# A genetic atlas for the butterflies of continental Canada and United States

**DOI:** 10.1371/journal.pone.0300811

**Published:** 2024-04-03

**Authors:** Jacopo D’Ercole, Leonardo Dapporto, Paul Opler, Christian B. Schmidt, Chris Ho, Mattia Menchetti, Evgeny V. Zakharov, John M. Burns, Paul D. N. Hebert

**Affiliations:** 1 Centre for Biodiversity Genomics, Guelph, Ontario, Canada; 2 Department of Integrative Biology, University of Guelph, Guelph, Ontario, Canada; 3 Department of Biology, University of Florence, Sesto Fiorentino, Italy; 4 Colorado State University, Fort Collins, Colorado, United States of America; 5 Canadian National Collection of Insects, Arachnids and Nematodes, Agriculture and Agri-Food Canada, Ottawa, Ontario, Canada; 6 Institut de Biologia Evolutiva (CSIC-UPF), Barcelona, Spain; 7 Department of Entomology, Smithsonian Institution, Washington, DC, United States of America; Institute of Systematics and Evolution of Animals Polish Academy of Sciences, POLAND

## Abstract

Multi-locus genetic data for phylogeographic studies is generally limited in geographic and taxonomic scope as most studies only examine a few related species. The strong adoption of DNA barcoding has generated large datasets of mtDNA COI sequences. This work examines the butterfly fauna of Canada and United States based on 13,236 COI barcode records derived from 619 species. It compiles i) geographic maps depicting the spatial distribution of haplotypes, ii) haplotype networks (minimum spanning trees), and iii) standard indices of genetic diversity such as nucleotide diversity (π), haplotype richness (H), and a measure of spatial genetic structure (G_ST_). High intraspecific genetic diversity and marked spatial structure were observed in the northwestern and southern North America, as well as in proximity to mountain chains. While species generally displayed concordance between genetic diversity and spatial structure, some revealed incongruence between these two metrics. Interestingly, most species falling in this category shared their barcode sequences with one at least other species. Aside from revealing large-scale phylogeographic patterns and shedding light on the processes underlying these patterns, this work also exposed cases of potential synonymy and hybridization.

## Introduction

Because of its high mutation rate and clean genealogical signal, mitochondrial DNA has long been used to detail the spatial configuration of genetic lineages [1]. However, the growing capacity to generate genetic data now makes it possible to also investigate multiple nuclear markers or entire genomes. While the latter approaches overcome limitations of a single marker [2–4] in accurately describing patterns of genetic diversity [5], their application is typically limited to only a few species. The broad adoption of the mitochondrial COI barcode region for animals [6] stimulated the development of BOLD (https://v4.boldsystems.org) [7], a data repository that now holds more than 16 million geo-referenced DNA barcode records. As particular effort has been directed towards Lepidoptera [e.g., 8–11], phylogeographic assessments are now possible for all species on a continental scale [12]. Founded on an updated checklist for the butterfly species of North America, the present study assembles a curated dataset of 13,236 DNA barcode records for this fauna, nearly 15% of which are new. Based on these data, it compiles haplotype maps and networks for each species. This information was integrated with standard indices of genetic diversity to characterize their genetic diversity and genetic structure.

## Methods

### Sampling and COI characterization

**Fig 1:**
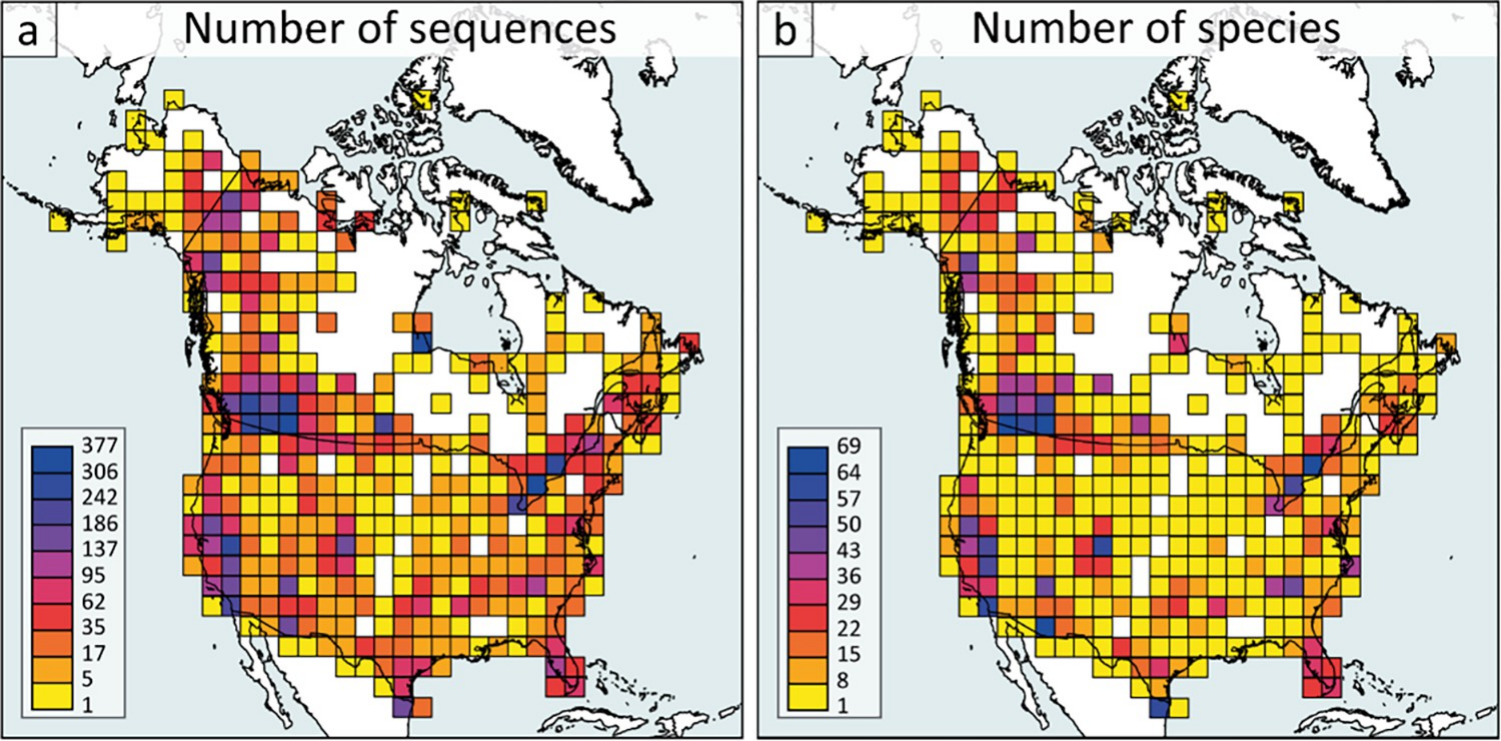
Sampling coverage. Number of sequences (a) and number of species (b) displayed on a grid of cells, where each cell is 200 x 200 km^2^.

The barcode data considered here mainly derive from an earlier compilation [8] reinforced with 1,750 additional records. A total of 13,236 georeferenced records (6838—Canada, 6398—USA) were assembled in the dataset “DS-ATLASNAB” (dx.doi.org/10.5883/DS-ATLASNAB). All 13,236 records included at least 500 unambiguous base pairs. This study provided barcode coverage for 619 species, 90% (619 of 691) of the butterfly taxa known to be resident in Canada and the continental United States (**Fig 1**). While sampling bias is inevitable, targeted selection of samples from two major natural history collections in North America, namely the Canadian National Collection and the Smithsonian’s National Museum of Natural History, helped to alleviate this issue. Sampling considered both the geographic and phylogenetic diversity of the species. The fauna is delineated by oceanic dispersal barriers to the east, west, and north and by the arid regions of northern Mexico to the south. The present checklist is largely based on D’Ercole et al., [8] augmented by recent taxonomic studies to refine the faunal list (S1 Appendix). In a few cases, where the evidence for revised taxonomic status was considered incomplete, proposed changes were not adopted, but these cases are highlighted in the checklist. Zhang et al. [13] provided low coverage (1x) genomic data for all North American butterfly species to advance understanding of their diversification and adaptation, but the results also revealed incongruencies with the current taxonomic system that were considered in this study.

DNA extraction, polymerase chain reaction (PCR), and sequencing followed the established procedures at the Centre for Biodiversity Genomics. DNA extraction utilized a silica-based method implemented in 96-well plate format [14]. PCR volumes and thermal cycling conditions followed methods in DeWaard et al. [15]. Although Sanger sequencing can recover small DNA fragments [16], the need to characterize numerous short amplicons is expensive and requires a substantial input DNA. High-throughput sequencing (HTS) not only provides the capability to analyze multiple sets of amplicons simultaneously but also, due to their ability to analyze single molecules, can produce reliable results with low concentrations of template DNA [17]. This study employed the Sequel platform to characterize short amplicons generated by multiplexing different primer sets and nested PCR [18]. CodonCode Aligner (CodonCode Corporation, http://www.codoncode.com) was employed to assemble trace files into contigs, thereby generating a sequence record for each specimen. Data validation revealed a few sequences that reflected either contamination by non-target species or nuclear mitochondrial elements (NUMTs); these records were excluded. A Neighbor-Joining tree [19] for the 619 species was built to search for unexpected placements, a situation that often reflects operational errors [20], but none were detected.

### Genetic analysis

The data were analyzed using methods described by Dapporto et al. [12]. Haplotype maps were constructed to depict the geographic distribution of genetic lineages. A matrix of p-distances (with pairwise deletion of missing sites) computed using the records for each species was subjected to Principal Coordinates Analysis (PCoA) (S1A Fig provides an example for *Limenitis lorquini*). The resultant 2-dimensional space was then overlapped with a colored square whose vertices were yellow, blue, white, and black. Shades of these colors defined the internal space of the square (e.g. S1B Fig). The overlap between the dimensional space of genetic distances and the colored square allowed assignment of colors to haplotypes—an approach that allowed color similarity to serve as a proxy for genetic similarity (e.g. S1C Fig). Color choice considered the most common types of color blindness. Haplotypes were then grouped by areas and displayed in circles whose size was proportional to the number of sequences (e.g. S2 Fig). In addition to haplotype maps [12], the present work established a framework for examining the spatial distribution of closely related species and revealing potential hybrid zones. This analysis treated two or more species as a single entity, so that their haplotypes were included in the same haplotype network and followed the same PCoA color scheme. However, specimens from different species were then displayed on separate maps to clearly locate areas of introgression (e.g., S3 Fig). Haplotype networks were used to examine relationships among the haplotypes detected in each species. The R package pegas (rmst function) was used to construct minimum spanning trees [21] with each haplotype represented by a circle whose size was proportional to the number of records. Numbers over the connections among haplotypes indicate the number of mutational steps separating them. These haplotype networks followed the color scheme established in the PCoA, which is the same than that employed for the haplotype maps (e.g., S4 Fig). Comprehensive understanding of the genetic structure of a species requires both the quantification of diversity and knowledge of its distribution across space. A bivariate bubble plot was employed to display the relationship between nucleotide diversity (π) [22] and G_ST_ [23], a measure of regional variation, for each species (e.g., S5 Fig). The function “nuc.div” of the pegas R package [24] was used to estimate nucleotide diversity. G_ST_ is defined as D_ST_/H_T_, where DST = (H_T_-HS)/H_T_, and H_T_ is the mean p-distance among all sequences while H_S_ is the mean p-distance among sequences of the single populations. G_ST_ ranges from -1 to 1. Because negative values typically represent artefacts due to low sample size [25], we set them to 0. Species represented by a single haplotype possessed a D_ST_ value of 0 and an undefined G_ST_, but G_ST_ was set to 0 in these cases as a species with a single haplotype lacks structure. Records were binned to cells of differently sized grids. It needs emphasis that G_ST_ values depend on the size of the grid cells and on the arbitrary placement of the grid on the geographic map. To overcome these limitations, G_ST_ was computed for five cell sizes (i.e., 100x100, 200x200, 300x300 and 400x400 km^2^) and replicate values were computed by moving cell centers to cell vertices. This approach produced 8 values for G_ST_ and the average of these values was retained for each species. G_ST_ was computed only for species having more than 10 specimens included in at least two areas, with no less than three specimens in each. Similar lower bounds were employed to estimate spatial structure in Scalercio et al. [26]. Following Dincă et al. [10], estimates of sampling completeness were obtained with the iNEXT R package [27]. This approach generates accumulation curves [28] that can be used to compare the asymptotic value with observed haplotype diversity. Because the accuracy of these estimates depends on sampling intensity, only species with more than ten specimens were analyzed. Following this approach, the number of specimens (N), the number of observed haplotypes (H), the estimated fraction of haplotype diversity retrieved (R), and the number of additional haplotypes which likely remain to be sampled (L) were computed for each species in the Atlas. In addition to estimates of sampling completeness, π, and G_ST_, Nei haplotype diversity (h) [29], the maximum intraspecific distance, and the nearest neighbor distance were also computed and included in S1 Appendix.

## Results and discussion

Taxonomic systems are dynamic, experiencing updates as knowledge of evolutionary relationships increases. This study was founded on a checklist assembled by D’Ercole et al. [8] which was updated based on genomic evidence [13] to produce a new reference list (S1 Appendix). The resulting new checklist includes 72 changes in taxonomic status—16 new names (9 species, 7 genera), 55 hierarchy changes (12 synonymies, 14 taxa moved to a lower rank, 29 taxa elevated to a higher rank). These changes impacted the taxonomic status of 158 North American butterfly species. In addition to these changes, 15 species were transferred between existing genera.

By examining the distribution of genetic diversity in 619 species, this study revealed three main phylogeographic patterns (S1 Appendix). Contrary to findings for Europe, where formerly glaciated areas tended to show low genetic variation [12], high genetic diversity and structure were particularly evident in the northwest (i.e., Alaska, Yukon, British Columbia) (e.g., *Erebia pawloskii*, *Boloria frigga*, *Oeneis uhleri*). Because Beringia in the north was a glacial refugium throughout much of the Pleistocene [30–32], populations of numerous species likely experienced recurrent isolation in this region, contributing to the observed diversity [32]. This pattern could also be influenced by other factors. Previous work on North American butterflies has shown that sharing of mitochondrial haplotypes is prevalent among closely related species in the north [8]. Consequently, it is probable that a portion of the diversity in this region derives from hybridization and introgression of mitochondrial lineages, rather than through intraspecific diversification. Notably, analysis of sequence variation for European butterflies [10,12] indicated the impact of barcode sharing is less than noted in this study [10,12]. It is possible that the varied landscape of Europe [33] limited genetic exchange among populations during the recurrent glacial cycles [34]. The southernmost regions of United States displayed high genetic diversity and structure (e.g., *Thorybes pylades*, *Calycopis cecrops*, *Hermeuptychia sosybius*). This pattern conforms not only with the traditional model of genetic diversity described for the European biota [35,36], but also with recent genetic findings pertaining to west Palearctic butterflies [8,12]. Second, species occurring in proximity to the western mountains typically showed both high genetic diversity and relatively high spatial structure (e.g., *Argynnis cybele*, *Neophasia menapia*, *Polites draco*). This agrees with patterns of genetic diversity observed for the cold-adapted species in Europe, where several differentiated endemic lineages are found in correspondence with the major mountain chains (i.e. Pyrennees, Alps, and Carpathians). These lineages often result from multiple centers of glacial survival in the perialpine areas [37,38]. This pattern could also reflect dispersal from glacial refugia and the aggregation of lineages near barriers [32,39] or relaxed natural selection on hybrid populations. Hybrid zones are frequently localized in regions with restricted gene flow and low population density where the negative effects of outbreeding depression are minimized [26,40,41]. The present results support this prediction as many species distributed along the western mountain chains share their barcodes with at least one other species (e.g., *Icaricia lupini*, *Euphydryas anicia*, *Argynnis zerene*). Third, although most species displayed either high genetic diversity/structure, or low diversity/structure, a considerable number showed a modest increase in genetic structure when compared to genetic diversity (e.g., *Euphilotes enoptes*, *Limenitis arthemis*, *Phyciodes cocyta*). As most species in this category displayed barcode sharing, it is probable that hybridization generates hotspots of diversity where different lineages congregate, a situation that results in a lack of genetic structure.

Aside from providing a glimpse of large-scale patterns of intraspecific genetic diversity, this study enables exploration of unique patterns displayed by single species linked to differing biological attributes and environmental scenarios [36,42] (S1 Appendix). For instance, while the primary response to glacial advances for species in western North America was either a retreat to southerly refugia or to the northern Beringian refuge [39], the present analysis highlights a few species (e.g., *Euchloe ausonides*, *Pieris marginalis*, *Boloria chariclea*) that likely retreated into micro-refugia on the margins of the west coast (i.e., Vancouver Island, Haida Gwaii, Alexander Archipelago). This agrees with past evidence supporting the existence of smaller refugia located off the coasts of British Columbia and Alaska [39]. Similar evidence is also found in Europe, where extra-Mediterranean source populations north of major mountain chains (i.e., Pyrenees, Alps, and Carpathians) helped to repopulate glaciated regions [43,44].

Studies of sequence variation have significant implications for conservation programs as they can provoke taxonomic revisions, impacting the fundamental units of conservation. The genus *Celastrina* provides an example as its taxonomy has been volatile over the last 30 years. Scott (1992) [45] viewed all North American specimens as belonging to the Eurasian species *C*. *argiolus*. However, subsequent workers argued that the North American *Celastrina* was composed of multiple species [46,47] and eventually, nine North American endemics were recognized [48] The present results indicate that all North American *Celastrina* species show close barcode congruence and share haplotypes with at least one other species. This result reinforces conclusions from detailed morphological and phenological analysis [49] which suggest that the supposed *Celastrina* species are actually conspecific (S1 Appendix). Another example of taxonomic and evolutionary interest involves the genus *Erynnis*. Two species, *E*. *propertius* and *E*. *horatius*, are allopatric and display clear morphological (including genitalia) and ecological differences [50]. While *E*. *propertius* exists in the far west (from southwest Canada to north Baja California), *E*. *horatius* is distributed in central and eastern North America (from New Mexico and Colorado up to the east coast). Zakharov et al., [51] detected *E*. *horatius* haplotypes in many populations of *E*. *propertius* and concluded that introgression from the western *E*. *horatius* to eastern *E*. *propertius* represented the most likely explanation. However, the increased sampling coverage in this study reveals a different scenario. A third, closely related species, *E*. *meridianus*, ranges between these two species (from south Nevada through much of Arizona and New Mexico to Texas) and displays the same haplotypes introgressed from *E*. *horatius* to *E*. *propertius*. It is probable that the present configuration of mitochondrial lineages arose with *E*. *meridianus* as an intermediary. More specifically, the ancestral species of *E*. *meridianus* might either have transferred its haplotypes to both *E*. *horatius* and *E*. *propertius*, or it might have acted as a stepping-stone from *E*. *horatius* to *E*. *propertius*. Additional barcode coverage coupled with studies on nuclear markers should resolve this case.

This phylogeographic assessment for all species in a large phylogenetic group demonstrates how the use of a well-curated reference library can aid understanding of biodiversity and its connection to underlying processes. Moreover, it can be used to improve the current classification system and to reveal cases of evolutionary interest such as hybridization and genetic introgression. It is worth noting that a similar atlas can be used as a routine practice to improve the quality of DNA barcode reference libraries of poorly known taxonomic groups where the impact of operational errors (e.g., inaccurate taxonomy, misidentified specimens) is large.

## Supporting information

S1 Appendix The genetic atlas for the butterflies of the continental Canada and United States. This pdf displays intraspecific genetic diversity for 619 butterfly species.
(PDF)

S1 Fig Principal coordinate analysis (PCoA) for *Limenitis arthemis* (a). The RGB (red blue green) square employed to assign colors to haplotypes (b) and the projection of the PCoA configuration in the RGB space (c).
(PNG)

S2 Fig The representation of PCoA in RGB space as showed in the Atlas (a) and the resulting haplotype map (b) for 36 specimens of *Limenitis lorquini*.
(PNG)

S3 Fig Haplotype maps for 36 specimens of *Limenitis lorquini* (a) and 102 specimens of *Limenitis arthemis* (b).
(PNG)

S4 Fig PCoA (a) and haplotype network (b) for 36 specimens of *Limenitis lorquini*. Because colors assigned to haplotypes are the same for the PCoA and the haplotype network, it is possible to easily identify same haplotypes in the two plots.
(PNG)

S5 Fig Bivariate plot of mitochondrial diversity (square root transformed nucleotide diversity) and standardised spatial structure (GST). Values for all species in the Atlas are depicted as grey bubbles and the species of interest (*Limenitis lorquini*) is represented by a purple dot. The black lines represent median values for all species of the Atlas.
(PNG)

## References

[1] Avise JC, Arnold J, Ball RM, Bermingham E, Lamb T, Neigel JE. Intraspecific phylogeography: the mitochondrial DNA bridge between population genetics and systematics. Annu Rev Ecol Syst. 1987;18:489–522.

[2] Gompert Z, Forister ML, Fordyce JA, Nice CC. Widespread mito-nuclear discordance with evidence for introgressive hybridization and selective sweeps in *Lycaeides*. Mol Ecol. 2008;17(24):5231–44. 19120997 10.1111/j.1365-294X.2008.03988.x

[3] Toews DPL, Brelsford A. The biogeography of mitochondrial and nuclear discordance in animals. Mol Ecol. 2012;21(16):3907–30. doi: 10.1111/j.1365-294X.2012.05664.x 22738314

[4] Wahlberg N, Weingartner E, Warren AD, Nylin S. Timing major conflict between mitochondrial and nuclear genes in species relationships of *Polygonia* butterflies (Nymphalidae: Nymphalini). BMC Evol Biol. 2009;9(1):92. 19422691 10.1186/1471-2148-9-92 PMC2688511

[5] Edwards SV, Robin VV, Ferrand N, Moritz C. The evolution of comparative phylogeography: putting the geography (and more) into comparative population genomics. Genome Biol Evol. 2022;14(1). doi: 10.1093/gbe/evab176 34347070 PMC8743039

[6] Hebert PDN, Cywinska A, Ball SL, deWaard JR. Biological identifications through DNA barcodes. Proc R Soc Lond B Biol Sci. 2003;270(1512):313–21. doi: 10.1098/rspb.2002.2218 12614582 PMC1691236

[7] Ratnasingham S, Hebert PDN. BOLD: The Barcode of Life Data System (www.barcodinglife.org). Mol Ecol Notes. 2007;7:355–64. 18784790 10.1111/j.1471-8286.2007.01678.x PMC1890991

[8] D’Ercole J, Dincă V, Opler PA, Kondla N, Schmidt C, Phillips JD, et al. A DNA barcode library for the butterflies of North America. PeerJ. 2021;9:e11157. doi: 10.7717/peerj.11157 33976967 PMC8061581

[9] D’Ercole J, Dapporto L, Schmidt CB, Dincă V, Talavera G, Vila R, et al. Patterns of DNA barcode diversity in butterfly species (Lepidoptera) introduced to the Nearctic. Eur J Entomol. 2022;119:379–87.

[10] Dincă V, Dapporto L, Somervuo P, Vodă R, Cuvelier S, Gascoigne-Pees M, et al. High resolution DNA barcode library for European butterflies reveals continental patterns of mitochondrial genetic diversity. Commun Biol. 2021;4(315). doi: 10.1038/s42003-021-01834-7 33750912 PMC7943782

[11] Zahiri R, Lafontaine DJ, Schmidt CB, deWaard JR, Zakharov EV, Hebert PDN. Probing planetary biodiversity with DNA barcodes: the Noctuoidea of North America. PLOS One. 2017;12(6). doi: 10.1371/journal.pone.0178548 28570635 PMC5453547

[12] Dapporto L, Menchetti M, Vodă R, Corbella C, Cuvelier S, Djemadi I, et al. The atlas of mitochondrial genetic diversity for Western Palaearctic butterflies. Global Ecology and Biogeography. 2022;31(11):2184–90.

[13] Zhang J, Cong Q, Shen J, Opler PA, Grishin N V. Genomics of a complete butterfly continent. bioRxiv. 2019;

[14] Ivanova NV., DeWaard JR, Hebert PDN. An inexpensive, automation-friendly protocol for recovering high-quality DNA. Mol Ecol Notes. 2006;6(4):998–1002.

[15] DeWaard J, Ivanova N, Hajibabaei M, Hebert P. Assembling DNA Barcodes. Analytical protocols. Methods Mol Biol. 2008;410:275–93. doi: 10.1007/978-1-59745-548-0_15 18642605

[16] Hebert PDN, DeWaard JR, Zakharov E V, Prosser SWJ, Sones JE, McKeown JTA, et al. A DNA “Barcode Blitz”: rapid digitization and sequencing of a natural history collection. PLOS One. 2013;8(7). doi: 10.1371/journal.pone.0068535 23874660 PMC3707885

[17] Prosser SWJ, DeWaard JR, Miller SE, Hebert PDN. DNA barcodes from century-old type specimens using next-generation sequencing. Mol Ecol Resour. 2016;16(2):487–97. doi: 10.1111/1755-0998.12474 26426290

[18] D’Ercole J, Prosser SWJ, Hebert PDN. A SMRT approach for targeted amplicon sequencing of museum specimens (Lepidoptera)—patterns of nucleotide misincorporation. PeerJ. 2021;9:e10420. doi: 10.7717/peerj.10420 33520432 PMC7811786

[19] Saitou N, Nei M. The neighbor-joining method: a new method for reconstructing phylogenetic trees. Mol Biol Evol. 1987;4(4):406–25. doi: 10.1093/oxfordjournals.molbev.a040454 3447015

[20] Mutanen M, Kivelä SM, Vos RA, Doorenweerd C, Ratnasingham S, Hausmann A, et al. Species-level para- and polyphyly in DNA barcode gene trees: strong operational bias in European lepidoptera. Syst Biol. 2016;65(6):1024–40. doi: 10.1093/sysbio/syw044 27288478 PMC5066064

[21] Paradis E. Analysis of haplotype networks: the randomized minimum spanning tree method. Methods Ecol Evol. 2018;9(5):1308–17.

[22] Tajima F. Evolutionary relationship relationship of DNA sequences in finite populations. Genetics. 1983;105(2):437–60. 6628982 10.1093/genetics/105.2.437 PMC1202167

[23] Nei M. Analysis of gene diversity in subdivided populations. Proc Natl Acad Sci USA. 1973;70(12):3321–3. doi: 10.1073/pnas.70.12.3321 4519626 PMC427228

[24] Paradis E. pegas: an R package for population genetics with an integrated–modular approach. Bioinformatics. 2010;26(3):419–20. doi: 10.1093/bioinformatics/btp696 20080509

[25] Meirmans PG, Hedrick PW. Assessing population structure: FST and related measures. Mol Ecol Resour. 2011;11(1):5–18. doi: 10.1111/j.1755-0998.2010.02927.x 21429096

[26] Scalercio S, Cini A, Menchetti M, Vodă R, Bonelli S, Bordoni A, et al. How long is 3 km for a butterfly? Ecological constraints and functional traits explain high mitochondrial genetic diversity between Sicily and the Italian peninsula. J Anim Ecol. 2020;89(9):2013–26. 32207150 10.1111/1365-2656.13196

[27] Hsieh TC, Ma KH, Chao A. iNEXT: an R package for rarefaction and extrapolation of species diversity (Hill numbers). Methods Ecol Evol. 2016;7(12):1451–6.

[28] Chao A. Nonparametric estimation of the number of classes in a population. Scandinavian Journal of Statistics. 1984;11(4):265–70.

[29] Nei M. Molecular evolutionary genetics. New York: Columbia University Press; 1987.

[30] Mikkola K, Lafontaine D, Kononenko V. Zoogeography of the Holarctic species of the Noctuidae (Lepidoptera): importance of the Beringian refuge. Entomol Fenn. 1991;2(3):157–73.

[31] Lafontaine JD, Wood DM. A zoogeographic analysis of the Noctuidae (Lepidoptera) of Beringia, and some inferences about past Beringian habitats. Memoirs of the Entomological Society of Canada. 1988;120(144):109–23.

[32] Swenson NG, Howard DJ. Clustering of contact zones, hybrid zones, and phylogeographic breaks in North America. Am Nat. 2005;166(5):581–91. doi: 10.1086/491688 16224723

[33] Blondel J, Aronson JF, Bodiou GB. The Mediterranean region: biological diversity in space and time. Oxford: Oxford University Press; 2010.

[34] Dapporto L, Cini A, Vodă R, Dincă V, Wiemers M, Menchetti M, et al. Integrating three comprehensive data sets shows that mitochondrial DNA variation is linked to species traits and paleogeographic events in European butterflies. Mol Ecol Resour. 2019;19(6):1623–36. doi: 10.1111/1755-0998.13059 31325412

[35] Hewitt G. The genetic legacy of the Quaternary ice ages. Nature. 2000;405(6789):907–13. doi: 10.1038/35016000 10879524

[36] Taberlet P, Fumagalli L, Wust-Saucy AG, Cosson JF. Comparative phylogeography and postglacial colonization routes in Europe. Mol Ecol. 1998;7(4):453–64. doi: 10.1046/j.1365-294x.1998.00289.x 9628000

[37] Schmitt T. Biogeographical and evolutionary importance of the European high mountain systems. Front Zool. 2009;6(9). doi: 10.1186/1742-9994-6-9 19480666 PMC2700098

[38] Minter M, Dasmahapatra K, Thomas C, Morecroft M, Tonhasca A, Schmitt T, et al. Past, current, and potential future distributions of unique genetic diversity in a cold-adapted mountain butterfly. Ecol Evol. 2020;10(20):11155–68. doi: 10.1002/ece3.6755 33144956 PMC7593187

[39] Shafer A, Cullimgham C, Côté SD, Coltman DW. Of glaciers and refugia: a decade of study sheds new light on the phylogeography of northwestern North America. Mol Ecol. 2010;19(21):4589–621. doi: 10.1111/j.1365-294X.2010.04828.x 20849561

[40] Harrison R. Hybrid zones and the evolutionary process. J Evol Biol. 1994;7(5):631–4.

[41] Rosser N, Shirai LT, Dasmahapatra KK, Mallet J, Freitas AVL. The Amazon river is a suture zone for a polyphyletic group of co-mimetic heliconiine butterflies. Ecography. 2021;44(2):177–87.

[42] Soltis DE, Morris Ashley B, McLachlan JS, Manos PS, Soltis PS. Comparative phylogeography of unglaciated eastern North America. Mol Ecol. 2006;15(14):4261–93. doi: 10.1111/j.1365-294X.2006.03061.x 17107465

[43] Stewart JR, Lister AM. Cryptic northern refugia and the origins of the modern biota. Trends Ecol Evol. 2001;16(11):608–13.

[44] Schmitt T, Varga Z. Extra-Mediterranean refugia: the rule and not the exception? Front Zool. 2012;9(1):2194–207. doi: 10.1186/1742-9994-9-22 22953783 PMC3462695

[45] Scott J. The butterflies of North America: a natural history and field guide. Stanford University Press; 1992.

[46] Pratt G, Wright D, Pavulaan H. The various taxa and hosts of the North American *Celastrina* (Lepidoptera: Lycaenidae). Proc Entomol Soc Wash. 1994;96:566–78.

[47] Pavulaan H. *Celastrina serotina* (Lycaenidae: Polyommatinae): a new butterfly species from the northeastern United States and eastern Canada. Taxonomic Report of the International Lepidoptera Survey. 2005;6:1–18.

[48] Pelham J. A catalogue of the butterflies of the United States and Canada [Internet]. 2022. Available from: https://butterfliesofamerica.com/US-Can-Cat.htm

[49] Schmidt B, Layberry R. What Azure Blues occur in Canada? A re-assessment of *Celastrina* Tutt species (Lepidoptera, Lycaenidae). Zookeys. 2016;584:135–64. 10.3897/zookeys.584.7882 PMC4857028 27199600

[50] Burns J. Evolution in skipper butterflies of the genus *Erynnis*. University of California Publication in Entomology. 1964;37:1–206.

[51] Zakharov E, Lobo N, Nowak C, Hellmann J. Introgression as a likely cause of mtDNA paraphyly in two allopatric skippers (Lepidoptera: Hesperiidae). Heredity. 2009;102(6):590–9. doi: 10.1038/hdy.2009.26 19293835

