# Supplementary material for: A genetic atlas for the butterflies of continental Canada and United States
Source: PLoS One. 2024 Apr 3;19(4):e0300811. doi: 10.1371/journal.pone.0300811 (PMC10990199; doi:10.1371/journal.pone.0300811)
Supplement: S1 Appendix — This pdf displays intraspecific genetic diversity for 619 butterfly species. (PDF) [file pone.0300811.s001.pdf]

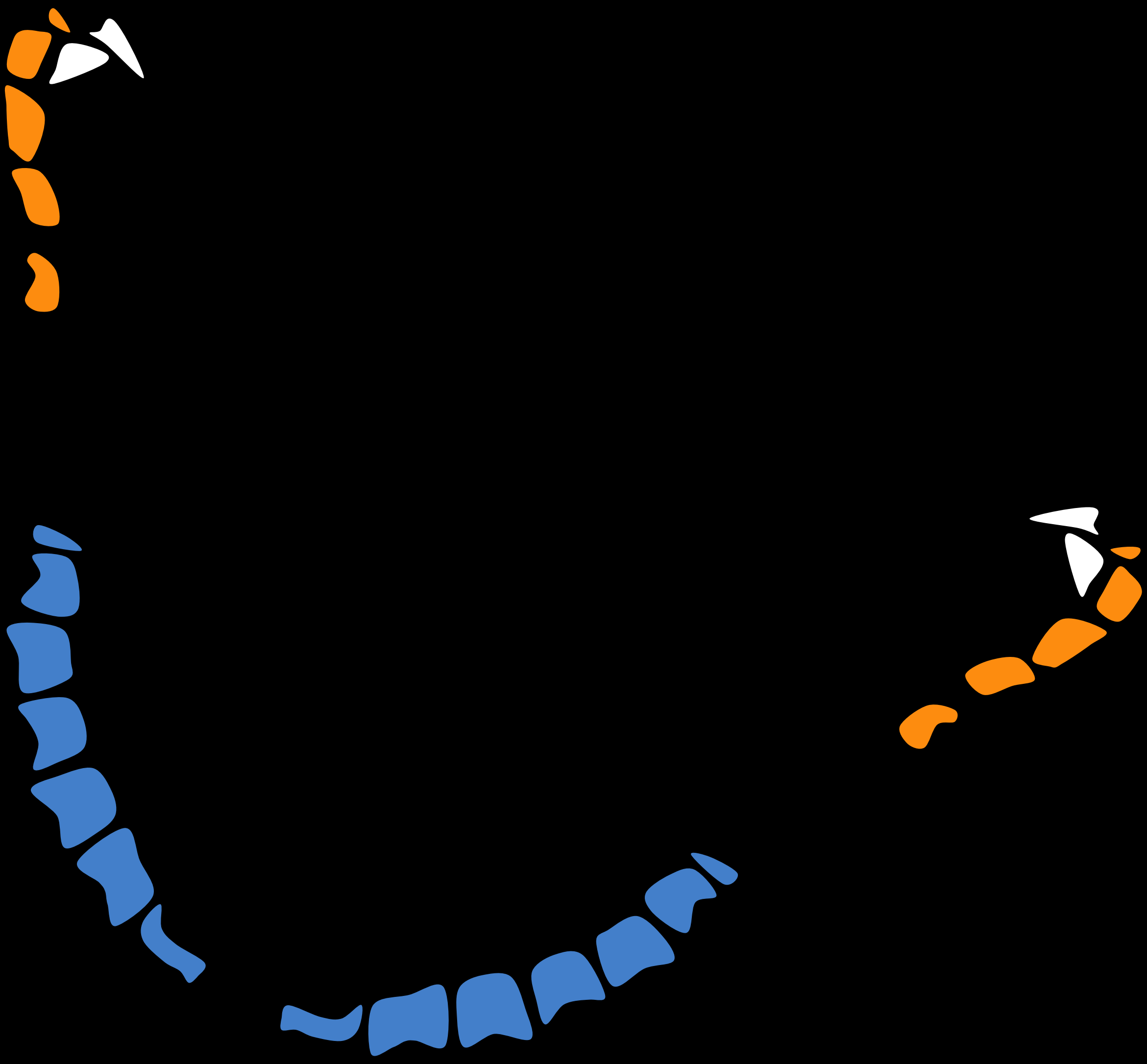

A Genetic Atlas for the Butterflies of the Continental Canada and United States

Jacopo D’Ercole, Leonardo Dapporto, Paul Opler, Christian B. Schmidt, Chris Ho, Mattia Menchetti, Evgeny V. Zakharov V, John M. Burns, Paul D. N. Hebert

A Genetic Atlas for the Butterflies of the Continental Canada and United States

Introduction

This Atlas describes different aspects of genetic diversity for the butterfly species of Canada and United States. Founded on an updated checklist (**Table 1**), this work assembles a curated dataset of 13,236 DNA barcode records (dx.doi.org/10.5883/DS-ATLASNAB), corresponding to 619 species (90% of the North American fauna). Conventional measures of genetic diversity to assess both the genetic diversity and genetic structure were computed for each species (**Table 2**). Based on these data, it compiles i) haplotype maps ii) haplotype networks iii) a bivariate plot showing the relationship between genetic diversity and genetic structure, iv) and a principal component analysis (PCoA) that provides a bidimensional representation of genetic distances among haplotypes of each species.

Methods

Sampling and COI characterization

The barcode data considered here mainly derive from an earlier compilation (1) reinforced with 1,750 additional records. A total of 13,236 georeferenced records (6838—Canada, 6398—USA) were assembled in the dataset "DS-ATLASNAB" (dx.doi.org/10.5883/DS-ATLASNAB). All 13,236 records included at least 500 unambiguous base pairs. This study provided barcode coverage for 619 species, 90% (619 of 691) of the butterfly taxa known to be resident in Canada and the continental United States (**Figure 1**). While sampling bias is inevitable, targeted selection of samples from two major natural history collections in North America, namely the Canadian National Collection and the Smithsonian's National Museum of Natural History, helped to alleviate this issue. Sampling in fact considered both the geographic and phylogenetic diversity of the examined species. The fauna is delineated by oceanic dispersal barriers to the east, west and north and by the arid regions of northern Mexico to the south. The present checklist is largely based on D'Ercole et al., (1) augmented by recent taxonomic studies to refine the faunal list (**Table 1**). In a few cases where the evidence for revised taxonomic status was considered incomplete, proposed changes were not adopted, but these cases are highlighted in the checklist. Zhang et al. (2) provided low coverage (1x) genomic data for all North American butterfly species to advance understanding of their diversification and adaptation, but the results also revealed incongruencies with the current taxonomic system that were considered in this study.

DNA extraction, polymerase chain reaction (PCR), and sequencing followed the established procedures at the Centre for Biodiversity Genomics. DNA extraction utilized a silica-based method implemented in a 96-well plate format (3). PCR volumes and thermal cycling conditions followed methods in DeWaard et al. (4). Although Sanger sequencing can recover small DNA fragments (5), the need to characterize numerous short amplicons is expensive and requires a substantial amount of input DNA. High-throughput sequencing (HTS) methods not only provide the capability to analyze multiple sets of amplicons simultaneously but also, due to their ability to analyze single molecules, can produce reliable results with low concentrations of template DNA (6). This study employed the Sequel platform to characterize short amplicons generated by multiplexing different primer sets and nested PCR (7). CodonCode Aligner (CodonCode Corporation, http://www.codoncode.com) was employed to assemble trace files into contigs, thereby generating a sequence record for each specimen. Data validation revealed a few sequences that reflected either contamination by non-target species or nuclear mitochondrial elements (NUMTs); these records were excluded. A Neighbor-Joining tree (8) for the 619 species was built to search for unexpected placements, a situation that often reflects operational errors (9), but none were detected.

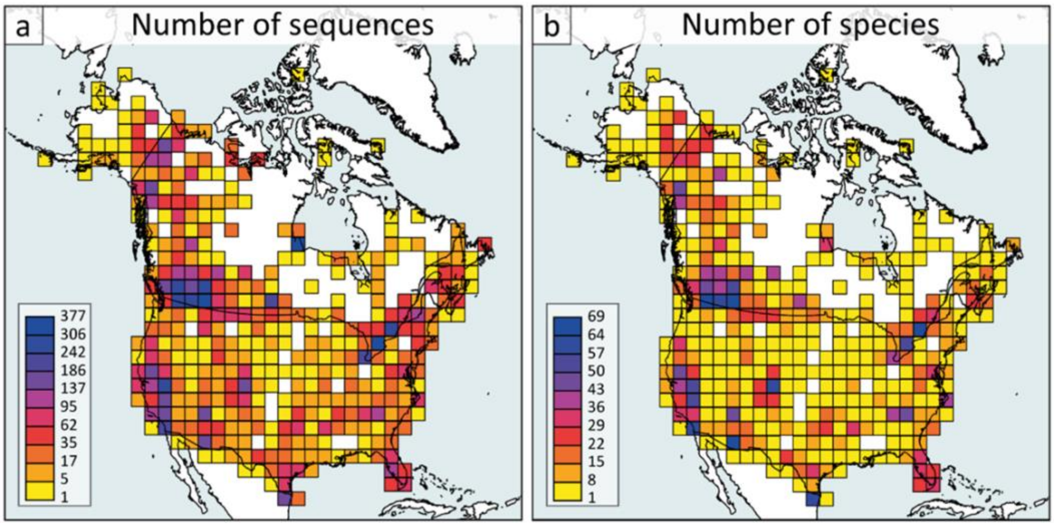

Figure 1. Sampling coverage. Number of sequences (a) and number of species (b) displayed on a grid of cells, where each cell is 200 x 200 km^2.

Genetic analysis

The data were analyzed using methods described by Dapporto et al. (10) . Haplotype maps were constructed to depict the geographic distribution of genetic lineages. A matrix of p-distances (with pairwise deletion of missing sites) computed using the records for each species was subjected to Principal Coordinates Analysis (PCoA) (**Figure 2a** provides an example for *Limenitis lorquini*). The resultant 2-dimensional space was then overlapped with a colored square whose vertices were

yellow, blue, white, and black. Shades of these colors defined the internal space of the square (e.g. **Figure 2b**). The overlap between the dimensional space of genetic distances and the colored square allowed assignment of colors to haplotypes—an approach that allowed color similarity to serve as a proxy for genetic similarity (e.g. **Figure 2c**). Color choice considered the most common types of color blindness.

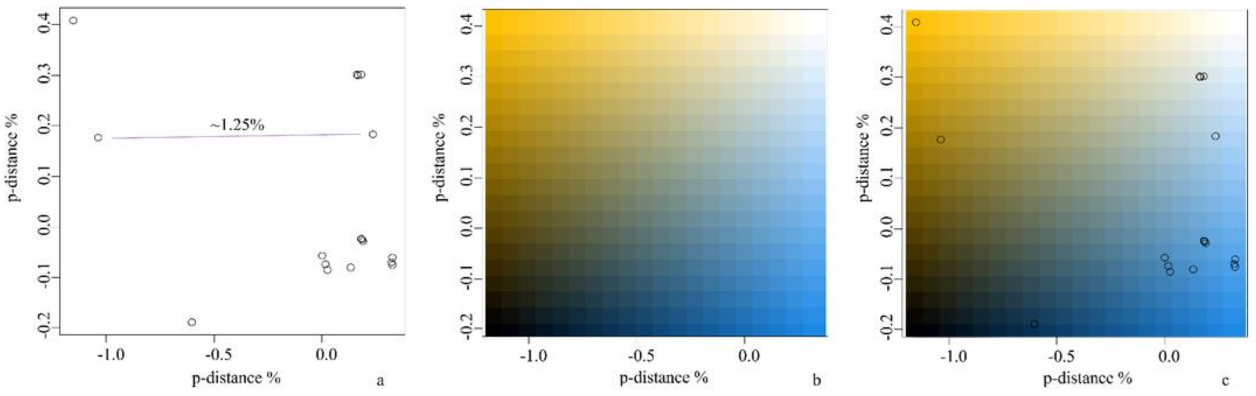

Figure 2. Principal coordinate analysis (PCoA) for *Limenitis arthemis* (a). The RGB (Red blue green) square employed to assign colors to haplotypes (b) and the projection of the PCoA configuration in the RGB space (c).

Haplotypes were then grouped by areas and displayed in circles whose size was proportional to the number of sequences (e.g. **Figure 3**).

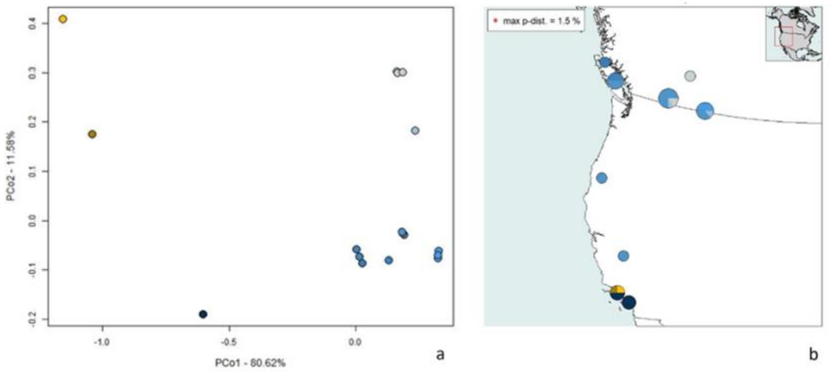

Figure 3. The representation of PcoA in RGB space as showed in the Atlas (a) and the resulting haplotype map (b) for 36 specimens of *Limenitis lorquini*.

In addition to haplotype maps (10), the present work established a framework for examining the spatial distribution of closely related species and revealing potential hybrid zones. This analysis treated two or more species as a single entity, so that their haplotypes were included in the same haplotype network and followed the same PCoA color scheme. However, specimens from different species were then displayed on separate maps to clearly locate area of introgression (e.g. **Figure 4**).

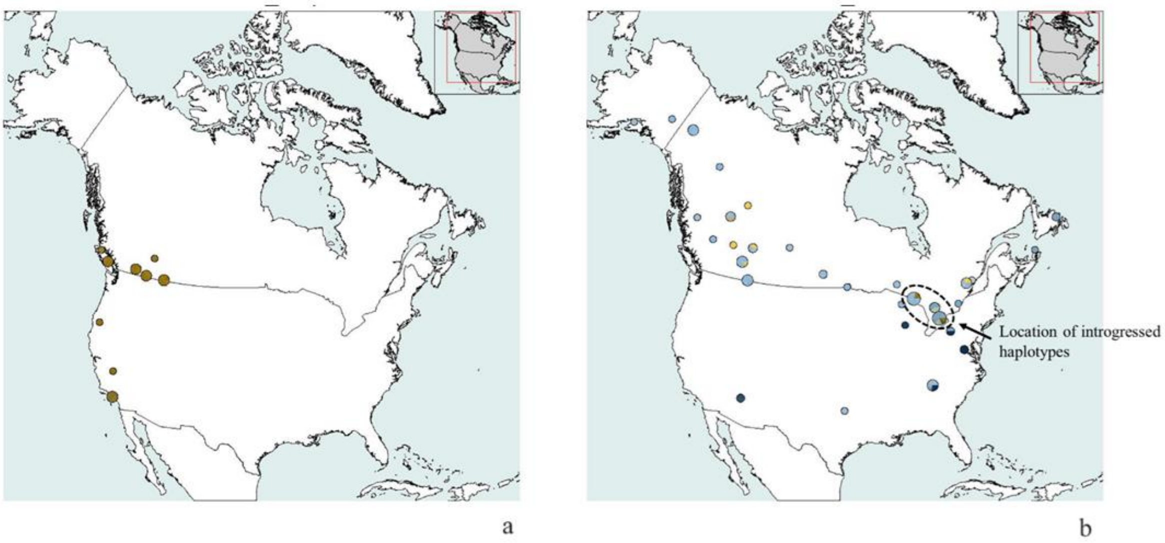

Figure 4. Haplotype maps for 36 specimens of *Limenitis lorquini* (a) and 102 specimens of *Limenitis arthemis* (b).

Haplotype networks were used to examine relationships among the haplotypes detected in each species. The R package pegas (rmst function) was used to construct minimum spanning trees (11) with each haplotype represented by a circle whose size was proportional to the number of records. Numbers over the connections among haplotypes indicate the number of mutational steps separating them. These haplotype networks followed the color scheme established in the PCoA, which is the same than that employed for the haplotype maps (e.g. **Figure 5**).

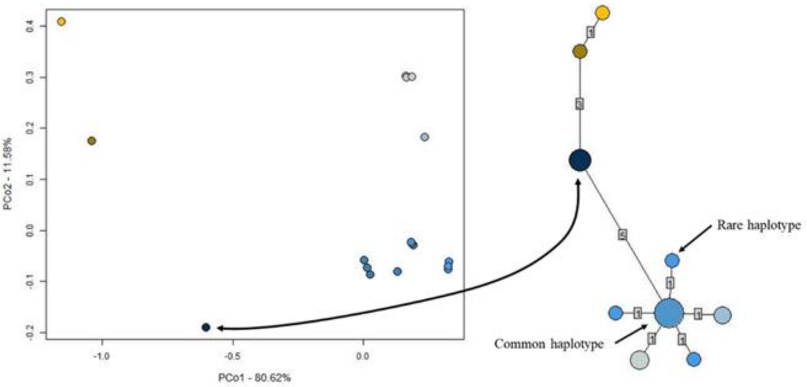

Figure 5. PCoA (a) and haplotype network (b) for 36 specimens of *Limenitis lorquini*. Because colors assigned to haplotypes are the same for the PCoA and the haplotype network, it is possible to easily identify same haplotypes in the two plots.

Comprehensive understanding of the genetic structure of a species requires both the quantification of diversity and knowledge of its distribution across space. A bivariate bubble plot was employed to

display the relationship between nucleotide diversity (π) (12) and G_{ST} (13), a measure of regional variation, for each species (e.g. **Figure 6**).

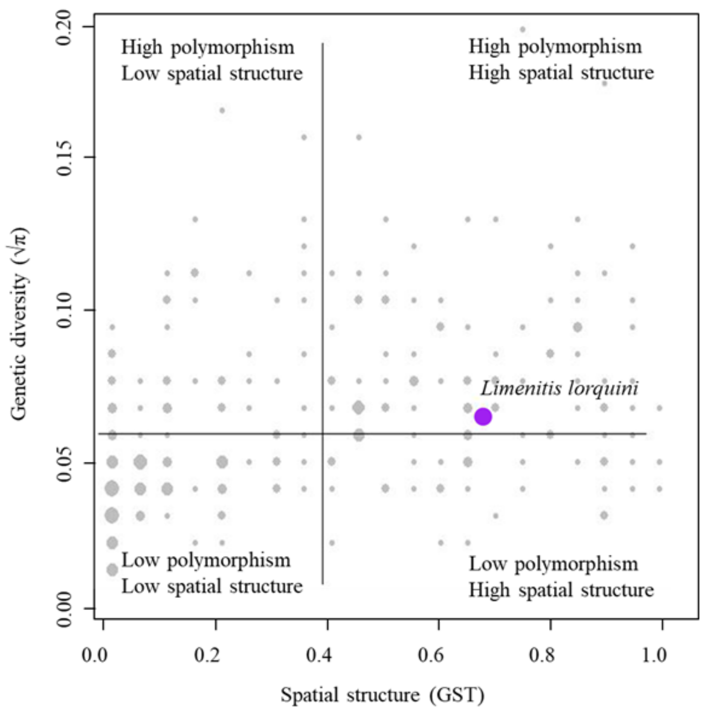

Figure 6. Bivariate plot of mitochondrial diversity (square root transformed nucleotide diversity) and standardised spatial structure (GST). Values for all species of the Atlas are depicted as grey bubbles and the species of interest (*Limenitis lorquini*) is represented by a purple dot. The black lines represent median values for all species of the Atlas.

The function "nuc.div" of the pegas R package (14) was used to estimate nucleotide diversity. G_{ST} is defined as D_{ST}/H_T, where DST = (H_T-HS)/H_T, and H_T is the mean p-distance among all sequences while H_S is the mean p-distance among sequences of the single populations. G_{ST} ranges from -1 to 1. Because negative values typically represent artefacts due to low sample size (15), we set them to 0. Species represented by a single haplotype possessed a DST value of 0 and an undefined GST, but G_{ST} was set to 0 in these cases as a species with a single haplotype lacks structure. Records were binned to cells of differently sized grids. It needs emphasis that G_{ST} values depend on the size of the grid cells and on the arbitrary placement of the grid on the geographic map. To overcome these limitations, G_{ST} was computed for five cell sizes (i.e., 100x100, 200x200, 300x300 and 400x400 km2) and replicate values were computed by moving cell centers to cell vertices. This approach produced 8 values for G_{ST} and the average of these values was retained for each species. G_{ST} was computed only for species having more than 10 specimens included in at least two areas, with no less than three specimens in each. Similar lower bounds were employed to estimate spatial structure in Scalercio et al. (16). Following Dincă et al. (17), estimates of sampling completeness were obtained with the iNEXT R package (18). This approach generates accumulation curves (19) that

can be used to compare the asymptotic value with observed haplotype diversity. Because the accuracy of these estimates depends on sampling intensity, only species with more than ten specimens were analyzed. Following this approach, the number of specimens (N), the number of observed haplotypes (H), the estimated fraction of haplotype diversity retrieved (R), and the number of additional haplotypes which likely remain to be sampled (L) were computed for each species in the Atlas. In addition to estimates of sampling completeness, π, and G_{ST}, Nei haplotype diversity (h) (8), the maximum intraspecific distance, and the nearest neighbor distance were also computed and included in **Table 2.**

Table 1. Checklist for the 691 butterfly species with resident populations in Canada and/or USA. Taxonomic changes proposed by authors detailed in the column "References" were applied to BOLD (https://v4.boldsystems.org), which previously followed the checklist proposed by D'Ercole et al. (2021).

Family	Genus	Species	Authorship	Changes in respect to D'Ercole et al. (2021)	
				**References**	**Notes**
Hesperiidae	*Aguna*	*Aguna mcguirei*	Grishin, 2023	(20)	North American records of *Aguna metophis* were transferred to the new species *Aguna mcguirei*
Hesperiidae	*Autochton*	*Autochton caballo*	Grishin, 2023	(20)	North American records of *Autochton potrillo* were transferred to thenew species *Autochton caballo*
Hesperiidae	*Cecropterus*	*Cecropterus casica*	(Herrich-Schäffer, 1869)	(21,22)	Transfer of species from *Thorybes* to *Cecropterus*. Tribe addition (Eudamini)
Hesperiidae	*Cecropterus*	*Cecropterus cincta*	Plötz, 1882	(21,22)	Transfer of species from *Thorybes* to *Cecropterus*. Tribe addition (Eudamini)
Hesperiidae	*Cecropterus*	*Cecropterus coyote*	(Skinner, 1892)	(23)	*Cecropterus coyote* is restored to species as it is distinct from *Cecropterus albociliatus* and *Cecropterus nigrociliata*
Hesperiidae	*Cecropterus*	*Cecropterus dorantes*	(Williams, 1926)	(22)	Transfer of species from *Urbanus* to *Cecropterus*
Hesperiidae	*Cecropterus*	*Cecropterus doryssus*	(Swainson, 1831)	(22)	Transfer of species from *Urbanus* to *Cecropterus*
Hesperiidae	*Cecropterus*	*Cecropterus drusius*	(Edwards, 1883)	(21,22)	Transfer of species from *Thorybes* to *Cecropterus*. Tribe addition (Eudamini)
Hesperiidae	*Cecropterus*	*Cecropterus toxeus*	(Plötz, 1882)	(22,24)	Demotion of *Murgaria* from genus to subgenus, and transfer of species to *Cecropterus*. Tribe addition (Eudamini)
Hesperiidae	*Chioides*	*Chioides albofasciatus*	(Hewitson, 1867)		
Hesperiidae	*Chioides*	*Chioides zilpa*	(Butler, 1872)		
Hesperiidae	*Codatractus*	*Codatractus arizonensis*	(Skinner, 1905)		
Hesperiidae	*Cogia*	*Cogia caicus*	(Herrich-Schäffer, 1869)		
Hesperiidae	*Cogia*	*Cogia calchas*	(Herrich-Schäffer, 1869)		
Hesperiidae	*Cogia*	*Cogia hippalus*	(Edwards, 1882)		
Hesperiidae	*Cogia*	*Cogia outis*	(Skinner, 1894)		
Hesperiidae	*Ectomis*	*Ectomis octomaculata*	(Sepp, 1844)	(22)	*Polythrix* is a junior synonym of *Ectomis*
Hesperiidae	*Epargyreus*	*Epargyreus clarus*	(Cramer, 1775)	(25)	Tribe addition (Eudamini)
Hesperiidae	*Epargyreus*	*Epargyreus huachuca*	Dixon, 1955	(26,27)	Elevation of *Epargyreus clarus huachuca* from subspecies to species
Hesperiidae	*Epargyreus*	*Epargyreus zestos*	(Geyer, 1832)	(26)	Tribe addition (Eudamini)
Hesperiidae	*Lobotractus*	*Lobotractus mysie*	(Dyar, 1904)	(28)	*Lobotractus mysie* is resurrected from synonymy and North American records of *Codatractus valeriana* are now referrable to *Lobotractus mysie*
Hesperiidae	*Phocides*	*Phocides batabano*	(Lucas, 1857)	(24,28)	Elevation of *Phocides pigmalion batabano* from subspecies to species, and transfer of North American records of *Phocides pigmalion* to *Phocides batabano*. Tribe addition (Phocidini)
Hesperiidae	*Phocides*	*Phocides lilea*	(Reakirt, 1867)	(24,27)	Elevation of *Phocides polybius lilea* from subspecies to species, and transfer of North American records to *Phocides liea*. Tribe addition (Phocidini)
Hesperiidae	*Polygonus*	*Polygonus arizonensis*	(Skinner, 1911)	(23)	Elevation of *Polygonus leo arizonensis* from subspecies to species
Hesperiidae	*Polygonus*	*Polygonus leo*	(Gmelin, 1790)		

Hesperiidae	*Spicauda*	*Spicauda atelis*	Grishin, 2023	(20,22)	Transfer of species from *Urbanus* to the new genus *Spicauda*. Transfer of North American records from *Spicauda teleus* to the new species *Spicauda atelis*
Hesperiidae	*Spicauda*	*Spicauda procne*	(Plötz, 1881)	(22)	Transfer of species from *Urbanus* to the new genus *Spicauda*.
Hesperiidae	*Telegonus*	*Telegonus anausis*	Godman & Salvin, 1896	(27)	Elevation of *Telegonus anaphus anausis* from subspecies to species, and transfer of North American records to *Telegonus anausis*
Hesperiidae	*Telegonus*	*Telegonus catemacoensis*	Freeman, 1967	(22)	*Telegonus* is resurrected from synonymy with *Astraptes*. *Telegonus catemacoensis* is resurrected from synonymy with *Telegonus azul* and transfer of North American records of *Astraptes fulgerator* to *Telegonus catemacoensis*
Hesperiidae	*Telegonus*	*Telegonus cellus*	(Boisduval & Le Conte, 1837)	(22)	Transfer of species from *Autochton* to *Telegonus*
Hesperiidae	*Thorybes*	*Thorybes bathyllus*	(Smith, 1797)	(21,22)	Tribe addition (Eudamini)
Hesperiidae	*Thorybes*	*Thorybes confusis*	(Bell, 1923)	(21,22)	Tribe addition (Eudamini)
Hesperiidae	*Thorybes*	*Thorybes diversus*	(Bell, 1927)	(21,22)	Tribe addition (Eudamini)
Hesperiidae	*Thorybes*	*Thorybes lyciades*	(Geyer, 1832)	(21,22,27)	Transfer of species from *Achalarus* to *Thorybes*. Tribe addition (Eudamini)
Hesperiidae	*Thorybes*	*Thorybes nevada*	(Scudder, 1872)	(21,22,27)	Elevation of *Thorybes mexicana nevada* from subspecies to species, and transfer of US records of *Thorybes mexicana* to *Thorybes nevada*
Hesperiidae	*Thorybes*	*Thorybes pylades*	(Scudder, 1870)	(21,22)	Tribe addition (Eudamini)
Hesperiidae	*Urbanus*	*Urbanus proteus*	(Linnaeus, 1758)		
Hesperiidae	*Zestusa*	*Zestusa dorus*	(Edwards, 1882)		
Hesperiidae	*Adopaeoides*	*Adopaeoides prittwitzi*	(Plötz, 1884)		
Hesperiidae	*Agathymus*	*Agathymus alliae*	(Stallings & Turner, 1957)		
Hesperiidae	*Agathymus*	*Agathymus aryxna*	(Dyar, 1905)		
Hesperiidae	*Agathymus*	*Agathymus baueri*	(Stallings & Turner, 1954)		
Hesperiidae	*Agathymus*	*Agathymus chisosensis*	(Freeman, 1952)		
Hesperiidae	*Agathymus*	*Agathymus estelleae*	(Stallings & Turner, 1958)		
Hesperiidae	*Agathymus*	*Agathymus evansi*	(H. Freeman, 1950)		
Hesperiidae	*Agathymus*	*Agathymus gentryi*	Roever, 1998		
Hesperiidae	*Agathymus*	*Agathymus mariae*	(Barnes & Benjamin, 1924)		
Hesperiidae	*Agathymus*	*Agathymus neumoegeni*	(Edwards, 1882)		
Hesperiidae	*Agathymus*	*Agathymus polingi*	(Skinner, 1905)		
Hesperiidae	*Agathymus*	*Agathymus stephensi*	(Skinner, 1912)		
Hesperiidae	*Amblyscirtes*	*Amblyscirtes aenus*	Edwards, 1878		
Hesperiidae	*Amblyscirtes*	*Amblyscirtes aesculapius*	(Fabricius, 1793)		
Hesperiidae	*Amblyscirtes*	*Amblyscirtes alternata*	(Grote & Robinson, 1867)		
Hesperiidae	*Amblyscirtes*	*Amblyscirtes arizonae*	Freeman, 1993		
Hesperiidae	*Amblyscirtes*	*Amblyscirtes belli*	Freeman, 1941		

Hesperiidae	*Amblyscirtes*	*Amblyscirtes carolina*	(Skinner, 1892)		
Hesperiidae	*Amblyscirtes*	*Amblyscirtes cassus*	Edwards, 1883		
Hesperiidae	*Amblyscirtes*	*Amblyscirtes celia*	Skinner, 1895		
Hesperiidae	*Amblyscirtes*	*Amblyscirtes eos*	(Edwards, 1871)		
Hesperiidae	*Amblyscirtes*	*Amblyscirtes exoteria*	(Herrich-Schäffer, 1869)		
Hesperiidae	*Amblyscirtes*	*Amblyscirtes fimbriata*	(Plötz, 1882)		
Hesperiidae	*Amblyscirtes*	*Amblyscirtes hegon*	(Scudder, 1863)		
Hesperiidae	*Amblyscirtes*	*Amblyscirtes linda*	Freeman, 1943		
Hesperiidae	*Amblyscirtes*	*Amblyscirtes nereus*	(Edwards, 1876)		
Hesperiidae	*Amblyscirtes*	*Amblyscirtes nysa*	Edwards, 1877		
Hesperiidae	*Amblyscirtes*	*Amblyscirtes oslari*	(Skinner, 1899)		
Hesperiidae	*Amblyscirtes*	*Amblyscirtes phylace*	(Edwards, 1878)		
Hesperiidae	*Amblyscirtes*	*Amblyscirtes reversa*	Jones, 1926		
Hesperiidae	*Amblyscirtes*	*Amblyscirtes texanae*	Bell, 1927		
Hesperiidae	*Amblyscirtes*	*Amblyscirtes tolteca*	Scudder, 1872		
Hesperiidae	*Amblyscirtes*	*Amblyscirtes vialis*	(Edwards, 1862)		
Hesperiidae	*Anatrytone*	*Anatrytone logan*	(Edwards, 1863)		
Hesperiidae	*Ancyloxypha*	*Ancyloxypha arene*	(Edwards, 1871)		
Hesperiidae	*Ancyloxypha*	*Ancyloxypha numitor*	(Fabricius, 1793)		
Hesperiidae	*Asbolis*	*Asbolis capucinus*	(Lucas, 1857)		
Hesperiidae	*Atalopedes*	*Atalopedes campestris*	(Boisduval, 1852)		
Hesperiidae	*Atrytone*	*Atrytone arogos*	(Boisduval & Le Conte, 1837)		
Hesperiidae	*Atrytone*	*Atrytone bulenta*	(Boisduval & Le Conte, 1837)	(25)	*Probema* is a synonym of *Atrytone*
Hesperiidae	*Atrytone*	*Atrytone byssus*	(Edwards, 1880)	(25)	*Probema* is a synonym of *Atrytone*
Hesperiidae	*Atrytonopsis*	*Atrytonopsis cestus*	(Edwards, 1884)		
Hesperiidae	*Atrytonopsis*	*Atrytonopsis deva*	(Edwards, 1877)		
Hesperiidae	*Atrytonopsis*	*Atrytonopsis edwardsi*	Barnes & McDunnough, 1916		
Hesperiidae	*Atrytonopsis*	*Atrytonopsis hianna*	(Scudder, 1868)		
Hesperiidae	*Atrytonopsis*	*Atrytonopsis loammi*	(Whitney, 1876)		
Hesperiidae	*Atrytonopsis*	*Atrytonopsis lunus*	(Edwards, 1884)		
Hesperiidae	*Atrytonopsis*	*Atrytonopsis margarita*	(Skinner, 1913)		

Hesperiidae	*Atrytonopsis*	*Atrytonopsis pittacus*	(Edwards, 1882)		
Hesperiidae	*Atrytonopsis*	*Atrytonopsis python*	(Edwards, 1882)		
Hesperiidae	*Atrytonopsis*	*Atrytonopsis quinteri*	(Burns, 2015)		
Hesperiidae	*Atrytonopsis*	*Atrytonopsis vierecki*	(Skinner, 1902)		
Hesperiidae	*Calpodes*	*Calpodes ethlius*	(Stoll, 1782)		
Hesperiidae	*Copaeodes*	*Copaeodes edwardsii*	(Barnes, 1897)	(24,25)	Transfer of species from *Oarisma* to *Copaeodes*. Tribe addition (Hesperiini)
Hesperiidae	*Cymaenes*	*Cymaenes tripunctus*	(Herrich-Schäffer, 1865)		
Hesperiidae	*Euphyes*	*Euphyes arpa*	(Boisduval & Le Conte, 1837)		
Hesperiidae	*Euphyes*	*Euphyes bayensis*	Shuey, 1989		
Hesperiidae	*Euphyes*	*Euphyes berryi*	(Bell, 1941)		
Hesperiidae	*Euphyes*	*Euphyes bimacula*	(Grote & Robinson, 1867)		
Hesperiidae	*Euphyes*	*Euphyes conspicua*	(Edwards, 1863)		
Hesperiidae	*Euphyes*	*Euphyes dion*	(Edwards, 1879)		
Hesperiidae	*Euphyes*	*Euphyes dukesi*	(Lindsey, 1923)		
Hesperiidae	*Euphyes*	*Euphyes pilatka*	(Edwards, 1867)		
Hesperiidae	*Euphyes*	*Euphyes vestris*	(Boisduval, 1852)		
Hesperiidae	*Hedone*	*Hedone vibex*	(Geyer, 1832)	(25)	Elevation of *Hedone* to genus level and transfer of species from *Polites* to *Hedone*
Hesperiidae	*Hesperia*	*Hesperia assiniboia*	(Lyman, 1892)		
Hesperiidae	*Hesperia*	*Hesperia attalus*	(Edwards, 1871)		
Hesperiidae	*Hesperia*	*Hesperia balcones*	Grishin, 2023	(20)	Transfer US records of *Hesperia woodgatei* to the new species *Hesperia balcones*
Hesperiidae	*Hesperia*	*Hesperia colorado*	(Scudder, 1874)		
Hesperiidae	*Hesperia*	*Hesperia columbia*	(Scudder, 1872)		
Hesperiidae	*Hesperia*	*Hesperia comma*	(Linnaeus, 1758)		
Hesperiidae	*Hesperia*	*Hesperia dacotae*	(Skinner, 1911)		
Hesperiidae	*Hesperia*	*Hesperia juba*	(Scudder, 1874)		
Hesperiidae	*Hesperia*	*Hesperia leonardus*	Harris, 1862		
Hesperiidae	*Hesperia*	*Hesperia lindseyi*	(Holland, 1930)		
Hesperiidae	*Hesperia*	*Hesperia meskei*	(Edwards, 1877)		
Hesperiidae	*Hesperia*	*Hesperia metea*	Scudder, 1863		
Hesperiidae	*Hesperia*	*Hesperia miriamae*	MacNeill, 1959		
Hesperiidae	*Hesperia*	*Hesperia nevada*	(Scudder, 1874)		
Hesperiidae	*Hesperia*	*Hesperia ottoe*	Edwards, 1866		

Hesperiidae	*Hesperia*	*Hesperia pahaska*	Leussler, 1938		
Hesperiidae	*Hesperia*	*Hesperia sassacus*	Harris, 1862		
Hesperiidae	*Hesperia*	*Hesperia uncas*	Edwards, 1863		
Hesperiidae	*Hesperia*	*Hesperia viridis*	(Edwards, 1883)		
Hesperiidae	*Hesperia*	*Hesperia woodgatei*	(Williams, 1914)		
Hesperiidae	*Hylephila*	*Hylephila phyleus*	(Drury, 1773)		
Hesperiidae	*Lerema*	*Lerema accius*	(Smith, 1797)		
Hesperiidae	*Lerema*	*Lerema liris*	Evans, 1955		
Hesperiidae	*Lerema*	*Lerema ochrius*	Grishin, 2023	(20)	Transfer south and south-east US populations of *Lerema accius* to the new species *Lerema ochrius*
Hesperiidae	*Lerodea*	*Lerodea arabus*	(Edwards, 1882)		
Hesperiidae	*Lerodea*	*Lerodea eufala*	(Edwards, 1869)		
Hesperiidae	*Limochores*	*Limochores sonora*	(Scudder, 1872)	(25)	Elevation of *Limochres* from subgenus to genus and transfer of species from *Polites* to *Limochres*
Hesperiidae	*Lon*	*Lon hobomok*	(Harris, 1862)	(29)	Transfer of species from *Panes* to the new genus *Lon*
Hesperiidae	*Lon*	*Lon melane*	(Edwards, 1869)	(29)	Transfer of species from *Panes* to the new genus *Lon*
Hesperiidae	*Lon*	*Lon taxiles*	(Edwards, 1881)	(29)	Transfer of species from *Panes* to the new genus *Lon*
Hesperiidae	*Lon*	*Lon zabulon*	(Boisduval & Le Conte, 1837)	(29)	Transfer of species from *Panes* to the new genus *Lon*
Hesperiidae	*Megathymus*	*Megathymus cofaqui*	(Strecker, 1876)		
Hesperiidae	*Megathymus*	*Megathymus streckeri*	(Skinner, 1895)		
Hesperiidae	*Megathymus*	*Megathymus ursus*	Poling, 1902		
Hesperiidae	*Megathymus*	*Megathymus yuccae*	(Boisduval & Le Conte, 1837)		
Hesperiidae	*Nastra*	*Nastra julia*	(Freeman, 1945)		
Hesperiidae	*Nastra*	*Nastra lherminier*	(Latreille, 1824)		
Hesperiidae	*Nastra*	*Nastra neamathla*	(Skinner & Williams, 1923)		
Hesperiidae	*Nastra*	*Nastra perigenes*	(Godman, 1900)	(25)	Transfer of species from *Vidius* to *Nastra*
Hesperiidae	*Notamblyscirtes*	*Notamblyscirtes simius*	(Edwards, 1881)		
Hesperiidae	*Nyctelius*	*Nyctelius nyctelius*	(Latreille, 1824)		
Hesperiidae	*Oarisma*	*Oarisma aurantiaca*	(Hewitson, 1868)	(25)	Transfer of species from *Copaeodes* to *Oarisma*
Hesperiidae	*Oarisma*	*Oarisma garita*	(Reakirt, 1866)		
Hesperiidae	*Oarisma*	*Oarisma minima*	(Edwards, 1870)	(25)	Transfer of species from *Copaeodes* to *Oarisma*
Hesperiidae	*Oarisma*	*Oarisma poweshiek*	(Parker, 1870)		
Hesperiidae	*Ochlodes*	*Ochlodes agricola*	(Boisduval, 1852)		
Hesperiidae	*Ochlodes*	*Ochlodes sylvanoides*	(Boisduval, 1852)		

Hesperiidae	*Ochlodes*	*Ochlodes yuma*	(Edwards, 1873)		
Hesperiidae	*Oligoria*	*Oligoria maculata*	(Edwards, 1865)		
Hesperiidae	*Oligoria*	*Oligoria percosius*	(Godman, 1900)	(25)	Transfer of species between genera, from *Decinea* to *Oligoria*
Hesperiidae	*Panoquina*	*Panoquina errans*	(Skinner, 1892)		
Hesperiidae	*Panoquina*	*Panoquina hecebolus*	(Scudder, 1872)		
Hesperiidae	*Panoquina*	*Panoquina ocola*	(Edwards, 1863)		
Hesperiidae	*Panoquina*	*Panoquina panoquin*	(Scudder, 1863)		
Hesperiidae	*Panoquina*	*Panoquina panoquinoides*	(Skinner, 1891)		
Hesperiidae	*Paratrytone*	*Paratrytone snowi*	(Edwards, 1877)		
Hesperiidae	*Poanes*	*Poanes aaroni*	(Skinner, 1890)		
Hesperiidae	*Poanes*	*Poanes massasoit*	(Scudder, 1863)		
Hesperiidae	*Poanes*	*Poanes viator*	(Edwards, 1865)		
Hesperiidae	*Poanes*	*Poanes yehl*	(Skinner, 1893)		
Hesperiidae	*Polites*	*Polites baracoa*	(Lucas, 1857)		
Hesperiidae	*Polites*	*Polites carus*	(Edwards, 1883)		
Hesperiidae	*Polites*	*Polites draco*	(Edwards, 1871)		
Hesperiidae	*Polites*	*Polites egeremet*	(Scudder, 1863)	(25)	Demotion of *Wallengrenia* from genus to subgenus, and transfer of species to *Polites*
Hesperiidae	*Polites*	*Polites mardon*	(Edwards, 1881)		
Hesperiidae	*Polites*	*Polites mystic*	(Edwards, 1863)		
Hesperiidae	*Polites*	*Polites origenes*	(Fabricius, 1793)		
Hesperiidae	*Polites*	*Polites otho*	(Smith, 1797)	(25)	Demotion of *Wallengrenia* from genus to subgenus, and transfer of species to *Polites*
Hesperiidae	*Polites*	*Polites peckius*	(Edwards, 1870)		
Hesperiidae	*Polites*	*Polites rhesus*	(Edwards, 1878)		
Hesperiidae	*Polites*	*Polites sabuleti*	(Boisduval, 1852)		
Hesperiidae	*Polites*	*Polites themistocles*	(Latreille, 1824)		
Hesperiidae	*Pseudocopaeodes*	*Pseudocopaeodes eunus*	(Edwards, 1881)		
Hesperiidae	*Quasimellana*	*Quasimellana eulogius*	(Plötz, 1882)		
Hesperiidae	*Stallingsia*	*Stallingsia maculosus*	(Freeman, 1955)	(22)	Tribe addition (Megathymini)
Hesperiidae	*Stinga*	*Stinga morrisoni*	(Edwards, 1878)		
Hesperiidae	*Synapte*	*Synapte pecta*	Evans, 1955		
Hesperiidae	*Thymelicus*	*Thymelicus lineola*	(Ochsenheimer, 1808)		
Hesperiidae	*Vernia*	*Vernia verna*	(Edwards, 1862)	(29)	Transfer of species from *Pompeius* to the new genus *Vernia*

Hesperiidae	*Carterocephalus*	*Carterocephalus mandan*	(Edwards, 1863)	(27)	Elevation of subspecies *Carterocephalus palaemon mandan* to the species *Carterocephalus mandan*
Hesperiidae	*Carterocephalus*	*Carterocephalus skada*	Edwards, 1870	(27)	Elevation of *Carterocephalus palaemon skada* from subspecies to species
Hesperiidae	*Piruna*	*Piruna aea*	(Dyar, 1912)		
Hesperiidae	*Piruna*	*Piruna haferniki*	Freeman, 1970		
Hesperiidae	*Piruna*	*Piruna pirus*	(Edwards, 1878)		
Hesperiidae	*Piruna*	*Piruna polingii*	(Barnes, 1900)		
Hesperiidae	*Arteurotia*	*Arteurotia artistella*	Grishin, 2023	(20)	Tansfer of US records of *Arteurotia tractipennis* to the new species *Arteurotia artistella*
Hesperiidae	*Burnsius*	*Burnsius albescens*	(Plötz, 1884)	(22)	Transfer of species from *Pyrgus* to the new genus *Burnsius*
Hesperiidae	*Burnsius*	*Burnsius communis*	(Grote, 1872)	(22)	Transfer of species from *Pyrgus* to the new genus *Burnsius*
Hesperiidae	*Burnsius*	*Burnsius oileus*	(Linnaeus, 1767)	(22)	Transfer of species from *Pyrgus* to the new genus *Burnsius*
Hesperiidae	*Burnsius*	*Burnsius philetas*	(Edwards, 1881)	(22)	Transfer of species from *Pyrgus* to the new genus *Burnsius*
Hesperiidae	*Canesia*	*Canesia canescens*	(Felder, 1869)	(29)	Transfer of species from *Carrhenes* to new genus *Canesia*
Hesperiidae	*Celotes*	*Celotes limpia*	Burns, 1974		
Hesperiidae	*Celotes*	*Celotes nessus*	(Edwards, 1877)		
Hesperiidae	*Chiothion*	*Chiothion georgina*	(Reakirt, 1868)	(29)	Transfer of species from *Chiomara* to the new genus *Chiotion*
Hesperiidae	*Clytius*	*Clytius clytius*	(Godman & Salvin, 1897)	(29)	Transfer of species from *Bolla* to the new genus *Clytius*
Hesperiidae	*Eantis*	*Eantis tamenund*	(Edwards, 1871)		
Hesperiidae	*Ephyriades*	*Ephyriades brunnea*	(Herrich-Schäffer, 1865)		
Hesperiidae	*Erynnis*	*Erynnis afranius*	(Lintner, 1878)	(24,29)	Maintain existing usage of genus as advocated by Pelham (2022), despite Cong et al. (2019)
Hesperiidae	*Erynnis*	*Erynnis baptisiae*	(Forbes, 1936)	(24,29)	Maintain existing usage of genus as advocated by Pelham (2022), despite Cong et al. (2019)
Hesperiidae	*Erynnis*	*Erynnis brizo*	(Boisduval & Le Conte, 1837)		
Hesperiidae	*Erynnis*	*Erynnis burgessi*	(Skinner, 1914)	(30)	Elevation of the subspecies *Erynnis brizo burgessi* to species
Hesperiidae	*Erynnis*	*Erynnis funeralis*	(Scudder & Burgess, 1870)	(24,29)	Maintain existing usage of genus as advocated by Pelham (2022), despite Cong et al. (2019)
Hesperiidae	*Erynnis*	*Erynnis horatius*	(Scudder & Burgess, 1870)	(24,29)	Maintain existing usage of genus as advocated by Pelham (2022), despite Cong et al. (2019)
Hesperiidae	*Erynnis*	*Erynnis icelus*	(Scudder & Burgess, 1870)		
Hesperiidae	*Erynnis*	*Erynnis juvenalis*	(Fabricius, 1793)	(24,29)	Maintain existing usage of genus as advocated by Pelham (2022), despite Cong et al. (2019)
Hesperiidae	*Erynnis*	*Erynnis lacustra*	(Wright, 1905)	(30)	Elevation of the subspecies *Erynnis brizo laustra* to species
Hesperiidae	*Erynnis*	*Erynnis lucilius*	(Scudder & Burgess, 1870)	(24,29)	Maintain existing usage of genus as advocated by Pelham (2022), despite Cong et al. (2019)
Hesperiidae	*Erynnis*	*Erynnis martialis*	(Scudder, 1869)	(24,29)	Maintain existing usage of genus as advocated by Pelham (2022), despite Cong et al. (2019)
Hesperiidae	*Erynnis*	*Erynnis meridianus*	Bell, 1927	(24,29)	Maintain existing usage of genus as advocated by Pelham (2022), despite Cong et al. (2019)
Hesperiidae	*Erynnis*	*Erynnis pacuvius*	(Lintner, 1878)	(24,29)	Maintain existing usage of genus as advocated by Pelham (2022), despite Cong et al. (2019)
Hesperiidae	*Erynnis*	*Erynnis persius*	(Scudder, 1863)	(24,29)	Maintain existing usage of genus as advocated by Pelham (2022), despite Cong et al. (2019)

Hesperiidae	*Erynnis*	*Erynnis propertius*	(Scudder & Burgess, 1870)	(24,29)	Maintain existing usage of genus as advocated by Pelham (2022), despite Cong et al. (2019)
Hesperiidae	*Erynnis*	*Erynnis scudderi*	(Skinner, 1914)	(24,29)	Maintain existing usage of genus as advocated by Pelham (2022), despite Cong et al. (2019)
Hesperiidae	*Erynnis*	*Erynnis somnus*	(Lintner, 1881)	(30)	Elevation of the subspecies *Erynnis brizo somnus* to species
Hesperiidae	*Erynnis*	*Erynnis telemachus*	Burns, 1960	(24,29)	Maintain existing usage of genus as advocated by Pelham (2022), despite Cong et al. (2019)
Hesperiidae	*Erynnis*	*Erynnis tristis*	(Boisduval, 1852)	(24,29)	Maintain existing usage of genus as advocated by Pelham (2022), despite Cong et al. (2019)
Hesperiidae	*Erynnis*	*Erynnis zarucco*	(Lucas, 1857)	(24,29)	Maintain existing usage of genus as advocated by Pelham (2022), despite Cong et al. (2019)
Hesperiidae	*Gesta*	*Gesta invisus*	(Butler & Druce, 1872)		
Hesperiidae	*Heliopetes*	*Heliopetes domicella*	(Erichson, 1849)	(22)	Demotion of *Heliopyrgus* from genus to subgenus and transfer of species to *Heliopetes*
Hesperiidae	*Heliopetes*	*Heliopetes elonmuski*	Grishin, 2023		North American records of *Heliopetes arsalte* should be transferred to the new species *Heliopetes elonmuski*
Hesperiidae	*Heliopetes*	*Heliopetes ericetorum*	(Boisduval, 1852)		
Hesperiidae	*Heliopetes*	*Heliopetes laviana*	(Hewitson, 1868)		
Hesperiidae	*Heliopetes*	*Heliopetes macaira*	(Reakirt, 1867)		
Hesperiidae	*Heliopetes*	*Heliopetes sublinea*	(Schaus, 1902)	(22)	Demotion of *Heliopyrgus* from genus to subgenus and transfer of species to *Heliopetes*
Hesperiidae	*Hesperopsis*	*Hesperopsis alpheus*	(Edwards, 1876)		
Hesperiidae	*Hesperopsis*	*Hesperopsis gracielae*	(MacNeill, 1970)		
Hesperiidae	*Hesperopsis*	*Hesperopsis libya*	(Scudder, 1878)		
Hesperiidae	*Pellicia*	*Pellicia arina*	Evans, 1953		
Hesperiidae	*Pholisora*	*Pholisora catullus*	(Fabricius, 1793)		
Hesperiidae	*Pholisora*	*Pholisora mejicanus*	(Reakirt, 1867)		
Hesperiidae	*Pyrgus*	*Pyrgus centaureae*	(Rambur, 1842)		
Hesperiidae	*Pyrgus*	*Pyrgus ruralis*	(Boisduval, 1852)		
Hesperiidae	*Pyrgus*	*Pyrgus scriptura*	(Boisduval, 1852)		
Hesperiidae	*Pyrgus*	*Pyrgus xanthus*	Edwards, 1878		
Hesperiidae	*Staphylus*	*Staphylus ceos*	(Edwards, 1882)		
Hesperiidae	*Staphylus*	*Staphylus hayhurstii*	(Edwards, 1870)		
Hesperiidae	*Staphylus*	*Staphylus mazans*	(Reakirt, 1867)		
Hesperiidae	*Systasea*	*Systasea pulverulenta*	(Felder, 1869)		
Hesperiidae	*Systasea*	*Systasea zampa*	(Edwards, 1876)		
Hesperiidae	*Timochares*	*Timochares ruptifasciata*	(Plötz, 1884)		
Hesperiidae	*Apyrrothrix*	*Apyrrothrix araxes*	(Hewitson, 1867)		
Lycaenidae	*Lycaena*	*Lycaena cupreus*	(Edwards, 1870)	(24)	Tribe addition (Lycaenini)
Lycaenidae	*Lycaena*	*Lycaena phlaeas*	Linnaeus, 1761	(24)	Tribe addition (Lycaenini)

Lycaenidae	*Tharsalea*	*Tharsalea arota*	(Boisduval, 1852)	(27)	Elevation of *Tharsalea* from subgenus to genus, and transfer of species from *Lycaena* to *Tharsalea*
Lycaenidae	*Tharsalea*	*Tharsalea dione*	(Scudder, 1868)	(27)	Elevation of *Tharsalea* from subgenus to genus, and transfer of species from *Lycaena* to *Tharsalea*
Lycaenidae	*Tharsalea*	*Tharsalea dorcas*	(Kirby, 1837)	(27)	Elevation of *Tharsalea* from subgenus to genus, and transfer of species from *Lycaena* to *Tharsalea*
Lycaenidae	*Tharsalea*	*Tharsalea dospassosi*	(McDunnough, 1940)	(27)	Elevation of *Tharsalea* from subgenus to genus, and transfer of species from *Lycaena* to *Tharsalea*
Lycaenidae	*Tharsalea*	*Tharsalea editha*	(Mead, 1878)	(27)	Elevation of *Tharsalea* from subgenus to genus, and transfer of species from *Lycaena* to *Tharsalea*
Lycaenidae	*Tharsalea*	*Tharsalea epixanthe*	(Boisduval & Le Conte, 1835)	(27)	Elevation of *Tharsalea* from subgenus to genus, and transfer of species from *Lycaena* to *Tharsalea*
Lycaenidae	*Tharsalea*	*Tharsalea gorgon*	(Boisduval, 1852)	(27)	Elevation of *Tharsalea* from subgenus to genus, and transfer of species from *Lycaena* to *Tharsalea*
Lycaenidae	*Tharsalea*	*Tharsalea helloides*	(Boisduval, 1852)	(27)	Elevation of *Tharsalea* from subgenus to genus, and transfer of species from *Lycaena* to *Tharsalea*
Lycaenidae	*Tharsalea*	*Tharsalea hermes*	(Edwards, 1870)	(27)	Elevation of *Tharsalea* from subgenus to genus, and transfer of species from *Lycaena* to *Tharsalea*
Lycaenidae	*Tharsalea*	*Tharsalea heteronea*	(Boisduval, 1852)	(27)	Elevation of *Tharsalea* from subgenus to genus, and transfer of species from *Lycaena* to *Tharsalea*
Lycaenidae	*Tharsalea*	*Tharsalea hyllus*	(Cramer, 1775)	(27)	Elevation of *Tharsalea* from subgenus to genus, and transfer of species from *Lycaena* to *Tharsalea*
Lycaenidae	*Tharsalea*	*Tharsalea mariposa*	(Reakirt, 1866)	(27)	Elevation of *Tharsalea* from subgenus to genus, and transfer of species from *Lycaena* to *Tharsalea*
Lycaenidae	*Tharsalea*	*Tharsalea nivalis*	(Boisduval, 1869)	(27)	Elevation of *Tharsalea* from subgenus to genus, and transfer of species from *Lycaena* to *Tharsalea*
Lycaenidae	*Tharsalea*	*Tharsalea rubidus*	(Behr, 1866)	(27)	Elevation of *Tharsalea* from subgenus to genus, and transfer of species from *Lycaena* to *Tharsalea*
Lycaenidae	*Tharsalea*	*Tharsalea xanthoides*	(Boisduval, 1852)	(27)	Elevation of *Tharsalea* from subgenus to genus, and transfer of species from *Lycaena* to *Tharsalea*
Lycaenidae	*Feniseca*	*Feniseca tarquinius*	(Fabricius, 1793)	(24)	Tribe addition (Miletini)
Lycaenidae	*Agriades*	*Agriades glandon*	(de Prunner, 1798)	(24)	Tribe addition (Polyommatini)
Lycaenidae	*Agriades*	*Agriades optilete*	(Knoch, 1781)	(24)	Tribe addition (Polyommatini)
Lycaenidae	*Agriades*	*Agriades podarce*	(Felder & Felder, 1865)	(24)	Tribe addition (Polyommatini)
Lycaenidae	*Brephidium*	*Brephidium exilis*	(Boisduval, 1852)	(24)	Tribe addition (Polyommatini)
Lycaenidae	*Brephidium*	*Brephidium pseudofea*	(Morrison, 1873)	(24)	Tribe addition (Polyommatini)
Lycaenidae	*Celastrina*	*Celastrina echo*	(Edwards, 1864)	(24)	Tribe addition (Polyommatini)
Lycaenidae	*Celastrina*	*Celastrina humulus*	Scott & Wright, 1998	(24)	Tribe addition (Polyommatini)
Lycaenidae	*Celastrina*	*Celastrina idella*	Wright & Pavulaan, 1999	(24)	Tribe addition (Polyommatini)
Lycaenidae	*Celastrina*	*Celastrina ladon*	(Cramer, 1780)	(24)	Tribe addition (Polyommatini)
Lycaenidae	*Celastrina*	*Celastrina lucia*	(Kirby, 1837)	(24)	Tribe addition (Polyommatini)
Lycaenidae	*Celastrina*	*Celastrina neglecta*	(Edwards, 1862)	(24)	Tribe addition (Polyommatini)
Lycaenidae	*Celastrina*	*Celastrina neglectamajor*	Tutt, 1908	(24)	Tribe addition (Polyommatini)
Lycaenidae	*Celastrina*	*Celastrina nigra*	(Forbes, 1960)	(24)	Tribe addition (Polyommatini)
Lycaenidae	*Celastrina*	*Celastrina serotina*	Pavulaan & Wright, 2005	(24)	Tribe addition (Polyommatini)
Lycaenidae	*Cupido*	*Cupido amyntula*	(Boisduval, 1852)	(24)	Tribe addition (Polyommatini)
Lycaenidae	*Cupido*	*Cupido comyntas*	(Godart, 1824)	(24)	Tribe addition (Polyommatini)

Lycaenidae	*Cyclargus*	*Cyclargus ammon*	(Lucas, 1857)	(24)	Tribe addition (Polyommatini)
Lycaenidae	*Cyclargus*	*Cyclargus thomasi*	(Clench, 1941)	(24)	Tribe addition (Polyommatini)
Lycaenidae	*Echinargus*	*Echinargus isola*	(Reakirt, 1867)	(24)	Tribe addition (Polyommatini)
Lycaenidae	*Euphilotes*	*Euphilotes allyni*	(Shields, 1975)	(24,31,32)	Elevation of *Euphilotes bernardino allyni* to *Euphilotes allyni*. Tribe addition (Polyommatini)
Lycaenidae	*Euphilotes*	*Euphilotes ancilla*	(Barnes & McDunnough, 1918)	(24)	Tribe addition (Polyommatini)
Lycaenidae	*Euphilotes*	*Euphilotes battoides*	(Behr, 1867)	(24)	Tribe addition (Polyommatini)
Lycaenidae	*Euphilotes*	*Euphilotes baueri*	(Shields, 1975)	(24)	Tribe addition (Polyommatini)
Lycaenidae	*Euphilotes*	*Euphilotes bernardino*	(Barnes & McDunnough, 1916)	(24)	Tribe addition (Polyommatini)
Lycaenidae	*Euphilotes*	*Euphilotes centralis*	(Barnes & McDunnough, 1917)	(24)	Tribe addition (Polyommatini)
Lycaenidae	*Euphilotes*	*Euphilotes columbiae*	(Mattoni, 1954)	(24)	Tribe addition (Polyommatini)
Lycaenidae	*Euphilotes*	*Euphilotes ellisii*	(Shields, 1975)	(24)	Tribe addition (Polyommatini)
Lycaenidae	*Euphilotes*	*Euphilotes enoptes*	(Boisduval, 1852)	(24)	Tribe addition (Polyommatini)
Lycaenidae	*Euphilotes*	*Euphilotes glaucon*	(Edwards, 1871)	(24)	Tribe addition (Polyommatini)
Lycaenidae	*Euphilotes*	*Euphilotes heracleoides*	Kohler & A. Warren, 2021	(24,33)	New species. Tribe addition (Polyommatini)
Lycaenidae	*Euphilotes*	*Euphilotes leona*	Hammond & McCorkle, 2000	(24,25)	Demotion of *Philotiella* from genus to subgenus of *Euphilotes*. Tribe addition (Polyommatini)
Lycaenidae	*Euphilotes*	*Euphilotes mojave*	(Watson & Comstock, 1920)	(24)	Tribe addition (Polyommatini)
Lycaenidae	*Euphilotes*	*Euphilotes oakleyi*	Kohler, 2021	(24,33)	Demotion of *Philotiella* from genus to subgenus of *Euphilotes*. Tribe addition (Polyommatini)
Lycaenidae	*Euphilotes*	*Euphilotes pallescens*	(Tilden & Downey, 1955)	(24)	Tribe addition (Polyommatini)
Lycaenidae	*Euphilotes*	*Euphilotes rita*	(Barnes & McDunnough, 1916)	(24)	Tribe addition (Polyommatini)
Lycaenidae	*Euphilotes*	*Euphilotes spaldingi*	(Barnes & McDunnough, 1917)	(24)	Tribe addition (Polyommatini)
Lycaenidae	*Euphilotes*	*Euphilotes speciosa*	Edwards, 1877	(24,25)	Demotion of *Philotiella* from genus to subgenus of *Euphilotes*. Tribe addition (Polyommatini)
Lycaenidae	*Euphilotes*	*Euphilotes stanfordorum*	Opler & A. Warren, 2009	(24)	Tribe addition (Polyommatini)
Lycaenidae	*Glaucopsyche*	*Glaucopsyche lygdamus*	(Doubleday, 1841)	(24)	Tribe addition (Polyommatini)
Lycaenidae	*Glaucopsyche*	*Glaucopsyche piasus*	(Boisduval, 1852)	(24)	Tribe addition (Polyommatini)
Lycaenidae	*Hemiargus*	*Hemiargus ceraunus*	(Fabricius, 1793)	(24)	Tribe addition (Polyommatini)
Lycaenidae	*Icaricia*	*Icaricia acmon*	(Westwood, 1851)	(24)	Tribe addition (Polyommatini)
Lycaenidae	*Icaricia*	*Icaricia chlorina*	(Skinner, 1902)	(24,31)	Elevation of *Icaricia lupini chlorina* from subspecies to species. Tribe addition (Polyommatini)
Lycaenidae	*Icaricia*	*Icaricia cotundra*	(Scott & Fisher, 2006)	(24)	Tribe addition (Polyommatini)
Lycaenidae	*Icaricia*	*Icaricia dedeckera*	Emmel, Emmel & Mattoon, 1998	(24,31)	Elevation of *Icaricia lupini dedeckera* from subspeies to species. Tribe addition (Polyommatini)
Lycaenidae	*Icaricia*	*Icaricia emigdionis*	Grinnell, 1905	(20,24)	Demotion of *Plebulina* to subgenus and transfer of species to *Icaricia*. Tribe addition (Polyommatini)

Lycaenidae	*Icaricia*	*Icaricia icarioides*	(Boisduval, 1852)	(24)	Tribe addition (Polyommatini)
Lycaenidae	*Icaricia*	*Icaricia lupini*	(Boisduval, 1869)	(24)	Tribe addition (Polyommatini)
Lycaenidae	*Icaricia*	*Icaricia monticola*	(Clemence, 1909)	(24,31)	Elevation of *Icaricia lupini monticola* from subspecies to species. Tribe addition (Polyommatini)
Lycaenidae	*Icaricia*	*Icaricia neurona*	(Skinner, 1902)	(24)	Tribe addition (Polyommatini)
Lycaenidae	*Icaricia*	*Icaricia saepiolus*	(Boisduval, 1852)	(24)	Tribe addition (Polyommatini)
Lycaenidae	*Icaricia*	*Icaricia shasta*	(Edwards, 1862)	(24)	Tribe addition (Polyommatini)
Lycaenidae	*Leptotes*	*Leptotes cassius*	(Cramer, 1775)	(24)	Tribe addition (Polyommatini)
Lycaenidae	*Leptotes*	*Leptotes marina*	(Reakirt, 1868)	(24)	Tribe addition (Polyommatini)
Lycaenidae	*Philotes*	*Philotes sonorensis*	(Felder & Felder, 1865)	(24)	Tribe addition (Polyommatini)
Lycaenidae	*Plebejus*	*Plebejus anna*	(Edwards, 1861)	(24)	Tribe addition (Polyommatini)
Lycaenidae	*Plebejus*	*Plebejus fridayi*	Chermock, 1945	(24)	Tribe addition (Polyommatini)
Lycaenidae	*Plebejus*	*Plebejus idas*	(Linnaeus, 1760)	(24)	Tribe addition (Polyommatini)
Lycaenidae	*Plebejus*	*Plebejus melissa*	(Edwards, 1873)	(24)	Tribe addition (Polyommatini)
Lycaenidae	*Plebejus*	*Plebejus samuelis*	(Nabokov, 1944)	(24)	Tribe addition (Polyommatini)
Lycaenidae	*Polyommatus*	*Polyommatus icarus*	(Rottemburg, 1775)	(24)	Tribe addition (Polyommatini)
Lycaenidae	*Zizula*	*Zizula cyna*	(Edwards, 1881)	(24)	Tribe addition (Polyommatini)
Lycaenidae	*Atlides*	*Atlides halesus*	(Cramer, 1777)		
Lycaenidae	*Callophrys*	*Callophrys affinis*	(Edwards, 1862)	(24)	Tribe addition (Riodinini)
Lycaenidae	*Callophrys*	*Callophrys augustinus*	(Westwood, 1852)	(24)	Tribe addition (Riodinini)
Lycaenidae	*Callophrys*	*Callophrys dumetorum*	(Boisduval, 1852)	(24)	Tribe addition (Riodinini)
Lycaenidae	*Callophrys*	*Callophrys eryphon*	(Boisduval, 1852)	(24)	Tribe addition (Riodinini)
Lycaenidae	*Callophrys*	*Callophrys fotis*	(Strecker, 1878)	(24)	Tribe addition (Riodinini)
Lycaenidae	*Callophrys*	*Callophrys gryneus*	(Hübner, 1819)	(24)	Tribe addition (Riodinini)
Lycaenidae	*Callophrys*	*Callophrys henrici*	(Grote & Robinson, 1867)	(24)	Tribe addition (Riodinini)
Lycaenidae	*Callophrys*	*Callophrys hesseli*	(Rawson & Ziegler, 1950)	(24)	Tribe addition (Riodinini)
Lycaenidae	*Callophrys*	*Callophrys irus*	(Godart, 1824)	(24)	Tribe addition (Riodinini)
Lycaenidae	*Callophrys*	*Callophrys johnsoni*	(Skinner, 1904)	(24)	Tribe addition (Riodinini)
Lycaenidae	*Callophrys*	*Callophrys lanoraieensis*	(Sheppard, 1934)	(24)	Tribe addition (Riodinini)
Lycaenidae	*Callophrys*	*Callophrys loki*	(Skinner, 1907)	(24)	Tribe addition (Riodinini)
Lycaenidae	*Callophrys*	*Callophrys mcfarlandi*	Ehrlich & Clench, 1960	(24)	Tribe addition (Riodinini)
Lycaenidae	*Callophrys*	*Callophrys mossii*	(Edwards, 1881)	(24)	Tribe addition (Riodinini)
Lycaenidae	*Callophrys*	*Callophrys muiri*	(Edwards, 1881)	(24)	Tribe addition (Riodinini)

Lycaenidae	*Callophrys*	*Callophrys niphon*	(Hübner, 1819)	(24)	Tribe addition (Riodinini)
Lycaenidae	*Callophrys*	*Callophrys polios*	(Cook & F. Watson, 1907)	(24)	Tribe addition (Riodinini)
Lycaenidae	*Callophrys*	*Callophrys sheridanii*	(Edwards, 1877)	(24)	Tribe addition (Riodinini)
Lycaenidae	*Callophrys*	*Callophrys spinetorum*	(Hewitson, 1867)	(24)	Tribe addition (Riodinini)
Lycaenidae	*Callophrys*	*Callophrys viridis*	(Edwards, 1862)	(24)	Tribe addition (Riodinini)
Lycaenidae	*Callophrys*	*Callophrys xami*	(Reakirt, 1867)	(24)	Tribe addition (Riodinini)
Lycaenidae	*Calycopis*	*Calycopis cecrops*	(Fabricius, 1793)		
Lycaenidae	*Calycopis*	*Calycopis isobeon*	(Butler & H. Druce, 1872)		
Lycaenidae	*Chlorostrymon*	*Chlorostrymon maesites*	(Herrich-Schäffer, 1865)	(24)	Tribe addition (Eumaeini)
Lycaenidae	*Chlorostrymon*	*Chlorostrymon simaethis*	(Drury, 1773)	(24)	Tribe addition (Eumaeini)
Lycaenidae	*Cyanophrys*	*Cyanophrys miserabilis*	(Clench, 1946)		
Lycaenidae	*Electrostrymon*	*Electrostrymon angelia*	(Hewitson, 1874)	(24)	Tribe addition (Eumaeini)
Lycaenidae	*Erora*	*Erora laeta*	(Edwards, 1862)		
Lycaenidae	*Erora*	*Erora quaderna*	(Hewitson, 1868)		
Lycaenidae	*Eumaeus*	*Eumaeus atala*	(Poey, 1832)		
Lycaenidae	*Habrodais*	*Habrodais grunus*	(Boisduval, 1852)	(24)	Tribe addition (Theclini)
Lycaenidae	*Hypaurotis*	*Hypaurotis crysalus*	(Edwards, 1873)		
Lycaenidae	*Ministrymon*	*Ministrymon clytie*	(Edwards, 1877)		
Lycaenidae	*Ministrymon*	*Ministrymon janevicroy*	Glassberg, 2013		
Lycaenidae	*Ministrymon*	*Ministrymon leda*	(Edwards, 1882)		
Lycaenidae	*Parrhasius*	*Parrhasius m-album*	(Boisduval & Le Conte, 1833)		
Lycaenidae	*Pendantus*	*Pendantus guzanta*	(Schaus, 1902)	(25)	Resurrected synonymy of *Pendantus guzanta*, and transfer of species from *Electrostrymon guzanta* to *Pendantus guzanta*
Lycaenidae	*Satyrium*	*Satyrium acadica*	(Edwards, 1862)	(24)	Tribe addition (Eumaeini)
Lycaenidae	*Satyrium*	*Satyrium alcestis*	(Edwards, 1871)	(24,25)	*Phaeostrymon* is now a junior synonym of *Satyrium*, and transfer of species from *Phaeostrymon* to *Satyrium*. Tribe addition (Eumaeini)
Lycaenidae	*Satyrium*	*Satyrium auretorum*	(Boisduval, 1852)	(24)	Tribe addition (Eumaeini)
Lycaenidae	*Satyrium*	*Satyrium behrii*	(Edwards, 1870)	(24)	Tribe addition (Eumaeini)
Lycaenidae	*Satyrium*	*Satyrium calanus*	(Hübner, 1809)	(24)	Tribe addition (Eumaeini)
Lycaenidae	*Satyrium*	*Satyrium californica*	(Edwards, 1862)	(24)	Tribe addition (Eumaeini)
Lycaenidae	*Satyrium*	*Satyrium caryaevorus*	(McDunnough, 1942)	(24)	Tribe addition (Eumaeini)
Lycaenidae	*Satyrium*	*Satyrium edwardsii*	(Grote & Robinson, 1867)	(24)	Tribe addition (Eumaeini)
Lycaenidae	*Satyrium*	*Satyrium favonius*	(Smith, 1797)	(24)	Tribe addition (Eumaeini)

Lycaenidae	*Satyrium*	*Satyrium fuliginosa*	(Edwards, 1861)	(24)	Tribe addition (Eumaeini)
Lycaenidae	*Satyrium*	*Satyrium ilavia*	(Beutenmüller, 1899)	(24)	Tribe addition (Eumaeini)
Lycaenidae	*Satyrium*	*Satyrium kingi*	(Klots & Clench, 1952)	(24)	Tribe addition (Eumaeini)
Lycaenidae	*Satyrium*	*Satyrium liparops*	(Le Conte, 1833)	(24)	Tribe addition (Eumaeini)
Lycaenidae	*Satyrium*	*Satyrium polingi*	(Barnes & Benjamin, 1926)	(24)	Tribe addition (Eumaeini)
Lycaenidae	*Satyrium*	*Satyrium saepium*	(Boisduval, 1852)	(24)	Tribe addition (Eumaeini)
Lycaenidae	*Satyrium*	*Satyrium semiluna*	Klots, 1930	(24)	Tribe addition (Eumaeini)
Lycaenidae	*Satyrium*	*Satyrium sylvinus*	Boisduval, 1852	(24)	Tribe addition (Eumaeini)
Lycaenidae	*Satyrium*	*Satyrium tetra*	(Edwards, 1870)	(24)	Tribe addition (Eumaeini)
Lycaenidae	*Satyrium*	*Satyrium titus*	(Fabricius, 1793)	(24)	Tribe addition (Eumaeini)
Lycaenidae	*Strymon*	*Strymon acis*	(Drury, 1773)		
Lycaenidae	*Strymon*	*Strymon alea*	(Godman & Salvin, 1887)		
Lycaenidae	*Strymon*	*Strymon avalona*	(Wright, 1905)		
Lycaenidae	*Strymon*	*Strymon bazochii*	(Godart, 1824)		
Lycaenidae	*Strymon*	*Strymon istapa*	(Reakirt, 1867)		
Lycaenidae	*Strymon*	*Strymon limenia*	(Hewitson, 1868)		
Lycaenidae	*Strymon*	*Strymon martialis*	(Herrich-Schäffer, 1865)		
Lycaenidae	*Strymon*	*Strymon melinus*	Hübner, 1818		
Lycaenidae	*Strymon*	*Strymon rufofusca*	(Hewitson, 1877)		
Lycaenidae	*Strymon*	*Strymon yojoa*	(Reakirt, 1867)		
Nymphalidae	*Asterocampa*	*Asterocampa celtis*	(Boisduval & Le Conte, 1835)		
Nymphalidae	*Asterocampa*	*Asterocampa clyton*	(Boisduval & Le Conte, 1835)		
Nymphalidae	*Asterocampa*	*Asterocampa leilia*	(Edwards, 1874)		
Nymphalidae	*Biblis*	*Biblis aganisa*	Boisduval, 1836	(24,34)	Elevation of *Biblis hyperia aganisa* from the subspecies to species. Tribe addition (Biblidini)
Nymphalidae	*Eunica*	*Eunica tatila*	(Herrich-Schäffer, 1855)	(24)	Tribe addition (Catonephelini)
Nymphalidae	*Mestra*	*Mestra amymone*	(Ménétriés, 1857)	(24)	Tribe addition (Biblidini)
Nymphalidae	*Myscelia*	*Myscelia ethusa*	(Doyère, 1840)		
Nymphalidae	*Anaea*	*Anaea aidea*	(Guérin-Méneville, 1844)		
Nymphalidae	*Anaea*	*Anaea andria*	Scudder, 1875		
Nymphalidae	*Anaea*	*Anaea troglodyta*	(Fabricius, 1775)		
Nymphalidae	*Memphis*	*Memphis pithyusa*	(Felder, 1869)		
Nymphalidae	*Marpesia*	*Marpesia petreus*	(Cramer, 1776)		

Nymphalidae	*Danaus*	*Danaus eresimus*	(Cramer, 1777)		
Nymphalidae	*Danaus*	*Danaus gilippus*	(Cramer, 1775)		
Nymphalidae	*Danaus*	*Danaus plexippus*	(Linnaeus, 1758)		
Nymphalidae	*Boloria*	*Boloria alaskensis*	(Holland, 1900)		
Nymphalidae	*Boloria*	*Boloria alberta*	(Edwards, 1890)		
Nymphalidae	*Boloria*	*Boloria astarte*	(Doubleday, 1847)		
Nymphalidae	*Boloria*	*Boloria bellona*	(Fabricius, 1775)		
Nymphalidae	*Boloria*	*Boloria chariclea*	(Schneider, 1794)		
Nymphalidae	*Boloria*	*Boloria epithore*	(Edwards, 1864)		
Nymphalidae	*Boloria*	*Boloria eunomia*	(Esper, 1800)		
Nymphalidae	*Boloria*	*Boloria freija*	(Thunberg, 1791)		
Nymphalidae	*Boloria*	*Boloria frigga*	(Thunberg, 1791)		
Nymphalidae	*Boloria*	*Boloria improba*	(Butler, 1877)		
Nymphalidae	*Boloria*	*Boloria kriemhild*	(Strecker, 1879)		
Nymphalidae	*Boloria*	*Boloria natazhati*	(Gibson, 1920)		
Nymphalidae	*Boloria*	*Boloria polaris*	(Boisduval, 1828)		
Nymphalidae	*Boloria*	*Boloria selene*	Denis & Schiffermüller, 1775		
Nymphalidae	*Dione*	*Dione incarnata*	Riley, 1926	(27)	Elevation of *Dione vanillae incarnata* from subspecies to species
Nymphalidae	*Dryas*	*Dryas alcionea*	(Cramer, 1779)		
Nymphalidae	*Euptoieta*	*Euptoieta claudia*	(Cramer, 1775)		
Nymphalidae	*Euptoieta*	*Euptoieta hegesia*	(Cramer, 1779)		
Nymphalidae	*Heliconius*	*Heliconius charithonia*	(Linnaeus, 1767)		
Nymphalidae	*Speyeria*	*Speyeria adiaste*	Edwards, 1864	(35,36)	Maintain existing usage of genus *Speyeria* (Simonsen et al. 2006 and DeMoya et al. 2017), despite genomic evidence (Zhang et al. 2020) suggesting to demote *Speyeria* from genus to subgenus. Tribe addition (Argynnini)
Nymphalidae	*Speyeria*	*Speyeria aphrodite*	Fabricius, 1787	(35,36)	Maintain existing usage of genus *Speyeria* (Simonsen et al. 2006 and DeMoya et al. 2017), despite genomic evidence (Zhang et al. 2020) suggesting to demote *Speyeria* from genus to subgenus. Tribe addition (Argynnini)
Nymphalidae	*Speyeria*	*Speyeria atlantis*	Edwards, 1862	(35,36)	Maintain existing usage of genus *Speyeria* (Simonsen et al. 2006 and DeMoya et al. 2017), despite genomic evidence (Zhang et al. 2020) suggesting to demote *Speyeria* from genus to subgenus. Tribe addition (Argynnini)
Nymphalidae	*Speyeria*	*Speyeria callippe*	Edwards, 1872	(35,36)	Maintain existing usage of genus *Speyeria* (Simonsen et al. 2006 and DeMoya et al. 2017), despite genomic evidence (Zhang et al. 2020) suggesting to demote *Speyeria* from genus to subgenus. Tribe addition (Argynnini)
Nymphalidae	*Speyeria*	*Speyeria coronis*	Behr, 1864	(35,36)	Maintain existing usage of genus *Speyeria* (Simonsen et al. 2006 and DeMoya et al. 2017), despite genomic evidence (Zhang et al. 2020) suggesting to demote *Speyeria* from genus to subgenus. Tribe addition (Argynnini)

Nymphalidae	*Speyeria*	*Speyeria cybele*	Fabricius, 1775	(35,36)	Maintain existing usage of genus *Speyeria* (Simonsen et al. 2006 and DeMoya et al. 2017), despite genomic evidence (Zhang et al. 2020) suggesting to demote *Speyeria* from genus to subgenus. Tribe addition (Argynnini)
Nymphalidae	*Speyeria*	*Speyeria diana*	Cramer, 1777	(35,36)	Maintain existing usage of genus *Speyeria* (Simonsen et al. 2006 and DeMoya et al. 2017), despite genomic evidence (Zhang et al. 2020) suggesting to demote *Speyeria* from genus to subgenus. Tribe addition (Argynnini)
Nymphalidae	*Speyeria*	*Speyeria edwardsii*	Reakirt, 1866	(35,36)	Maintain existing usage of genus *Speyeria* (Simonsen et al. 2006 and DeMoya et al. 2017), despite genomic evidence (Zhang et al. 2020) suggesting to demote *Speyeria* from genus to subgenus. Tribe addition (Argynnini)
Nymphalidae	*Speyeria*	*Speyeria egleis*	Behr, 1862	(35,36)	Maintain existing usage of genus *Speyeria* (Simonsen et al. 2006 and DeMoya et al. 2017), despite genomic evidence (Zhang et al. 2020) suggesting to demote *Speyeria* from genus to subgenus. Tribe addition (Argynnini)
Nymphalidae	*Speyeria*	*Speyeria hesperis*	Edwards, 1864	(35,36)	Maintain existing usage of genus *Speyeria* (Simonsen et al. 2006 and DeMoya et al. 2017), despite genomic evidence (Zhang et al. 2020) suggesting to demote *Speyeria* from genus to subgenus. Tribe addition (Argynnini)
Nymphalidae	*Speyeria*	*Speyeria hydaspe*	Comstock, 1925	(35,36)	Maintain existing usage of genus *Speyeria* (Simonsen et al. 2006 and DeMoya et al. 2017), despite genomic evidence (Zhang et al. 2020) suggesting to demote *Speyeria* from genus to subgenus. Tribe addition (Argynnini)
Nymphalidae	*Speyeria*	*Speyeria idalia*	Drury, 1773	(35,36)	Maintain existing usage of genus *Speyeria* (Simonsen et al. 2006 and DeMoya et al. 2017), despite genomic evidence (Zhang et al. 2020) suggesting to demote *Speyeria* from genus to subgenus. Tribe addition (Argynnini)
Nymphalidae	*Speyeria*	*Speyeria mormonia*	Boisduval, 1869	(35,36)	Maintain existing usage of genus *Speyeria* (Simonsen et al. 2006 and DeMoya et al. 2017), despite genomic evidence (Zhang et al. 2020) suggesting to demote *Speyeria* from genus to subgenus. Tribe addition (Argynnini)
Nymphalidae	*Speyeria*	*Speyeria nokomis*	Edwards, 1862	(35,36)	Maintain existing usage of genus *Speyeria* (Simonsen et al. 2006 and DeMoya et al. 2017), despite genomic evidence (Zhang et al. 2020) suggesting to demote *Speyeria* from genus to subgenus. Tribe addition (Argynnini)
Nymphalidae	*Speyeria*	*Speyeria zerene*	Hammond & Harry & McCorkle, 2001	(35,36)	Maintain existing usage of genus *Speyeria* (Simonsen et al. 2006 and DeMoya et al. 2017), despite genomic evidence (Zhang et al. 2020) suggesting to demote *Speyeria* from genus to subgenus. Tribe addition (Argynnini)
Nymphalidae	*Libytheana*	*Libytheana carinenta*	(Cramer, 1777)		
Nymphalidae	*Adelpha*	*Adelpha basiloides*	(Bates, 1865)		
Nymphalidae	*Adelpha*	*Adelpha californica*	(Butler, 1865)		
Nymphalidae	*Adelpha*	*Adelpha eulalia*	(Doubleday, 1848)		
Nymphalidae	*Limenitis*	*Limenitis archippus*	(Cramer, 1775)		
Nymphalidae	*Limenitis*	*Limenitis arthemis*	(Drury, 1773)		
Nymphalidae	*Limenitis*	*Limenitis lorquini*	Boisduval, 1852		
Nymphalidae	*Limenitis*	*Limenitis weidemeyerii*	Edwards, 1861		
Nymphalidae	*Aglais*	*Aglais io*	(Linnaeus, 1758)	(24)	Tribe addition (Nymphalini)
Nymphalidae	*Aglais*	*Aglais milberti*	(Godart, 1819)	(24)	Tribe addition (Nymphalini)
Nymphalidae	*Anartia*	*Anartia fatima*	(Fabricius, 1793)	(24)	Tribe addition (Victorinini)
Nymphalidae	*Anartia*	*Anartia jatrophae*	(Linnaeus, 1763)	(24)	Tribe addition (Victorinini)
Nymphalidae	*Anthanassa*	*Anthanassa texana*	(Edwards, 1863)	(24)	Tribe addition (Melitaeini)

Nymphalidae	*Anthanassa*	*Anthanassa tulcis*	(Bates, 1864)	(24)	Tribe addition (Melitaeini)
Nymphalidae	*Chlosyne*	*Chlosyne acastus*	(Edwards, 1874)		
Nymphalidae	*Chlosyne*	*Chlosyne californica*	(Wright, 1905)		
Nymphalidae	*Chlosyne*	*Chlosyne chinatiensis*	(Tinkham, 1944)		
Nymphalidae	*Chlosyne*	*Chlosyne coronado*	(Smith & Brock, 1988)		
Nymphalidae	*Chlosyne*	*Chlosyne cyneas*	(Godman & Salvin, 1878)		
Nymphalidae	*Chlosyne*	*Chlosyne damoetas*	(Skinner, 1902)		
Nymphalidae	*Chlosyne*	*Chlosyne definita*	(Aaron, 1885)		
Nymphalidae	*Chlosyne*	*Chlosyne endeis*	(Godman & Salvin, 1894)		
Nymphalidae	*Chlosyne*	*Chlosyne flavula*	Barnes & McDunnough, 1918	(20)	Elevation of *Chlosyne palla flavula* from subspecies to species
Nymphalidae	*Chlosyne*	*Chlosyne fulvia*	(Edwards, 1879)		
Nymphalidae	*Chlosyne*	*Chlosyne gabbii*	(Behr, 1863)		
Nymphalidae	*Chlosyne*	*Chlosyne gorgone*	(Hübner, 1810)		
Nymphalidae	*Chlosyne*	*Chlosyne harrisii*	(Scudder, 1863)		
Nymphalidae	*Chlosyne*	*Chlosyne hoffmanni*	(Behr, 1863)		
Nymphalidae	*Chlosyne*	*Chlosyne janais*	(Drury, 1782)		
Nymphalidae	*Chlosyne*	*Chlosyne lacinia*	(Geyer, 1837)		
Nymphalidae	*Chlosyne*	*Chlosyne leanira*	(Felder & Felder, 1860)		
Nymphalidae	*Chlosyne*	*Chlosyne nycteis*	(Doubleday, 1847)		
Nymphalidae	*Chlosyne*	*Chlosyne palla*	(Boisduval, 1852)		
Nymphalidae	*Chlosyne*	*Chlosyne theona*	(Ménétriés, 1855)		
Nymphalidae	*Chlosyne*	*Chlosyne whitneyi*	(Behr, 1863)		
Nymphalidae	*Euphydryas*	*Euphydryas anicia*	(Doubleday, 1847)	(28)	Tribe addition (Polyommatini)
Nymphalidae	*Euphydryas*	*Euphydryas chalcedona*	(Doubleday, 1847)	(28)	Tribe addition (Polyommatini)
Nymphalidae	*Euphydryas*	*Euphydryas colon*	(Edwards, 1881)	(28)	Tribe addition (Polyommatini)
Nymphalidae	*Euphydryas*	*Euphydryas editha*	(Boisduval, 1852)	(28)	Tribe addition (Polyommatini)
Nymphalidae	*Euphydryas*	*Euphydryas gillettii*	(Barnes, 1897)	(28)	Tribe addition (Polyommatini)
Nymphalidae	*Euphydryas*	*Euphydryas phaeton*	(Drury, 1773)	(28)	Tribe addition (Polyommatini)
Nymphalidae	*Junonia*	*Junonia grisea*	Austin & Emmel, 1998	(24,37)	Elevation of *Junonia coenia grisea* from subspecies to species. Tribe addition (Junoniini)
Nymphalidae	*Junonia*	*Junonia neildi*	Brévignon, 2004	(24,27)	Elevation of *Junonia genoveva neildi* from subspecies to species. Tribe addition (Junoniini)
Nymphalidae	*Junonia*	*Junonia nigrosuffusa*	Barnes & McDunnough, 1916	(24,27)	Elevation of *Junonia evarete nigrosoffusa* from subspecies to species. Tribe addition (Junoniini)

Nymphalidae	*Junonia*	*Junonia stemosa*	Grishin, 2020	(24,27)	Central and South Texas records of *Junonia nigrosoffusa* shuold be transferred to the new species *Junonia stemosa*. Tribe addition (Junonoiini)
Nymphalidae	*Junonia*	*Junonia zonalis*	Felder & Felder, 1867	(24,27)	Elevation of *Junonia evarete zonalis* from subspecies to species. Tribe addition (Junoniini)
Nymphalidae	*Microtia*	*Microtia dymas*	(Edwards, 1877)	(24,28)	*Dymasia* is a junior synonym of *Microtia* and transfer *of* species to *Microtia*. Tribe addition (Melitaeini)
Nymphalidae	*Microtia*	*Microtia elada*	(Hewitson, 1868)	(24,28)	*Dymasia* is a junior synonym of *Microtia* and transfer *of* species to *Microtia*. Tribe addition (Melitaeini)
Nymphalidae	*Microtia*	*Microtia perse*	(Edwards, 1882)	(24,28)	*Dymasia* is a junior synonym of *Microtia* and transfer *of* species to *Microtia*. Tribe addition (Melitaeini)
Nymphalidae	*Nymphalis*	*Nymphalis antiopa*	(Linnaeus, 1758)		
Nymphalidae	*Nymphalis*	*Nymphalis californica*	(Boisduval, 1852)		
Nymphalidae	*Nymphalis*	*Nymphalis l-album*	(Esper, 1781)		
Nymphalidae	*Phyciodes*	*Phyciodes batesii*	(Reakirt, [1866])	(28)	Tribe addition (Melitaeini)
Nymphalidae	*Phyciodes*	*Phyciodes cocyta*	(Cramer, 1777)	(28)	Tribe addition (Melitaeini)
Nymphalidae	*Phyciodes*	*Phyciodes graphica*	(Felder, 1869)	(28)	Tribe addition (Melitaeini)
Nymphalidae	*Phyciodes*	*Phyciodes mylitta*	(Edwards, 1861)	(28)	Tribe addition (Melitaeini)
Nymphalidae	*Phyciodes*	*Phyciodes orseis*	Edwards, 1871	(28)	Tribe addition (Melitaeini)
Nymphalidae	*Phyciodes*	*Phyciodes pallescens*	(Felder, 1869)	(28)	Tribe addition (Melitaeini)
Nymphalidae	*Phyciodes*	*Phyciodes pallida*	(Edwards, 1864)	(28)	Tribe addition (Melitaeini)
Nymphalidae	*Phyciodes*	*Phyciodes phaon*	(Edwards, 1864)	(28)	Tribe addition (Melitaeini)
Nymphalidae	*Phyciodes*	*Phyciodes picta*	(Edwards, 1865)	(28)	Tribe addition (Melitaeini)
Nymphalidae	*Phyciodes*	*Phyciodes pulchella*	(Boisduval, 1852)	(28)	Tribe addition (Melitaeini)
Nymphalidae	*Phyciodes*	*Phyciodes tharos*	(Drury, 1773)	(28)	Tribe addition (Melitaeini)
Nymphalidae	*Poladryas*	*Poladryas arachne*	(Edwards, 1869)	(28)	Tribe addition (Melitaeini)
Nymphalidae	*Poladryas*	*Poladryas minuta*	(Edwards, 1861)	(28)	Tribe addition (Melitaeini)
Nymphalidae	*Polygonia*	*Polygonia comma*	(Harris, 1841)	(24)	Tribe addition (Nymphalini)
Nymphalidae	*Polygonia*	*Polygonia faunus*	(Edwards, 1862)	(24)	Tribe addition (Nymphalini)
Nymphalidae	*Polygonia*	*Polygonia gracilis*	(Grote & Robinson, 1867)	(24)	Tribe addition (Nymphalini)
Nymphalidae	*Polygonia*	*Polygonia interrogationis*	(Fabricius, 1798)	(24)	Tribe addition (Nymphalini)
Nymphalidae	*Polygonia*	*Polygonia oreas*	(Edwards, 1869)	(24)	Tribe addition (Nymphalini)
Nymphalidae	*Polygonia*	*Polygonia progne*	(Cramer, 1775)	(24)	Tribe addition (Nymphalini)
Nymphalidae	*Polygonia*	*Polygonia satyrus*	(Edwards, 1869)	(24)	Tribe addition (Nymphalini)
Nymphalidae	*Siproeta*	*Siproeta stelenes*	(Linnaeus, 1758)	(24)	Tribe addition (Victorinini)
Nymphalidae	*Vanessa*	*Vanessa annabella*	(Field, 1971)		
Nymphalidae	*Vanessa*	*Vanessa atalanta*	(Linnaeus, 1758)		

Nymphalidae	*Vanessa*	*Vanessa cardui*	(Linnaeus, 1758)		
Nymphalidae	*Vanessa*	*Vanessa virginiensis*	(Drury, 1773)		
Nymphalidae	*Cercyonis*	*Cercyonis hypoleuca*	(Hawks & Emmel, 1998)	(23)	Elevation of *Cercyonis sthenele hypoleuca* from subspecies to species
Nymphalidae	*Cercyonis*	*Cercyonis incognita*	Emmel, Emmel & Mattoon, 2012	(24)	Tribe addition (Satytini)
Nymphalidae	*Cercyonis*	*Cercyonis meadii*	(Edwards, 1872)	(24)	Tribe addition (Satytini)
Nymphalidae	*Cercyonis*	*Cercyonis oetus*	(Boisduval, 1869)	(24)	Tribe addition (Satytini)
Nymphalidae	*Cercyonis*	*Cercyonis pegala*	(Fabricius, 1775)	(24)	Tribe addition (Satytini)
Nymphalidae	*Cercyonis*	*Cercyonis sthenele*	(Boisduval, 1852)	(24)	Tribe addition (Satytini)
Nymphalidae	*Cissia*	*Cissia rubricata*	(Edwards, 1871)	(38)	Transfer of species from *Megisto* to *Cissia*
Nymphalidae	*Coenonympha*	*Coenonympha california*	Westwood, 1851	(24)	Tribe addition (Satyrini)
Nymphalidae	*Coenonympha*	*Coenonympha haydenii*	Edwards, 1872	(24)	Tribe addition (Satyrini)
Nymphalidae	*Coenonympha*	*Coenonympha tullia*	(Müller, 1764)	(24)	Tribe addition (Satyrini)
Nymphalidae	*Cyllopsis*	*Cyllopsis gemma*	(Hübner, 1818)		
Nymphalidae	*Cyllopsis*	*Cyllopsis pertepida*	(Dyar, 1912)		
Nymphalidae	*Cyllopsis*	*Cyllopsis pyracmon*	(Butler, 1867)		
Nymphalidae	*Erebia*	*Erebia callias*	Edwards, 1871	(24)	Tribe addition (Satyrini)
Nymphalidae	*Erebia*	*Erebia disa*	(Thunberg, 1791)	(24)	Tribe addition (Satyrini)
Nymphalidae	*Erebia*	*Erebia discoidalis*	(Kirby, 1837)	(24)	Tribe addition (Satyrini)
Nymphalidae	*Erebia*	*Erebia epipsodea*	Butler, 1868	(24)	Tribe addition (Satyrini)
Nymphalidae	*Erebia*	*Erebia fasciata*	Butler, 1868	(24)	Tribe addition (Satyrini)
Nymphalidae	*Erebia*	*Erebia lafontainei*	Troubridge & Philip, 1983	(24)	Tribe addition (Satyrini)
Nymphalidae	*Erebia*	*Erebia mackinleyensis*	Gunder, 1932	(24)	Tribe addition (Satyrini)
Nymphalidae	*Erebia*	*Erebia magdalena*	Strecker, 1880	(24)	Tribe addition (Satyrini)
Nymphalidae	*Erebia*	*Erebia mancinus*	Doubleday, 1849	(24)	Tribe addition (Satyrini)
Nymphalidae	*Erebia*	*Erebia occulta*	Roos & Kimmich, 1983	(24)	Tribe addition (Satyrini)
Nymphalidae	*Erebia*	*Erebia pawloskii*	Ménétriés, 1859	(24)	Tribe addition (Satyrini)
Nymphalidae	*Erebia*	*Erebia rossii*	(Curtis, 1835)	(24)	Tribe addition (Satyrini)
Nymphalidae	*Erebia*	*Erebia vidleri*	Elwes, 1898	(24)	Tribe addition (Satyrini)
Nymphalidae	*Erebia*	*Erebia youngi*	Holland, 1900	(24)	Tribe addition (Satyrini)
Nymphalidae	*Gyrocheilus*	*Gyrocheilus patrobas*	(Hewitson, 1862)	(24)	Tribe addition (Satyrini)
Nymphalidae	*Hermeuptychia*	*Hermeuptychia hermybius*	(Grishin, 2014)		
Nymphalidae	*Hermeuptychia*	*Hermeuptychia intricata*	(Grishin, 2014)		

Nymphalidae	*Hermeuptychia*	*Hermeuptychia sosybius*	(Fabricius, 1793)		
Nymphalidae	*Lethe*	*Lethe anthedon*	(Clark, 1936)	(24)	Demotion of *Enodia* from genus to subgenus, and transfer of species from *Enodia* to *Lethe*. Tribe addition (Satyrini)
Nymphalidae	*Lethe*	*Lethe appalachia*	Chermock, 1947	(24)	Tribe addition (Satyrini)
Nymphalidae	*Lethe*	*Lethe creola*	(Skinner, 1897)	(24)	Tribe addition (Satyrini)
Nymphalidae	*Lethe*	*Lethe eurydice*	(Linnaeus, 1763)	(24)	Tribe addition (Satyrini)
Nymphalidae	*Lethe*	*Lethe portlandia*	(Fabricius, 1781)		
Nymphalidae	*Megisto*	*Megisto cymela*	(Cramer, 1777)		
Nymphalidae	*Neonympha*	*Neonympha areolatus*	(Smith, 1797)	(24,39)	*Neonympha helicta* is a synonym of *Neonympha areolatus*, transfer of species to *Neonympha areolatus*. Tribe addition (Satyrini)
Nymphalidae	*Neonympha*	*Neonympha mitchellii*	French, 1889	(24)	Tribe addition (Satyrini)
Nymphalidae	*Oeneis*	*Oeneis alberta*	Elwes, 1893		
Nymphalidae	*Oeneis*	*Oeneis alpina*	Kurentsov, 1970		
Nymphalidae	*Oeneis*	*Oeneis bore*	(Schneider, 1792)		
Nymphalidae	*Oeneis*	*Oeneis chryxus*	(Doubleday, 1849)		
Nymphalidae	*Oeneis*	*Oeneis jutta*	(Hübner, 1806)		
Nymphalidae	*Oeneis*	*Oeneis macounii*	(Edwards, 1885)		
Nymphalidae	*Oeneis*	*Oeneis melissa*	(Fabricius, 1775)		
Nymphalidae	*Oeneis*	*Oeneis nevadensis*	(Felder & Felder, 1867)		
Nymphalidae	*Oeneis*	*Oeneis philipi*	Troubridge, 1988		
Nymphalidae	*Oeneis*	*Oeneis polixenes*	(Fabricius, 1775)		
Nymphalidae	*Oeneis*	*Oeneis ridingsii*	(Edwards, 1865)	(25,40)	Demotion of *Neominois* from genus to subgenus, and transfer of species to *Oeneis*
Nymphalidae	*Oeneis*	*Oeneis tanana*	Warren & Nakahara, 2016		
Nymphalidae	*Oeneis*	*Oeneis uhleri*	(Reakirt, 1866)		
Nymphalidae	*Paramacera*	*Paramacera allyni*	Miller, 1972	(24,34)	Elevation of *Paramacera xicaque allyni* to *Paramacera allyni*. Tribe addition (Satyrini)
Papilionidae	*Battus*	*Battus philenor*	(Linnaeus, 1771)	(24)	Tribe addition (Troidini)
Papilionidae	*Battus*	*Battus polydamas*	(Linnaeus, 1758)	(24)	Tribe addition (Troidini)
Papilionidae	*Eurytides*	*Eurytides marcellus*	(Cramer, 1777)	(24)	Demotion of *Mimoides* from genus to subgenus and transfer of species to *Eurytides*. Tribe addition (Leptocircini)
Papilionidae	*Heraclides*	*Heraclides anchisiades*	(Esper, 1788)	(24,28,41)	Elevation of *Heraclides* from subgenus to genus and transfer of species from *Papilio* to *Heraclides*. Tribe addition (Papilionini)
Papilionidae	*Heraclides*	*Heraclides andraemon*	Hübner, 1823	(24,28,41)	Elevation of *Heraclides* from subgenus to genus and transfer of species from *Papilio* to *Heraclides*. Tribe addition (Papilionini)
Papilionidae	*Heraclides*	*Heraclides caiguanabus*	(Poey, 1852)	(24,28,41)	Elevation of *Heraclides* from subgenus to genus and transfer of species from *Papilio* to *Heraclides*. Tribe addition (Papilionini)
Papilionidae	*Heraclides*	*Heraclides cresphontes*	(Cramer, 1777)	(24,28,41)	Elevation of *Heraclides* from subgenus to genus and transfer of species from *Papilio* to *Heraclides*. Tribe addition (Papilionini)

Papilionidae	*Heraclides*	*Heraclides ornythion*	(Boisduval, 1836)	(24,28,41)	Elevation of *Heraclides* from subgenus to genus and transfer of species from *Papilio* to *Heraclides*. Tribe addition (Papilionini)
Papilionidae	*Heraclides*	*Heraclides ponceana*	Schaus, 1911	(24,28,41)	Elevation of *Heraclides aristodemus ponceana* to *Heraclides ponceana*. Tribe addition (Papilionini)
Papilionidae	*Heraclides*	*Heraclides rogeri*	(Boisduval, 1836)	(24,28,41)	Elevation of *Heraclides* from subgenus to genus and transfer of species from *Papilio* to *Heraclides*. Tribe addition (Papilionini)
Papilionidae	*Heraclides*	*Heraclides rumiko*	Shiraiwa & Grishin, 2014	(24,28,41)	Elevation of *Heraclides* from subgenus to genus and transfer of species from *Papilio* to *Heraclides*. Tribe addition (Papilionini)
Papilionidae	*Heraclides*	*Heraclides thoas*	(Linnaeus, 1771)	(24,28,41)	Elevation of *Heraclides* from subgenus to genus and transfer of species from *Papilio* to *Heraclides*. Tribe addition (Papilionini)
Papilionidae	*Papilio*	*Papilio brevicauda*	Saunders, 1868		
Papilionidae	*Papilio*	*Papilio indra*	Reakirt, 1866		
Papilionidae	*Papilio*	*Papilio joanae*	Heitzman, 1973		
Papilionidae	*Papilio*	*Papilio machaon*	Linnaeus, 1758		
Papilionidae	*Papilio*	*Papilio polyxenes*	Fabricius, 1775		
Papilionidae	*Papilio*	*Papilio zelicaon*	Lucas, 1852		
Papilionidae	*Pterourus*	*Pterourus alexiares*	(Hopffer, 1866)	(24,28,41)	Elevation of *Pterourus* from subgenus to genus, and transfer of species from *Papilio* to *Pterourus*. Tribe addition (Papilionini)
Papilionidae	*Pterourus*	*Pterourus appalachiensis*	Pavulaan & Wright, 2002	(24,28,41)	Elevation of *Pterourus* from subgenus to genus, and transfer of species from *Papilio* to *Pterourus*. Tribe addition (Papilionini)
Papilionidae	*Pterourus*	*Pterourus canadensis*	(Rothschild & Jordan, 1906)	(24,28,41)	Elevation of *Pterourus* from subgenus to genus, and transfer of species from *Papilio* to *Pterourus*. Tribe addition (Papilionini)
Papilionidae	*Pterourus*	*Pterourus eurymedon*	(Lucas, 1852)	(24,28,41)	Elevation of *Pterourus* from subgenus to genus, and transfer of species from *Papilio* to *Pterourus*. Tribe addition (Papilionini)
Papilionidae	*Pterourus*	*Pterourus glaucus*	(Linnaeus, 1758)	(24,28,41)	Elevation of *Pterourus* from subgenus to genus, and transfer of species from *Papilio* to *Pterourus*. Tribe addition (Papilionini)
Papilionidae	*Pterourus*	*Pterourus multicaudata*	(Kirby, 1884)	(24,28,41)	Elevation of *Pterourus* from subgenus to genus, and transfer of species from *Papilio* to *Pterourus*. Tribe addition (Papilionini)
Papilionidae	*Pterourus*	*Pterourus palamedes*	(Drury, 1773)	(24,28,41)	Elevation of *Pterourus* from subgenus to genus, and transfer of species from *Papilio* to *Pterourus*. Tribe addition (Papilionini)
Papilionidae	*Pterourus*	*Pterourus rutulus*	(Lucas, 1852)	(24,28,41)	Elevation of *Pterourus* from subgenus to genus, and transfer of species from *Papilio* to *Pterourus*. Tribe addition (Papilionini)
Papilionidae	*Pterourus*	*Pterourus troilus*	(Linnaeus, 1758)	(24,28,41)	Elevation of *Pterourus* from subgenus to genus, and transfer of species from *Papilio* to *Pterourus*. Tribe addition (Papilionini)
Papilionidae	*Parnassius*	*Parnassius behrii*	Edwards, 1870	(24)	Tribe addition (Parnassiini)
Papilionidae	*Parnassius*	*Parnassius clodius*	Ménétriés, 1857	(24)	Tribe addition (Parnassiini)
Papilionidae	*Parnassius*	*Parnassius eversmanni*	Ménétriés, 1850	(24)	Tribe addition (Parnassiini)
Papilionidae	*Parnassius*	*Parnassius phoebus*	(Fabricius, 1793)	(24)	Tribe addition (Parnassiini)
Papilionidae	*Parnassius*	*Parnassius smintheus*	Doubleday, 1847	(24)	Tribe addition (Parnassiini)
Pieridae	*Abaeis*	*Abaeis boisduvaliana*	(Felder & Felder, 1865)	(25)	Transfer of species from *Eurema* to *Abaeis*
Pieridae	*Abaeis*	*Abaeis mexicana*	(Boisduval, 1836)	(25)	Transfer of species from *Eurema* to *Abaeis*
Pieridae	*Abaeis*	*Abaeis nicippe*	(Cramer, 1779)		

Pieridae	*Anteos*	*Anteos clorinde*	(Godart, 1824)		
Pieridae	*Anteos*	*Anteos maerula*	(Fabricius, 1775)		
Pieridae	*Aphrissa*	*Aphrissa neleis*	(Boisduval, 1836)		
Pieridae	*Aphrissa*	*Aphrissa statira*	(Cramer, 1777)		
Pieridae	*Colias*	*Colias alexandra*	Edwards, 1863		
Pieridae	*Colias*	*Colias behrii*	Edwards, 1866		
Pieridae	*Colias*	*Colias boothii*	Curtis, 1835	(42)	Demoting *Colias canadensis* to *Colias boothii canadensis*. Elevation of *Colias thyche boothii* to *Colias boothii*
Pieridae	*Colias*	*Colias christina*	Edwards, 1863		
Pieridae	*Colias*	*Colias eriphyle*	Edwards, 1876		
Pieridae	*Colias*	*Colias eurytheme*	Boisduval, 1852		
Pieridae	*Colias*	*Colias gigantea*	Strecker, 1900		
Pieridae	*Colias*	*Colias harfordii*	Edwards, 1877		
Pieridae	*Colias*	*Colias hecla*	Lefèbvre, 1836		
Pieridae	*Colias*	*Colias interior*	Scudder, 1862		
Pieridae	*Colias*	*Colias johanseni*	Troubridge & Philip, 1990		
Pieridae	*Colias*	*Colias meadii*	Edwards, 1871		
Pieridae	*Colias*	*Colias nastes*	Boisduval, 1832		
Pieridae	*Colias*	*Colias occidentalis*	Scudder, 1862		
Pieridae	*Colias*	*Colias palaeno*	(Linnaeus, 1761)		
Pieridae	*Colias*	*Colias pelidne*	Boisduval & Le Conte, 1830		
Pieridae	*Colias*	*Colias philodice*	Godart, 1819		
Pieridae	*Colias*	*Colias rankinensis*	Verhulst, 2009		
Pieridae	*Colias*	*Colias scudderii*	Reakirt, 1865		
Pieridae	*Colias*	*Colias skinneri*	Barnes, 1897		
Pieridae	*Colias*	*Colias vitabunda*	Hovanitz, 1943		
Pieridae	*Eurema*	*Eurema daira*	(Godart, 1819)		
Pieridae	*Kricogonia*	*Kricogonia lyside*	(Godart, 1819)		
Pieridae	*Nathalis*	*Nathalis iole*	Boisduval, 1836		
Pieridae	*Phoebis*	*Phoebis agarithe*	(Boisduval, 1836)		
Pieridae	*Phoebis*	*Phoebis philea*	(Linnaeus, 1763)		
Pieridae	*Phoebis*	*Phoebis sennae*	(Linnaeus, 1758)		
Pieridae	*Pyrisitia*	*Pyrisitia dina*	(Poey, 1832)		

Pieridae	*Pyrisitia*	*Pyrisitia lisa*	(Boisduval & Le Conte, 1830)		
Pieridae	*Pyrisitia*	*Pyrisitia nise*	(Cramer, 1775)		
Pieridae	*Pyrisitia*	*Pyrisitia proterpia*	(Fabricius, 1775)		
Pieridae	*Zerene*	*Zerene cesonia*	(Stoll, 1790)		
Pieridae	*Zerene*	*Zerene eurydice*	(Boisduval, 1855)		
Pieridae	*Anthocharis*	*Anthocharis cethura*	Felder & Felder, 1865	(24)	Tribe addition (Anthocharidini)
Pieridae	*Anthocharis*	*Anthocharis julia*	Edwards, 1872	(24)	Tribe addition (Anthocharidini)
Pieridae	*Anthocharis*	*Anthocharis lanceolata*	Lucas, 1852	(24)	Tribe addition (Anthocharidini)
Pieridae	*Anthocharis*	*Anthocharis midea*	(Hübner, 1809)	(24)	Tribe addition (Anthocharidini)
Pieridae	*Anthocharis*	*Anthocharis sara*	Lucas, 1852	(24)	Tribe addition (Anthocharidini)
Pieridae	*Anthocharis*	*Anthocharis thoosa*	(Scudder, 1878)	(24)	Tribe addition (Anthocharidini)
Pieridae	*Archonias*	*Archonias nimbice*	(Boisduval, 1836)	(24,28)	*Catasticta* is now a junior synonym of *Archonias*. Tribe addition (Pierini)
Pieridae	*Ascia*	*Ascia monuste*	(Linnaeus, 1764)	(24)	Tribe addition (Pierini)
Pieridae	*Euchloe*	*Euchloe ausonides*	(Lucas, 1852)	(24)	Tribe addition (Anthocharidini)
Pieridae	*Euchloe*	*Euchloe creusa*	(Doubleday, 1847)	(24)	Tribe addition (Anthocharidini)
Pieridae	*Euchloe*	*Euchloe hyantis*	(Edwards, 1871)	(24)	Tribe addition (Anthocharidini)
Pieridae	*Euchloe*	*Euchloe lotta*	Beutenmüller, 1898	(24)	Tribe addition (Anthocharidini)
Pieridae	*Euchloe*	*Euchloe ochracea*	(Trybom, 1877)	(24,43)	*Euchloe naina* is now a synonym of *Euchloe ochracea*
Pieridae	*Euchloe*	*Euchloe olympia*	(Edwards, 1871)	(24)	Tribe addition (Anthocharidini)
Pieridae	*Ganyra*	*Ganyra josephina*	(Godart, 1819)	(24)	Tribe addition (Pierini)
Pieridae	*Glutophrissa*	*Glutophrissa drusilla*	(Cramer, 1777)	(24)	Tribe addition (Pierini)
Pieridae	*Neophasia*	*Neophasia menapia*	(Felder & Felder, 1859)	(24)	Tribe addition (Pierini)
Pieridae	*Neophasia*	*Neophasia terlooii*	Behr, 1869	(24)	Tribe addition (Pierini)
Pieridae	*Pieris*	*Pieris angelika*	Eitschberger, 1984		
Pieridae	*Pieris*	*Pieris marginalis*	Scudder, 1861		
Pieridae	*Pieris*	*Pieris oleracea*	(Harris, 1829)		
Pieridae	*Pieris*	*Pieris rapae*	(Linnaeus, 1758)		
Pieridae	*Pieris*	*Pieris virginiensis*	Edwards, 1870		
Pieridae	*Pontia*	*Pontia beckerii*	Edwards, 1871		
Pieridae	*Pontia*	*Pontia occidentalis*	(Reakirt, 1866)		
Pieridae	*Pontia*	*Pontia protodice*	(Boisduval & Le Conte, 1830)		
Pieridae	*Pontia*	*Pontia sisymbrii*	Boisduval, 1852		

Riodinidae	*Apodemia*	*Apodemia ares*	(Edwards, 1882)	(25)	Transfer between genera, from *Emesis* to *Apodemia*. Tribe addition (Emesidini)
Riodinidae	*Apodemia*	*Apodemia chisosensis*	(Freeman, 1964)	(25)	Demotion of *Neoapodemia* from genus to subgenus, and transfer of species to *Apodemia*. Tribe addition (Emesidini)
Riodinidae	*Apodemia*	*Apodemia duryi*	(Edwards, 1882)	(25)	Tribe addition (Emesidini)
Riodinidae	*Apodemia*	*Apodemia hepburni*	(Godman & Salvin, 1886)	(25)	Demotion of *Plesioarida* from genus to subgenus, and transfer of species to *Apodemia*. Tribe addition (Emesidini)
Riodinidae	*Apodemia*	*Apodemia mejicanus*	(Behr, 1865)	(25)	Tribe addition (Emesidini)
Riodinidae	*Apodemia*	*Apodemia mormo*	(Felder & Felder, 1859)	(25)	Tribe addition (Emesidini)
Riodinidae	*Apodemia*	*Apodemia nais*	(Edwards, 1877)	(25)	Demotion of *Neoapodemia* from genus to subgenus, and transfer of species to *Apodemia*. Tribe addition (Emesidini)
Riodinidae	*Apodemia*	*Apodemia palmerii*	(Edwards, 1870)	(25)	Demotion of *Plesioarida* from genus to subgenus and transfer of species to *Apodemia*. Tribe addition (Emesidini)
Riodinidae	*Apodemia*	*Apodemia virgulti*	(Behr, 1865)	(25)	Tribe addition (Emesidini)
Riodinidae	*Apodemia*	*Apodemia zela*	Butler, 1870	(25)	Transfer of species from *Emesis* to *Apodemia*. Tribe addition (Emesidini)
Riodinidae	*Calephelis*	*Calephelis arizonensis*	McAlpine, 1971	(24)	Tribe addition (Riodinini)
Riodinidae	*Calephelis*	*Calephelis borealis*	(Grote & Robinson, 1866)	(24)	Tribe addition (Riodinini)
Riodinidae	*Calephelis*	*Calephelis freemani*	McAlpine, 1971	(24)	Tribe addition (Riodinini)
Riodinidae	*Calephelis*	*Calephelis muticum*	McAlpine, 1937	(24)	Tribe addition (Riodinini)
Riodinidae	*Calephelis*	*Calephelis nemesis*	(Edwards, 1871)	(24)	Tribe addition (Riodinini)
Riodinidae	*Calephelis*	*Calephelis perditalis*	Barnes & McDunnough, 1918	(24)	Tribe addition (Riodinini)
Riodinidae	*Calephelis*	*Calephelis rawsoni*	McAlpine, 1939	(24)	Tribe addition (Riodinini)
Riodinidae	*Calephelis*	*Calephelis virginiensis*	(Guérin-Méneville, 1832)	(24)	Tribe addition (Riodinini)
Riodinidae	*Calephelis*	*Calephelis wrighti*	Holland, 1930	(24)	Tribe addition (Riodinini)
Riodinidae	*Caria*	*Caria ino*	(Godman & Salvin, 1886)		
Riodinidae	*Lasaia*	*Lasaia sula*	Staudinger, 1888		
Riodinidae	*Melanis*	*Melanis pixe*	(Boisduval, 1836)		

Table 2. Indices of genetic diversity for 691 butterfly species with resident populations in Canada and/or the USA. N indicates the number of specimens analyzed; h indicates haplotype diversity (Nei, 1987); **π** indicates nucleotide diversity (Tajima, 1983); G_{ST} represents a measure of spatial genetic structure; Max intra refers to the maximum intraspecific distance (p-distance); NN refers to the nearest neighbor distance (p-distance); H indicates the number of observed unique haplotypes; T indicates the total number of estimated haplotypes; R indicates the estimated proportion of haplotype diversity retrieved; distribution refers to the geographic distribution in respect of the last glacial maximum (LGM); NC indicates that values could not be computed due to insufficient sampling. The asterisk indicates species extending their distribution to Central and/or South American countries. Indices h, **π**, GST, Max intra, T, and R were computed only for species with ≥ 10 specimens.

**Family**	**Genus**	**Species**	**N**	**h**	**π**	**G_{ST}**	**Max intra**	**NN**	**H**	**T**	**R**	**Distribution**
Hesperiidae	*Adopaeoides*	*Adopaeoides prittwitzi**	3	NC	NC	NC	NC	8.33	1	NC	NC	South
Hesperiidae	*Agathymus*	*Agathymus alliae*	1	NC	NC	NC	NC	3.82	NC	NC	NC	South
Hesperiidae	*Agathymus*	*Agathymus aryxna**	2	NC	NC	NC	NC	1.22	2	NC	NC	South
Hesperiidae	*Agathymus*	*Agathymus baueri*	5	NC	NC	NC	NC	1.22	2	NC	NC	South
Hesperiidae	*Agathymus*	*Agathymus chisosensis*	0	NC	NC	NC	NC	NC	NC	NC	NC	South
Hesperiidae	*Agathymus*	*Agathymus estelleae**	0	NC	NC	NC	NC	NC	NC	NC	NC	South
Hesperiidae	*Agathymus*	*Agathymus evansi*	4	NC	NC	NC	NC	3.67	1	NC	NC	South
Hesperiidae	*Agathymus*	*Agathymus gentryi*	0	NC	NC	NC	NC	NC	NC	NC	NC	South
Hesperiidae	*Agathymus*	*Agathymus mariae*	1	NC	NC	NC	NC	5.88	NC	NC	NC	South
Hesperiidae	*Agathymus*	*Agathymus neumoegeni*	0	NC	NC	NC	NC	NC	NC	NC	NC	South
Hesperiidae	*Agathymus*	*Agathymus polingi**	1	NC	NC	NC	NC	3.67	NC	NC	NC	South
Hesperiidae	*Agathymus*	*Agathymus stephensi**	1	NC	NC	NC	NC	5.2	NC	NC	NC	South
Hesperiidae	*Aguna*	*Aguna mcguirei**	1	NC	NC	NC	NC	6.42	NC	NC	NC	South
Hesperiidae	*Amblyscirtes*	*Amblyscirtes aenus**	3	NC	NC	NC	NC	0	2	NC	NC	South
Hesperiidae	*Amblyscirtes*	*Amblyscirtes aesculapius*	3	NC	NC	NC	NC	4.74	1	NC	NC	South
Hesperiidae	*Amblyscirtes*	*Amblyscirtes alternata*	3	NC	NC	NC	NC	5.2	3	NC	NC	South
Hesperiidae	*Amblyscirtes*	*Amblyscirtes arizonae**	4	NC	NC	NC	NC	3.53	1	NC	NC	South
Hesperiidae	*Amblyscirtes*	*Amblyscirtes belli*	6	NC	NC	NC	NC	4.43	2	NC	NC	South
Hesperiidae	*Amblyscirtes*	*Amblyscirtes carolina**	7	NC	NC	NC	NC	2.47	3	NC	NC	South
Hesperiidae	*Amblyscirtes*	*Amblyscirtes cassus**	3	NC	NC	NC	NC	2.91	2	NC	NC	South
Hesperiidae	*Amblyscirtes*	*Amblyscirtes celia**	4	NC	NC	NC	NC	3.82	3	NC	NC	South

Hesperiidae	*Amblyscirtes*	*Amblyscirtes eos**	4	NC	NC	NC	NC	5.66	3	NC	NC	South
Hesperiidae	*Amblyscirtes*	*Amblyscirtes exoteria*	2	NC	NC	NC	NC	3.98	1	NC	NC	South
Hesperiidae	*Amblyscirtes*	*Amblyscirtes fimbriata*	2	NC	NC	NC	NC	6.18	2	NC	NC	North/alpine
Hesperiidae	*Amblyscirtes*	*Amblyscirtes hegon*	6	NC	NC	NC	NC	4.06	5	NC	NC	South
Hesperiidae	*Amblyscirtes*	*Amblyscirtes linda*	0	NC	NC	NC	NC	NC	NC	NC	NC	South
Hesperiidae	*Amblyscirtes*	*Amblyscirtes nereus**	2	NC	NC	NC	NC	4.28	1	NC	NC	South
Hesperiidae	*Amblyscirtes*	*Amblyscirtes nysa**	1	NC	NC	NC	NC	5.33	NC	NC	NC	South
Hesperiidae	*Amblyscirtes*	*Amblyscirtes oslari*	8	NC	NC	NC	NC	2.11	6	NC	NC	Mid-latitude
Hesperiidae	*Amblyscirtes*	*Amblyscirtes phylace*	2	NC	NC	NC	NC	2.11	2	NC	NC	South
Hesperiidae	*Amblyscirtes*	*Amblyscirtes reversa*	3	NC	NC	NC	NC	2.47	2	NC	NC	South
Hesperiidae	*Amblyscirtes*	*Amblyscirtes texanae*	1	NC	NC	NC	NC	2.91	NC	NC	NC	South
Hesperiidae	*Amblyscirtes*	*Amblyscirtes tolteca**	0	NC	NC	NC	NC	NC	NC	NC	NC	South
Hesperiidae	*Amblyscirtes*	*Amblyscirtes vialis*	25	0.74	0	0.67	0.5	0	6	8	0.76	Mid-latitude
Hesperiidae	*Anatrytone*	*Anatrytone logan**	22	0.61	0	0	0.6	7.19	9	36	0.25	Mid-latitude
Hesperiidae	*Ancyloxypha*	*Ancyloxypha arene**	3	NC	NC	NC	NC	6.72	2	NC	NC	South
Hesperiidae	*Ancyloxypha*	*Ancyloxypha numitor*	28	0.14	0	0.24	0.3	6.72	3	4	0.76	Mid-latitude
Hesperiidae	*Apyrrothrix*	*Apyrrothrix araxes**	2	NC	NC	NC	NC	9.2	1	NC	NC	South
Hesperiidae	*Arteurotia*	*Arteurotia artistella**	1	NC	NC	NC	NC	9.17	NC	NC	NC	South
Hesperiidae	*Asbolis*	*Asbolis capucinus**	5	NC	NC	NC	NC	7.1	1	NC	NC	South
Hesperiidae	*Atalopedes*	*Atalopedes campestris**	30	0.64	0	0.48	1.7	3.99	11	46	0.24	Mid-latitude
Hesperiidae	*Atrytone*	*Atrytone arogos*	6	NC	NC	NC	NC	5.21	6	NC	NC	South
Hesperiidae	*Atrytone*	*Atrytone bulenta*	4	NC	NC	NC	NC	4.13	3	NC	NC	South
Hesperiidae	*Atrytone*	*Atrytone byssus*	14	0.85	0	0.1	0.8	4.13	6	7	0.87	Mid-latitude
Hesperiidae	*Atrytonopsis*	*Atrytonopsis cestus**	1	NC	NC	NC	NC	4.59	NC	NC	NC	South
Hesperiidae	*Atrytonopsis*	*Atrytonopsis deva**	0	NC	NC	NC	NC	NC	NC	NC	NC	South
Hesperiidae	*Atrytonopsis*	*Atrytonopsis edwardsi**	2	NC	NC	NC	NC	3.67	1	NC	NC	South
Hesperiidae	*Atrytonopsis*	*Atrytonopsis hianna*	15	0.84	0	0.44	0.8	4.69	6	8	0.76	Mid-latitude
Hesperiidae	*Atrytonopsis*	*Atrytonopsis loammi*	0	NC	NC	NC	NC	NC	NC	NC	NC	South
Hesperiidae	*Atrytonopsis*	*Atrytonopsis lunus**	3	NC	NC	NC	NC	5.81	1	NC	NC	South
Hesperiidae	*Atrytonopsis*	*Atrytonopsis margarita*	0	NC	NC	NC	NC	NC	NC	NC	NC	South
Hesperiidae	*Atrytonopsis*	*Atrytonopsis pittacus**	2	NC	NC	NC	NC	3.67	1	NC	NC	South
Hesperiidae	*Atrytonopsis*	*Atrytonopsis python*	1	NC	NC	NC	NC	4.28	NC	NC	NC	South

Hesperiidae	*Atrytonopsis*	*Atrytonopsis quinteri*	0	NC	NC	NC	NC	NC	NC	NC	NC	South
Hesperiidae	*Atrytonopsis*	*Atrytonopsis vierecki*	3	NC	NC	NC	NC	4.59	1	NC	NC	South
Hesperiidae	*Autochton*	*Autochton caballo**	4	NC	NC	NC	NC	9.22	3	NC	NC	South
Hesperiidae	*Burnsius*	*Burnsius albescens**	11	0.51	0	NC	0.2	0	2	2	1	South
Hesperiidae	*Burnsius*	*Burnsius communis**	166	0.54	0	0.12	0.5	0	10	22	0.45	Mid-latitude
Hesperiidae	*Burnsius*	*Burnsius oileus**	13	0.59	0	0.92	0.9	4.77	3	3	1	South
Hesperiidae	*Burnsius*	*Burnsius philetas**	3	NC	NC	NC	NC	5.05	3	NC	NC	South
Hesperiidae	*Calpodes*	*Calpodes ethlius**	3	NC	NC	NC	NC	0.15	2	NC	NC	South
Hesperiidae	*Canesia*	*Canesia canescens**	0	NC	NC	NC	NC	NC	NC	NC	NC	South
Hesperiidae	*Carterocephalus*	*Carterocephalus mandan*	29	0.84	0	0.35	0.8	0	11	17	0.65	Mid-latitude
Hesperiidae	*Carterocephalus*	*Carterocephalus skada*	48	0.34	0	0.12	1	5.35	9	33	0.27	Mid-latitude
Hesperiidae	*Cecropterus*	*Cecropterus casica**	2	NC	NC	NC	NC	5.35	1	NC	NC	South
Hesperiidae	*Cecropterus*	*Cecropterus cincta**	1	NC	NC	NC	NC	6.73	NC	NC	NC	South
Hesperiidae	*Cecropterus*	*Cecropterus coyote**	1	NC	NC	NC	NC	3.67	NC	NC	NC	South
Hesperiidae	*Cecropterus*	*Cecropterus dorantes**	4	NC	NC	NC	NC	2.6	3	NC	NC	South
Hesperiidae	*Cecropterus*	*Cecropterus doryssus**	0	NC	NC	NC	NC	NC	NC	NC	NC	South
Hesperiidae	*Cecropterus*	*Cecropterus drusius**	2	NC	NC	NC	NC	0	2	NC	NC	South
Hesperiidae	*Cecropterus*	*Cecropterus toxeus**	0	NC	NC	NC	NC	NC	NC	NC	NC	South
Hesperiidae	*Celotes*	*Celotes limpia**	2	NC	NC	NC	NC	5.2	2	NC	NC	South
Hesperiidae	*Celotes*	*Celotes nessus**	2	NC	NC	NC	NC	0.17	1	NC	NC	South
Hesperiidae	*Chioides*	*Chioides albofasciatus**	3	NC	NC	NC	NC	6.12	1	NC	NC	Mid-latitude
Hesperiidae	*Chioides*	*Chioides zilpa**	1	NC	NC	NC	NC	7.8	NC	NC	NC	South
Hesperiidae	*Chiothion*	*Chiothion georgina**	5	NC	NC	NC	NC	5.1	2	NC	NC	South
Hesperiidae	*Clytius*	*Clytius clytius**	0	NC	NC	NC	NC	NC	NC	NC	NC	South
Hesperiidae	*Codatractus*	*Codatractus arizonensis**	4	NC	NC	NC	NC	0	4	NC	NC	South
Hesperiidae	*Cogia*	*Cogia caicus**	2	NC	NC	NC	NC	6.57	1	NC	NC	South
Hesperiidae	*Cogia*	*Cogia calchas**	3	NC	NC	NC	NC	2.6	1	NC	NC	South
Hesperiidae	*Cogia*	*Cogia hippalus**	3	NC	NC	NC	NC	2.6	3	NC	NC	South
Hesperiidae	*Cogia*	*Cogia outis*	5	NC	NC	NC	NC	0	3	NC	NC	South
Hesperiidae	*Copaeodes*	*Copaeodes edwardsii**	4	NC	NC	NC	NC	1.18	3	NC	NC	South
Hesperiidae	*Cymaenes*	*Cymaenes tripunctus**	3	NC	NC	NC	NC	6.88	1	NC	NC	South
Hesperiidae	*Eantis*	*Eantis tamenund**	2	NC	NC	NC	NC	5.5	1	NC	NC	South

Hesperiidae	*Ectomis*	*Ectomis octomaculata**	0	NC	NC	NC	NC	NC	NC	NC	NC	South
Hesperiidae	*Epargyreus*	*Epargyreus clarus*	53	0.73	0.01	0.24	2.4	4.5	13	53	0.25	Mid-latitude
Hesperiidae	*Epargyreus*	*Epargyreus huachuca**	0	NC	NC	NC	NC	NC	NC	NC	NC	South
Hesperiidae	*Epargyreus*	*Epargyreus zestos**	1	NC	NC	NC	NC	6.42	NC	NC	NC	South
Hesperiidae	*Ephyriades*	*Ephyriades brunnea**	1	NC	NC	NC	NC	6.61	NC	NC	NC	South
Hesperiidae	*Erynnis*	*Erynnis afranius**	5	NC	NC	NC	NC	0	3	NC	NC	Mid-latitude
Hesperiidae	*Erynnis*	*Erynnis baptisiae*	7	NC	NC	NC	NC	0.15	3	NC	NC	Mid-latitude
Hesperiidae	*Erynnis*	*Erynnis brizo**	31	0.78	0.01	0.15	1.3	0.71	6	7	0.86	Mid-latitude
Hesperiidae	*Erynnis*	*Erynnis burgessi**	11	0.84	0.01	NC	4	0	5	5	0.97	South
Hesperiidae	*Erynnis*	*Erynnis funeralis**	8	NC	NC	NC	NC	0	2	NC	NC	South
Hesperiidae	*Erynnis*	*Erynnis horatius*	34	0.59	0	0.22	0.8	2.55	12	61	0.2	Mid-latitude
Hesperiidae	*Erynnis*	*Erynnis icelus*	54	0.87	0	0.47	1.1	1.59	13	19	0.68	Mid-latitude
Hesperiidae	*Erynnis*	*Erynnis juvenalis**	36	0.65	0	0.77	0.8	1.64	10	16	0.62	Mid-latitude
Hesperiidae	*Erynnis*	*Erynnis lacustra**	2	NC	NC	NC	NC	0	2	NC	NC	South
Hesperiidae	*Erynnis*	*Erynnis lucilius*	11	0.44	0	NC	0.2	4.4	2	2	1	North/alpine
Hesperiidae	*Erynnis*	*Erynnis martialis*	14	0.79	0	NC	2.3	0	6	10	0.59	Mid-latitude
Hesperiidae	*Erynnis*	*Erynnis meridianus**	21	0.63	0	0.08	0.5	4.53	9	36	0.25	South
Hesperiidae	*Erynnis*	*Erynnis pacuvius**	30	0.19	0	0.69	0.3	0.15	4	7	0.58	Mid-latitude
Hesperiidae	*Erynnis*	*Erynnis persius*	57	0.65	0.02	0.75	6.8	0	14	34	0.41	Mid-latitude
Hesperiidae	*Erynnis*	*Erynnis propertius**	62	0.7	0.01	0.35	7.2	3.72	20	41	0.49	Mid-latitude
Hesperiidae	*Erynnis*	*Erynnis scudderi**	1	NC	NC	NC	NC	0.15	NC	NC	NC	South
Hesperiidae	*Erynnis*	*Erynnis somnus*	20	0.71	0	0.59	1.1	1.59	6	10	0.58	South
Hesperiidae	*Erynnis*	*Erynnis telemachus*	29	0.14	0	0	1.4	3.63	3	4	0.76	South
Hesperiidae	*Erynnis*	*Erynnis tristis**	13	0.63	0	NC	0.6	0	5	9	0.55	South
Hesperiidae	*Erynnis*	*Erynnis zarucco**	5	NC	NC	NC	NC	3.06	2	NC	NC	South
Hesperiidae	*Euphyes*	*Euphyes arpa*	2	NC	NC	NC	NC	0	2	NC	NC	South
Hesperiidae	*Euphyes*	*Euphyes bayensis*	1	NC	NC	NC	NC	3.06	NC	NC	NC	South
Hesperiidae	*Euphyes*	*Euphyes berryi*	1	NC	NC	NC	NC	1.76	NC	NC	NC	South
Hesperiidae	*Euphyes*	*Euphyes bimacula*	6	NC	NC	NC	NC	3.88	4	NC	NC	Mid-latitude
Hesperiidae	*Euphyes*	*Euphyes conspicua*	1	NC	NC	NC	NC	0	NC	NC	NC	Mid-latitude
Hesperiidae	*Euphyes*	*Euphyes dion*	11	0.71	0	NC	1.5	3.56	4	5	0.82	Mid-latitude
Hesperiidae	*Euphyes*	*Euphyes dukesi*	7	NC	NC	NC	NC	4.28	3	NC	NC	Mid-latitude

Hesperiidae	*Euphyes*	*Euphyes pilatka*	2	NC	NC	NC	NC	2.1	1	NC	NC	South
Hesperiidae	*Euphyes*	*Euphyes vestris*	44	0.85	0	0.45	1.7	10.11	15	35	0.43	Mid-latitude
Hesperiidae	*Gesta*	*Gesta invisus**	1	NC	NC	NC	NC	3.55	NC	NC	NC	South
Hesperiidae	*Hedone*	*Hedone vibex**	6	NC	NC	NC	NC	8.58	2	NC	NC	South
Hesperiidae	*Heliopetes*	*Heliopetes domicella**	1	NC	NC	NC	NC	6.42	NC	NC	NC	South
Hesperiidae	*Heliopetes*	*Heliopetes elonmuski**	2	NC	NC	NC	NC	3.52	1	NC	NC	South
Hesperiidae	*Heliopetes*	*Heliopetes ericetorum**	14	0	0	0	0	3.52	1	NC	NC	South
Hesperiidae	*Heliopetes*	*Heliopetes laviana**	3	NC	NC	NC	NC	8.26	2	NC	NC	South
Hesperiidae	*Heliopetes*	*Heliopetes macaira**	2	NC	NC	NC	NC	5.58	1	NC	NC	South
Hesperiidae	*Heliopetes*	*Heliopetes sublinea**	0	NC	NC	NC	NC	NC	NC	NC	NC	South
Hesperiidae	*Hesperia*	*Hesperia assiniboia*	77	0.65	0	0.61	1.4	1.41	13	62	0.21	North/alpine
Hesperiidae	*Hesperia*	*Hesperia attalus*	9	NC	NC	NC	NC	0.15	4	NC	NC	South
Hesperiidae	*Hesperia*	*Hesperia balcones*	2	NC	NC	NC	NC	0	2	NC	NC	South
Hesperiidae	*Hesperia*	*Hesperia colorado*	138	0.83	0.01	0.69	1.9	1.68	24	41	0.59	Mid-latitude
Hesperiidae	*Hesperia*	*Hesperia columbia*	9	NC	NC	NC	NC	0	1	NC	NC	South
Hesperiidae	*Hesperia*	*Hesperia comma*	239	0.92	0.01	0.88	4	2.45	48	89	0.54	North/alpine
Hesperiidae	*Hesperia*	*Hesperia dacotae*	36	0.26	0	0.01	0.6	2.25	6	16	0.38	North/alpine
Hesperiidae	*Hesperia*	*Hesperia juba*	34	0.73	0	0.13	0.8	1.59	9	21	0.43	Mid-latitude
Hesperiidae	*Hesperia*	*Hesperia leonardus*	28	0.7	0	0.93	0.5	2.6	5	6	0.84	Mid-latitude
Hesperiidae	*Hesperia*	*Hesperia lindseyi*	6	NC	NC	NC	NC	2.12	3	NC	NC	South
Hesperiidae	*Hesperia*	*Hesperia meskei*	8	NC	NC	NC	NC	1.41	2	NC	NC	South
Hesperiidae	*Hesperia*	*Hesperia metea*	8	NC	NC	NC	NC	2.91	3	NC	NC	Mid-latitude
Hesperiidae	*Hesperia*	*Hesperia miriamae*	1	NC	NC	NC	NC	2.61	NC	NC	NC	North/alpine
Hesperiidae	*Hesperia*	*Hesperia nevada*	28	0.53	0.01	0.99	2.3	1.59	5	5	0.95	Mid-latitude
Hesperiidae	*Hesperia*	*Hesperia ottoe*	6	NC	NC	NC	NC	1.83	2	NC	NC	Mid-latitude
Hesperiidae	*Hesperia*	*Hesperia pahaska**	5	NC	NC	NC	NC	2.63	2	NC	NC	Mid-latitude
Hesperiidae	*Hesperia*	*Hesperia sassacus*	19	0.6	0	0.12	0.8	2.22	6	12	0.51	Mid-latitude
Hesperiidae	*Hesperia*	*Hesperia uncas**	13	0.72	0	0	0.5	1.53	4	4	0.9	Mid-latitude
Hesperiidae	*Hesperia*	*Hesperia viridis**	3	NC	NC	NC	NC	0.15	1	NC	NC	South
Hesperiidae	*Hesperia*	*Hesperia woodgatei**	4	NC	NC	NC	NC	0	1	NC	NC	South
Hesperiidae	*Hesperopsis*	*Hesperopsis alpheus**	5	NC	NC	NC	NC	0	4	NC	NC	South
Hesperiidae	*Hesperopsis*	*Hesperopsis gracielae*	13	0.83	0	0.24	0.5	4.43	6	10	0.59	South

Hesperiidae	*Hesperopsis*	*Hesperopsis libya**	7	NC	NC	NC	NC	3.98	5	NC	NC	Mid-latitude
Hesperiidae	*Hylephila*	*Hylephila phyleus**	36	0.4	0	0.15	1.3	3.21	8	26	0.31	Mid-latitude
Hesperiidae	*Lerema*	*Lerema accius**	10	0.2	0	NC	0.2	0	2	2	1	South
Hesperiidae	*Lerema*	*Lerema liris**	9	NC	NC	NC	NC	0	4	NC	NC	South
Hesperiidae	*Lerema*	*Lerema ochrius**	2	NC	NC	NC	NC	6.08	2	NC	NC	South
Hesperiidae	*Lerodea*	*Lerodea arabus**	0	NC	NC	NC	NC	NC	NC	NC	NC	South
Hesperiidae	*Lerodea*	*Lerodea eufala**	8	NC	NC	NC	NC	4.28	3	NC	NC	South
Hesperiidae	*Limochores*	*Limochores sonora*	10	0.73	0	NC	0.7	8.56	4	4	0.95	Mid-latitude
Hesperiidae	*Lobotractus*	*Lobotractus mysie*	2	NC	NC	NC	NC	2.6	1	NC	NC	South
Hesperiidae	*Lon*	*Lon hobomok*	136	0.84	0	0.43	1.1	3.65	29	57	0.51	Mid-latitude
Hesperiidae	*Lon*	*Lon melane**	11	0	0	0	0	2.6	1	NC	NC	South
Hesperiidae	*Lon*	*Lon taxiles**	3	NC	NC	NC	NC	3.19	1	NC	NC	South
Hesperiidae	*Lon*	*Lon zabulon**	15	0.53	0	0.98	0.5	3.16	3	3	1	South
Hesperiidae	*Megathymus*	*Megathymus cofaqui*	3	NC	NC	NC	NC	3.36	3	NC	NC	South
Hesperiidae	*Megathymus*	*Megathymus streckeri*	2	NC	NC	NC	NC	6.36	2	NC	NC	Mid-latitude
Hesperiidae	*Megathymus*	*Megathymus ursus**	0	NC	NC	NC	NC	NC	NC	NC	NC	South
Hesperiidae	*Megathymus*	*Megathymus yuccae**	4	NC	NC	NC	NC	6.03	3	NC	NC	South
Hesperiidae	*Nastra*	*Nastra julia**	2	NC	NC	NC	NC	5.81	2	NC	NC	South
Hesperiidae	*Nastra*	*Nastra lherminier*	9	NC	NC	NC	NC	2.1	4	NC	NC	Mid-latitude
Hesperiidae	*Nastra*	*Nastra neamathla**	4	NC	NC	NC	NC	6.36	3	NC	NC	South
Hesperiidae	*Nastra*	*Nastra perigenes**	3	NC	NC	NC	NC	10.45	2	NC	NC	South
Hesperiidae	*Notamblyscirtes*	*Notamblyscirtes simius**	4	NC	NC	NC	NC	5.48	2	NC	NC	Mid-latitude
Hesperiidae	*Nyctelius*	*Nyctelius nyctelius**	1	NC	NC	NC	NC	3.91	NC	NC	NC	South
Hesperiidae	*Oarisma*	*Oarisma aurantiaca**	2	NC	NC	NC	NC	1.96	2	NC	NC	South
Hesperiidae	*Oarisma*	*Oarisma garita**	30	0.85	0	0.65	1.5	6.3	12	47	0.26	Mid-latitude
Hesperiidae	*Oarisma*	*Oarisma minima**	5	NC	NC	NC	NC	1.96	1	NC	NC	South
Hesperiidae	*Oarisma*	*Oarisma poweshiek*	5	NC	NC	NC	NC	3.62	2	NC	NC	Mid-latitude
Hesperiidae	*Ochlodes*	*Ochlodes agricola*	7	NC	NC	NC	NC	4.5	2	NC	NC	South
Hesperiidae	*Ochlodes*	*Ochlodes sylvanoides*	79	0.91	0	0.45	2.5	4.78	25	44	0.56	Mid-latitude
Hesperiidae	*Ochlodes*	*Ochlodes yuma*	4	NC	NC	NC	NC	0	4	NC	NC	South
Hesperiidae	*Oligoria*	*Oligoria maculata*	12	0.32	0	NC	0.3	1.85	3	4	0.77	South
Hesperiidae	*Oligoria*	*Oligoria percosius**	0	NC	NC	NC	NC	NC	NC	NC	NC	South

Hesperiidae	*Panoquina*	*Panoquina errans**	3	NC	NC	NC	NC	2.75	3	NC	NC	South
Hesperiidae	*Panoquina*	*Panoquina hecebolus**	3	NC	NC	NC	NC	2.75	2	NC	NC	South
Hesperiidae	*Panoquina*	*Panoquina ocola**	8	NC	NC	NC	NC	2.04	3	NC	NC	South
Hesperiidae	*Panoquina*	*Panoquina panoquin*	13	0.42	0	NC	1.1	1.85	4	7	0.59	South
Hesperiidae	*Panoquina*	*Panoquina panoquinoides**	4	NC	NC	NC	NC	0	3	NC	NC	South
Hesperiidae	*Paratrytone*	*Paratrytone snowi*	5	NC	NC	NC	NC	0.31	3	NC	NC	North/alpine
Hesperiidae	*Pellicia*	*Pellicia arina**	0	NC	NC	NC	NC	NC	NC	NC	NC	South
Hesperiidae	*Phocides*	*Phocides batabano**	2	NC	NC	NC	NC	6.57	1	NC	NC	South
Hesperiidae	*Phocides*	*Phocides lilea**	4	NC	NC	NC	NC	6.12	1	NC	NC	South
Hesperiidae	*Pholisora*	*Pholisora catullus**	42	0.9	0.01	0.47	2.6	0.92	21	162	0.13	Mid-latitude
Hesperiidae	*Pholisora*	*Pholisora mejicanus**	3	NC	NC	NC	NC	0	3	NC	NC	South
Hesperiidae	*Piruna*	*Piruna aea**	3	NC	NC	NC	NC	7.07	2	NC	NC	South
Hesperiidae	*Piruna*	*Piruna haferniki**	0	NC	NC	NC	NC	NC	NC	NC	NC	South
Hesperiidae	*Piruna*	*Piruna pirus*	2	NC	NC	NC	NC	7.14	2	NC	NC	South
Hesperiidae	*Piruna*	*Piruna polingii**	5	NC	NC	NC	NC	0	4	NC	NC	South
Hesperiidae	*Poanes*	*Poanes aaroni*	5	NC	NC	NC	NC	1.69	1	NC	NC	South
Hesperiidae	*Poanes*	*Poanes massasoit*	2	NC	NC	NC	NC	1.69	2	NC	NC	Mid-latitude
Hesperiidae	*Poanes*	*Poanes viator*	21	0.73	0	0.78	1.2	2.6	6	10	0.58	Mid-latitude
Hesperiidae	*Poanes*	*Poanes yehl*	7	NC	NC	NC	NC	7.34	2	NC	NC	South
Hesperiidae	*Polites*	*Polites baracoa**	4	NC	NC	NC	NC	0.46	2	NC	NC	South
Hesperiidae	*Polites*	*Polites carus**	0	NC	NC	NC	NC	NC	NC	NC	NC	South
Hesperiidae	*Polites*	*Polites draco*	33	0.84	0.01	0.76	1.4	1.53	10	22	0.45	North/alpine
Hesperiidae	*Polites*	*Polites egeremet*	35	0.27	0	0.24	0.6	0.72	6	16	0.38	Mid-latitude
Hesperiidae	*Polites*	*Polites mardon*	6	NC	NC	NC	NC	0.31	2	NC	NC	South
Hesperiidae	*Polites*	*Polites mystic*	38	0.5	0	0.69	0.5	2.94	6	12	0.51	Mid-latitude
Hesperiidae	*Polites*	*Polites origenes*	19	0.68	0	0.15	0.5	1.53	6	12	0.51	Mid-latitude
Hesperiidae	*Polites*	*Polites otho**	13	0.15	0	NC	0.2	0.98	2	2	1	South
Hesperiidae	*Polites*	*Polites peckius*	34	0.68	0.01	0.91	3.9	2.2	11	35	0.32	Mid-latitude
Hesperiidae	*Polites*	*Polites rhesus**	6	NC	NC	NC	NC	0.46	2	NC	NC	Mid-latitude
Hesperiidae	*Polites*	*Polites sabuleti**	26	0.73	0.01	0.64	1.7	1.49	7	9	0.76	Mid-latitude
Hesperiidae	*Polites*	*Polites themistocles*	50	0.8	0.01	0.44	1.8	1.83	9	11	0.82	Mid-latitude
Hesperiidae	*Polygonus*	*Polygonus arizonensis**	0	NC	NC	NC	NC	NC	NC	NC	NC	South

Hesperiidae	*Polygonus*	*Polygonus leo**	2	NC	NC	NC	NC	4.92	1	NC	NC	South
Hesperiidae	*Pseudocopaeodes*	*Pseudocopaeodes eunus**	3	NC	NC	NC	NC	0	2	NC	NC	South
Hesperiidae	*Pyrgus*	*Pyrgus centaureae*	46	0.69	0	0.97	1.4	2.82	9	17	0.54	North/alpine
Hesperiidae	*Pyrgus*	*Pyrgus ruralis*	2	NC	NC	NC	NC	4.13	2	NC	NC	Mid-latitude
Hesperiidae	*Pyrgus*	*Pyrgus scriptura**	2	NC	NC	NC	NC	2.82	1	NC	NC	South
Hesperiidae	*Pyrgus*	*Pyrgus xanthus*	3	NC	NC	NC	NC	8.8	3	NC	NC	North/alpine
Hesperiidae	*Quasimellana*	*Quasimellana eulogius**	1	NC	NC	NC	NC	2.29	NC	NC	NC	South
Hesperiidae	*Spicauda*	*Spicauda atelis**	2	NC	NC	NC	NC	5.2	2	NC	NC	South
Hesperiidae	*Spicauda*	*Spicauda procne**	1	NC	NC	NC	NC	5.65	NC	NC	NC	South
Hesperiidae	*Stallingsia*	*Stallingsia maculosus**	2	NC	NC	NC	NC	6.12	1	NC	NC	South
Hesperiidae	*Staphylus*	*Staphylus ceos**	6	NC	NC	NC	NC	3.82	3	NC	NC	South
Hesperiidae	*Staphylus*	*Staphylus hayhurstii*	4	NC	NC	NC	NC	3.82	1	NC	NC	Mid-latitude
Hesperiidae	*Staphylus*	*Staphylus mazans**	2	NC	NC	NC	NC	5.05	1	NC	NC	South
Hesperiidae	*Stinga*	*Stinga morrisoni**	2	NC	NC	NC	NC	8.1	2	NC	NC	South
Hesperiidae	*Synapte*	*Synapte pecta**	0	NC	NC	NC	NC	NC	NC	NC	NC	South
Hesperiidae	*Systasea*	*Systasea pulverulenta**	2	NC	NC	NC	NC	3.37	2	NC	NC	South
Hesperiidae	*Systasea*	*Systasea zampa**	4	NC	NC	NC	NC	5.98	1	NC	NC	South
Hesperiidae	*Telegonus*	*Telegonus anausis**	0	NC	NC	NC	NC	NC	NC	NC	NC	South
Hesperiidae	*Telegonus*	*Telegonus catemacoensis**	2	NC	NC	NC	NC	4.89	2	NC	NC	South
Hesperiidae	*Telegonus*	*Telegonus cellus**	11	0.85	0	NC	1.4	4.14	6	8	0.75	South
Hesperiidae	*Thorybes*	*Thorybes bathyllus*	19	0.78	0	NC	0.9	4.74	7	13	0.55	South
Hesperiidae	*Thorybes*	*Thorybes confusis*	8	NC	NC	NC	NC	3.27	2	NC	NC	South
Hesperiidae	*Thorybes*	*Thorybes diversus*	6	NC	NC	NC	NC	3.06	2	NC	NC	South
Hesperiidae	*Thorybes*	*Thorybes lyciades*	17	0.4	0	0.11	0.7	4	3	3	1	South
Hesperiidae	*Thorybes*	*Thorybes nevada*	7	NC	NC	NC	NC	2.6	2	NC	NC	South
Hesperiidae	*Thorybes*	*Thorybes pylades*	61	0.8	0.01	0.96	2.8	5.27	16	32	0.49	Mid-latitude
Hesperiidae	*Thymelicus*	*Thymelicus lineola*	176	0.31	0	0.05	0.5	9.54	8	20	0.39	Mid-latitude
Hesperiidae	*Timochares*	*Timochares ruptifasciata**	2	NC	NC	NC	NC	4.89	2	NC	NC	South
Hesperiidae	*Urbanus*	*Urbanus proteus**	8	NC	NC	NC	NC	5.44	3	NC	NC	South
Hesperiidae	*Vernia*	*Vernia verna*	14	0.87	0.02	0.89	4.6	2.61	8	14	0.58	South
Hesperiidae	*Zestusa*	*Zestusa dorus**	3	NC	NC	NC	NC	NC	3	NC	NC	South
Lycaenidae	*Agriades*	*Agriades glandon*	143	0.91	0.01	0.68	3.2	0.61	37	110	0.34	North/alpine

Lycaenidae	*Agriades*	*Agriades optilete*	61	0.67	0	0.02	0.8	2.67	18	60	0.3	North/alpine
Lycaenidae	*Agriades*	*Agriades podarce*	30	0.89	0	0.39	1	0.61	13	44	0.3	North/alpine
Lycaenidae	*Atlides*	*Atlides halesus**	4	NC	NC	NC	NC	6.14	4	NC	NC	South
Lycaenidae	*Brephidium*	*Brephidium exilis**	28	0.14	0	0.01	0.6	0.15	3	4	0.76	South
Lycaenidae	*Brephidium*	*Brephidium pseudofea*	1	NC	NC	NC	NC	0.15	NC	NC	NC	South
Lycaenidae	*Callophrys*	*Callophrys affinis**	8	NC	NC	NC	NC	0	3	NC	NC	Mid-latitude
Lycaenidae	*Callophrys*	*Callophrys augustinus**	34	0.67	0.01	0.55	1.6	0.69	9	21	0.43	Mid-latitude
Lycaenidae	*Callophrys*	*Callophrys dumetorum*	11	0.78	0	NC	0.4	1.12	5	8	0.65	South
Lycaenidae	*Callophrys*	*Callophrys eryphon*	29	0.07	0	0.08	2	0.31	2	2	1	Mid-latitude
Lycaenidae	*Callophrys*	*Callophrys fotis*	1	NC	NC	NC	NC	0	NC	NC	NC	South
Lycaenidae	*Callophrys*	*Callophrys gryneus**	59	0.91	0.01	0.88	1.8	0.31	18	34	0.53	Mid-latitude
Lycaenidae	*Callophrys*	*Callophrys henrici*	20	0.27	0	0	0.5	2.6	2	2	1	Mid-latitude
Lycaenidae	*Callophrys*	*Callophrys hesseli*	3	NC	NC	NC	NC	1.07	1	NC	NC	Mid-latitude
Lycaenidae	*Callophrys*	*Callophrys irus*	5	NC	NC	NC	NC	2.33	4	NC	NC	Mid-latitude
Lycaenidae	*Callophrys*	*Callophrys johnsoni*	3	NC	NC	NC	NC	1.05	1	NC	NC	Mid-latitude
Lycaenidae	*Callophrys*	*Callophrys lanoraieensis*	17	0.44	0	0	0.2	1.05	2	2	1	North/alpine
Lycaenidae	*Callophrys*	*Callophrys loki**	0	NC	NC	NC	NC	NC	NC	NC	NC	South
Lycaenidae	*Callophrys*	*Callophrys mcfarlandi**	1	NC	NC	NC	NC	0	NC	NC	NC	South
Lycaenidae	*Callophrys*	*Callophrys mossii*	9	NC	NC	NC	NC	0	3	NC	NC	Mid-latitude
Lycaenidae	*Callophrys*	*Callophrys muiri**	1	NC	NC	NC	NC	1.12	NC	NC	NC	South
Lycaenidae	*Callophrys*	*Callophrys niphon*	9	NC	NC	NC	NC	1.05	2	NC	NC	Mid-latitude
Lycaenidae	*Callophrys*	*Callophrys polios*	21	0.73	0	0.47	1.1	0	4	4	1	Mid-latitude
Lycaenidae	*Callophrys*	*Callophrys sheridanii*	3	NC	NC	NC	NC	2.55	2	NC	NC	North/alpine
Lycaenidae	*Callophrys*	*Callophrys spinetorum**	2	NC	NC	NC	NC	0	1	NC	NC	Mid-latitude
Lycaenidae	*Callophrys*	*Callophrys viridis*	1	NC	NC	NC	NC	6.01	NC	NC	NC	South
Lycaenidae	*Callophrys*	*Callophrys xami**	0	NC	NC	NC	NC	NC	NC	NC	NC	South
Lycaenidae	*Calycopis*	*Calycopis cecrops*	41	0.8	0.01	0.13	3.1	0.15	14	34	0.42	Mid-latitude
Lycaenidae	*Calycopis*	*Calycopis isobeon**	7	NC	NC	NC	NC	9.79	5	NC	NC	South
Lycaenidae	*Celastrina*	*Celastrina echo**	33	0	0	0	0	0	1	NC	NC	Mid-latitude
Lycaenidae	*Celastrina*	*Celastrina humulus*	4	NC	NC	NC	NC	0	2	NC	NC	South
Lycaenidae	*Celastrina*	*Celastrina idella*	6	NC	NC	NC	NC	0	3	NC	NC	South
Lycaenidae	*Celastrina*	*Celastrina ladon*	29	0.38	0	0	0.5	0	7	21	0.33	Mid-latitude

Lycaenidae	*Celastrina*	*Celastrina lucia*	42	0.05	0	0	0.4	0	2	2	1	North/alpine
Lycaenidae	*Celastrina*	*Celastrina neglecta*	23	0.09	0	0	0.2	0	2	2	1	Mid-latitude
Lycaenidae	*Celastrina*	*Celastrina neglectamajor*	1	NC	NC	NC	NC	0	NC	NC	NC	South
Lycaenidae	*Celastrina*	*Celastrina nigra*	5	NC	NC	NC	NC	0	2	NC	NC	South
Lycaenidae	*Celastrina*	*Celastrina serotina*	1	NC	NC	NC	NC	5.2	NC	NC	NC	North/alpine
Lycaenidae	*Chlorostrymon*	*Chlorostrymon maesites**	0	NC	NC	NC	NC	NC	NC	NC	NC	South
Lycaenidae	*Chlorostrymon*	*Chlorostrymon simaethis**	1	NC	NC	NC	NC	0.61	NC	NC	NC	South
Lycaenidae	*Cupido*	*Cupido amyntula*	131	0.89	0.01	0.12	2.3	1.18	37	218	0.17	Mid-latitude
Lycaenidae	*Cupido*	*Cupido comyntas**	57	0.41	0	0.04	1.1	3.54	12	32	0.38	Mid-latitude
Lycaenidae	*Cyanophrys*	*Cyanophrys miserabilis**	2	NC	NC	NC	NC	0.61	1	NC	NC	South
Lycaenidae	*Cyclargus*	*Cyclargus ammon**	1	NC	NC	NC	NC	0.61	NC	NC	NC	South
Lycaenidae	*Cyclargus*	*Cyclargus thomasi**	7	NC	NC	NC	NC	6.22	3	NC	NC	South
Lycaenidae	*Echinargus*	*Echinargus isola**	46	0.32	0	0.1	0.3	6.27	6	10	0.58	South
Lycaenidae	*Electrostrymon*	*Electrostrymon angelia**	1	NC	NC	NC	NC	4.5	NC	NC	NC	South
Lycaenidae	*Erora*	*Erora laeta*	9	NC	NC	NC	NC	0.92	2	NC	NC	Mid-latitude
Lycaenidae	*Erora*	*Erora quaderna**	1	NC	NC	NC	NC	0	NC	NC	NC	South
Lycaenidae	*Eumaeus*	*Eumaeus atala**	4	NC	NC	NC	NC	0.47	1	NC	NC	South
Lycaenidae	*Euphilotes*	*Euphilotes allyni**	45	0.13	0	NC	0.3	0	3	3	0.86	South
Lycaenidae	*Euphilotes*	*Euphilotes ancilla*	40	0.91	0.01	0.61	3.7	0	17	46	0.37	Mid-latitude
Lycaenidae	*Euphilotes*	*Euphilotes battoides*	13	0.85	0.01	0.53	3.5	0	7	11	0.66	North/alpine
Lycaenidae	*Euphilotes*	*Euphilotes baueri*	4	NC	NC	NC	NC	0	4	NC	NC	South
Lycaenidae	*Euphilotes*	*Euphilotes bernardino**	5	NC	NC	NC	NC	0.15	2	NC	NC	South
Lycaenidae	*Euphilotes*	*Euphilotes centralis*	5	NC	NC	NC	NC	0.15	1	NC	NC	South
Lycaenidae	*Euphilotes*	*Euphilotes columbiae*	1	NC	NC	NC	NC	0.92	NC	NC	NC	South
Lycaenidae	*Euphilotes*	*Euphilotes ellisii*	4	NC	NC	NC	NC	0	2	NC	NC	South
Lycaenidae	*Euphilotes*	*Euphilotes enoptes**	31	0.87	0.01	0.26	1.7	0	13	44	0.3	South
Lycaenidae	*Euphilotes*	*Euphilotes glaucon*	8	NC	NC	NC	NC	0.95	5	NC	NC	North/alpine
Lycaenidae	*Euphilotes*	*Euphilotes heracleoides*	0	NC	NC	NC	NC	NC	NC	NC	NC	South
Lycaenidae	*Euphilotes*	*Euphilotes leona*	6	NC	NC	NC	NC	0	1	NC	NC	South
Lycaenidae	*Euphilotes*	*Euphilotes mojave**	7	NC	NC	NC	NC	0.16	6	NC	NC	South
Lycaenidae	*Euphilotes*	*Euphilotes oakleyi*	0	NC	NC	NC	NC	NC	NC	NC	NC	South
Lycaenidae	*Euphilotes*	*Euphilotes pallescens*	24	0.37	0	0.61	0.6	0.15	4	5	0.81	South

Lycaenidae	*Euphilotes*	*Euphilotes rita**	19	0.88	0.01	0.17	2.1	0.16	9	10	0.91	South
Lycaenidae	*Euphilotes*	*Euphilotes spaldingi*	11	0.78	0	0.49	0.6	1.68	5	8	0.65	North/alpine
Lycaenidae	*Euphilotes*	*Euphilotes speciosa*	3	NC	NC	NC	NC	0	2	NC	NC	South
Lycaenidae	*Euphilotes*	*Euphilotes stanfordorum**	3	NC	NC	NC	NC	0	3	NC	NC	South
Lycaenidae	*Feniseca*	*Feniseca tarquinius*	161	0.66	0	0.12	1.6	9.52	36	127	0.28	Mid-latitude
Lycaenidae	*Glaucopsyche*	*Glaucopsyche lygdamus**	250	0.78	0	0.27	7.7	2.91	94	599	0.16	Mid-latitude
Lycaenidae	*Glaucopsyche*	*Glaucopsyche piasus*	5	NC	NC	NC	NC	9.31	4	NC	NC	Mid-latitude
Lycaenidae	*Habrodais*	*Habrodais grunus*	17	0.71	0	0.06	0.6	4.42	7	16	0.43	South
Lycaenidae	*Hemiargus*	*Hemiargus ceraunus**	35	0.91	0.01	0.58	2.3	2.26	15	35	0.43	South
Lycaenidae	*Hypaurotis*	*Hypaurotis crysalus*	3	NC	NC	NC	NC	0	3	NC	NC	South
Lycaenidae	*Icaricia*	*Icaricia acmon**	53	0.96	0.01	0.5	3.2	0.15	31	172	0.18	South
Lycaenidae	*Icaricia*	*Icaricia chlorina*	2	NC	NC	NC	NC	0.31	2	NC	NC	South
Lycaenidae	*Icaricia*	*Icaricia cotundra*	1	NC	NC	NC	NC	5.58	NC	NC	NC	North/alpine
Lycaenidae	*Icaricia*	*Icaricia dedeckera*	0	NC	NC	NC	NC	NC	NC	NC	NC	South
Lycaenidae	*Icaricia*	*Icaricia emigdionis*	6	NC	NC	NC	NC	3.52	4	NC	NC	South
Lycaenidae	*Icaricia*	*Icaricia icarioides**	83	0.94	0.02	0.5	4.9	0	41	81	0.51	Mid-latitude
Lycaenidae	*Icaricia*	*Icaricia lupini**	158	0.95	0.01	0.34	3.2	0	55	131	0.42	Mid-latitude
Lycaenidae	*Icaricia*	*Icaricia monticola*	8	NC	NC	NC	NC	0.76	3	NC	NC	South
Lycaenidae	*Icaricia*	*Icaricia neurona*	11	0.56	0	0.7	0.9	2.91	3	3	1	South
Lycaenidae	*Icaricia*	*Icaricia saepiolus*	147	0.87	0.01	0.52	2.5	2.91	34	94	0.36	Mid-latitude
Lycaenidae	*Icaricia*	*Icaricia shasta*	46	0.94	0.01	0.24	1.5	0	21	44	0.47	Mid-latitude
Lycaenidae	*Leptotes*	*Leptotes cassius**	24	0.76	0	0.68	0.6	0.46	6	9	0.68	South
Lycaenidae	*Leptotes*	*Leptotes marina**	34	0.37	0	0.11	0.6	1.79	8	28	0.28	South
Lycaenidae	*Lycaena*	*Lycaena cupreus*	29	0.76	0	1	0.9	4.52	5	5	1	North/alpine
Lycaenidae	*Lycaena*	*Lycaena phlaeas*	20	0.74	0	0.89	0.8	9.33	5	6	0.84	Mid-latitude
Lycaenidae	*Ministrymon*	*Ministrymon clytie**	0	NC	NC	NC	NC	NC	NC	NC	NC	South
Lycaenidae	*Ministrymon*	*Ministrymon janevicroy**	0	NC	NC	NC	NC	NC	NC	NC	NC	South
Lycaenidae	*Ministrymon*	*Ministrymon leda**	1	NC	NC	NC	NC	5.81	NC	NC	NC	South
Lycaenidae	*Parrhasius*	*Parrhasius m-album*	3	NC	NC	NC	NC	2.91	3	NC	NC	Mid-latitude
Lycaenidae	*Pendantus*	*Pendantus guzanta**	0	NC	NC	NC	NC	NC	NC	NC	NC	South
Lycaenidae	*Philotes*	*Philotes sonorensis**	4	NC	NC	NC	NC	6.57	2	NC	NC	South
Lycaenidae	*Plebejus*	*Plebejus anna*	9	NC	NC	NC	NC	0	4	NC	NC	Mid-latitude

Lycaenidae	*Plebejus*	*Plebejus fridayi*	5	NC	NC	NC	NC	0	3	NC	NC	South
Lycaenidae	*Plebejus*	*Plebejus idas*	110	0.59	0	0.68	3	0	19	34	0.56	North/alpine
Lycaenidae	*Plebejus*	*Plebejus melissa**	39	0.6	0	0.04	0.5	0.49	8	23	0.35	Mid-latitude
Lycaenidae	*Plebejus*	*Plebejus samuelis*	1	NC	NC	NC	NC	2.6	NC	NC	NC	Mid-latitude
Lycaenidae	*Polyommatus*	*Polyommatus icarus*	8	NC	NC	NC	NC	6.36	1	NC	NC	Mid-latitude
Lycaenidae	*Satyrium*	*Satyrium acadica*	23	0.55	0	0.34	0.5	3.25	5	8	0.64	Mid-latitude
Lycaenidae	*Satyrium*	*Satyrium alcestis*	7	NC	NC	NC	NC	2.62	2	NC	NC	South
Lycaenidae	*Satyrium*	*Satyrium auretorum**	3	NC	NC	NC	NC	4.12	3	NC	NC	South
Lycaenidae	*Satyrium*	*Satyrium behrii*	17	0.66	0.01	0	2.1	1.68	7	19	0.37	Mid-latitude
Lycaenidae	*Satyrium*	*Satyrium calanus*	15	0.86	0	0.49	0.8	0	7	9	0.77	Mid-latitude
Lycaenidae	*Satyrium*	*Satyrium californica*	19	0.53	0	1	0.9	1.53	5	8	0.64	Mid-latitude
Lycaenidae	*Satyrium*	*Satyrium caryaevorus*	5	NC	NC	NC	NC	1.53	3	NC	NC	Mid-latitude
Lycaenidae	*Satyrium*	*Satyrium edwardsii*	10	0.64	0	NC	0.5	1.99	3	3	1	Mid-latitude
Lycaenidae	*Satyrium*	*Satyrium favonius*	13	0.86	0.01	NC	1.8	1.14	8	25	0.33	Mid-latitude
Lycaenidae	*Satyrium*	*Satyrium fuliginosa*	8	NC	NC	NC	NC	2.15	5	NC	NC	South
Lycaenidae	*Satyrium*	*Satyrium ilavia*	1	NC	NC	NC	NC	3.72	NC	NC	NC	South
Lycaenidae	*Satyrium*	*Satyrium kingi*	2	NC	NC	NC	NC	3.72	2	NC	NC	South
Lycaenidae	*Satyrium*	*Satyrium liparops*	10	0.93	0	NC	1.3	1.99	7	9	0.75	Mid-latitude
Lycaenidae	*Satyrium*	*Satyrium polingi**	2	NC	NC	NC	NC	3.51	1	NC	NC	South
Lycaenidae	*Satyrium*	*Satyrium saepium**	15	0.83	0.01	0.11	2.9	1.14	8	22	0.36	Mid-latitude
Lycaenidae	*Satyrium*	*Satyrium semiluna*	9	NC	NC	NC	NC	0	4	NC	NC	Mid-latitude
Lycaenidae	*Satyrium*	*Satyrium sylvinus*	16	0.46	0	0.95	0.2	3.51	2	2	1	Mid-latitude
Lycaenidae	*Satyrium*	*Satyrium tetra**	2	NC	NC	NC	NC	3.14	2	NC	NC	South
Lycaenidae	*Satyrium*	*Satyrium titus*	14	0.4	0	NC	0.4	8.67	4	7	0.59	Mid-latitude
Lycaenidae	*Strymon*	*Strymon acis**	1	NC	NC	NC	NC	1.15	NC	NC	NC	South
Lycaenidae	*Strymon*	*Strymon alea**	0	NC	NC	NC	NC	NC	NC	NC	NC	South
Lycaenidae	*Strymon*	*Strymon avalona*	4	NC	NC	NC	NC	5.36	1	NC	NC	South
Lycaenidae	*Strymon*	*Strymon bazochii**	0	NC	NC	NC	NC	NC	NC	NC	NC	South
Lycaenidae	*Strymon*	*Strymon istapa**	2	NC	NC	NC	NC	4.89	1	NC	NC	South
Lycaenidae	*Strymon*	*Strymon limenia**	0	NC	NC	NC	NC	NC	NC	NC	NC	South
Lycaenidae	*Strymon*	*Strymon martialis**	1	NC	NC	NC	NC	1.15	NC	NC	NC	South
Lycaenidae	*Strymon*	*Strymon melinus**	62	0.5	0	0.22	0.8	3.37	14	27	0.51	Mid-latitude

Lycaenidae	*Strymon*	*Strymon rufofusca**	0	NC	NC	NC	NC	NC	NC	NC	NC	South
Lycaenidae	*Strymon*	*Strymon yojoa**	0	NC	NC	NC	NC	NC	NC	NC	NC	South
Lycaenidae	*Tharsalea*	*Tharsalea arota**	14	0.87	0.01	0.74	1.5	1.7	8	22	0.37	South
Lycaenidae	*Tharsalea*	*Tharsalea dione*	11	0.89	0	NC	0.8	0	6	7	0.91	Mid-latitude
Lycaenidae	*Tharsalea*	*Tharsalea dorcas*	49	0.45	0	0.64	0.5	0	8	23	0.35	North/alpine
Lycaenidae	*Tharsalea*	*Tharsalea dospassosi*	6	NC	NC	NC	NC	1.33	1	NC	NC	North/alpine
Lycaenidae	*Tharsalea*	*Tharsalea editha*	8	NC	NC	NC	NC	2.45	4	NC	NC	Mid-latitude
Lycaenidae	*Tharsalea*	*Tharsalea epixanthe*	16	0.72	0	0.68	0.8	2.48	5	6	0.84	Mid-latitude
Lycaenidae	*Tharsalea*	*Tharsalea gorgon*	10	0.51	0.01	NC	1.5	0	3	3	0.87	South
Lycaenidae	*Tharsalea*	*Tharsalea helloides*	41	0.72	0	0.66	1	4.89	5	5	1	Mid-latitude
Lycaenidae	*Tharsalea*	*Tharsalea hermes**	3	NC	NC	NC	NC	2.48	2	NC	NC	South
Lycaenidae	*Tharsalea*	*Tharsalea heteronea*	29	0.78	0	0.59	1.1	4.07	11	23	0.48	Mid-latitude
Lycaenidae	*Tharsalea*	*Tharsalea hyllus*	10	0	0	NC	0	3.52	1	NC	NC	Mid-latitude
Lycaenidae	*Tharsalea*	*Tharsalea mariposa*	42	0.49	0	0.33	1.2	2.45	10	34	0.3	Mid-latitude
Lycaenidae	*Tharsalea*	*Tharsalea nivalis*	5	NC	NC	NC	NC	2.82	4	NC	NC	Mid-latitude
Lycaenidae	*Tharsalea*	*Tharsalea rubidus*	23	0.32	0	0.22	0.3	1.33	4	6	0.68	Mid-latitude
Lycaenidae	*Tharsalea*	*Tharsalea xanthoides*	4	NC	NC	NC	NC	3.27	4	NC	NC	South
Lycaenidae	*Zizula*	*Zizula cyna**	0	NC	NC	NC	NC	NC	NC	NC	NC	South
Nymphalidae	*Adelpha*	*Adelpha basiloides**	0	NC	NC	NC	NC	NC	NC	NC	NC	South
Nymphalidae	*Adelpha*	*Adelpha californica*	6	NC	NC	NC	NC	1.53	5	NC	NC	South
Nymphalidae	*Adelpha*	*Adelpha eulalia**	2	NC	NC	NC	NC	1.53	2	NC	NC	South
Nymphalidae	*Aglais*	*Aglais io*	3	NC	NC	NC	NC	5.61	1	NC	NC	Mid-latitude
Nymphalidae	*Aglais*	*Aglais milberti*	20	0.45	0	NC	0.6	5.29	6	16	0.39	Mid-latitude
Nymphalidae	*Anaea*	*Anaea aidea**	3	NC	NC	NC	NC	2.75	3	NC	NC	South
Nymphalidae	*Anaea*	*Anaea andria**	9	NC	NC	NC	NC	2.75	4	NC	NC	Mid-latitude
Nymphalidae	*Anaea*	*Anaea troglodyta**	1	NC	NC	NC	NC	7.03	NC	NC	NC	Mid-latitude
Nymphalidae	*Anartia*	*Anartia fatima**	0	NC	NC	NC	NC	NC	NC	NC	NC	South
Nymphalidae	*Anartia*	*Anartia jatrophae*	7	NC	NC	NC	NC	7.03	3	NC	NC	South
Nymphalidae	*Anthanassa*	*Anthanassa texana*	3	NC	NC	NC	NC	6.27	2	NC	NC	South
Nymphalidae	*Anthanassa*	*Anthanassa tulcis**	3	NC	NC	NC	NC	6.88	1	NC	NC	South
Nymphalidae	*Asterocampa*	*Asterocampa celtis**	34	0.86	0	0.55	1.1	2.29	15	74	0.2	South
Nymphalidae	*Asterocampa*	*Asterocampa clyton**	21	0.8	0.01	0.62	2.2	3.06	9	23	0.39	Mid-latitude

Nymphalidae	*Asterocampa*	*Asterocampa leilia**	12	0.83	0	0	0.9	2.29	7	18	0.38	South
Nymphalidae	*Biblis*	*Biblis aganisa**	1	NC	NC	NC	NC	9.23	NC	NC	NC	South
Nymphalidae	*Boloria*	*Boloria alaskensis*	36	0.76	0	0.35	0.6	6.5	7	8	0.88	North/alpine
Nymphalidae	*Boloria*	*Boloria alberta*	6	NC	NC	NC	NC	0.63	2	NC	NC	North/alpine
Nymphalidae	*Boloria*	*Boloria astarte*	12	0.67	0	NC	0.5	4.96	3	3	1	North/alpine
Nymphalidae	*Boloria*	*Boloria bellona*	17	0.51	0	NC	1	2.82	6	15	0.39	Mid-latitude
Nymphalidae	*Boloria*	*Boloria chariclea*	335	0.8	0	0.35	1.9	4.92	42	146	0.29	North/alpine
Nymphalidae	*Boloria*	*Boloria epithore*	22	0.63	0	0.8	0.8	2.66	6	10	0.58	Mid-latitude
Nymphalidae	*Boloria*	*Boloria eunomia*	96	0.58	0	0	0.7	6.5	18	60	0.3	North/alpine
Nymphalidae	*Boloria*	*Boloria freija*	54	0.52	0	0.55	0.8	0	7	10	0.7	North/alpine
Nymphalidae	*Boloria*	*Boloria frigga*	29	0.84	0.02	0.96	4	2.54	10	16	0.62	North/alpine
Nymphalidae	*Boloria*	*Boloria improba*	19	0.47	0	0	2.5	2.29	6	15	0.39	North/alpine
Nymphalidae	*Boloria*	*Boloria kriemhild*	1	NC	NC	NC	NC	2.29	NC	NC	NC	North/alpine
Nymphalidae	*Boloria*	*Boloria natazhati*	23	0.7	0	0.92	0.3	0	4	4	1	North/alpine
Nymphalidae	*Boloria*	*Boloria polaris*	79	0.61	0	0.85	0.9	0.63	9	10	0.9	North/alpine
Nymphalidae	*Boloria*	*Boloria selene**	31	0.89	0.01	0.57	3.7	5.05	13	44	0.3	Mid-latitude
Nymphalidae	*Cercyonis*	*Cercyonis hypoleuca*	0	NC	NC	NC	NC	NC	NC	NC	NC	South
Nymphalidae	*Cercyonis*	*Cercyonis incognita*	1	NC	NC	NC	NC	2.3	NC	NC	NC	South
Nymphalidae	*Cercyonis*	*Cercyonis meadii**	4	NC	NC	NC	NC	0.17	2	NC	NC	Mid-latitude
Nymphalidae	*Cercyonis*	*Cercyonis oetus*	140	0.58	0	0.1	0.6	5.39	13	21	0.62	Mid-latitude
Nymphalidae	*Cercyonis*	*Cercyonis pegala*	126	0.96	0.02	0.38	4	2.3	63	136	0.46	Mid-latitude
Nymphalidae	*Cercyonis*	*Cercyonis sthenele*	47	0.52	0	0.86	0.9	6.12	8	16	0.51	Mid-latitude
Nymphalidae	*Chlosyne*	*Chlosyne acastus**	15	0.8	0.01	0.89	2.6	4.89	9	35	0.26	Mid-latitude
Nymphalidae	*Chlosyne*	*Chlosyne californica**	2	NC	NC	NC	NC	2.91	2	NC	NC	South
Nymphalidae	*Chlosyne*	*Chlosyne chinatiensis**	0	NC	NC	NC	NC	NC	NC	NC	NC	South
Nymphalidae	*Chlosyne*	*Chlosyne coronado**	0	NC	NC	NC	NC	NC	NC	NC	NC	South
Nymphalidae	*Chlosyne*	*Chlosyne cyneas**	4	NC	NC	NC	NC	1.22	1	NC	NC	South
Nymphalidae	*Chlosyne*	*Chlosyne damoetas*	5	NC	NC	NC	NC	2.45	2	NC	NC	North/alpine
Nymphalidae	*Chlosyne*	*Chlosyne definita**	2	NC	NC	NC	NC	2.45	1	NC	NC	South
Nymphalidae	*Chlosyne*	*Chlosyne endeis**	2	NC	NC	NC	NC	2.6	1	NC	NC	South
Nymphalidae	*Chlosyne*	*Chlosyne flavula*	0	NC	NC	NC	NC	NC	NC	NC	NC	South
Nymphalidae	*Chlosyne*	*Chlosyne fulvia**	3	NC	NC	NC	NC	1.53	1	NC	NC	South

Nymphalidae	*Chlosyne*	*Chlosyne gabbii**	5	NC	NC	NC	NC	4.76	4	NC	NC	South
Nymphalidae	*Chlosyne*	*Chlosyne gorgone*	15	0.81	0	NC	1.4	1.59	6	7	0.87	Mid-latitude
Nymphalidae	*Chlosyne*	*Chlosyne harrisii**	13	0.62	0	NC	0.3	1.59	4	4	0.95	Mid-latitude
Nymphalidae	*Chlosyne*	*Chlosyne hoffmanni*	13	0.29	0	NC	0.3	6.12	3	4	0.77	Mid-latitude
Nymphalidae	*Chlosyne*	*Chlosyne janais**	1	NC	NC	NC	NC	4.89	NC	NC	NC	South
Nymphalidae	*Chlosyne*	*Chlosyne lacinia**	5	NC	NC	NC	NC	2.6	4	NC	NC	South
Nymphalidae	*Chlosyne*	*Chlosyne leanira**	9	NC	NC	NC	NC	4.28	5	NC	NC	South
Nymphalidae	*Chlosyne*	*Chlosyne nycteis*	11	0.87	0	NC	1	1.22	6	10	0.6	Mid-latitude
Nymphalidae	*Chlosyne*	*Chlosyne palla*	28	0.86	0.01	0.67	1.2	4.43	12	43	0.28	Mid-latitude
Nymphalidae	*Chlosyne*	*Chlosyne theona**	2	NC	NC	NC	NC	0.61	2	NC	NC	South
Nymphalidae	*Chlosyne*	*Chlosyne whitneyi*	1	NC	NC	NC	NC	9.22	NC	NC	NC	North/alpine
Nymphalidae	*Cissia*	*Cissia rubricata**	13	0.9	0.04	0.75	10.2	8.47	8	14	0.58	South
Nymphalidae	*Coenonympha*	*Coenonympha california*	257	0.94	0.01	0.55	1.6	8.1	56	184	0.31	South
Nymphalidae	*Coenonympha*	*Coenonympha haydenii*	3	NC	NC	NC	NC	0	3	NC	NC	South
Nymphalidae	*Coenonympha*	*Coenonympha tullia*	27	0.5	0	0.05	0.3	5.35	6	12	0.51	Mid-latitude
Nymphalidae	*Cyllopsis*	*Cyllopsis gemma**	3	NC	NC	NC	NC	5.96	3	NC	NC	South
Nymphalidae	*Cyllopsis*	*Cyllopsis pertepida**	2	NC	NC	NC	NC	5.96	1	NC	NC	South
Nymphalidae	*Cyllopsis*	*Cyllopsis pyracmon**	3	NC	NC	NC	NC	6.08	1	NC	NC	South
Nymphalidae	*Danaus*	*Danaus eresimus**	0	NC	NC	NC	NC	NC	NC	NC	NC	South
Nymphalidae	*Danaus*	*Danaus gilippus**	19	0.65	0	0.22	0.6	6.88	5	8	0.64	South
Nymphalidae	*Danaus*	*Danaus plexippus**	14	0.38	0	NC	0.3	8.33	3	3	0.87	Mid-latitude
Nymphalidae	*Dione*	*Dione incarnata**	16	0.78	0	0.34	0.6	8.71	6	10	0.59	South
Nymphalidae	*Dryas*	*Dryas alcionea**	0	NC	NC	NC	NC	NC	NC	NC	NC	South
Nymphalidae	*Erebia*	*Erebia callias*	7	NC	NC	NC	NC	0	1	NC	NC	North/alpine
Nymphalidae	*Erebia*	*Erebia disa*	13	0.29	0	NC	0.3	2.14	3	4	0.77	North/alpine
Nymphalidae	*Erebia*	*Erebia discoidalis*	14	0.74	0	NC	0.6	5.96	6	12	0.52	North/alpine
Nymphalidae	*Erebia*	*Erebia epipsodea*	38	0.73	0	0.7	1.1	0.15	10	19	0.53	North/alpine
Nymphalidae	*Erebia*	*Erebia fasciata*	18	0.66	0	0.98	0.8	1.48	5	7	0.73	North/alpine
Nymphalidae	*Erebia*	*Erebia lafontainei*	19	0.53	0	0.09	0.3	0.15	5	8	0.64	North/alpine
Nymphalidae	*Erebia*	*Erebia mackinleyensis*	25	0.91	0.01	0.3	1.1	0.92	11	25	0.43	North/alpine
Nymphalidae	*Erebia*	*Erebia magdalena*	3	NC	NC	NC	NC	0	3	NC	NC	North/alpine
Nymphalidae	*Erebia*	*Erebia mancinus*	20	0.58	0.01	0.39	2.9	0.15	7	19	0.37	North/alpine

Nymphalidae	*Erebia*	*Erebia occulta*	14	0	0	0	0	4.72	1	NC	NC	North/alpine
Nymphalidae	*Erebia*	*Erebia pawloskii*	45	0.65	0	0.91	1.9	5.1	9	30	0.31	North/alpine
Nymphalidae	*Erebia*	*Erebia rossii*	36	0.4	0	0	0.5	4.72	6	7	0.9	North/alpine
Nymphalidae	*Erebia*	*Erebia vidleri*	7	NC	NC	NC	NC	0.15	1	NC	NC	North/alpine
Nymphalidae	*Erebia*	*Erebia youngi*	36	0.64	0	0.36	0.8	0.92	7	15	0.47	North/alpine
Nymphalidae	*Eunica*	*Eunica tatila**	0	NC	NC	NC	NC	NC	NC	NC	NC	South
Nymphalidae	*Euphydryas*	*Euphydryas anicia*	44	0.93	0.01	0.7	1.7	0	18	42	0.42	Mid-latitude
Nymphalidae	*Euphydryas*	*Euphydryas chalcedona**	21	0.72	0	0.11	1.8	0	8	11	0.76	South
Nymphalidae	*Euphydryas*	*Euphydryas colon*	8	NC	NC	NC	NC	0	2	NC	NC	Mid-latitude
Nymphalidae	*Euphydryas*	*Euphydryas editha**	65	0.88	0.01	0.27	3.2	4.89	18	24	0.75	Mid-latitude
Nymphalidae	*Euphydryas*	*Euphydryas gillettii*	1	NC	NC	NC	NC	1.29	NC	NC	NC	Mid-latitude
Nymphalidae	*Euphydryas*	*Euphydryas phaeton*	12	0.76	0	NC	1.2	1.76	6	13	0.45	Mid-latitude
Nymphalidae	*Euptoieta*	*Euptoieta claudia**	25	0.72	0	0.05	0.8	8.38	9	26	0.34	Mid-latitude
Nymphalidae	*Euptoieta*	*Euptoieta hegesia**	0	NC	NC	NC	NC	NC	NC	NC	NC	South
Nymphalidae	*Gyrocheilus*	*Gyrocheilus patrobas**	4	NC	NC	NC	NC	3.21	2	NC	NC	South
Nymphalidae	*Heliconius*	*Heliconius charithonia**	3	NC	NC	NC	NC	4.59	1	NC	NC	South
Nymphalidae	*Hermeuptychia*	*Hermeuptychia hermybius**	19	0.2	0	0	0.2	2.82	2	2	1	South
Nymphalidae	*Hermeuptychia*	*Hermeuptychia intricata*	17	0.51	0	0.95	0.5	1.69	5	9	0.54	South
Nymphalidae	*Hermeuptychia*	*Hermeuptychia sosybius**	87	0.44	0	0.51	1	0	12	16	0.74	South
Nymphalidae	*Junonia*	*Junonia grisea**	20	0.52	0	0.23	1.1	0.15	7	21	0.33	South
Nymphalidae	*Junonia*	*Junonia neildi**	3	NC	NC	NC	NC	0	1	NC	NC	South
Nymphalidae	*Junonia*	*Junonia nigrosuffusa**	0	NC	NC	NC	NC	NC	NC	NC	NC	South
Nymphalidae	*Junonia*	*Junonia stemosa**	34	0.84	0	0	2.1	0.29	16	36	0.45	South
Nymphalidae	*Junonia*	*Junonia zonalis**	1	NC	NC	NC	NC	7.8	NC	NC	NC	South
Nymphalidae	*Lethe*	*Lethe anthedon*	66	0.9	0	0.24	1.7	0.76	21	36	0.59	Mid-latitude
Nymphalidae	*Lethe*	*Lethe appalachia*	15	0.42	0	1	0.3	4.28	2	2	1	Mid-latitude
Nymphalidae	*Lethe*	*Lethe creola*	3	NC	NC	NC	NC	0.76	2	NC	NC	South
Nymphalidae	*Lethe*	*Lethe eurydice*	11	0.89	0	NC	0.8	7.65	7	16	0.44	Mid-latitude
Nymphalidae	*Lethe*	*Lethe portlandia*	0	NC	NC	NC	NC	NC	NC	NC	NC	South
Nymphalidae	*Libytheana*	*Libytheana carinenta**	32	0.87	0.01	0.43	1.9	1.59	15	31	0.48	Mid-latitude
Nymphalidae	*Limenitis*	*Limenitis archippus**	22	0.92	0.01	0.88	2.3	0	11	15	0.73	Mid-latitude
Nymphalidae	*Limenitis*	*Limenitis arthemis**	102	0.94	0.02	0.16	4.3	0	55	404	0.14	Mid-latitude

Nymphalidae	*Limenitis*	*Limenitis lorquini**	36	0.76	0	0.7	1.5	0.92	11	29	0.39	Mid-latitude
Nymphalidae	*Limenitis*	*Limenitis weidemeyerii*	6	NC	NC	NC	NC	0.31	4	NC	NC	Mid-latitude
Nymphalidae	*Marpesia*	*Marpesia petreus**	4	NC	NC	NC	NC	3.36	1	NC	NC	South
Nymphalidae	*Megisto*	*Megisto cymela*	203	0.73	0	0.44	1.7	7.32	32	68	0.47	Mid-latitude
Nymphalidae	*Memphis*	*Memphis pithyusa**	0	NC	NC	NC	NC	NC	NC	NC	NC	South
Nymphalidae	*Mestra*	*Mestra amymone**	0	NC	NC	NC	NC	NC	NC	NC	NC	South
Nymphalidae	*Microtia*	*Microtia dymas*	4	NC	NC	NC	NC	3.82	3	NC	NC	South
Nymphalidae	*Microtia*	*Microtia elada**	1	NC	NC	NC	NC	3.82	NC	NC	NC	South
Nymphalidae	*Microtia*	*Microtia perse*	9	NC	NC	NC	NC	5.49	5	NC	NC	South
Nymphalidae	*Myscelia*	*Myscelia ethusa**	0	NC	NC	NC	NC	NC	NC	NC	NC	South
Nymphalidae	*Neonympha*	*Neonympha areolatus*	5	NC	NC	NC	NC	6.58	3	NC	NC	South
Nymphalidae	*Neonympha*	*Neonympha mitchellii*	0	NC	NC	NC	NC	NC	NC	NC	NC	Mid-latitude
Nymphalidae	*Nymphalis*	*Nymphalis antiopa**	58	0.43	0	0.19	0.6	3.91	7	15	0.47	Mid-latitude
Nymphalidae	*Nymphalis*	*Nymphalis californica**	11	0.18	0	NC	0.2	4.93	2	2	1	Mid-latitude
Nymphalidae	*Nymphalis*	*Nymphalis l-album*	60	0.83	0	0.03	1.5	4.95	18	89	0.2	Mid-latitude
Nymphalidae	*Oeneis*	*Oeneis alberta*	26	0.78	0.01	0.8	1.5	3.36	10	37	0.27	Mid-latitude
Nymphalidae	*Oeneis*	*Oeneis alpina*	20	0.51	0	0.76	0.3	0	3	3	1	North/alpine
Nymphalidae	*Oeneis*	*Oeneis bore*	66	0.85	0	0.52	1.5	0	20	62	0.33	North/alpine
Nymphalidae	*Oeneis*	*Oeneis chryxus*	52	0.93	0.01	0.86	1.8	0.15	20	36	0.55	North/alpine
Nymphalidae	*Oeneis*	*Oeneis jutta*	56	0.56	0.01	0.41	1.9	2.91	12	43	0.28	North/alpine
Nymphalidae	*Oeneis*	*Oeneis macounii*	12	0.76	0	NC	0.3	0.15	5	8	0.65	North/alpine
Nymphalidae	*Oeneis*	*Oeneis melissa*	166	0.76	0.01	0.81	3.7	0.31	20	36	0.56	North/alpine
Nymphalidae	*Oeneis*	*Oeneis nevadensis*	12	0.76	0	NC	0.6	0	5	8	0.65	Mid-latitude
Nymphalidae	*Oeneis*	*Oeneis philipi*	9	NC	NC	NC	NC	0	4	NC	NC	North/alpine
Nymphalidae	*Oeneis*	*Oeneis polixenes*	54	0.85	0.02	0.52	4.3	4.89	14	45	0.31	North/alpine
Nymphalidae	*Oeneis*	*Oeneis ridingsii*	44	0.82	0.01	0.56	1.4	0.15	10	20	0.51	Mid-latitude
Nymphalidae	*Oeneis*	*Oeneis tanana*	4	NC	NC	NC	NC	4.74	1	NC	NC	North/alpine
Nymphalidae	*Oeneis*	*Oeneis uhleri*	28	0.91	0.01	0.83	1.7	5.23	13	21	0.62	North/alpine
Nymphalidae	*Paramacera*	*Paramacera allyni**	0	NC	NC	NC	NC	NC	NC	NC	NC	South
Nymphalidae	*Phyciodes*	*Phyciodes batesii*	25	0.81	0.02	0.8	3.6	0	8	16	0.51	Mid-latitude
Nymphalidae	*Phyciodes*	*Phyciodes cocyta*	150	0.81	0.01	0.19	3.8	4.71	35	65	0.54	Mid-latitude
Nymphalidae	*Phyciodes*	*Phyciodes graphica**	4	NC	NC	NC	NC	0.18	2	NC	NC	South

Nymphalidae	*Phyciodes*	*Phyciodes mylitta**	20	0.73	0.01	0.1	3.8	1.52	6	10	0.58	Mid-latitude
Nymphalidae	*Phyciodes*	*Phyciodes orseis*	7	NC	NC	NC	NC	1.99	4	NC	NC	South
Nymphalidae	*Phyciodes*	*Phyciodes pallescens**	3	NC	NC	NC	NC	0.18	2	NC	NC	South
Nymphalidae	*Phyciodes*	*Phyciodes pallida*	32	0.86	0.01	0.52	1.8	1.82	11	23	0.48	South
Nymphalidae	*Phyciodes*	*Phyciodes phaon**	27	0.57	0.01	0.86	1.8	1.99	4	4	1	South
Nymphalidae	*Phyciodes*	*Phyciodes picta**	4	NC	NC	NC	NC	0	3	NC	NC	South
Nymphalidae	*Phyciodes*	*Phyciodes pulchella*	84	0.76	0.01	0.54	3.4	0	20	27	0.75	Mid-latitude
Nymphalidae	*Phyciodes*	*Phyciodes tharos**	49	0.8	0	0.31	0.8	0	10	25	0.41	Mid-latitude
Nymphalidae	*Poladryas*	*Poladryas arachne**	6	NC	NC	NC	NC	3.57	6	NC	NC	South
Nymphalidae	*Poladryas*	*Poladryas minuta**	0	NC	NC	NC	NC	NC	NC	NC	NC	South
Nymphalidae	*Polygonia*	*Polygonia comma*	16	0.34	0	NC	0.3	2.75	3	3	0.87	Mid-latitude
Nymphalidae	*Polygonia*	*Polygonia faunus*	61	0.56	0	0.51	0.6	0	10	16	0.62	Mid-latitude
Nymphalidae	*Polygonia*	*Polygonia gracilis*	38	0.73	0	0.47	1.6	1.83	11	29	0.39	Mid-latitude
Nymphalidae	*Polygonia*	*Polygonia interrogationis**	13	0.15	0	NC	0.2	0	2	2	1	Mid-latitude
Nymphalidae	*Polygonia*	*Polygonia oreas*	8	NC	NC	NC	NC	0.76	3	NC	NC	Mid-latitude
Nymphalidae	*Polygonia*	*Polygonia progne*	22	0.41	0	0	0.8	0.31	6	16	0.39	Mid-latitude
Nymphalidae	*Polygonia*	*Polygonia satyrus*	40	0.76	0	0.35	0.7	8.26	10	28	0.36	Mid-latitude
Nymphalidae	*Siproeta*	*Siproeta stelenes**	4	NC	NC	NC	NC	0.46	3	NC	NC	South
Nymphalidae	*Speyeria*	*Speyeria adiaste*	4	NC	NC	NC	NC	1.96	2	NC	NC	South
Nymphalidae	*Speyeria*	*Speyeria aphrodite*	41	0.43	0	0.94	0.7	1.83	4	5	0.8	Mid-latitude
Nymphalidae	*Speyeria*	*Speyeria atlantis*	46	0.87	0	0.25	0.6	0	17	47	0.37	Mid-latitude
Nymphalidae	*Speyeria*	*Speyeria callippe*	21	0.89	0.01	0.43	3.7	0	10	14	0.72	Mid-latitude
Nymphalidae	*Speyeria*	*Speyeria coronis**	24	0.9	0.03	0.94	5.9	2.14	12	43	0.28	Mid-latitude
Nymphalidae	*Speyeria*	*Speyeria cybele*	70	0.68	0.01	0.86	1.6	3.52	8	14	0.58	Mid-latitude
Nymphalidae	*Speyeria*	*Speyeria diana*	2	NC	NC	NC	NC	0	1	NC	NC	Mid-latitude
Nymphalidae	*Speyeria*	*Speyeria edwardsii*	13	0.77	0.01	0.04	1.5	0	4	4	1	Mid-latitude
Nymphalidae	*Speyeria*	*Speyeria egleis*	8	NC	NC	NC	NC	0.15	5	NC	NC	Mid-latitude
Nymphalidae	*Speyeria*	*Speyeria hesperis*	69	0.17	0.01	0.98	4.9	0.46	6	14	0.43	Mid-latitude
Nymphalidae	*Speyeria*	*Speyeria hydaspe*	32	0.8	0	0.66	0.8	5.22	8	10	0.79	Mid-latitude
Nymphalidae	*Speyeria*	*Speyeria idalia*	4	NC	NC	NC	NC	0.76	2	NC	NC	Mid-latitude
Nymphalidae	*Speyeria*	*Speyeria mormonia*	93	0.83	0.01	0.45	1.8	4.13	21	111	0.19	North/alpine
Nymphalidae	*Speyeria*	*Speyeria nokomis**	6	NC	NC	NC	NC	0	1	NC	NC	South

Nymphalidae	*Speyeria*	*Speyeria zerene*	34	0.91	0.02	0.35	4.9	4.62	21	52	0.4	Mid-latitude
Nymphalidae	*Vanessa*	*Vanessa annabella**	10	0.2	0	NC	0.2	4.51	2	2	1	Mid-latitude
Nymphalidae	*Vanessa*	*Vanessa atalanta**	53	0.75	0	0.09	0.8	4.13	13	21	0.62	Mid-latitude
Nymphalidae	*Vanessa*	*Vanessa cardui**	45	0.39	0	0.41	0.9	4.13	8	20	0.4	Mid-latitude
Nymphalidae	*Vanessa*	*Vanessa virginiensis**	18	0.22	0	NC	0.5	6.18	3	4	0.76	Mid-latitude
Papilionidae	*Battus*	*Battus philenor**	23	0.89	0	0.01	1.5	8.95	12	20	0.61	South
Papilionidae	*Battus*	*Battus polydamas**	0	NC	NC	NC	NC	NC	NC	NC	NC	South
Papilionidae	*Eurytides*	*Eurytides marcellus*	12	0.32	0	NC	0.3	13.06	3	4	0.77	Mid-latitude
Papilionidae	*Heraclides*	*Heraclides anchisiades**	0	NC	NC	NC	NC	NC	NC	NC	NC	South
Papilionidae	*Heraclides*	*Heraclides andraemon**	0	NC	NC	NC	NC	NC	NC	NC	NC	South
Papilionidae	*Heraclides*	*Heraclides caiguanabus**	0	NC	NC	NC	NC	NC	NC	NC	NC	South
Papilionidae	*Heraclides*	*Heraclides cresphontes**	68	0.14	0	0.17	0.3	6.49	4	4	0.94	Mid-latitude
Papilionidae	*Heraclides*	*Heraclides ornythion**	0	NC	NC	NC	NC	NC	NC	NC	NC	South
Papilionidae	*Heraclides*	*Heraclides ponceana**	5	NC	NC	NC	NC	2.26	3	NC	NC	South
Papilionidae	*Heraclides*	*Heraclides rogeri**	0	NC	NC	NC	NC	NC	NC	NC	NC	South
Papilionidae	*Heraclides*	*Heraclides rumiko**	90	0.59	0	0.56	0.7	1.69	10	22	0.45	South
Papilionidae	*Heraclides*	*Heraclides thoas**	0	NC	NC	NC	NC	NC	NC	NC	NC	South
Papilionidae	*Papilio*	*Papilio brevicauda*	11	0.69	0	NC	0.3	4.91	4	4	0.95	North/alpine
Papilionidae	*Papilio*	*Papilio indra*	5	NC	NC	NC	NC	0	1	NC	NC	Mid-latitude
Papilionidae	*Papilio*	*Papilio joanae*	4	NC	NC	NC	NC	0	1	NC	NC	South
Papilionidae	*Papilio*	*Papilio machaon*	46	0.64	0	0.1	2.4	3.21	10	13	0.79	Mid-latitude
Papilionidae	*Papilio*	*Papilio polyxenes**	29	0.55	0	0.73	0.3	3.21	4	4	0.89	Mid-latitude
Papilionidae	*Papilio*	*Papilio zelicaon**	34	0.06	0	0	0.4	6.42	2	2	1	Mid-latitude
Papilionidae	*Parnassius*	*Parnassius behrii*	5	NC	NC	NC	NC	1.78	1	NC	NC	North/alpine
Papilionidae	*Parnassius*	*Parnassius clodius*	16	0.57	0	0.62	0.3	1.78	3	3	1	Mid-latitude
Papilionidae	*Parnassius*	*Parnassius eversmanni*	10	0	0	NC	0	1.07	1	NC	NC	North/alpine
Papilionidae	*Parnassius*	*Parnassius phoebus*	8	NC	NC	NC	NC	0.31	4	NC	NC	North/alpine
Papilionidae	*Parnassius*	*Parnassius smintheus*	25	0.89	0	0.22	0.8	4.33	11	20	0.56	North/alpine
Papilionidae	*Pterourus*	*Pterourus alexiares*	0	NC	NC	NC	NC	NC	NC	NC	NC	South
Papilionidae	*Pterourus*	*Pterourus appalachiensis*	1	NC	NC	NC	NC	1.47	NC	NC	NC	North/alpine
Papilionidae	*Pterourus*	*Pterourus canadensis*	51	0.32	0	0.09	0.8	0	7	17	0.42	North/alpine
Papilionidae	*Pterourus*	*Pterourus eurymedon*	22	0.82	0.01	0.09	1.1	0	7	9	0.79	Mid-latitude

Papilionidae	*Pterourus*	*Pterourus glaucus*	27	0.6	0	0.06	0.8	2.14	9	29	0.31	Mid-latitude
Papilionidae	*Pterourus*	*Pterourus multicaudata**	10	0.87	0	NC	0.6	4.48	6	11	0.53	Mid-latitude
Papilionidae	*Pterourus*	*Pterourus palamedes**	12	0.94	0	0.12	0.9	0	9	31	0.29	South
Papilionidae	*Pterourus*	*Pterourus rutulus*	21	0.86	0	0.3	0.9	4.48	9	21	0.43	South
Papilionidae	*Pterourus*	*Pterourus troilus*	21	0.96	0.01	0	1.4	4.13	16	103	0.16	Mid-latitude
Pieridae	*Abaeis*	*Abaeis boisduvaliana*	0	NC	NC	NC	NC	NC	NC	NC	NC	South
Pieridae	*Abaeis*	*Abaeis mexicana**	8	NC	NC	NC	NC	7.34	3	NC	NC	South
Pieridae	*Abaeis*	*Abaeis nicippe**	43	0.34	0	0.1	0.5	8.56	6	10	0.58	South
Pieridae	*Anteos*	*Anteos clorinde**	1	NC	NC	NC	NC	9.17	NC	NC	NC	South
Pieridae	*Anteos*	*Anteos maerula**	3	NC	NC	NC	NC	10.42	1	NC	NC	South
Pieridae	*Anthocharis*	*Anthocharis cethura**	8	NC	NC	NC	NC	6	4	NC	NC	South
Pieridae	*Anthocharis*	*Anthocharis julia*	17	0.81	0	0.9	0.5	1.99	6	9	0.68	Mid-latitude
Pieridae	*Anthocharis*	*Anthocharis lanceolata**	2	NC	NC	NC	NC	6.56	1	NC	NC	South
Pieridae	*Anthocharis*	*Anthocharis midea*	5	NC	NC	NC	NC	6	4	NC	NC	South
Pieridae	*Anthocharis*	*Anthocharis sara**	16	0.69	0	0	1.1	2.2	7	19	0.37	South
Pieridae	*Anthocharis*	*Anthocharis thoosa*	2	NC	NC	NC	NC	1.99	1	NC	NC	South
Pieridae	*Aphrissa*	*Aphrissa neleis**	2	NC	NC	NC	NC	1.07	1	NC	NC	South
Pieridae	*Aphrissa*	*Aphrissa statira**	5	NC	NC	NC	NC	1.07	2	NC	NC	South
Pieridae	*Archonias*	*Archonias nimbice**	0	NC	NC	NC	NC	NC	NC	NC	NC	South
Pieridae	*Ascia*	*Ascia monuste**	10	0.89	0	NC	0.5	7.14	6	8	0.75	South
Pieridae	*Colias*	*Colias alexandra*	32	0.81	0.01	0.16	7.2	0	7	9	0.78	Mid-latitude
Pieridae	*Colias*	*Colias behrii*	10	0.47	0	NC	0.2	0	2	2	1	North/alpine
Pieridae	*Colias*	*Colias boothii*	12	0.82	0.01	NC	2.8	0	6	8	0.74	North/alpine
Pieridae	*Colias*	*Colias christina*	59	0.8	0.01	0.38	7.8	0	14	18	0.78	Mid-latitude
Pieridae	*Colias*	*Colias eriphyle*	25	0.66	0	0.06	1.4	0	5	7	0.72	Mid-latitude
Pieridae	*Colias*	*Colias eurytheme**	23	0.56	0	0	0.6	0	4	5	0.81	Mid-latitude
Pieridae	*Colias*	*Colias gigantea*	55	0.8	0.01	0.51	2.8	0	16	26	0.6	Mid-latitude
Pieridae	*Colias*	*Colias harfordii*	8	NC	NC	NC	NC	0	3	NC	NC	South
Pieridae	*Colias*	*Colias hecla*	9	NC	NC	NC	NC	0	5	NC	NC	North/alpine
Pieridae	*Colias*	*Colias interior*	68	0.76	0.01	0.13	3.2	0	16	65	0.25	North/alpine
Pieridae	*Colias*	*Colias johanseni*	6	NC	NC	NC	NC	0	2	NC	NC	North/alpine
Pieridae	*Colias*	*Colias meadii*	17	0.88	0.01	0.74	1.1	0	7	8	0.88	North/alpine

Pieridae	*Colias*	*Colias nastes*	29	0.89	0.03	0.24	7.8	0	11	14	0.81	North/alpine
Pieridae	*Colias*	*Colias occidentalis*	9	NC	NC	NC	NC	0	2	NC	NC	Mid-latitude
Pieridae	*Colias*	*Colias palaeno*	86	0.56	0.01	0.61	7.8	0	13	15	0.84	North/alpine
Pieridae	*Colias*	*Colias pelidne*	18	0.81	0.02	NC	3.5	0	10	40	0.25	North/alpine
Pieridae	*Colias*	*Colias philodice*	78	0.65	0	0.46	2	0	21	53	0.39	Mid-latitude
Pieridae	*Colias*	*Colias rankinensis*	2	NC	NC	NC	NC	0	1	NC	NC	North/alpine
Pieridae	*Colias*	*Colias scudderii*	5	NC	NC	NC	NC	0	3	NC	NC	North/alpine
Pieridae	*Colias*	*Colias skinneri*	2	NC	NC	NC	NC	0	1	NC	NC	North/alpine
Pieridae	*Colias*	*Colias vitabunda*	7	NC	NC	NC	NC	4.95	3	NC	NC	North/alpine
Pieridae	*Euchloe*	*Euchloe ausonides*	33	0.65	0	0.66	1.2	2.13	8	9	0.85	Mid-latitude
Pieridae	*Euchloe*	*Euchloe creusa*	25	0.63	0.01	0.39	2.6	0.15	8	22	0.36	North/alpine
Pieridae	*Euchloe*	*Euchloe hyantis*	7	NC	NC	NC	NC	0.15	2	NC	NC	South
Pieridae	*Euchloe*	*Euchloe lotta*	10	0.78	0.01	NC	1.1	3.06	6	15	0.4	Mid-latitude
Pieridae	*Euchloe*	*Euchloe ochracea*	8	NC	NC	NC	NC	3.52	4	NC	NC	North/alpine
Pieridae	*Euchloe*	*Euchloe olympia*	10	0.71	0	NC	0.5	6.81	4	5	0.82	Mid-latitude
Pieridae	*Eurema*	*Eurema daira**	12	0.53	0	NC	0.2	10.4	2	2	1	South
Pieridae	*Ganyra*	*Ganyra josephina**	1	NC	NC	NC	NC	6.19	NC	NC	NC	South
Pieridae	*Glutophrissa*	*Glutophrissa drusilla**	4	NC	NC	NC	NC	10.14	3	NC	NC	South
Pieridae	*Kricogonia*	*Kricogonia lyside**	21	0.97	0	0	0.9	6.27	17	124	0.14	South
Pieridae	*Nathalis*	*Nathalis iole**	35	0.11	0	0.44	0.3	9.14	3	4	0.76	South
Pieridae	*Neophasia*	*Neophasia menapia*	58	0.84	0.01	0.75	2.9	6.58	19	102	0.19	Mid-latitude
Pieridae	*Neophasia*	*Neophasia terlooii**	2	NC	NC	NC	NC	6.37	2	NC	NC	South
Pieridae	*Phoebis*	*Phoebis agarithe**	13	0.15	0	NC	0.3	5.36	2	2	1	South
Pieridae	*Phoebis*	*Phoebis philea*	3	NC	NC	NC	NC	5.36	2	NC	NC	South
Pieridae	*Phoebis*	*Phoebis sennae**	17	0.74	0	0.93	0.8	0.92	6	10	0.59	South
Pieridae	*Pieris*	*Pieris angelika*	39	0.58	0	0.27	1.7	0	9	21	0.43	North/alpine
Pieridae	*Pieris*	*Pieris marginalis*	76	0.73	0.01	0.47	2.6	0	20	27	0.75	Mid-latitude
Pieridae	*Pieris*	*Pieris oleracea*	33	0.88	0	0.13	1.6	6.32	15	21	0.71	North/alpine
Pieridae	*Pieris*	*Pieris rapae*	416	0.57	0	0.08	2	1.01	30	84	0.36	Mid-latitude
Pieridae	*Pieris*	*Pieris virginiensis*	11	0.6	0	NC	0.7	7.07	4	6	0.69	Mid-latitude
Pieridae	*Pontia*	*Pontia beckerii*	12	0.53	0	NC	0.2	4.17	2	2	1	South
Pieridae	*Pontia*	*Pontia occidentalis*	50	0.62	0	0.22	0.8	4.17	11	20	0.56	Mid-latitude

Pieridae	*Pontia*	*Pontia protodice**	48	0.67	0	0.01	1.4	6.54	9	17	0.54	South
Pieridae	*Pontia*	*Pontia sisymbrii*	17	0.92	0.02	0.67	3.8	4.42	12	59	0.2	South
Pieridae	*Pyrisitia*	*Pyrisitia dina**	3	NC	NC	NC	NC	5.48	2	NC	NC	South
Pieridae	*Pyrisitia*	*Pyrisitia lisa**	16	0.87	0	0.03	0.8	5.48	8	17	0.46	Mid-latitude
Pieridae	*Pyrisitia*	*Pyrisitia nise**	5	NC	NC	NC	NC	7.57	4	NC	NC	South
Pieridae	*Pyrisitia*	*Pyrisitia proterpia**	2	NC	NC	NC	NC	6.73	2	NC	NC	South
Pieridae	*Zerene*	*Zerene cesonia**	11	0.78	0	NC	0.9	2.61	4	4	1	South
Pieridae	*Zerene*	*Zerene eurydice*	7	NC	NC	NC	NC	8.6	1	NC	NC	South
Riodinidae	*Apodemia*	*Apodemia ares*	4	NC	NC	NC	NC	5.35	1	NC	NC	South
Riodinidae	*Apodemia*	*Apodemia chisosensis*	0	NC	NC	NC	NC	NC	NC	NC	NC	South
Riodinidae	*Apodemia*	*Apodemia duryi**	1	NC	NC	NC	NC	0.46	NC	NC	NC	South
Riodinidae	*Apodemia*	*Apodemia hepburni**	0	NC	NC	NC	NC	NC	NC	NC	NC	South
Riodinidae	*Apodemia*	*Apodemia mejicanus**	9	NC	NC	NC	NC	0.15	6	NC	NC	South
Riodinidae	*Apodemia*	*Apodemia mormo*	21	0.55	0.01	0.58	4.6	0.15	5	7	0.72	Mid-latitude
Riodinidae	*Apodemia*	*Apodemia nais**	2	NC	NC	NC	NC	5.35	2	NC	NC	South
Riodinidae	*Apodemia*	*Apodemia palmerii**	8	NC	NC	NC	NC	5.97	8	NC	NC	South
Riodinidae	*Apodemia*	*Apodemia virgulti**	19	0.94	0.01	0.05	2.4	0.31	11	20	0.56	South
Riodinidae	*Apodemia*	*Apodemia zela**	4	NC	NC	NC	NC	6.03	2	NC	NC	South
Riodinidae	*Calephelis*	*Calephelis arizonensis*	3	NC	NC	NC	NC	2.04	3	NC	NC	South
Riodinidae	*Calephelis*	*Calephelis borealis*	11	0	0	NC	0	2.41	1	NC	NC	Mid-latitude
Riodinidae	*Calephelis*	*Calephelis freemani*	0	NC	NC	NC	NC	1.47	NC	NC	NC	South
Riodinidae	*Calephelis*	*Calephelis muticum*	1	NC	NC	NC	NC	2.04	NC	NC	NC	Mid-latitude
Riodinidae	*Calephelis*	*Calephelis nemesis**	19	0.7	0.01	0.93	1.4	2.72	5	5	0.91	South
Riodinidae	*Calephelis*	*Calephelis perditalis**	5	NC	NC	NC	NC	1.47	1	NC	NC	South
Riodinidae	*Calephelis*	*Calephelis rawsoni**	0	NC	NC	NC	NC	NC	NC	NC	NC	South
Riodinidae	*Calephelis*	*Calephelis virginiensis*	9	NC	NC	NC	NC	3.23	2	NC	NC	South
Riodinidae	*Calephelis*	*Calephelis wrighti**	5	NC	NC	NC	NC	0	3	NC	NC	South
Riodinidae	*Caria*	*Caria ino**	3	NC	NC	NC	NC	0	1	NC	NC	South
Riodinidae	*Lasaia*	*Lasaia sula**	1	NC	NC	NC	NC	0.46	NC	NC	NC	South
Riodinidae	*Melanis*	*Melanis pixe**	3	NC	NC	NC	NC	5.14	2	NC	NC	South

References

1. D'Ercole J, Dincă V, Opler PA, Kondla N, Schmidt C, Phillips JD, et al. A DNA barcode library for the butterflies of North America. PeerJ. 2021;9:e11157.

2. Zhang J, Cong Q, Shen J, Opler PA, Grishin NV. Genomics of a complete butterfly continent. bioRxiv. 2019.

3. Ivanova NV, Dewaard JR, Hebert PDN. An inexpensive, automation-friendly protocol for recovering high-quality DNA. Mol Ecol Notes. 2006;6(4):998–1002.

4. DeWaard J, Ivanova N, Hajibabaei M, Hebert PDN. Assembling DNA Barcodes. Analytical protocols. Methods Mol Biol. 2008;410:275–93.

5. Hebert PDN, DeWaard JR, Zakharov E V, Prosser SWJ, Sones JE, McKeown JTA, et al. A DNA 'Barcode Blitz': rapid digitization and sequencing of a natural history collection. PLoS One. 2013;8(7).

6. Prosser SWJ, DeWaard JR, Miller SE, Hebert PDN. DNA barcodes from century-old type specimens using next-generation sequencing. Mol Ecol Resour. 2016;16(2):487–97.

7. D'Ercole J, Prosser SWJ, Hebert PDN. A SMRT approach for targeted amplicon sequencing of museum specimens (Lepidoptera)—patterns of nucleotide misincorporation. PeerJ. 2021;9:e10420.

8. Saitou N, Nei M. The neighbor-joining method: a new method for reconstructing phylogenetic trees. Mol Biol Evol. 1987;4(4):406–25.

9. Mutanen M, Kivelä SM, Vos RA, Doorenweerd C, Ratnasingham S, Hausmann A, et al. Species-level para- and polyphyly in DNA barcode gene trees: strong operational bias in European lepidoptera. Syst Biol. 2016;65(6):1024–40.

10. Dapporto L, Menchetti M, Vodă R, Corbella C, Cuvelier S, Djemadi I, et al. The atlas of mitochondrial genetic diversity for Western Palaearctic butterflies. Glob Ecol Biogeogr. 2022;31(11):2184–90.

11. Paradis E. Analysis of haplotype networks: the randomized minimum spanning tree method. Methods Ecol Evol. 2018;9(5):1308–17.

12. Tajima F. Evolutionary relationship of DNA sequences in finite populations. Genetics. 1983;105(2):437–60.

13. Nei M. Analysis of gene diversity in subdivided populations. Proc Natl Acad Sci. 1973;70(12):3321–3.

14. Paradis E. pegas: an R package for population genetics with an integrated–modular approach. Bioinformatics. 2010;26(3):419–20.

15. Meirmans PG, Hedrick PW. Assessing population structure: FST and related measures. Mol Ecol Resour. 2011;11(1):5–18.

16. Scalercio S, Cini A, Menchetti M, Vodă R, Bonelli S, Bordoni A, et al. How long is 3 km for a butterfly? Ecological constraints and functional traits explain high mitochondrial genetic diversity between Sicily and the Italian Peninsula. J Anim Ecol. 2020;89(9):2013–26.

17. Dincă V, Dapporto L, Somervuo P, Vodă R, Cuvelier S, Gascoigne-Pees M, et al. High resolution DNA barcode library for European butterflies reveals continental patterns of mitochondrial genetic diversity. Commun Biol. 2021;4(315).

18. Hsieh TC, Ma KH, Chao A. iNEXT: an R package for rarefaction and extrapolation of species diversity (Hill numbers). Methods Ecol Evol. 2016;7(12):1451–6.

19. Chao A. Nonparametric estimation of the number of classes in a population. Scand JStat. 1984;11(4):265–70.

20. Zhang J, Cong Q, Grishin NV. Thirteen new species of butterflies (Lepidoptera: Hesperiidae) from Texas. Insecta mundi. 2023;0969:1–58.

21. Grishin N. Expanded phenotypic diagnoses for 24 recently named new taxa of Hesperiidae (Lepidoptera). Taxon Rep Int Lepid Surv. 2019;8:1–15.

22. Li W, Cong Q, Shen J, Zhang J, Hallwachs W, Janzen DH, et al. Genomes of skipper butterflies reveal extensive convergence of wing patterns. Proc Natl Acad Sci. 2019;116(13):6232–7.

23. Zhang J, Cong Q, Shen J, Song L, Opler P, Grishin N. Additional taxonomic refinements suggested by genomic analysis of butterflies. Taxon Rep Int Lepid Surv. 2023;11(1).

24. Warren AD, Davis KJ, Grishin NV, Pelham JP, Stangeland EM. Illustrated Lists of American Butterflies [Internet]. 2012. Available from: http://www.butterfliesofamerica.com/

25. Zhang J, Cong Q, Shen J, Opler PA, Grishin NV. Changes to North American butterfly names. Taxon Rep Int Lepid Surv. 2019;8:1–12.

26. Zhang J, Shen J, Cong Q, Grishin NV. Genomic analysis of the tribe Emesidini (Lepidoptera: Riodinidae). Zootaxa. 2019;4668.

27. Zhang J, Cong Q, Shen J, Opler PA, Grishin NV. Genomic evidence suggests further changes of butterfly names. Taxon Rep Int Lepid Surv. 2020;8:7.

28. Zhang J, Cong Q, Shen J, Opler PA, Grishin NV. Genomics-guided refinement of butterfly taxonomy. Taxon Rep Int Lepid Surv. 2021;9:1–54.

29. Cong Q, Zhang J, Shen J, Grishin NV. Fifty new genera of Hesperiidae (Lepidoptera). Insecta mundi. 2019;0731:1–56.

30. Burns JM. Taxonomic status of a Florida differentiate in the *Erynnis brizo* species group: classical evidence (Lepidoptera: Hesperiidae: Pyrginae). Proc Entomol Soc Wash. 2020;122(1):25–41.

31. Davenport K. Lepidoptera of North America 15. Butterflies of southern California in 2018: updating Emmel and Emmel’s 1973 butterflies of southern California. Fort Collins: Gilette Museum of Arthropod Diversity, Colorado State University; 2018.

32. Rubinoff D, Longcore T, Dupuis JR, Osborne KH. Genomic data support the elevation of the federally listed El Segundo Blue (*Euphilotes bernardino*/*Battoides allyni*) to species status. J Lepid Soc. 2021;75(2):161–4.

33. Kohler S, Warren A. Review of Montana *Euphilotes* Mattoni, [1978], with descriptions of new taxa (Lepidoptera: Lycaenidae: Polyommatinae). Taxon Rep Int Lepid Surv. 2021;9:1–79.

34. Zhang J, Cong Q, Shen J, Song L, Gott R, Boyer P, et al. Taxonomic discoveries enabled by genomic analysis of butterflies. Taxon Rep Int Lepid Surv. 2022;10:1–59.

35. Brower A, Wahlberg N, Simonsen T, de Jong R. Morphology, molecules and fritillaries: approaching a stable phylogeny for Argynnini (Lepidoptera: Nymphalidae). Insect Syst Evol. 2006;37(4):405–18.

36. De Moya R, Savage W, Tenney C, Bao X, Wahlberg N, Hill R. Interrelationships and diversification of *Argynnis* Fabricius and Speyeria Scudder butterflies. Syst Entomol. 2017;42(4):635–49.

37. Lalonde MML, McCullagh BS, Marcus JM. The Taxonomy and population structure of the buckeye butterflies (Genus *Junonia*, Nymphalidae: Nymphalini) of Florida, USA. J Lepid Soc. 2018;72(2):97–115.

38. Zacca T, Casagrande M, Mielke O, Huertas B, Barbosa E, Freitas A, et al. Systematics of the butterfly genus *Cissia* Doubleday, 1848 (Lepidoptera: Nymphalidae: Satyrinae) using an integrative approach. Arthropod Syst Phylogeny. 2018;76:349–76.

39. Calhoun J. John Abbot, Jacob Hübner and *Oreas helicta* (Nymphalidae: Satyrinae). News Lepid Soc. 2018;60:159–63.

40. Kleckova I, Cesanek M, Fric Z, Pellissier L. Diversification of the cold-adapted butterfly genus *Oeneis* related to Holarctic biogeography and climatic niche shifts. Mol Phylogenet Evol. 2015;92:255–65.

41. Zakharov E V, Caterino MS, Sperling FAH. Molecular phylogeny, historical biogeography, and divergence time estimates for swallowtail butterflies of the genus *Papili*o (Lepidoptera: Papilionidae). Syst Biol. 2004;53(2):193–215.

42. Hammond P, Mccorkle D. Taxonomy, ecology, and evolutionary theory of the genus *Colias* (Lepidoptera: Pieridae: Coliadinae). Corvalis: The Franklin Press; 2017.

43. Korb S, Bolshakov LV. A systematic catalogue of butterflies of the former Soviet Union (Armenia, Azerbaijan, Belarus, Estonia, Georgia, Kyrgyzstan, Kazakhstan, Latvia, Lituania, Moldova, Russia, Tajikistan, Turkmenistan, Ukraine, Uzbekistan) with special account to their type specimens (Lepidoptera: Hesperioidea, Papilionoidea). Zootaxa. 2016;4160:1–324.

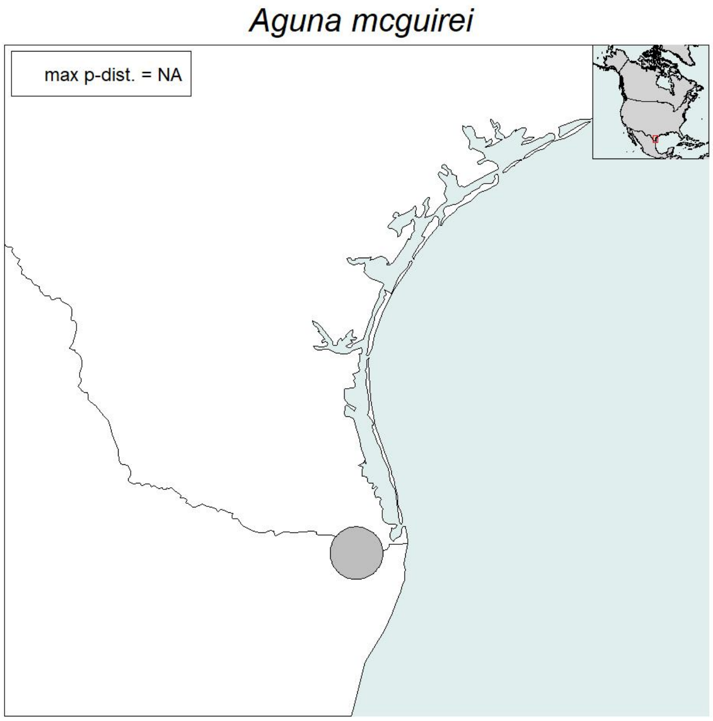

Figure 1: Map of *Aguna mcguirei* showing the localities of the sequenced specimens. Nearby localities are grouped in pies. Due to the presence of a single haplotype PCoA projection was not done and a single grey colour was plotted on the map. Sequences= 1; Hap obs.= NA; Hap asympt.= NA; Hap % obs.= NA; GST= NaN; DST= NaN; HD= NA; ND= NA; max p-dist= NA.

Haplotype network analysis and bubble plot of *Aguna mcguirei* were not possible. Sequences > 599 bp = 1.

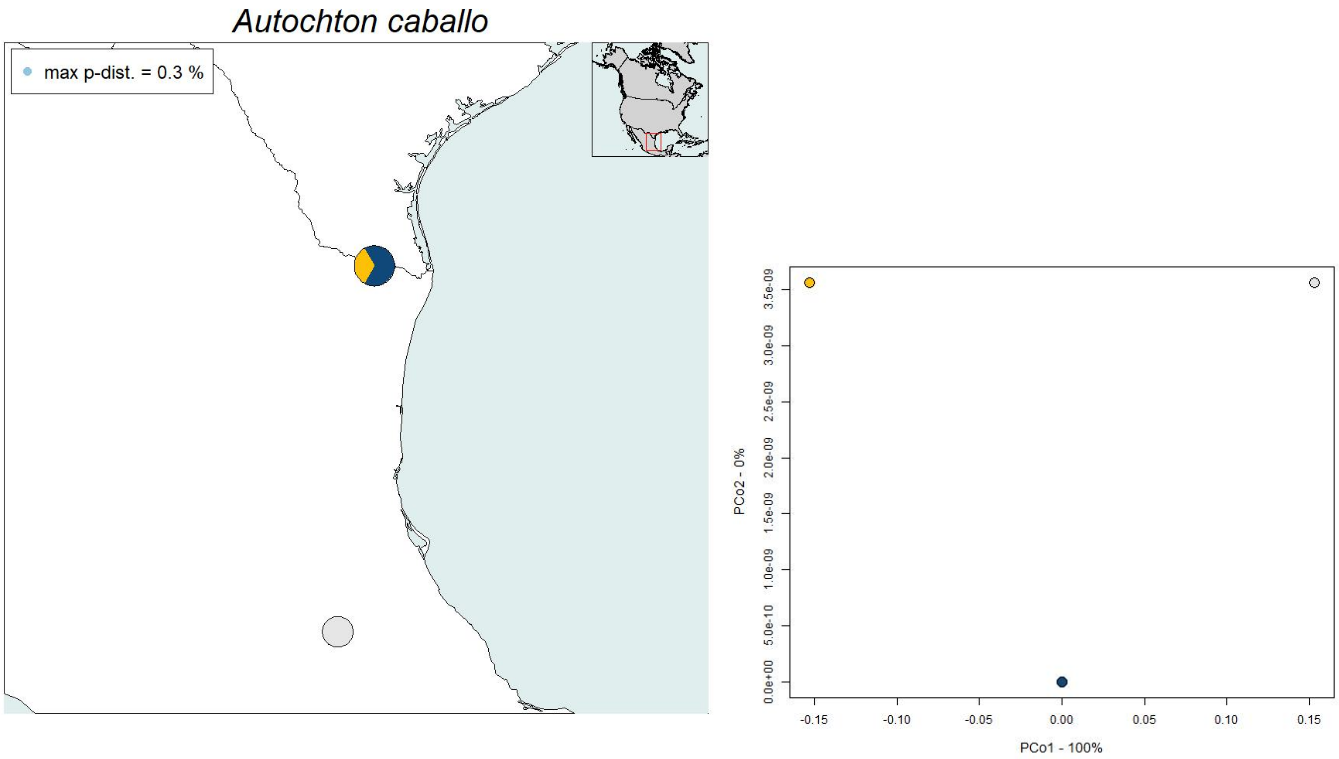

Figure 2 Map of *Autochton caballo* showing the localities of the sequenced specimens (left). Nearby localities are grouped in pies. Colours match the bidimensional colour space of the PCoA projection (right) of max p-dists among sequences (dots). Sequences= 4; Hap obs.= 3; Hap asympt.= NA; Hap % obs.= NA%; GST= NaN; DST= NaN; HD= NA; ND= NA; max p-dist= 0.3%.

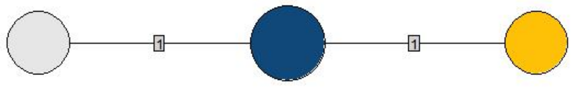

Figure 3: Haplotype network of *Autochton caballo*. Sequences > 599 bp= 4.

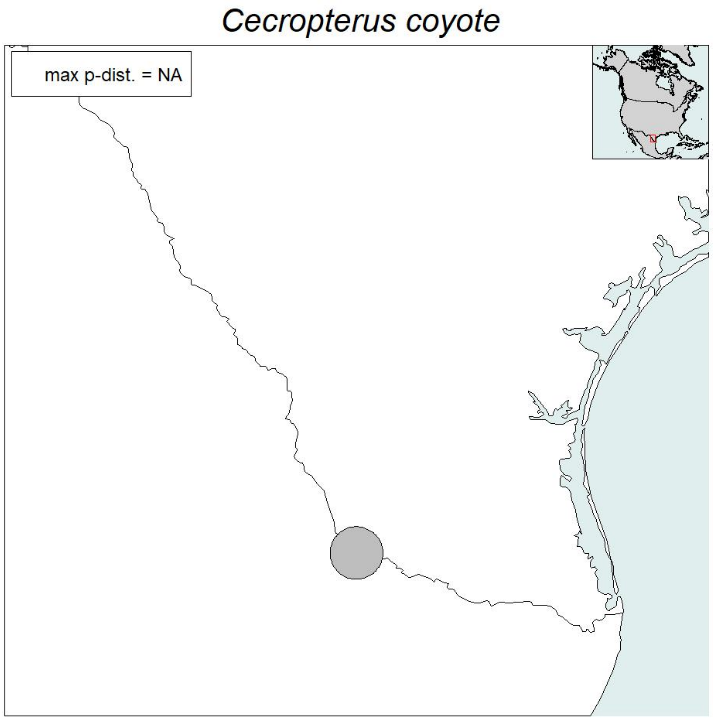

Figure 4: Map of *Cecropterus coyote* showing the localities of the sequenced specimens. Nearby localities are grouped in pies. Due to the presence of a single haplotype PCoA projection was not done and a single grey colour was plotted on the map. Sequences= 1; Hap obs.= NA; Hap asympt.= NA; Hap % obs.= NA; GST= NaN; DST= NaN; HD= NA; ND= NA; max p-dist= NA.

Haplotype network analysis and bubble plot of *Cecropterus coyote* were not possible. Sequences > 599 bp = 1.

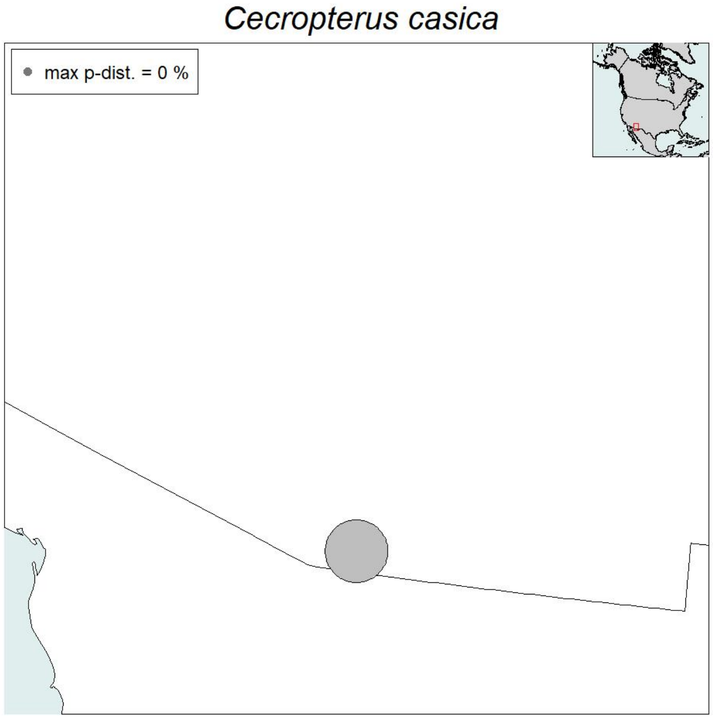

Figure 5: Map of *Cecropterus casica* showing the localities of the sequenced specimens. Nearby localities are grouped in pies. Due to the presence of a single haplotype PCoA projection was not done and a single grey colour was plotted on the map. Sequences= 2; Hap obs.= 1; Hap asympt.= NA; Hap % obs.= NA%; GST= NaN; DST= NaN; HD= NA; ND= NA; max p-dist= 0%.

Haplotype network analysis and bubble plot of *Cecropterus casica* were not possible. Sequences > 599 bp = 2.

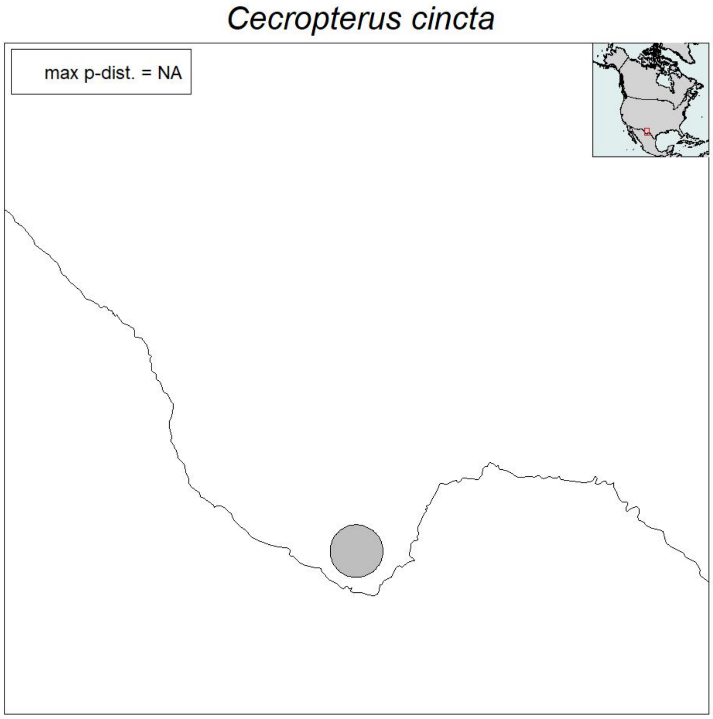

Figure 6: Map of *Cecropterus cincta* showing the localities of the sequenced specimens. Nearby localities are grouped in pies. Due to the presence of a single haplotype PCoA projection was not done and a single grey colour was plotted on the map. Sequences= 1; Hap obs.= NA; Hap asympt.= NA; Hap % obs.= NA; GST= NaN; DST= NaN; HD= NA; ND= NA; max p-dist= NA.

Haplotype network analysis and bubble plot of *Cecropterus cincta* were not possible. Sequences > 599 bp = 1.

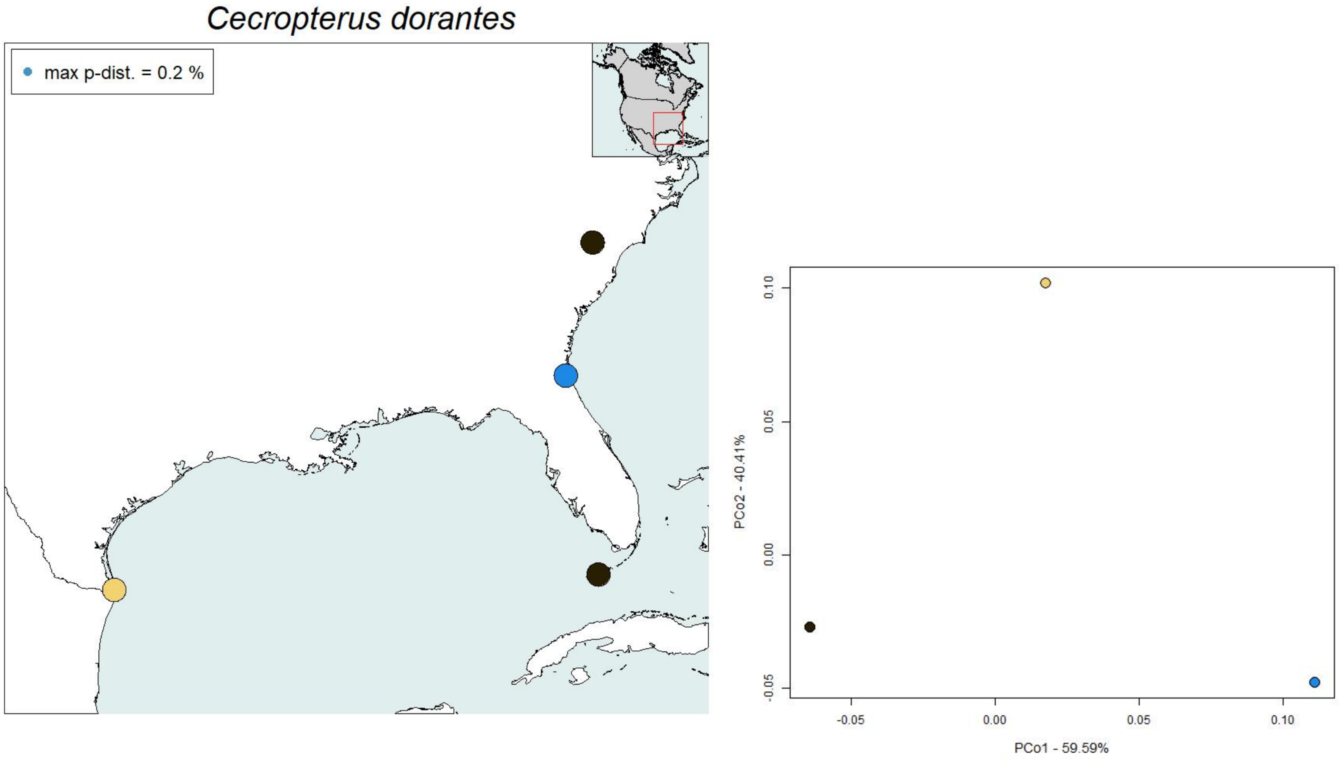

Figure 7 Map of *Cecropterus dorantes* showing the localities of the sequenced specimens (left). Nearby localities are grouped in pies. Colours match the bidimensional colour space of the PCoA projection (right) of max p-dists among sequences (dots). Sequences= 4; Hap obs.= 3; Hap asympt.= NA; Hap % obs.= NA%; GST= NaN; DST= NaN; HD= NA; ND= NA; max p-dist= 0.2%.

Haplotype network analysis and bubble plot of *Cecropterus dorantes* were not possible. Sequences > 599 bp = 3.

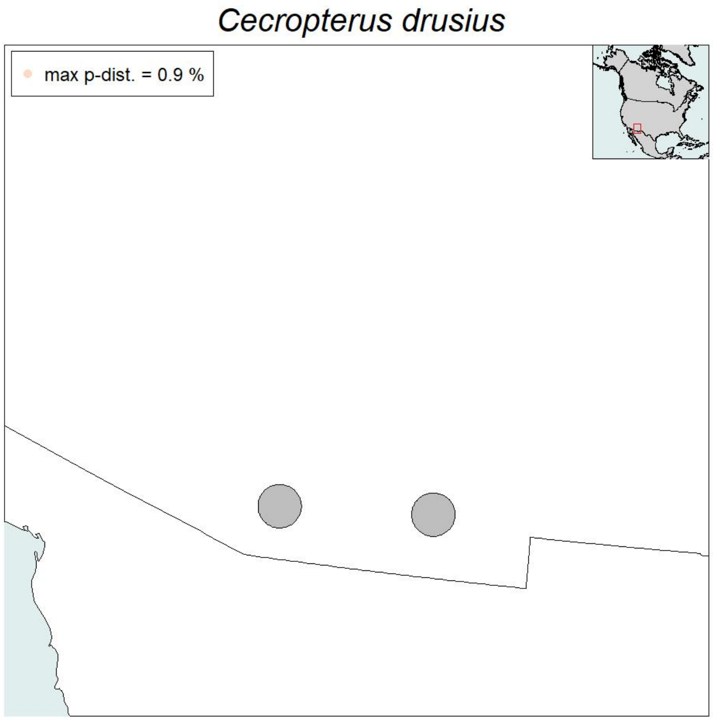

Figure 8: Map of *Cecropterus drusius* showing the localities of the sequenced specimens. Nearby localities are grouped in pies. Due to the presence of a single haplotype PCoA projection was not done and a single grey colour was plotted on the map. Sequences= 2; Hap obs.= 2; Hap asympt.= NA; Hap % obs.= NA%; GST= NaN; DST= NaN; HD= NA; ND= NA; max p-dist= 0.9%.

Haplotype network analysis and bubble plot of *Cecropterus drusius* were not possible. Sequences > 599 bp = 2.

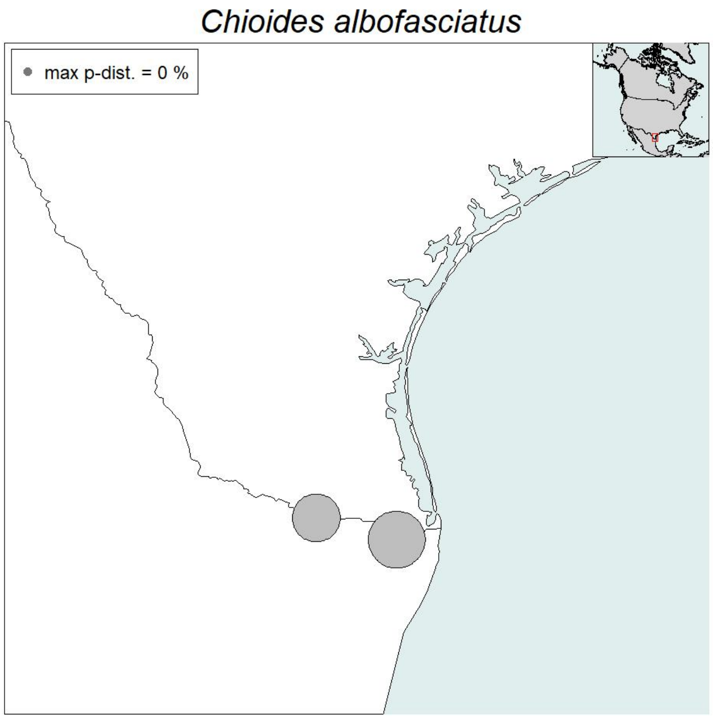

Figure 9: Map of *Chioides albofasciatus* showing the localities of the sequenced specimens. Nearby localities are grouped in pies. Due to the presence of a single haplotype PCoA projection was not done and a single grey colour was plotted on the map. Sequences= 3; Hap obs.= 1; Hap asympt.= NA; Hap % obs.= NA%; GST= NaN; DST= NaN; HD= NA; ND= NA; max p-dist= 0%.

Haplotype network analysis and bubble plot of *Chioides albofasciatus* were not possible. Sequences > 599 bp = 3.

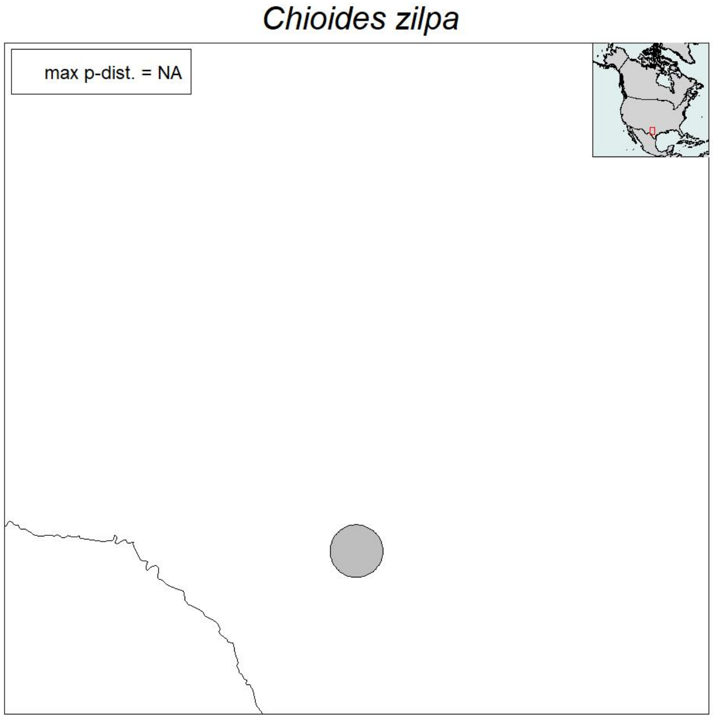

Figure 10: Map of *Chioides zilpa* showing the localities of the sequenced specimens. Nearby localities are grouped in pies. Due to the presence of a single haplotype PCoA projection was not done and a single grey colour was plotted on the map. Sequences= 1; Hap obs.= NA; Hap asympt.= NA; Hap % obs.= NA; GST= NaN; DST= NaN; HD= NA; ND= NA; max p-dist= NA.

Haplotype network analysis and bubble plot of *Chioides zilpa* were not possible. Sequences > 599 bp = 1.

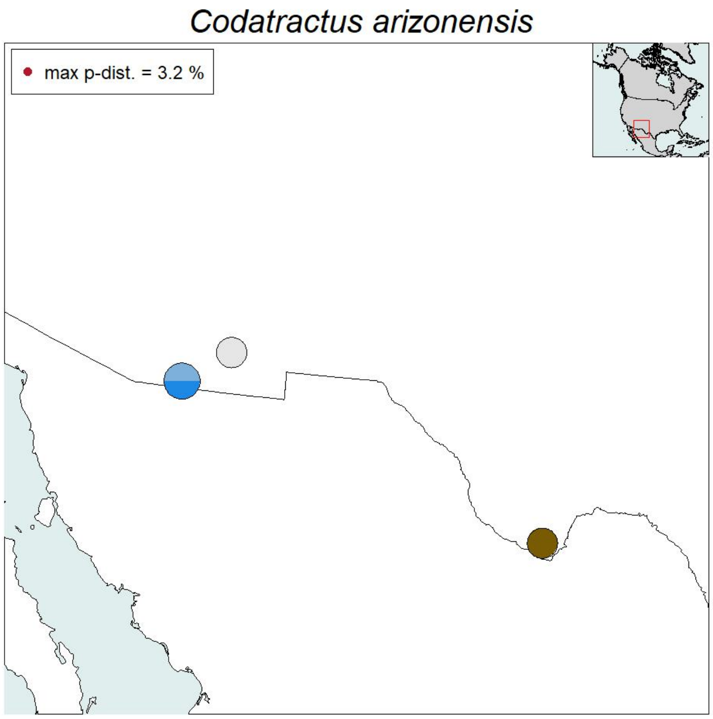

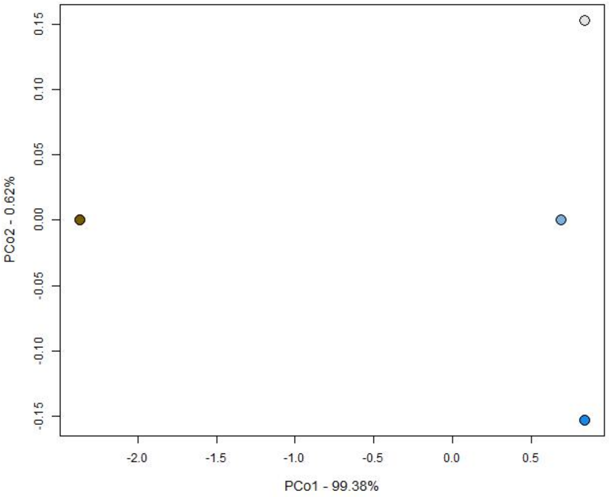

Figure 11 Map of *Codatractus arizonensis* showing the localities of the sequenced specimens (left). Nearby localities are grouped in pies. Colours match the bidimensional colour space of the PCoA projection (right) of max p-dists among sequences (dots). Sequences= 4; Hap obs.= 4; Hap asympt.= NA; Hap % obs.= NA%; GST= NaN; DST= NaN; HD= NA; ND= NA; max p-dist= 3.2%.

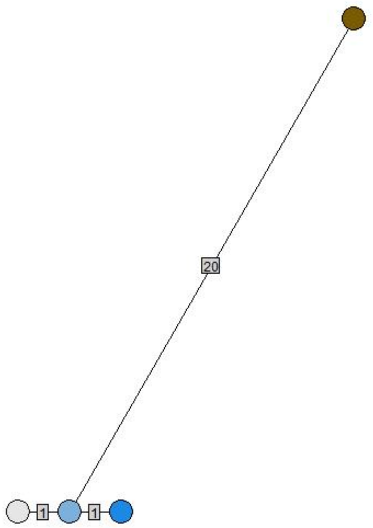

Figure 12: Haplotype network of *Codatractus arizonensis*. Sequences > 599 bp= 4.

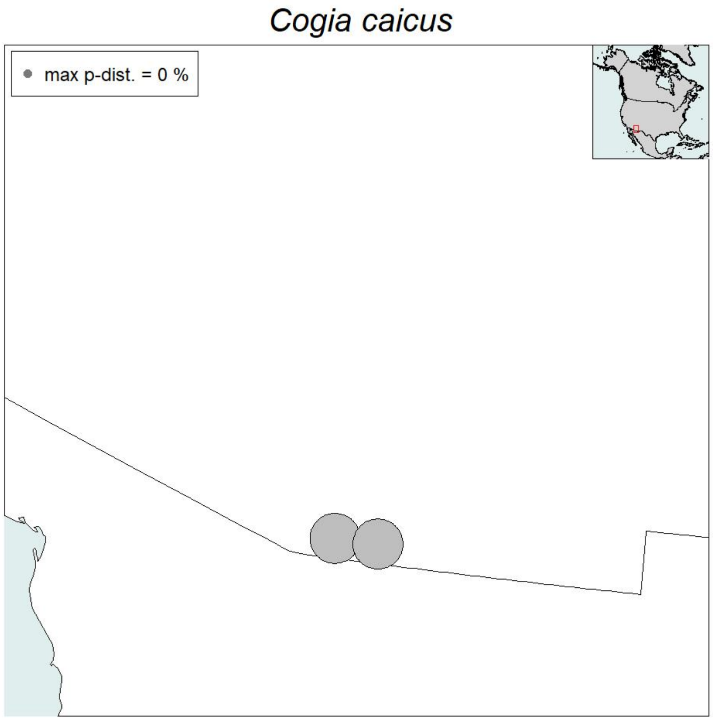

Figure 13: Map of *Cogia caicus* showing the localities of the sequenced specimens. Nearby localities are grouped in pies. Due to the presence of a single haplotype PCoA projection was not done and a single grey colour was plotted on the map. Sequences= 2; Hap obs.= 1; Hap asympt.= NA; Hap % obs.= NA%; GST= NaN; DST= NaN; HD= NA; ND= NA; max p-dist= 0%.

Haplotype network analysis and bubble plot of *Cogia caicus* were not possible. Sequences > 599 bp = 2.

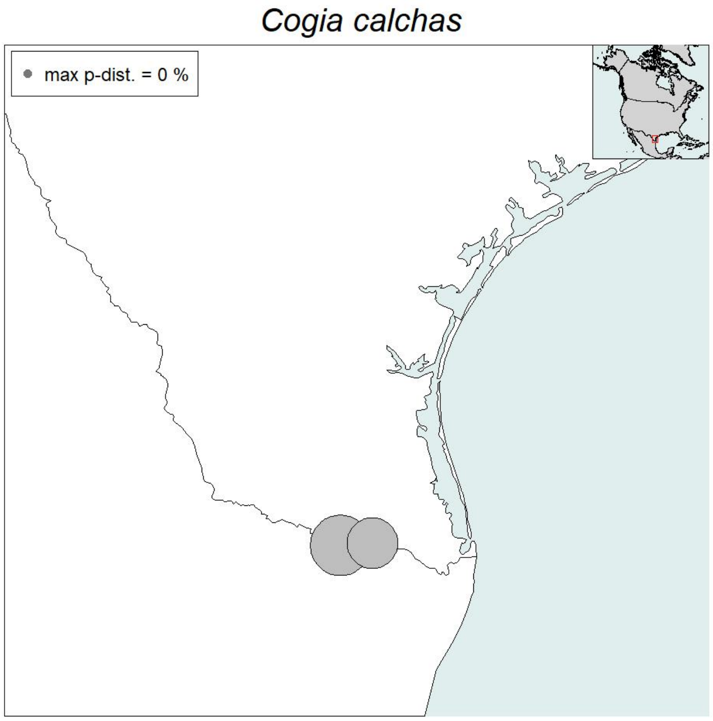

Figure 14: Map of *Cogia calchas* showing the localities of the sequenced specimens. Nearby localities are grouped in pies. Due to the presence of a single haplotype PCoA projection was not done and a single grey colour was plotted on the map. Sequences= 3; Hap obs.= 1; Hap asympt.= NA; Hap % obs.= NA%; GST= NaN; DST= NaN; HD= NA; ND= NA; max p-dist= 0%.

Haplotype network analysis and bubble plot of *Cogia calchas* were not possible. Sequences > 599 bp = 3.

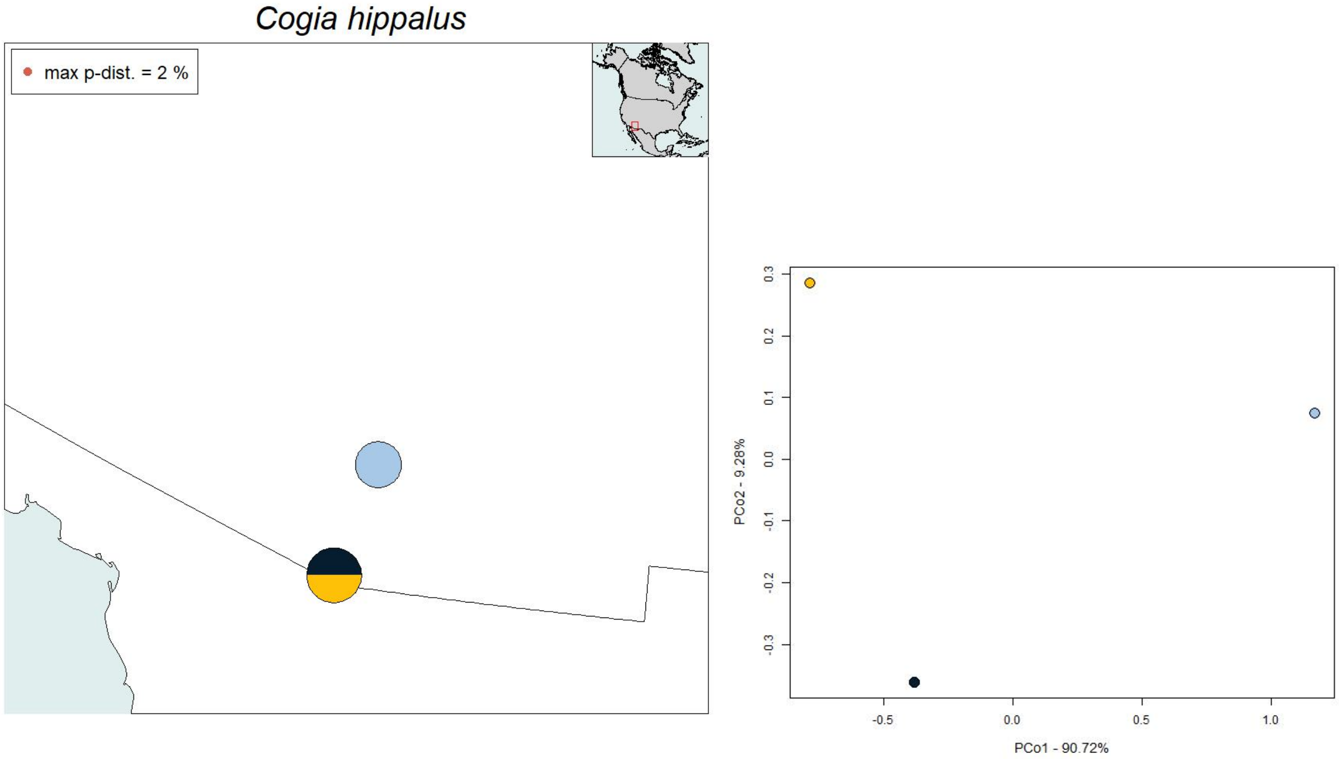

Figure 15 Map of *Cogia hippalus* showing the localities of the sequenced specimens (left). Nearby localities are grouped in pies. Colours match the bidimensional colour space of the PCoA projection (right) of max p-dists among sequences (dots). Sequences= 3; Hap obs.= 3; Hap asympt.= NA; Hap % obs.= NA%; GST= NaN; DST= NaN; HD= NA; ND= NA; max p-dist= 2%.

Haplotype network analysis and bubble plot of *Cogia hippalus* were not possible. Sequences > 599 bp = 2.

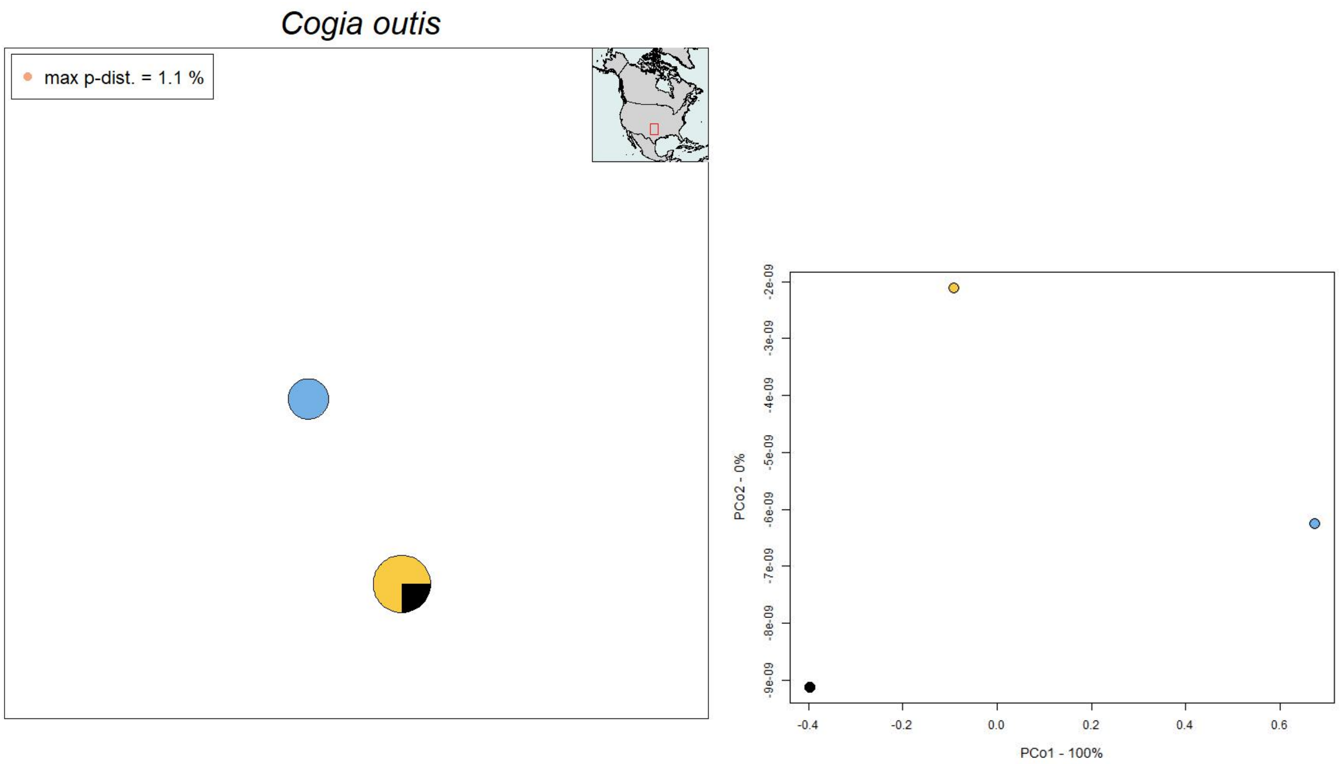

Figure 16 Map of *Cogia outis* showing the localities of the sequenced specimens (left). Nearby localities are grouped in pies. Colours match the bidimensional colour space of the PCoA projection (right) of max p-dists among sequences (dots). Sequences= 5; Hap obs.= 3; Hap asympt.= NA; Hap % obs.= NA%; GST= NaN; DST= NaN; HD= NA; ND= NA; max p-dist= 1.1%.

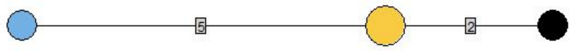

Figure 17: Haplotype network of *Cogia outis*. Sequences > 599 bp= 5.

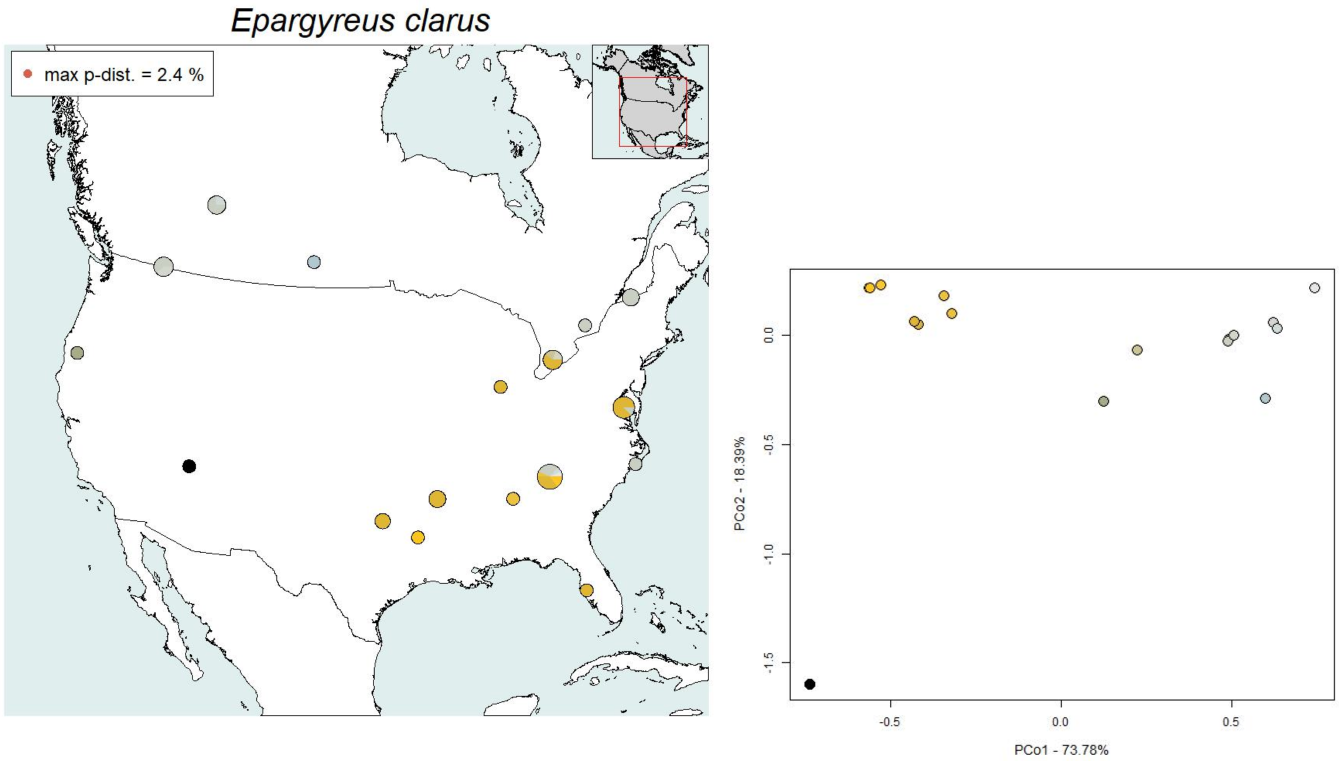

Figure 18 Map of *Epargyreus clarus* showing the localities of the sequenced specimens (left). Nearby localities are grouped in pies. Colours match the bidimensional colour space of the PCoA projection (right) of max p-dists among sequences (dots). Sequences= 53; Hap obs.= 13; Hap asympt.= 52.7; Hap % obs.= 24.7%; GST= 0.243; DST= 0.0013; HD= 0.734; ND= 0.0058; max p-dist= 2.4%.

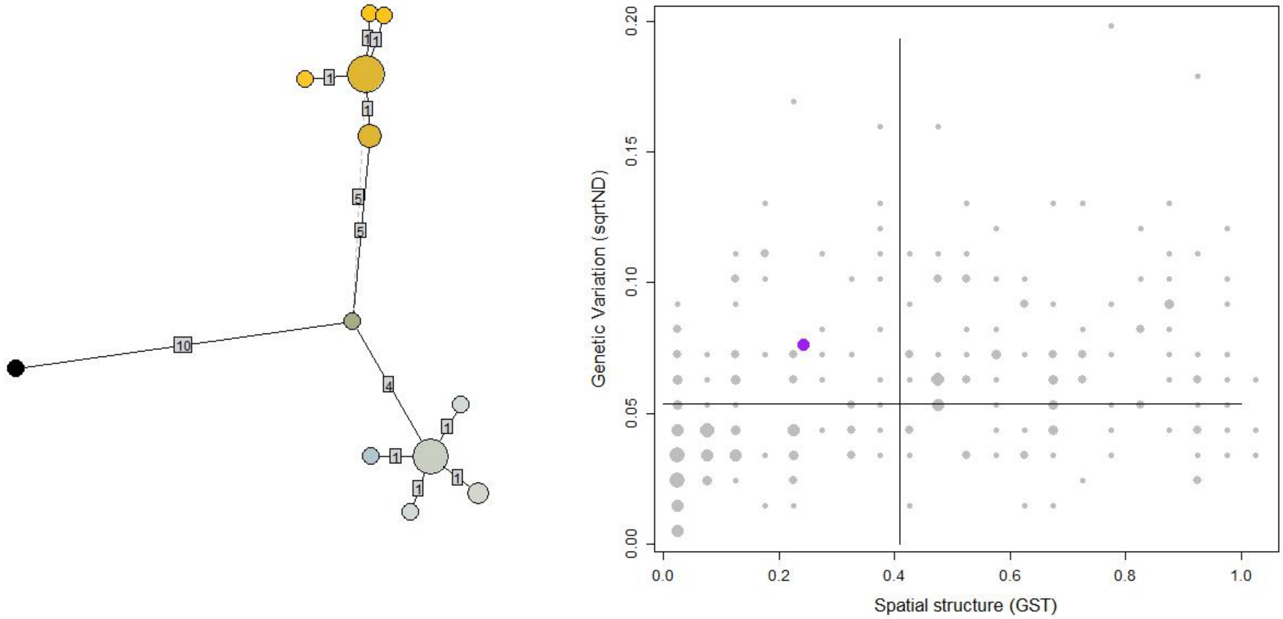

Figure 19: Haplotype network (left) of *Epargyreus clarus* sequences > 599 bp with colours matching the PCoA colour space (above). The bubble plot for mt-DNA polymorphism (square root transformed nucleotide diversity) and spatial structure (GST) among all species in the atlas and values for *Epargyreus clarus* (purple dot). The horizontal and vertical lines represent median values of nucleotide diversity and GST, respectively. Sequences > 599 bp= 51.

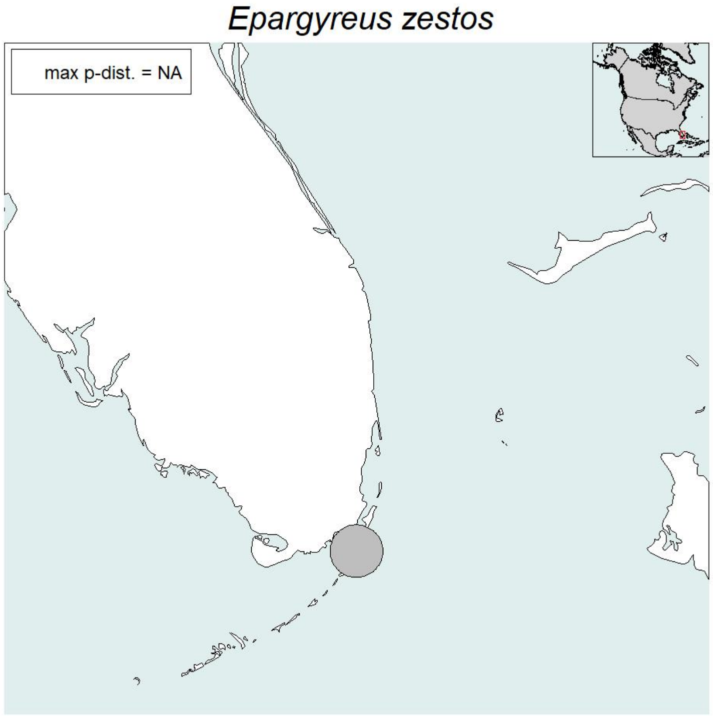

Figure 20: Map of *Epargyreus zestos* showing the localities of the sequenced specimens. Nearby localities are grouped in pies. Due to the presence of a single haplotype PCoA projection was not done and a single grey colour was plotted on the map. Sequences= 1; Hap obs.= NA; Hap asympt.= NA; Hap % obs.= NA; GST= NaN; DST= NaN; HD= NA; ND= NA; max p-dist= NA.

Haplotype network analysis and bubble plot of *Epargyreus zestos* were not possible. Sequences > 599 bp = 1.

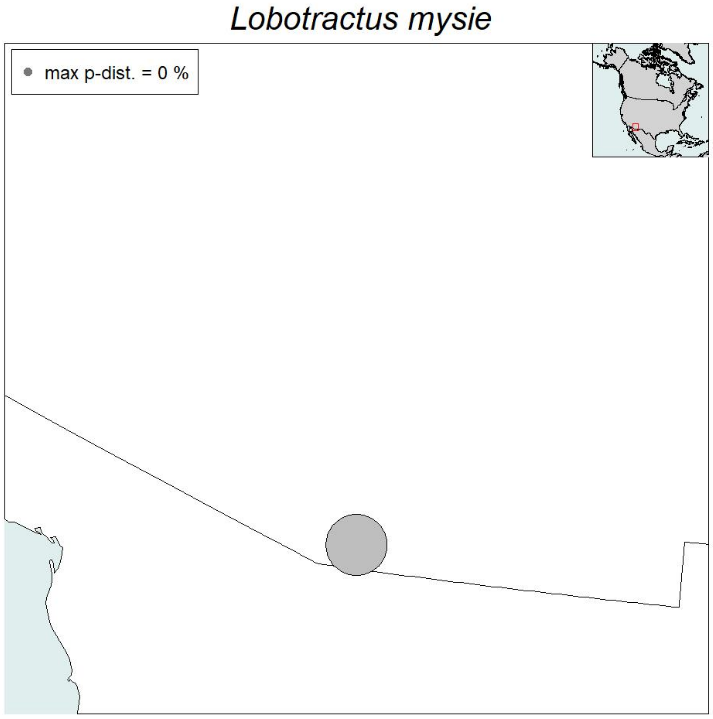

Figure 21: Map of *Lobotractus mysie* showing the localities of the sequenced specimens. Nearby localities are grouped in pies. Due to the presence of a single haplotype PCoA projection was not done and a single grey colour was plotted on the map. Sequences= 2; Hap obs.= 1; Hap asympt.= NA; Hap % obs.= NA%; GST= NaN; DST= NaN; HD= NA; ND= NA; max p-dist= 0%.

Haplotype network analysis and bubble plot of *Lobotractus mysie* were not possible. Sequences > 599 bp = 2.

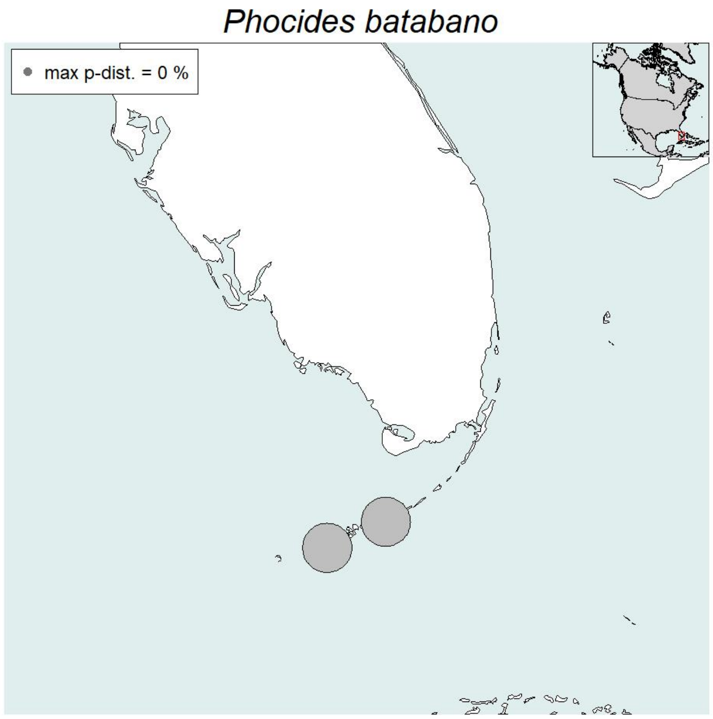

Figure 22: Map of *Phocides batabano* showing the localities of the sequenced specimens. Nearby localities are grouped in pies. Due to the presence of a single haplotype PCoA projection was not done and a single grey colour was plotted on the map. Sequences= 2; Hap obs.= 1; Hap asympt.= NA; Hap % obs.= NA%; GST= NaN; DST= NaN; HD= NA; ND= NA; max p-dist= 0%.

Haplotype network analysis and bubble plot of *Phocides batabano* were not possible. Sequences > 599 bp = 2.

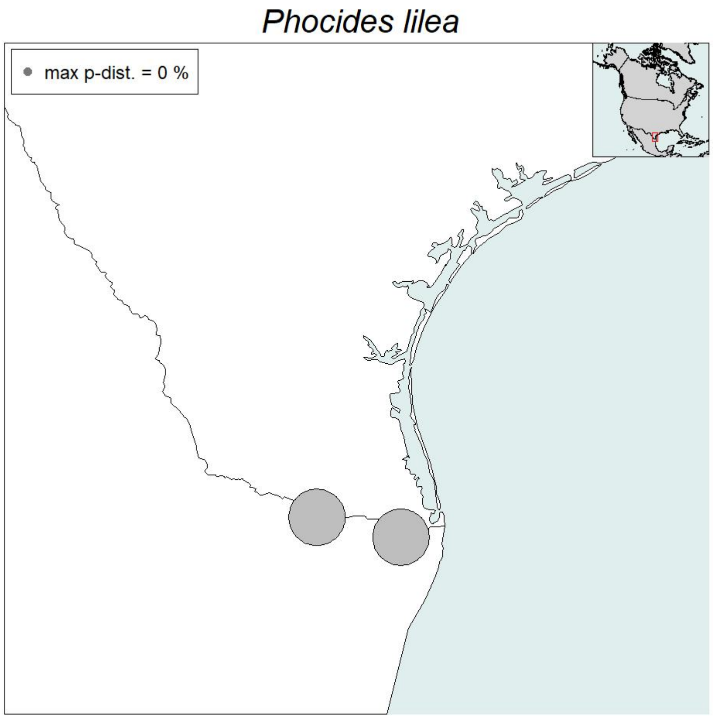

Figure 23: Map of *Phocides lilea* showing the localities of the sequenced specimens. Nearby localities are grouped in pies. Due to the presence of a single haplotype PCoA projection was not done and a single grey colour was plotted on the map. Sequences= 4; Hap obs.= 1; Hap asympt.= NA; Hap % obs.= NA%; GST= NaN; DST= NaN; HD= NA; ND= NA; max p-dist= 0%.

Haplotype network analysis and bubble plot of *Phocides lilea* were not possible. Sequences > 599 bp = 4.

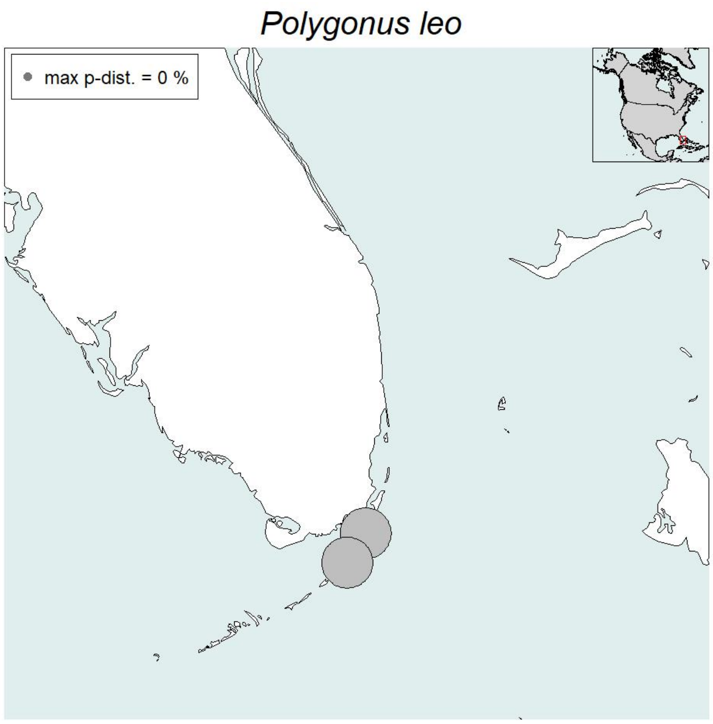

Figure 24: Map of *Polygonus leo* showing the localities of the sequenced specimens. Nearby localities are grouped in pies. Due to the presence of a single haplotype PCoA projection was not done and a single grey colour was plotted on the map. Sequences= 2; Hap obs.= 1; Hap asympt.= NA; Hap % obs.= NA%; GST= NaN; DST= NaN; HD= NA; ND= NA; max p-dist= 0%.

Haplotype network analysis and bubble plot of *Polygonus leo* were not possible. Sequences > 599 bp = 2.

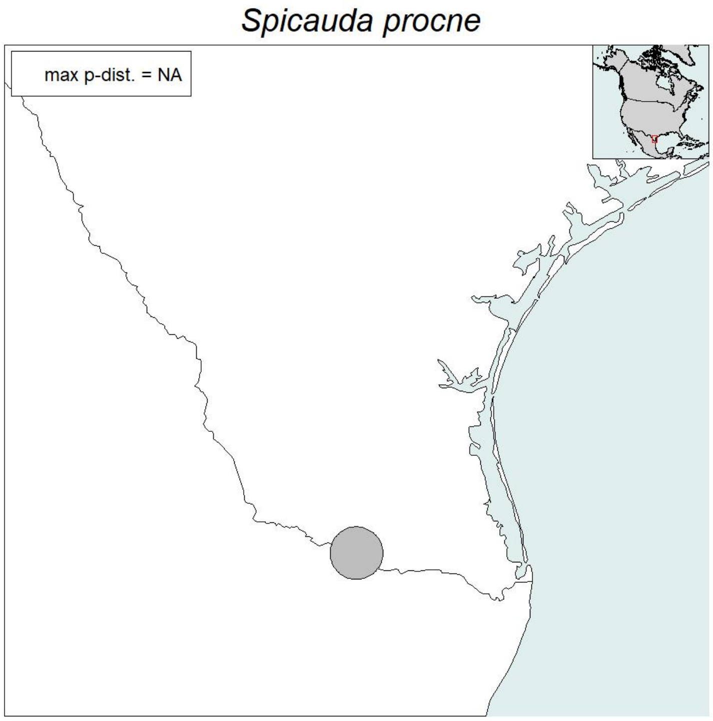

Figure 25: Map of *Spicauda procne* showing the localities of the sequenced specimens. Nearby localities are grouped in pies. Due to the presence of a single haplotype PCoA projection was not done and a single grey colour was plotted on the map. Sequences= 1; Hap obs.= NA; Hap asympt.= NA; Hap % obs.= NA; GST= NaN; DST= NaN; HD= NA; ND= NA; max p-dist= NA.

Haplotype network analysis and bubble plot of *Spicauda procne* were not possible. Sequences > 599 bp = 1.

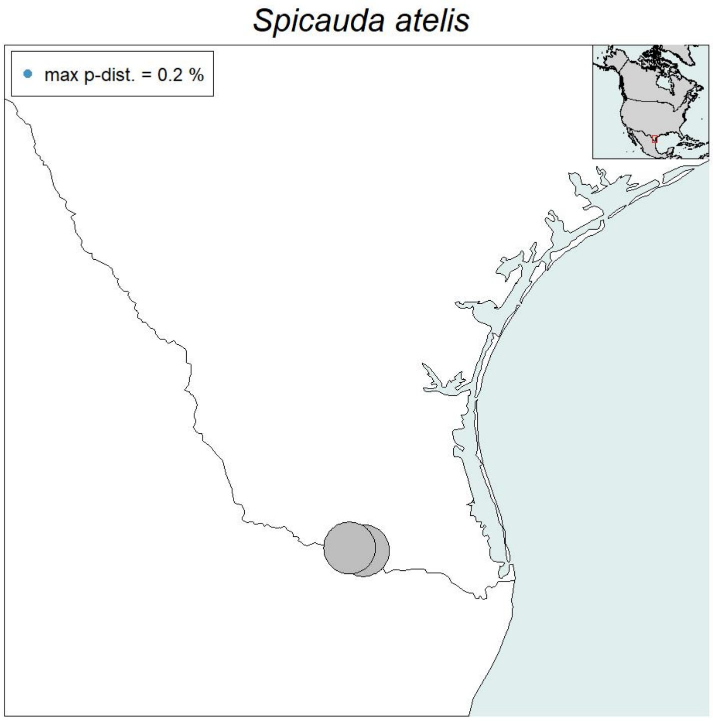

Figure 26: Map of *Spicauda atelis* showing the localities of the sequenced specimens. Nearby localities are grouped in pies. Due to the presence of a single haplotype PCoA projection was not done and a single grey colour was plotted on the map. Sequences= 2; Hap obs.= 2; Hap asympt.= NA; Hap % obs.= NA%; GST= NaN; DST= NaN; HD= NA; ND= NA; max p-dist= 0.2%.

Haplotype network analysis and bubble plot of *Spicauda atelis* were not possible. Sequences > 599 bp = 2.

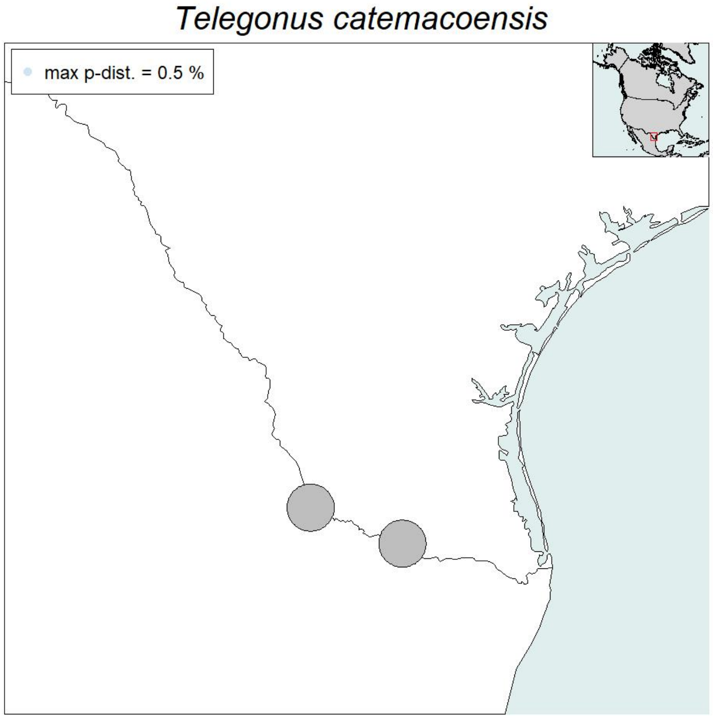

Figure 27: Map of *Telegonus catemacoensis* showing the localities of the sequenced specimens. Nearby localities are grouped in pies. Due to the presence of a single haplotype PCoA projection was not done and a single grey colour was plotted on the map. Sequences= 2; Hap obs.= 2; Hap asympt.= NA; Hap % obs.= NA%; GST= NaN; DST= NaN; HD= NA; ND= NA; max p-dist= 0.5%.

Haplotype network analysis and bubble plot of *Telegonus catemacoensis* were not possible. Sequences > 599 bp = 1.

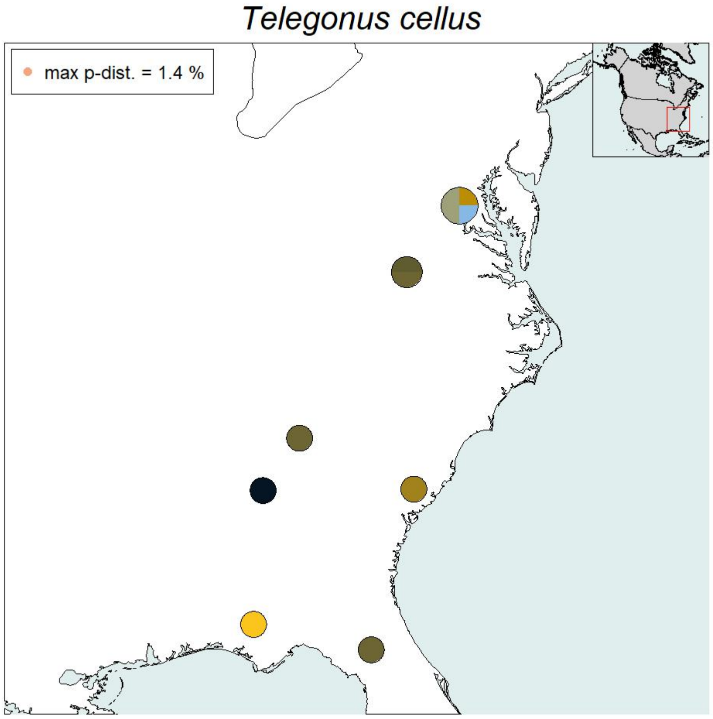

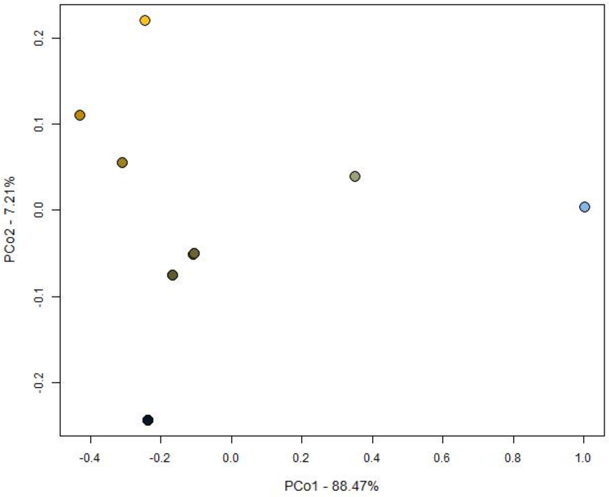

Figure 28 Map of *Telegonus cellus* showing the localities of the sequenced specimens (left). Nearby localities are grouped in pies. Colours match the bidimensional colour space of the PCoA projection (right) of max p-dists among sequences (dots). Sequences= 11; Hap obs.= 6; Hap asympt.= 8; Hap % obs.= 74.6%; GST= NaN; DST= NaN; HD= 0.855; ND= 0.0045; max p-dist= 1.4%.

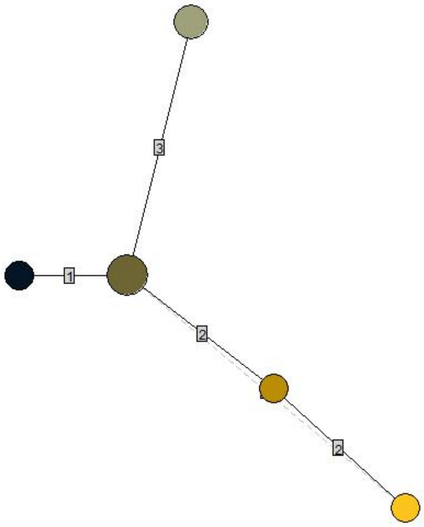

Figure 29: Haplotype network of *Telegonus cellus*. Sequences > 599 bp= 9.

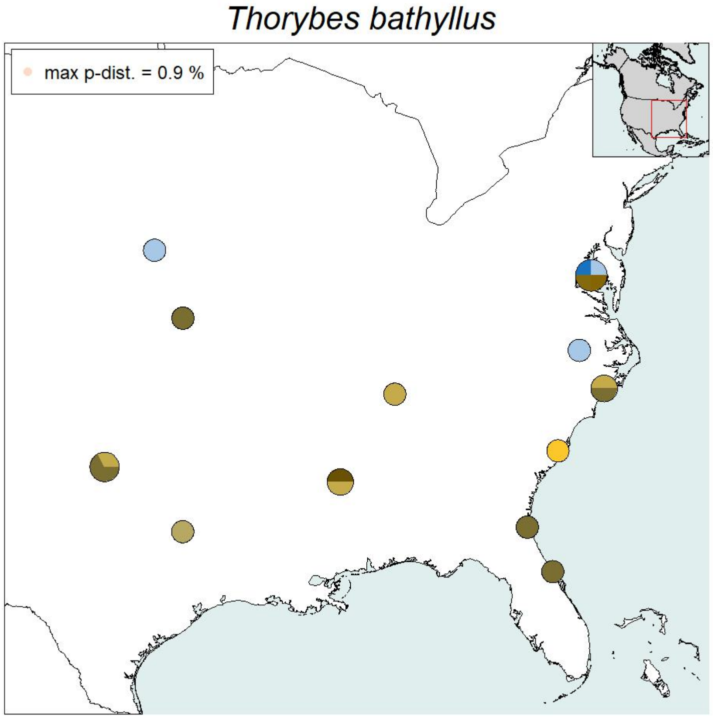

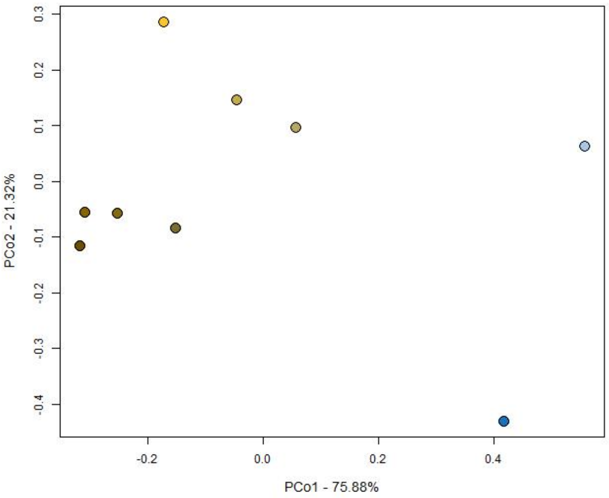

Figure 30 Map of *Thorybes bathyllus* showing the localities of the sequenced specimens (left). Nearby localities are grouped in pies. Colours match the bidimensional colour space of the PCoA projection (right) of max p-dists among sequences (dots). Sequences= 19; Hap obs.= 7; Hap asympt.= 12.7; Hap % obs.= 55.2%; GST= NaN; DST= NaN; HD= 0.784; ND= 0.0031; max p-dist= 0.9%.

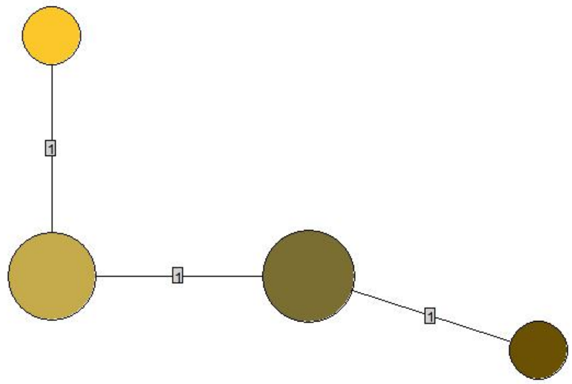

Figure 31: Haplotype network of *Thorybes bathyllus*. Sequences > 599 bp= 13.

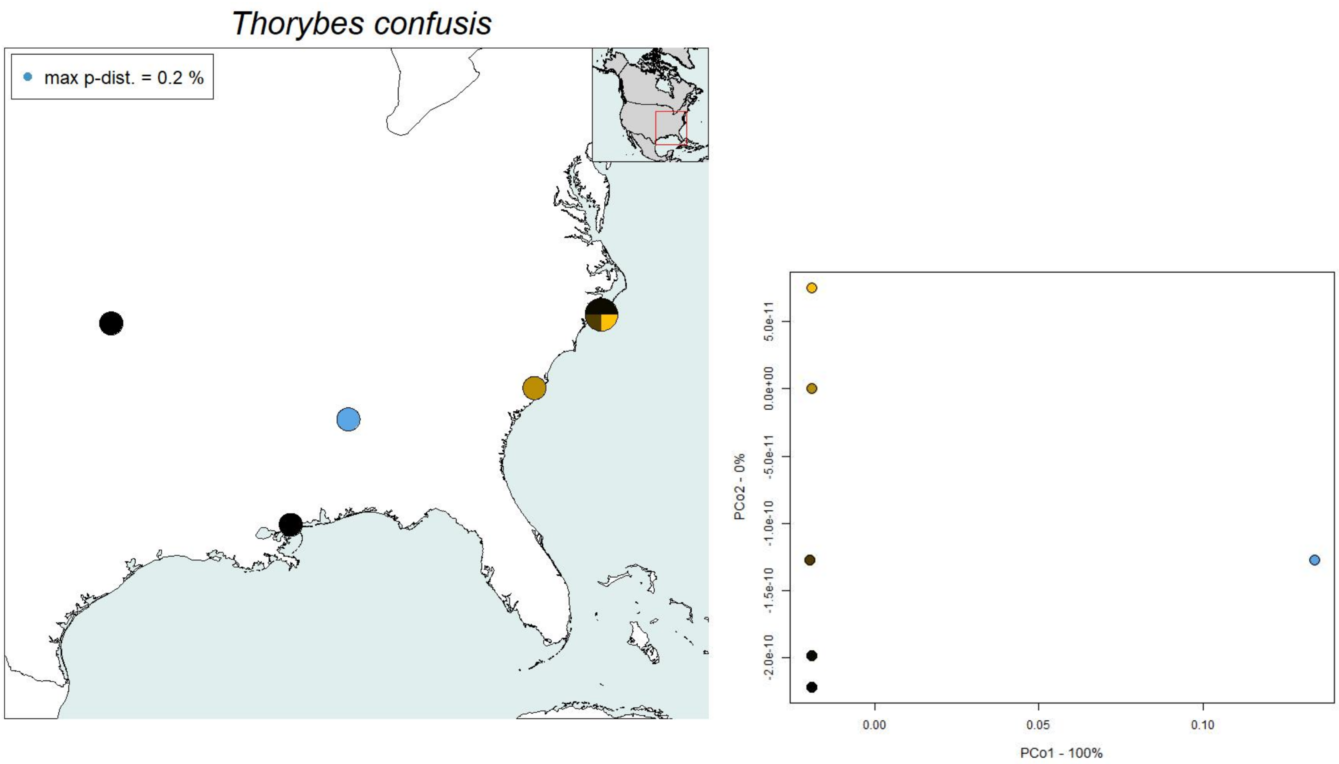

Figure 32 Map of *Thorybes confusis* showing the localities of the sequenced specimens (left). Nearby localities are grouped in pies. Colours match the bidimensional colour space of the PCoA projection (right) of max p-dists among sequences (dots). Sequences= 8; Hap obs.= 2; Hap asympt.= NA; Hap % obs.= NA%; GST= NaN; DST= NaN; HD= NA; ND= NA; max p-dist= 0.2%.

Haplotype network analysis and bubble plot of *Thorybes confusis* were not possible. Sequences > 599 bp = 8.

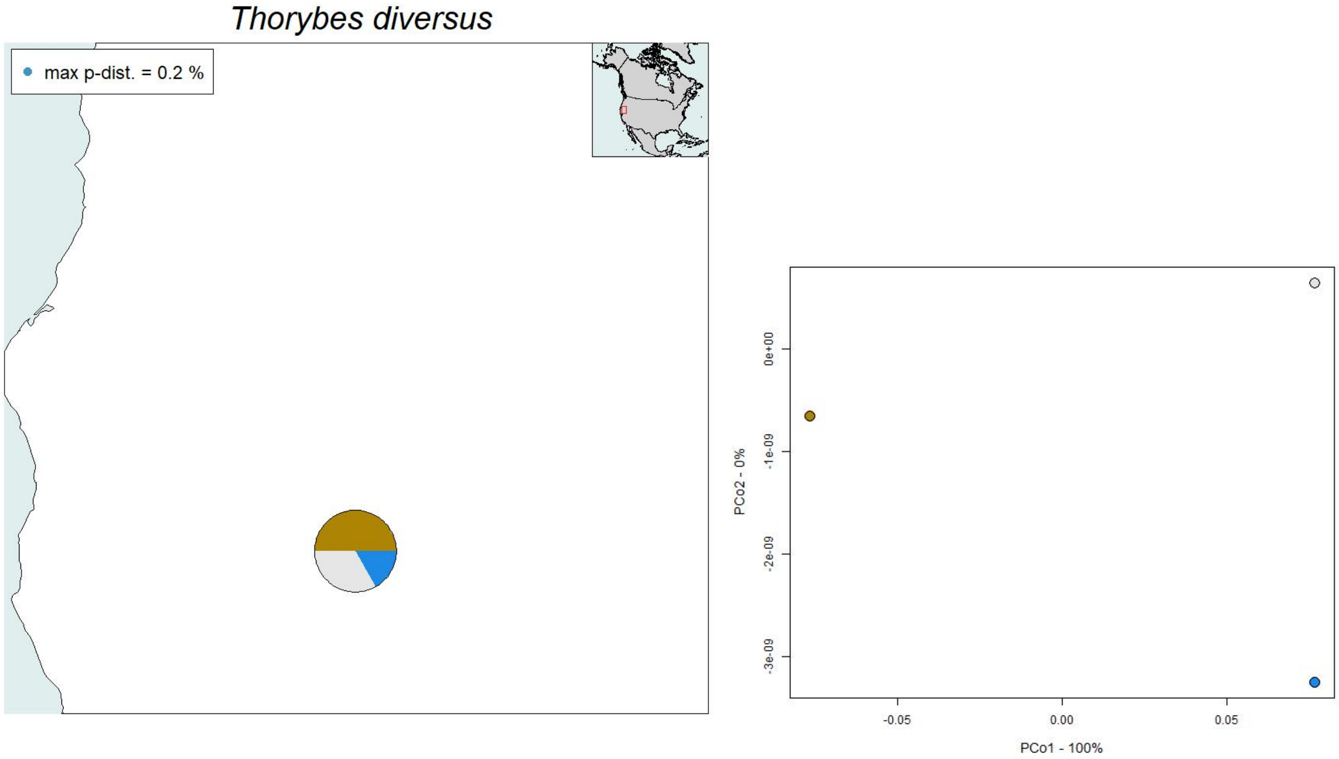

Figure 33 Map of *Thorybes diversus* showing the localities of the sequenced specimens (left). Nearby localities are grouped in pies. Colours match the bidimensional colour space of the PCoA projection (right) of max p-dists among sequences (dots). Sequences= 6; Hap obs.= 2; Hap asympt.= NA; Hap % obs.= NA%; GST= NaN; DST= NaN; HD= NA; ND= NA; max p-dist= 0.2%.

Haplotype network analysis and bubble plot of *Thorybes diversus* were not possible. Sequences > 599 bp = 6.

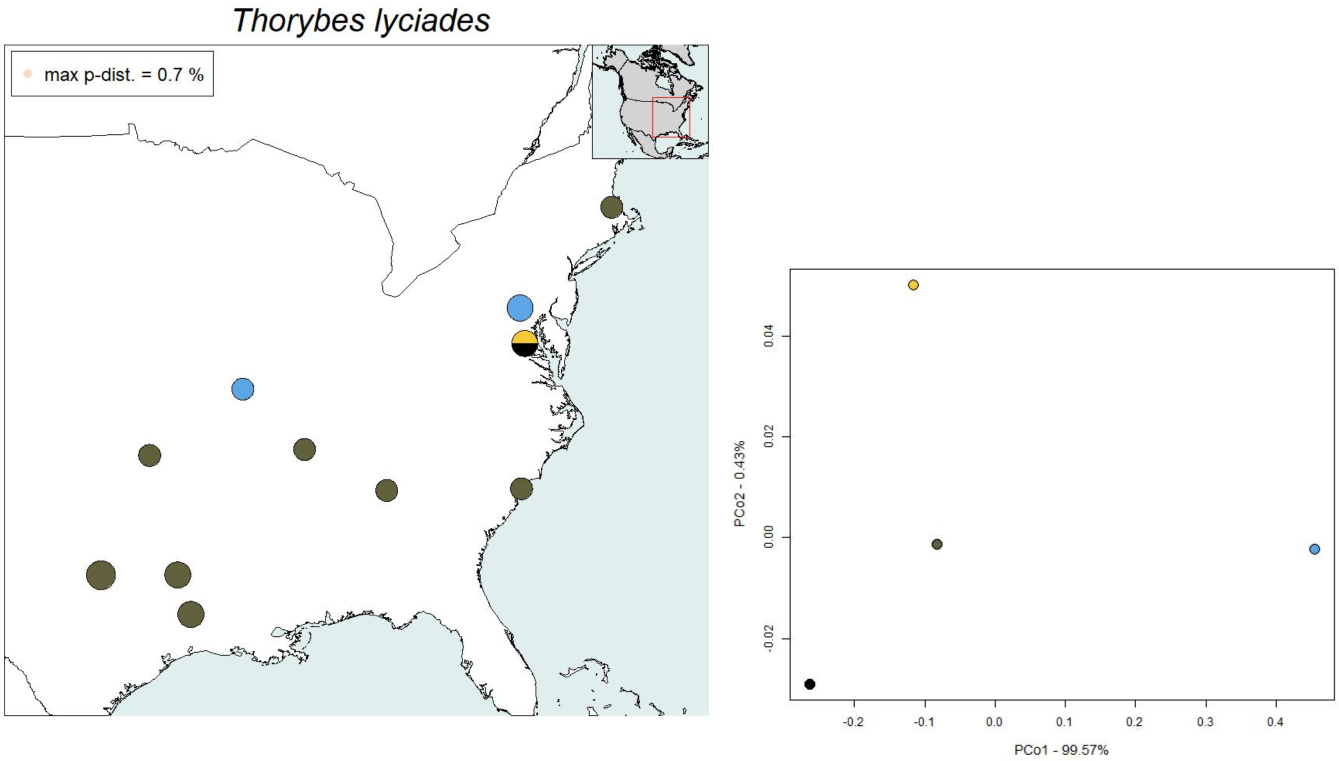

Figure 34 Map of *Thorybes lyciades* showing the localities of the sequenced specimens (left). Nearby localities are grouped in pies. Colours match the bidimensional colour space of the PCoA projection (right) of max p-dists among sequences (dots). Sequences= 17; Hap obs.= 3; Hap asympt.= 3; Hap % obs.= 100%; GST= 0.113; DST= 0.0002; HD= 0.404; ND= 0.0018; max p-dist= 0.7%.

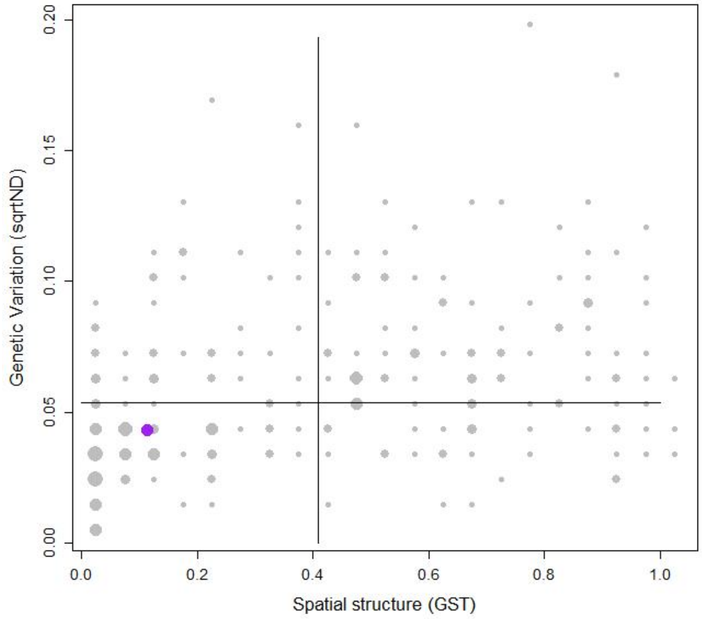

Figure 35: The bubble plot for mt-DNA polymorphism (square root transformed nucleotide diversity) and spatial structuree (GST) among all species in the atlas and values for *Thorybes lyciades* (purple dot). The horizontal and vertical lines represent median values of nucleotide diversity and GST, respectively. Haplotype network analysis was not possible. Sequences > 599 bp = 14.

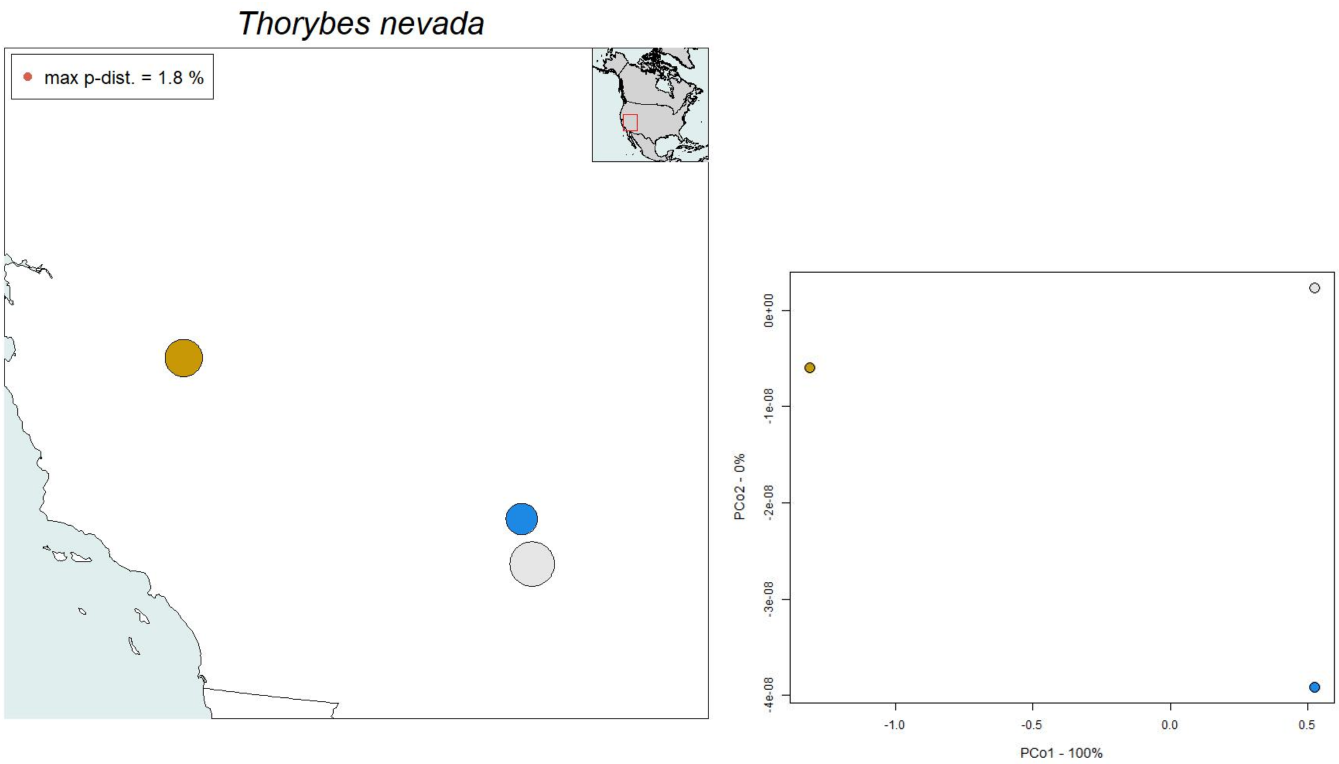

Figure 36 Map of *Thorybes nevada* showing the localities of the sequenced specimens (left). Nearby localities are grouped in pies. Colours match the bidimensional colour space of the PCoA projection (right) of max p-dists among sequences (dots). Sequences= 7; Hap obs.= 2; Hap asympt.= NA; Hap % obs.= NA%; GST= NaN; DST= NaN; HD= NA; ND= NA; max p-dist= 1.8%.

Haplotype network analysis and bubble plot of *Thorybes nevada* were not possible. Sequences > 599 bp = 7.

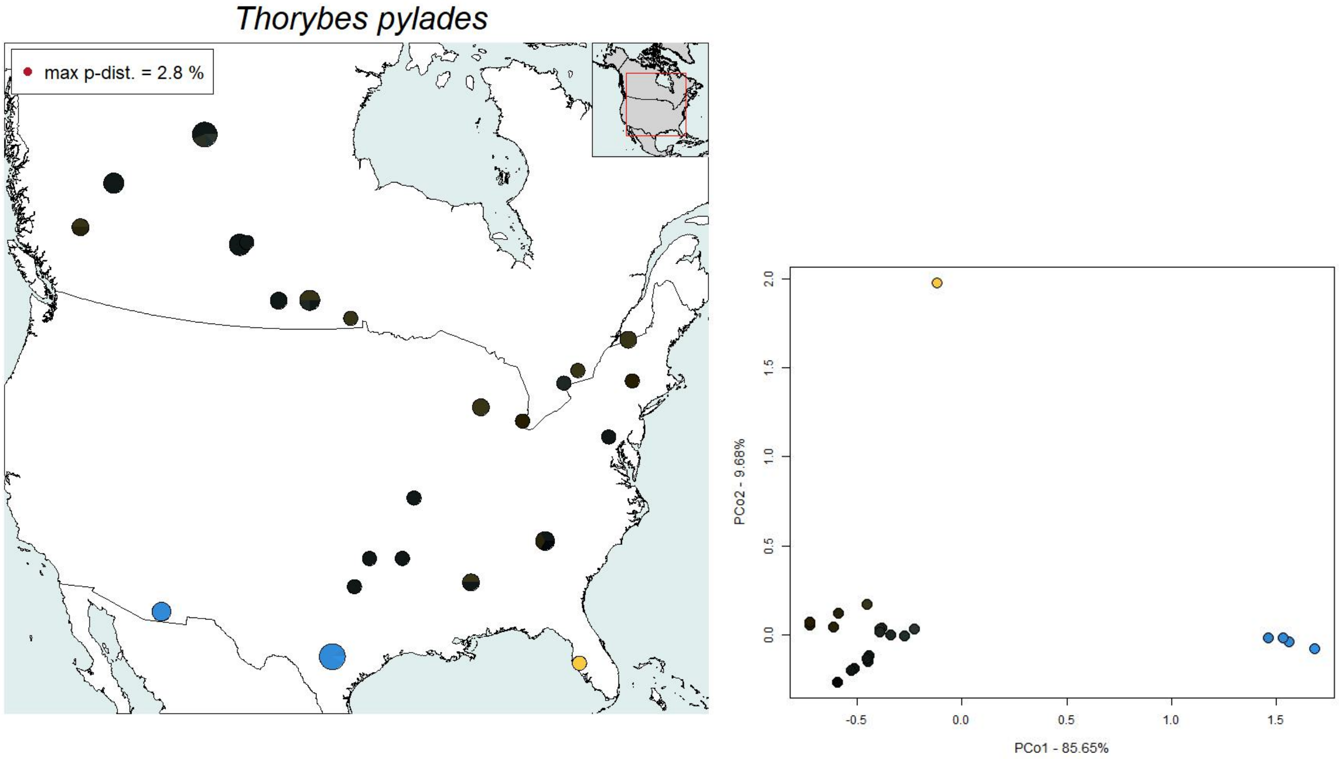

Figure 37 Map of *Thorybes pylades* showing the localities of the sequenced specimens (left). Nearby localities are grouped in pies. Colours match the bidimensional colour space of the PCoA projection (right) of max p-dists among sequences (dots). Sequences= 61; Hap obs.= 16; Hap asympt.= 32.4; Hap % obs.= 49.4%; GST= 0.958; DST= 0.0096; HD= 0.8; ND= 0.0089; max p-dist= 2.8%.

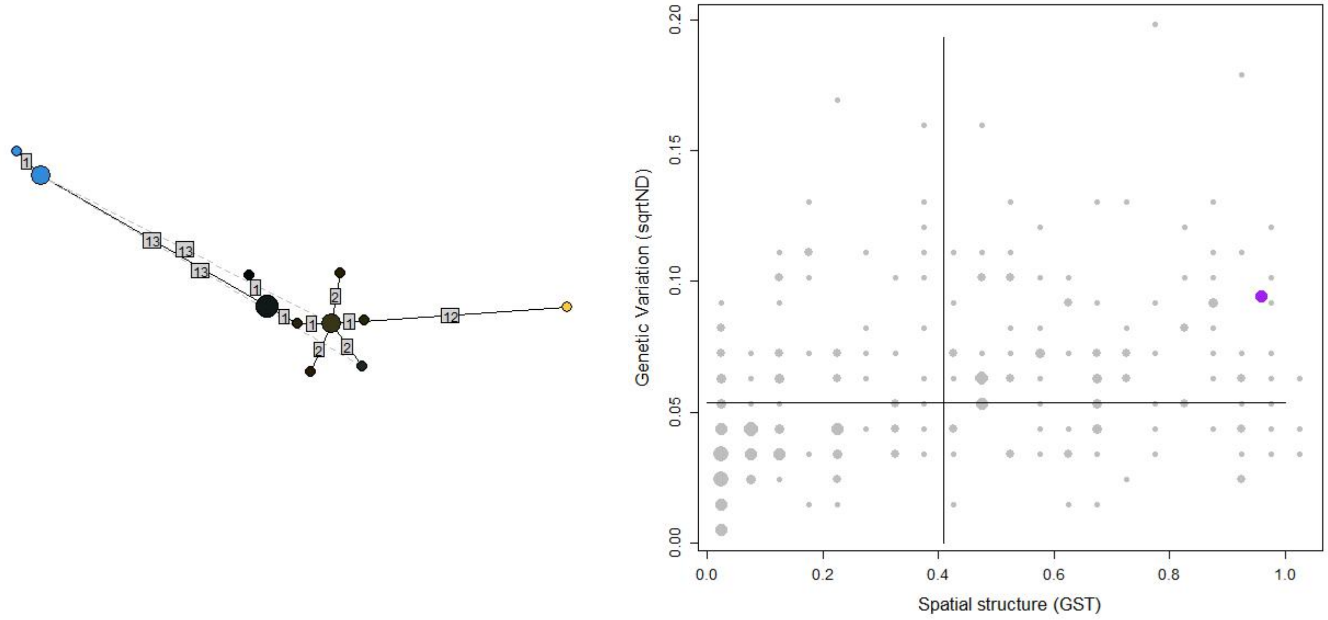

Figure 38: Haplotype network (left) of *Thorybes pylades* sequences > 599 bp with colours matching the PCoA colour space (above). The bubble plot for mt-DNA polymorphism (square root transformed nucleotide diversity) and spatial structure (GST) among all species in the atlas and values for *Thorybes pylades* (purple dot). The horizontal and vertical lines represent median values of nucleotide diversity and GST, respectively. Sequences > 599 bp= 56.

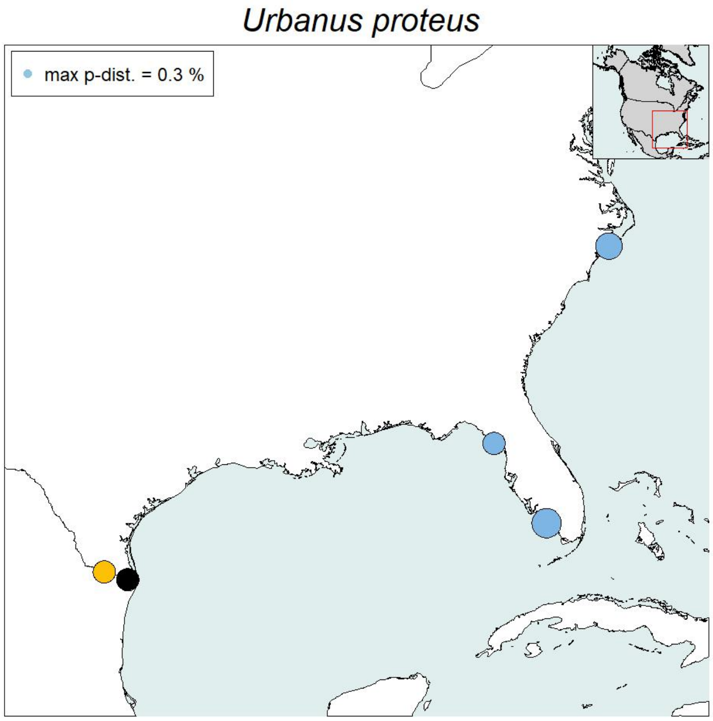

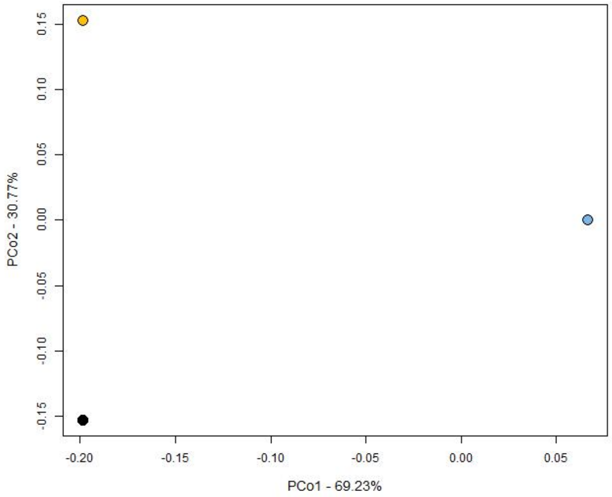

Figure 39 Map of *Urbanus proteus* showing the localities of the sequenced specimens (left). Nearby localities are grouped in pies. Colours match the bidimensional colour space of the PCoA projection (right) of max p-dists among sequences (dots). Sequences= 8; Hap obs.= 3; Hap asympt.= NA; Hap % obs.= NA%; GST= NaN; DST= NaN; HD= NA; ND= NA; max p-dist= 0.3%.

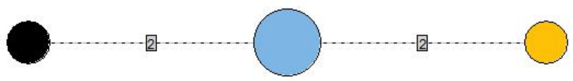

Figure 40: Haplotype network of *Urbanus proteus*. Sequences > 599 bp= 8.

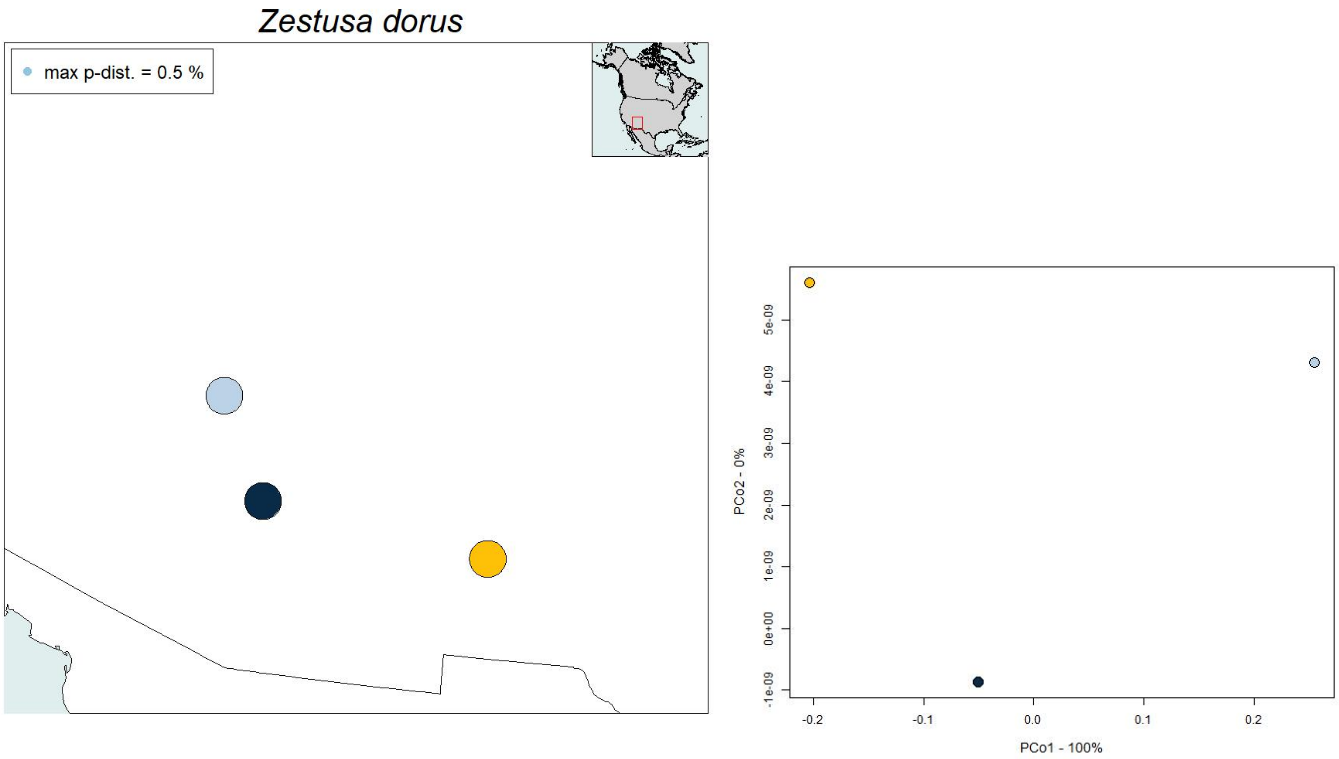

Figure 41 Map of *Zestusa dorus* showing the localities of the sequenced specimens (left). Nearby localities are grouped in pies. Colours match the bidimensional colour space of the PCoA projection (right) of max p-dists among sequences (dots). Sequences= 3; Hap obs.= 3; Hap asympt.= NA; Hap % obs.= NA%; GST= NaN; DST= NaN; HD= NA; ND= NA; max p-dist= 0.5%.

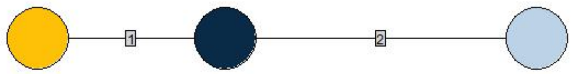

Figure 42: Haplotype network of *Zestusa dorus*. Sequences > 599 bp= 3.

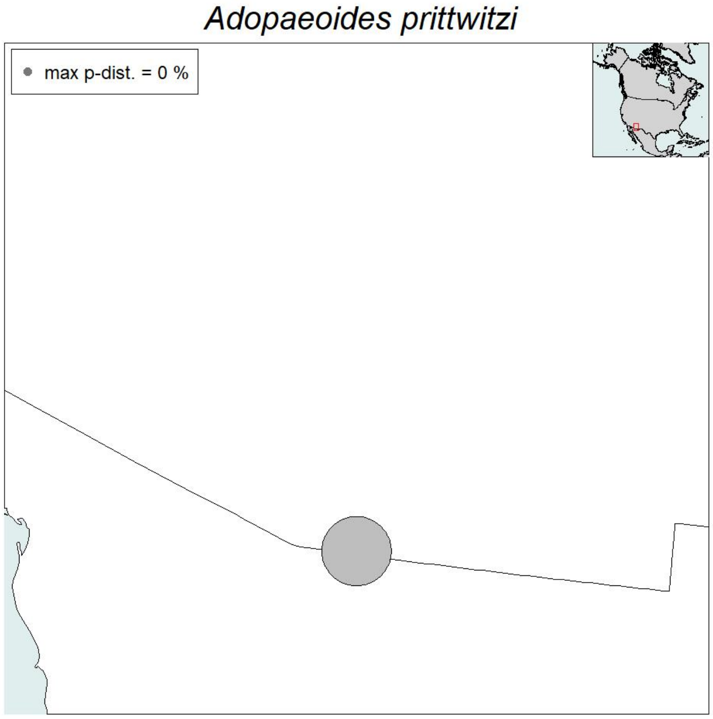

Figure 43: Map of *Adopaeoides prittwitzi* showing the localities of the sequenced specimens. Nearby localities are grouped in pies. Due to the presence of a single haplotype PCoA projection was not done and a single grey colour was plotted on the map. Sequences= 3; Hap obs.= 1; Hap asympt.= NA; Hap % obs.= NA%; GST= NaN; DST= NaN; HD= NA; ND= NA; max p-dist= 0%.

Haplotype network analysis and bubble plot of *Adopaeoides prittwitzi* were not possible. Sequences > 599 bp = 3.

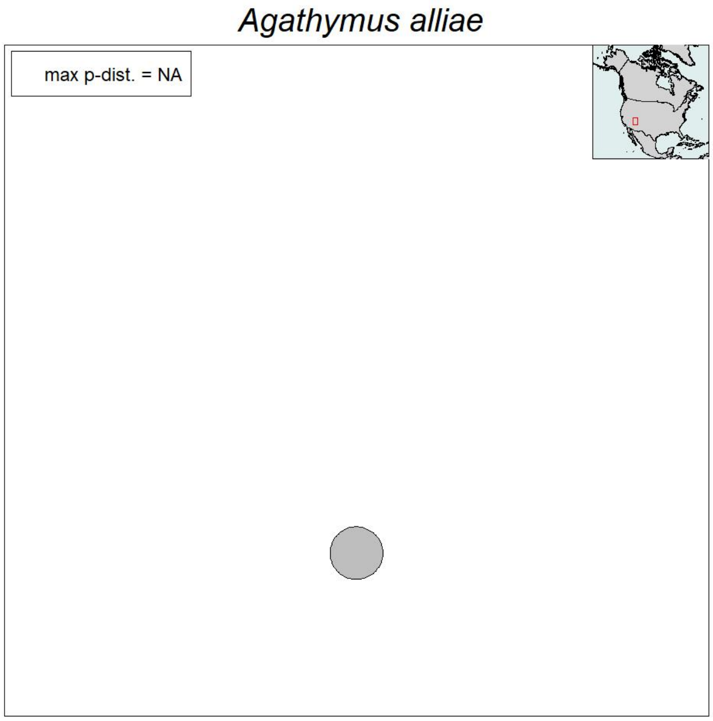

Figure 44: Map of *Agathymus alliae* showing the localities of the sequenced specimens. Nearby localities are grouped in pies. Due to the presence of a single haplotype PCoA projection was not done and a single grey colour was plotted on the map. Sequences= 1; Hap obs.= NA; Hap asympt.= NA; Hap % obs.= NA; GST= NaN; DST= NaN; HD= NA; ND= NA; max p-dist= NA.

Haplotype network analysis and bubble plot of *Agathymus alliae* were not possible. Sequences > 599 bp = 1.

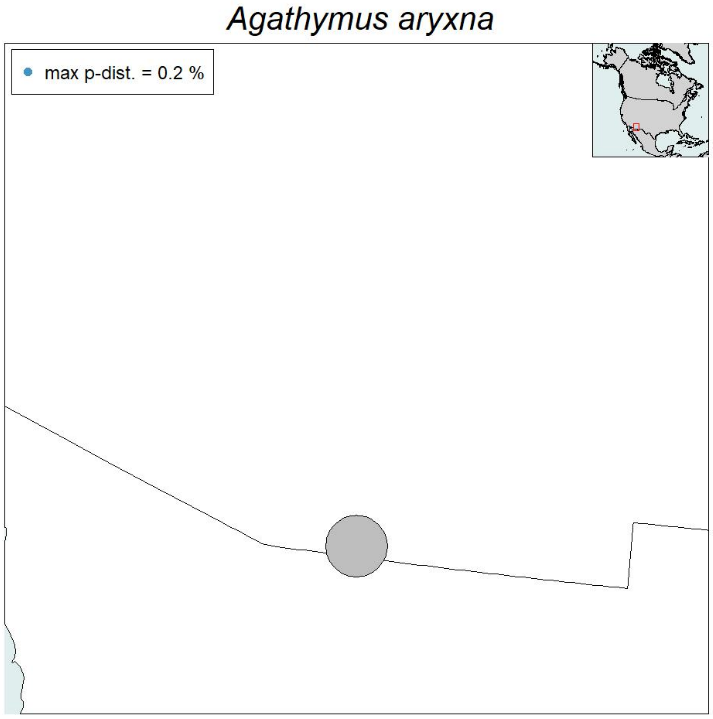

Figure 45: Map of *Agathymus aryxna* showing the localities of the sequenced specimens. Nearby localities are grouped in pies. Due to the presence of a single haplotype PCoA projection was not done and a single grey colour was plotted on the map. Sequences= 2; Hap obs.= 2; Hap asympt.= NA; Hap % obs.= NA%; GST= NaN; DST= NaN; HD= NA; ND= NA; max p-dist= 0.2%.

Haplotype network analysis and bubble plot of *Agathymus aryxna* were not possible. Sequences > 599 bp = 2.

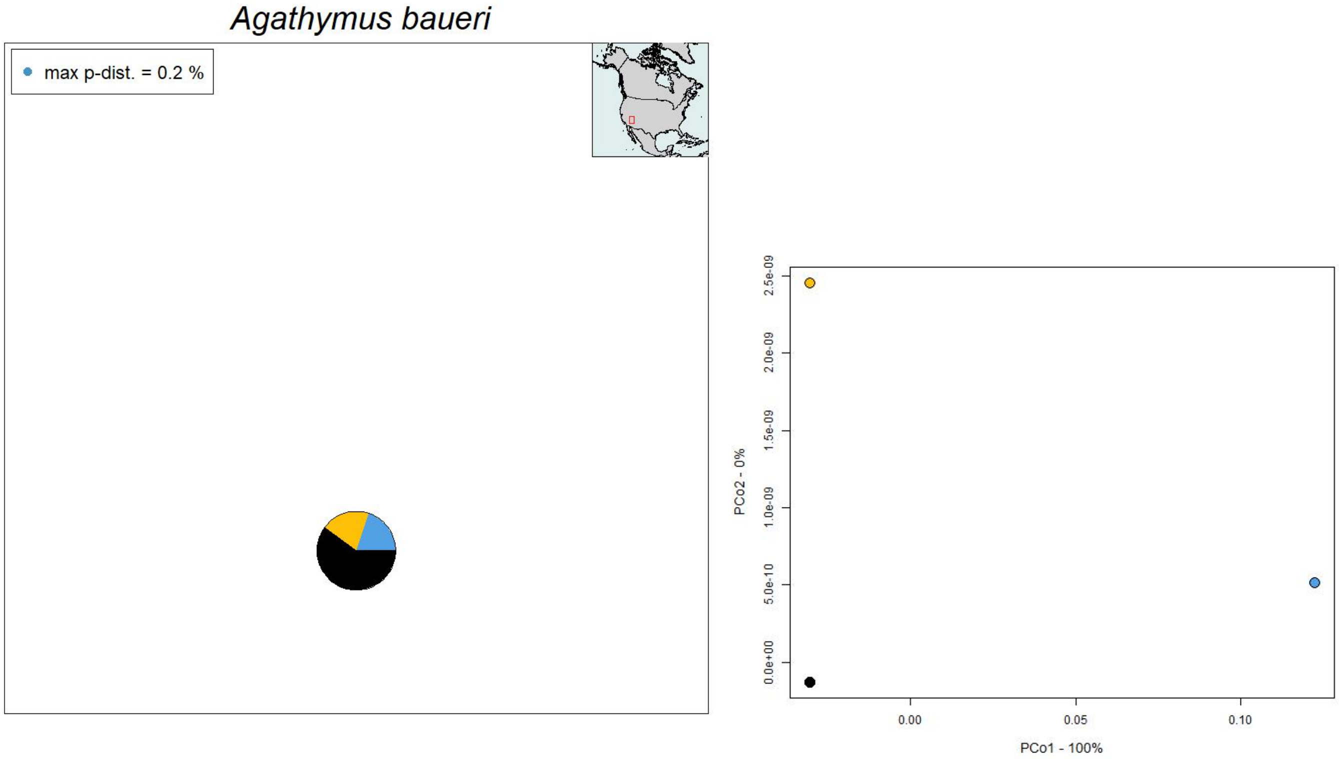

Figure 46 Map of *Agathymus baueri* showing the localities of the sequenced specimens (left). Nearby localities are grouped in pies. Colours match the bidimensional colour space of the PCoA projection (right) of max p-dists among sequences (dots). Sequences= 5; Hap obs.= 2; Hap asympt.= NA; Hap % obs.= NA%; GST= NaN; DST= NaN; HD= NA; ND= NA; max p-dist= 0.2%.

Haplotype network analysis and bubble plot of *Agathymus baueri* were not possible. Sequences > 599 bp = 5.

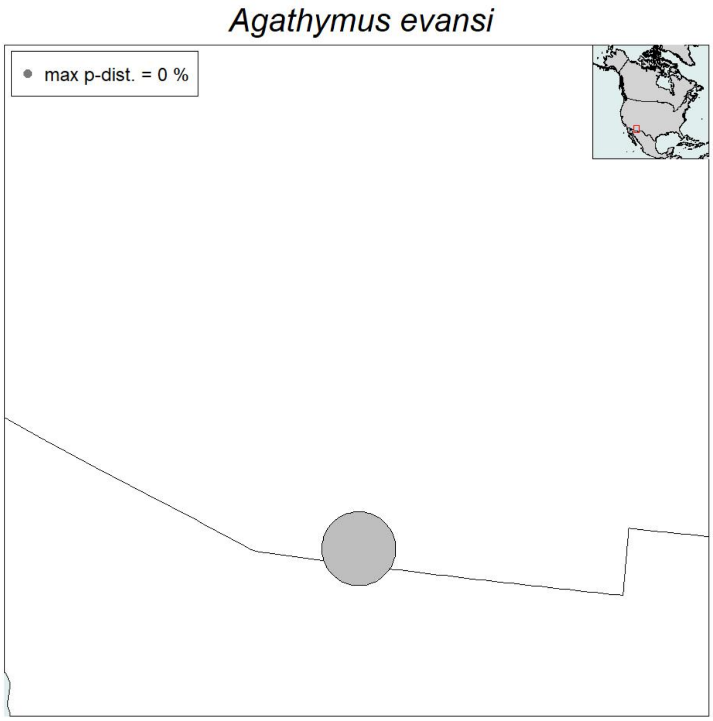

Figure 47: Map of *Agathymus evansi* showing the localities of the sequenced specimens. Nearby localities are grouped in pies. Due to the presence of a single haplotype PCoA projection was not done and a single grey colour was plotted on the map. Sequences= 4; Hap obs.= 1; Hap asympt.= NA; Hap % obs.= NA%; GST= NaN; DST= NaN; HD= NA; ND= NA; max p-dist= 0%.

Haplotype network analysis and bubble plot of *Agathymus evansi* were not possible. Sequences > 599 bp = 4.

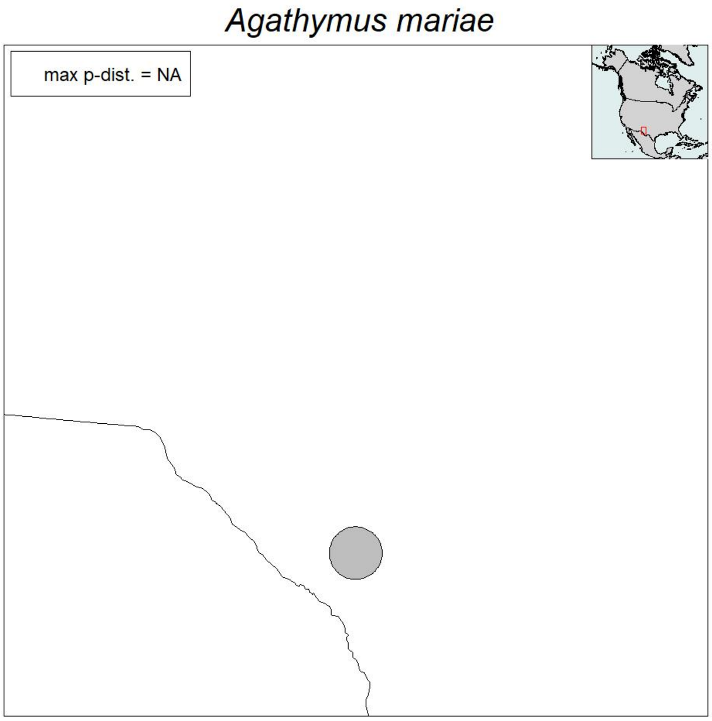

Figure 48: Map of *Agathymus mariae* showing the localities of the sequenced specimens. Nearby localities are grouped in pies. Due to the presence of a single haplotype PCoA projection was not done and a single grey colour was plotted on the map. Sequences= 1; Hap obs.= NA; Hap asympt.= NA; Hap % obs.= NA; GST= NaN; DST= NaN; HD= NA; ND= NA; max p-dist= NA.

Haplotype network analysis and bubble plot of *Agathymus mariae* were not possible. Sequences > 599 bp = 1.

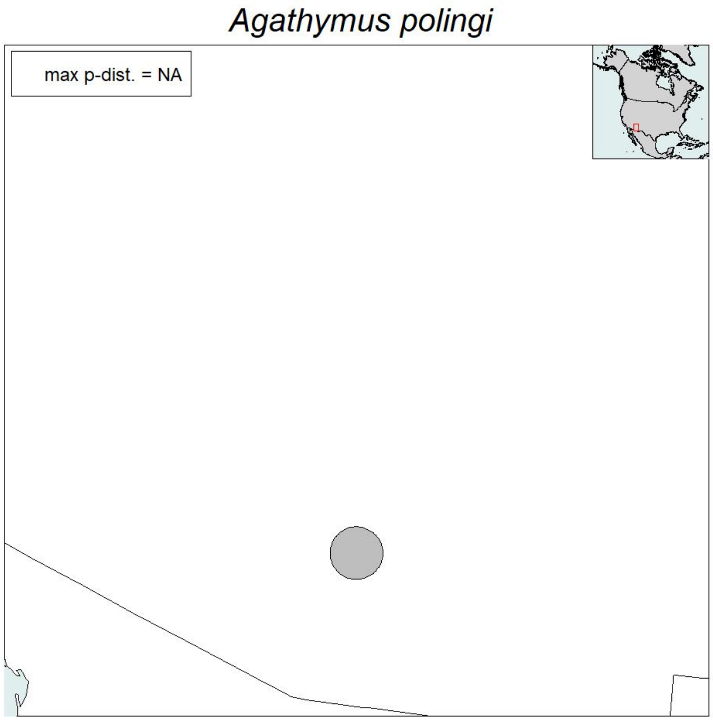

Figure 49: Map of *Agathymus polingi* showing the localities of the sequenced specimens. Nearby localities are grouped in pies. Due to the presence of a single haplotype PCoA projection was not done and a single grey colour was plotted on the map. Sequences= 1; Hap obs.= NA; Hap asympt.= NA; Hap % obs.= NA; GST= NaN; DST= NaN; HD= NA; ND= NA; max p-dist= NA.

Haplotype network analysis and bubble plot of *Agathymus polingi* were not possible. Sequences > 599 bp = 1.

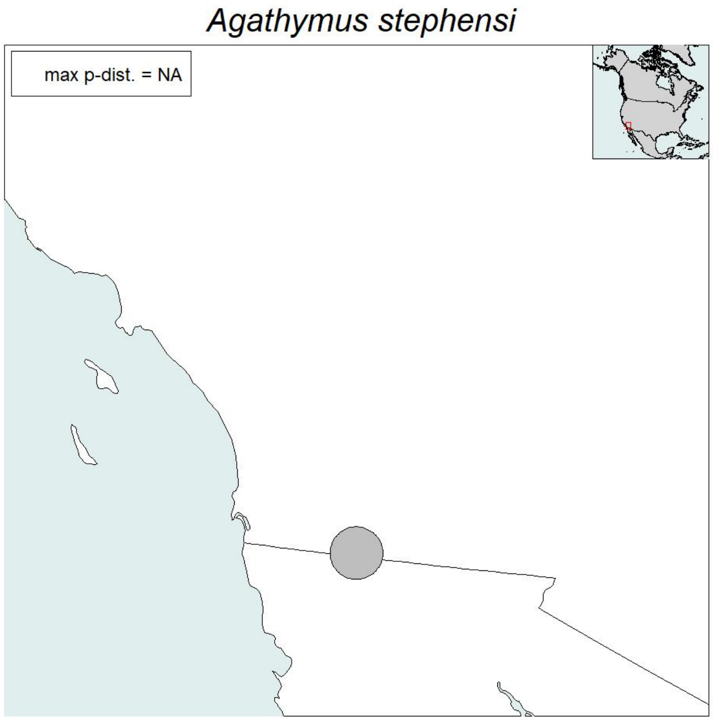

Figure 50: Map of *Agathymus stephensi* showing the localities of the sequenced specimens. Nearby localities are grouped in pies. Due to the presence of a single haplotype PCoA projection was not done and a single grey colour was plotted on the map. Sequences= 1; Hap obs.= NA; Hap asympt.= NA; Hap % obs.= NA; GST= NaN; DST= NaN; HD= NA; ND= NA; max p-dist= NA.

Haplotype network analysis and bubble plot of *Agathymus stephensi* were not possible. Sequences > 599 bp = 1.

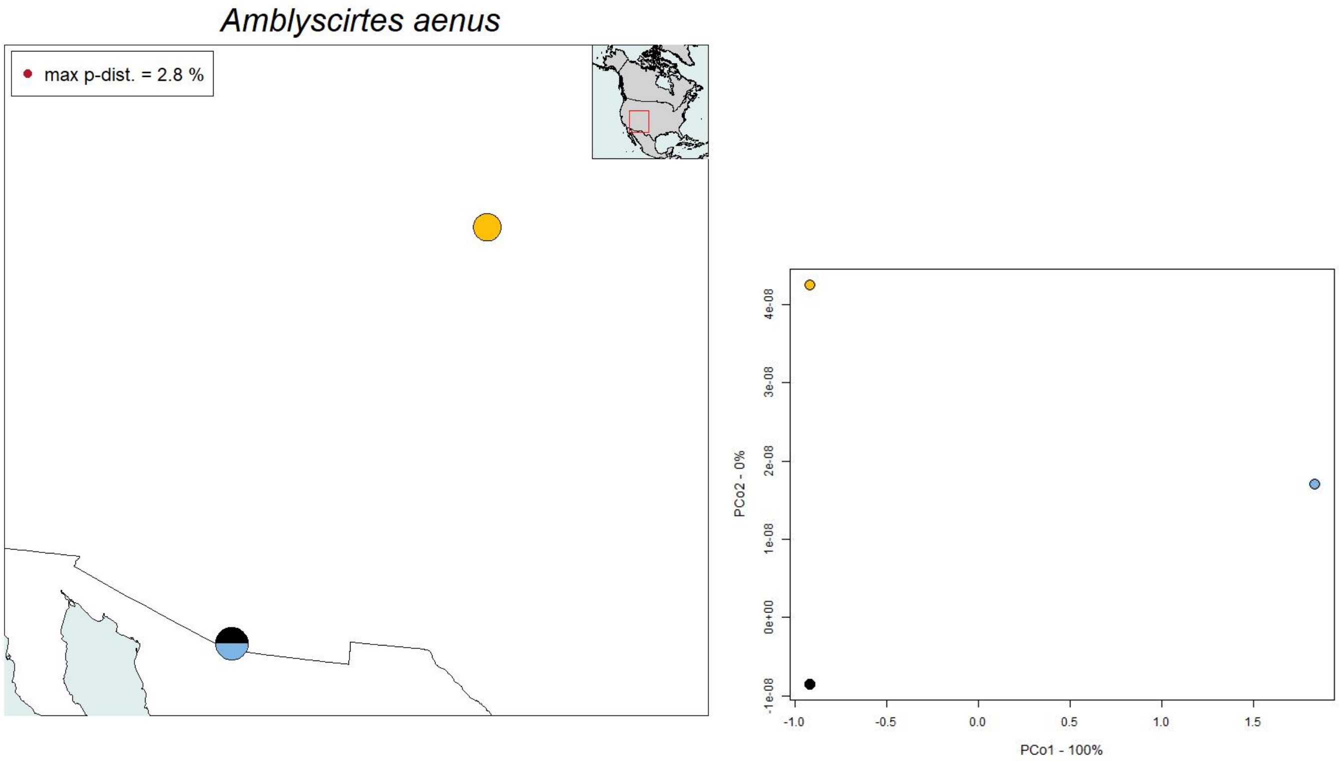

Figure 51 Map of *Amblyscirtes aenus* showing the localities of the sequenced specimens (left). Nearby localities are grouped in pies. Colours match the bidimensional colour space of the PCoA projection (right) of max p-dists among sequences (dots). Sequences= 3; Hap obs.= 2; Hap asympt.= NA; Hap % obs.= NA%; GST= NaN; DST= NaN; HD= NA; ND= NA; max p-dist= 2.8%.

Haplotype network analysis and bubble plot of *Amblyscirtes aenus* were not possible. Sequences > 599 bp = 3.

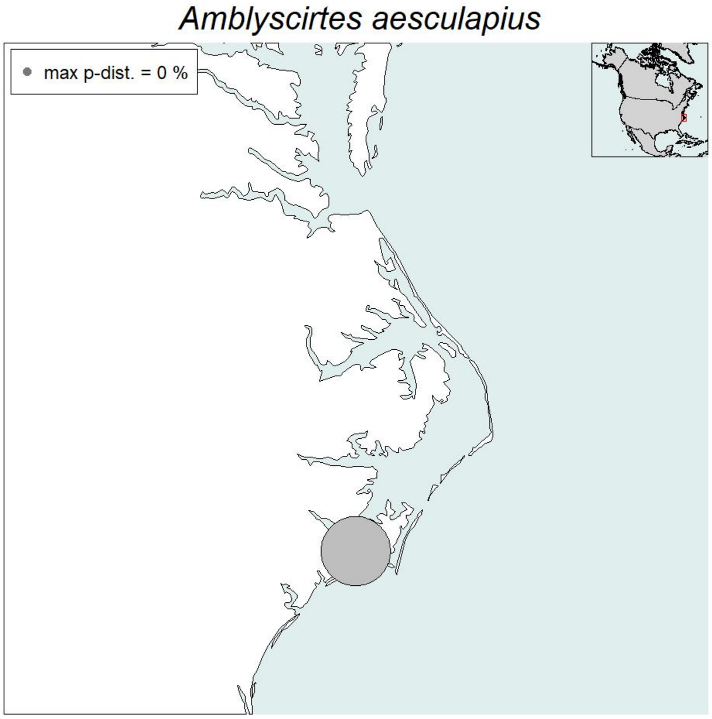

Figure 52: Map of *Amblyscirtes aesculapius* showing the localities of the sequenced specimens. Nearby localities are grouped in pies. Due to the presence of a single haplotype PCoA projection was not done and a single grey colour was plotted on the map. Sequences= 3; Hap obs.= 1; Hap asympt.= NA; Hap % obs.= NA%; GST= NaN; DST= NaN; HD= NA; ND= NA; max p-dist= 0%.

Haplotype network analysis and bubble plot of *Amblyscirtes aesculapius* were not possible. Sequences > 599 bp = 3.

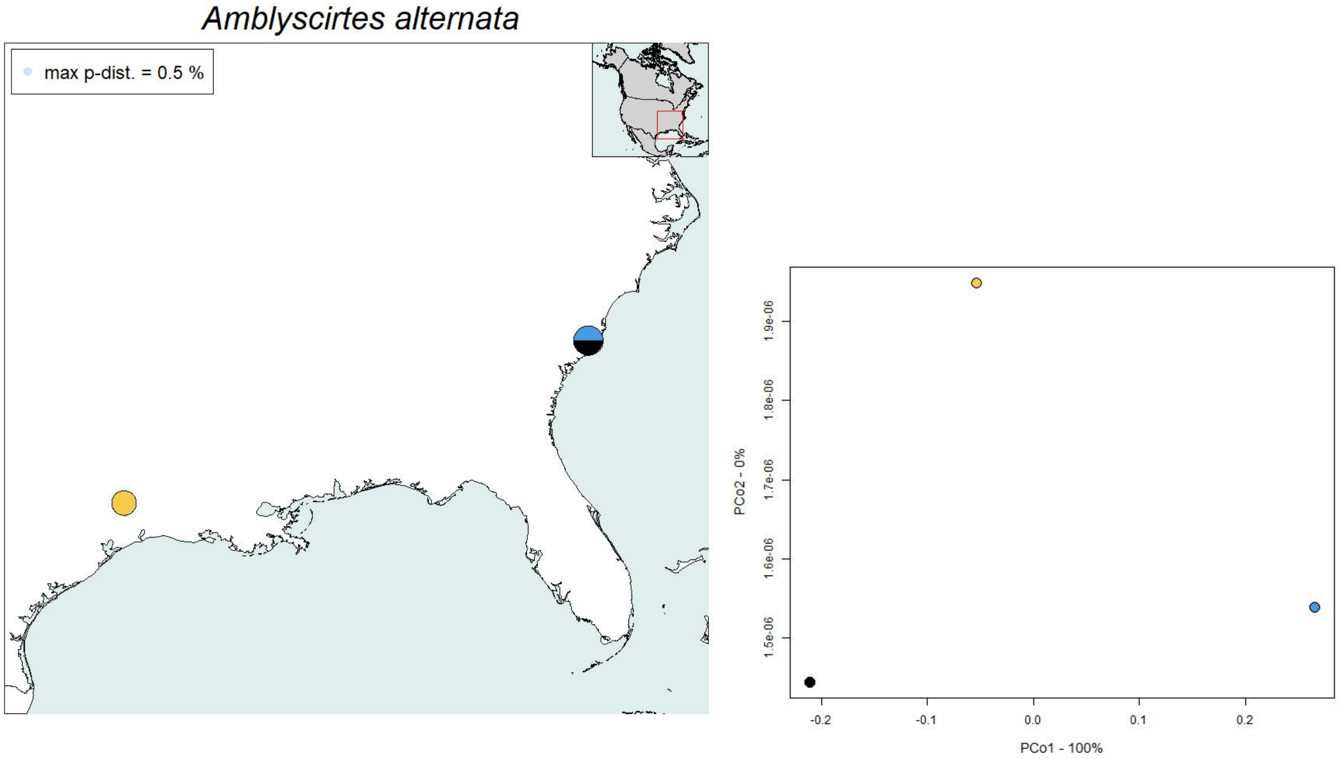

Figure 53 Map of *Amblyscirtes alternata* showing the localities of the sequenced specimens (left). Nearby localities are grouped in pies. Colours match the bidimensional colour space of the PCoA projection (right) of max p-dists among sequences (dots). Sequences= 3; Hap obs.= 3; Hap asympt.= NA; Hap % obs.= NA%; GST= NaN; DST= NaN; HD= NA; ND= NA; max p-dist= 0.5%.

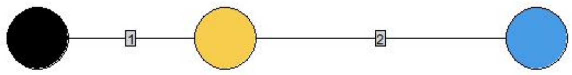

Figure 54: Haplotype network of *Amblyscirtes alternata*. Sequences > 599 bp= 3.

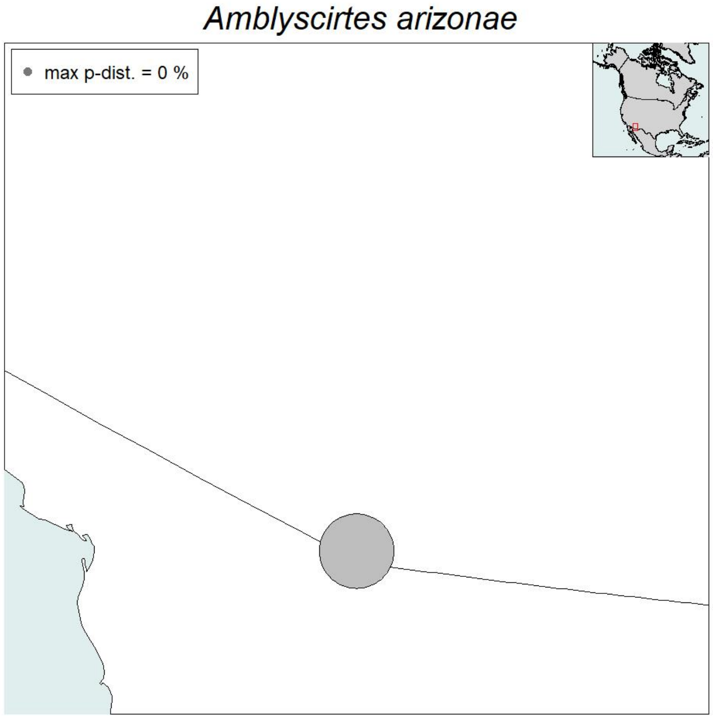

Figure 55: Map of *Amblyscirtes arizonae* showing the localities of the sequenced specimens. Nearby localities are grouped in pies. Due to the presence of a single haplotype PCoA projection was not done and a single grey colour was plotted on the map. Sequences= 4; Hap obs.= 1; Hap asympt.= NA; Hap % obs.= NA%; GST= NaN; DST= NaN; HD= NA; ND= NA; max p-dist= 0%.

Haplotype network analysis and bubble plot of *Amblyscirtes arizonae* were not possible. Sequences > 599 bp = 4.

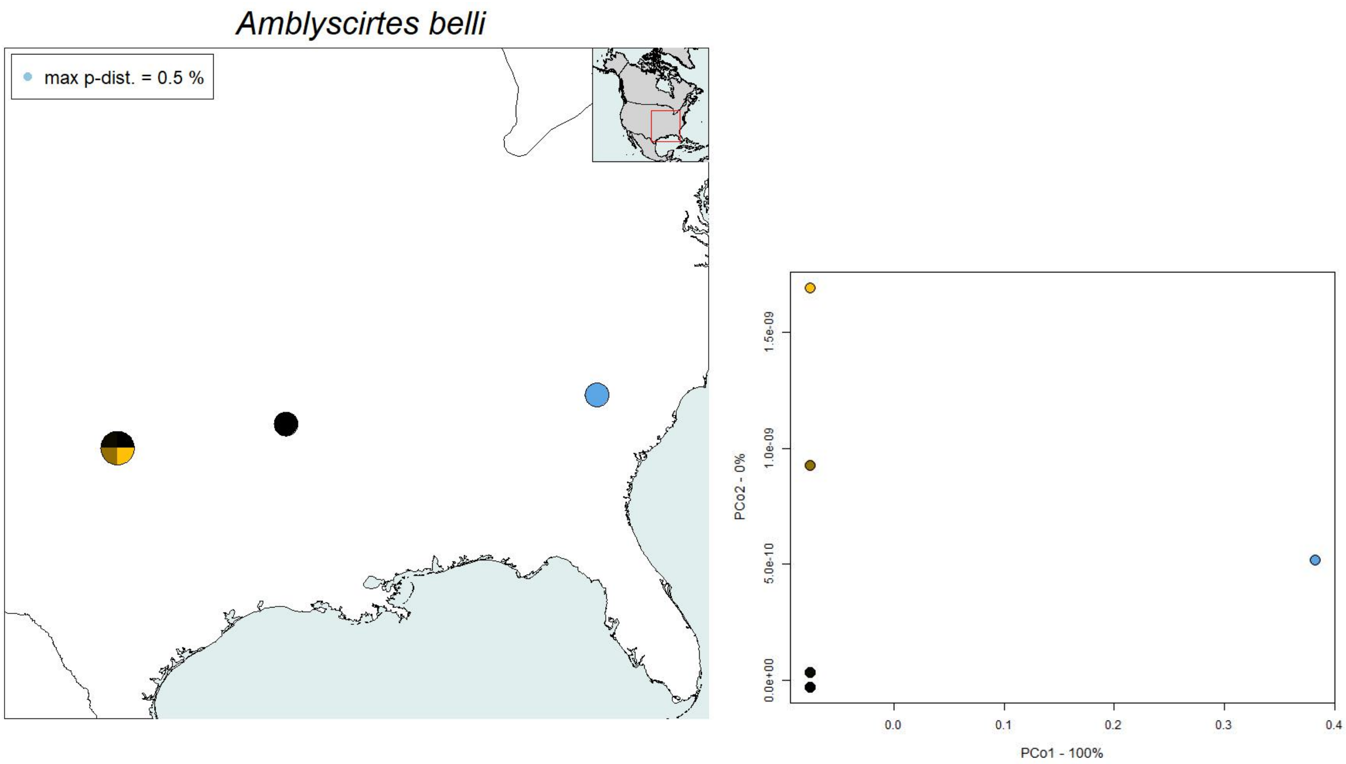

Figure 56 Map of *Amblyscirtes belli* showing the localities of the sequenced specimens (left). Nearby localities are grouped in pies. Colours match the bidimensional colour space of the PCoA projection (right) of max p-dists among sequences (dots). Sequences= 6; Hap obs.= 2; Hap asympt.= NA; Hap % obs.= NA%; GST= NaN; DST= NaN; HD= NA; ND= NA; max p-dist= 0.5%.

Haplotype network analysis and bubble plot of *Amblyscirtes belli* were not possible. Sequences > 599 bp = 6.

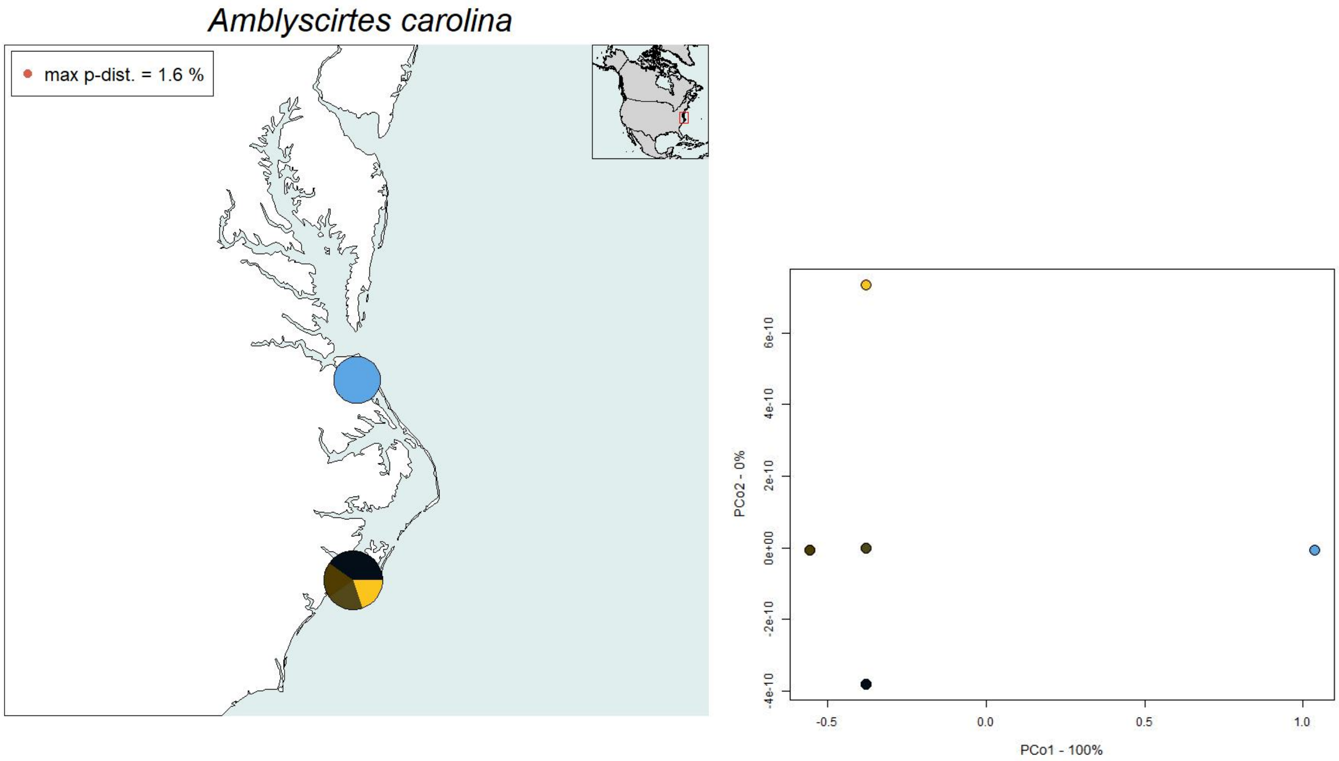

Figure 57 Map of *Amblyscirtes carolina* showing the localities of the sequenced specimens (left). Nearby localities are grouped in pies. Colours match the bidimensional colour space of the PCoA projection (right) of max p-dists among sequences (dots). Sequences= 7; Hap obs.= 3; Hap asympt.= NA; Hap % obs.= NA%; GST= NaN; DST= NaN; HD= NA; ND= NA; max p-dist= 1.6%.

Haplotype network analysis and bubble plot of *Amblyscirtes carolina* were not possible. Sequences > 599 bp = 5.

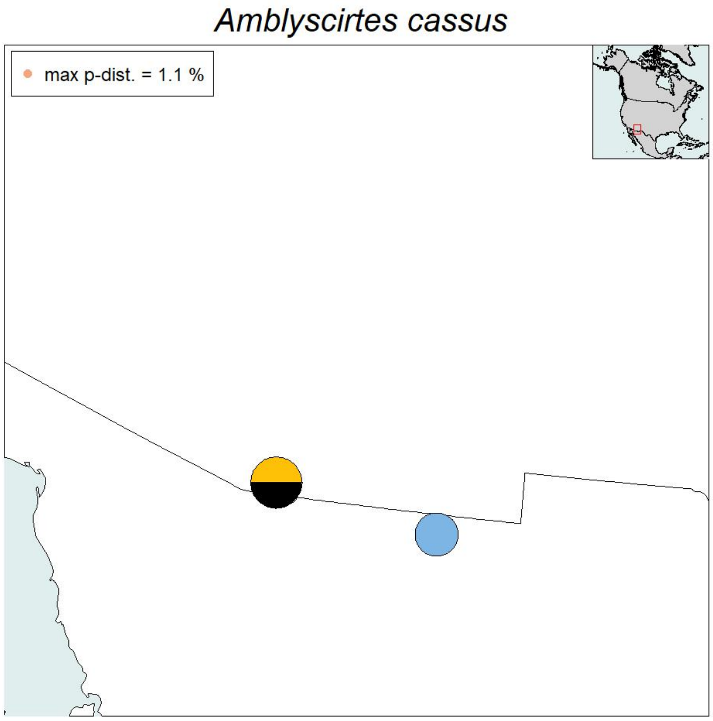

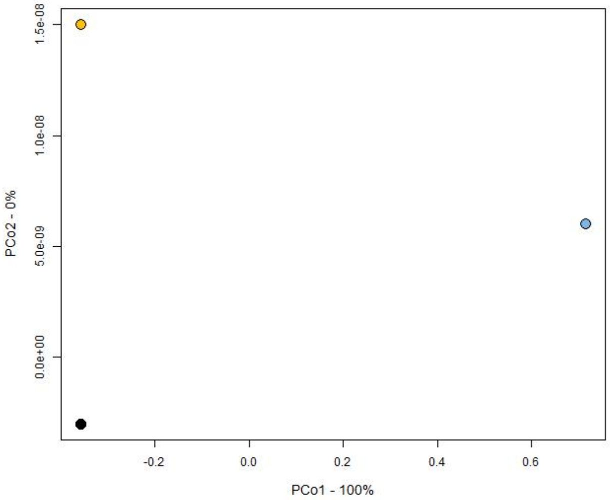

Figure 58 Map of *Amblyscirtes cassus* showing the localities of the sequenced specimens (left). Nearby localities are grouped in pies. Colours match the bidimensional colour space of the PCoA projection (right) of max p-dists among sequences (dots). Sequences= 3; Hap obs.= 2; Hap asympt.= NA; Hap % obs.= NA%; GST= NaN; DST= NaN; HD= NA; ND= NA; max p-dist= 1.1%.

Haplotype network analysis and bubble plot of *Amblyscirtes cassus* were not possible. Sequences > 599 bp = 3.

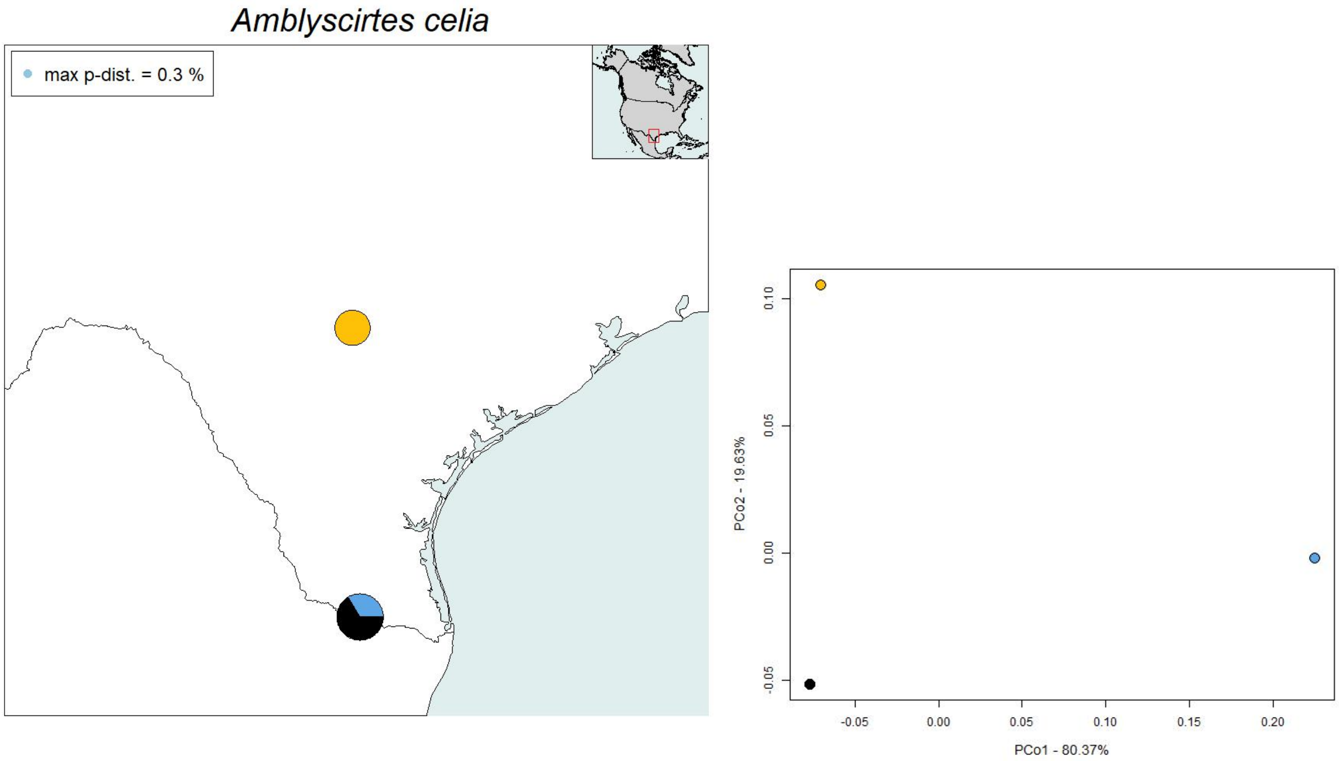

Figure 59 Map of *Amblyscirtes celia* showing the localities of the sequenced specimens (left). Nearby localities are grouped in pies. Colours match the bidimensional colour space of the PCoA projection (right) of max p-dists among sequences (dots). Sequences= 4; Hap obs.= 3; Hap asympt.= NA; Hap % obs.= NA%; GST= NaN; DST= NaN; HD= NA; ND= NA; max p-dist= 0.3%.

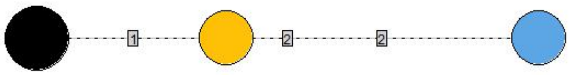

Figure 60: Haplotype network of *Amblyscirtes celia*. Sequences > 599 bp= 4.

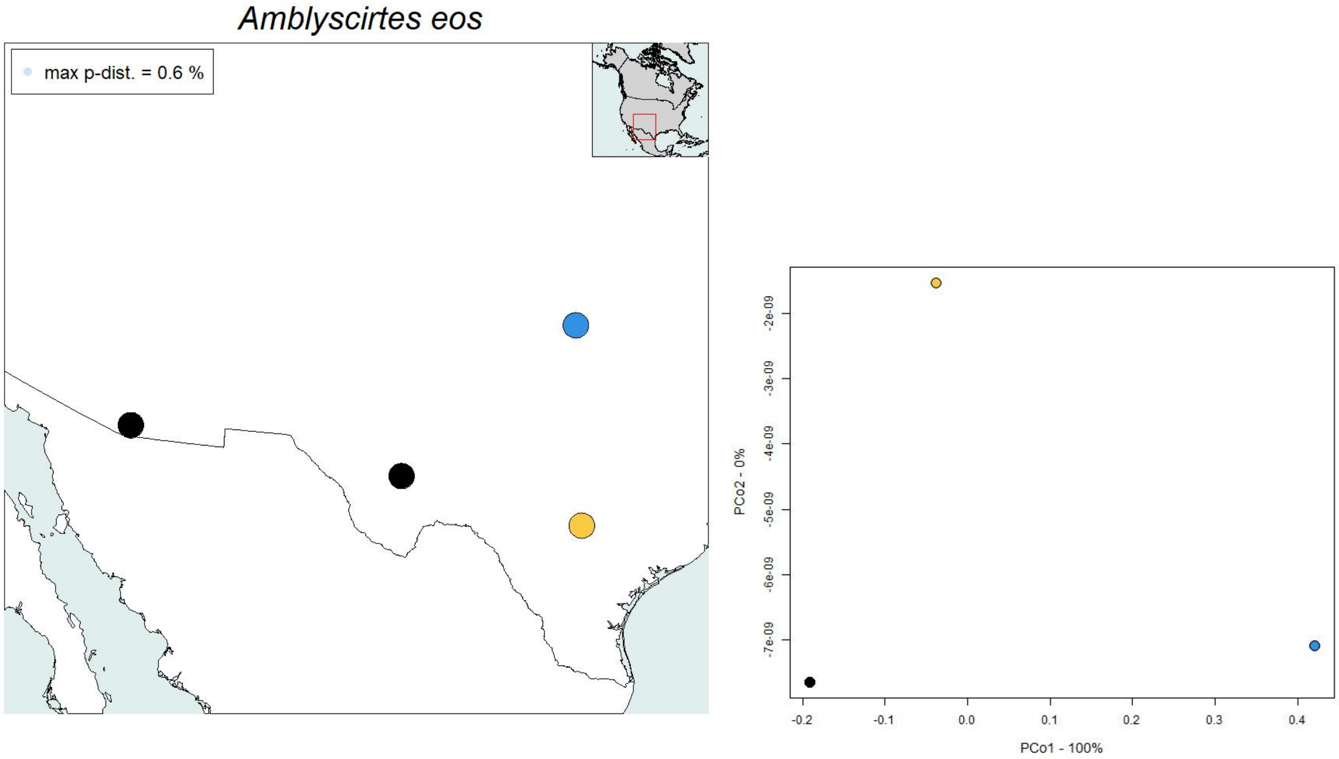

Figure 61 Map of *Amblyscirtes eos* showing the localities of the sequenced specimens (left). Nearby localities are grouped in pies. Colours match the bidimensional colour space of the PCoA projection (right) of max p-dists among sequences (dots). Sequences= 4; Hap obs.= 3; Hap asympt.= NA; Hap % obs.= NA%; GST= NaN; DST= NaN; HD= NA; ND= NA; max p-dist= 0.6%.

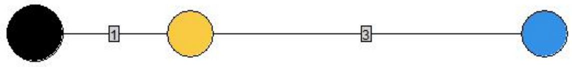

Figure 62: Haplotype network of *Amblyscirtes eos*. Sequences > 599 bp= 4.

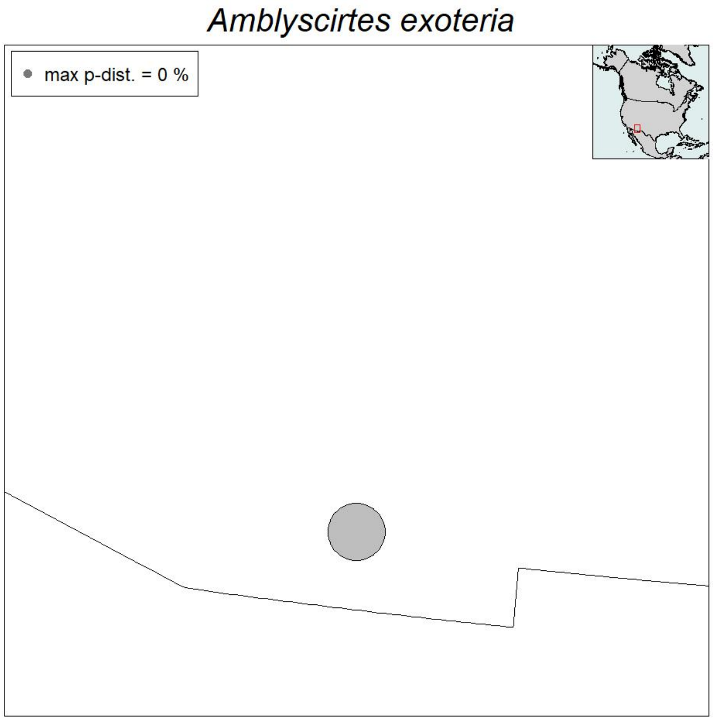

Figure 63: Map of *Amblyscirtes exoteria* showing the localities of the sequenced specimens. Nearby localities are grouped in pies. Due to the presence of a single haplotype PCoA projection was not done and a single grey colour was plotted on the map. Sequences= 2; Hap obs.= 1; Hap asympt.= NA; Hap % obs.= NA%; GST= NaN; DST= NaN; HD= NA; ND= NA; max p-dist= 0%.

Haplotype network analysis and bubble plot of *Amblyscirtes exoteria* were not possible. Sequences > 599 bp = 2.

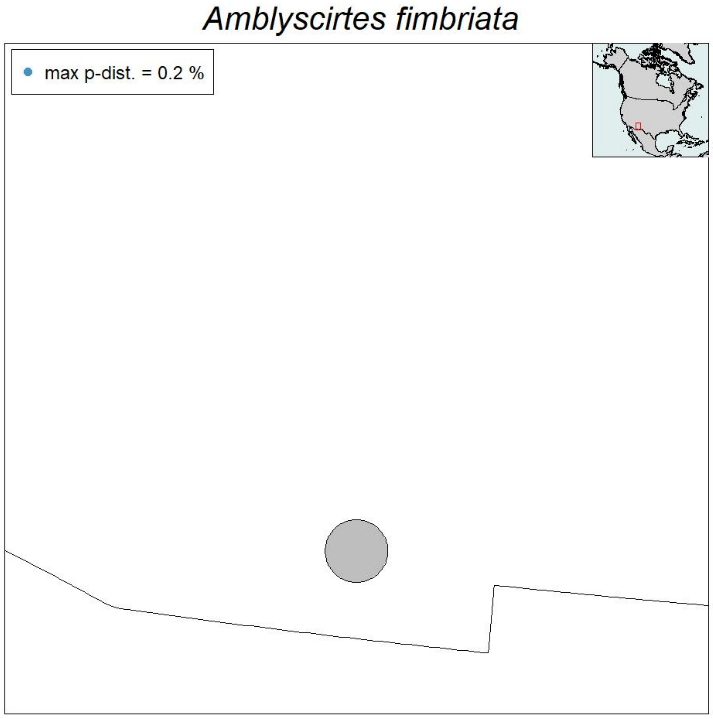

Figure 64: Map of *Amblyscirtes fimbriata* showing the localities of the sequenced specimens. Nearby localities are grouped in pies. Due to the presence of a single haplotype PCoA projection was not done and a single grey colour was plotted on the map. Sequences= 2; Hap obs.= 2; Hap asympt.= NA; Hap % obs.= NA%; GST= NaN; DST= NaN; HD= NA; ND= NA; max p-dist= 0.2%.

Haplotype network analysis and bubble plot of *Amblyscirtes fimbriata* were not possible. Sequences > 599 bp = 0.

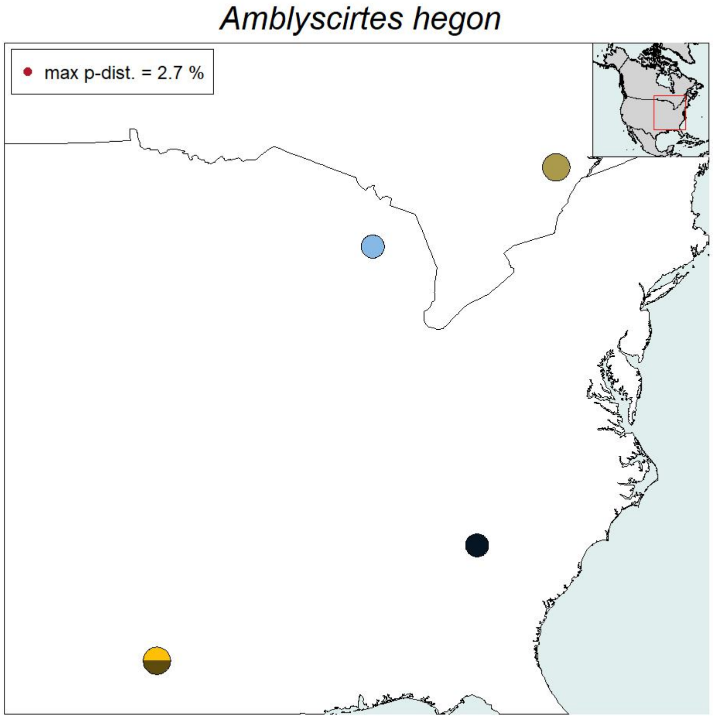

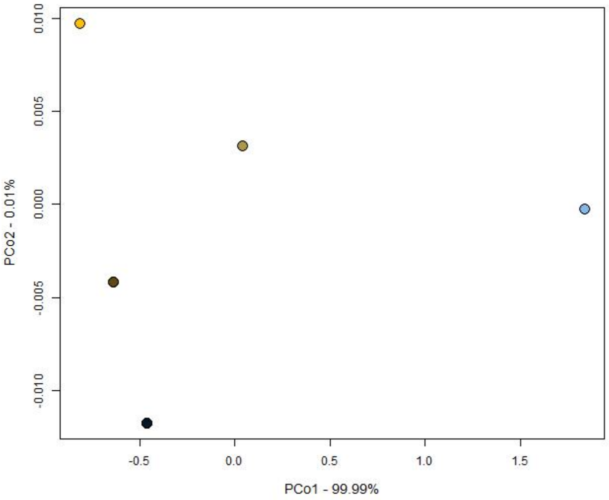

Figure 65 Map of *Amblyscirtes hegon* showing the localities of the sequenced specimens (left). Nearby localities are grouped in pies. Colours match the bidimensional colour space of the PCoA projection (right) of max p-dists among sequences (dots). Sequences= 6; Hap obs.= 5; Hap asympt.= NA; Hap % obs.= NA%; GST= NaN; DST= NaN; HD= NA; ND= NA; max p-dist= 2.7%.

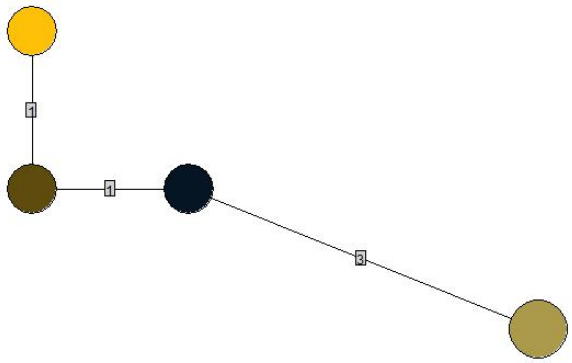

Figure 66: Haplotype network of *Amblyscirtes hegon*. Sequences > 599 bp= 5.

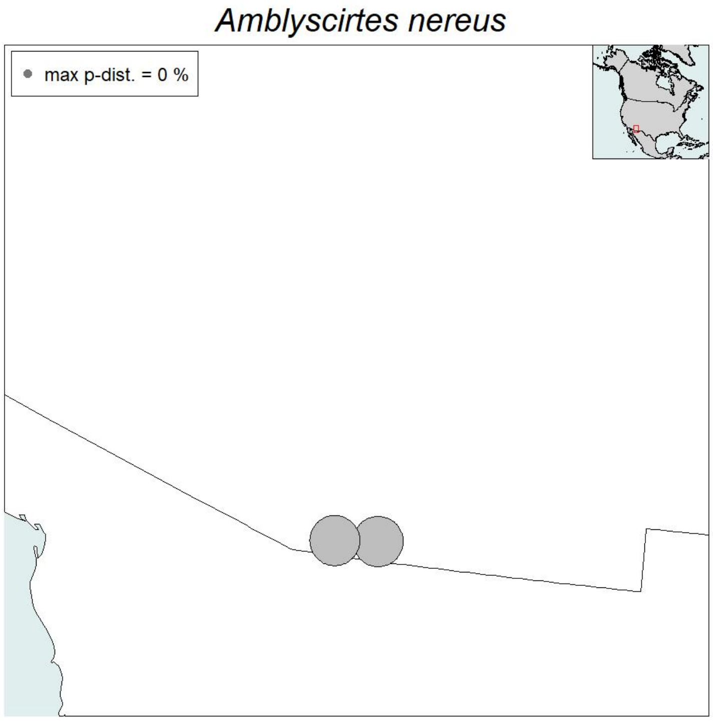

Figure 67: Map of *Amblyscirtes nereus* showing the localities of the sequenced specimens. Nearby localities are grouped in pies. Due to the presence of a single haplotype PCoA projection was not done and a single grey colour was plotted on the map. Sequences= 2; Hap obs.= 1; Hap asympt.= NA; Hap % obs.= NA%; GST= NaN; DST= NaN; HD= NA; ND= NA; max p-dist= 0%.

Haplotype network analysis and bubble plot of *Amblyscirtes nereus* were not possible. Sequences > 599 bp = 2.

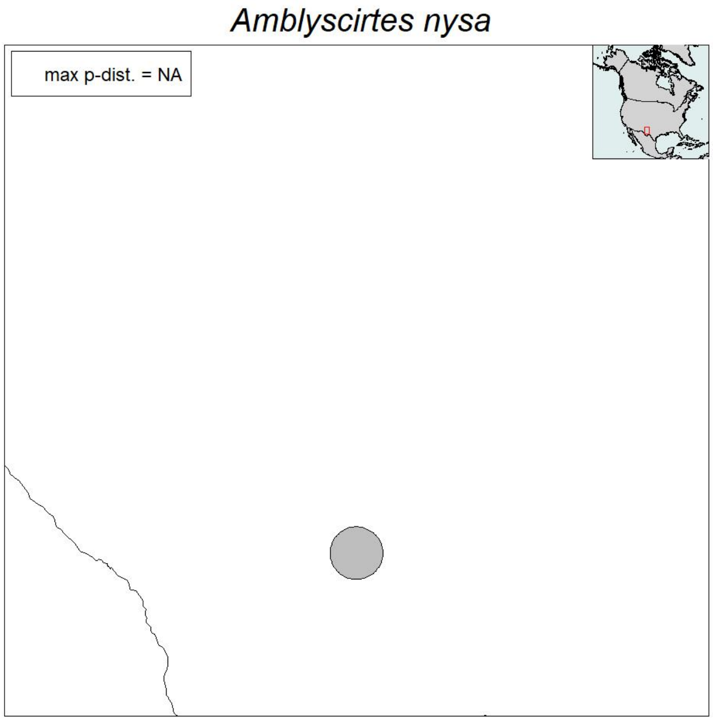

Figure 68: Map of *Amblyscirtes nysa* showing the localities of the sequenced specimens. Nearby localities are grouped in pies. Due to the presence of a single haplotype PCoA projection was not done and a single grey colour was plotted on the map. Sequences= 1; Hap obs.= NA; Hap asympt.= NA; Hap % obs.= NA; GST= NaN; DST= NaN; HD= NA; ND= NA; max p-dist= NA.

Haplotype network analysis and bubble plot of *Amblyscirtes nysa* were not possible. Sequences > 599 bp = 1.

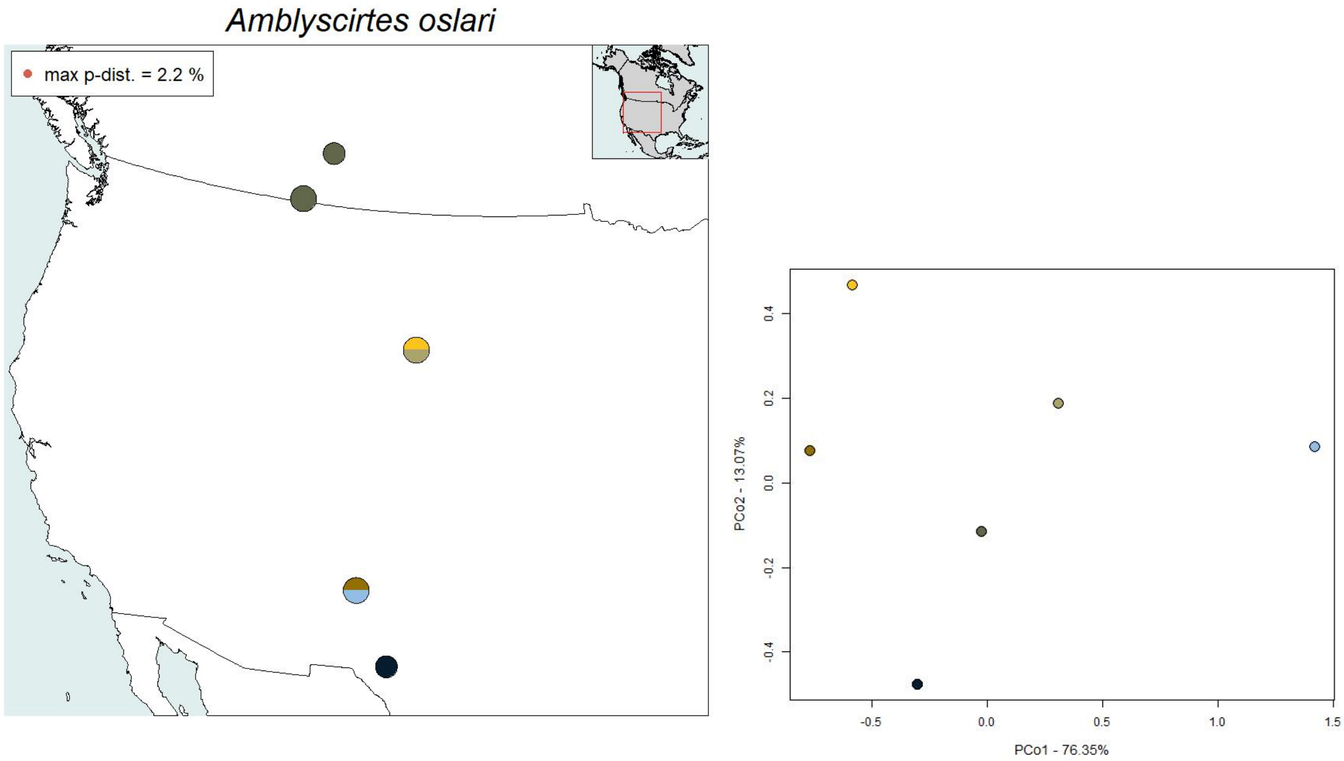

Figure 69 Map of *Amblyscirtes oslari* showing the localities of the sequenced specimens (left). Nearby localities are grouped in pies. Colours match the bidimensional colour space of the PCoA projection (right) of max p-dists among sequences (dots). Sequences= 8; Hap obs.= 6; Hap asympt.= NA; Hap % obs.= NA%; GST= NaN; DST= NaN; HD= NA; ND= NA; max p-dist= 2.2%.

Haplotype network analysis and bubble plot of *Amblyscirtes oslari* were not possible. Sequences > 599 bp = 4.

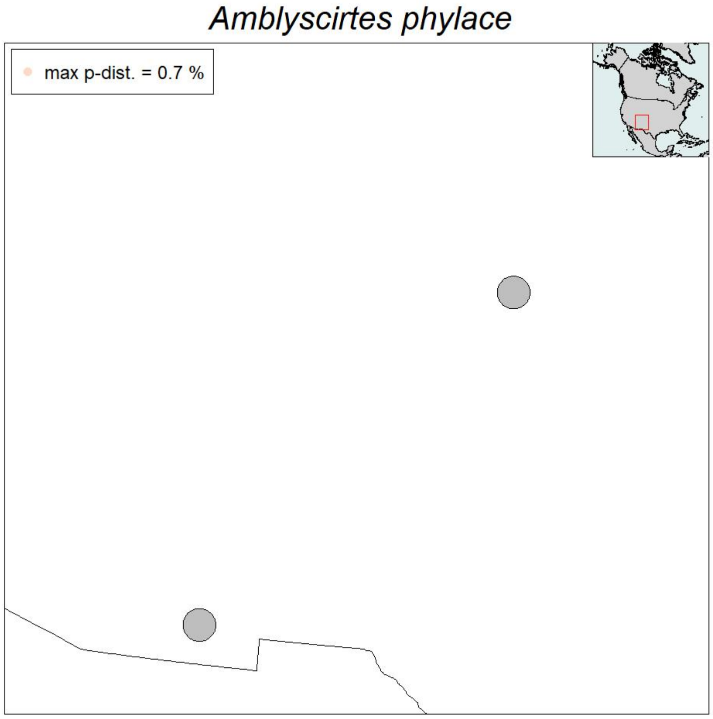

Figure 70: Map of *Amblyscirtes phylace* showing the localities of the sequenced specimens. Nearby localities are grouped in pies. Due to the presence of a single haplotype PCoA projection was not done and a single grey colour was plotted on the map. Sequences= 2; Hap obs.= 2; Hap asympt.= NA; Hap % obs.= NA%; GST= NaN; DST= NaN; HD= NA; ND= NA; max p-dist= 0.7%.

Haplotype network analysis and bubble plot of *Amblyscirtes phylace* were not possible. Sequences > 599 bp = 1.

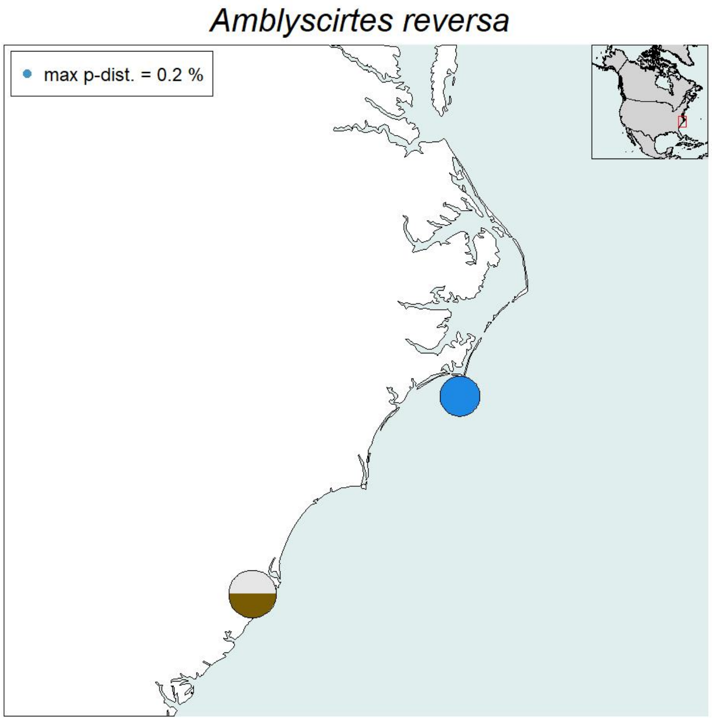

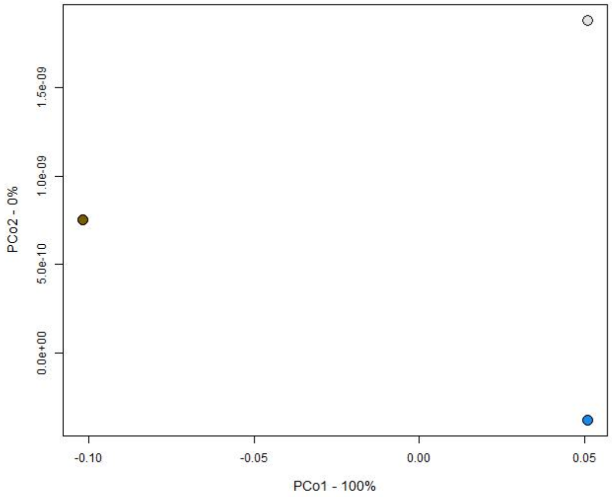

Figure 71 Map of *Amblyscirtes reversa* showing the localities of the sequenced specimens (left). Nearby localities are grouped in pies. Colours match the bidimensional colour space of the PCoA projection (right) of max p-dists among sequences (dots). Sequences= 3; Hap obs.= 2; Hap asympt.= NA; Hap % obs.= NA%; GST= NaN; DST= NaN; HD= NA; ND= NA; max p-dist= 0.2%.

Haplotype network analysis and bubble plot of *Amblyscirtes reversa* were not possible. Sequences > 599 bp = 3.

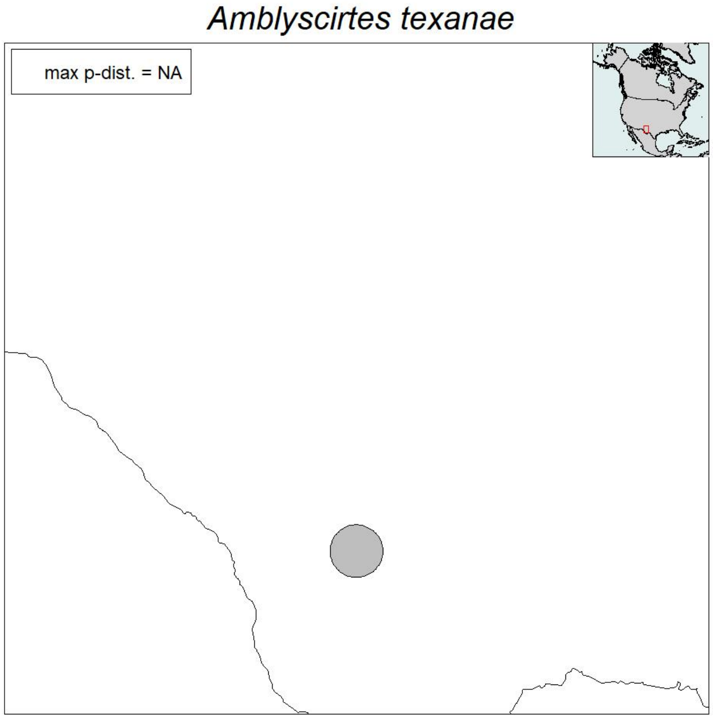

Figure 72: Map of *Amblyscirtes texanae* showing the localities of the sequenced specimens. Nearby localities are grouped in pies. Due to the presence of a single haplotype PCoA projection was not done and a single grey colour was plotted on the map. Sequences= 1; Hap obs.= NA; Hap asympt.= NA; Hap % obs.= NA; GST= NaN; DST= NaN; HD= NA; ND= NA; max p-dist= NA.

Haplotype network analysis and bubble plot of *Amblyscirtes texanae* were not possible. Sequences > 599 bp = 1.

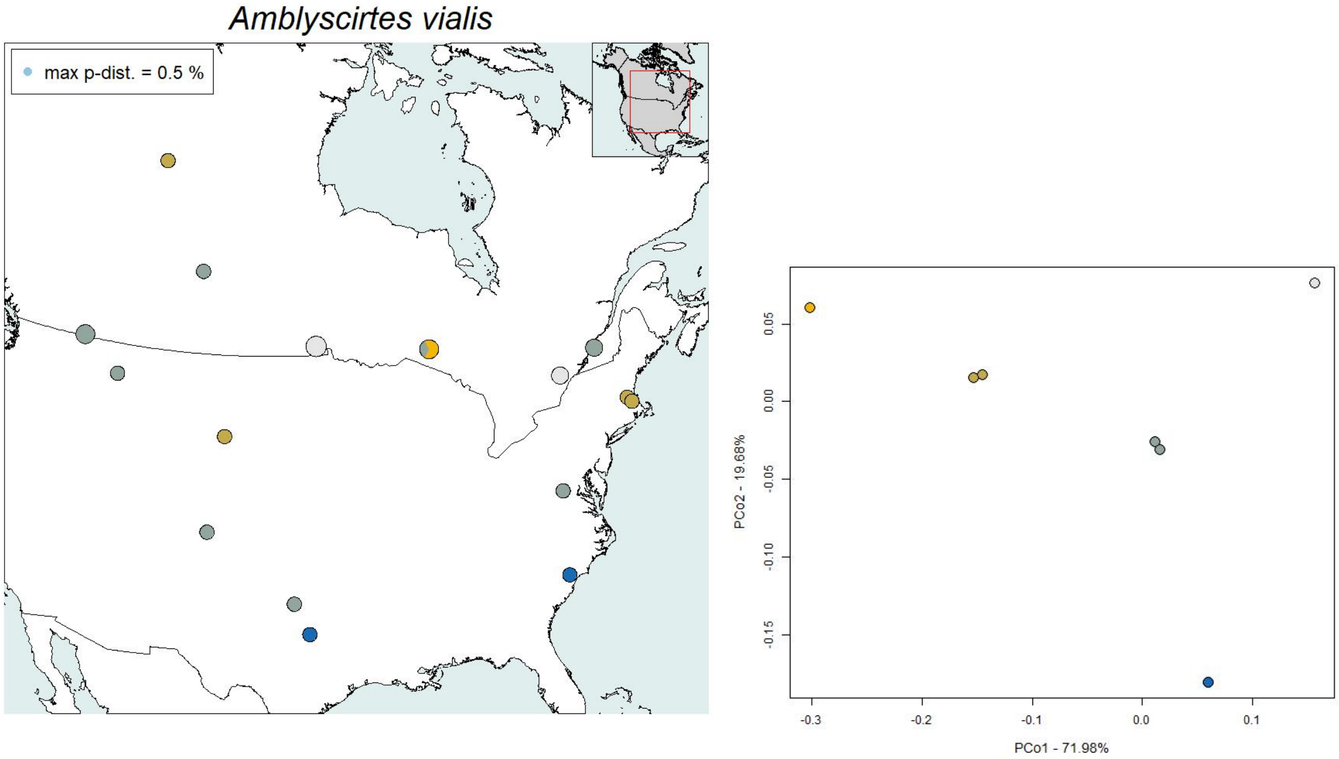

Figure 73 Map of *Amblyscirtes vialis* showing the localities of the sequenced specimens (left). Nearby localities are grouped in pies. Colours match the bidimensional colour space of the PCoA projection (right) of max p-dists among sequences (dots). Sequences= 25; Hap obs.= 6; Hap asympt.= 7.9; Hap % obs.= 75.8%; GST= 0.665; DST= 0.0013; HD= 0.743; ND= 0.0016; max p-dist= 0.5%.

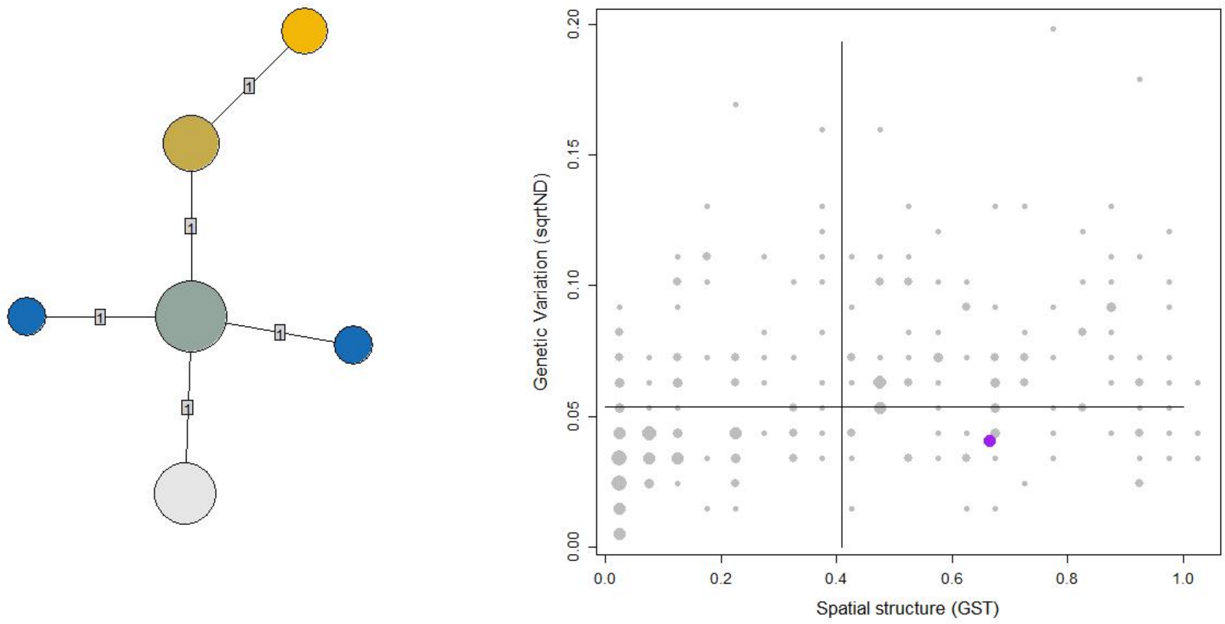

Figure 74: Haplotype network (left) of *Amblyscirtes vialis* sequences > 599 bp with colours matching the PCoA colour space (above). The bubble plot for mt-DNA polymorphism (square root transformed nucleotide diversity) and spatial structure (GST) among all species in the atlas and values for *Amblyscirtes vialis* (purple dot). The horizontal and vertical lines represent median values of nucleotide diversity and GST, respectively. Sequences > 599 bp= 25.

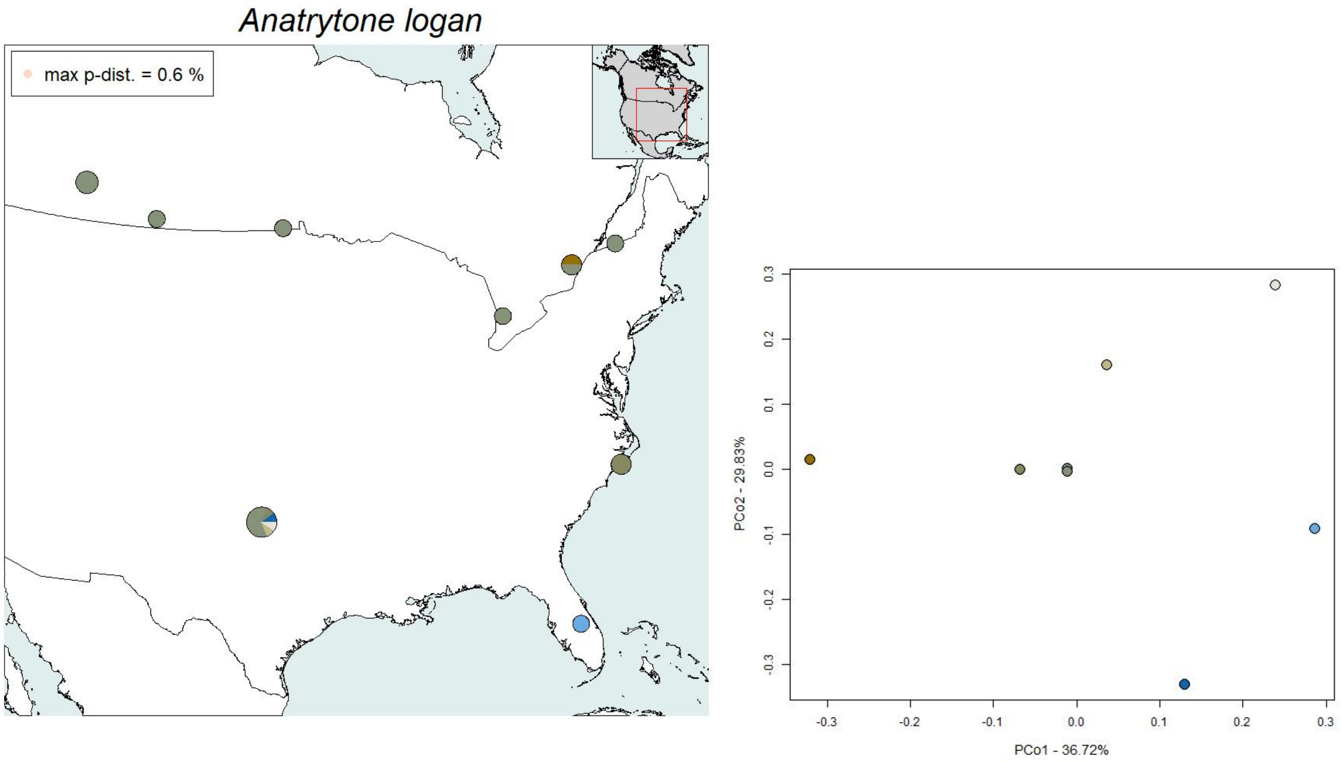

Figure 75 Map of *Anatrytone logan* showing the localities of the sequenced specimens (left). Nearby localities are grouped in pies. Colours match the bidimensional colour space of the PCoA projection (right) of max p-dists among sequences (dots). Sequences= 22; Hap obs.= 9; Hap asympt.= 35.7; Hap % obs.= 25.2%; GST= 0; DST= 0; HD= 0.606; ND= 0.0016; max p-dist= 0.6%.

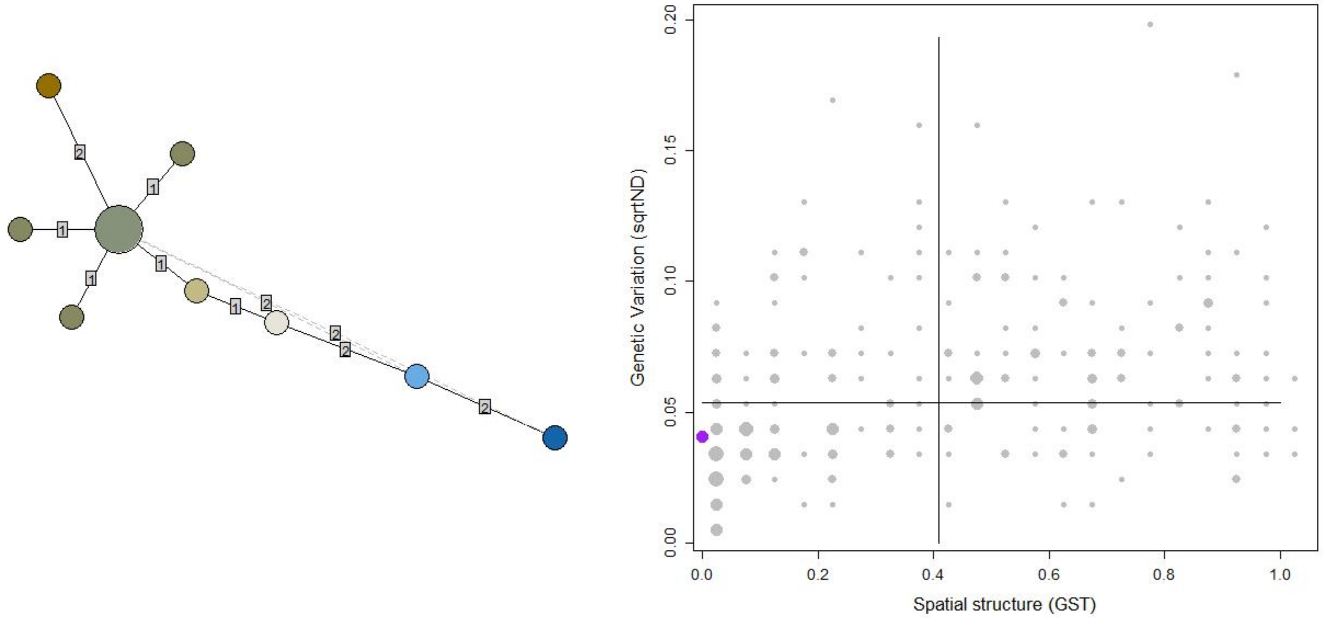

Figure 76: Haplotype network (left) of *Anatrytone logan* sequences > 599 bp with colours matching the PCoA colour space (above). The bubble plot for mt-DNA polymorphism (square root transformed nucleotide diversity) and spatial structure (GST) among all species in the atlas and values for *Anatrytone logan* (purple dot). The horizontal and vertical lines represent median values of nucleotide diversity and GST, respectively. Sequences > 599 bp= 22.

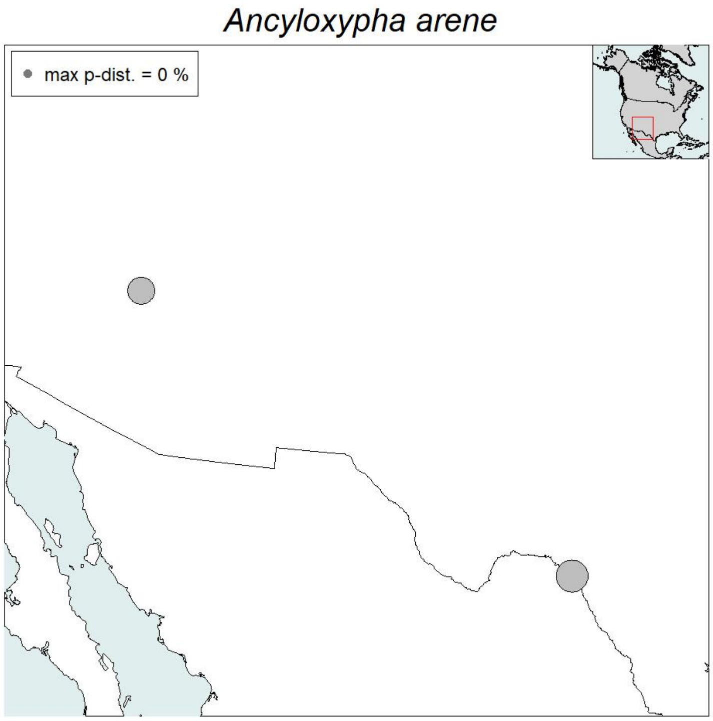

Figure 77: Map of *Ancyloxypha arene* showing the localities of the sequenced specimens. Nearby localities are grouped in pies. Due to the presence of a single haplotype PCoA projection was not done and a single grey colour was plotted on the map. Sequences= 3; Hap obs.= 2; Hap asympt.= NA; Hap % obs.= NA%; GST= NaN; DST= NaN; HD= NA; ND= NA; max p-dist= 0%.

Haplotype network analysis and bubble plot of *Ancyloxypha arene* were not possible. Sequences > 599 bp = 1.

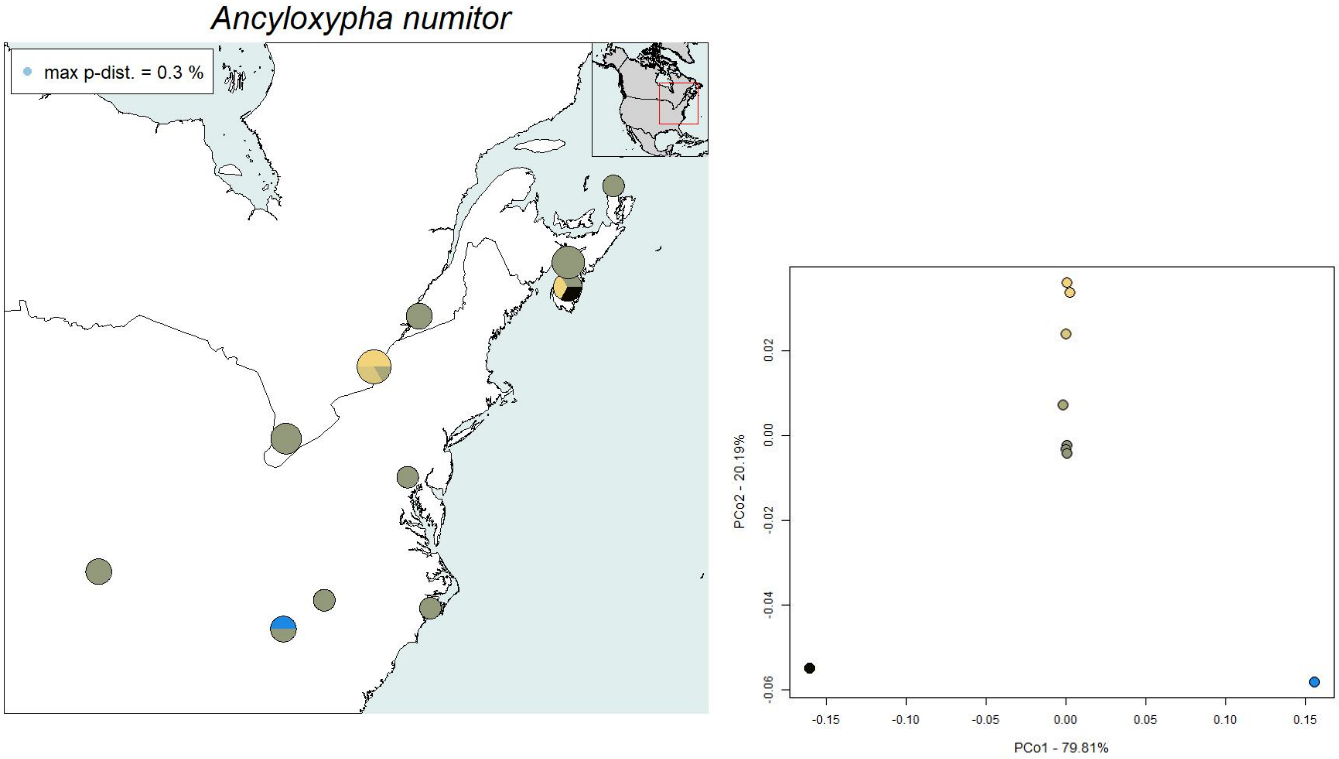

Figure 78 Map of *Ancyloxypha numitor* showing the localities of the sequenced specimens (left). Nearby localities are grouped in pies. Colours match the bidimensional colour space of the PCoA projection (right) of max p-dists among sequences (dots). Sequences= 28; Hap obs.= 3; Hap asympt.= 4; Hap % obs.= 75.7%; GST= 0.236; DST= 0.0001; HD= 0.14; ND= 0.0002; max p-dist= 0.3%.

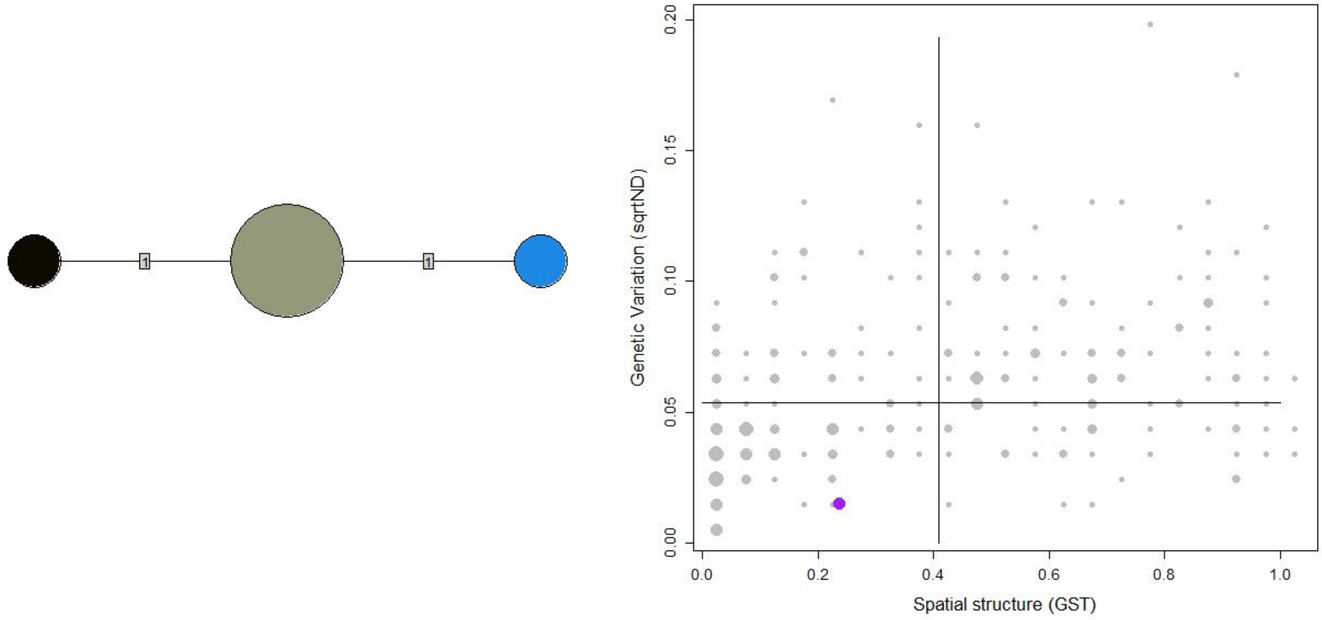

Figure 79: Haplotype network (left) of *Ancyloxypha numitor* sequences > 599 bp with colours matching the PCoA colour space (above). The bubble plot for mt-DNA polymorphism (square root transformed nucleotide diversity) and spatial structure (GST) among all species in the atlas and values for *Ancyloxypha numitor* (purple dot). The horizontal and vertical lines represent median values of nucleotide diversity and GST, respectively. Sequences > 599 bp= 22.

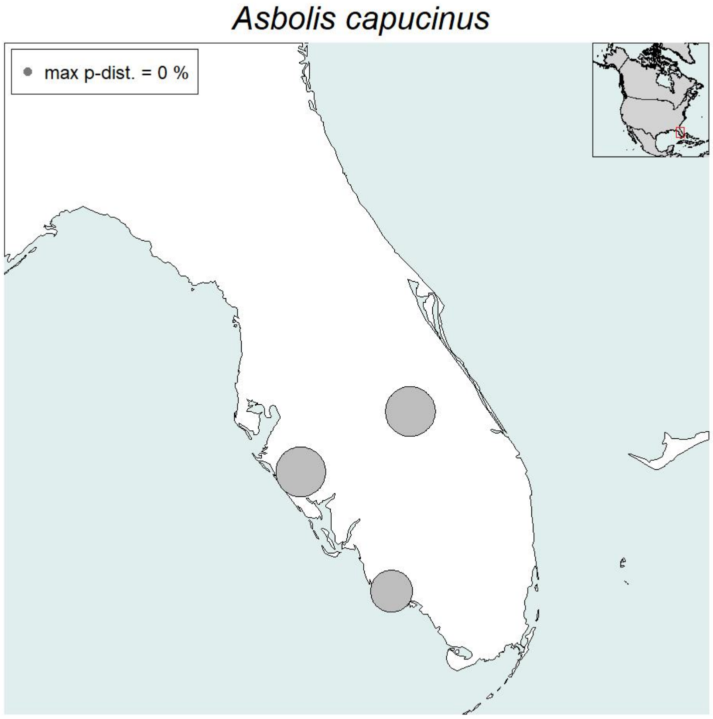

Figure 80: Map of *Asbolis capucinus* showing the localities of the sequenced specimens. Nearby localities are grouped in pies. Due to the presence of a single haplotype PCoA projection was not done and a single grey colour was plotted on the map. Sequences= 5; Hap obs.= 1; Hap asympt.= NA; Hap % obs.= NA%; GST= NaN; DST= NaN; HD= NA; ND= NA; max p-dist= 0%.

Haplotype network analysis and bubble plot of *Asbolis capucinus* were not possible. Sequences > 599 bp = 5.

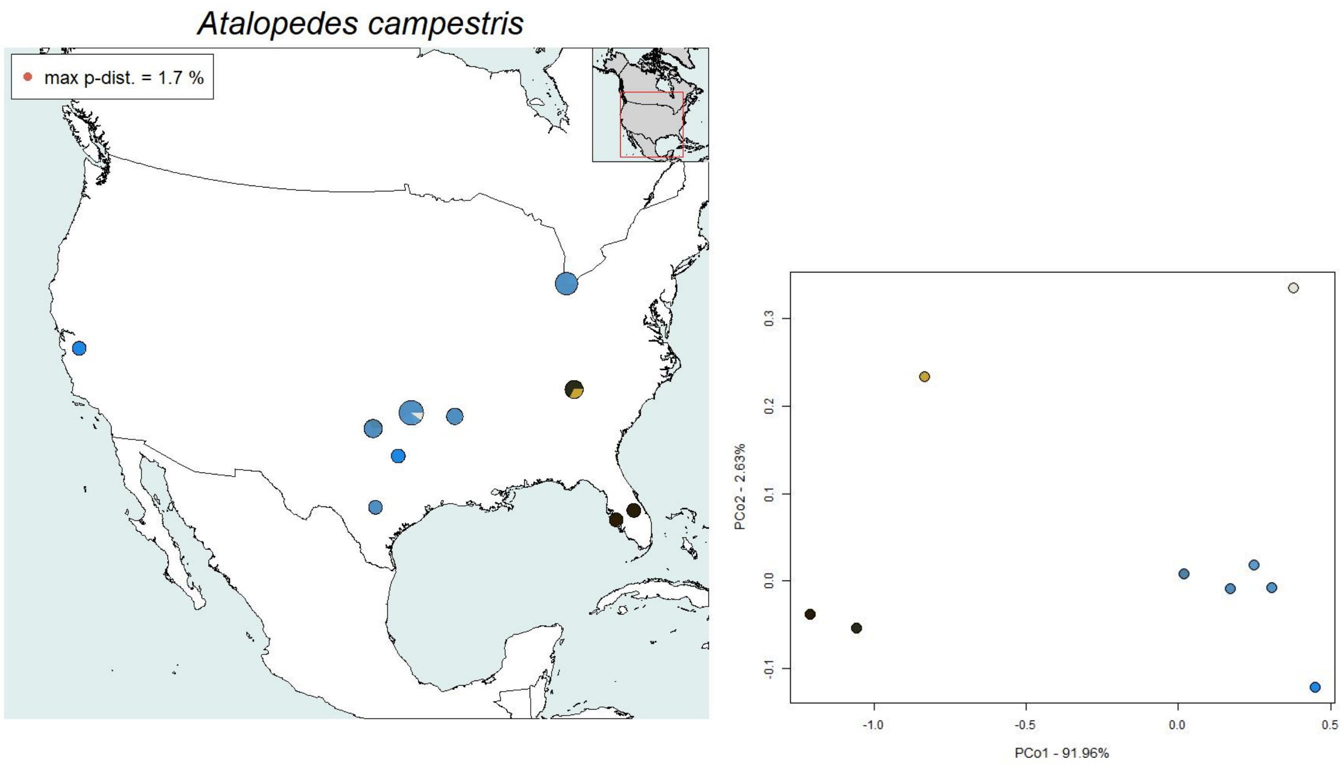

Figure 81 Map of *Atalopedes campestris* showing the localities of the sequenced specimens (left). Nearby localities are grouped in pies. Colours match the bidimensional colour space of the PCoA projection (right) of max p-dists among sequences (dots). Sequences= 30; Hap obs.= 11; Hap asympt.= 45.8; Hap % obs.= 24%; GST= 0.48; DST= 0.0016; HD= 0.641; ND= 0.0045; max p-dist= 1.7%.

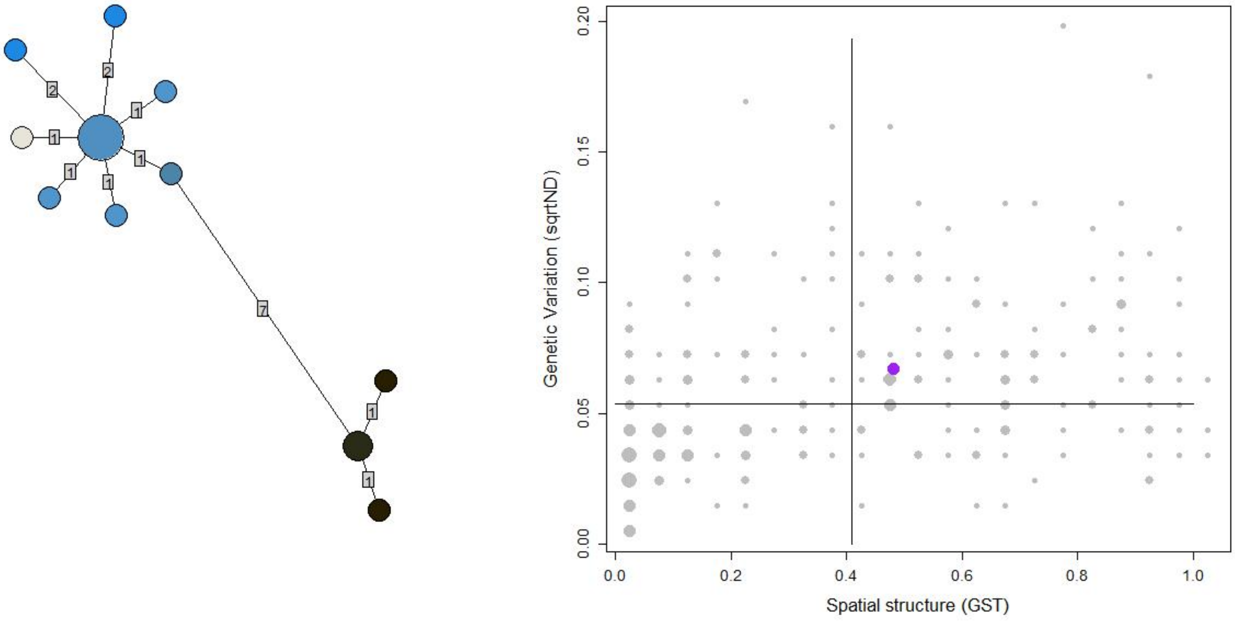

Figure 82: Haplotype network (left) of *Atalopedes campestris* sequences > 599 bp with colours matching the PCoA colour space (above). The bubble plot for mt-DNA polymorphism (square root transformed nucleotide diversity) and spatial structure (GST) among all species in the atlas and values for *Atalopedes campestris* (purple dot). The horizontal and vertical lines represent median values of nucleotide diversity and GST, respectively. Sequences > 599 bp= 30.

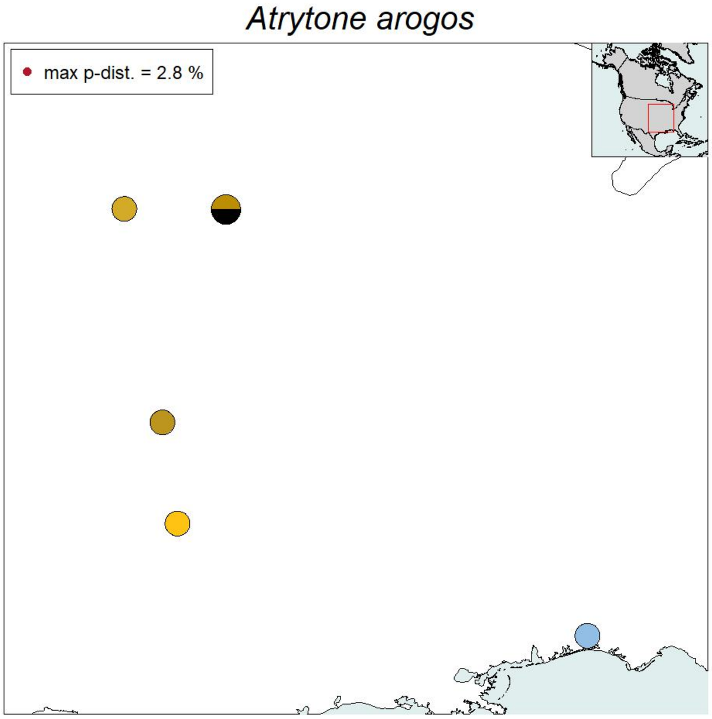

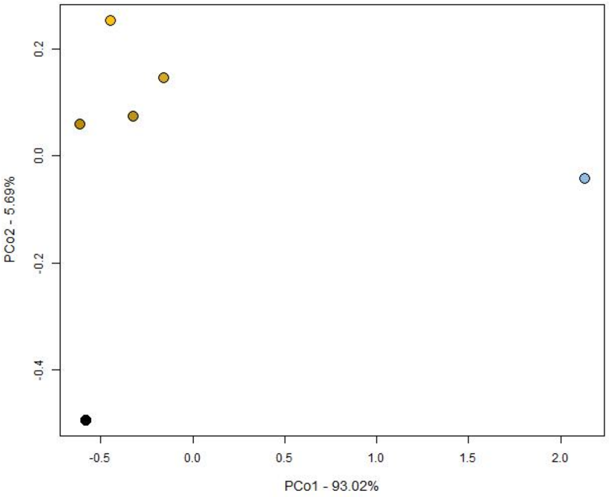

Figure 83 Map of *Atrytone arogos* showing the localities of the sequenced specimens (left). Nearby localities are grouped in pies. Colours match the bidimensional colour space of the PCoA projection (right) of max p-dists among sequences (dots). Sequences= 6; Hap obs.= 6; Hap asympt.= NA; Hap % obs.= NA%; GST= NaN; DST= NaN; HD= NA; ND= NA; max p-dist= 2.8%.

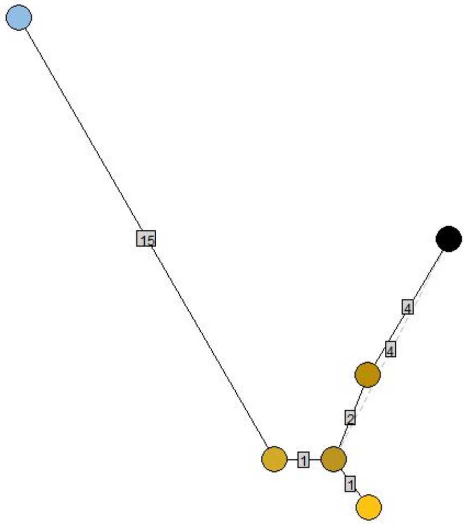

Figure 84: Haplotype network of *Atrytone arogos*. Sequences > 599 bp= 6.

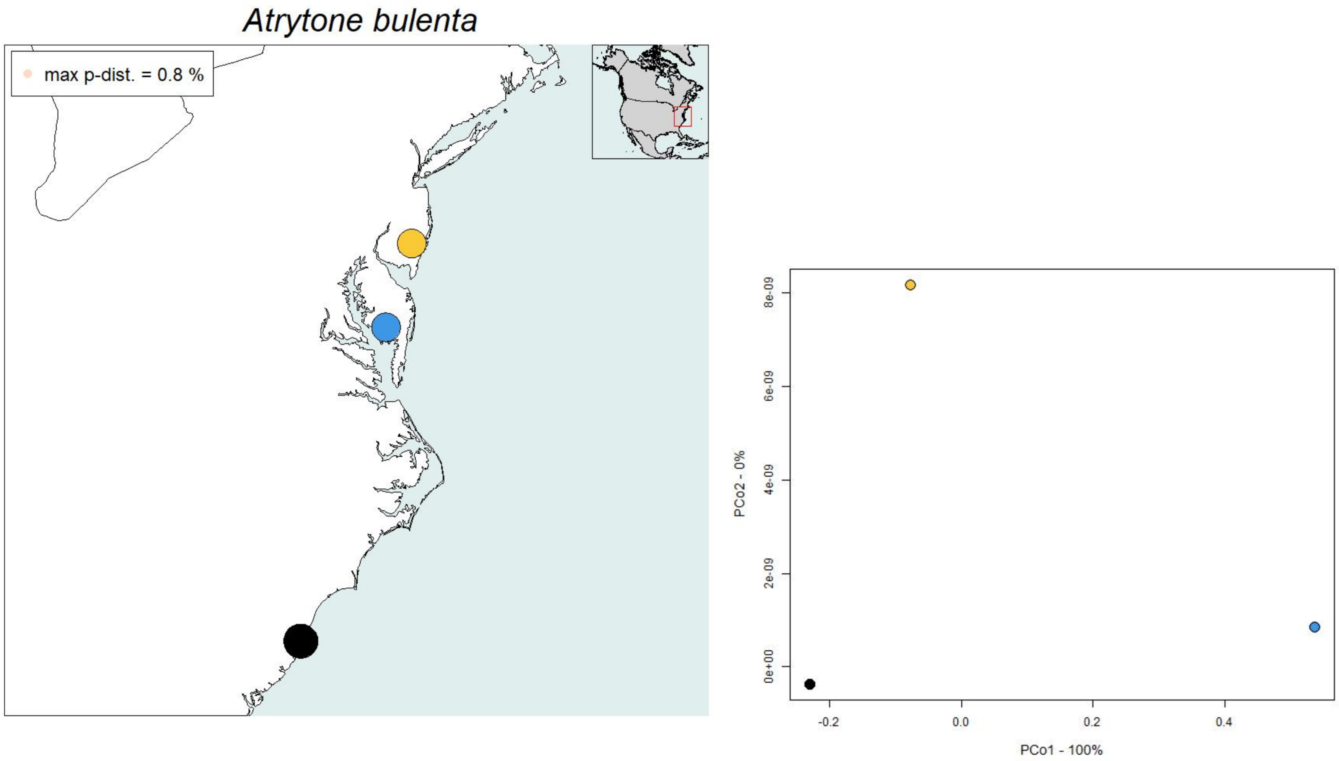

Figure 85 Map of *Atrytone bulenta* showing the localities of the sequenced specimens (left). Nearby localities are grouped in pies. Colours match the bidimensional colour space of the PCoA projection (right) of max p-dists among sequences (dots). Sequences= 4; Hap obs.= 3; Hap asympt.= NA; Hap % obs.= NA%; GST= NaN; DST= NaN; HD= NA; ND= NA; max p-dist= 0.8%.

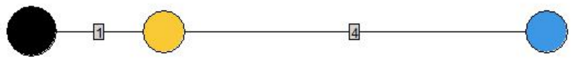

Figure 86: Haplotype network of *Atrytone bulenta*. Sequences > 599 bp= 4.

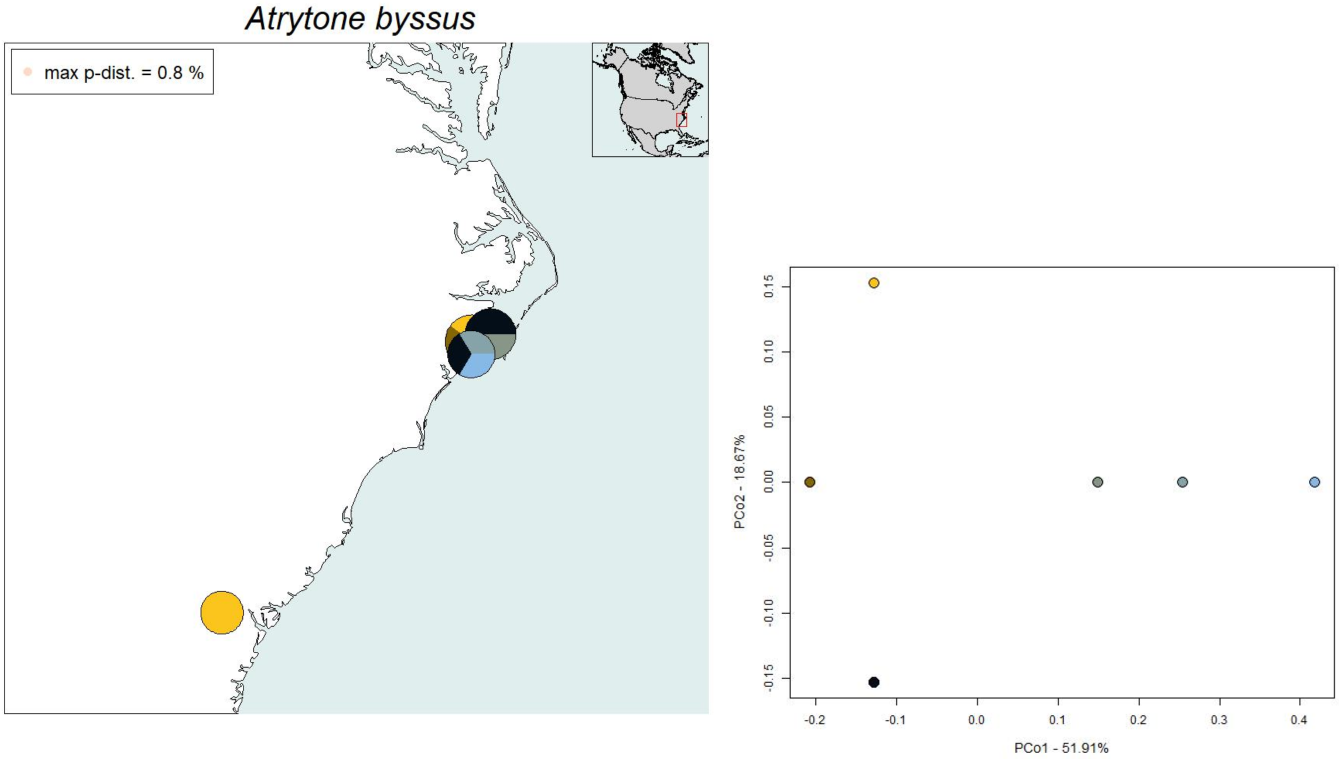

Figure 87 Map of *Atrytone byssus* showing the localities of the sequenced specimens (left). Nearby localities are grouped in pies. Colours match the bidimensional colour space of the PCoA projection (right) of max p-dists among sequences (dots). Sequences= 14; Hap obs.= 6; Hap asympt.= 6.9; Hap % obs.= 86.6%; GST= 0.098; DST= 0.0003; HD= 0.846; ND= 0.0034; max p-dist= 0.8%.

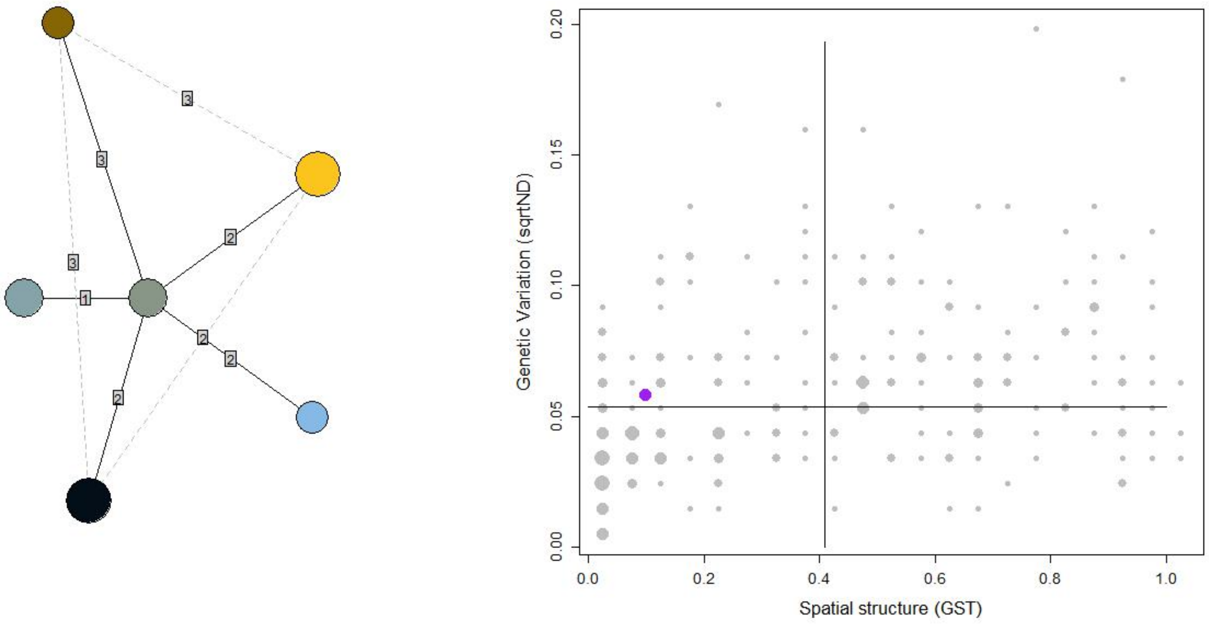

Figure 88: Haplotype network (left) of *Atrytone byssus* sequences > 599 bp with colours matching the PCoA colour space (above). The bubble plot for mt-DNA polymorphism (square root transformed nucleotide diversity) and spatial structure (GST) among all species in the atlas and values for *Atrytone byssus* (purple dot). The horizontal and vertical lines represent median values of nucleotide diversity and GST, respectively. Sequences > 599 bp= 14.

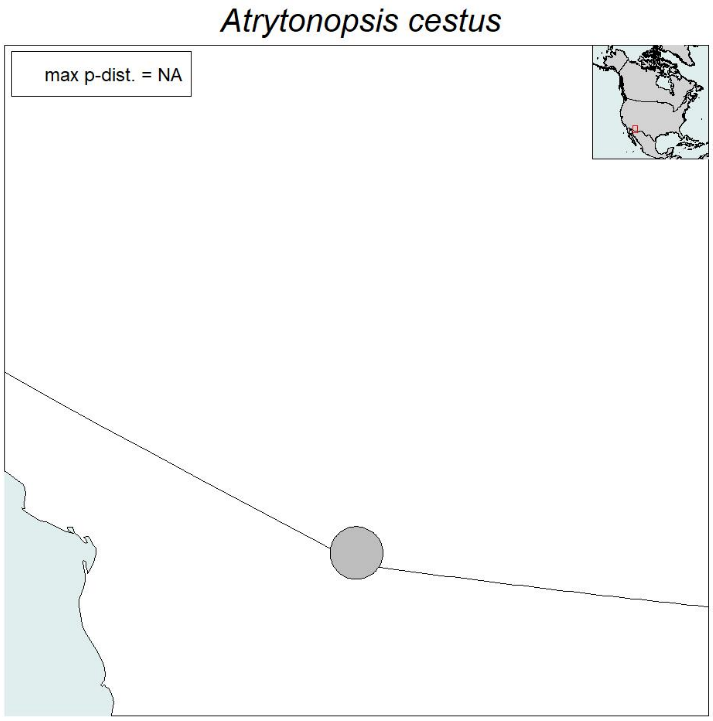

Figure 89: Map of *Atrytonopsis cestus* showing the localities of the sequenced specimens. Nearby localities are grouped in pies. Due to the presence of a single haplotype PCoA projection was not done and a single grey colour was plotted on the map. Sequences= 1; Hap obs.= NA; Hap asympt.= NA; Hap % obs.= NA; GST= NaN; DST= NaN; HD= NA; ND= NA; max p-dist= NA.

Haplotype network analysis and bubble plot of *Atrytonopsis cestus* were not possible. Sequences > 599 bp = 1.

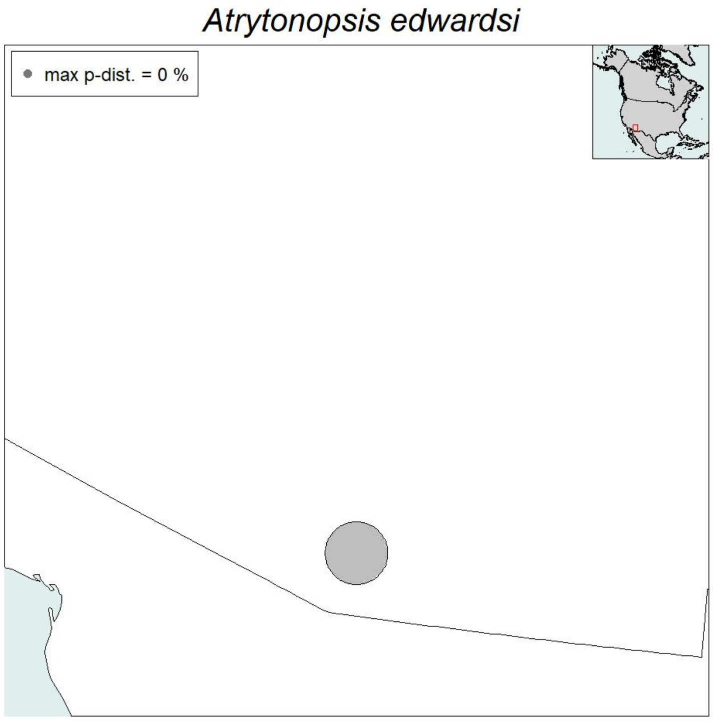

Figure 90: Map of *Atrytonopsis edwardsi* showing the localities of the sequenced specimens. Nearby localities are grouped in pies. Due to the presence of a single haplotype PCoA projection was not done and a single grey colour was plotted on the map. Sequences= 2; Hap obs.= 1; Hap asympt.= NA; Hap % obs.= NA%; GST= NaN; DST= NaN; HD= NA; ND= NA; max p-dist= 0%.

Haplotype network analysis and bubble plot of *Atrytonopsis edwardsi* were not possible. Sequences > 599 bp = 2.

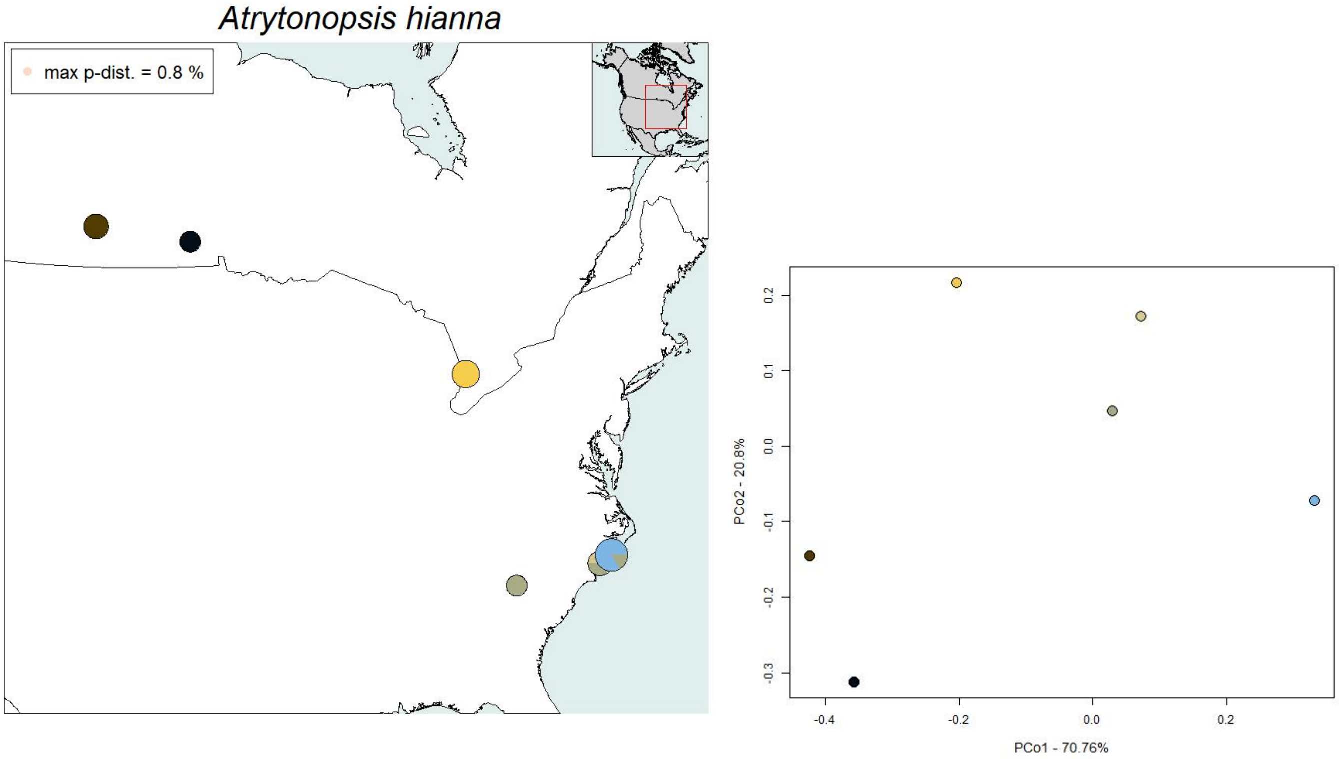

Figure 91 Map of *Atrytonopsis hianna* showing the localities of the sequenced specimens (left). Nearby localities are grouped in pies. Colours match the bidimensional colour space of the PCoA projection (right) of max p-dists among sequences (dots). Sequences= 15; Hap obs.= 6; Hap asympt.= 7.9; Hap % obs.= 76.3%; GST= 0.444; DST= 0.0015; HD= 0.838; ND= 0.0041; max p-dist= 0.8%.

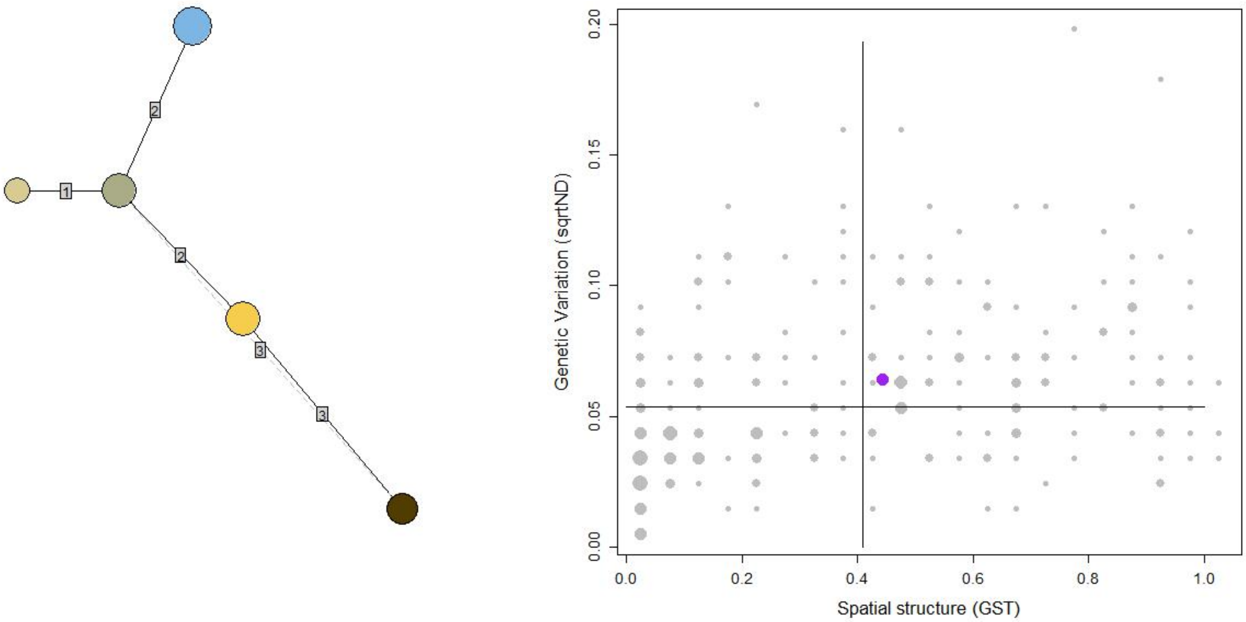

Figure 92: Haplotype network (left) of *Atrytonopsis hianna* sequences > 599 bp with colours matching the PCoA colour space (above). The bubble plot for mt-DNA polymorphism (square root transformed nucleotide diversity) and spatial structure (GST) among all species in the atlas and values for *Atrytonopsis hianna* (purple dot). The horizontal and vertical lines represent median values of nucleotide diversity and GST, respectively. Sequences > 599 bp= 14.

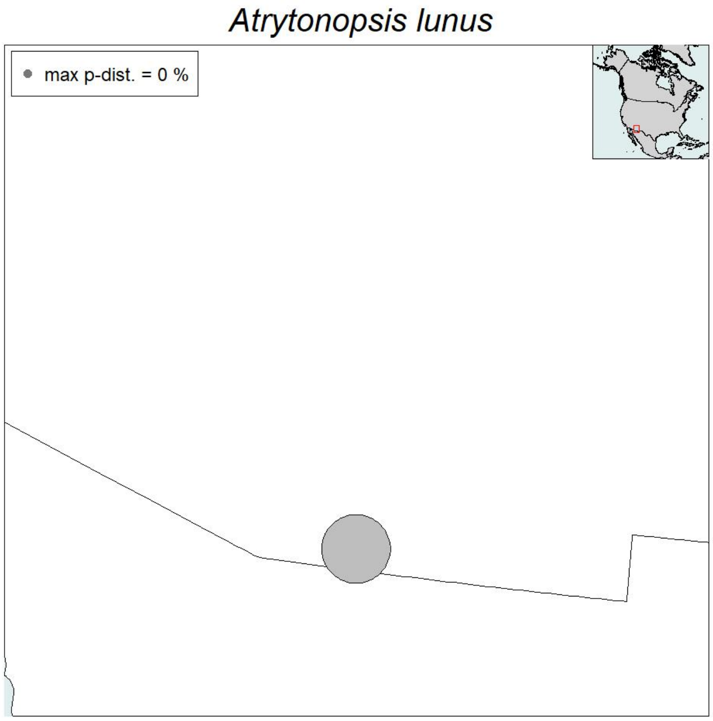

Figure 93: Map of *Atrytonopsis lunus* showing the localities of the sequenced specimens. Nearby localities are grouped in pies. Due to the presence of a single haplotype PCoA projection was not done and a single grey colour was plotted on the map. Sequences= 3; Hap obs.= 1; Hap asympt.= NA; Hap % obs.= NA%; GST= NaN; DST= NaN; HD= NA; ND= NA; max p-dist= 0%.

Haplotype network analysis and bubble plot of *Atrytonopsis lunus* were not possible. Sequences > 599 bp = 3.

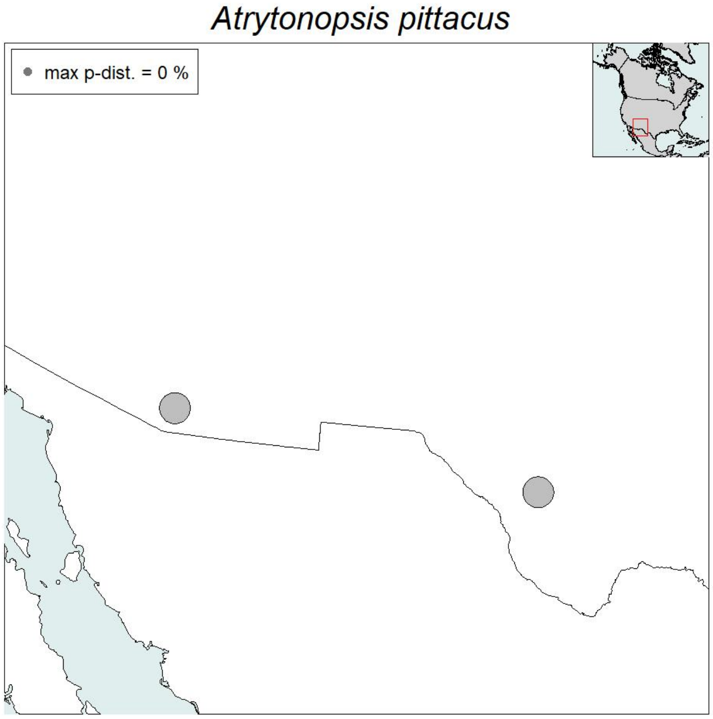

Figure 94: Map of *Atrytonopsis pittacus* showing the localities of the sequenced specimens. Nearby localities are grouped in pies. Due to the presence of a single haplotype PCoA projection was not done and a single grey colour was plotted on the map. Sequences= 2; Hap obs.= 1; Hap asympt.= NA; Hap % obs.= NA%; GST= NaN; DST= NaN; HD= NA; ND= NA; max p-dist= 0%.

Haplotype network analysis and bubble plot of *Atrytonopsis pittacus* were not possible. Sequences > 599 bp = 2.

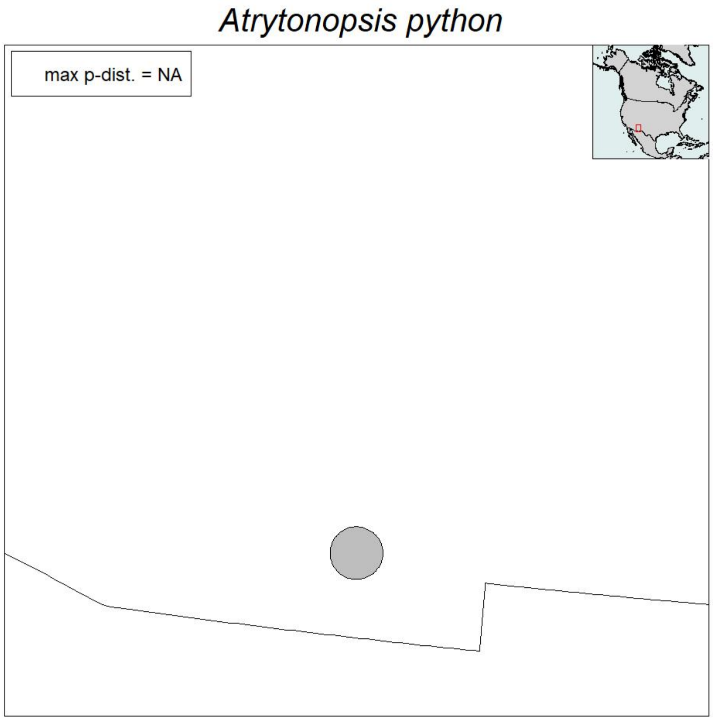

Figure 95: Map of *Atrytonopsis python* showing the localities of the sequenced specimens. Nearby localities are grouped in pies. Due to the presence of a single haplotype PCoA projection was not done and a single grey colour was plotted on the map. Sequences= 1; Hap obs.= NA; Hap asympt.= NA; Hap % obs.= NA; GST= NaN; DST= NaN; HD= NA; ND= NA; max p-dist= NA.

Haplotype network analysis and bubble plot of *Atrytonopsis python* were not possible. Sequences > 599 bp = 1.

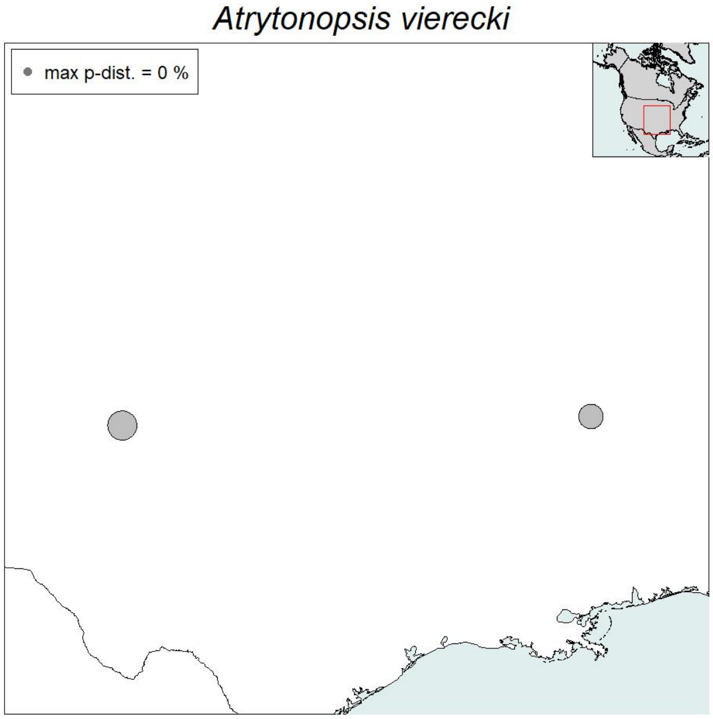

Figure 96: Map of *Atrytonopsis vierecki* showing the localities of the sequenced specimens. Nearby localities are grouped in pies. Due to the presence of a single haplotype PCoA projection was not done and a single grey colour was plotted on the map. Sequences= 3; Hap obs.= 1; Hap asympt.= NA; Hap % obs.= NA%; GST= NaN; DST= NaN; HD= NA; ND= NA; max p-dist= 0%.

Haplotype network analysis and bubble plot of *Atrytonopsis vierecki* were not possible. Sequences > 599 bp = 3.

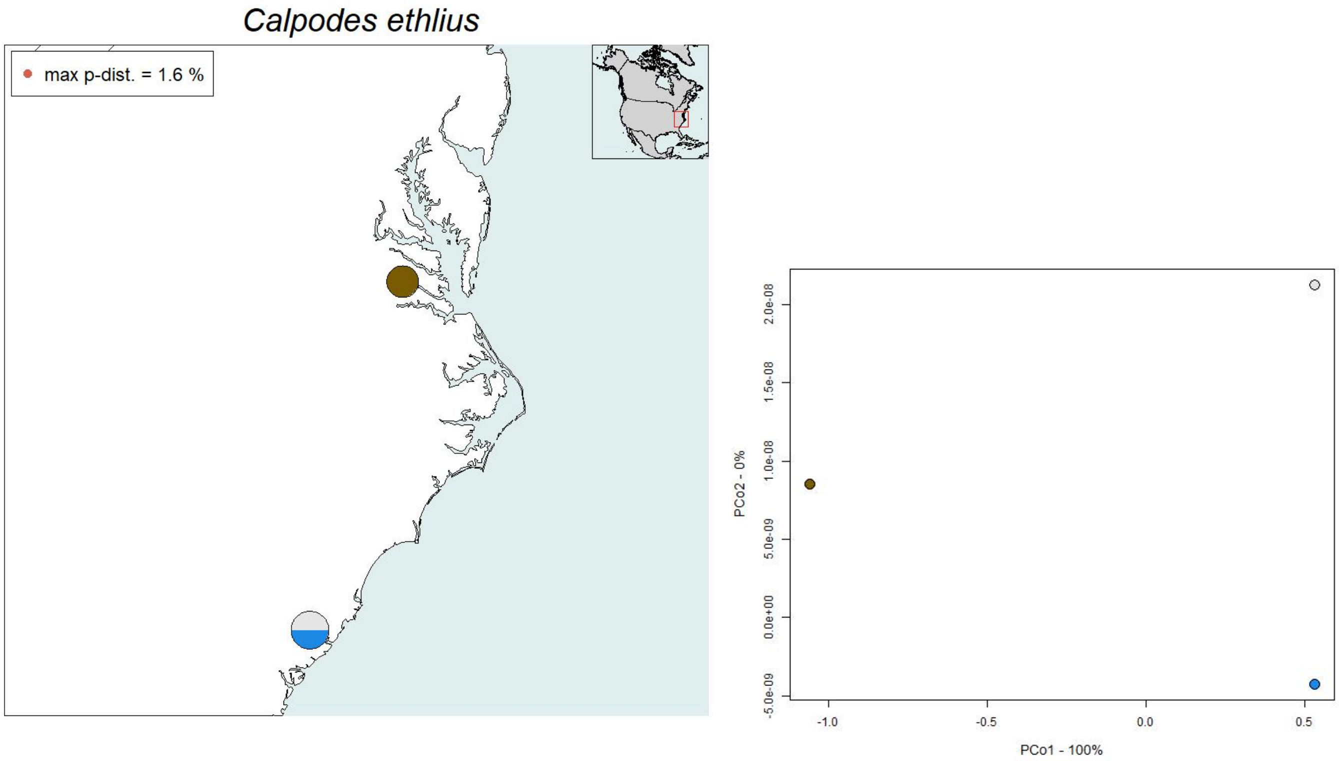

Figure 97 Map of *Calpodes ethlius* showing the localities of the sequenced specimens (left). Nearby localities are grouped in pies. Colours match the bidimensional colour space of the PCoA projection (right) of max p-dists among sequences (dots). Sequences= 3; Hap obs.= 2; Hap asympt.= NA; Hap % obs.= NA%; GST= NaN; DST= NaN; HD= NA; ND= NA; max p-dist= 1.6%.

Haplotype network analysis and bubble plot of *Calpodes ethlius* were not possible. Sequences > 599 bp = 1.

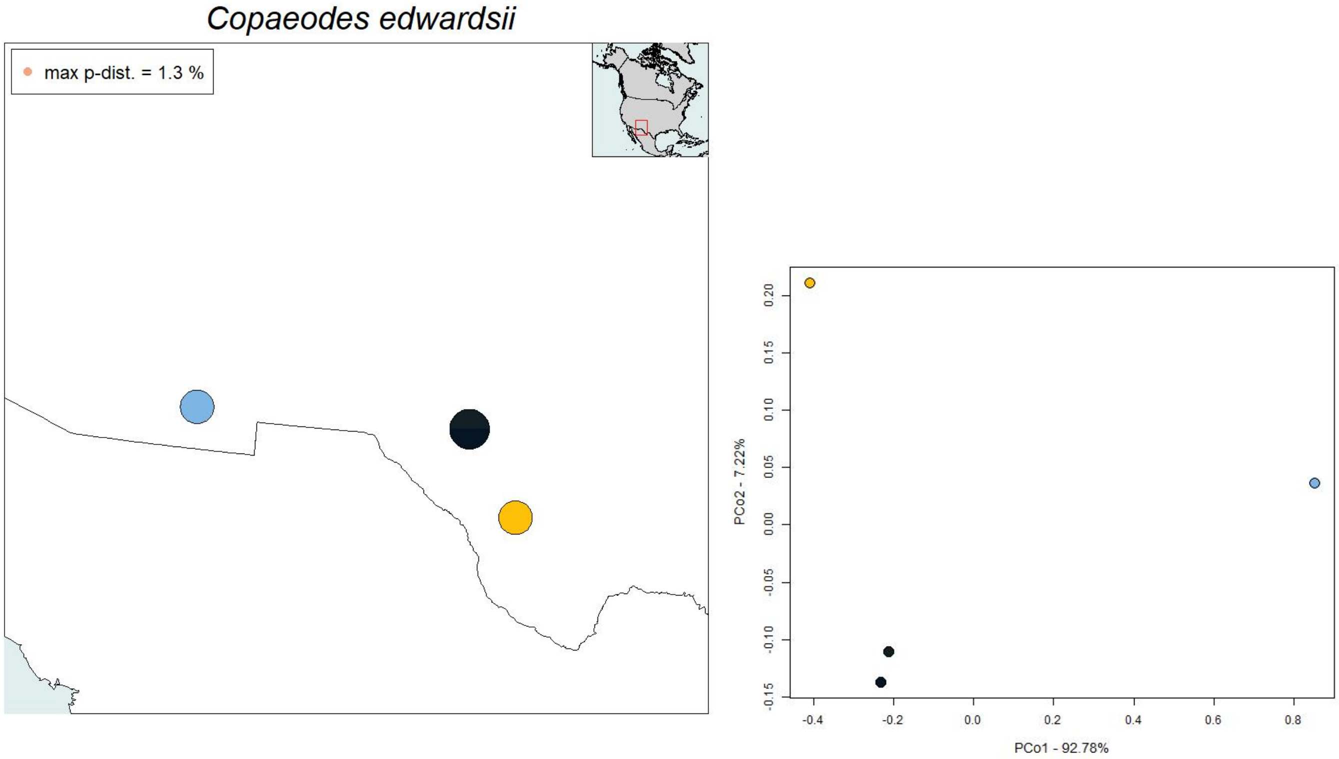

Figure 98 Map of *Copaeodes edwardsii* showing the localities of the sequenced specimens (left). Nearby localities are grouped in pies. Colours match the bidimensional colour space of the PCoA projection (right) of max p-dists among sequences (dots). Sequences= 4; Hap obs.= 3; Hap asympt.= NA; Hap % obs.= NA%; GST= NaN; DST= NaN; HD= NA; ND= NA; max p-dist= 1.3%.

Haplotype network analysis and bubble plot of *Copaeodes edwardsii* were not possible. Sequences > 599 bp = 2.

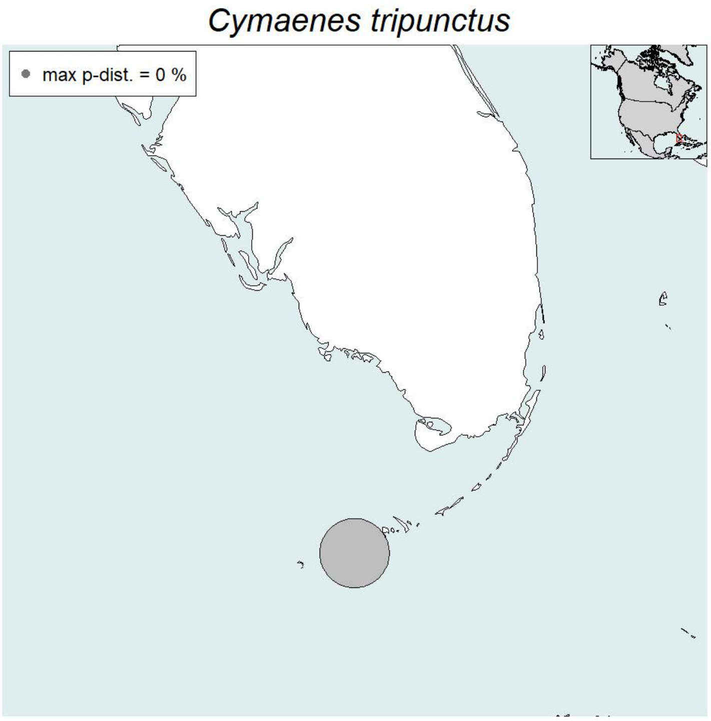

Figure 99: Map of *Cymaenes tripunctus* showing the localities of the sequenced specimens. Nearby localities are grouped in pies. Due to the presence of a single haplotype PCoA projection was not done and a single grey colour was plotted on the map. Sequences= 3; Hap obs.= 1; Hap asympt.= NA; Hap % obs.= NA%; GST= NaN; DST= NaN; HD= NA; ND= NA; max p-dist= 0%.

Haplotype network analysis and bubble plot of *Cymaenes tripunctus* were not possible. Sequences > 599 bp = 3.

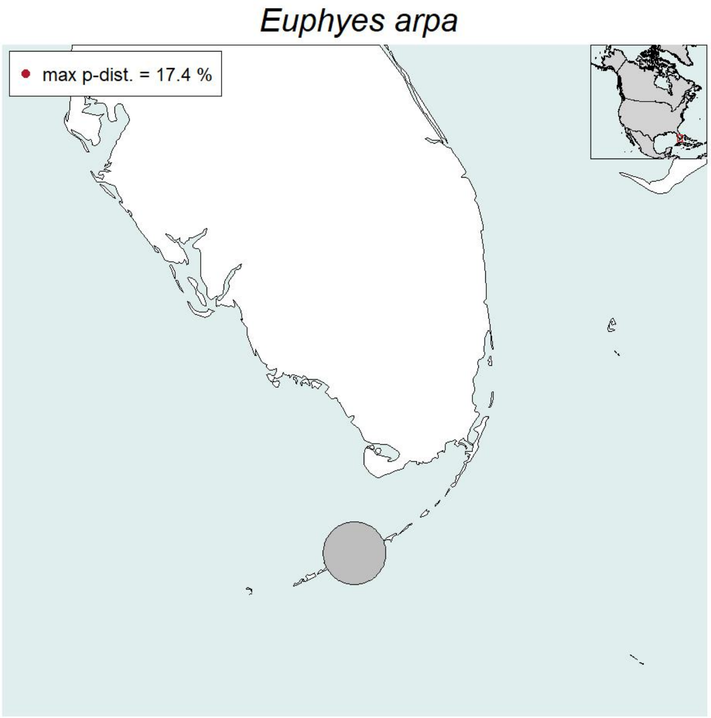

Figure 100: Map of *Euphyes arpa* showing the localities of the sequenced specimens. Nearby localities are grouped in pies. Due to the presence of a single haplotype PCoA projection was not done and a single grey colour was plotted on the map. Sequences= 2; Hap obs.= 2; Hap asympt.= NA; Hap % obs.= NA%; GST= NaN; DST= NaN; HD= NA; ND= NA; max p-dist= 17.4%.

Haplotype network analysis and bubble plot of *Euphyes arpa* were not possible. Sequences > 599 bp = 1.

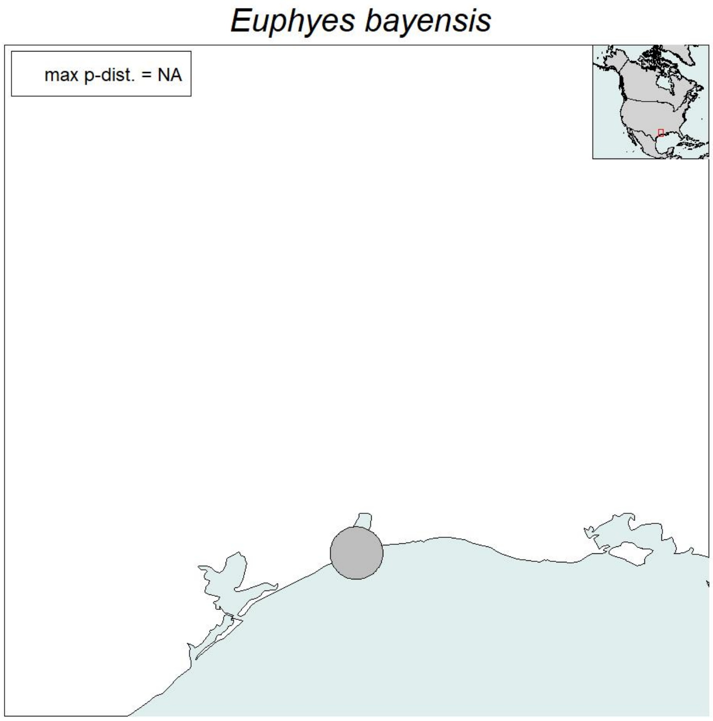

Figure 101: Map of *Euphyes bayensis* showing the localities of the sequenced specimens. Nearby localities are grouped in pies. Due to the presence of a single haplotype PCoA projection was not done and a single grey colour was plotted on the map. Sequences= 1; Hap obs.= NA; Hap asympt.= NA; Hap % obs.= NA; GST= NaN; DST= NaN; HD= NA; ND= NA; max p-dist= NA.

Haplotype network analysis and bubble plot of *Euphyes bayensis* were not possible. Sequences > 599 bp = 1.

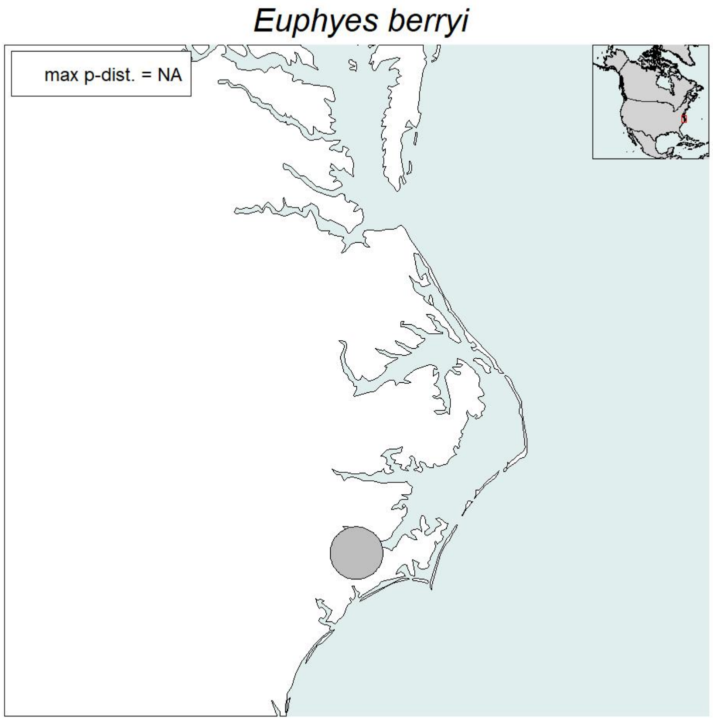

Figure 102: Map of *Euphyes berryi* showing the localities of the sequenced specimens. Nearby localities are grouped in pies. Due to the presence of a single haplotype PCoA projection was not done and a single grey colour was plotted on the map. Sequences= 1; Hap obs.= NA; Hap asympt.= NA; Hap % obs.= NA; GST= NaN; DST= NaN; HD= NA; ND= NA; max p-dist= NA.

Haplotype network analysis and bubble plot of *Euphyes berryi* were not possible. Sequences > 599 bp = 1.

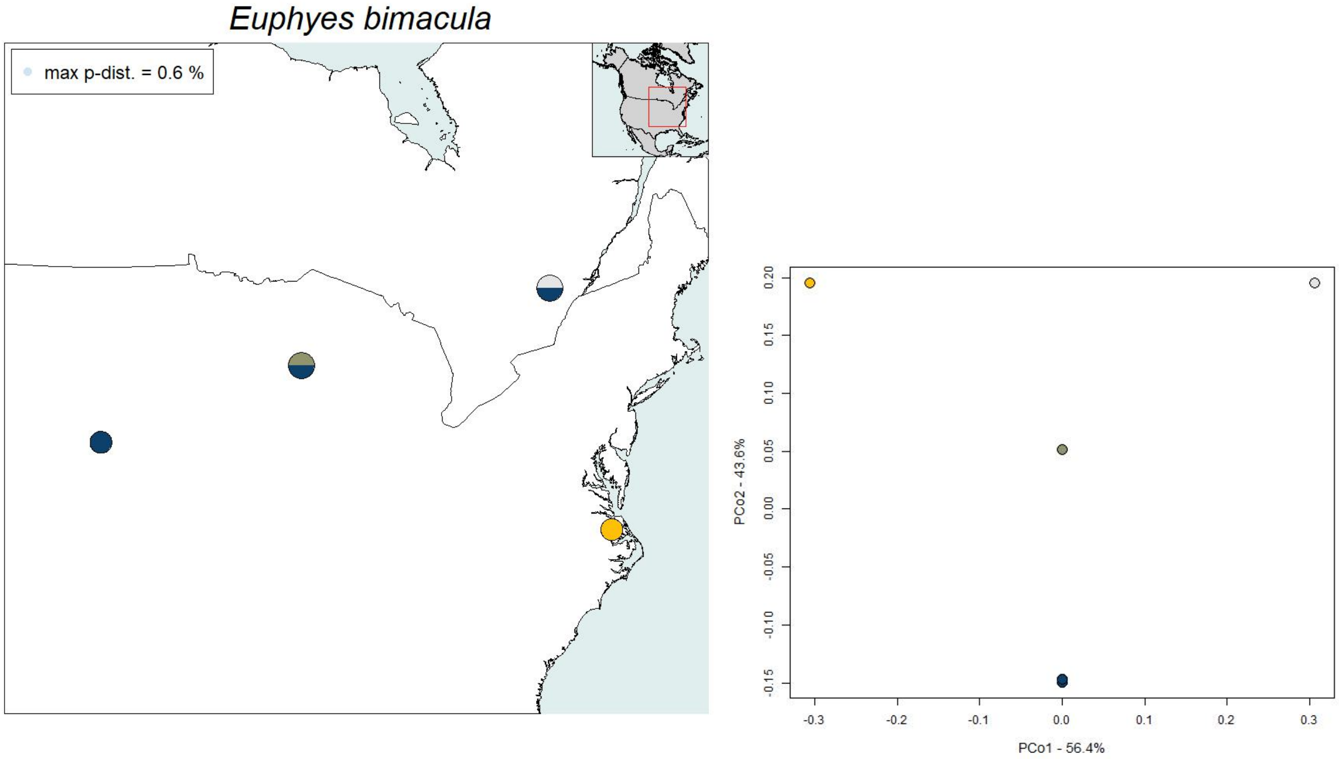

Figure 103 Map of *Euphyes bimacula* showing the localities of the sequenced specimens (left). Nearby localities are grouped in pies. Colours match the bidimensional colour space of the PCoA projection (right) of max p-dists among sequences (dots). Sequences= 6; Hap obs.= 4; Hap asympt.= NA; Hap % obs.= NA%; GST= NaN; DST= NaN; HD= NA; ND= NA; max p-dist= 0.6%.

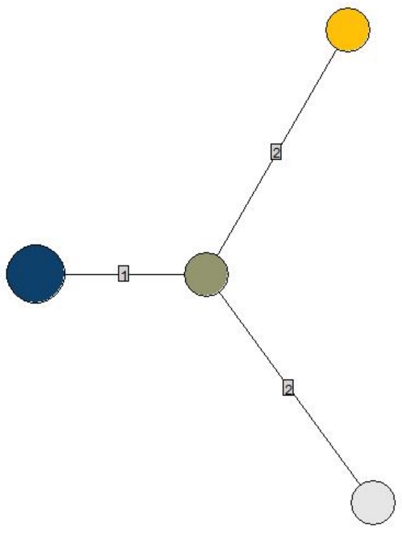

Figure 104: Haplotype network of *Euphyes bimacula*. Sequences > 599 bp= 6.

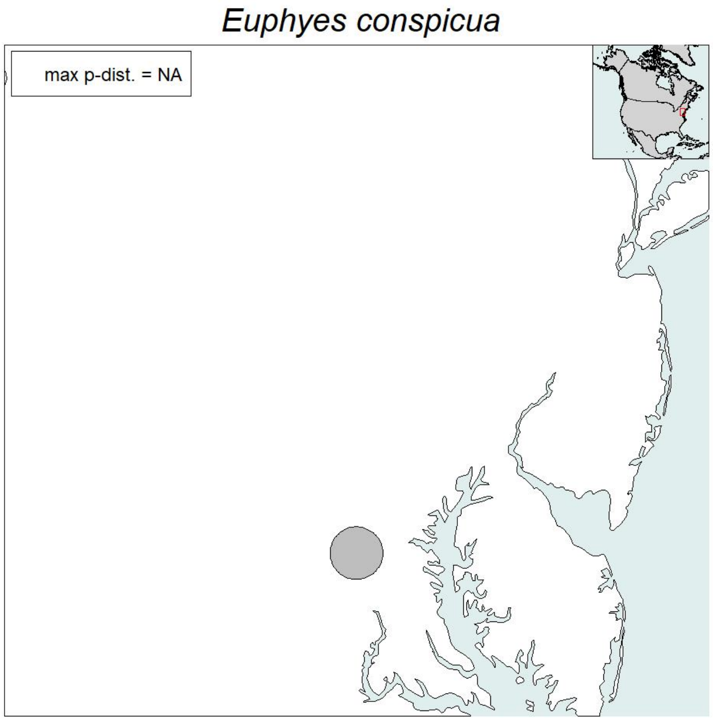

Figure 105: Map of *Euphyes conspicua* showing the localities of the sequenced specimens. Nearby localities are grouped in pies. Due to the presence of a single haplotype PCoA projection was not done and a single grey colour was plotted on the map. Sequences= 1; Hap obs.= NA; Hap asympt.= NA; Hap % obs.= NA; GST= NaN; DST= NaN; HD= NA; ND= NA; max p-dist= NA.

Haplotype network analysis and bubble plot of *Euphyes conspicua* were not possible. Sequences > 599 bp = 1.

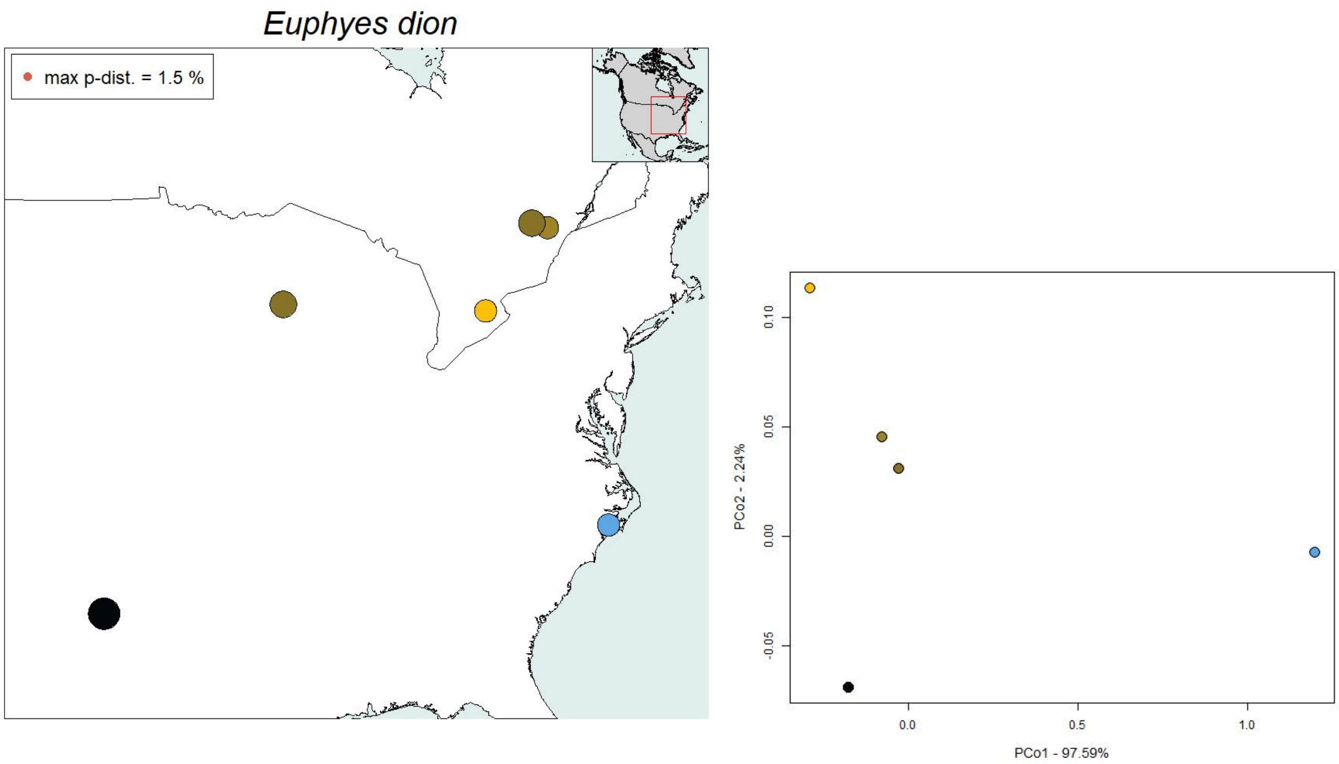

Figure 106 Map of *Euphyes dion* showing the localities of the sequenced specimens (left). Nearby localities are grouped in pies. Colours match the bidimensional colour space of the PCoA projection (right) of max p-dists among sequences (dots). Sequences= 11; Hap obs.= 4; Hap asympt.= 4.9; Hap % obs.= 81.5%; GST= NaN; DST= NaN; HD= 0.709; ND= 0.0033; max p-dist= 1.5%.

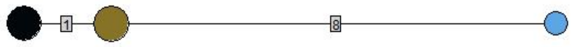

Figure 107: Haplotype network of *Euphyes dion*. Sequences > 599 bp= 10.

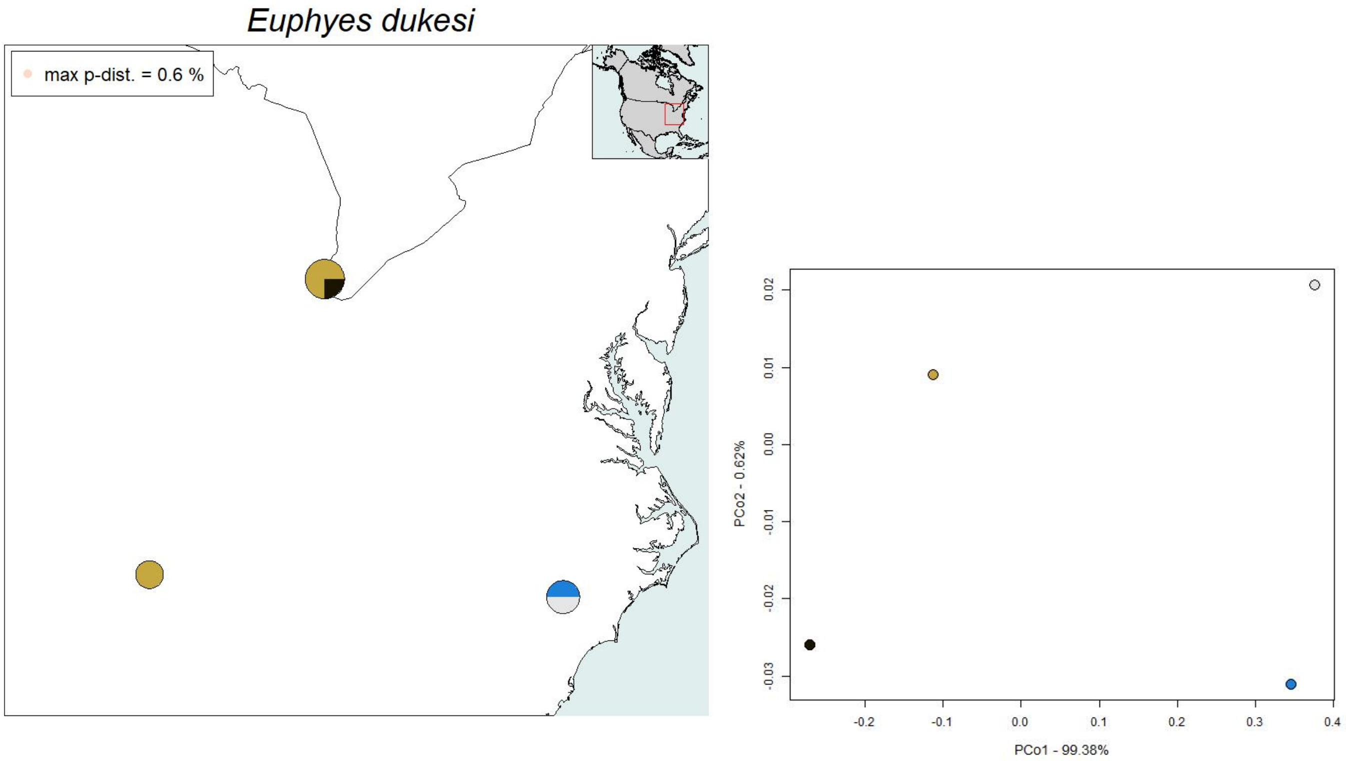

Figure 108 Map of *Euphyes dukesi* showing the localities of the sequenced specimens (left). Nearby localities are grouped in pies. Colours match the bidimensional colour space of the PCoA projection (right) of max p-dists among sequences (dots). Sequences= 7; Hap obs.= 3; Hap asympt.= NA; Hap % obs.= NA%; GST= NaN; DST= NaN; HD= NA; ND= NA; max p-dist= 0.6%.

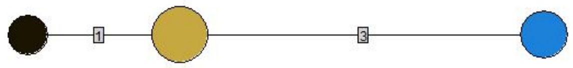

Figure 109: Haplotype network of *Euphyes dukesi*. Sequences > 599 bp= 7.

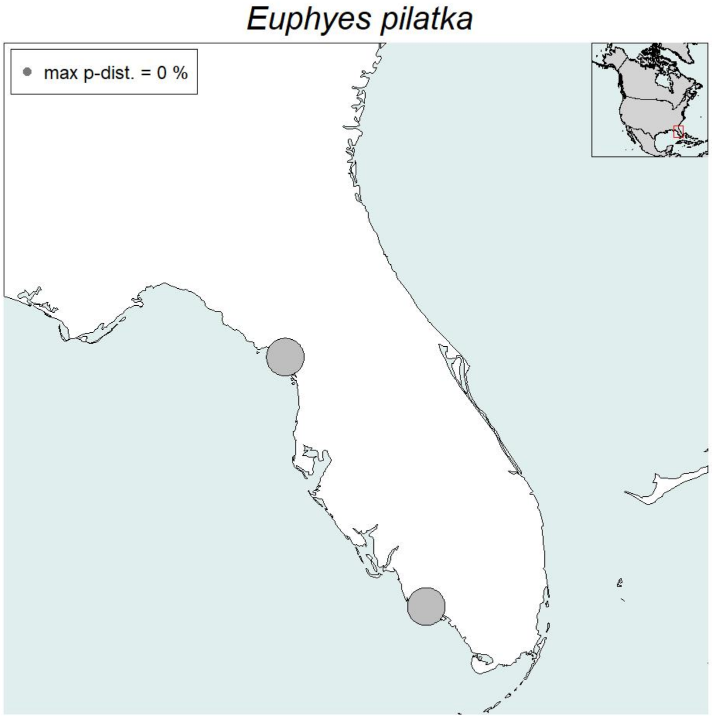

Figure 110: Map of *Euphyes pilatka* showing the localities of the sequenced specimens. Nearby localities are grouped in pies. Due to the presence of a single haplotype PCoA projection was not done and a single grey colour was plotted on the map. Sequences= 2; Hap obs.= 1; Hap asympt.= NA; Hap % obs.= NA%; GST= NaN; DST= NaN; HD= NA; ND= NA; max p-dist= 0%.

Haplotype network analysis and bubble plot of *Euphyes pilatka* were not possible. Sequences > 599 bp = 2.

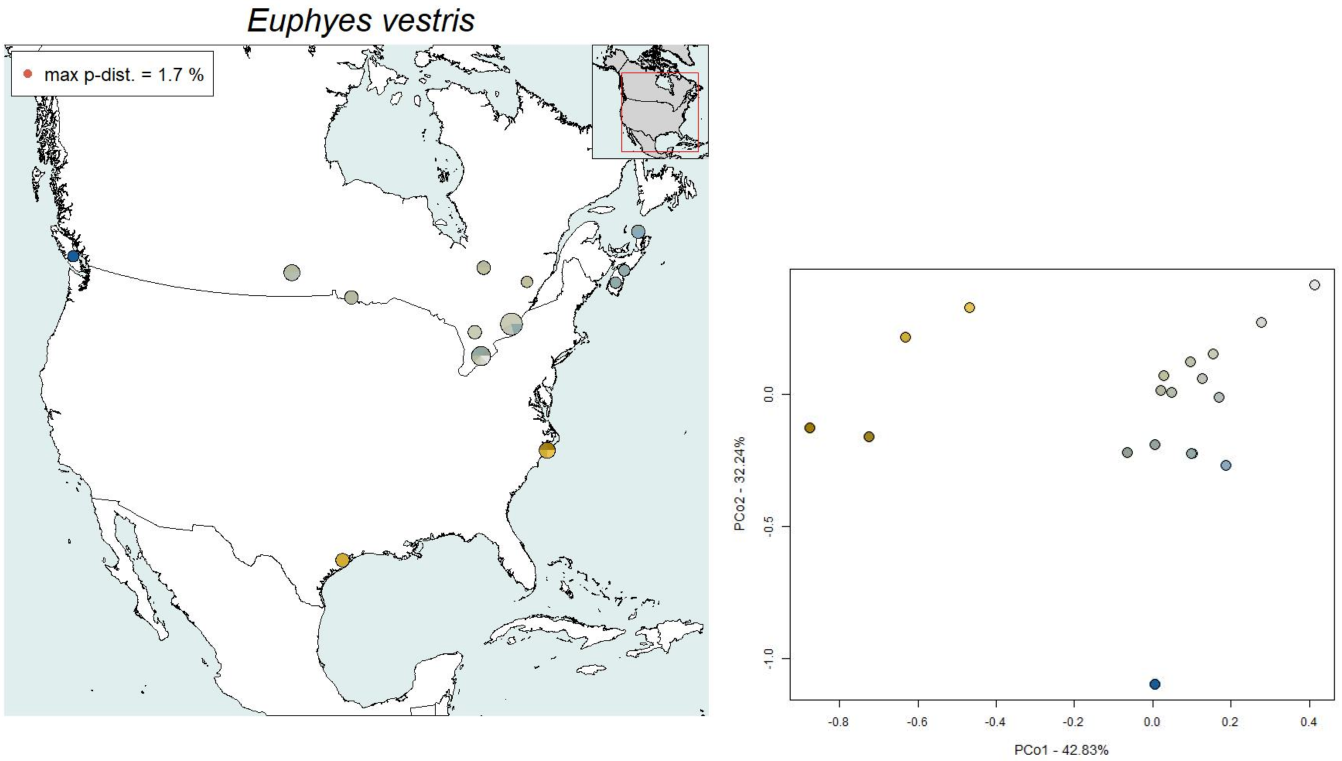

Figure 111 Map of *Euphyes vestris* showing the localities of the sequenced specimens (left). Nearby localities are grouped in pies. Colours match the bidimensional colour space of the PCoA projection (right) of max p-dists among sequences (dots). Sequences= 44; Hap obs.= 15; Hap asympt.= 34.8; Hap % obs.= 43.1%; GST= 0.454; DST= 0.0017; HD= 0.854; ND= 0.0043; max p-dist= 1.7%.

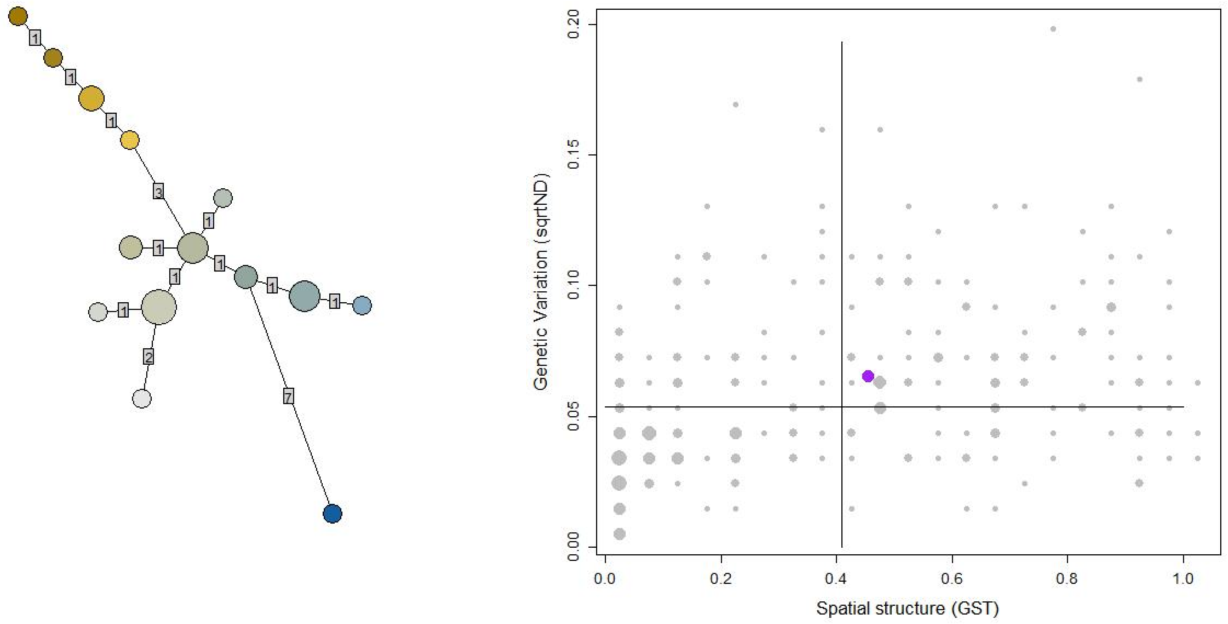

Figure 112: Haplotype network (left) of *Euphyes vestris* sequences > 599 bp with colours matching the PCoA colour space (above). The bubble plot for mt-DNA polymorphism (square root transformed nucleotide diversity) and spatial structure (GST) among all species in the atlas and values for *Euphyes vestris* (purple dot). The horizontal and vertical lines represent median values of nucleotide diversity and GST, respectively. Sequences > 599 bp= 40.

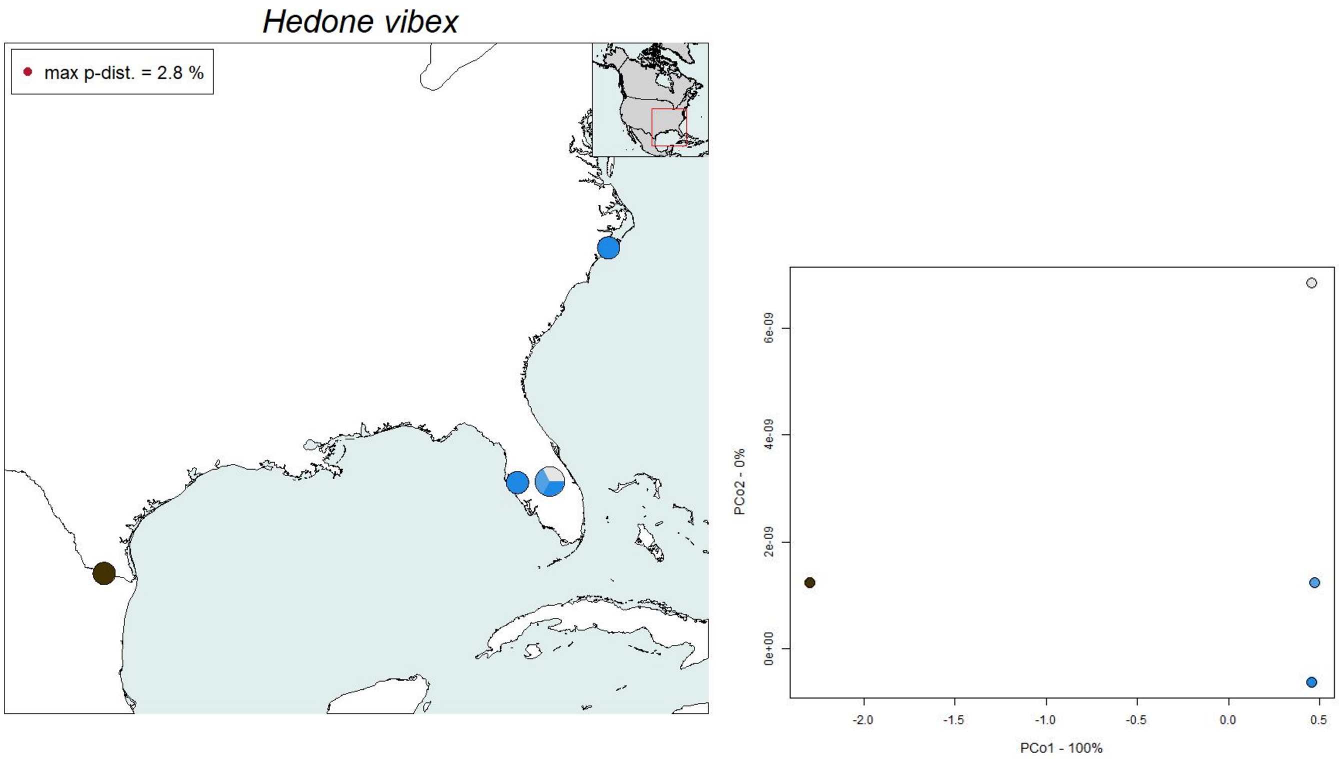

Figure 113 Map of *Hedone vibex* showing the localities of the sequenced specimens (left). Nearby localities are grouped in pies. Colours match the bidimensional colour space of the PCoA projection (right) of max p-dists among sequences (dots). Sequences= 6; Hap obs.= 2; Hap asympt.= NA; Hap % obs.= NA%; GST= NaN; DST= NaN; HD= NA; ND= NA; max p-dist= 2.8%.

Haplotype network analysis and bubble plot of *Hedone vibex* were not possible. Sequences > 599 bp = 6.

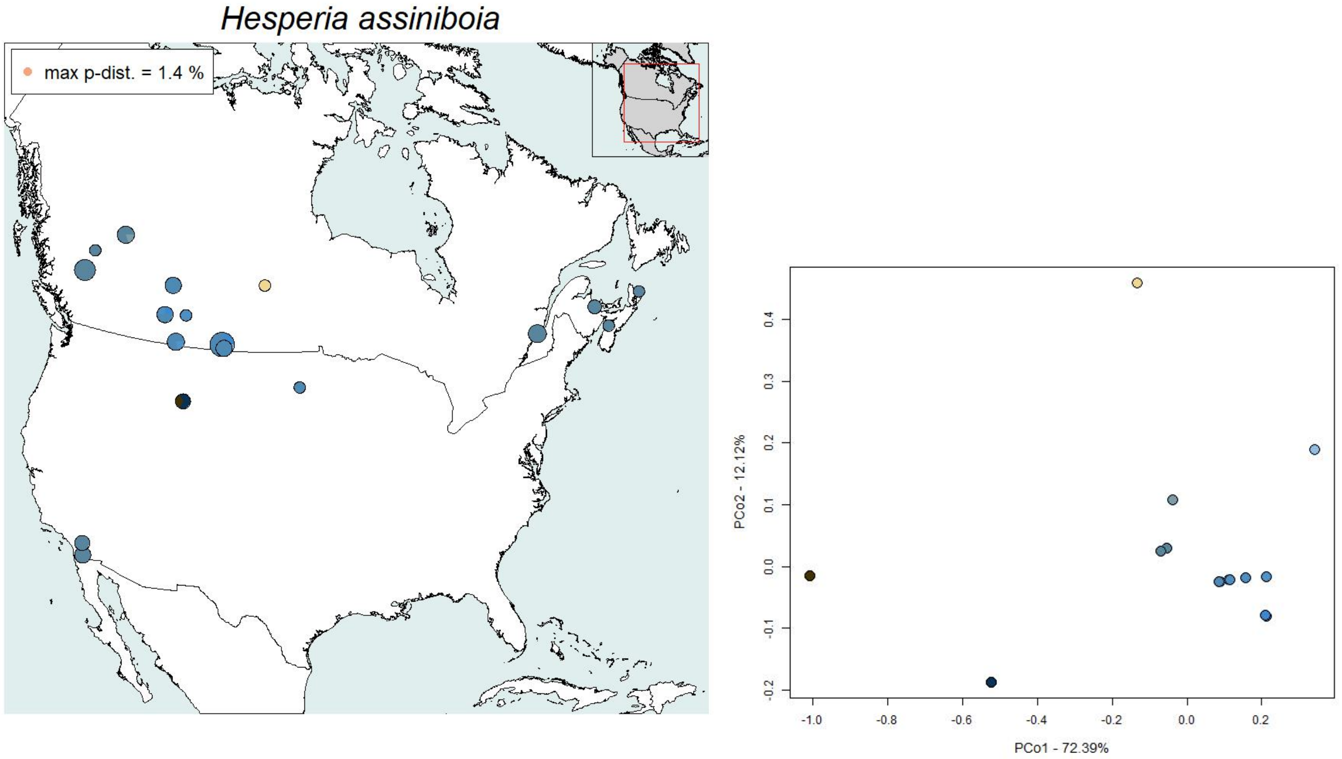

Figure 114 Map of *Hesperia assiniboia* showing the localities of the sequenced specimens (left). Nearby localities are grouped in pies. Colours match the bidimensional colour space of the PCoA projection (right) of max p-dists among sequences (dots). Sequences= 77; Hap obs.= 13; Hap asympt.= 62.4; Hap % obs.= 20.8%; GST= 0.61; DST= 0.001; HD= 0.65; ND= 0.0016; max p-dist= 1.4%.

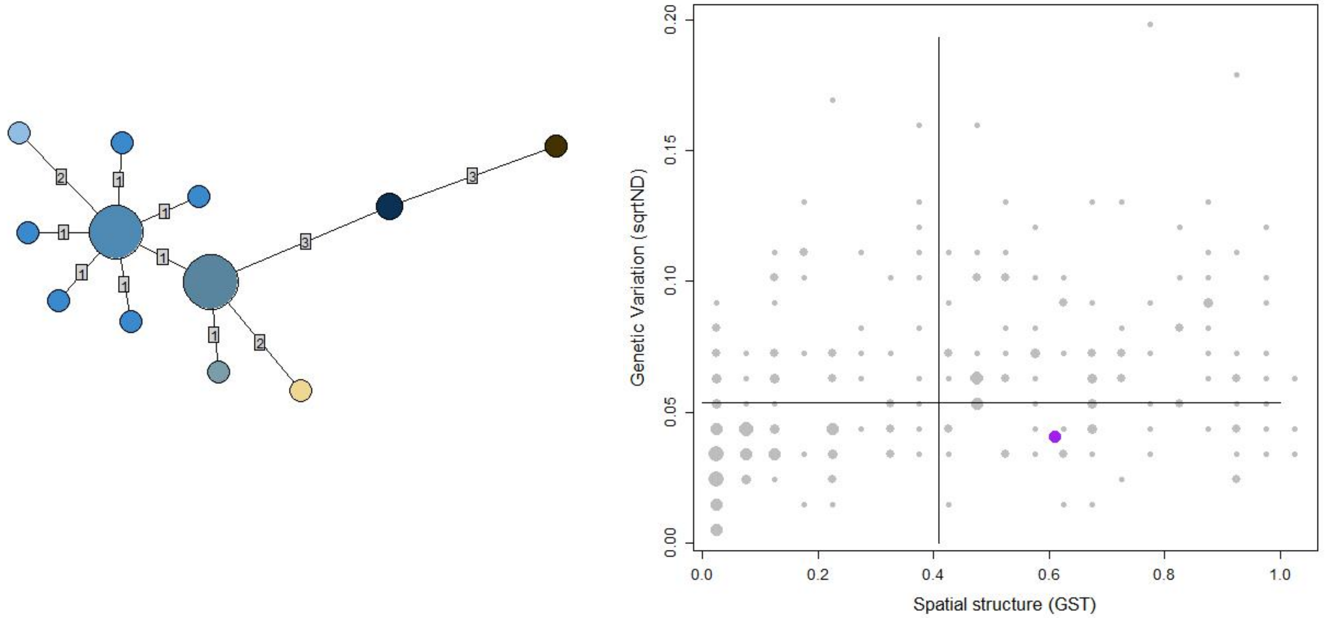

Figure 115: Haplotype network (left) of *Hesperia assiniboia* sequences > 599 bp with colours matching the PCoA colour space (above). The bubble plot for mt-DNA polymorphism (square root transformed nucleotide diversity) and spatial structure (GST) among all species in the atlas and values for *Hesperia assiniboia* (purple dot). The horizontal and vertical lines represent median values of nucleotide diversity and GST, respectively. Sequences > 599 bp= 75.

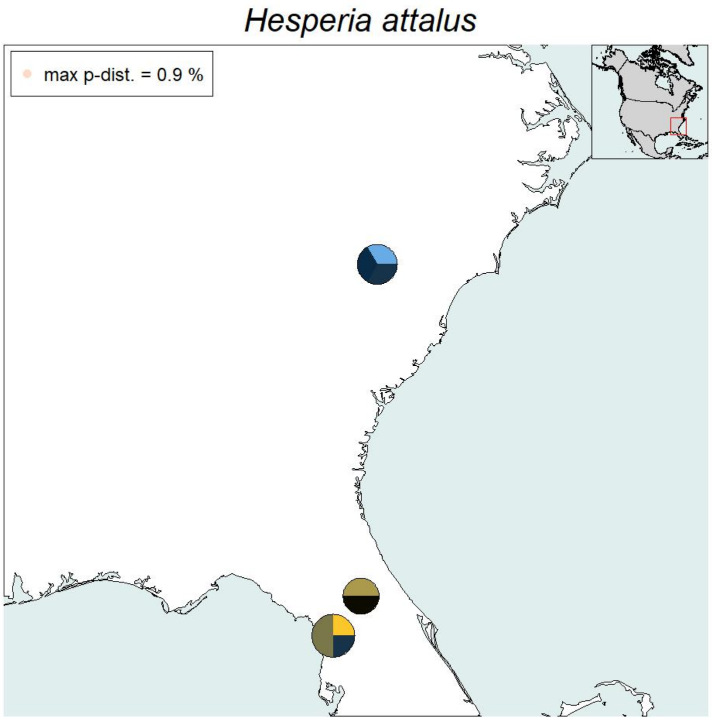

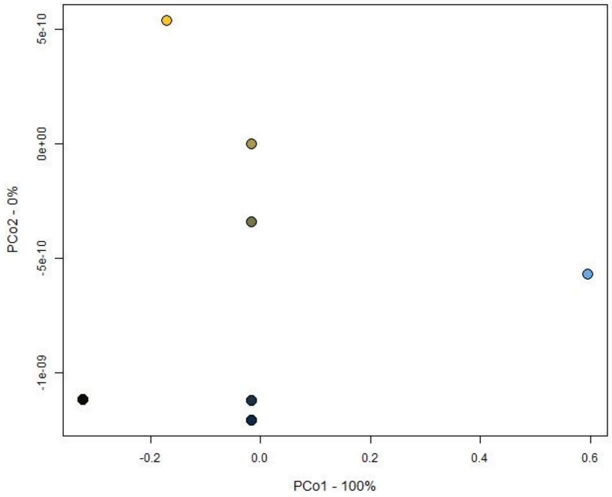

Figure 116 Map of *Hesperia attalus* showing the localities of the sequenced specimens (left). Nearby localities are grouped in pies. Colours match the bidimensional colour space of the PCoA projection (right) of max p-dists among sequences (dots). Sequences= 9; Hap obs.= 4; Hap asympt.= NA; Hap % obs.= NA%; GST= NaN; DST= NaN; HD= NA; ND= NA; max p-dist= 0.9%.

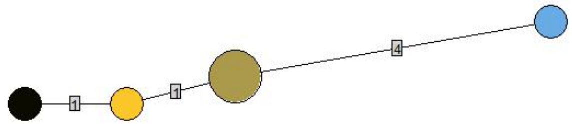

Figure 117: Haplotype network of *Hesperia attalus*. Sequences > 599 bp= 9.

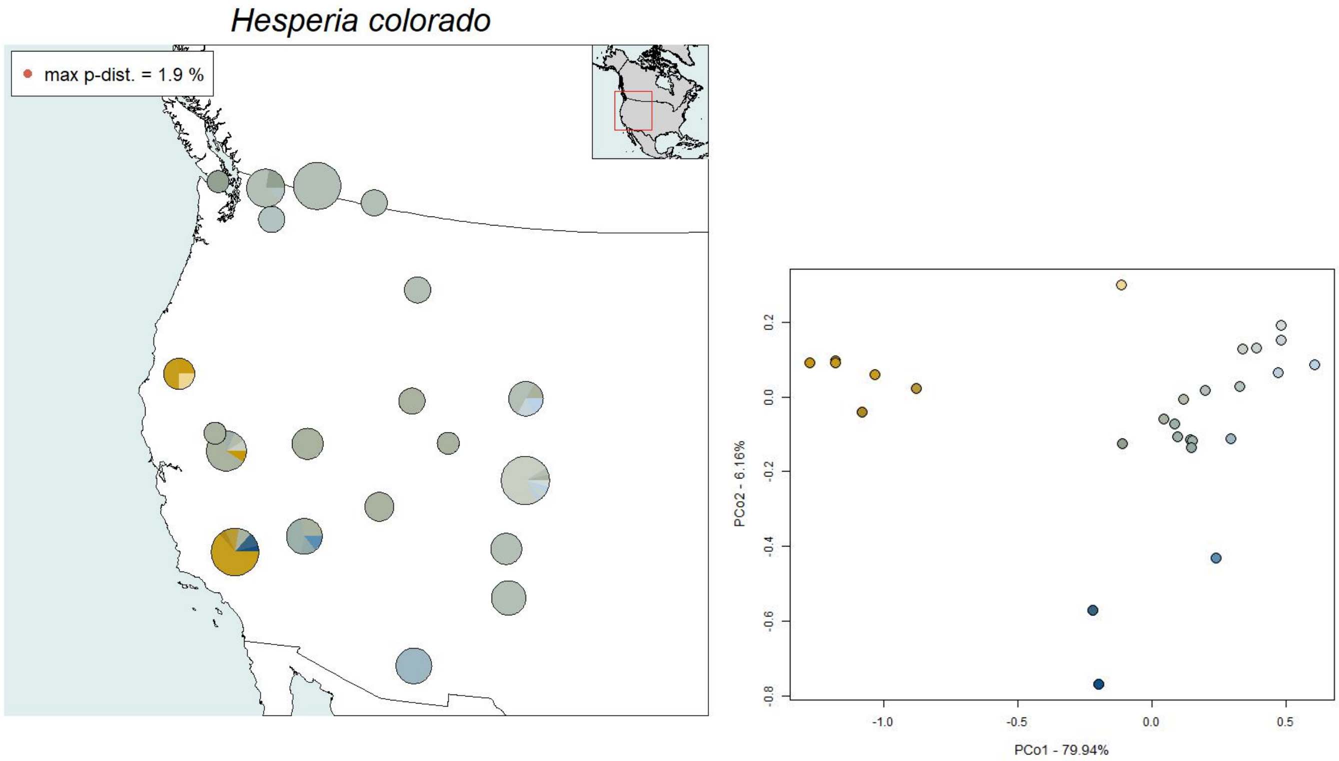

Figure 118 Map of *Hesperia colorado* showing the localities of the sequenced specimens (left). Nearby localities are grouped in pies. Colours match the bidimensional colour space of the PCoA projection (right) of max p-dists among sequences (dots). Sequences= 138; Hap obs.= 24; Hap asympt.= 40.8; Hap % obs.= 58.9%; GST= 0.694; DST= 0.0036; HD= 0.829; ND= 0.005; max p-dist= 1.9%.

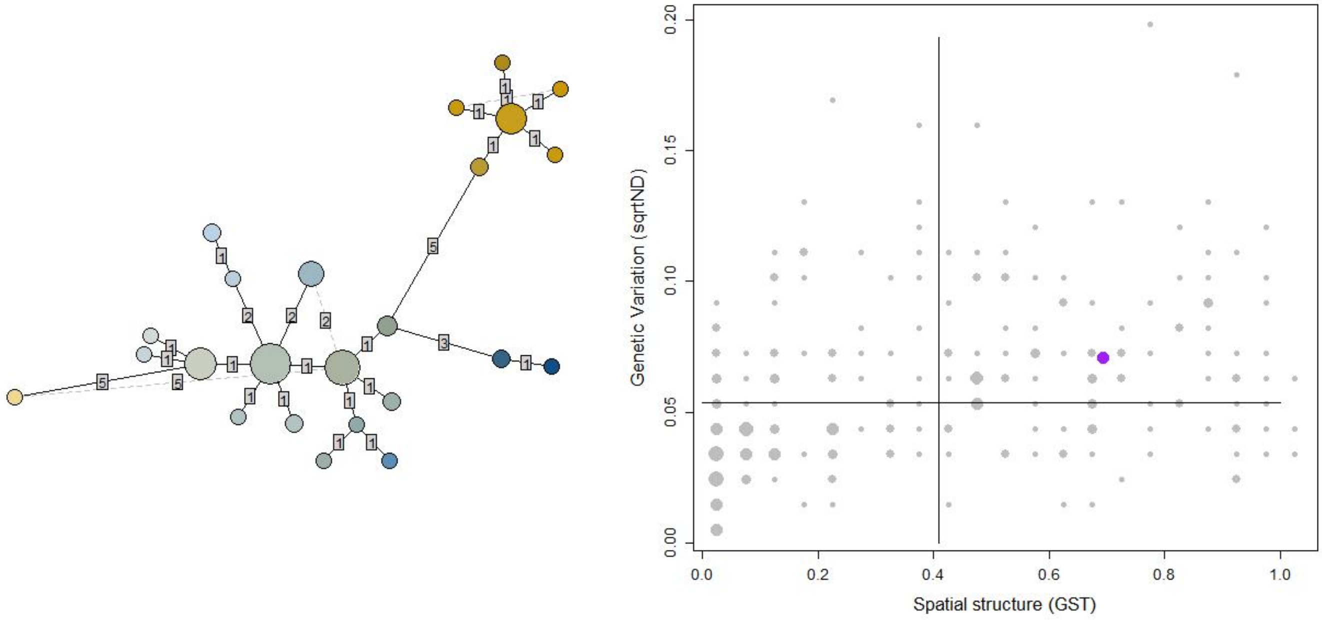

Figure 119: Haplotype network (left) of *Hesperia colorado* sequences > 599 bp with colours matching the PCoA colour space (above). The bubble plot for mt-DNA polymorphism (square root transformed nucleotide diversity) and spatial structure (GST) among all species in the atlas and values for *Hesperia colorado* (purple dot). The horizontal and vertical lines represent median values of nucleotide diversity and GST, respectively. Sequences > 599 bp= 138.

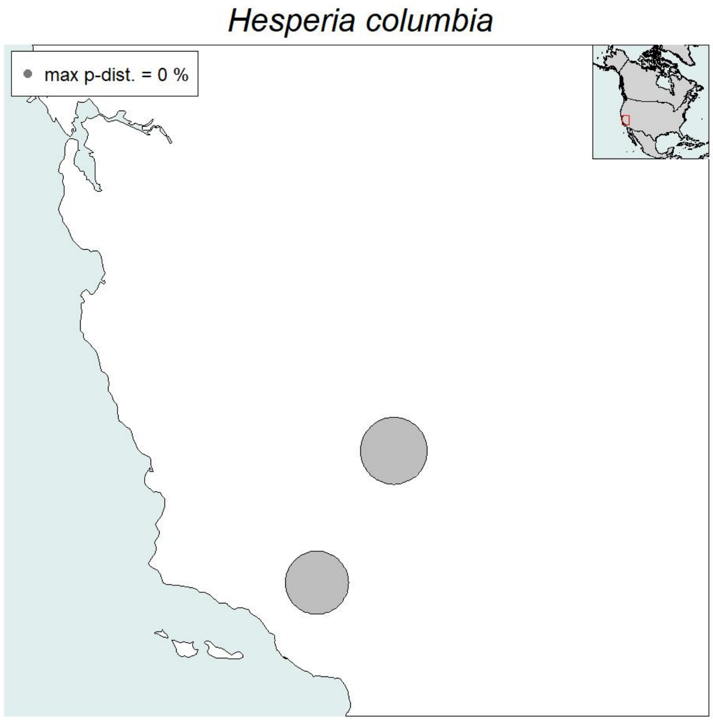

Figure 120: Map of *Hesperia columbia* showing the localities of the sequenced specimens. Nearby localities are grouped in pies. Due to the presence of a single haplotype PCoA projection was not done and a single grey colour was plotted on the map. Sequences= 9; Hap obs.= 1; Hap asympt.= NA; Hap % obs.= NA%; GST= NaN; DST= NaN; HD= NA; ND= NA; max p-dist= 0%.

Haplotype network analysis and bubble plot of *Hesperia columbia* were not possible. Sequences > 599 bp = 9.

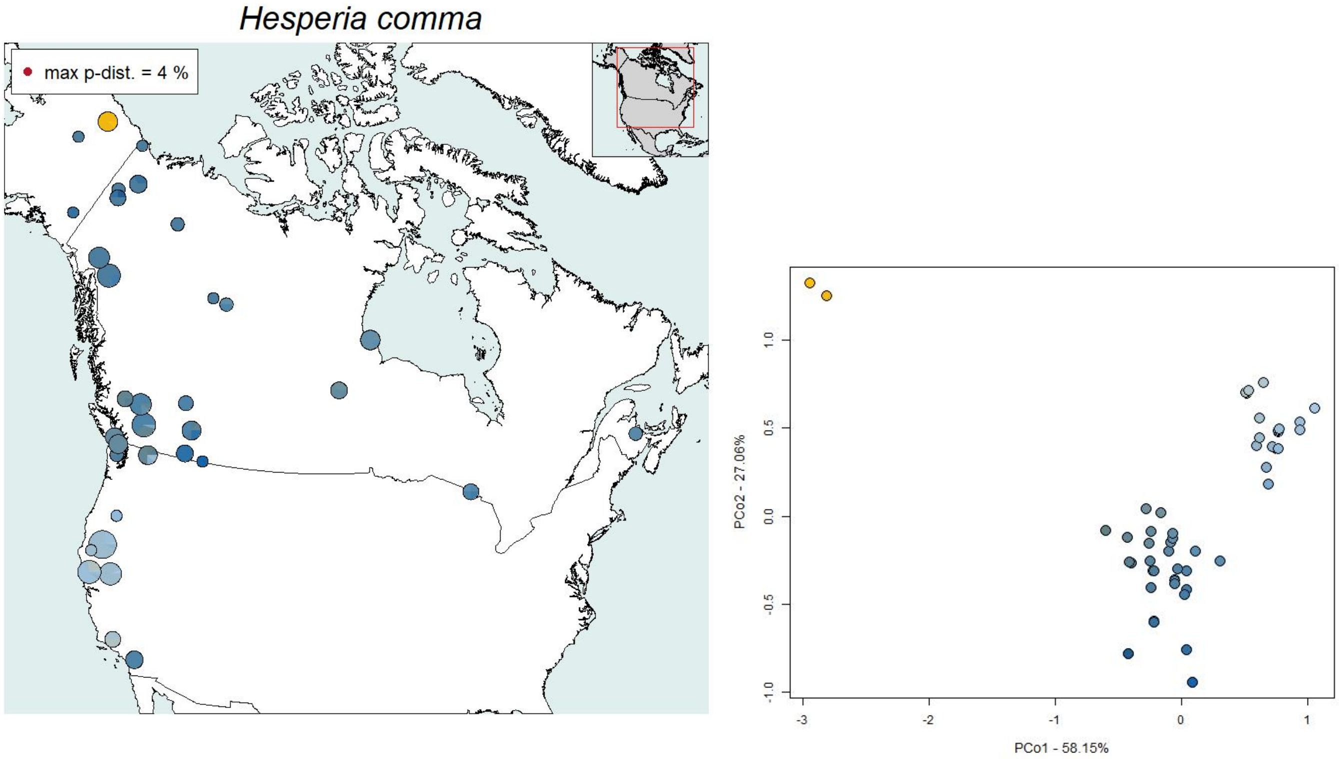

Figure 121 Map of *Hesperia comma* showing the localities of the sequenced specimens (left). Nearby localities are grouped in pies. Colours match the bidimensional colour space of the PCoA projection (right) of max p-dists among sequences (dots). Sequences= 239; Hap obs.= 48; Hap asympt.= 89; Hap % obs.= 54%; GST= 0.882; DST= 0.0085; HD= 0.918; ND= 0.0094; max p-dist= 4%.

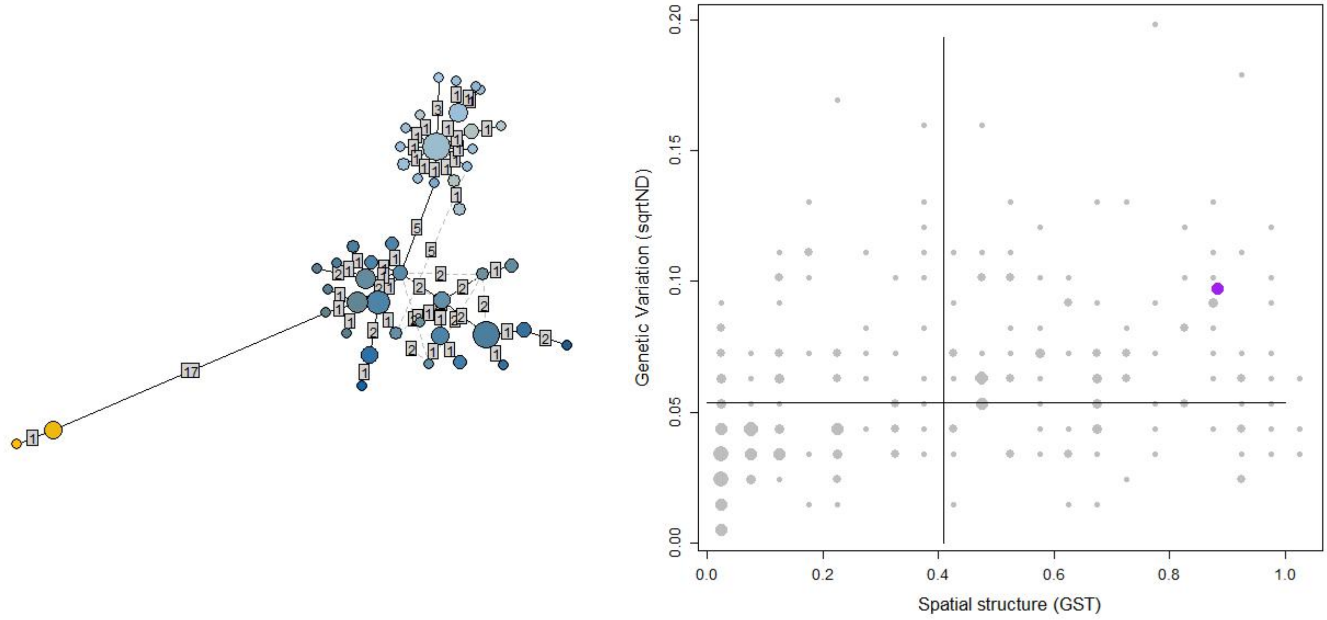

Figure 122: Haplotype network (left) of *Hesperia comma* sequences > 599 bp with colours matching the PCoA colour space (above). The bubble plot for mt-DNA polymorphism (square root transformed nucleotide diversity) and spatial structure (GST) among all species in the atlas and values for *Hesperia comma* (purple dot). The horizontal and vertical lines represent median values of nucleotide diversity and GST, respectively. Sequences > 599 bp= 235.

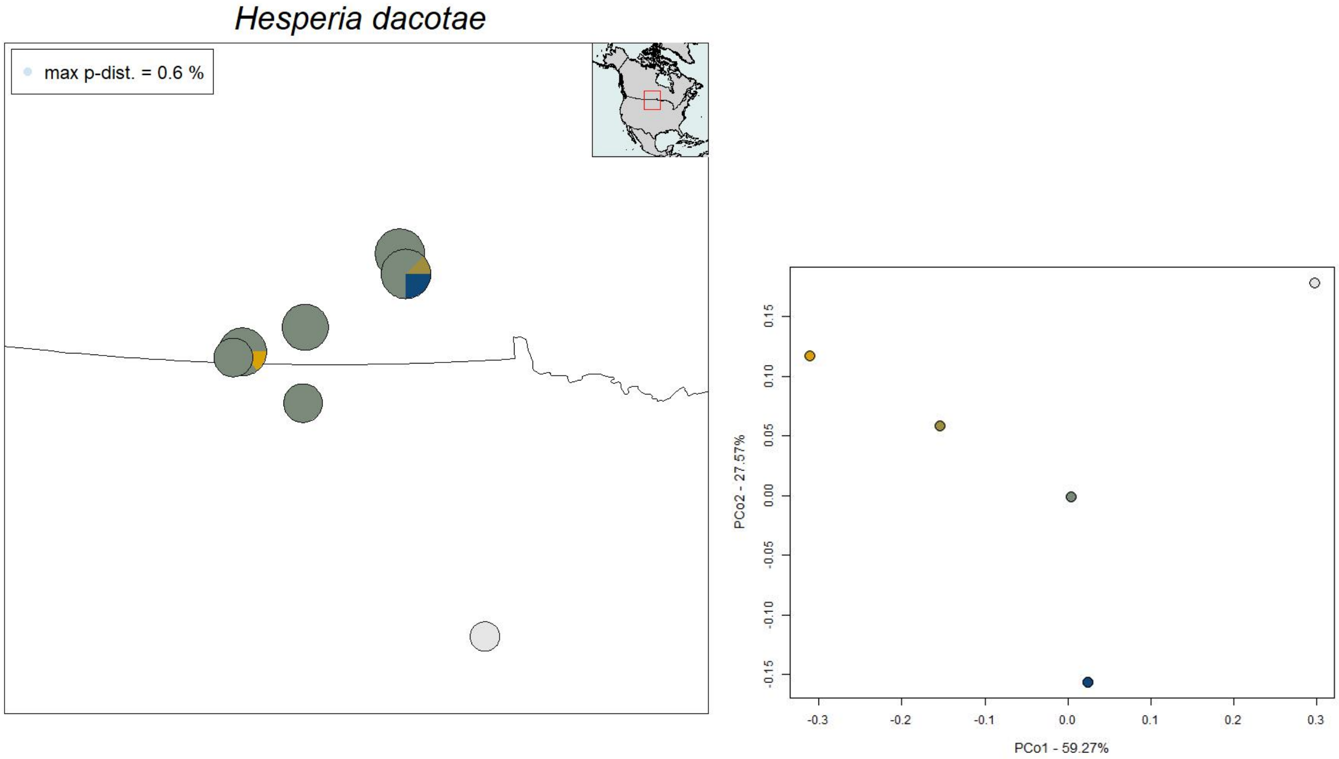

Figure 123 Map of *Hesperia dacotae* showing the localities of the sequenced specimens (left). Nearby localities are grouped in pies. Colours match the bidimensional colour space of the PCoA projection (right) of max p-dists among sequences (dots). Sequences= 36; Hap obs.= 6; Hap asympt.= 15.7; Hap % obs.= 38.2%; GST= 0.011; DST= 0; HD= 0.262; ND= 0.0006; max p-dist= 0.6%.

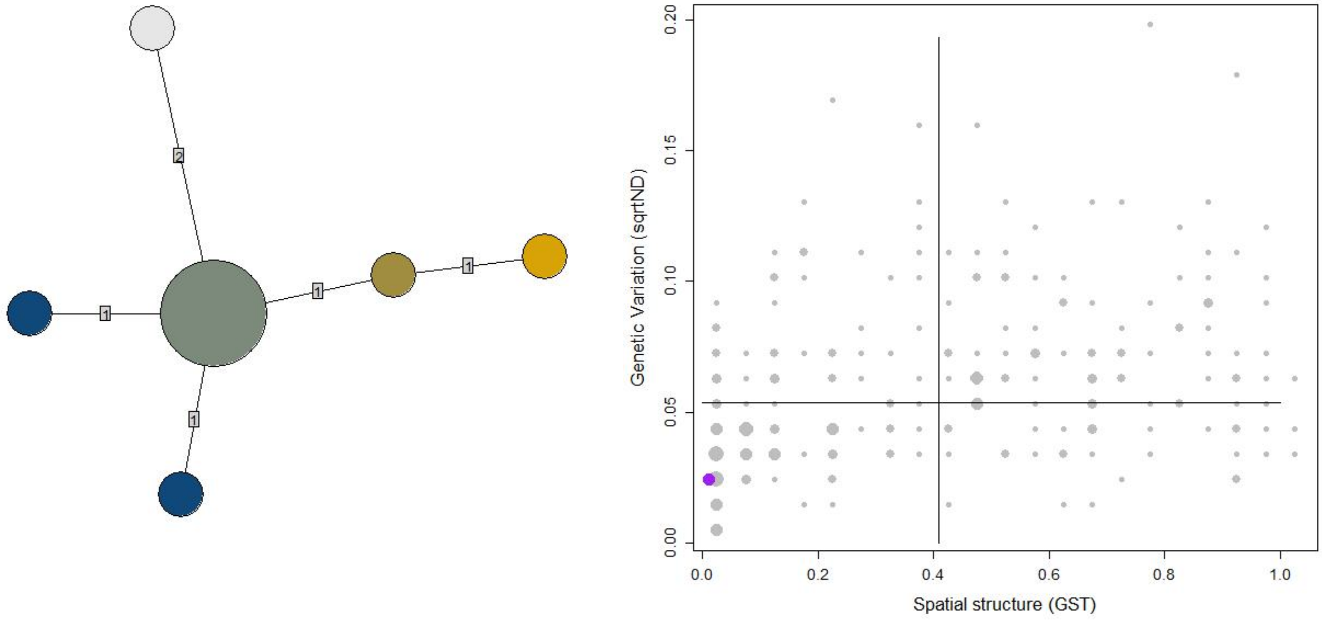

Figure 124: Haplotype network (left) of *Hesperia dacotae* sequences > 599 bp with colours matching the PCoA colour space (above). The bubble plot for mt-DNA polymorphism (square root transformed nucleotide diversity) and spatial structure (GST) among all species in the atlas and values for *Hesperia dacotae* (purple dot). The horizontal and vertical lines represent median values of nucleotide diversity and GST, respectively. Sequences > 599 bp= 36.

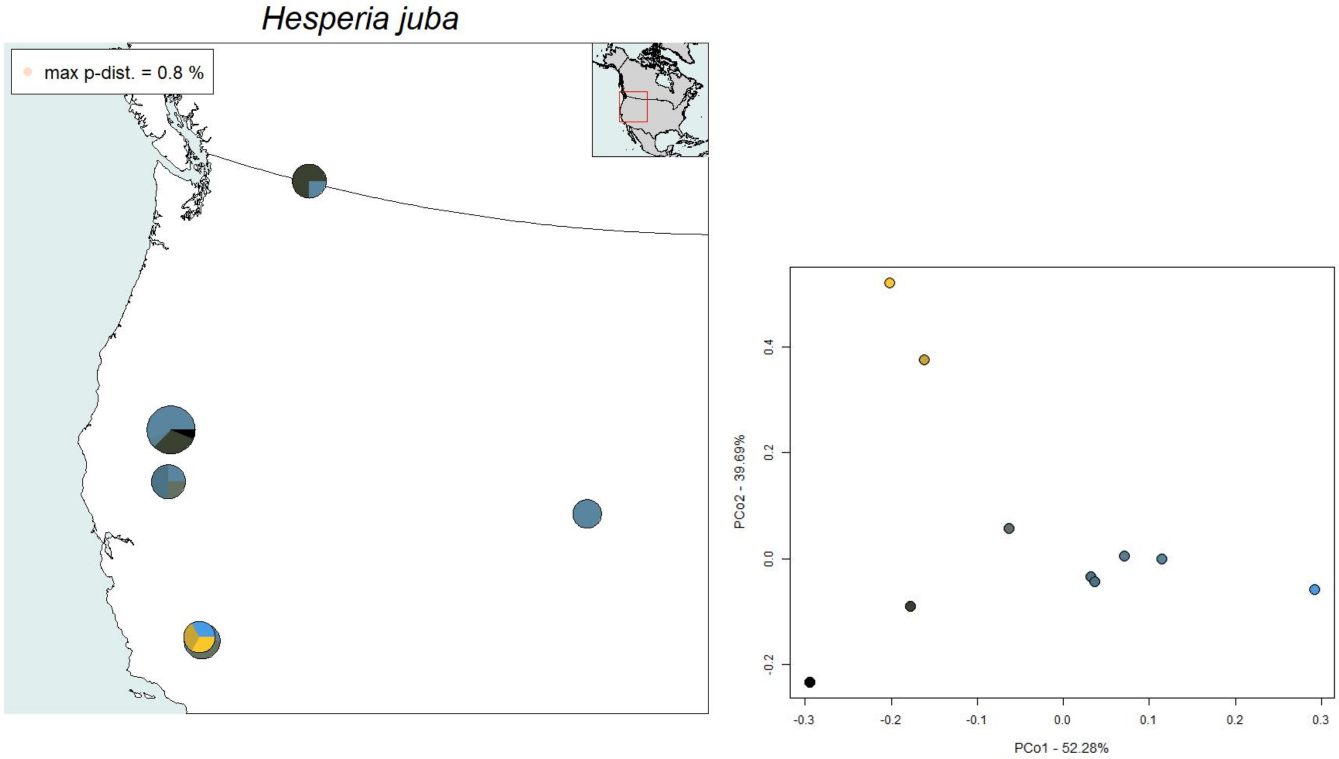

Figure 125 Map of *Hesperia juba* showing the localities of the sequenced specimens (left). Nearby localities are grouped in pies. Colours match the bidimensional colour space of the PCoA projection (right) of max p-dists among sequences (dots). Sequences= 34; Hap obs.= 9; Hap asympt.= 21.1; Hap % obs.= 42.6%; GST= 0.129; DST= 0.0003; HD= 0.729; ND= 0.002; max p-dist= 0.8%.

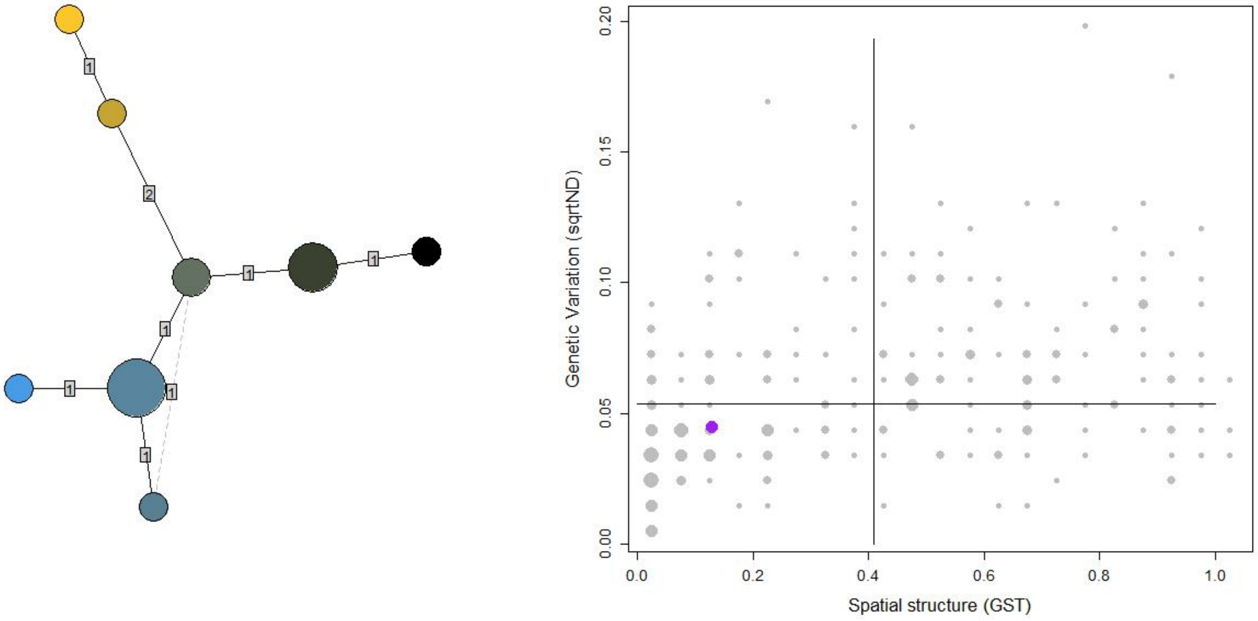

Figure 126: Haplotype network (left) of *Hesperia juba* sequences > 599 bp with colours matching the PCoA colour space (above). The bubble plot for mt-DNA polymorphism (square root transformed nucleotide diversity) and spatial structure (GST) among all species in the atlas and values for *Hesperia juba* (purple dot). The horizontal and vertical lines represent median values of nucleotide diversity and GST, respectively. Sequences > 599 bp= 32.

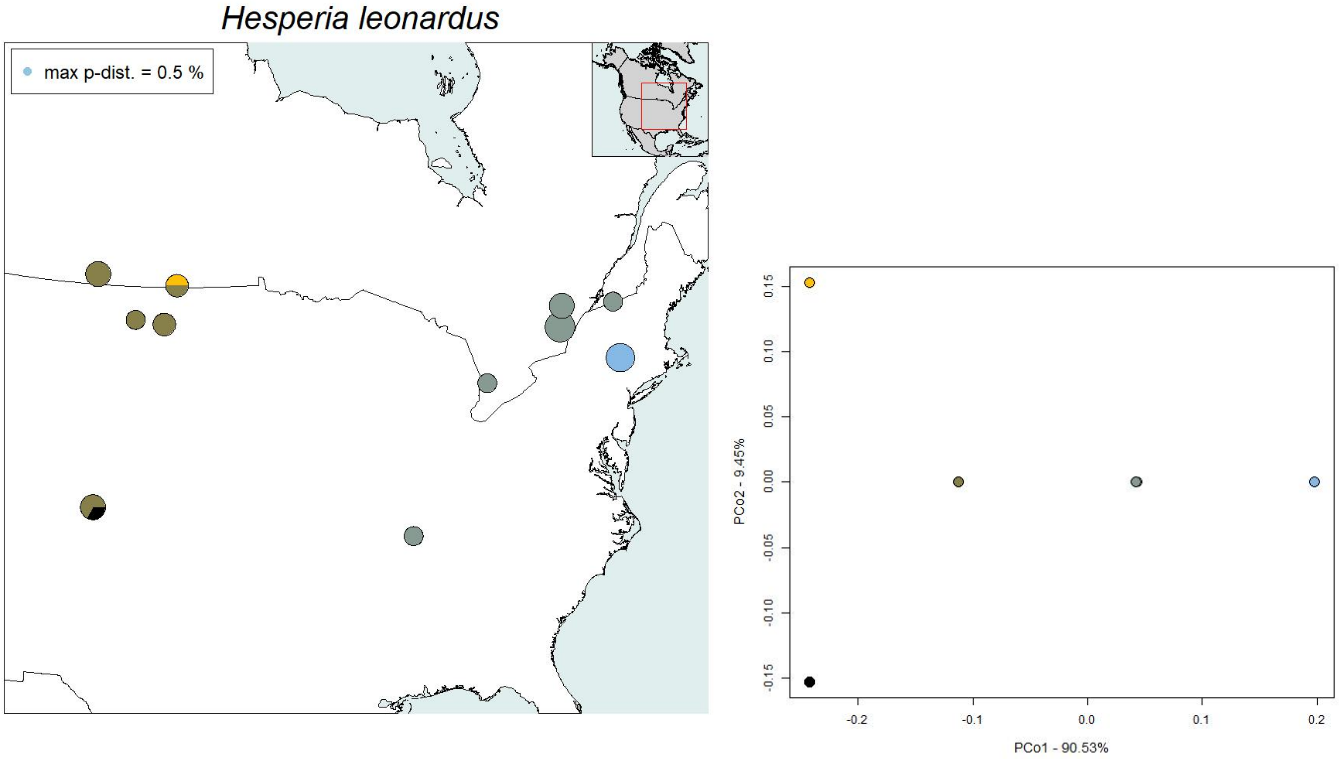

Figure 127 Map of *Hesperia leonardus* showing the localities of the sequenced specimens (left). Nearby localities are grouped in pies. Colours match the bidimensional colour space of the PCoA projection (right) of max p-dists among sequences (dots). Sequences= 28; Hap obs.= 5; Hap asympt.= 6; Hap % obs.= 83.8%; GST= 0.928; DST= 0.0014; HD= 0.704; ND= 0.0014; max p-dist= 0.5%.

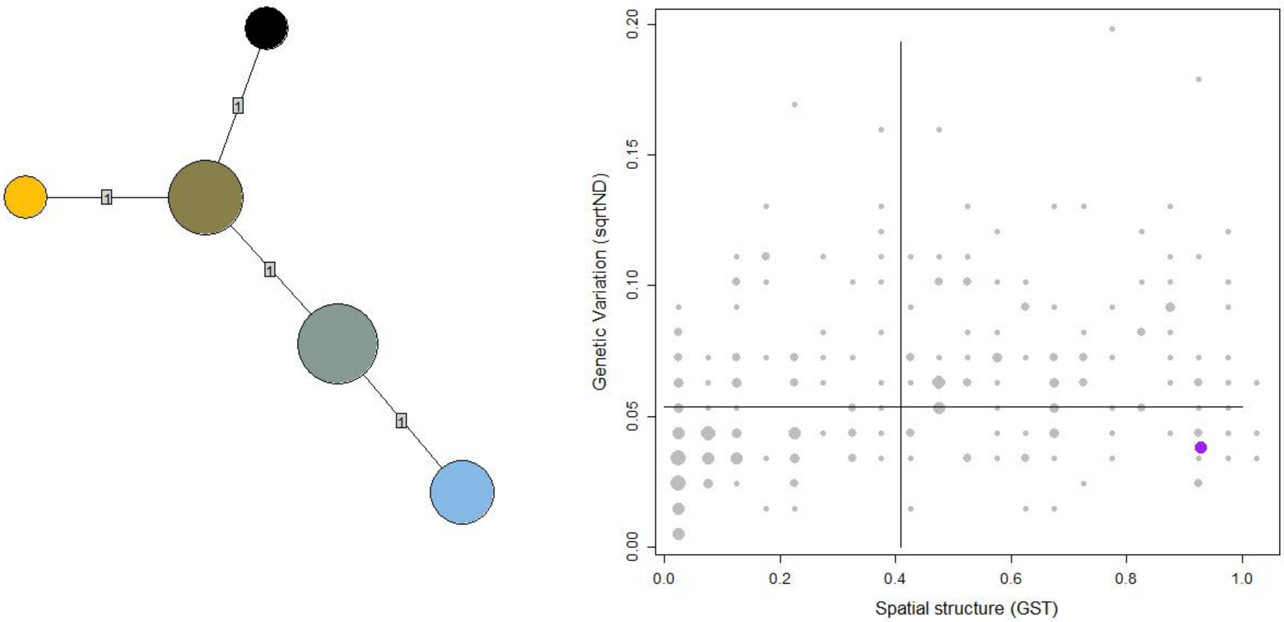

Figure 128: Haplotype network (left) of *Hesperia leonardus* sequences > 599 bp with colours matching the PCoA colour space (above). The bubble plot for mt-DNA polymorphism (square root transformed nucleotide diversity) and spatial structure (GST) among all species in the atlas and values for *Hesperia leonardus* (purple dot). The horizontal and vertical lines represent median values of nucleotide diversity and GST, respectively. Sequences > 599 bp= 28.

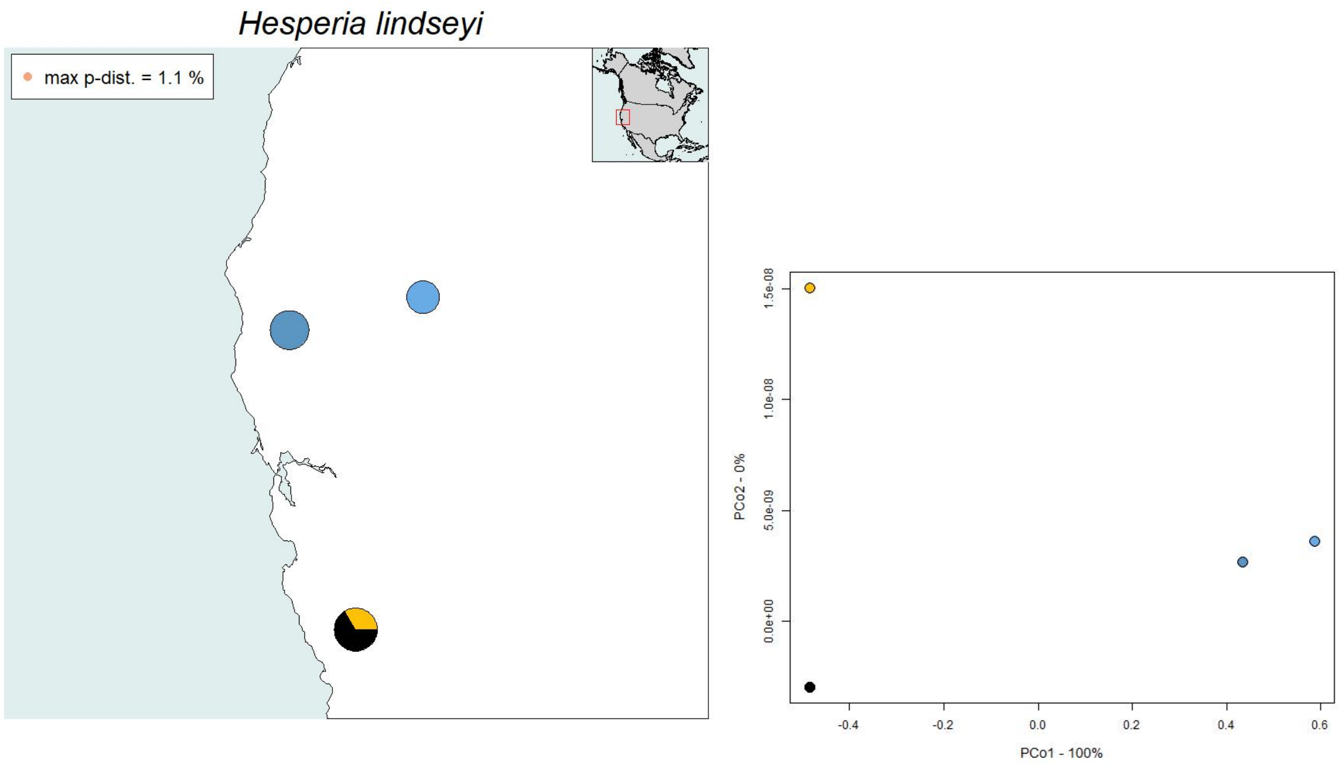

Figure 129 Map of *Hesperia lindseyi* showing the localities of the sequenced specimens (left). Nearby localities are grouped in pies. Colours match the bidimensional colour space of the PCoA projection (right) of max p-dists among sequences (dots). Sequences= 6; Hap obs.= 3; Hap asympt.= NA; Hap % obs.= NA%; GST= NaN; DST= NaN; HD= NA; ND= NA; max p-dist= 1.1%.

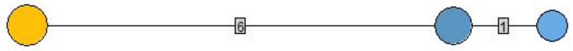

Figure 130: Haplotype network of *Hesperia lindseyi*. Sequences > 599 bp= 6.

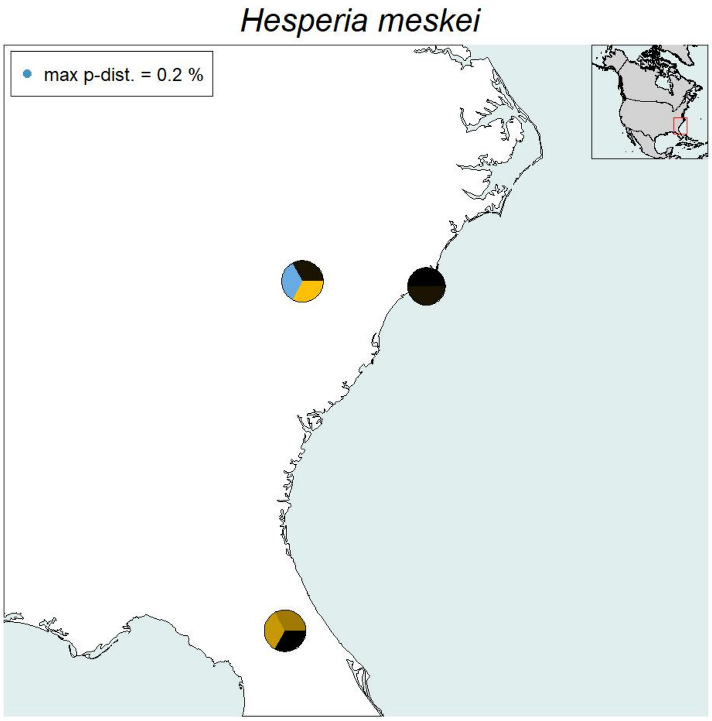

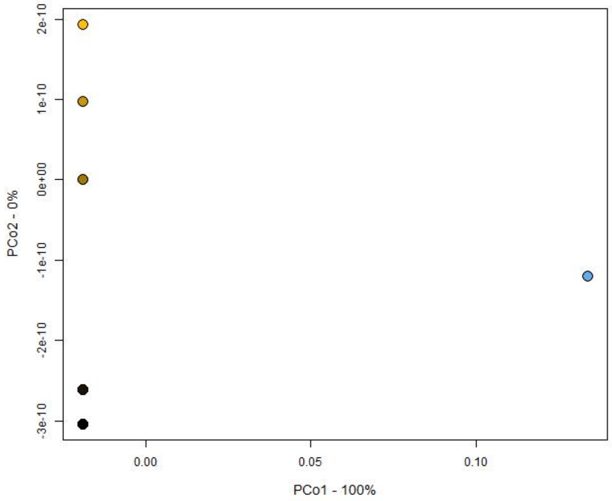

Figure 131 Map of *Hesperia meskei* showing the localities of the sequenced specimens (left). Nearby localities are grouped in pies. Colours match the bidimensional colour space of the PCoA projection (right) of max p-dists among sequences (dots). Sequences= 8; Hap obs.= 2; Hap asympt.= NA; Hap % obs.= NA%; GST= NaN; DST= NaN; HD= NA; ND= NA; max p-dist= 0.2%.

Haplotype network analysis and bubble plot of *Hesperia meskei* were not possible. Sequences > 599 bp = 8.

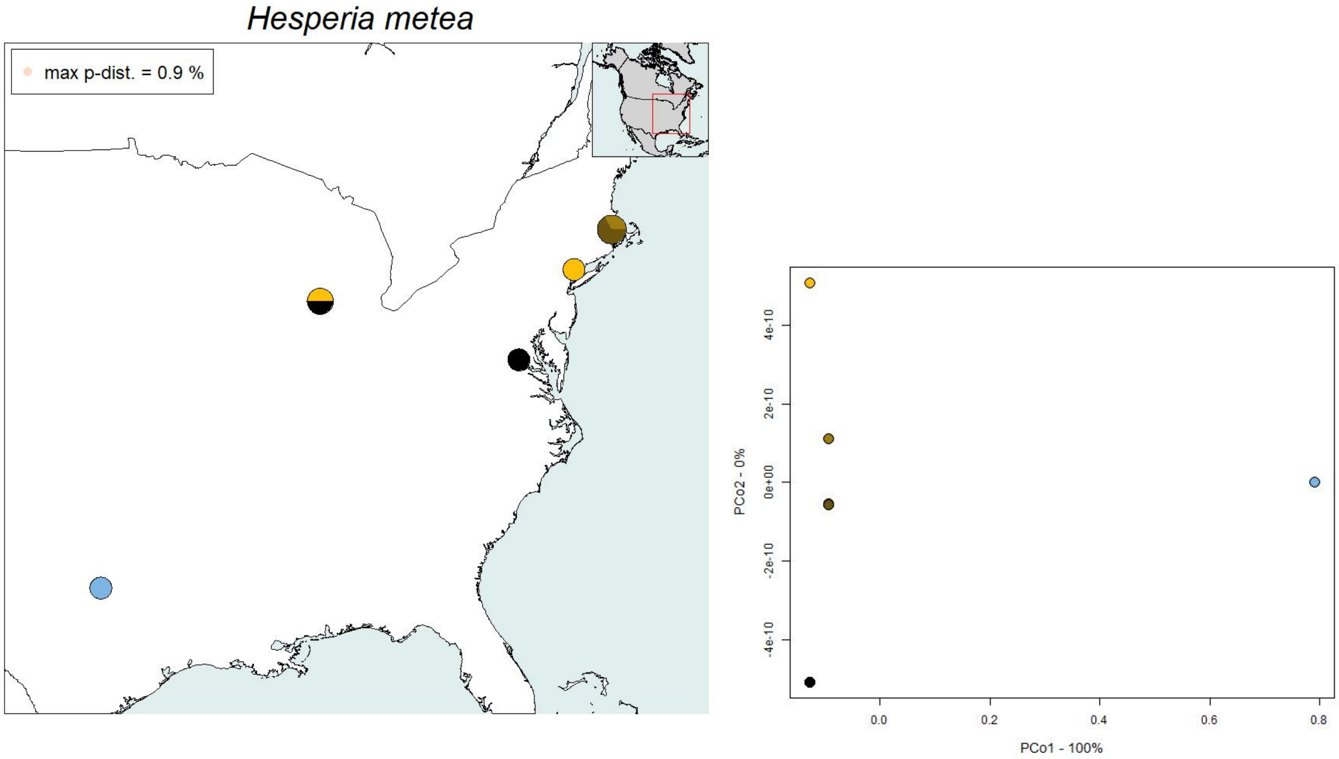

Figure 132 Map of *Hesperia metea* showing the localities of the sequenced specimens (left). Nearby localities are grouped in pies. Colours match the bidimensional colour space of the PCoA projection (right) of max p-dists among sequences (dots). Sequences= 8; Hap obs.= 3; Hap asympt.= NA; Hap % obs.= NA%; GST= NaN; DST= NaN; HD= NA; ND= NA; max p-dist= 0.9%.

Haplotype network analysis and bubble plot of *Hesperia metea* were not possible. Sequences > 599 bp = 5.

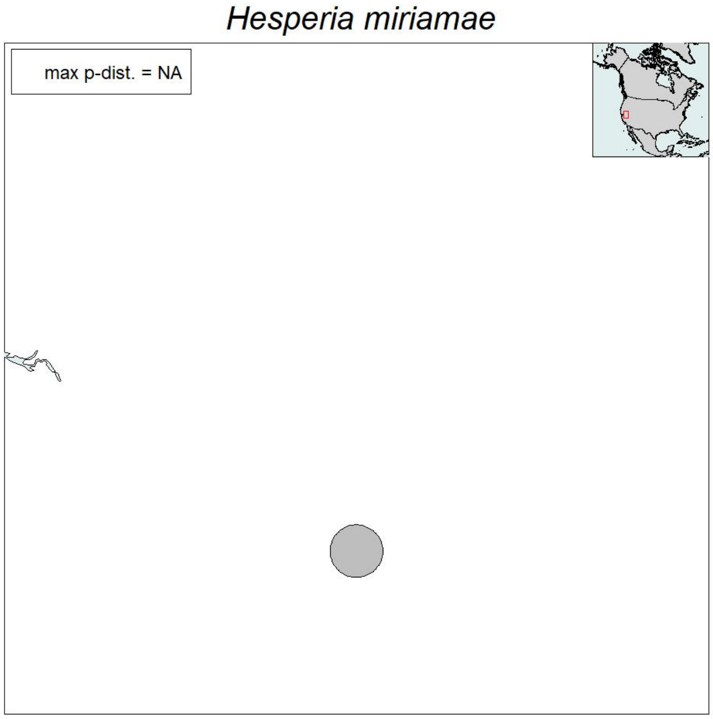

Figure 133: Map of *Hesperia miriamae* showing the localities of the sequenced specimens. Nearby localities are grouped in pies. Due to the presence of a single haplotype PCoA projection was not done and a single grey colour was plotted on the map. Sequences= 1; Hap obs.= NA; Hap asympt.= NA; Hap % obs.= NA; GST= NaN; DST= NaN; HD= NA; ND= NA; max p-dist= NA.

Haplotype network analysis and bubble plot of *Hesperia miriamae* were not possible. Sequences > 599 bp = 1.

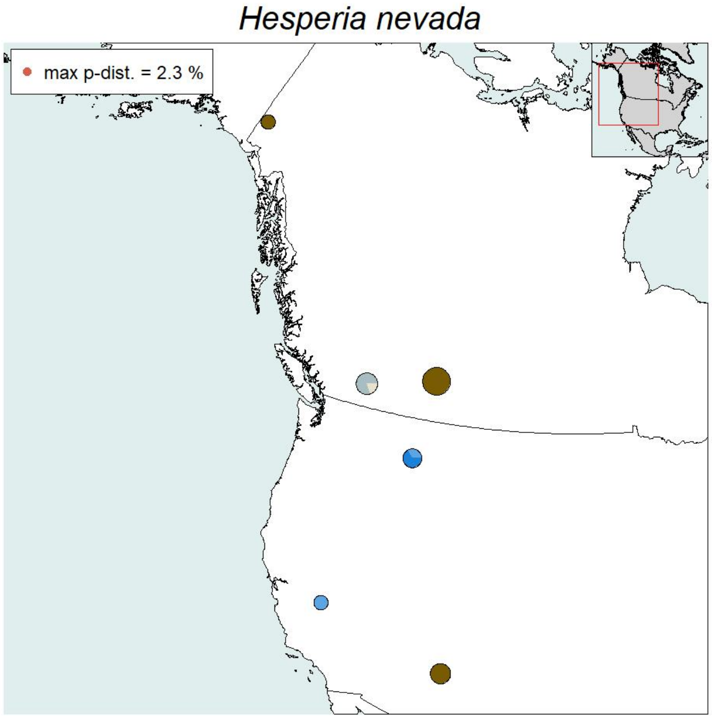

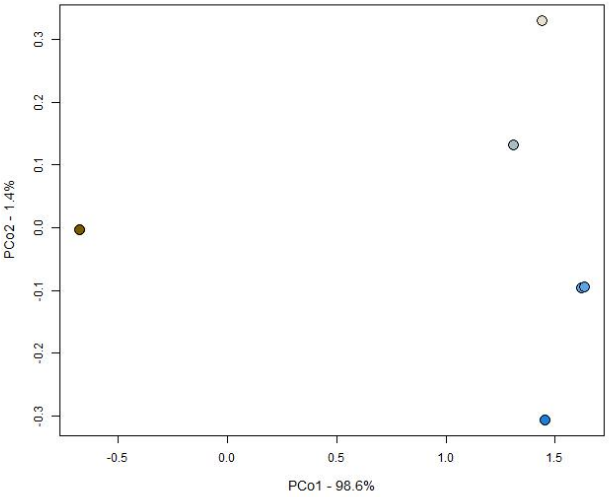

Figure 134 Map of *Hesperia nevada* showing the localities of the sequenced specimens (left). Nearby localities are grouped in pies. Colours match the bidimensional colour space of the PCoA projection (right) of max p-dists among sequences (dots). Sequences= 28; Hap obs.= 5; Hap asympt.= 5.2; Hap % obs.= 95.4%; GST= 0.987; DST= 0.0094; HD= 0.526; ND= 0.0098; max p-dist= 2.3%.

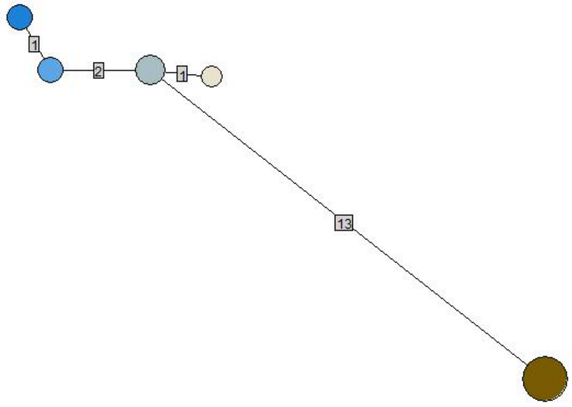

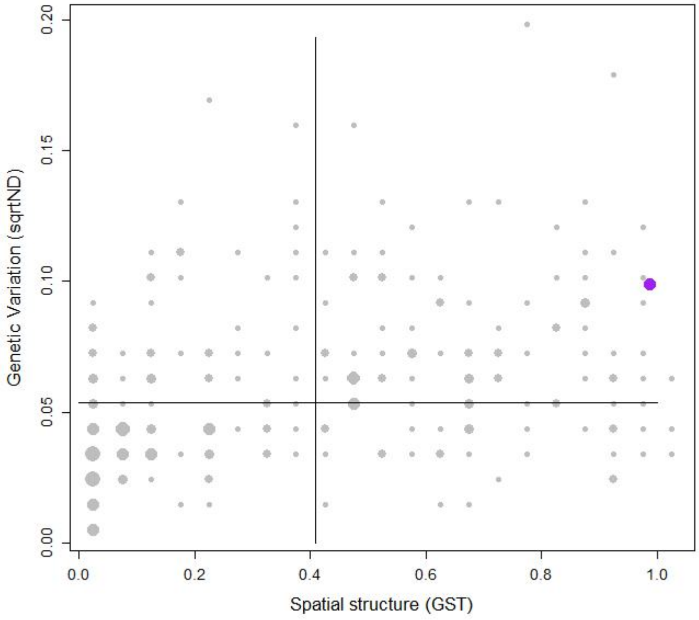

Figure 135: Haplotype network (left) of *Hesperia nevada* sequences > 599 bp with colours matching the PCoA colour space (above). The bubble plot for mt-DNA polymorphism (square root transformed nucleotide diversity) and spatial structure (GST) among all species in the atlas and values for *Hesperia nevada* (purple dot). The horizontal and vertical lines represent median values of nucleotide diversity and GST, respectively. Sequences > 599 bp= 28.

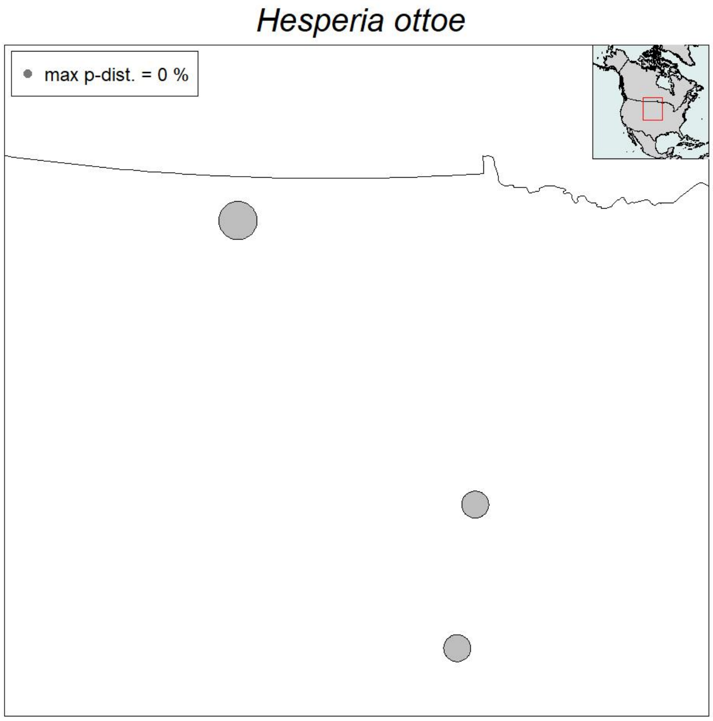

Figure 136: Map of *Hesperia ottoe* showing the localities of the sequenced specimens. Nearby localities are grouped in pies. Due to the presence of a single haplotype PCoA projection was not done and a single grey colour was plotted on the map. Sequences= 6; Hap obs.= 2; Hap asympt.= NA; Hap % obs.= NA%; GST= NaN; DST= NaN; HD= NA; ND= NA; max p-dist= 0%.

Haplotype network analysis and bubble plot of *Hesperia ottoe* were not possible. Sequences > 599 bp = 5.

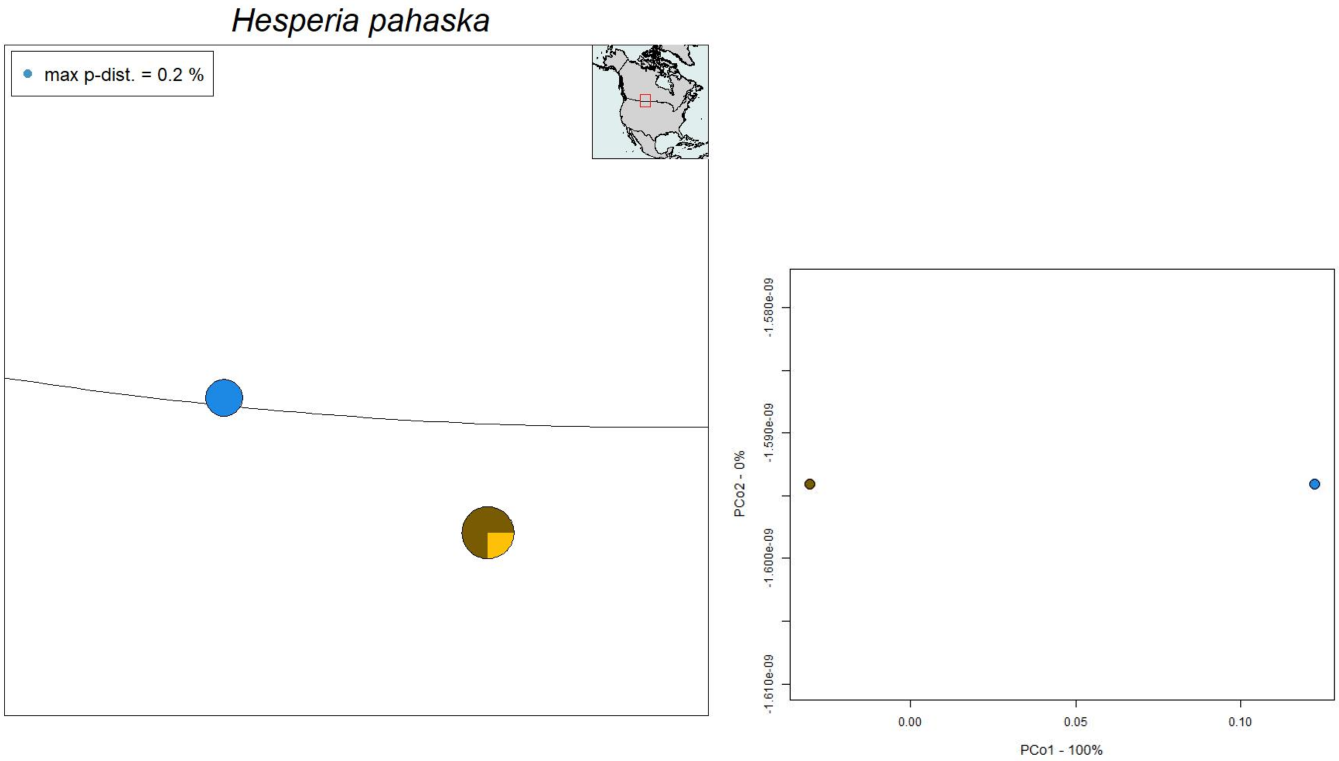

Figure 137 Map of *Hesperia pahaska* showing the localities of the sequenced specimens (left). Nearby localities are grouped in pies. Colours match the bidimensional colour space of the PCoA projection (right) of max p-dists among sequences (dots). Sequences= 5; Hap obs.= 2; Hap asympt.= NA; Hap % obs.= NA%; GST= NaN; DST= NaN; HD= NA; ND= NA; max p-dist= 0.2%.

Haplotype network analysis and bubble plot of *Hesperia pahaska* were not possible. Sequences > 599 bp = 5.

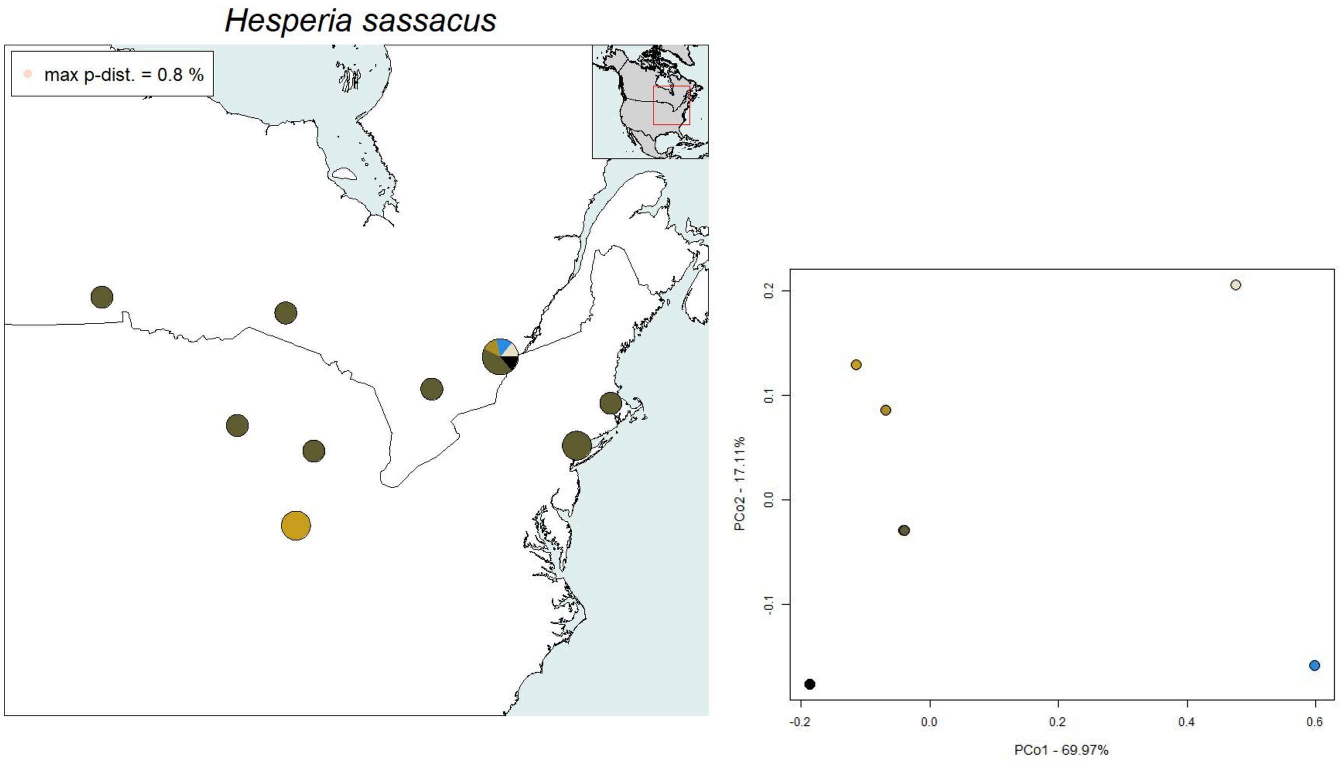

Figure 138 Map of *Hesperia sassacus* showing the localities of the sequenced specimens (left). Nearby localities are grouped in pies. Colours match the bidimensional colour space of the PCoA projection (right) of max p-dists among sequences (dots). Sequences= 19; Hap obs.= 6; Hap asympt.= 11.7; Hap % obs.= 51.4%; GST= 0.118; DST= 0.0002; HD= 0.596; ND= 0.0019; max p-dist= 0.8%.

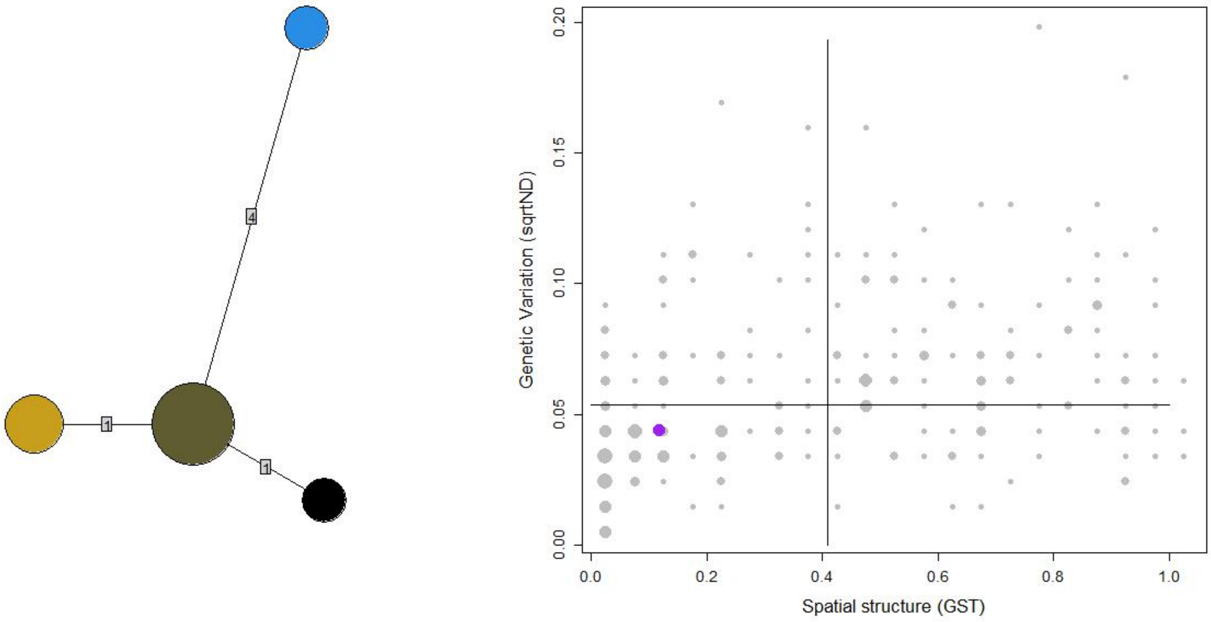

Figure 139: Haplotype network (left) of *Hesperia sassacus* sequences > 599 bp with colours matching the PCoA colour space (above). The bubble plot for mt-DNA polymorphism (square root transformed nucleotide diversity) and spatial structure (GST) among all species in the atlas and values for *Hesperia sassacus* (purple dot). The horizontal and vertical lines represent median values of nucleotide diversity and GST, respectively. Sequences > 599 bp= 17.

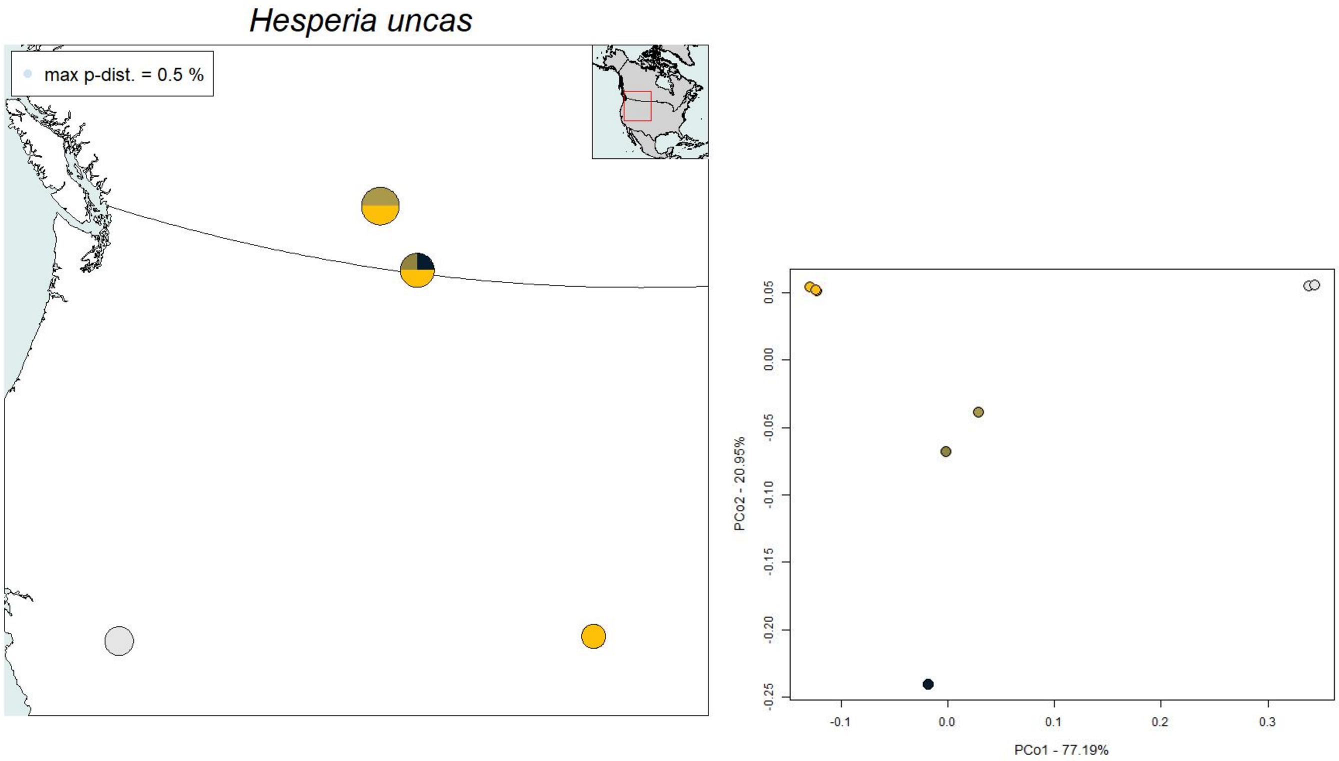

Figure 140 Map of *Hesperia uncas* showing the localities of the sequenced specimens (left). Nearby localities are grouped in pies. Colours match the bidimensional colour space of the PCoA projection (right) of max p-dists among sequences (dots). Sequences= 13; Hap obs.= 4; Hap asympt.= 4.5; Hap % obs.= 89.6%; GST= 0; DST= 0; HD= 0.718; ND= 0.002; max p-dist= 0.5%.

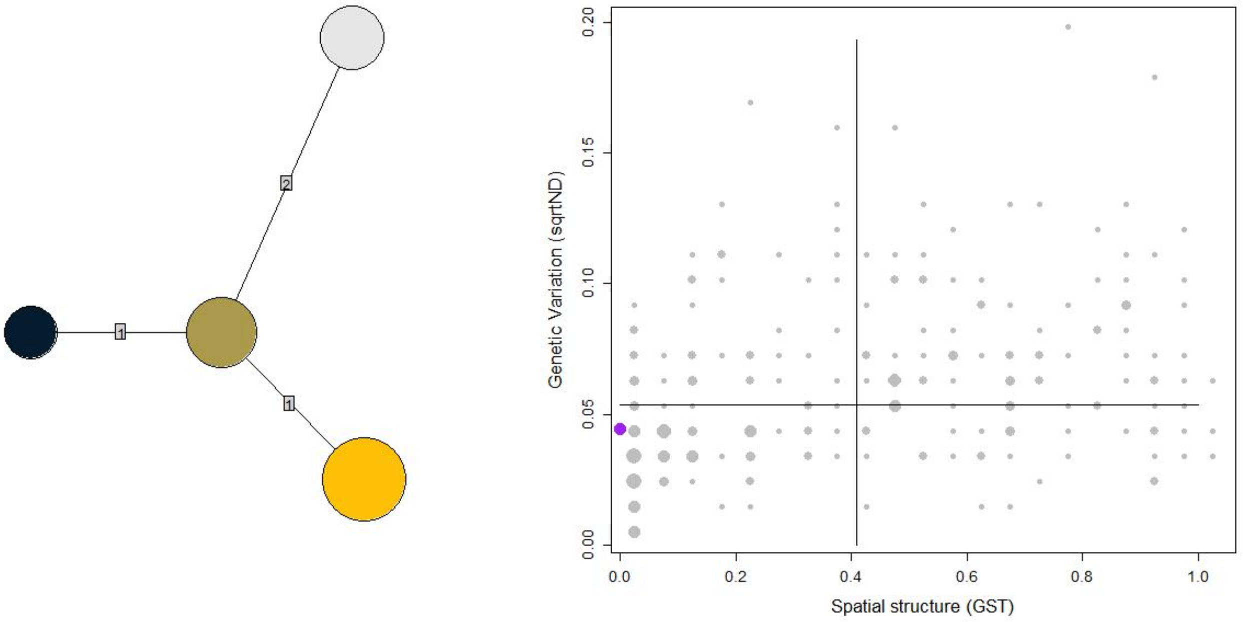

Figure 141: Haplotype network (left) of *Hesperia uncas* sequences > 599 bp with colours matching the PCoA colour space (above). The bubble plot for mt-DNA polymorphism (square root transformed nucleotide diversity) and spatial structure (GST) among all species in the atlas and values for *Hesperia uncas* (purple dot). The horizontal and vertical lines represent median values of nucleotide diversity and GST, respectively. Sequences > 599 bp= 12.

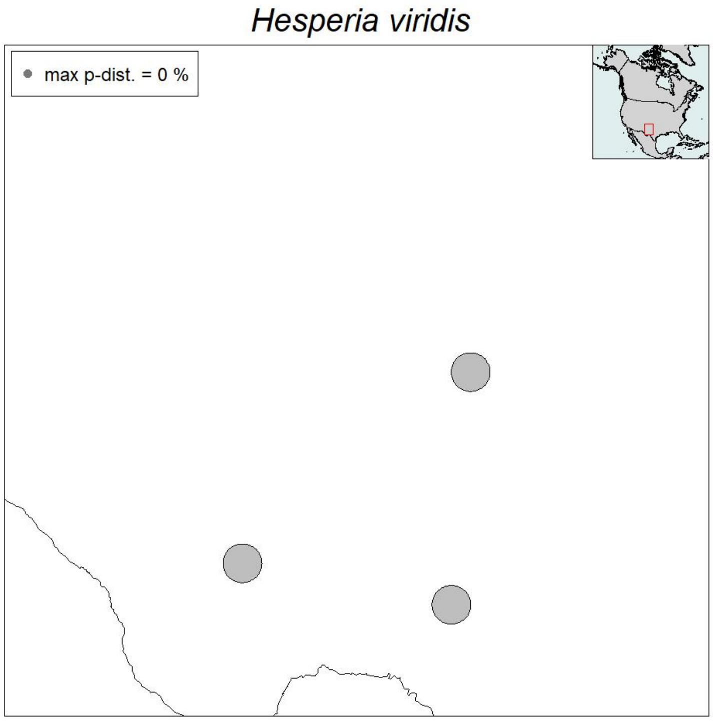

Figure 142: Map of *Hesperia viridis* showing the localities of the sequenced specimens. Nearby localities are grouped in pies. Due to the presence of a single haplotype PCoA projection was not done and a single grey colour was plotted on the map. Sequences= 3; Hap obs.= 1; Hap asympt.= NA; Hap % obs.= NA%; GST= NaN; DST= NaN; HD= NA; ND= NA; max p-dist= 0%.

Haplotype network analysis and bubble plot of *Hesperia viridis* were not possible. Sequences > 599 bp = 3.

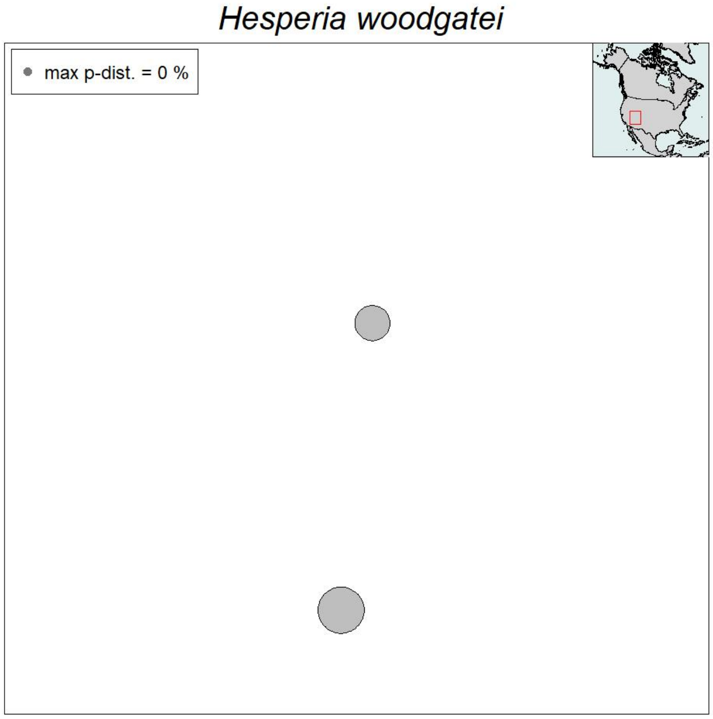

Figure 143: Map of *Hesperia woodgatei* showing the localities of the sequenced specimens. Nearby localities are grouped in pies. Due to the presence of a single haplotype PCoA projection was not done and a single grey colour was plotted on the map. Sequences= 4; Hap obs.= 1; Hap asympt.= NA; Hap % obs.= NA%; GST= NaN; DST= NaN; HD= NA; ND= NA; max p-dist= 0%.

Haplotype network analysis and bubble plot of *Hesperia woodgatei* were not possible. Sequences > 599 bp = 4.

Hesperia balcones

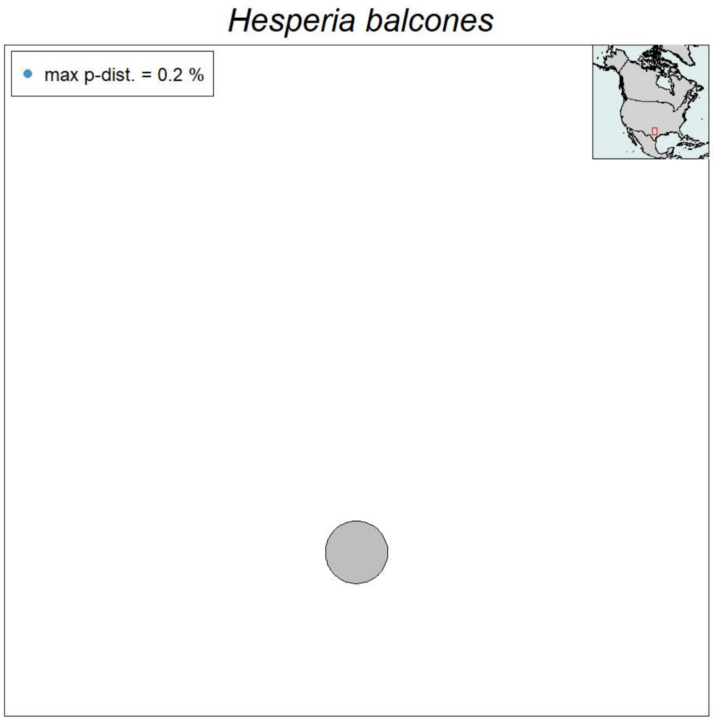

Figure 144: Map of *Hesperia balcones* showing the localities of the sequenced specimens. Nearby localities are grouped in pies. Due to the presence of a single haplotype PCoA projection was not done and a single grey colour was plotted on the map. Sequences= 2; Hap obs.= 2; Hap asympt.= NA; Hap % obs.= NA%; GST= NaN; DST= NaN; HD= NA; ND= NA; max p-dist= 0.2%.

Haplotype network analysis and bubble plot of *Hesperia balcones* were not possible. Sequences > 599 bp = 2.

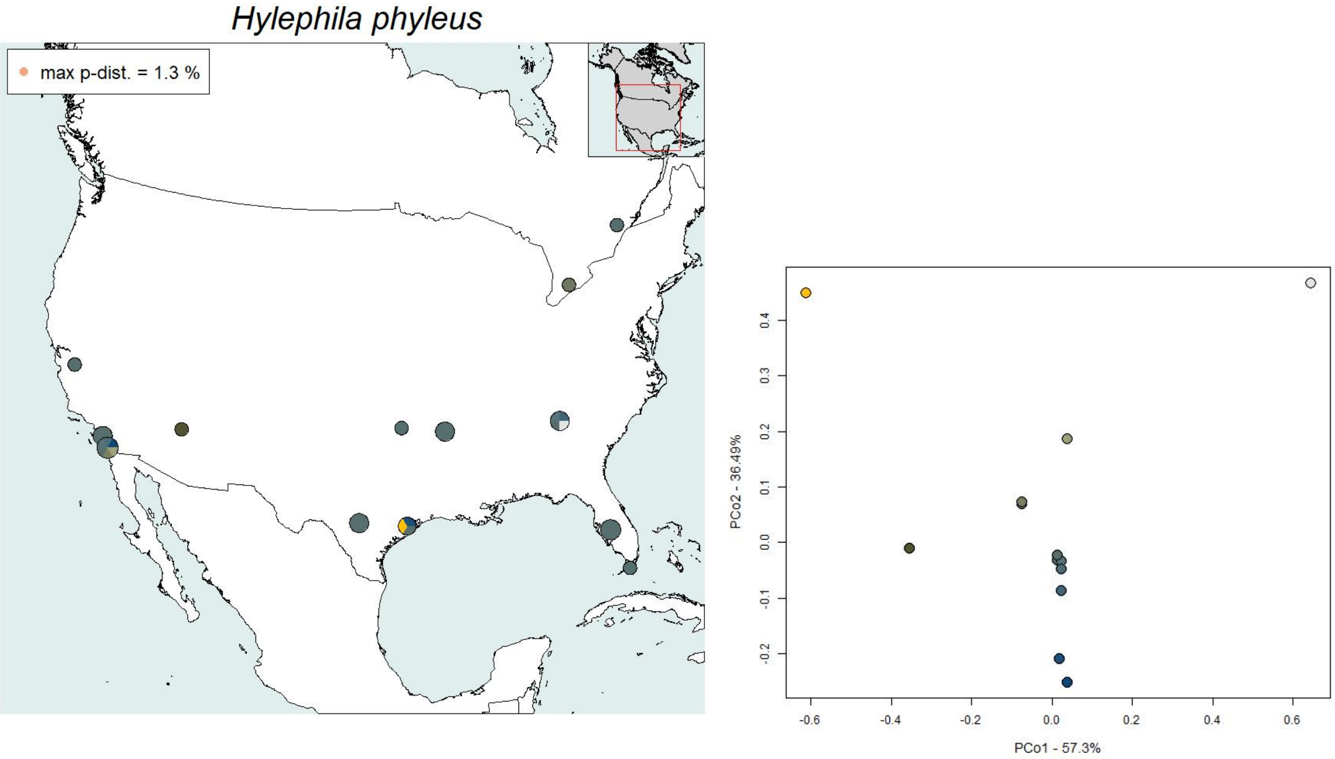

Figure 145 Map of *Hylephila phyleus* showing the localities of the sequenced specimens (left). Nearby localities are grouped in pies. Colours match the bidimensional colour space of the PCoA projection (right) of max p-dists among sequences (dots). Sequences= 36; Hap obs.= 8; Hap asympt.= 25.5; Hap % obs.= 31.4%; GST= 0.148; DST= 0.0002; HD= 0.398; ND= 0.0013; max p-dist= 1.3%.

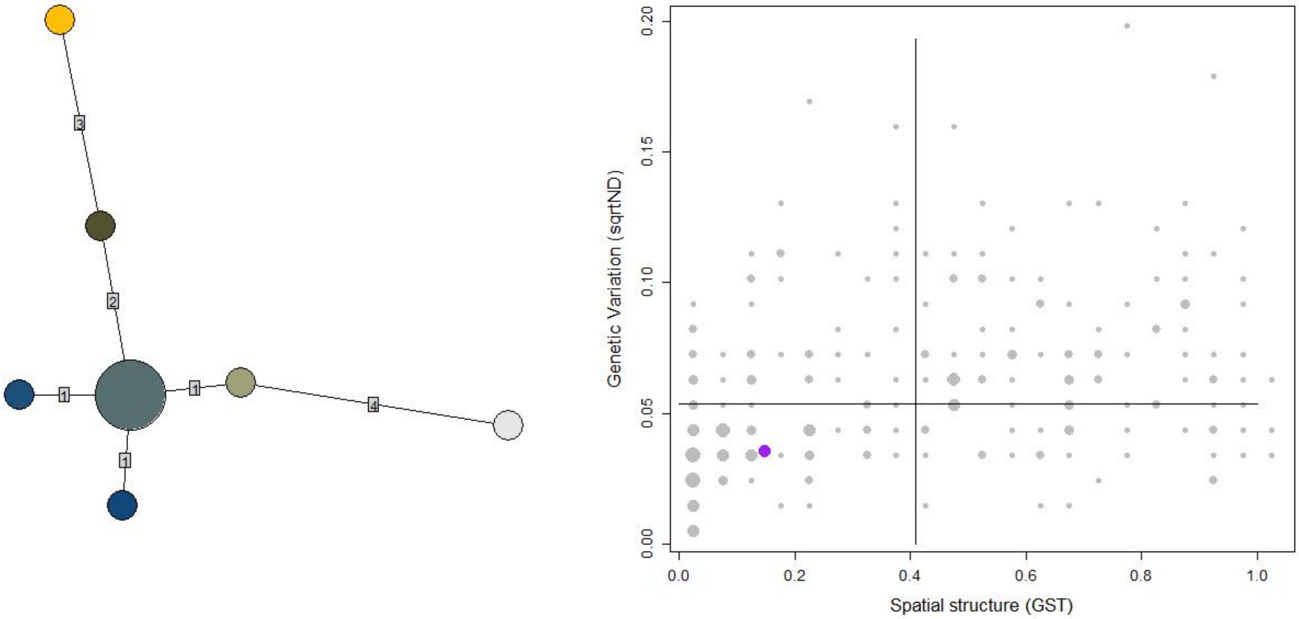

Figure 146: Haplotype network (left) of *Hylephila phyleus* sequences > 599 bp with colours matching the PCoA colour space (above). The bubble plot for mt-DNA polymorphism (square root transformed nucleotide diversity) and spatial structure (GST) among all species in the atlas and values for *Hylephila phyleus* (purple dot). The horizontal and vertical lines represent median values of nucleotide diversity and GST, respectively. Sequences > 599 bp= 34.

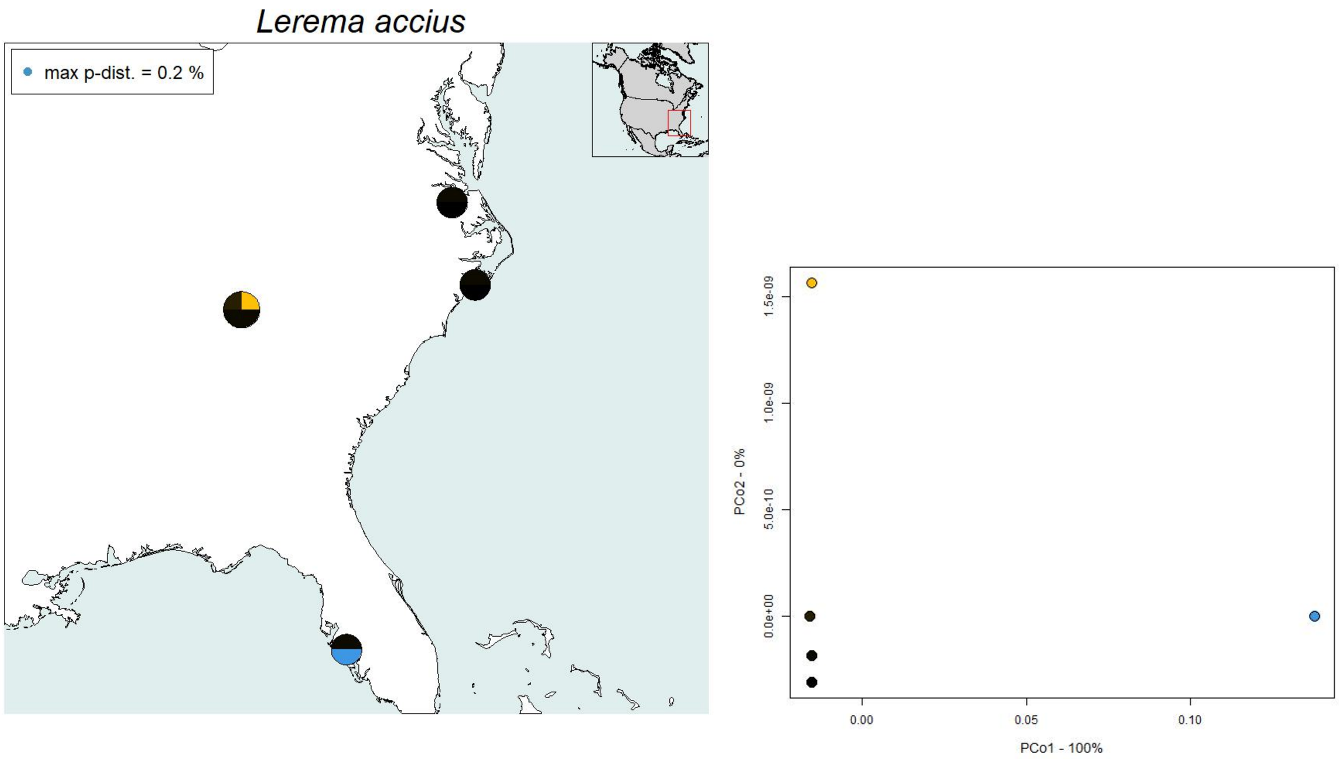

Figure 147 Map of *Lerema accius* showing the localities of the sequenced specimens (left). Nearby localities are grouped in pies. Colours match the bidimensional colour space of the PCoA projection (right) of max p-dists among sequences (dots). Sequences= 10; Hap obs.= 2; Hap asympt.= 2; Hap % obs.= 100%; GST= NaN; DST= NaN; HD= 0.2; ND= 0.0003; max p-dist= 0.2%.

Haplotype network analysis and bubble plot of *Lerema accius* were not possible. Sequences > 599 bp = 10.

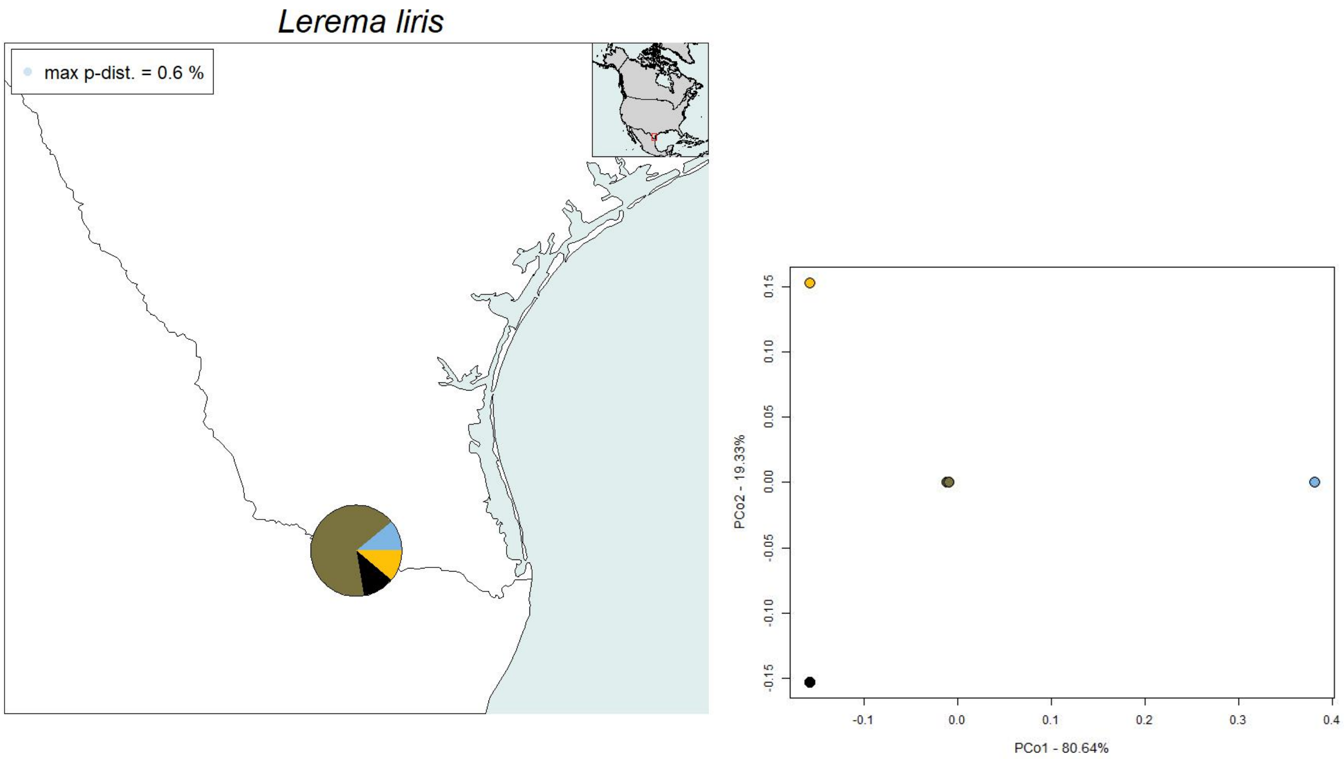

Figure 148 Map of *Lerema liris* showing the localities of the sequenced specimens (left). Nearby localities are grouped in pies. Colours match the bidimensional colour space of the PCoA projection (right) of max p-dists among sequences (dots). Sequences= 9; Hap obs.= 4; Hap asympt.= NA; Hap % obs.= NA%; GST= NaN; DST= NaN; HD= NA; ND= NA; max p-dist= 0.6%.

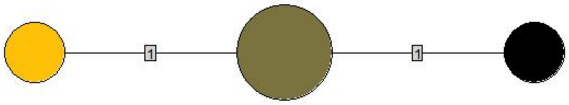

Figure 149: Haplotype network of *Lerema liris*. Sequences > 599 bp= 8.

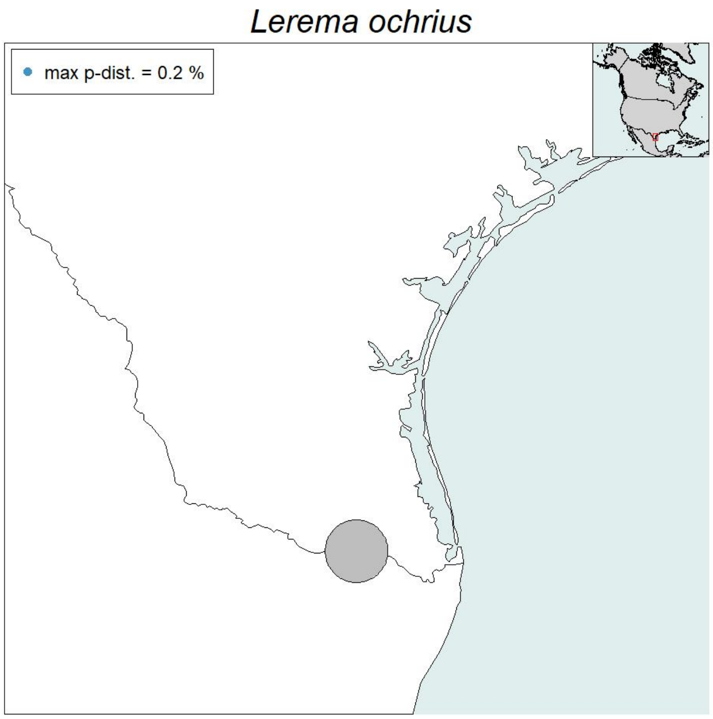

Figure 150: Map of *Lerema ochrius* showing the localities of the sequenced specimens. Nearby localities are grouped in pies. Due to the presence of a single haplotype PCoA projection was not done and a single grey colour was plotted on the map. Sequences= 2; Hap obs.= 2; Hap asympt.= NA; Hap % obs.= NA%; GST= NaN; DST= NaN; HD= NA; ND= NA; max p-dist= 0.2%.

Haplotype network analysis and bubble plot of *Lerema ochrius* were not possible. Sequences > 599 bp = 2.

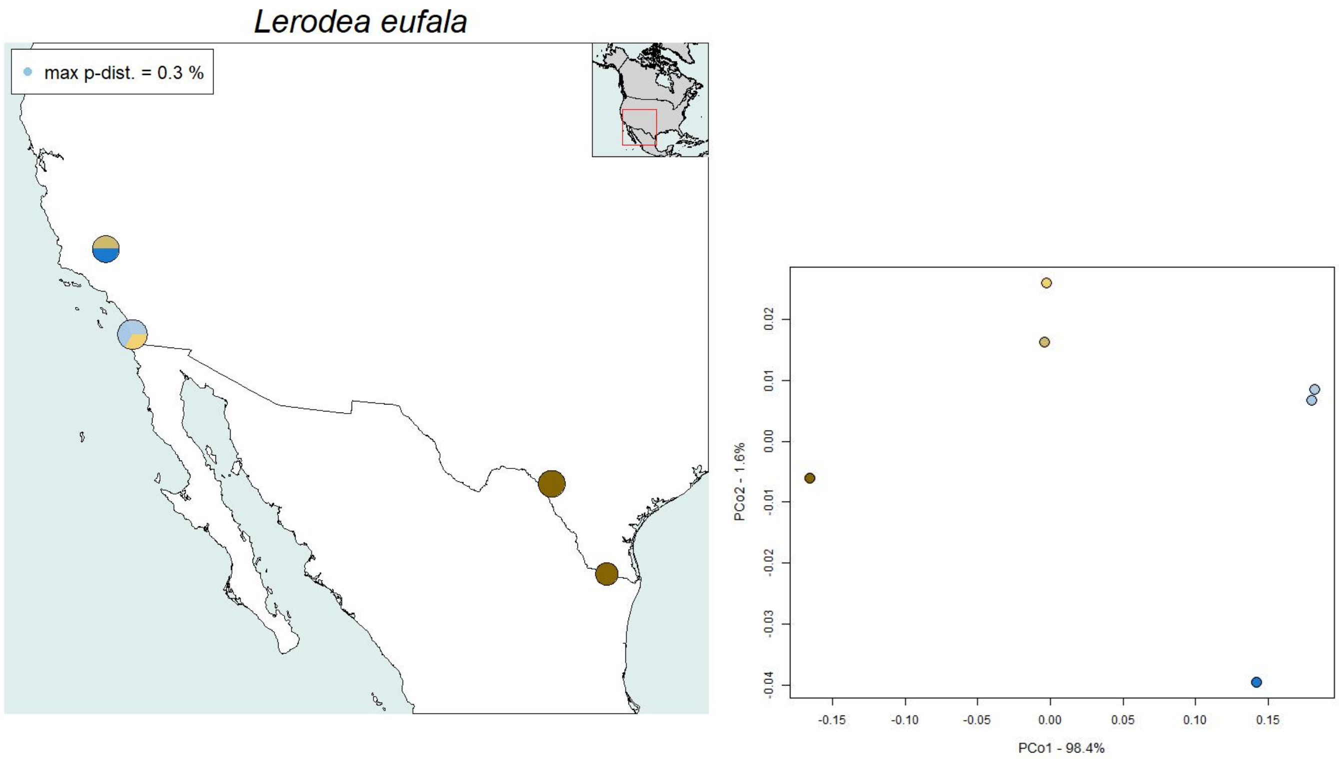

Figure 151 Map of *Lerodea eufala* showing the localities of the sequenced specimens (left). Nearby localities are grouped in pies. Colours match the bidimensional colour space of the PCoA projection (right) of max p-dists among sequences (dots). Sequences= 8; Hap obs.= 3; Hap asympt.= NA; Hap % obs.= NA%; GST= NaN; DST= NaN; HD= NA; ND= NA; max p-dist= 0.3%.

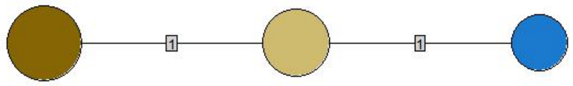

Figure 152: Haplotype network of *Lerodea eufala*. Sequences > 599 bp= 6.

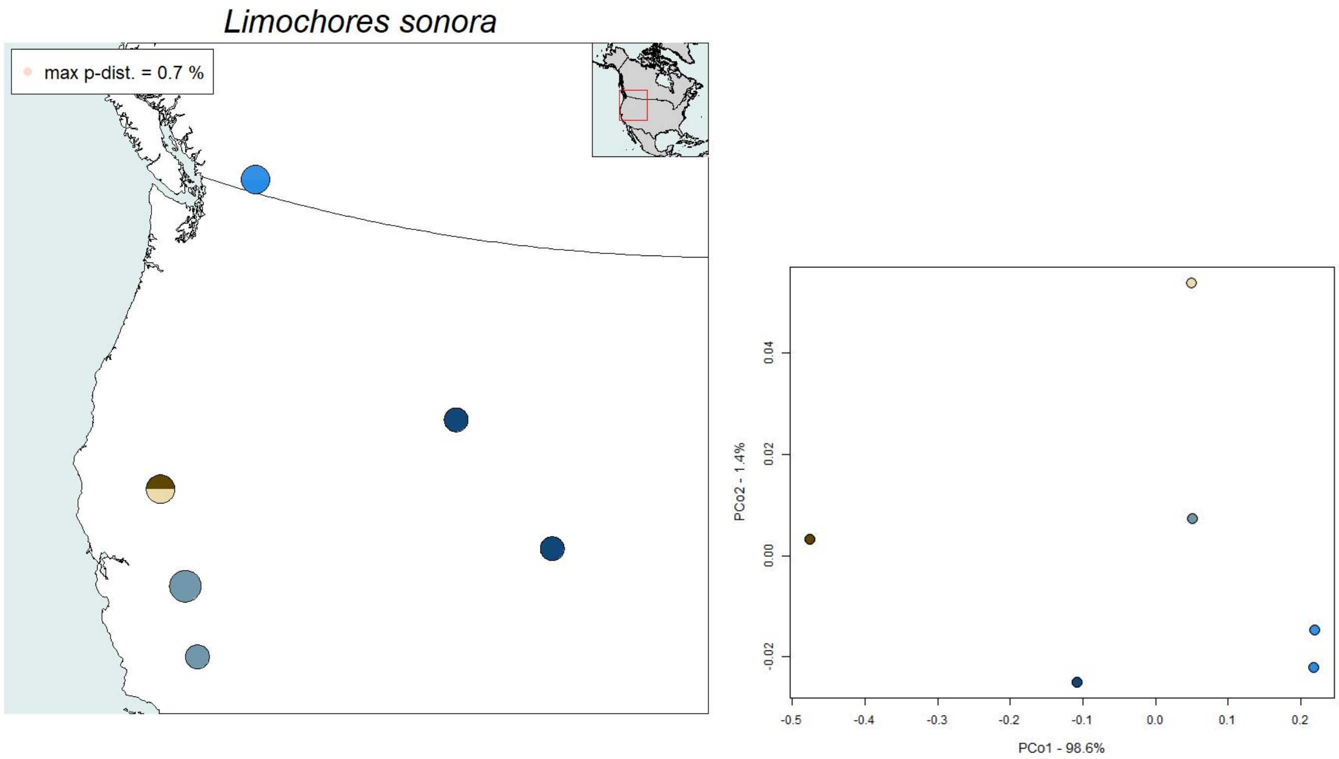

Figure 153 Map of *Limochores sonora* showing the localities of the sequenced specimens (left). Nearby localities are grouped in pies. Colours match the bidimensional colour space of the PCoA projection (right) of max p-dists among sequences (dots). Sequences= 10; Hap obs.= 4; Hap asympt.= 4.2; Hap % obs.= 94.7%; GST= NaN; DST= NaN; HD= 0.733; ND= 0.002; max p-dist= 0.7%.

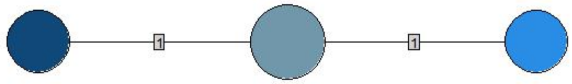

Figure 154: Haplotype network of *Limochores sonora*. Sequences > 599 bp= 8.

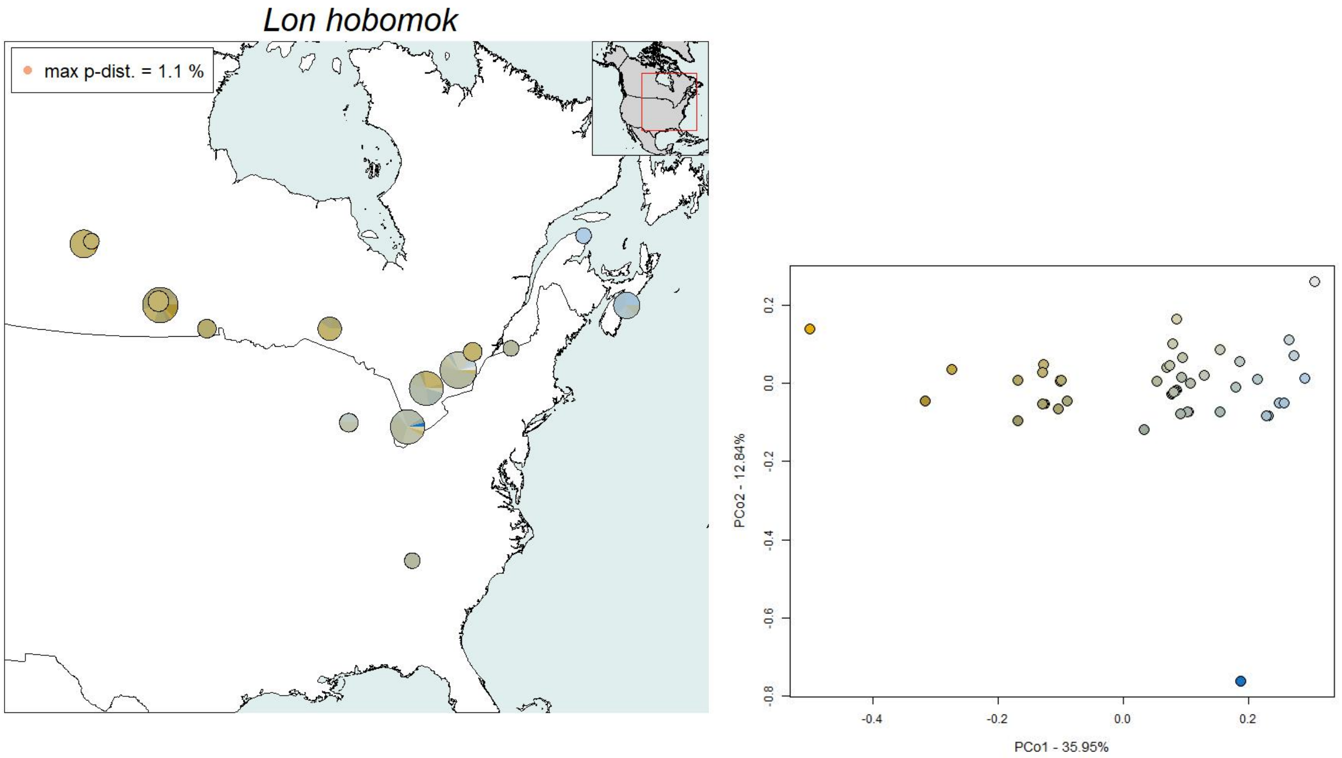

Figure 155 Map of *Lon hobomok* showing the localities of the sequenced specimens (left). Nearby localities are grouped in pies. Colours match the bidimensional colour space of the PCoA projection (right) of max p-dists among sequences (dots). Sequences= 136; Hap obs.= 29; Hap asympt.= 56.9; Hap % obs.= 51%; GST= 0.435; DST= 0.0009; HD= 0.845; ND= 0.002; max p-dist= 1.1%.

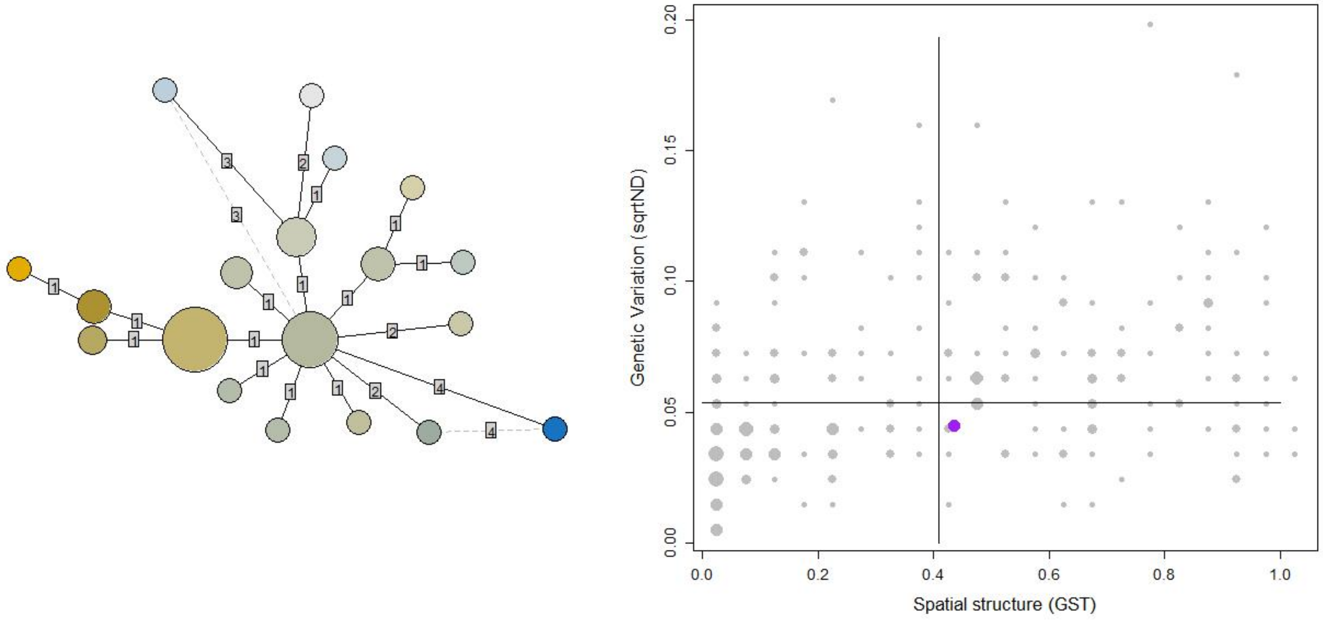

Figure 156: Haplotype network (left) of *Lon hobomok* sequences > 599 bp with colours matching the PCoA colour space (above). The bubble plot for mt-DNA polymorphism (square root transformed nucleotide diversity) and spatial structure (GST) among all species in the atlas and values for *Lon hobomok* (purple dot). The horizontal and vertical lines represent median values of nucleotide diversity and GST, respectively. Sequences > 599 bp= 107.

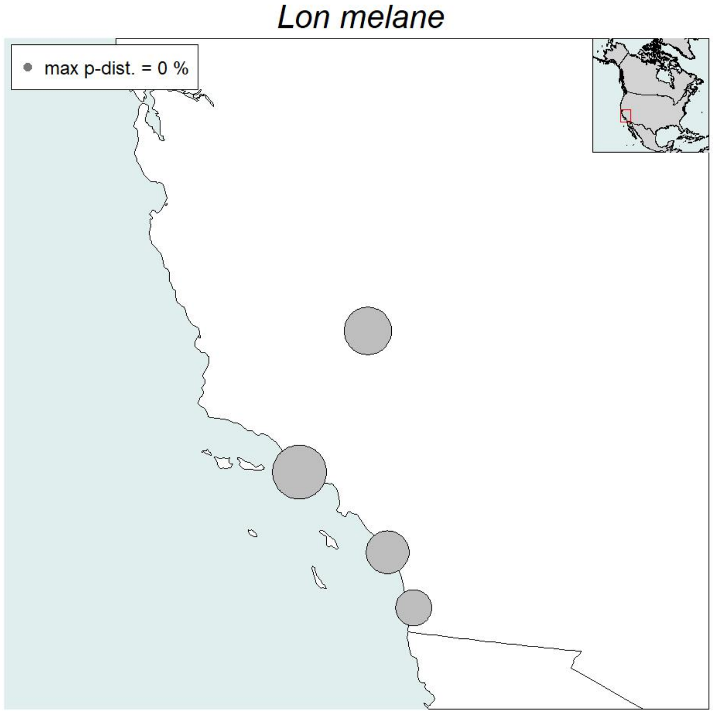

Figure 157: Map of *Lon melane* showing the localities of the sequenced specimens. Nearby localities are grouped in pies. Due to the presence of a single haplotype PCoA projection was not done and a single grey colour was plotted on the map. Sequences= 11; Hap obs.= 1; Hap asympt.= NA; Hap % obs.= NA%; GST= 0; DST= 0; HD= 0; ND= 0; max p-dist= 0%.

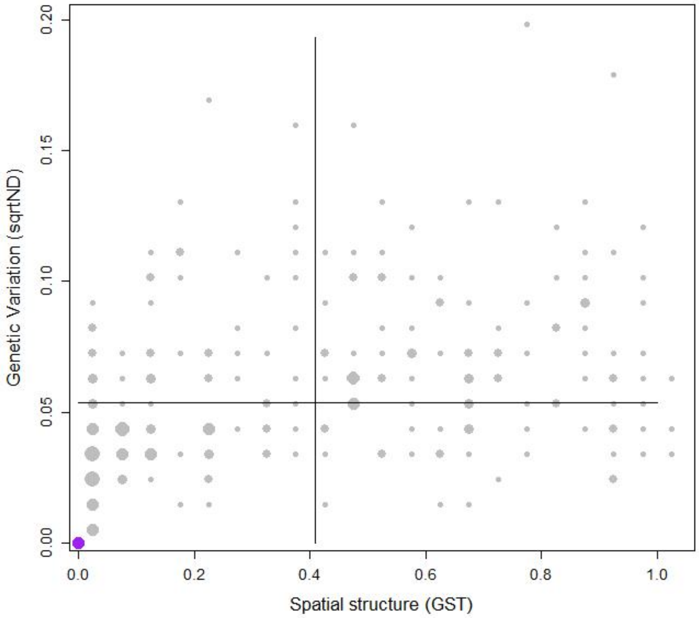

Figure 158: The bubble plot for mt-DNA polymorphism (square root transformed nucleotide diversity) and spatial structuree (GST) among all species in the atlas and values for *Lon melane* (purple dot). The horizontal and vertical lines represent median values of nucleotide diversity and GST, respectively. Haplotype network analysis was not possible. Sequences > 599 bp = 11.

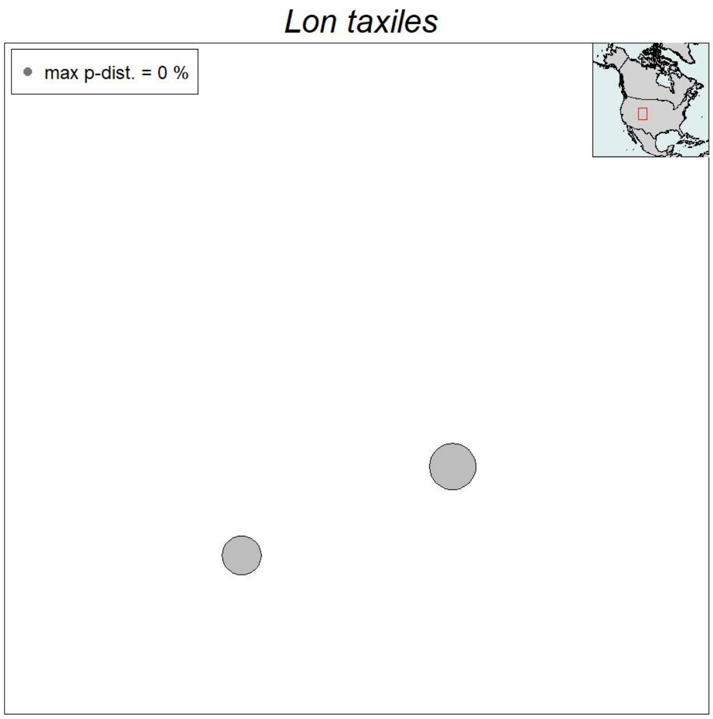

Figure 159: Map of *Lon taxiles* showing the localities of the sequenced specimens. Nearby localities are grouped in pies. Due to the presence of a single haplotype PCoA projection was not done and a single grey colour was plotted on the map. Sequences= 3; Hap obs.= 1; Hap asympt.= NA; Hap % obs.= NA%; GST= NaN; DST= NaN; HD= NA; ND= NA; max p-dist= 0%.

Haplotype network analysis and bubble plot of *Lon taxiles* were not possible. Sequences > 599 bp = 3.

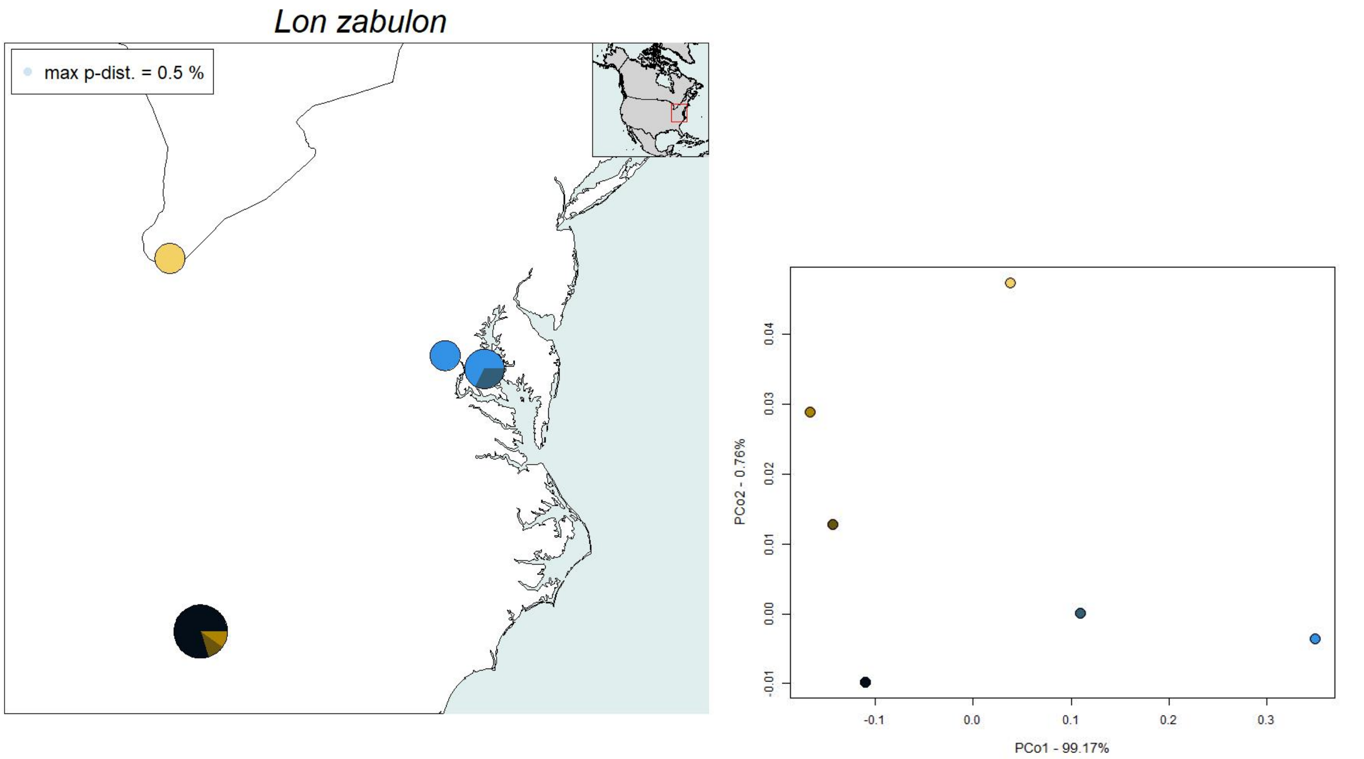

Figure 160 Map of *Lon zabulon* showing the localities of the sequenced specimens (left). Nearby localities are grouped in pies. Colours match the bidimensional colour space of the PCoA projection (right) of max p-dists among sequences (dots). Sequences= 15; Hap obs.= 3; Hap asympt.= 3; Hap % obs.= 100%; GST= 0.979; DST= 0.0018; HD= 0.533; ND= 0.0018; max p-dist= 0.5%.

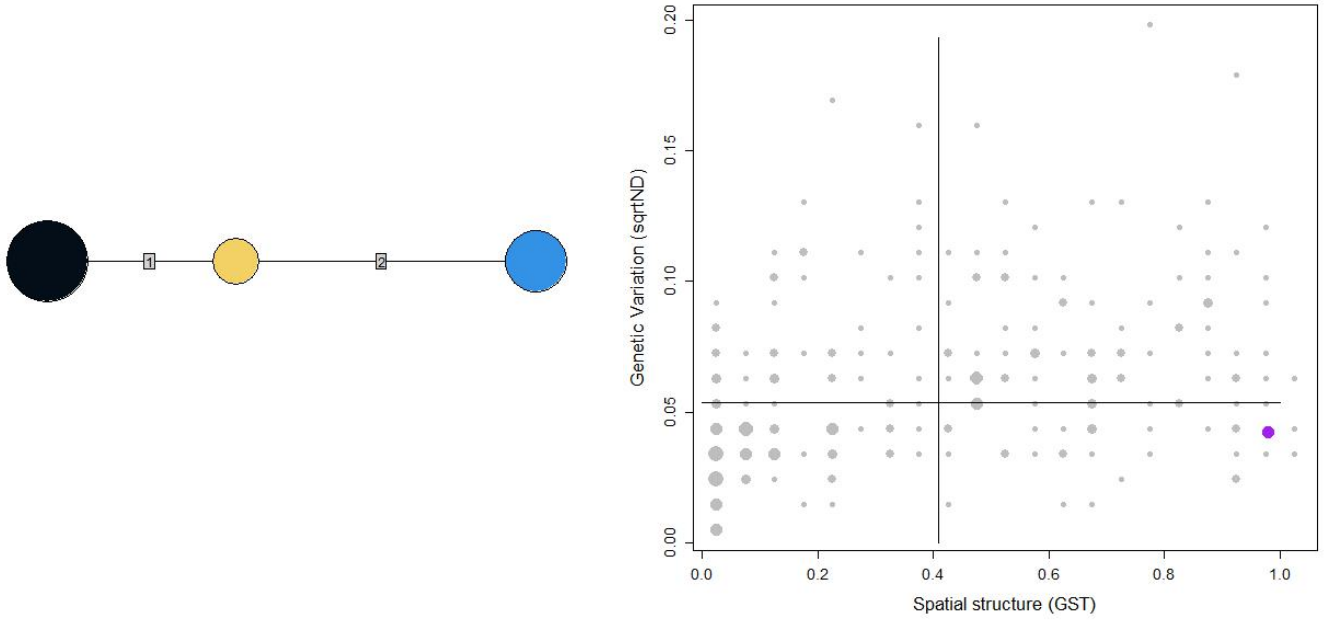

Figure 161: Haplotype network (left) of *Lon zabulon* sequences > 599 bp with colours matching the PCoA colour space (above). The bubble plot for mt-DNA polymorphism (square root transformed nucleotide diversity) and spatial structure (GST) among all species in the atlas and values for *Lon zabulon* (purple dot). The horizontal and vertical lines represent median values of nucleotide diversity and GST, respectively. Sequences > 599 bp= 13.

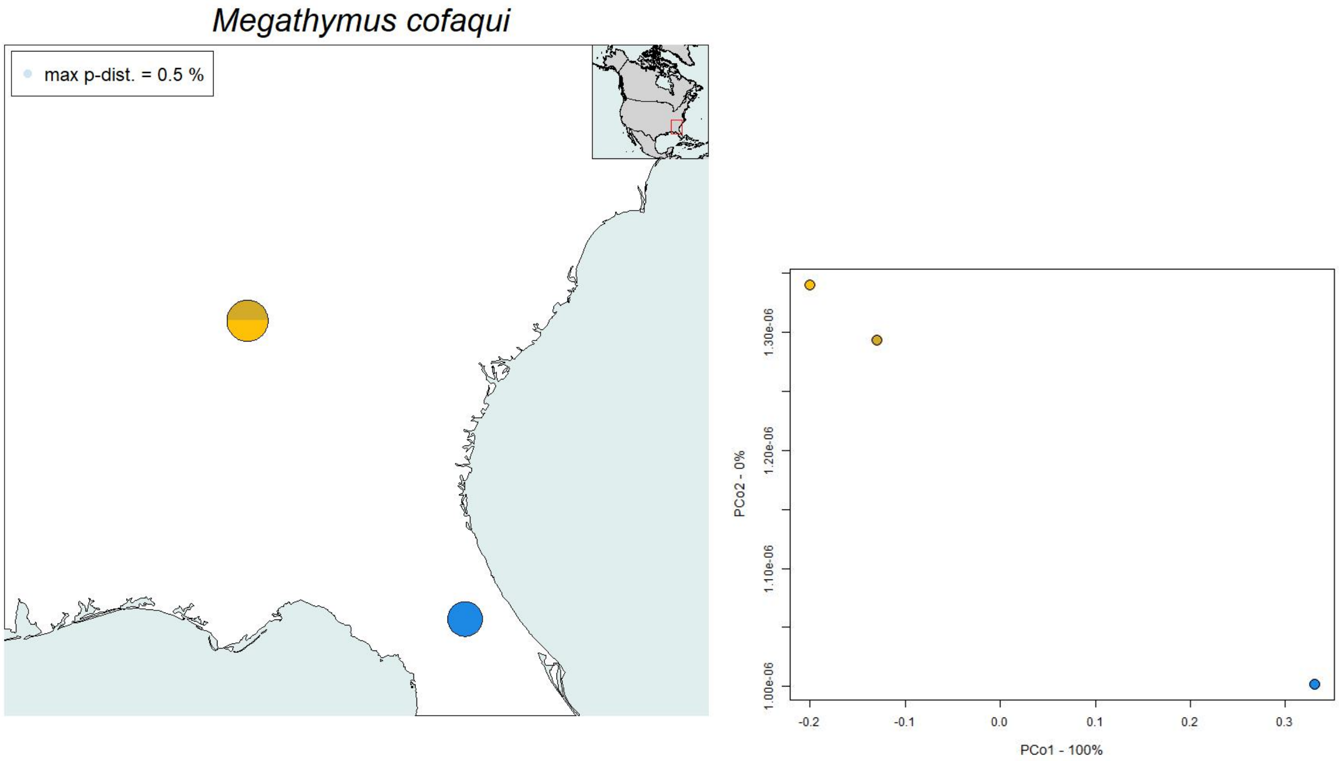

Figure 162 Map of *Megathymus cofaqui* showing the localities of the sequenced specimens (left). Nearby localities are grouped in pies. Colours match the bidimensional colour space of the PCoA projection (right) of max p-dists among sequences (dots). Sequences= 3; Hap obs.= 3; Hap asympt.= NA; Hap % obs.= NA%; GST= NaN; DST= NaN; HD= NA; ND= NA; max p-dist= 0.5%.

Haplotype network analysis and bubble plot of *Megathymus cofaqui* were not possible. Sequences > 599 bp = 2.

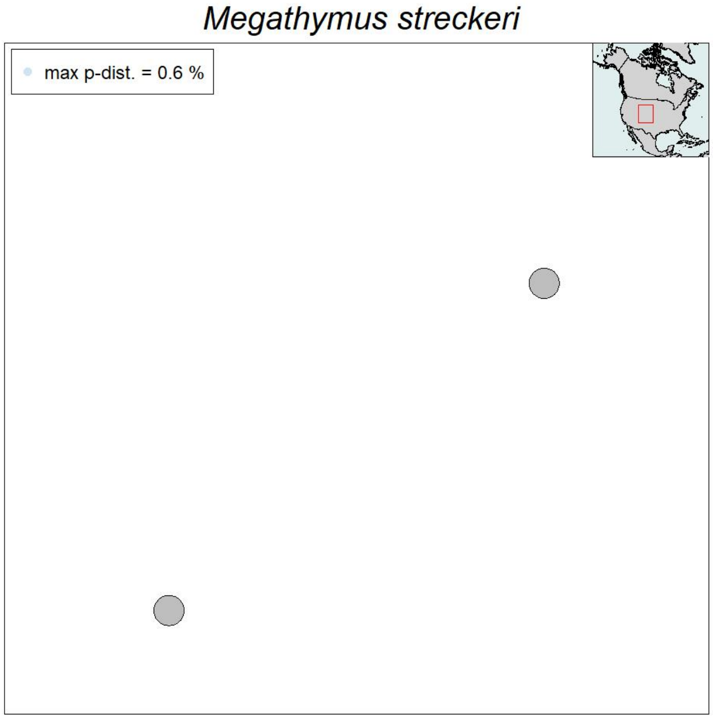

Figure 163: Map of *Megathymus streckeri* showing the localities of the sequenced specimens. Nearby localities are grouped in pies. Due to the presence of a single haplotype PCoA projection was not done and a single grey colour was plotted on the map. Sequences= 2; Hap obs.= 2; Hap asympt.= NA; Hap % obs.= NA%; GST= NaN; DST= NaN; HD= NA; ND= NA; max p-dist= 0.6%.

Haplotype network analysis and bubble plot of *Megathymus streckeri* were not possible. Sequences > 599 bp = 2.

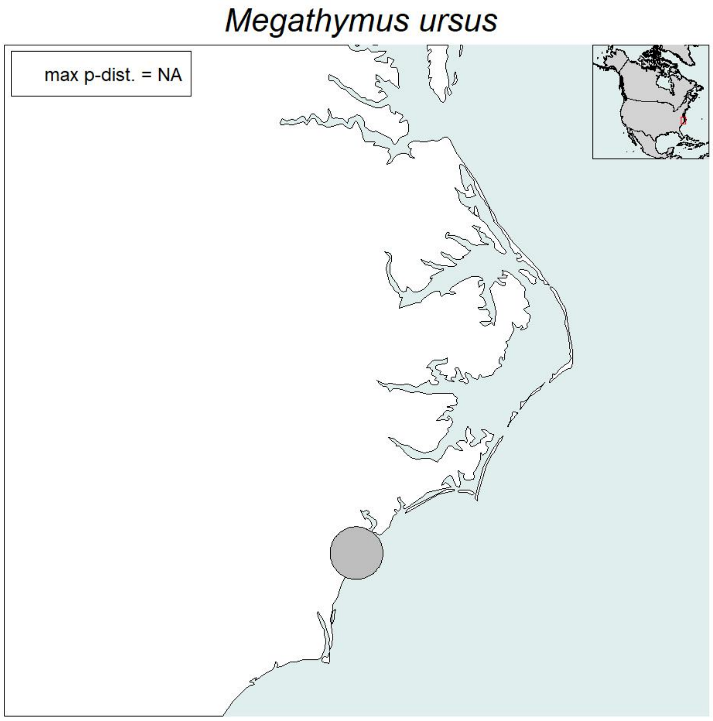

Figure 164: Map of *Megathymus ursus* showing the localities of the sequenced specimens. Nearby localities are grouped in pies. Due to the presence of a single haplotype PCoA projection was not done and a single grey colour was plotted on the map. Sequences= 1; Hap obs.= NA; Hap asympt.= NA; Hap % obs.= NA; GST= NaN; DST= NaN; HD= NA; ND= NA; max p-dist= NA.

Haplotype network analysis and bubble plot of *Megathymus ursus* were not possible. Sequences > 599 bp = 0.

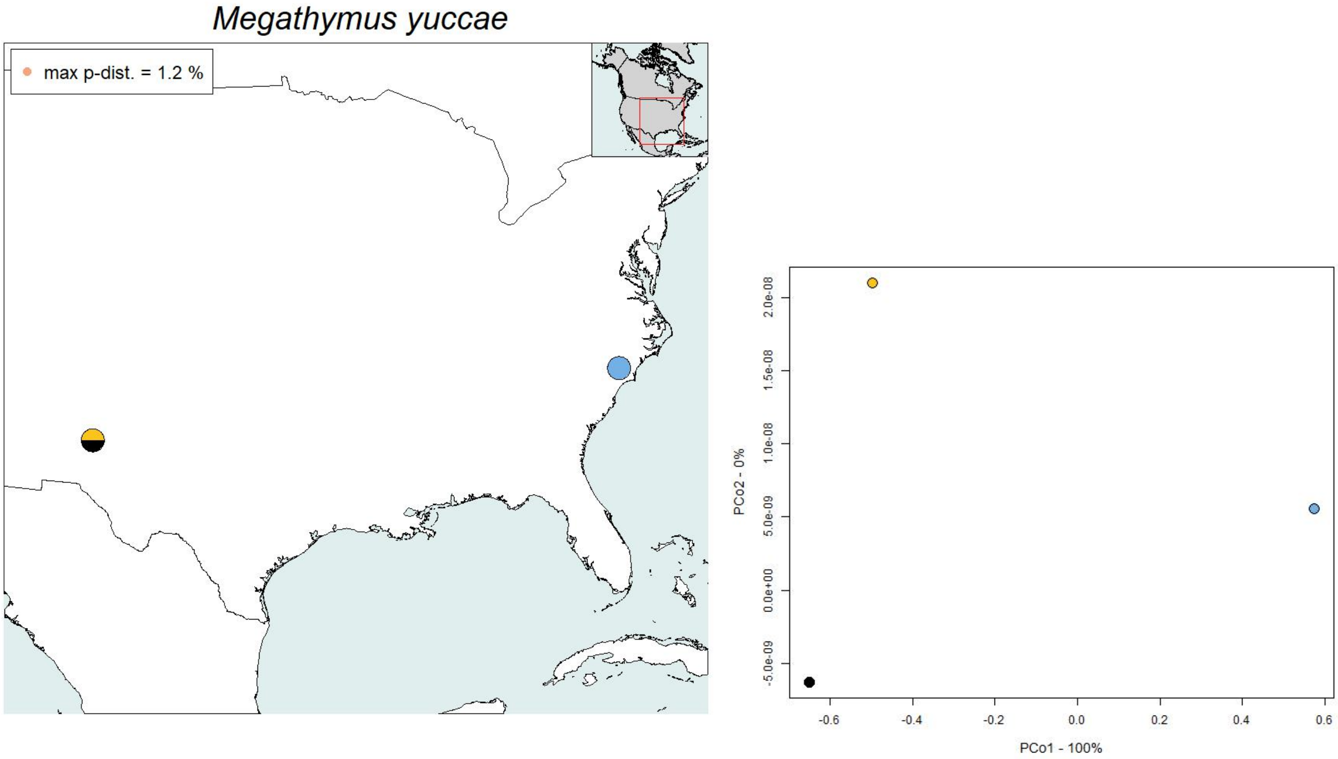

Figure 165 Map of *Megathymus yuccae* showing the localities of the sequenced specimens (left). Nearby localities are grouped in pies. Colours match the bidimensional colour space of the PCoA projection (right) of max p-dists among sequences (dots). Sequences= 4; Hap obs.= 3; Hap asympt.= NA; Hap % obs.= NA%; GST= NaN; DST= NaN; HD= NA; ND= NA; max p-dist= 1.2%.

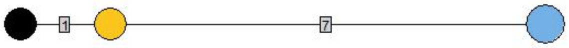

Figure 166: Haplotype network of *Megathymus yuccae*. Sequences > 599 bp= 4.

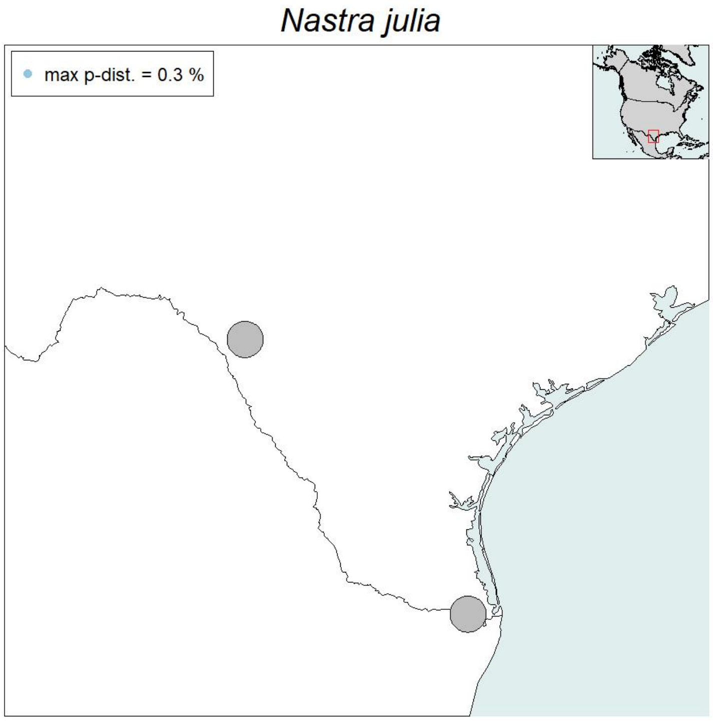

Figure 167: Map of *Nastra julia* showing the localities of the sequenced specimens. Nearby localities are grouped in pies. Due to the presence of a single haplotype PCoA projection was not done and a single grey colour was plotted on the map. Sequences= 2; Hap obs.= 2; Hap asympt.= NA; Hap % obs.= NA%; GST= NaN; DST= NaN; HD= NA; ND= NA; max p-dist= 0.3%.

Haplotype network analysis and bubble plot of *Nastra julia* were not possible. Sequences > 599 bp = 2.

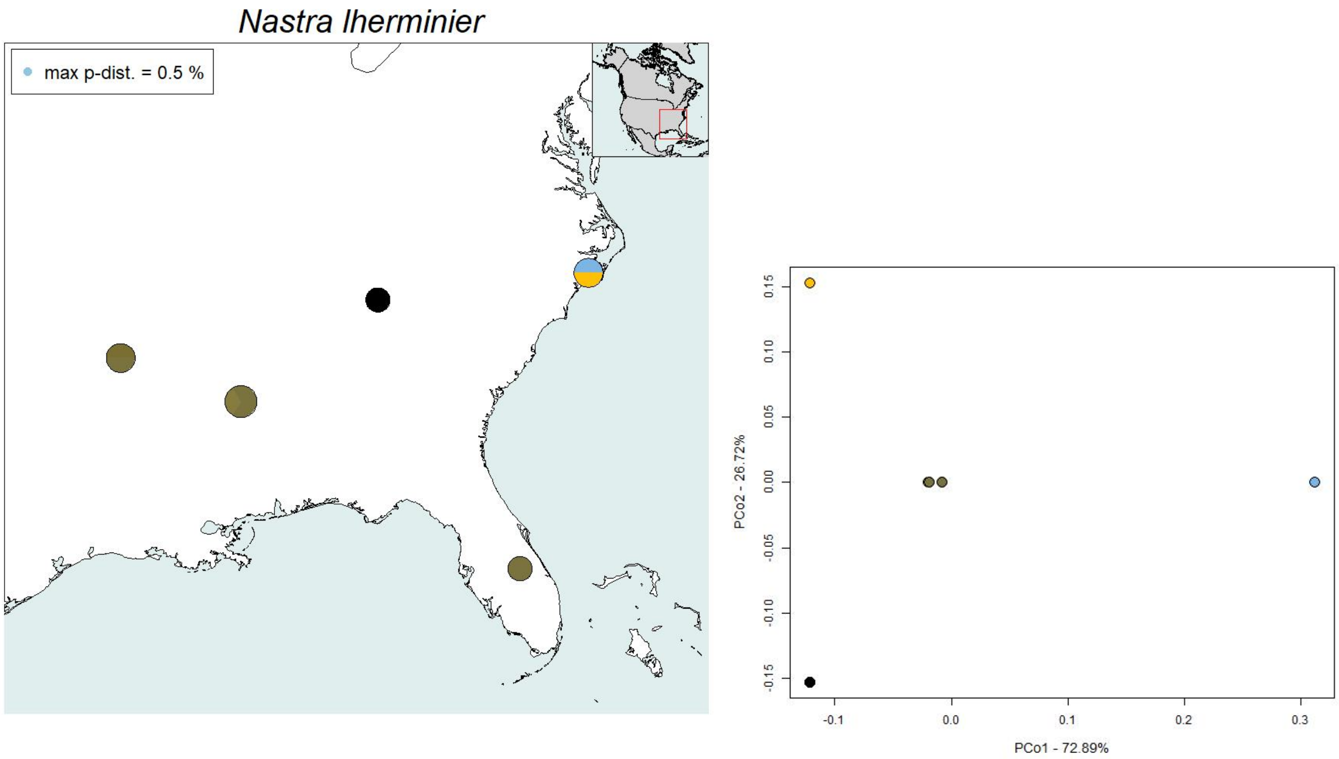

Figure 168 Map of *Nastra lherminier* showing the localities of the sequenced specimens (left). Nearby localities are grouped in pies. Colours match the bidimensional colour space of the PCoA projection (right) of max p-dists among sequences (dots). Sequences= 9; Hap obs.= 4; Hap asympt.= NA; Hap % obs.= NA%; GST= NaN; DST= NaN; HD= NA; ND= NA; max p-dist= 0.5%.

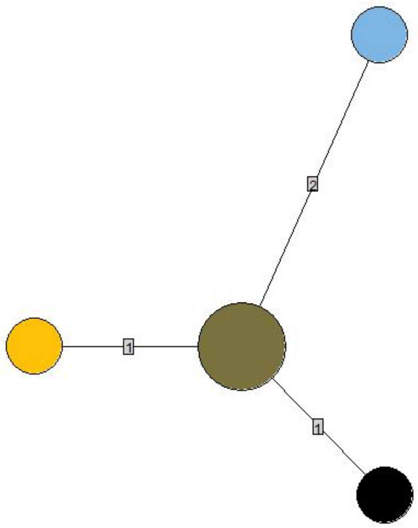

Figure 169: Haplotype network of *Nastra lherminier*. Sequences > 599 bp= 9.

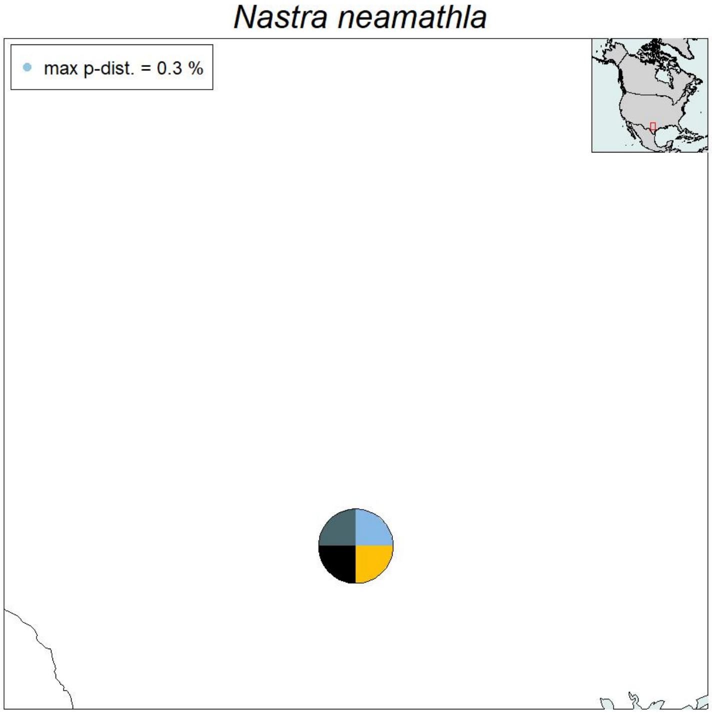

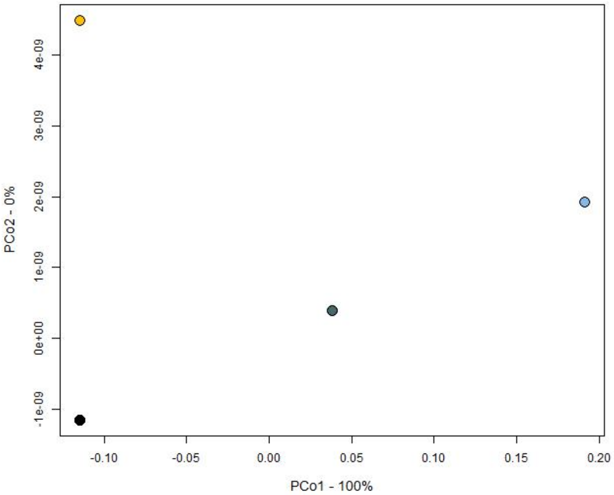

Figure 170 Map of *Nastra neamathla* showing the localities of the sequenced specimens (left). Nearby localities are grouped in pies. Colours match the bidimensional colour space of the PCoA projection (right) of max p-dists among sequences (dots). Sequences= 4; Hap obs.= 3; Hap asympt.= NA; Hap % obs.= NA%; GST= NaN; DST= NaN; HD= NA; ND= NA; max p-dist= 0.3%.

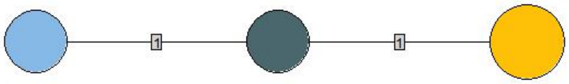

Figure 171: Haplotype network of *Nastra neamathla*. Sequences > 599 bp= 4.

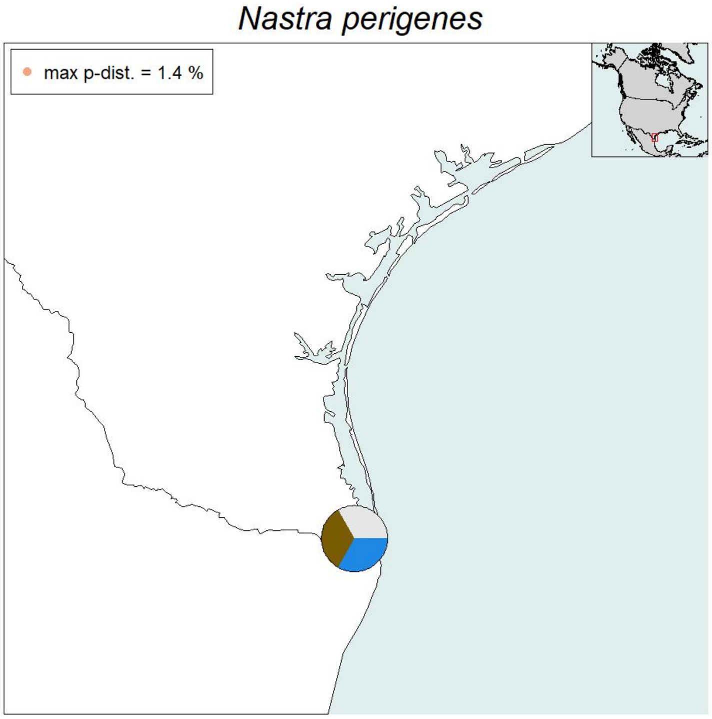

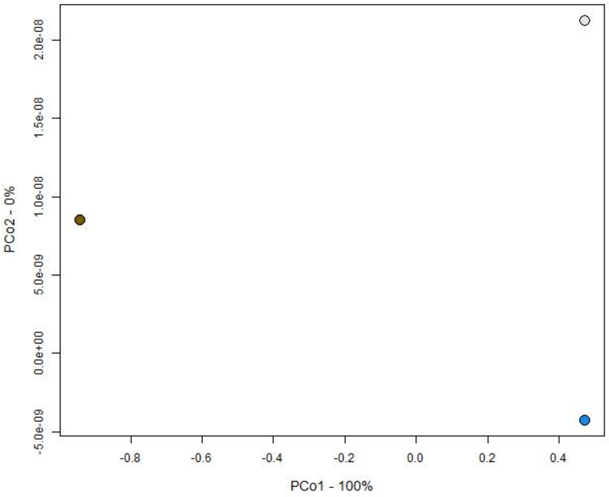

Figure 172 Map of *Nastra perigenes* showing the localities of the sequenced specimens (left). Nearby localities are grouped in pies. Colours match the bidimensional colour space of the PCoA projection (right) of max p-dists among sequences (dots). Sequences= 3; Hap obs.= 2; Hap asympt.= NA; Hap % obs.= NA%; GST= NaN; DST= NaN; HD= NA; ND= NA; max p-dist= 1.4%.

Haplotype network analysis and bubble plot of *Nastra perigenes* were not possible. Sequences > 599 bp = 2.

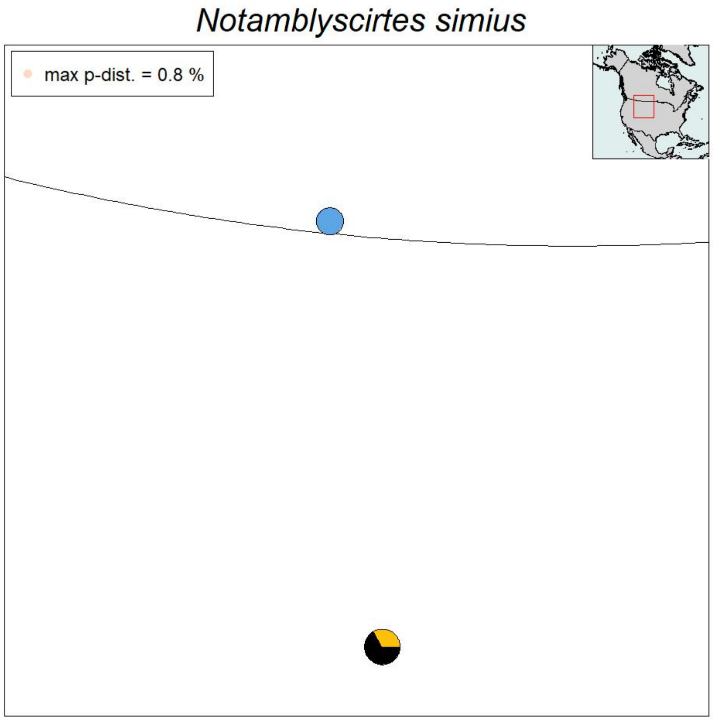

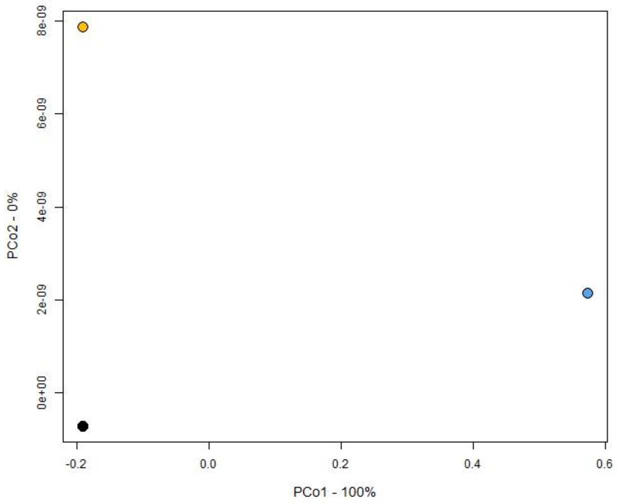

Figure 173 Map of *Notamblyscirtes simius* showing the localities of the sequenced specimens (left). Nearby localities are grouped in pies. Colours match the bidimensional colour space of the PCoA projection (right) of max p-dists among sequences (dots). Sequences= 4; Hap obs.= 2; Hap asympt.= NA; Hap % obs.= NA%; GST= NaN; DST= NaN; HD= NA; ND= NA; max p-dist= 0.8%.

Haplotype network analysis and bubble plot of *Notamblyscirtes simius* were not possible. Sequences > 599 bp = 4.

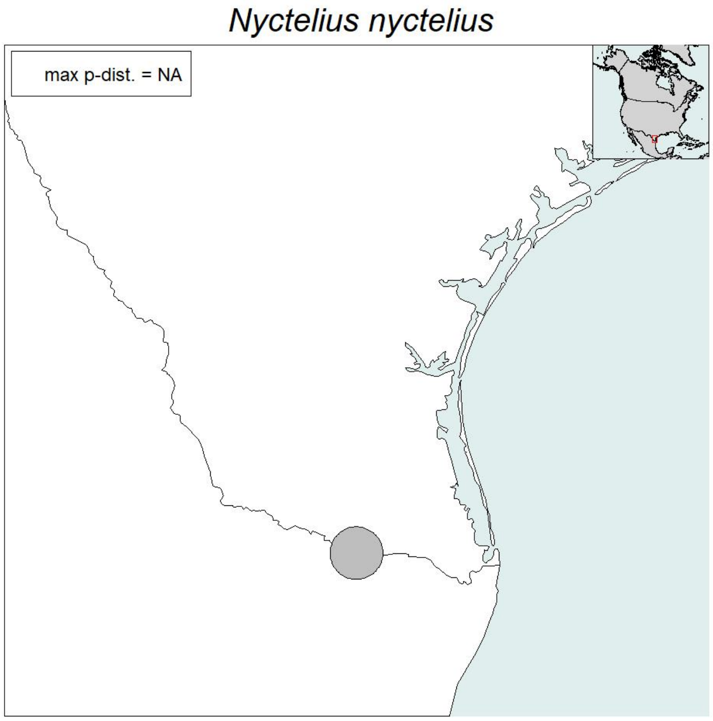

Figure 174: Map of *Nyctelius nyctelius* showing the localities of the sequenced specimens. Nearby localities are grouped in pies. Due to the presence of a single haplotype PCoA projection was not done and a single grey colour was plotted on the map. Sequences= 1; Hap obs.= NA; Hap asympt.= NA; Hap % obs.= NA; GST= NaN; DST= NaN; HD= NA; ND= NA; max p-dist= NA.

Haplotype network analysis and bubble plot of *Nyctelius nyctelius* were not possible. Sequences > 599 bp = 1.

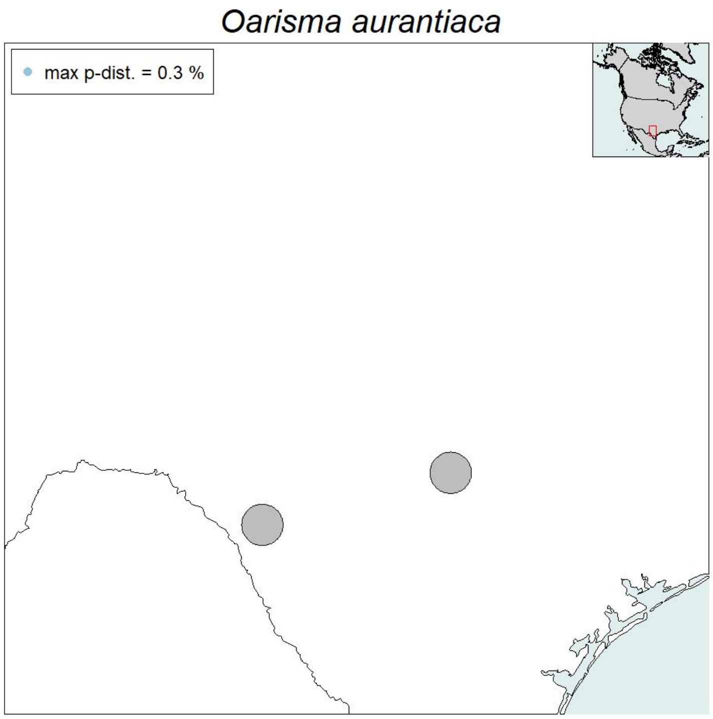

Figure 175: Map of *Oarisma aurantiaca* showing the localities of the sequenced specimens. Nearby localities are grouped in pies. Due to the presence of a single haplotype PCoA projection was not done and a single grey colour was plotted on the map. Sequences= 2; Hap obs.= 2; Hap asympt.= NA; Hap % obs.= NA%; GST= NaN; DST= NaN; HD= NA; ND= NA; max p-dist= 0.3%.

Haplotype network analysis and bubble plot of *Oarisma aurantiaca* were not possible. Sequences > 599 bp = 2.

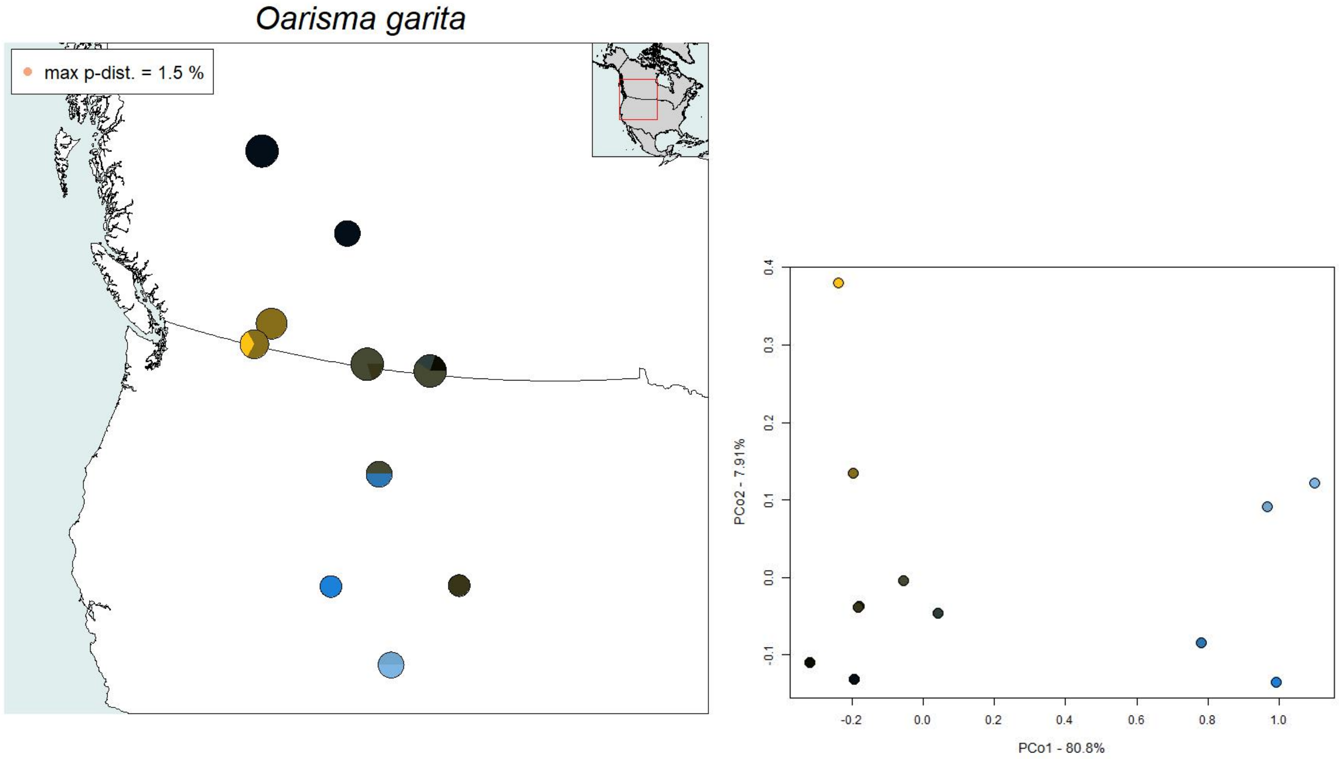

Figure 176 Map of *Oarisma garita* showing the localities of the sequenced specimens (left). Nearby localities are grouped in pies. Colours match the bidimensional colour space of the PCoA projection (right) of max p-dists among sequences (dots). Sequences= 30; Hap obs.= 12; Hap asympt.= 46.8; Hap % obs.= 25.6%; GST= 0.655; DST= 0.0013; HD= 0.853; ND= 0.0042; max p-dist= 1.5%.

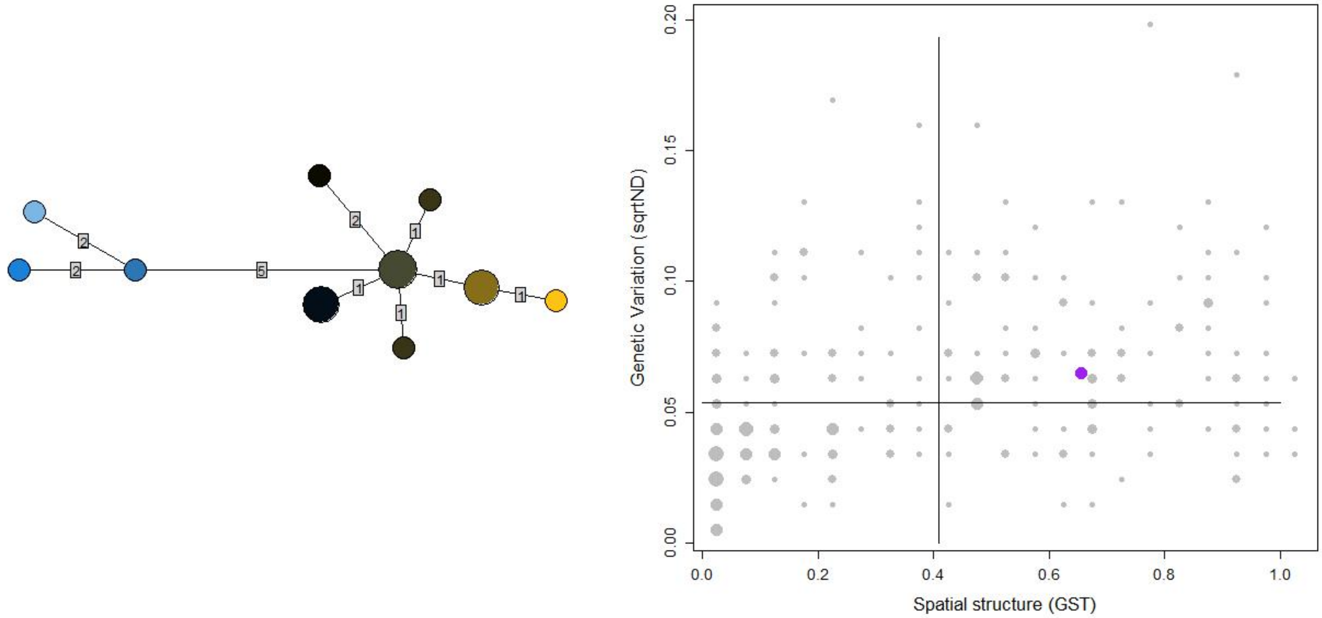

Figure 177: Haplotype network (left) of *Oarisma garita* sequences > 599 bp with colours matching the PCoA colour space (above). The bubble plot for mt-DNA polymorphism (square root transformed nucleotide diversity) and spatial structure (GST) among all species in the atlas and values for *Oarisma garita* (purple dot). The horizontal and vertical lines represent median values of nucleotide diversity and GST, respectively. Sequences > 599 bp= 28.

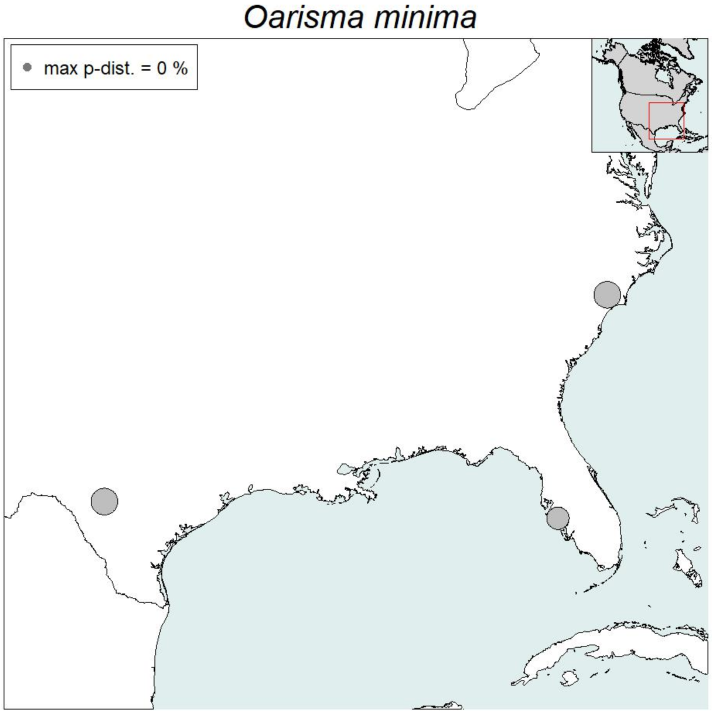

Figure 178: Map of *Oarisma minima* showing the localities of the sequenced specimens. Nearby localities are grouped in pies. Due to the presence of a single haplotype PCoA projection was not done and a single grey colour was plotted on the map. Sequences= 5; Hap obs.= 1; Hap asympt.= NA; Hap % obs.= NA%; GST= NaN; DST= NaN; HD= NA; ND= NA; max p-dist= 0%.

Haplotype network analysis and bubble plot of *Oarisma minima* were not possible. Sequences > 599 bp = 4.

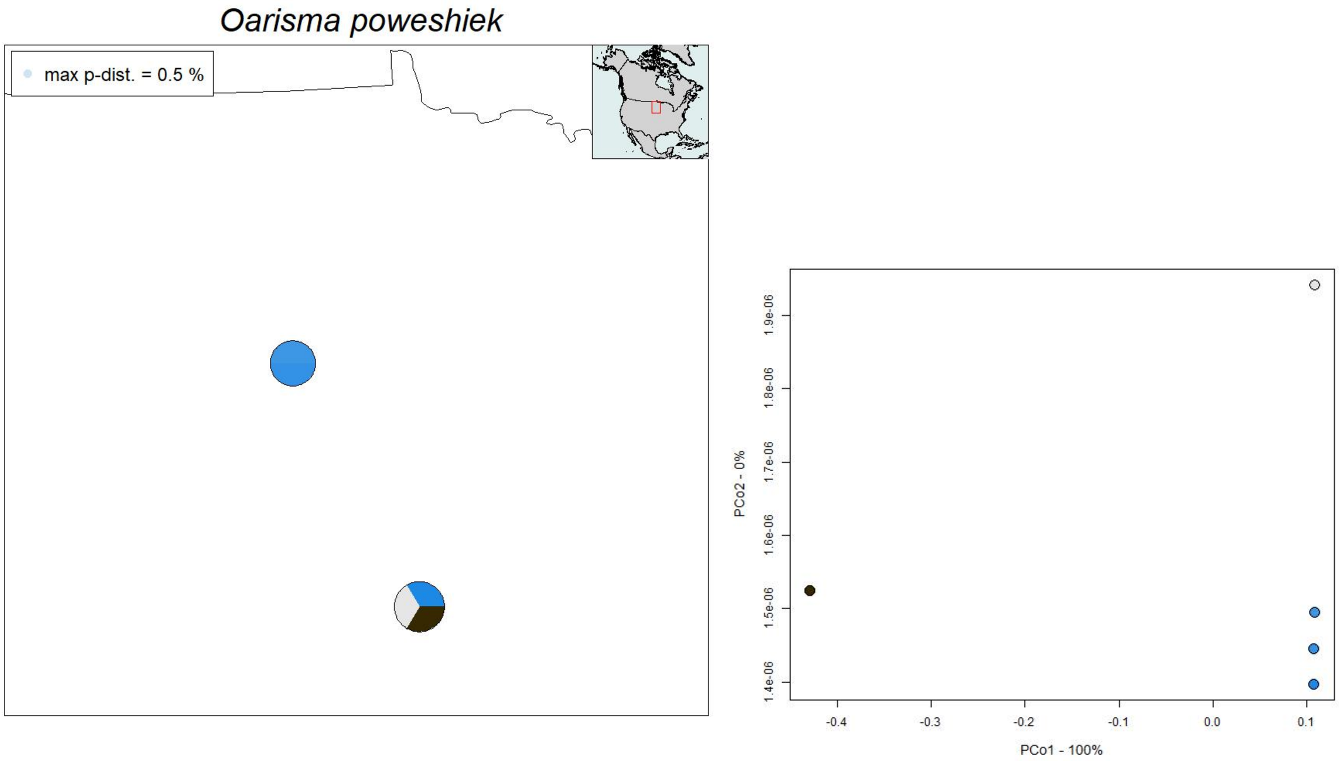

Figure 179 Map of *Oarisma poweshiek* showing the localities of the sequenced specimens (left). Nearby localities are grouped in pies. Colours match the bidimensional colour space of the PCoA projection (right) of max p-dists among sequences (dots). Sequences= 5; Hap obs.= 2; Hap asympt.= NA; Hap % obs.= NA%; GST= NaN; DST= NaN; HD= NA; ND= NA; max p-dist= 0.5%.

Haplotype network analysis and bubble plot of *Oarisma poweshiek* were not possible. Sequences > 599 bp = 4.

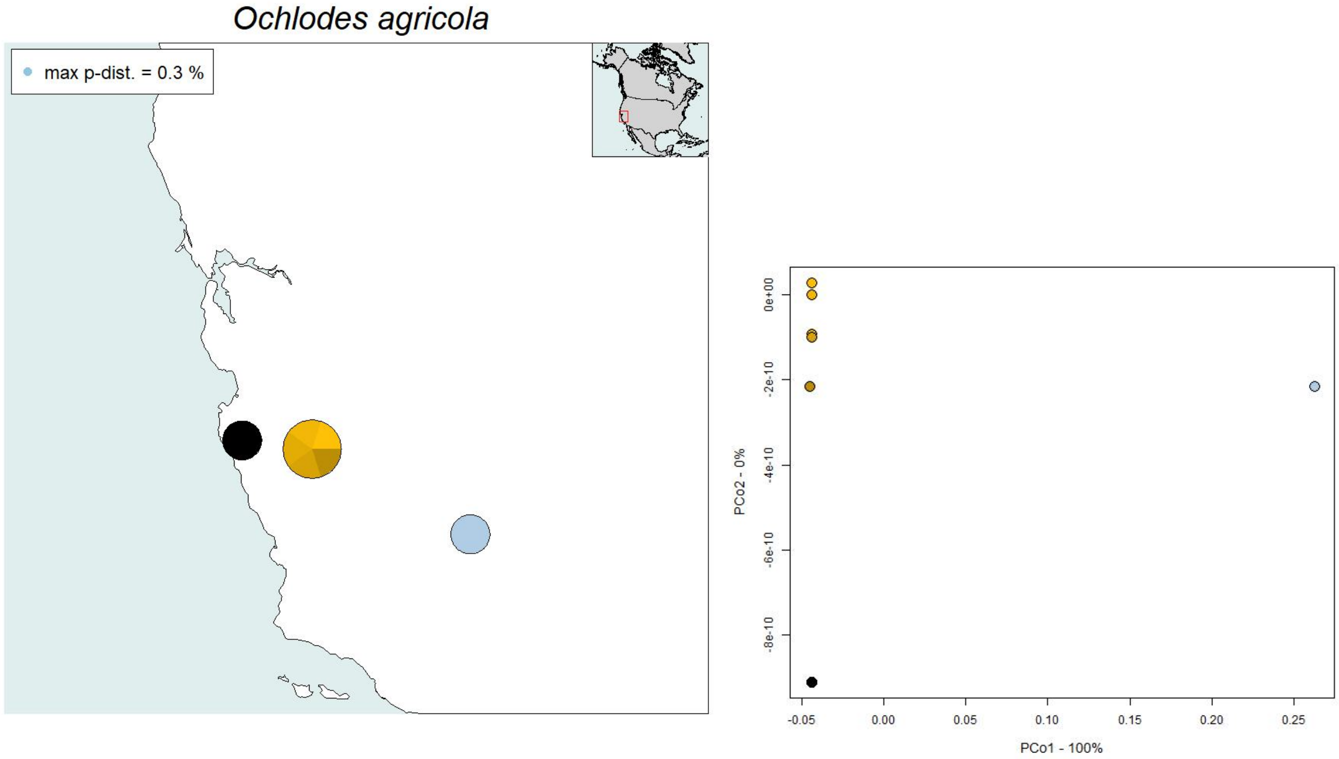

Figure 180 Map of *Ochlodes agricola* showing the localities of the sequenced specimens (left). Nearby localities are grouped in pies. Colours match the bidimensional colour space of the PCoA projection (right) of max p-dists among sequences (dots). Sequences= 7; Hap obs.= 2; Hap asympt.= NA; Hap % obs.= NA%; GST= NaN; DST= NaN; HD= NA; ND= NA; max p-dist= 0.3%.

Haplotype network analysis and bubble plot of *Ochlodes agricola* were not possible. Sequences > 599 bp = 7.

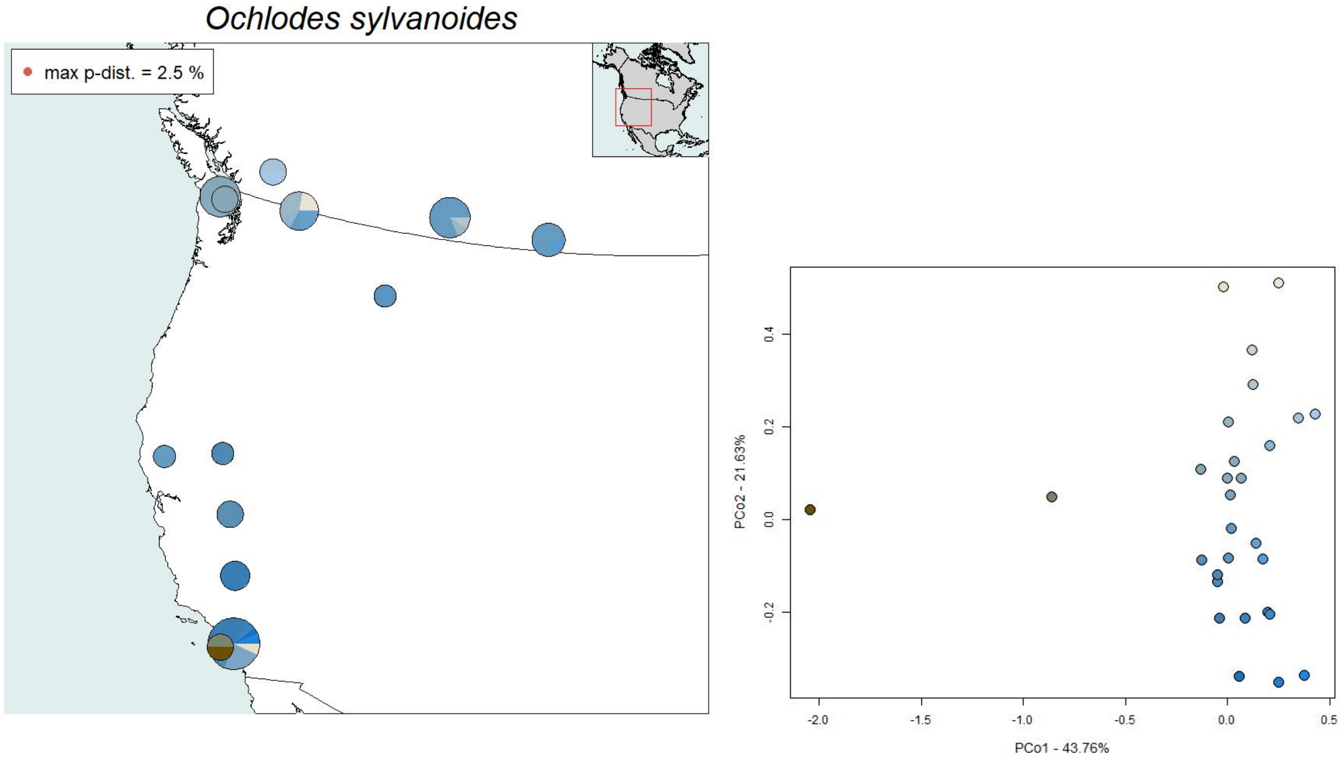

Figure 181 Map of *Ochlodes sylvanoides* showing the localities of the sequenced specimens (left). Nearby localities are grouped in pies. Colours match the bidimensional colour space of the PCoA projection (right) of max p-dists among sequences (dots). Sequences= 79; Hap obs.= 25; Hap asympt.= 44.4; Hap % obs.= 56.4%; GST= 0.454; DST= 0.0015; HD= 0.914; ND= 0.0039; max p-dist= 2.5%.

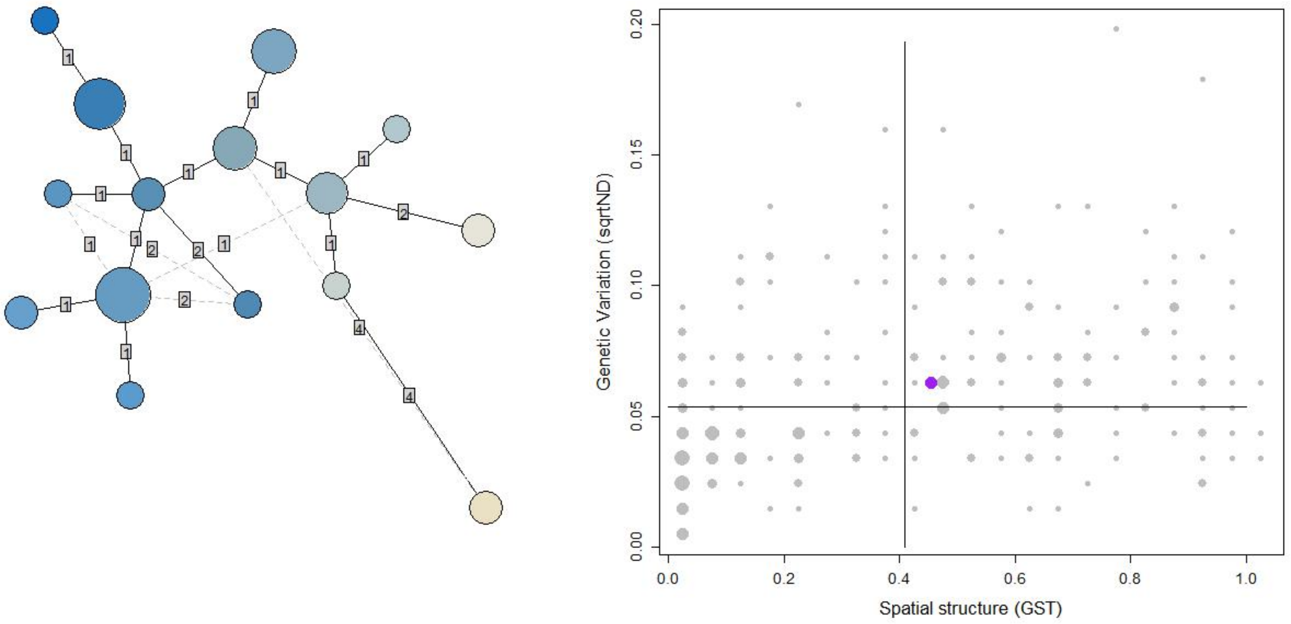

Figure 182: Haplotype network (left) of *Ochlodes sylvanoides* sequences > 599 bp with colours matching the PCoA colour space (above). The bubble plot for mt-DNA polymorphism (square root transformed nucleotide diversity) and spatial structure (GST) among all species in the atlas and values for *Ochlodes sylvanoides* (purple dot). The horizontal and vertical lines represent median values of nucleotide diversity and GST, respectively. Sequences > 599 bp= 59.

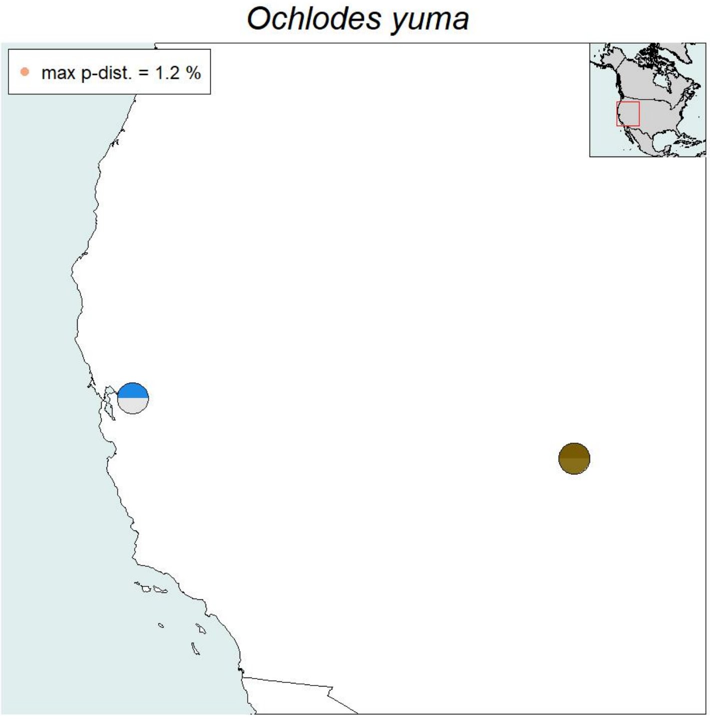

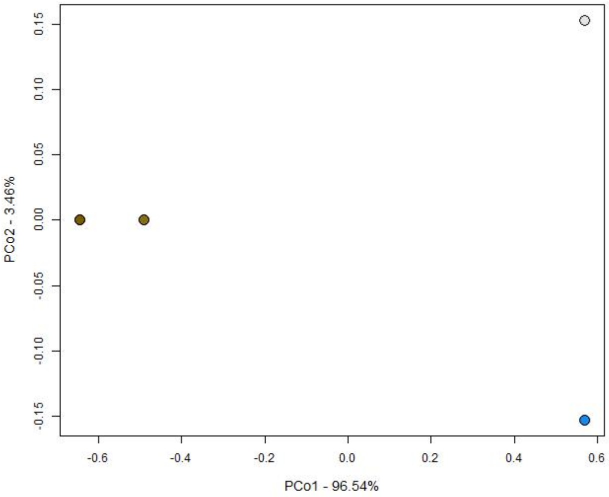

Figure 183 Map of *Ochlodes yuma* showing the localities of the sequenced specimens (left). Nearby localities are grouped in pies. Colours match the bidimensional colour space of the PCoA projection (right) of max p-dists among sequences (dots). Sequences= 4; Hap obs.= 4; Hap asympt.= NA; Hap % obs.= NA%; GST= NaN; DST= NaN; HD= NA; ND= NA; max p-dist= 1.2%.

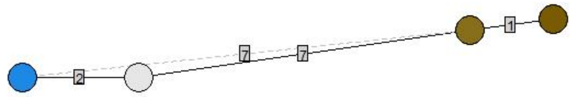

Figure 184: Haplotype network of *Ochlodes yuma*. Sequences > 599 bp= 4.

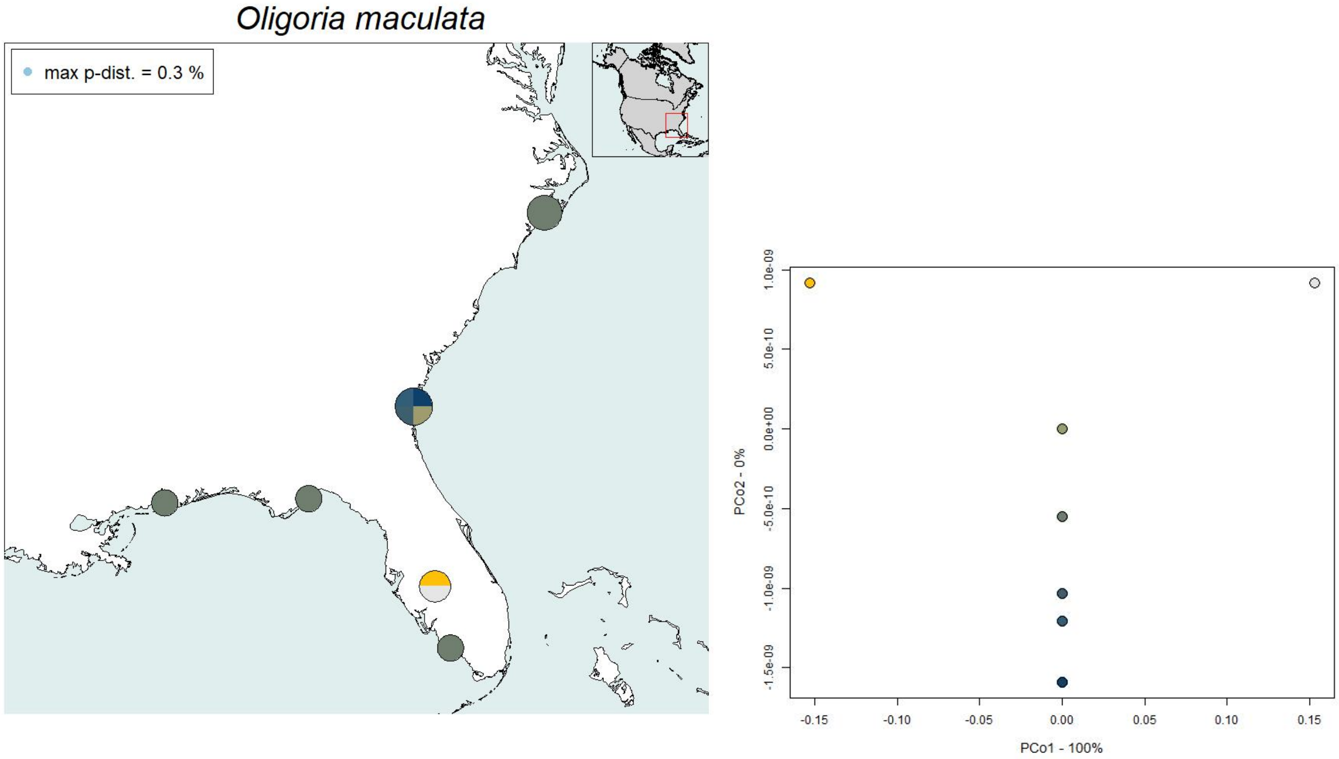

Figure 185 Map of *Oligoria maculata* showing the localities of the sequenced specimens (left). Nearby localities are grouped in pies. Colours match the bidimensional colour space of the PCoA projection (right) of max p-dists among sequences (dots). Sequences= 12; Hap obs.= 3; Hap asympt.= 3.9; Hap % obs.= 76.6%; GST= NaN; DST= NaN; HD= 0.318; ND= 0.0005; max p-dist= 0.3%.

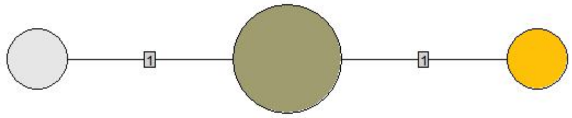

Figure 186: Haplotype network of *Oligoria maculata*. Sequences > 599 bp= 12.

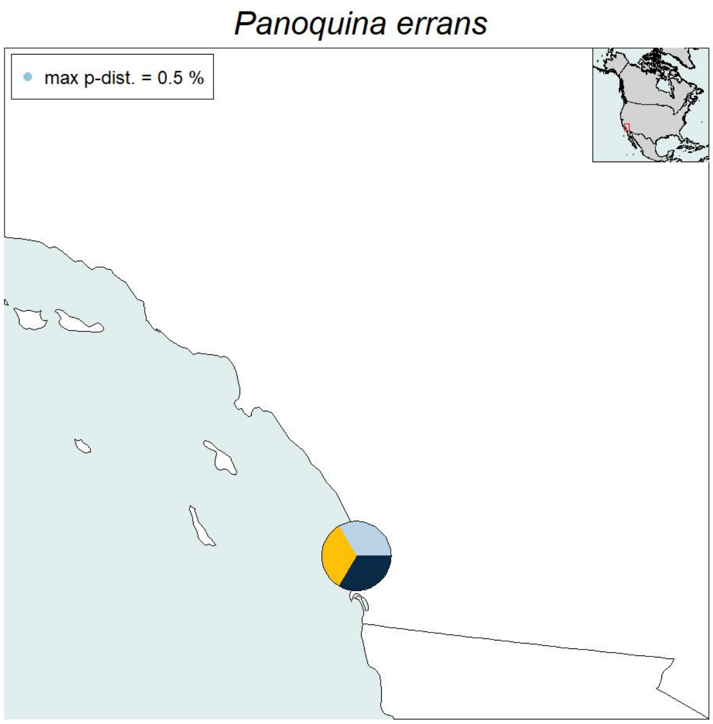

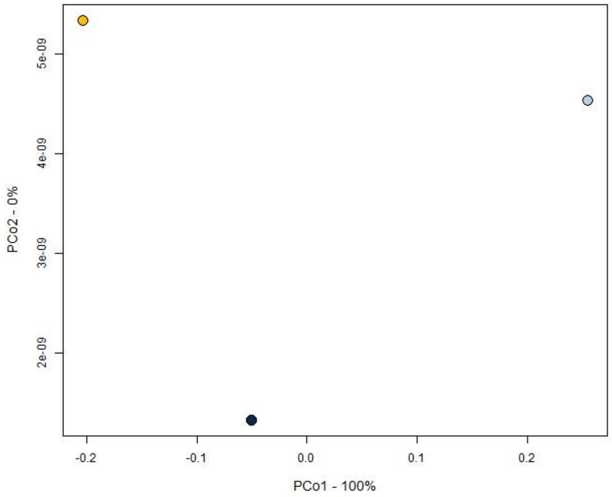

Figure 187 Map of *Panoquina errans* showing the localities of the sequenced specimens (left). Nearby localities are grouped in pies. Colours match the bidimensional colour space of the PCoA projection (right) of max p-dists among sequences (dots). Sequences= 3; Hap obs.= 3; Hap asympt.= NA; Hap % obs.= NA%; GST= NaN; DST= NaN; HD= NA; ND= NA; max p-dist= 0.5%.

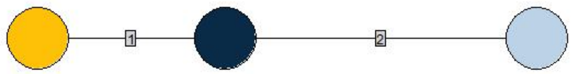

Figure 188: Haplotype network of *Panoquina errans*. Sequences > 599 bp= 3.

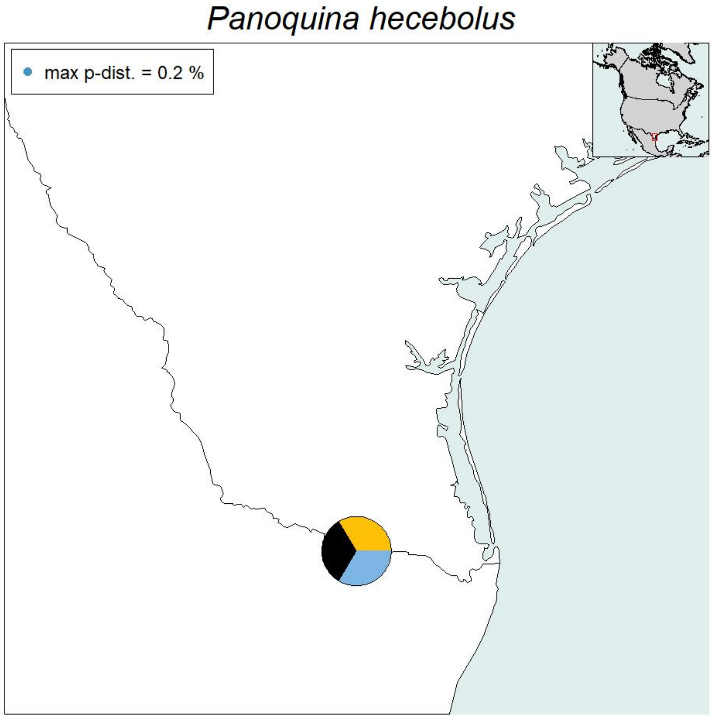

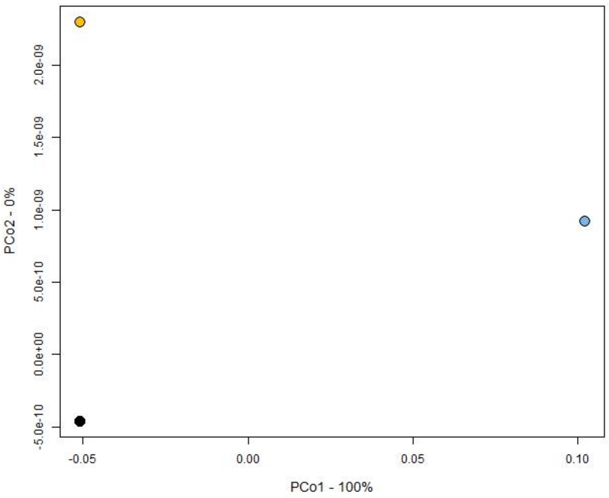

Figure 189 Map of *Panoquina hecebolus* showing the localities of the sequenced specimens (left). Nearby localities are grouped in pies. Colours match the bidimensional colour space of the PCoA projection (right) of max p-dists among sequences (dots). Sequences= 3; Hap obs.= 2; Hap asympt.= NA; Hap % obs.= NA%; GST= NaN; DST= NaN; HD= NA; ND= NA; max p-dist= 0.2%.

Haplotype network analysis and bubble plot of *Panoquina hecebolus* were not possible. Sequences > 599 bp = 3.

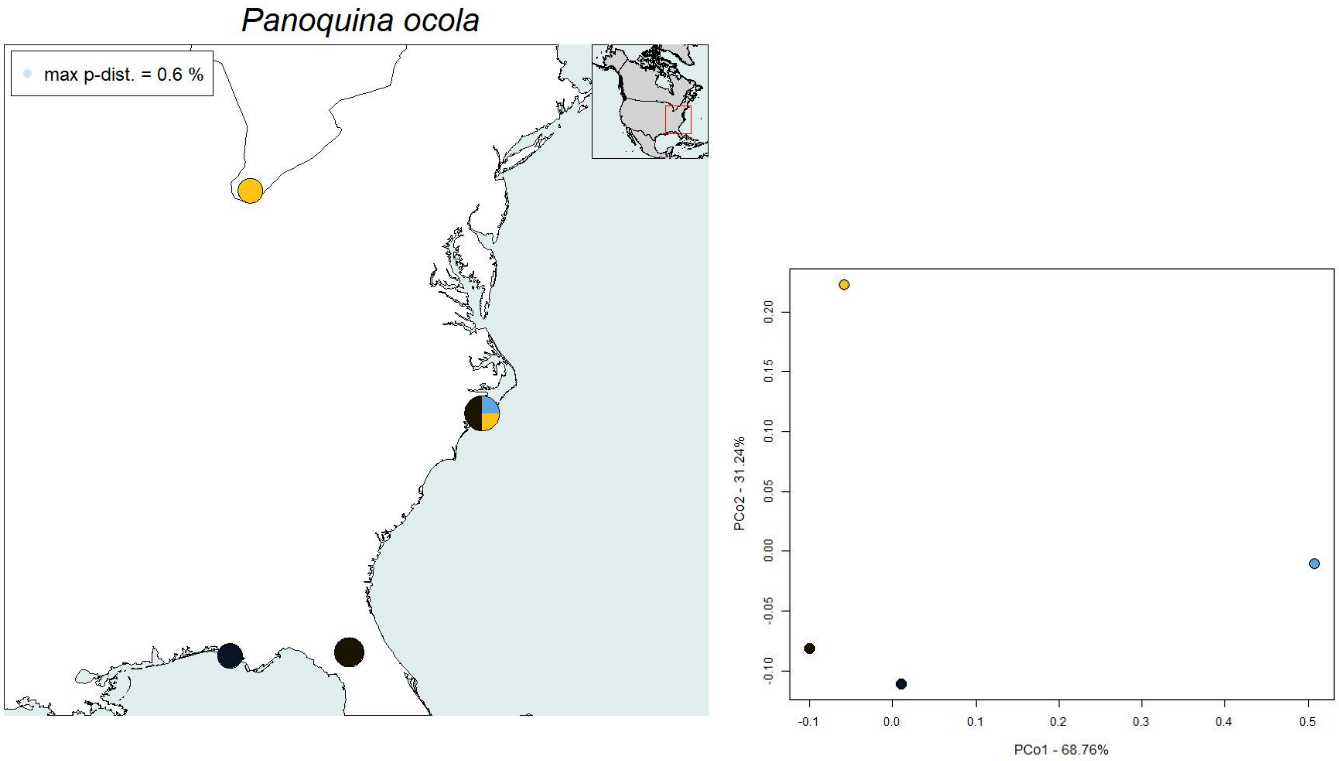

Figure 190 Map of *Panoquina ocola* showing the localities of the sequenced specimens (left). Nearby localities are grouped in pies. Colours match the bidimensional colour space of the PCoA projection (right) of max p-dists among sequences (dots). Sequences= 8; Hap obs.= 3; Hap asympt.= NA; Hap % obs.= NA%; GST= NaN; DST= NaN; HD= NA; ND= NA; max p-dist= 0.6%.

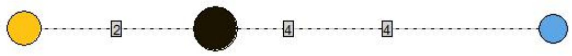

Figure 191: Haplotype network of *Panoquina ocola*. Sequences > 599 bp= 8.

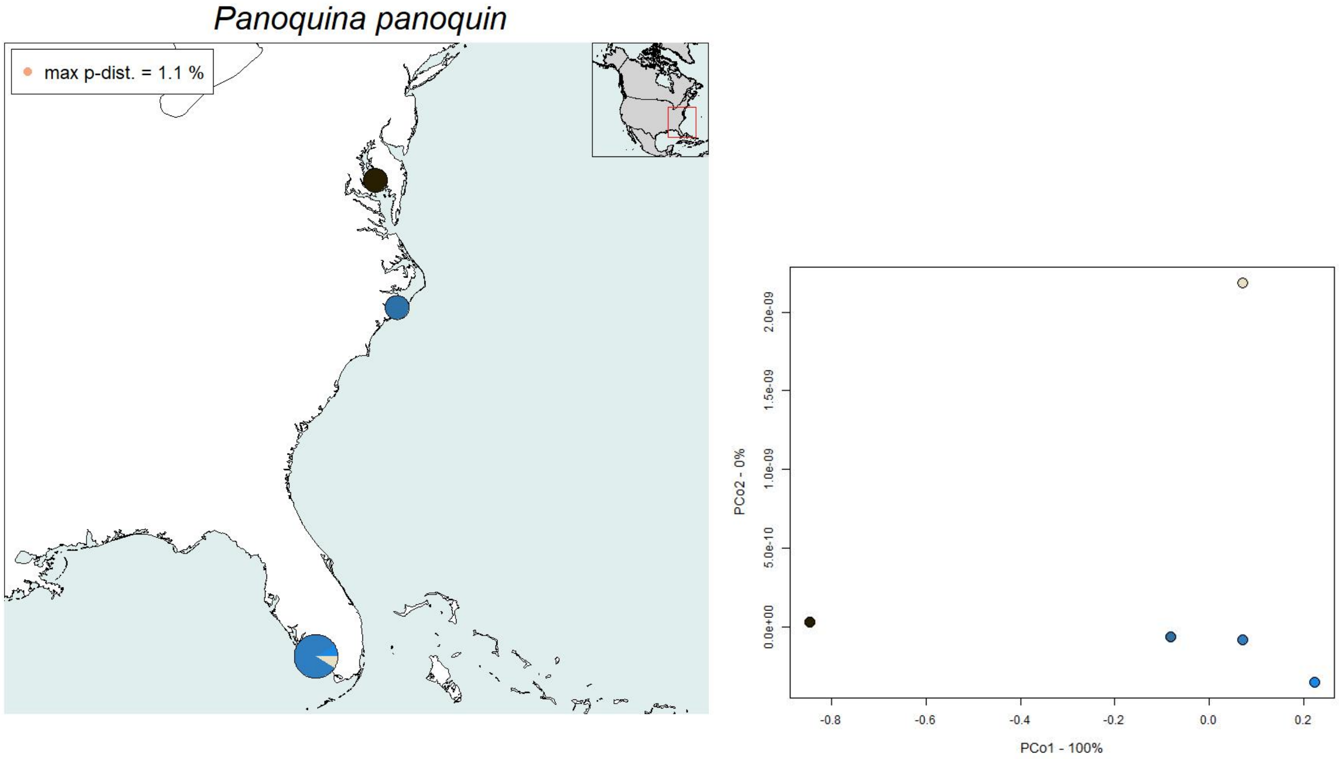

Figure 192 Map of *Panoquina panoquin* showing the localities of the sequenced specimens (left). Nearby localities are grouped in pies. Colours match the bidimensional colour space of the PCoA projection (right) of max p-dists among sequences (dots). Sequences= 13; Hap obs.= 4; Hap asympt.= 6.8; Hap % obs.= 59.1%; GST= NaN; DST= NaN; HD= 0.423; ND= 0.0018; max p-dist= 1.1%.

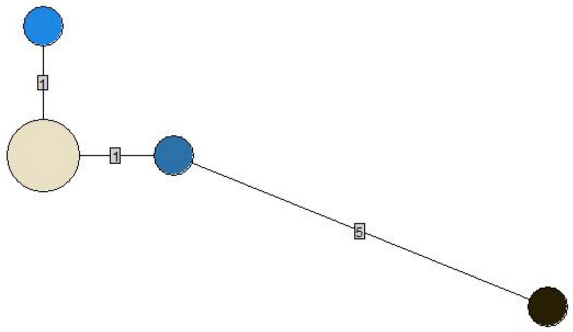

Figure 193: Haplotype network of *Panoquina panoquin*. Sequences > 599 bp= 13.

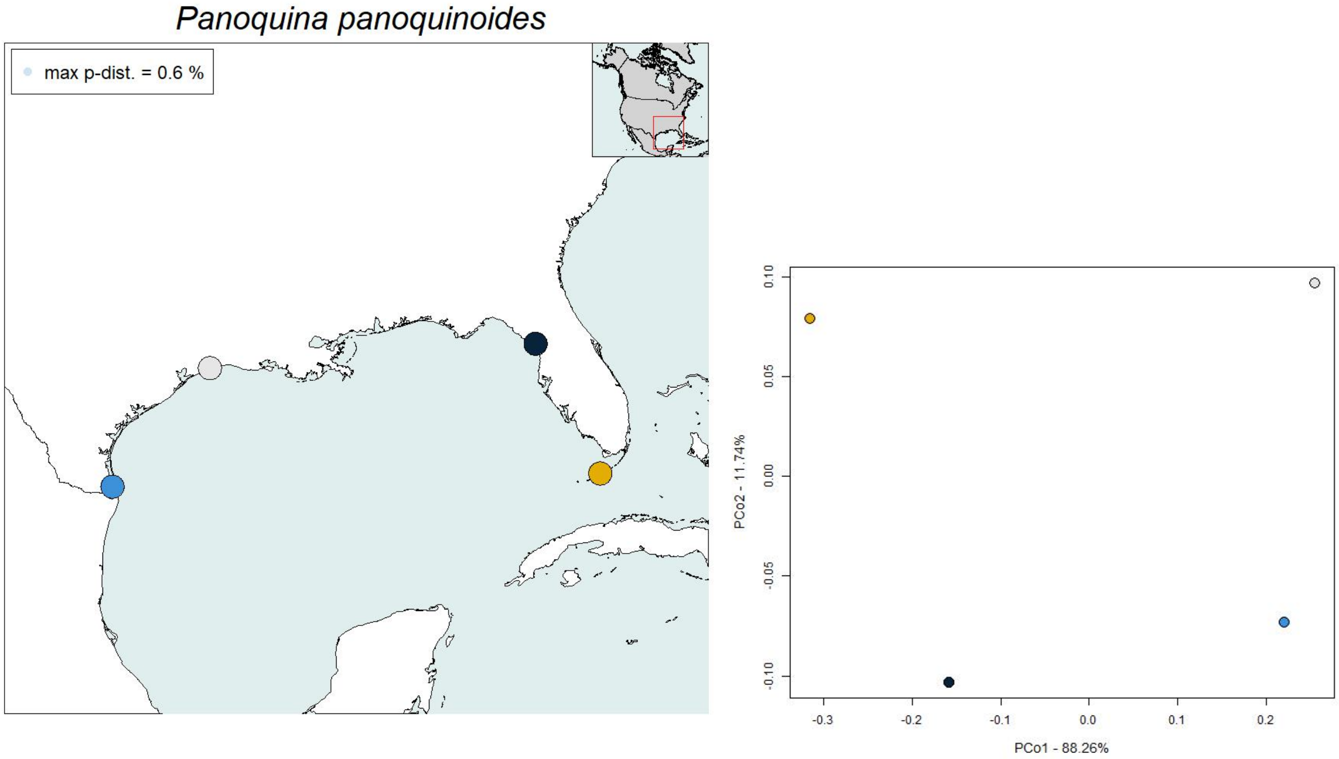

Figure 194 Map of *Panoquina panoquinoides* showing the localities of the sequenced specimens (left). Nearby localities are grouped in pies. Colours match the bidimensional colour space of the PCoA projection (right) of max p-dists among sequences (dots). Sequences= 4; Hap obs.= 3; Hap asympt.= NA; Hap % obs.= NA%; GST= NaN; DST= NaN; HD= NA; ND= NA; max p-dist= 0.6%.

Haplotype network analysis and bubble plot of *Panoquina panoquinoides* were not possible. Sequences > 599 bp = 3.

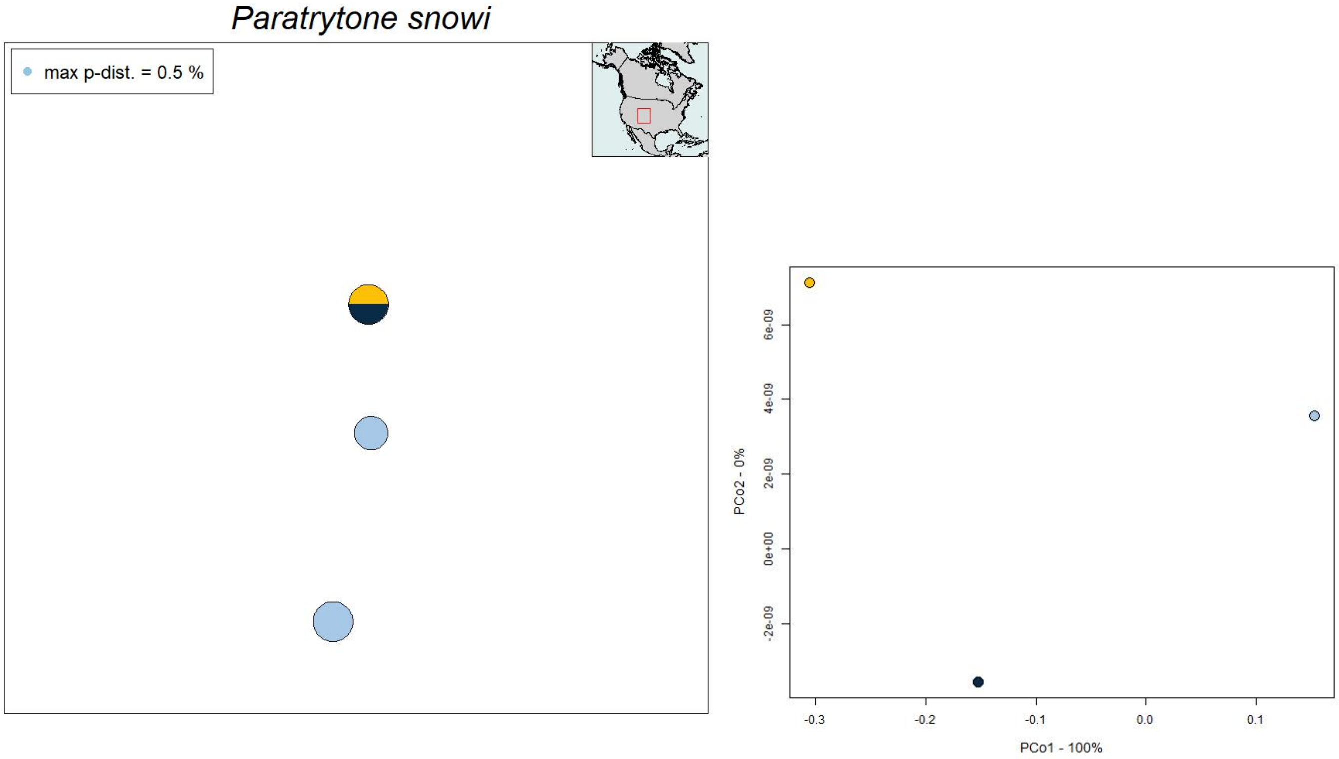

Figure 195 Map of *Paratrytone snowi* showing the localities of the sequenced specimens (left). Nearby localities are grouped in pies. Colours match the bidimensional colour space of the PCoA projection (right) of max p-dists among sequences (dots). Sequences= 5; Hap obs.= 3; Hap asympt.= NA; Hap % obs.= NA%; GST= NaN; DST= NaN; HD= NA; ND= NA; max p-dist= 0.5%.

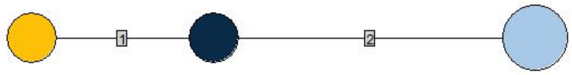

Figure 196: Haplotype network of *Paratrytone snowi*. Sequences > 599 bp= 5.

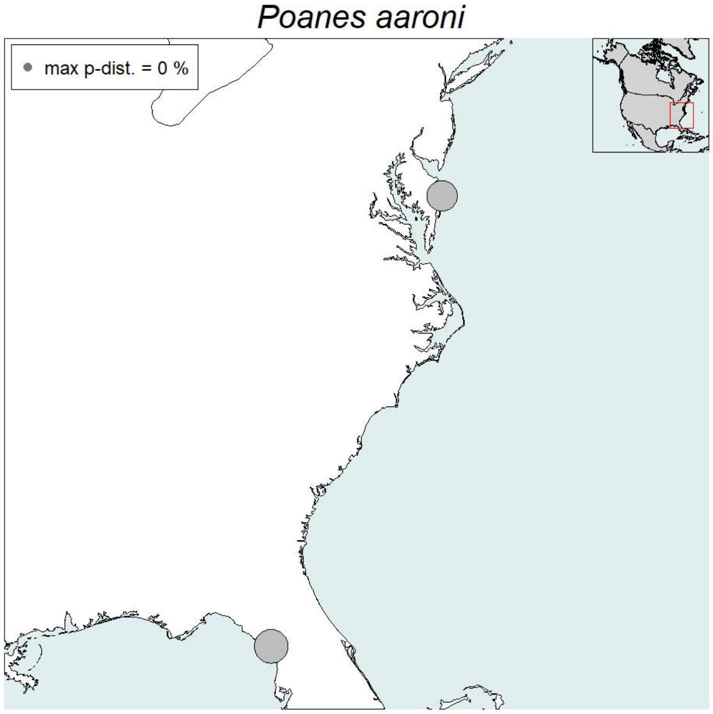

Figure 197: Map of *Poanes aaroni* showing the localities of the sequenced specimens. Nearby localities are grouped in pies. Due to the presence of a single haplotype PCoA projection was not done and a single grey colour was plotted on the map. Sequences= 5; Hap obs.= 1; Hap asympt.= NA; Hap % obs.= NA%; GST= NaN; DST= NaN; HD= NA; ND= NA; max p-dist= 0%.

Haplotype network analysis and bubble plot of *Poanes aaroni* were not possible. Sequences > 599 bp = 5.

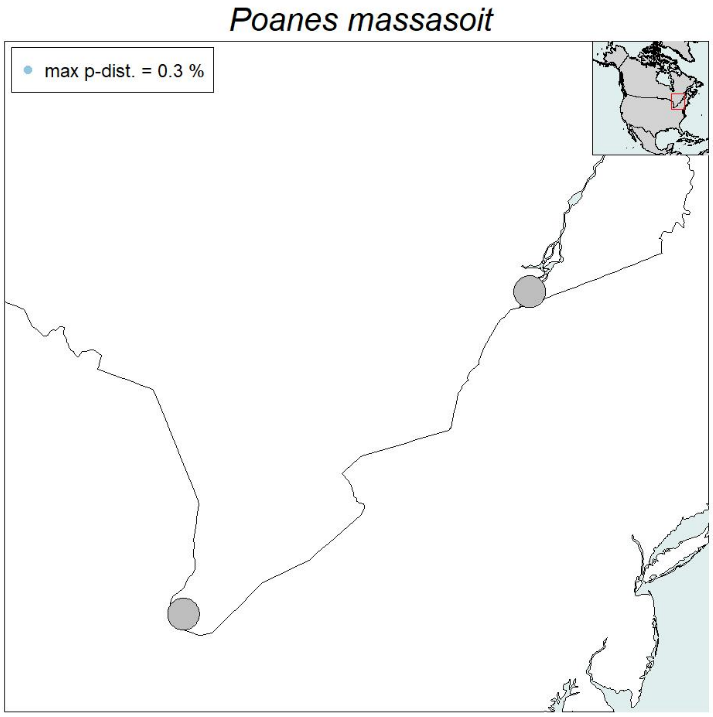

Figure 198: Map of *Poanes massasoit* showing the localities of the sequenced specimens. Nearby localities are grouped in pies. Due to the presence of a single haplotype PCoA projection was not done and a single grey colour was plotted on the map. Sequences= 2; Hap obs.= 2; Hap asympt.= NA; Hap % obs.= NA%; GST= NaN; DST= NaN; HD= NA; ND= NA; max p-dist= 0.3%.

Haplotype network analysis and bubble plot of *Poanes massasoit* were not possible. Sequences > 599 bp = 2.

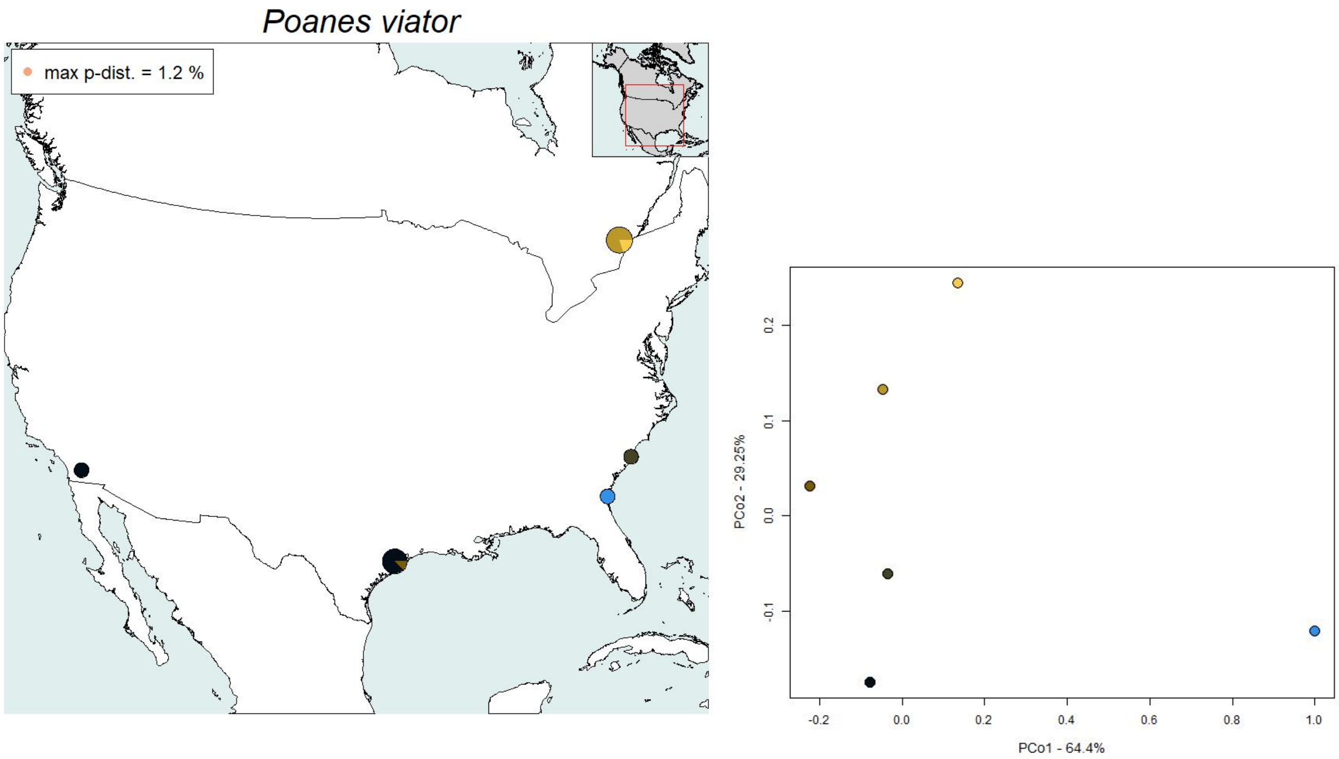

Figure 199 Map of *Poanes viator* showing the localities of the sequenced specimens (left). Nearby localities are grouped in pies. Colours match the bidimensional colour space of the PCoA projection (right) of max p-dists among sequences (dots). Sequences= 21; Hap obs.= 6; Hap asympt.= 10.3; Hap % obs.= 58.3%; GST= 0.779; DST= 0.0015; HD= 0.729; ND= 0.0029; max p-dist= 1.2%.

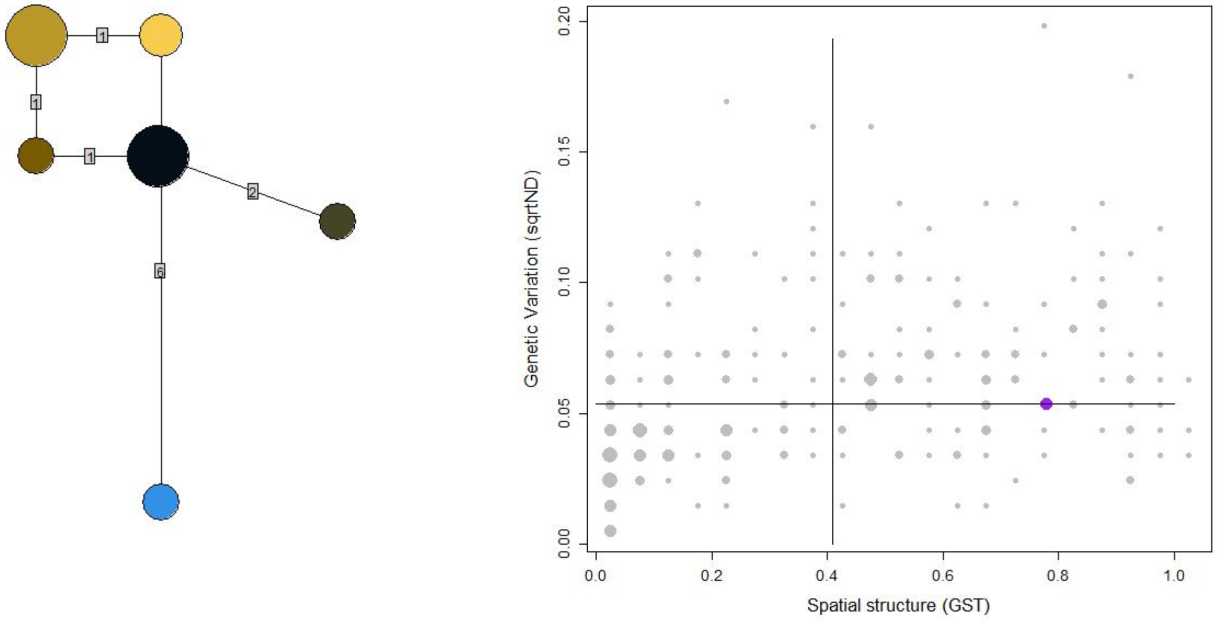

Figure 200: Haplotype network (left) of *Poanes viator* sequences > 599 bp with colours matching the PCoA colour space (above). The bubble plot for mt-DNA polymorphism (square root transformed nucleotide diversity) and spatial structure (GST) among all species in the atlas and values for *Poanes viator* (purple dot). The horizontal and vertical lines represent median values of nucleotide diversity and GST, respectively. Sequences > 599 bp= 21.

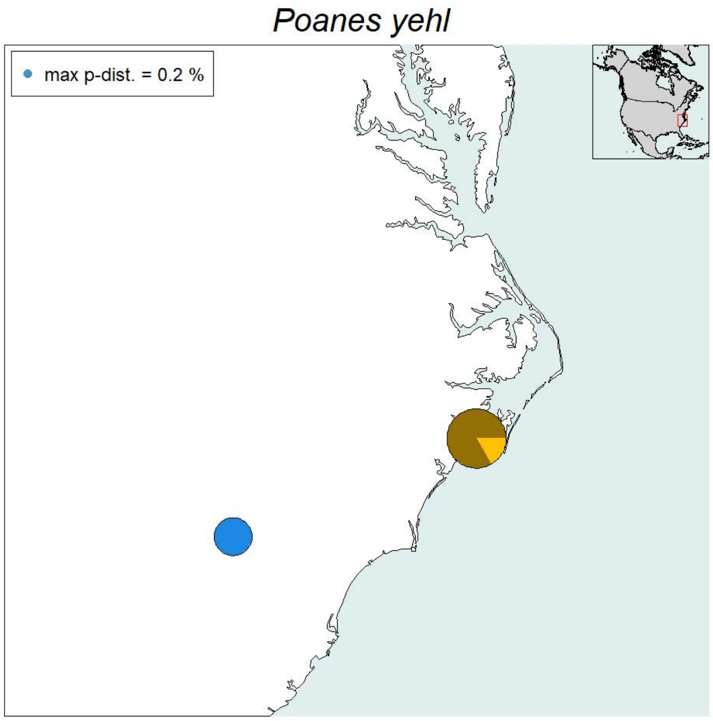

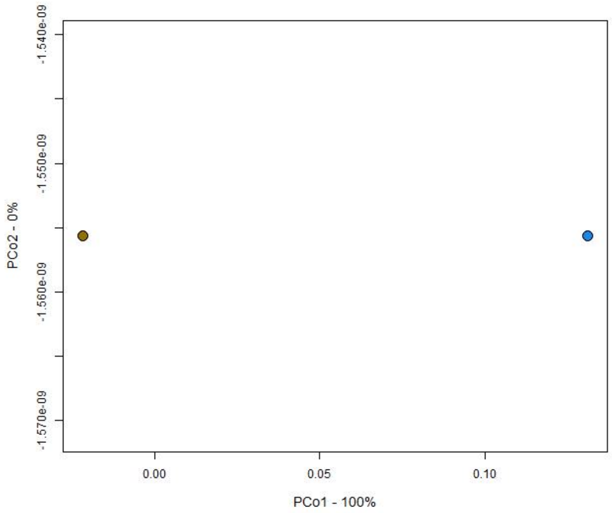

Figure 201 Map of *Poanes yehl* showing the localities of the sequenced specimens (left). Nearby localities are grouped in pies. Colours match the bidimensional colour space of the PCoA projection (right) of max p-dists among sequences (dots). Sequences= 7; Hap obs.= 2; Hap asympt.= NA; Hap % obs.= NA%; GST= NaN; DST= NaN; HD= NA; ND= NA; max p-dist= 0.2%.

Haplotype network analysis and bubble plot of *Poanes yehl* were not possible. Sequences > 599 bp = 7.

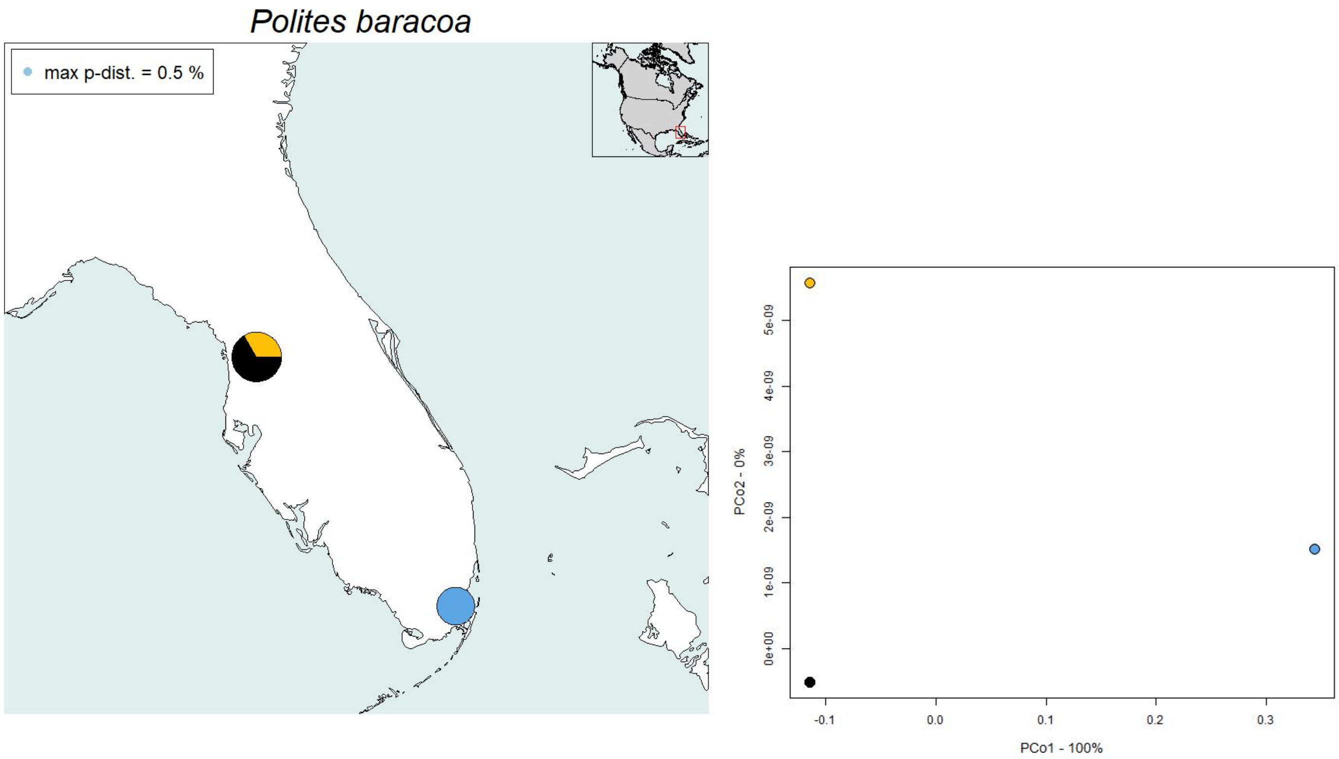

Figure 202 Map of *Polites baracoa* showing the localities of the sequenced specimens (left). Nearby localities are grouped in pies. Colours match the bidimensional colour space of the PCoA projection (right) of max p-dists among sequences (dots). Sequences= 4; Hap obs.= 2; Hap asympt.= NA; Hap % obs.= NA%; GST= NaN; DST= NaN; HD= NA; ND= NA; max p-dist= 0.5%.

Haplotype network analysis and bubble plot of *Polites baracoa* were not possible. Sequences > 599 bp = 4.

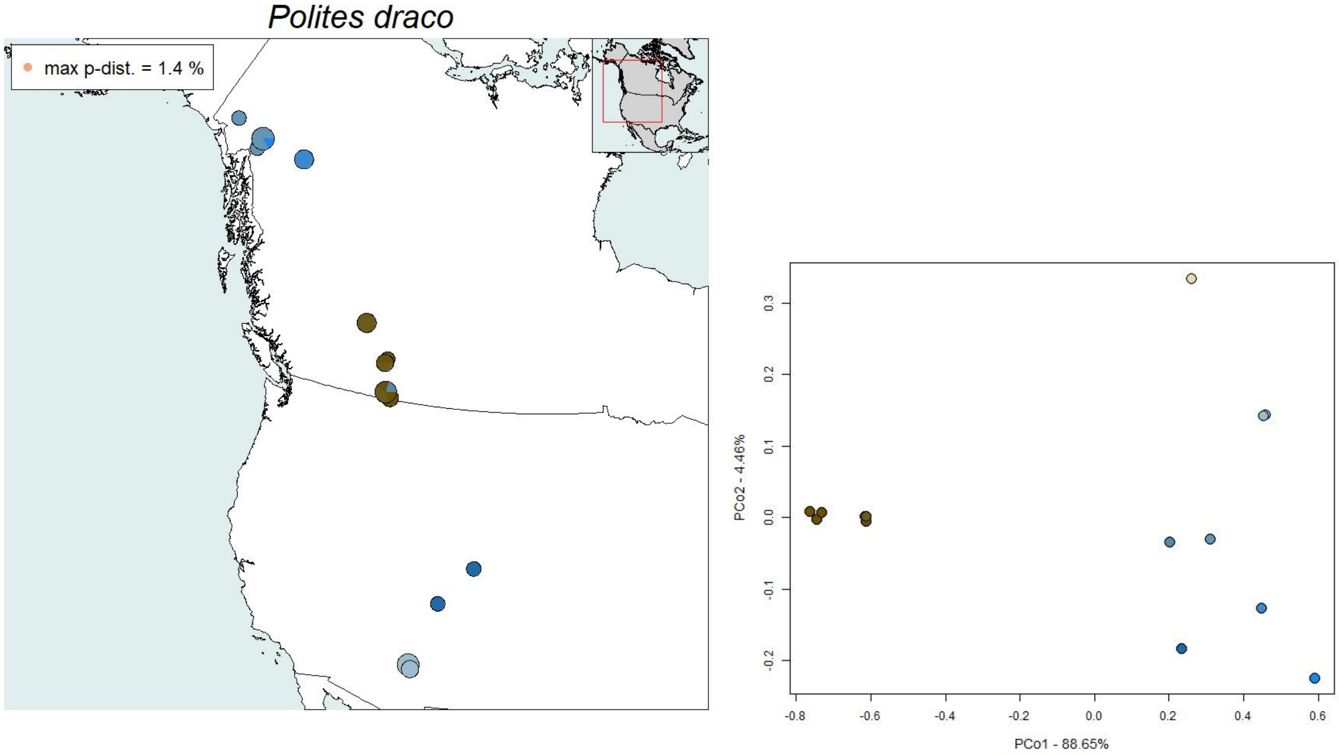

Figure 203 Map of *Polites draco* showing the localities of the sequenced specimens (left). Nearby localities are grouped in pies. Colours match the bidimensional colour space of the PCoA projection (right) of max p-dists among sequences (dots). Sequences= 33; Hap obs.= 10; Hap asympt.= 22.1; Hap % obs.= 45.2%; GST= 0.762; DST= 0.0045; HD= 0.839; ND= 0.0059; max p-dist= 1.4%.

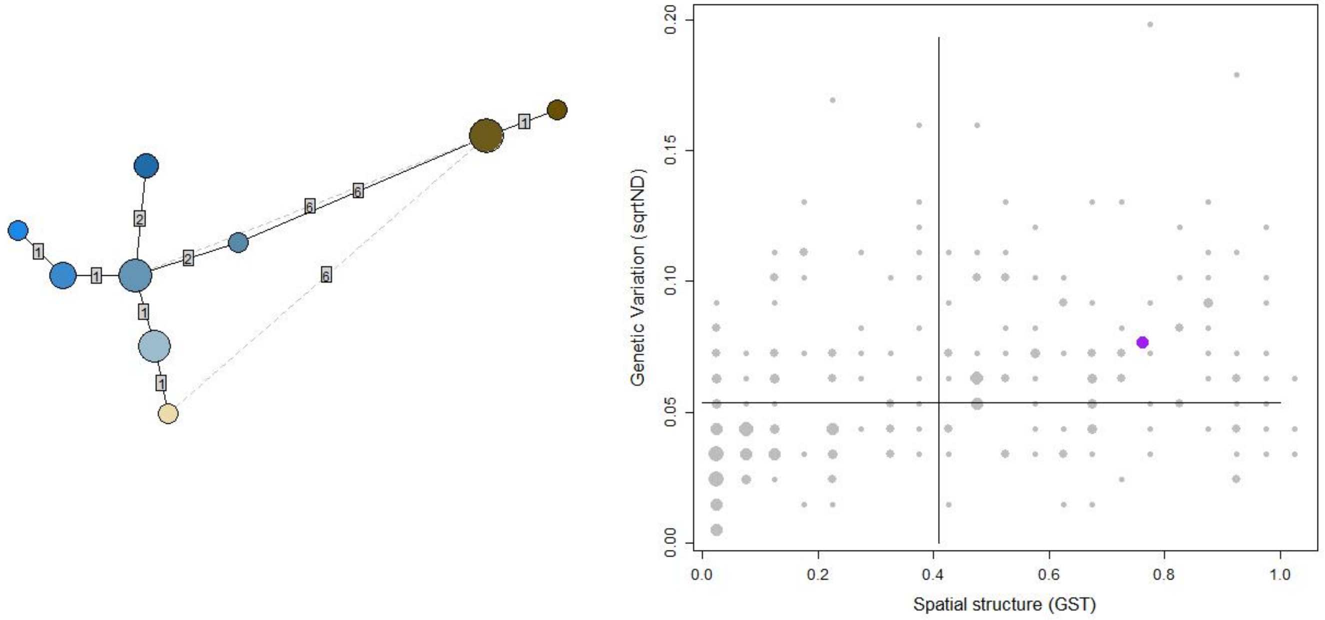

Figure 204: Haplotype network (left) of *Polites draco* sequences > 599 bp with colours matching the PCoA colour space (above). The bubble plot for mt-DNA polymorphism (square root transformed nucleotide diversity) and spatial structure (GST) among all species in the atlas and values for *Polites draco* (purple dot). The horizontal and vertical lines represent median values of nucleotide diversity and GST, respectively. Sequences > 599 bp= 30.

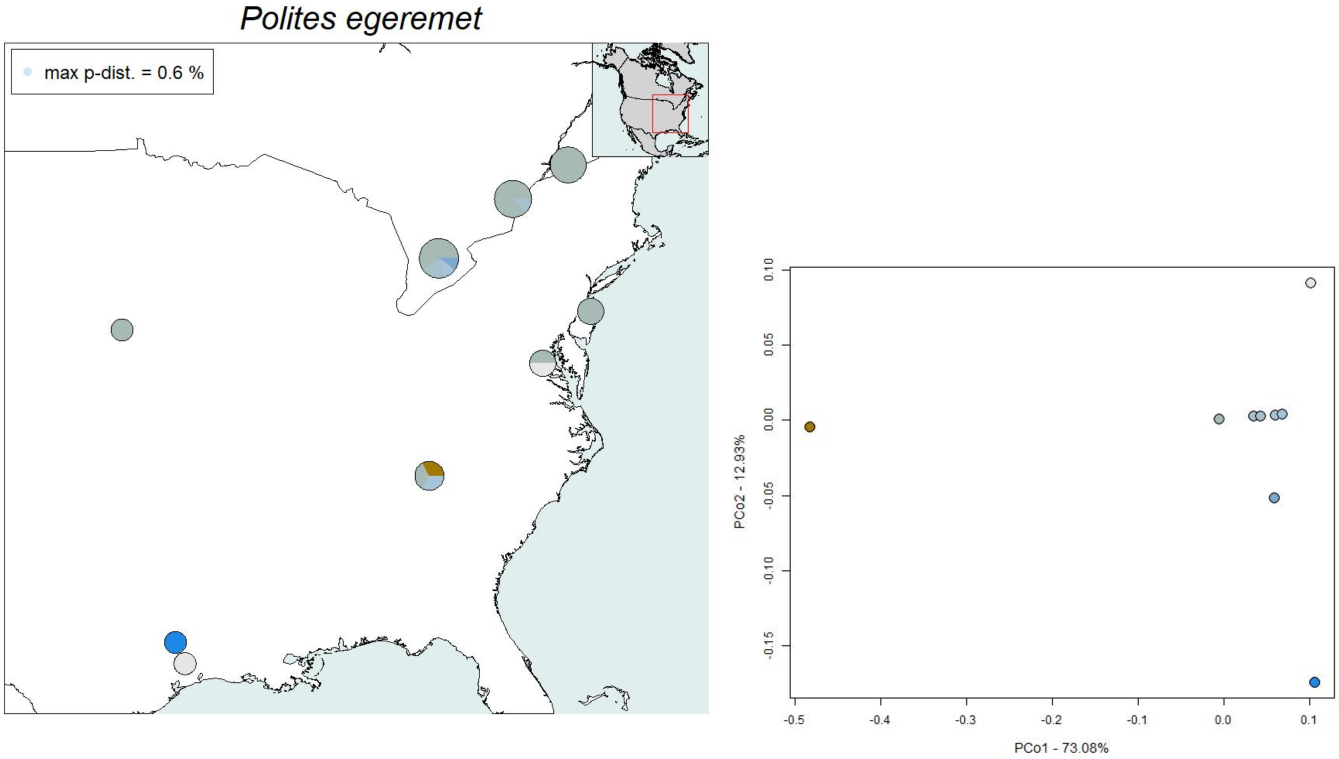

Figure 205 Map of *Polites egeremet* showing the localities of the sequenced specimens (left). Nearby localities are grouped in pies. Colours match the bidimensional colour space of the PCoA projection (right) of max p-dists among sequences (dots). Sequences= 35; Hap obs.= 6; Hap asympt.= 15.7; Hap % obs.= 38.2%; GST= 0.244; DST= 0.0001; HD= 0.269; ND= 0.0005; max p-dist= 0.6%.

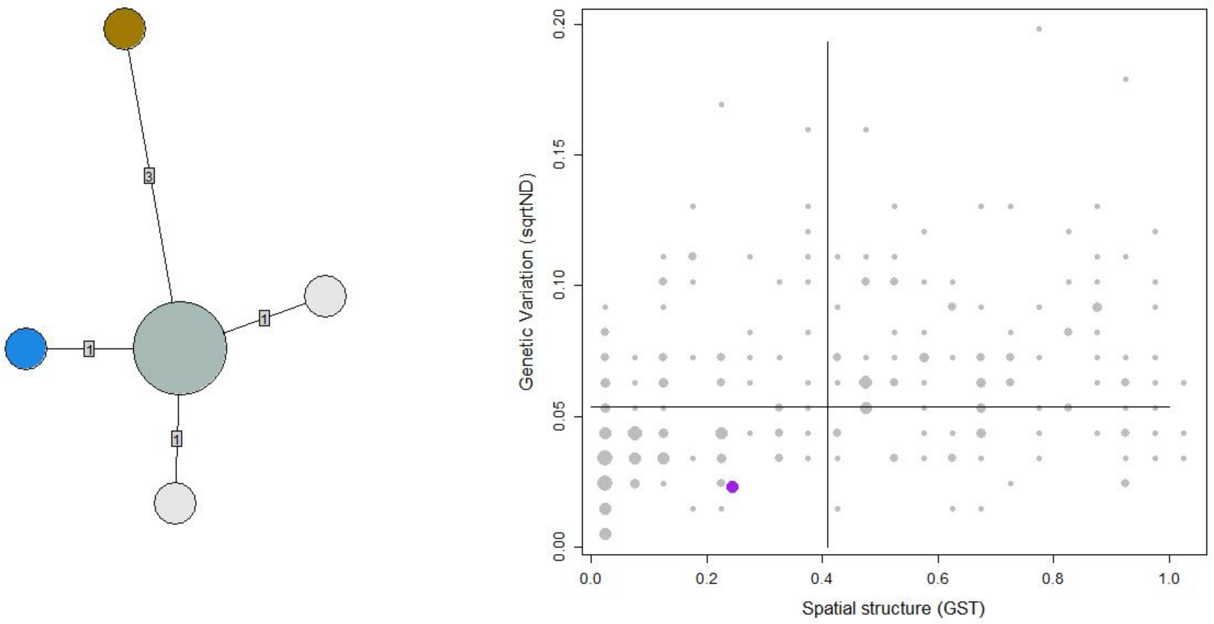

Figure 206: Haplotype network (left) of *Polites egeremet* sequences > 599 bp with colours matching the PCoA colour space (above). The bubble plot for mt-DNA polymorphism (square root transformed nucleotide diversity) and spatial structure (GST) among all species in the atlas and values for *Polites egeremet* (purple dot). The horizontal and vertical lines represent median values of nucleotide diversity and GST, respectively. Sequences > 599 bp= 29.

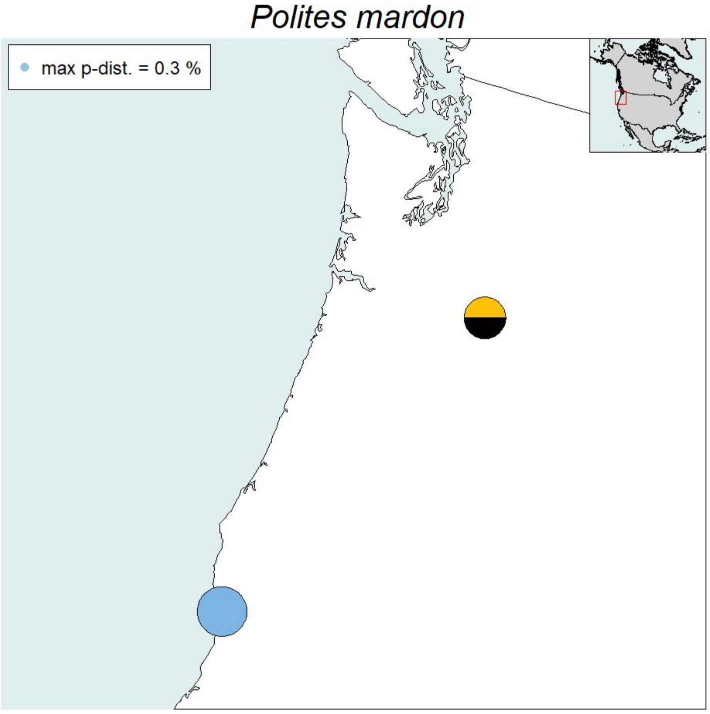

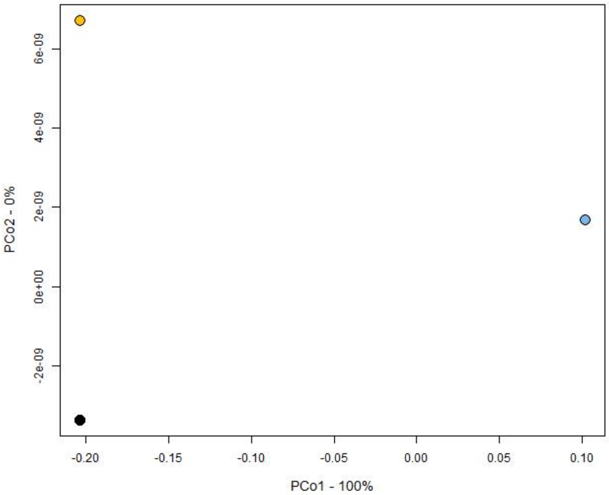

Figure 207 Map of *Polites mardon* showing the localities of the sequenced specimens (left). Nearby localities are grouped in pies. Colours match the bidimensional colour space of the PCoA projection (right) of max p-dists among sequences (dots). Sequences= 6; Hap obs.= 2; Hap asympt.= NA; Hap % obs.= NA%; GST= NaN; DST= NaN; HD= NA; ND= NA; max p-dist= 0.3%.

Haplotype network analysis and bubble plot of *Polites mardon* were not possible. Sequences > 599 bp = 6.

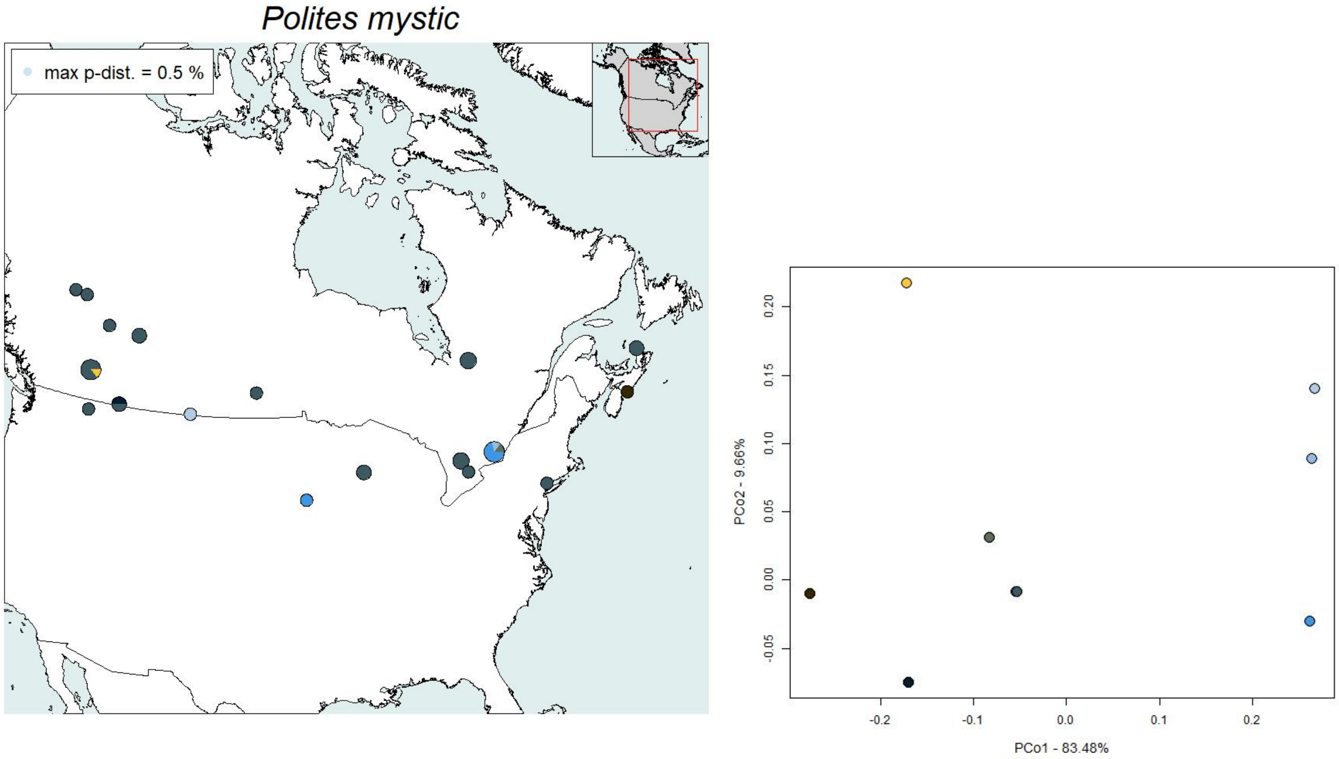

Figure 208 Map of *Polites mystic* showing the localities of the sequenced specimens (left). Nearby localities are grouped in pies. Colours match the bidimensional colour space of the PCoA projection (right) of max p-dists among sequences (dots). Sequences= 38; Hap obs.= 6; Hap asympt.= 11.8; Hap % obs.= 50.7%; GST= 0.689; DST= 0.001; HD= 0.498; ND= 0.0013; max p-dist= 0.5%.

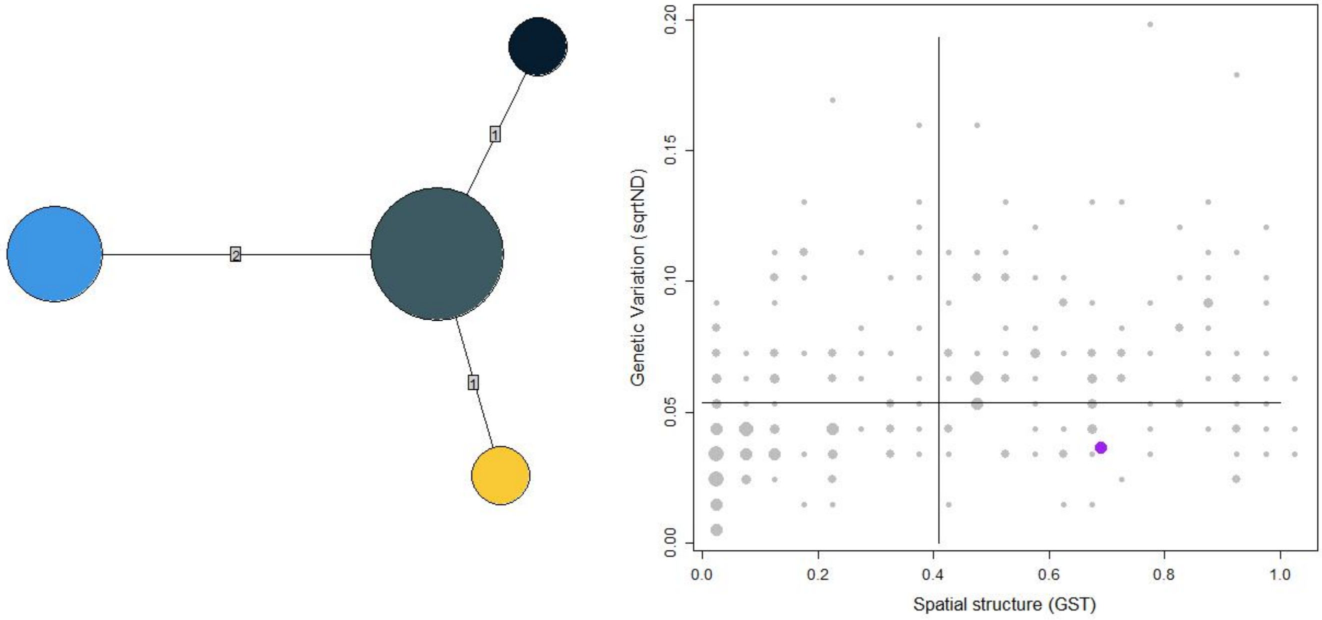

Figure 209: Haplotype network (left) of *Polites mystic* sequences > 599 bp with colours matching the PCoA colour space (above). The bubble plot for mt-DNA polymorphism (square root transformed nucleotide diversity) and spatial structure (GST) among all species in the atlas and values for *Polites mystic* (purple dot). The horizontal and vertical lines represent median values of nucleotide diversity and GST, respectively. Sequences > 599 bp= 35.

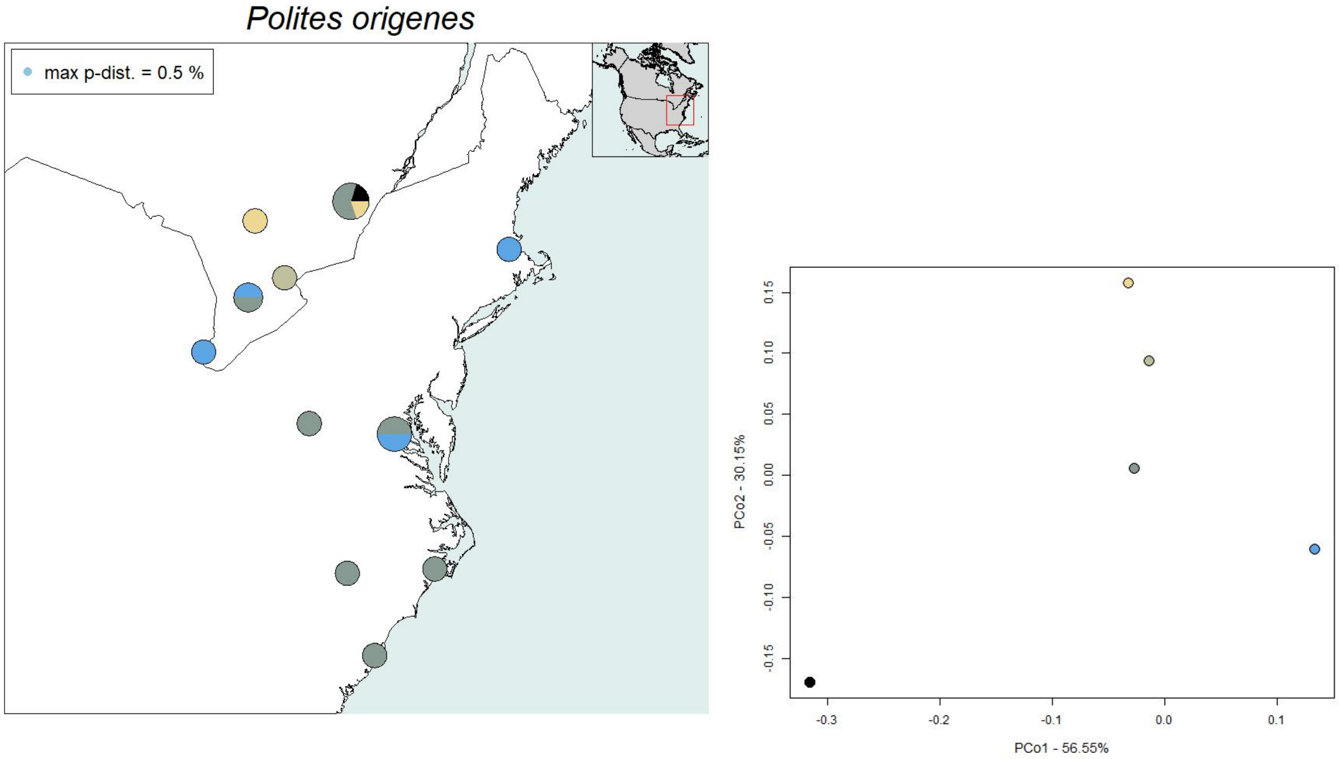

Figure 210 Map of *Polites origenes* showing the localities of the sequenced specimens (left). Nearby localities are grouped in pies. Colours match the bidimensional colour space of the PCoA projection (right) of max p-dists among sequences (dots). Sequences= 19; Hap obs.= 6; Hap asympt.= 11.7; Hap % obs.= 51.4%; GST= 0.149; DST= 0.0002; HD= 0.678; ND= 0.0013; max p-dist= 0.5%.

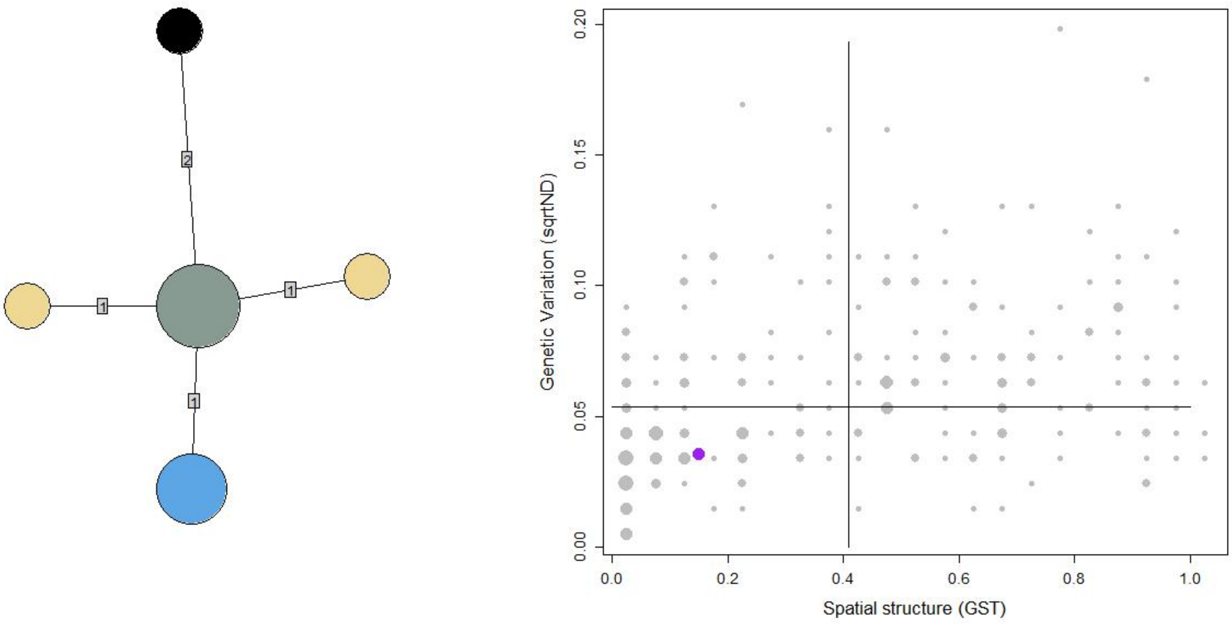

Figure 211: Haplotype network (left) of *Polites origenes* sequences > 599 bp with colours matching the PCoA colour space (above). The bubble plot for mt-DNA polymorphism (square root transformed nucleotide diversity) and spatial structure (GST) among all species in the atlas and values for *Polites origenes* (purple dot). The horizontal and vertical lines represent median values of nucleotide diversity and GST, respectively. Sequences > 599 bp= 18.

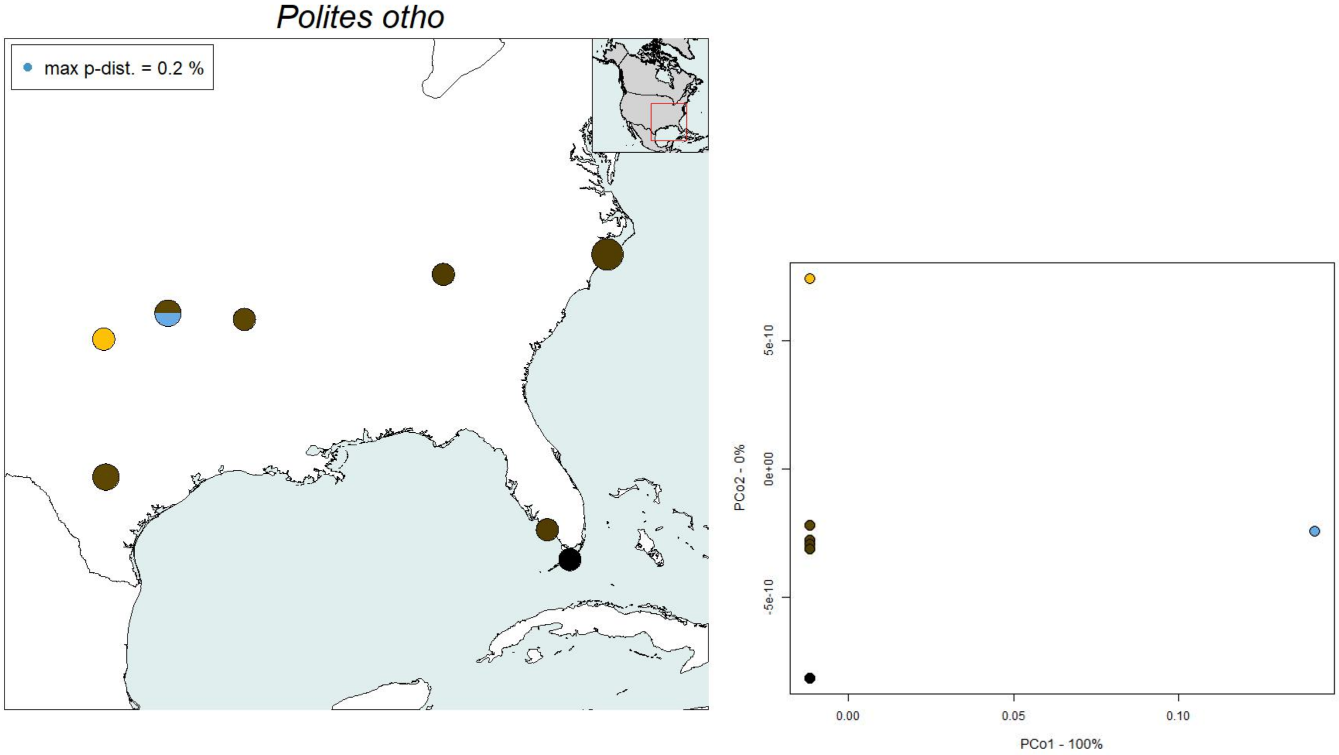

Figure 212 Map of *Polites otho* showing the localities of the sequenced specimens (left). Nearby localities are grouped in pies. Colours match the bidimensional colour space of the PCoA projection (right) of max p-dists among sequences (dots). Sequences= 13; Hap obs.= 2; Hap asympt.= 2; Hap % obs.= 100%; GST= NaN; DST= NaN; HD= 0.154; ND= 0.0002; max p-dist= 0.2%.

Haplotype network analysis and bubble plot of *Polites otho* were not possible. Sequences > 599 bp = 13.

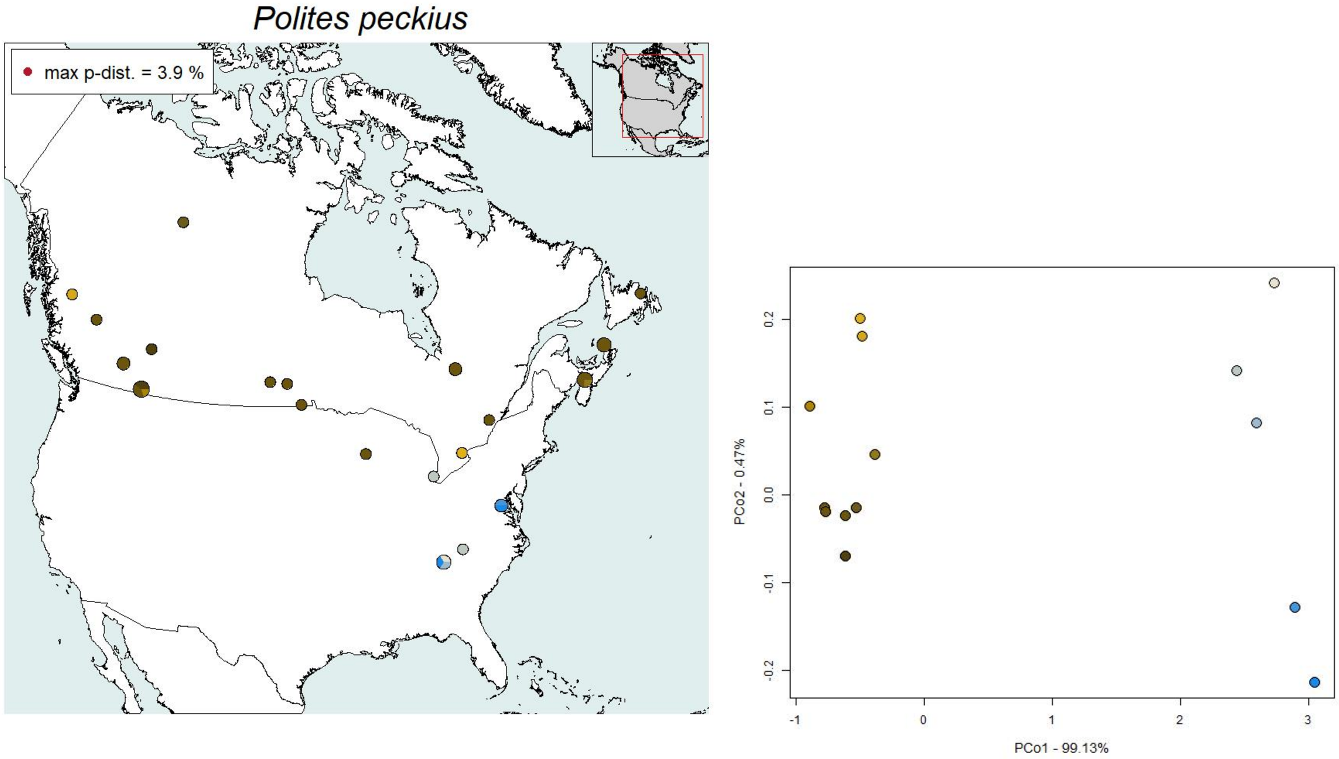

Figure 213 Map of *Polites peckius* showing the localities of the sequenced specimens (left). Nearby localities are grouped in pies. Colours match the bidimensional colour space of the PCoA projection (right) of max p-dists among sequences (dots). Sequences= 34; Hap obs.= 11; Hap asympt.= 34.8; Hap % obs.= 31.6%; GST= 0.911; DST= 0.013; HD= 0.683; ND= 0.0121; max p-dist= 3.9%.

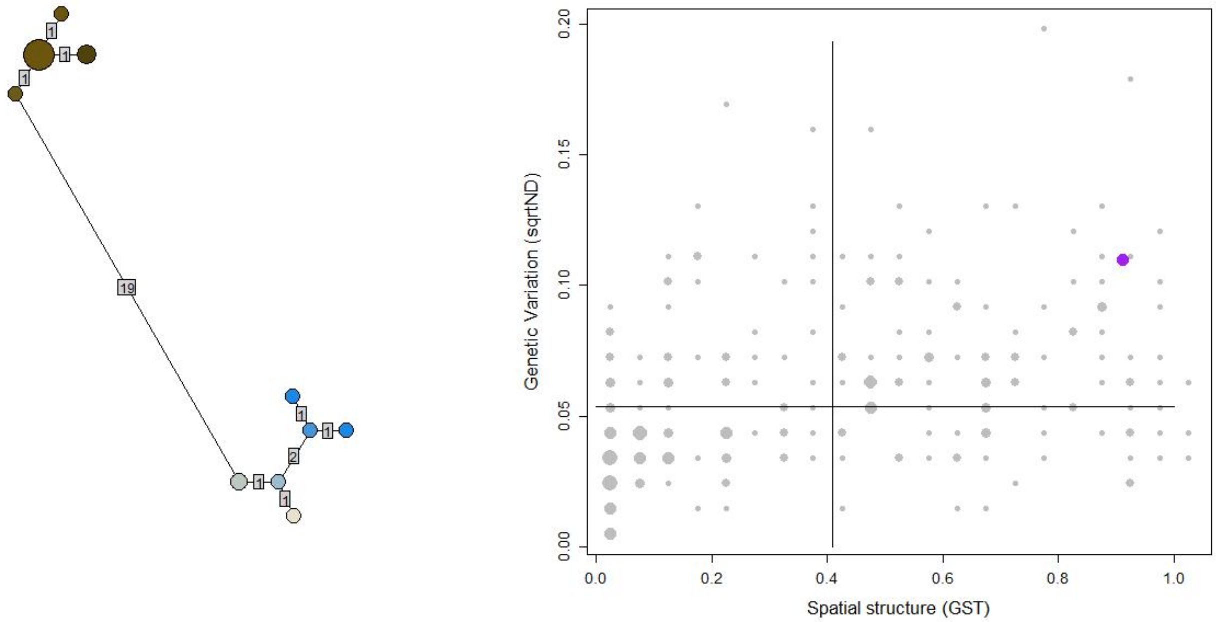

Figure 214: Haplotype network (left) of *Polites peckius* sequences > 599 bp with colours matching the PCoA colour space (above). The bubble plot for mt-DNA polymorphism (square root transformed nucleotide diversity) and spatial structure (GST) among all species in the atlas and values for *Polites peckius* (purple dot). The horizontal and vertical lines represent median values of nucleotide diversity and GST, respectively. Sequences > 599 bp= 32.

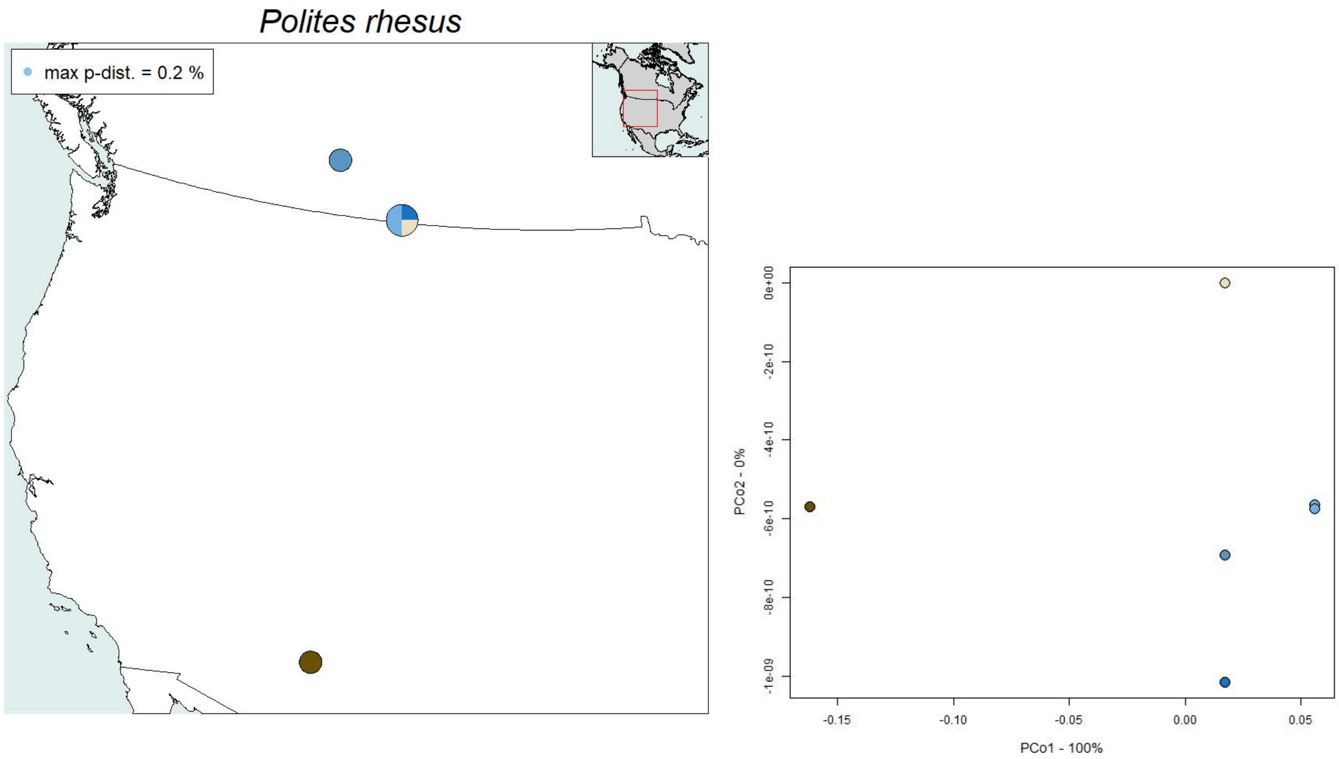

Figure 215 Map of *Polites rhesus* showing the localities of the sequenced specimens (left). Nearby localities are grouped in pies. Colours match the bidimensional colour space of the PCoA projection (right) of max p-dists among sequences (dots). Sequences= 6; Hap obs.= 2; Hap asympt.= NA; Hap % obs.= NA%; GST= NaN; DST= NaN; HD= NA; ND= NA; max p-dist= 0.2%.

Haplotype network analysis and bubble plot of *Polites rhesus* were not possible. Sequences > 599 bp = 3.

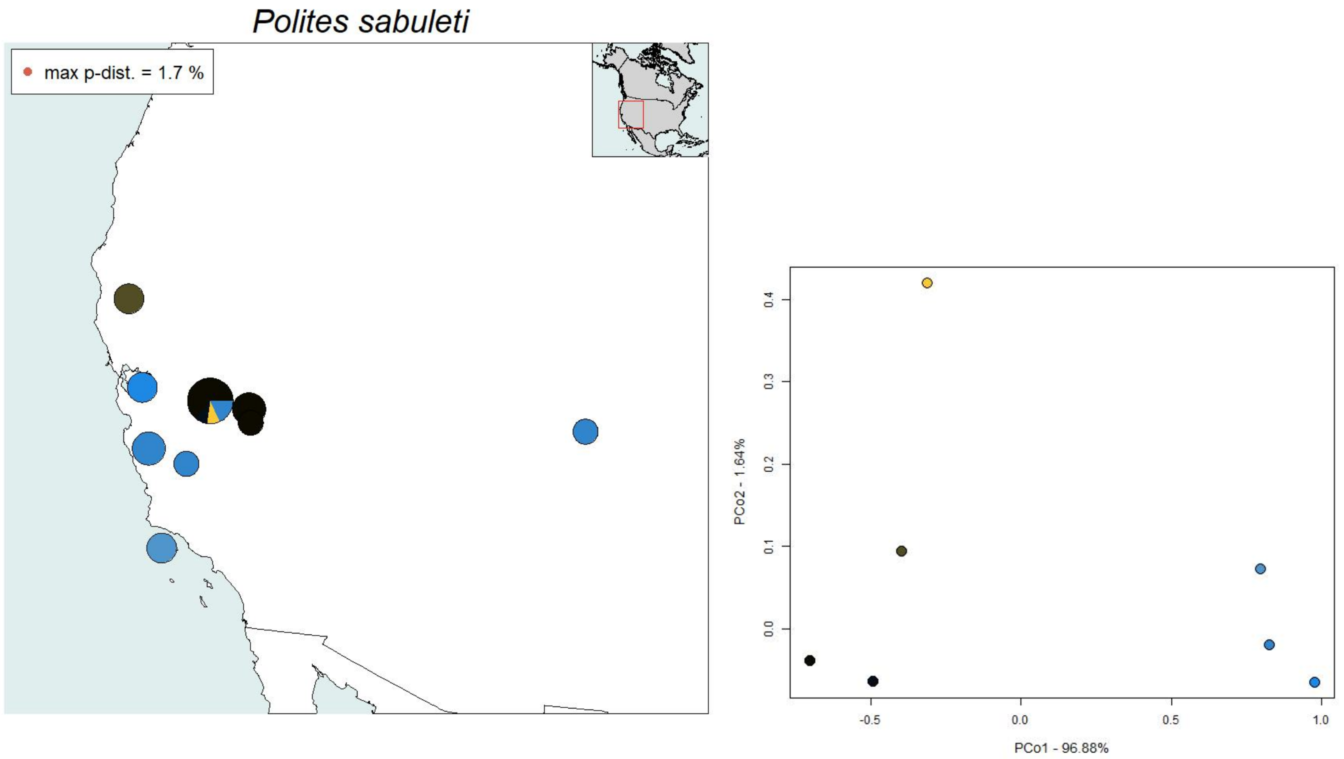

Figure 216 Map of *Polites sabuleti* showing the localities of the sequenced specimens (left). Nearby localities are grouped in pies. Colours match the bidimensional colour space of the PCoA projection (right) of max p-dists among sequences (dots). Sequences= 26; Hap obs.= 7; Hap asympt.= 9.2; Hap % obs.= 76.4%; GST= 0.638; DST= 0.0047; HD= 0.726; ND= 0.0082; max p-dist= 1.7%.

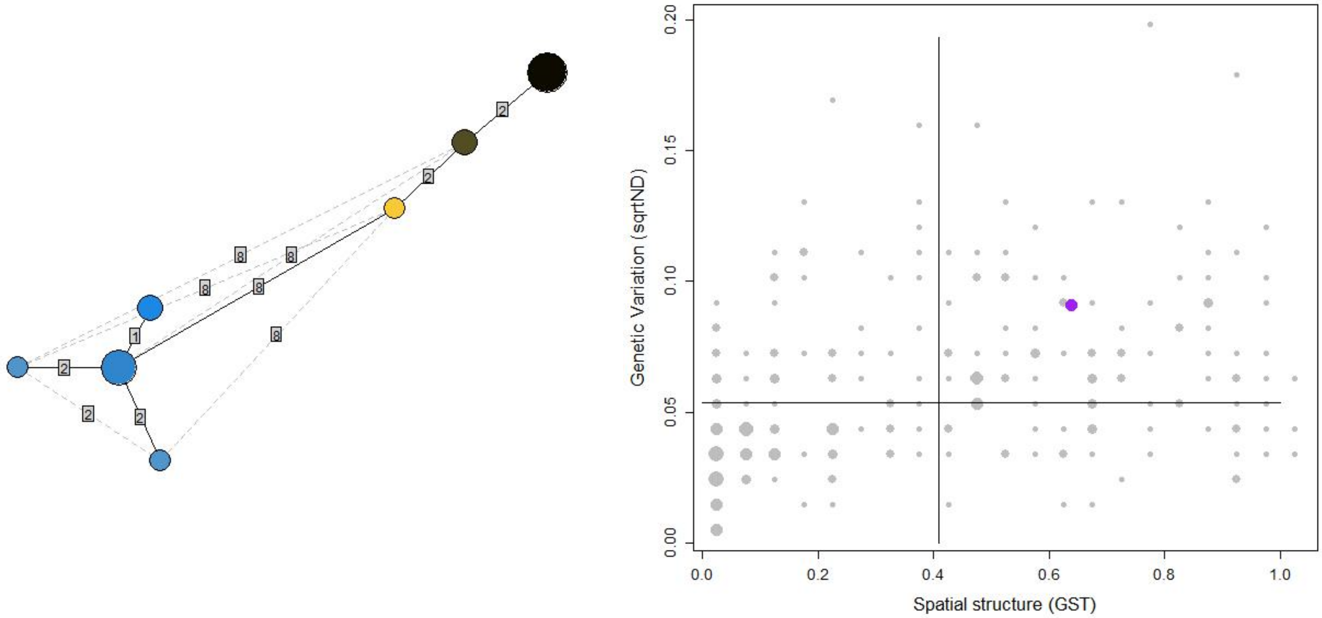

Figure 217: Haplotype network (left) of *Polites sabuleti* sequences > 599 bp with colours matching the PCoA colour space (above). The bubble plot for mt-DNA polymorphism (square root transformed nucleotide diversity) and spatial structure (GST) among all species in the atlas and values for *Polites sabuleti* (purple dot). The horizontal and vertical lines represent median values of nucleotide diversity and GST, respectively. Sequences > 599 bp= 26.

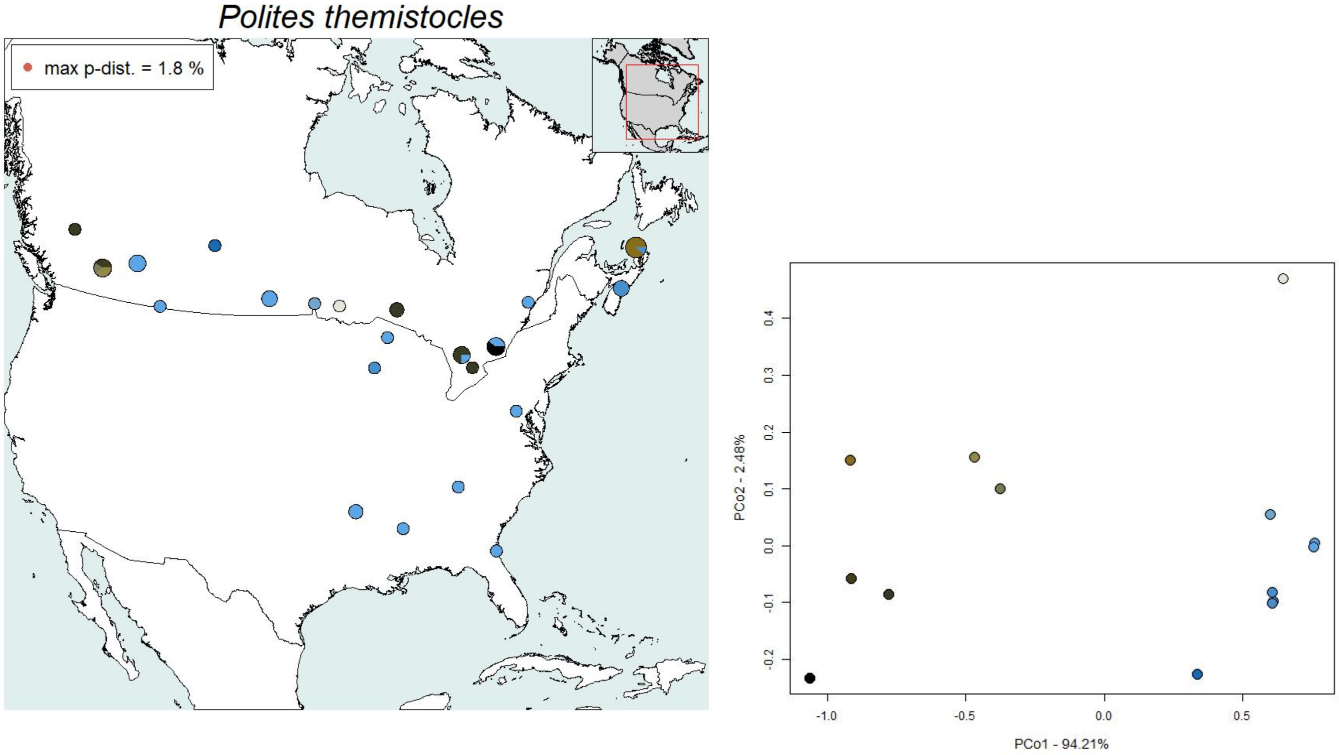

Figure 218 Map of *Polites themistocles* showing the localities of the sequenced specimens (left). Nearby localities are grouped in pies. Colours match the bidimensional colour space of the PCoA projection (right) of max p-dists among sequences (dots). Sequences= 50; Hap obs.= 9; Hap asympt.= 11; Hap % obs.= 82.1%; GST= 0.439; DST= 0.0035; HD= 0.798; ND= 0.0083; max p-dist= 1.8%.

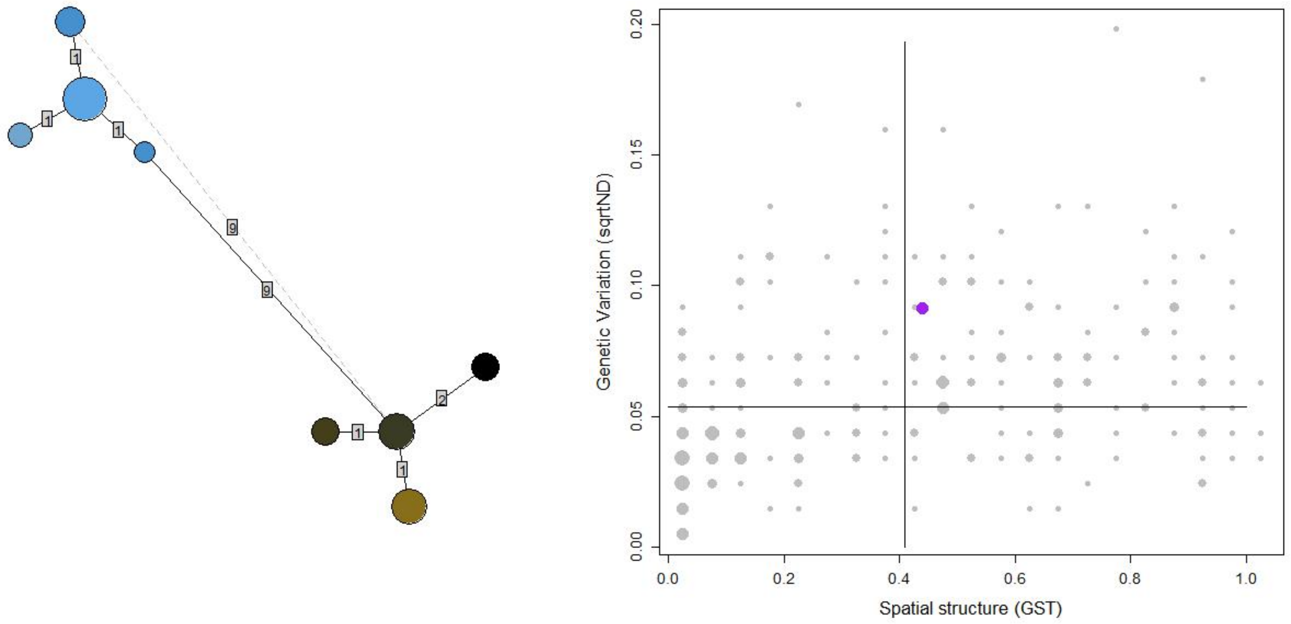

Figure 219: Haplotype network (left) of *Polites themistocles* sequences > 599 bp with colours matching the PCoA colour space (above). The bubble plot for mt-DNA polymorphism (square root transformed nucleotide diversity) and spatial structure (GST) among all species in the atlas and values for *Polites themistocles* (purple dot). The horizontal and vertical lines represent median values of nucleotide diversity and GST, respectively. Sequences > 599 bp= 49.

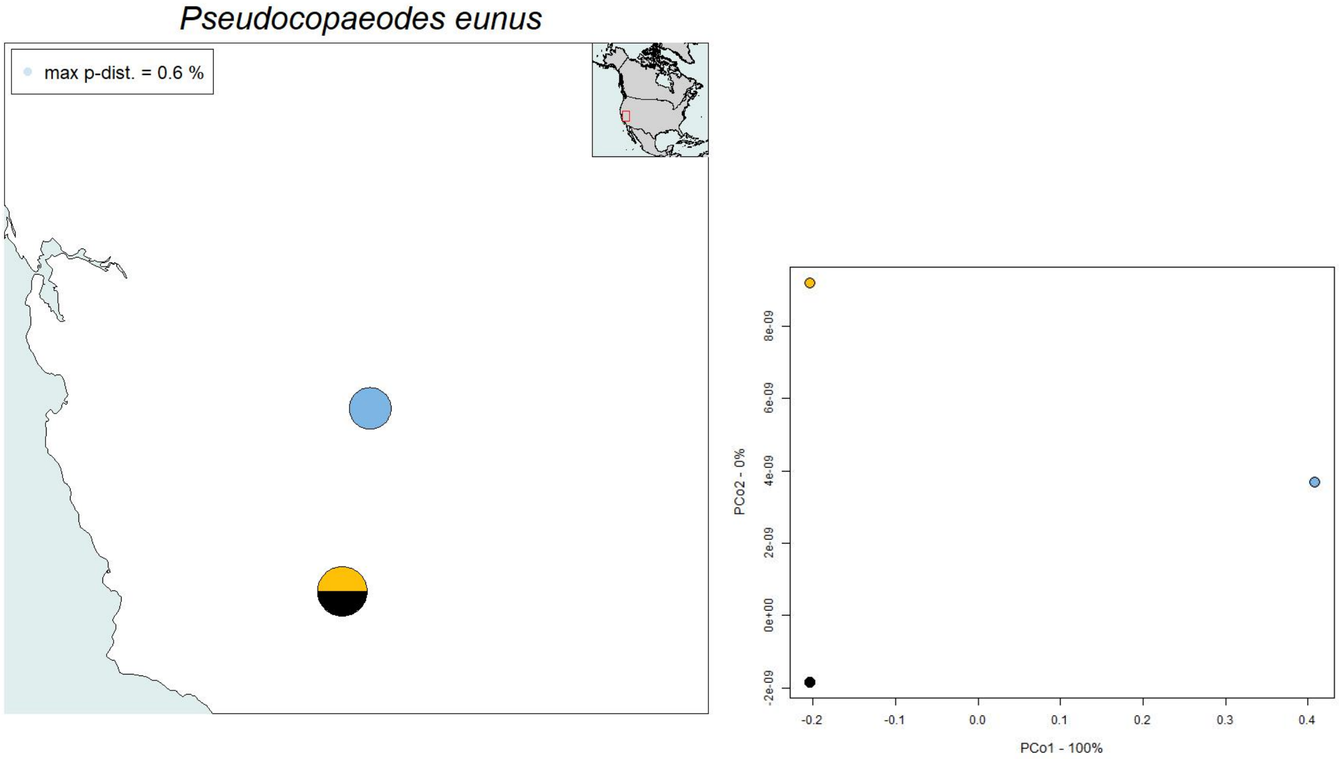

Figure 220 Map of *Pseudocopaeodes eunus* showing the localities of the sequenced specimens (left). Nearby localities are grouped in pies. Colours match the bidimensional colour space of the PCoA projection (right) of max p-dists among sequences (dots). Sequences= 3; Hap obs.= 2; Hap asympt.= NA; Hap % obs.= NA%; GST= NaN; DST= NaN; HD= NA; ND= NA; max p-dist= 0.6%.

Haplotype network analysis and bubble plot of *Pseudocopaeodes eunus* were not possible. Sequences > 599 bp = 3.

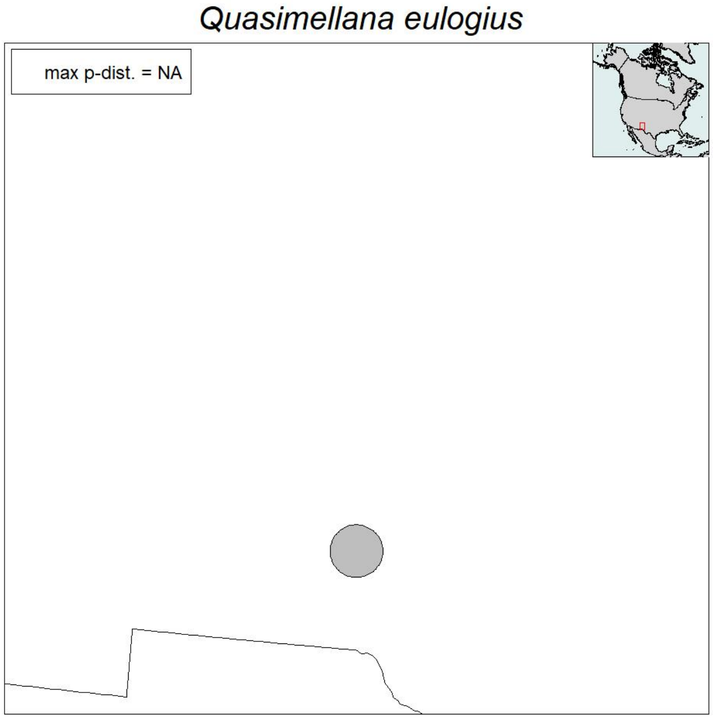

Figure 221: Map of *Quasimellana eulogius* showing the localities of the sequenced specimens. Nearby localities are grouped in pies. Due to the presence of a single haplotype PCoA projection was not done and a single grey colour was plotted on the map. Sequences= 1; Hap obs.= NA; Hap asympt.= NA; Hap % obs.= NA; GST= NaN; DST= NaN; HD= NA; ND= NA; max p-dist= NA.

Haplotype network analysis and bubble plot of *Quasimellana eulogius* were not possible. Sequences > 599 bp = 1.

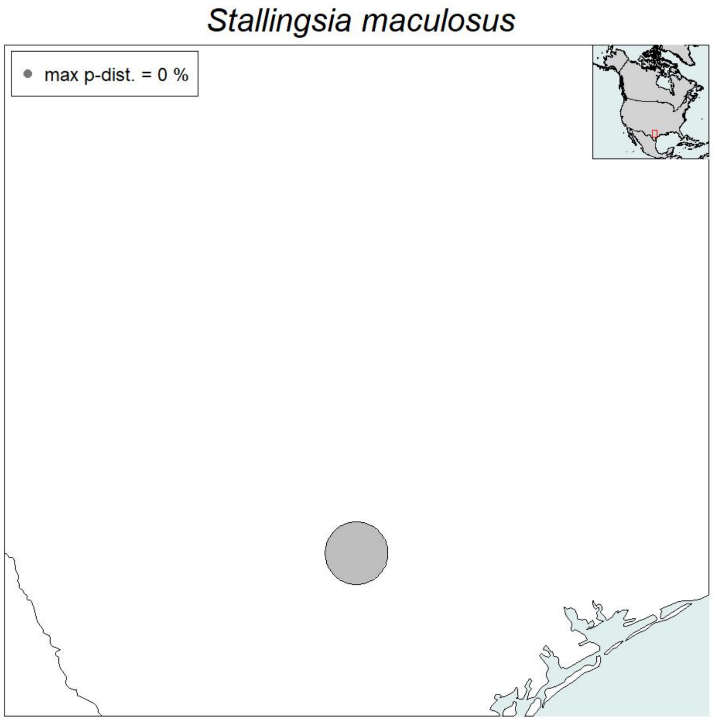

Figure 222: Map of *Stallingsia maculosus* showing the localities of the sequenced specimens. Nearby localities are grouped in pies. Due to the presence of a single haplotype PCoA projection was not done and a single grey colour was plotted on the map. Sequences= 2; Hap obs.= 1; Hap asympt.= NA; Hap % obs.= NA%; GST= NaN; DST= NaN; HD= NA; ND= NA; max p-dist= 0%.

Haplotype network analysis and bubble plot of *Stallingsia maculosus* were not possible. Sequences > 599 bp = 2.

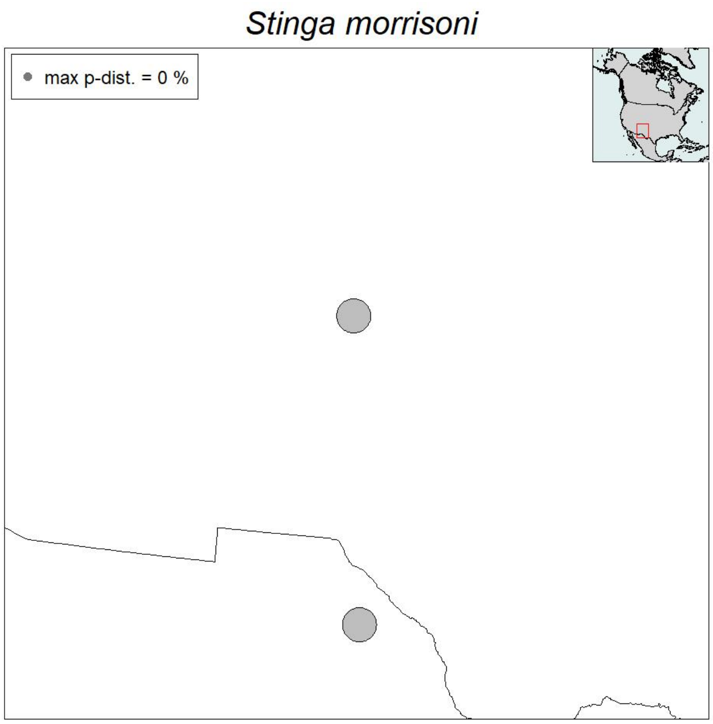

Figure 223: Map of *Stinga morrisoni* showing the localities of the sequenced specimens. Nearby localities are grouped in pies. Due to the presence of a single haplotype PCoA projection was not done and a single grey colour was plotted on the map. Sequences= 2; Hap obs.= 2; Hap asympt.= NA; Hap % obs.= NA%; GST= NaN; DST= NaN; HD= NA; ND= NA; max p-dist= 0%.

Haplotype network analysis and bubble plot of *Stinga morrisoni* were not possible. Sequences > 599 bp = 1.

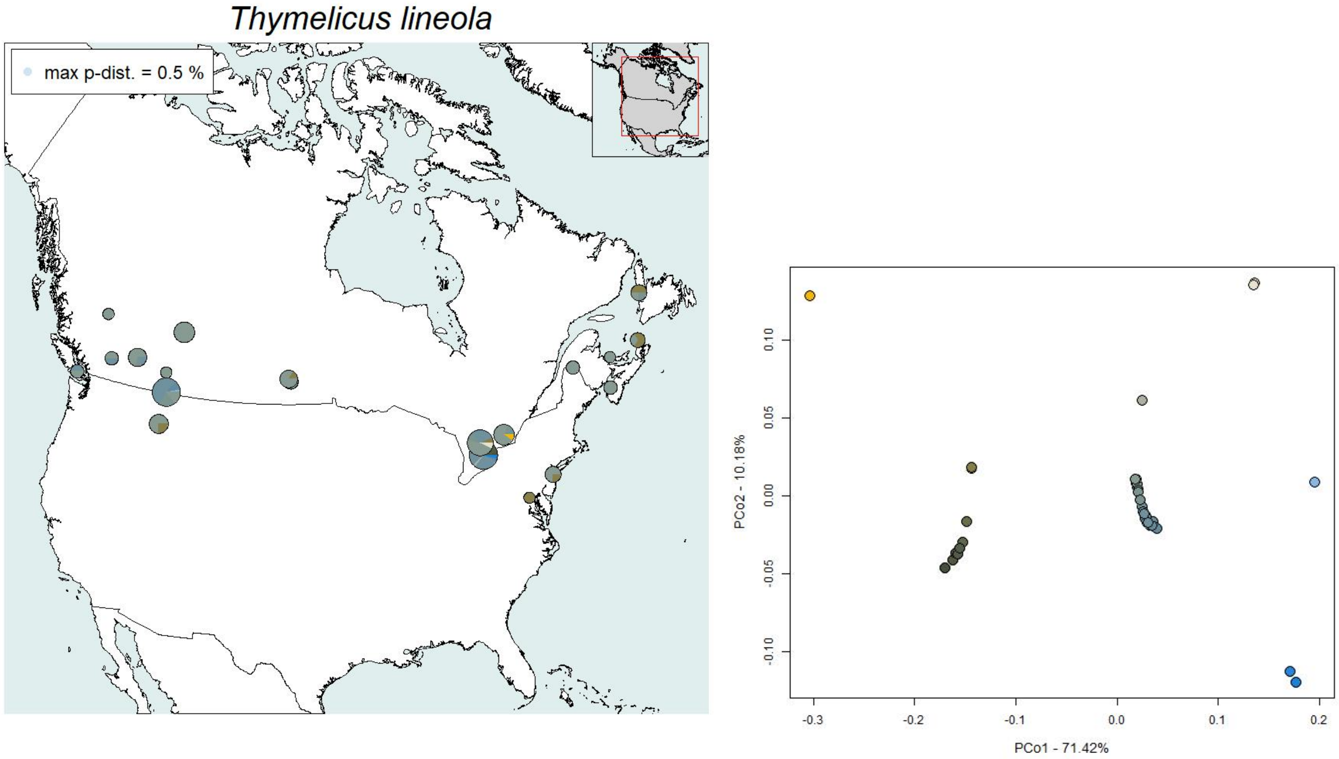

Figure 224 Map of *Thymelicus lineola* showing the localities of the sequenced specimens (left). Nearby localities are grouped in pies. Colours match the bidimensional colour space of the PCoA projection (right) of max p-dists among sequences (dots). Sequences= 176; Hap obs.= 8; Hap asympt.= 20.4; Hap % obs.= 39.2%; GST= 0.047; DST= 0; HD= 0.312; ND= 0.0005; max p-dist= 0.5%.

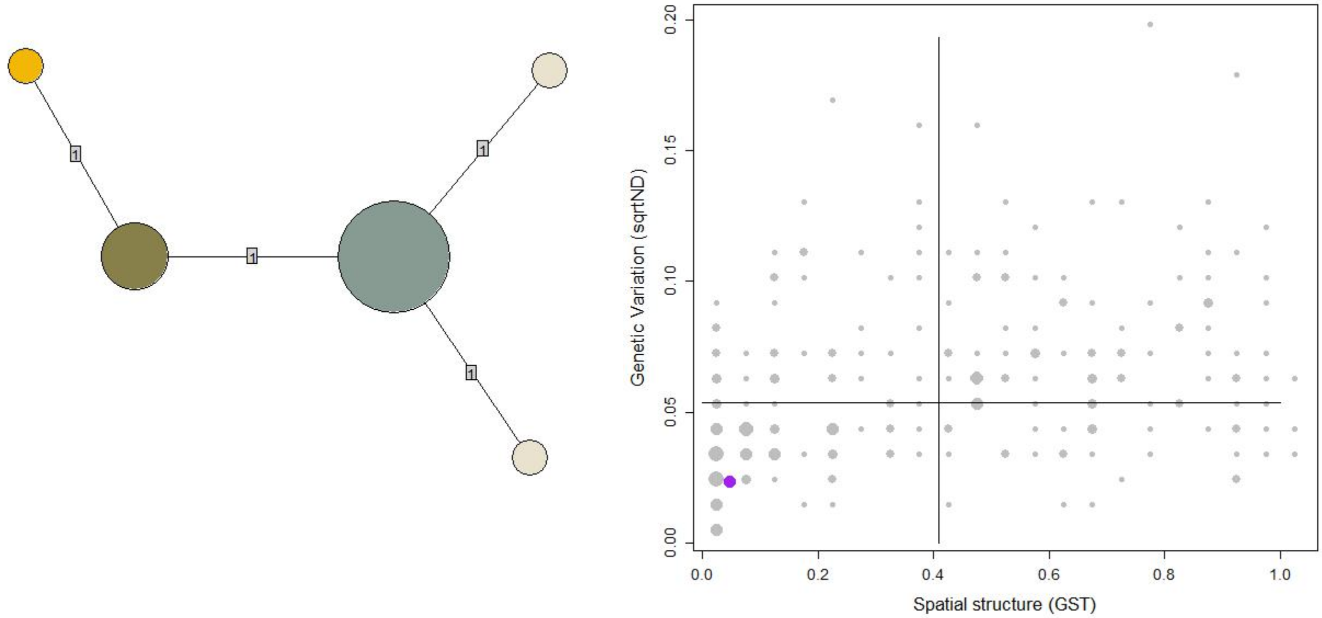

Figure 225: Haplotype network (left) of *Thymelicus lineola* sequences > 599 bp with colours matching the PCoA colour space (above). The bubble plot for mt-DNA polymorphism (square root transformed nucleotide diversity) and spatial structure (GST) among all species in the atlas and values for *Thymelicus lineola* (purple dot). The horizontal and vertical lines represent median values of nucleotide diversity and GST, respectively. Sequences > 599 bp= 105.

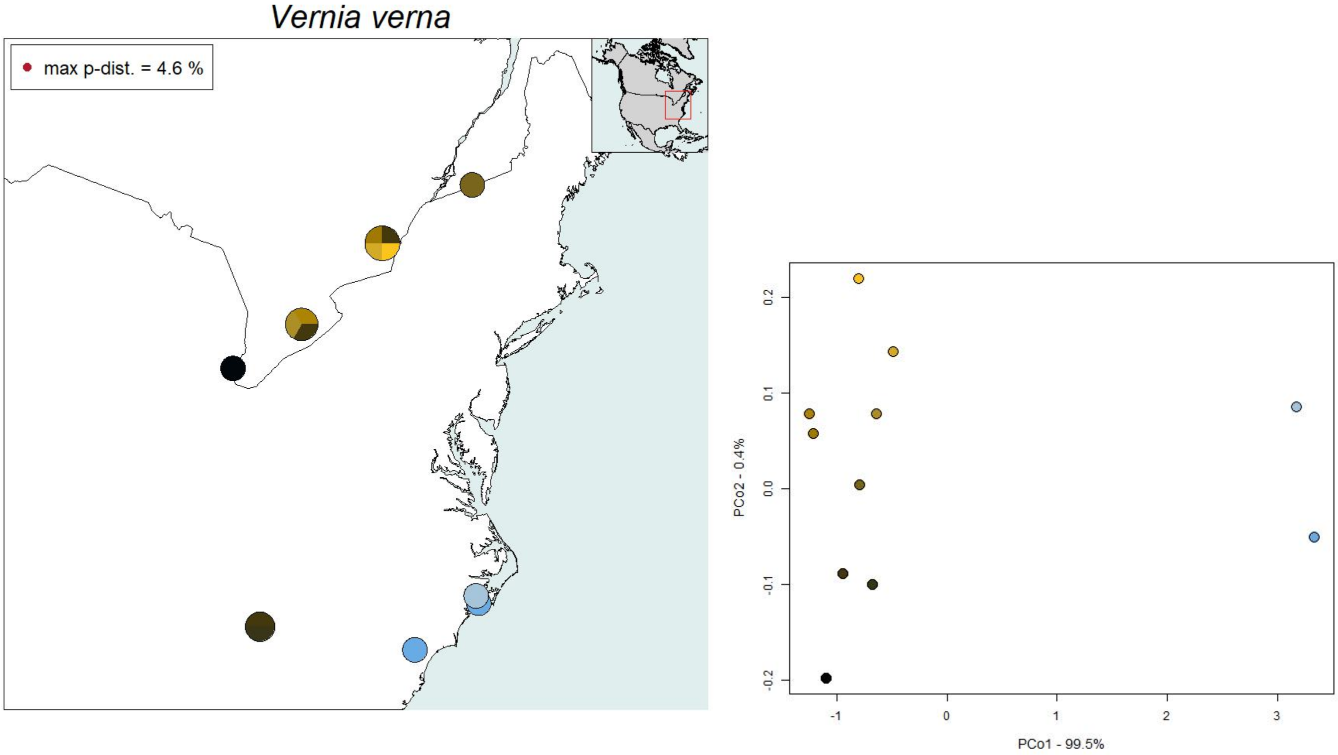

Figure 226 Map of *Vernia verna* showing the localities of the sequenced specimens (left). Nearby localities are grouped in pies. Colours match the bidimensional colour space of the PCoA projection (right) of max p-dists among sequences (dots). Sequences= 14; Hap obs.= 8; Hap asympt.= 13.8; Hap % obs.= 58%; GST= 0.886; DST= 0.0172; HD= 0.868; ND= 0.0164; max p-dist= 4.6%.

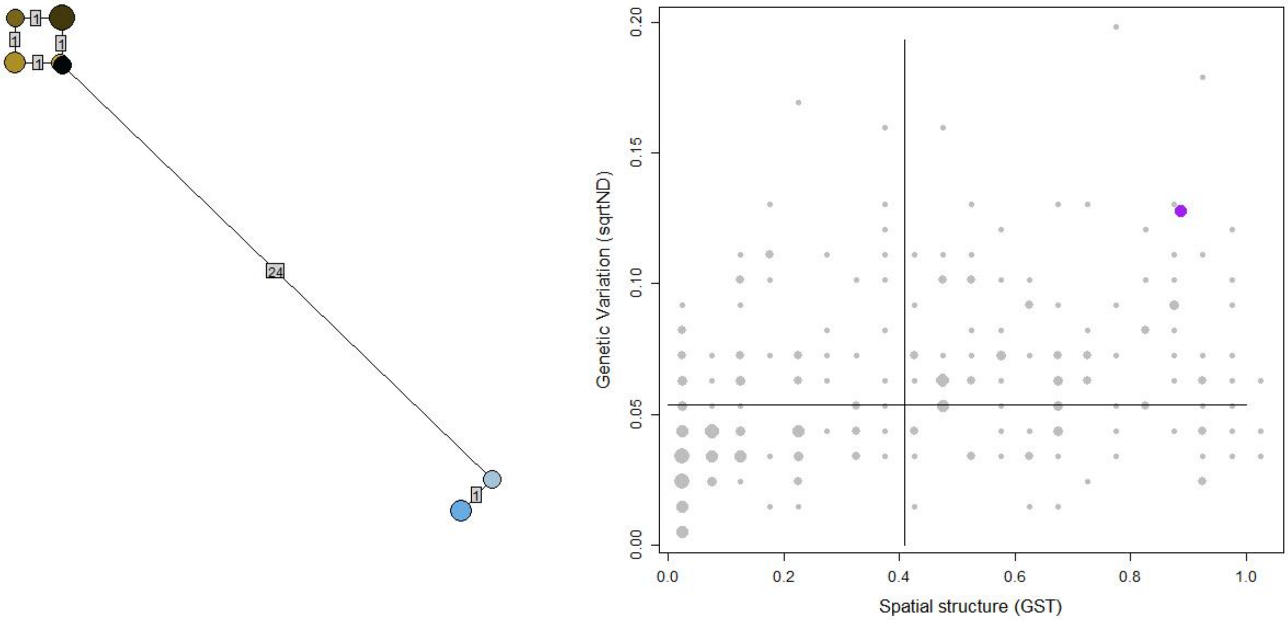

Figure 227: Haplotype network (left) of *Vernia verna* sequences > 599 bp with colours matching the PCoA colour space (above). The bubble plot for mt-DNA polymorphism (square root transformed nucleotide diversity) and spatial structure (GST) among all species in the atlas and values for *Vernia verna* (purple dot). The horizontal and vertical lines represent median values of nucleotide diversity and GST, respectively. Sequences > 599 bp= 12.

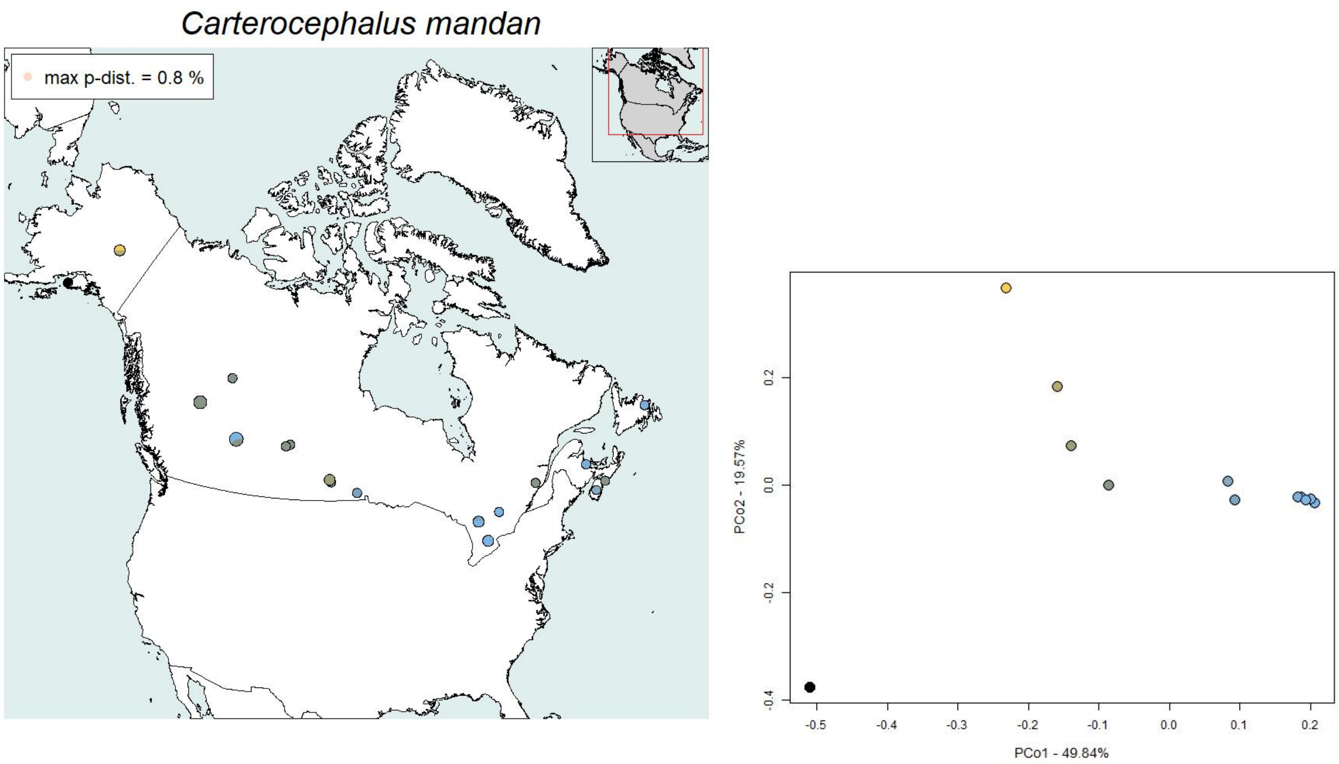

Figure 228 Map of *Carterocephalus mandan* showing the localities of the sequenced specimens (left). Nearby localities are grouped in pies. Colours match the bidimensional colour space of the PCoA projection (right) of max p-dists among sequences (dots). Sequences= 29; Hap obs.= 11; Hap asympt.= 17; Hap % obs.= 64.6%; GST= 0.351; DST= 0.0008; HD= 0.837; ND= 0.0026; max p-dist= 0.8%.

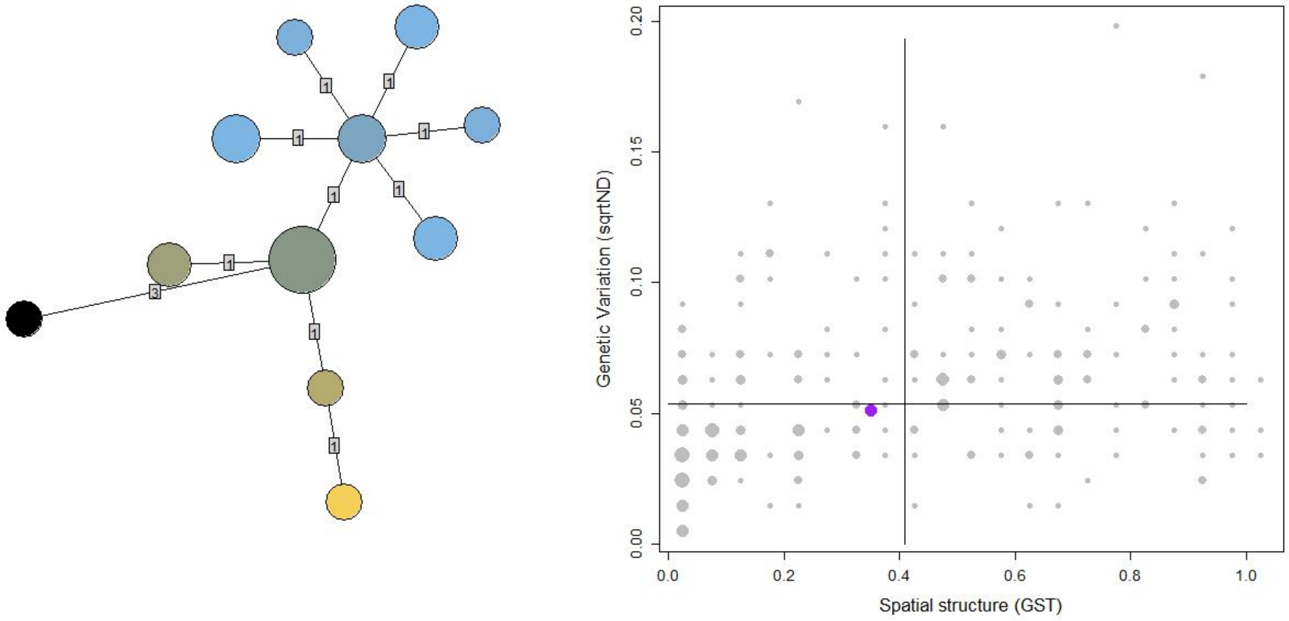

Figure 229: Haplotype network (left) of *Carterocephalus mandan* sequences > 599 bp with colours matching the PCoA colour space (above). The bubble plot for mt-DNA polymorphism (square root transformed nucleotide diversity) and spatial structure (GST) among all species in the atlas and values for *Carterocephalus mandan* (purple dot). The horizontal and vertical lines represent median values of nucleotide diversity and GST, respectively. Sequences > 599 bp= 28.

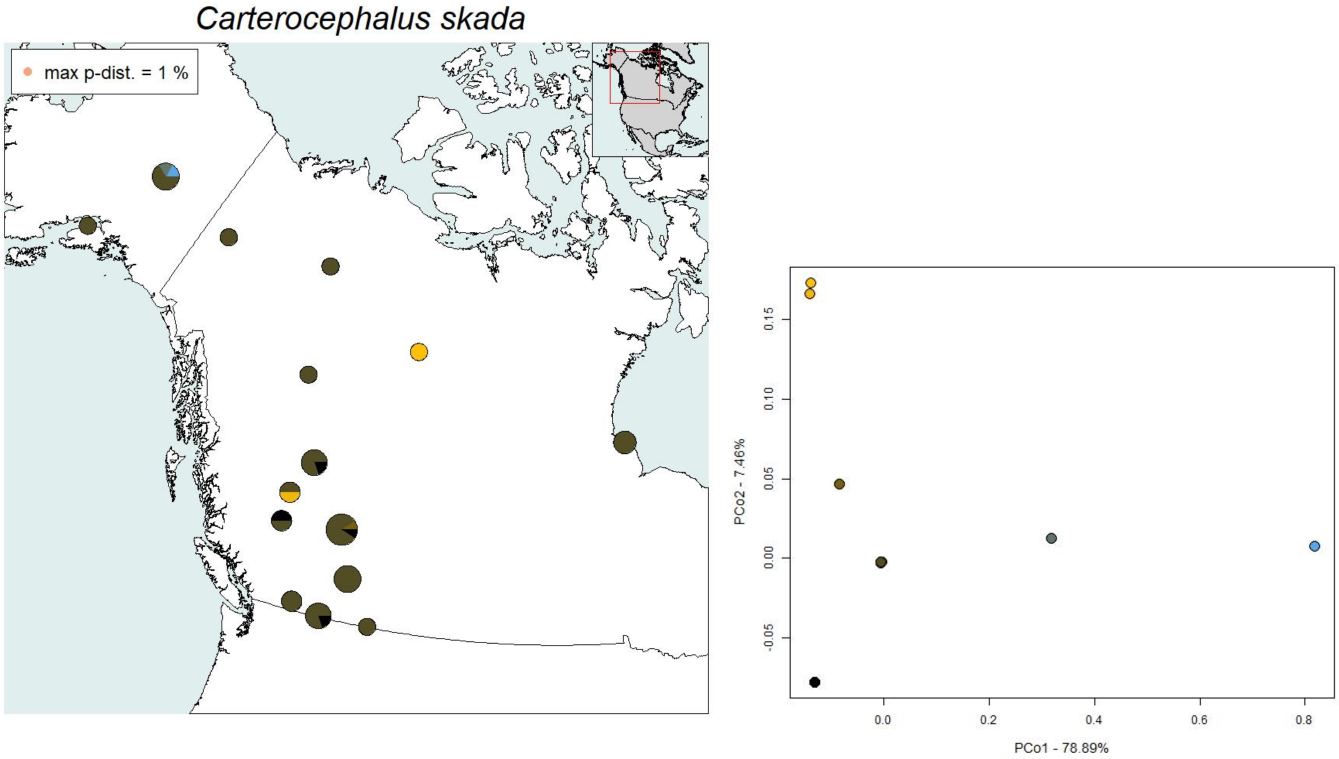

Figure 230 Map of *Carterocephalus skada* showing the localities of the sequenced specimens (left). Nearby localities are grouped in pies. Colours match the bidimensional colour space of the PCoA projection (right) of max p-dists among sequences (dots). Sequences= 48; Hap obs.= 9; Hap asympt.= 33; Hap % obs.= 27.3%; GST= 0.115; DST= 0.0001; HD= 0.342; ND= 0.0008; max p-dist= 1%.

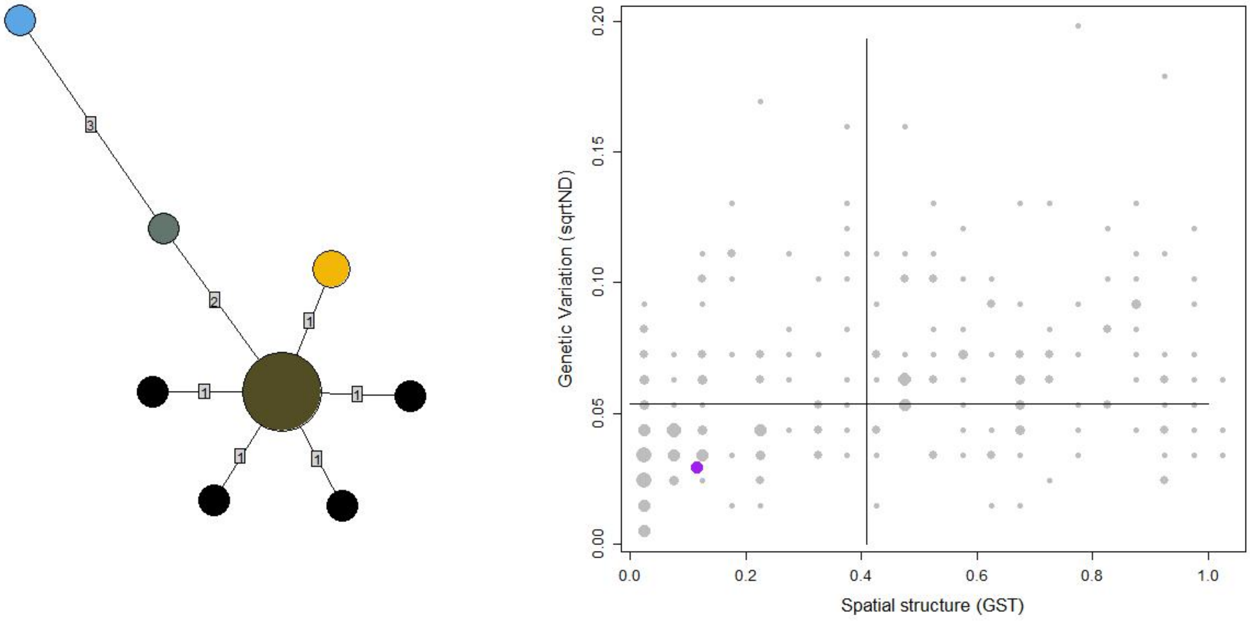

Figure 231: Haplotype network (left) of *Carterocephalus skada* sequences > 599 bp with colours matching the PCoA colour space (above). The bubble plot for mt-DNA polymorphism (square root transformed nucleotide diversity) and spatial structure (GST) among all species in the atlas and values for *Carterocephalus skada* (purple dot). The horizontal and vertical lines represent median values of nucleotide diversity and GST, respectively. Sequences > 599 bp= 47.

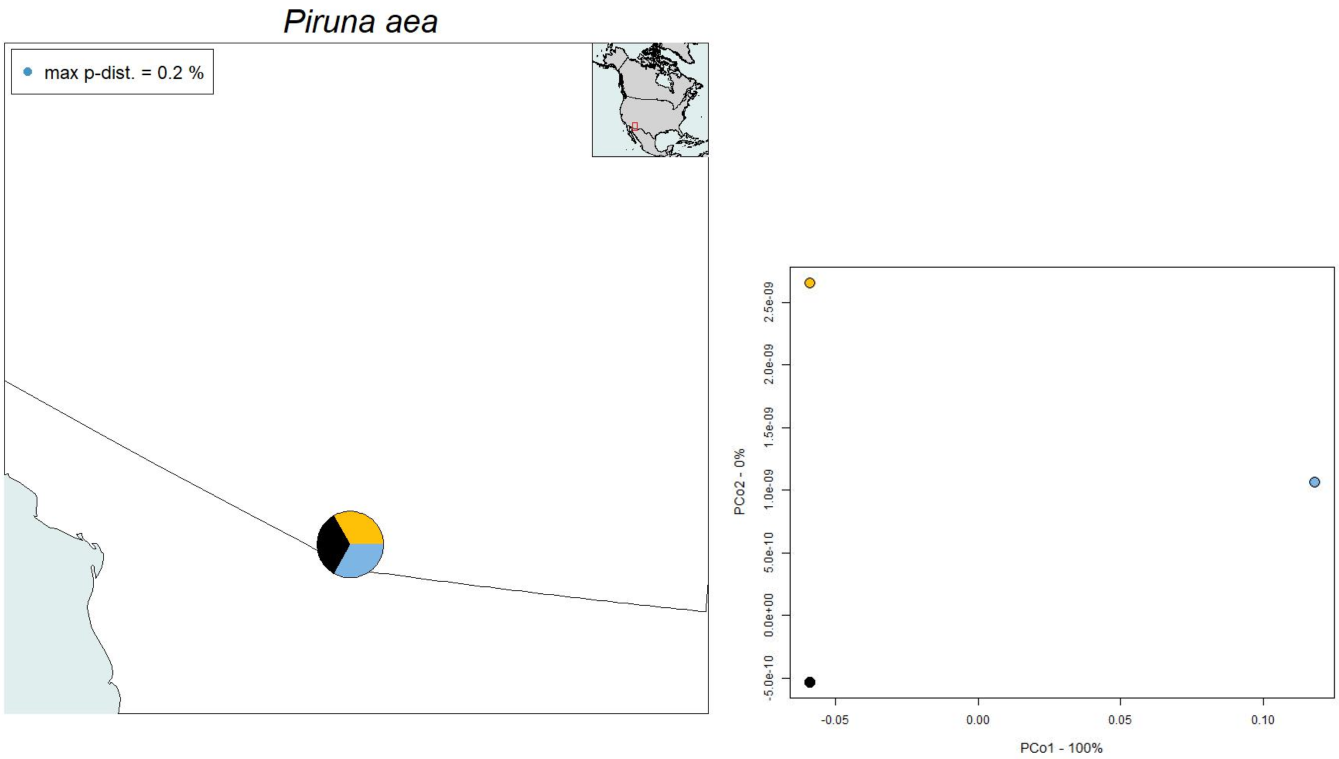

Figure 232 Map of *Piruna aea* showing the localities of the sequenced specimens (left). Nearby localities are grouped in pies. Colours match the bidimensional colour space of the PCoA projection (right) of max p-dists among sequences (dots). Sequences= 3; Hap obs.= 2; Hap asympt.= NA; Hap % obs.= NA%; GST= NaN; DST= NaN; HD= NA; ND= NA; max p-dist= 0.2%.

Haplotype network analysis and bubble plot of *Piruna aea* were not possible. Sequences > 599 bp = 2.

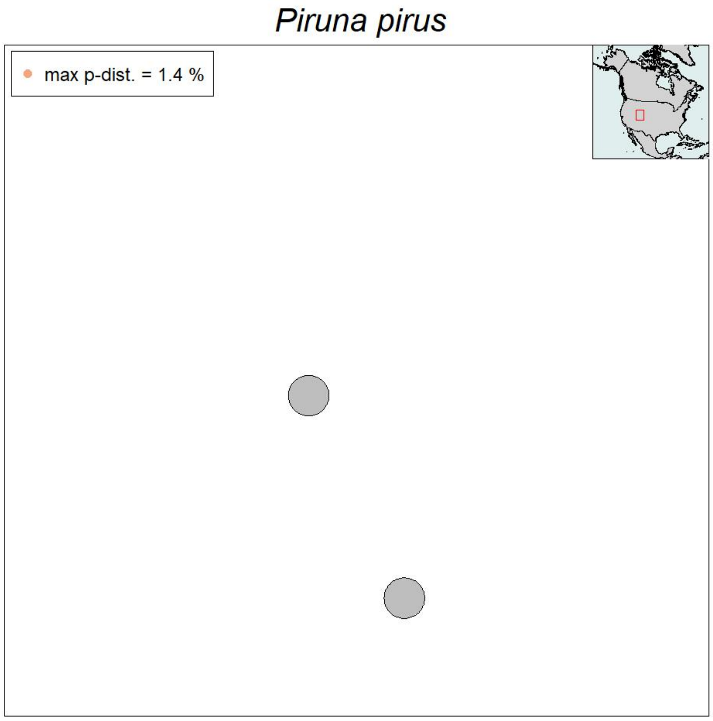

Figure 233: Map of *Piruna pirus* showing the localities of the sequenced specimens. Nearby localities are grouped in pies. Due to the presence of a single haplotype PCoA projection was not done and a single grey colour was plotted on the map. Sequences= 2; Hap obs.= 2; Hap asympt.= NA; Hap % obs.= NA%; GST= NaN; DST= NaN; HD= NA; ND= NA; max p-dist= 1.4%.

Haplotype network analysis and bubble plot of *Piruna pirus* were not possible. Sequences > 599 bp = 1.

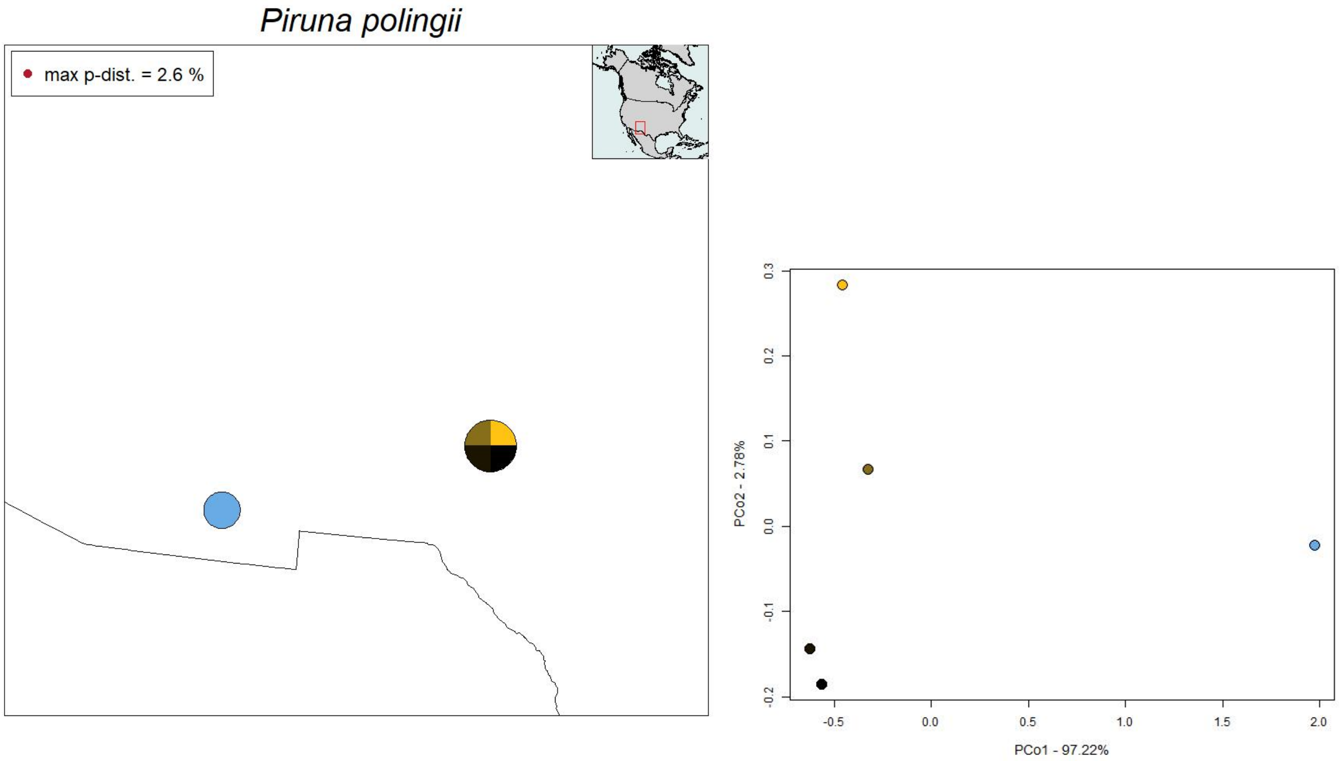

Figure 234 Map of *Piruna polingii* showing the localities of the sequenced specimens (left). Nearby localities are grouped in pies. Colours match the bidimensional colour space of the PCoA projection (right) of max p-dists among sequences (dots). Sequences= 5; Hap obs.= 4; Hap asympt.= NA; Hap % obs.= NA%; GST= NaN; DST= NaN; HD= NA; ND= NA; max p-dist= 2.6%.

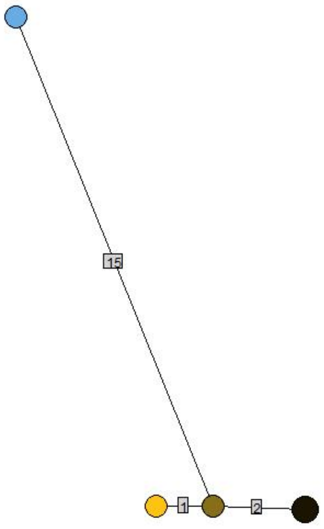

Figure 235: Haplotype network of *Piruna polingii.* Sequences > 599 bp= 5.

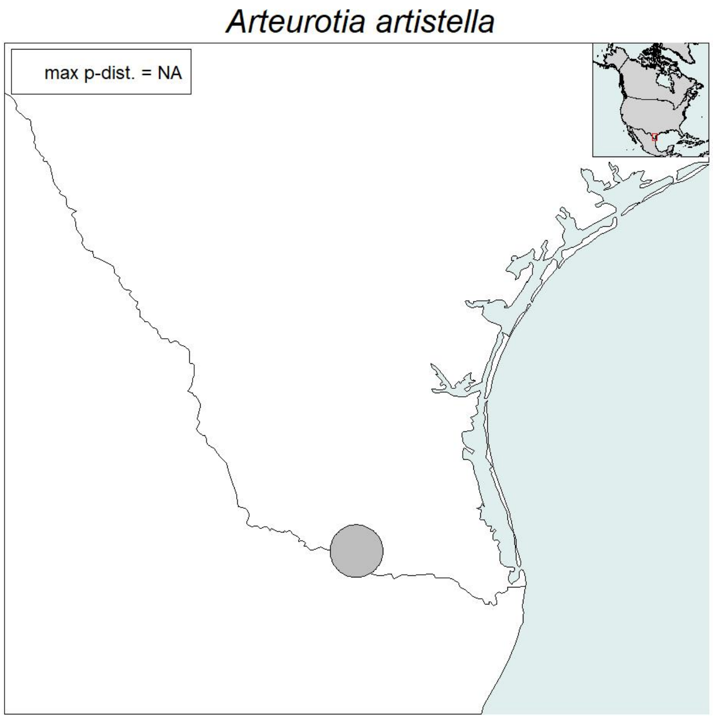

Figure 236: Map of *Arteurotia artistella* showing the localities of the sequenced specimens. Nearby localities are grouped in pies. Due to the presence of a single haplotype PCoA projection was not done and a single grey colour was plotted on the map. Sequences= 1; Hap obs.= NA; Hap asympt.= NA; Hap % obs.= NA; GST= NaN; DST= NaN; HD= NA; ND= NA; max p-dist= NA.

Haplotype network analysis and bubble plot of *Arteurotia artistella* were not possible. Sequences > 599 bp = 1.

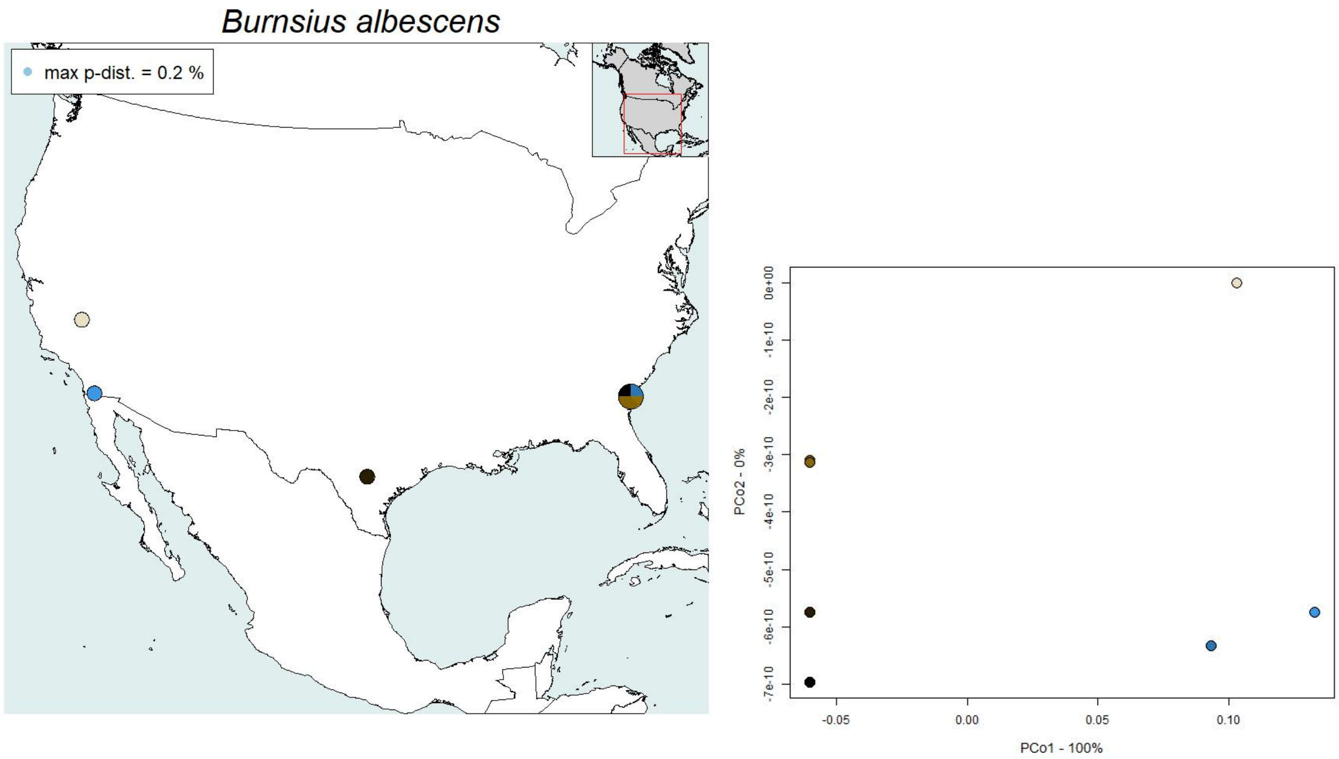

Figure 237 Map of *Burnsius albescens* showing the localities of the sequenced specimens (left). Nearby localities are grouped in pies. Colours match the bidimensional colour space of the PCoA projection (right) of max p-dists among sequences (dots). Sequences= 11; Hap obs.= 2; Hap asympt.= 2; Hap % obs.= 100%; GST= NaN; DST= NaN; HD= 0.509; ND= 0.0008; max p-dist= 0.2%.

Haplotype network analysis and bubble plot of *Burnsius albescens* were not possible. Sequences > 599 bp = 10.

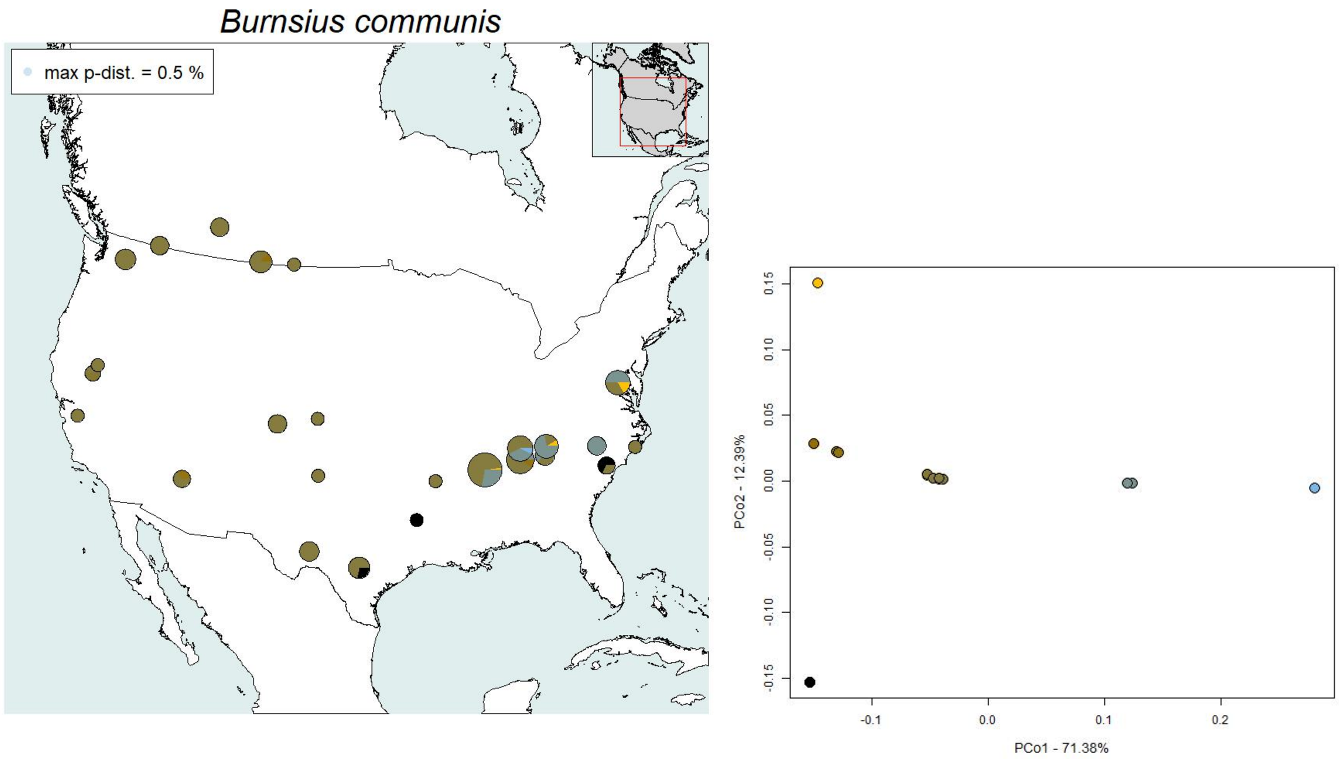

Figure 238 Map of *Burnsius communis* showing the localities of the sequenced specimens (left). Nearby localities are grouped in pies. Colours match the bidimensional colour space of the PCoA projection (right) of max p-dists among sequences (dots). Sequences= 166; Hap obs.= 10; Hap asympt.= 22.4; Hap % obs.= 44.6%; GST= 0.122; DST= 0.0001; HD= 0.54; ND= 0.0009; max p-dist= 0.5%.

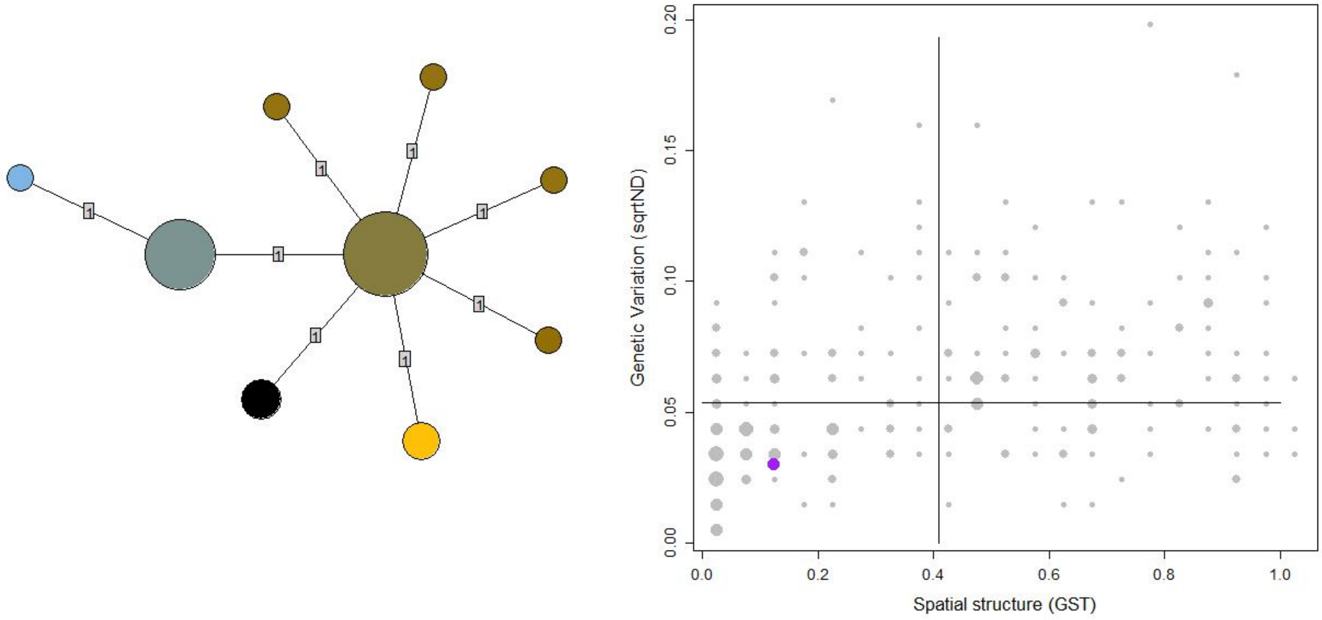

Figure 239: Haplotype network (left) of *Burnsius communis* sequences > 599 bp with colours matching the PCoA colour space (above). The bubble plot for mt-DNA polymorphism (square root transformed nucleotide diversity) and spatial structure (GST) among all species in the atlas and values for *Burnsius communis* (purple dot). The horizontal and vertical lines represent median values of nucleotide diversity and GST, respectively. Sequences > 599 bp= 164.

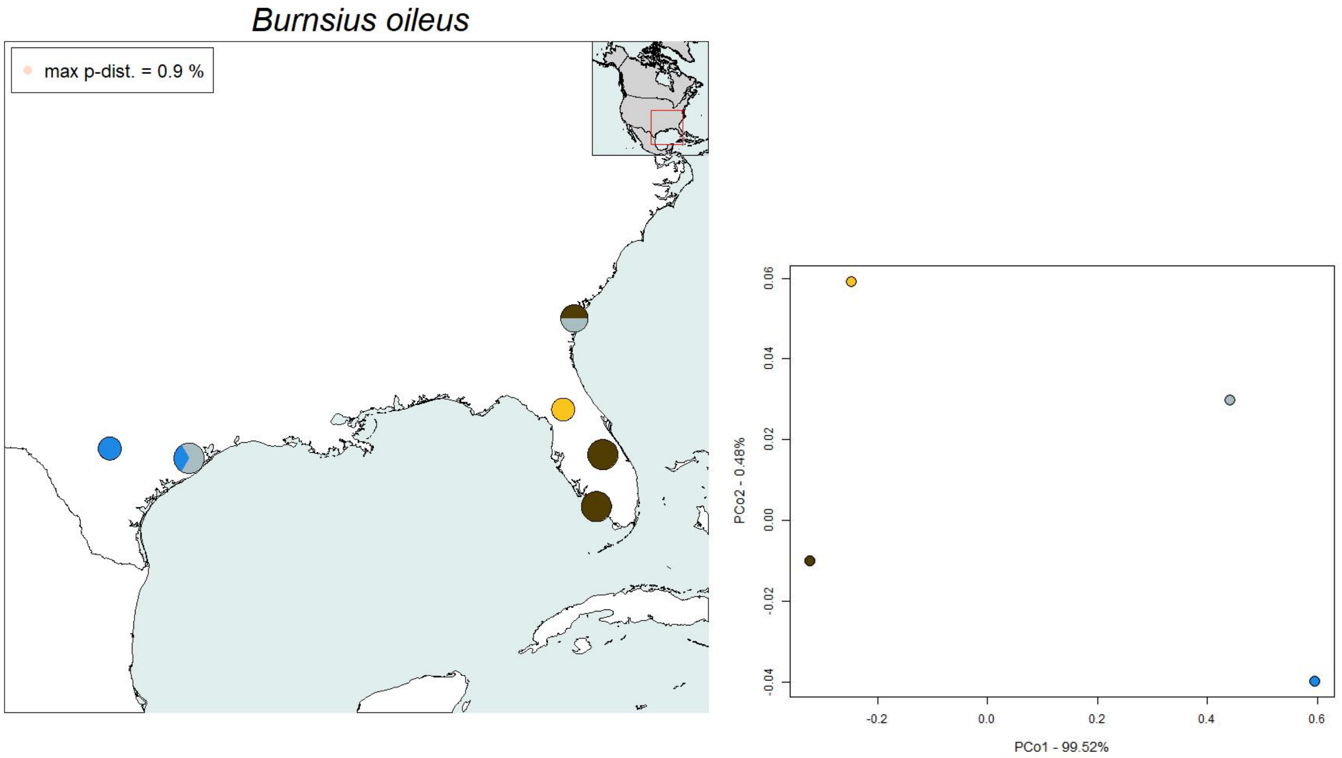

Figure 240 Map of *Burnsius oileus* showing the localities of the sequenced specimens (left). Nearby localities are grouped in pies. Colours match the bidimensional colour space of the PCoA projection (right) of max p-dists among sequences (dots). Sequences= 13; Hap obs.= 3; Hap asympt.= 3; Hap % obs.= 100%; GST= 0.92; DST= 0.0037; HD= 0.59; ND= 0.0043; max p-dist= 0.9%.

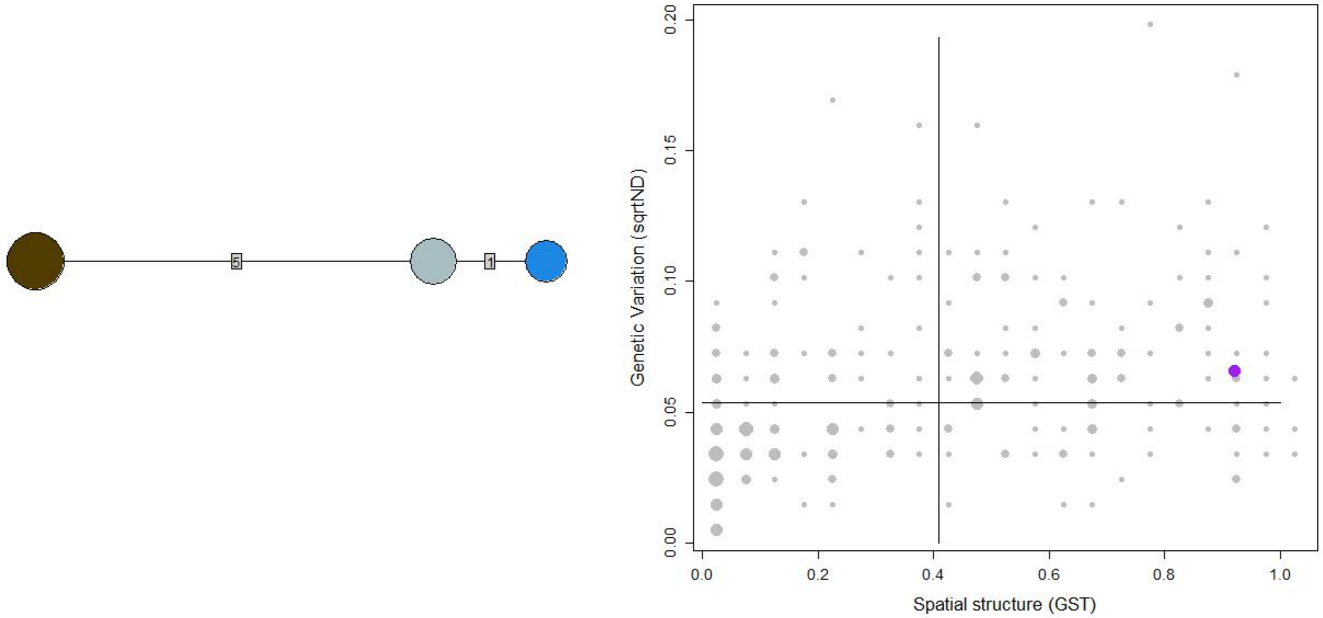

Figure 241: Haplotype network (left) of *Burnsius oileus* sequences > 599 bp with colours matching the PCoA colour space (above). The bubble plot for mt-DNA polymorphism (square root transformed nucleotide diversity) and spatial structure (GST) among all species in the atlas and values for *Burnsius oileus* (purple dot). The horizontal and vertical lines represent median values of nucleotide diversity and GST, respectively. Sequences > 599 bp= 12.

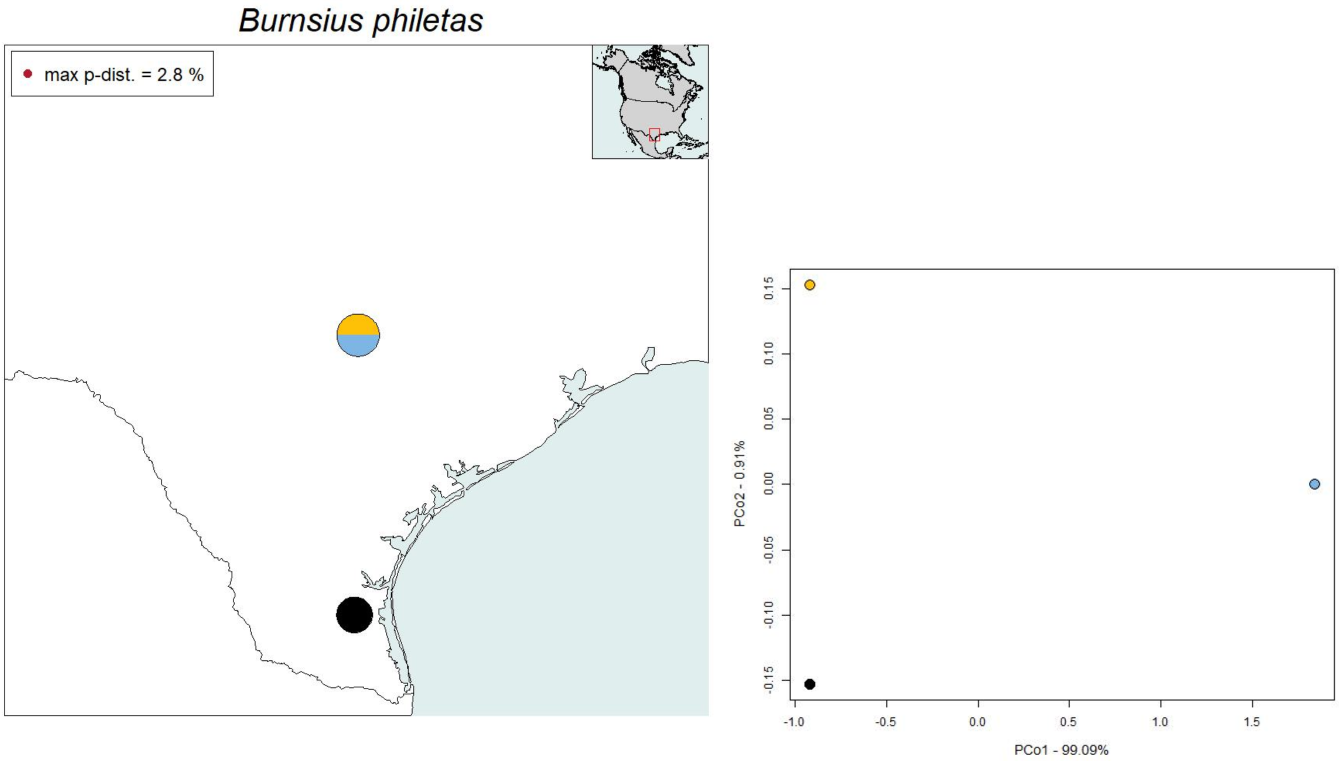

Figure 242 Map of *Burnsius philetas* showing the localities of the sequenced specimens (left). Nearby localities are grouped in pies. Colours match the bidimensional colour space of the PCoA projection (right) of max p-dists among sequences (dots). Sequences= 3; Hap obs.= 3; Hap asympt.= NA; Hap % obs.= NA%; GST= NaN; DST= NaN; HD= NA; ND= NA; max p-dist= 2.8%.

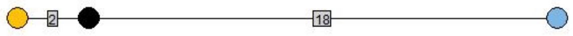

Figure 243: Haplotype network of *Burnsius philetas*. Sequences > 599 bp= 3.

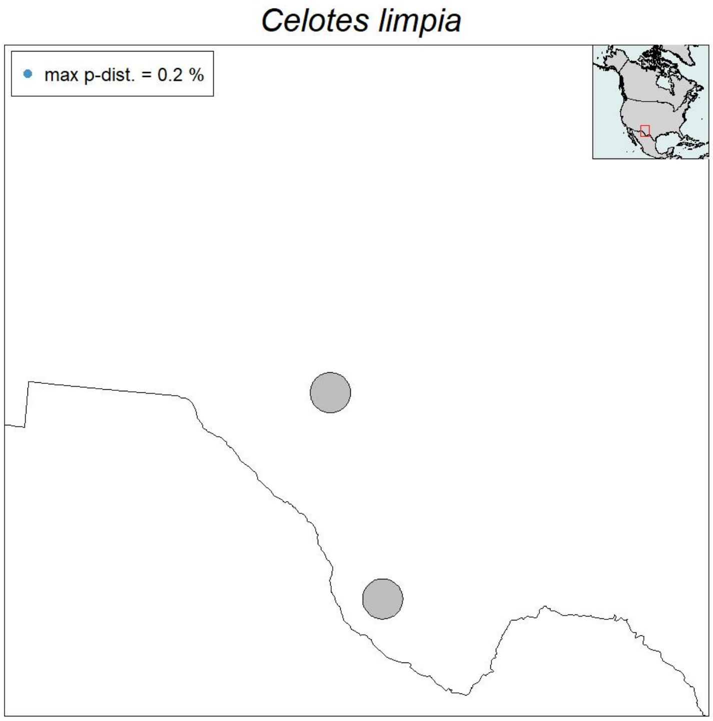

Figure 244: Map of *Celotes limpia* showing the localities of the sequenced specimens. Nearby localities are grouped in pies. Due to the presence of a single haplotype PCoA projection was not done and a single grey colour was plotted on the map. Sequences= 2; Hap obs.= 2; Hap asympt.= NA; Hap % obs.= NA%; GST= NaN; DST= NaN; HD= NA; ND= NA; max p-dist= 0.2%.

Haplotype network analysis and bubble plot of *Celotes limpia* were not possible. Sequences > 599 bp = 2.

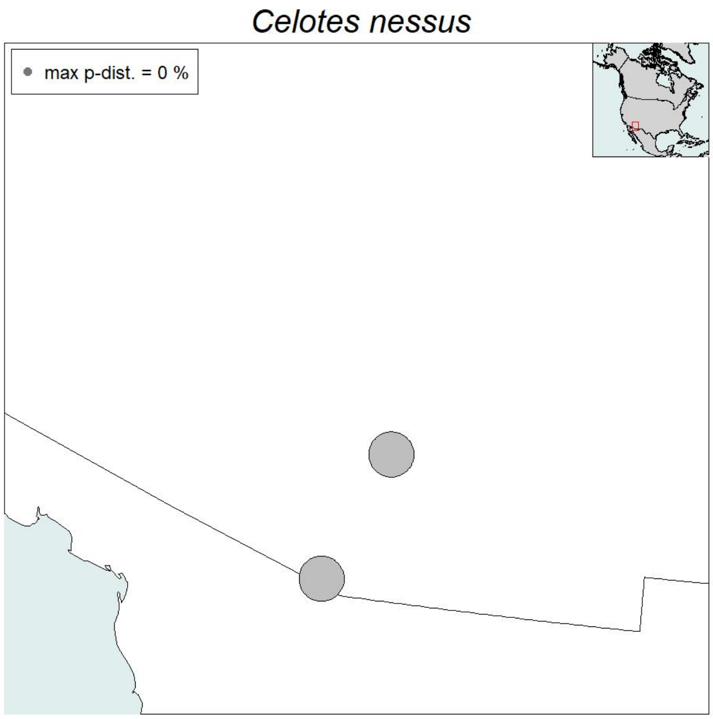

Figure 245: Map of *Celotes nessus* showing the localities of the sequenced specimens. Nearby localities are grouped in pies. Due to the presence of a single haplotype PCoA projection was not done and a single grey colour was plotted on the map. Sequences= 2; Hap obs.= 1; Hap asympt.= NA; Hap % obs.= NA%; GST= NaN; DST= NaN; HD= NA; ND= NA; max p-dist= 0%.

Haplotype network analysis and bubble plot of *Celotes nessus* were not possible. Sequences > 599 bp = 2.

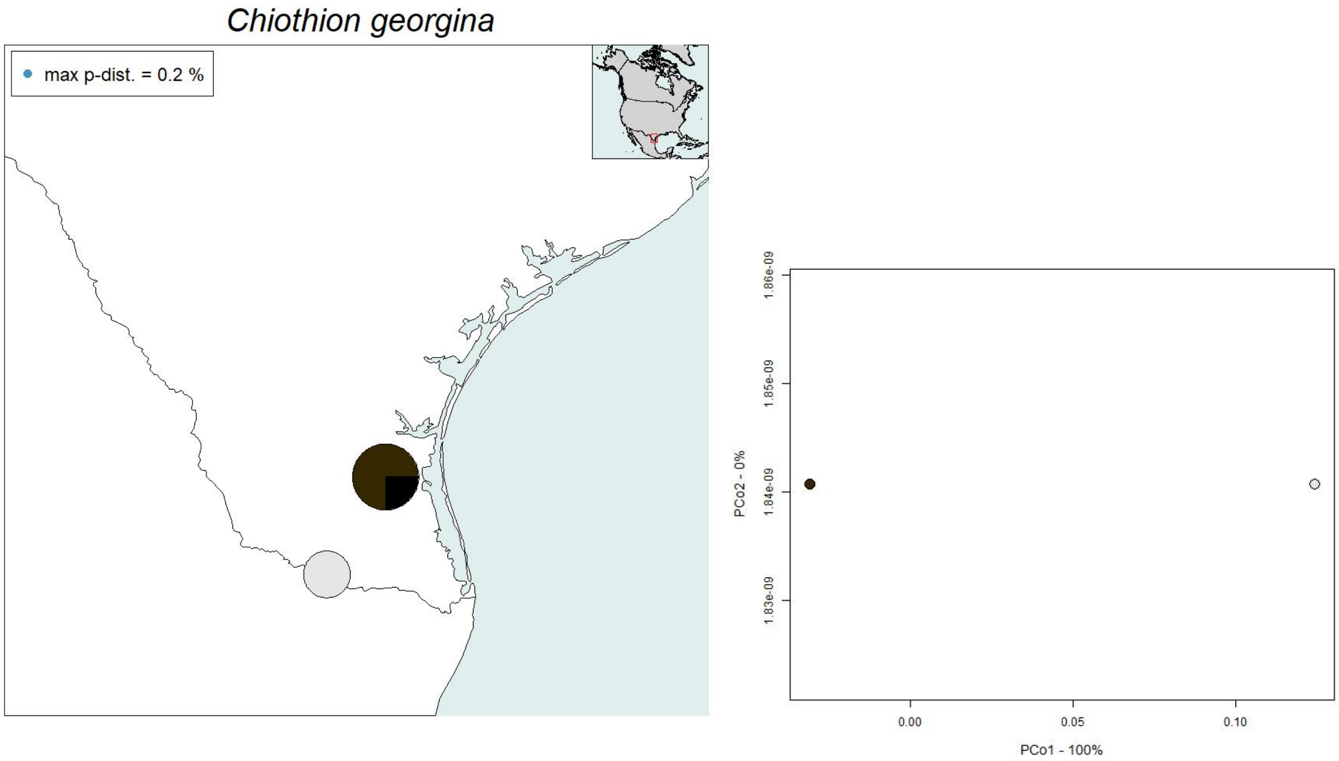

Figure 246 Map of *Chiothion georgina* showing the localities of the sequenced specimens (left). Nearby localities are grouped in pies. Colours match the bidimensional colour space of the PCoA projection (right) of max p-dists among sequences (dots). Sequences= 5; Hap obs.= 2; Hap asympt.= NA; Hap % obs.= NA%; GST= NaN; DST= NaN; HD= NA; ND= NA; max p-dist= 0.2%.

Haplotype network analysis and bubble plot of *Chiothion georgina* were not possible. Sequences > 599 bp = 5.

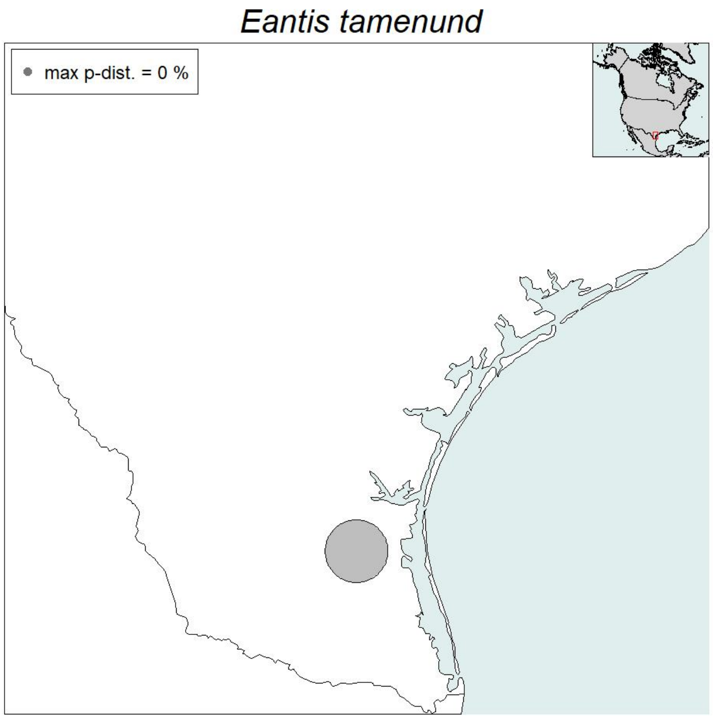

Figure 247: Map of *Eantis tamenund* showing the localities of the sequenced specimens. Nearby localities are grouped in pies. Due to the presence of a single haplotype PCoA projection was not done and a single grey colour was plotted on the map. Sequences= 2; Hap obs.= 1; Hap asympt.= NA; Hap % obs.= NA%; GST= NaN; DST= NaN; HD= NA; ND= NA; max p-dist= 0%.

Haplotype network analysis and bubble plot of *Eantis tamenund* were not possible. Sequences > 599 bp = 2.

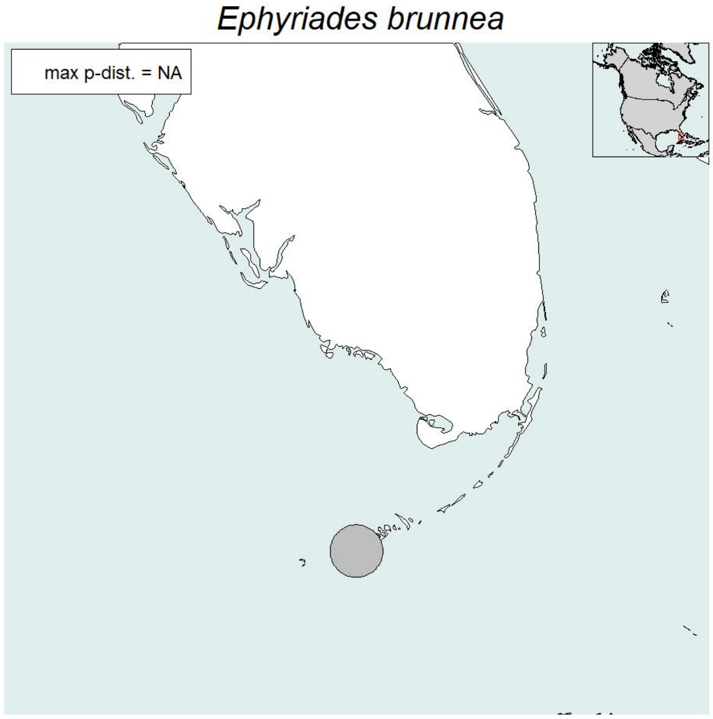

Figure 248: Map of *Ephyriades brunnea* showing the localities of the sequenced specimens. Nearby localities are grouped in pies. Due to the presence of a single haplotype PCoA projection was not done and a single grey colour was plotted on the map. Sequences= 1; Hap obs.= NA; Hap asympt.= NA; Hap % obs.= NA; GST= NaN; DST= NaN; HD= NA; ND= NA; max p-dist= NA.

Haplotype network analysis and bubble plot of *Ephyriades brunnea* were not possible. Sequences > 599 bp = 1.

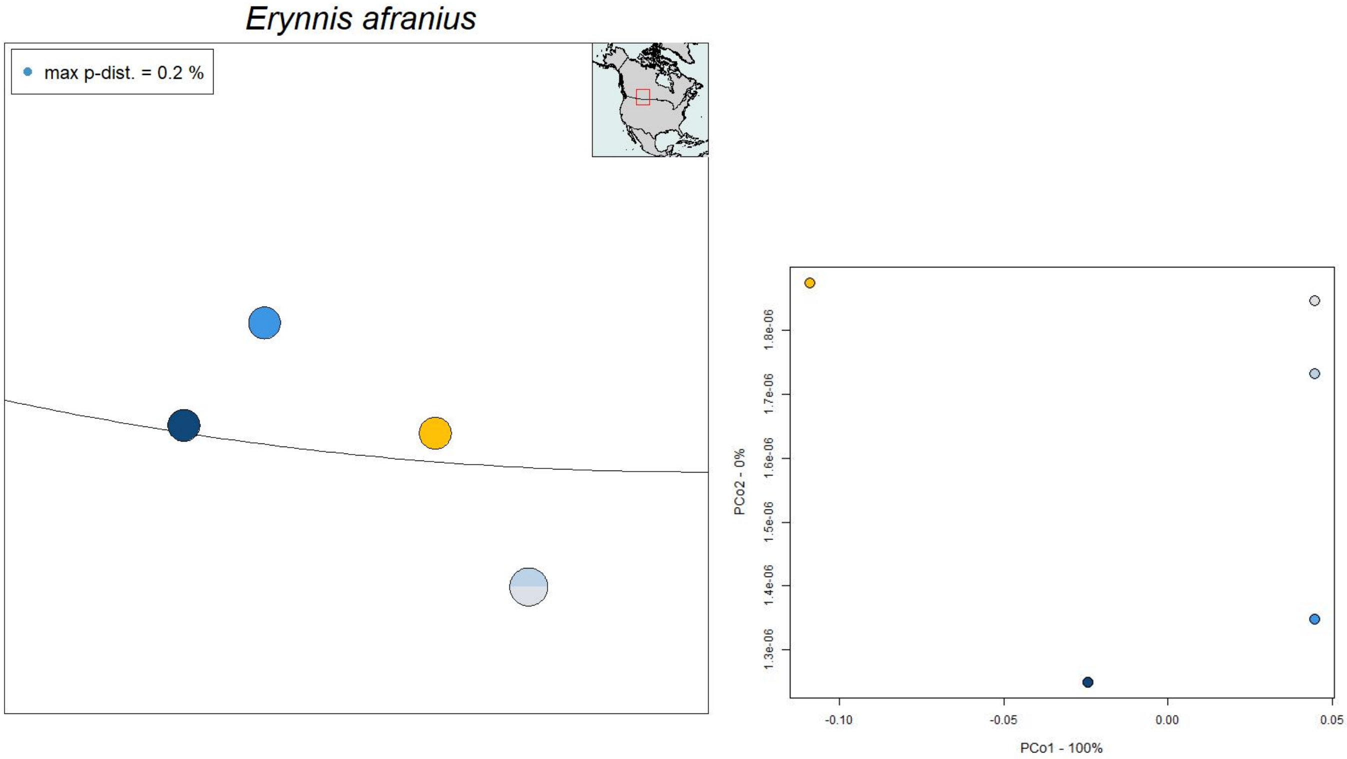

Figure 249 Map of *Erynnis afranius* showing the localities of the sequenced specimens (left). Nearby localities are grouped in pies. Colours match the bidimensional colour space of the PCoA projection (right) of max p-dists among sequences (dots). Sequences= 5; Hap obs.= 3; Hap asympt.= NA; Hap % obs.= NA%; GST= NaN; DST= NaN; HD= NA; ND= NA; max p-dist= 0.2%.

Haplotype network analysis and bubble plot of *Erynnis afranius* were not possible. Sequences > 599 bp = 4.

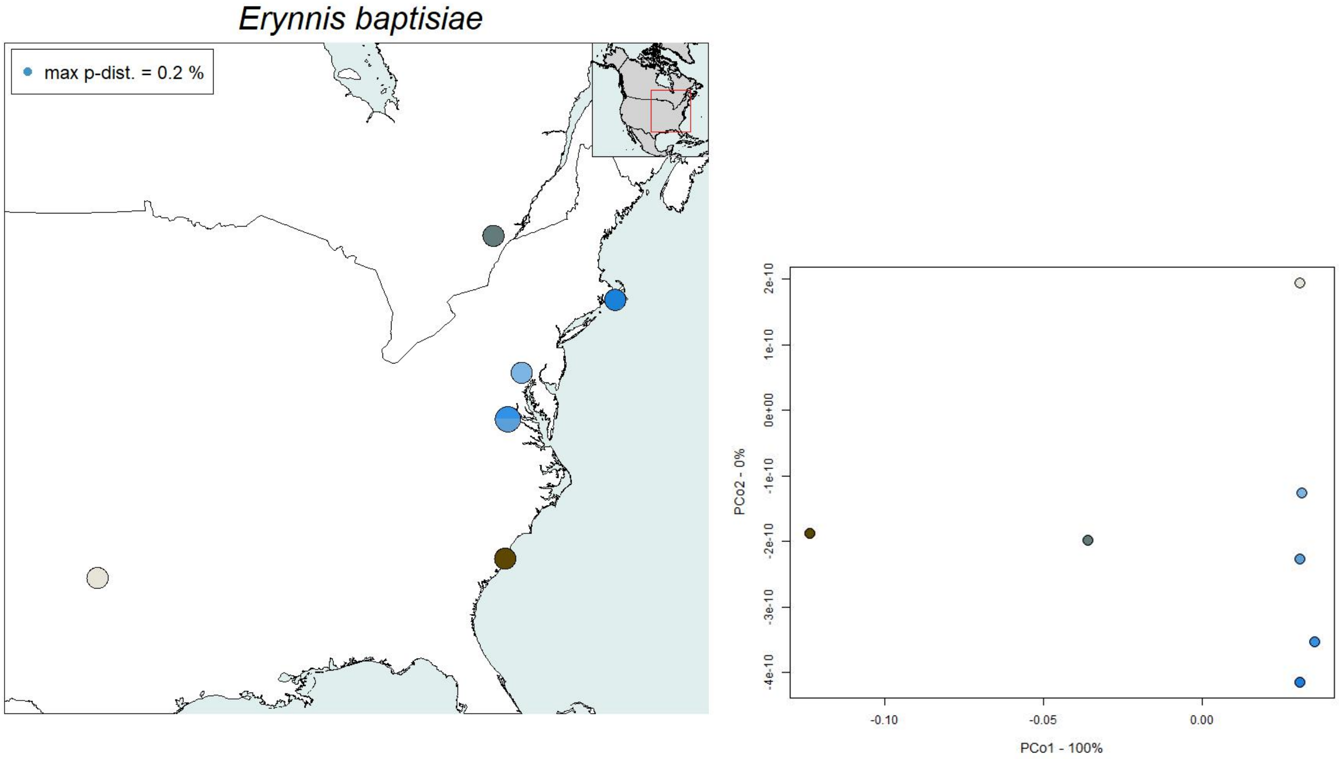

Figure 250 Map of *Erynnis baptisiae* showing the localities of the sequenced specimens (left). Nearby localities are grouped in pies. Colours match the bidimensional colour space of the PCoA projection (right) of max p-dists among sequences (dots). Sequences= 7; Hap obs.= 3; Hap asympt.= NA; Hap % obs.= NA%; GST= NaN; DST= NaN; HD= NA; ND= NA; max p-dist= 0.2%.

Haplotype network analysis and bubble plot of *Erynnis baptisiae* were not possible. Sequences > 599 bp = 7.

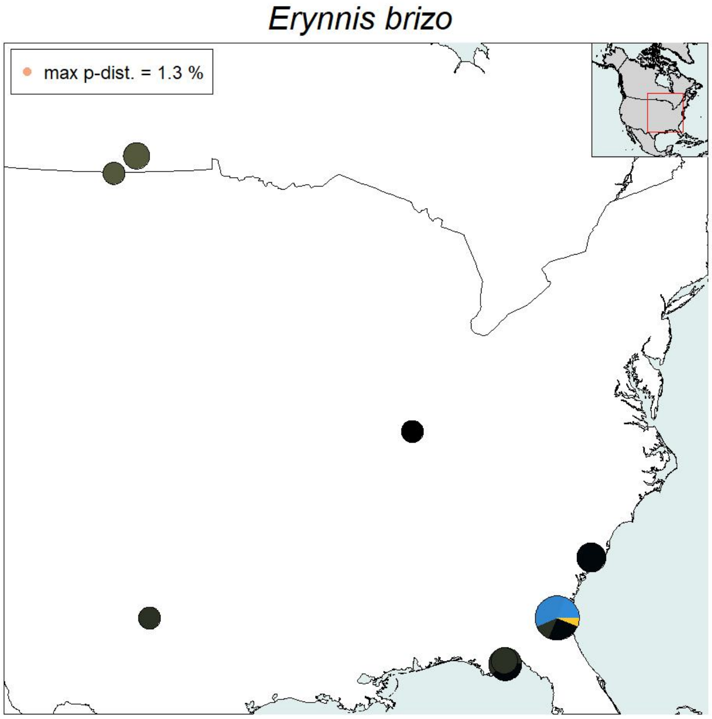

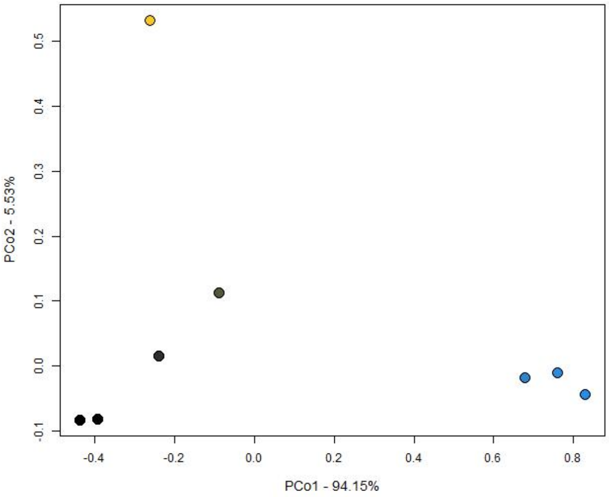

Figure 251 Map of *Erynnis brizo* showing the localities of the sequenced specimens (left). Nearby localities are grouped in pies. Colours match the bidimensional colour space of the PCoA projection (right) of max p-dists among sequences (dots). Sequences= 31; Hap obs.= 6; Hap asympt.= 7; Hap % obs.= 86.1%; GST= 0.154; DST= 0.0008; HD= 0.776; ND= 0.0051; max p-dist= 1.3%.

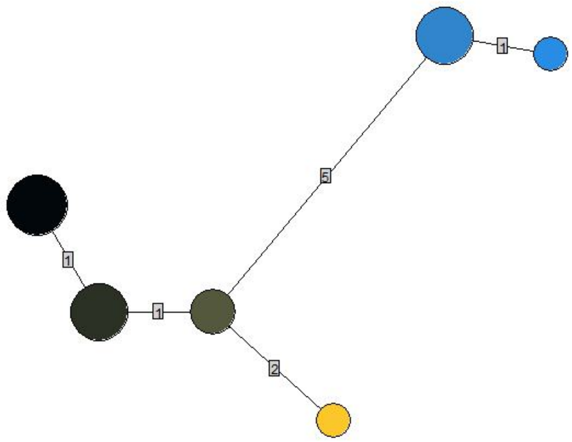

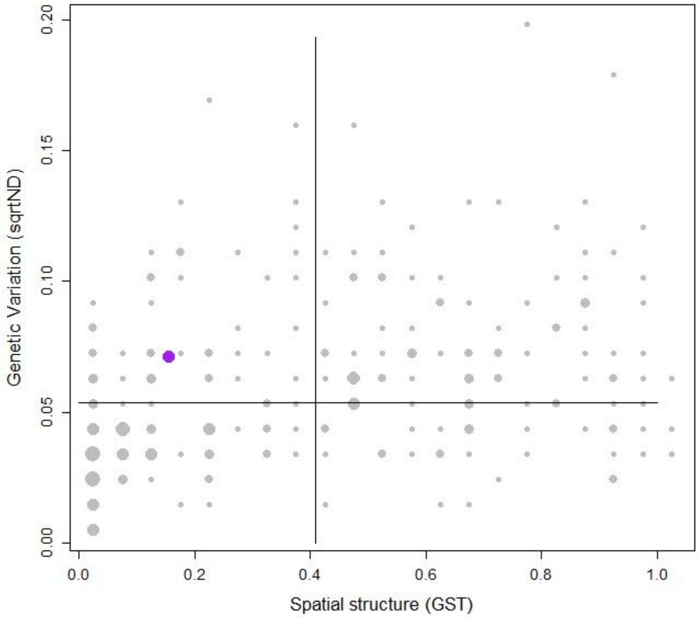

Figure 252: Haplotype network (left) of *Erynnis brizo* sequences > 599 bp with colours matching the PCoA colour space (above). The bubble plot for mt-DNA polymorphism (square root transformed nucleotide diversity) and spatial structure (GST) among all species in the atlas and values for *Erynnis brizo* (purple dot). The horizontal and vertical lines represent median values of nucleotide diversity and GST, respectively. Sequences > 599 bp= 31.

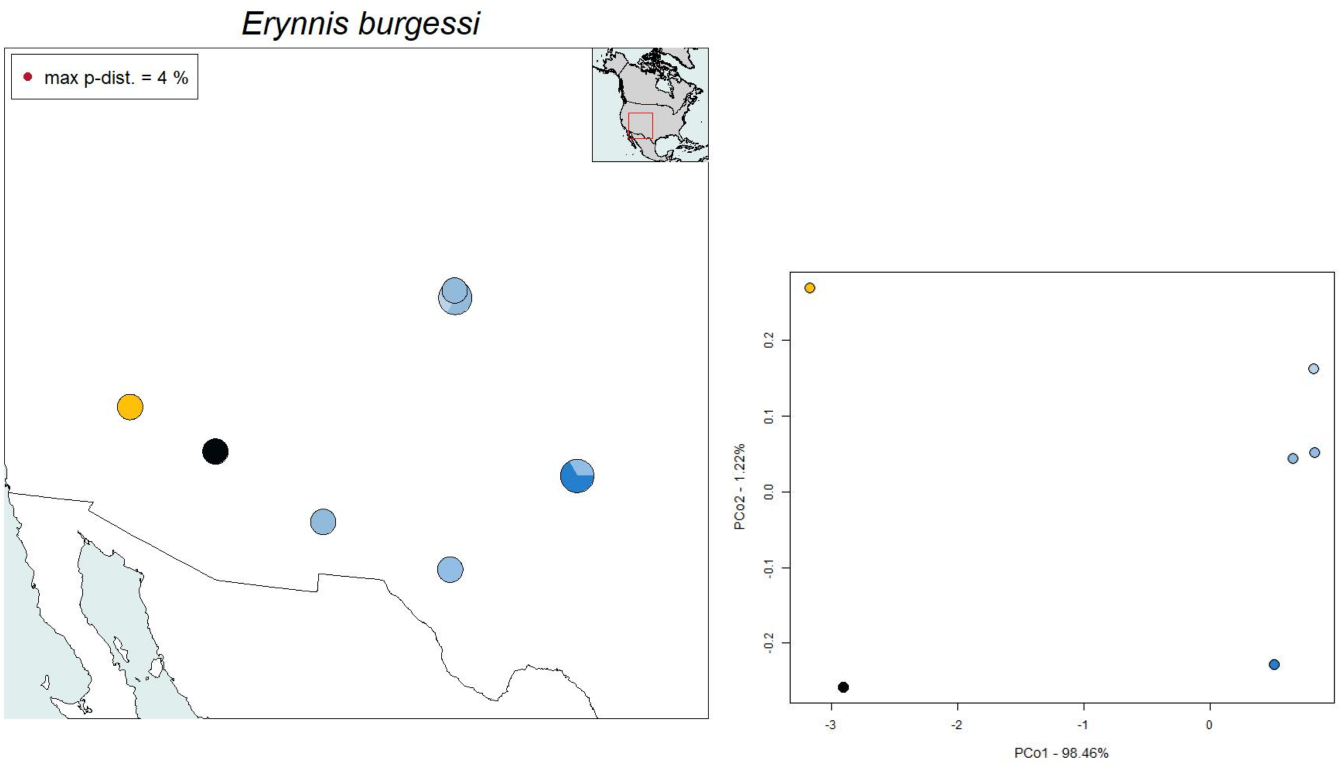

Figure 253 Map of *Erynnis burgessi* showing the localities of the sequenced specimens (left). Nearby localities are grouped in pies. Colours match the bidimensional colour space of the PCoA projection (right) of max p-dists among sequences (dots). Sequences= 11; Hap obs.= 5; Hap asympt.= 5.2; Hap % obs.= 97%; GST= NaN; DST= NaN; HD= 0.836; ND= 0.013; max p-dist= 4%.

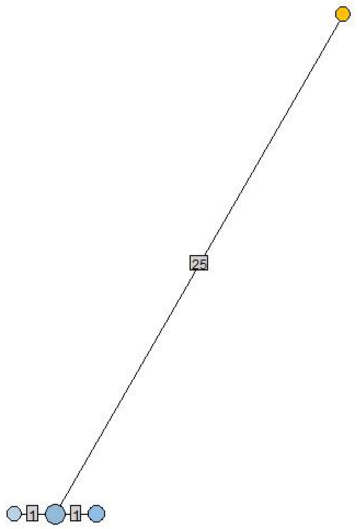

Figure 254: Haplotype network of *Erynnis burgessi*. Sequences > 599 bp= 8.

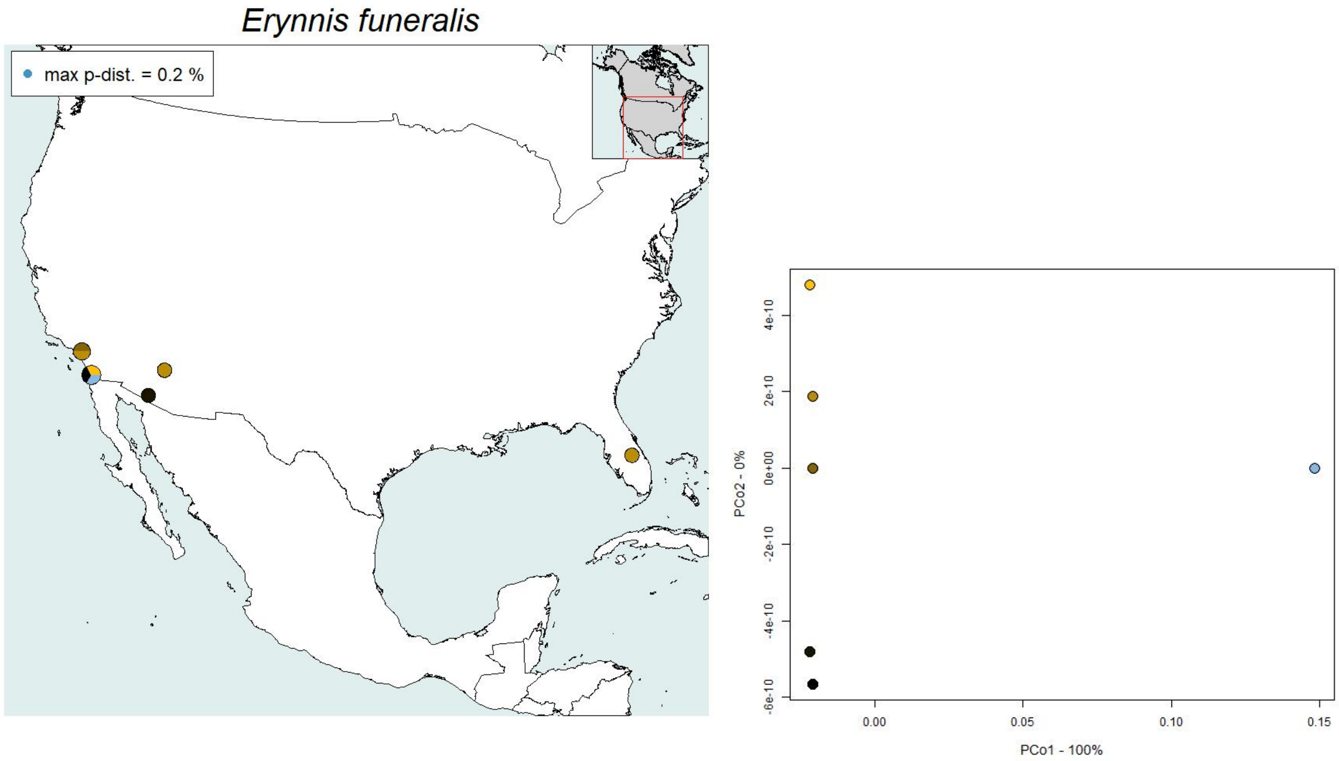

Figure 255 Map of *Erynnis funeralis* showing the localities of the sequenced specimens (left). Nearby localities are grouped in pies. Colours match the bidimensional colour space of the PCoA projection (right) of max p-dists among sequences (dots). Sequences= 8; Hap obs.= 2; Hap asympt.= NA; Hap % obs.= NA%; GST= NaN; DST= NaN; HD= NA; ND= NA; max p-dist= 0.2%.

Haplotype network analysis and bubble plot of *Erynnis funeralis* were not possible. Sequences > 599 bp = 5.

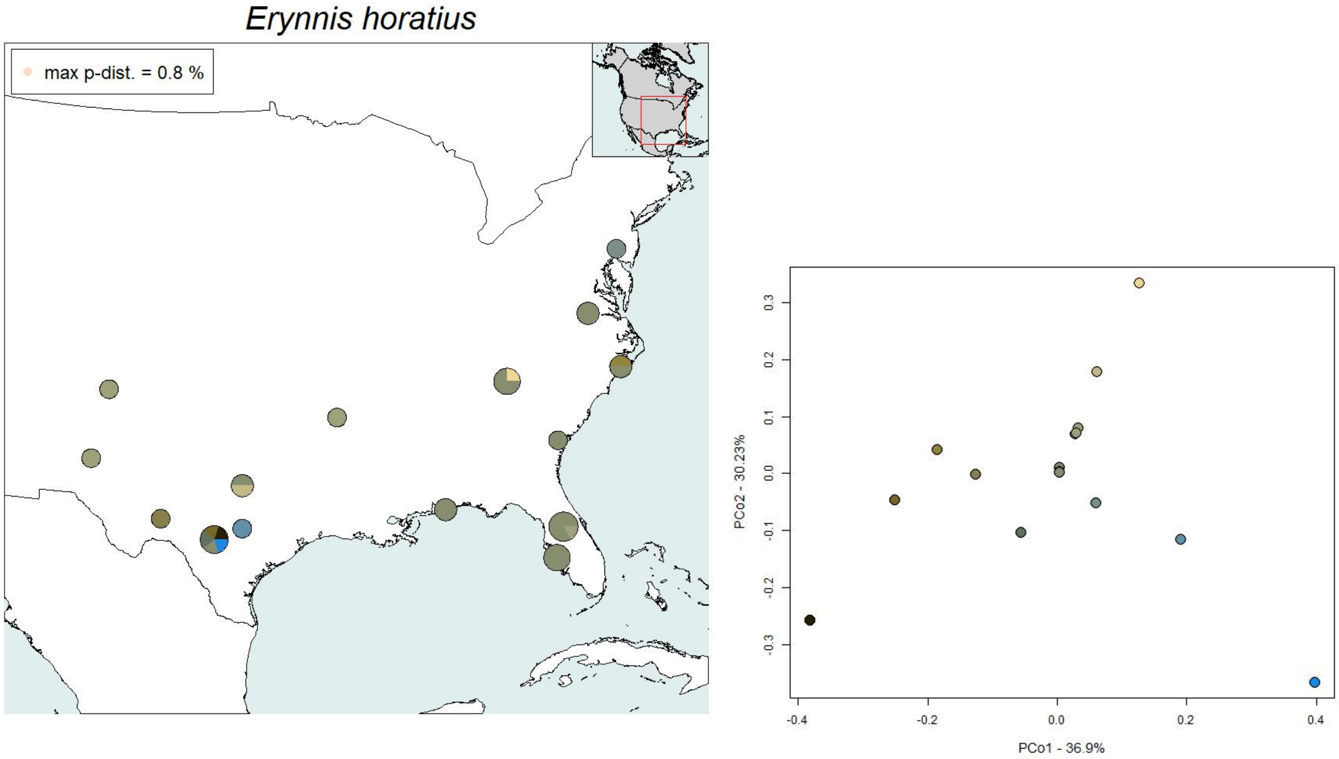

Figure 256 Map of *Erynnis horatius* showing the localities of the sequenced specimens (left). Nearby localities are grouped in pies. Colours match the bidimensional colour space of the PCoA projection (right) of max p-dists among sequences (dots). Sequences= 34; Hap obs.= 12; Hap asympt.= 60.5; Hap % obs.= 19.8%; GST= 0.217; DST= 0.0003; HD= 0.586; ND= 0.0016; max p-dist= 0.8%.

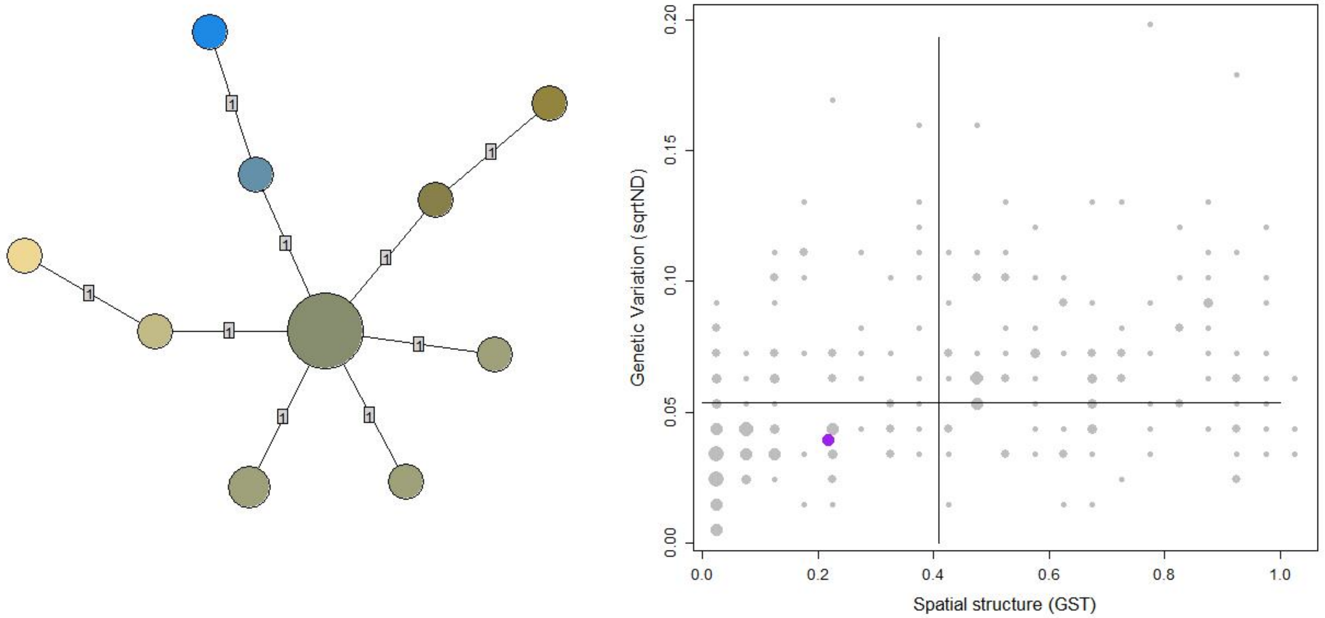

Figure 257: Haplotype network (left) of *Erynnis horatius* sequences > 599 bp with colours matching the PCoA colour space (above). The bubble plot for mt-DNA polymorphism (square root transformed nucleotide diversity) and spatial structure (GST) among all species in the atlas and values for *Erynnis horatius* (purple dot). The horizontal and vertical lines represent median values of nucleotide diversity and GST, respectively. Sequences > 599 bp= 31.

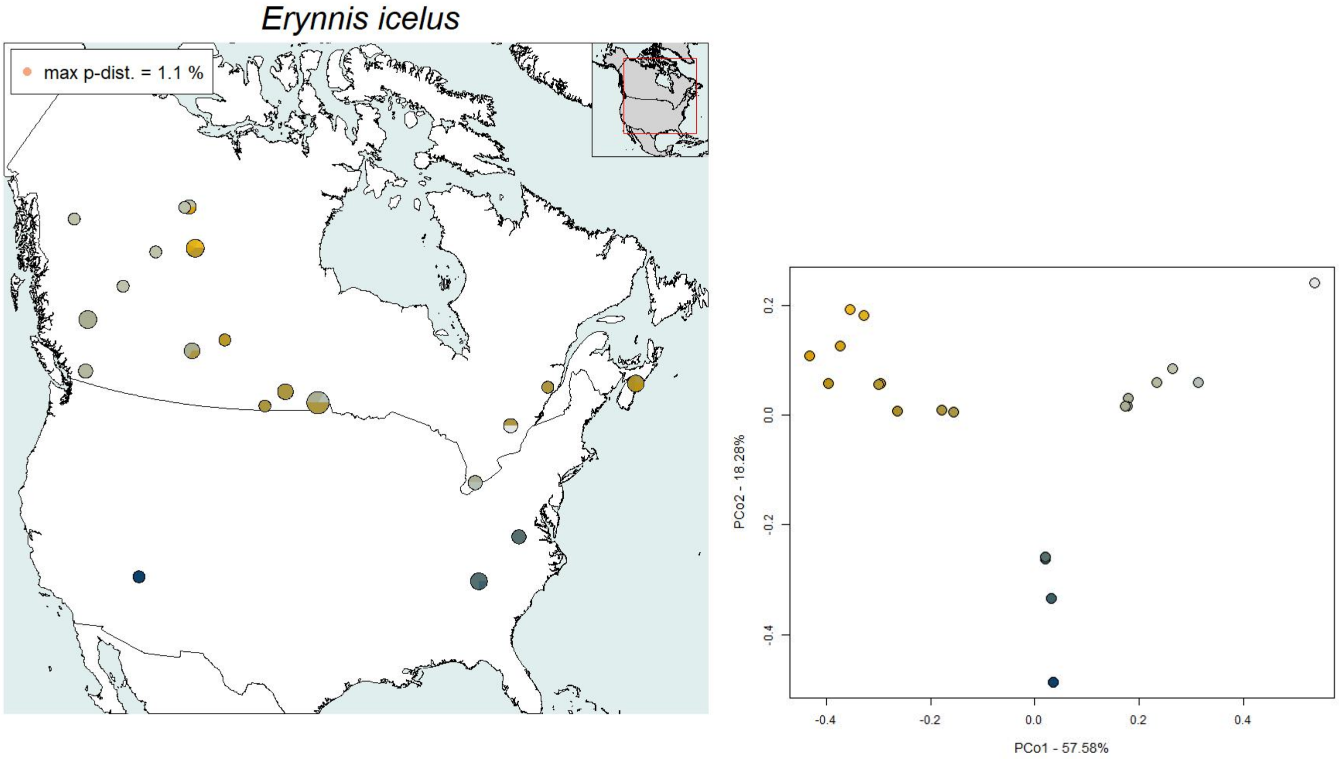

Figure 258 Map of *Erynnis icelus* showing the localities of the sequenced specimens (left). Nearby localities are grouped in pies. Colours match the bidimensional colour space of the PCoA projection (right) of max p-dists among sequences (dots). Sequences= 54; Hap obs.= 13; Hap asympt.= 19.1; Hap % obs.= 67.9%; GST= 0.472; DST= 0.0014; HD= 0.867; ND= 0.0034; max p-dist= 1.1%.

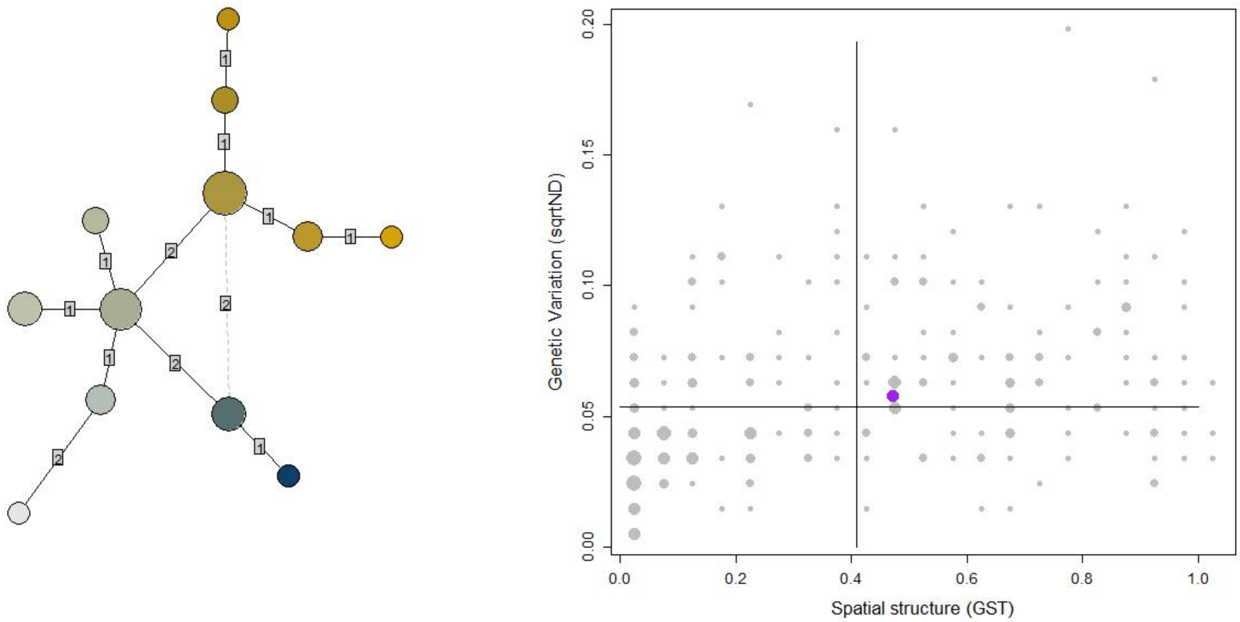

Figure 259: Haplotype network (left) of *Erynnis icelus* sequences > 599 bp with colours matching the PCoA colour space (above). The bubble plot for mt-DNA polymorphism (square root transformed nucleotide diversity) and spatial structure (GST) among all species in the atlas and values for *Erynnis icelus* (purple dot). The horizontal and vertical lines represent median values of nucleotide diversity and GST, respectively. Sequences > 599 bp= 50.

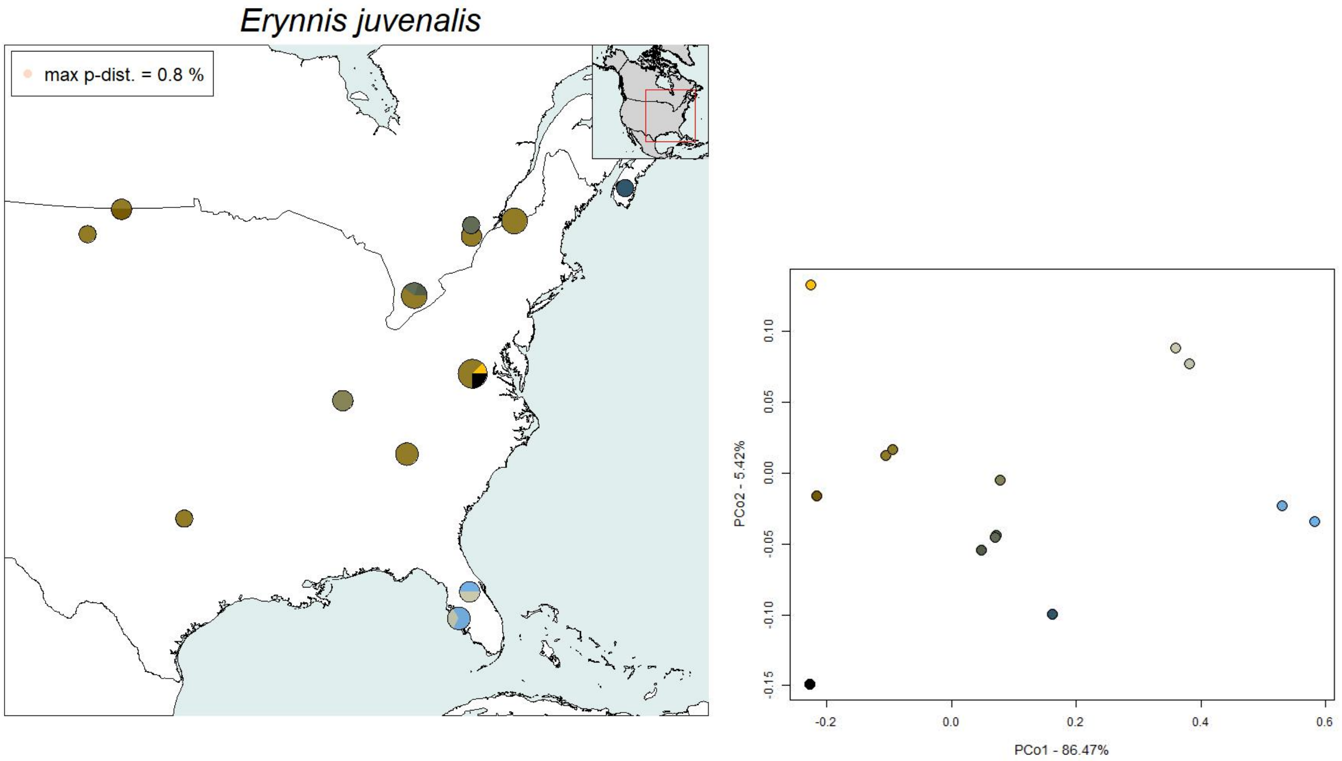

Figure 260 Map of *Erynnis juvenalis* showing the localities of the sequenced specimens (left). Nearby localities are grouped in pies. Colours match the bidimensional colour space of the PCoA projection (right) of max p-dists among sequences (dots). Sequences= 36; Hap obs.= 10; Hap asympt.= 16.1; Hap % obs.= 62.2%; GST= 0.773; DST= 0.0014; HD= 0.654; ND= 0.0017; max p-dist= 0.8%.

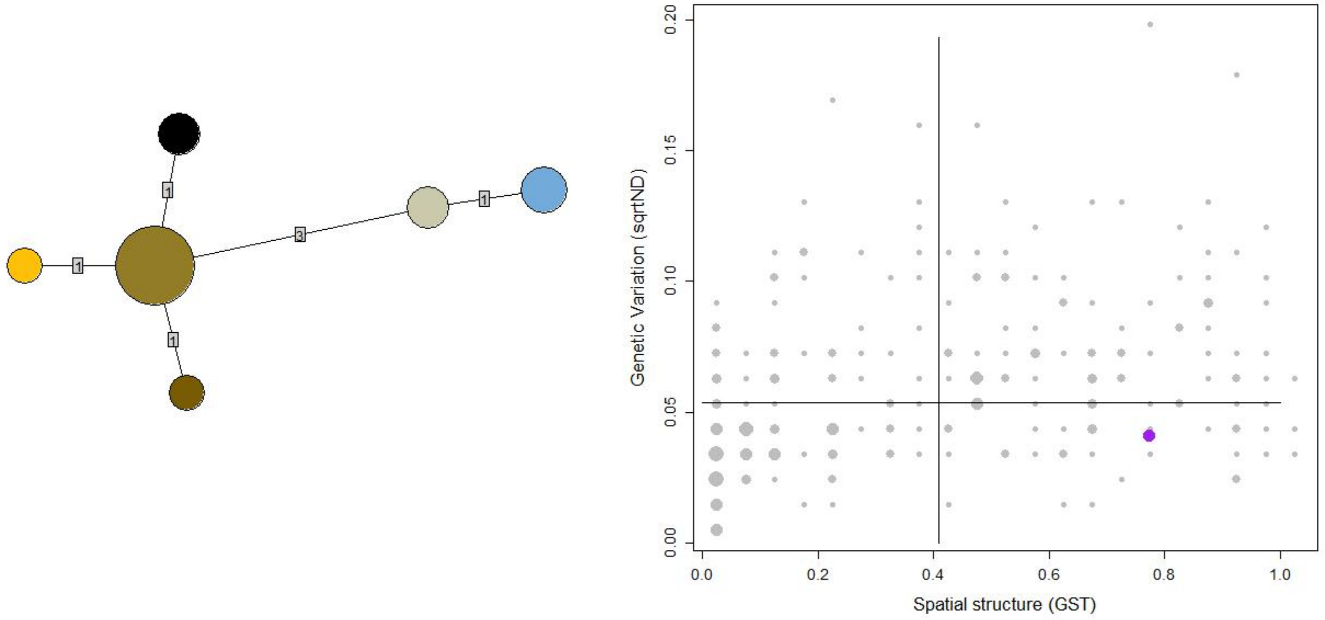

Figure 261: Haplotype network (left) of *Erynnis juvenalis* sequences > 599 bp with colours matching the PCoA colour space (above). The bubble plot for mt-DNA polymorphism (square root transformed nucleotide diversity) and spatial structure (GST) among all species in the atlas and values for *Erynnis juvenalis* (purple dot). The horizontal and vertical lines represent median values of nucleotide diversity and GST, respectively. Sequences > 599 bp= 34.

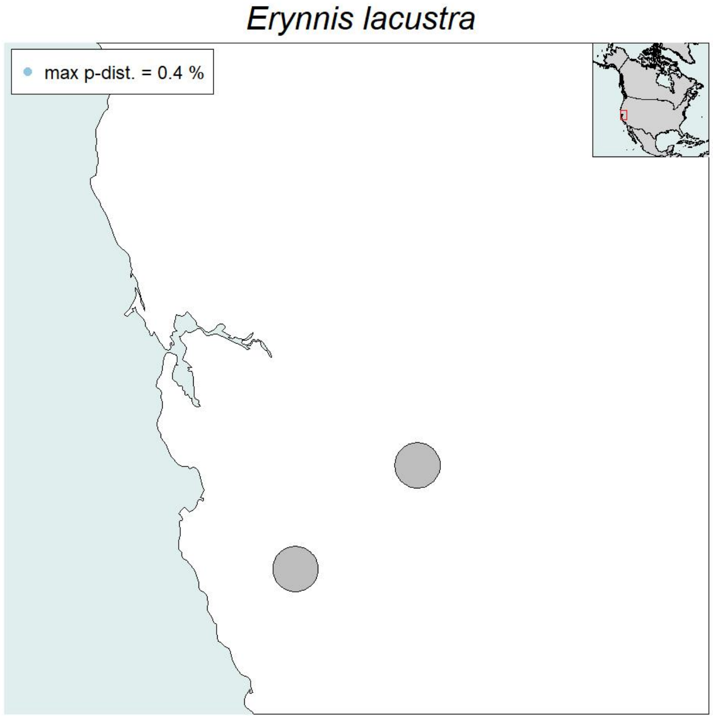

Figure 262: Map of *Erynnis lacustra* showing the localities of the sequenced specimens. Nearby localities are grouped in pies. Due to the presence of a single haplotype PCoA projection was not done and a single grey colour was plotted on the map. Sequences= 2; Hap obs.= 2; Hap asympt.= NA; Hap % obs.= NA%; GST= NaN; DST= NaN; HD= NA; ND= NA; max p-dist= 0.4%.

Haplotype network analysis and bubble plot of *Erynnis lacustra* were not possible. Sequences > 599 bp = 1.

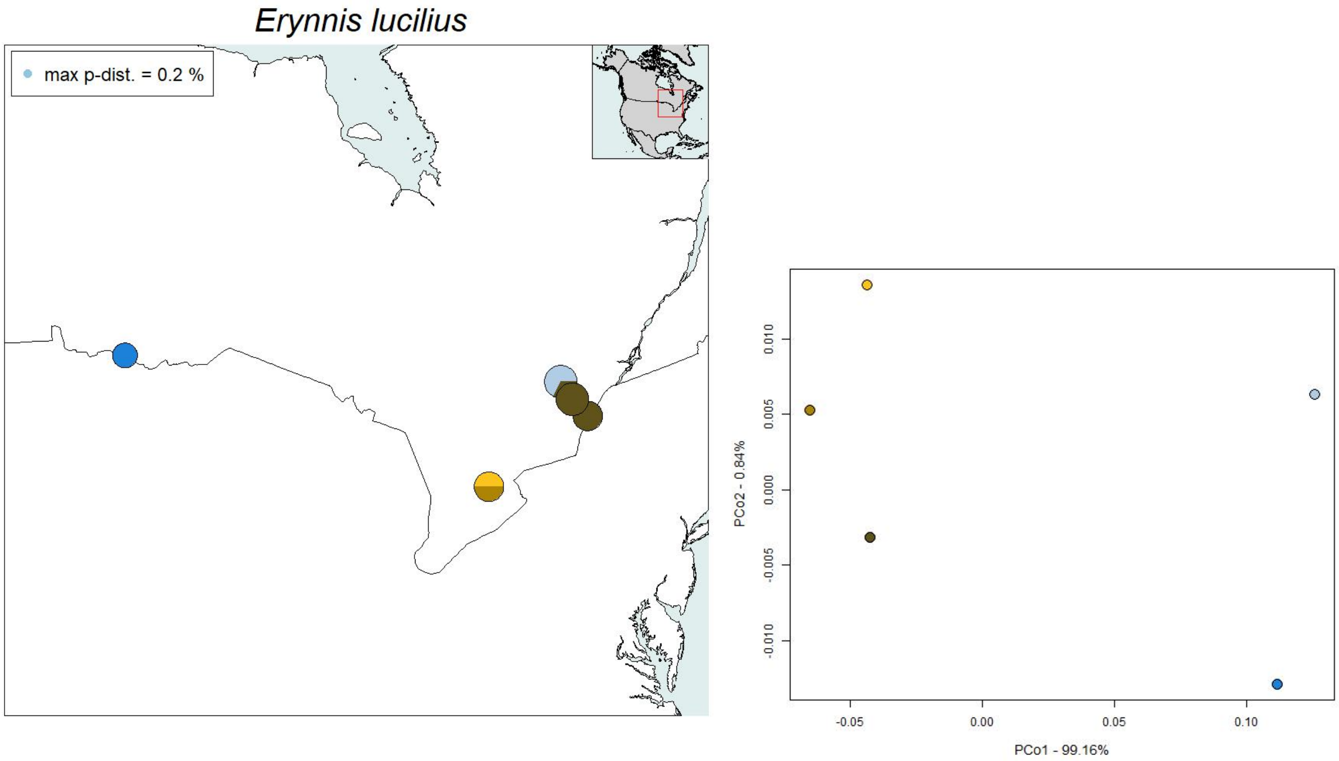

Figure 263 Map of *Erynnis lucilius* showing the localities of the sequenced specimens (left). Nearby localities are grouped in pies. Colours match the bidimensional colour space of the PCoA projection (right) of max p-dists among sequences (dots). Sequences= 11; Hap obs.= 2; Hap asympt.= 2; Hap % obs.= 100%; GST= NaN; DST= NaN; HD= 0.436; ND= 0.0007; max p-dist= 0.2%.

Haplotype network analysis and bubble plot of *Erynnis lucilius* were not possible. Sequences > 599 bp = 8.

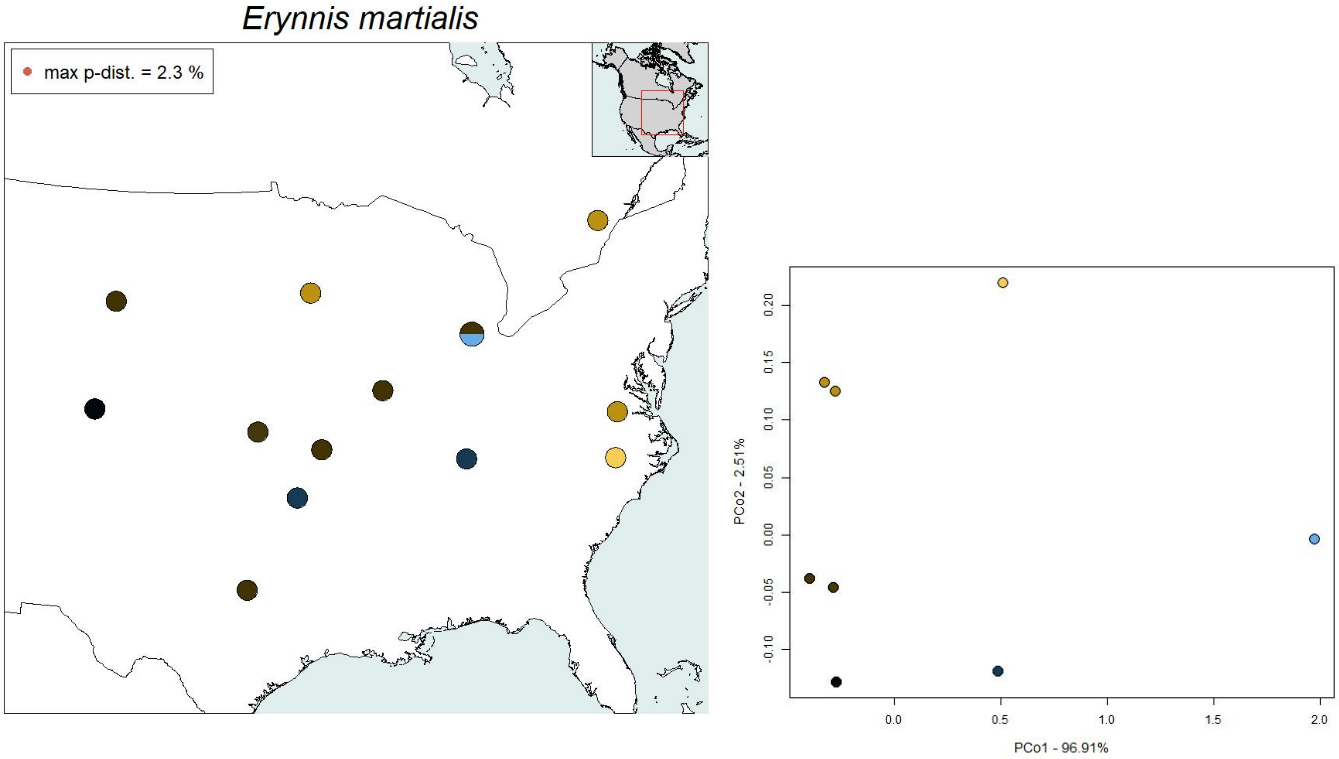

Figure 264 Map of *Erynnis martialis* showing the localities of the sequenced specimens (left). Nearby localities are grouped in pies. Colours match the bidimensional colour space of the PCoA projection (right) of max p-dists among sequences (dots). Sequences= 14; Hap obs.= 6; Hap asympt.= 10.2; Hap % obs.= 58.9%; GST= NaN; DST= NaN; HD= 0.791; ND= 0.0035; max p-dist= 2.3%.

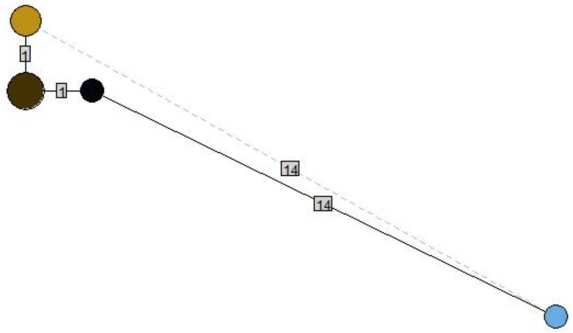

Figure 265: Haplotype network of *Erynnis martialis*. Sequences > 599 bp= 11.

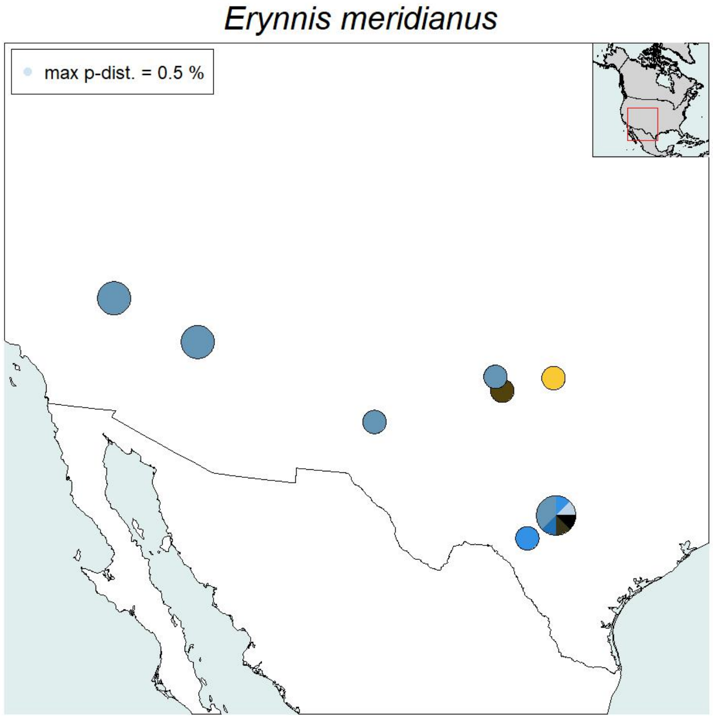

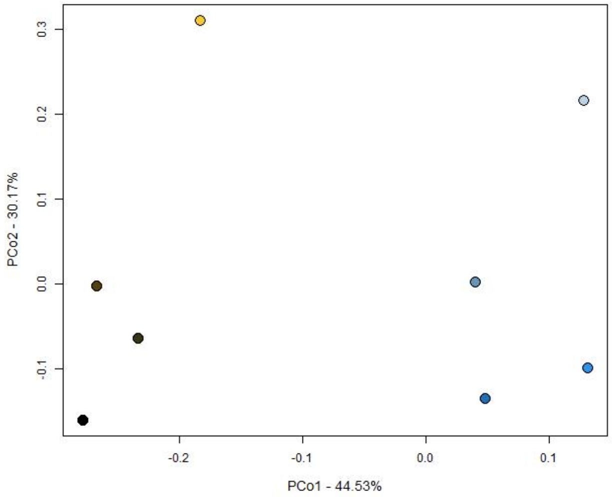

Figure 266 Map of *Erynnis meridianus* showing the localities of the sequenced specimens (left). Nearby localities are grouped in pies. Colours match the bidimensional colour space of the PCoA projection (right) of max p-dists among sequences (dots). Sequences= 21; Hap obs.= 9; Hap asympt.= 35.7; Hap % obs.= 25.2%; GST= 0.08; DST= 0.0001; HD= 0.629; ND= 0.0017; max p-dist= 0.5%.

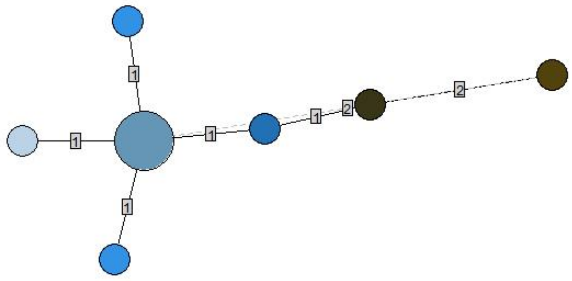

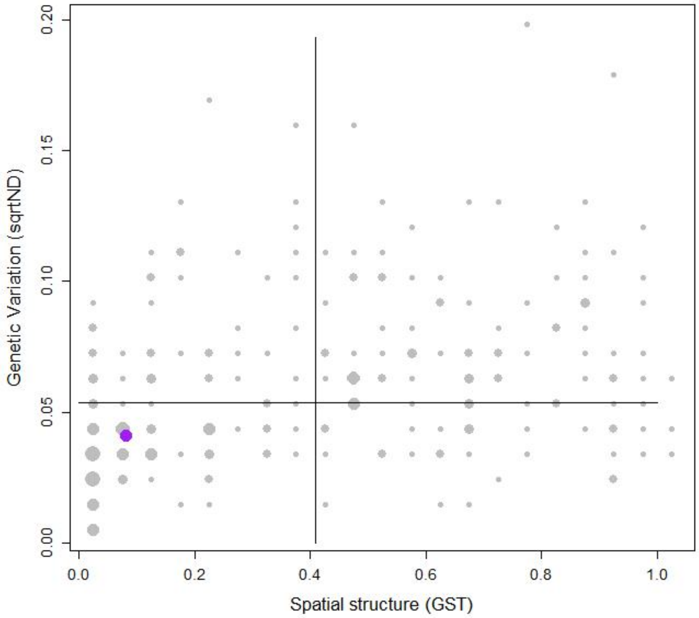

Figure 267: Haplotype network (left) of *Erynnis meridianus* sequences > 599 bp with colours matching the PCoA colour space (above). The bubble plot for mt-DNA polymorphism (square root transformed nucleotide diversity) and spatial structure (GST) among all species in the atlas and values for *Erynnis meridianus* (purple dot). The horizontal and vertical lines represent median values of nucleotide diversity and GST, respectively. Sequences > 599 bp= 19.

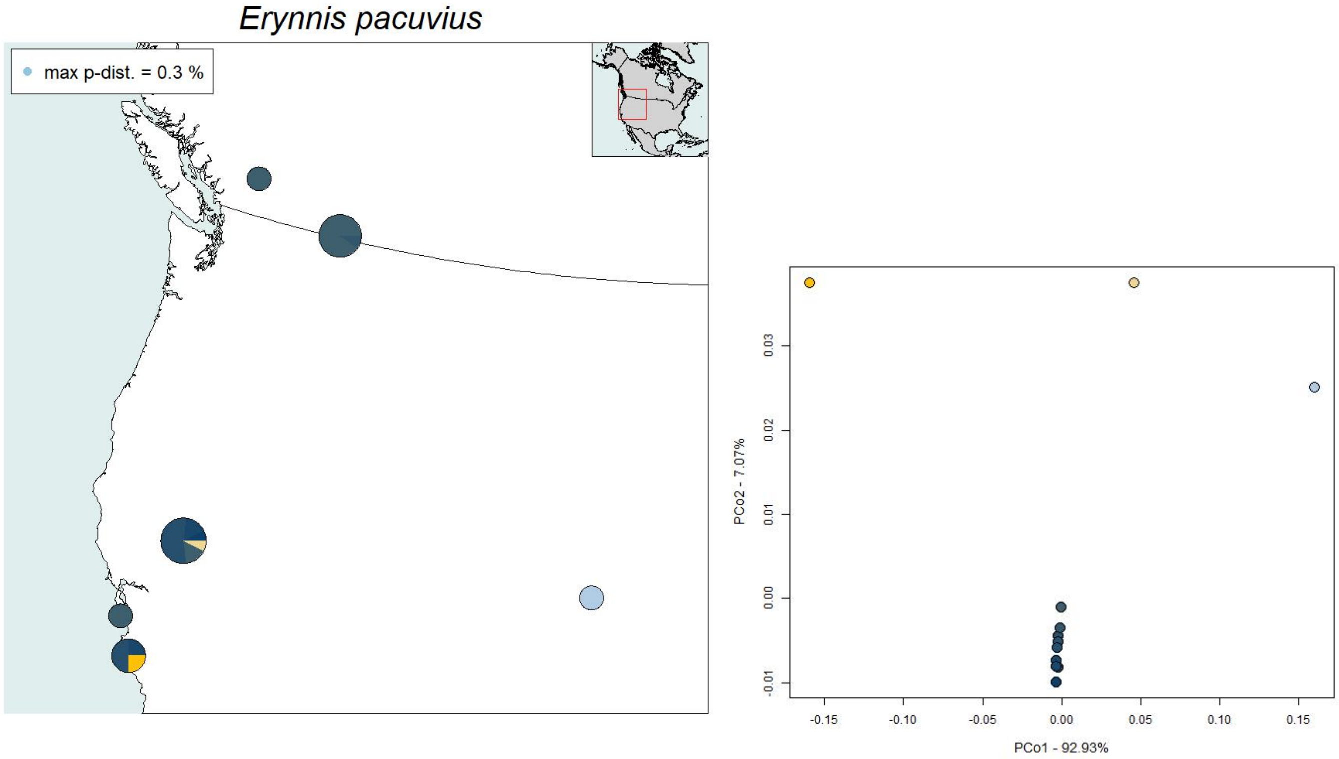

Figure 268 Map of *Erynnis pacuvius* showing the localities of the sequenced specimens (left). Nearby localities are grouped in pies. Colours match the bidimensional colour space of the PCoA projection (right) of max p-dists among sequences (dots). Sequences= 30; Hap obs.= 4; Hap asympt.= 6.9; Hap % obs.= 58%; GST= 0.695; DST= 0.0001; HD= 0.193; ND= 0.0002; max p-dist= 0.3%.

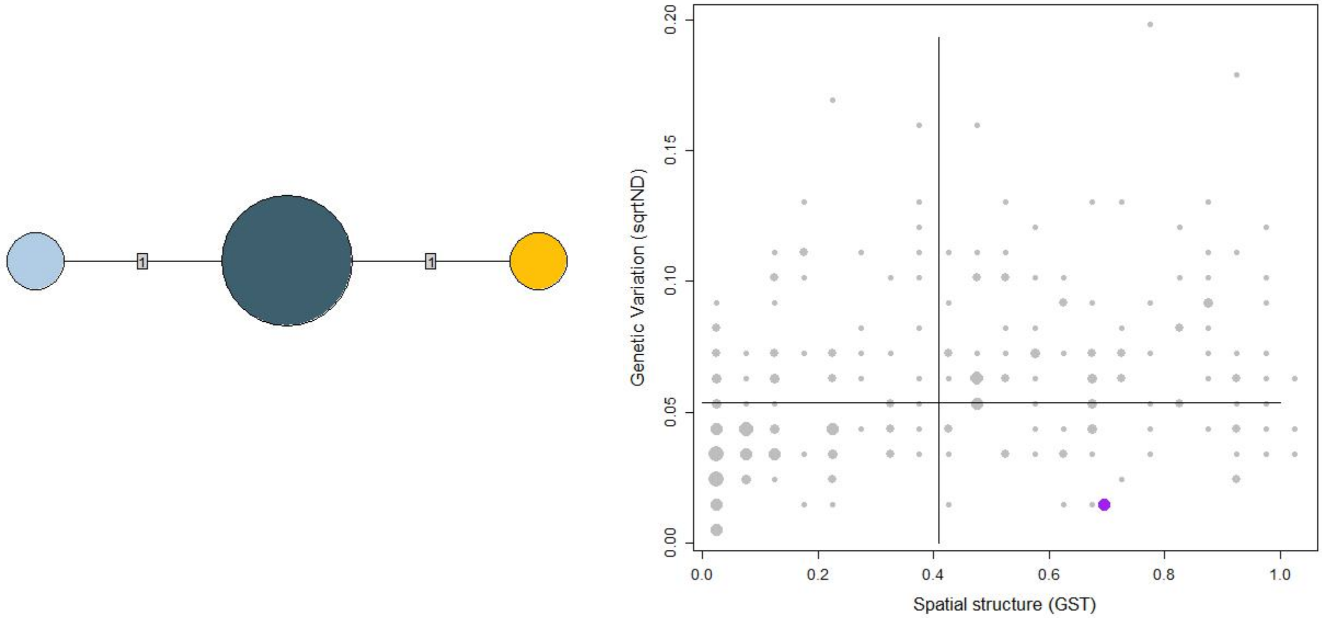

Figure 269: Haplotype network (left) of *Erynnis pacuvius* sequences > 599 bp with colours matching the PCoA colour space (above). The bubble plot for mt-DNA polymorphism (square root transformed nucleotide diversity) and spatial structure (GST) among all species in the atlas and values for *Erynnis pacuvius* (purple dot). The horizontal and vertical lines represent median values of nucleotide diversity and GST, respectively. Sequences > 599 bp= 29.

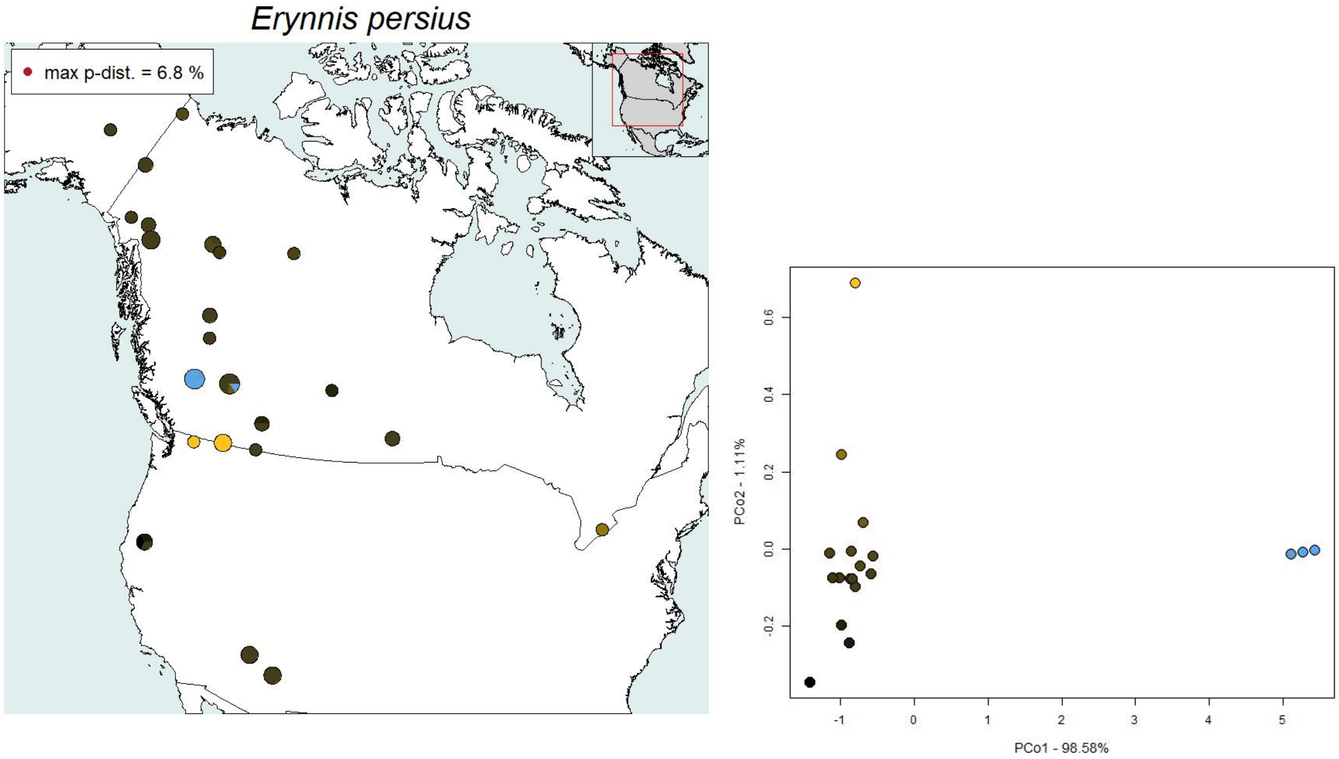

Figure 270 Map of *Erynnis persius* showing the localities of the sequenced specimens (left). Nearby localities are grouped in pies. Colours match the bidimensional colour space of the PCoA projection (right) of max p-dists among sequences (dots). Sequences= 57; Hap obs.= 14; Hap asympt.= 33.9; Hap % obs.= 41.3%; GST= 0.748; DST= 0.0142; HD= 0.652; ND= 0.0165; max p-dist= 6.8%.

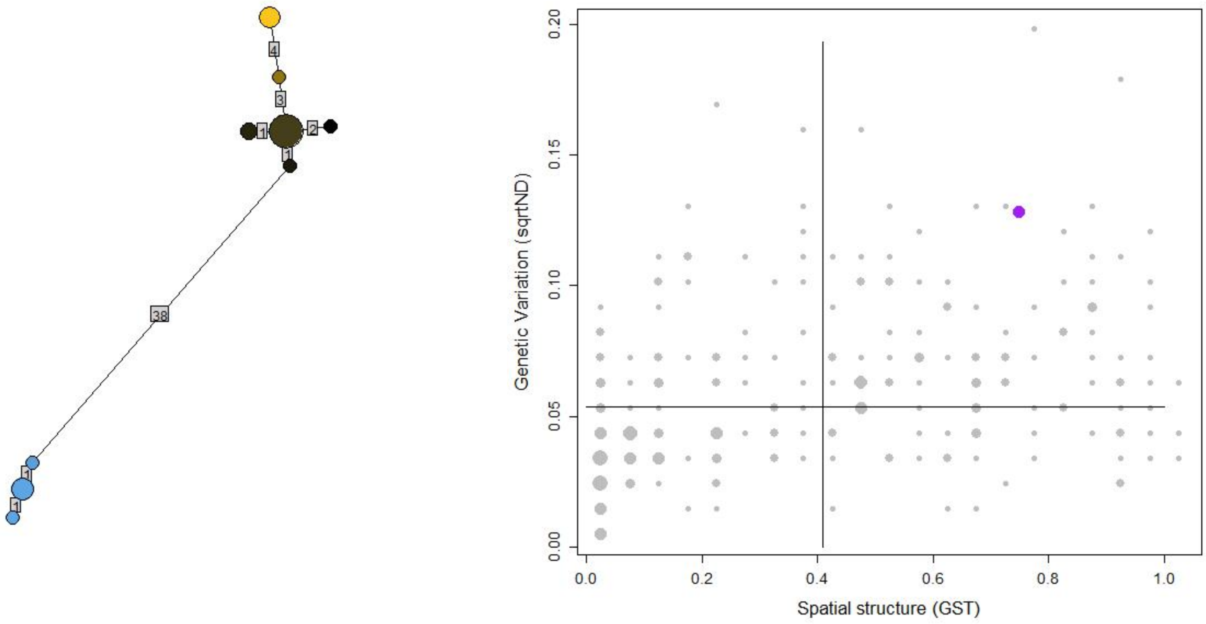

Figure 271: Haplotype network (left) of *Erynnis persius* sequences > 599 bp with colours matching the PCoA colour space (above). The bubble plot for mt-DNA polymorphism (square root transformed nucleotide diversity) and spatial structure (GST) among all species in the atlas and values for *Erynnis persius* (purple dot). The horizontal and vertical lines represent median values of nucleotide diversity and GST, respectively. Sequences > 599 bp= 52.

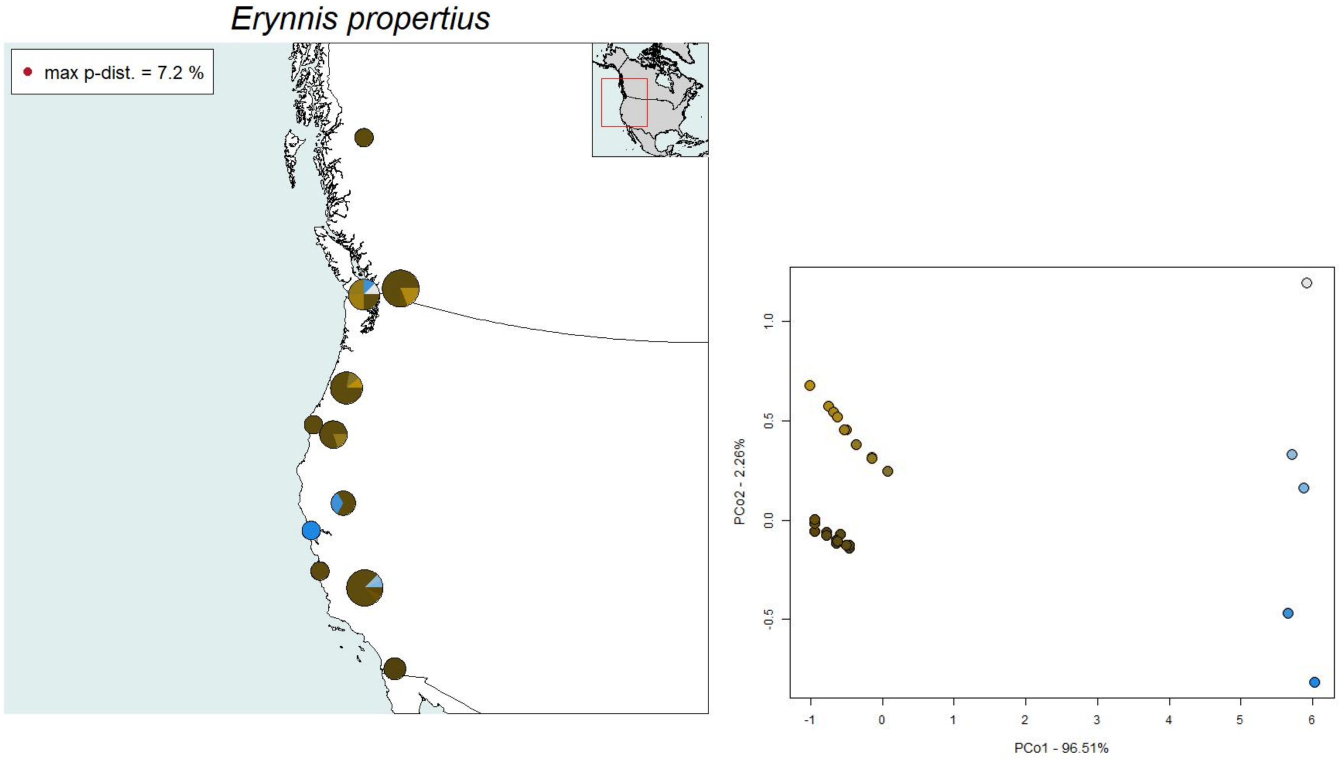

Figure 272 Map of *Erynnis propertius* showing the localities of the sequenced specimens (left). Nearby localities are grouped in pies. Colours match the bidimensional colour space of the PCoA projection (right) of max p-dists among sequences (dots). Sequences= 62; Hap obs.= 20; Hap asympt.= 40.8; Hap % obs.= 49%; GST= 0.35; DST= 0.0038; HD= 0.696; ND= 0.0121; max p-dist= 7.2%.

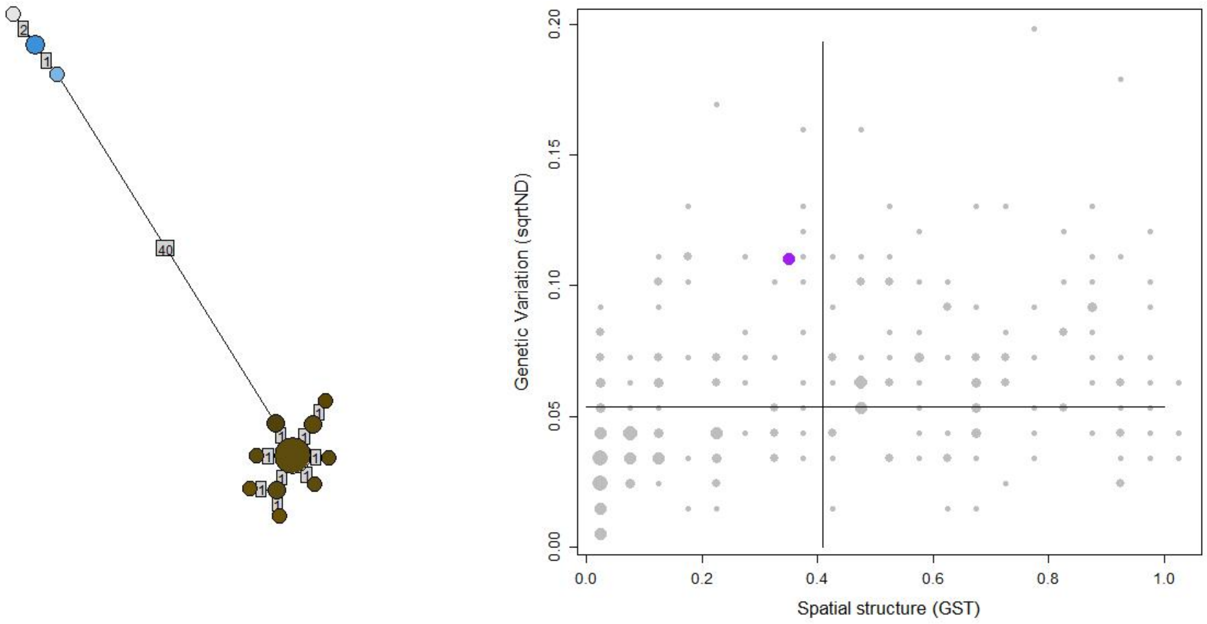

Figure 273: Haplotype network (left) of *Erynnis propertius* sequences > 599 bp with colours matching the PCoA colour space (above). The bubble plot for mt-DNA polymorphism (square root transformed nucleotide diversity) and spatial structure (GST) among all species in the atlas and values for *Erynnis propertius* (purple dot). The horizontal and vertical lines represent median values of nucleotide diversity and GST, respectively. Sequences > 599 bp= 54.

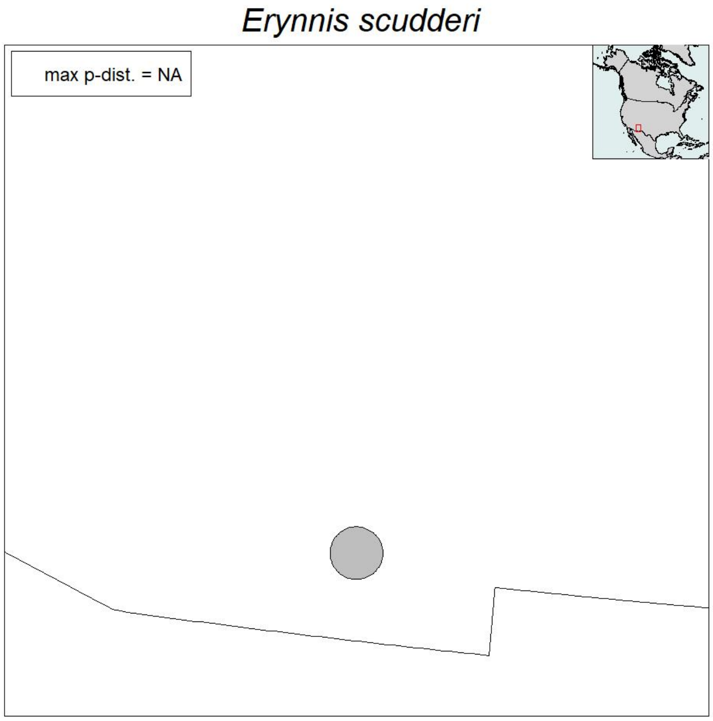

Figure 274: Map of *Erynnis scudderi* showing the localities of the sequenced specimens. Nearby localities are grouped in pies. Due to the presence of a single haplotype PCoA projection was not done and a single grey colour was plotted on the map. Sequences= 1; Hap obs.= NA; Hap asympt.= NA; Hap % obs.= NA; GST= NaN; DST= NaN; HD= NA; ND= NA; max p-dist= NA.

Haplotype network analysis and bubble plot of *Erynnis scudderi* were not possible. Sequences > 599 bp = 1.

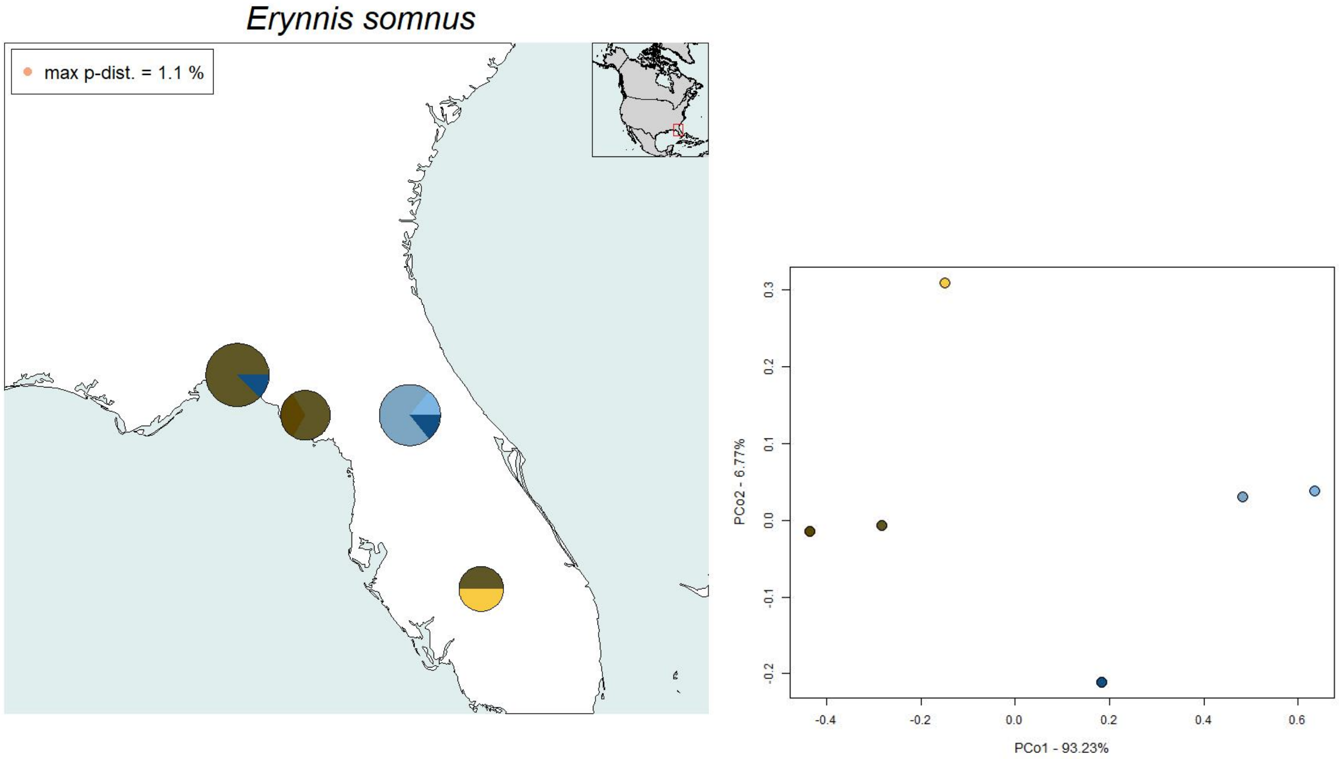

Figure 275 Map of *Erynnis somnus* showing the localities of the sequenced specimens (left). Nearby localities are grouped in pies. Colours match the bidimensional colour space of the PCoA projection (right) of max p-dists among sequences (dots). Sequences= 20; Hap obs.= 6; Hap asympt.= 10.3; Hap % obs.= 58.4%; GST= 0.586; DST= 0.0024; HD= 0.705; ND= 0.004; max p-dist= 1.1%.

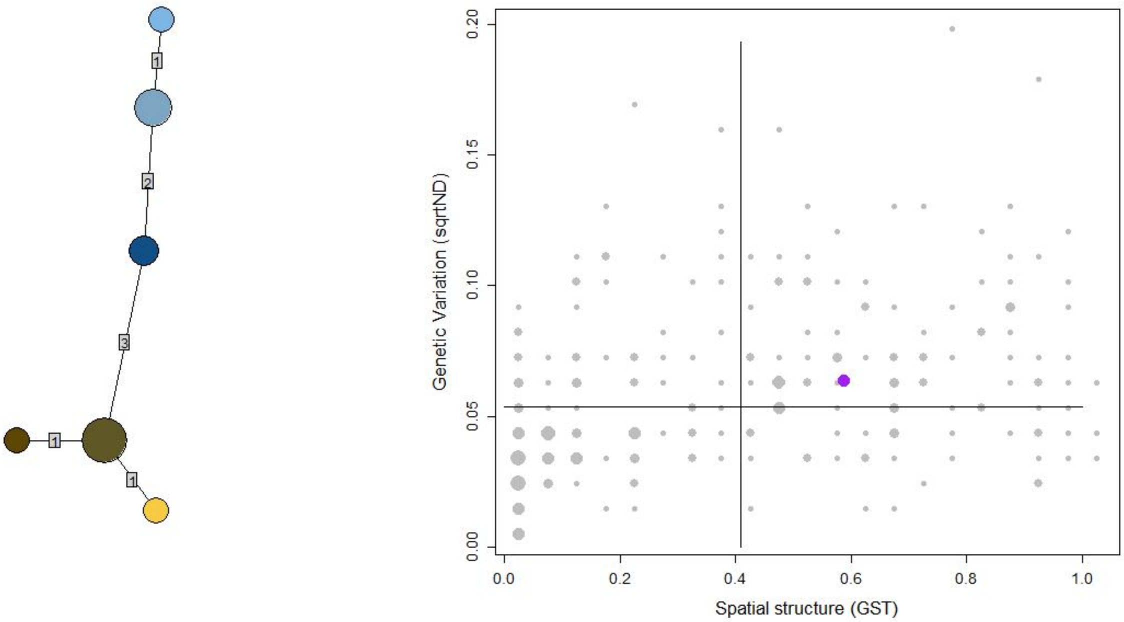

Figure 276: Haplotype network (left) of *Erynnis somnus* sequences > 599 bp with colours matching the PCoA colour space (above). The bubble plot for mt-DNA polymorphism (square root transformed nucleotide diversity) and spatial structure (GST) among all species in the atlas and values for *Erynnis somnus* (purple dot). The horizontal and vertical lines represent median values of nucleotide diversity and GST, respectively. Sequences > 599 bp= 20.

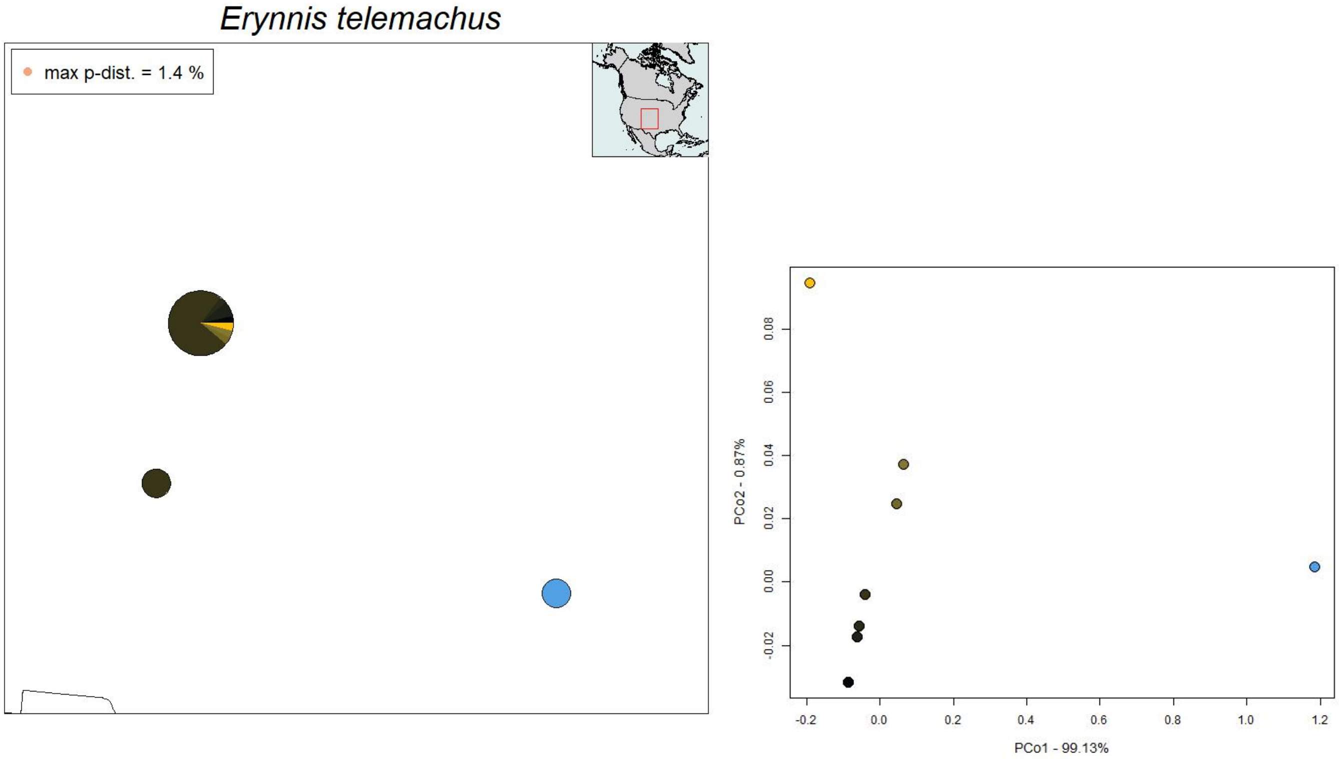

Figure 277 Map of *Erynnis telemachus* showing the localities of the sequenced specimens (left). Nearby localities are grouped in pies. Colours match the bidimensional colour space of the PCoA projection (right) of max p-dists among sequences (dots). Sequences= 29; Hap obs.= 3; Hap asympt.= 4; Hap % obs.= 75.6%; GST= 0; DST= 0; HD= 0.135; ND= 0.0009; max p-dist= 1.4%.

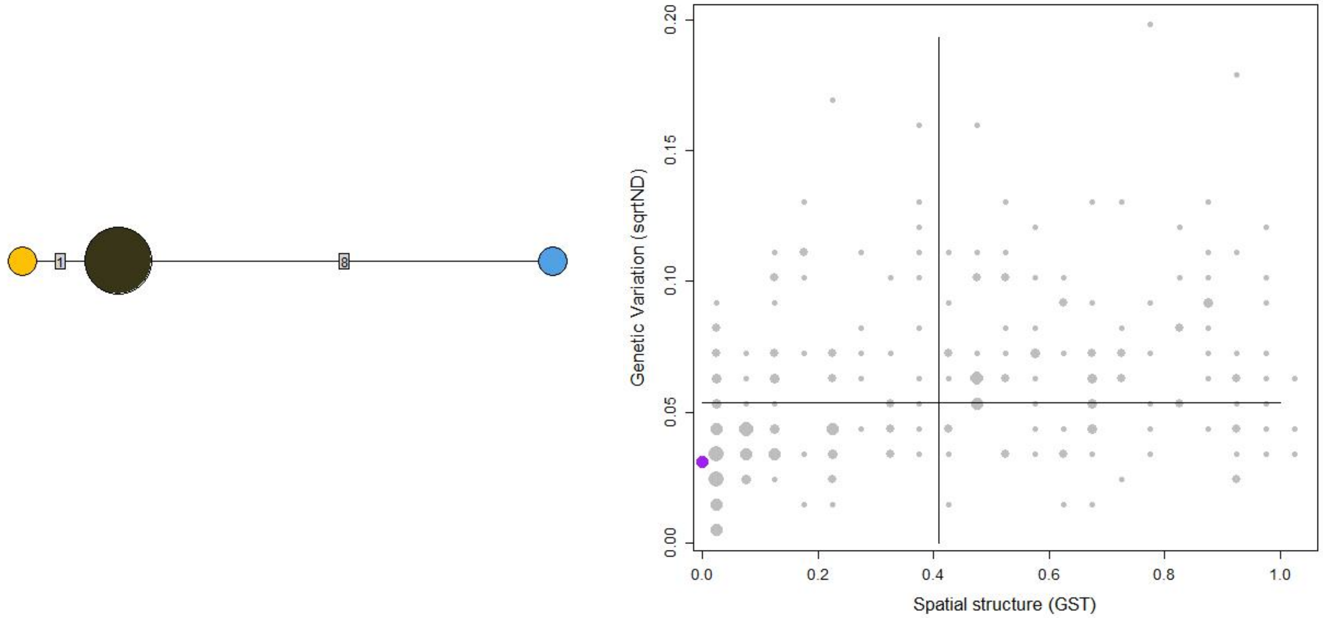

Figure 278: Haplotype network (left) of *Erynnis telemachus* sequences > 599 bp with colours matching the PCoA colour space (above). The bubble plot for mt-DNA polymorphism (square root transformed nucleotide diversity) and spatial structure (GST) among all species in the atlas and values for *Erynnis telemachus* (purple dot). The horizontal and vertical lines represent median values of nucleotide diversity and GST, respectively. Sequences > 599 bp= 29.

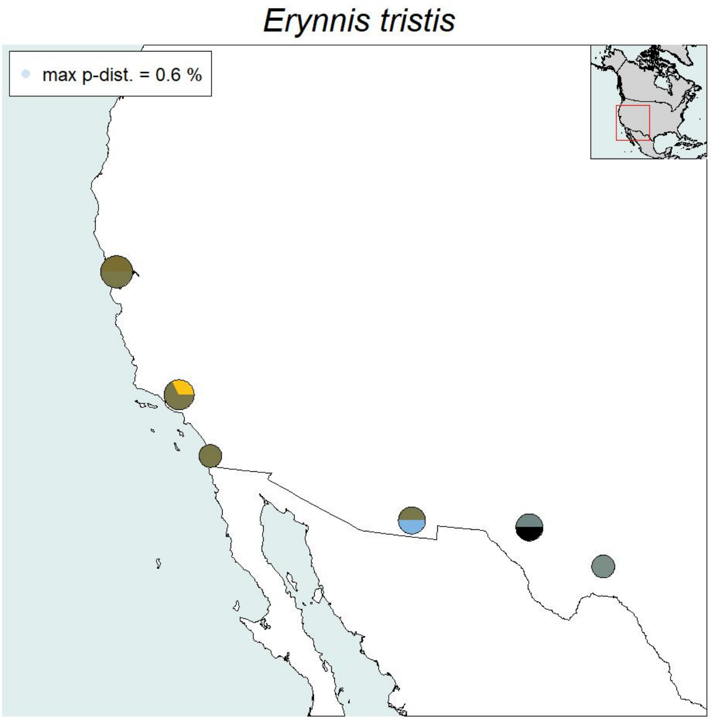

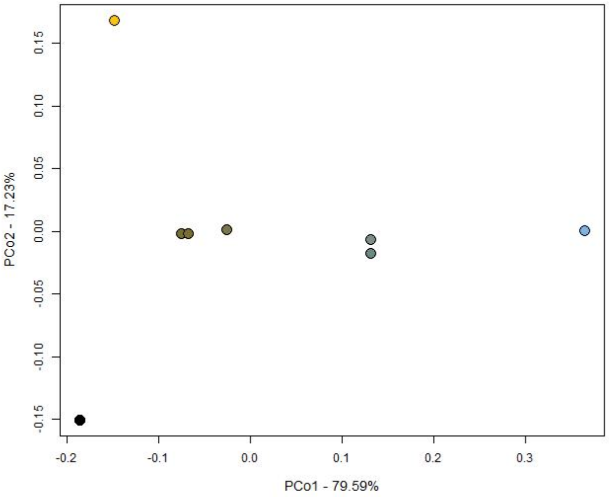

Figure 279 Map of *Erynnis tristis* showing the localities of the sequenced specimens (left). Nearby localities are grouped in pies. Colours match the bidimensional colour space of the PCoA projection (right) of max p-dists among sequences (dots). Sequences= 13; Hap obs.= 5; Hap asympt.= 9.2; Hap % obs.= 54.6%; GST= NaN; DST= NaN; HD= 0.628; ND= 0.0014; max p-dist= 0.6%.

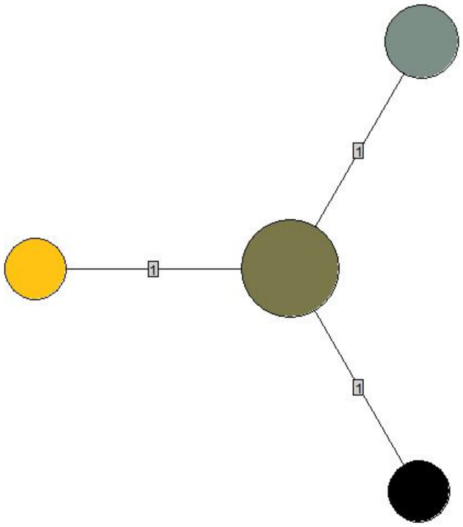

Figure 280: Haplotype network of *Erynnis tristis*. Sequences > 599 bp= 10.

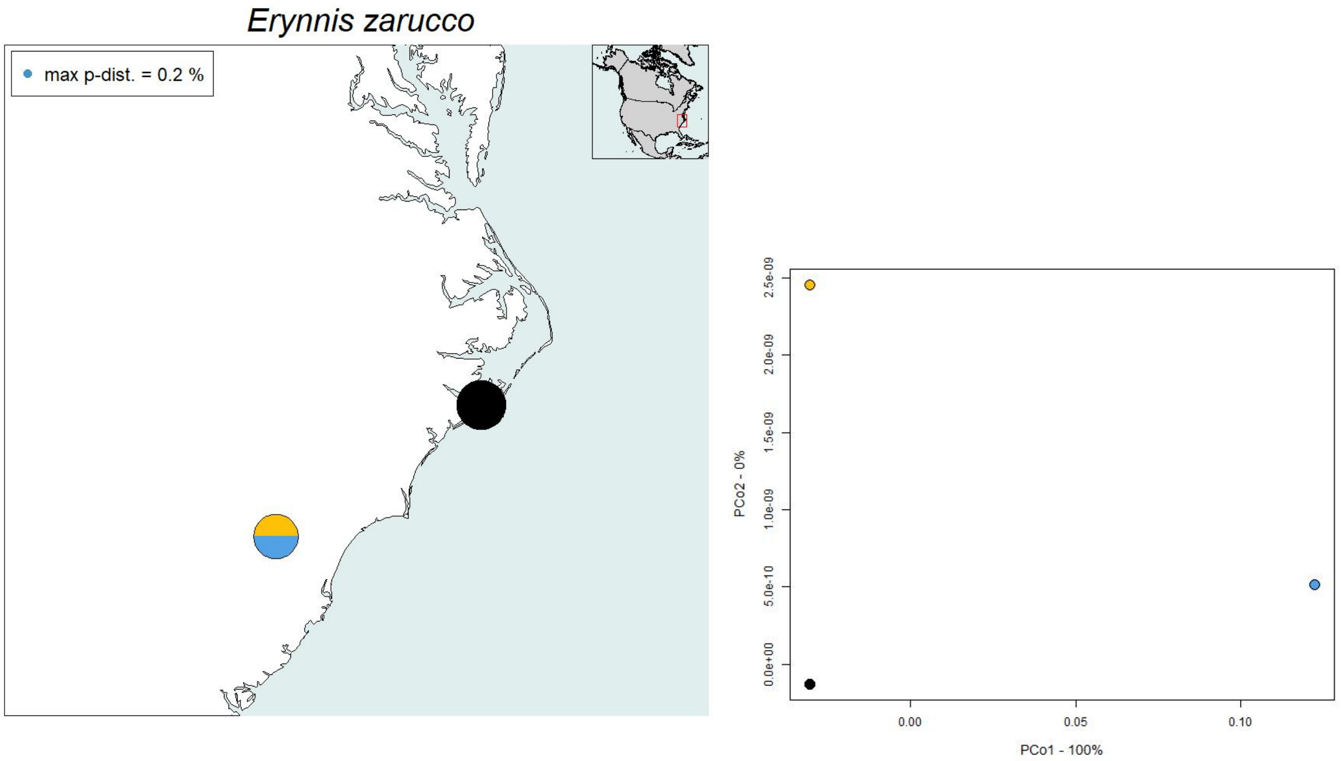

Figure 281 Map of *Erynnis zarucco* showing the localities of the sequenced specimens (left). Nearby localities are grouped in pies. Colours match the bidimensional colour space of the PCoA projection (right) of max p-dists among sequences (dots). Sequences= 5; Hap obs.= 2; Hap asympt.= NA; Hap % obs.= NA%; GST= NaN; DST= NaN; HD= NA; ND= NA; max p-dist= 0.2%.

Haplotype network analysis and bubble plot of *Erynnis zarucco* were not possible. Sequences > 599 bp = 5.

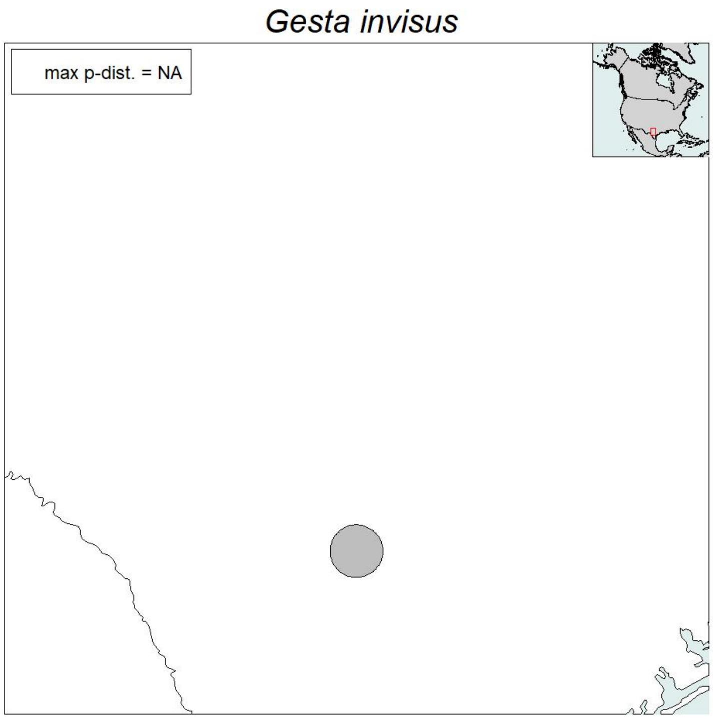

Figure 282: Map of *Gesta invisus* showing the localities of the sequenced specimens. Nearby localities are grouped in pies. Due to the presence of a single haplotype PCoA projection was not done and a single grey colour was plotted on the map. Sequences= 1; Hap obs.= NA; Hap asympt.= NA; Hap % obs.= NA; GST= NaN; DST= NaN; HD= NA; ND= NA; max p-dist= NA.

Haplotype network analysis and bubble plot of *Gesta invisus* were not possible. Sequences > 599 bp = 1.

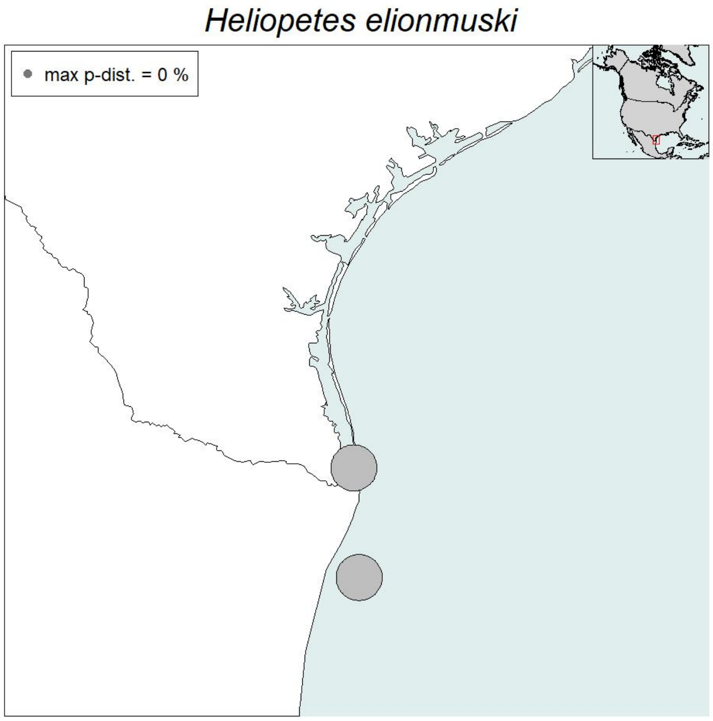

Figure 283: Map of *Heliopetes elionmuski* showing the localities of the sequenced specimens. Nearby localities are grouped in pies. Due to the presence of a single haplotype PCoA projection was not done and a single grey colour was plotted on the map. Sequences= 2; Hap obs.= 1; Hap asympt.= NA; Hap % obs.= NA%; GST= NaN; DST= NaN; HD= NA; ND= NA; max p-dist= 0%.

Haplotype network analysis and bubble plot of *Heliopetes elionmuski* were not possible. Sequences $>$ 599 bp = 2.

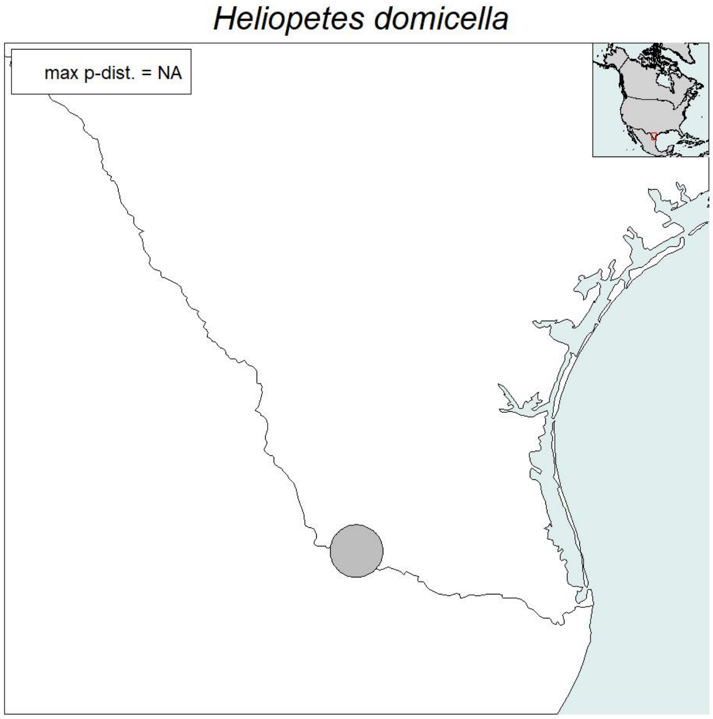

Figure 284: Map of *Heliopetes domicella* showing the localities of the sequenced specimens. Nearby localities are grouped in pies. Due to the presence of a single haplotype PCoA projection was not done and a single grey colour was plotted on the map. Sequences= 1; Hap obs.= NA; Hap asympt.= NA; Hap % obs.= NA; GST= NaN; DST= NaN; HD= NA; ND= NA; max p-dist= NA.

Haplotype network analysis and bubble plot of *Heliopetes domicella* were not possible. Sequences > 599 bp = 1.

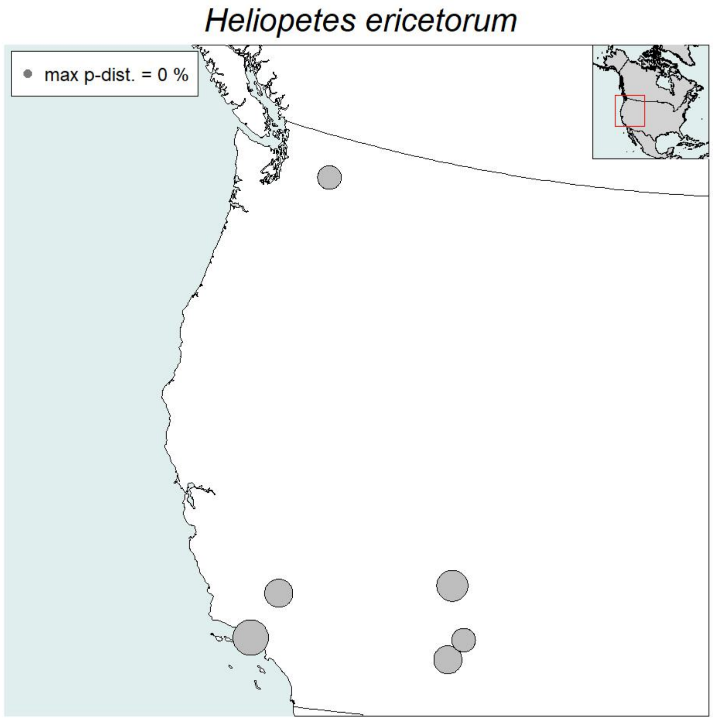

Figure 285: Map of *Heliopetes ericetorum* showing the localities of the sequenced specimens. Nearby localities are grouped in pies. Due to the presence of a single haplotype PCoA projection was not done and a single grey colour was plotted on the map. Sequences= 14; Hap obs.= 1; Hap asympt.= NA; Hap % obs.= NA%; GST= 0; DST= 0; HD= 0; ND= 0; max p-dist= 0%.

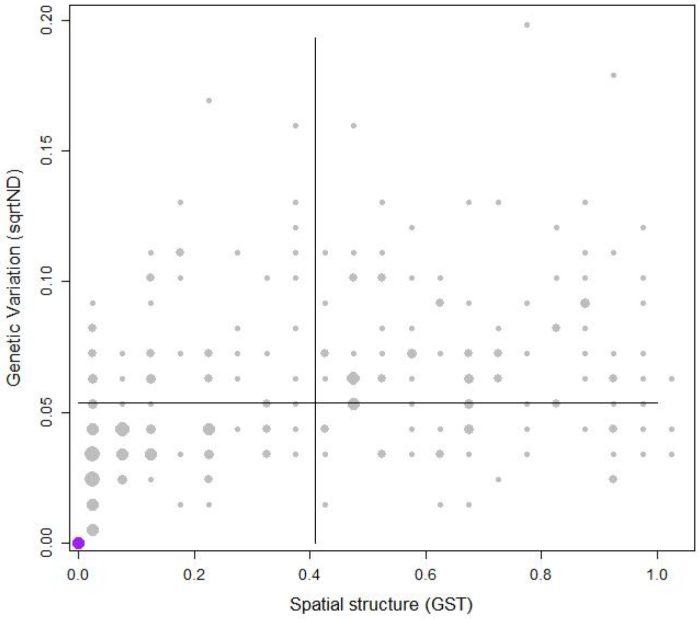

Figure 286: The bubble plot for mt-DNA polymorphism (square root transformed nucleotide diversity) and spatial structuree (GST) among all species in the atlas and values for *Heliopetes ericetorum* (purple dot). The horizontal and vertical lines represent median values of nucleotide diversity and GST, respectively. Haplotype network analysis was not possible. Sequences > 599 bp = 14.

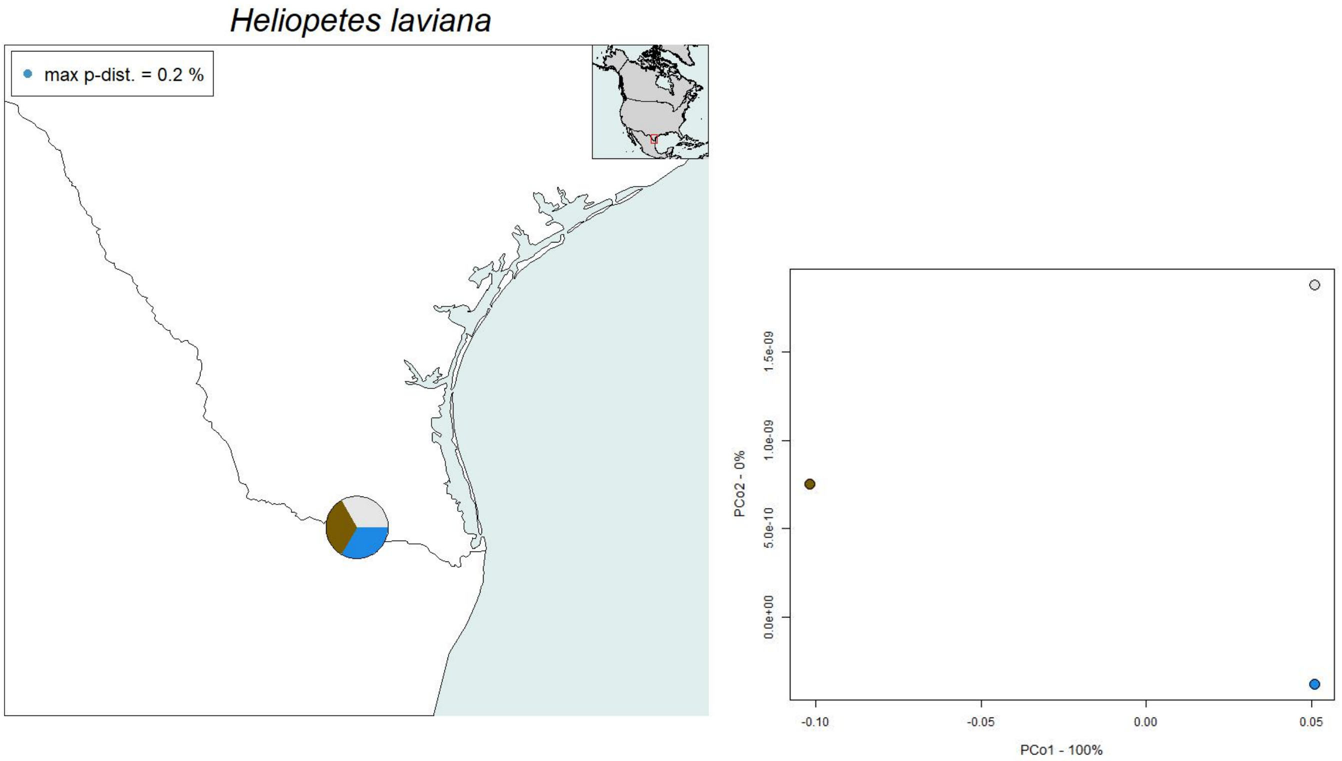

Figure 287 Map of *Heliopetes laviana* showing the localities of the sequenced specimens (left). Nearby localities are grouped in pies. Colours match the bidimensional colour space of the PCoA projection (right) of max p-dists among sequences (dots). Sequences= 3; Hap obs.= 2; Hap asympt.= NA; Hap % obs.= NA%; GST= NaN; DST= NaN; HD= NA; ND= NA; max p-dist= 0.2%.

Haplotype network analysis and bubble plot of *Heliopetes laviana* were not possible. Sequences > 599 bp = 3.

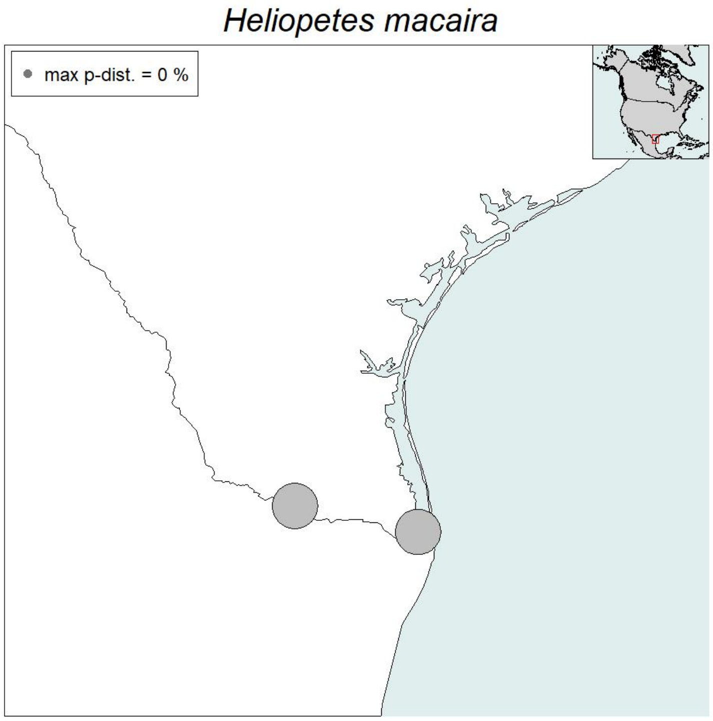

Figure 288: Map of *Heliopetes macaira* showing the localities of the sequenced specimens. Nearby localities are grouped in pies. Due to the presence of a single haplotype PCoA projection was not done and a single grey colour was plotted on the map. Sequences= 2; Hap obs.= 1; Hap asympt.= NA; Hap % obs.= NA%; GST= NaN; DST= NaN; HD= NA; ND= NA; max p-dist= 0%.

Haplotype network analysis and bubble plot of *Heliopetes macaira* were not possible. Sequences > 599 bp = 2.

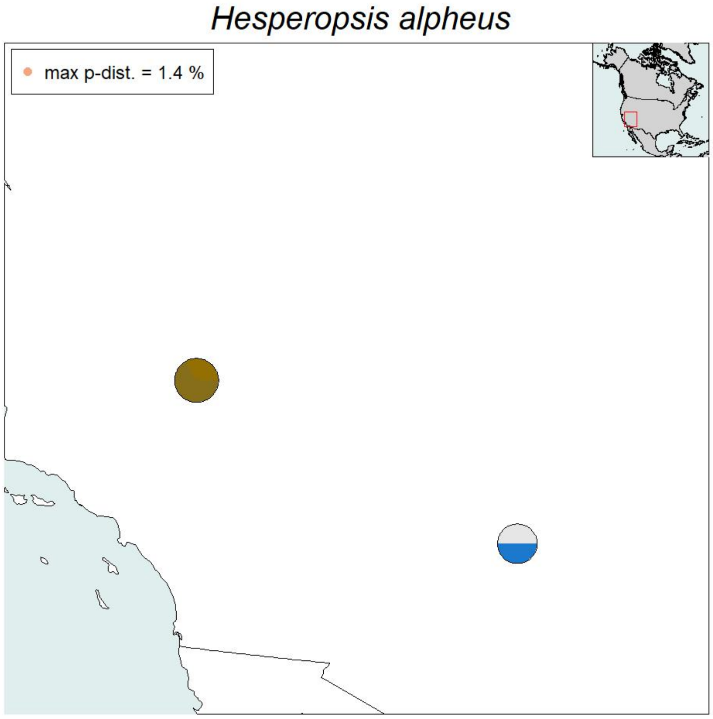

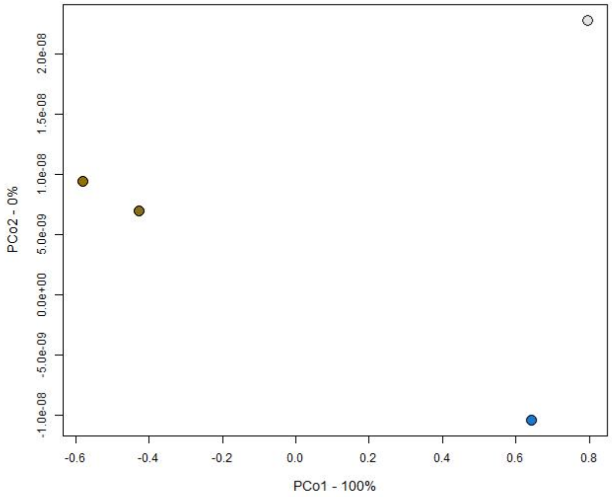

Figure 289 Map of *Hesperopsis alpheus* showing the localities of the sequenced specimens (left). Nearby localities are grouped in pies. Colours match the bidimensional colour space of the PCoA projection (right) of max p-dists among sequences (dots). Sequences= 5; Hap obs.= 4; Hap asympt.= NA; Hap % obs.= NA%; GST= NaN; DST= NaN; HD= NA; ND= NA; max p-dist= 1.4%.

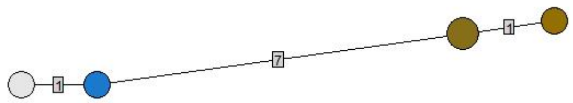

Figure 290: Haplotype network of *Hesperopsis alpheus*. Sequences > 599 bp= 5.

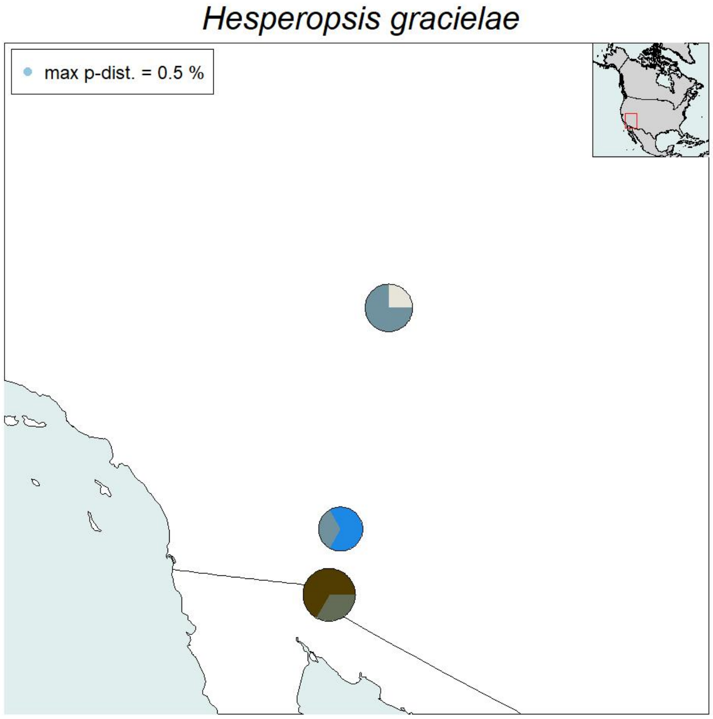

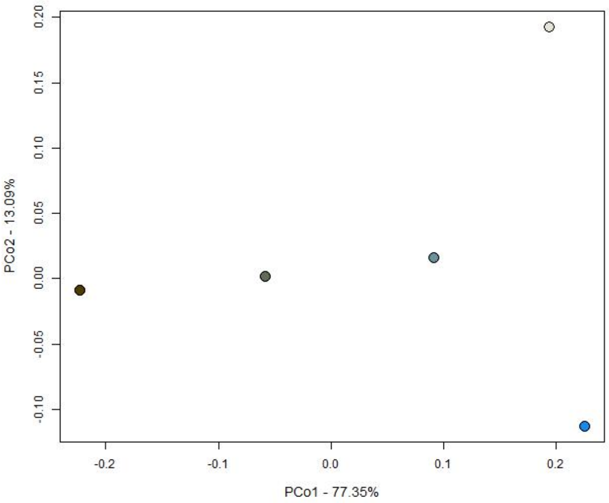

Figure 291 Map of *Hesperopsis gracielae* showing the localities of the sequenced specimens (left). Nearby localities are grouped in pies. Colours match the bidimensional colour space of the PCoA projection (right) of max p-dists among sequences (dots). Sequences= 13; Hap obs.= 6; Hap asympt.= 10.2; Hap % obs.= 59.1%; GST= 0.24; DST= 0.0005; HD= 0.833; ND= 0.0022; max p-dist= 0.5%.

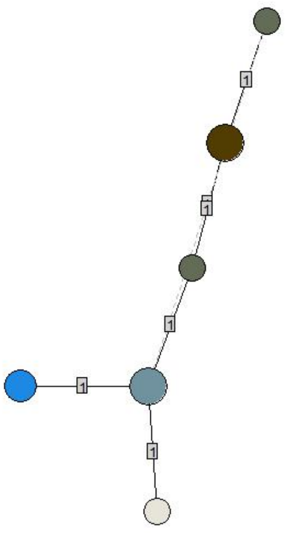

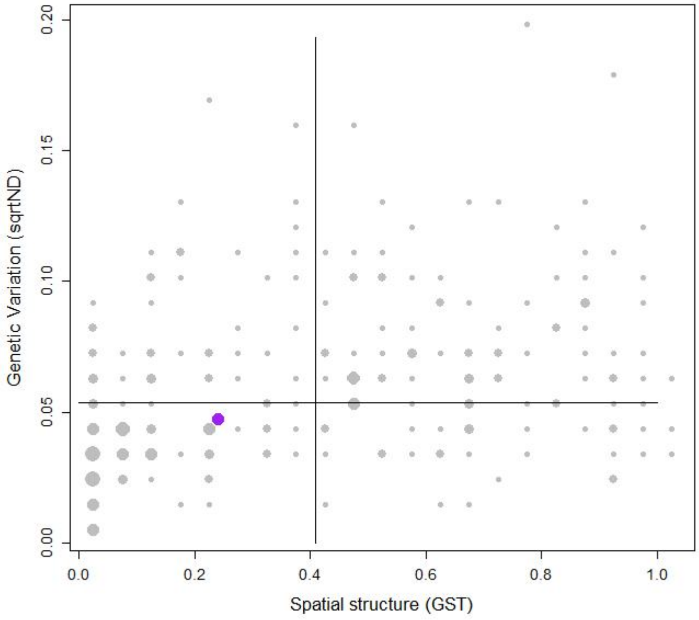

Figure 292: Haplotype network (left) of *Hesperopsis gracielae* sequences > 599 bp with colours matching the PCoA colour space (above). The bubble plot for mt-DNA polymorphism (square root transformed nucleotide diversity) and spatial structure (GST) among all species in the atlas and values for *Hesperopsis gracielae* (purple dot). The horizontal and vertical lines represent median values of nucleotide diversity and GST, respectively. Sequences > 599 bp= 13.

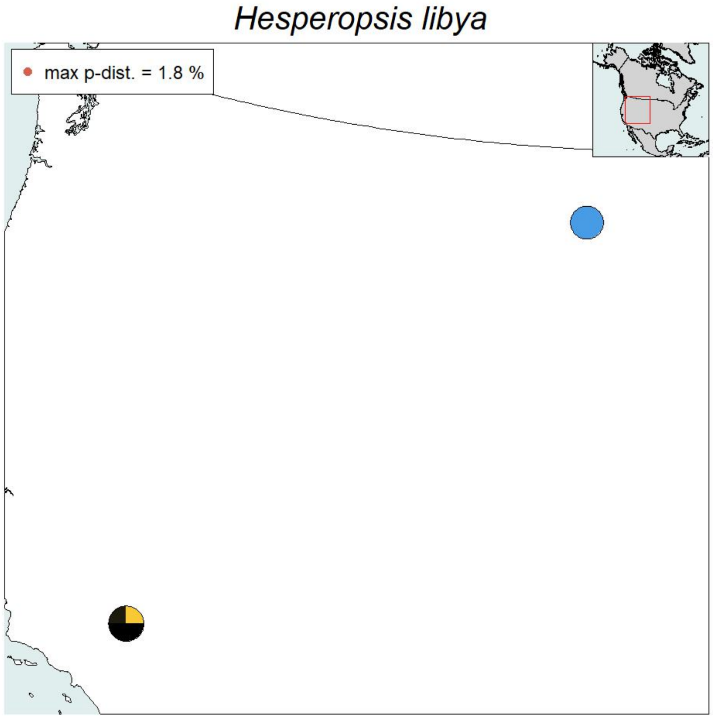

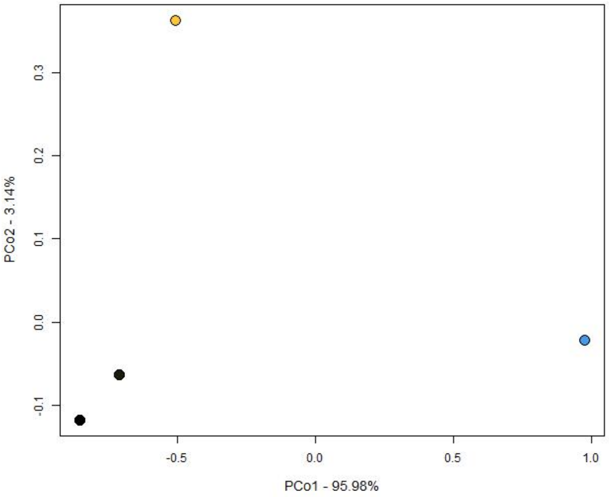

Figure 293 Map of *Hesperopsis libya* showing the localities of the sequenced specimens (left). Nearby localities are grouped in pies. Colours match the bidimensional colour space of the PCoA projection (right) of max p-dists among sequences (dots). Sequences= 7; Hap obs.= 5; Hap asympt.= NA; Hap % obs.= NA%; GST= NaN; DST= NaN; HD= NA; ND= NA; max p-dist= 1.8%.

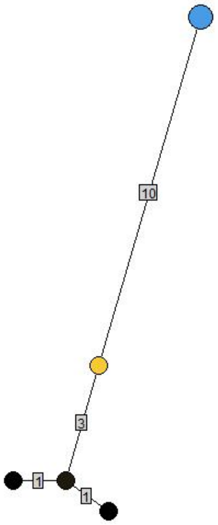

Figure 294: Haplotype network of *Hesperopsis libya*. Sequences > 599 bp= 7.

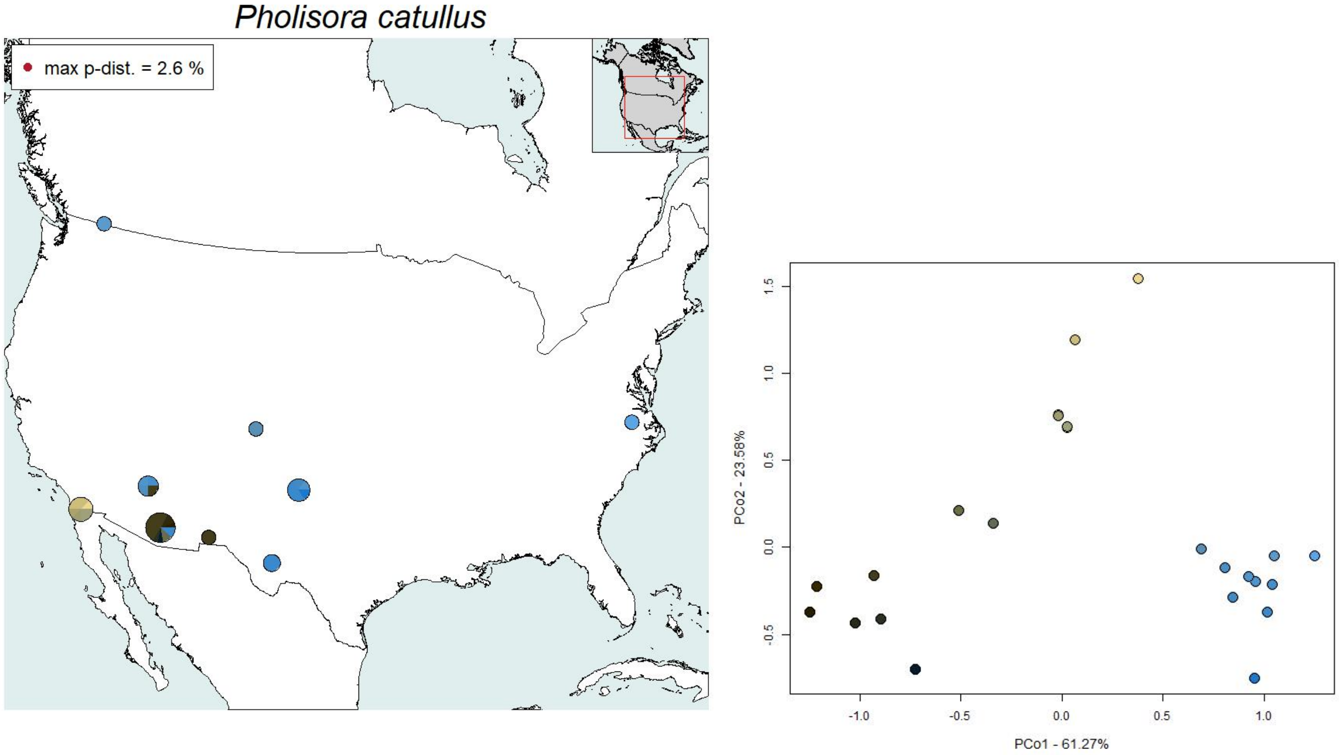

Figure 295 Map of *Pholisora catullus* showing the localities of the sequenced specimens (left). Nearby localities are grouped in pies. Colours match the bidimensional colour space of the PCoA projection (right) of max p-dists among sequences (dots). Sequences= 42; Hap obs.= 21; Hap asympt.= 162.1; Hap % obs.= 13%; GST= 0.47; DST= 0.0059; HD= 0.899; ND= 0.0128; max p-dist= 2.6%.

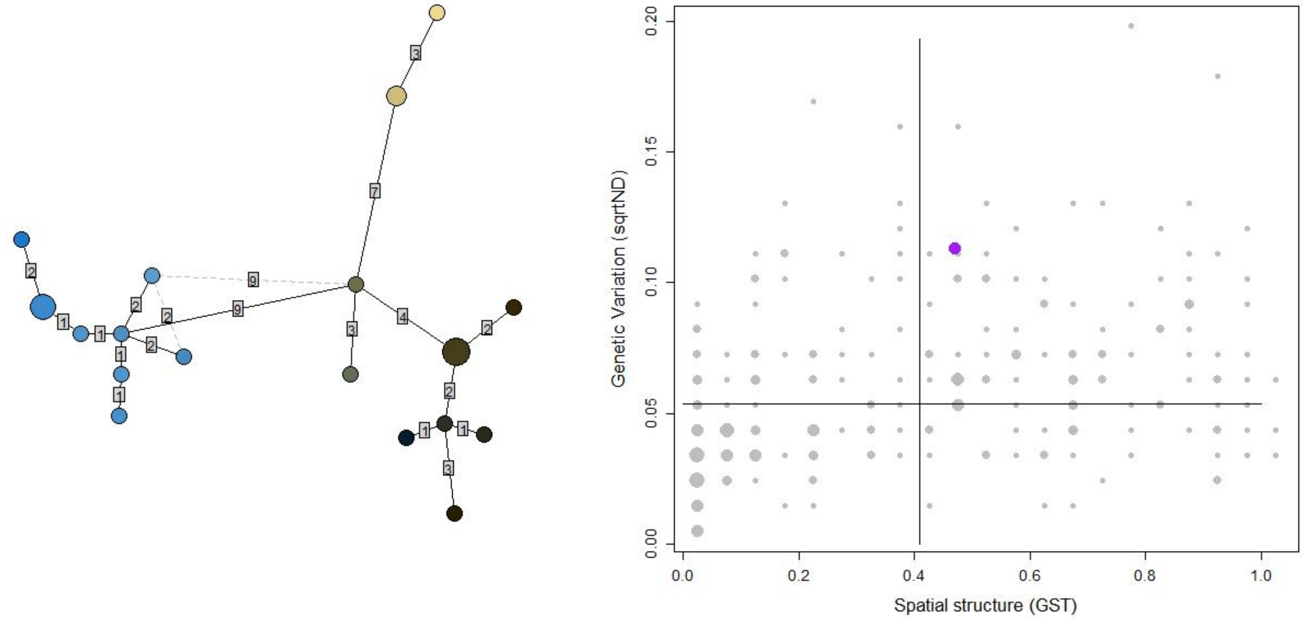

Figure 296: Haplotype network (left) of *Pholisora catullus* sequences > 599 bp with colours matching the PCoA colour space (above). The bubble plot for mt-DNA polymorphism (square root transformed nucleotide diversity) and spatial structure (GST) among all species in the atlas and values for *Pholisora catullus* (purple dot). The horizontal and vertical lines represent median values of nucleotide diversity and GST, respectively. Sequences > 599 bp= 36.

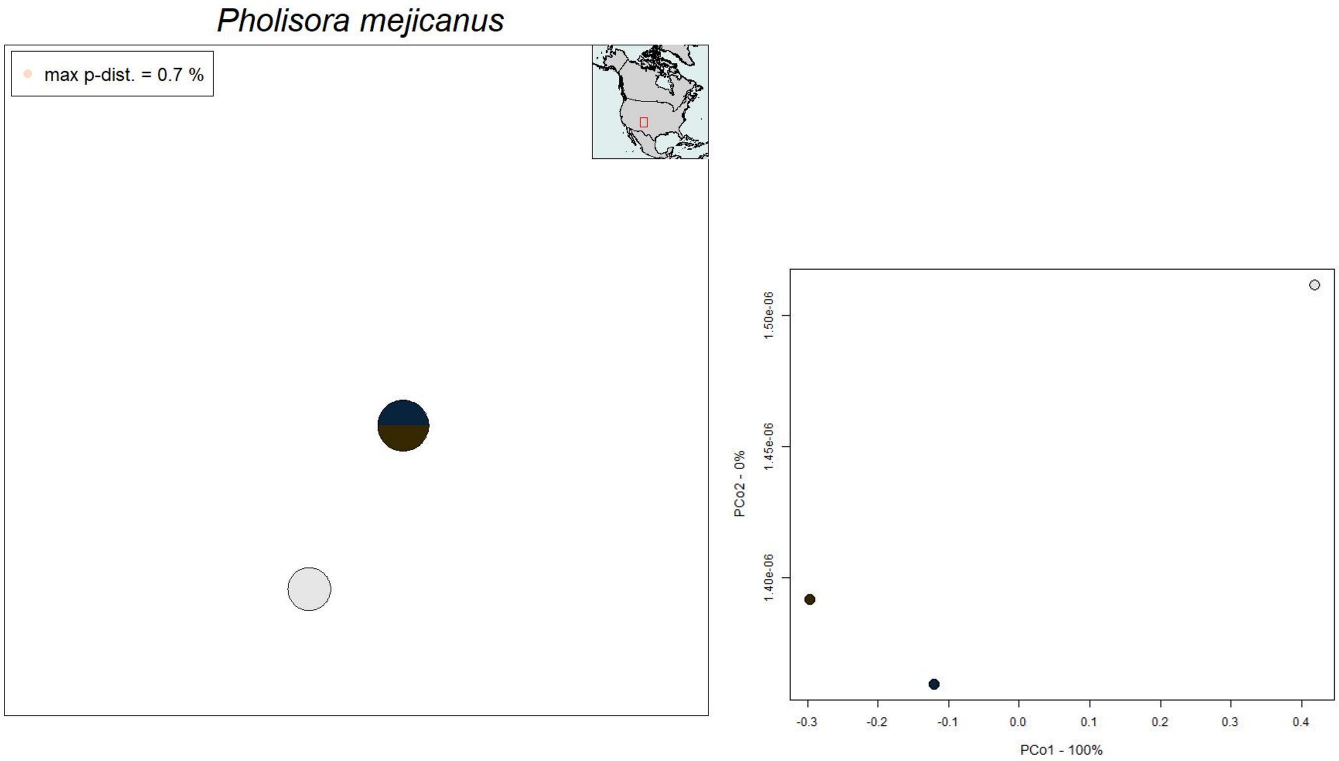

Figure 297 Map of *Pholisora mejicanus* showing the localities of the sequenced specimens (left). Nearby localities are grouped in pies. Colours match the bidimensional colour space of the PCoA projection (right) of max p-dists among sequences (dots). Sequences= 3; Hap obs.= 3; Hap asympt.= NA; Hap % obs.= NA%; GST= NaN; DST= NaN; HD= NA; ND= NA; max p-dist= 0.7%.

Haplotype network analysis and bubble plot of *Pholisora mejicanus* were not possible. Sequences > 599 bp = 2.

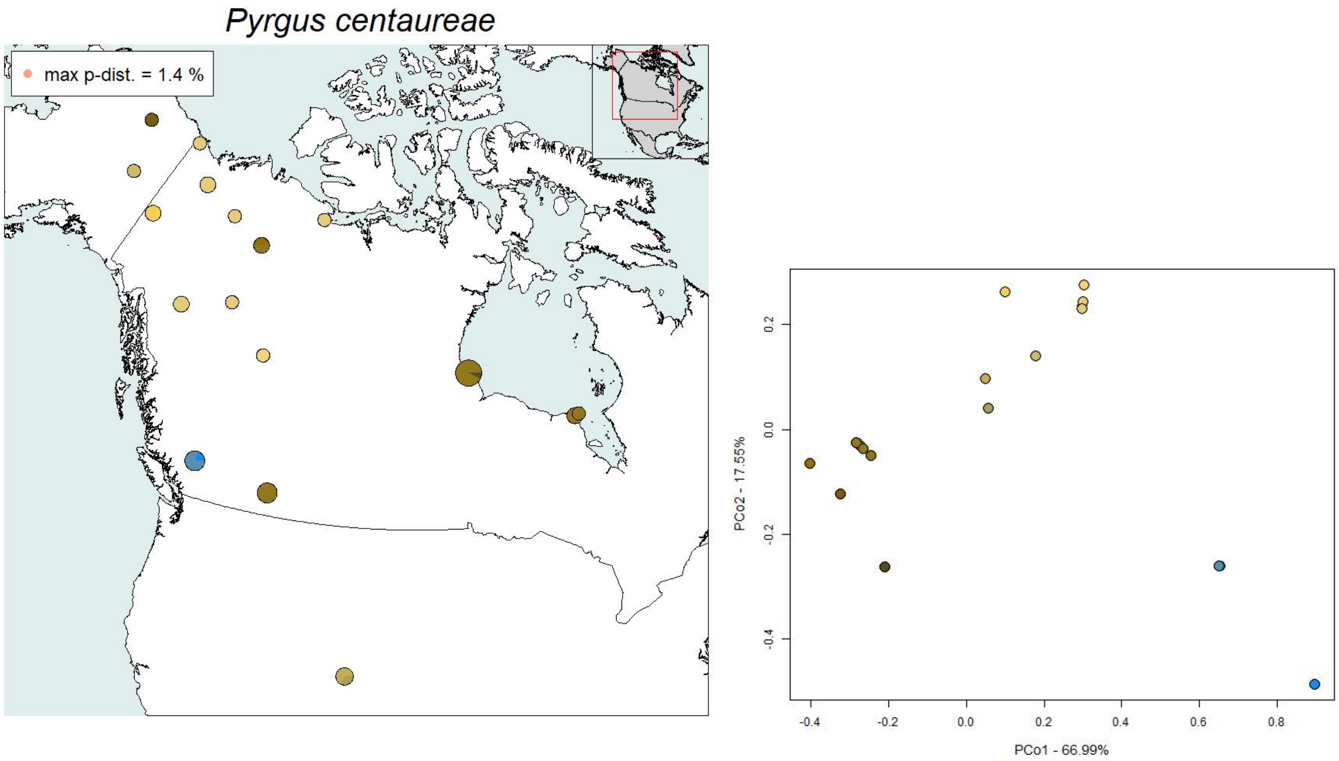

Figure 298 Map of *Pyrgus centaureae* showing the localities of the sequenced specimens (left). Nearby localities are grouped in pies. Colours match the bidimensional colour space of the PCoA projection (right) of max p-dists among sequences (dots). Sequences= 46; Hap obs.= 9; Hap asympt.= 16.8; Hap % obs.= 53.5%; GST= 0.965; DST= 0.0037; HD= 0.689; ND= 0.0044; max p-dist= 1.4%.

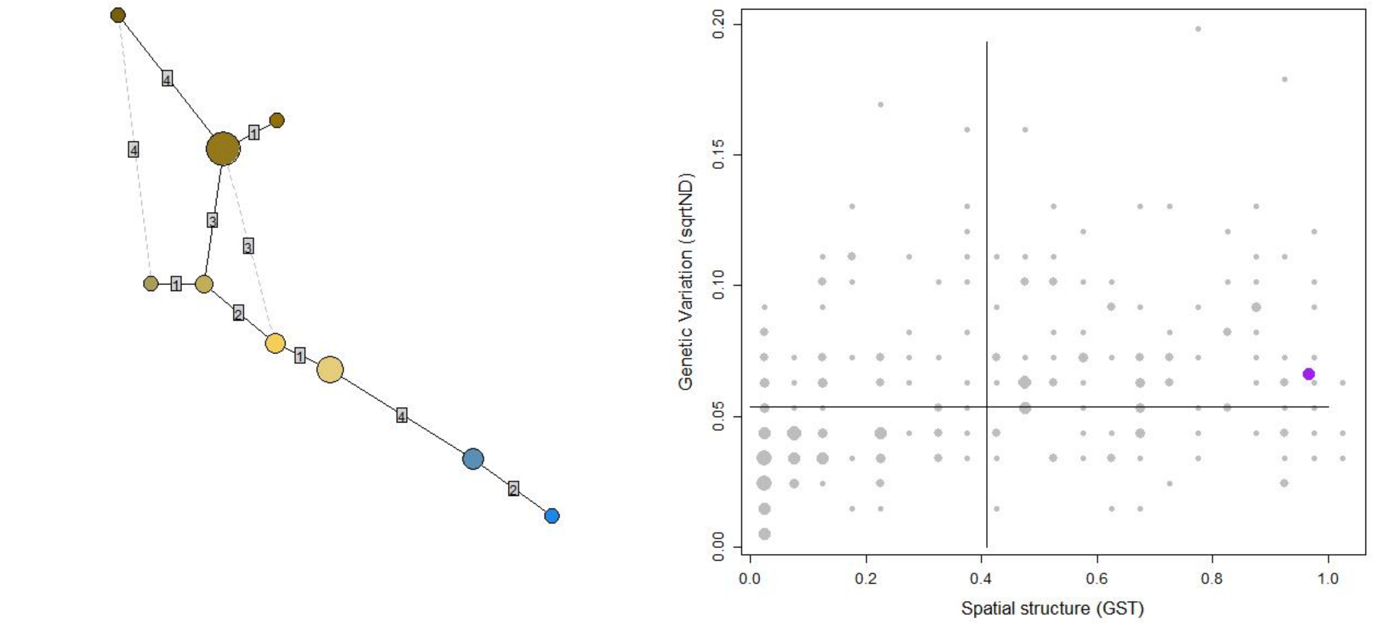

Figure 299: Haplotype network (left) of *Pyrgus centaureae* sequences > 599 bp with colours matching the PCoA colour space (above). The bubble plot for mt-DNA polymorphism (square root transformed nucleotide diversity) and spatial structure (GST) among all species in the atlas and values for *Pyrgus centaureae* (purple dot). The horizontal and vertical lines represent median values of nucleotide diversity and GST, respectively. Sequences > 599 bp= 45.

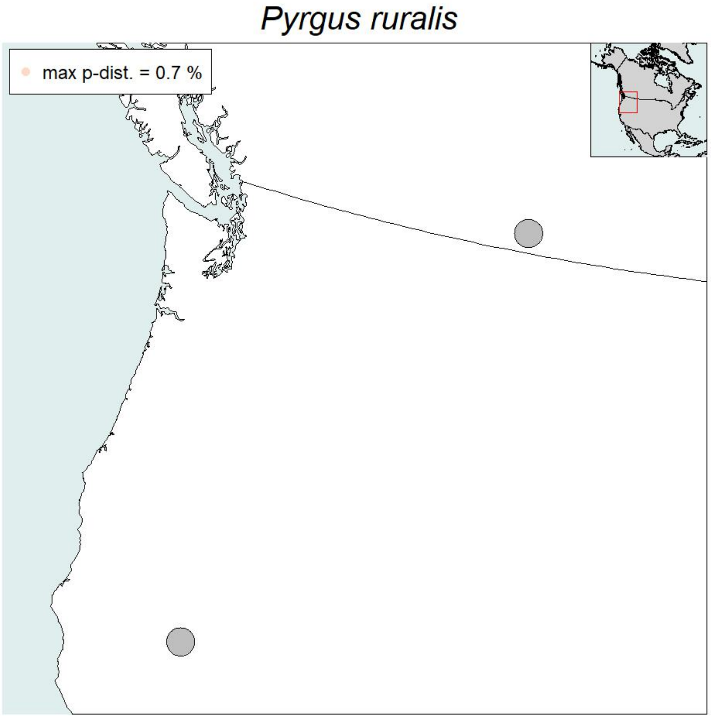

Figure 300: Map of *Pyrgus ruralis* showing the localities of the sequenced specimens. Nearby localities are grouped in pies. Due to the presence of a single haplotype PCoA projection was not done and a single grey colour was plotted on the map. Sequences= 2; Hap obs.= 2; Hap asympt.= NA; Hap % obs.= NA%; GST= NaN; DST= NaN; HD= NA; ND= NA; max p-dist= 0.7%.

Haplotype network analysis and bubble plot of *Pyrgus ruralis* were not possible. Sequences > 599 bp = 1.

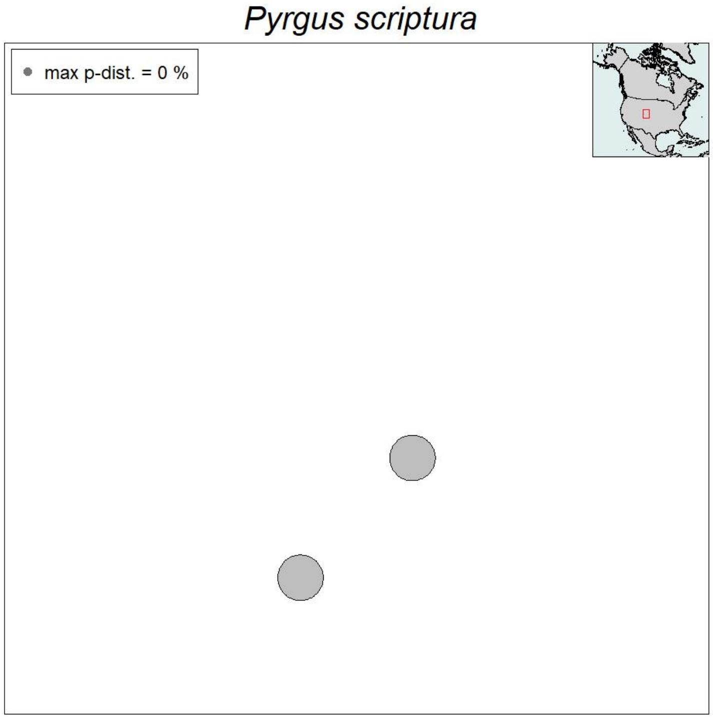

Figure 301: Map of *Pyrgus scriptura* showing the localities of the sequenced specimens. Nearby localities are grouped in pies. Due to the presence of a single haplotype PCoA projection was not done and a single grey colour was plotted on the map. Sequences= 2; Hap obs.= 1; Hap asympt.= NA; Hap % obs.= NA%; GST= NaN; DST= NaN; HD= NA; ND= NA; max p-dist= 0%.

Haplotype network analysis and bubble plot of *Pyrgus scriptura* were not possible. Sequences > 599 bp = 2.

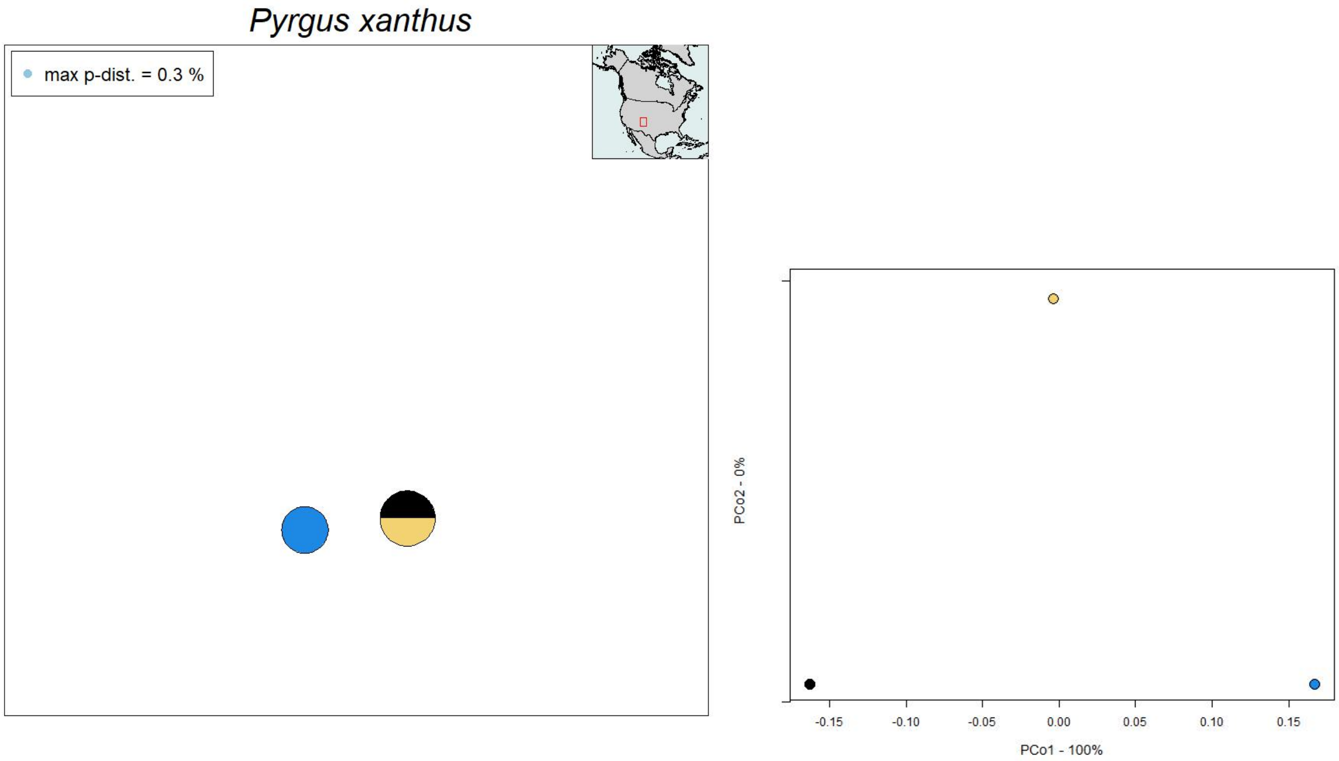

Figure 302 Map of *Pyrgus xanthus* showing the localities of the sequenced specimens (left). Nearby localities are grouped in pies. Colours match the bidimensional colour space of the PCoA projection (right) of max p-dists among sequences (dots). Sequences= 3; Hap obs.= 3; Hap asympt.= NA; Hap % obs.= NA%; GST= NaN; DST= NaN; HD= NA; ND= NA; max p-dist= 0.3%.

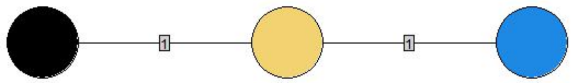

Figure 303: Haplotype network of *Pyrgus xanthus*. Sequences > 599 bp= 3.

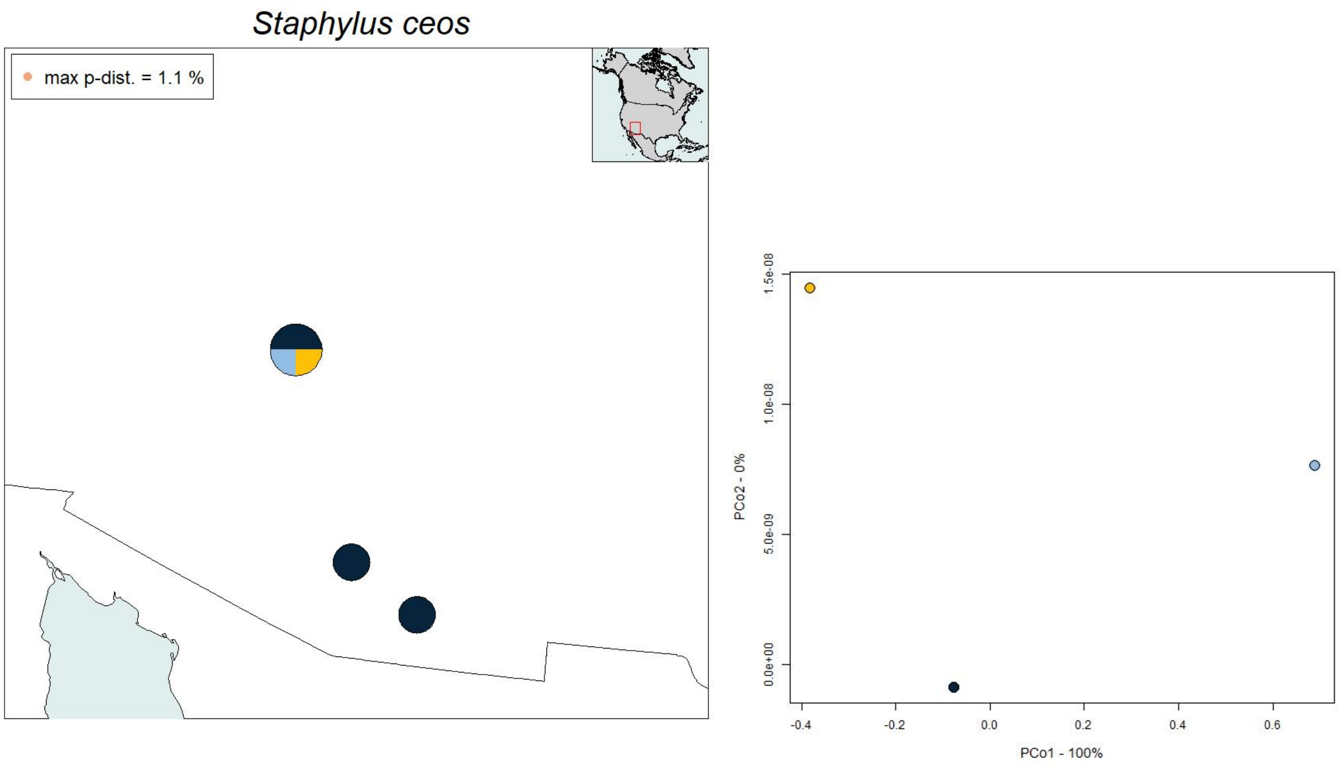

Figure 304 Map of *Staphylus ceos* showing the localities of the sequenced specimens (left). Nearby localities are grouped in pies. Colours match the bidimensional colour space of the PCoA projection (right) of max p-dists among sequences (dots). Sequences= 6; Hap obs.= 3; Hap asympt.= NA; Hap % obs.= NA%; GST= NaN; DST= NaN; HD= NA; ND= NA; max p-dist= 1.1%.

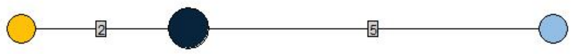

Figure 305: Haplotype network of *Staphylus ceos*. Sequences > 599 bp= 6.

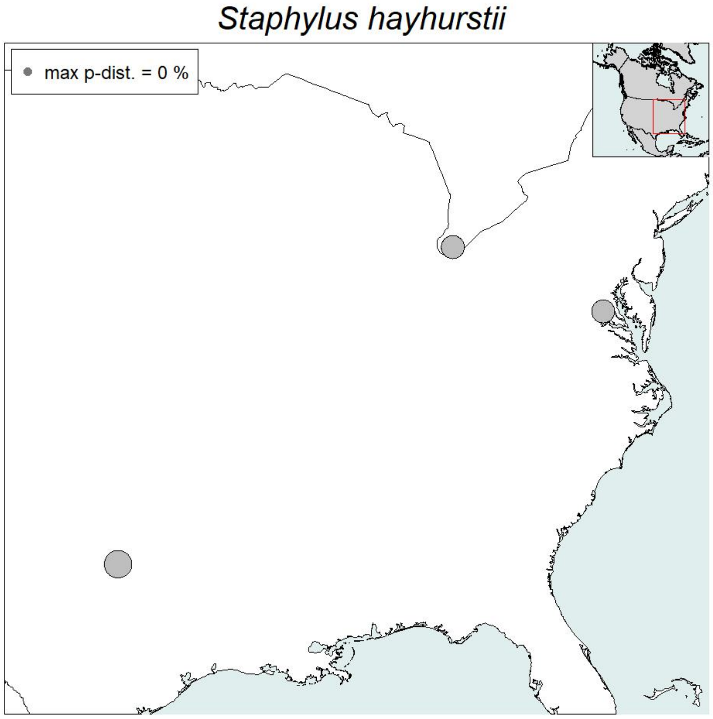

Figure 306: Map of *Staphylus hayhurstii* showing the localities of the sequenced specimens. Nearby localities are grouped in pies. Due to the presence of a single haplotype PCoA projection was not done and a single grey colour was plotted on the map. Sequences= 4; Hap obs.= 1; Hap asympt.= NA; Hap % obs.= NA%; GST= NaN; DST= NaN; HD= NA; ND= NA; max p-dist= 0%.

Haplotype network analysis and bubble plot of *Staphylus hayhurstii* were not possible. Sequences > 599 bp = 3.

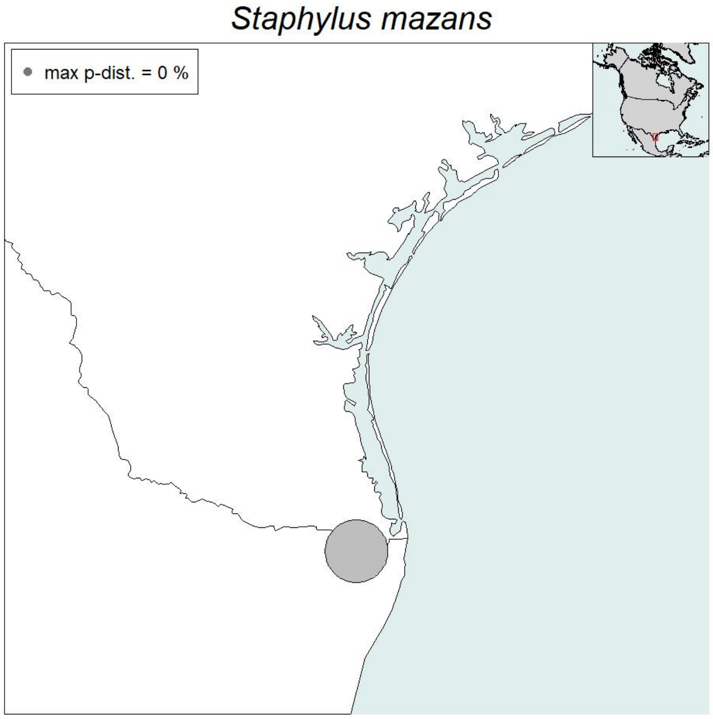

Figure 307: Map of *Staphylus mazans* showing the localities of the sequenced specimens. Nearby localities are grouped in pies. Due to the presence of a single haplotype PCoA projection was not done and a single grey colour was plotted on the map. Sequences= 2; Hap obs.= 1; Hap asympt.= NA; Hap % obs.= NA%; GST= NaN; DST= NaN; HD= NA; ND= NA; max p-dist= 0%.

Haplotype network analysis and bubble plot of *Staphylus mazans* were not possible. Sequences > 599 bp = 2.

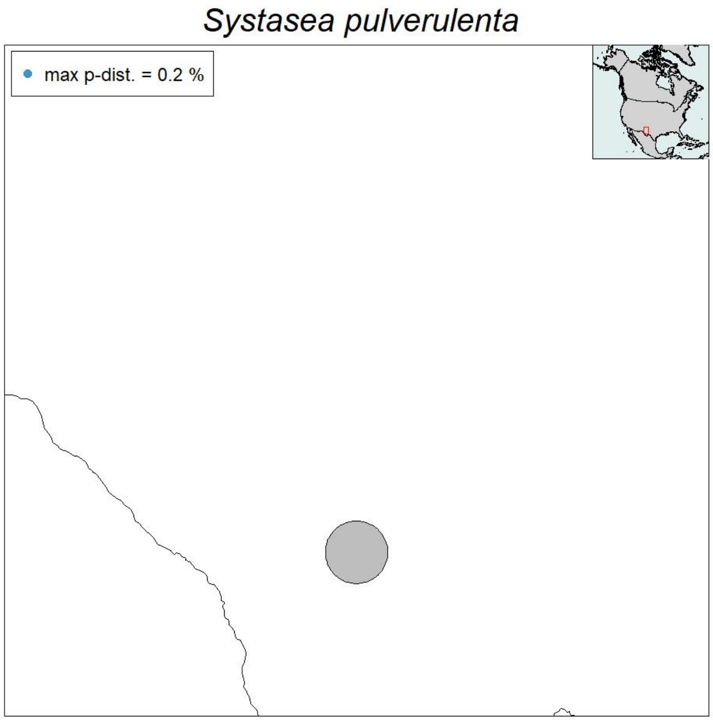

Figure 308: Map of *Systasea pulverulenta* showing the localities of the sequenced specimens. Nearby localities are grouped in pies. Due to the presence of a single haplotype PCoA projection was not done and a single grey colour was plotted on the map. Sequences= 2; Hap obs.= 2; Hap asympt.= NA; Hap % obs.= NA%; GST= NaN; DST= NaN; HD= NA; ND= NA; max p-dist= 0.2%.

Haplotype network analysis and bubble plot of *Systasea pulverulenta* were not possible. Sequences > 599 bp = 1.

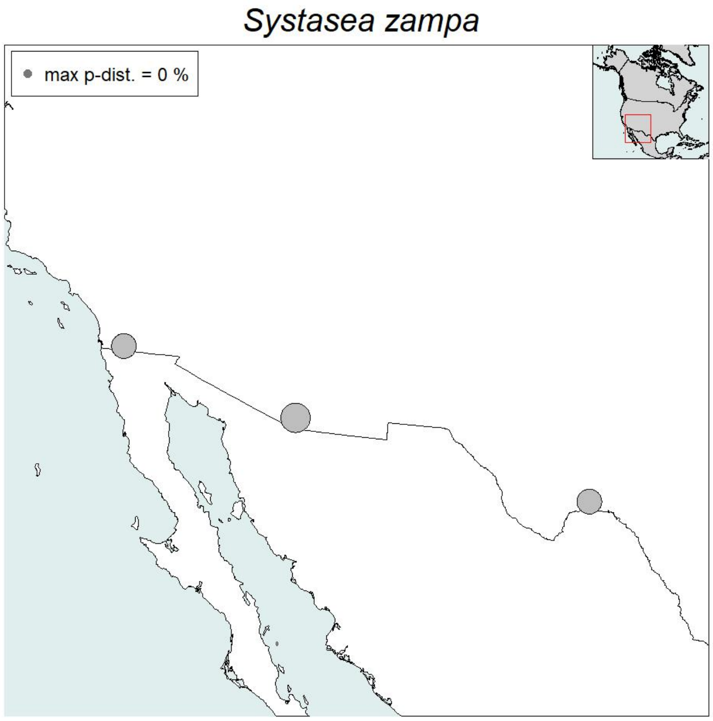

Figure 309: Map of *Systasea zampa* showing the localities of the sequenced specimens. Nearby localities are grouped in pies. Due to the presence of a single haplotype PCoA projection was not done and a single grey colour was plotted on the map. Sequences= 4; Hap obs.= 1; Hap asympt.= NA; Hap % obs.= NA%; GST= NaN; DST= NaN; HD= NA; ND= NA; max p-dist= 0%.

Haplotype network analysis and bubble plot of *Systasea zampa* were not possible. Sequences > 599 bp = 3.

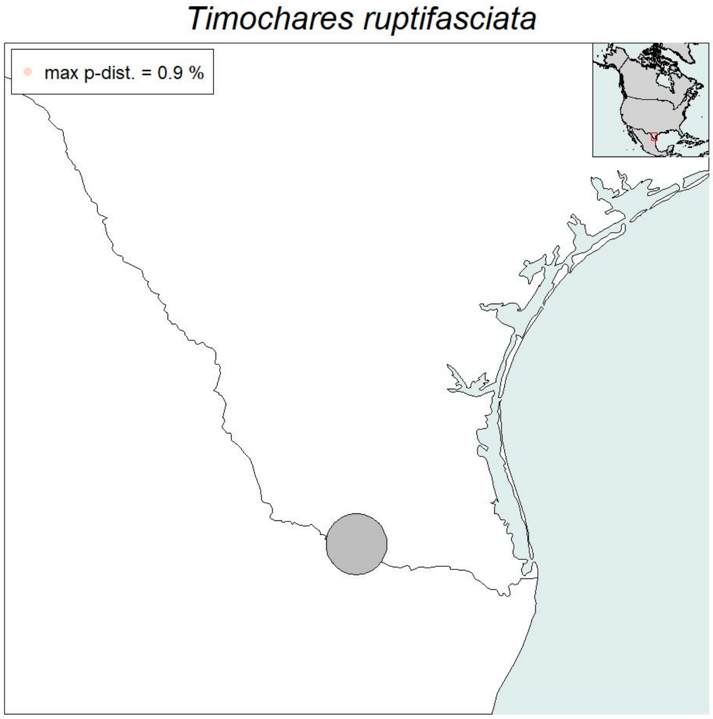

Figure 310: Map of *Timochares ruptifasciata* showing the localities of the sequenced specimens. Nearby localities are grouped in pies. Due to the presence of a single haplotype PCoA projection was not done and a single grey colour was plotted on the map. Sequences= 2; Hap obs.= 2; Hap asympt.= NA; Hap % obs.= NA%; GST= NaN; DST= NaN; HD= NA; ND= NA; max p-dist= 0.9%.

Haplotype network analysis and bubble plot of *Timochares ruptifasciata* were not possible. Sequences > 599 bp = 1.

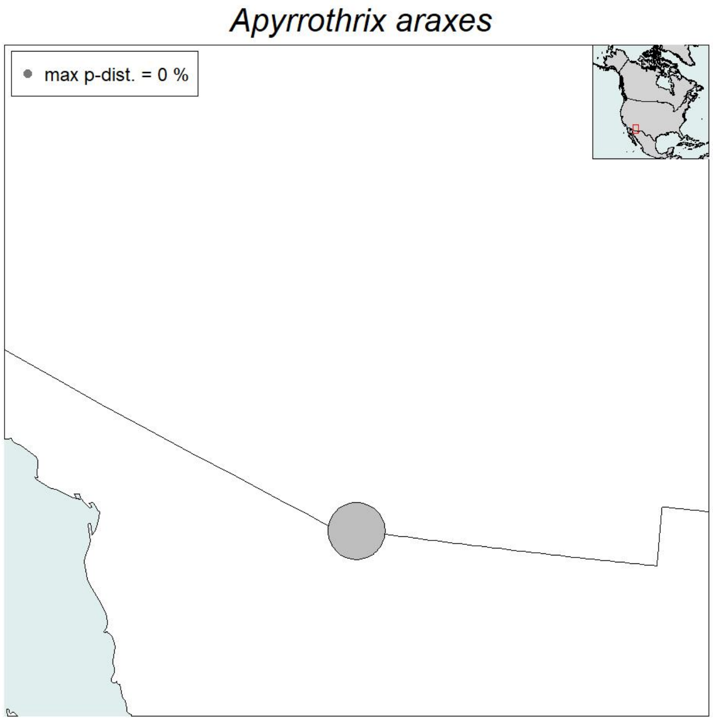

Figure 311: Map of *Apyrrothrix araxes* showing the localities of the sequenced specimens. Nearby localities are grouped in pies. Due to the presence of a single haplotype PCoA projection was not done and a single grey colour was plotted on the map. Sequences= 2; Hap obs.= 1; Hap asympt.= NA; Hap % obs.= NA%; GST= NaN; DST= NaN; HD= NA; ND= NA; max p-dist= 0%.

Haplotype network analysis and bubble plot of *Apyrrothrix araxes* were not possible. Sequences > 599 bp = 2.

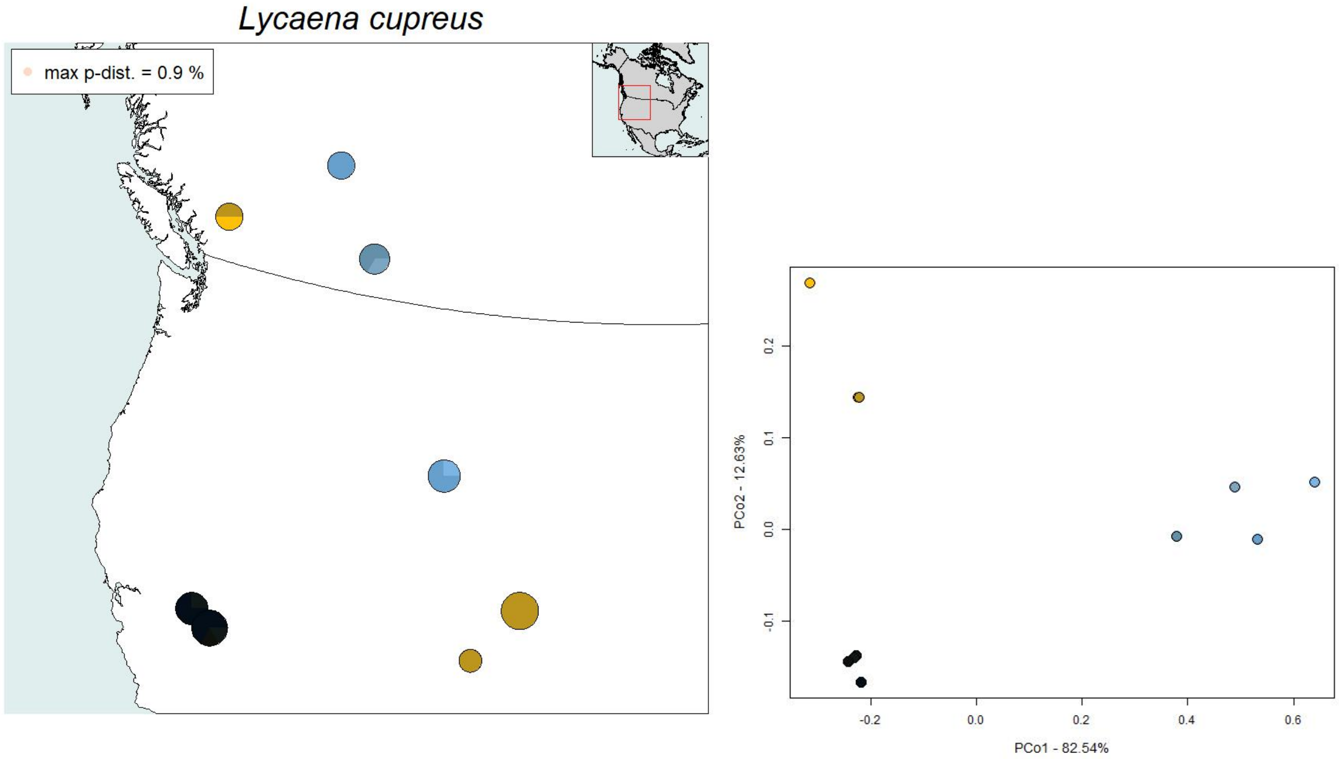

Figure 312 Map of *Lycaena cupreus* showing the localities of the sequenced specimens (left). Nearby localities are grouped in pies. Colours match the bidimensional colour space of the PCoA projection (right) of max p-dists among sequences (dots). Sequences= 29; Hap obs.= 5; Hap asympt.= 5; Hap % obs.= 100%; GST= 1; DST= 0.0038; HD= 0.756; ND= 0.0042; max p-dist= 0.9%.

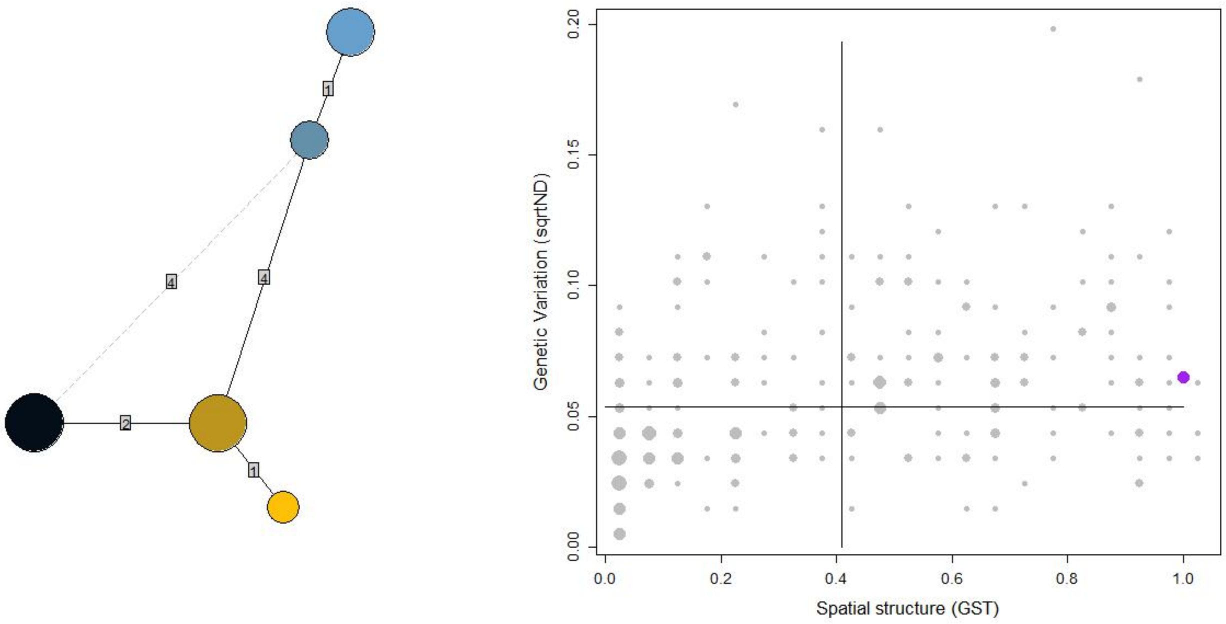

Figure 313: Haplotype network (left) of *Lycaena cupreus* sequences > 599 bp with colours matching the PCoA colour space (above). The bubble plot for mt-DNA polymorphism (square root transformed nucleotide diversity) and spatial structure (GST) among all species in the atlas and values for *Lycaena cupreus* (purple dot). The horizontal and vertical lines represent median values of nucleotide diversity and GST, respectively. Sequences > 599 bp= 27.

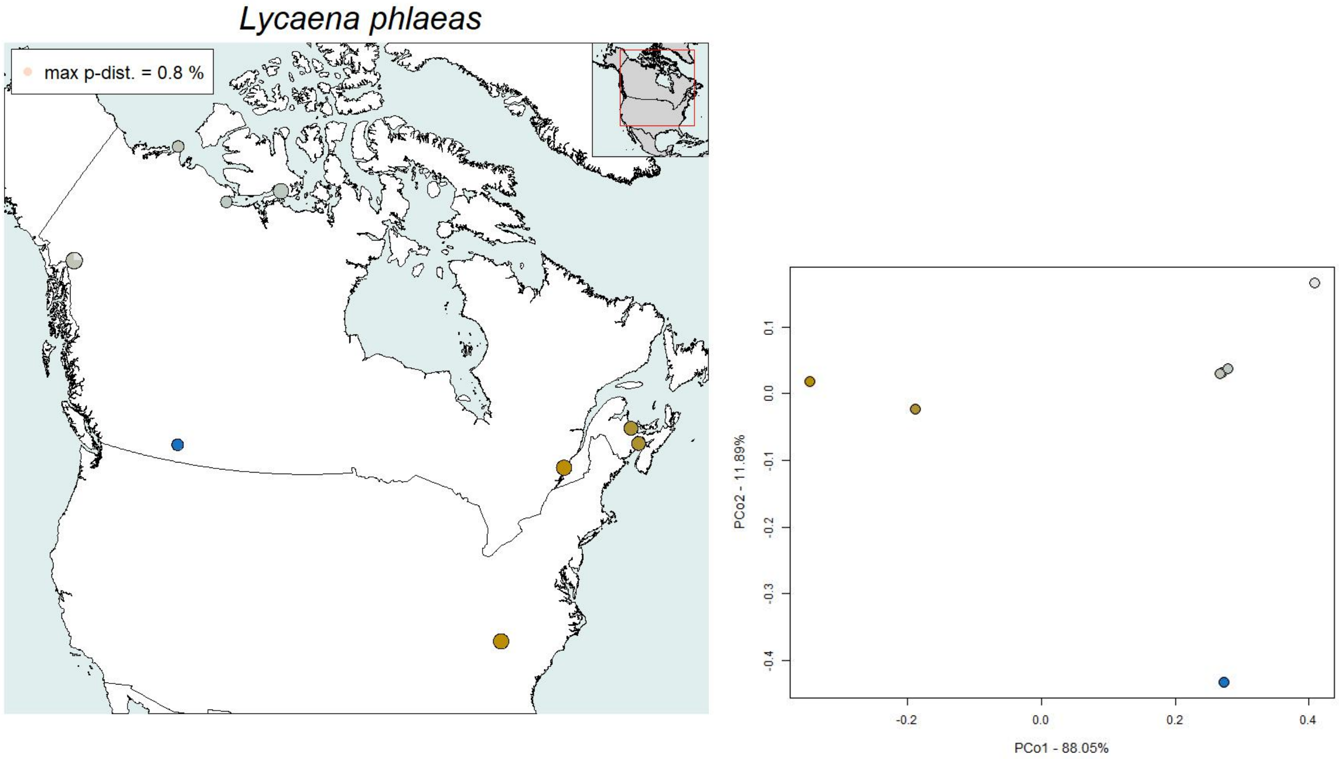

Figure 314 Map of *Lycaena phlaeas* showing the localities of the sequenced specimens (left). Nearby localities are grouped in pies. Colours match the bidimensional colour space of the PCoA projection (right) of max p-dists among sequences (dots). Sequences= 20; Hap obs.= 5; Hap asympt.= 6; Hap % obs.= 84%; GST= 0.893; DST= 0.0029; HD= 0.742; ND= 0.0036; max p-dist= 0.8%.

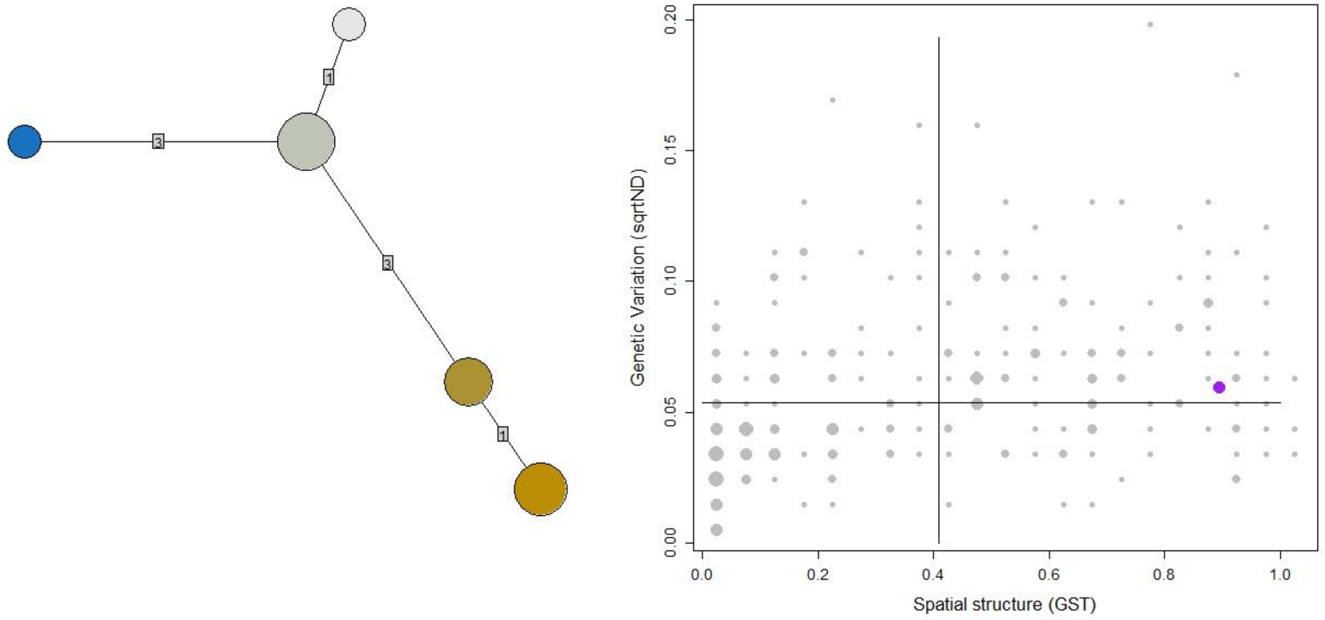

Figure 315: Haplotype network (left) of *Lycaena phlaeas* sequences > 599 bp with colours matching the PCoA colour space (above). The bubble plot for mt-DNA polymorphism (square root transformed nucleotide diversity) and spatial structure (GST) among all species in the atlas and values for *Lycaena phlaeas* (purple dot). The horizontal and vertical lines represent median values of nucleotide diversity and GST, respectively. Sequences > 599 bp= 20.

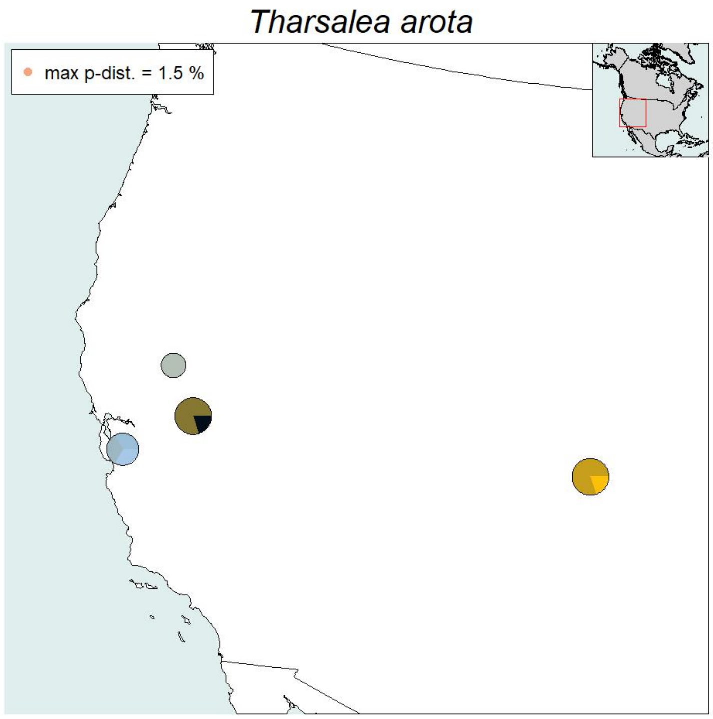

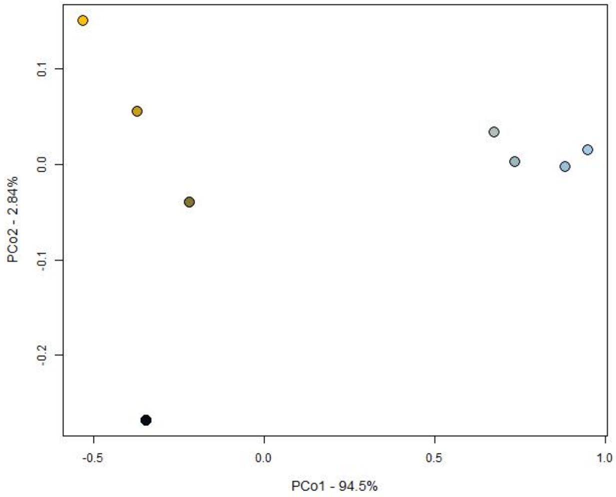

Figure 316 Map of *Tharsalea arota* showing the localities of the sequenced specimens (left). Nearby localities are grouped in pies. Colours match the bidimensional colour space of the PCoA projection (right) of max p-dists among sequences (dots). Sequences= 14; Hap obs.= 8; Hap asympt.= 21.9; Hap % obs.= 36.5%; GST= 0.739; DST= 0.0041; HD= 0.868; ND= 0.0059; max p-dist= 1.5%.

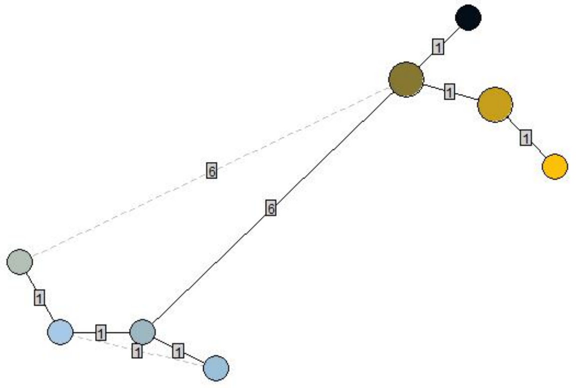

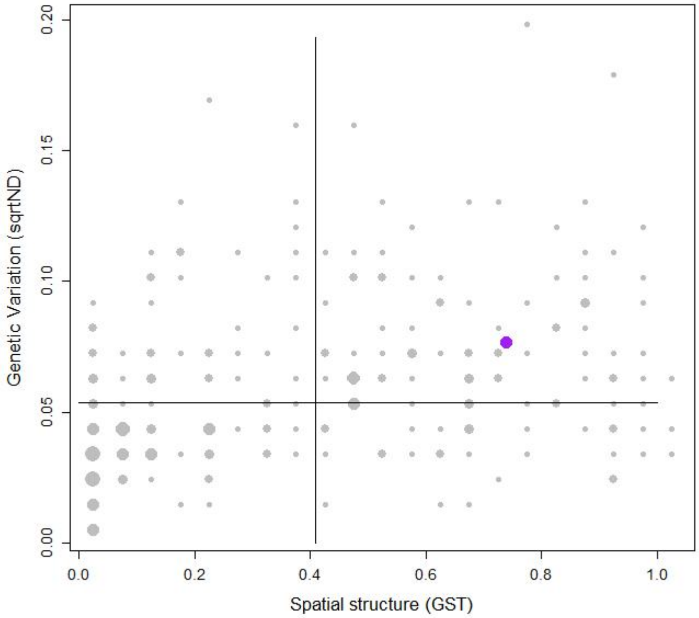

Figure 317: Haplotype network (left) of *Tharsalea arota* sequences > 599 bp with colours matching the PCoA colour space (above). The bubble plot for mt-DNA polymorphism (square root transformed nucleotide diversity) and spatial structure (GST) among all species in the atlas and values for *Tharsalea arota* (purple dot). The horizontal and vertical lines represent median values of nucleotide diversity and GST, respectively. Sequences > 599 bp= 14.

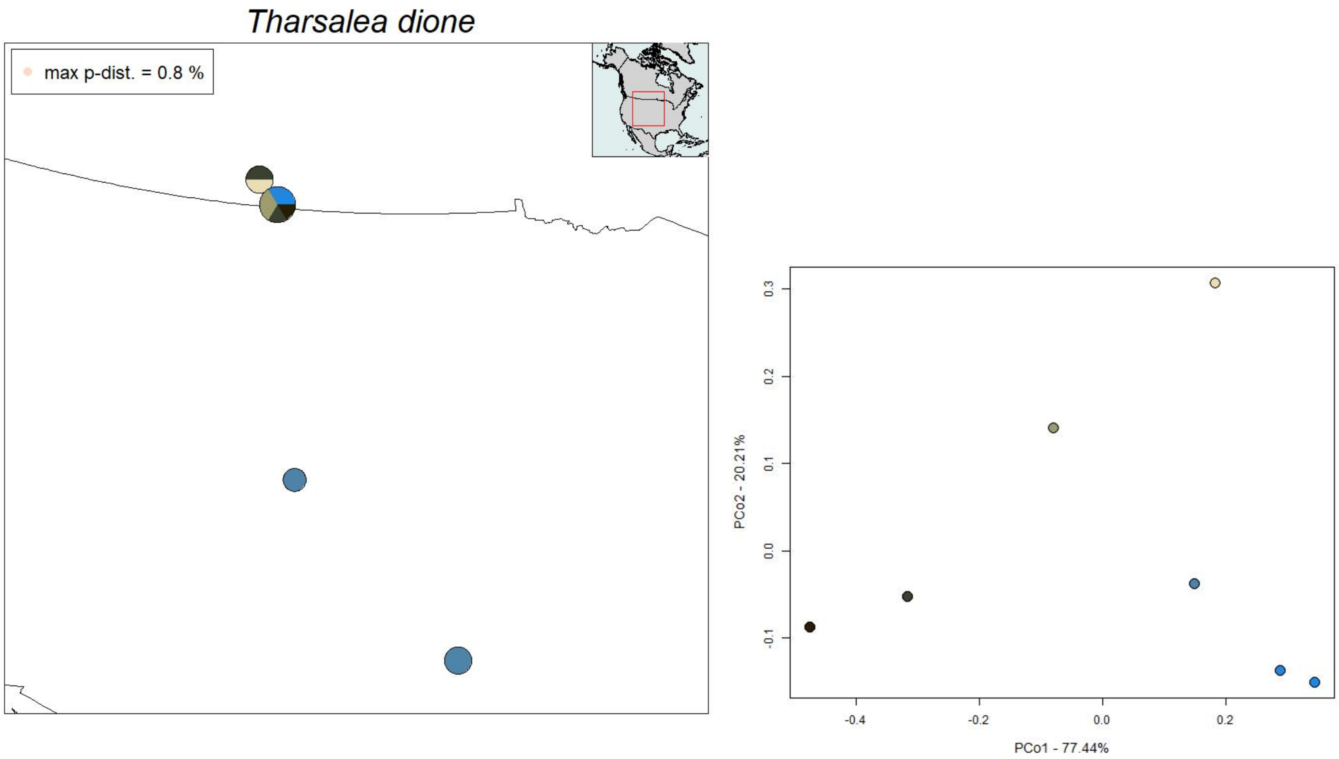

Figure 318 Map of *Tharsalea dione* showing the localities of the sequenced specimens (left). Nearby localities are grouped in pies. Colours match the bidimensional colour space of the PCoA projection (right) of max p-dists among sequences (dots). Sequences= 11; Hap obs.= 6; Hap asympt.= 6.6; Hap % obs.= 90.8%; GST= NaN; DST= NaN; HD= 0.891; ND= 0.0037; max p-dist= 0.8%.

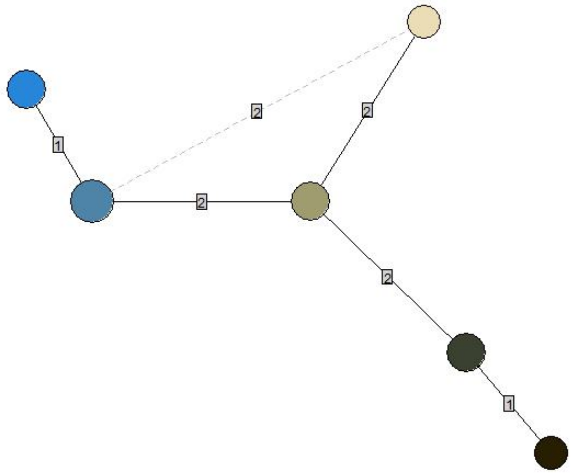

Figure 319: Haplotype network of *Tharsalea dione*. Sequences > 599 bp= 11.

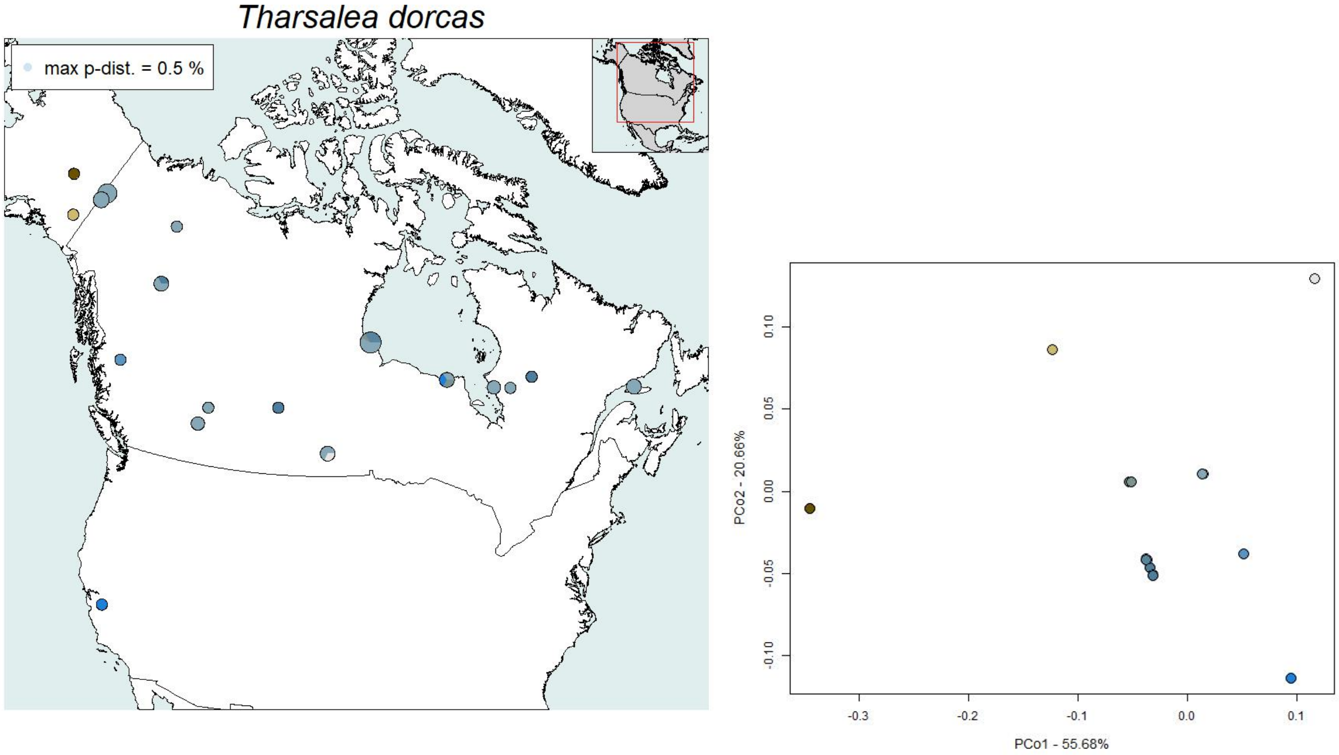

Figure 320 Map of *Tharsalea dorcas* showing the localities of the sequenced specimens (left). Nearby localities are grouped in pies. Colours match the bidimensional colour space of the PCoA projection (right) of max p-dists among sequences (dots). Sequences= 49; Hap obs.= 8; Hap asympt.= 22.7; Hap % obs.= 35.3%; GST= 0.644; DST= 0.0001; HD= 0.446; ND= 0.0003; max p-dist= 0.5%.

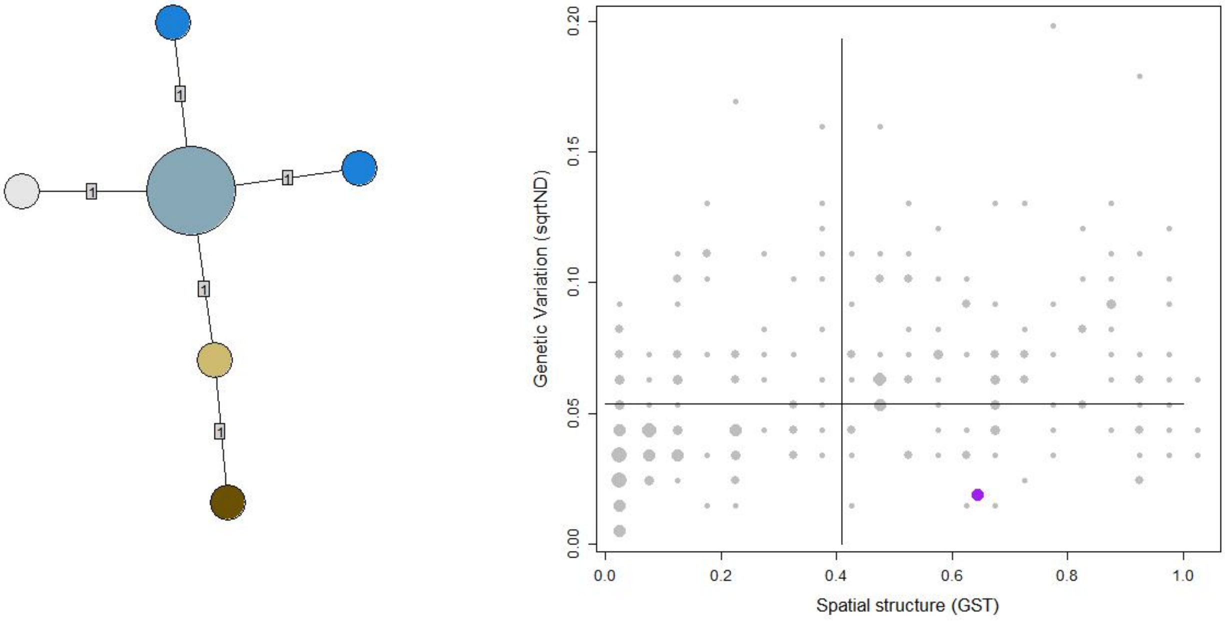

Figure 321: Haplotype network (left) of *Tharsalea dorcas* sequences > 599 bp with colours matching the PCoA colour space (above). The bubble plot for mt-DNA polymorphism (square root transformed nucleotide diversity) and spatial structure (GST) among all species in the atlas and values for *Tharsalea dorcas* (purple dot). The horizontal and vertical lines represent median values of nucleotide diversity and GST, respectively. Sequences > 599 bp= 41.

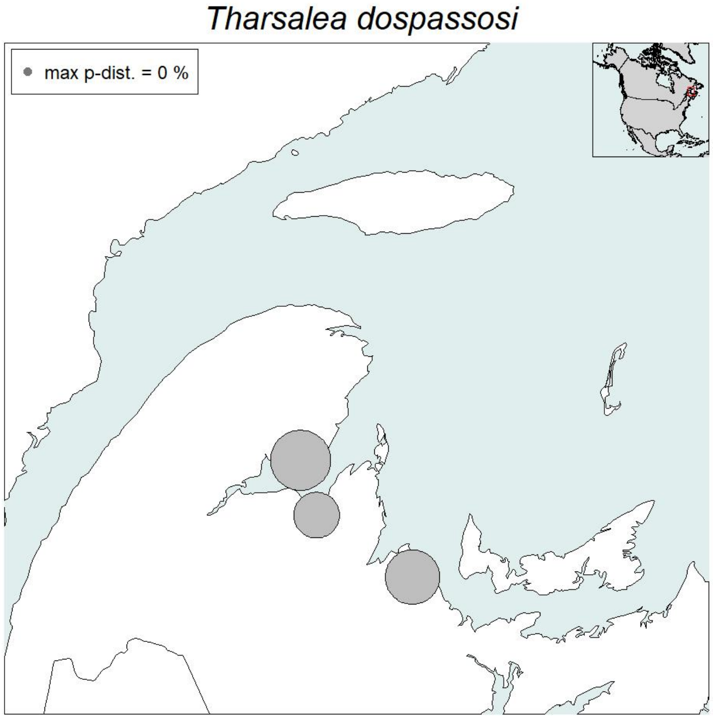

Figure 322: Map of *Tharsalea dospassosi* showing the localities of the sequenced specimens. Nearby localities are grouped in pies. Due to the presence of a single haplotype PCoA projection was not done and a single grey colour was plotted on the map. Sequences= 6; Hap obs.= 1; Hap asympt.= NA; Hap % obs.= NA%; GST= NaN; DST= NaN; HD= NA; ND= NA; max p-dist= 0%.

Haplotype network analysis and bubble plot of *Tharsalea dospassosi* were not possible. Sequences > 599 bp = 6.

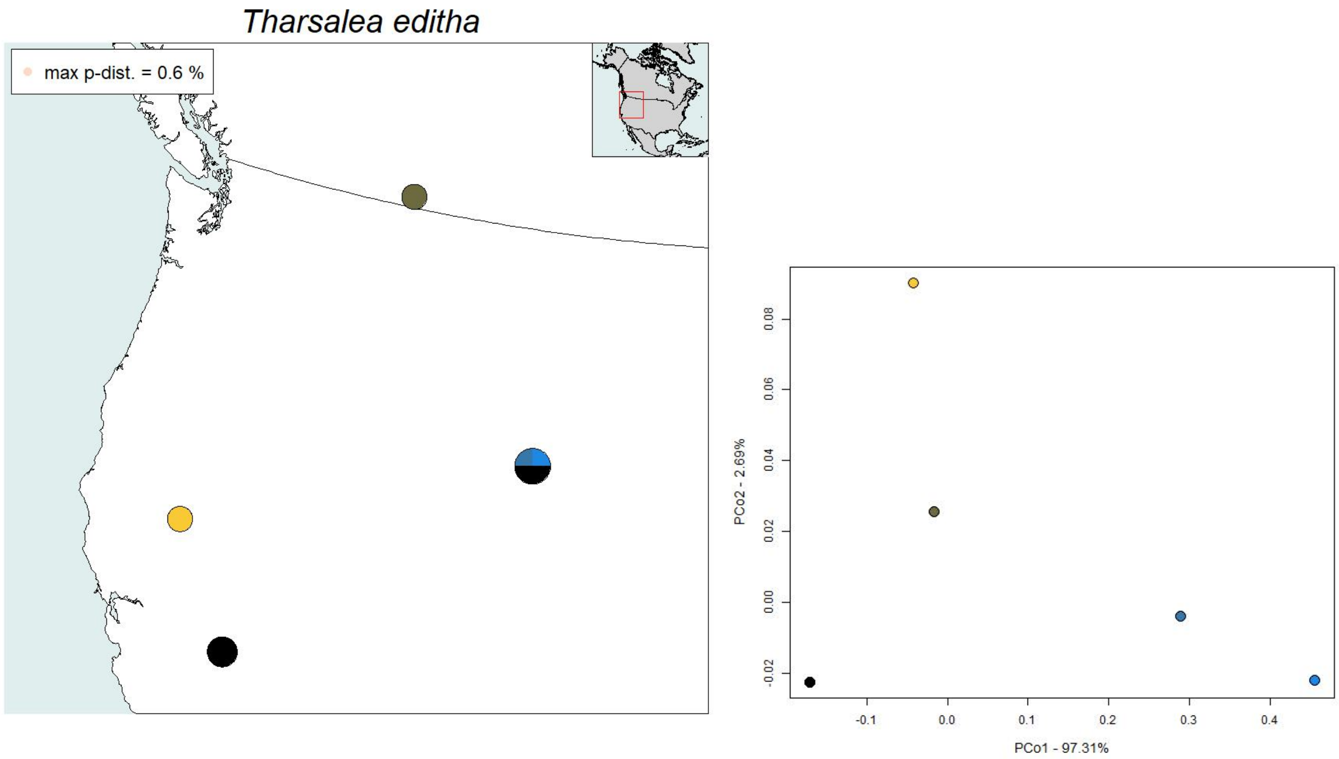

Figure 323 Map of *Tharsalea editha* showing the localities of the sequenced specimens (left). Nearby localities are grouped in pies. Colours match the bidimensional colour space of the PCoA projection (right) of max p-dists among sequences (dots). Sequences= 8; Hap obs.= 4; Hap asympt.= NA; Hap % obs.= NA%; GST= NaN; DST= NaN; HD= NA; ND= NA; max p-dist= 0.6%.

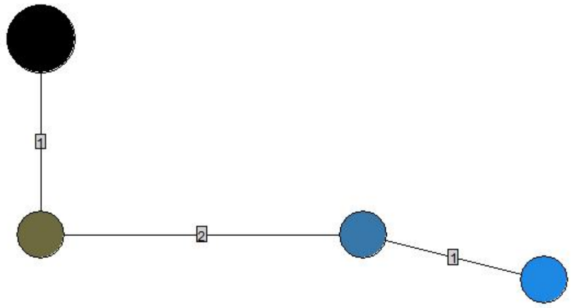

Figure 324: Haplotype network of *Tharsalea editha*. Sequences > 599 bp= 7.

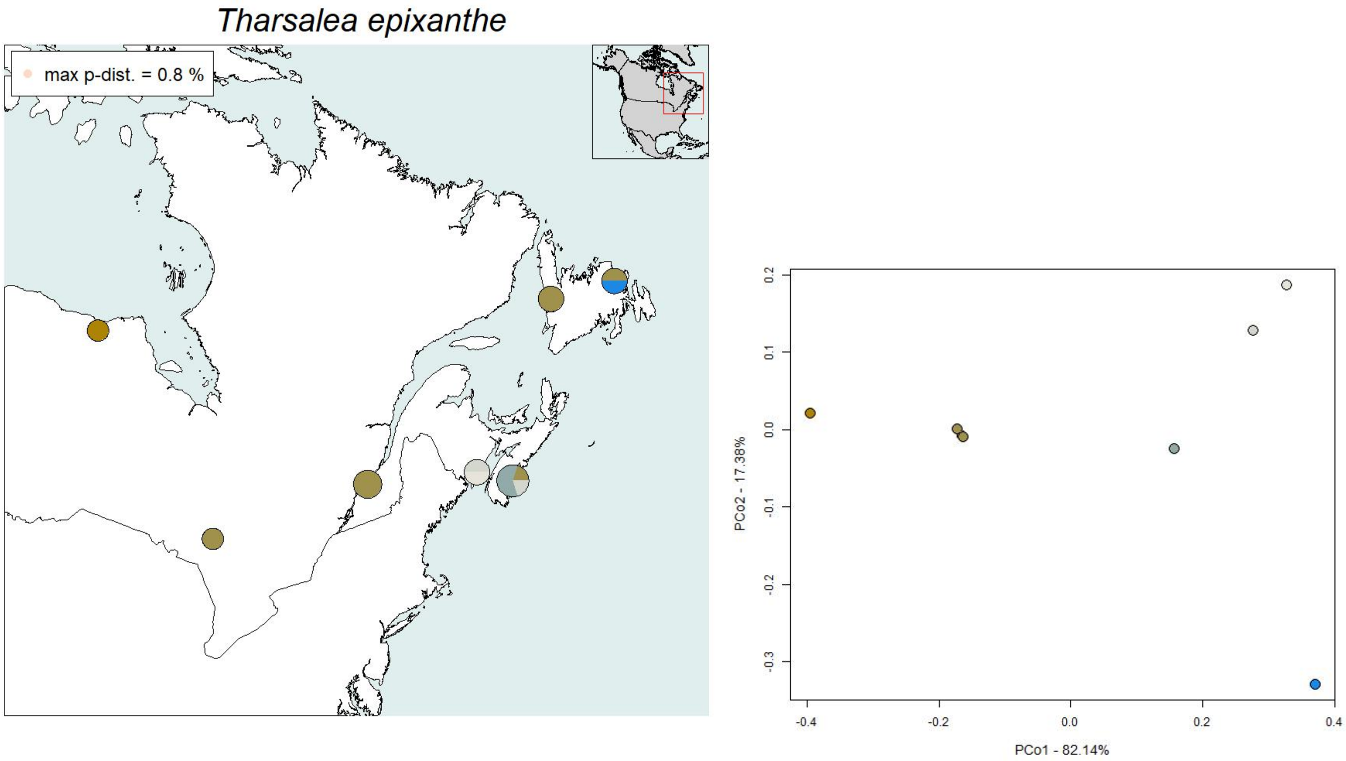

Figure 325 Map of *Tharsalea epixanthe* showing the localities of the sequenced specimens (left). Nearby localities are grouped in pies. Colours match the bidimensional colour space of the PCoA projection (right) of max p-dists among sequences (dots). Sequences= 16; Hap obs.= 5; Hap asympt.= 5.9; Hap % obs.= 84.2%; GST= 0.683; DST= 0.0016; HD= 0.717; ND= 0.0028; max p-dist= 0.8%.

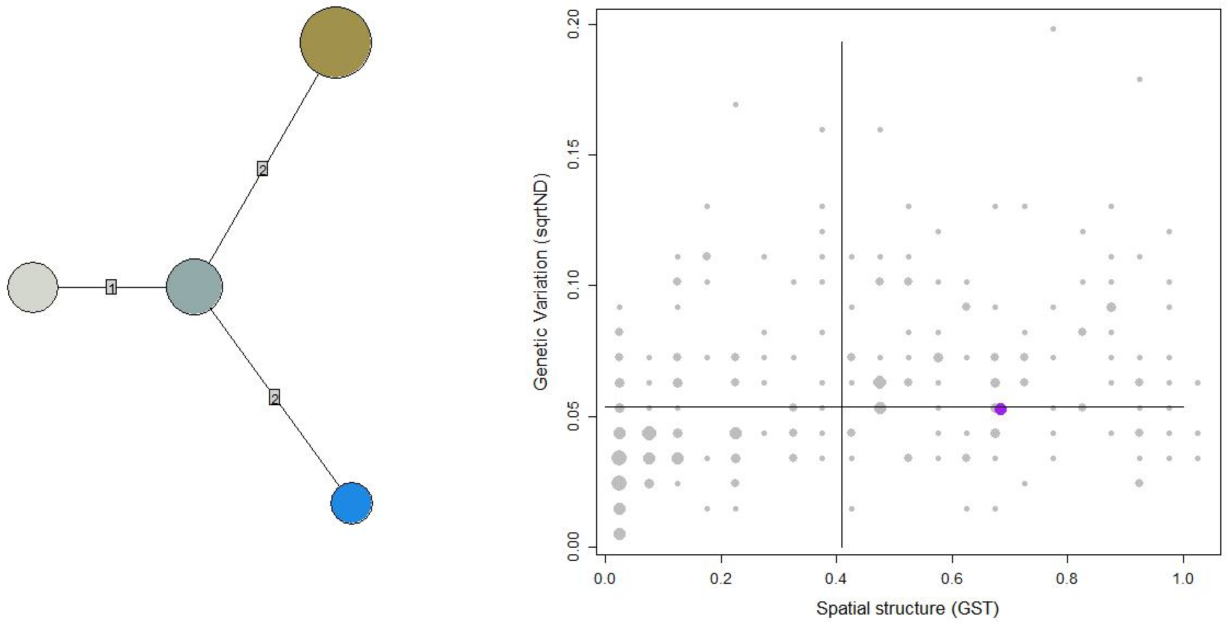

Figure 326: Haplotype network (left) of *Tharsalea epixanthe* sequences > 599 bp with colours matching the PCoA colour space (above). The bubble plot for mt-DNA polymorphism (square root transformed nucleotide diversity) and spatial structure (GST) among all species in the atlas and values for *Tharsalea epixanthe* (purple dot). The horizontal and vertical lines represent median values of nucleotide diversity and GST, respectively. Sequences > 599 bp= 14.

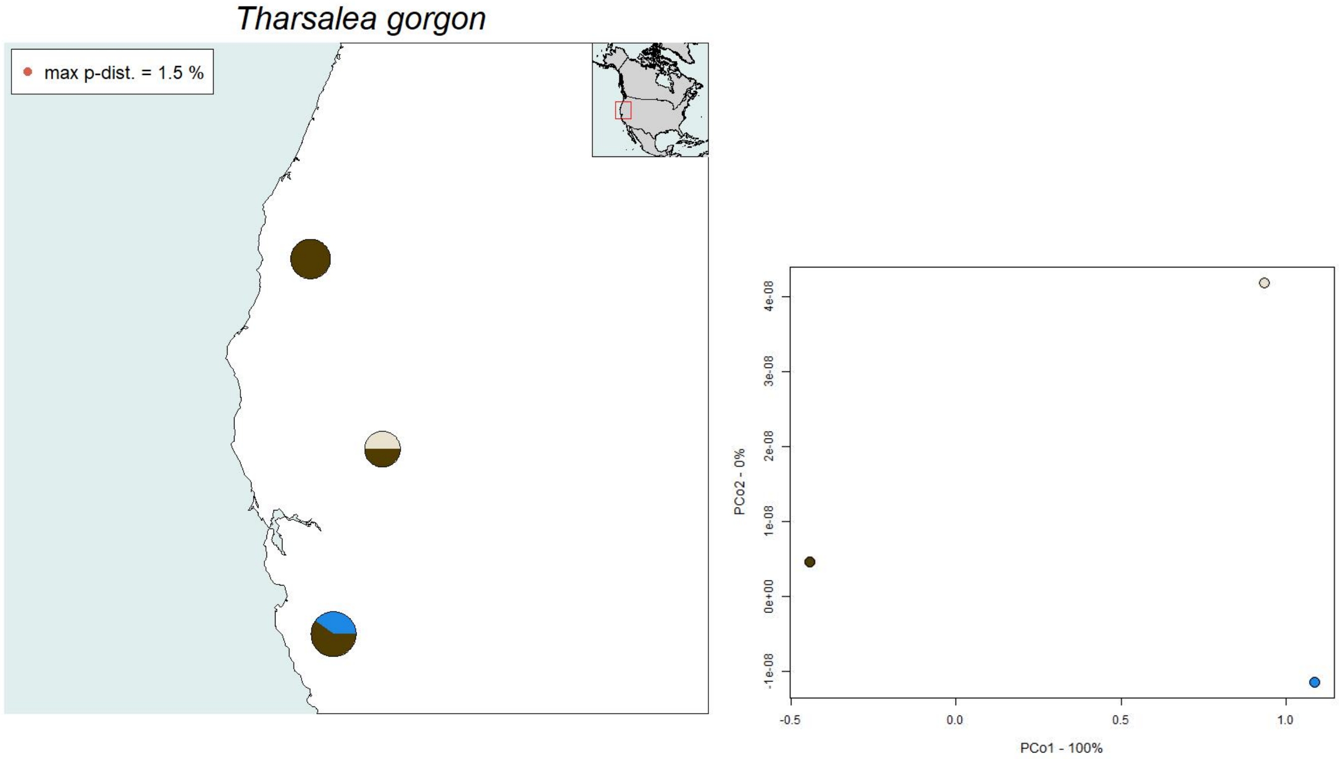

Figure 327 Map of *Tharsalea gorgon* showing the localities of the sequenced specimens (left). Nearby localities are grouped in pies. Colours match the bidimensional colour space of the PCoA projection (right) of max p-dists among sequences (dots). Sequences= 10; Hap obs.= 3; Hap asympt.= 3.5; Hap % obs.= 87%; GST= NaN; DST= NaN; HD= 0.511; ND= 0.007; max p-dist= 1.5%.

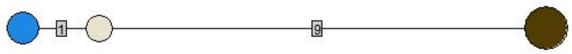

Figure 328: Haplotype network of *Tharsalea gorgon*. Sequences > 599 bp= 10.

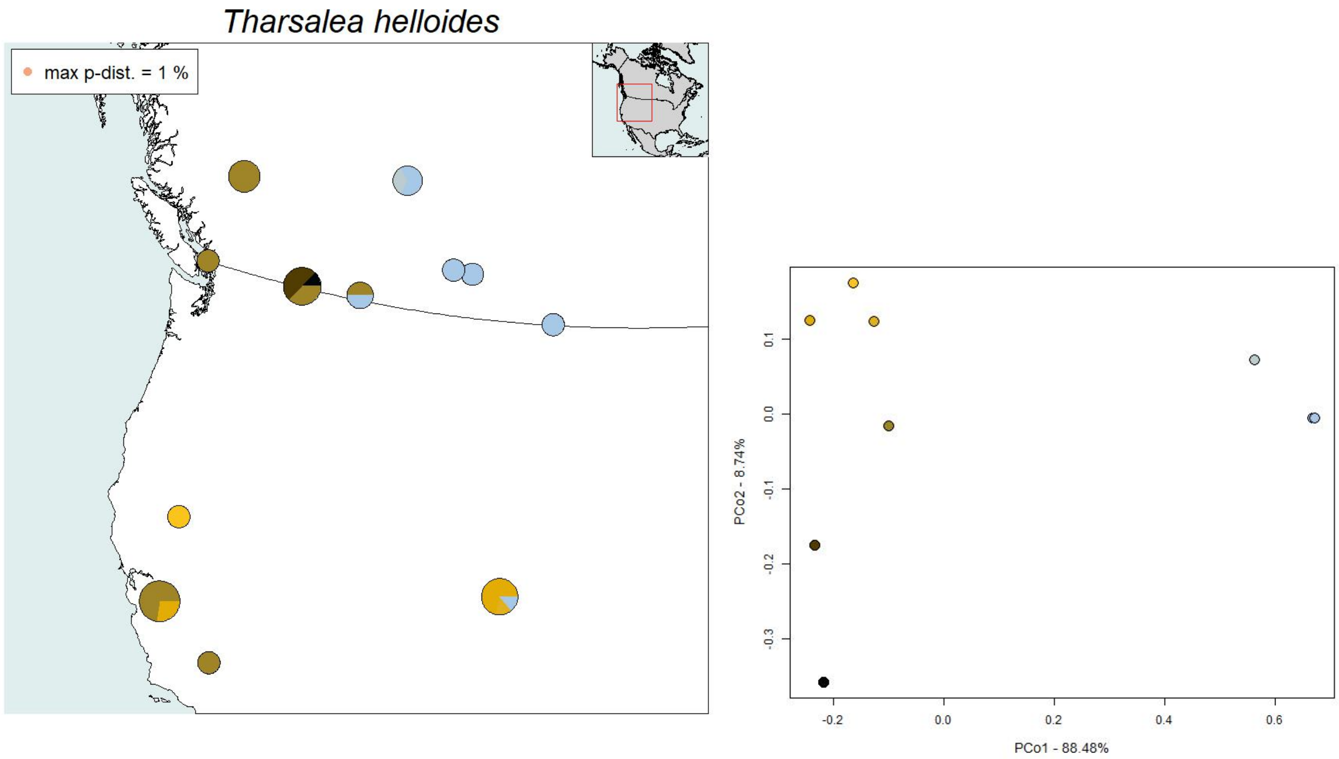

Figure 329 Map of *Tharsalea helloides* showing the localities of the sequenced specimens (left). Nearby localities are grouped in pies. Colours match the bidimensional colour space of the PCoA projection (right) of max p-dists among sequences (dots). Sequences= 41; Hap obs.= 5; Hap asympt.= 5; Hap % obs.= 100%; GST= 0.665; DST= 0.0017; HD= 0.717; ND= 0.0034; max p-dist= 1%.

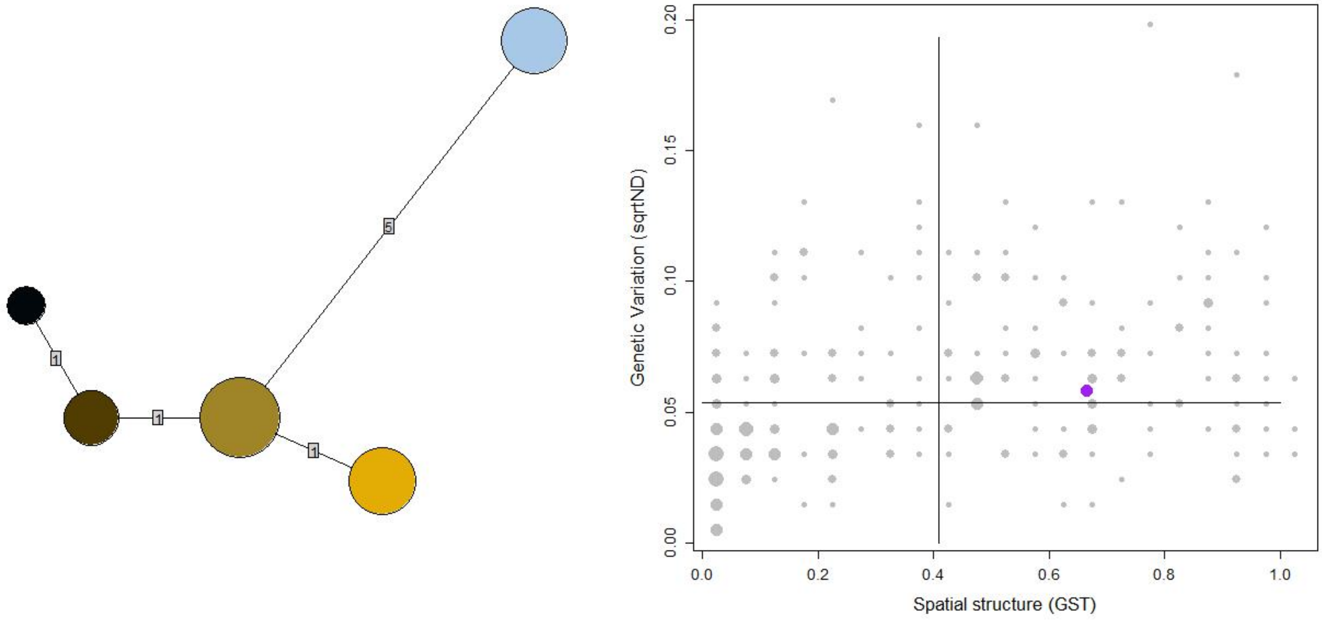

Figure 330: Haplotype network (left) of *Tharsalea helloides* sequences > 599 bp with colours matching the PCoA colour space (above). The bubble plot for mt-DNA polymorphism (square root transformed nucleotide diversity) and spatial structure (GST) among all species in the atlas and values for *Tharsalea helloides* (purple dot). The horizontal and vertical lines represent median values of nucleotide diversity and GST, respectively. Sequences > 599 bp= 40.

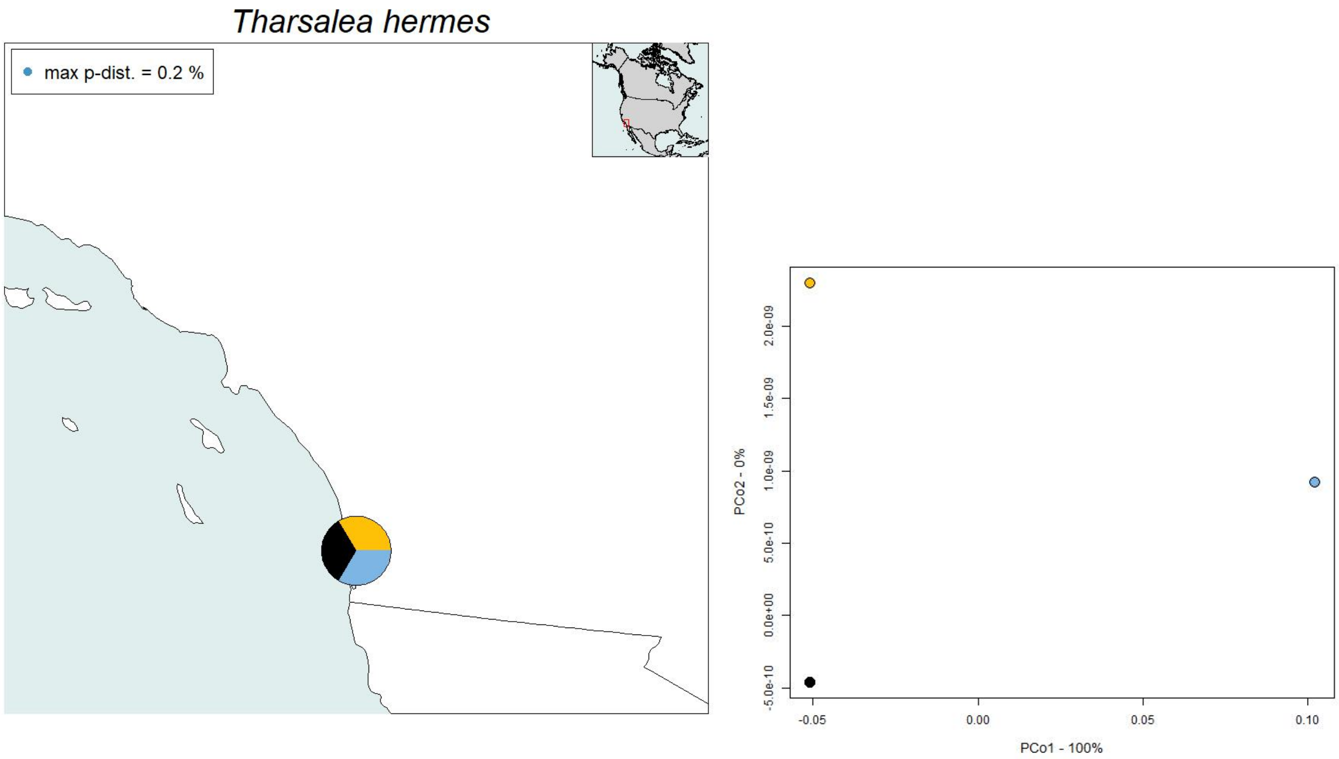

Figure 331 Map of *Tharsalea hermes* showing the localities of the sequenced specimens (left). Nearby localities are grouped in pies. Colours match the bidimensional colour space of the PCoA projection (right) of max p-dists among sequences (dots). Sequences= 3; Hap obs.= 2; Hap asympt.= NA; Hap % obs.= NA%; GST= NaN; DST= NaN; HD= NA; ND= NA; max p-dist= 0.2%.

Haplotype network analysis and bubble plot of *Tharsalea hermes* were not possible. Sequences > 599 bp = 3.

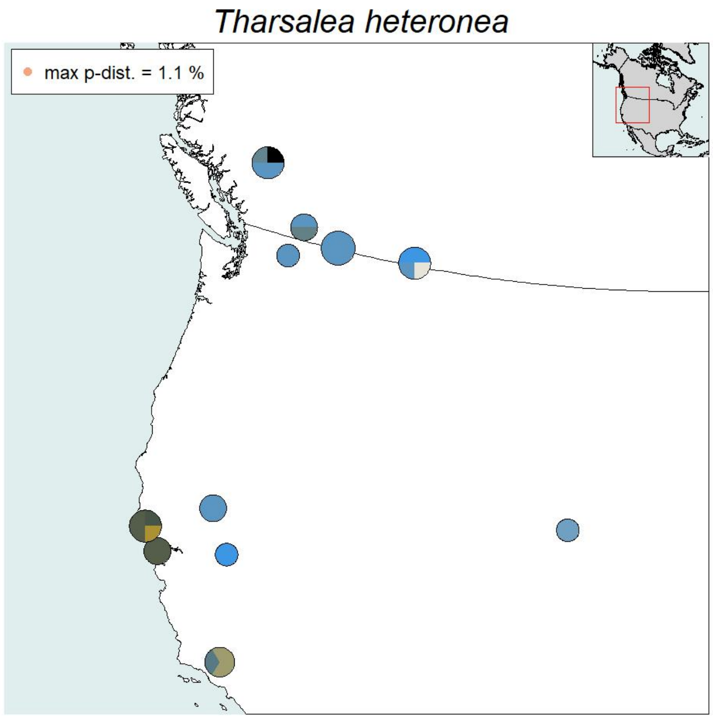

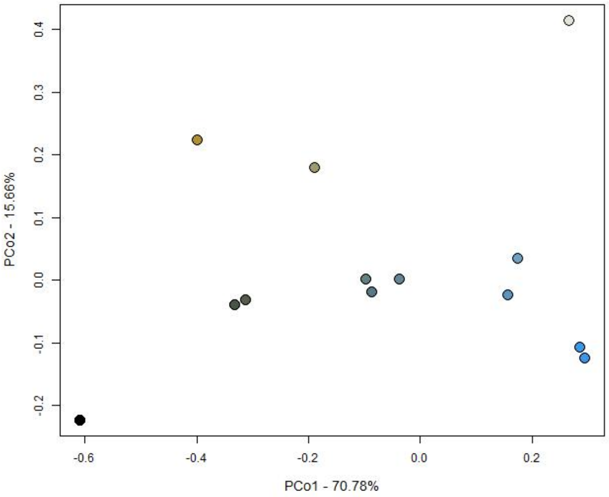

Figure 332 Map of *Tharsalea heteronea* showing the localities of the sequenced specimens (left). Nearby localities are grouped in pies. Colours match the bidimensional colour space of the PCoA projection (right) of max p-dists among sequences (dots). Sequences= 29; Hap obs.= 11; Hap asympt.= 22.8; Hap % obs.= 48.2%; GST= 0.59; DST= 0.0018; HD= 0.778; ND= 0.0029; max p-dist= 1.1%.

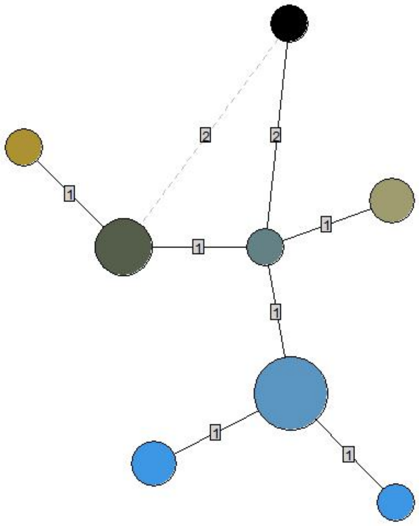

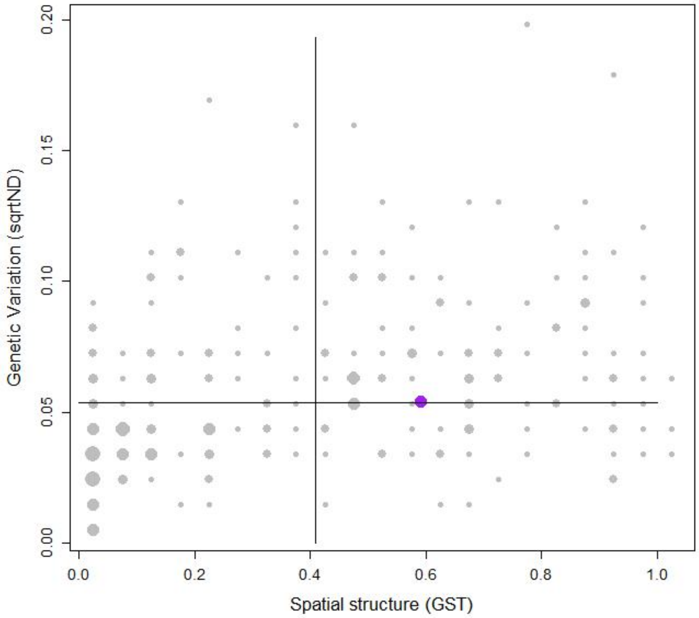

Figure 333: Haplotype network (left) of *Tharsalea heteronea* sequences > 599 bp with colours matching the PCoA colour space (above). The bubble plot for mt-DNA polymorphism (square root transformed nucleotide diversity) and spatial structure (GST) among all species in the atlas and values for *Tharsalea heteronea* (purple dot). The horizontal and vertical lines represent median values of nucleotide diversity and GST, respectively. Sequences > 599 bp= 27.

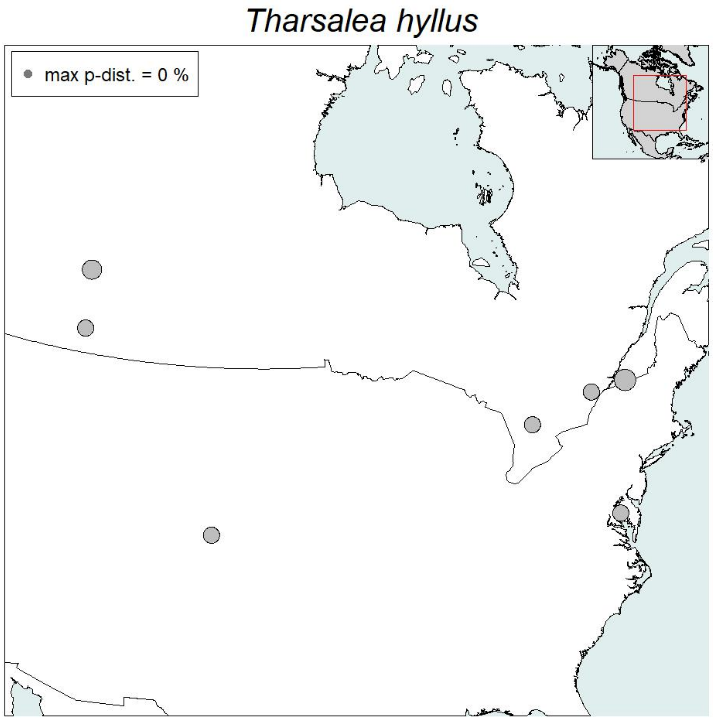

Figure 334: Map of *Tharsalea hyllus* showing the localities of the sequenced specimens. Nearby localities are grouped in pies. Due to the presence of a single haplotype PCoA projection was not done and a single grey colour was plotted on the map. Sequences= 10; Hap obs.= 1; Hap asympt.= NA; Hap % obs.= NA%; GST= NaN; DST= NaN; HD= 0; ND= 0; max p-dist= 0%.

Haplotype network analysis and bubble plot of *Tharsalea hyllus* were not possible. Sequences > 599 bp = 10.

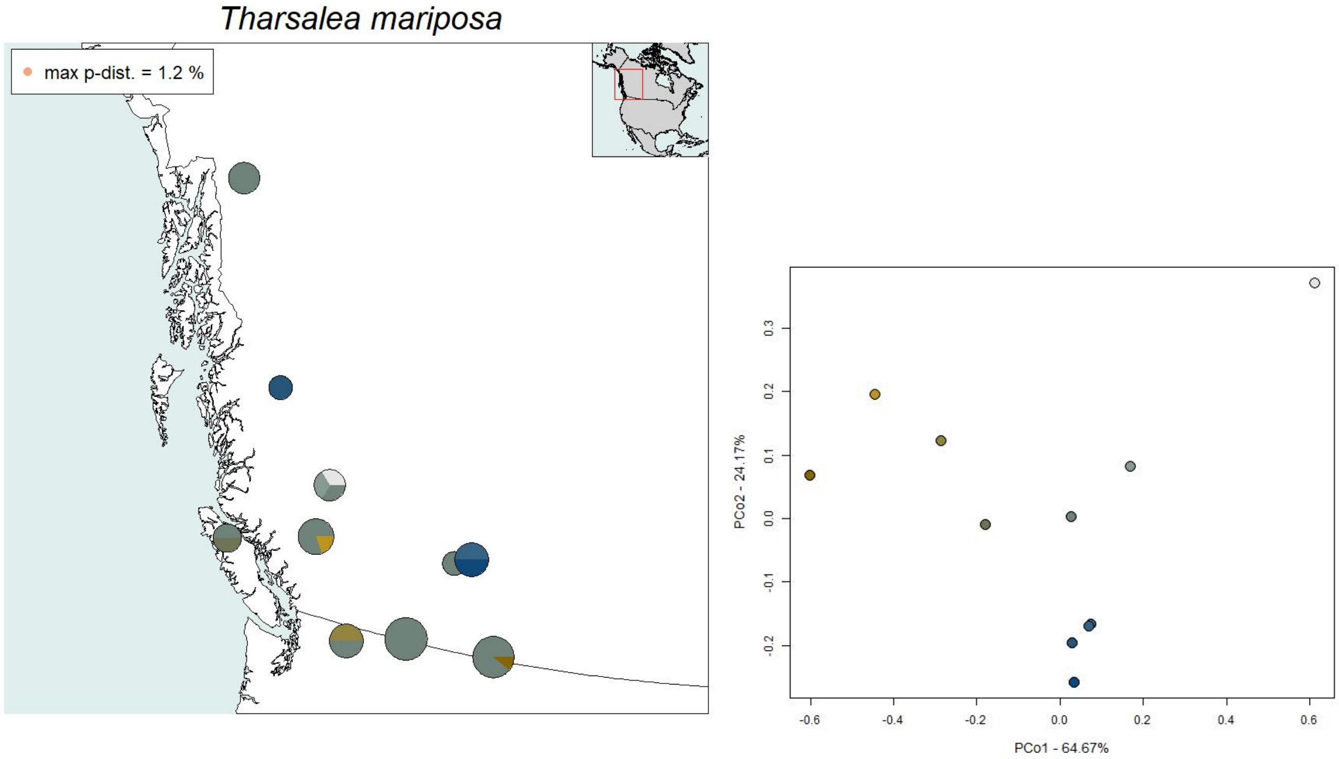

Figure 335 Map of *Tharsalea mariposa* showing the localities of the sequenced specimens (left). Nearby localities are grouped in pies. Colours match the bidimensional colour space of the PCoA projection (right) of max p-dists among sequences (dots). Sequences= 42; Hap obs.= 10; Hap asympt.= 33.9; Hap % obs.= 29.5%; GST= 0.332; DST= 0.0005; HD= 0.49; ND= 0.0015; max p-dist= 1.2%.

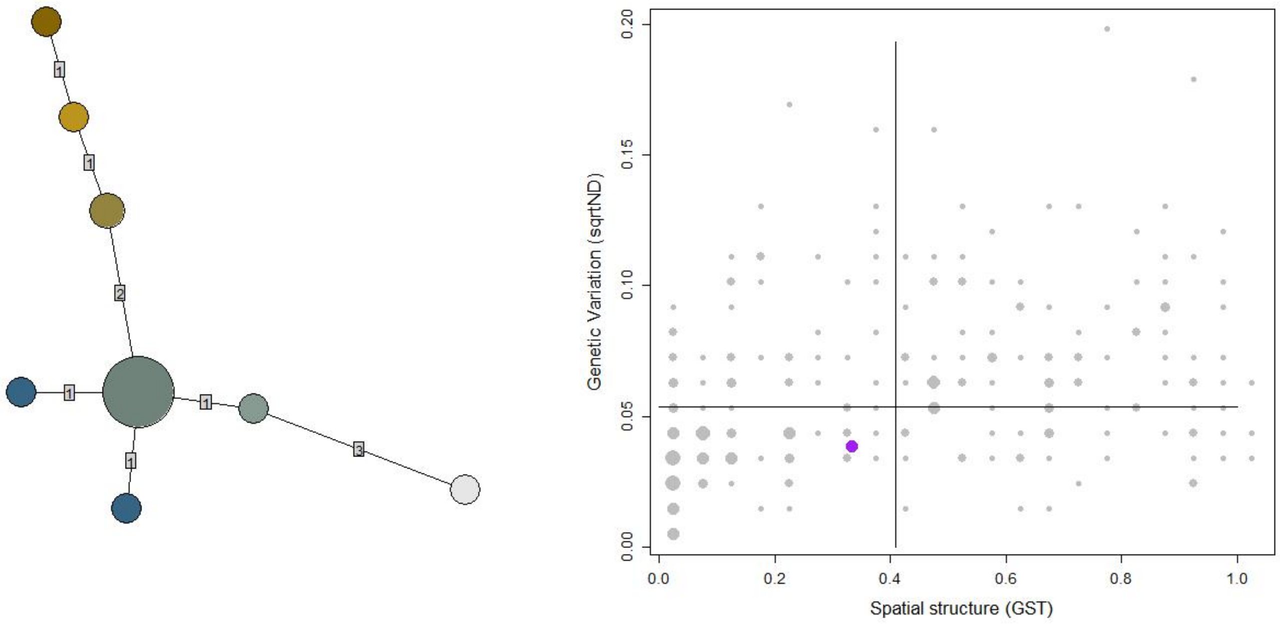

Figure 336: Haplotype network (left) of *Tharsalea mariposa* sequences > 599 bp with colours matching the PCoA colour space (above). The bubble plot for mt-DNA polymorphism (square root transformed nucleotide diversity) and spatial structure (GST) among all species in the atlas and values for *Tharsalea mariposa* (purple dot). The horizontal and vertical lines represent median values of nucleotide diversity and GST, respectively. Sequences > 599 bp= 38.

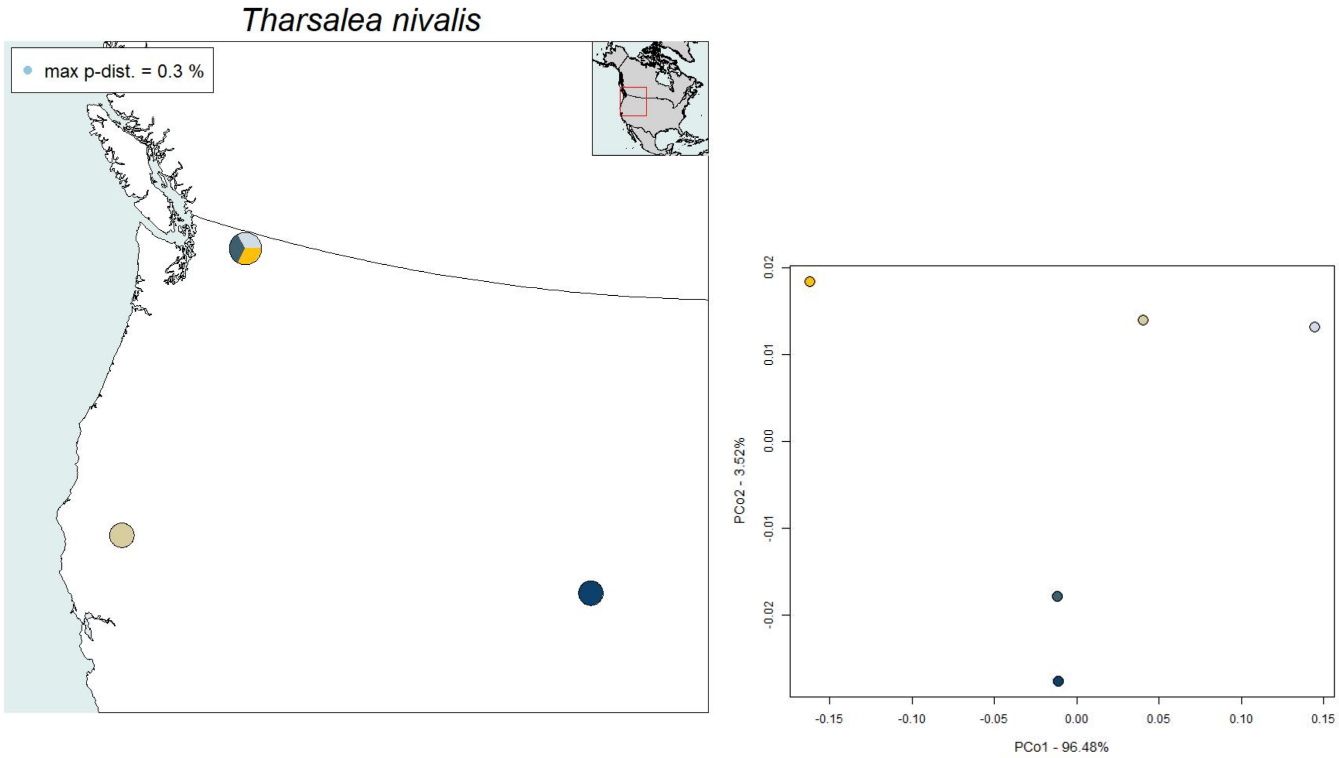

Figure 337 Map of *Tharsalea nivalis* showing the localities of the sequenced specimens (left). Nearby localities are grouped in pies. Colours match the bidimensional colour space of the PCoA projection (right) of max p-dists among sequences (dots). Sequences= 5; Hap obs.= 4; Hap asympt.= NA; Hap % obs.= NA%; GST= NaN; DST= NaN; HD= NA; ND= NA; max p-dist= 0.3%.

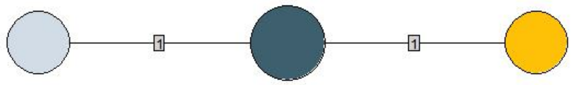

Figure 338: Haplotype network of *Tharsalea nivalis*. Sequences > 599 bp= 4.

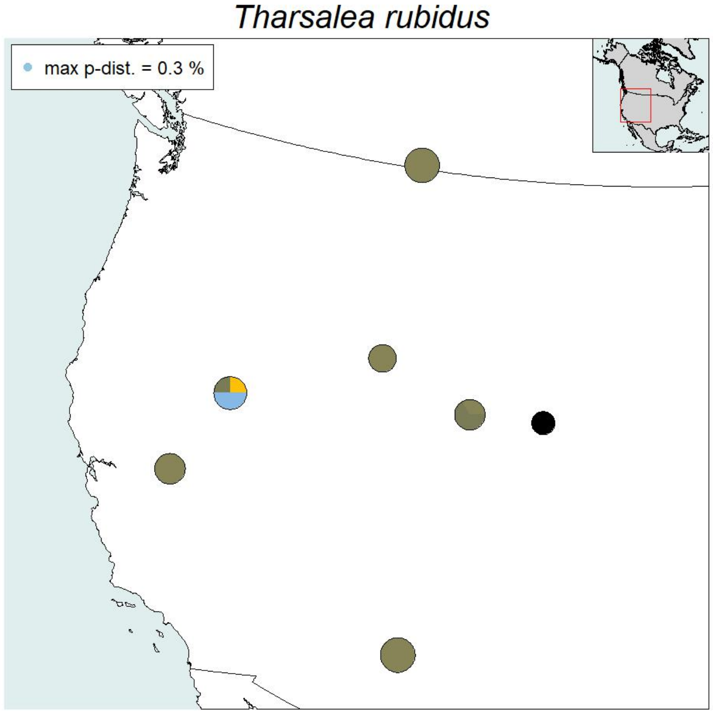

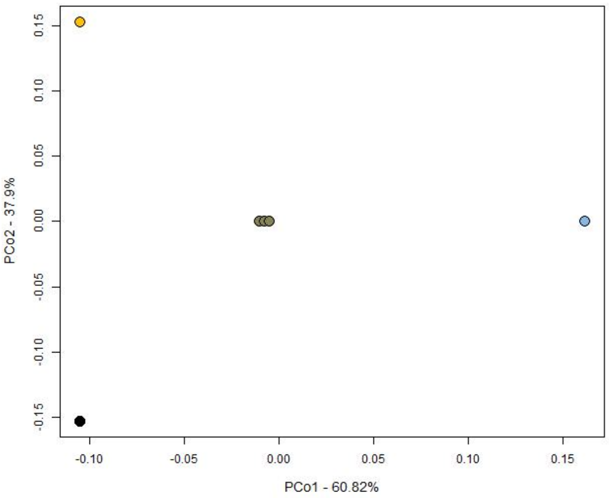

Figure 339 Map of *Tharsalea rubidus* showing the localities of the sequenced specimens (left). Nearby localities are grouped in pies. Colours match the bidimensional colour space of the PCoA projection (right) of max p-dists among sequences (dots). Sequences= 23; Hap obs.= 4; Hap asympt.= 5.9; Hap % obs.= 67.6%; GST= 0.221; DST= 0.0001; HD= 0.32; ND= 0.0005; max p-dist= 0.3%.

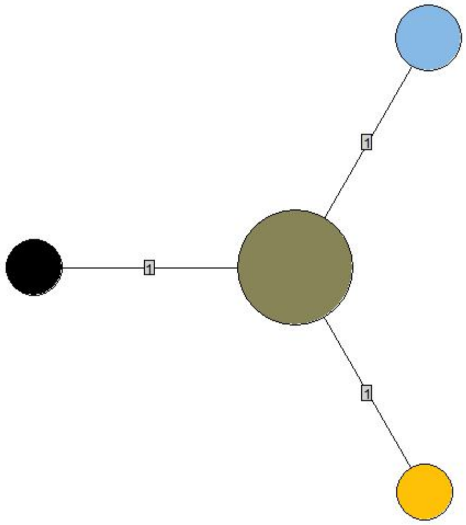

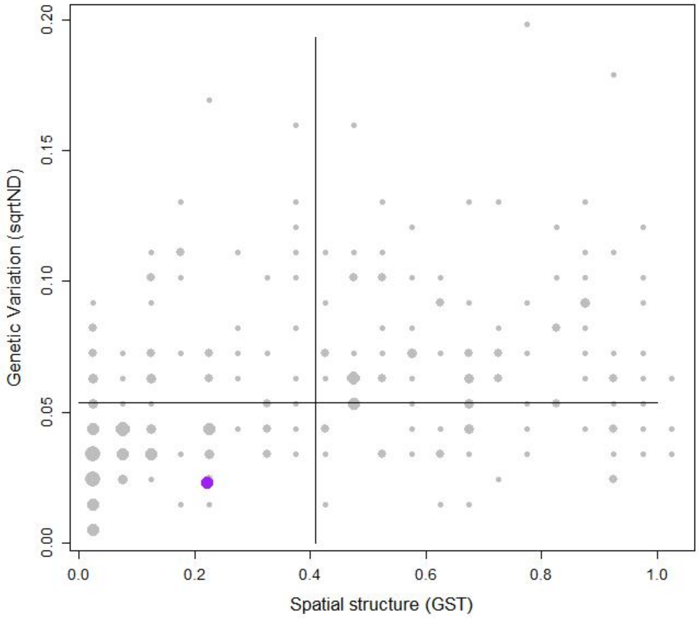

Figure 340: Haplotype network (left) of *Tharsalea rubidus* sequences > 599 bp with colours matching the PCoA colour space (above). The bubble plot for mt-DNA polymorphism (square root transformed nucleotide diversity) and spatial structure (GST) among all species in the atlas and values for *Tharsalea rubidus* (purple dot). The horizontal and vertical lines represent median values of nucleotide diversity and GST, respectively. Sequences > 599 bp= 22.

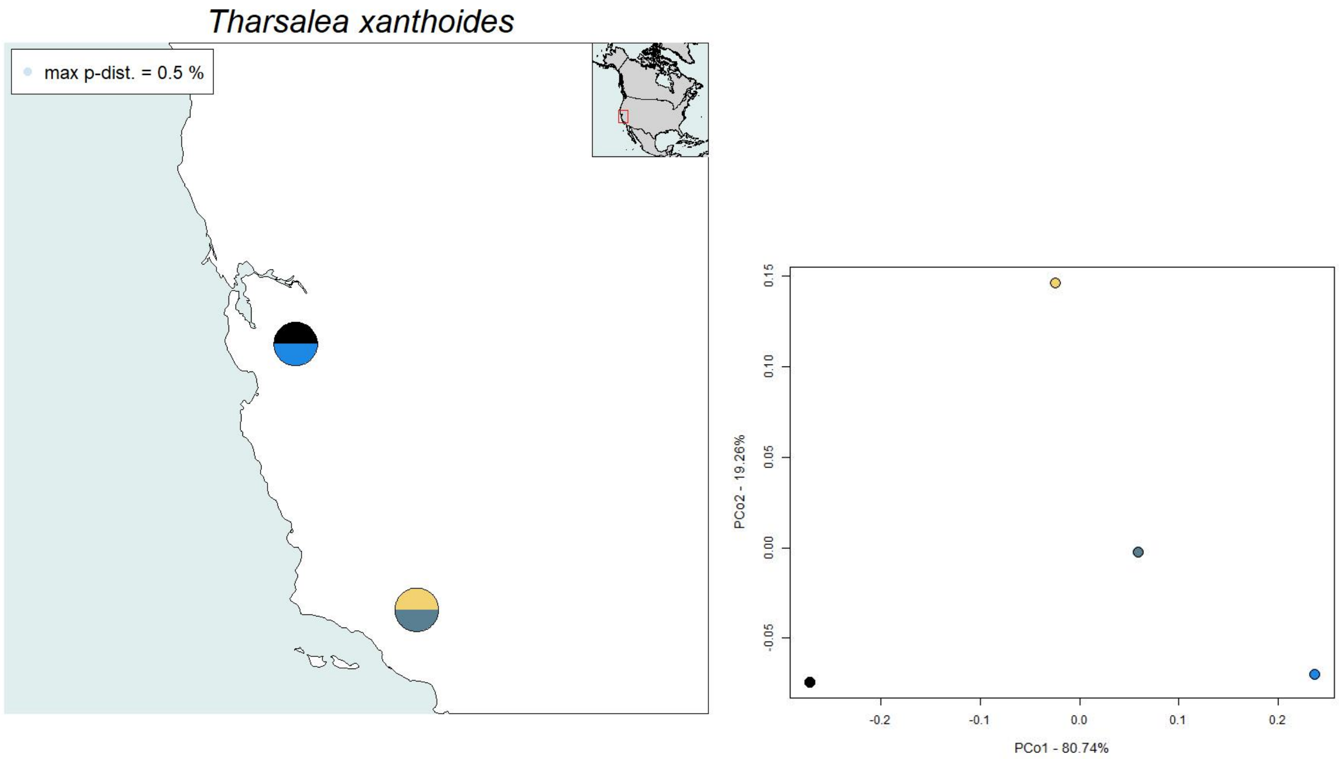

Figure 341 Map of *Tharsalea xanthoides* showing the localities of the sequenced specimens (left). Nearby localities are grouped in pies. Colours match the bidimensional colour space of the PCoA projection (right) of max p-dists among sequences (dots). Sequences= 4; Hap obs.= 4; Hap asympt.= NA; Hap % obs.= NA%; GST= NaN; DST= NaN; HD= NA; ND= NA; max p-dist= 0.5%.

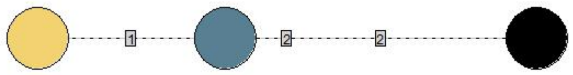

Figure 342: Haplotype network of *Tharsalea xanthoides*. Sequences > 599 bp= 3.

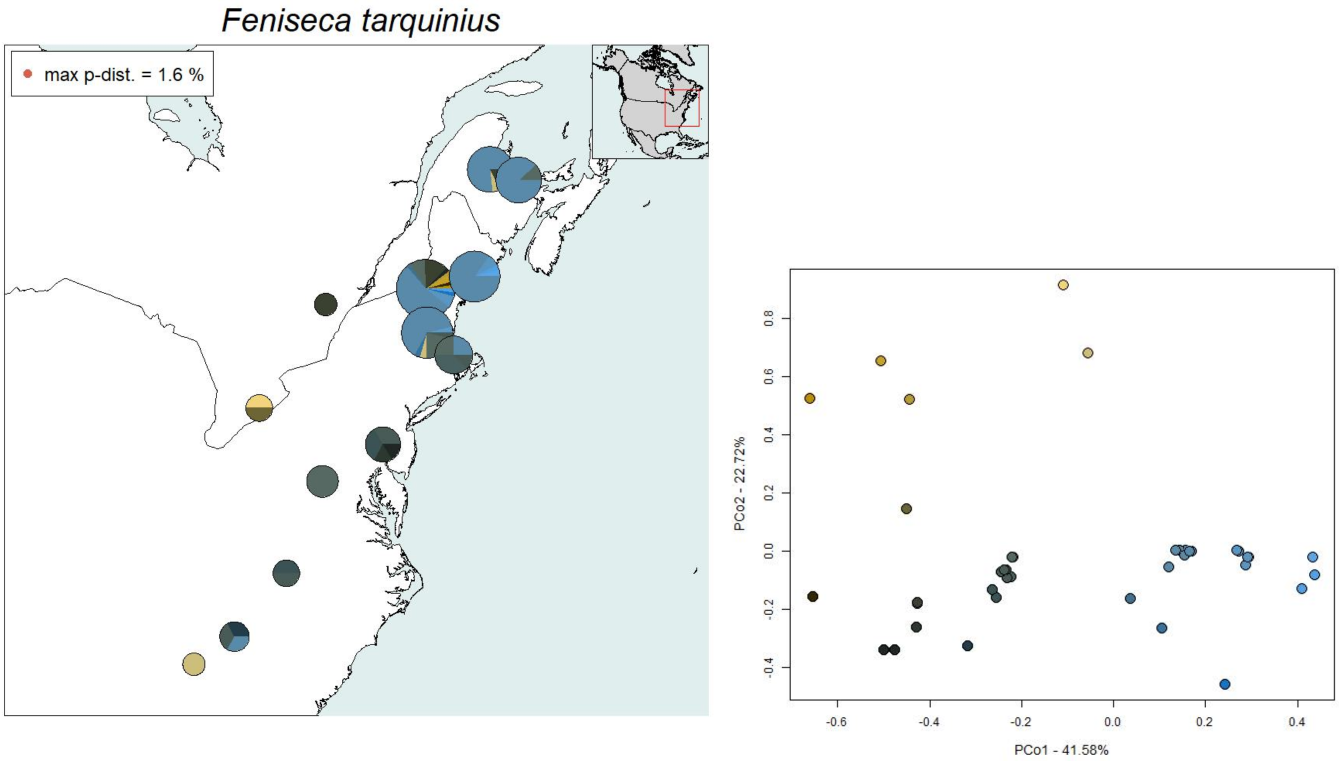

Figure 343 Map of *Feniseca tarquinius* showing the localities of the sequenced specimens (left). Nearby localities are grouped in pies. Colours match the bidimensional colour space of the PCoA projection (right) of max p-dists among sequences (dots). Sequences= 161; Hap obs.= 36; Hap asympt.= 126.6; Hap % obs.= 28.4%; GST= 0.117; DST= 0.0004; HD= 0.659; ND= 0.0033; max p-dist= 1.6%.

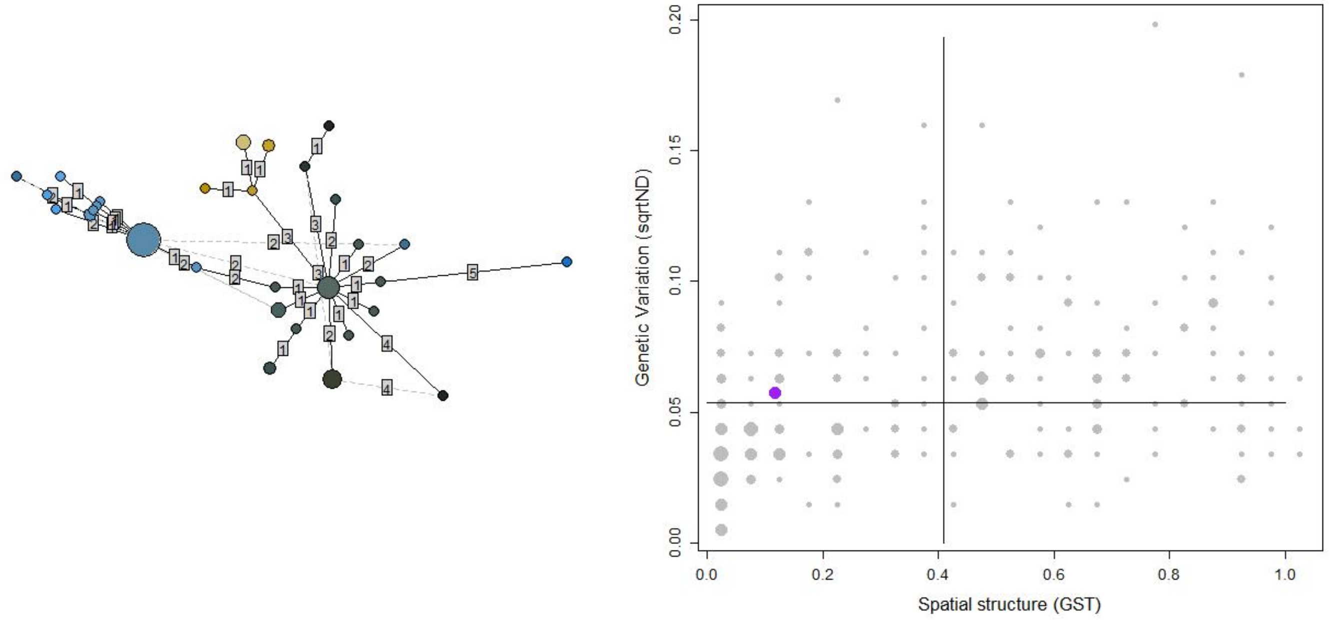

Figure 344: Haplotype network (left) of *Feniseca tarquinius* sequences > 599 bp with colours matching the PCoA colour space (above). The bubble plot for mt-DNA polymorphism (square root transformed nucleotide diversity) and spatial structure (GST) among all species in the atlas and values for *Feniseca tarquinius* (purple dot). The horizontal and vertical lines represent median values of nucleotide diversity and GST, respectively. Sequences > 599 bp= 157.

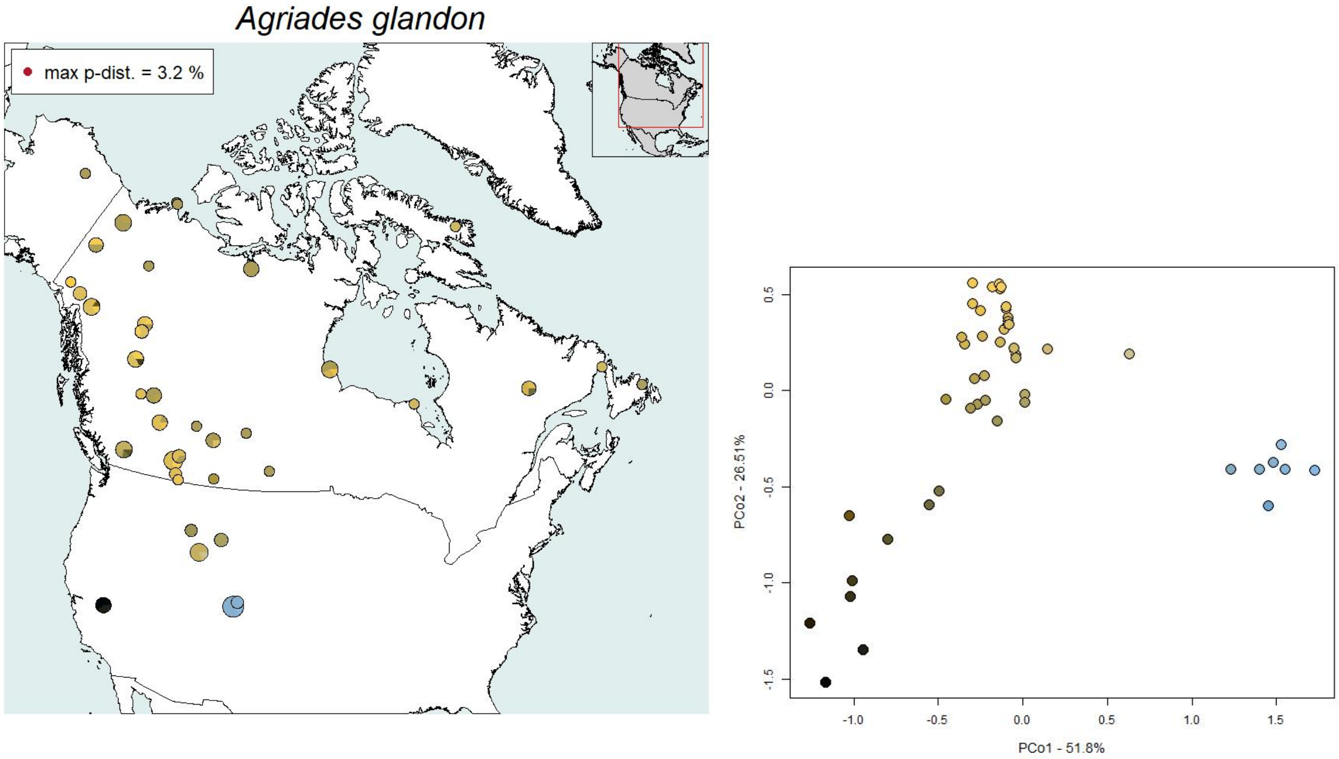

Figure 345 Map of *Agriades glandon* showing the localities of the sequenced specimens (left). Nearby localities are grouped in pies. Colours match the bidimensional colour space of the PCoA projection (right) of max p-dists among sequences (dots). Sequences= 143; Hap obs.= 37; Hap asympt.= 110; Hap % obs.= 33.6%; GST= 0.675; DST= 0.0064; HD= 0.914; ND= 0.009; max p-dist= 3.2%.

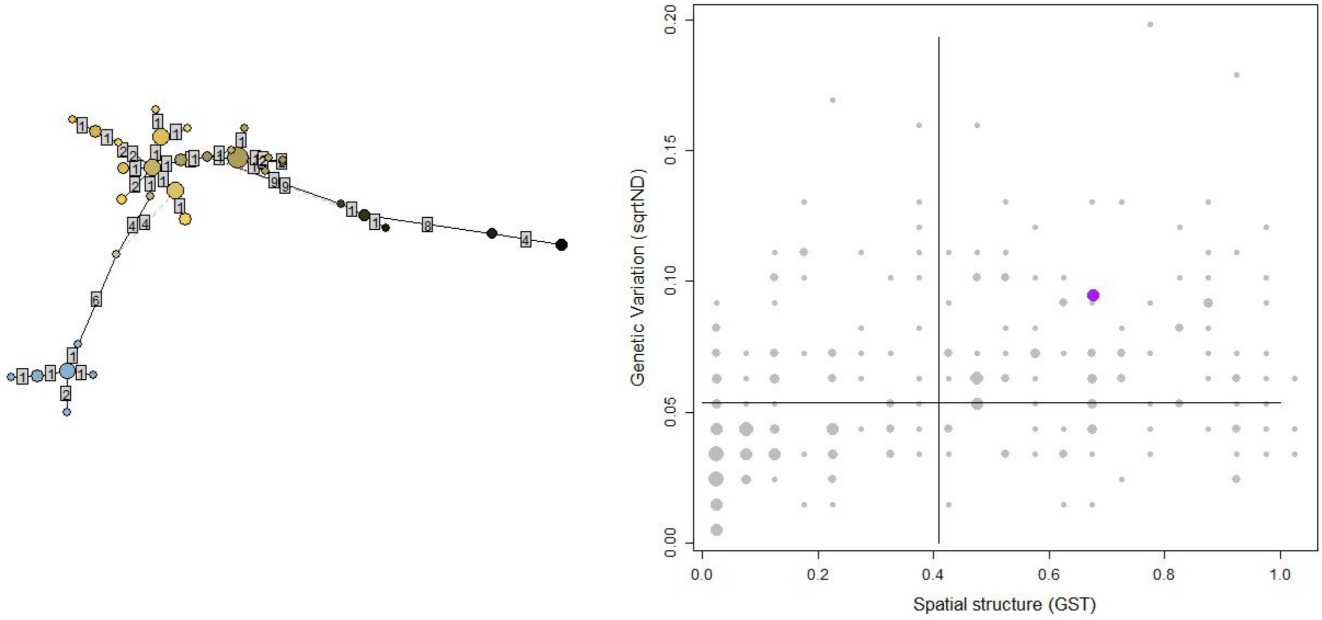

Figure 346: Haplotype network (left) of *Agriades glandon* sequences > 599 bp with colours matching the PCoA colour space (above). The bubble plot for mt-DNA polymorphism (square root transformed nucleotide diversity) and spatial structure (GST) among all species in the atlas and values for *Agriades glandon* (purple dot). The horizontal and vertical lines represent median values of nucleotide diversity and GST, respectively. Sequences > 599 bp= 135.

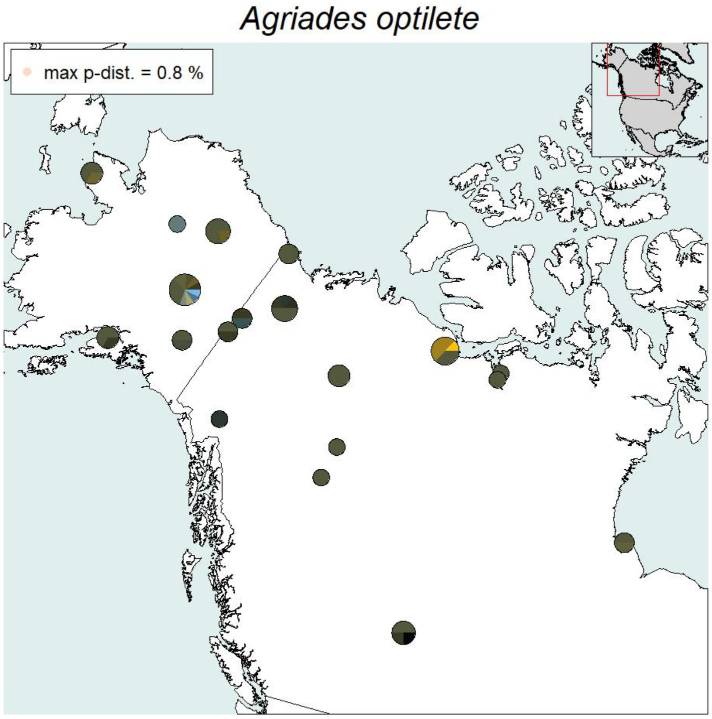

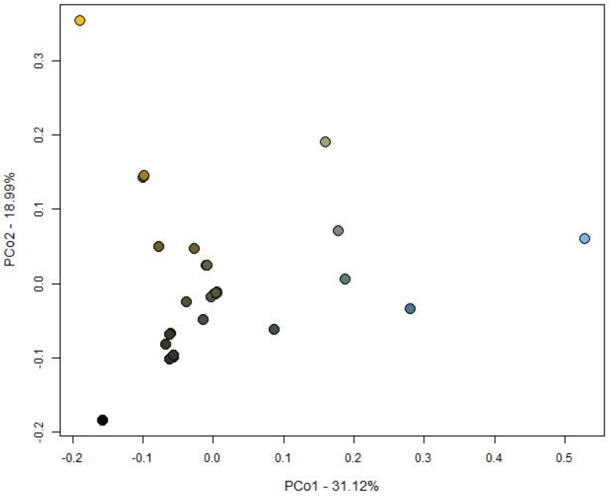

Figure 347 Map of *Agriades optilete* showing the localities of the sequenced specimens (left). Nearby localities are grouped in pies. Colours match the bidimensional colour space of the PCoA projection (right) of max p-dists among sequences (dots). Sequences= 61; Hap obs.= 18; Hap asympt.= 59.6; Hap % obs.= 30.2%; GST= 0.022; DST= 0; HD= 0.665; ND= 0.0013; max p-dist= 0.8%.

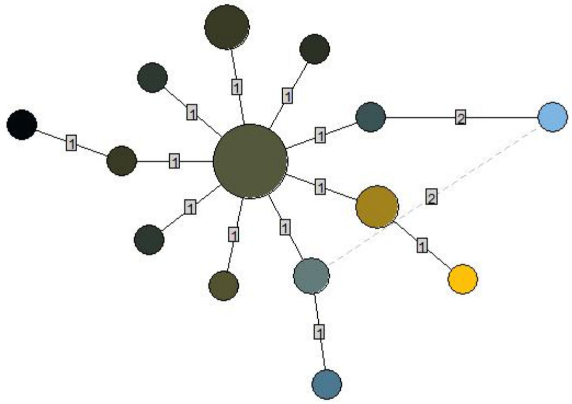

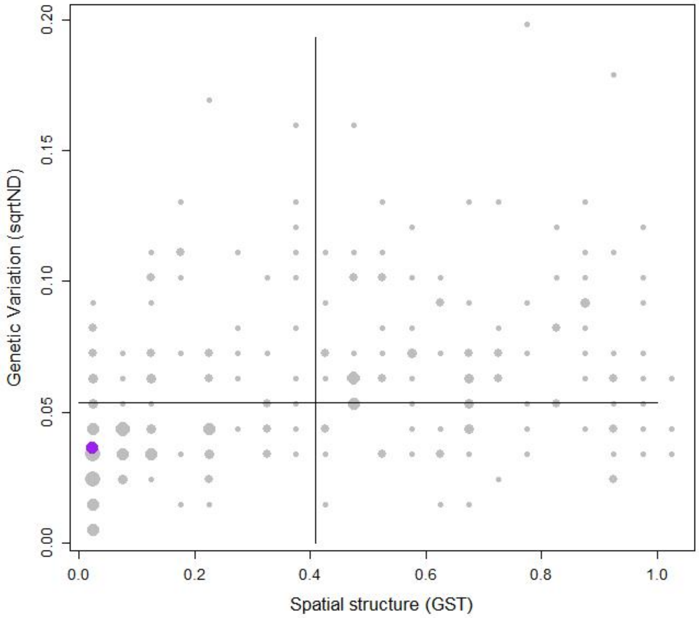

Figure 348: Haplotype network (left) of *Agriades optilete* sequences > 599 bp with colours matching the PCoA colour space (above). The bubble plot for mt-DNA polymorphism (square root transformed nucleotide diversity) and spatial structure (GST) among all species in the atlas and values for *Agriades optilete* (purple dot). The horizontal and vertical lines represent median values of nucleotide diversity and GST, respectively. Sequences > 599 bp= 56.

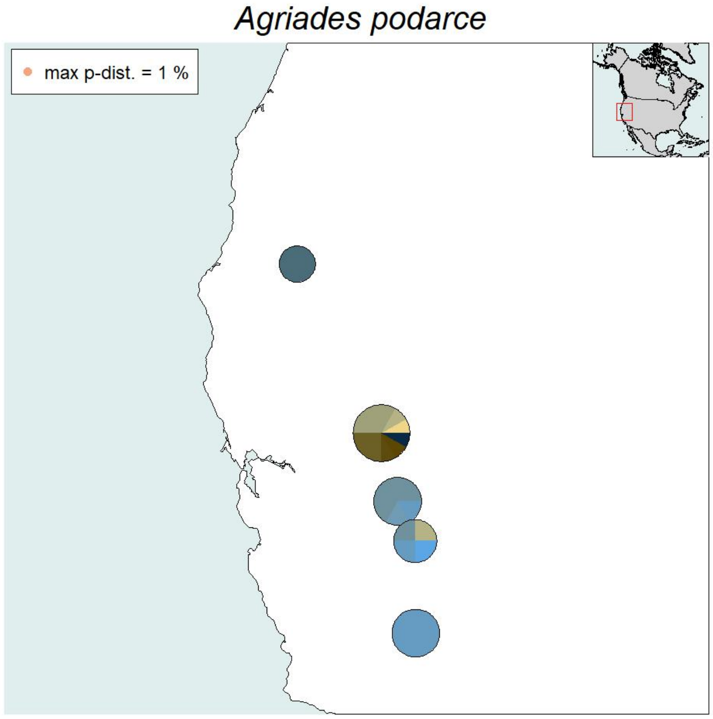

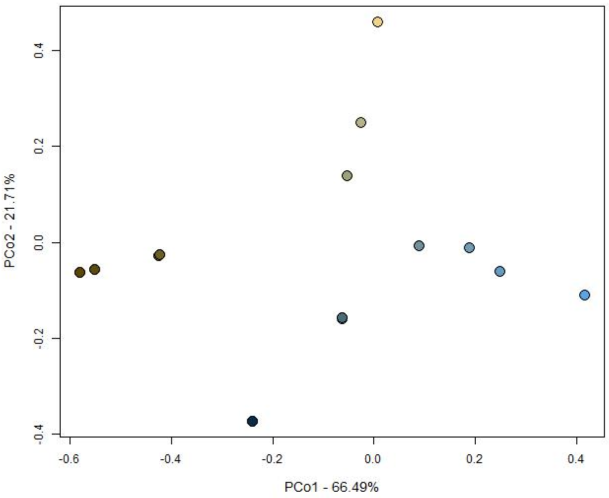

Figure 349 Map of *Agriades podarce* showing the localities of the sequenced specimens (left). Nearby localities are grouped in pies. Colours match the bidimensional colour space of the PCoA projection (right) of max p-dists among sequences (dots). Sequences= 30; Hap obs.= 13; Hap asympt.= 43.9; Hap % obs.= 29.6%; GST= 0.393; DST= 0.0014; HD= 0.89; ND= 0.0035; max p-dist= 1%.

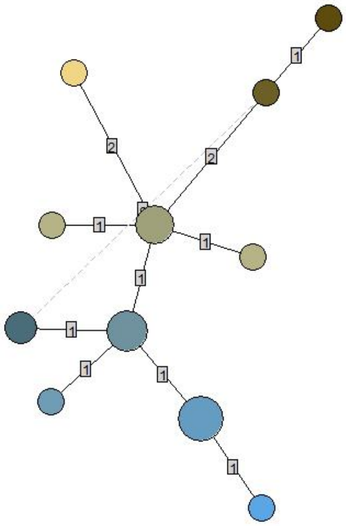

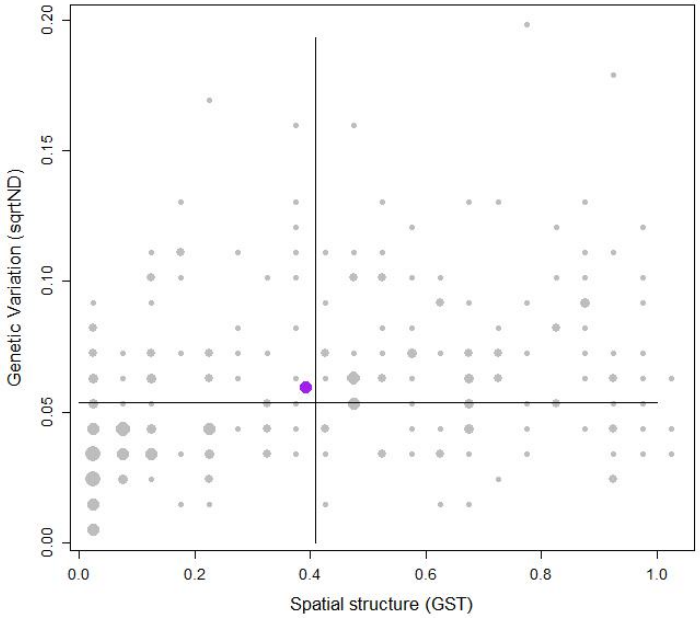

Figure 350: Haplotype network (left) of *Agriades podarce* sequences > 599 bp with colours matching the PCoA colour space (above). The bubble plot for mt-DNA polymorphism (square root transformed nucleotide diversity) and spatial structure (GST) among all species in the atlas and values for *Agriades podarce* (purple dot). The horizontal and vertical lines represent median values of nucleotide diversity and GST, respectively. Sequences > 599 bp= 26.

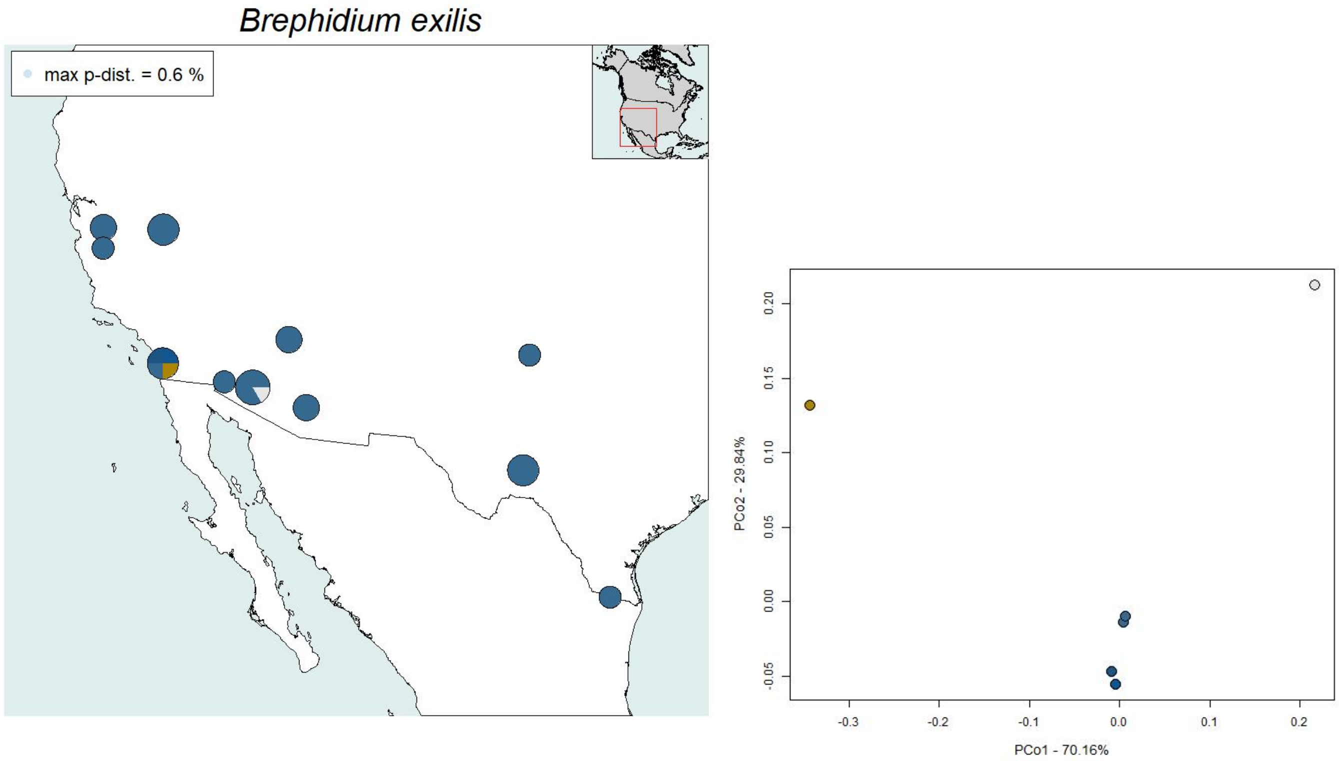

Figure 351 Map of *Brephidium exilis* showing the localities of the sequenced specimens (left). Nearby localities are grouped in pies. Colours match the bidimensional colour space of the PCoA projection (right) of max p-dists among sequences (dots). Sequences= 28; Hap obs.= 3; Hap asympt.= 4; Hap % obs.= 75.7%; GST= 0.005; DST= 0; HD= 0.14; ND= 0.0005; max p-dist= 0.6%.

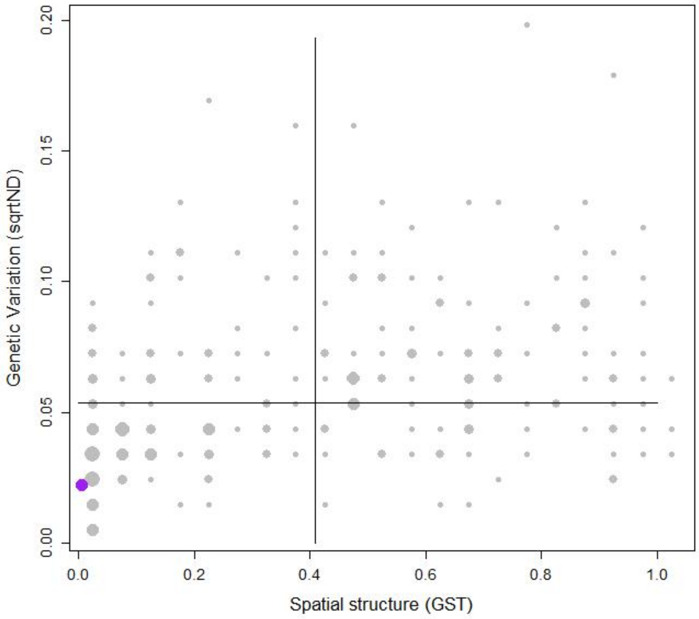

Figure 352: The bubble plot for mt-DNA polymorphism (square root transformed nucleotide diversity) and spatial structuree (GST) among all species in the atlas and values for *Brephidium exilis* (purple dot). The horizontal and vertical lines represent median values of nucleotide diversity and GST, respectively. Haplotype network analysis was not possible. Sequences > 599 bp = 25.

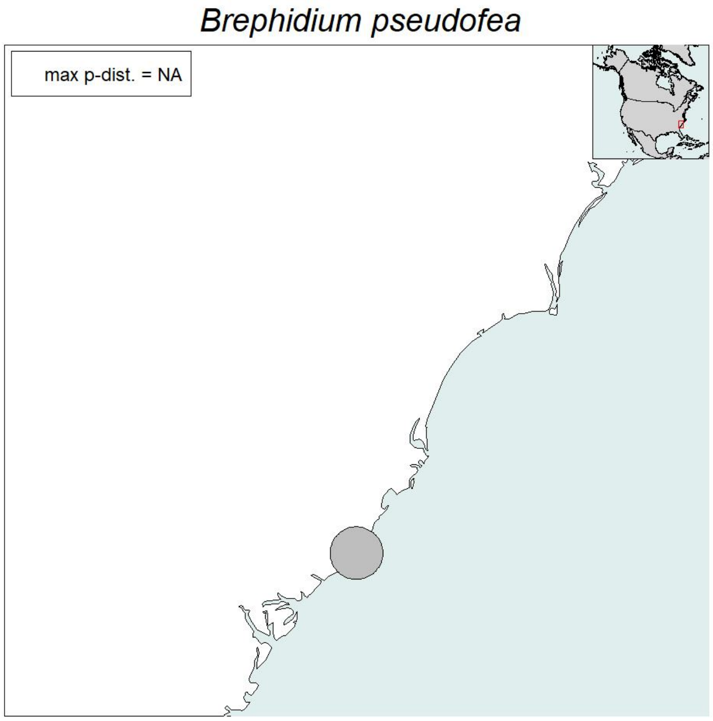

Figure 353: Map of *Brephidium pseudofea* showing the localities of the sequenced specimens. Nearby localities are grouped in pies. Due to the presence of a single haplotype PCoA projection was not done and a single grey colour was plotted on the map. Sequences= 1; Hap obs.= NA; Hap asympt.= NA; Hap % obs.= NA; GST= NaN; DST= NaN; HD= NA; ND= NA; max p-dist= NA.

Haplotype network analysis and bubble plot of *Brephidium pseudofea* were not possible. Sequences > 599 bp = 1.

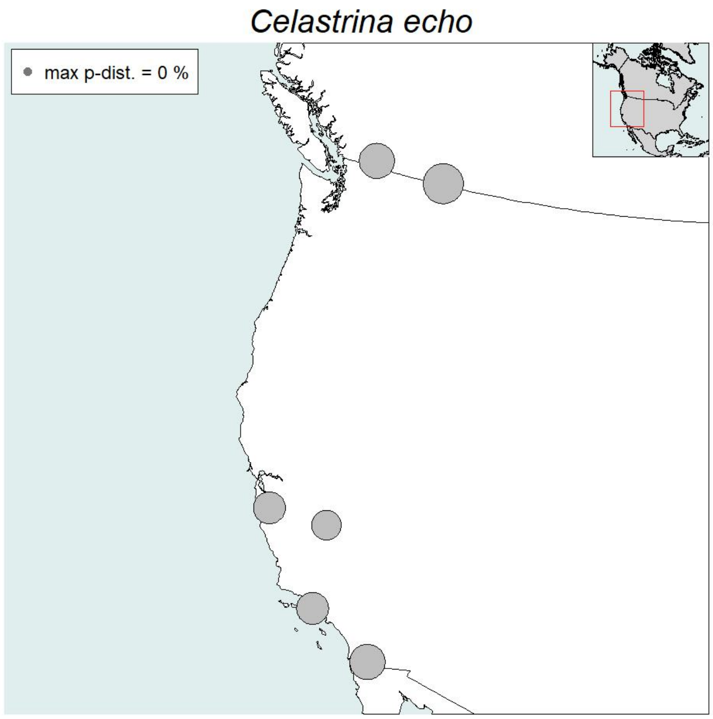

Figure 354: Map of *Celastrina echo* showing the localities of the sequenced specimens. Nearby localities are grouped in pies. Due to the presence of a single haplotype PCoA projection was not done and a single grey colour was plotted on the map. Sequences= 33; Hap obs.= 1; Hap asympt.= NA; Hap % obs.= NA%; GST= 0; DST= 0; HD= 0; ND= 0; max p-dist= 0%.

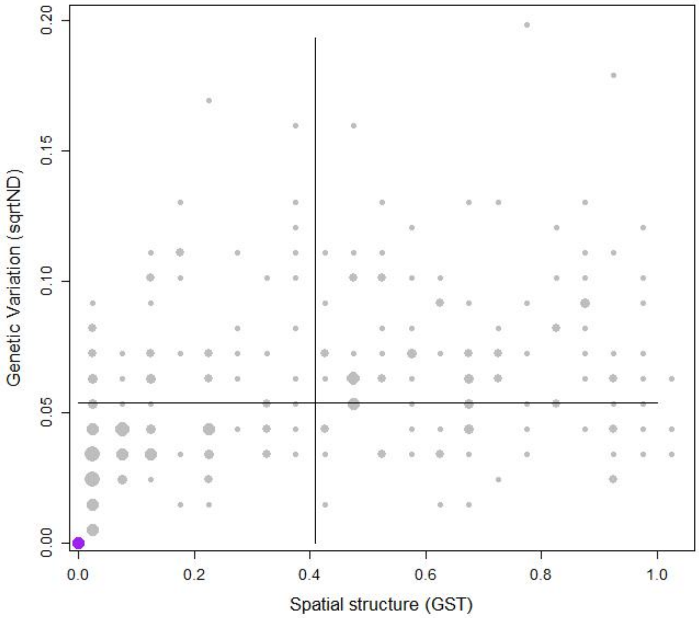

Figure 355: The bubble plot for mt-DNA polymorphism (square root transformed nucleotide diversity) and spatial structuree (GST) among all species in the atlas and values for *Celastrina echo* (purple dot). The horizontal and vertical lines represent median values of nucleotide diversity and GST, respectively. Haplotype network analysis was not possible. Sequences > 599 bp = 29.

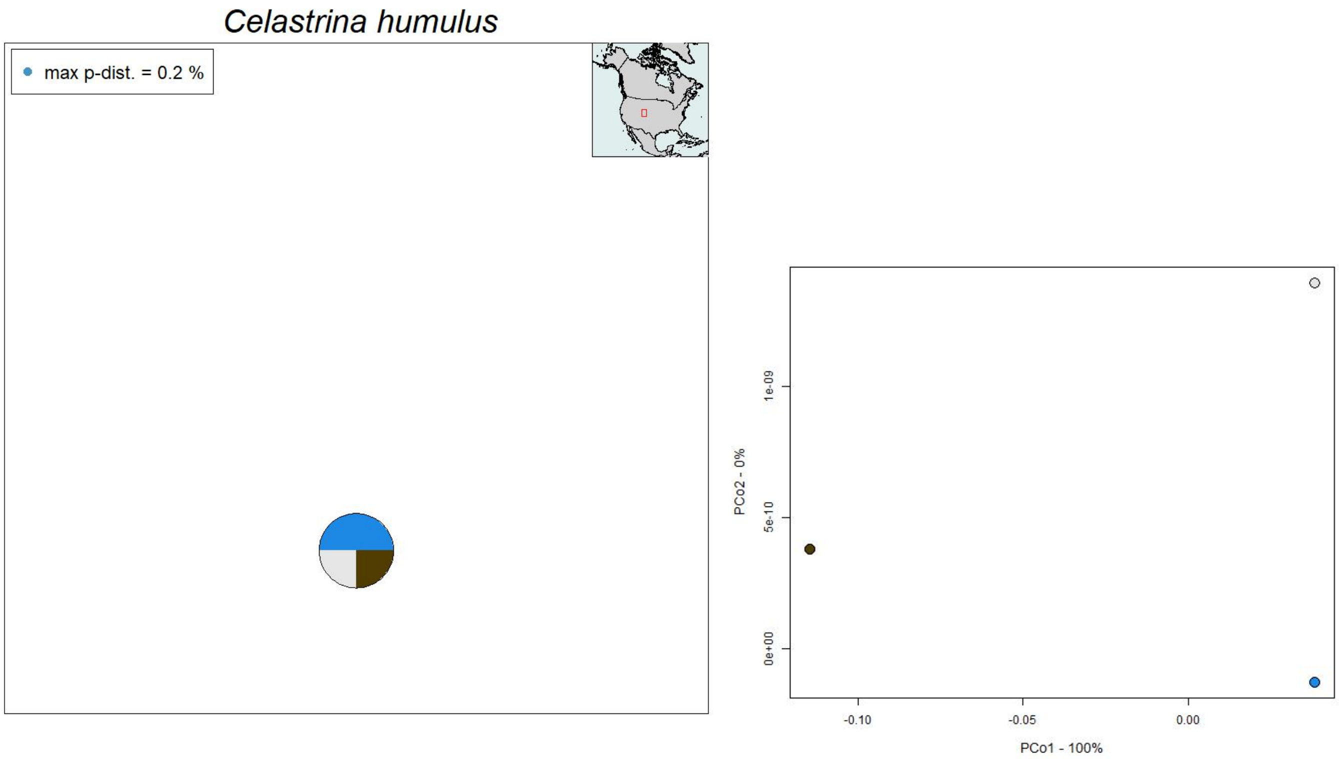

Figure 356 Map of *Celastrina humulus* showing the localities of the sequenced specimens (left). Nearby localities are grouped in pies. Colours match the bidimensional colour space of the PCoA projection (right) of max p-dists among sequences (dots). Sequences= 4; Hap obs.= 2; Hap asympt.= NA; Hap % obs.= NA%; GST= NaN; DST= NaN; HD= NA; ND= NA; max p-dist= 0.2%.

Haplotype network analysis and bubble plot of *Celastrina humulus* were not possible. Sequences > 599 bp = 4.

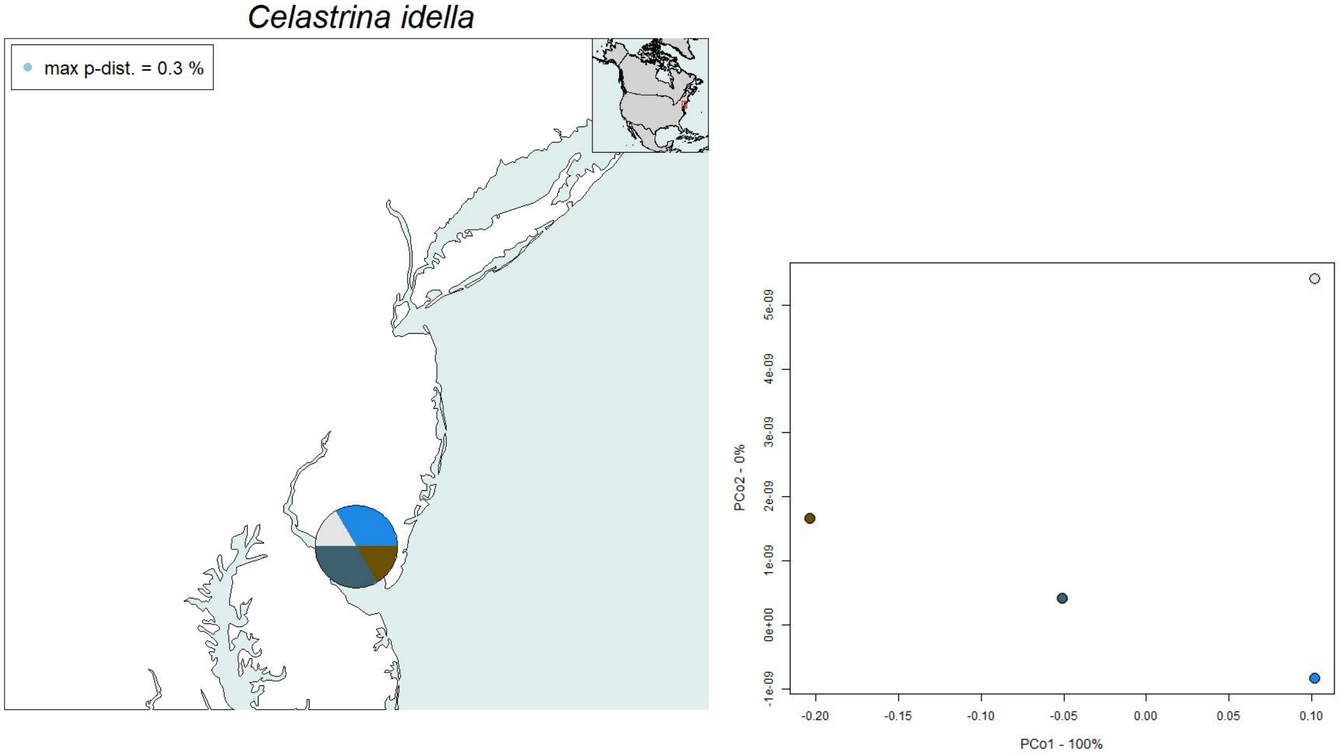

Figure 357 Map of *Celastrina idella* showing the localities of the sequenced specimens (left). Nearby localities are grouped in pies. Colours match the bidimensional colour space of the PCoA projection (right) of max p-dists among sequences (dots). Sequences= 6; Hap obs.= 3; Hap asympt.= NA; Hap % obs.= NA%; GST= NaN; DST= NaN; HD= NA; ND= NA; max p-dist= 0.3%.

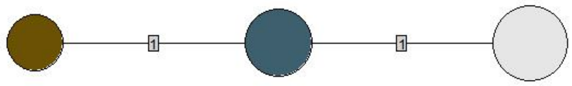

Figure 358: Haplotype network of *Celastrina idella*. Sequences > 599 bp= 6.

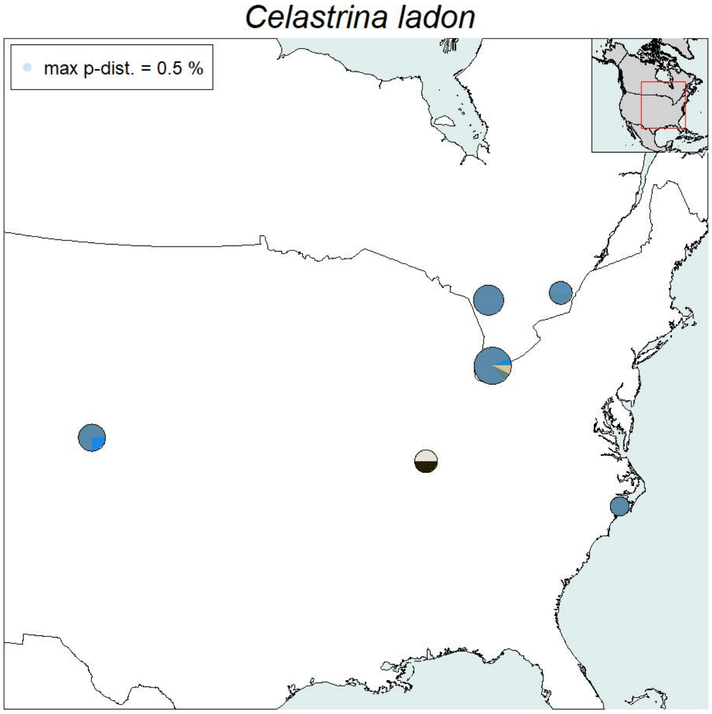

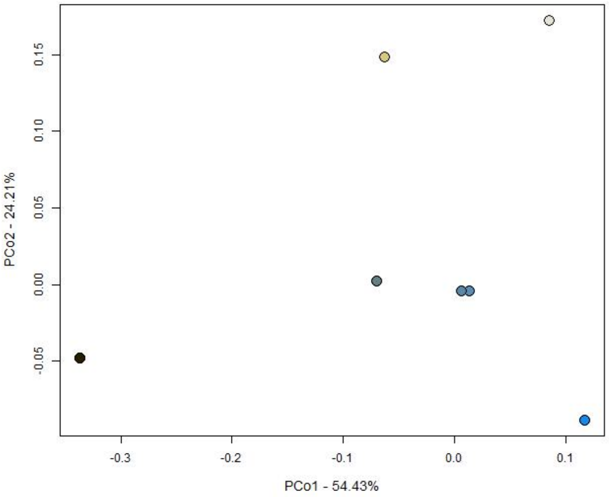

Figure 359 Map of *Celastrina ladon* showing the localities of the sequenced specimens (left). Nearby localities are grouped in pies. Colours match the bidimensional colour space of the PCoA projection (right) of max p-dists among sequences (dots). Sequences= 29; Hap obs.= 7; Hap asympt.= 21.5; Hap % obs.= 32.6%; GST= 0; DST= 0; HD= 0.377; ND= 0.0006; max p-dist= 0.5%.

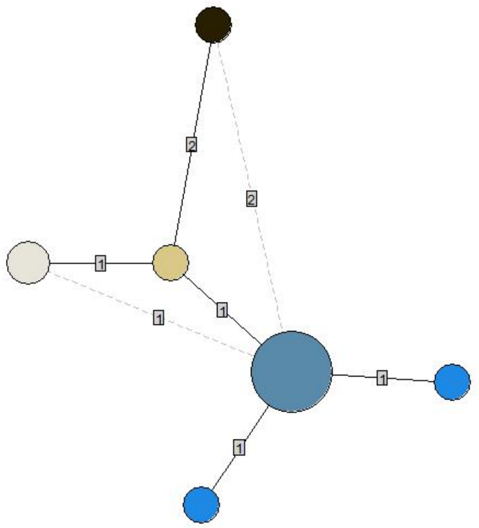

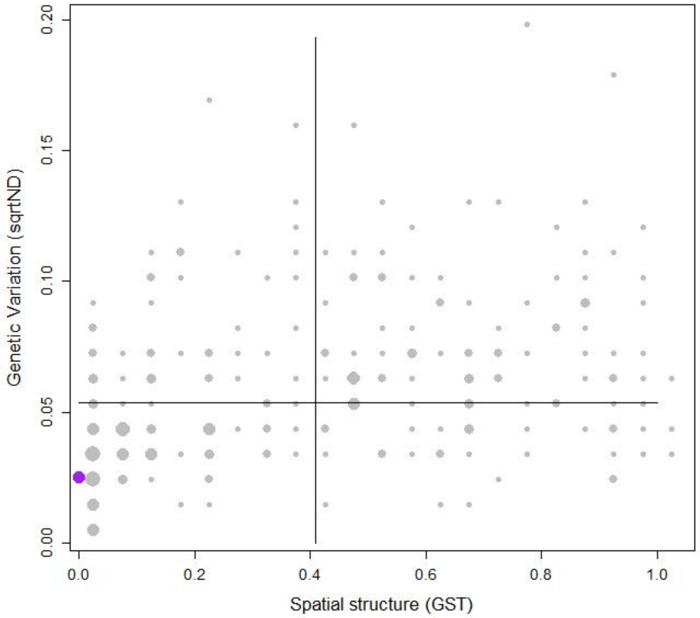

Figure 360: Haplotype network (left) of *Celastrina ladon* sequences > 599 bp with colours matching the PCoA colour space (above). The bubble plot for mt-DNA polymorphism (square root transformed nucleotide diversity) and spatial structure (GST) among all species in the atlas and values for *Celastrina ladon* (purple dot). The horizontal and vertical lines represent median values of nucleotide diversity and GST, respectively. Sequences > 599 bp= 29.

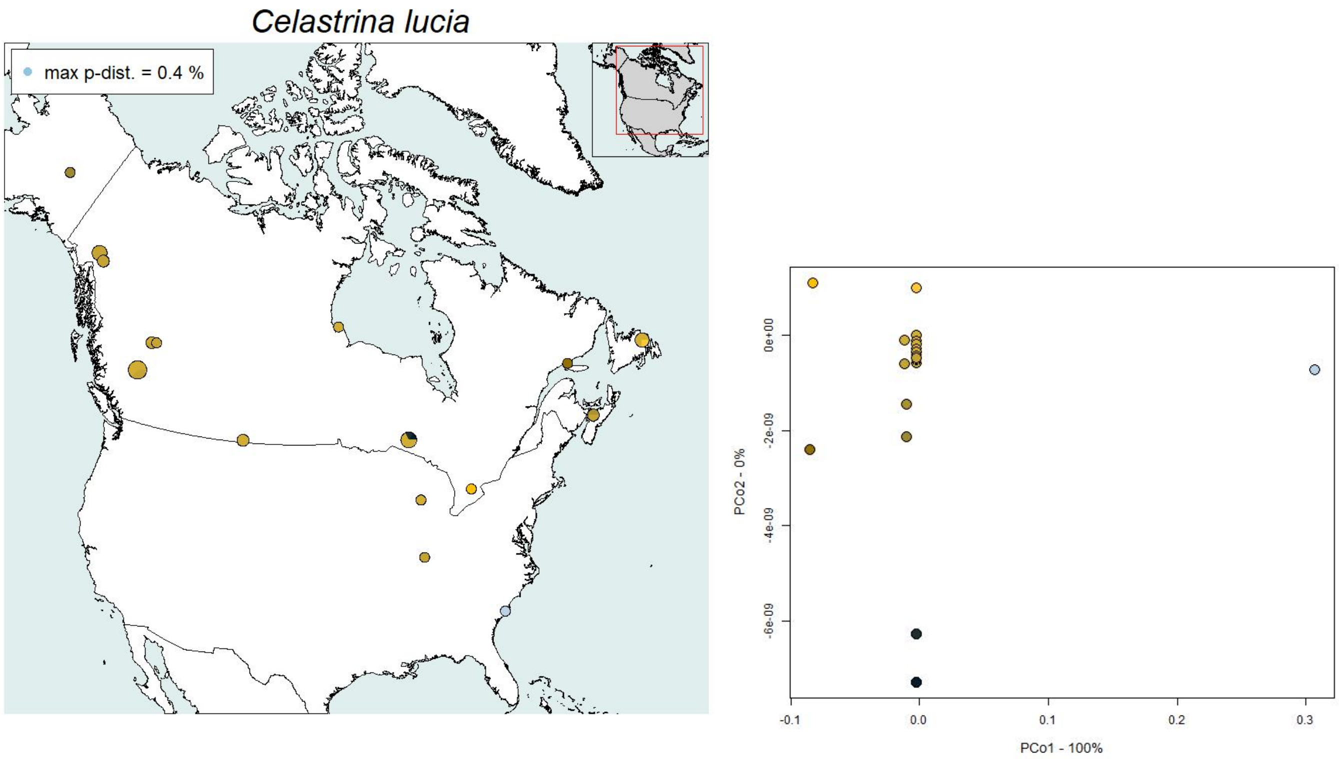

Figure 361 Map of *Celastrina lucia* showing the localities of the sequenced specimens (left). Nearby localities are grouped in pies. Colours match the bidimensional colour space of the PCoA projection (right) of max p-dists among sequences (dots). Sequences= 42; Hap obs.= 2; Hap asympt.= 2; Hap % obs.= 100%; GST= 0; DST= 0; HD= 0.048; ND= 0.0001; max p-dist= 0.4%.

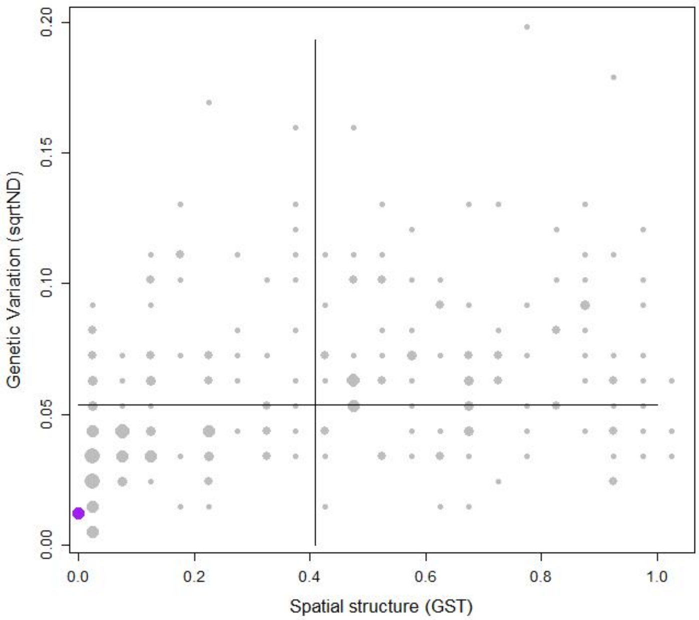

Figure 362: The bubble plot for mt-DNA polymorphism (square root transformed nucleotide diversity) and spatial structuree (GST) among all species in the atlas and values for *Celastrina lucia* (purple dot). The horizontal and vertical lines represent median values of nucleotide diversity and GST, respectively. Haplotype network analysis was not possible. Sequences > 599 bp = 40.

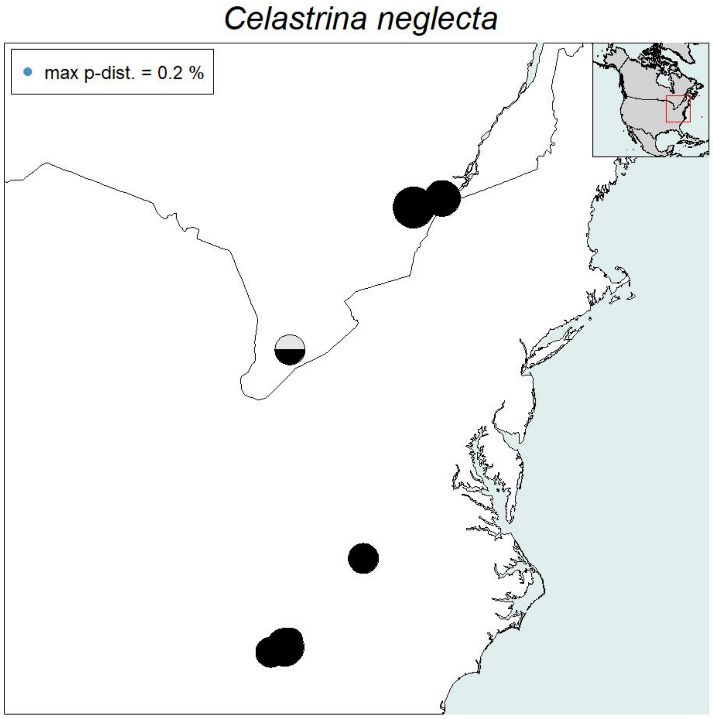

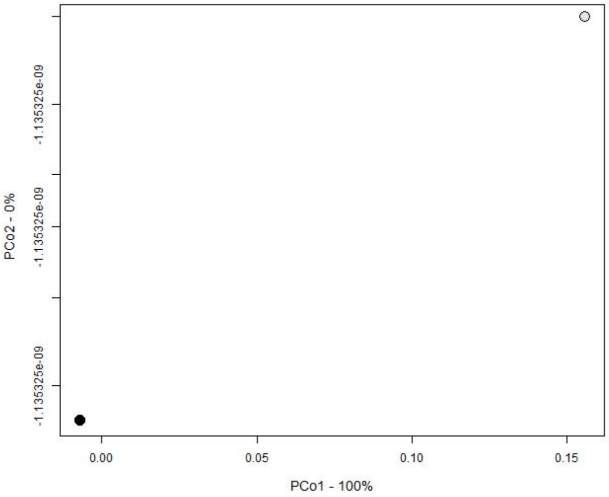

Figure 363 Map of *Celastrina neglecta* showing the localities of the sequenced specimens (left). Nearby localities are grouped in pies. Colours match the bidimensional colour space of the PCoA projection (right) of max p-dists among sequences (dots). Sequences= 23; Hap obs.= 2; Hap asympt.= 2; Hap % obs.= 100%; GST= 0; DST= 0; HD= 0.087; ND= 0.0001; max p-dist= 0.2%.

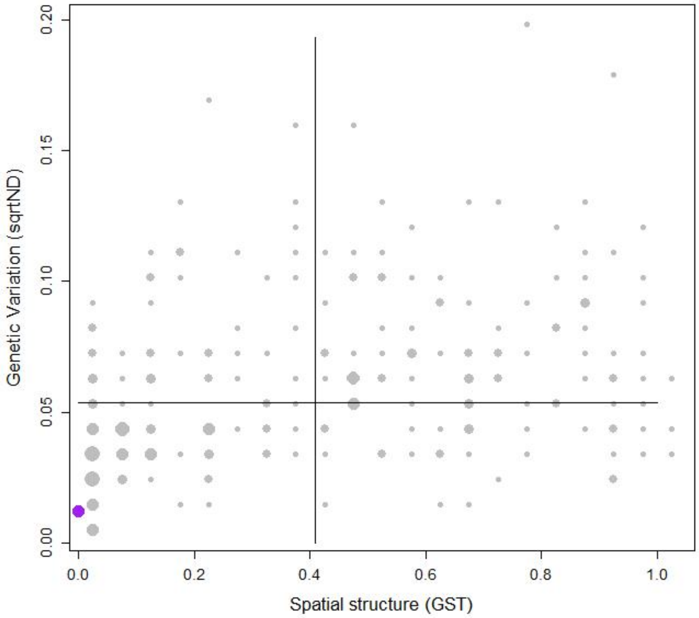

Figure 364: The bubble plot for mt-DNA polymorphism (square root transformed nucleotide diversity) and spatial structuree (GST) among all species in the atlas and values for *Celastrina neglecta* (purple dot). The horizontal and vertical lines represent median values of nucleotide diversity and GST, respectively. Haplotype network analysis was not possible. Sequences > 599 bp = 23.

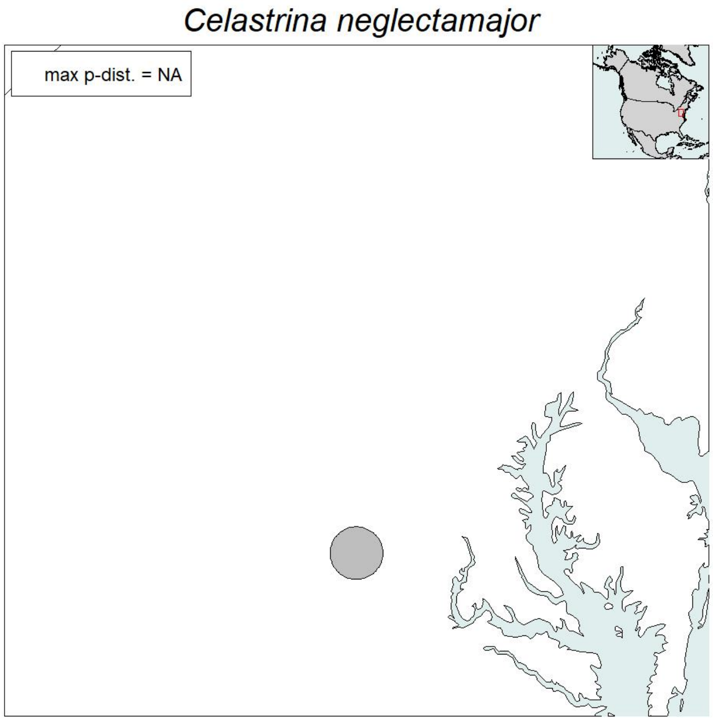

Figure 365: Map of *Celastrina neglectamajor* showing the localities of the sequenced specimens. Nearby localities are grouped in pies. Due to the presence of a single haplotype PCoA projection was not done and a single grey colour was plotted on the map. Sequences= 1; Hap obs.= NA; Hap asympt.= NA; Hap % obs.= NA; GST= NaN; DST= NaN; HD= NA; ND= NA; max p-dist= NA.

Haplotype network analysis and bubble plot of *Celastrina neglectamajor* were not possible. Sequences > 599 bp = 1.

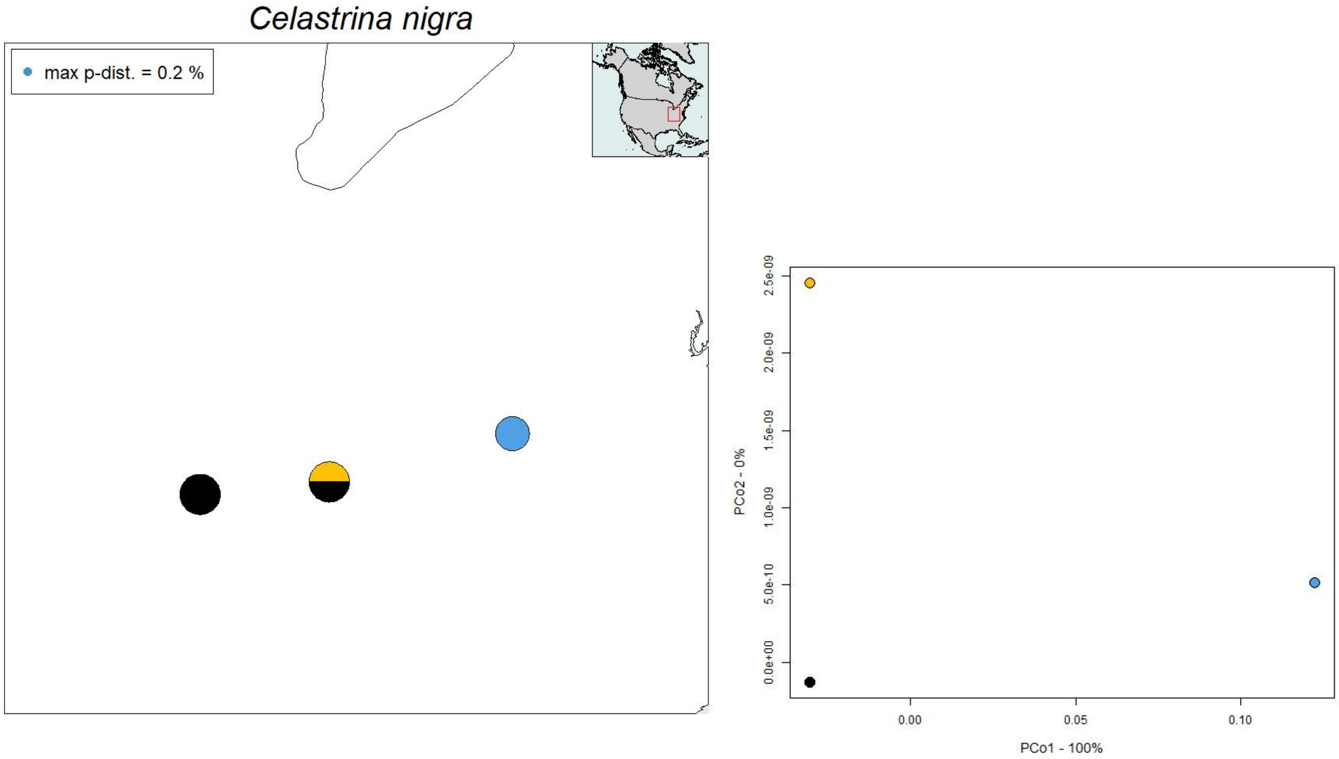

Figure 366 Map of *Celastrina nigra* showing the localities of the sequenced specimens (left). Nearby localities are grouped in pies. Colours match the bidimensional colour space of the PCoA projection (right) of max p-dists among sequences (dots). Sequences= 5; Hap obs.= 2; Hap asympt.= NA; Hap % obs.= NA%; GST= NaN; DST= NaN; HD= NA; ND= NA; max p-dist= 0.2%.

Haplotype network analysis and bubble plot of *Celastrina nigra* were not possible. Sequences > 599 bp = 5.

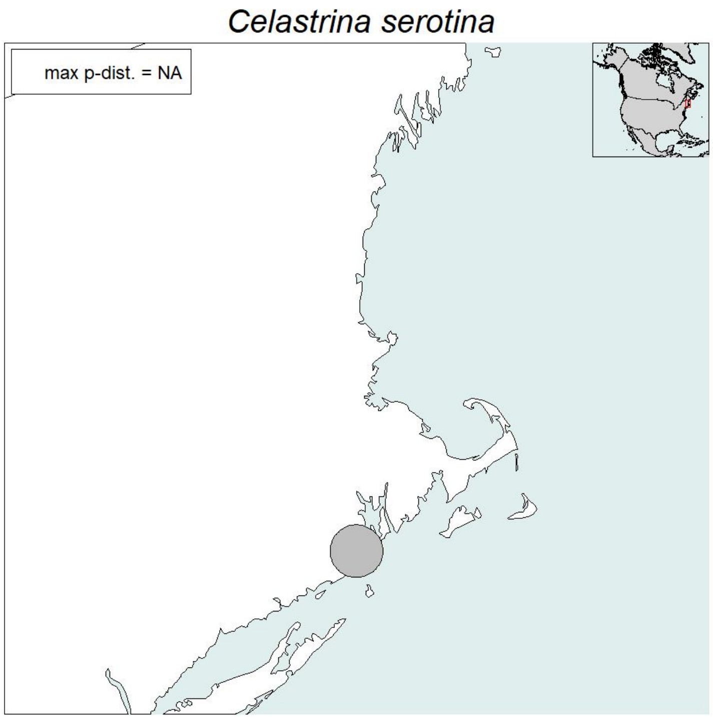

Figure 367: Map of *Celastrina serotina* showing the localities of the sequenced specimens. Nearby localities are grouped in pies. Due to the presence of a single haplotype PCoA projection was not done and a single grey colour was plotted on the map. Sequences= 1; Hap obs.= NA; Hap asympt.= NA; Hap % obs.= NA; GST= NaN; DST= NaN; HD= NA; ND= NA; max p-dist= NA.

Haplotype network analysis and bubble plot of *Celastrina serotina* were not possible. Sequences > 599 bp = 1.

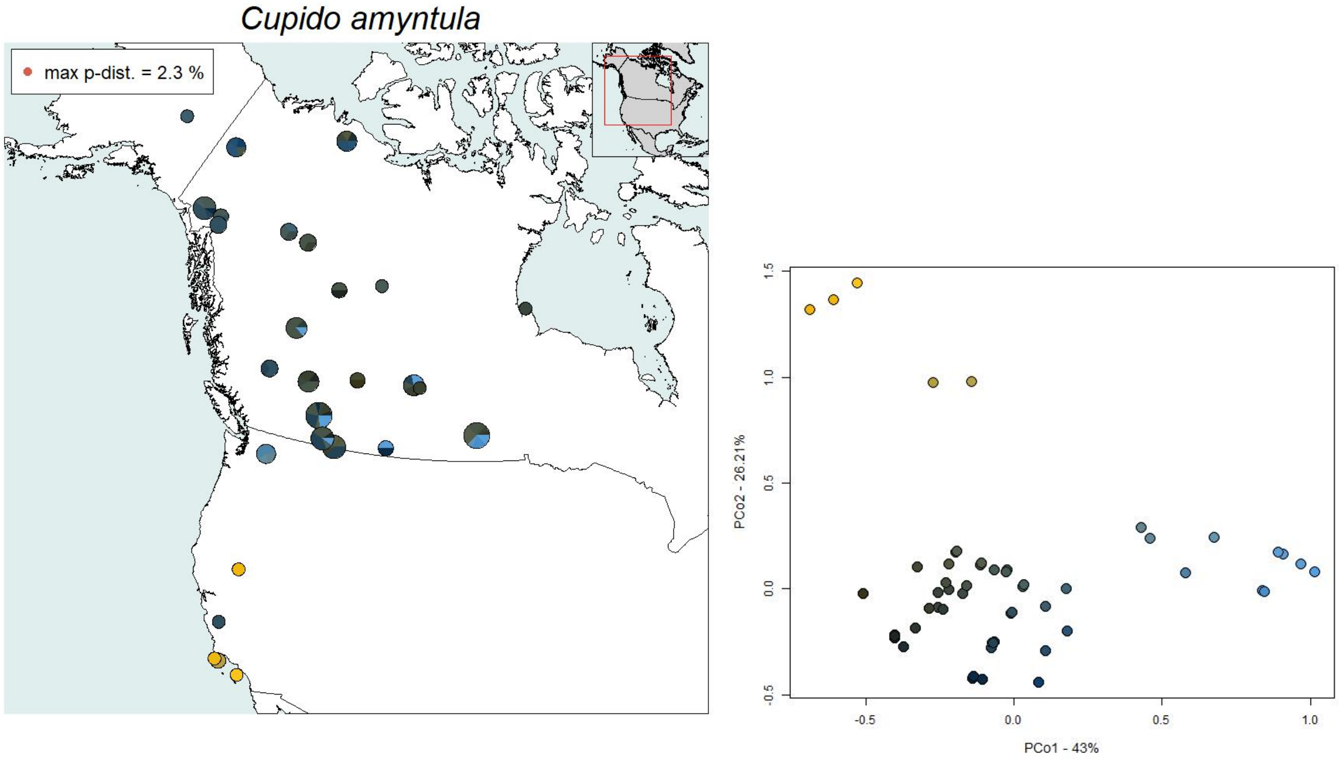

Figure 368 Map of *Cupido amyntula* showing the localities of the sequenced specimens (left). Nearby localities are grouped in pies. Colours match the bidimensional colour space of the PCoA projection (right) of max p-dists among sequences (dots). Sequences= 131; Hap obs.= 37; Hap asympt.= 217.9; Hap % obs.= 17%; GST= 0.12; DST= 0.0006; HD= 0.888; ND= 0.0054; max p-dist= 2.3%.

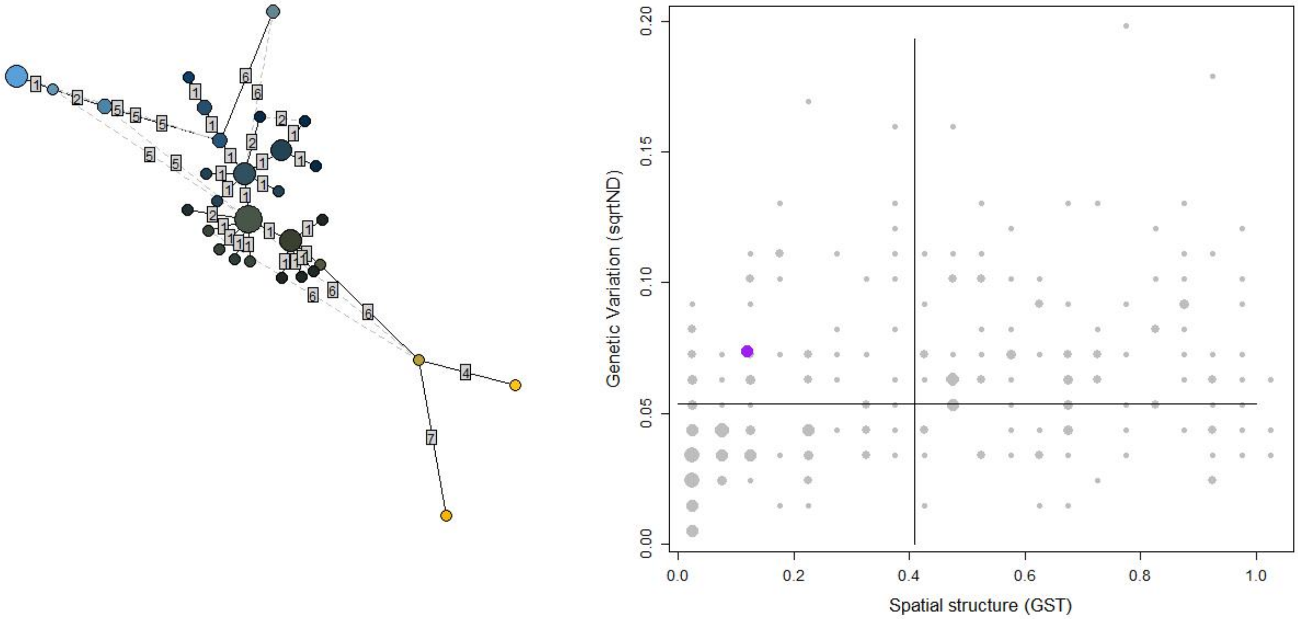

Figure 369: Haplotype network (left) of *Cupido amyntula* sequences > 599 bp with colours matching the PCoA colour space (above). The bubble plot for mt-DNA polymorphism (square root transformed nucleotide diversity) and spatial structure (GST) among all species in the atlas and values for *Cupido amyntula* (purple dot). The horizontal and vertical lines represent median values of nucleotide diversity and GST, respectively. Sequences > 599 bp= 118.

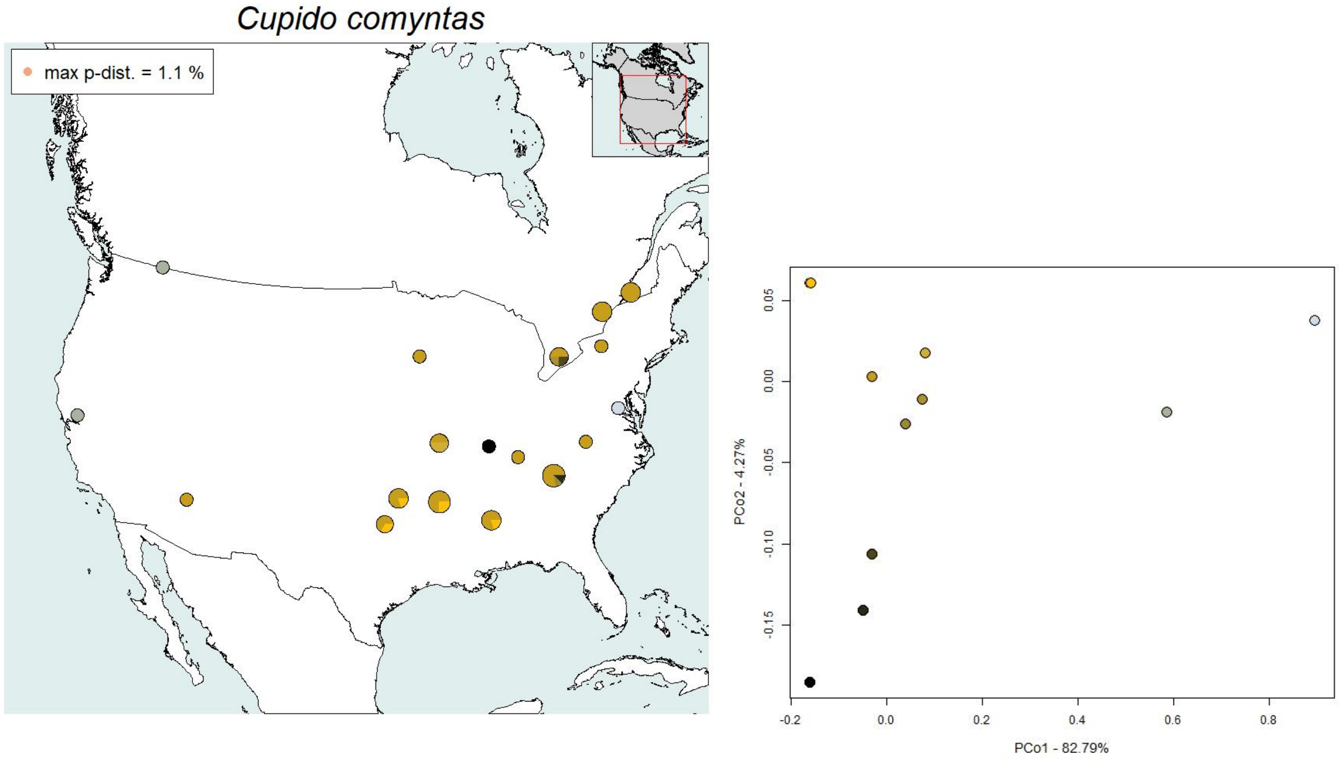

Figure 370 Map of *Cupido comyntas* showing the localities of the sequenced specimens (left). Nearby localities are grouped in pies. Colours match the bidimensional colour space of the PCoA projection (right) of max p-dists among sequences (dots). Sequences= 57; Hap obs.= 12; Hap asympt.= 31.9; Hap % obs.= 37.6%; GST= 0.041; DST= 0; HD= 0.406; ND= 0.0011; max p-dist= 1.1%.

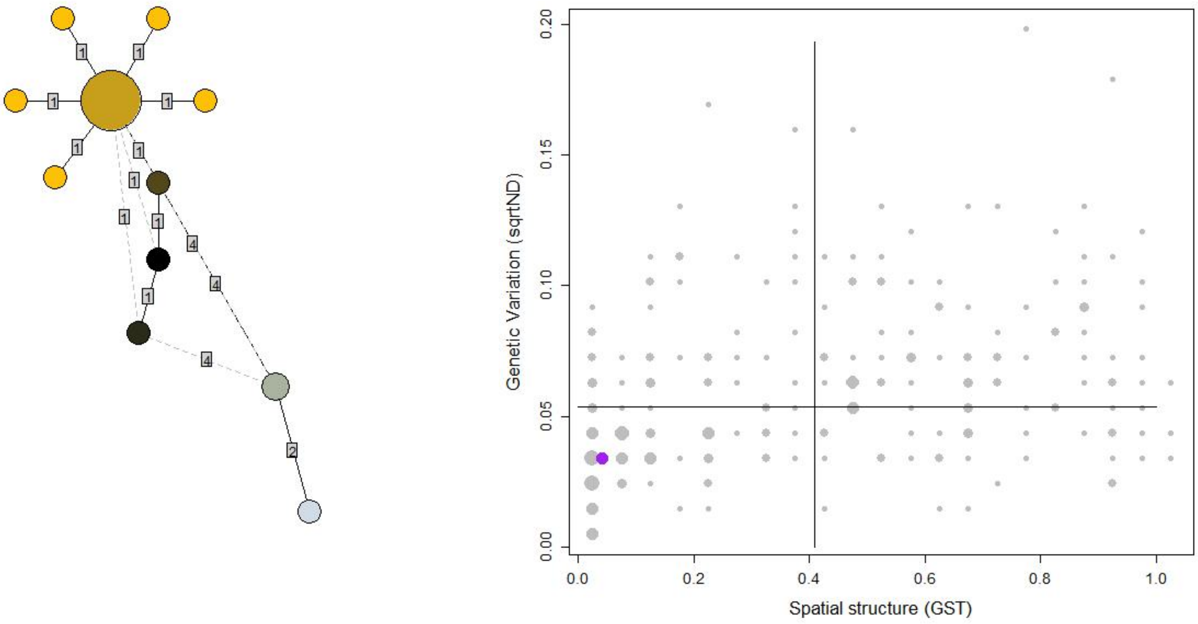

Figure 371: Haplotype network (left) of *Cupido comyntas* sequences > 599 bp with colours matching the PCoA colour space (above). The bubble plot for mt-DNA polymorphism (square root transformed nucleotide diversity) and spatial structure (GST) among all species in the atlas and values for *Cupido comyntas* (purple dot). The horizontal and vertical lines represent median values of nucleotide diversity and GST, respectively. Sequences > 599 bp= 56.

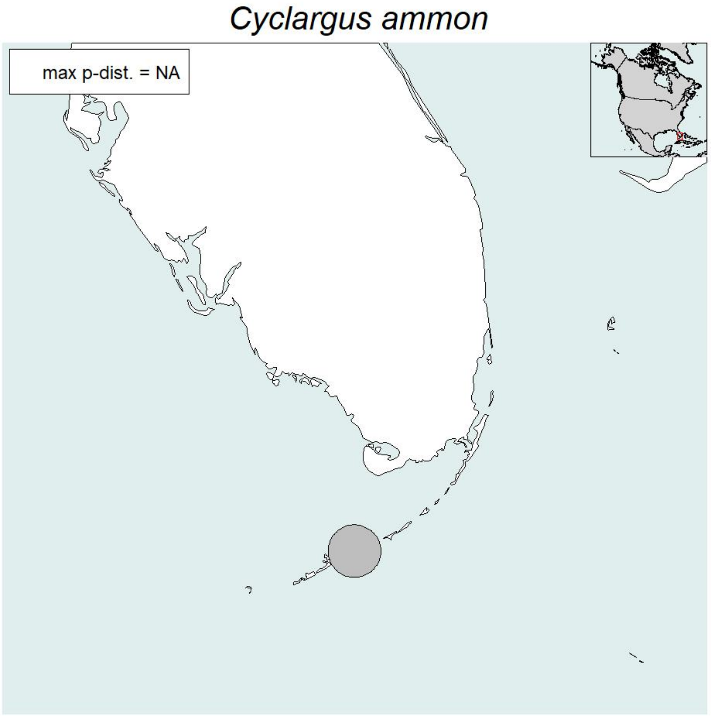

Figure 372: Map of *Cyclargus ammon* showing the localities of the sequenced specimens. Nearby localities are grouped in pies. Due to the presence of a single haplotype PCoA projection was not done and a single grey colour was plotted on the map. Sequences= 1; Hap obs.= NA; Hap asympt.= NA; Hap % obs.= NA; GST= NaN; DST= NaN; HD= NA; ND= NA; max p-dist= NA.

Haplotype network analysis and bubble plot of *Cyclargus ammon* were not possible. Sequences > 599 bp = 1.

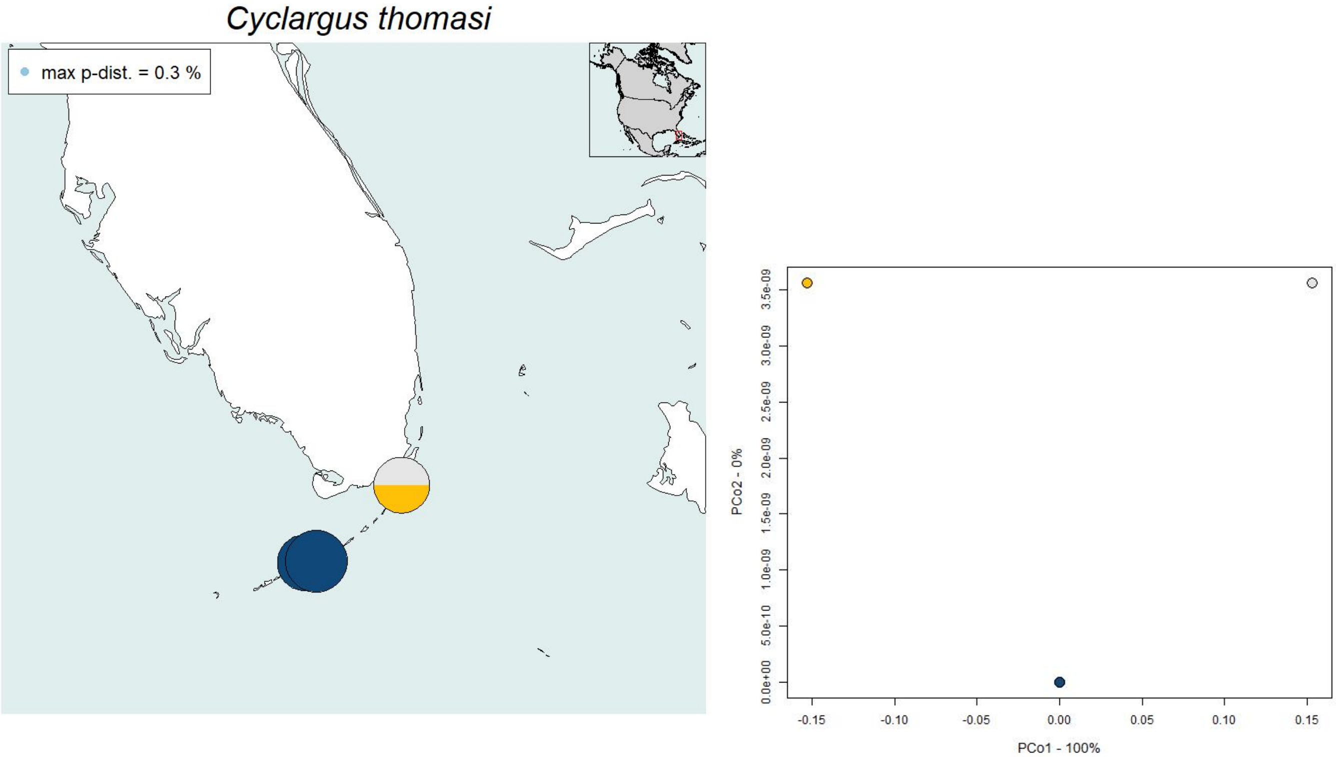

Figure 373 Map of *Cyclargus thomasi* showing the localities of the sequenced specimens (left). Nearby localities are grouped in pies. Colours match the bidimensional colour space of the PCoA projection (right) of max p-dists among sequences (dots). Sequences= 7; Hap obs.= 3; Hap asympt.= NA; Hap % obs.= NA%; GST= NaN; DST= NaN; HD= NA; ND= NA; max p-dist= 0.3%.

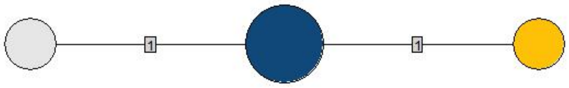

Figure 374: Haplotype network of *Cyclargus thomasi*. Sequences > 599 bp= 7.

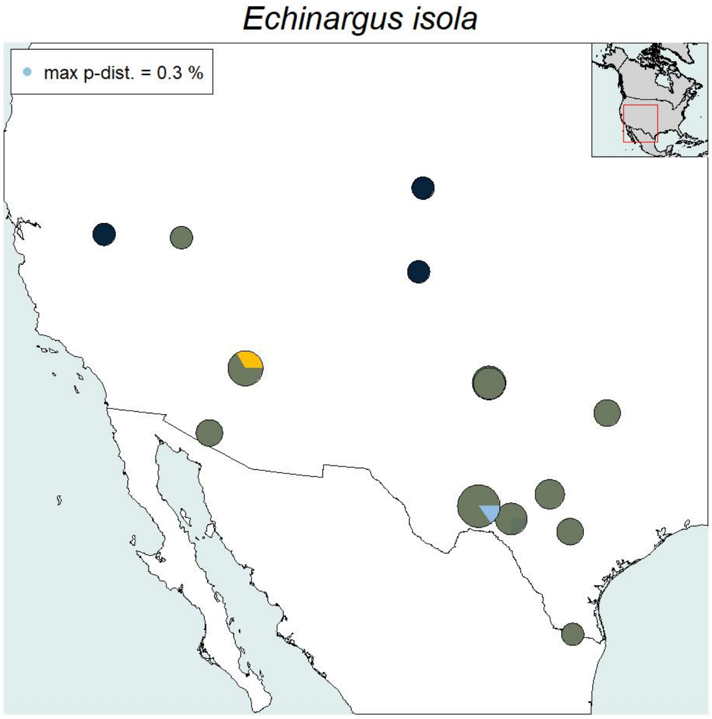

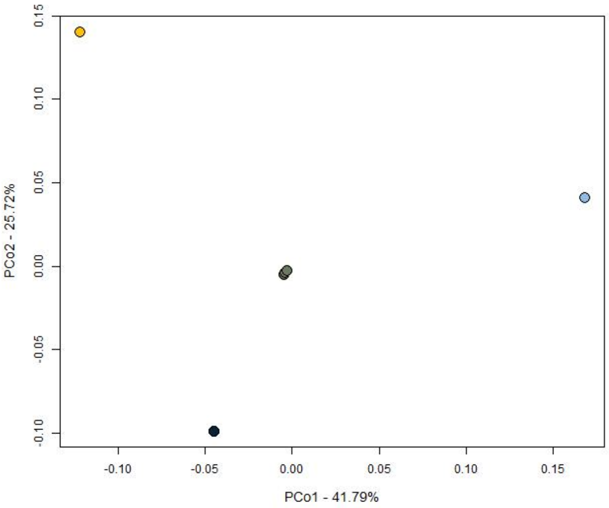

Figure 375 Map of *Echinargus isola* showing the localities of the sequenced specimens (left). Nearby localities are grouped in pies. Colours match the bidimensional colour space of the PCoA projection (right) of max p-dists among sequences (dots). Sequences= 46; Hap obs.= 6; Hap asympt.= 10.4; Hap % obs.= 57.7%; GST= 0.099; DST= 0; HD= 0.317; ND= 0.0005; max p-dist= 0.3%.

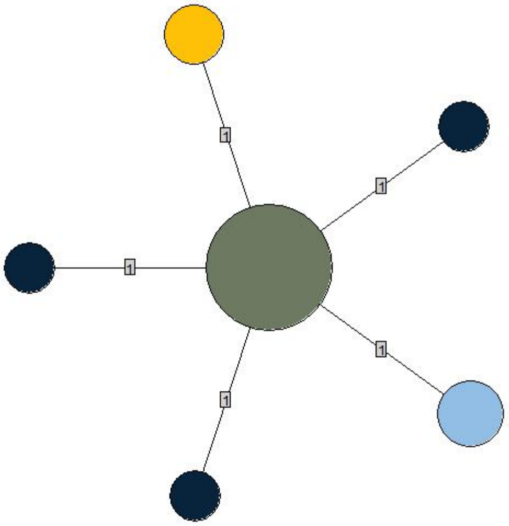
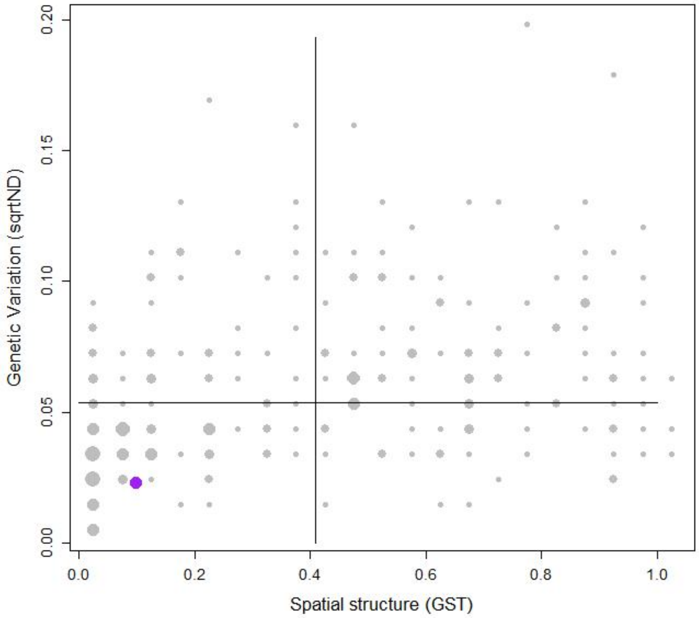

Figure 376: Haplotype network (left) of *Echinargus isola* sequences > 599 bp with colours matching the PCoA colour space (above). The bubble plot for mt-DNA polymorphism (square root transformed nucleotide diversity) and spatial structure (GST) among all species in the atlas and values for *Echinargus isola* (purple dot). The horizontal and vertical lines represent median values of nucleotide diversity and GST, respectively. Sequences > 599 bp= 46.

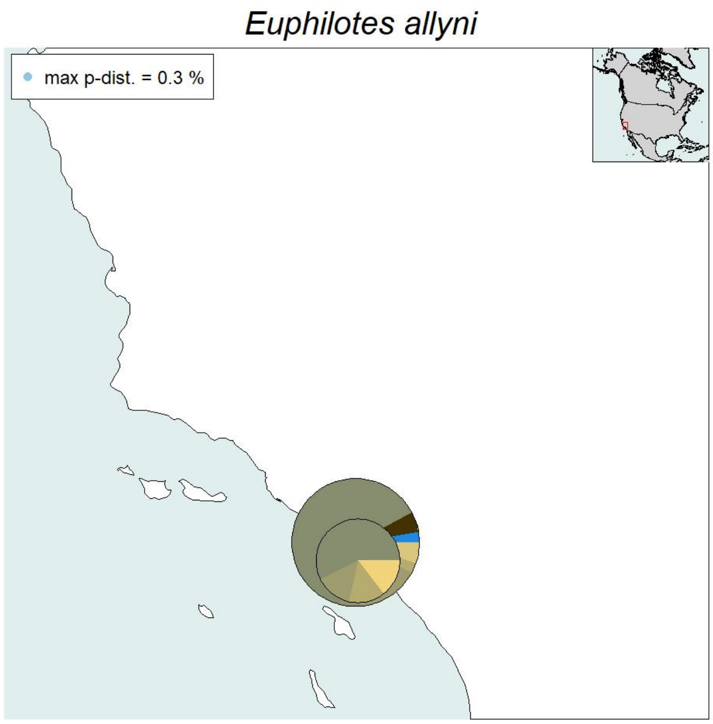

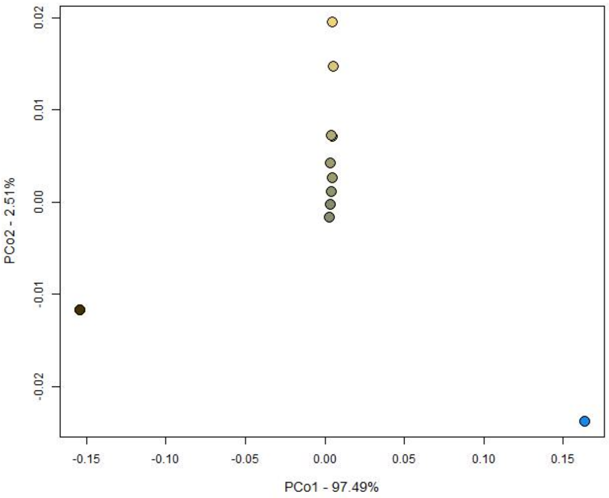

Figure 377 Map of *Euphilotes allyni* showing the localities of the sequenced specimens (left). Nearby localities are grouped in pies. Colours match the bidimensional colour space of the PCoA projection (right) of max p-dists among sequences (dots). Sequences= 45; Hap obs.= 3; Hap asympt.= 3.5; Hap % obs.= 86%; GST= NaN; DST= NaN; HD= 0.129; ND= 0.0002; max p-dist= 0.3%.

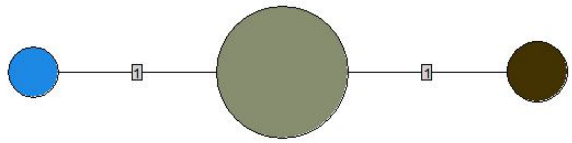

Figure 378: Haplotype network of *Euphilotes allyni*. Sequences > 599 bp= 45.

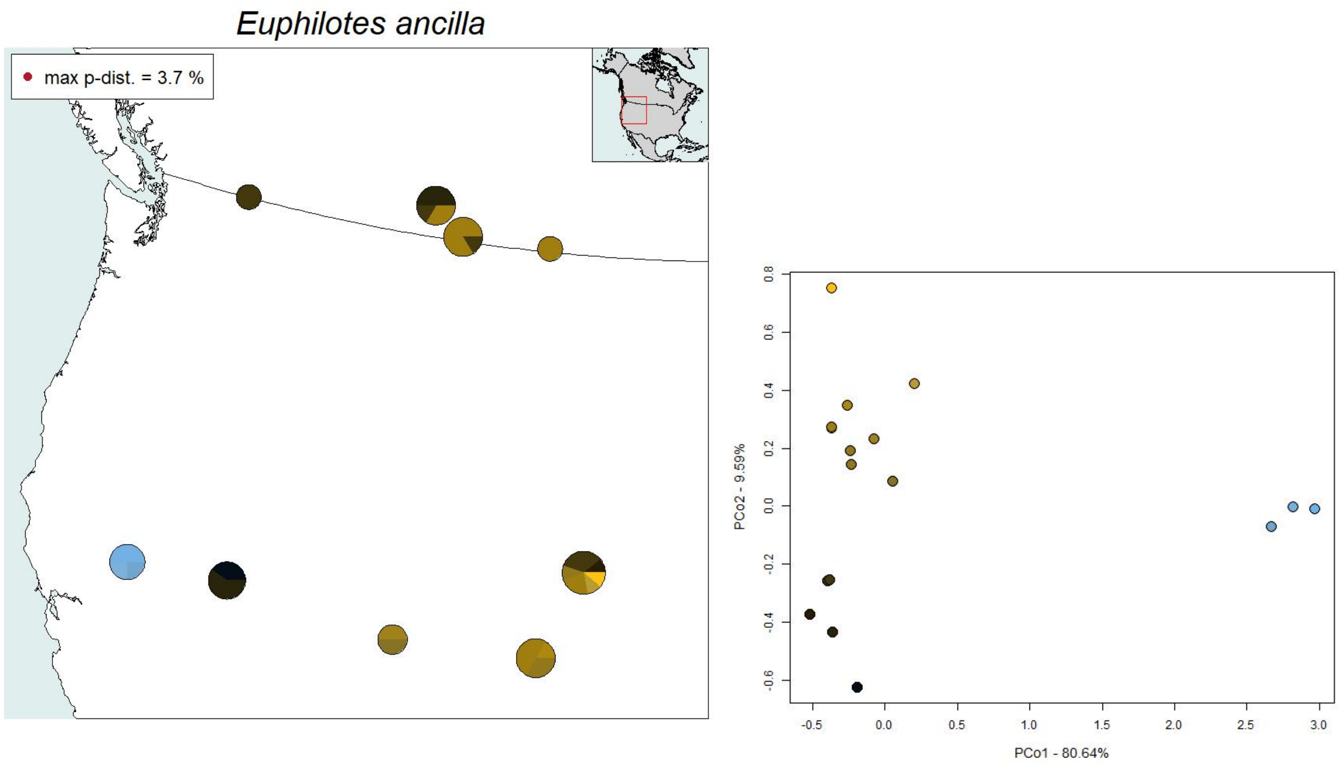

Figure 379 Map of *Euphilotes ancilla* showing the localities of the sequenced specimens (left). Nearby localities are grouped in pies. Colours match the bidimensional colour space of the PCoA projection (right) of max p-dists among sequences (dots). Sequences= 40; Hap obs.= 17; Hap asympt.= 46.5; Hap % obs.= 36.6%; GST= 0.613; DST= 0.0068; HD= 0.91; ND= 0.0104; max p-dist= 3.7%.

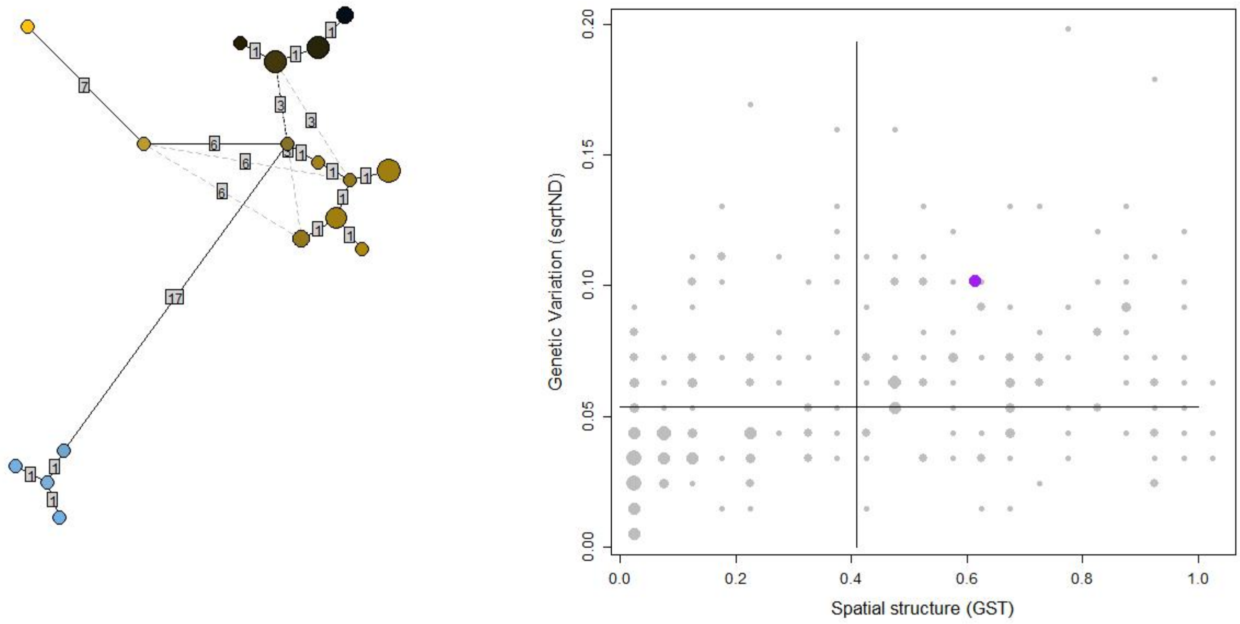

Figure 380: Haplotype network (left) of *Euphilotes ancilla* sequences > 599 bp with colours matching the PCoA colour space (above). The bubble plot for mt-DNA polymorphism (square root transformed nucleotide diversity) and spatial structure (GST) among all species in the atlas and values for *Euphilotes ancilla* (purple dot). The horizontal and vertical lines represent median values of nucleotide diversity and GST, respectively. Sequences > 599 bp= 40.

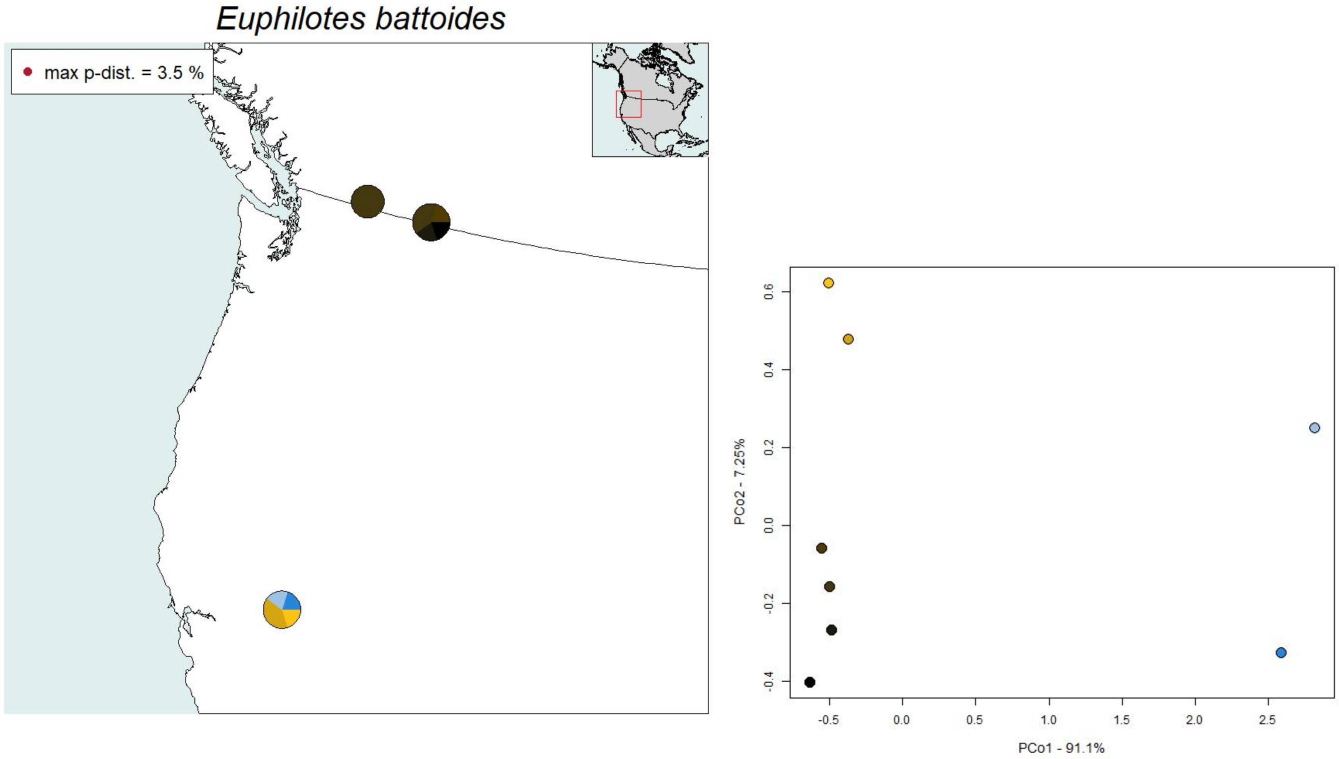

Figure 381 Map of *Euphilotes battoides* showing the localities of the sequenced specimens (left). Nearby localities are grouped in pies. Colours match the bidimensional colour space of the PCoA projection (right) of max p-dists among sequences (dots). Sequences= 13; Hap obs.= 7; Hap asympt.= 10.7; Hap % obs.= 65.5%; GST= 0.526; DST= 0.0039; HD= 0.846; ND= 0.0119; max p-dist= 3.5%.

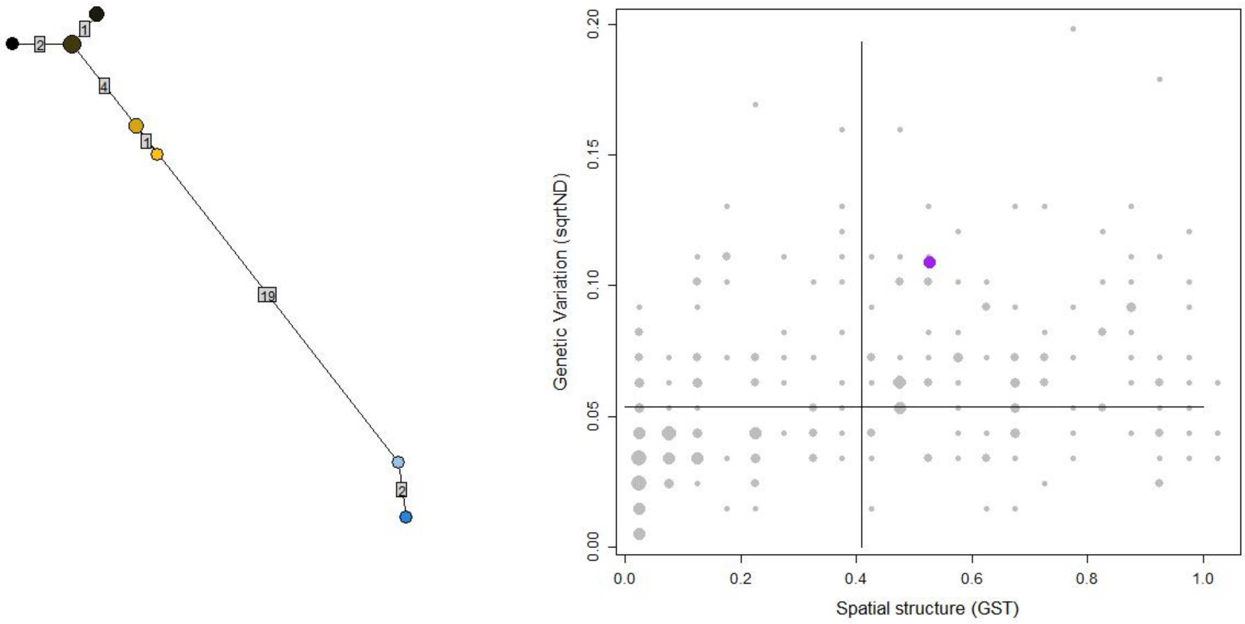

Figure 382: Haplotype network (left) of *Euphilotes battoides* sequences > 599 bp with colours matching the PCoA colour space (above). The bubble plot for mt-DNA polymorphism (square root transformed nucleotide diversity) and spatial structure (GST) among all species in the atlas and values for *Euphilotes battoides* (purple dot). The horizontal and vertical lines represent median values of nucleotide diversity and GST, respectively. Sequences > 599 bp= 13.

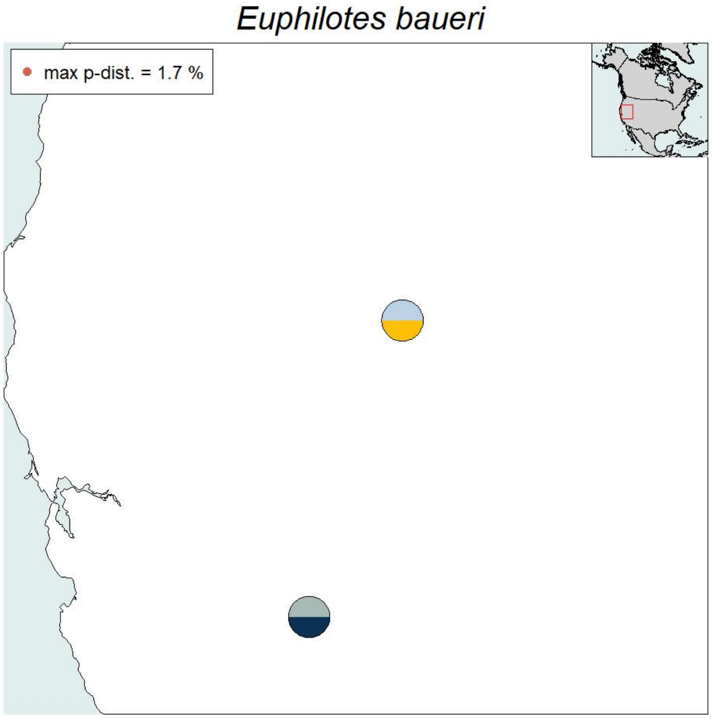

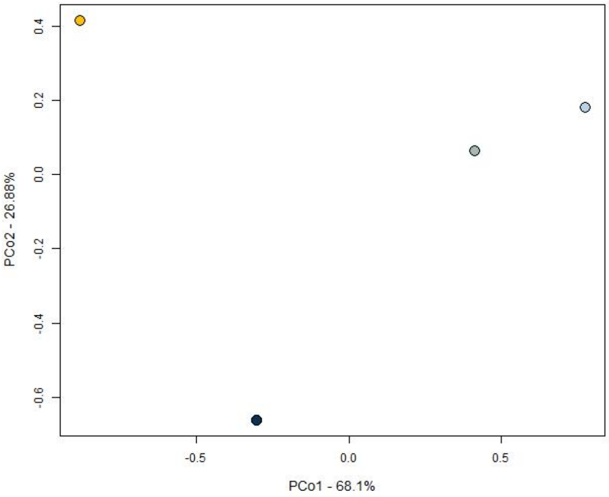

Figure 383 Map of *Euphilotes baueri* showing the localities of the sequenced specimens (left). Nearby localities are grouped in pies. Colours match the bidimensional colour space of the PCoA projection (right) of max p-dists among sequences (dots). Sequences= 4; Hap obs.= 4; Hap asympt.= NA; Hap % obs.= NA%; GST= NaN; DST= NaN; HD= NA; ND= NA; max p-dist= 1.7%.

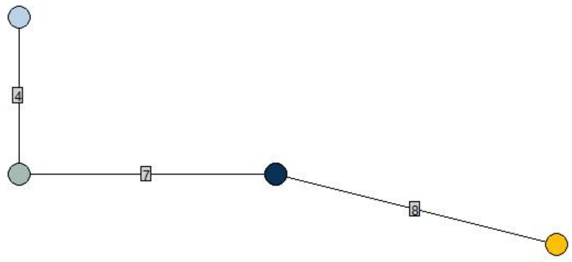

Figure 384: Haplotype network of *Euphilotes baueri*. Sequences > 599 bp= 4.

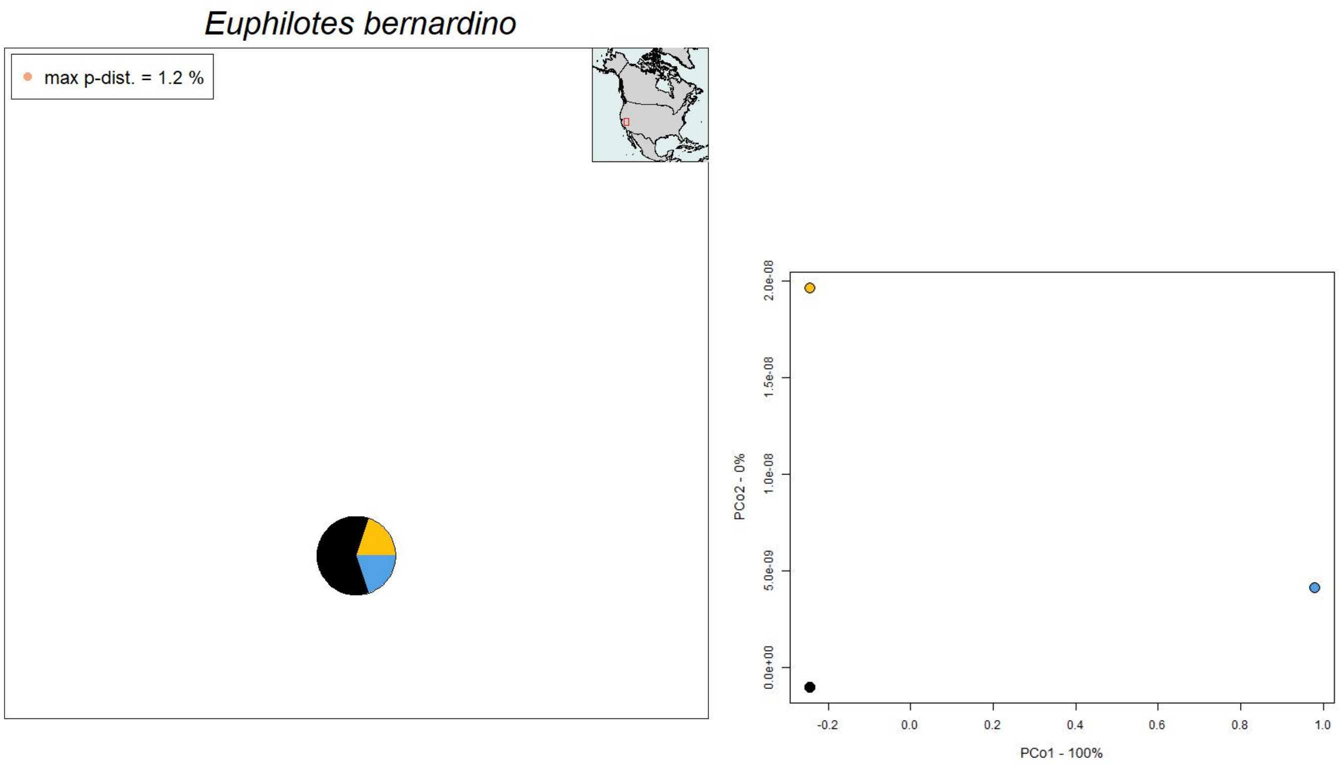

Figure 385 Map of *Euphilotes bernardino* showing the localities of the sequenced specimens (left). Nearby localities are grouped in pies. Colours match the bidimensional colour space of the PCoA projection (right) of max p-dists among sequences (dots). Sequences= 5; Hap obs.= 2; Hap asympt.= NA; Hap % obs.= NA%; GST= NaN; DST= NaN; HD= NA; ND= NA; max p-dist= 1.2%.

Haplotype network analysis and bubble plot of *Euphilotes bernardino* were not possible. Sequences > 599 bp = 5.

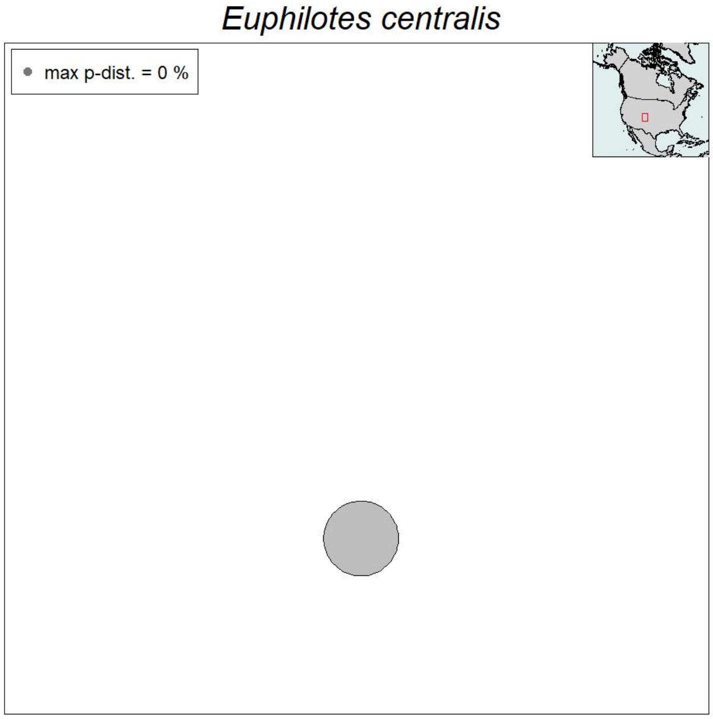

Figure 386: Map of *Euphilotes centralis* showing the localities of the sequenced specimens. Nearby localities are grouped in pies. Due to the presence of a single haplotype PCoA projection was not done and a single grey colour was plotted on the map. Sequences= 5; Hap obs.= 1; Hap asympt.= NA; Hap % obs.= NA%; GST= NaN; DST= NaN; HD= NA; ND= NA; max p-dist= 0%.

Haplotype network analysis and bubble plot of *Euphilotes centralis* were not possible. Sequences > 599 bp = 5.

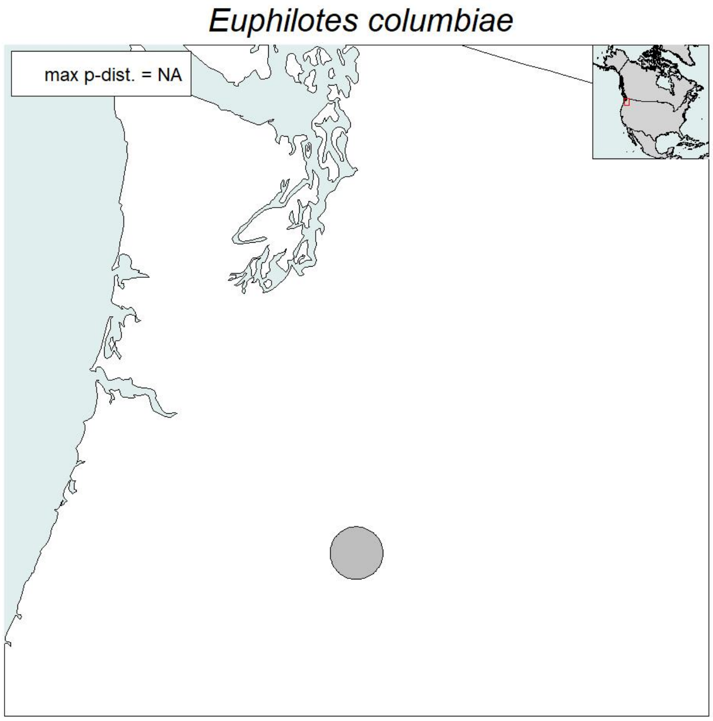

Figure 387: Map of *Euphilotes columbiae* showing the localities of the sequenced specimens. Nearby localities are grouped in pies. Due to the presence of a single haplotype PCoA projection was not done and a single grey colour was plotted on the map. Sequences= 1; Hap obs.= NA; Hap asympt.= NA; Hap % obs.= NA; GST= NaN; DST= NaN; HD= NA; ND= NA; max p-dist= NA.

Haplotype network analysis and bubble plot of *Euphilotes columbiae* were not possible. Sequences > 599 bp = 1.

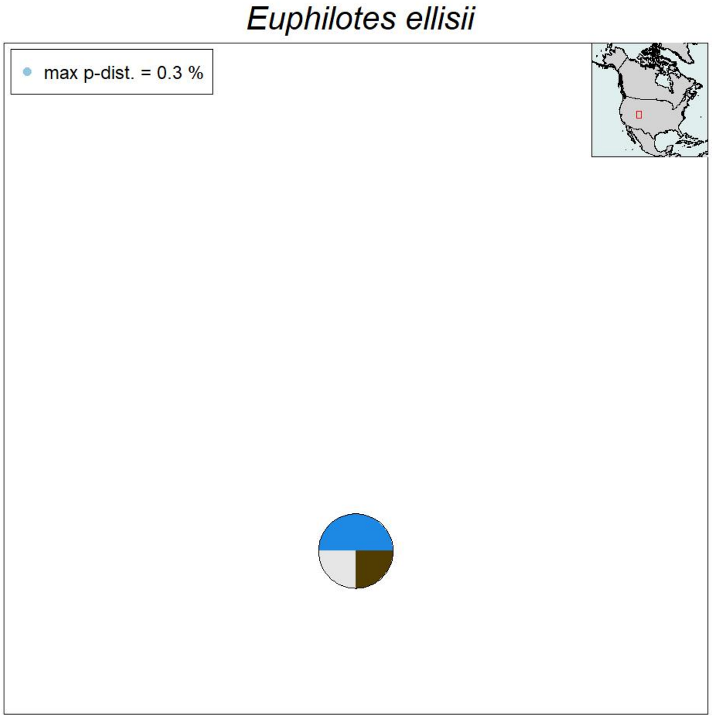

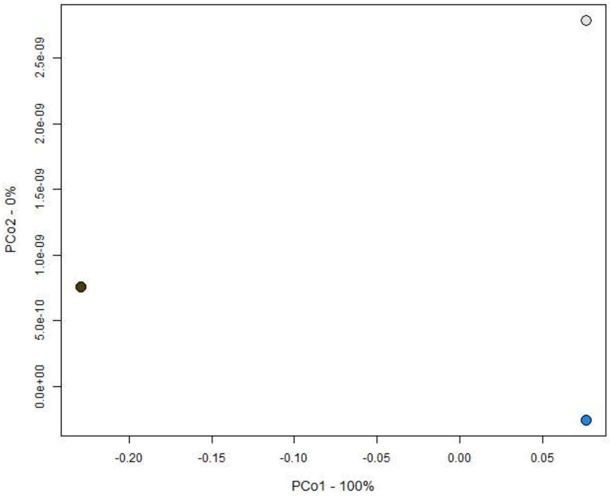

Figure 388 Map of *Euphilotes ellisii* showing the localities of the sequenced specimens (left). Nearby localities are grouped in pies. Colours match the bidimensional colour space of the PCoA projection (right) of max p-dists among sequences (dots). Sequences= 4; Hap obs.= 2; Hap asympt.= NA; Hap % obs.= NA%; GST= NaN; DST= NaN; HD= NA; ND= NA; max p-dist= 0.3%.

Haplotype network analysis and bubble plot of *Euphilotes ellisii* were not possible. Sequences > 599 bp = 4.

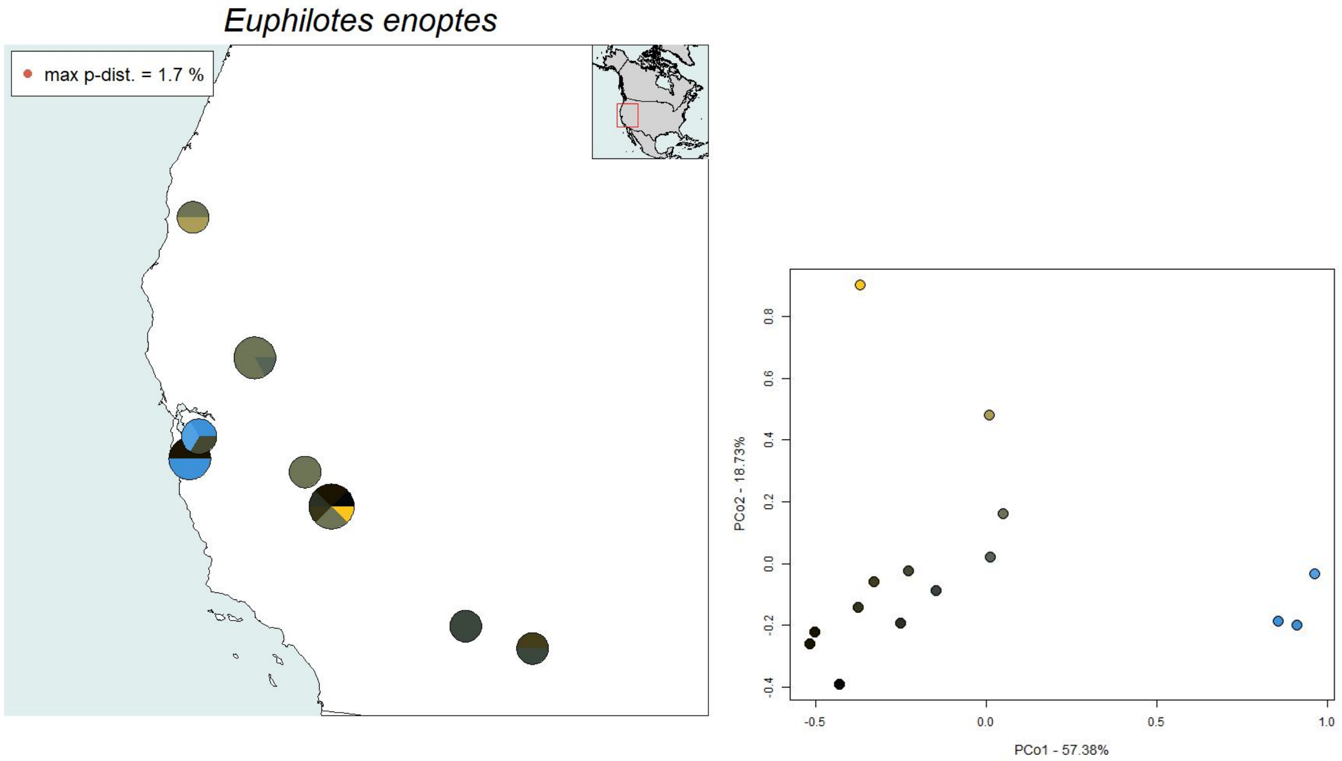

Figure 389 Map of *Euphilotes enoptes* showing the localities of the sequenced specimens (left). Nearby localities are grouped in pies. Colours match the bidimensional colour space of the PCoA projection (right) of max p-dists among sequences (dots). Sequences= 31; Hap obs.= 13; Hap asympt.= 44; Hap % obs.= 29.6%; GST= 0.261; DST= 0.0019; HD= 0.867; ND= 0.007; max p-dist= 1.7%.

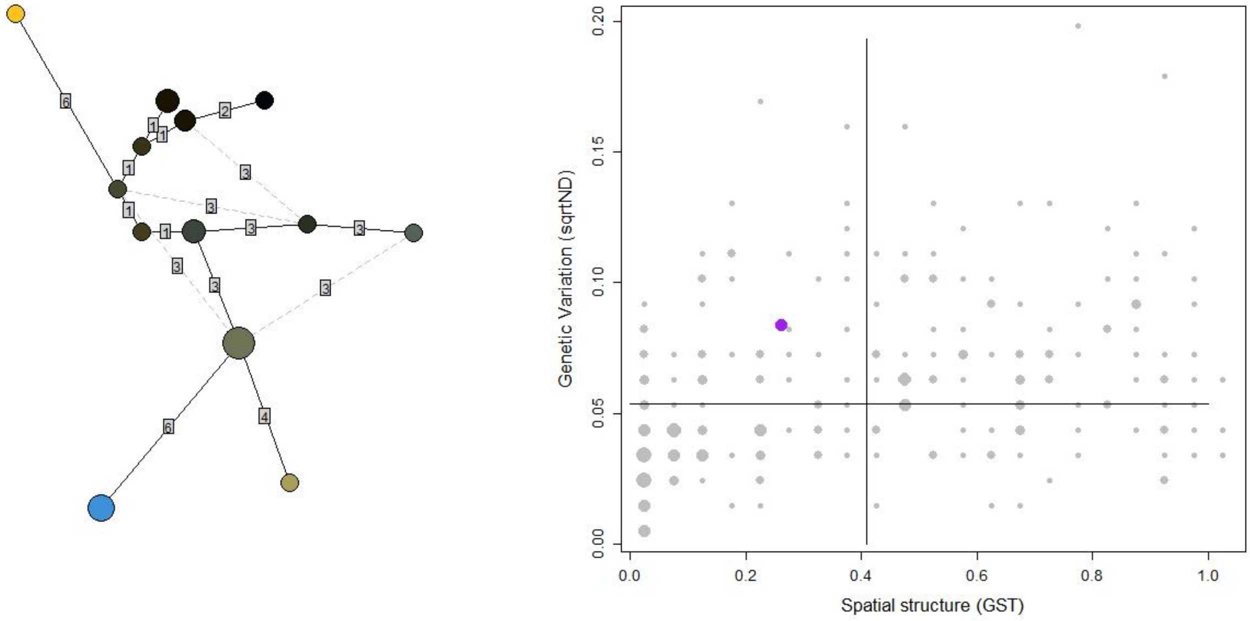

Figure 390: Haplotype network (left) of *Euphilotes enoptes* sequences > 599 bp with colours matching the PCoA colour space (above). The bubble plot for mt-DNA polymorphism (square root transformed nucleotide diversity) and spatial structure (GST) among all species in the atlas and values for *Euphilotes enoptes* (purple dot). The horizontal and vertical lines represent median values of nucleotide diversity and GST, respectively. Sequences > 599 bp= 31.

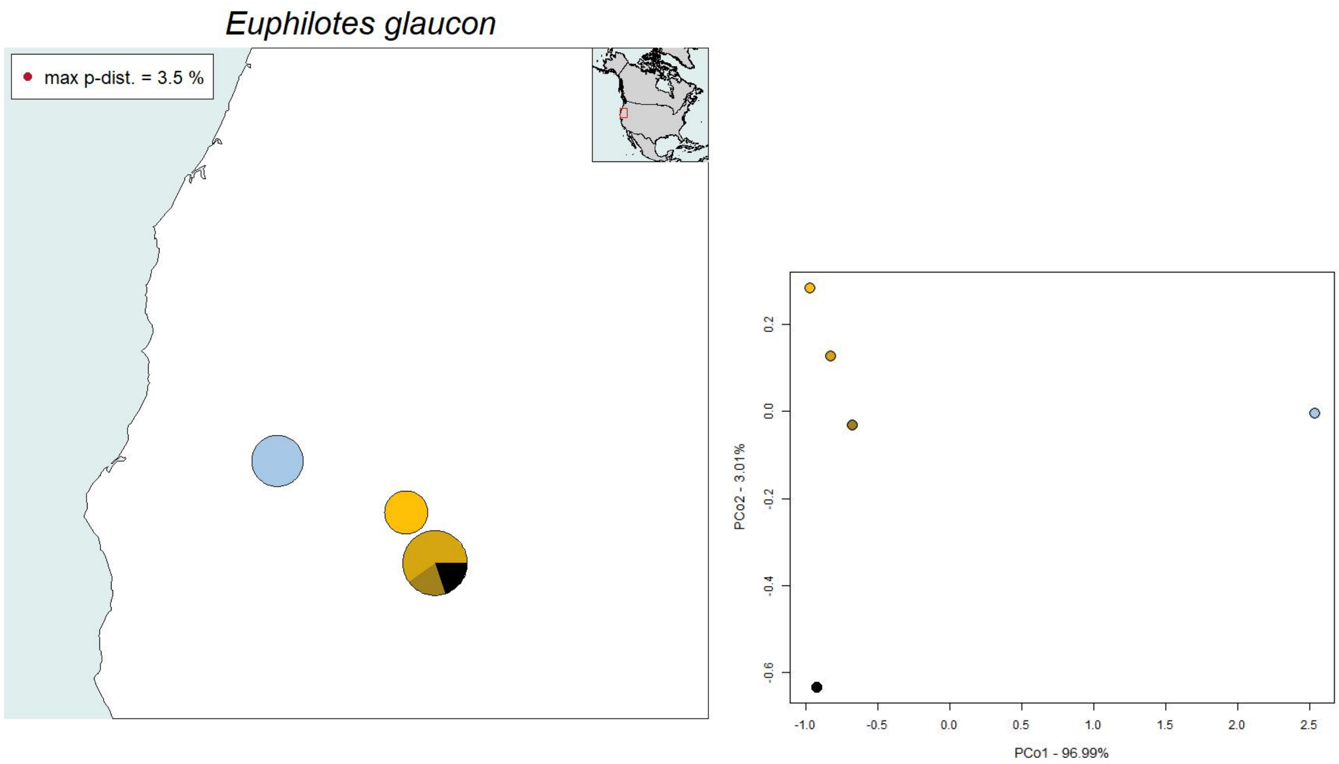

Figure 391 Map of *Euphilotes glaucon* showing the localities of the sequenced specimens (left). Nearby localities are grouped in pies. Colours match the bidimensional colour space of the PCoA projection (right) of max p-dists among sequences (dots). Sequences= 8; Hap obs.= 5; Hap asympt.= NA; Hap % obs.= NA%; GST= NaN; DST= NaN; HD= NA; ND= NA; max p-dist= 3.5%.

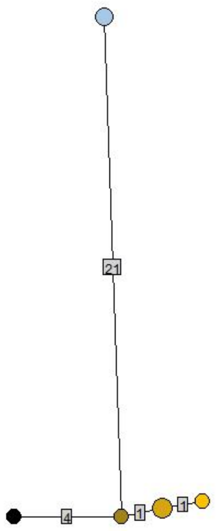

Figure 392: Haplotype network of *Euphilotes glaucon*. Sequences > 599 bp= 8.

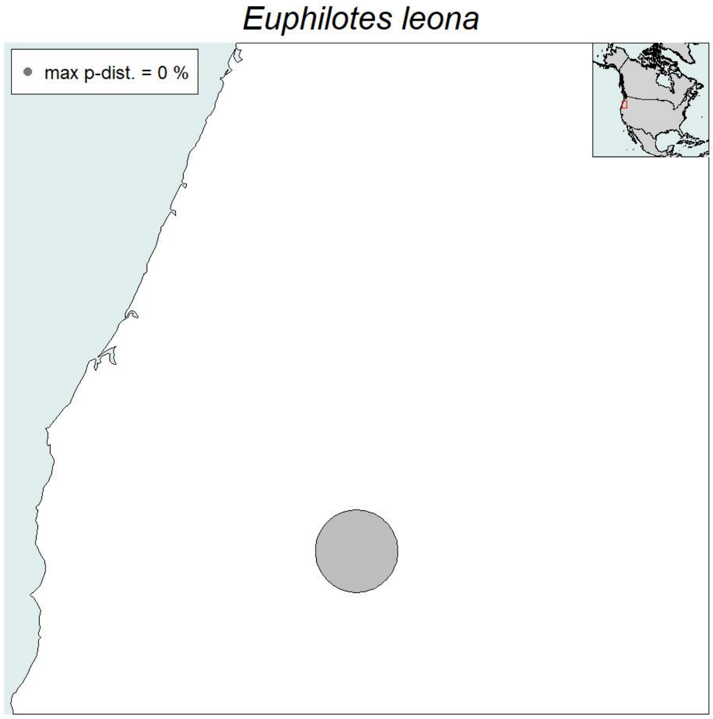

Figure 393: Map of *Euphilotes leona* showing the localities of the sequenced specimens. Nearby localities are grouped in pies. Due to the presence of a single haplotype PCoA projection was not done and a single grey colour was plotted on the map. Sequences= 6; Hap obs.= 1; Hap asympt.= NA; Hap % obs.= NA%; GST= NaN; DST= NaN; HD= NA; ND= NA; max p-dist= 0%.

Haplotype network analysis and bubble plot of *Euphilotes leona* were not possible. Sequences > 599 bp = 6.

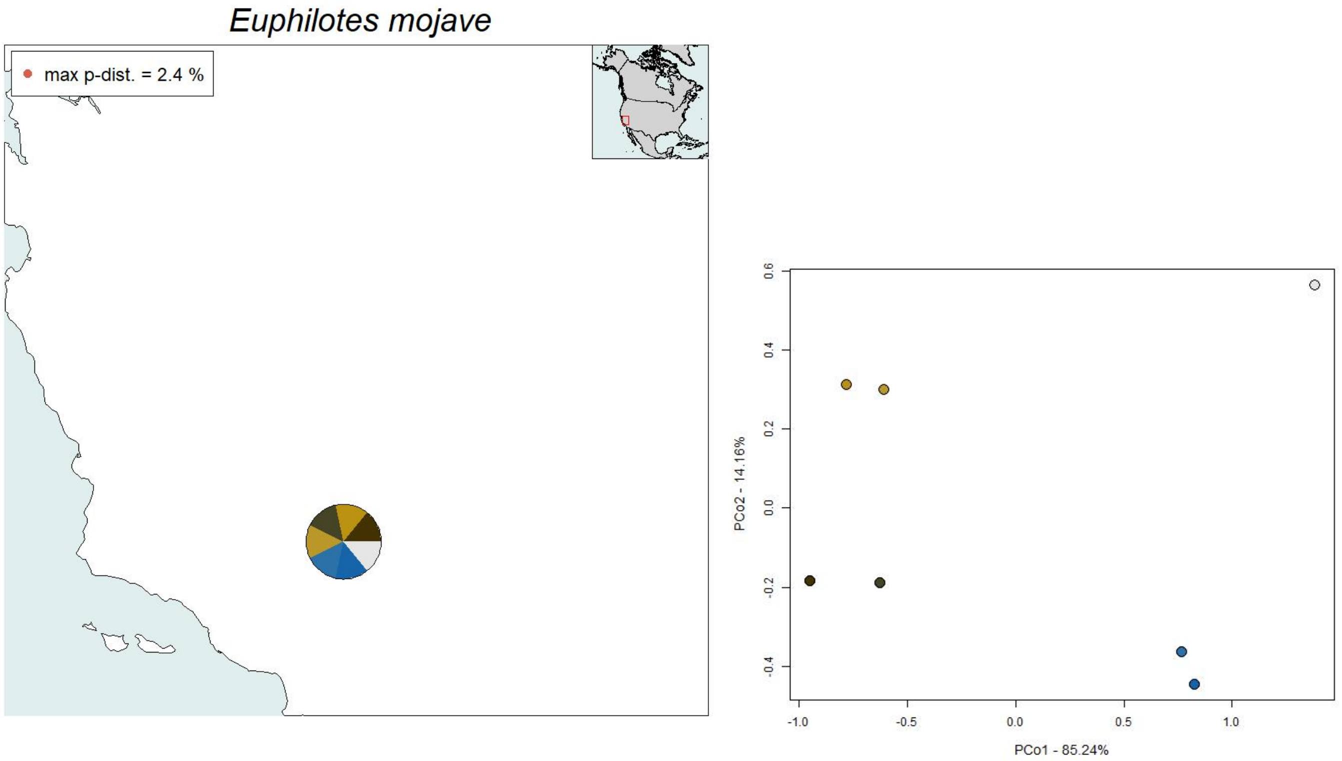

Figure 394 Map of *Euphilotes mojave* showing the localities of the sequenced specimens (left). Nearby localities are grouped in pies. Colours match the bidimensional colour space of the PCoA projection (right) of max p-dists among sequences (dots). Sequences= 7; Hap obs.= 6; Hap asympt.= NA; Hap % obs.= NA%; GST= NaN; DST= NaN; HD= NA; ND= NA; max p-dist= 2.4%.

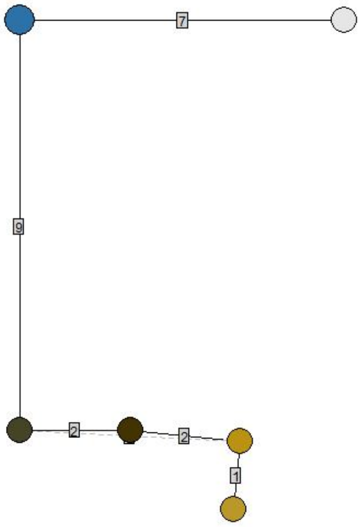

Figure 395: Haplotype network of *Euphilotes mojave*. Sequences > 599 bp= 7.

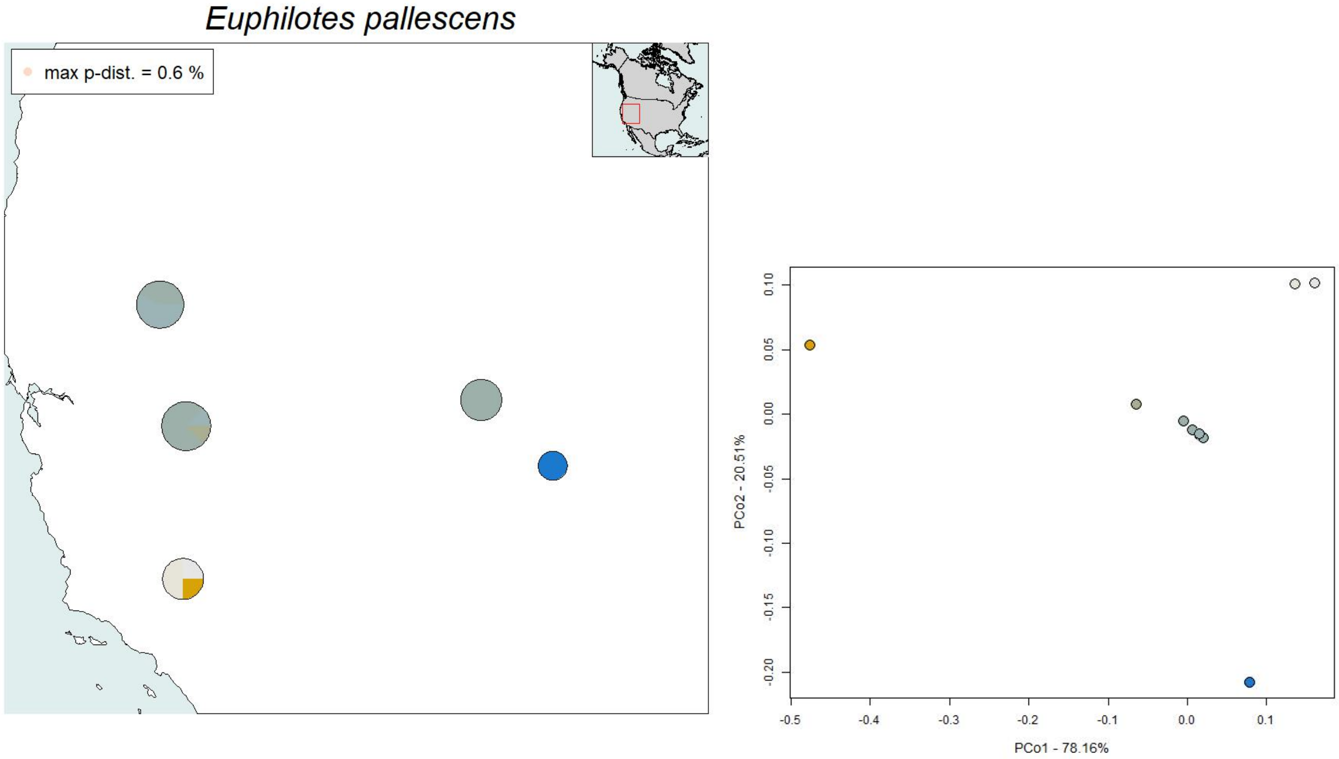

Figure 396 Map of *Euphilotes pallescens* showing the localities of the sequenced specimens (left). Nearby localities are grouped in pies. Colours match the bidimensional colour space of the PCoA projection (right) of max p-dists among sequences (dots). Sequences= 24; Hap obs.= 4; Hap asympt.= 5; Hap % obs.= 80.7%; GST= 0.614; DST= 0.0005; HD= 0.37; ND= 0.0009; max p-dist= 0.6%.

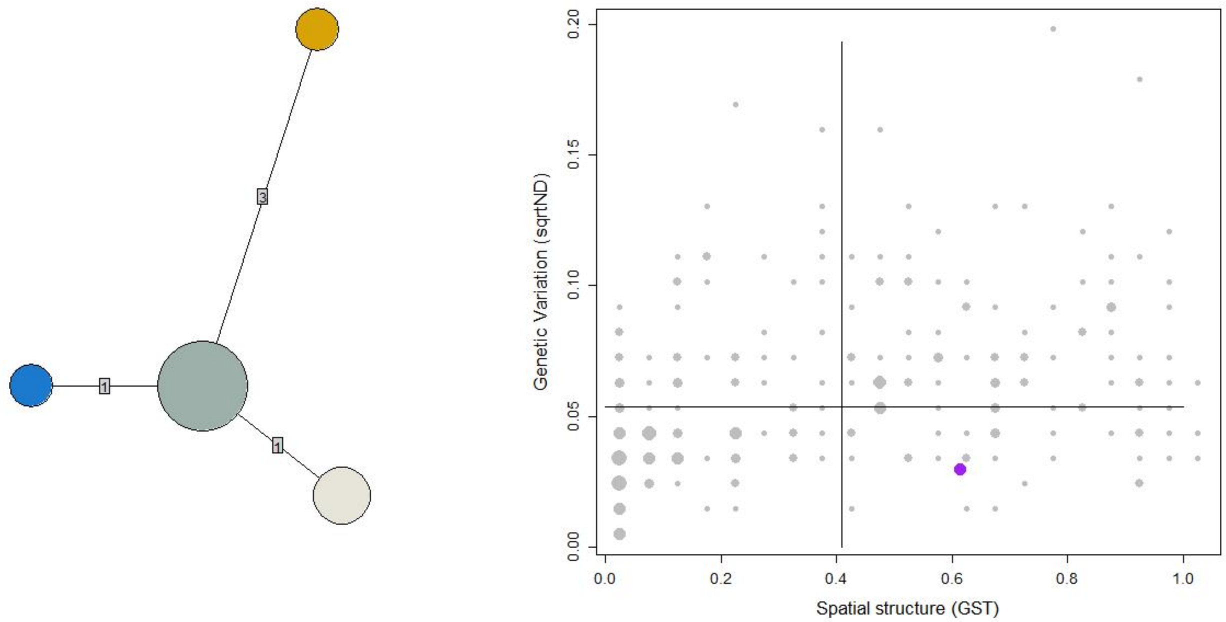

Figure 397: Haplotype network (left) of *Euphilotes pallescens* sequences > 599 bp with colours matching the PCoA colour space (above). The bubble plot for mt-DNA polymorphism (square root transformed nucleotide diversity) and spatial structure (GST) among all species in the atlas and values for *Euphilotes pallescens* (purple dot). The horizontal and vertical lines represent median values of nucleotide diversity and GST, respectively. Sequences > 599 bp= 23.

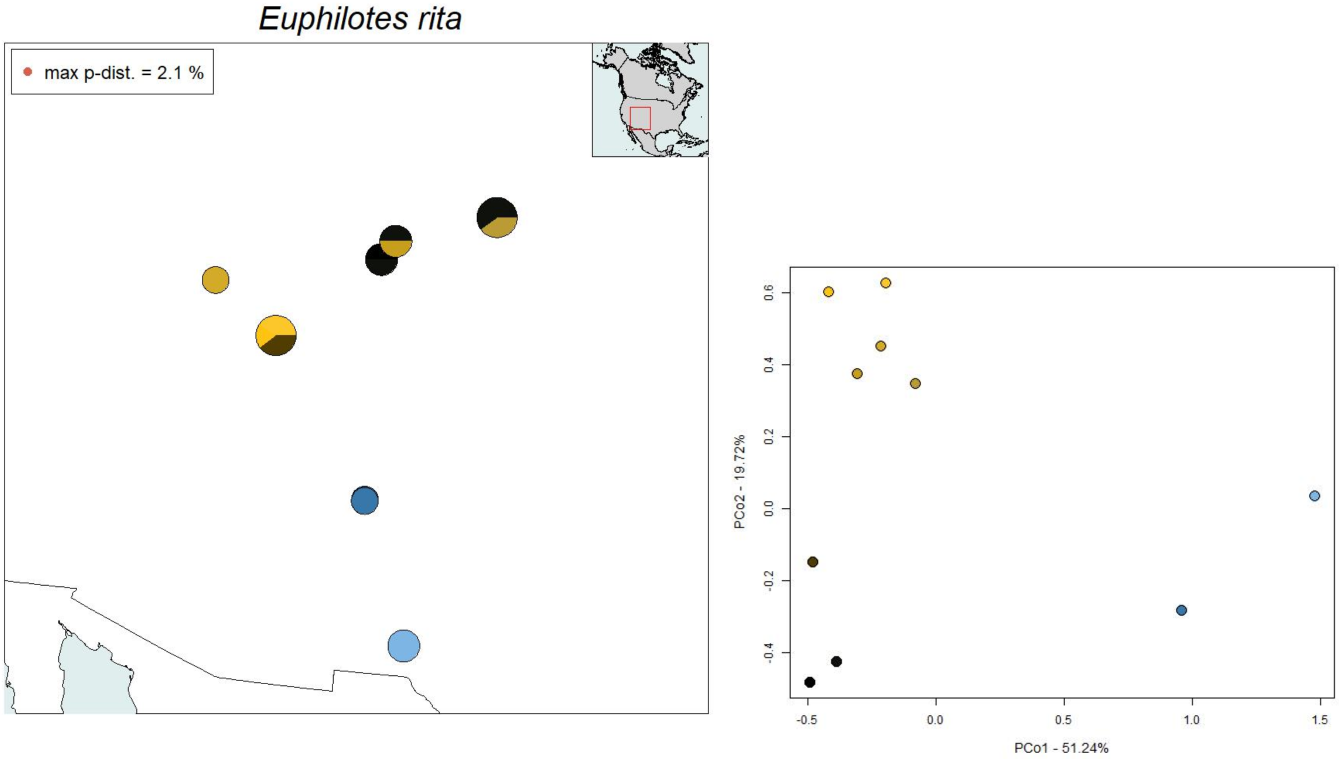

Figure 398 Map of *Euphilotes rita* showing the localities of the sequenced specimens (left). Nearby localities are grouped in pies. Colours match the bidimensional colour space of the PCoA projection (right) of max p-dists among sequences (dots). Sequences= 19; Hap obs.= 9; Hap asympt.= 9.9; Hap % obs.= 91.3%; GST= 0.174; DST= 0.0015; HD= 0.883; ND= 0.0115; max p-dist= 2.1%.

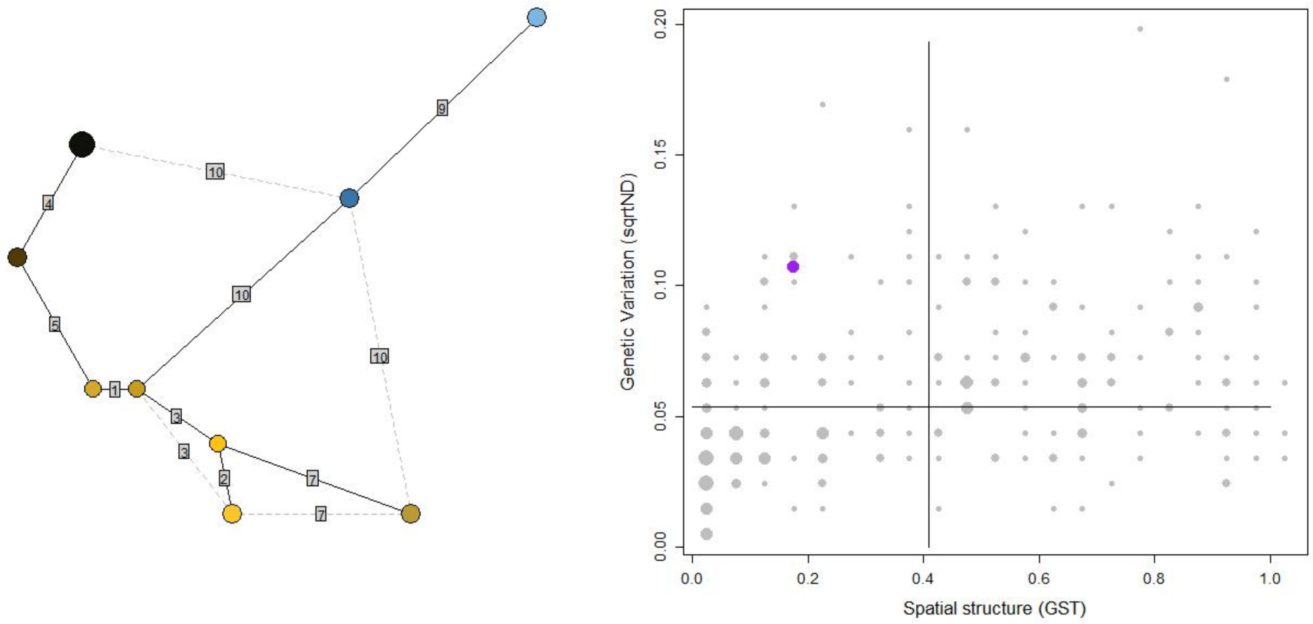

Figure 399: Haplotype network (left) of *Euphilotes rita* sequences > 599 bp with colours matching the PCoA colour space (above). The bubble plot for mt-DNA polymorphism (square root transformed nucleotide diversity) and spatial structure (GST) among all species in the atlas and values for *Euphilotes rita* (purple dot). The horizontal and vertical lines represent median values of nucleotide diversity and GST, respectively. Sequences > 599 bp= 19.

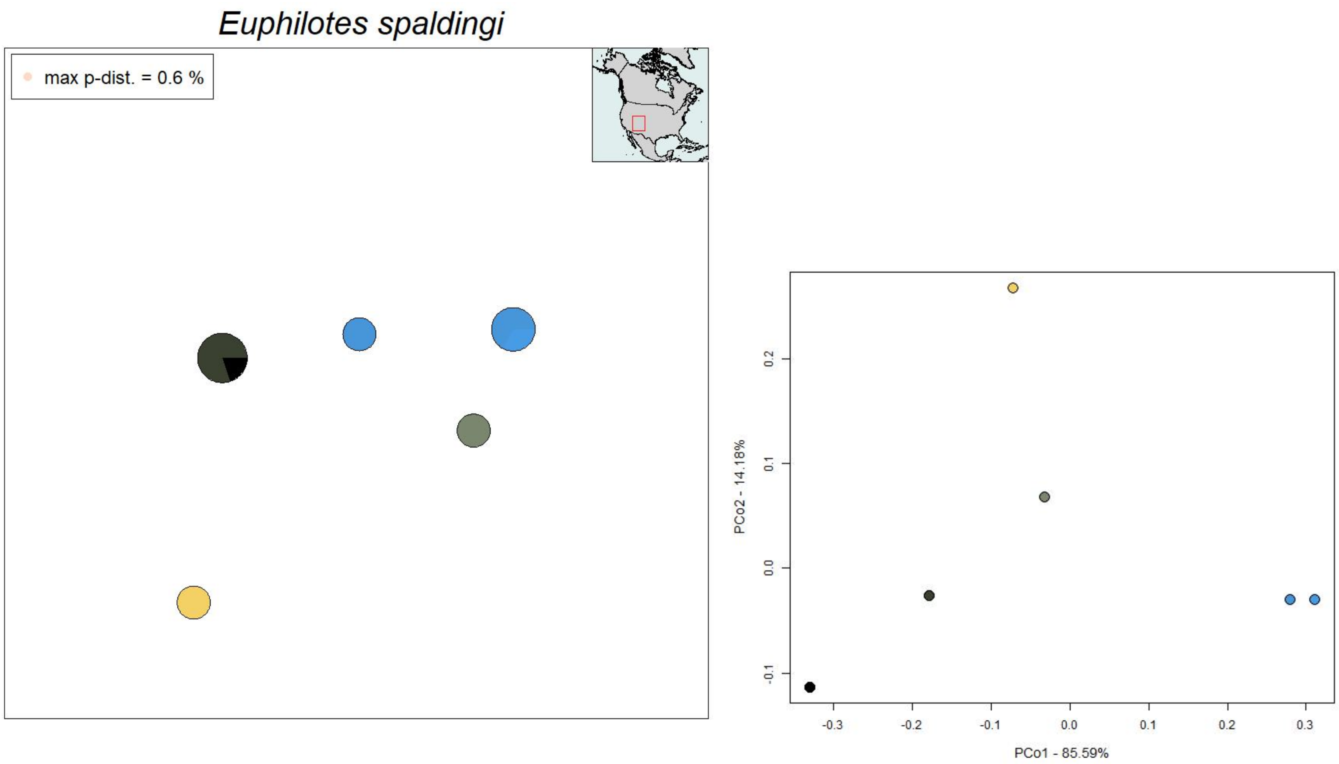

Figure 400 Map of *Euphilotes spaldingi* showing the localities of the sequenced specimens (left). Nearby localities are grouped in pies. Colours match the bidimensional colour space of the PCoA projection (right) of max p-dists among sequences (dots). Sequences= 11; Hap obs.= 5; Hap asympt.= 7.7; Hap % obs.= 64.7%; GST= 0.495; DST= 0.0014; HD= 0.782; ND= 0.003; max p-dist= 0.6%.

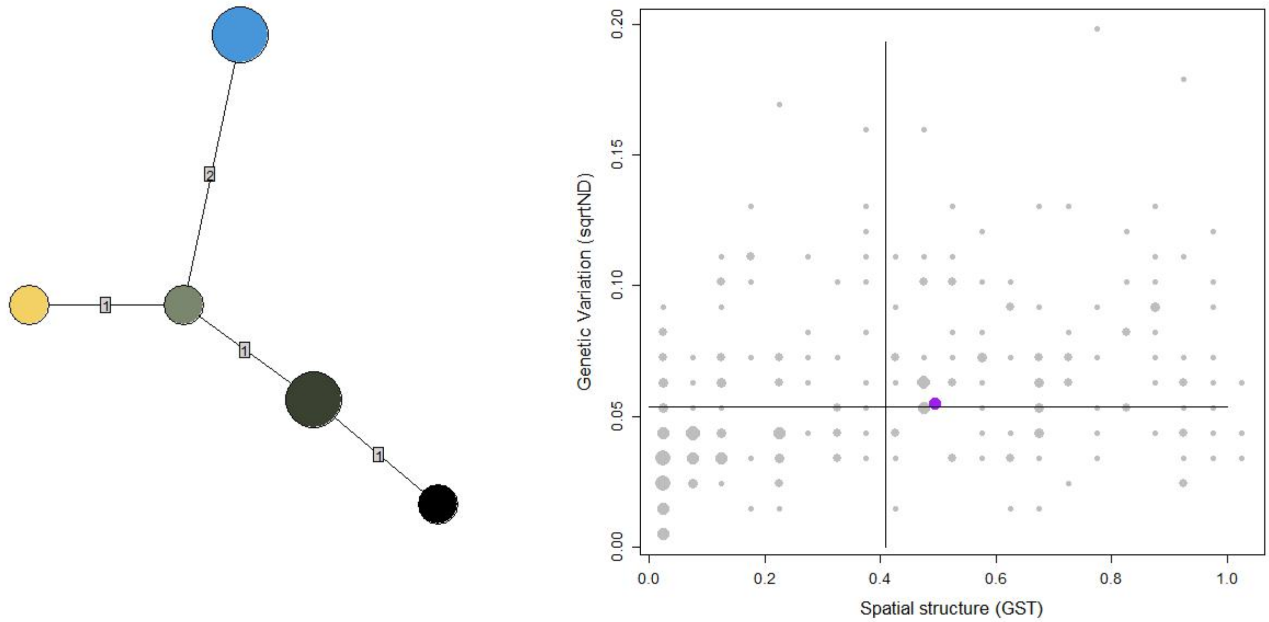

Figure 401: Haplotype network (left) of *Euphilotes spaldingi* sequences > 599 bp with colours matching the PCoA colour space (above). The bubble plot for mt-DNA polymorphism (square root transformed nucleotide diversity) and spatial structure (GST) among all species in the atlas and values for *Euphilotes spaldingi* (purple dot). The horizontal and vertical lines represent median values of nucleotide diversity and GST, respectively. Sequences > 599 bp= 11.

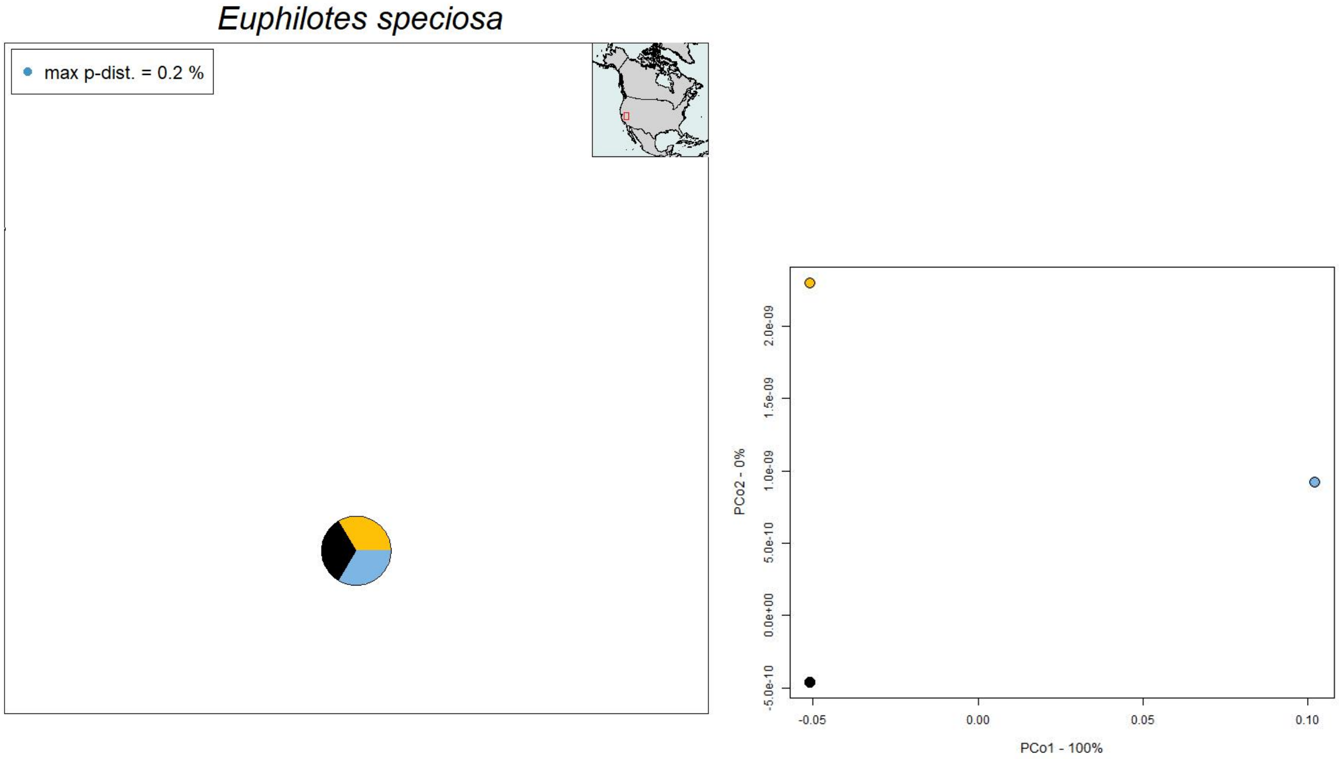

Figure 402 Map of *Euphilotes speciosa* showing the localities of the sequenced specimens (left). Nearby localities are grouped in pies. Colours match the bidimensional colour space of the PCoA projection (right) of max p-dists among sequences (dots). Sequences= 3; Hap obs.= 2; Hap asympt.= NA; Hap % obs.= NA%; GST= NaN; DST= NaN; HD= NA; ND= NA; max p-dist= 0.2%.

Haplotype network analysis and bubble plot of *Euphilotes speciosa* were not possible. Sequences > 599 bp = 3.

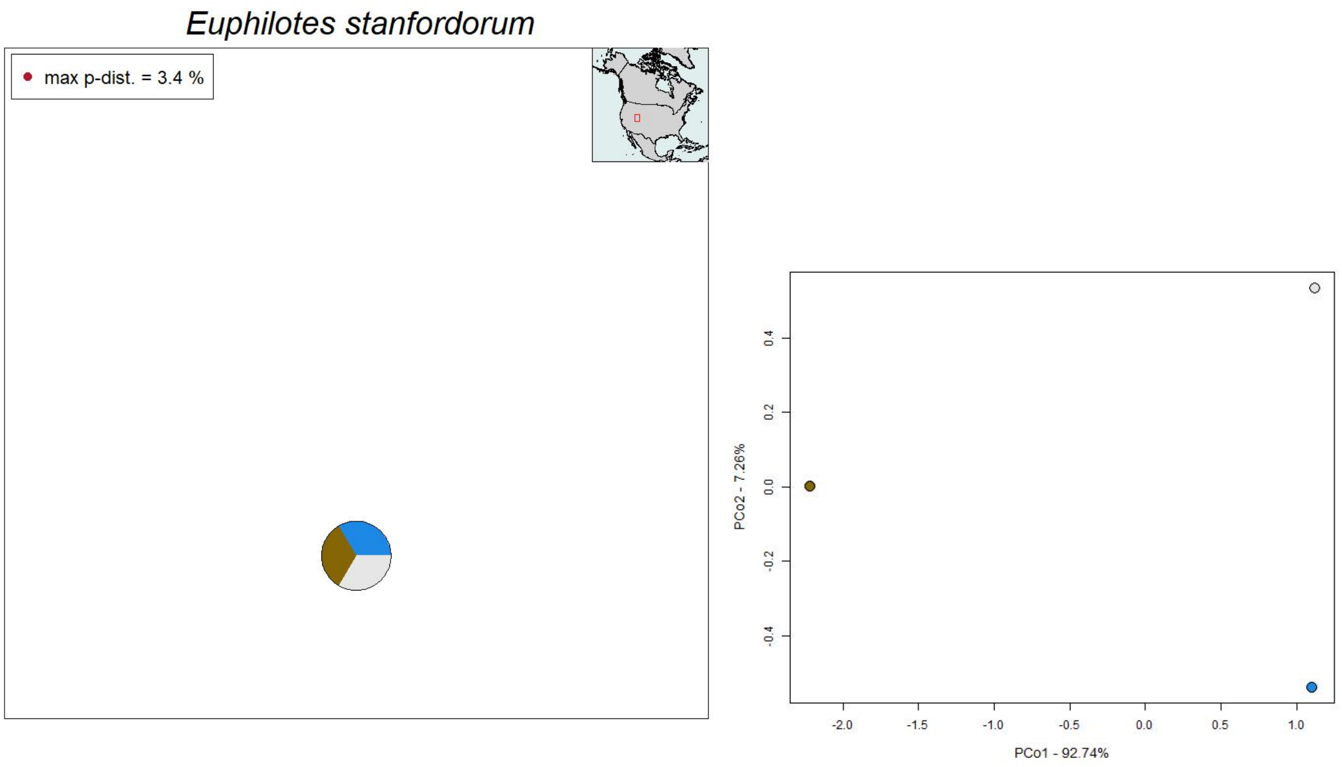

Figure 403 Map of *Euphilotes stanfordorum* showing the localities of the sequenced specimens (left). Nearby localities are grouped in pies. Colours match the bidimensional colour space of the PCoA projection (right) of max p-dists among sequences (dots). Sequences= 3; Hap obs.= 3; Hap asympt.= NA; Hap % obs.= NA%; GST= NaN; DST= NaN; HD= NA; ND= NA; max p-dist= 3.4%.

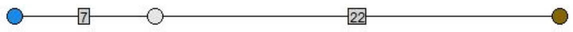

Figure 404: Haplotype network of *Euphilotes stanfordorum*. Sequences > 599 bp= 3.

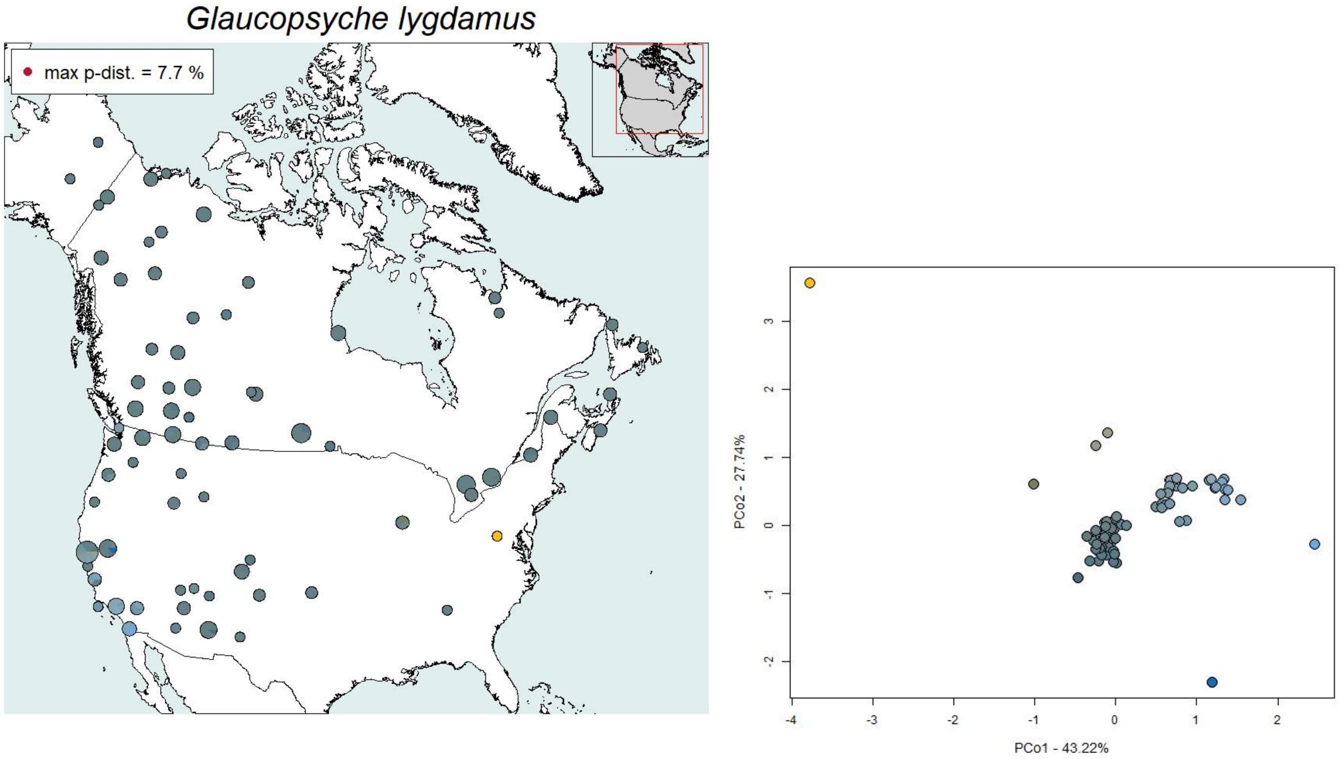

Figure 405 Map of *Glaucopsyche lygdamus* showing the localities of the sequenced specimens (left). Nearby localities are grouped in pies. Colours match the bidimensional colour space of the PCoA projection (right) of max p-dists among sequences (dots). Sequences= 250; Hap obs.= 94; Hap asympt.= 599; Hap % obs.= 15.7%; GST= 0.274; DST= 0.0013; HD= 0.775; ND= 0.0049; max p-dist= 7.7%.

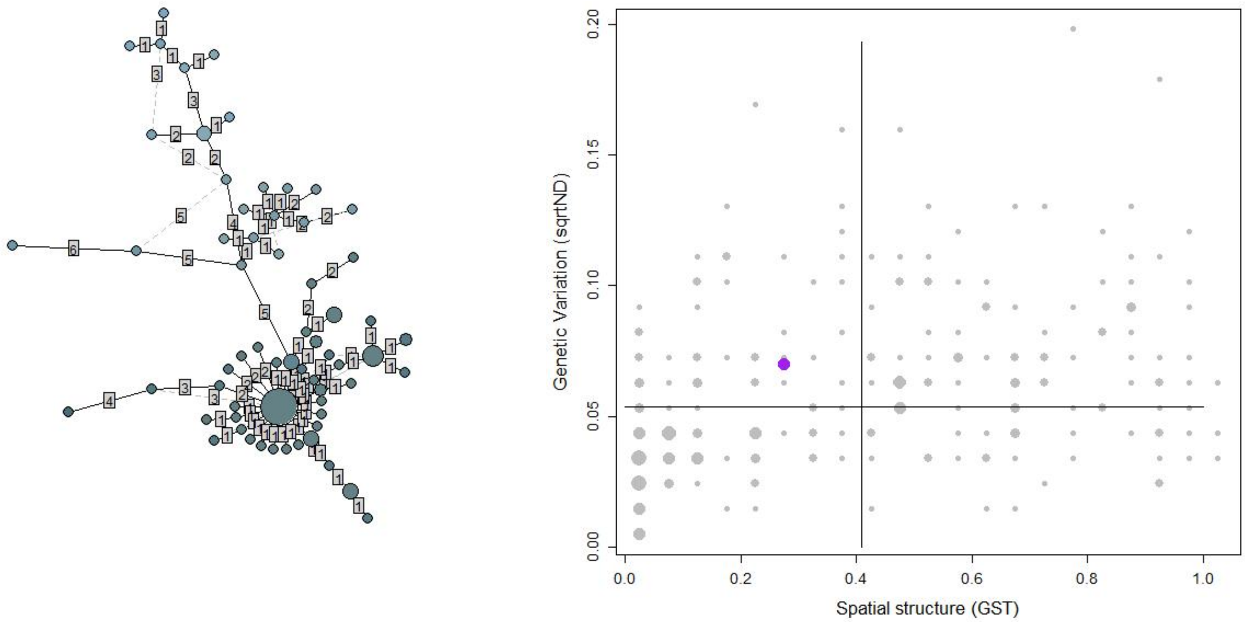

Figure 406: Haplotype network (left) of *Glaucopsyche lygdamus* sequences > 599 bp with colours matching the PCoA colour space (above). The bubble plot for mt-DNA polymorphism (square root transformed nucleotide diversity) and spatial structure (GST) among all species in the atlas and values for *Glaucopsyche lygdamus* (purple dot). The horizontal and vertical lines represent median values of nucleotide diversity and GST, respectively. Sequences > 599 bp= 222.

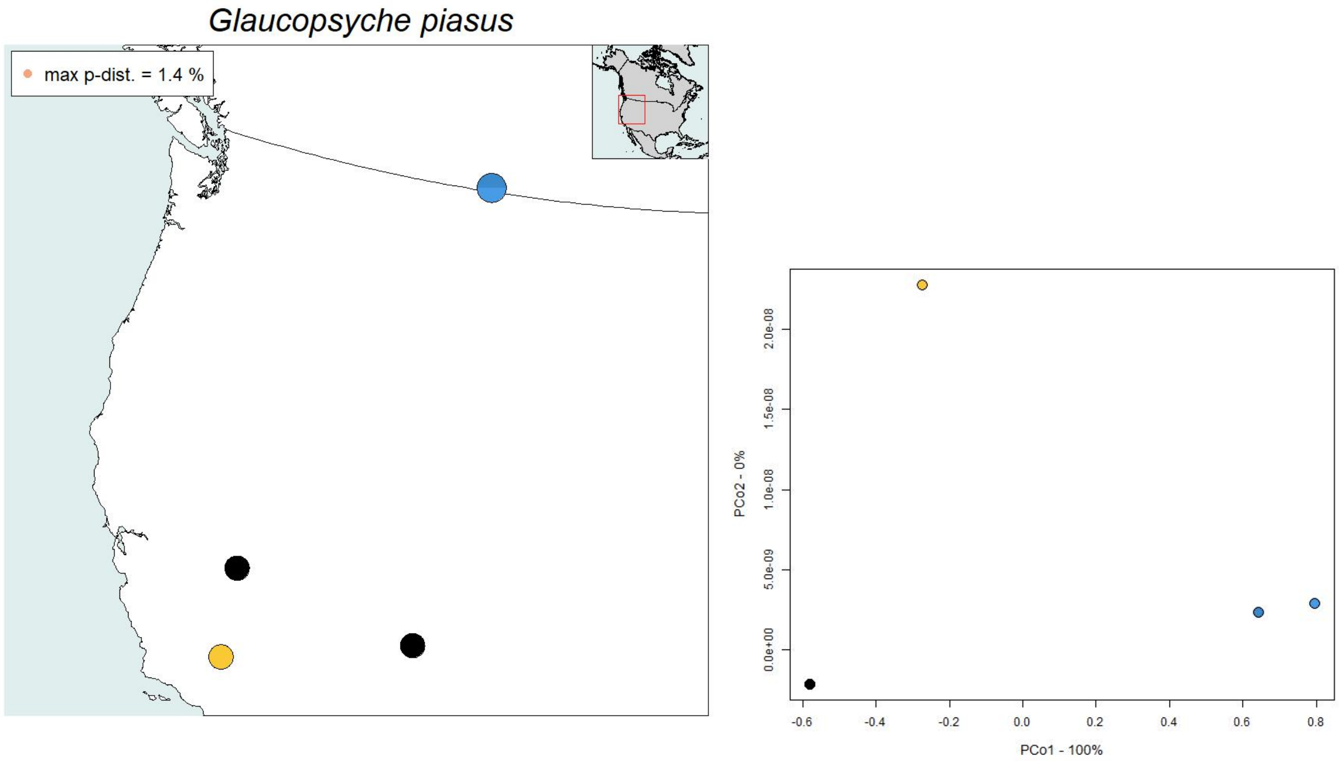

Figure 407 Map of *Glaucopsyche piasus* showing the localities of the sequenced specimens (left). Nearby localities are grouped in pies. Colours match the bidimensional colour space of the PCoA projection (right) of max p-dists among sequences (dots). Sequences= 5; Hap obs.= 4; Hap asympt.= NA; Hap % obs.= NA%; GST= NaN; DST= NaN; HD= NA; ND= NA; max p-dist= 1.4%.

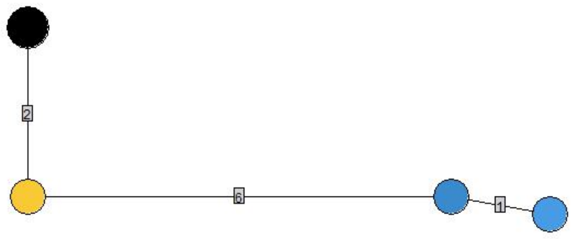

Figure 408: Haplotype network of *Glaucopsyche piasus*. Sequences > 599 bp= 5.

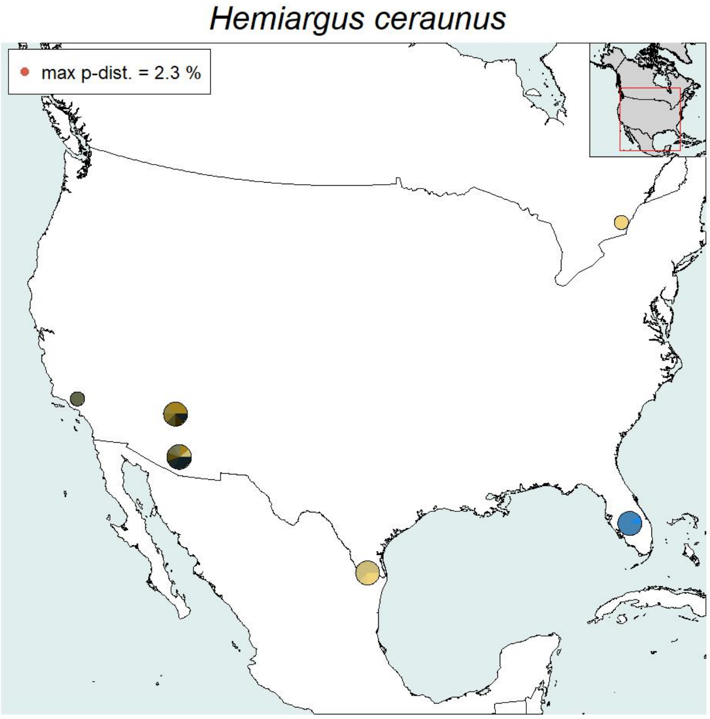

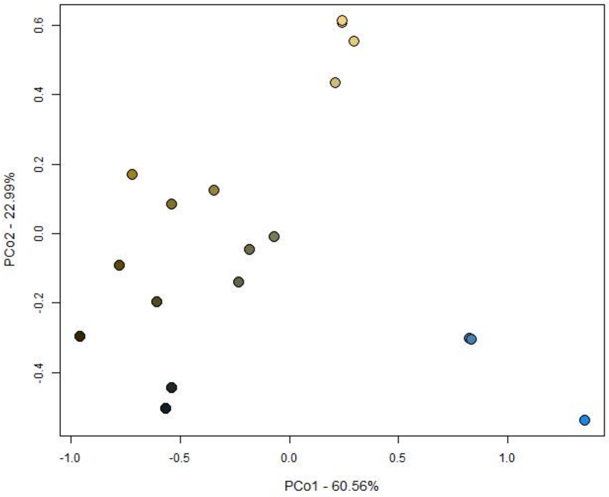

Figure 409 Map of *Hemiargus ceraunus* showing the localities of the sequenced specimens (left). Nearby localities are grouped in pies. Colours match the bidimensional colour space of the PCoA projection (right) of max p-dists among sequences (dots). Sequences= 35; Hap obs.= 15; Hap asympt.= 34.7; Hap % obs.= 43.3%; GST= 0.576; DST= 0.0056; HD= 0.909; ND= 0.0095; max p-dist= 2.3%.

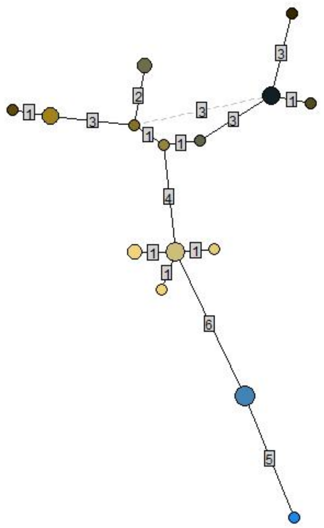

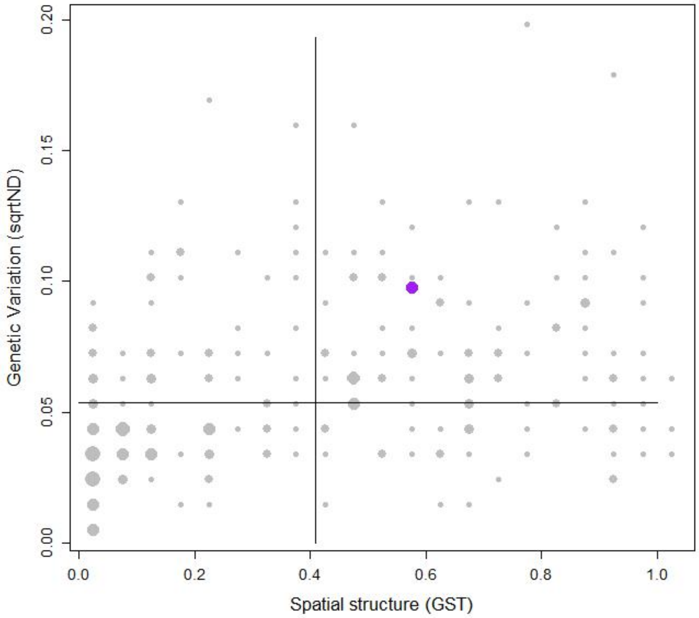

Figure 410: Haplotype network (left) of *Hemiargus ceraunus* sequences > 599 bp with colours matching the PCoA colour space (above). The bubble plot for mt-DNA polymorphism (square root transformed nucleotide diversity) and spatial structure (GST) among all species in the atlas and values for *Hemiargus ceraunus* (purple dot). The horizontal and vertical lines represent median values of nucleotide diversity and GST, respectively. Sequences > 599 bp= 35.

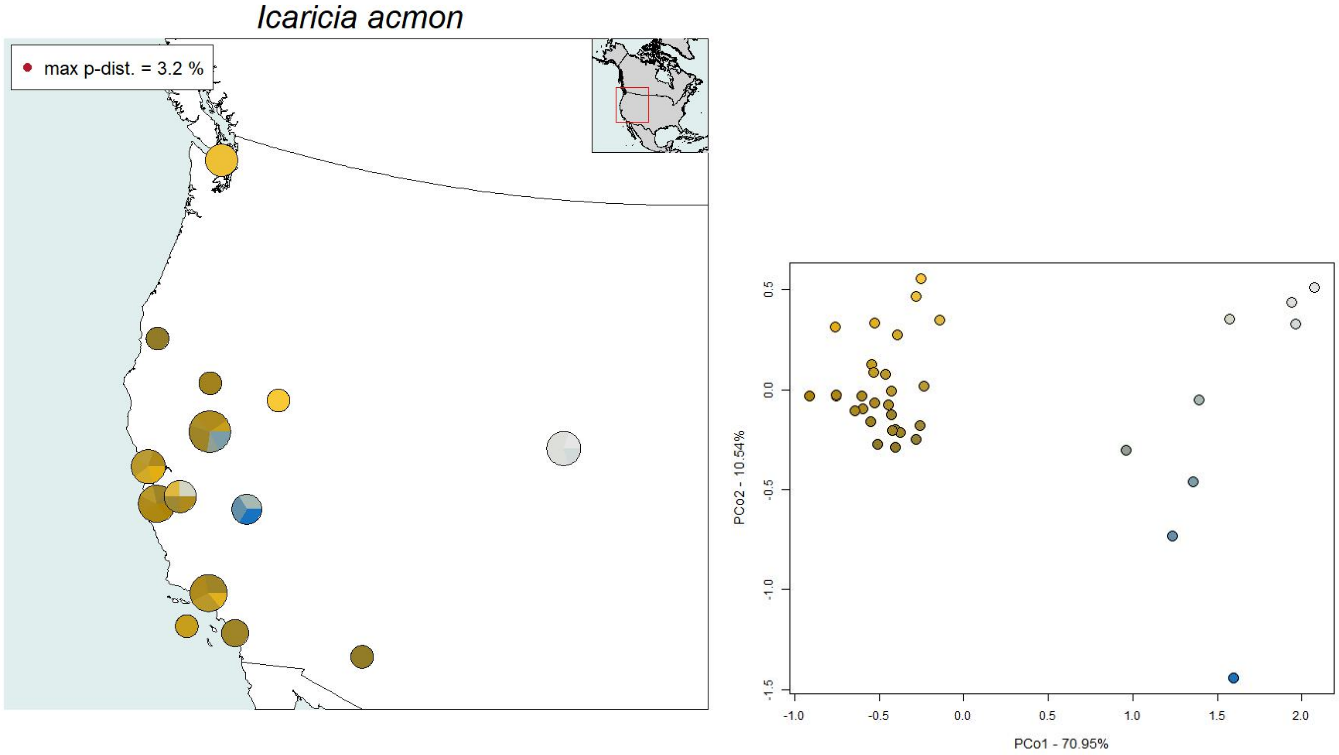

Figure 411 Map of *Icaricia acmon* showing the localities of the sequenced specimens (left). Nearby localities are grouped in pies. Colours match the bidimensional colour space of the PCoA projection (right) of max p-dists among sequences (dots). Sequences= 53; Hap obs.= 31; Hap asympt.= 172.3; Hap % obs.= 18%; GST= 0.497; DST= 0.0058; HD= 0.959; ND= 0.0109; max p-dist= 3.2%.

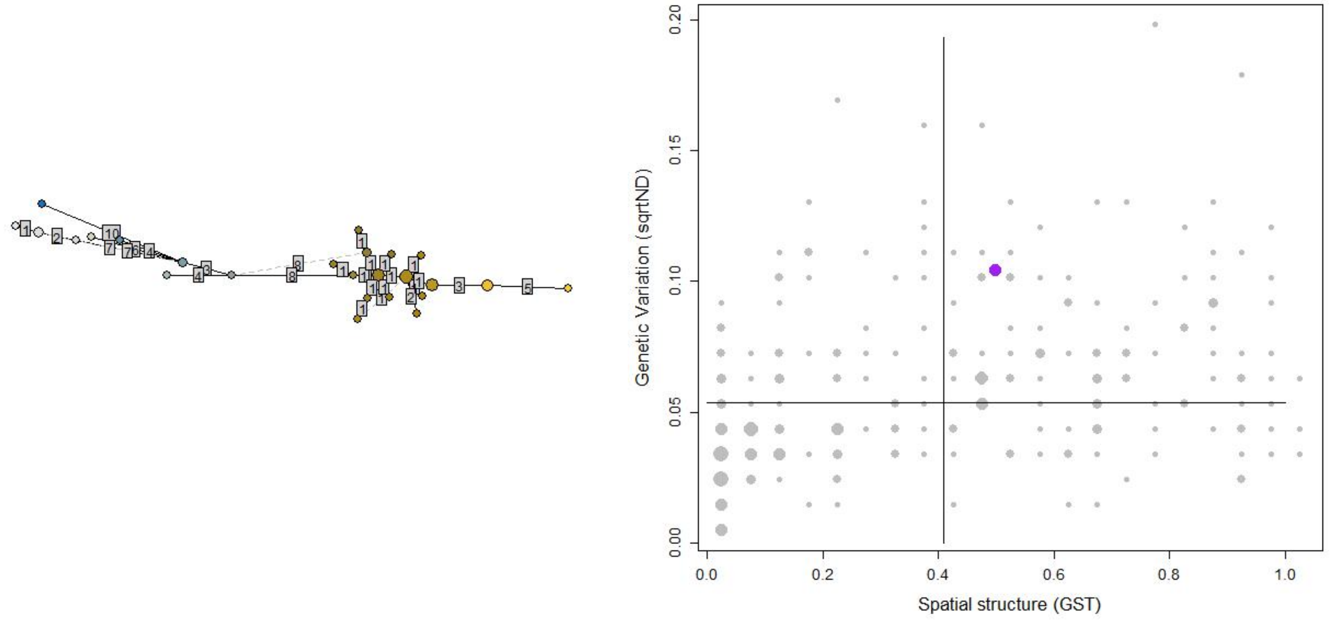

Figure 412: Haplotype network (left) of *Icaricia acmon* sequences > 599 bp with colours matching the PCoA colour space (above). The bubble plot for mt-DNA polymorphism (square root transformed nucleotide diversity) and spatial structure (GST) among all species in the atlas and values for *Icaricia acmon* (purple dot). The horizontal and vertical lines represent median values of nucleotide diversity and GST, respectively. Sequences > 599 bp= 49.

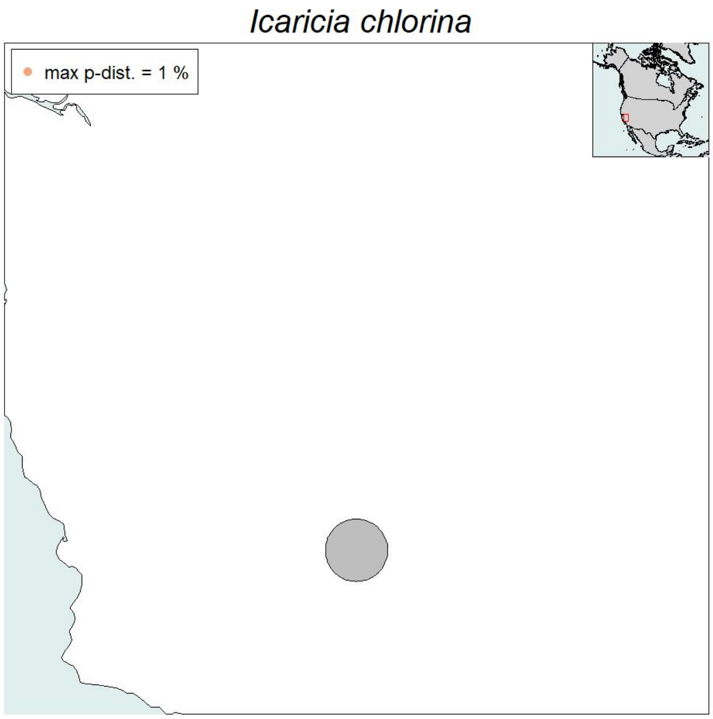

Figure 413: Map of *Icaricia chlorina* showing the localities of the sequenced specimens. Nearby localities are grouped in pies. Due to the presence of a single haplotype PCoA projection was not done and a single grey colour was plotted on the map. Sequences= 2; Hap obs.= 2; Hap asympt.= NA; Hap % obs.= NA%; GST= NaN; DST= NaN; HD= NA; ND= NA; max p-dist= 1%.

Haplotype network analysis and bubble plot of *Icaricia chlorina* were not possible. Sequences > 599 bp = 2.

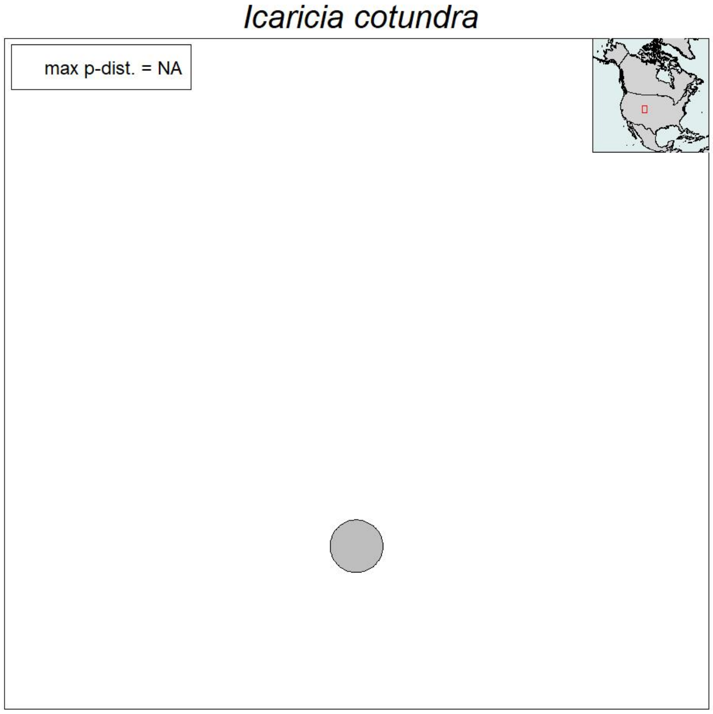

Figure 414: Map of *Icaricia cotundra* showing the localities of the sequenced specimens. Nearby localities are grouped in pies. Due to the presence of a single haplotype PCoA projection was not done and a single grey colour was plotted on the map. Sequences= 1; Hap obs.= NA; Hap asympt.= NA; Hap % obs.= NA; GST= NaN; DST= NaN; HD= NA; ND= NA; max p-dist= NA.

Haplotype network analysis and bubble plot of *Icaricia cotundra* were not possible. Sequences > 599 bp = 1.

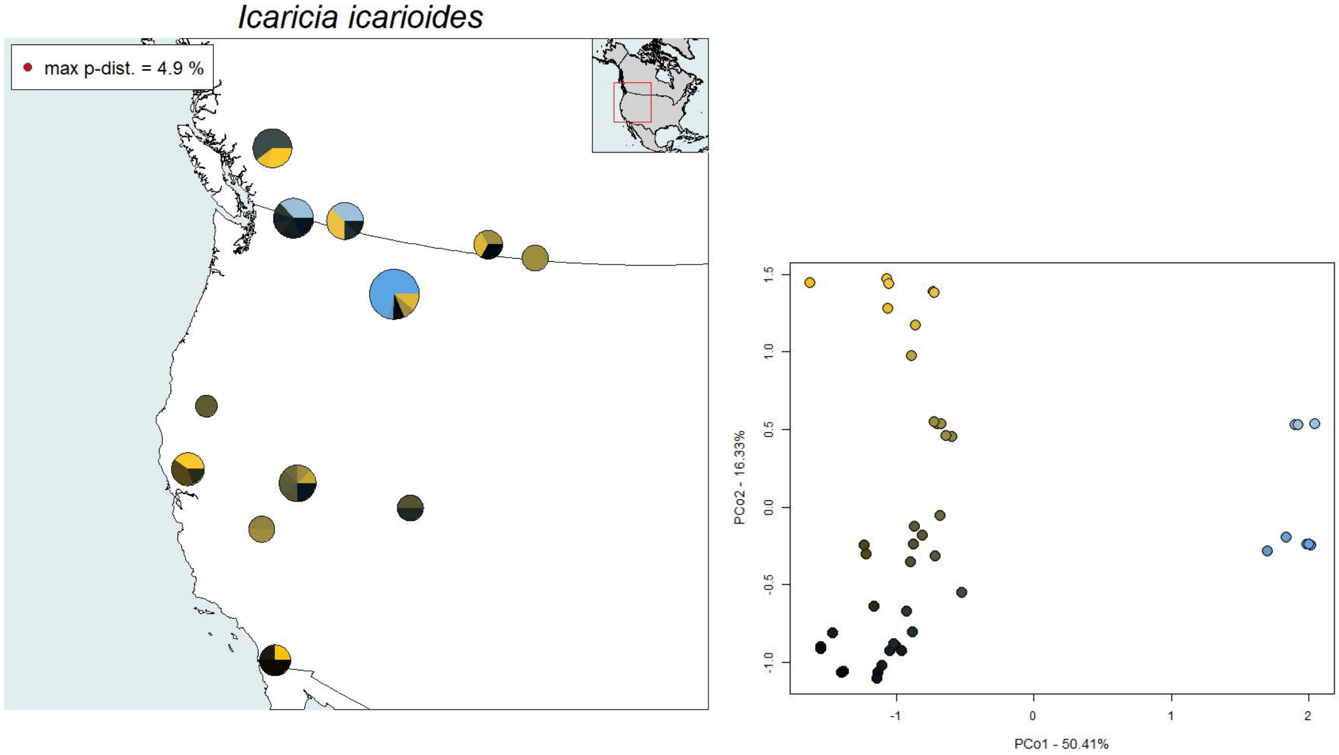

Figure 415 Map of *Icaricia icarioides* showing the localities of the sequenced specimens (left). Nearby localities are grouped in pies. Colours match the bidimensional colour space of the PCoA projection (right) of max p-dists among sequences (dots). Sequences= 83; Hap obs.= 41; Hap asympt.= 81; Hap % obs.= 50.6%; GST= 0.498; DST= 0.0122; HD= 0.94; ND= 0.0243; max p-dist= 4.9%.

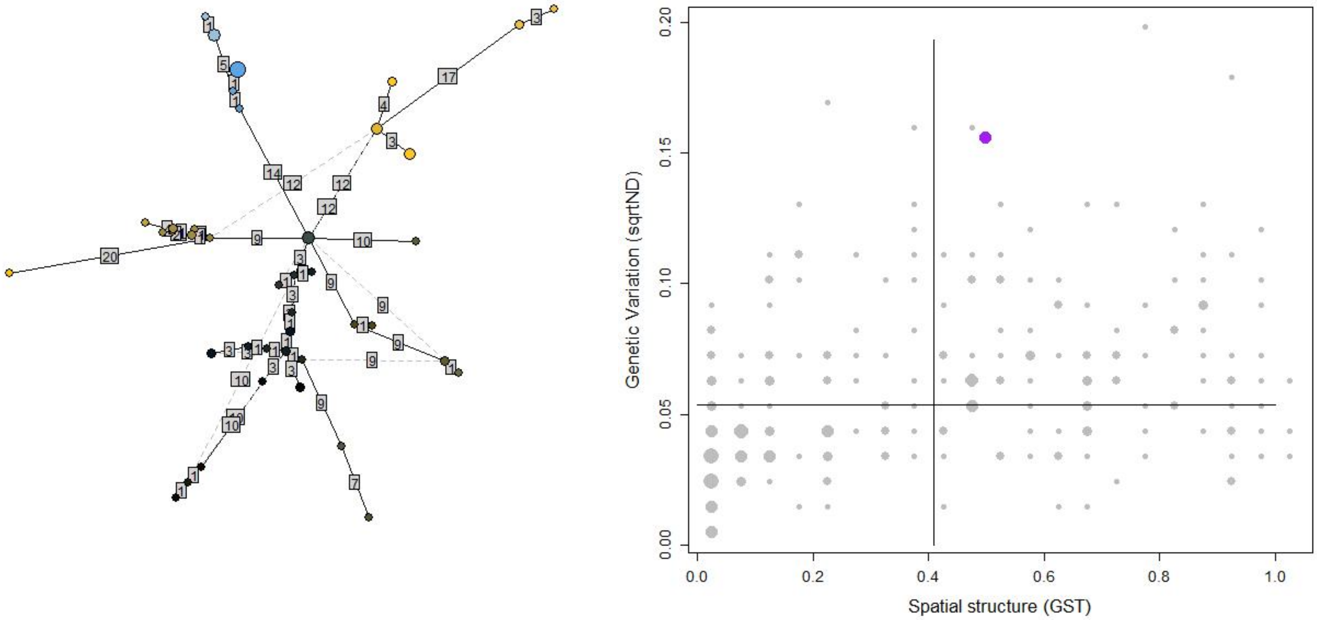

Figure 416: Haplotype network (left) of *Icaricia icarioides* sequences > 599 bp with colours matching the PCoA colour space (above). The bubble plot for mt-DNA polymorphism (square root transformed nucleotide diversity) and spatial structure (GST) among all species in the atlas and values for *Icaricia icarioides* (purple dot). The horizontal and vertical lines represent median values of nucleotide diversity and GST, respectively. Sequences > 599 bp= 83.

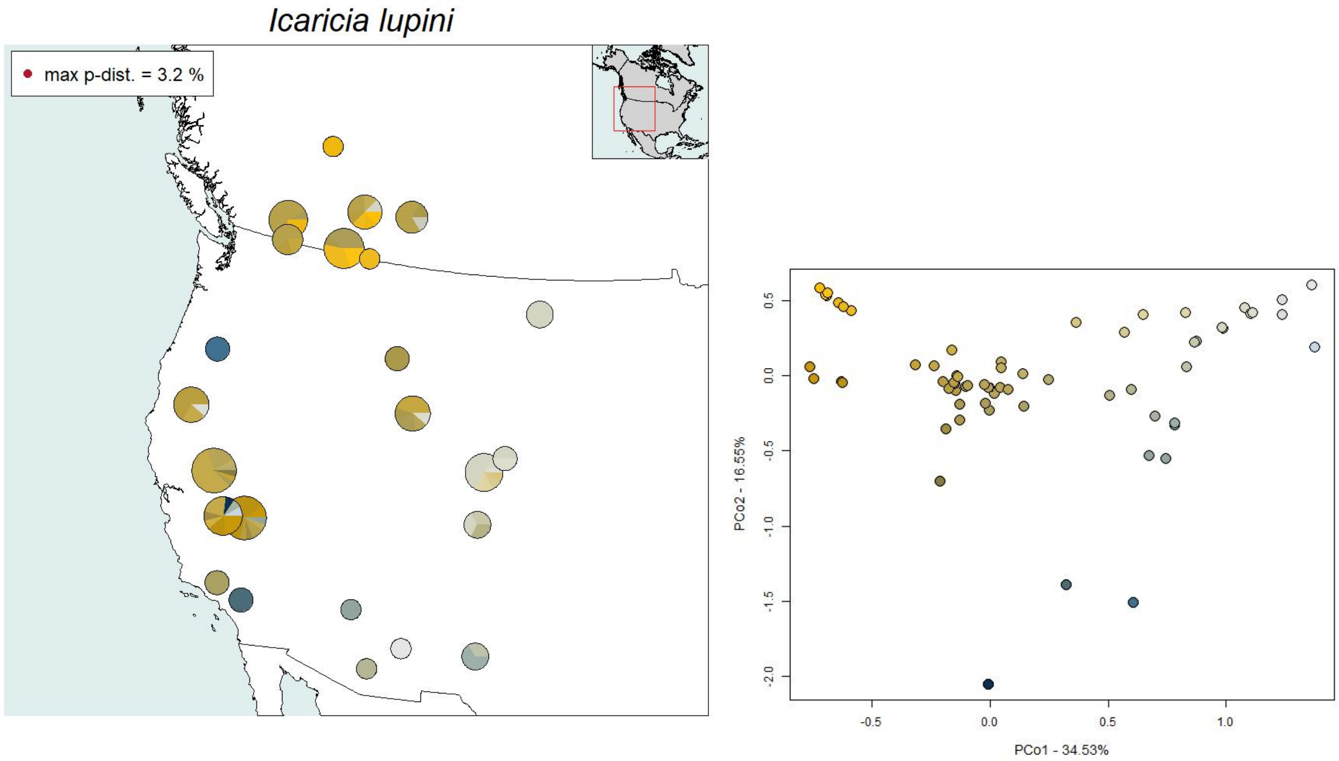

Figure 417 Map of *Icaricia lupini* showing the localities of the sequenced specimens (left). Nearby localities are grouped in pies. Colours match the bidimensional colour space of the PCoA projection (right) of max p-dists among sequences (dots). Sequences= 158; Hap obs.= 55; Hap asympt.= 131.1; Hap % obs.= 42%; GST= 0.343; DST= 0.0034; HD= 0.947; ND= 0.0106; max p-dist= 3.2%.

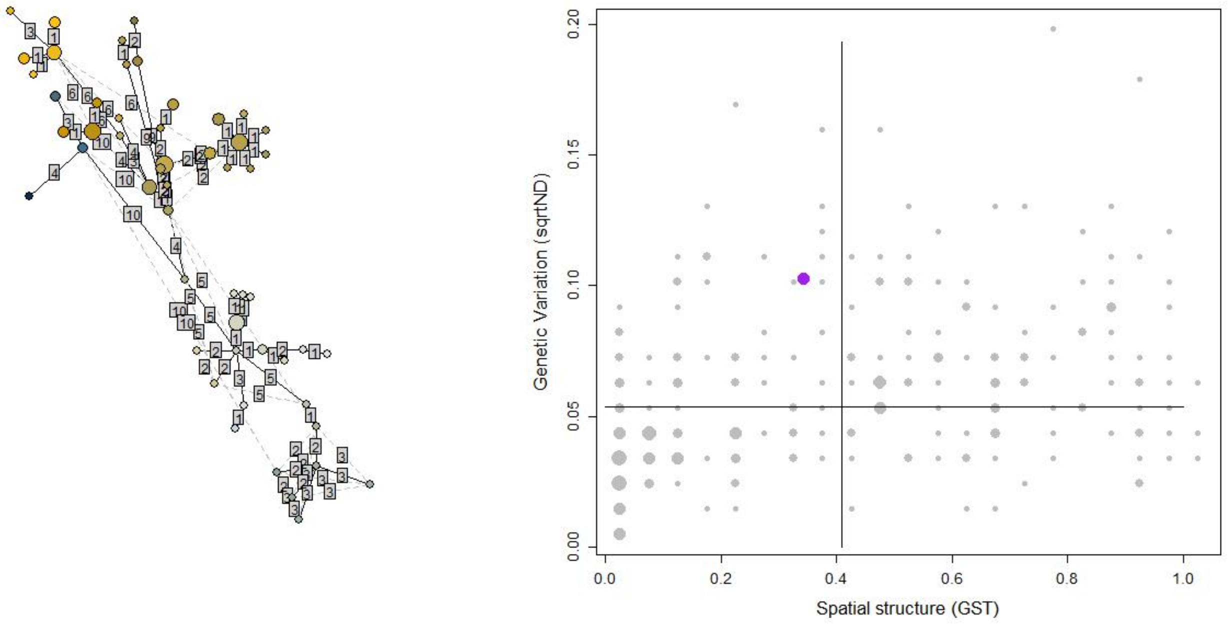

Figure 418: Haplotype network (left) of *Icaricia lupini* sequences > 599 bp with colours matching the PCoA colour space (above). The bubble plot for mt-DNA polymorphism (square root transformed nucleotide diversity) and spatial structure (GST) among all species in the atlas and values for *Icaricia lupini* (purple dot). The horizontal and vertical lines represent median values of nucleotide diversity and GST, respectively. Sequences > 599 bp= 154.

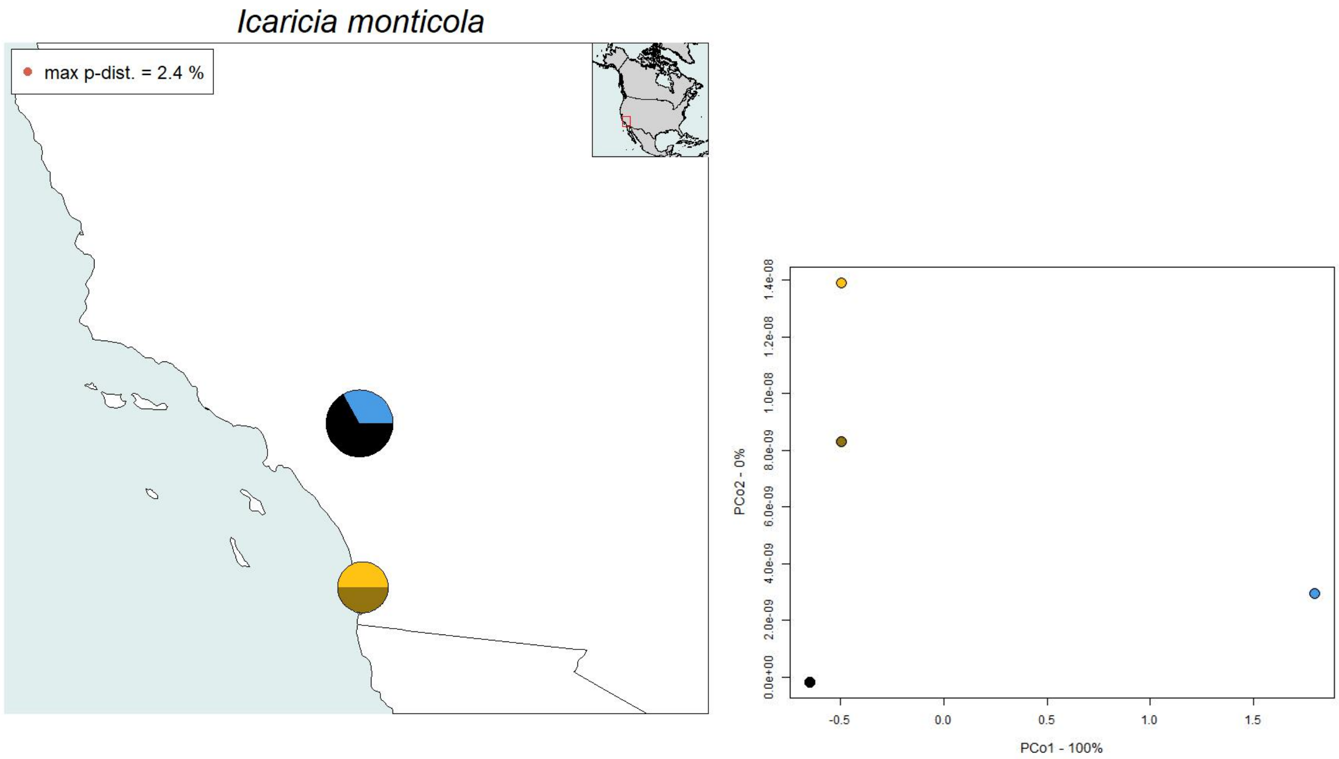

Figure 419 Map of *Icaricia monticola* showing the localities of the sequenced specimens (left). Nearby localities are grouped in pies. Colours match the bidimensional colour space of the PCoA projection (right) of max p-dists among sequences (dots). Sequences= 8; Hap obs.= 3; Hap asympt.= NA; Hap % obs.= NA%; GST= NaN; DST= NaN; HD= NA; ND= NA; max p-dist= 2.4%.

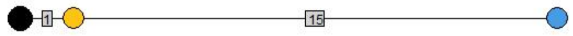

Figure 420: Haplotype network of *Icaricia monticola*. Sequences > 599 bp= 8.

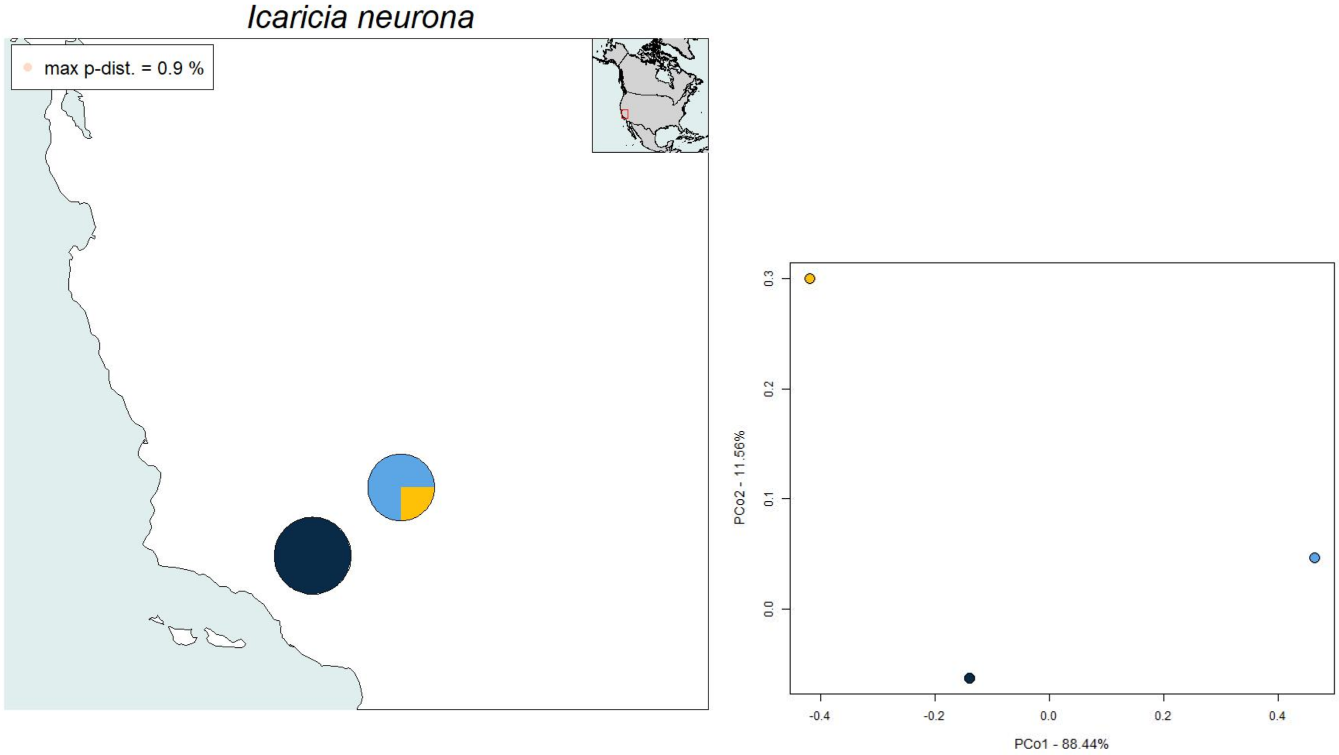

Figure 421 Map of *Icaricia neurona* showing the localities of the sequenced specimens (left). Nearby localities are grouped in pies. Colours match the bidimensional colour space of the PCoA projection (right) of max p-dists among sequences (dots). Sequences= 11; Hap obs.= 3; Hap asympt.= 3; Hap % obs.= 100%; GST= 0.702; DST= 0.0024; HD= 0.564; ND= 0.0034; max p-dist= 0.9%.

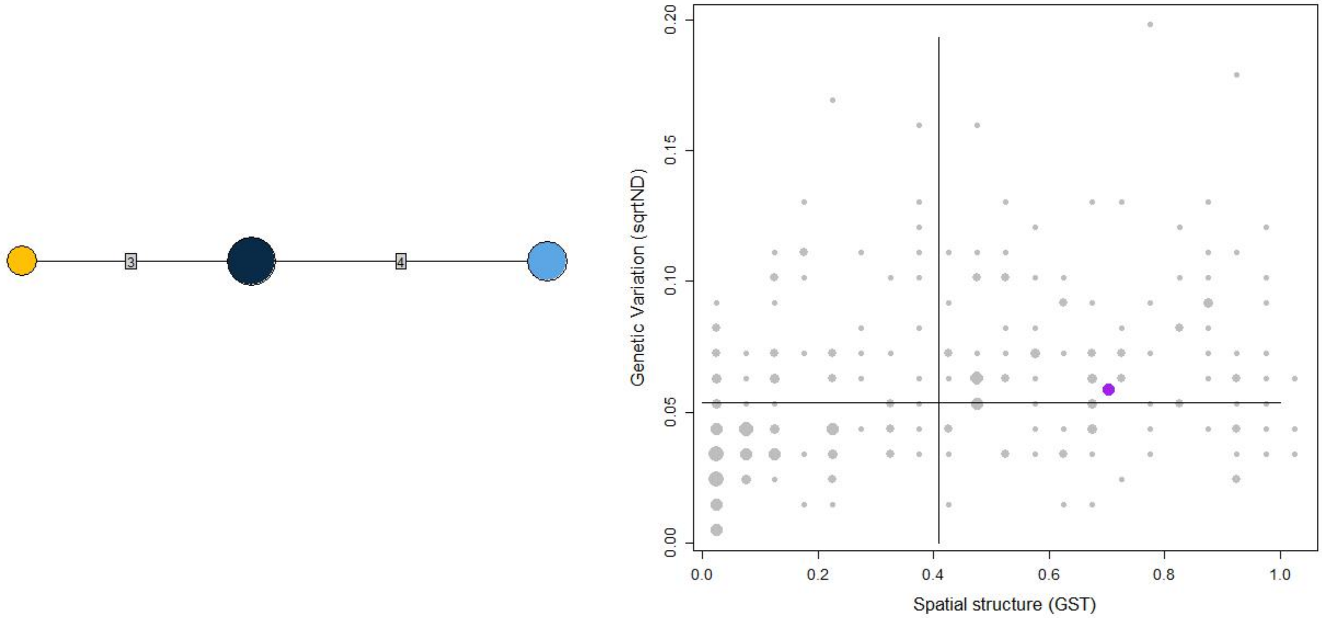

Figure 422: Haplotype network (left) of *Icaricia neurona* sequences > 599 bp with colours matching the PCoA colour space (above). The bubble plot for mt-DNA polymorphism (square root transformed nucleotide diversity) and spatial structure (GST) among all species in the atlas and values for *Icaricia neurona* (purple dot). The horizontal and vertical lines represent median values of nucleotide diversity and GST, respectively. Sequences > 599 bp= 11.

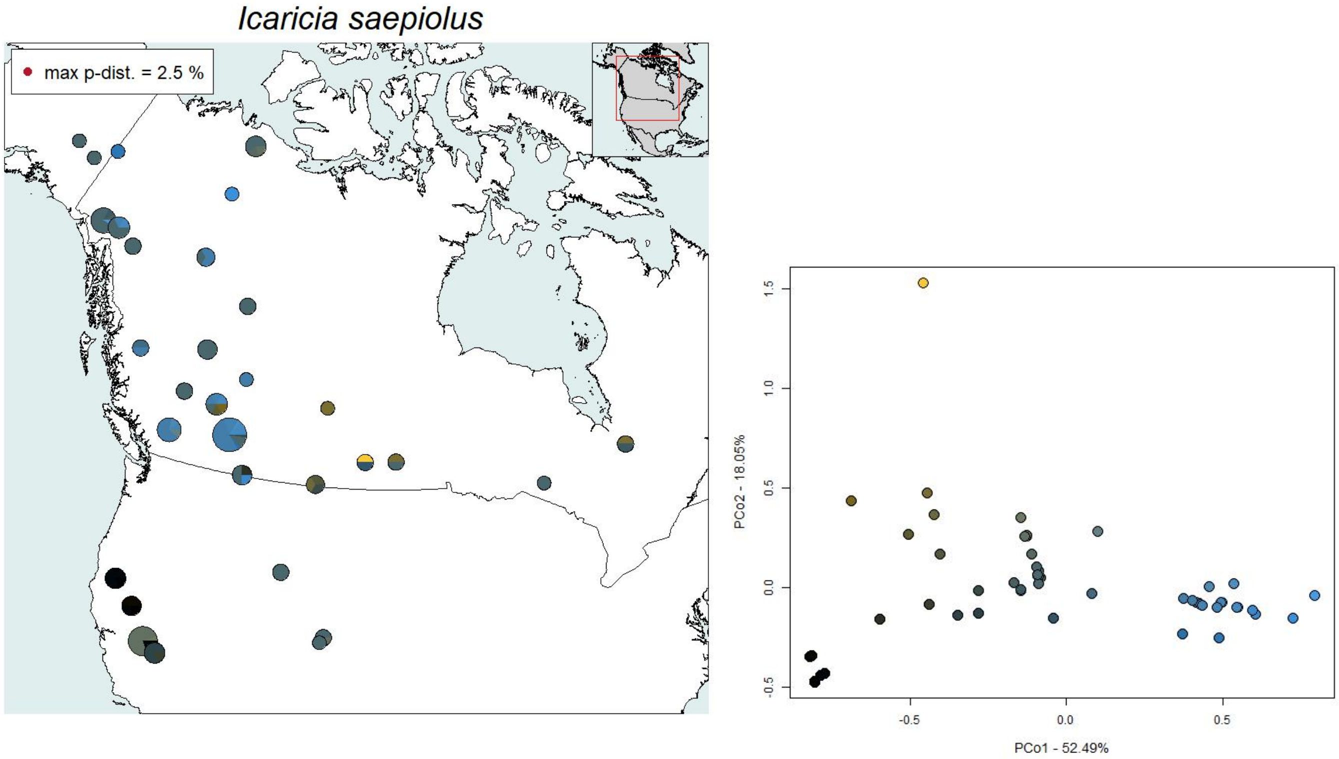

Figure 423 Map of *Icaricia saepiolus* showing the localities of the sequenced specimens (left). Nearby localities are grouped in pies. Colours match the bidimensional colour space of the PCoA projection (right) of max p-dists among sequences (dots). Sequences= 147; Hap obs.= 34; Hap asympt.= 94.1; Hap % obs.= 36.1%; GST= 0.524; DST= 0.0029; HD= 0.865; ND= 0.0057; max p-dist= 2.5%.

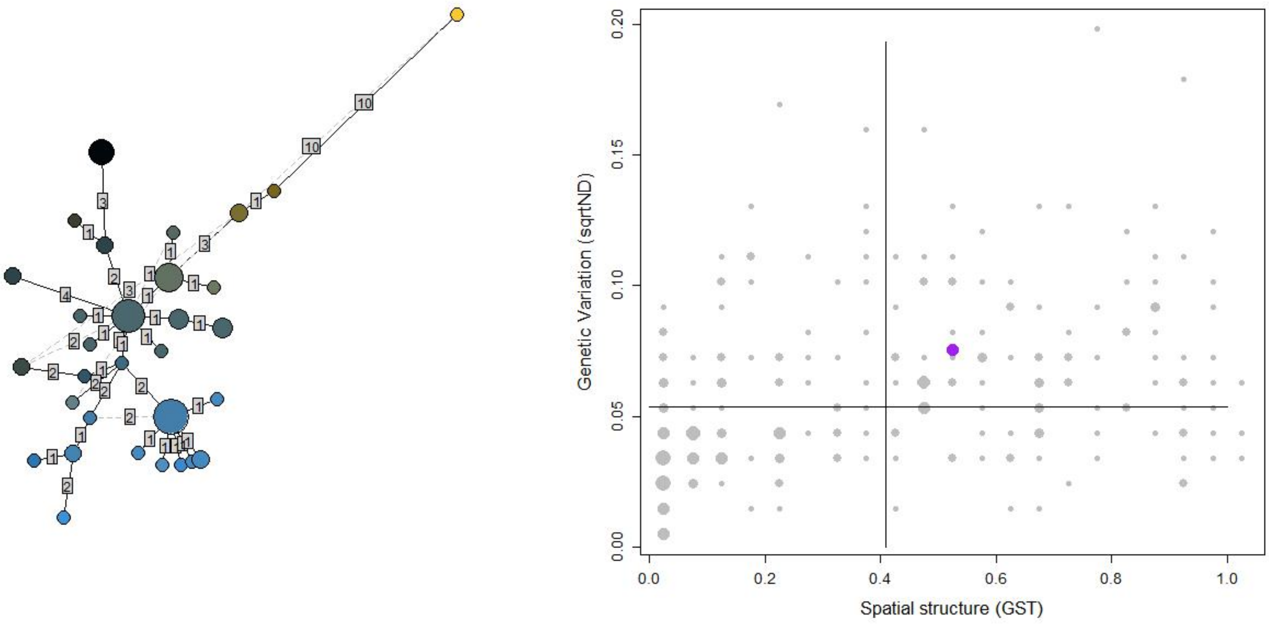

Figure 424: Haplotype network (left) of *Icaricia saepiolus* sequences > 599 bp with colours matching the PCoA colour space (above). The bubble plot for mt-DNA polymorphism (square root transformed nucleotide diversity) and spatial structure (GST) among all species in the atlas and values for *Icaricia saepiolus* (purple dot). The horizontal and vertical lines represent median values of nucleotide diversity and GST, respectively. Sequences > 599 bp= 136.

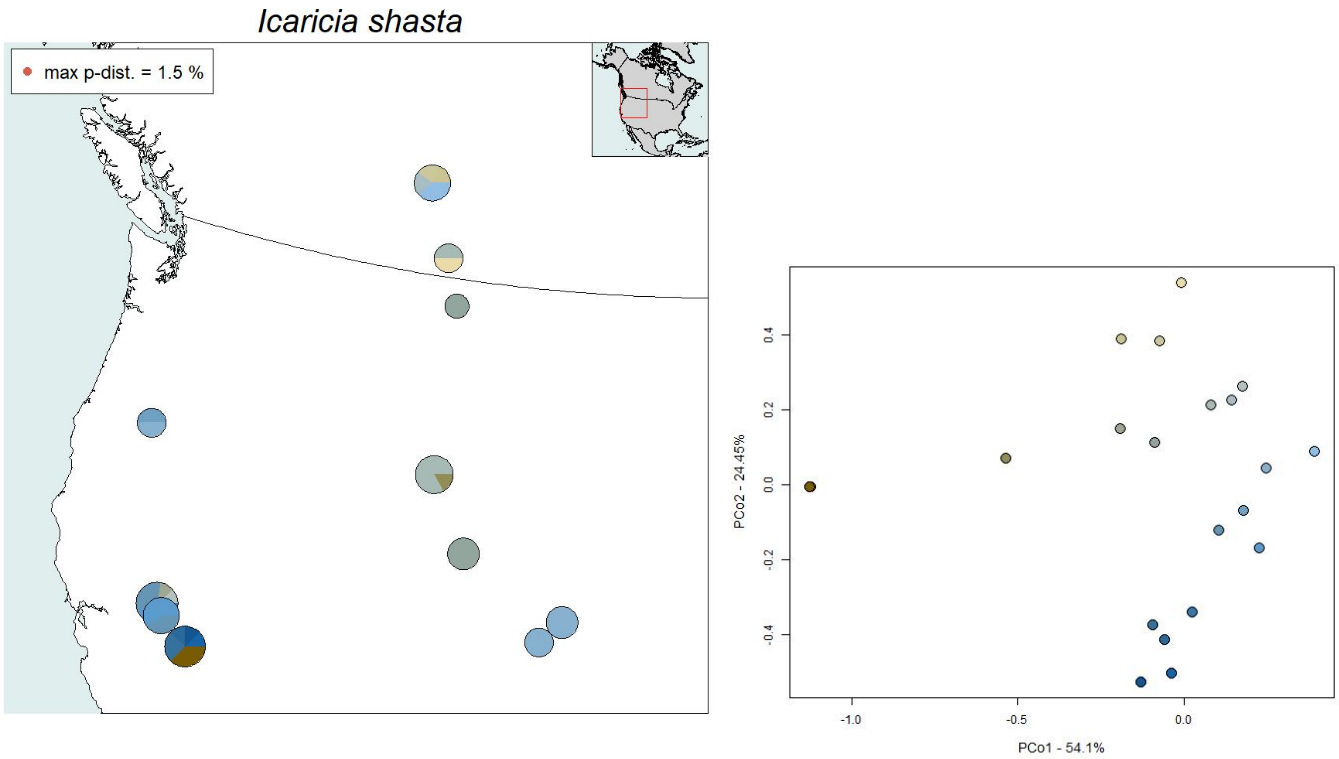

Figure 425 Map of *Icaricia shasta* showing the localities of the sequenced specimens (left). Nearby localities are grouped in pies. Colours match the bidimensional colour space of the PCoA projection (right) of max p-dists among sequences (dots). Sequences= 46; Hap obs.= 21; Hap asympt.= 44.5; Hap % obs.= 47.2%; GST= 0.242; DST= 0.0013; HD= 0.941; ND= 0.005; max p-dist= 1.5%.

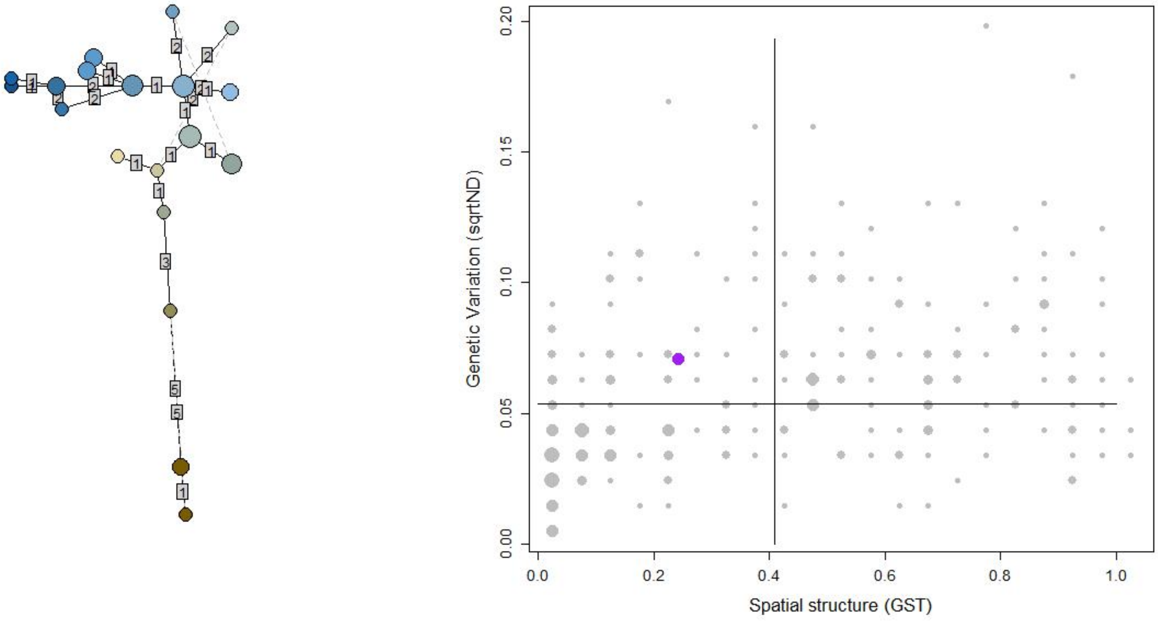

Figure 426: Haplotype network (left) of *Icaricia shasta* sequences > 599 bp with colours matching the PCoA colour space (above). The bubble plot for mt-DNA polymorphism (square root transformed nucleotide diversity) and spatial structure (GST) among all species in the atlas and values for *Icaricia shasta* (purple dot). The horizontal and vertical lines represent median values of nucleotide diversity and GST, respectively. Sequences > 599 bp= 45.

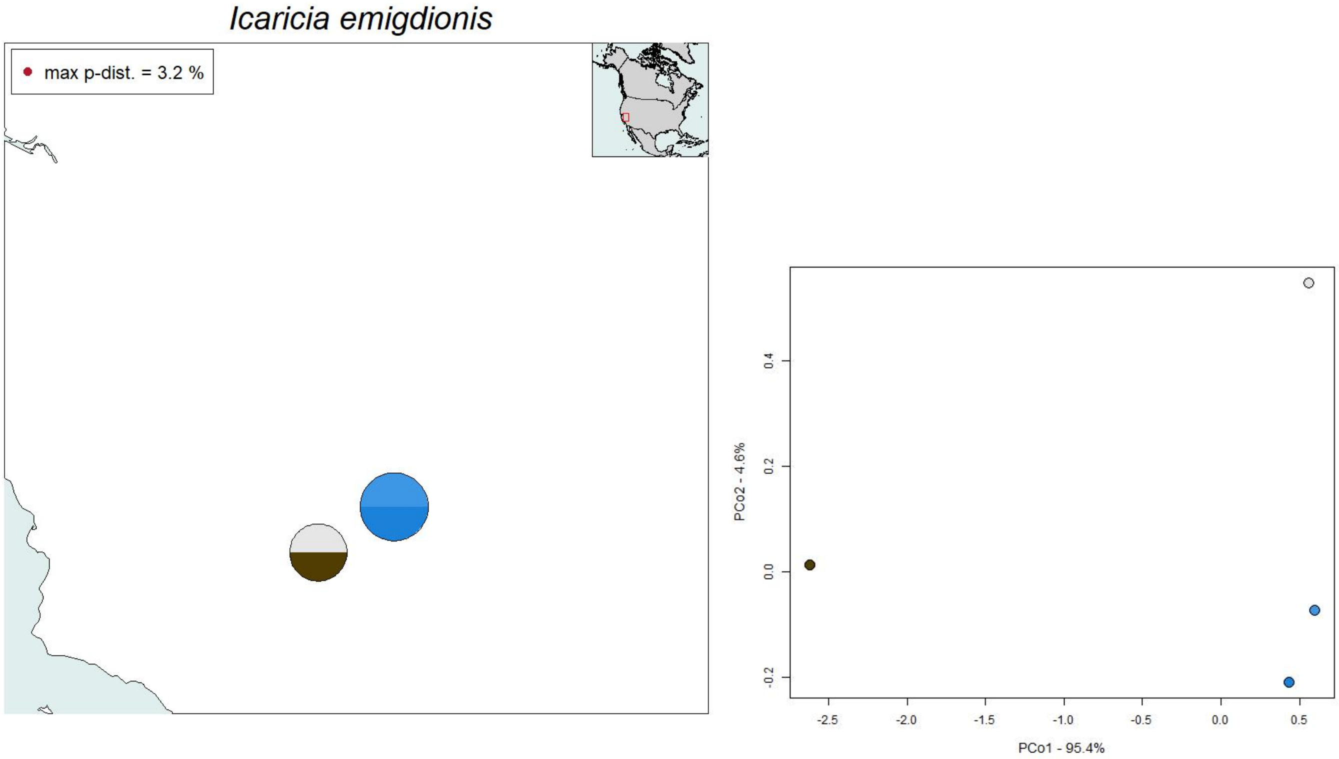

Figure 427 Map of *Icaricia emigdionis* showing the localities of the sequenced specimens (left). Nearby localities are grouped in pies. Colours match the bidimensional colour space of the PCoA projection (right) of max p-dists among sequences (dots). Sequences= 6; Hap obs.= 4; Hap asympt.= NA; Hap % obs.= NA%; GST= NaN; DST= NaN; HD= NA; ND= NA; max p-dist= 3.2%.

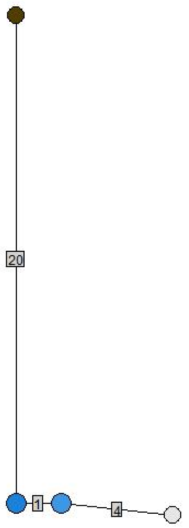

Figure 428: Haplotype network of *Icaricia emigdionis*. Sequences > 599 bp= 6.

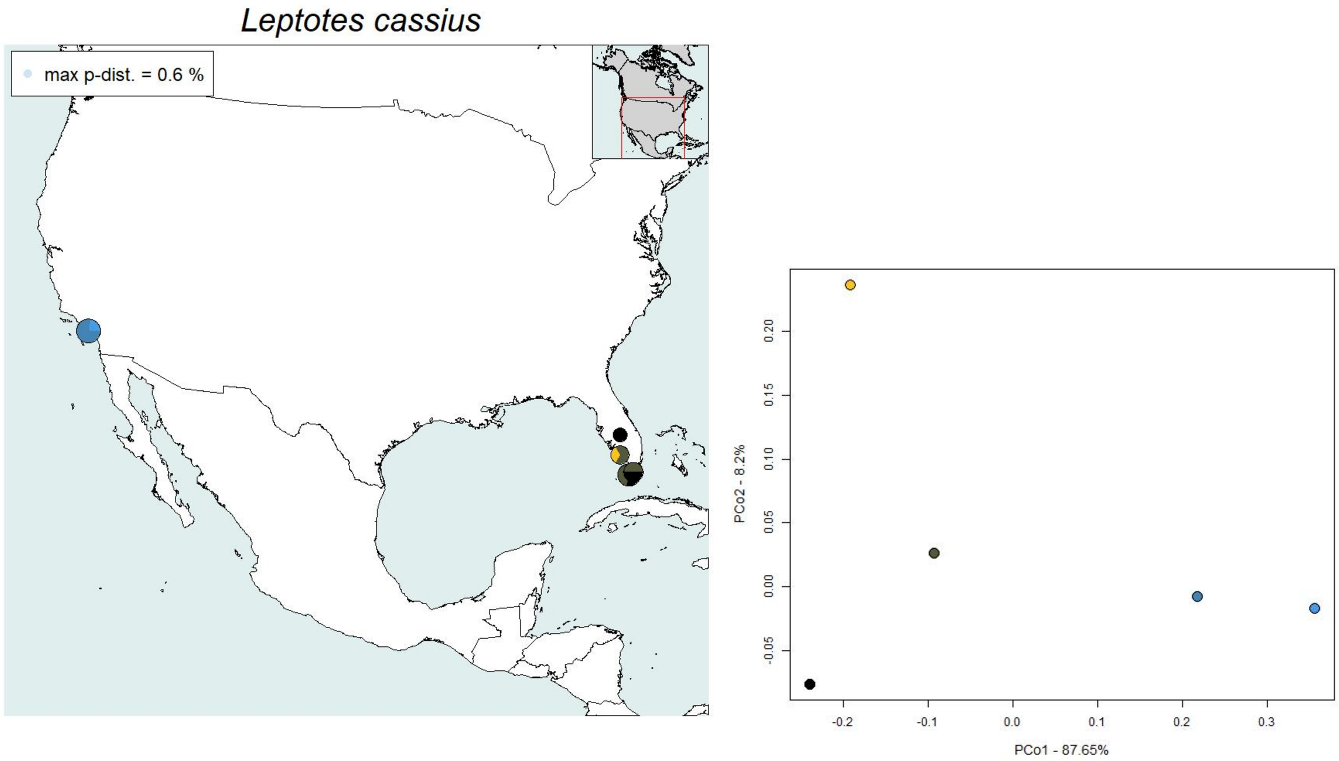

Figure 429 Map of *Leptotes cassius* showing the localities of the sequenced specimens (left). Nearby localities are grouped in pies. Colours match the bidimensional colour space of the PCoA projection (right) of max p-dists among sequences (dots). Sequences= 24; Hap obs.= 6; Hap asympt.= 8.9; Hap % obs.= 67.6%; GST= 0.684; DST= 0.0016; HD= 0.757; ND= 0.0024; max p-dist= 0.6%.

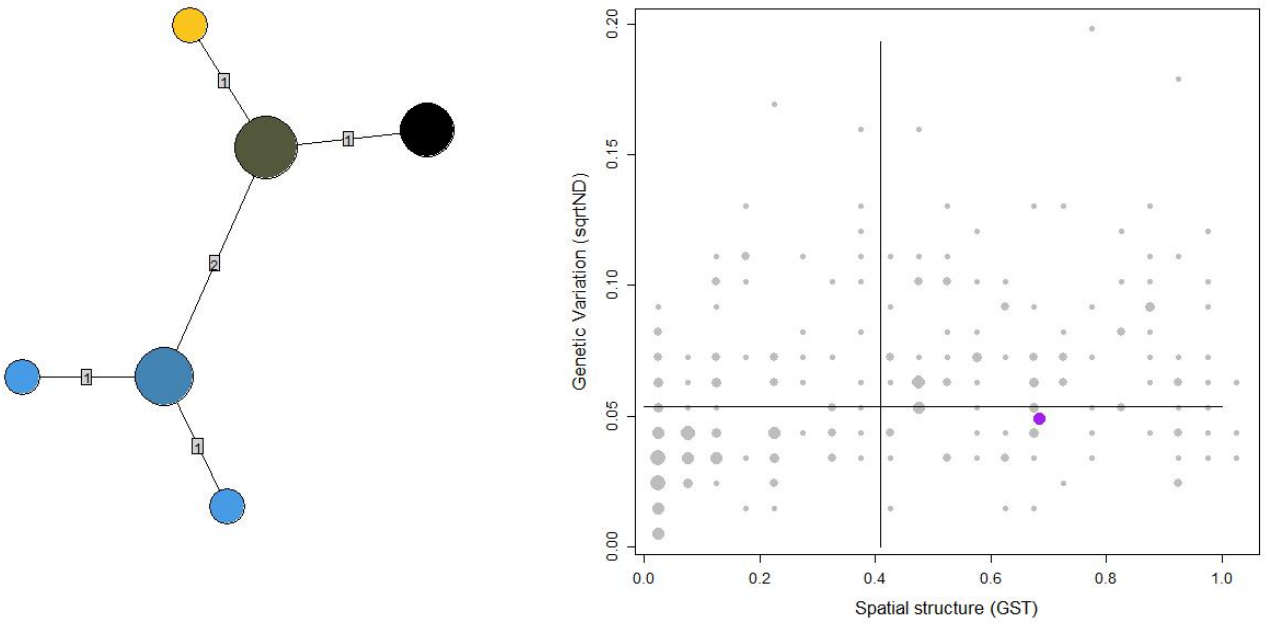

Figure 430: Haplotype network (left) of *Leptotes cassius* sequences > 599 bp with colours matching the PCoA colour space (above). The bubble plot for mt-DNA polymorphism (square root transformed nucleotide diversity) and spatial structure (GST) among all species in the atlas and values for *Leptotes cassius* (purple dot). The horizontal and vertical lines represent median values of nucleotide diversity and GST, respectively. Sequences > 599 bp= 24.

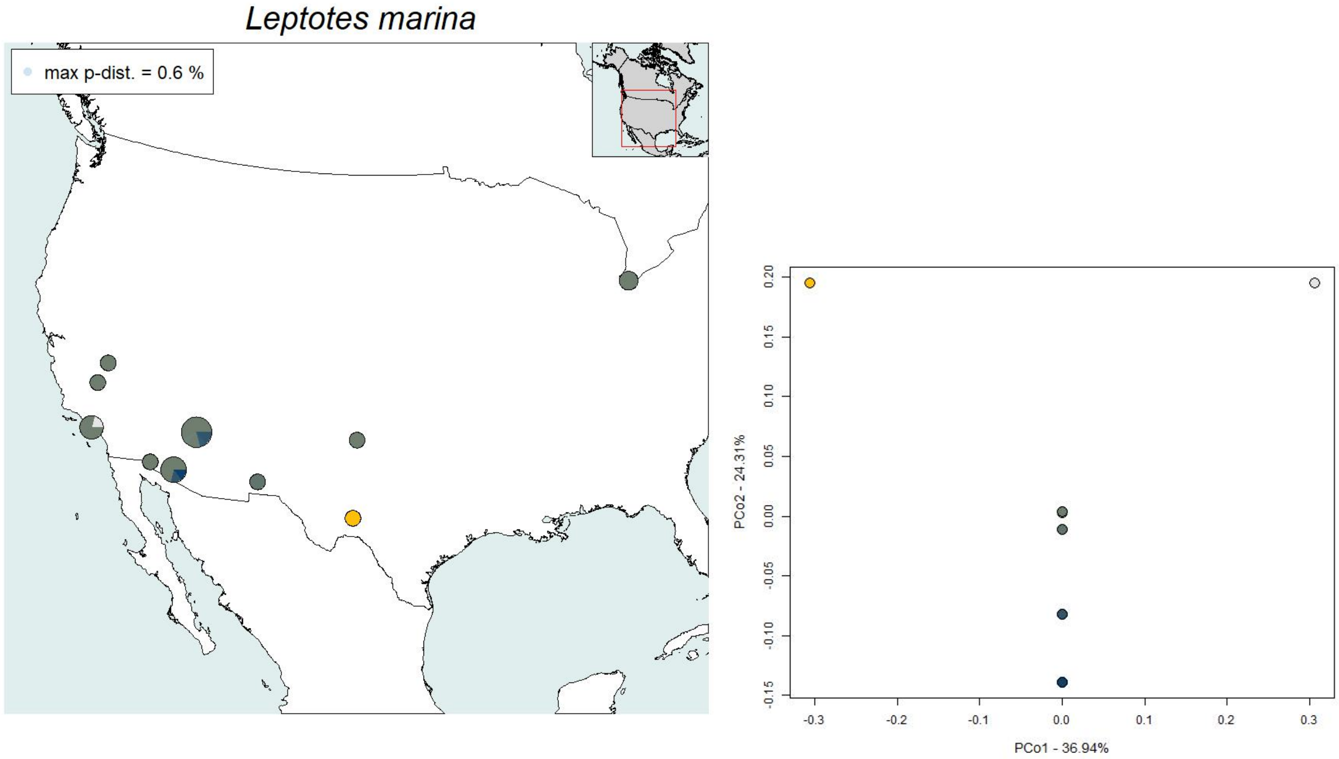

Figure 431 Map of *Leptotes marina* showing the localities of the sequenced specimens (left). Nearby localities are grouped in pies. Colours match the bidimensional colour space of the PCoA projection (right) of max p-dists among sequences (dots). Sequences= 34; Hap obs.= 8; Hap asympt.= 28.4; Hap % obs.= 28.2%; GST= 0.112; DST= 0.0001; HD= 0.374; ND= 0.0008; max p-dist= 0.6%.

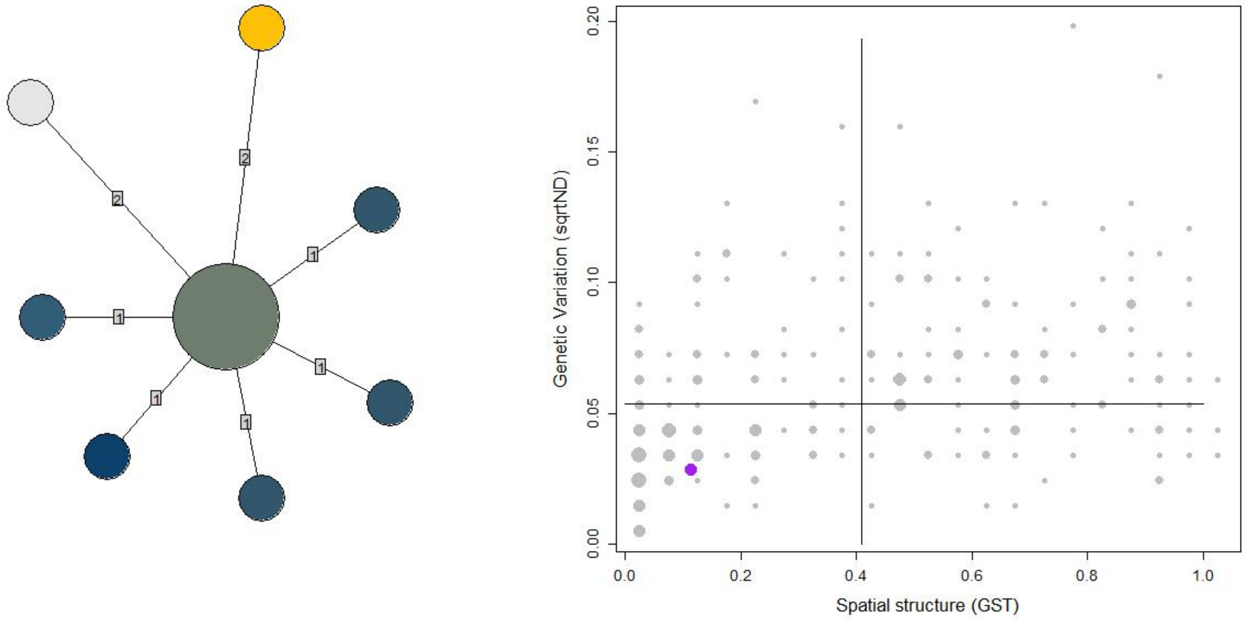

Figure 432: Haplotype network (left) of *Leptotes marina* sequences > 599 bp with colours matching the PCoA colour space (above). The bubble plot for mt-DNA polymorphism (square root transformed nucleotide diversity) and spatial structure (GST) among all species in the atlas and values for *Leptotes marina* (purple dot). The horizontal and vertical lines represent median values of nucleotide diversity and GST, respectively. Sequences > 599 bp= 34.

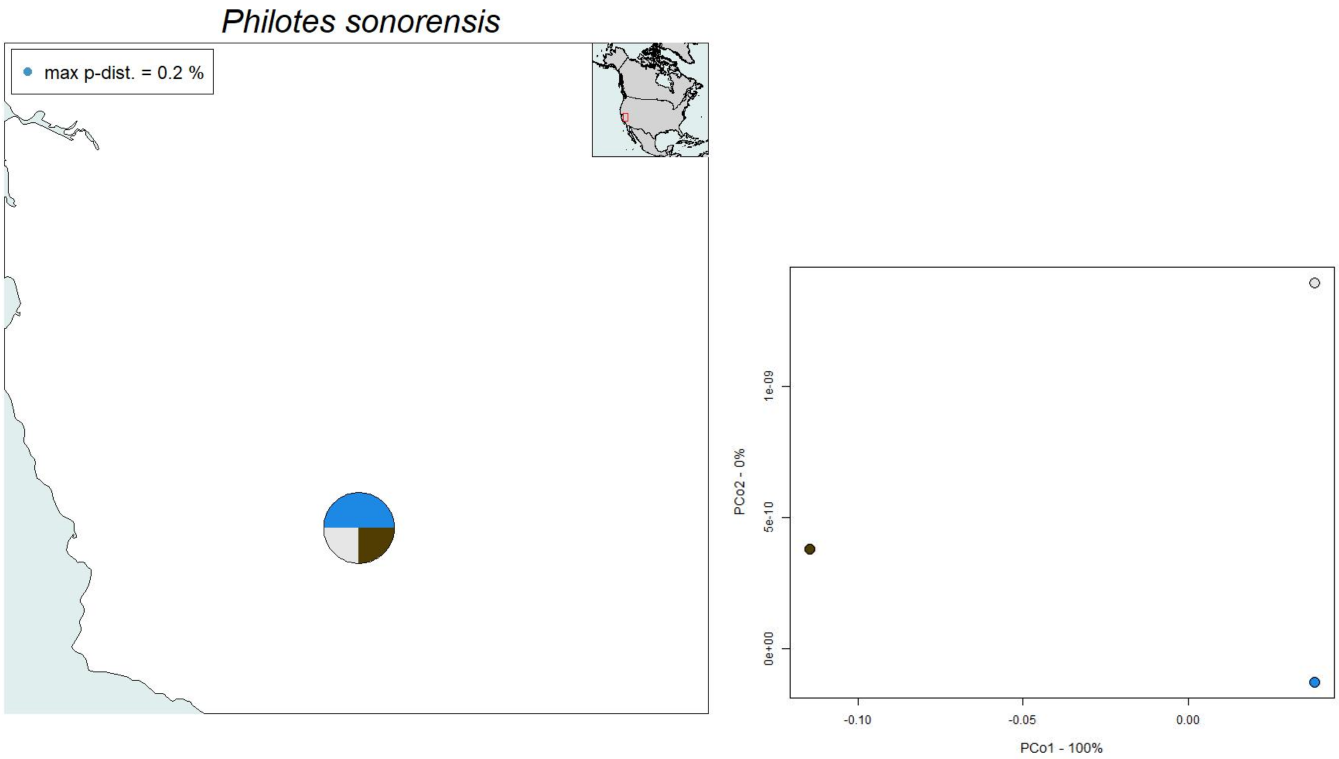

Figure 433 Map of *Philotes sonorensis* showing the localities of the sequenced specimens (left). Nearby localities are grouped in pies. Colours match the bidimensional colour space of the PCoA projection (right) of max p-dists among sequences (dots). Sequences= 4; Hap obs.= 2; Hap asympt.= NA; Hap % obs.= NA%; GST= NaN; DST= NaN; HD= NA; ND= NA; max p-dist= 0.2%.

Haplotype network analysis and bubble plot of *Philotes sonorensis* were not possible. Sequences > 599 bp = 4.

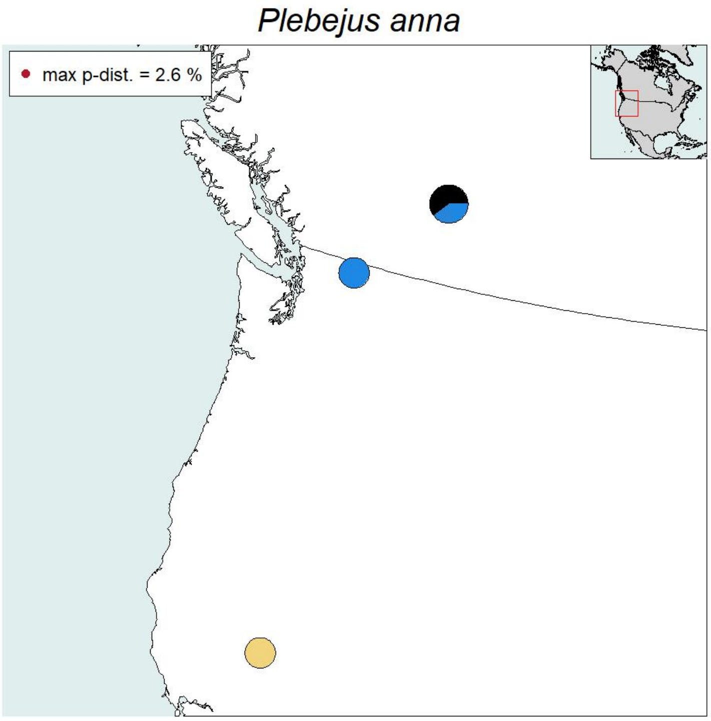

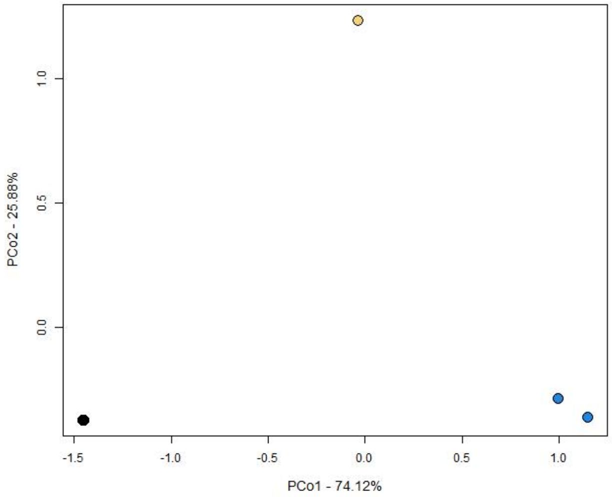

Figure 434 Map of *Plebejus anna* showing the localities of the sequenced specimens (left). Nearby localities are grouped in pies. Colours match the bidimensional colour space of the PCoA projection (right) of max p-dists among sequences (dots). Sequences= 9; Hap obs.= 4; Hap asympt.= NA; Hap % obs.= NA%; GST= NaN; DST= NaN; HD= NA; ND= NA; max p-dist= 2.6%.

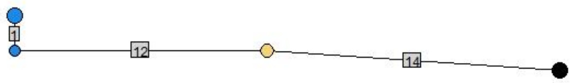

Figure 435: Haplotype network of *Plebejus anna*. Sequences > 599 bp= 9.

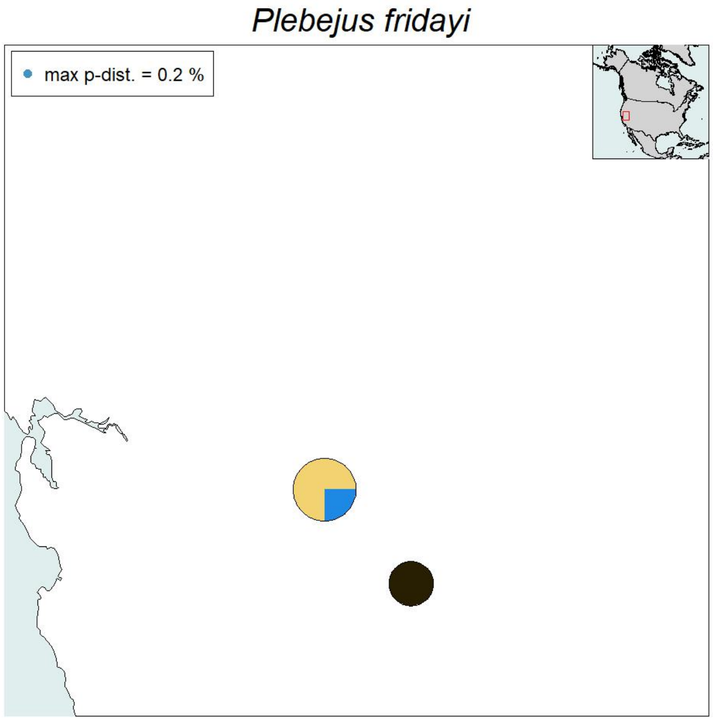

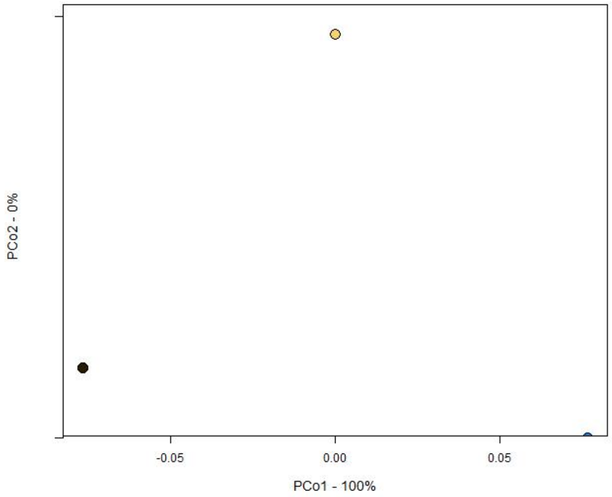

Figure 436 Map of *Plebejus fridayi* showing the localities of the sequenced specimens (left). Nearby localities are grouped in pies. Colours match the bidimensional colour space of the PCoA projection (right) of max p-dists among sequences (dots). Sequences= 5; Hap obs.= 3; Hap asympt.= NA; Hap % obs.= NA%; GST= NaN; DST= NaN; HD= NA; ND= NA; max p-dist= 0.2%.

Haplotype network analysis and bubble plot of *Plebejus fridayi* were not possible. Sequences > 599 bp = 4.

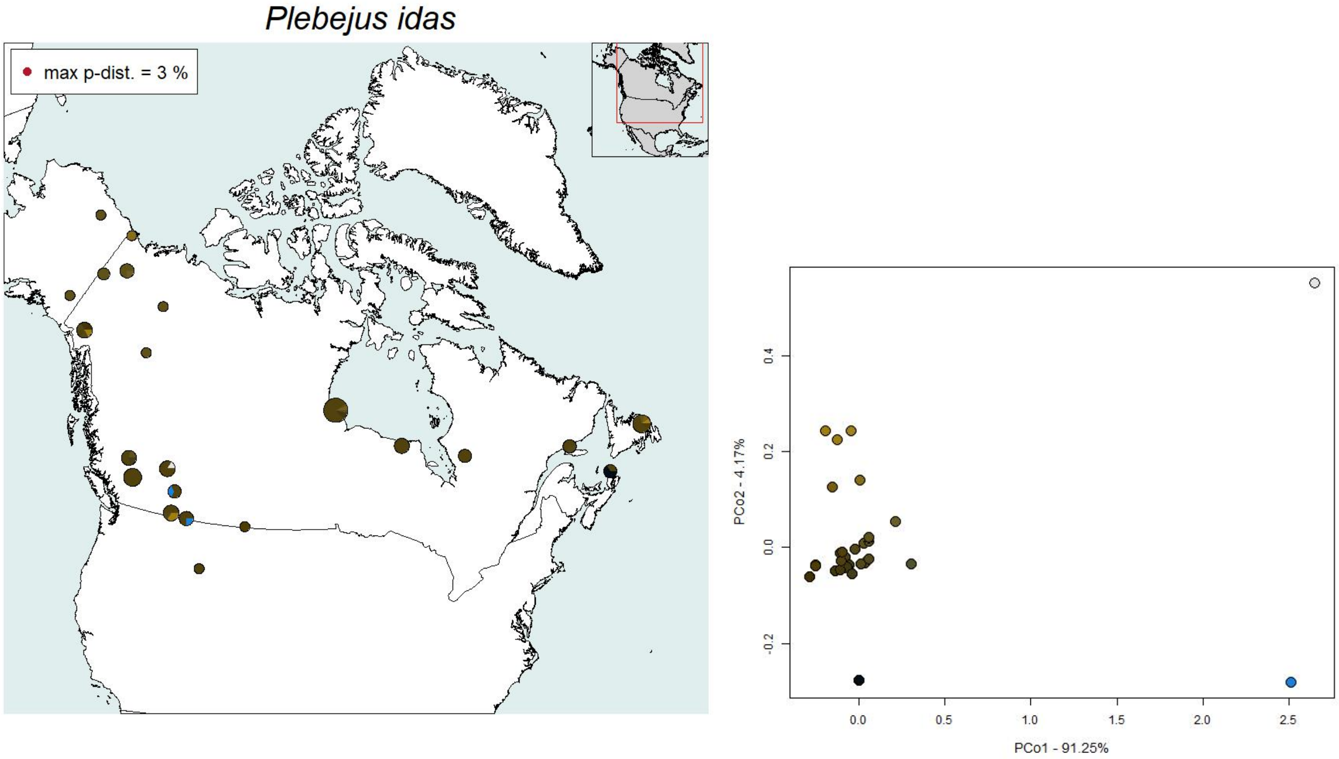

Figure 437 Map of *Plebejus idas* showing the localities of the sequenced specimens (left). Nearby localities are grouped in pies. Colours match the bidimensional colour space of the PCoA projection (right) of max p-dists among sequences (dots). Sequences= 110; Hap obs.= 19; Hap asympt.= 34; Hap % obs.= 55.9%; GST= 0.677; DST= 0.0013; HD= 0.592; ND= 0.0021; max p-dist= 3%.

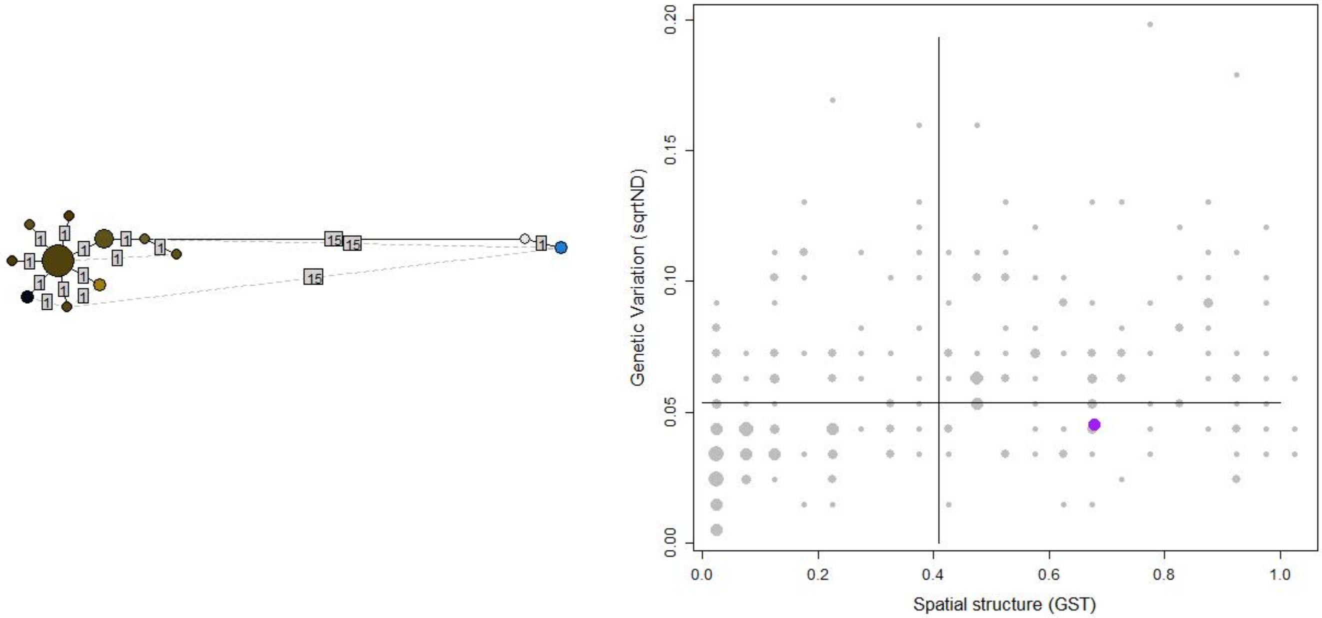

Figure 438: Haplotype network (left) of *Plebejus idas* sequences > 599 bp with colours matching the PCoA colour space (above). The bubble plot for mt-DNA polymorphism (square root transformed nucleotide diversity) and spatial structure (GST) among all species in the atlas and values for *Plebejus idas* (purple dot). The horizontal and vertical lines represent median values of nucleotide diversity and GST, respectively. Sequences > 599 bp= 97.

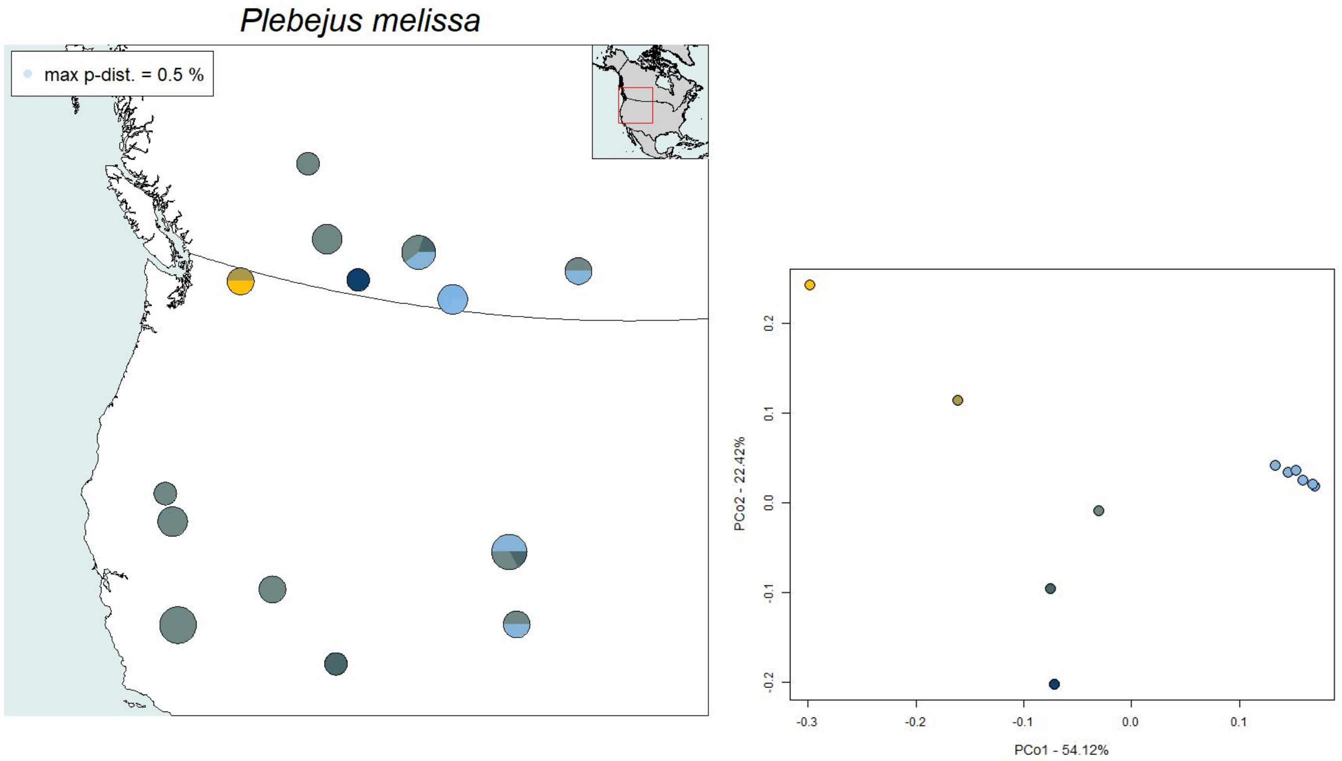

Figure 439 Map of *Plebejus melissa* showing the localities of the sequenced specimens (left). Nearby localities are grouped in pies. Colours match the bidimensional colour space of the PCoA projection (right) of max p-dists among sequences (dots). Sequences= 39; Hap obs.= 8; Hap asympt.= 22.6; Hap % obs.= 35.4%; GST= 0.038; DST= 0; HD= 0.598; ND= 0.0012; max p-dist= 0.5%.

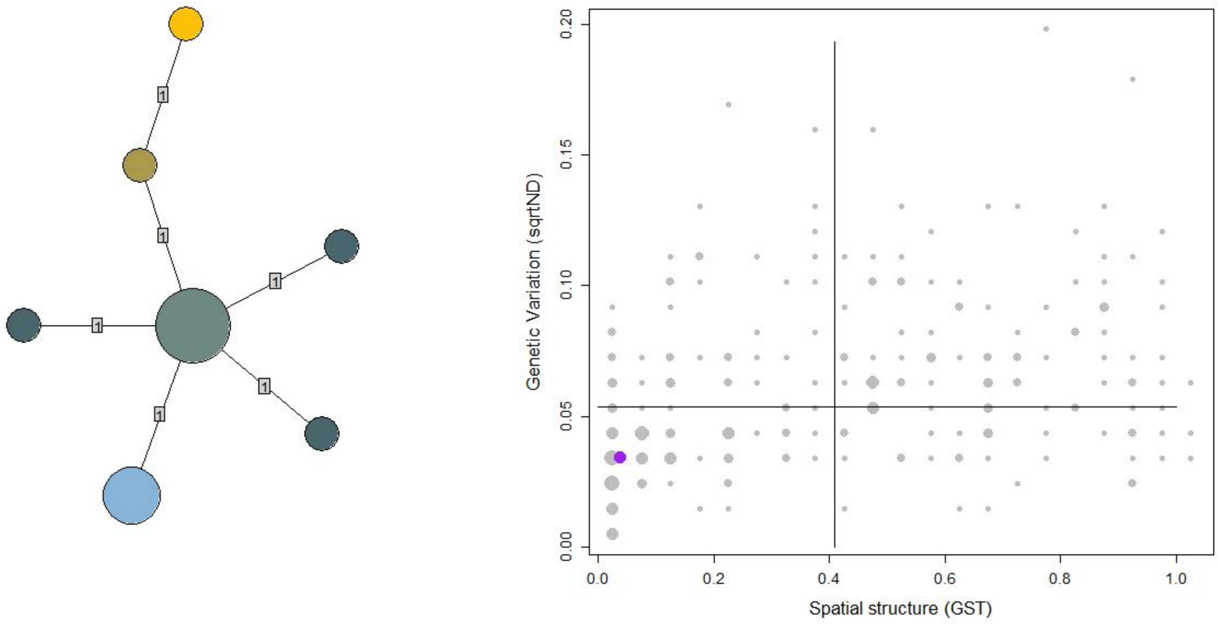

Figure 440: Haplotype network (left) of *Plebejus melissa* sequences > 599 bp with colours matching the PCoA colour space (above). The bubble plot for mt-DNA polymorphism (square root transformed nucleotide diversity) and spatial structure (GST) among all species in the atlas and values for *Plebejus melissa* (purple dot). The horizontal and vertical lines represent median values of nucleotide diversity and GST, respectively. Sequences > 599 bp= 36.

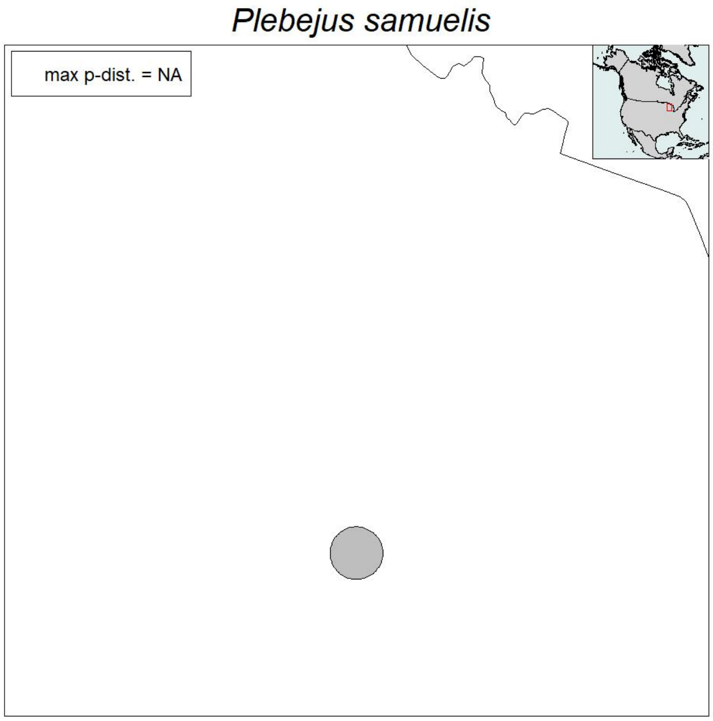

Figure 441: Map of *Plebejus samuelis* showing the localities of the sequenced specimens. Nearby localities are grouped in pies. Due to the presence of a single haplotype PCoA projection was not done and a single grey colour was plotted on the map. Sequences= 1; Hap obs.= NA; Hap asympt.= NA; Hap % obs.= NA; GST= NaN; DST= NaN; HD= NA; ND= NA; max p-dist= NA.

Haplotype network analysis and bubble plot of *Plebejus samuelis* were not possible. Sequences > 599 bp = 1.

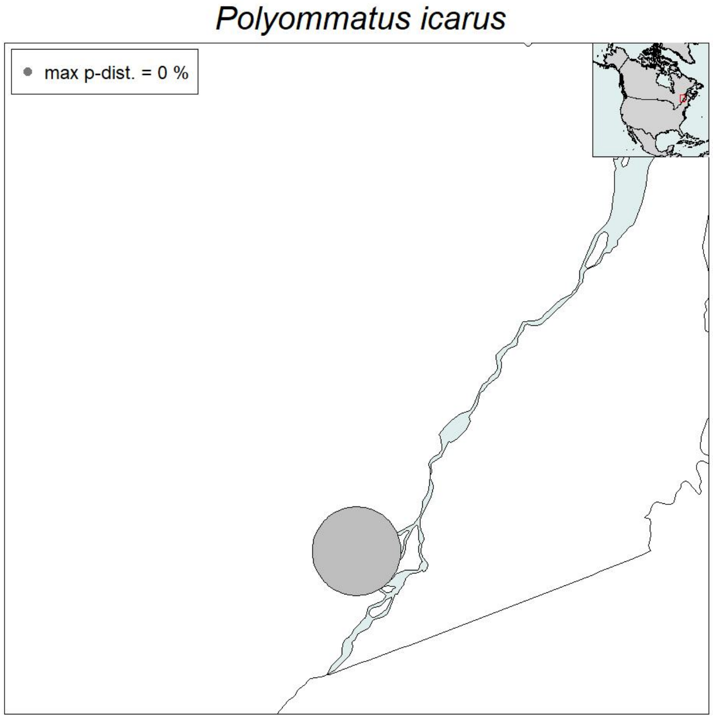

Figure 442: Map of *Polyommatus icarus* showing the localities of the sequenced specimens. Nearby localities are grouped in pies. Due to the presence of a single haplotype PCoA projection was not done and a single grey colour was plotted on the map. Sequences= 8; Hap obs.= 1; Hap asympt.= NA; Hap % obs.= NA%; GST= NaN; DST= NaN; HD= NA; ND= NA; max p-dist= 0%.

Haplotype network analysis and bubble plot of *Polyommatus icarus* were not possible. Sequences > 599 bp = 8.

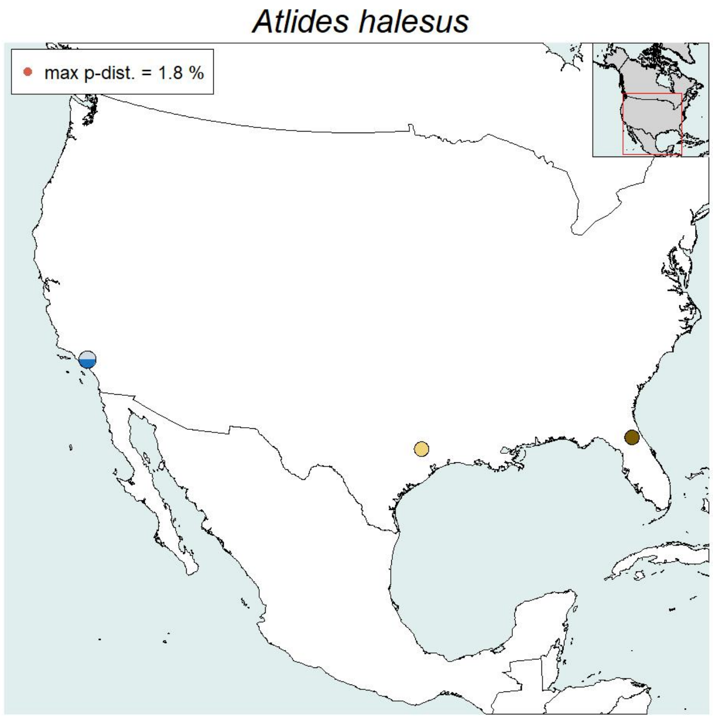

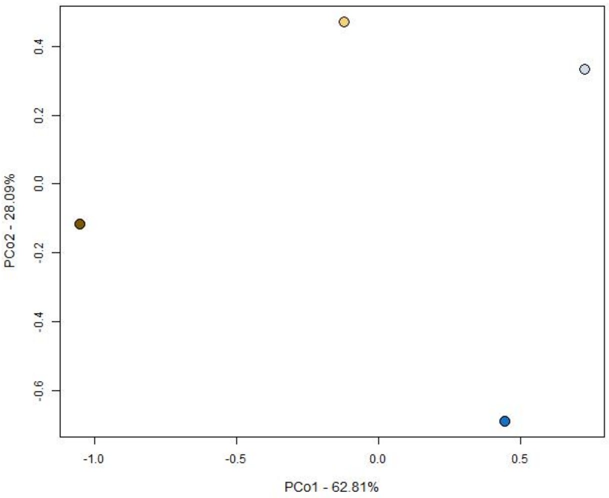

Figure 443 Map of *Atlides halesus* showing the localities of the sequenced specimens (left). Nearby localities are grouped in pies. Colours match the bidimensional colour space of the PCoA projection (right) of max p-dists among sequences (dots). Sequences= 4; Hap obs.= 4; Hap asympt.= NA; Hap % obs.= NA%; GST= NaN; DST= NaN; HD= NA; ND= NA; max p-dist= 1.8%.

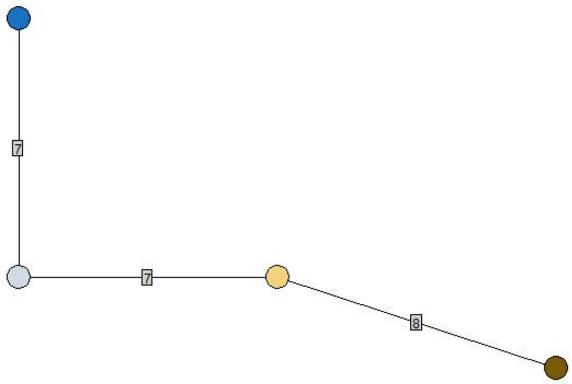

Figure 444: Haplotype network of *Atlides halesus*. Sequences > 599 bp= 4.

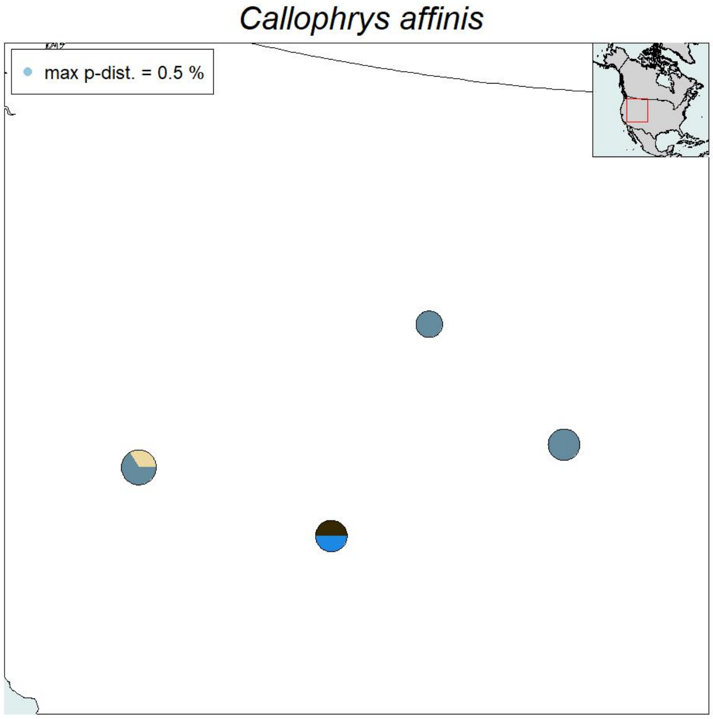

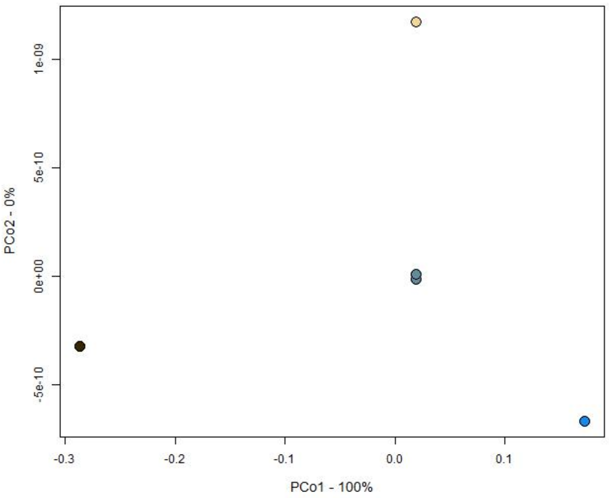

Figure 445 Map of *Callophrys affinis* showing the localities of the sequenced specimens (left). Nearby localities are grouped in pies. Colours match the bidimensional colour space of the PCoA projection (right) of max p-dists among sequences (dots). Sequences= 8; Hap obs.= 3; Hap asympt.= NA; Hap % obs.= NA%; GST= NaN; DST= NaN; HD= NA; ND= NA; max p-dist= 0.5%.

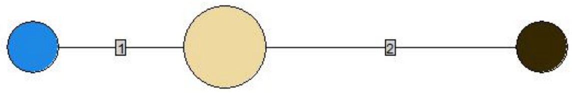

Figure 446: Haplotype network of *Callophrys affinis*. Sequences > 599 bp= 8.

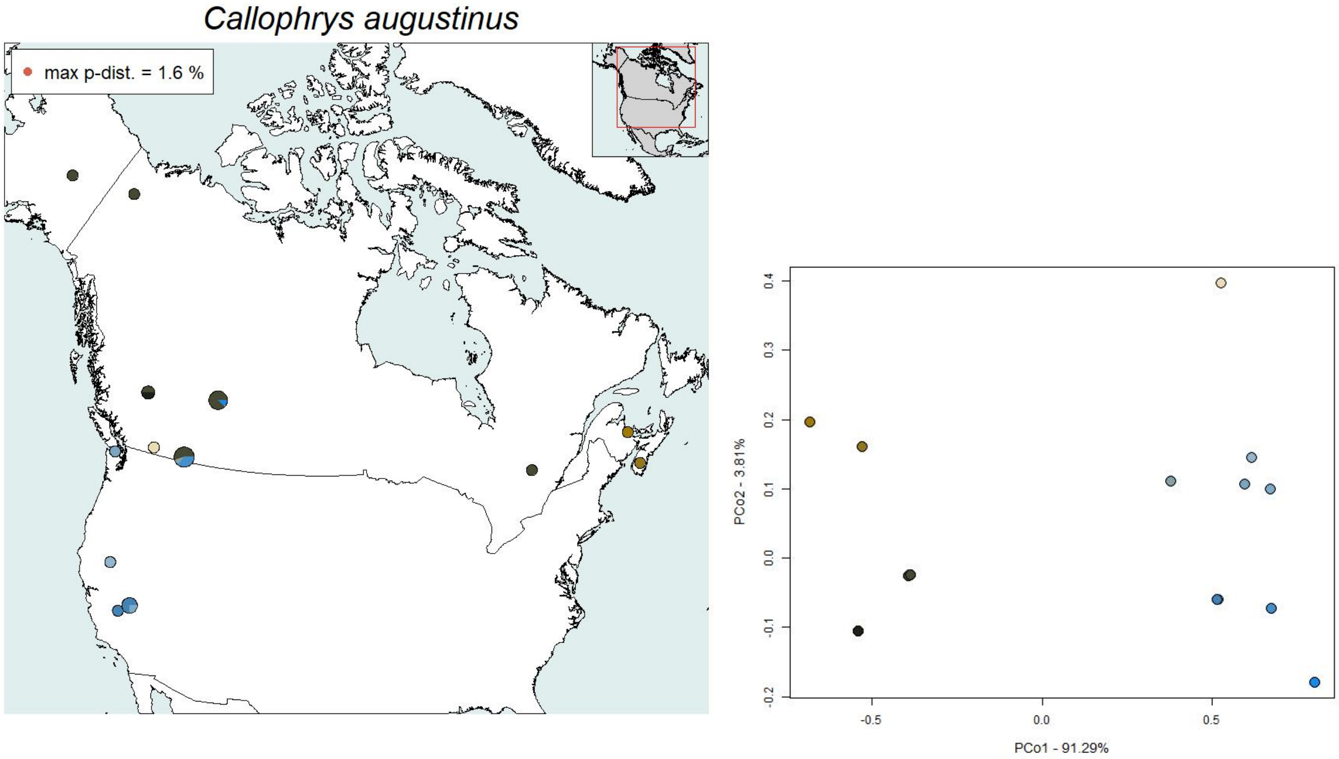

Figure 447 Map of *Callophrys augustinus* showing the localities of the sequenced specimens (left). Nearby localities are grouped in pies. Colours match the bidimensional colour space of the PCoA projection (right) of max p-dists among sequences (dots). Sequences= 34; Hap obs.= 9; Hap asympt.= 21.1; Hap % obs.= 42.6%; GST= 0.553; DST= 0.0032; HD= 0.672; ND= 0.0056; max p-dist= 1.6%.

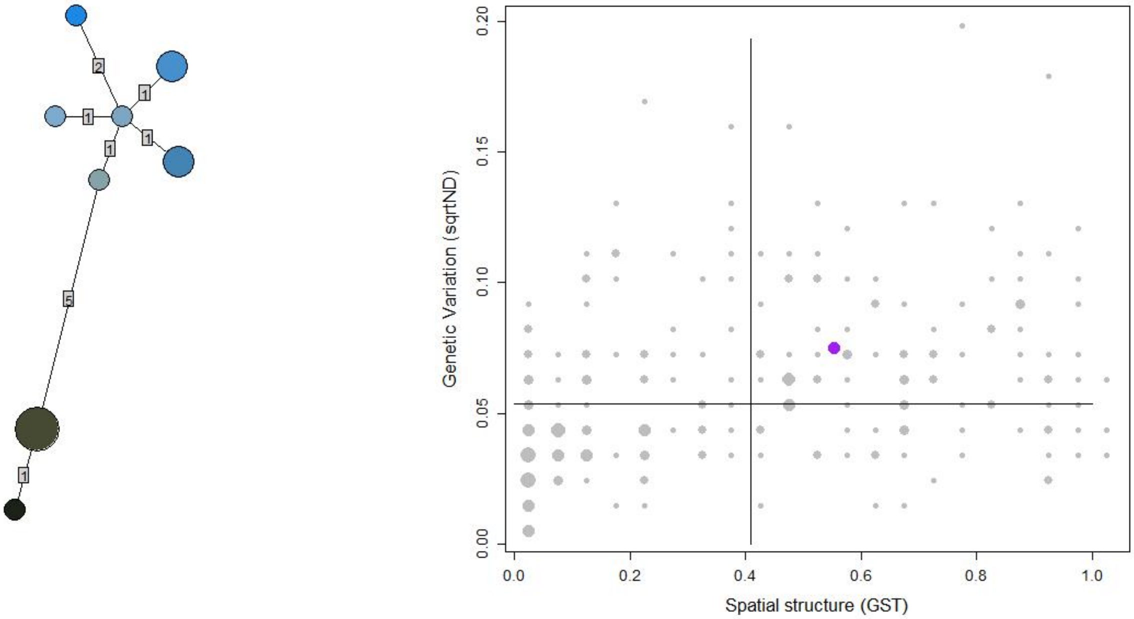

Figure 448: Haplotype network (left) of *Callophrys augustinus* sequences > 599 bp with colours matching the PCoA colour space (above). The bubble plot for mt-DNA polymorphism (square root transformed nucleotide diversity) and spatial structure (GST) among all species in the atlas and values for *Callophrys augustinus* (purple dot). The horizontal and vertical lines represent median values of nucleotide diversity and GST, respectively. Sequences > 599 bp= 30.

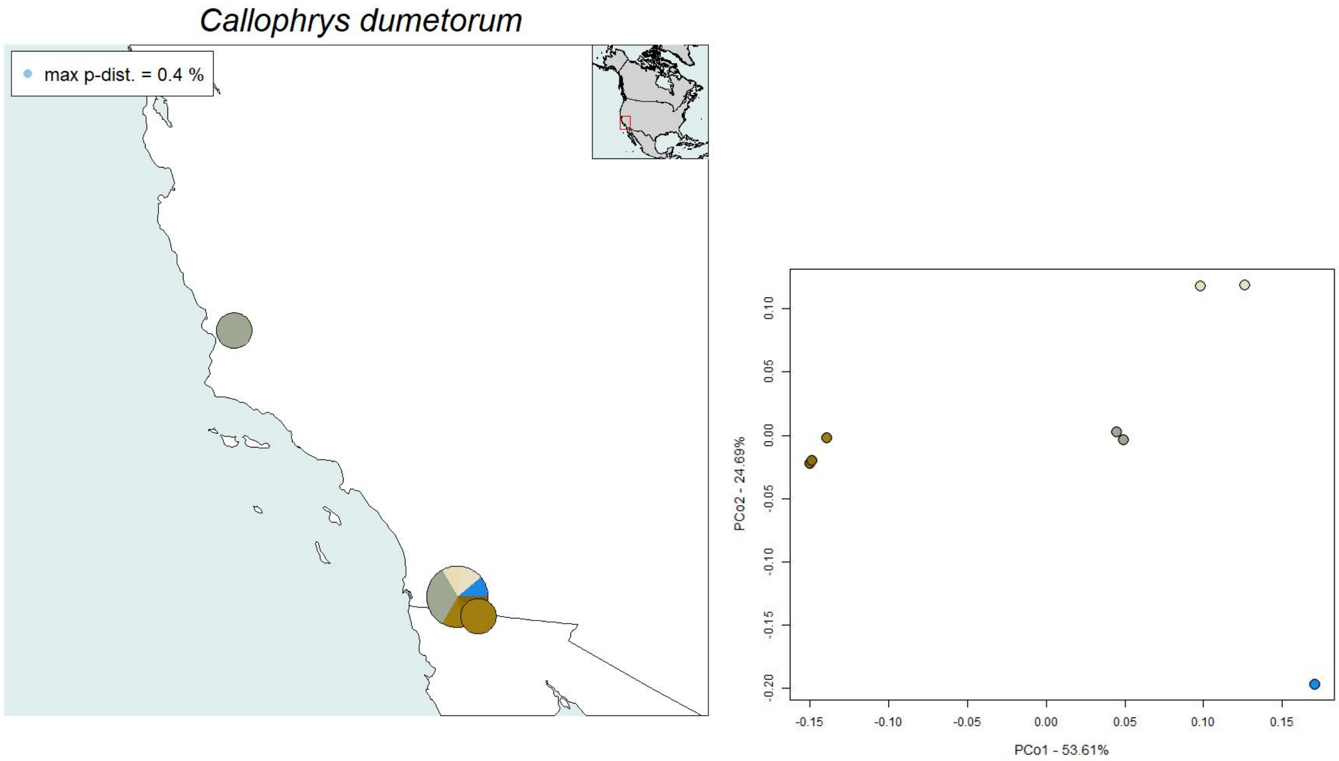

Figure 449 Map of *Callophrys dumetorum* showing the localities of the sequenced specimens (left). Nearby localities are grouped in pies. Colours match the bidimensional colour space of the PCoA projection (right) of max p-dists among sequences (dots). Sequences= 11; Hap obs.= 5; Hap asympt.= 7.7; Hap % obs.= 64.7%; GST= NaN; DST= NaN; HD= 0.782; ND= 0.0018; max p-dist= 0.4%.

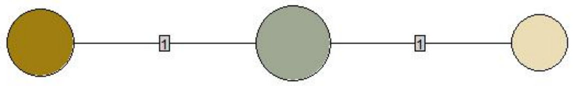

Figure 450: Haplotype network of *Callophrys dumetorum*. Sequences > 599 bp= 6.

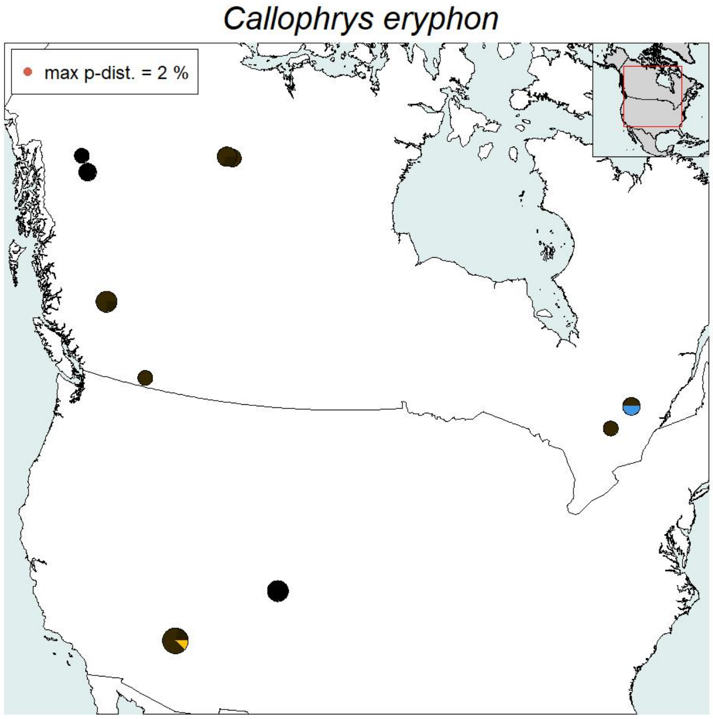

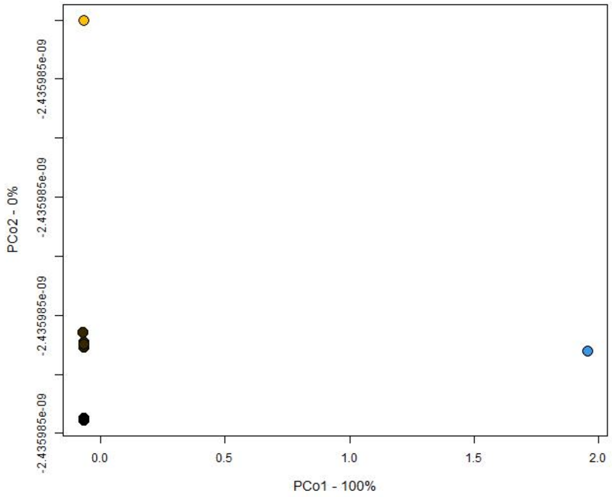

Figure 451 Map of *Callophrys eryphon* showing the localities of the sequenced specimens (left). Nearby localities are grouped in pies. Colours match the bidimensional colour space of the PCoA projection (right) of max p-dists among sequences (dots). Sequences= 29; Hap obs.= 2; Hap asympt.= 2; Hap % obs.= 100%; GST= 0.077; DST= 0.0001; HD= 0.069; ND= 0.0014; max p-dist= 2%.

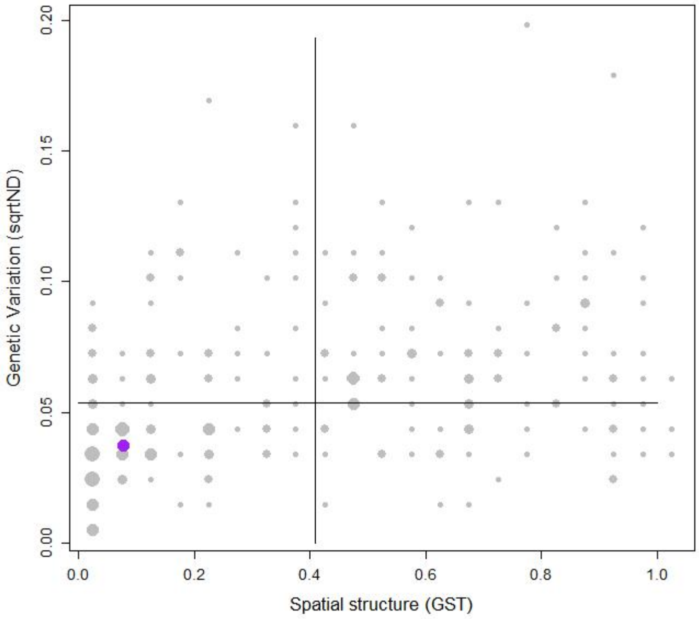

Figure 452: The bubble plot for mt-DNA polymorphism (square root transformed nucleotide diversity) and spatial structuree (GST) among all species in the atlas and values for *Callophrys eryphon* (purple dot). The horizontal and vertical lines represent median values of nucleotide diversity and GST, respectively. Haplotype network analysis was not possible. Sequences > 599 bp = 28.

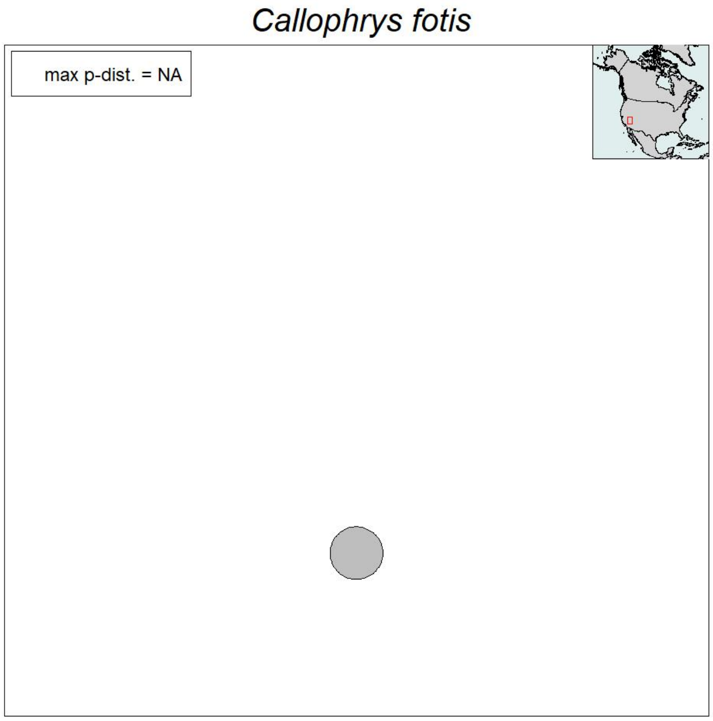

Figure 453: Map of *Callophrys fotis* showing the localities of the sequenced specimens. Nearby localities are grouped in pies. Due to the presence of a single haplotype PCoA projection was not done and a single grey colour was plotted on the map. Sequences= 1; Hap obs.= NA; Hap asympt.= NA; Hap % obs.= NA; GST= NaN; DST= NaN; HD= NA; ND= NA; max p-dist= NA.

Haplotype network analysis and bubble plot of *Callophrys fotis* were not possible. Sequences > 599 bp = 1.

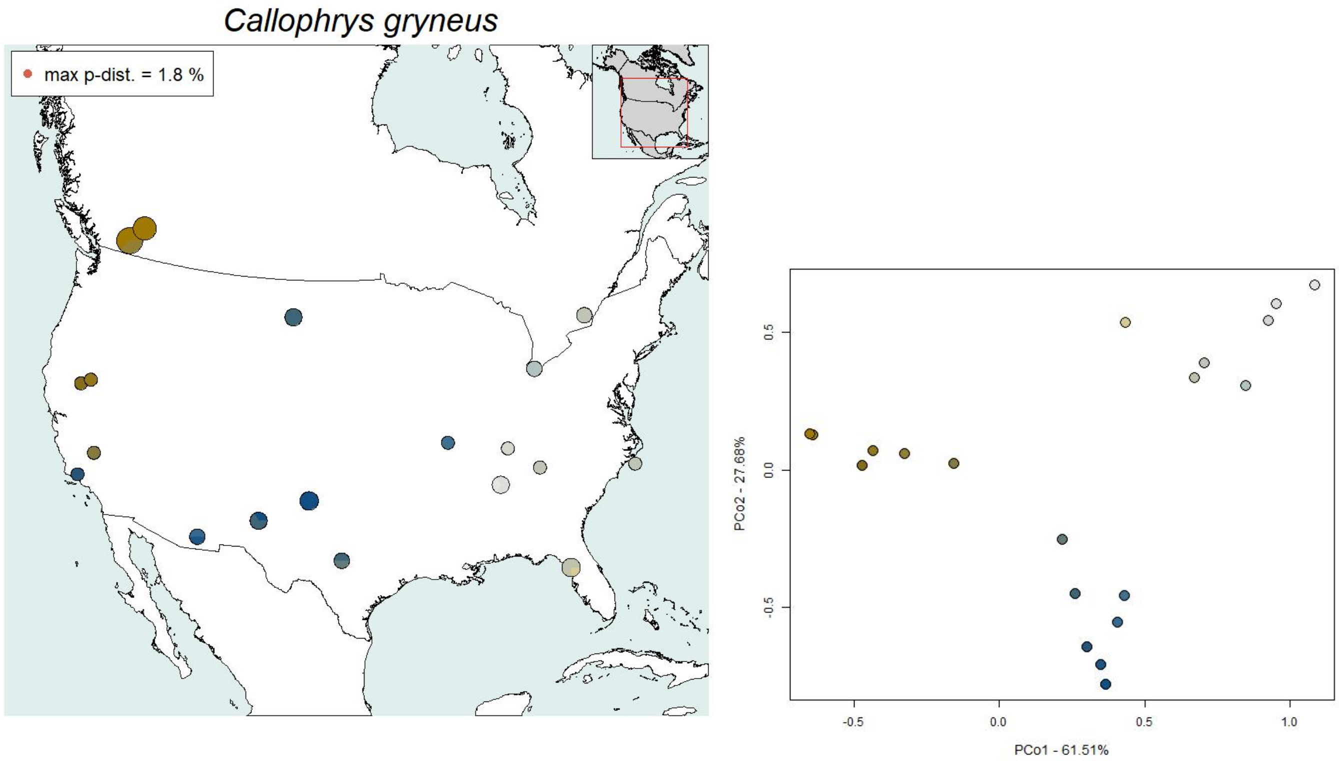

Figure 454 Map of *Callophrys gryneus* showing the localities of the sequenced specimens (left). Nearby localities are grouped in pies. Colours match the bidimensional colour space of the PCoA projection (right) of max p-dists among sequences (dots). Sequences= 59; Hap obs.= 18; Hap asympt.= 33.7; Hap % obs.= 53.4%; GST= 0.88; DST= 0.0072; HD= 0.911; ND= 0.0088; max p-dist= 1.8%.

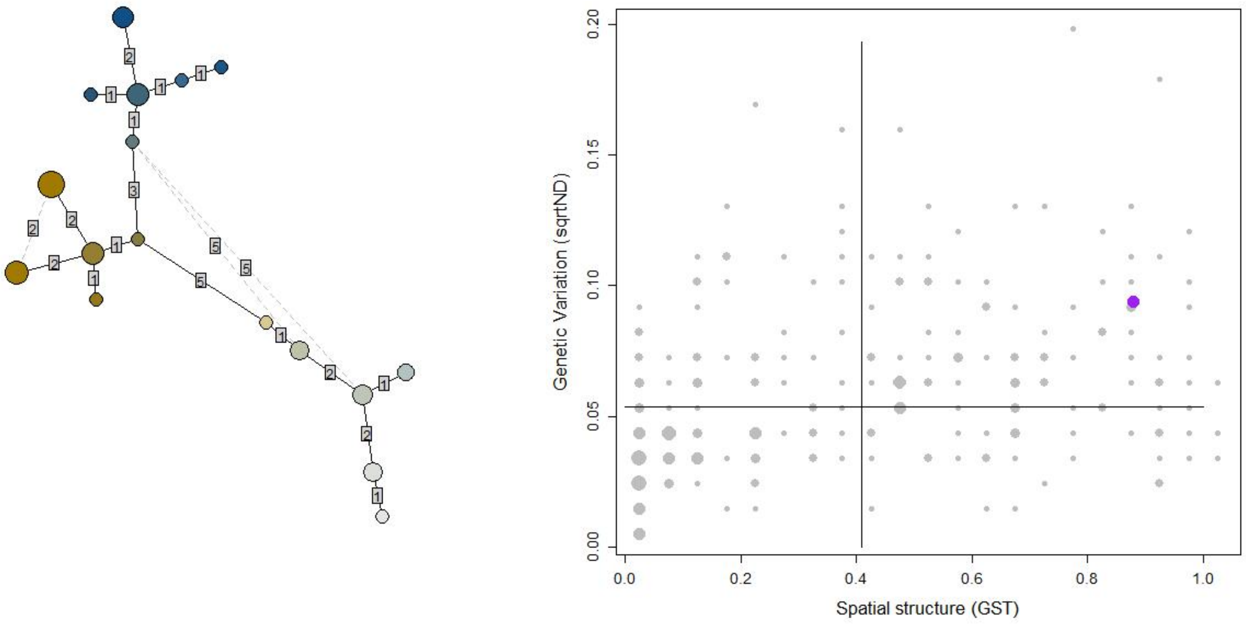

Figure 455: Haplotype network (left) of *Callophrys gryneus* sequences > 599 bp with colours matching the PCoA colour space (above). The bubble plot for mt-DNA polymorphism (square root transformed nucleotide diversity) and spatial structure (GST) among all species in the atlas and values for *Callophrys gryneus* (purple dot). The horizontal and vertical lines represent median values of nucleotide diversity and GST, respectively. Sequences > 599 bp= 57.

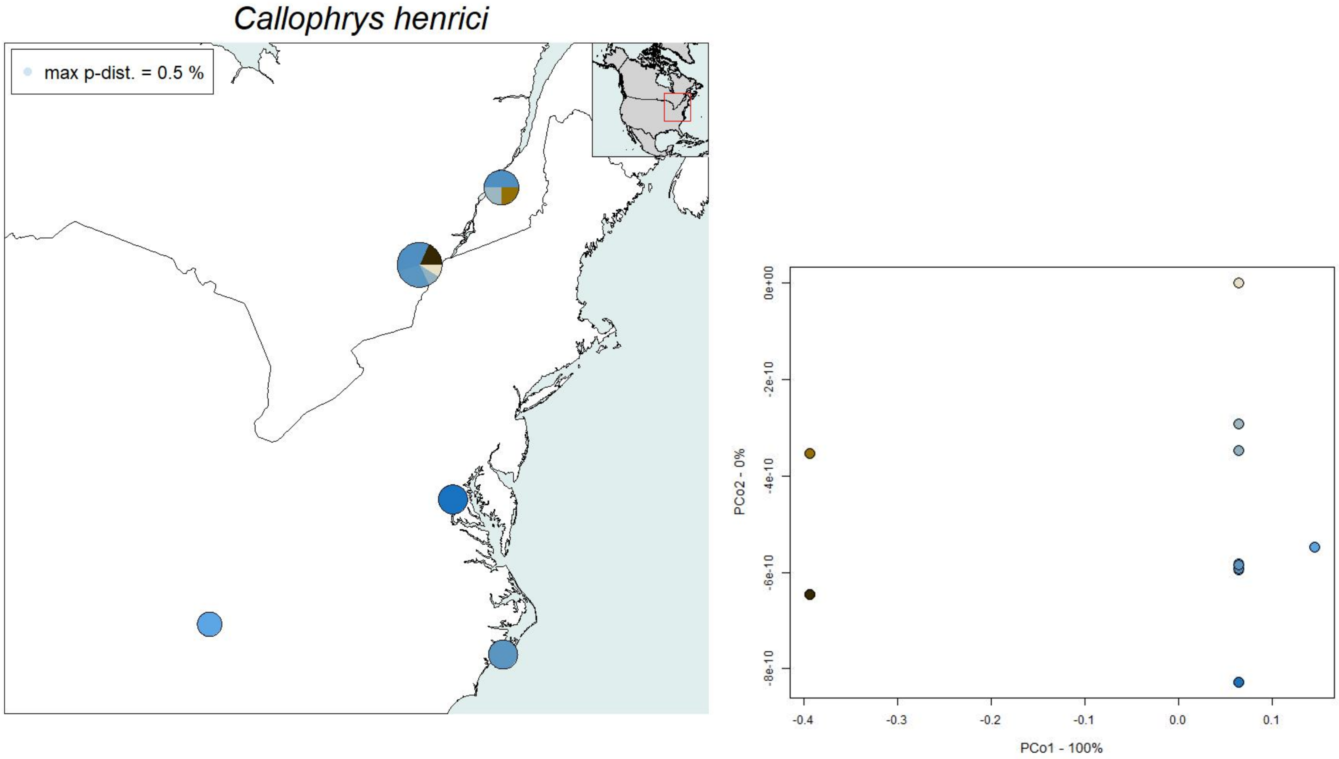

Figure 456 Map of *Callophrys henrici* showing the localities of the sequenced specimens (left). Nearby localities are grouped in pies. Colours match the bidimensional colour space of the PCoA projection (right) of max p-dists among sequences (dots). Sequences= 20; Hap obs.= 2; Hap asympt.= 2; Hap % obs.= 100%; GST= 0; DST= 0; HD= 0.268; ND= 0.0012; max p-dist= 0.5%.

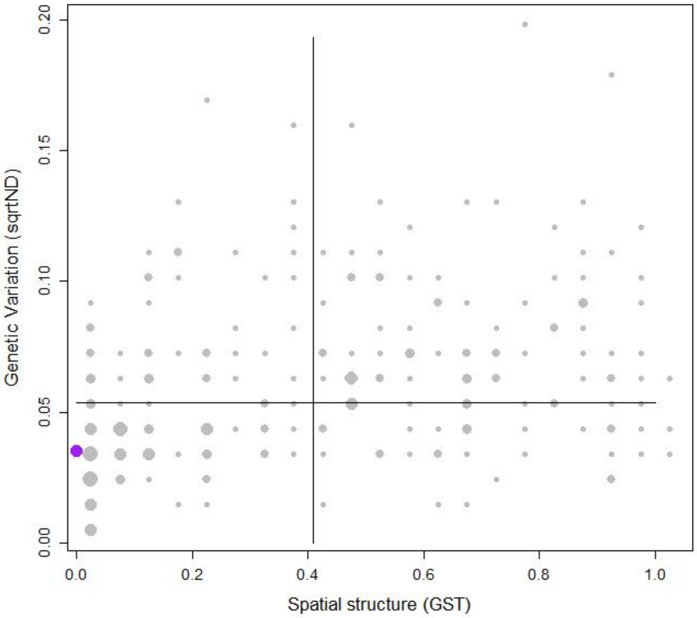

Figure 457: The bubble plot for mt-DNA polymorphism (square root transformed nucleotide diversity) and spatial structuree (GST) among all species in the atlas and values for *Callophrys henrici* (purple dot). The horizontal and vertical lines represent median values of nucleotide diversity and GST, respectively. Haplotype network analysis was not possible. Sequences > 599 bp = 19.

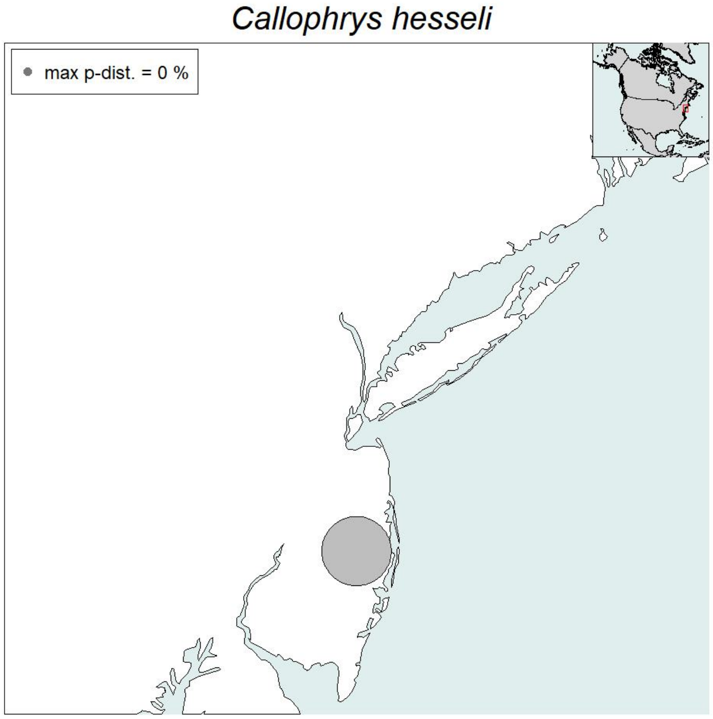

Figure 458: Map of *Callophrys hesseli* showing the localities of the sequenced specimens. Nearby localities are grouped in pies. Due to the presence of a single haplotype PCoA projection was not done and a single grey colour was plotted on the map. Sequences= 3; Hap obs.= 1; Hap asympt.= NA; Hap % obs.= NA%; GST= NaN; DST= NaN; HD= NA; ND= NA; max p-dist= 0%.

Haplotype network analysis and bubble plot of *Callophrys hesseli* were not possible. Sequences > 599 bp = 3.

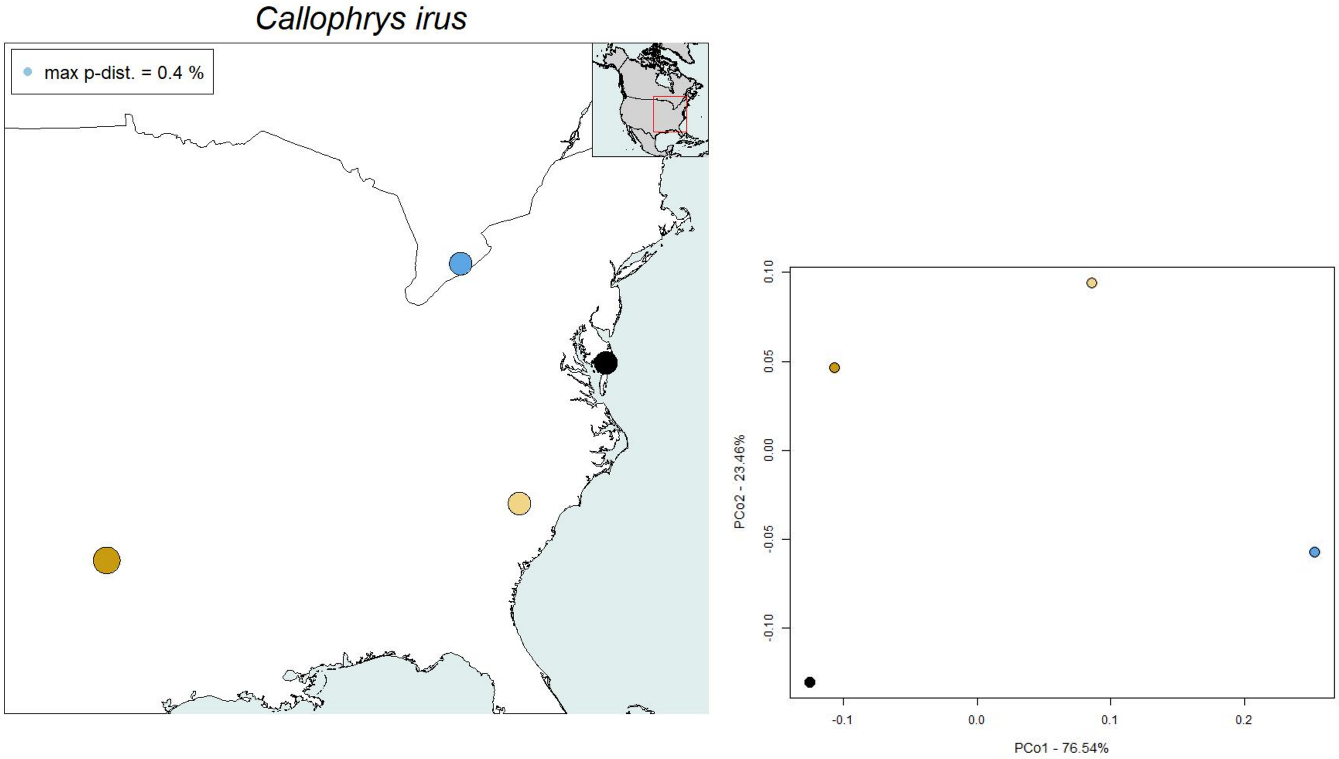

Figure 459 Map of *Callophrys irus* showing the localities of the sequenced specimens (left). Nearby localities are grouped in pies. Colours match the bidimensional colour space of the PCoA projection (right) of max p-dists among sequences (dots). Sequences= 5; Hap obs.= 4; Hap asympt.= NA; Hap % obs.= NA%; GST= NaN; DST= NaN; HD= NA; ND= NA; max p-dist= 0.4%.

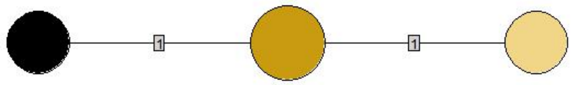

Figure 460: Haplotype network of *Callophrys irus*. Sequences > 599 bp= 4.

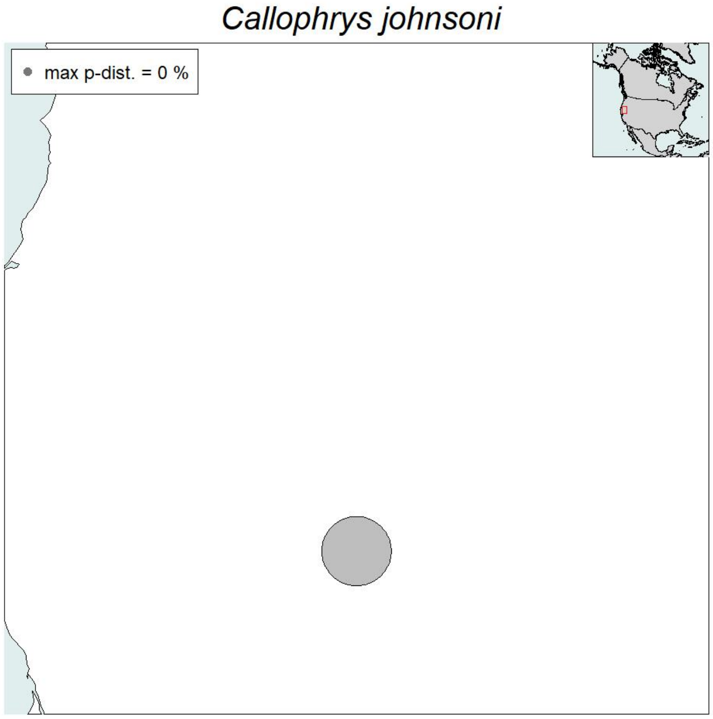

Figure 461: Map of *Callophrys johnsoni* showing the localities of the sequenced specimens. Nearby localities are grouped in pies. Due to the presence of a single haplotype PCoA projection was not done and a single grey colour was plotted on the map. Sequences= 3; Hap obs.= 1; Hap asympt.= NA; Hap % obs.= NA%; GST= NaN; DST= NaN; HD= NA; ND= NA; max p-dist= 0%.

Haplotype network analysis and bubble plot of *Callophrys johnsoni* were not possible. Sequences > 599 bp = 3.

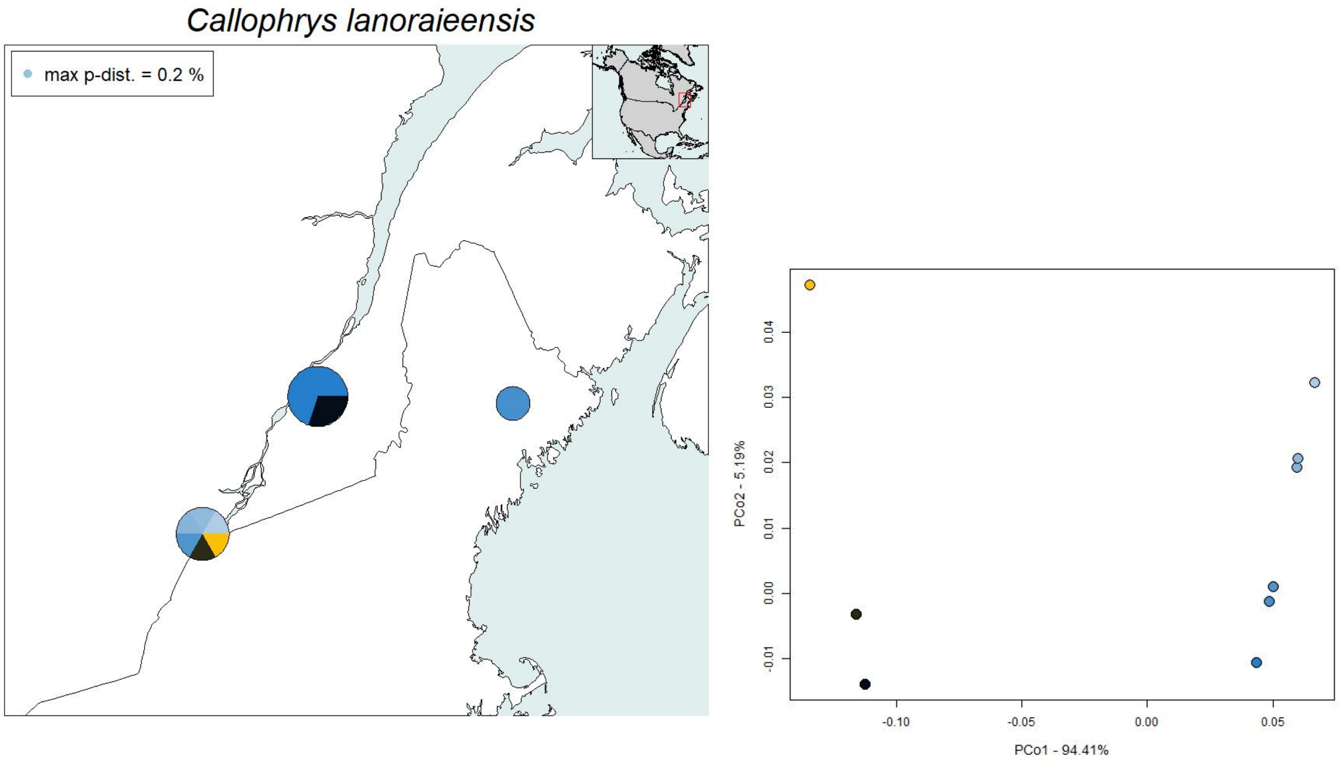

Figure 462 Map of *Callophrys lanoraieensis* showing the localities of the sequenced specimens (left). Nearby localities are grouped in pies. Colours match the bidimensional colour space of the PCoA projection (right) of max p-dists among sequences (dots). Sequences= 17; Hap obs.= 2; Hap asympt.= 2; Hap % obs.= 100%; GST= 0; DST= 0; HD= 0.441; ND= 0.0007; max p-dist= 0.2%.

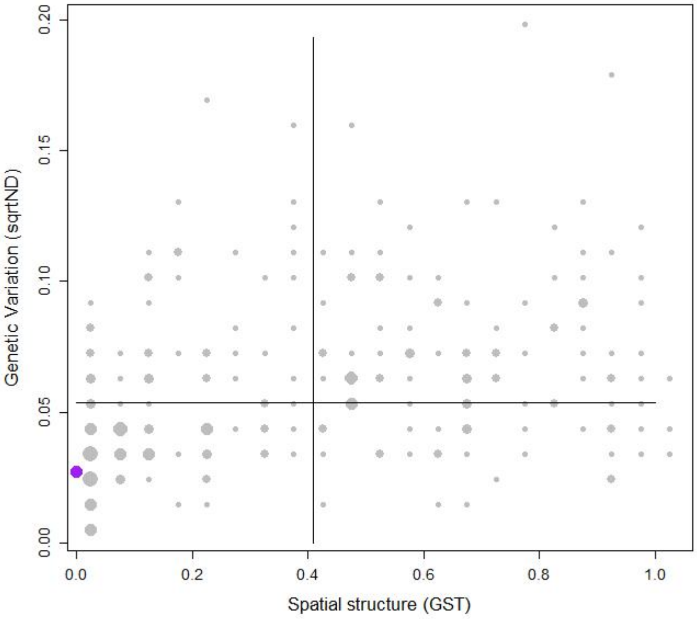

Figure 463: The bubble plot for mt-DNA polymorphism (square root transformed nucleotide diversity) and spatial structuree (GST) among all species in the atlas and values for *Callophrys lanoraieensis* (purple dot). The horizontal and vertical lines represent median values of nucleotide diversity and GST, respectively. Haplotype network analysis was not possible. Sequences > 599 bp = 13.

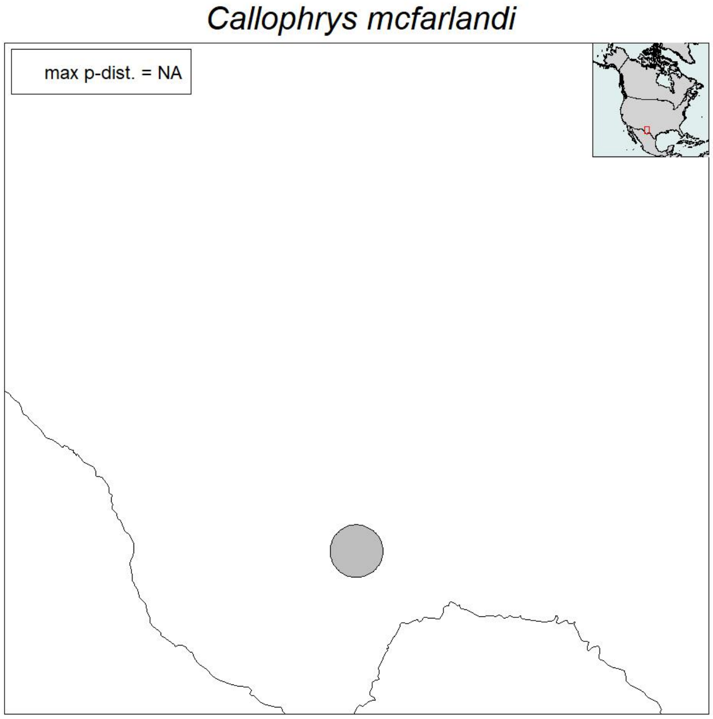

Figure 464: Map of *Callophrys mcfarlandi* showing the localities of the sequenced specimens. Nearby localities are grouped in pies. Due to the presence of a single haplotype PCoA projection was not done and a single grey colour was plotted on the map. Sequences= 1; Hap obs.= NA; Hap asympt.= NA; Hap % obs.= NA; GST= NaN; DST= NaN; HD= NA; ND= NA; max p-dist= NA.

Haplotype network analysis and bubble plot of *Callophrys mcfarlandi* were not possible. Sequences > 599 bp = 1.

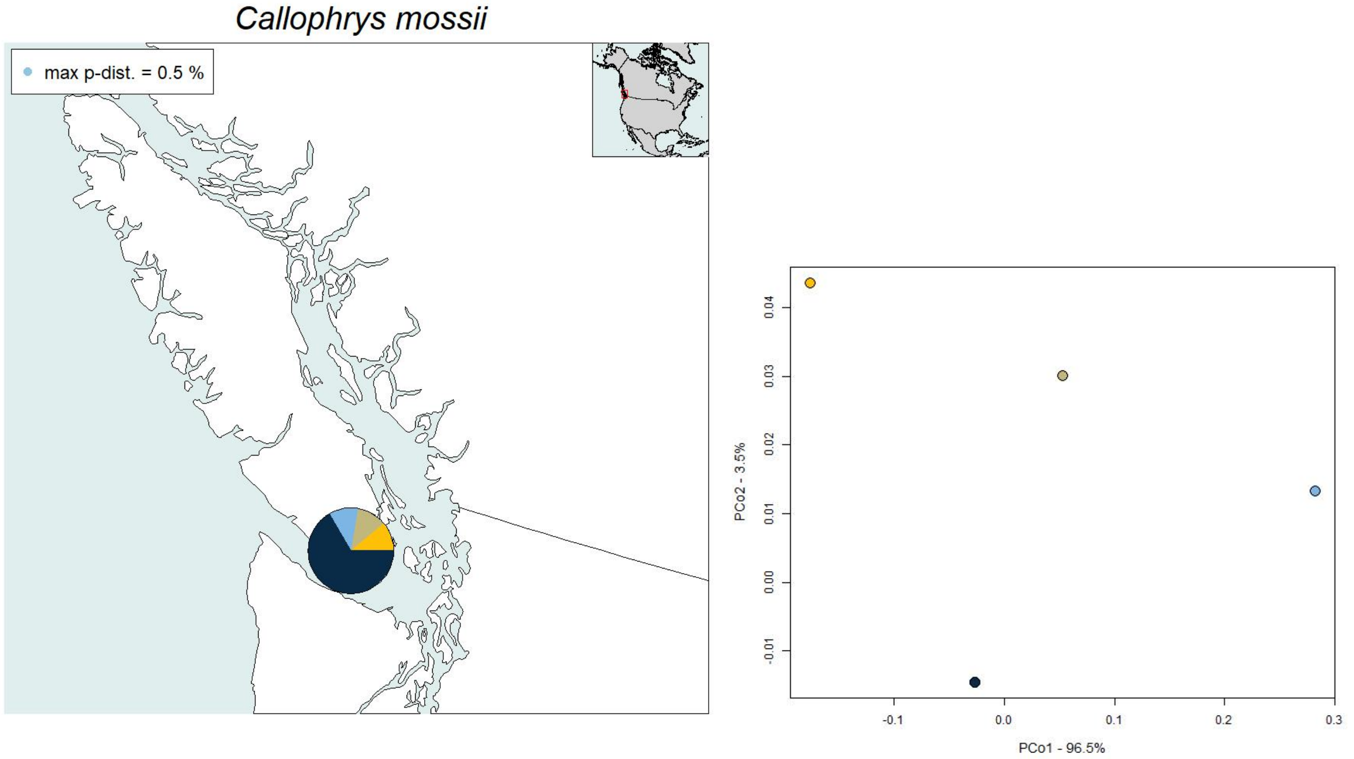

Figure 465 Map of *Callophrys mossii* showing the localities of the sequenced specimens (left). Nearby localities are grouped in pies. Colours match the bidimensional colour space of the PCoA projection (right) of max p-dists among sequences (dots). Sequences= 9; Hap obs.= 3; Hap asympt.= NA; Hap % obs.= NA%; GST= NaN; DST= NaN; HD= NA; ND= NA; max p-dist= 0.5%.

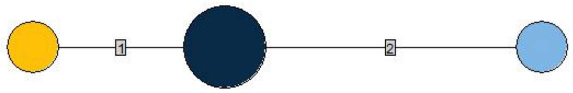

Figure 466: Haplotype network of *Callophrys mossii*. Sequences > 599 bp= 8.

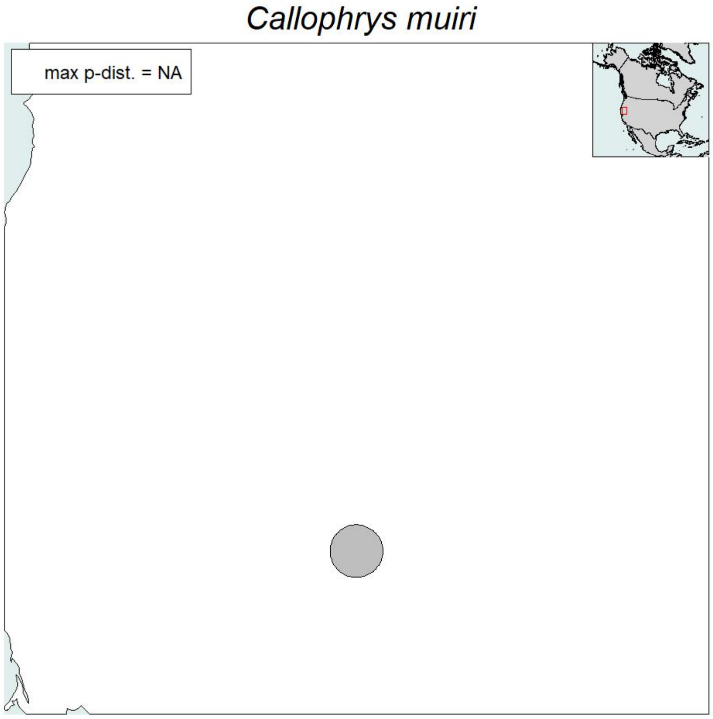

Figure 467: Map of *Callophrys muiri* showing the localities of the sequenced specimens. Nearby localities are grouped in pies. Due to the presence of a single haplotype PCoA projection was not done and a single grey colour was plotted on the map. Sequences= 1; Hap obs.= NA; Hap asympt.= NA; Hap % obs.= NA; GST= NaN; DST= NaN; HD= NA; ND= NA; max p-dist= NA.

Haplotype network analysis and bubble plot of *Callophrys muiri* were not possible. Sequences > 599 bp = 1.

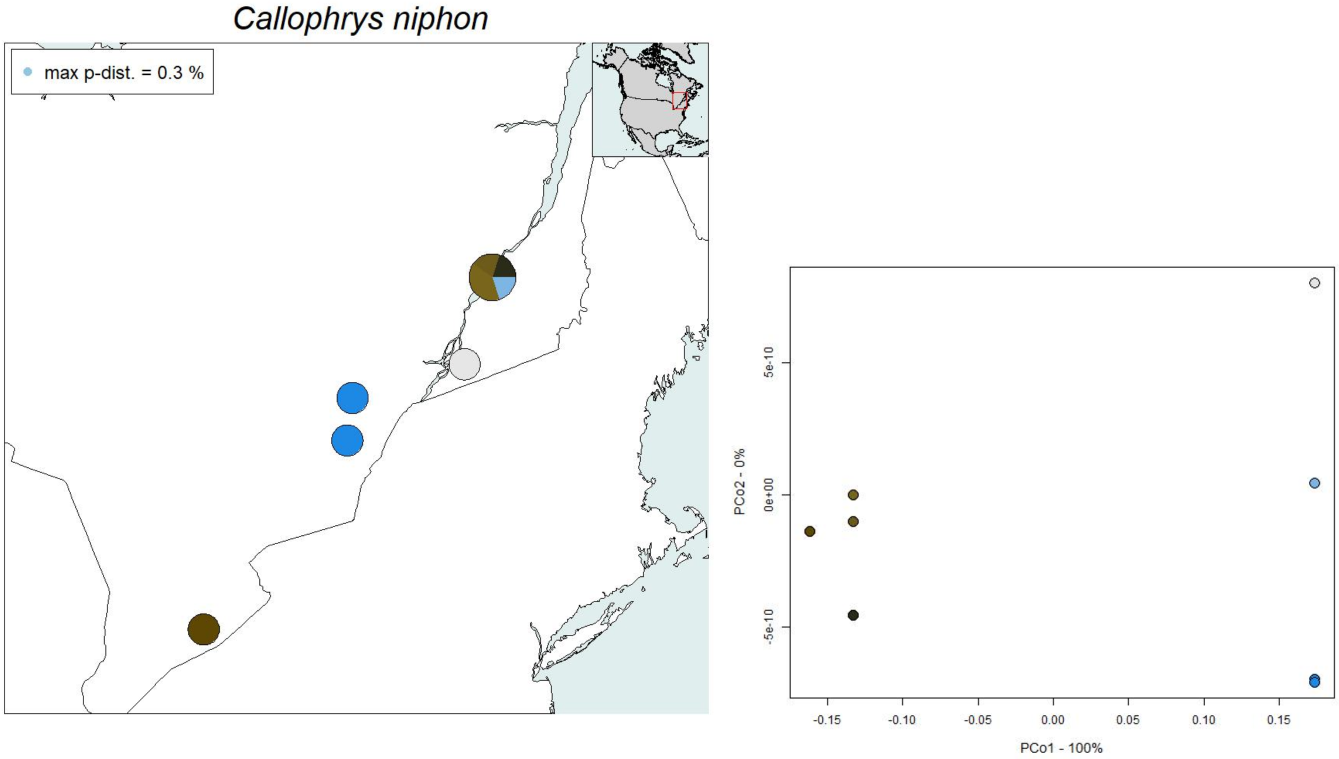

Figure 468 Map of *Callophrys niphon* showing the localities of the sequenced specimens (left). Nearby localities are grouped in pies. Colours match the bidimensional colour space of the PCoA projection (right) of max p-dists among sequences (dots). Sequences= 9; Hap obs.= 2; Hap asympt.= NA; Hap % obs.= NA%; GST= NaN; DST= NaN; HD= NA; ND= NA; max p-dist= 0.3%.

Haplotype network analysis and bubble plot of *Callophrys niphon* were not possible. Sequences > 599 bp = 8.

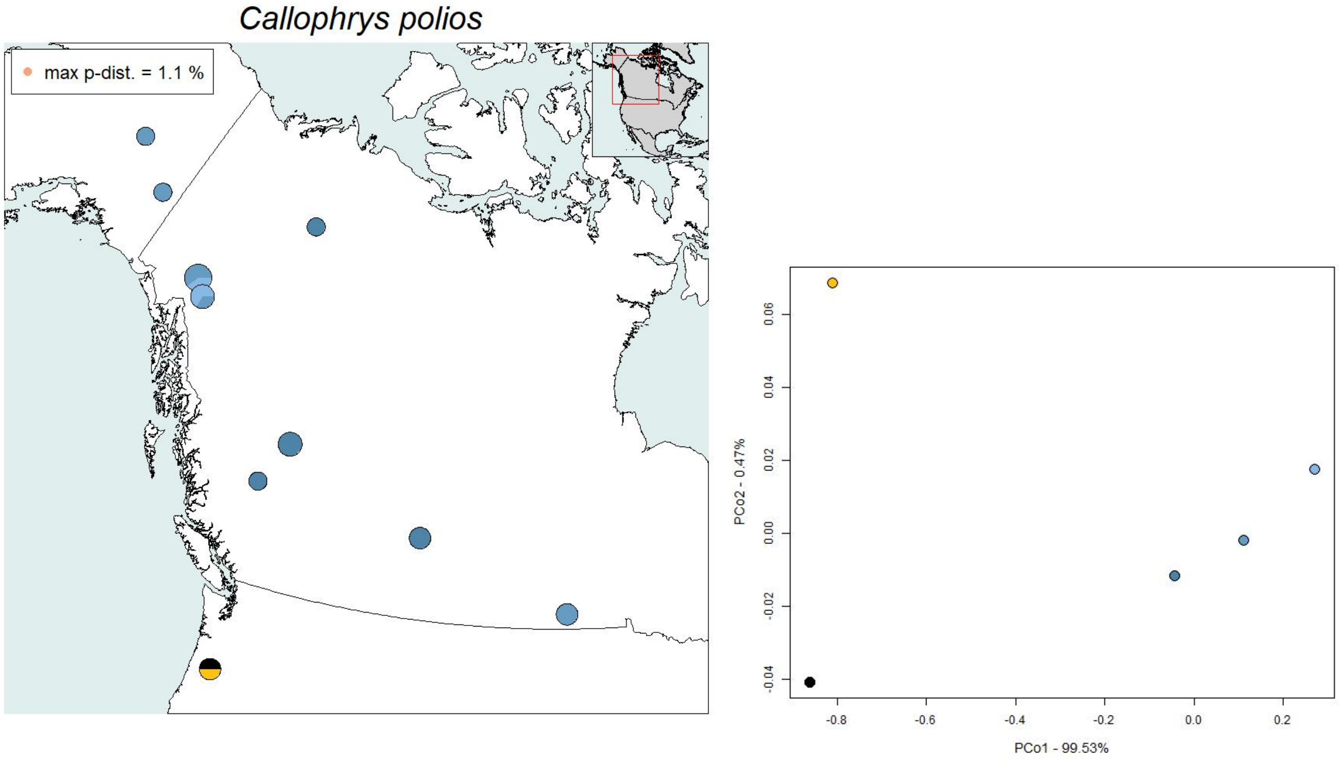

Figure 469 Map of *Callophrys polios* showing the localities of the sequenced specimens (left). Nearby localities are grouped in pies. Colours match the bidimensional colour space of the PCoA projection (right) of max p-dists among sequences (dots). Sequences= 21; Hap obs.= 4; Hap asympt.= 4; Hap % obs.= 100%; GST= 0.474; DST= 0.0007; HD= 0.733; ND= 0.0027; max p-dist= 1.1%.

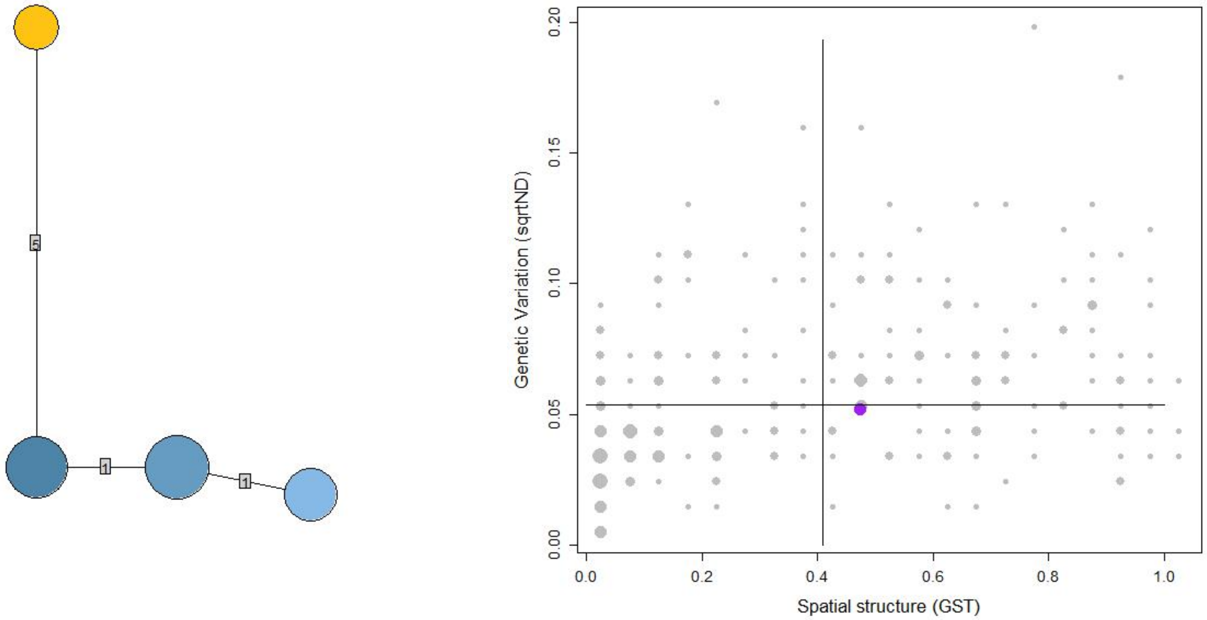

Figure 470: Haplotype network (left) of *Callophrys polios* sequences > 599 bp with colours matching the PCoA colour space (above). The bubble plot for mt-DNA polymorphism (square root transformed nucleotide diversity) and spatial structure (GST) among all species in the atlas and values for *Callophrys polios* (purple dot). The horizontal and vertical lines represent median values of nucleotide diversity and GST, respectively. Sequences > 599 bp= 21.

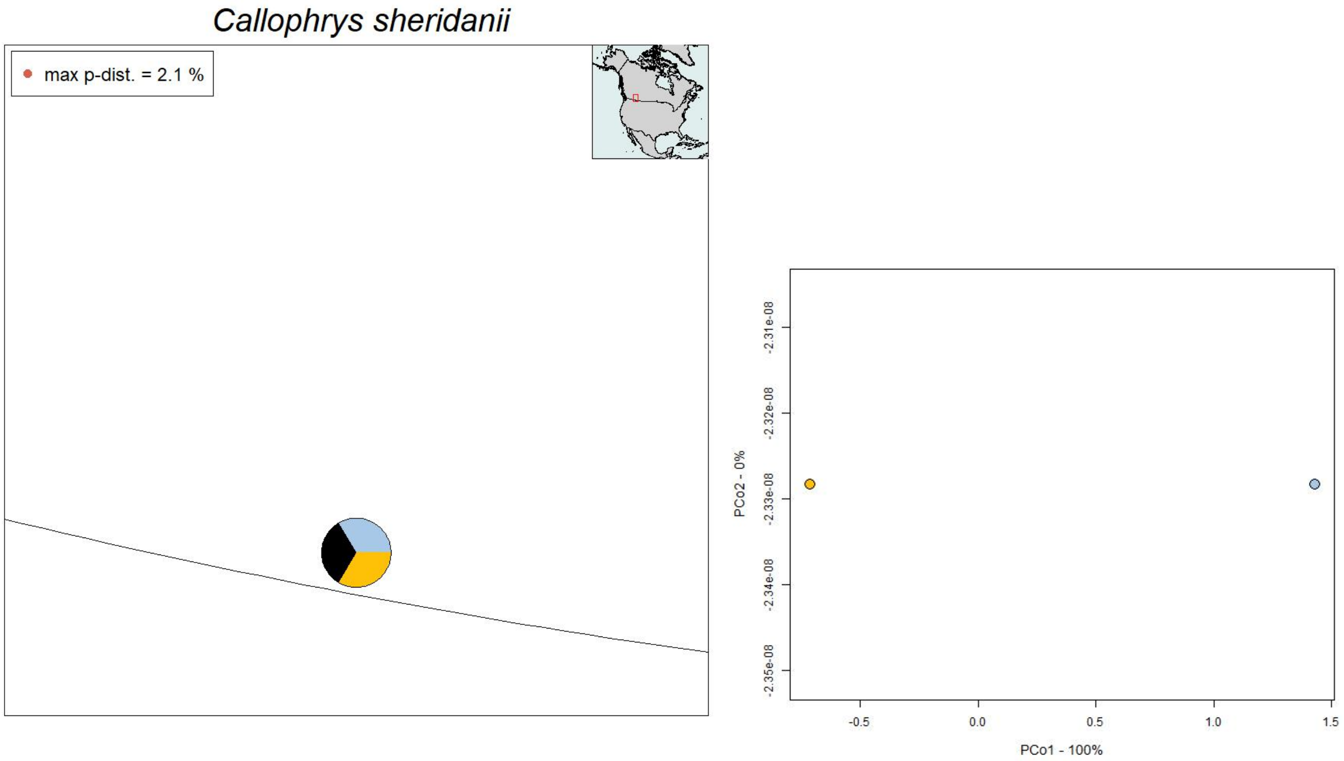

Figure 471 Map of *Callophrys sheridanii* showing the localities of the sequenced specimens (left). Nearby localities are grouped in pies. Colours match the bidimensional colour space of the PCoA projection (right) of max p-dists among sequences (dots). Sequences= 3; Hap obs.= 2; Hap asympt.= NA; Hap % obs.= NA%; GST= NaN; DST= NaN; HD= NA; ND= NA; max p-dist= 2.1%.

Haplotype network analysis and bubble plot of *Callophrys sheridanii* were not possible. Sequences > 599 bp = 3.

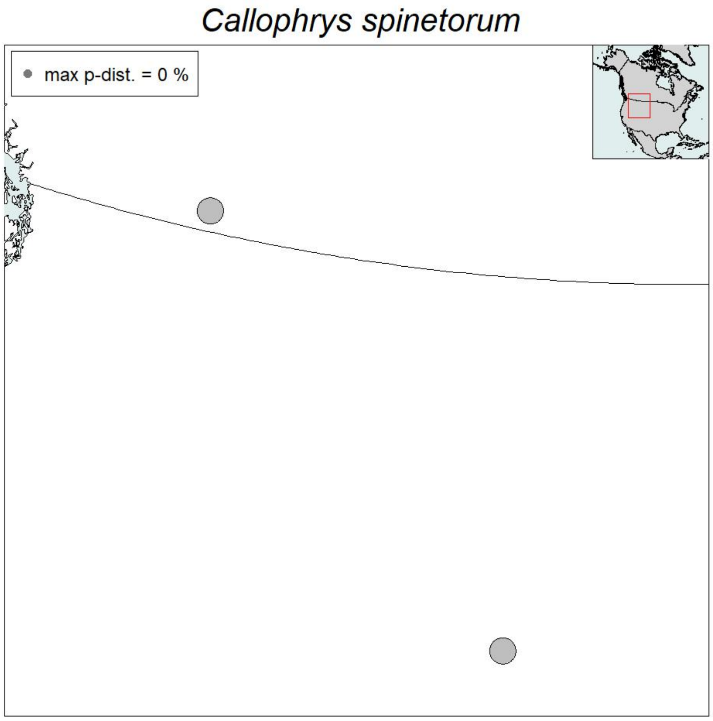

Figure 472: Map of *Callophrys spinetorum* showing the localities of the sequenced specimens. Nearby localities are grouped in pies. Due to the presence of a single haplotype PCoA projection was not done and a single grey colour was plotted on the map. Sequences= 2; Hap obs.= 1; Hap asympt.= NA; Hap % obs.= NA%; GST= NaN; DST= NaN; HD= NA; ND= NA; max p-dist= 0%.

Haplotype network analysis and bubble plot of *Callophrys spinetorum* were not possible. Sequences > 599 bp = 2.

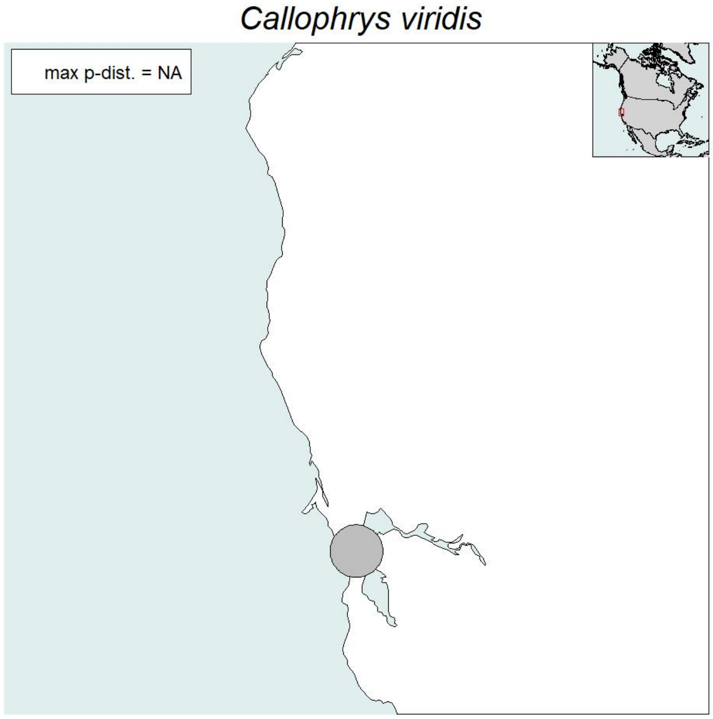

Figure 473: Map of *Callophrys viridis* showing the localities of the sequenced specimens. Nearby localities are grouped in pies. Due to the presence of a single haplotype PCoA projection was not done and a single grey colour was plotted on the map. Sequences= 1; Hap obs.= NA; Hap asympt.= NA; Hap % obs.= NA; GST= NaN; DST= NaN; HD= NA; ND= NA; max p-dist= NA.

Haplotype network analysis and bubble plot of *Callophrys viridis* were not possible. Sequences > 599 bp = 0.

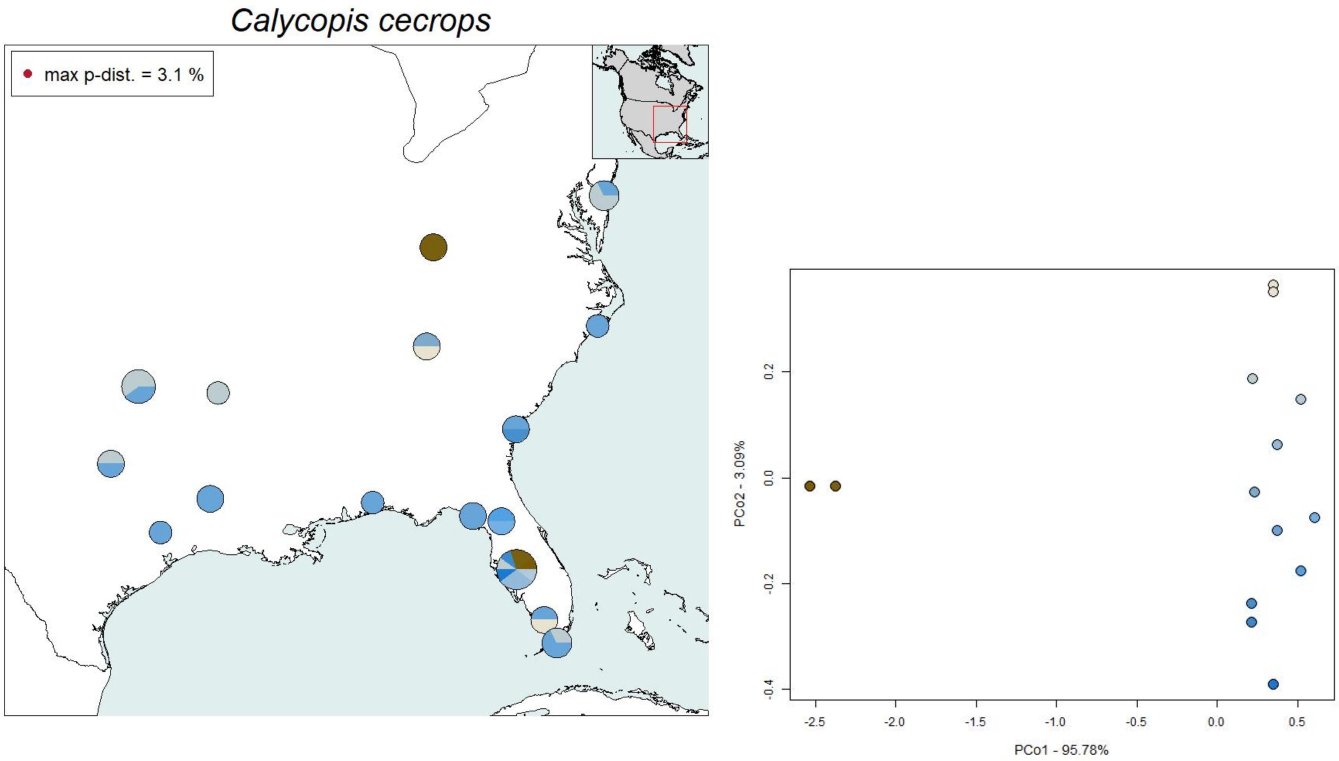

Figure 474 Map of *Calycopis cecrops* showing the localities of the sequenced specimens (left). Nearby localities are grouped in pies. Colours match the bidimensional colour space of the PCoA projection (right) of max p-dists among sequences (dots). Sequences= 41; Hap obs.= 14; Hap asympt.= 33.8; Hap % obs.= 41.5%; GST= 0.126; DST= 0.001; HD= 0.804; ND= 0.0076; max p-dist= 3.1%.

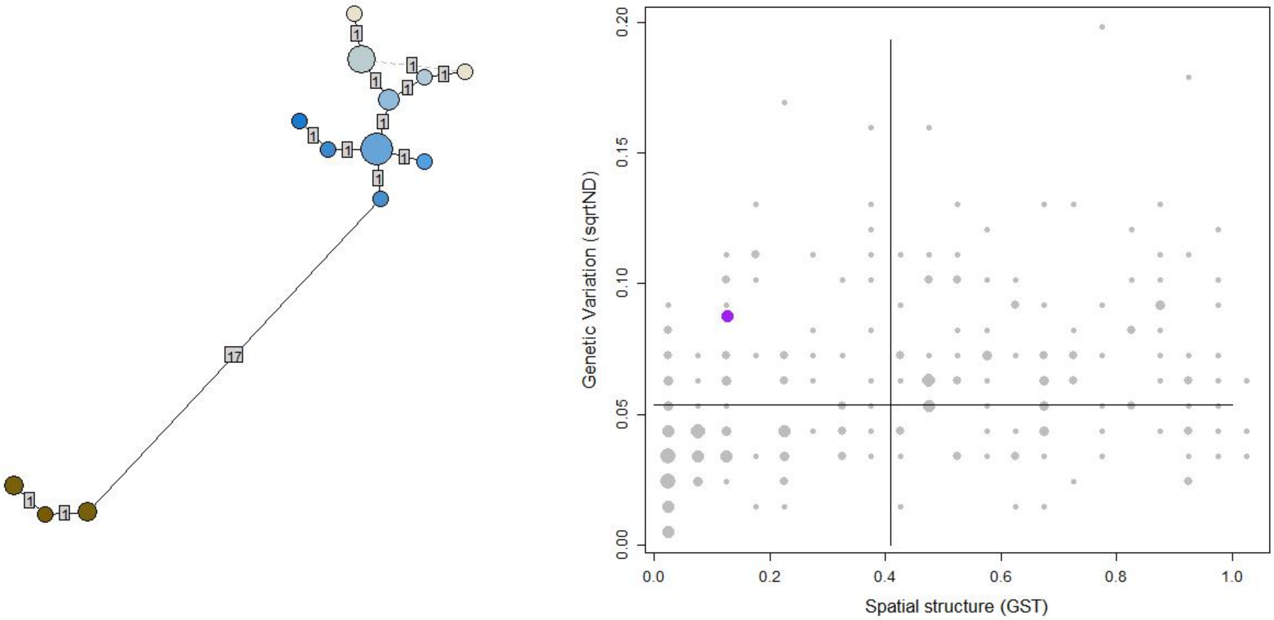

Figure 475: Haplotype network (left) of *Calycopis cecrops* sequences > 599 bp with colours matching the PCoA colour space (above). The bubble plot for mt-DNA polymorphism (square root transformed nucleotide diversity) and spatial structure (GST) among all species in the atlas and values for *Calycopis cecrops* (purple dot). The horizontal and vertical lines represent median values of nucleotide diversity and GST, respectively. Sequences > 599 bp= 41.

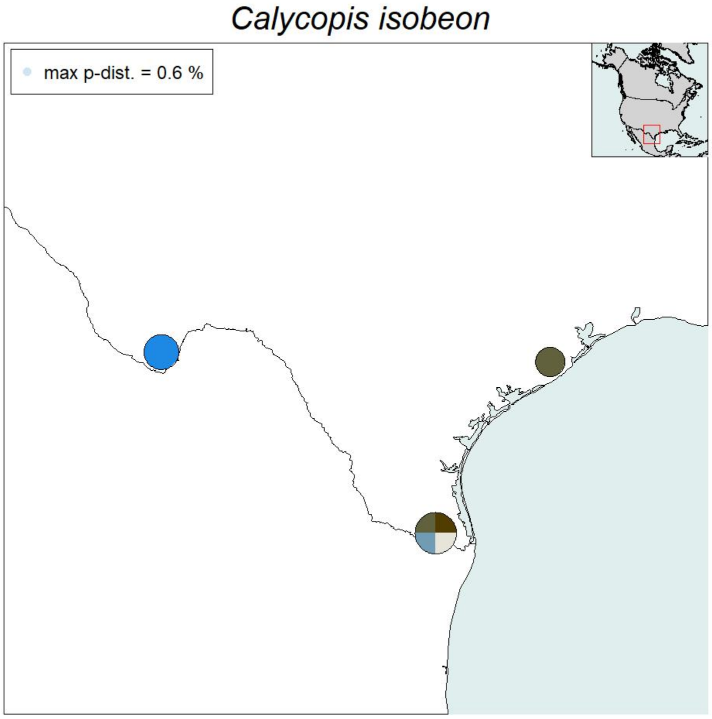

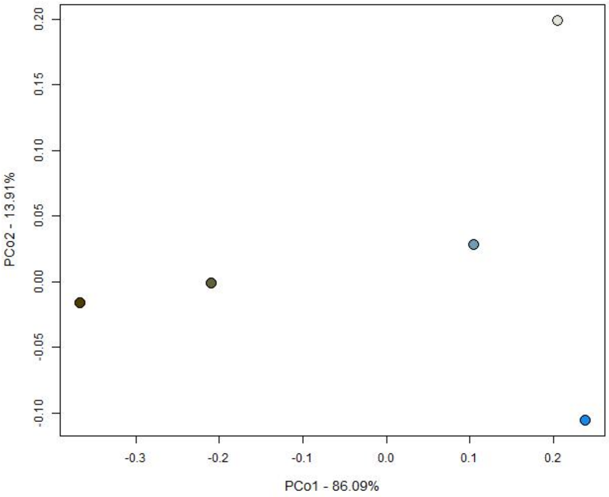

Figure 476 Map of *Calycopis isobeon* showing the localities of the sequenced specimens (left). Nearby localities are grouped in pies. Colours match the bidimensional colour space of the PCoA projection (right) of max p-dists among sequences (dots). Sequences= 7; Hap obs.= 5; Hap asympt.= NA; Hap % obs.= NA%; GST= NaN; DST= NaN; HD= NA; ND= NA; max p-dist= 0.6%.

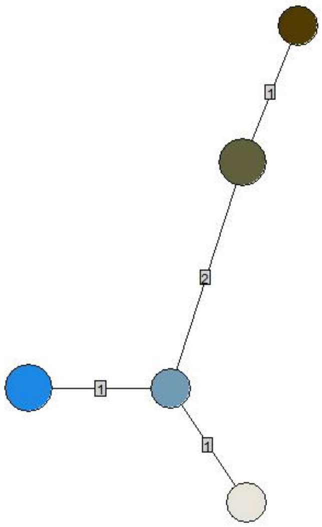

Figure 477: Haplotype network of *Calycopis isobeon*. Sequences > 599 bp= 7.

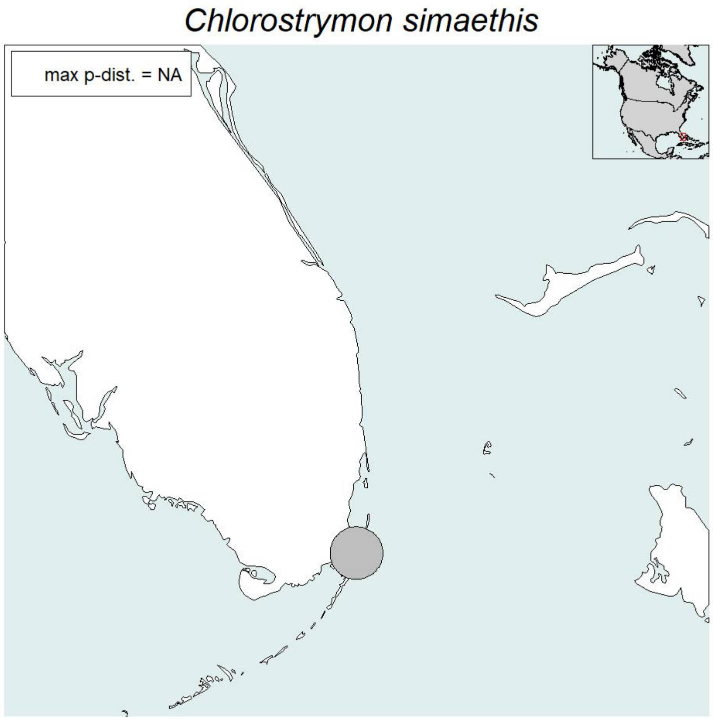

Figure 478: Map of *Chlorostrymon simaethis* showing the localities of the sequenced specimens. Nearby localities are grouped in pies. Due to the presence of a single haplotype PCoA projection was not done and a single grey colour was plotted on the map. Sequences= 1; Hap obs.= NA; Hap asympt.= NA; Hap % obs.= NA; GST= NaN; DST= NaN; HD= NA; ND= NA; max p-dist= NA.

Haplotype network analysis and bubble plot of *Chlorostrymon simaethis* were not possible. Sequences > 599 bp = 1.

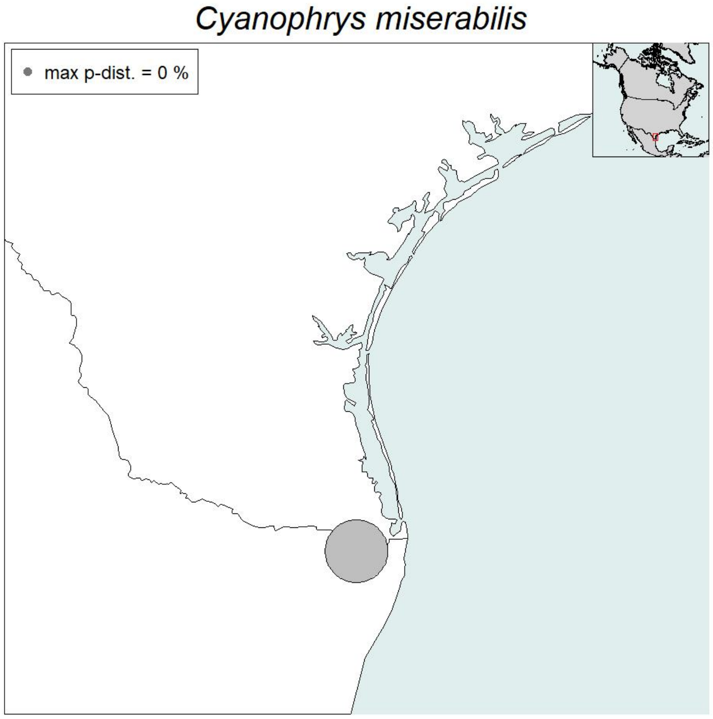

Figure 479: Map of *Cyanophrys miserabilis* showing the localities of the sequenced specimens. Nearby localities are grouped in pies. Due to the presence of a single haplotype PCoA projection was not done and a single grey colour was plotted on the map. Sequences= 2; Hap obs.= 1; Hap asympt.= NA; Hap % obs.= NA%; GST= NaN; DST= NaN; HD= NA; ND= NA; max p-dist= 0%.

Haplotype network analysis and bubble plot of *Cyanophrys miserabilis* were not possible. Sequences > 599 bp = 2.

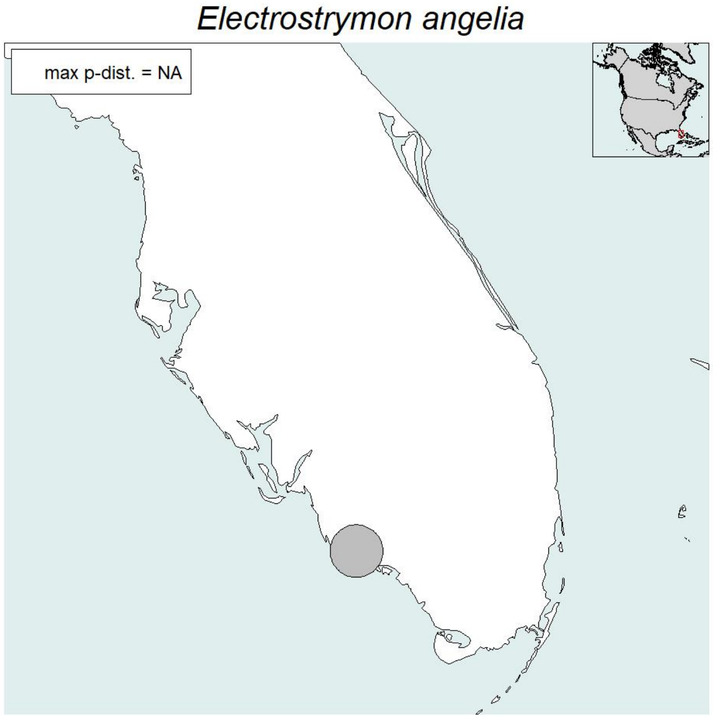

Figure 480: Map of *Electrostrymon angelia* showing the localities of the sequenced specimens. Nearby localities are grouped in pies. Due to the presence of a single haplotype PCoA projection was not done and a single grey colour was plotted on the map. Sequences= 1; Hap obs.= NA; Hap asympt.= NA; Hap % obs.= NA; GST= NaN; DST= NaN; HD= NA; ND= NA; max p-dist= NA.

Haplotype network analysis and bubble plot of *Electrostrymon angelia* were not possible. Sequences > 599 bp = 1.

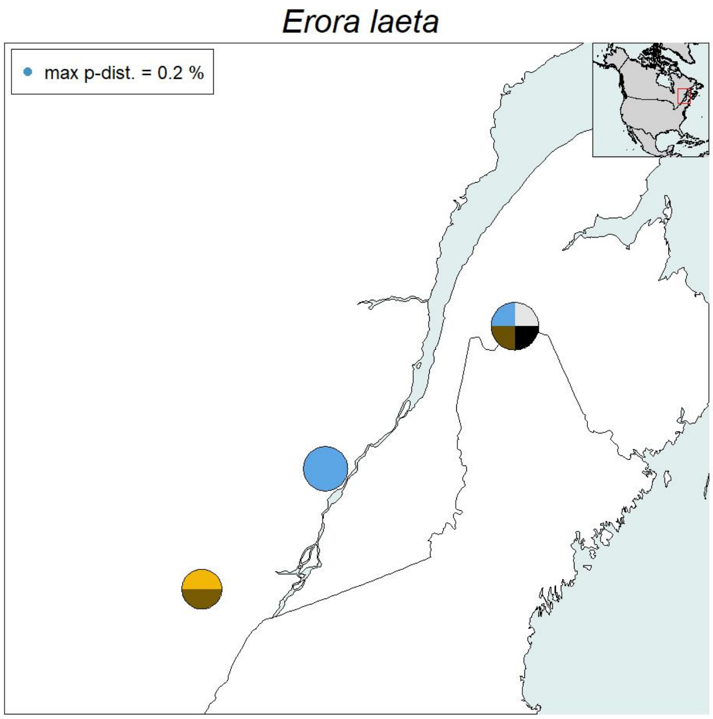

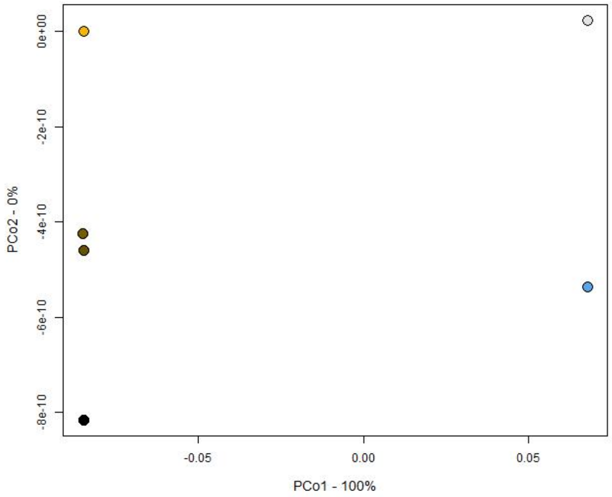

Figure 481 Map of *Erora laeta* showing the localities of the sequenced specimens (left). Nearby localities are grouped in pies. Colours match the bidimensional colour space of the PCoA projection (right) of max p-dists among sequences (dots). Sequences= 9; Hap obs.= 2; Hap asympt.= NA; Hap % obs.= NA%; GST= NaN; DST= NaN; HD= NA; ND= NA; max p-dist= 0.2%.

Haplotype network analysis and bubble plot of *Erora laeta* were not possible. Sequences > 599 bp = 9.

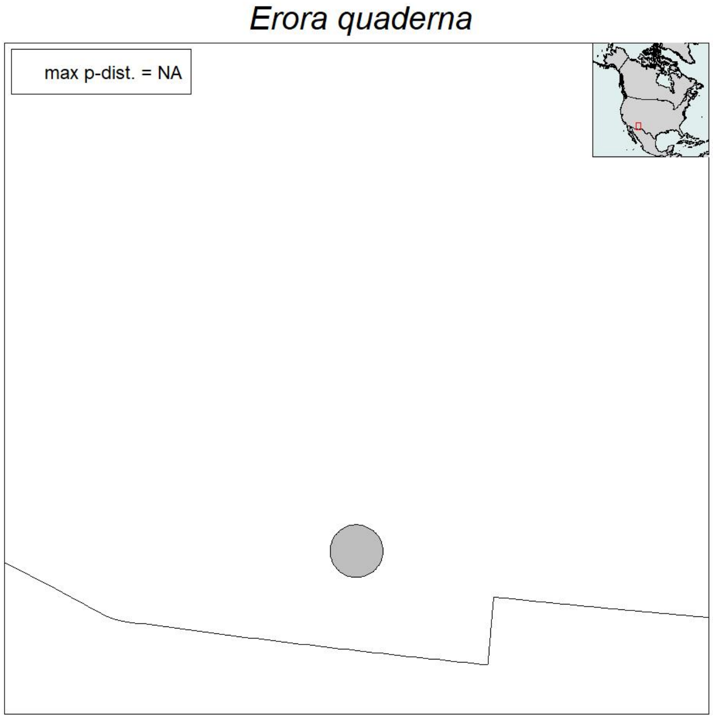

Figure 482: Map of *Erora quaderna* showing the localities of the sequenced specimens. Nearby localities are grouped in pies. Due to the presence of a single haplotype PCoA projection was not done and a single grey colour was plotted on the map. Sequences= 1; Hap obs.= NA; Hap asympt.= NA; Hap % obs.= NA; GST= NaN; DST= NaN; HD= NA; ND= NA; max p-dist= NA.

Haplotype network analysis and bubble plot of *Erora quaderna* were not possible. Sequences > 599 bp = 1.

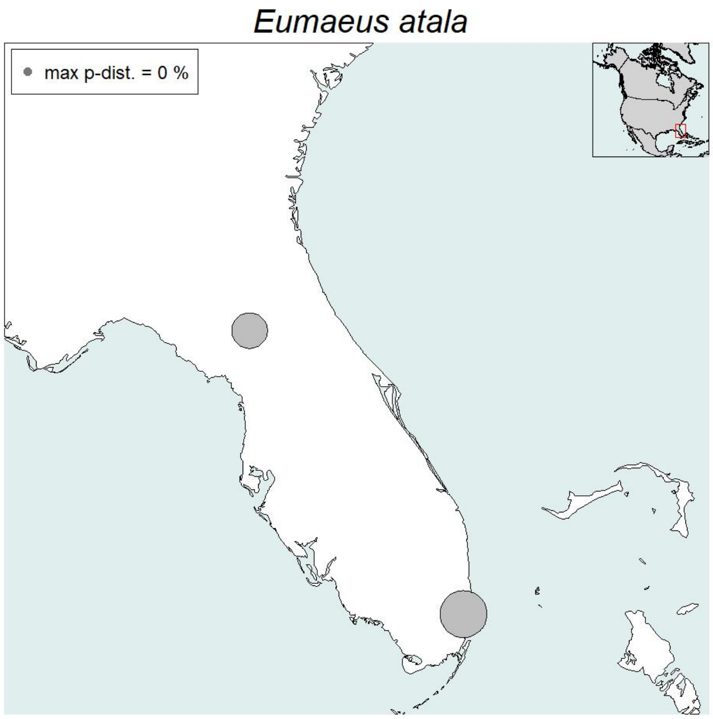

Figure 483: Map of *Eumaeus atala* showing the localities of the sequenced specimens. Nearby localities are grouped in pies. Due to the presence of a single haplotype PCoA projection was not done and a single grey colour was plotted on the map. Sequences= 4; Hap obs.= 1; Hap asympt.= NA; Hap % obs.= NA%; GST= NaN; DST= NaN; HD= NA; ND= NA; max p-dist= 0%.

Haplotype network analysis and bubble plot of *Eumaeus atala* were not possible. Sequences > 599 bp = 4.

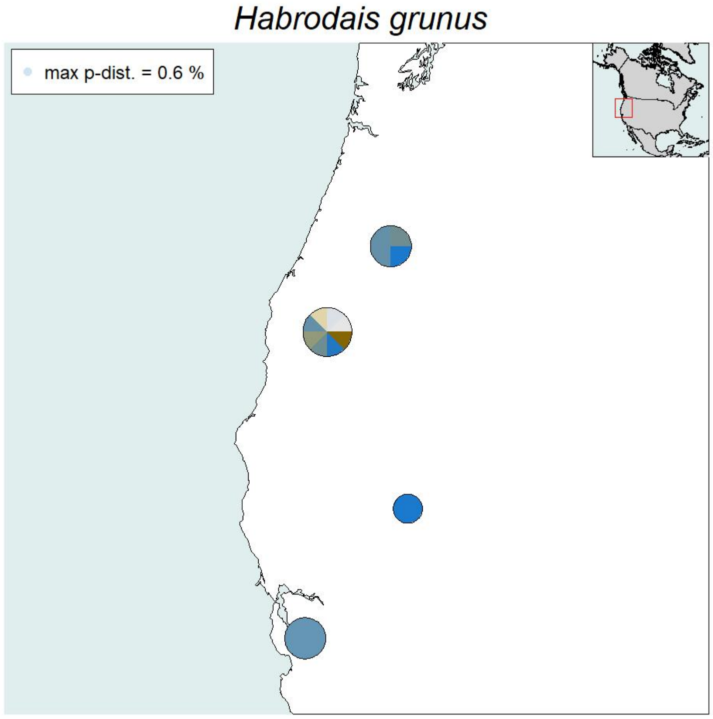

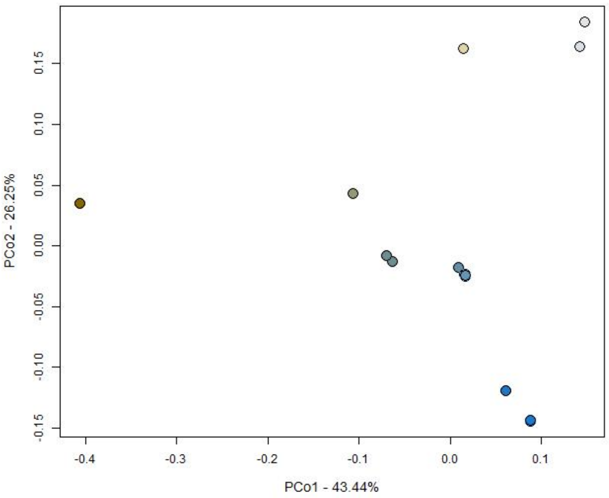

Figure 484 Map of *Habrodais grunus* showing the localities of the sequenced specimens (left). Nearby localities are grouped in pies. Colours match the bidimensional colour space of the PCoA projection (right) of max p-dists among sequences (dots). Sequences= 17; Hap obs.= 7; Hap asympt.= 16.4; Hap % obs.= 42.7%; GST= 0.055; DST= 0.0001; HD= 0.713; ND= 0.0017; max p-dist= 0.6%.

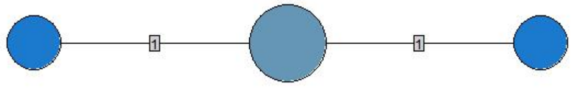

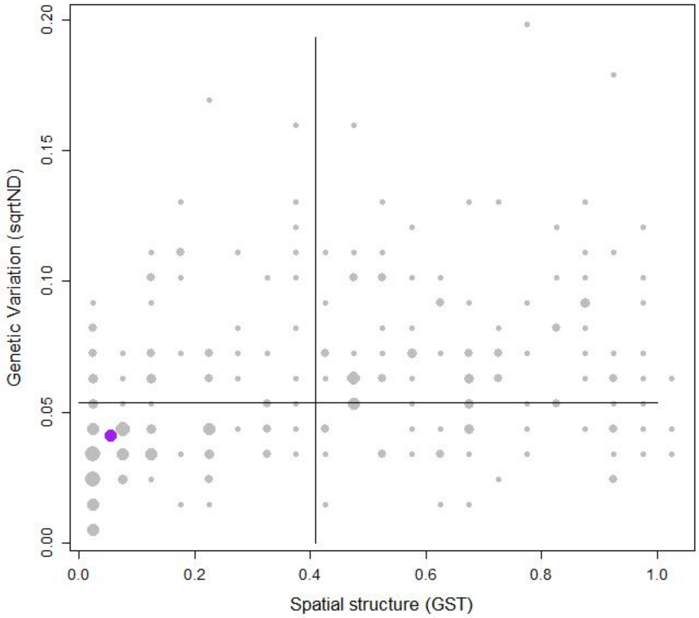

Figure 485: Haplotype network (left) of *Habrodais grunus* sequences > 599 bp with colours matching the PCoA colour space (above). The bubble plot for mt-DNA polymorphism (square root transformed nucleotide diversity) and spatial structure (GST) among all species in the atlas and values for *Habrodais grunus* (purple dot). The horizontal and vertical lines represent median values of nucleotide diversity and GST, respectively. Sequences > 599 bp= 6.

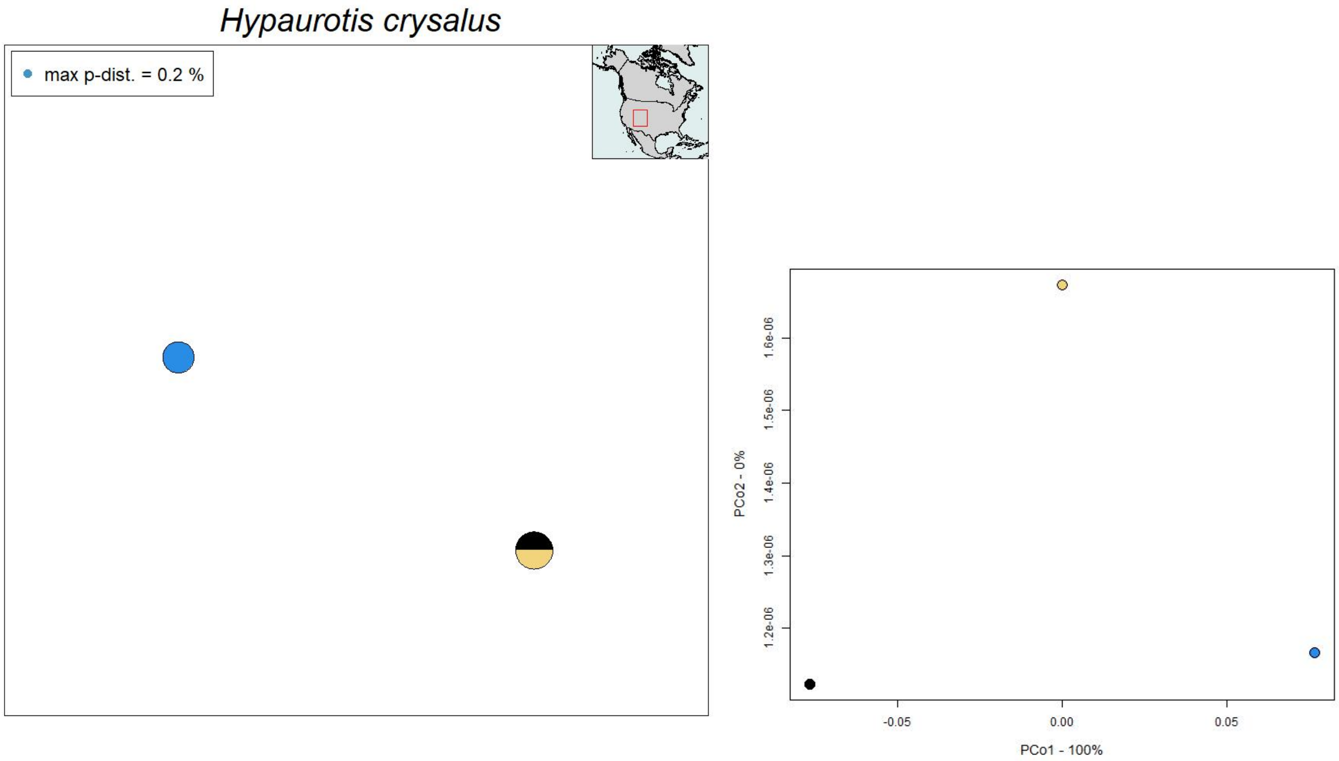

Figure 486 Map of *Hypaurotis crysalus* showing the localities of the sequenced specimens (left). Nearby localities are grouped in pies. Colours match the bidimensional colour space of the PCoA projection (right) of max p-dists among sequences (dots). Sequences= 3; Hap obs.= 3; Hap asympt.= NA; Hap % obs.= NA%; GST= NaN; DST= NaN; HD= NA; ND= NA; max p-dist= 0.2%.

Haplotype network analysis and bubble plot of *Hypaurotis crysalus* were not possible. Sequences > 599 bp = 3.

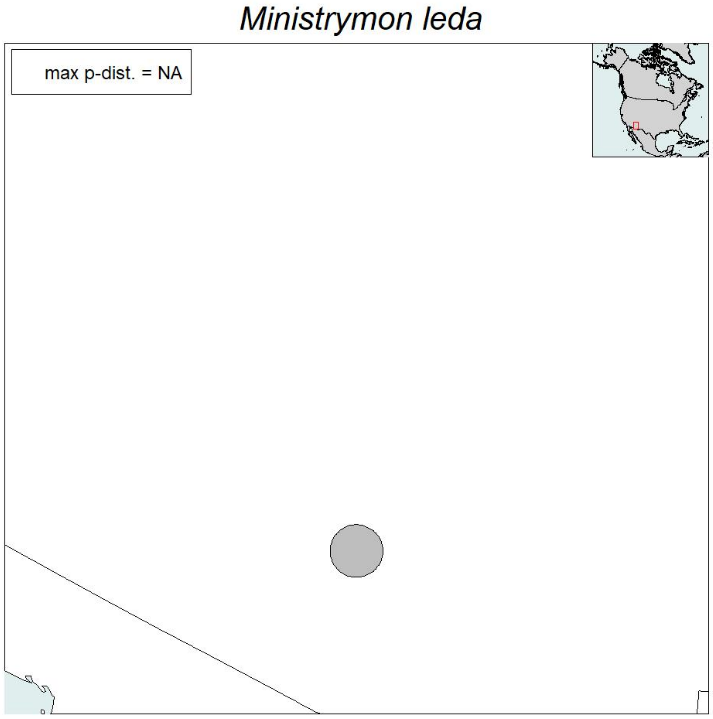

Figure 487: Map of *Ministrymon leda* showing the localities of the sequenced specimens. Nearby localities are grouped in pies. Due to the presence of a single haplotype PCoA projection was not done and a single grey colour was plotted on the map. Sequences= 1; Hap obs.= NA; Hap asympt.= NA; Hap % obs.= NA; GST= NaN; DST= NaN; HD= NA; ND= NA; max p-dist= NA.

Haplotype network analysis and bubble plot of *Ministrymon leda* were not possible. Sequences > 599 bp = 1.

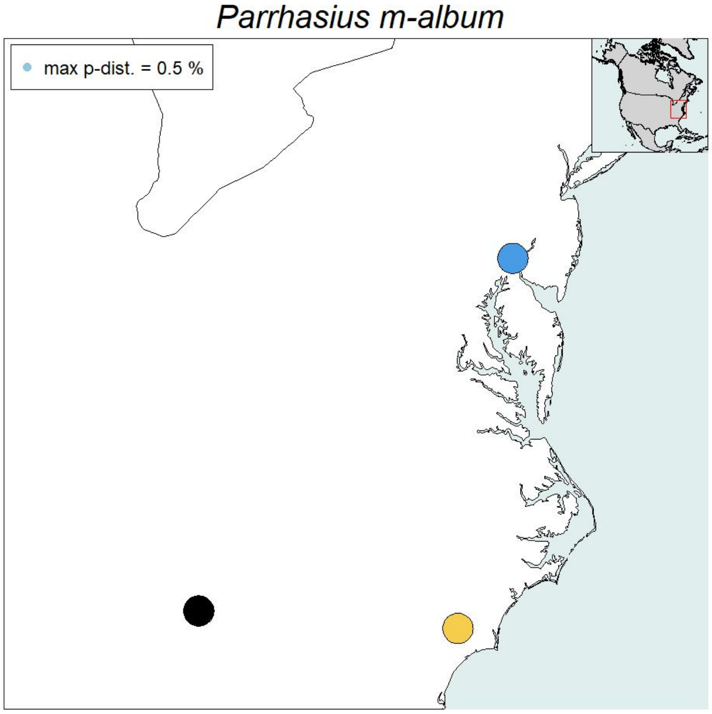

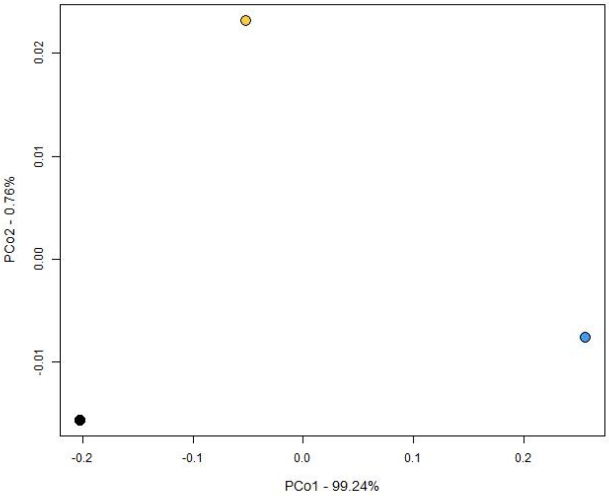

Figure 488 Map of *Parrhasius m-album* showing the localities of the sequenced specimens (left). Nearby localities are grouped in pies. Colours match the bidimensional colour space of the PCoA projection (right) of max p-dists among sequences (dots). Sequences= 3; Hap obs.= 3; Hap asympt.= NA; Hap % obs.= NA%; GST= NaN; DST= NaN; HD= NA; ND= NA; max p-dist= 0.5%.

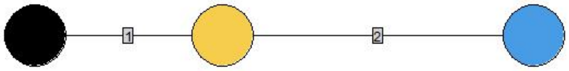

Figure 489: Haplotype network of *Parrhasius m-album*. Sequences > 599 bp= 3.

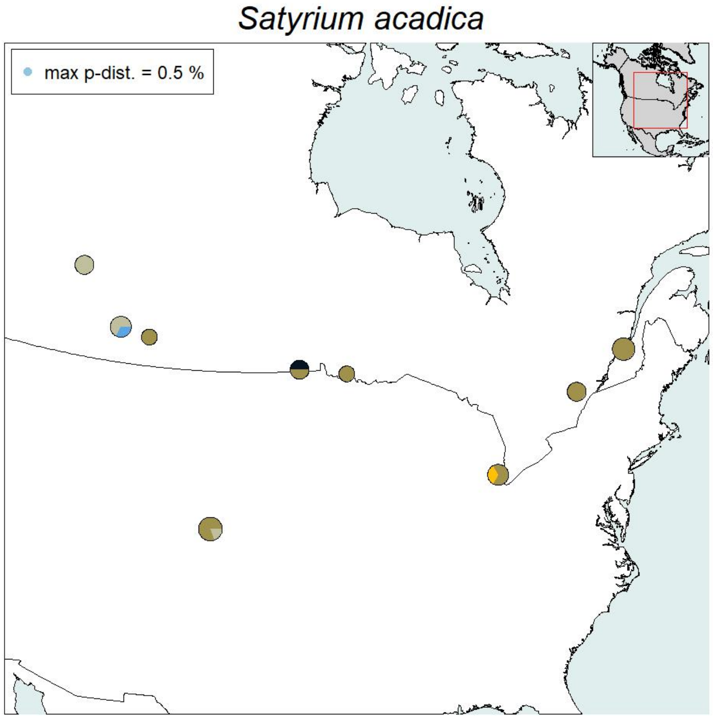

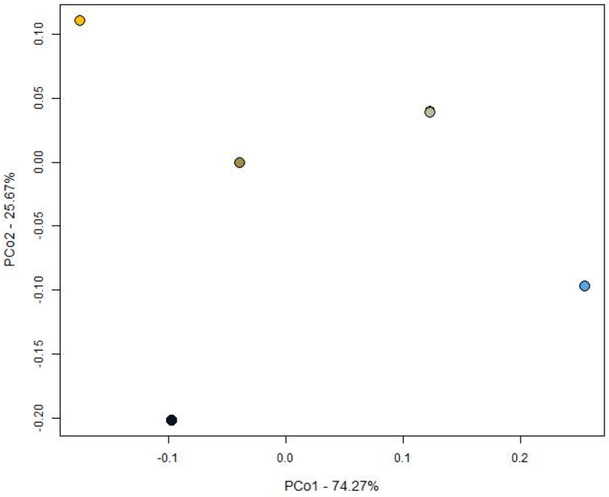

Figure 490 Map of *Satyrium acadica* showing the localities of the sequenced specimens (left). Nearby localities are grouped in pies. Colours match the bidimensional colour space of the PCoA projection (right) of max p-dists among sequences (dots). Sequences= 23; Hap obs.= 5; Hap asympt.= 7.9; Hap % obs.= 63.5%; GST= 0.345; DST= 0.0004; HD= 0.545; ND= 0.001; max p-dist= 0.5%.

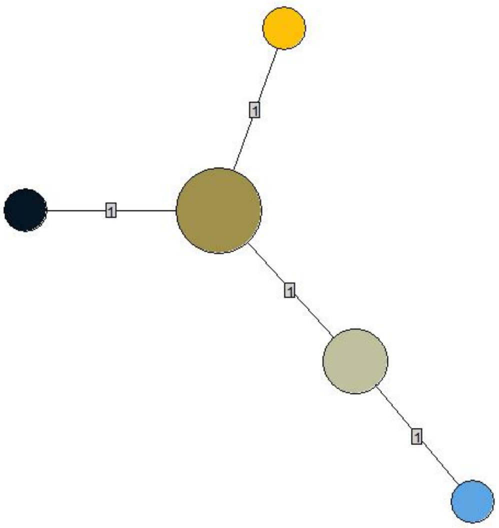

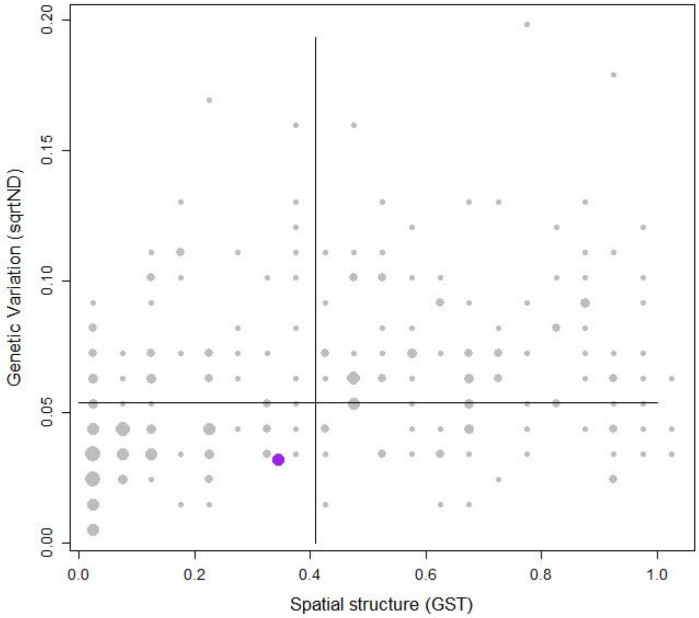

Figure 491: Haplotype network (left) of *Satyrium acadica* sequences > 599 bp with colours matching the PCoA colour space (above). The bubble plot for mt-DNA polymorphism (square root transformed nucleotide diversity) and spatial structure (GST) among all species in the atlas and values for *Satyrium acadica* (purple dot). The horizontal and vertical lines represent median values of nucleotide diversity and GST, respectively. Sequences > 599 bp= 23.

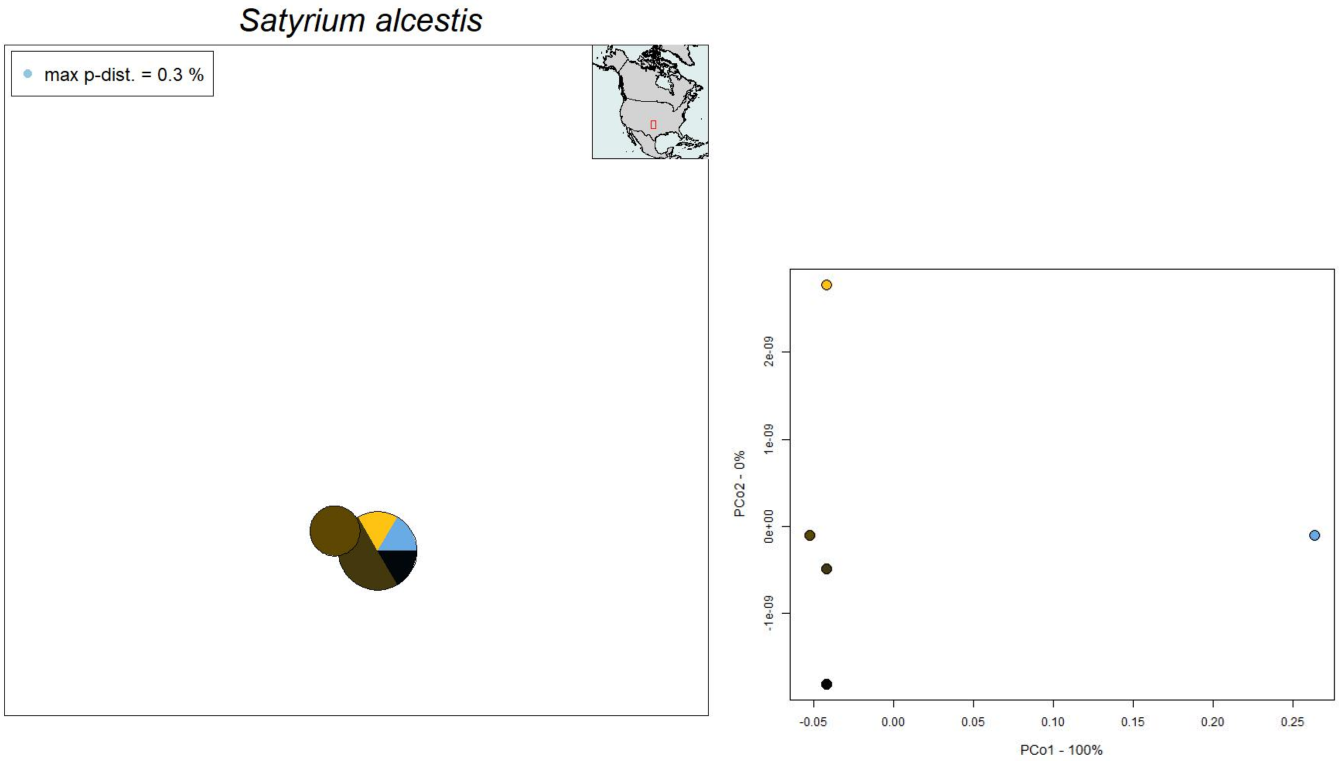

Figure 492 Map of *Satyrium alcestis* showing the localities of the sequenced specimens (left). Nearby localities are grouped in pies. Colours match the bidimensional colour space of the PCoA projection (right) of max p-dists among sequences (dots). Sequences= 7; Hap obs.= 2; Hap asympt.= NA; Hap % obs.= NA%; GST= NaN; DST= NaN; HD= NA; ND= NA; max p-dist= 0.3%.

Haplotype network analysis and bubble plot of *Satyrium alcestis* were not possible. Sequences > 599 bp = 7.

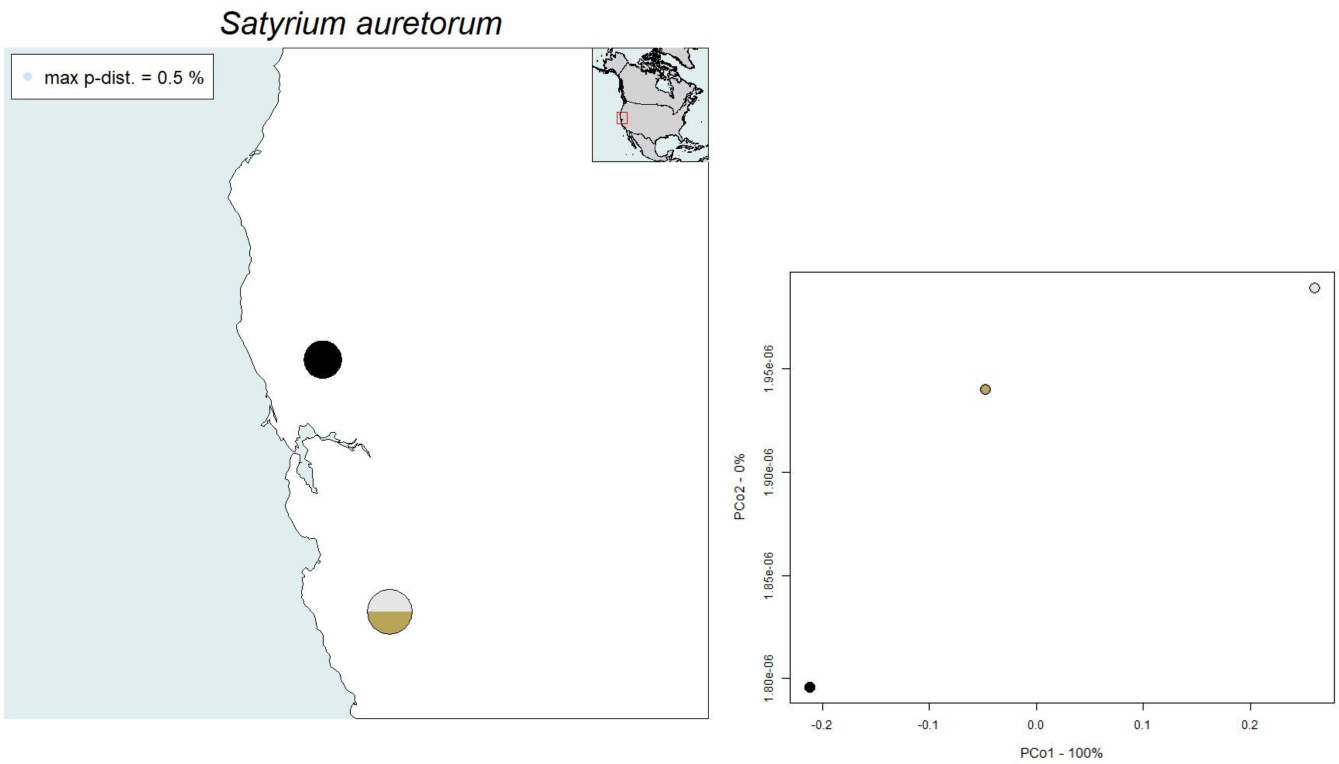

Figure 493 Map of *Satyrium auretorum* showing the localities of the sequenced specimens (left). Nearby localities are grouped in pies. Colours match the bidimensional colour space of the PCoA projection (right) of max p-dists among sequences (dots). Sequences= 3; Hap obs.= 3; Hap asympt.= NA; Hap % obs.= NA%; GST= NaN; DST= NaN; HD= NA; ND= NA; max p-dist= 0.5%.

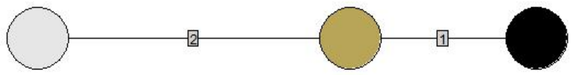

Figure 494: Haplotype network of *Satyrium auretorum*. Sequences > 599 bp= 3.

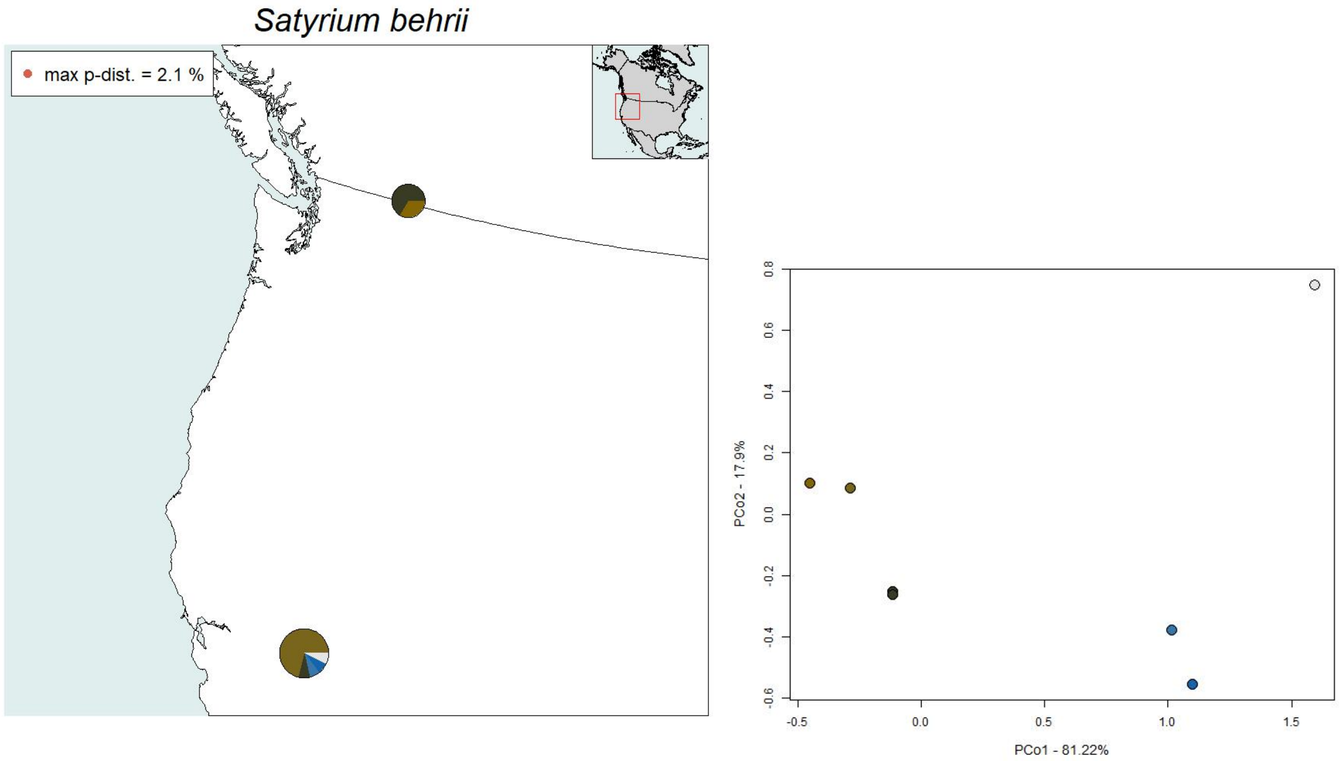

Figure 495 Map of *Satyrium behrii* showing the localities of the sequenced specimens (left). Nearby localities are grouped in pies. Colours match the bidimensional colour space of the PCoA projection (right) of max p-dists among sequences (dots). Sequences= 17; Hap obs.= 7; Hap asympt.= 18.8; Hap % obs.= 37.3%; GST= 0; DST= 0; HD= 0.662; ND= 0.0061; max p-dist= 2.1%.

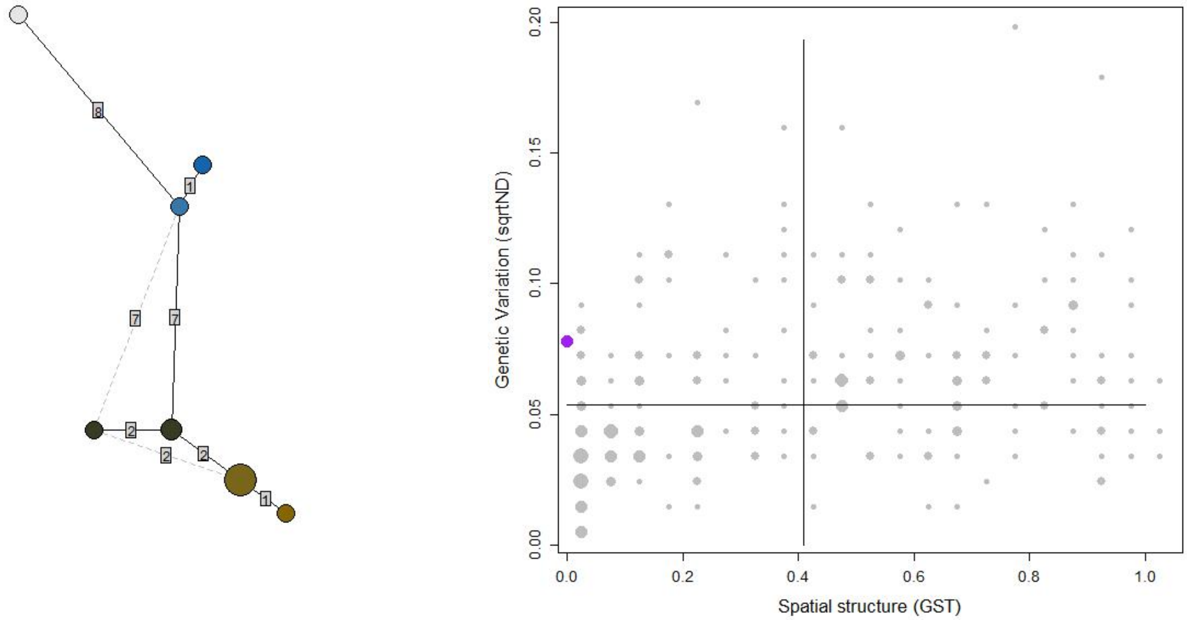

Figure 496: Haplotype network (left) of *Satyrium behrii* sequences > 599 bp with colours matching the PCoA colour space (above). The bubble plot for mt-DNA polymorphism (square root transformed nucleotide diversity) and spatial structure (GST) among all species in the atlas and values for *Satyrium behrii* (purple dot). The horizontal and vertical lines represent median values of nucleotide diversity and GST, respectively. Sequences > 599 bp= 17.

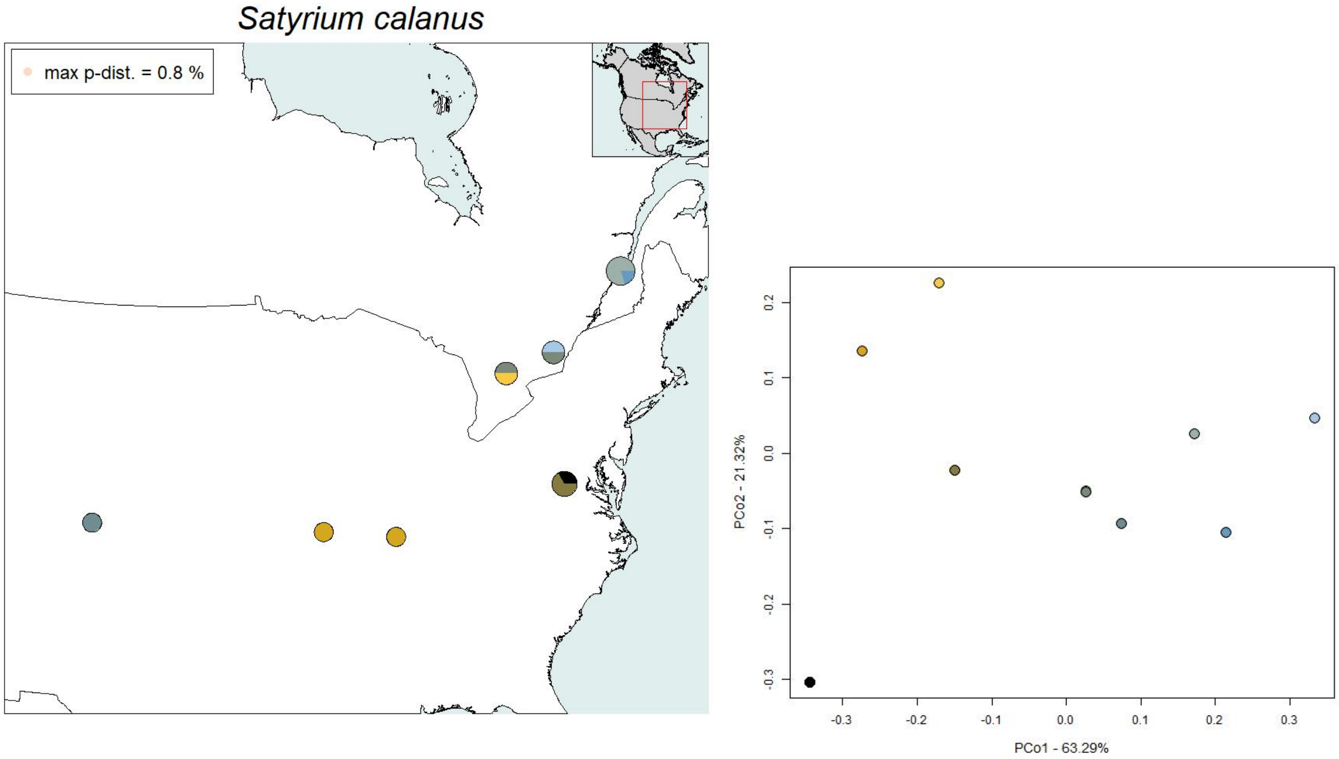

Figure 497 Map of *Satyrium calanus* showing the localities of the sequenced specimens (left). Nearby localities are grouped in pies. Colours match the bidimensional colour space of the PCoA projection (right) of max p-dists among sequences (dots). Sequences= 15; Hap obs.= 7; Hap asympt.= 9.1; Hap % obs.= 76.9%; GST= 0.494; DST= 0.0013; HD= 0.857; ND= 0.0029; max p-dist= 0.8%.

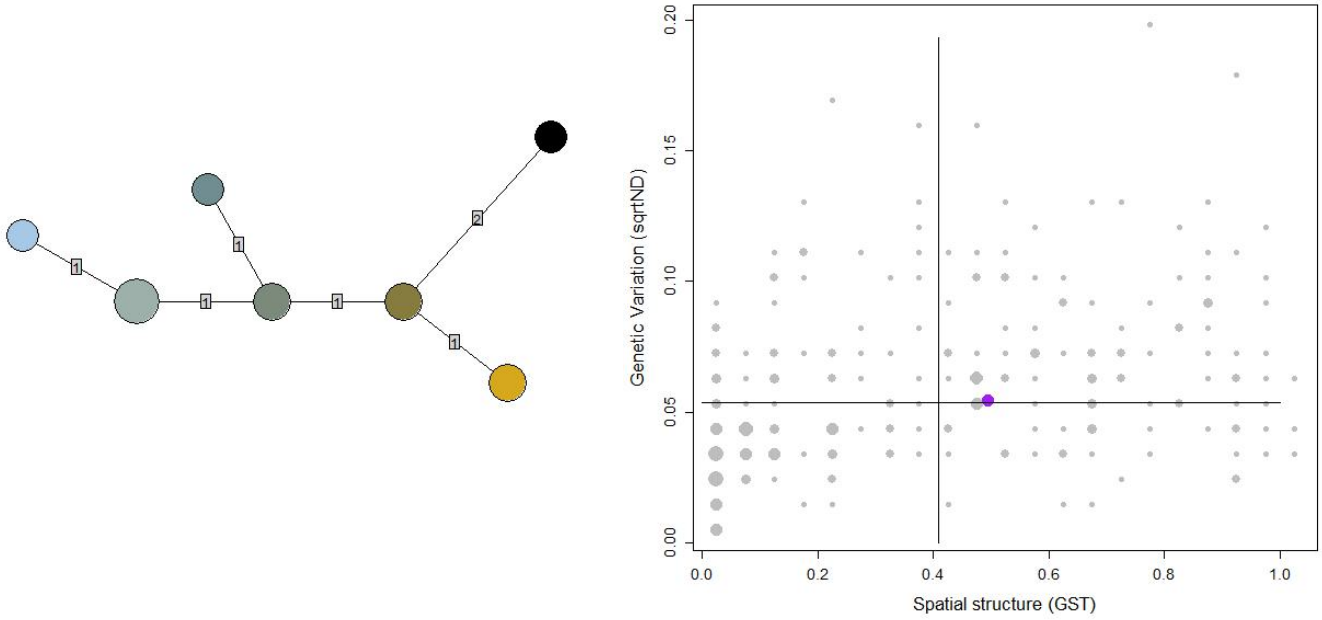

Figure 498: Haplotype network (left) of *Satyrium calanus* sequences > 599 bp with colours matching the PCoA colour space (above). The bubble plot for mt-DNA polymorphism (square root transformed nucleotide diversity) and spatial structure (GST) among all species in the atlas and values for *Satyrium calanus* (purple dot). The horizontal and vertical lines represent median values of nucleotide diversity and GST, respectively. Sequences > 599 bp= 13.

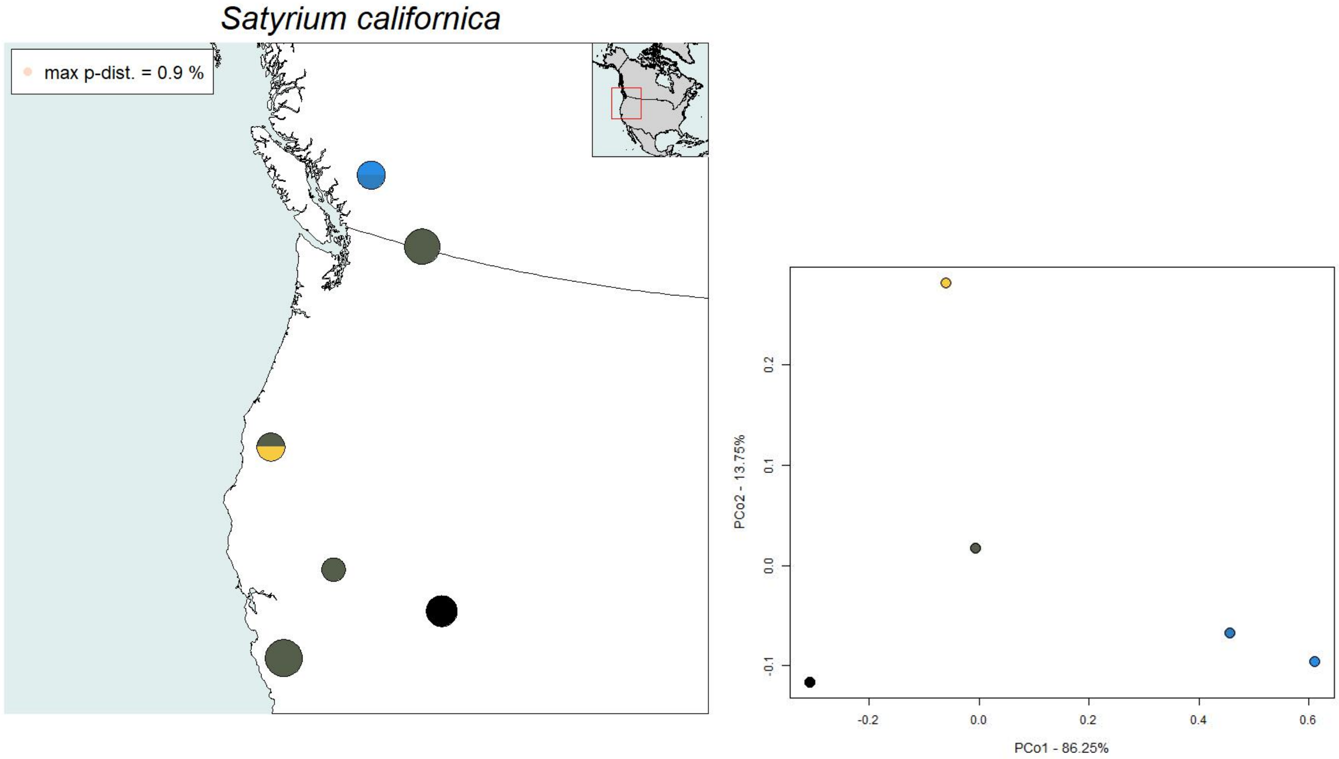

Figure 499 Map of *Satyrium californica* showing the localities of the sequenced specimens (left). Nearby localities are grouped in pies. Colours match the bidimensional colour space of the PCoA projection (right) of max p-dists among sequences (dots). Sequences= 19; Hap obs.= 5; Hap asympt.= 7.8; Hap % obs.= 63.8%; GST= 1; DST= 0.0011; HD= 0.526; ND= 0.0021; max p-dist= 0.9%.

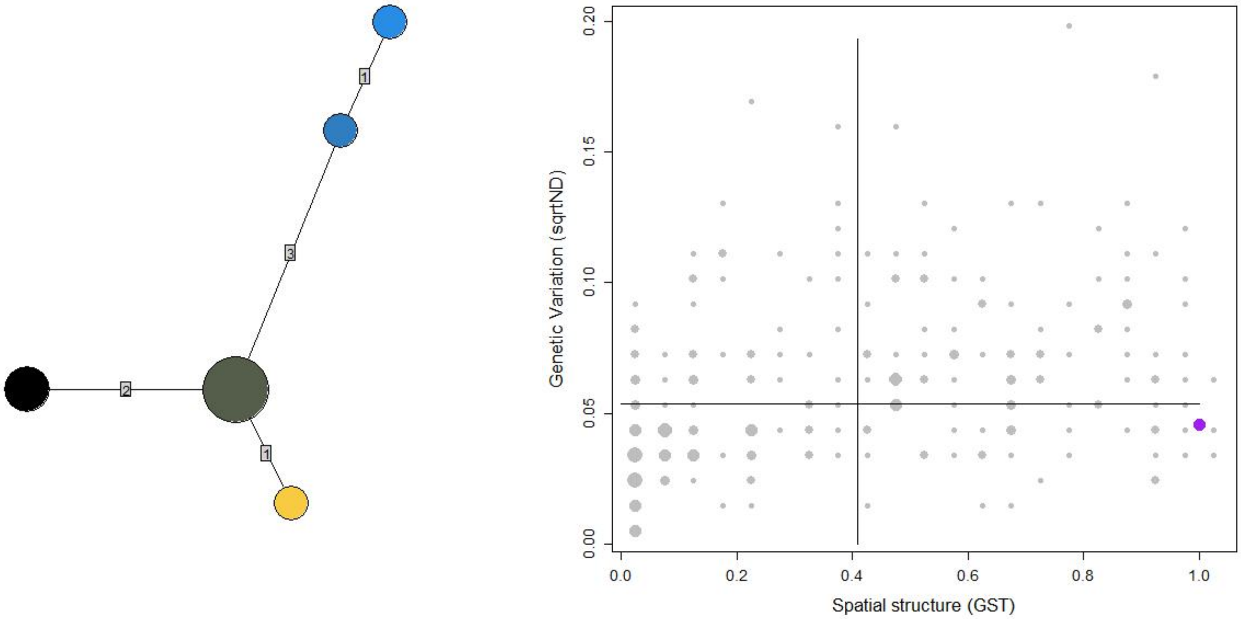

Figure 500: Haplotype network (left) of *Satyrium californica* sequences > 599 bp with colours matching the PCoA colour space (above). The bubble plot for mt-DNA polymorphism (square root transformed nucleotide diversity) and spatial structure (GST) among all species in the atlas and values for *Satyrium californica* (purple dot). The horizontal and vertical lines represent median values of nucleotide diversity and GST, respectively. Sequences > 599 bp= 19.

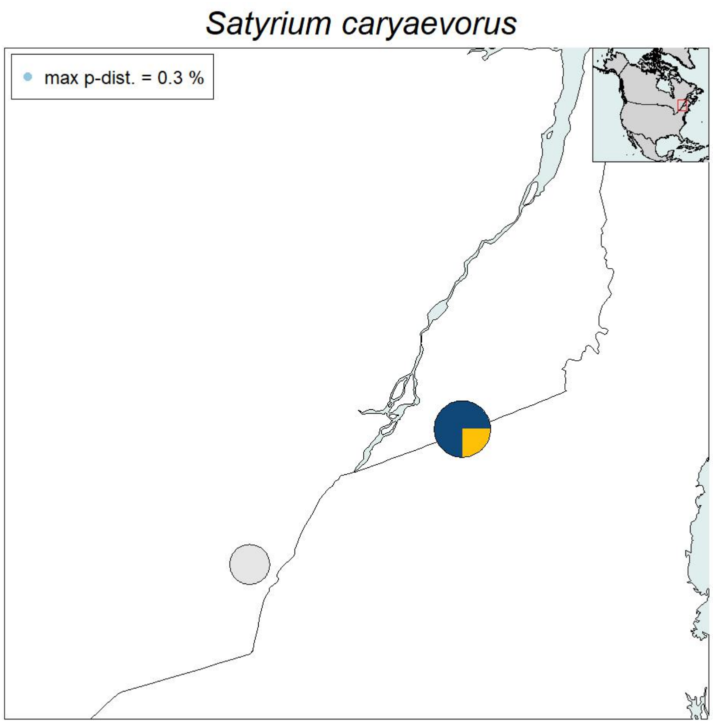

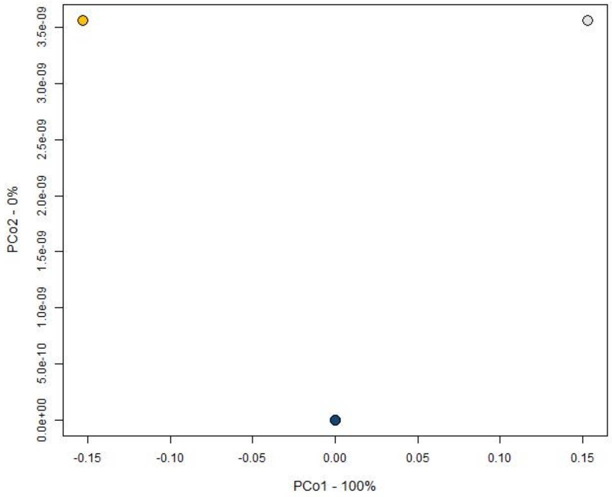

Figure 501 Map of *Satyrium caryaevorus* showing the localities of the sequenced specimens (left). Nearby localities are grouped in pies. Colours match the bidimensional colour space of the PCoA projection (right) of max p-dists among sequences (dots). Sequences= 5; Hap obs.= 3; Hap asympt.= NA; Hap % obs.= NA%; GST= NaN; DST= NaN; HD= NA; ND= NA; max p-dist= 0.3%.

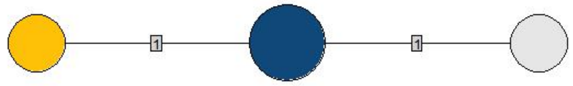

Figure 502: Haplotype network of *Satyrium caryaevorus*. Sequences > 599 bp= 5.

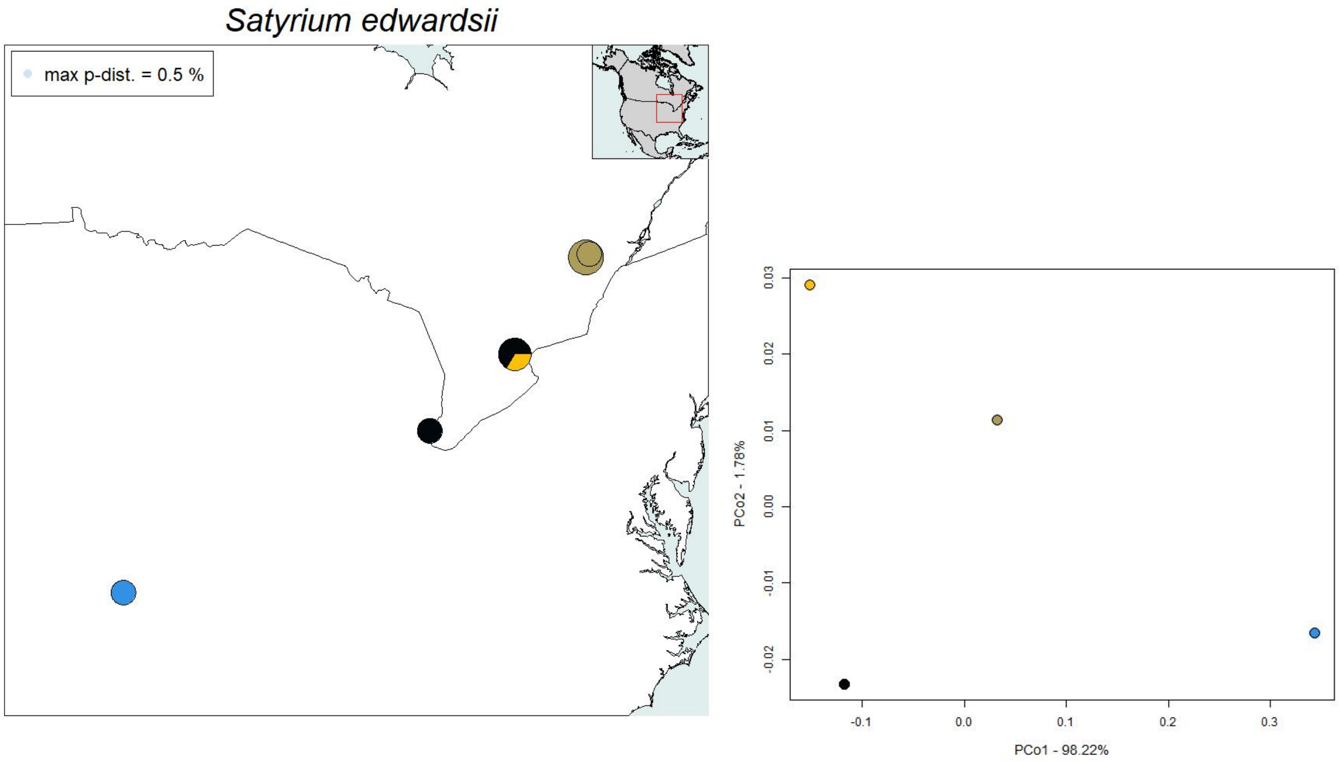

Figure 503 Map of *Satyrium edwardsii* showing the localities of the sequenced specimens (left). Nearby localities are grouped in pies. Colours match the bidimensional colour space of the PCoA projection (right) of max p-dists among sequences (dots). Sequences= 10; Hap obs.= 3; Hap asympt.= 3; Hap % obs.= 100%; GST= NaN; DST= NaN; HD= 0.644; ND= 0.0014; max p-dist= 0.5%.

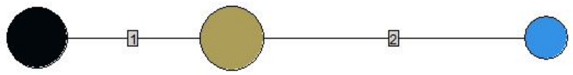

Figure 504: Haplotype network of *Satyrium edwardsii*. Sequences > 599 bp= 10.

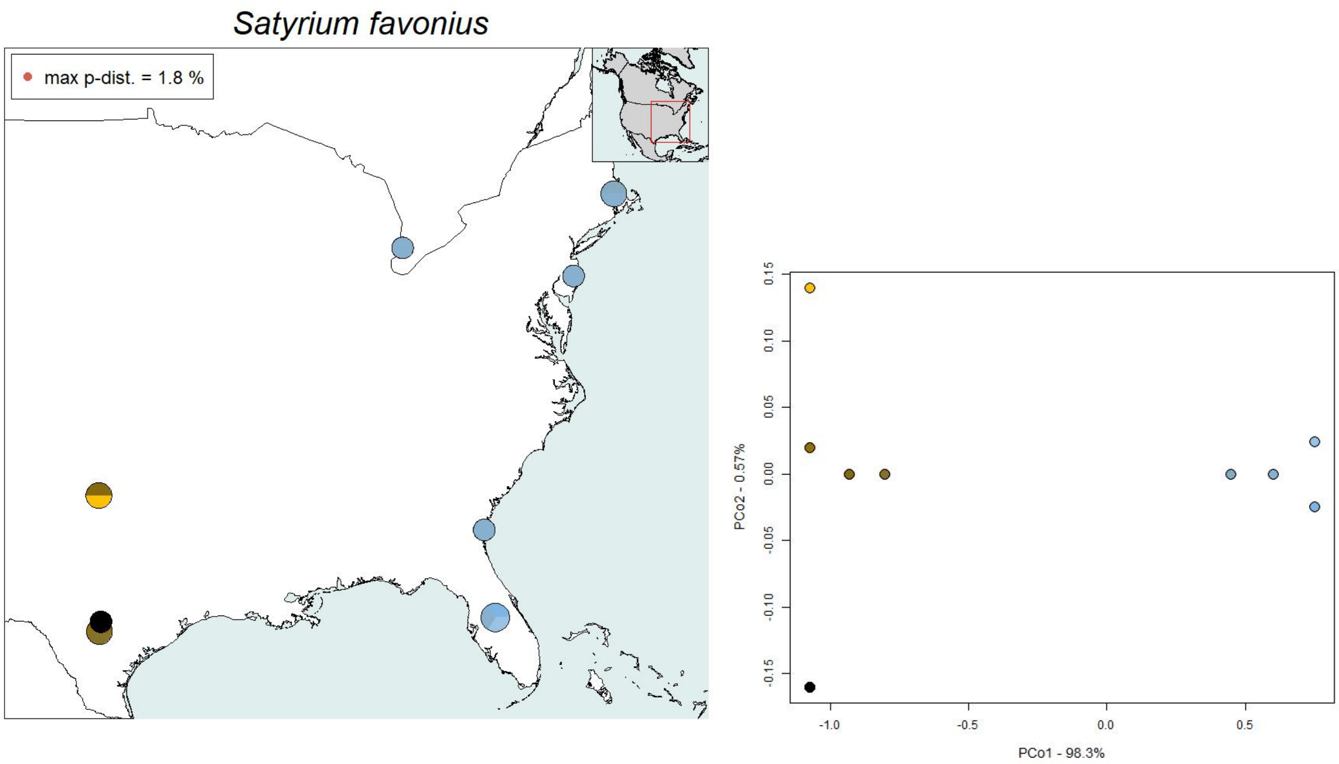

Figure 505 Map of *Satyrium favonius* showing the localities of the sequenced specimens (left). Nearby localities are grouped in pies. Colours match the bidimensional colour space of the PCoA projection (right) of max p-dists among sequences (dots). Sequences= 13; Hap obs.= 8; Hap asympt.= 24.6; Hap % obs.= 32.5%; GST= NaN; DST= NaN; HD= 0.859; ND= 0.0089; max p-dist= 1.8%.

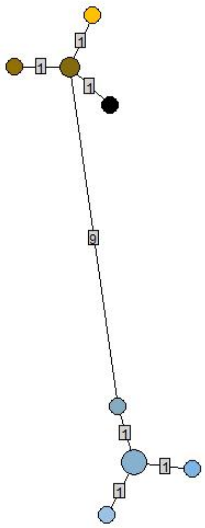

Figure 506: Haplotype network of *Satyrium favonius*. Sequences > 599 bp= 13.

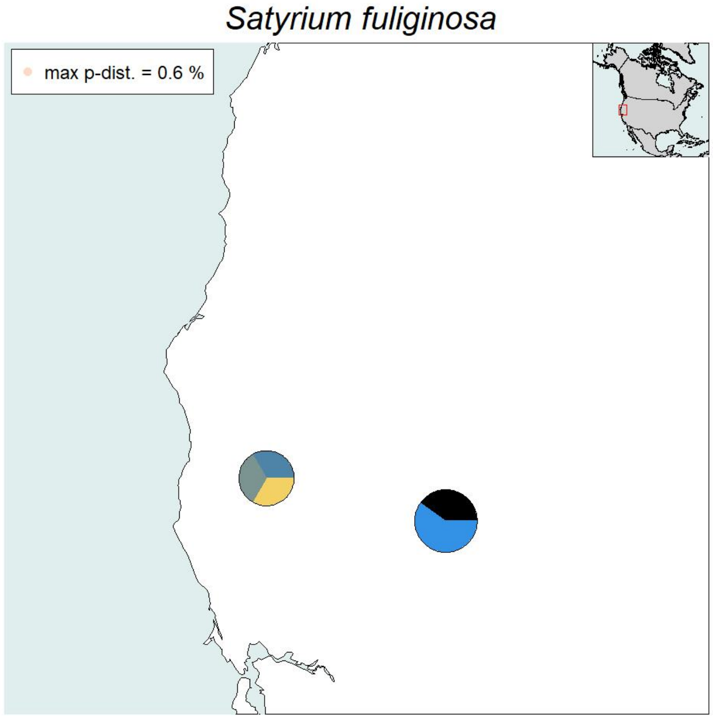

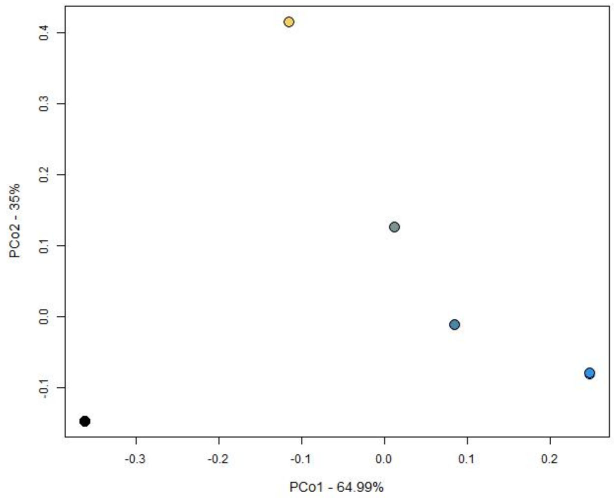

Figure 507 Map of *Satyrium fuliginosa* showing the localities of the sequenced specimens (left). Nearby localities are grouped in pies. Colours match the bidimensional colour space of the PCoA projection (right) of max p-dists among sequences (dots). Sequences= 8; Hap obs.= 5; Hap asympt.= NA; Hap % obs.= NA%; GST= NaN; DST= NaN; HD= NA; ND= NA; max p-dist= 0.6%.

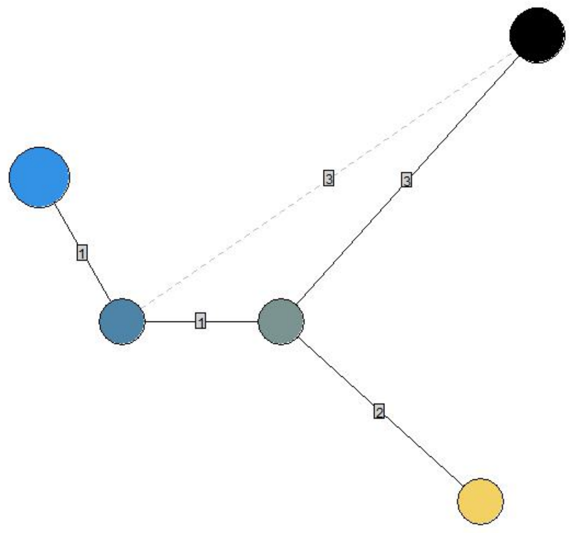

Figure 508: Haplotype network of *Satyrium fuliginosa*. Sequences > 599 bp= 8.

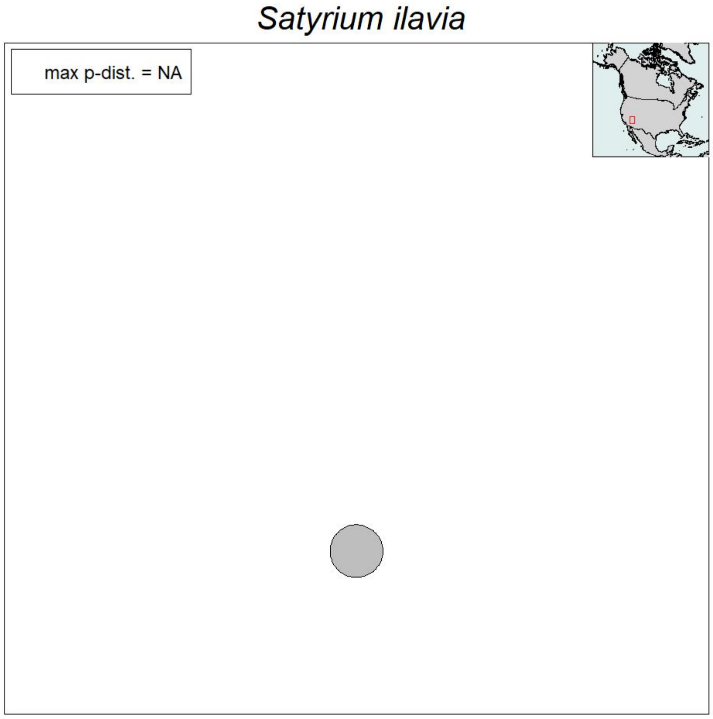

Figure 509: Map of *Satyrium ilavia* showing the localities of the sequenced specimens. Nearby localities are grouped in pies. Due to the presence of a single haplotype PCoA projection was not done and a single grey colour was plotted on the map. Sequences= 1; Hap obs.= NA; Hap asympt.= NA; Hap % obs.= NA; GST= NaN; DST= NaN; HD= NA; ND= NA; max p-dist= NA.

Haplotype network analysis and bubble plot of *Satyrium ilavia* were not possible. Sequences > 599 bp = 0.

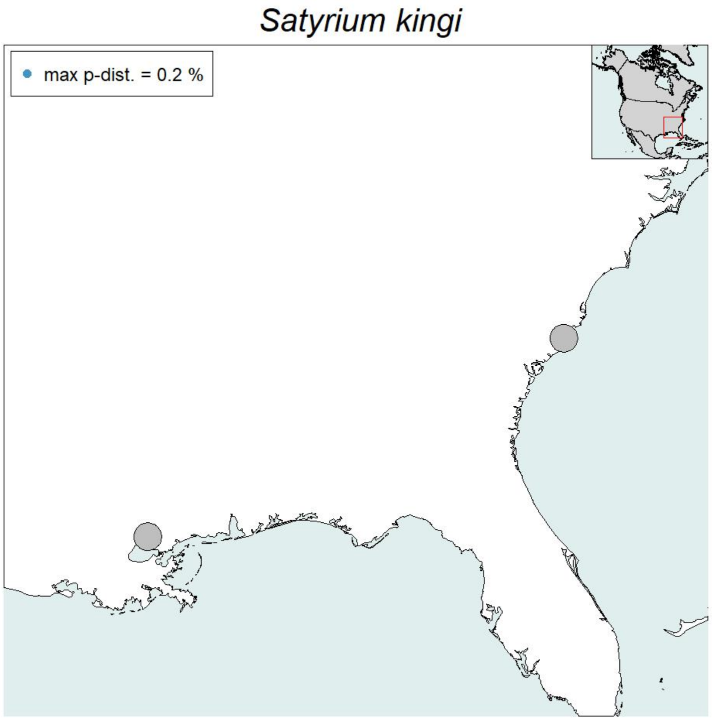

Figure 510: Map of *Satyrium kingi* showing the localities of the sequenced specimens. Nearby localities are grouped in pies. Due to the presence of a single haplotype PCoA projection was not done and a single grey colour was plotted on the map. Sequences= 2; Hap obs.= 2; Hap asympt.= NA; Hap % obs.= NA%; GST= NaN; DST= NaN; HD= NA; ND= NA; max p-dist= 0.2%.

Haplotype network analysis and bubble plot of *Satyrium kingi* were not possible. Sequences > 599 bp = 2.

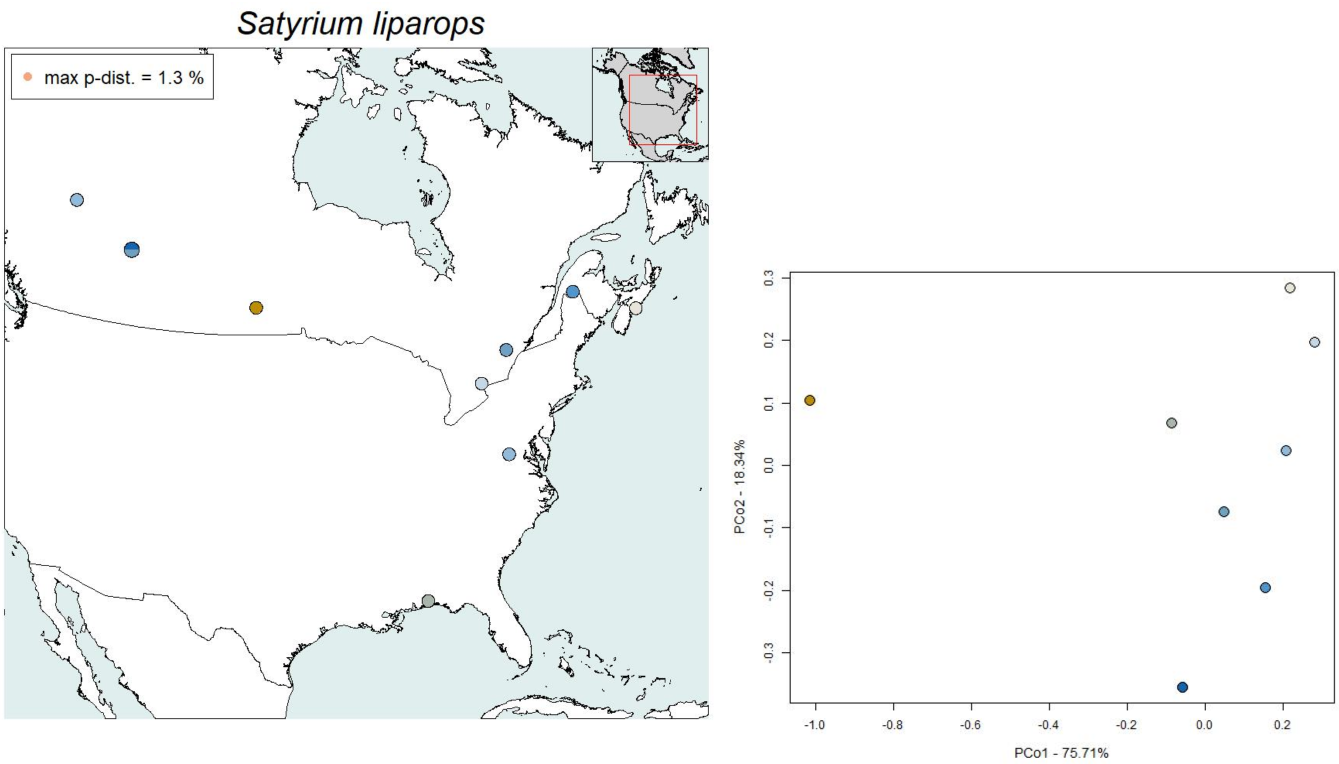

Figure 511 Map of *Satyrium liparops* showing the localities of the sequenced specimens (left). Nearby localities are grouped in pies. Colours match the bidimensional colour space of the PCoA projection (right) of max p-dists among sequences (dots). Sequences= 10; Hap obs.= 7; Hap asympt.= 9.4; Hap % obs.= 74.5%; GST= NaN; DST= NaN; HD= 0.933; ND= 0.0046; max p-dist= 1.3%.

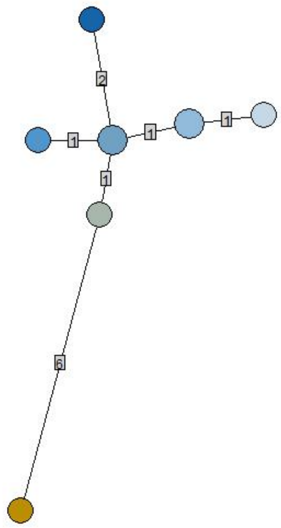

Figure 512: Haplotype network of *Satyrium liparops*. Sequences > 599 bp= 9.

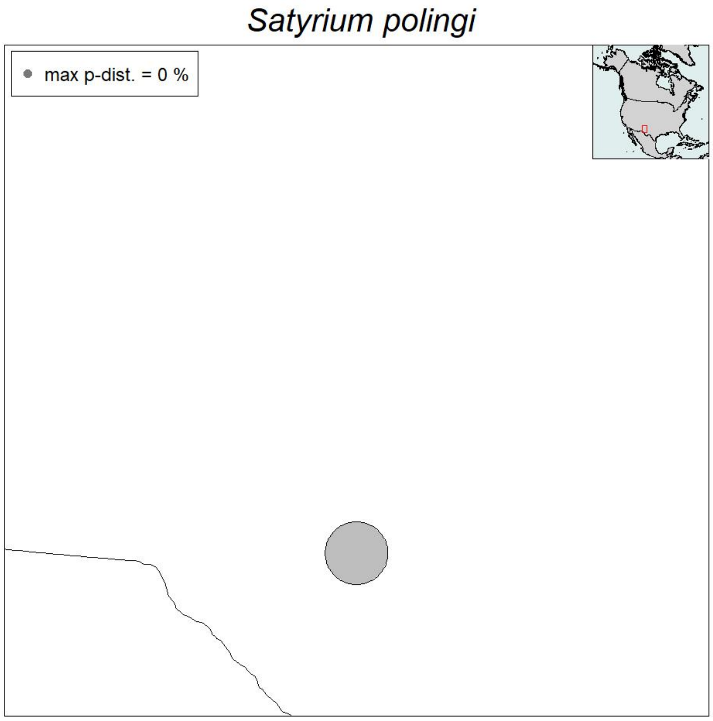

Figure 513: Map of *Satyrium polingi* showing the localities of the sequenced specimens. Nearby localities are grouped in pies. Due to the presence of a single haplotype PCoA projection was not done and a single grey colour was plotted on the map. Sequences= 2; Hap obs.= 1; Hap asympt.= NA; Hap % obs.= NA%; GST= NaN; DST= NaN; HD= NA; ND= NA; max p-dist= 0%.

Haplotype network analysis and bubble plot of *Satyrium polingi* were not possible. Sequences > 599 bp = 2.

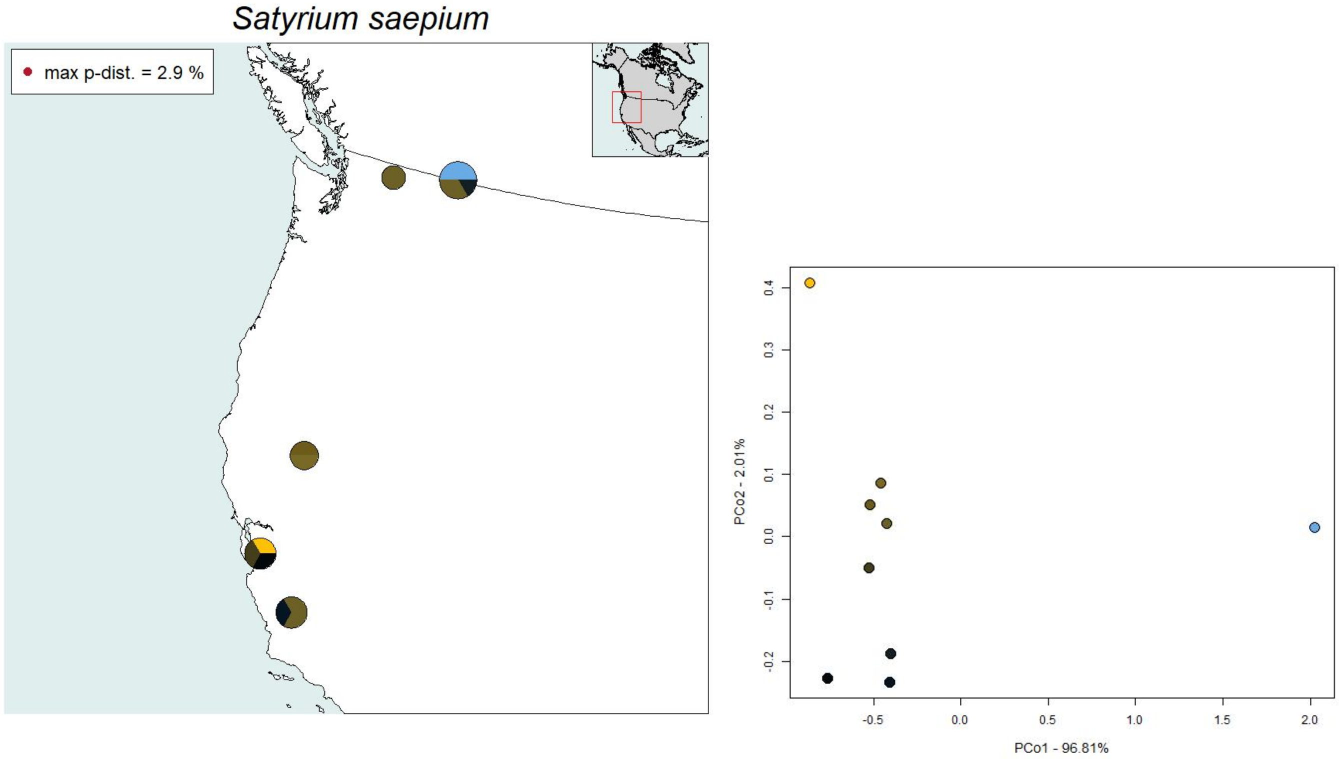

Figure 514 Map of *Satyrium saepium* showing the localities of the sequenced specimens (left). Nearby localities are grouped in pies. Colours match the bidimensional colour space of the PCoA projection (right) of max p-dists among sequences (dots). Sequences= 15; Hap obs.= 8; Hap asympt.= 22; Hap % obs.= 36.4%; GST= 0.113; DST= 0.0014; HD= 0.829; ND= 0.01; max p-dist= 2.9%.

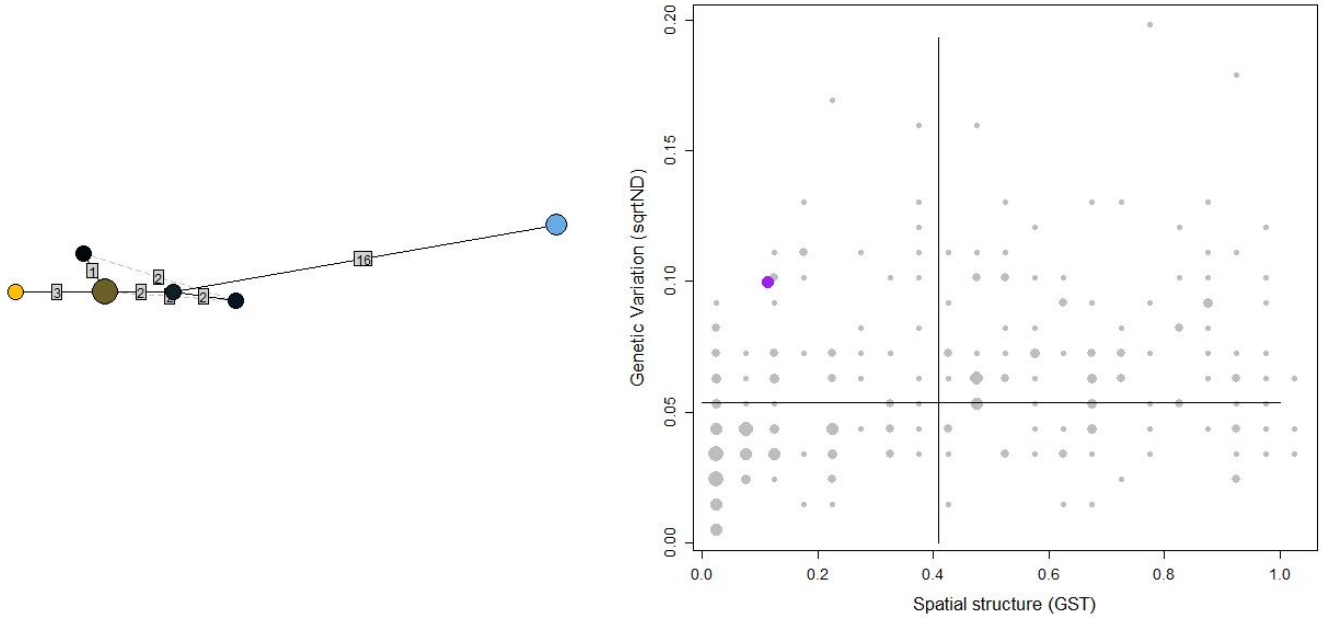

Figure 515: Haplotype network (left) of *Satyrium saepium* sequences > 599 bp with colours matching the PCoA colour space (above). The bubble plot for mt-DNA polymorphism (square root transformed nucleotide diversity) and spatial structure (GST) among all species in the atlas and values for *Satyrium saepium* (purple dot). The horizontal and vertical lines represent median values of nucleotide diversity and GST, respectively. Sequences > 599 bp= 13.

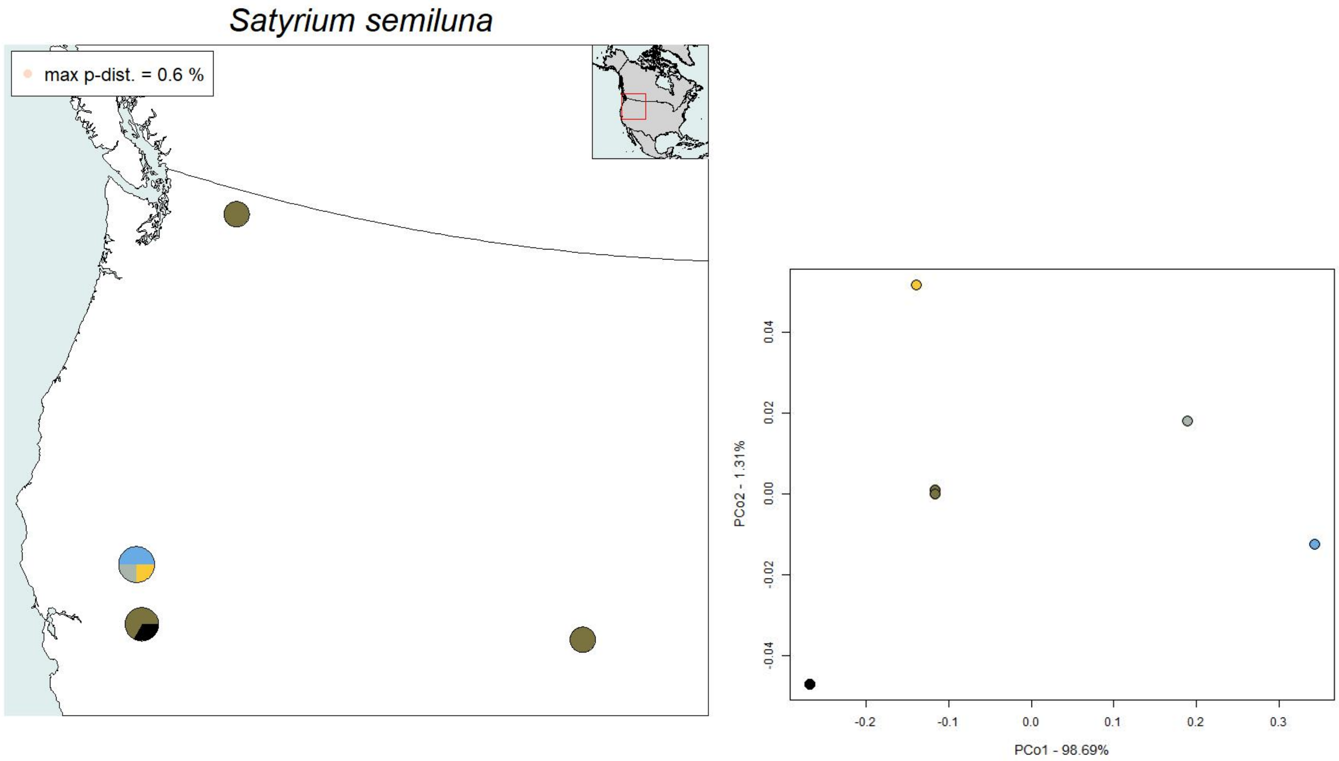

Figure 516 Map of *Satyrium semiluna* showing the localities of the sequenced specimens (left). Nearby localities are grouped in pies. Colours match the bidimensional colour space of the PCoA projection (right) of max p-dists among sequences (dots). Sequences= 9; Hap obs.= 4; Hap asympt.= NA; Hap % obs.= NA%; GST= NaN; DST= NaN; HD= NA; ND= NA; max p-dist= 0.6%.

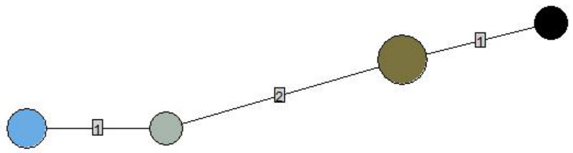

Figure 517: Haplotype network of *Satyrium semiluna*. Sequences > 599 bp= 9.

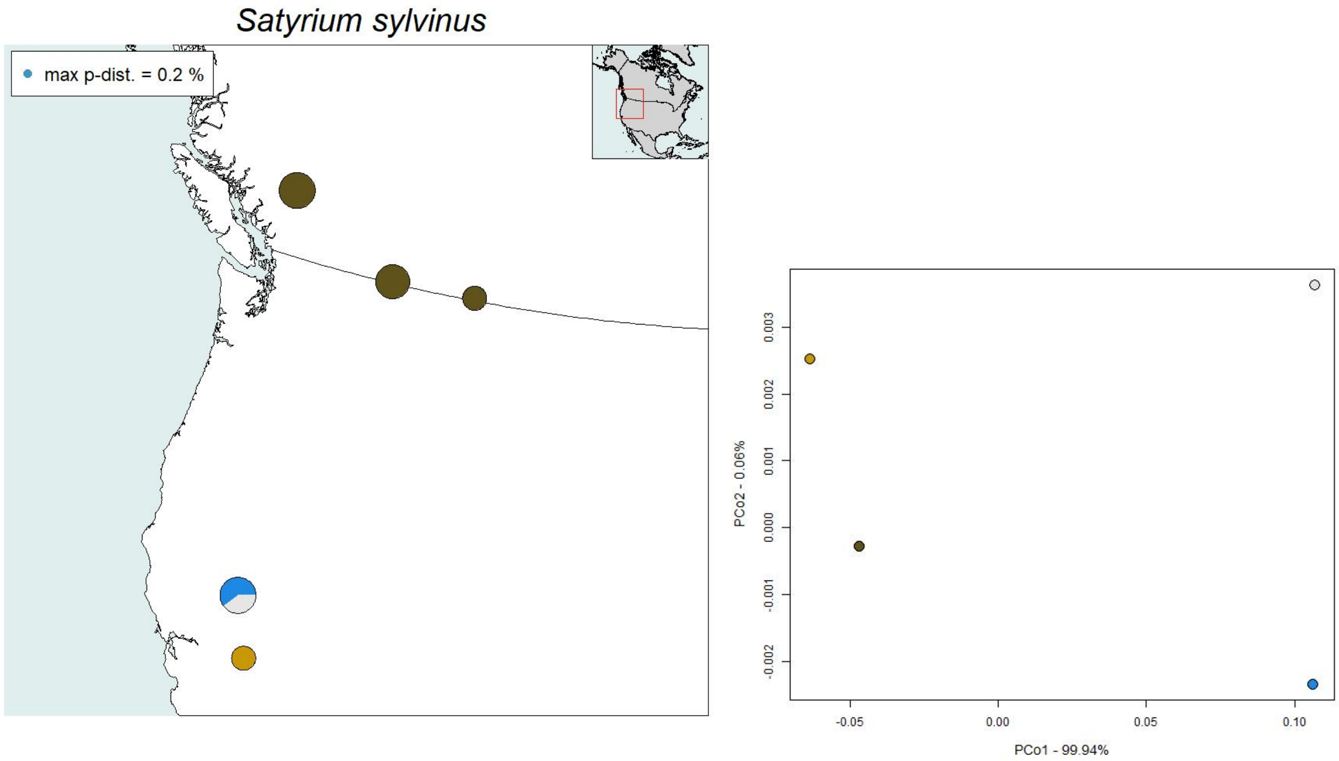

Figure 518 Map of *Satyrium sylvinus* showing the localities of the sequenced specimens (left). Nearby localities are grouped in pies. Colours match the bidimensional colour space of the PCoA projection (right) of max p-dists among sequences (dots). Sequences= 16; Hap obs.= 2; Hap asympt.= 2; Hap % obs.= 100%; GST= 0.947; DST= 0.0007; HD= 0.458; ND= 0.0007; max p-dist= 0.2%.

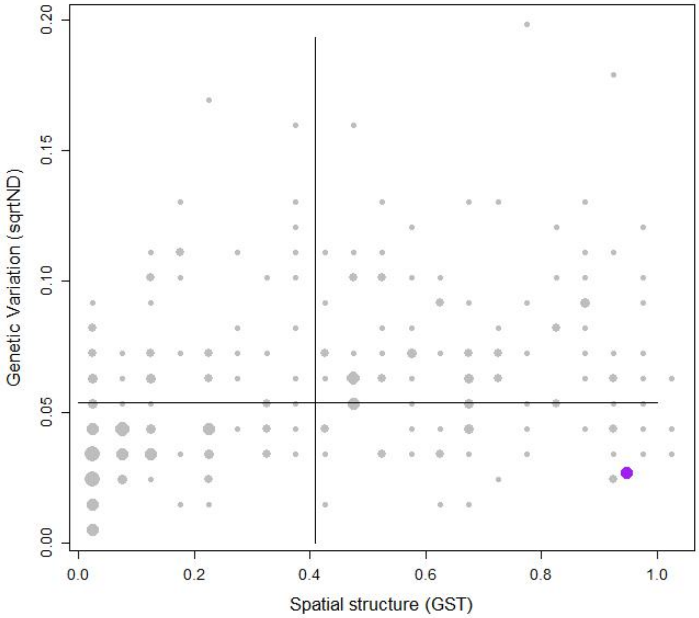

Figure 519: The bubble plot for mt-DNA polymorphism (square root transformed nucleotide diversity) and spatial structuree (GST) among all species in the atlas and values for *Satyrium sylvinus* (purple dot). The horizontal and vertical lines represent median values of nucleotide diversity and GST, respectively. Haplotype network analysis was not possible. Sequences > 599 bp = 15.

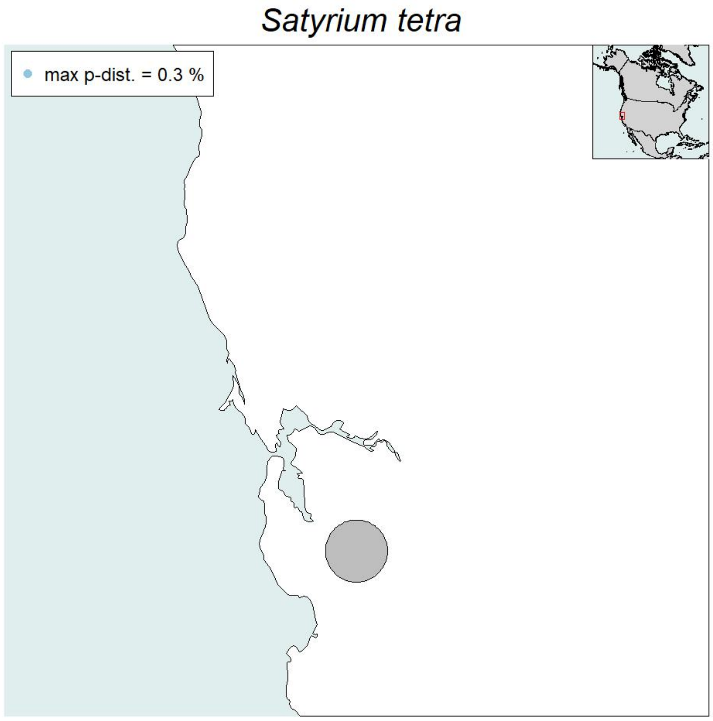

Figure 520: Map of *Satyrium tetra* showing the localities of the sequenced specimens. Nearby localities are grouped in pies. Due to the presence of a single haplotype PCoA projection was not done and a single grey colour was plotted on the map. Sequences= 2; Hap obs.= 2; Hap asympt.= NA; Hap % obs.= NA%; GST= NaN; DST= NaN; HD= NA; ND= NA; max p-dist= 0.3%.

Haplotype network analysis and bubble plot of *Satyrium tetra* were not possible. Sequences > 599 bp = 2.

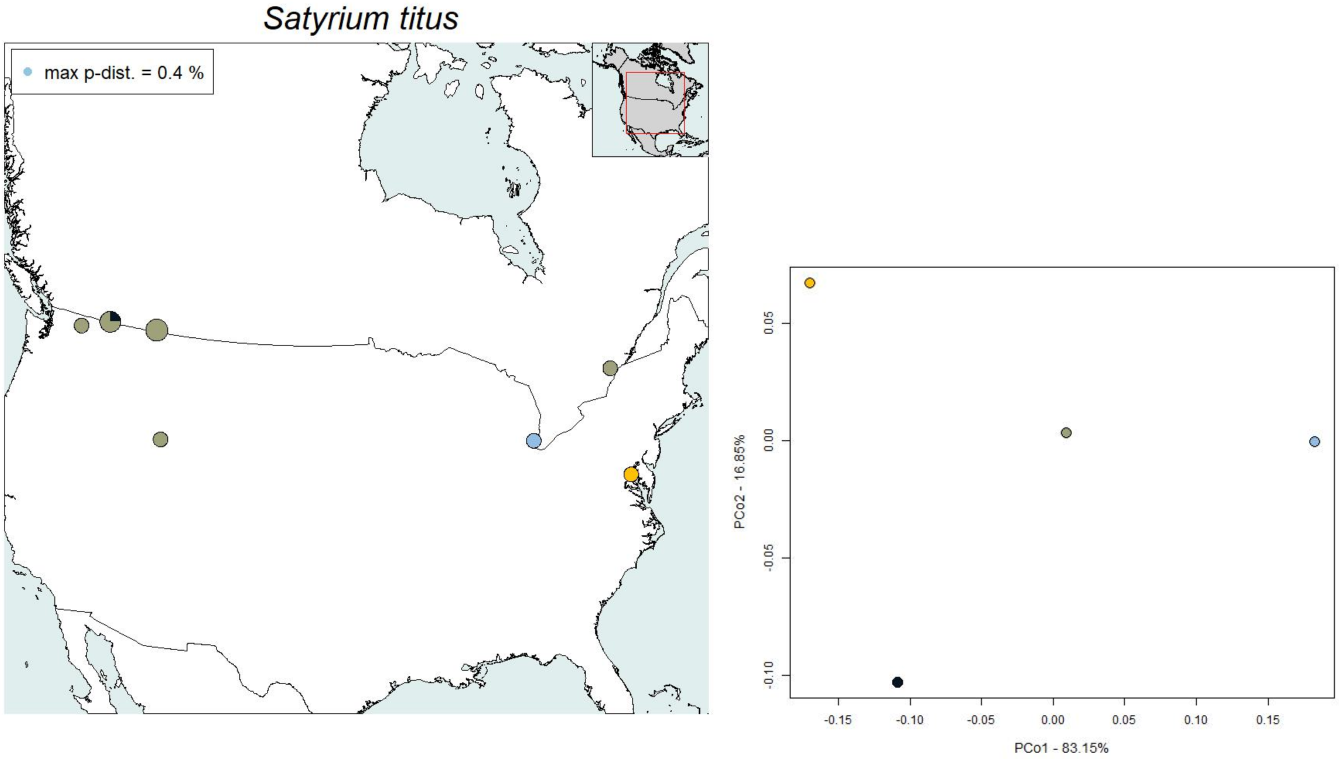

Figure 521 Map of *Satyrium titus* showing the localities of the sequenced specimens (left). Nearby localities are grouped in pies. Colours match the bidimensional colour space of the PCoA projection (right) of max p-dists among sequences (dots). Sequences= 14; Hap obs.= 4; Hap asympt.= 6.8; Hap % obs.= 58.9%; GST= NaN; DST= NaN; HD= 0.396; ND= 0.0007; max p-dist= 0.4%.

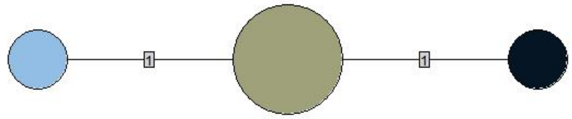

Figure 522: Haplotype network of *Satyrium titus*. Sequences > 599 bp= 13.

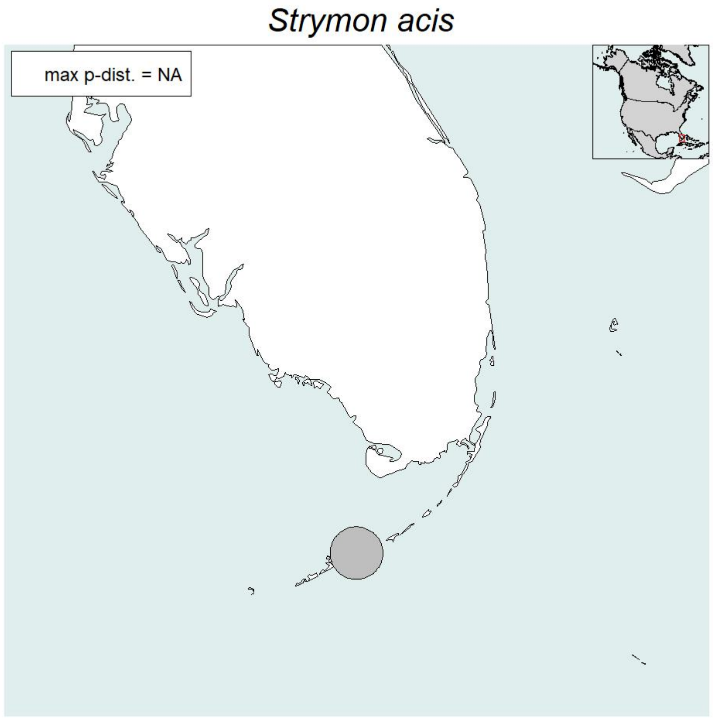

Figure 523: Map of *Strymon acis* showing the localities of the sequenced specimens. Nearby localities are grouped in pies. Due to the presence of a single haplotype PCoA projection was not done and a single grey colour was plotted on the map. Sequences= 1; Hap obs.= NA; Hap asympt.= NA; Hap % obs.= NA; GST= NaN; DST= NaN; HD= NA; ND= NA; max p-dist= NA.

Haplotype network analysis and bubble plot of *Strymon acis* were not possible. Sequences > 599 bp = 1.

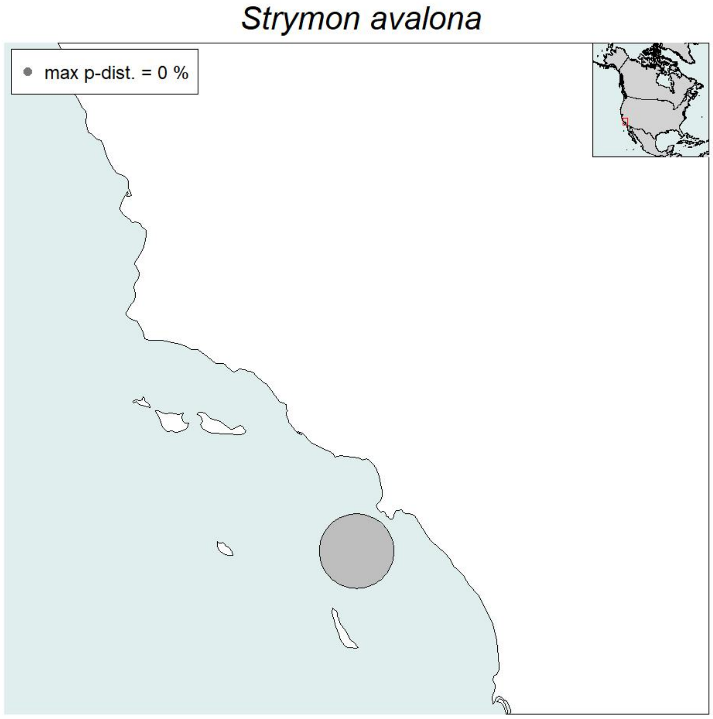

Figure 524: Map of *Strymon avalona* showing the localities of the sequenced specimens. Nearby localities are grouped in pies. Due to the presence of a single haplotype PCoA projection was not done and a single grey colour was plotted on the map. Sequences= 4; Hap obs.= 1; Hap asympt.= NA; Hap % obs.= NA%; GST= NaN; DST= NaN; HD= NA; ND= NA; max p-dist= 0%.

Haplotype network analysis and bubble plot of *Strymon avalona* were not possible. Sequences > 599 bp = 4.

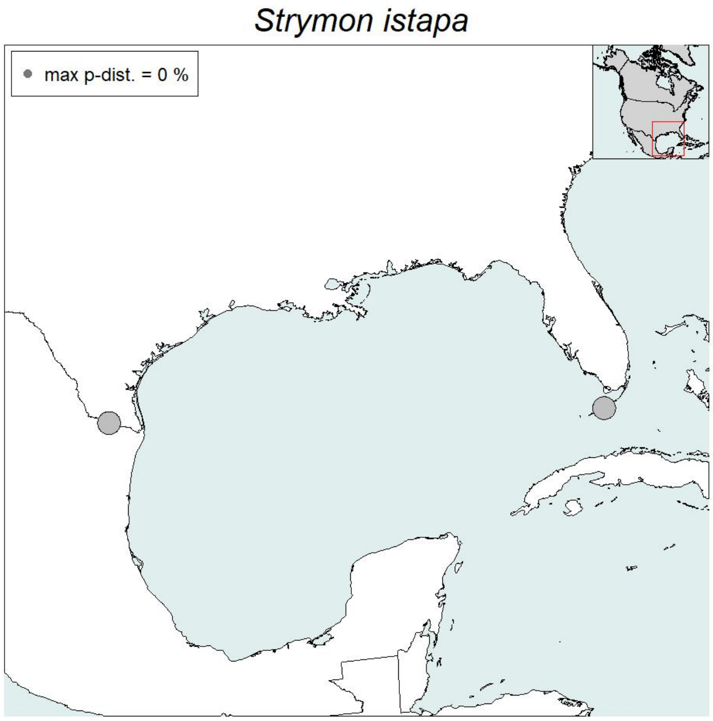

Figure 525: Map of *Strymon istapa* showing the localities of the sequenced specimens. Nearby localities are grouped in pies. Due to the presence of a single haplotype PCoA projection was not done and a single grey colour was plotted on the map. Sequences= 2; Hap obs.= 1; Hap asympt.= NA; Hap % obs.= NA%; GST= NaN; DST= NaN; HD= NA; ND= NA; max p-dist= 0%.

Haplotype network analysis and bubble plot of *Strymon istapa* were not possible. Sequences > 599 bp = 2.

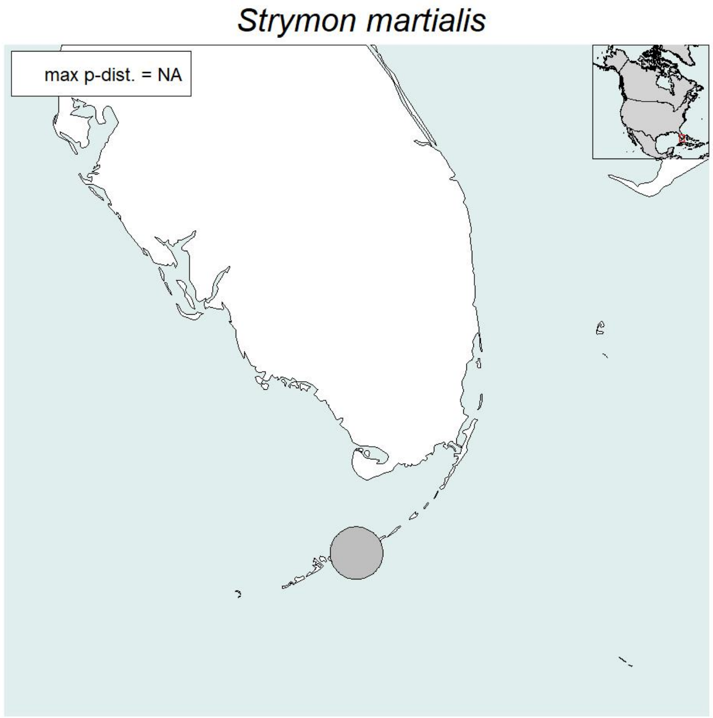

Figure 526: Map of *Strymon martialis* showing the localities of the sequenced specimens. Nearby localities are grouped in pies. Due to the presence of a single haplotype PCoA projection was not done and a single grey colour was plotted on the map. Sequences= 1; Hap obs.= NA; Hap asympt.= NA; Hap % obs.= NA; GST= NaN; DST= NaN; HD= NA; ND= NA; max p-dist= NA.

Haplotype network analysis and bubble plot of *Strymon martialis* were not possible. Sequences > 599 bp = 1.

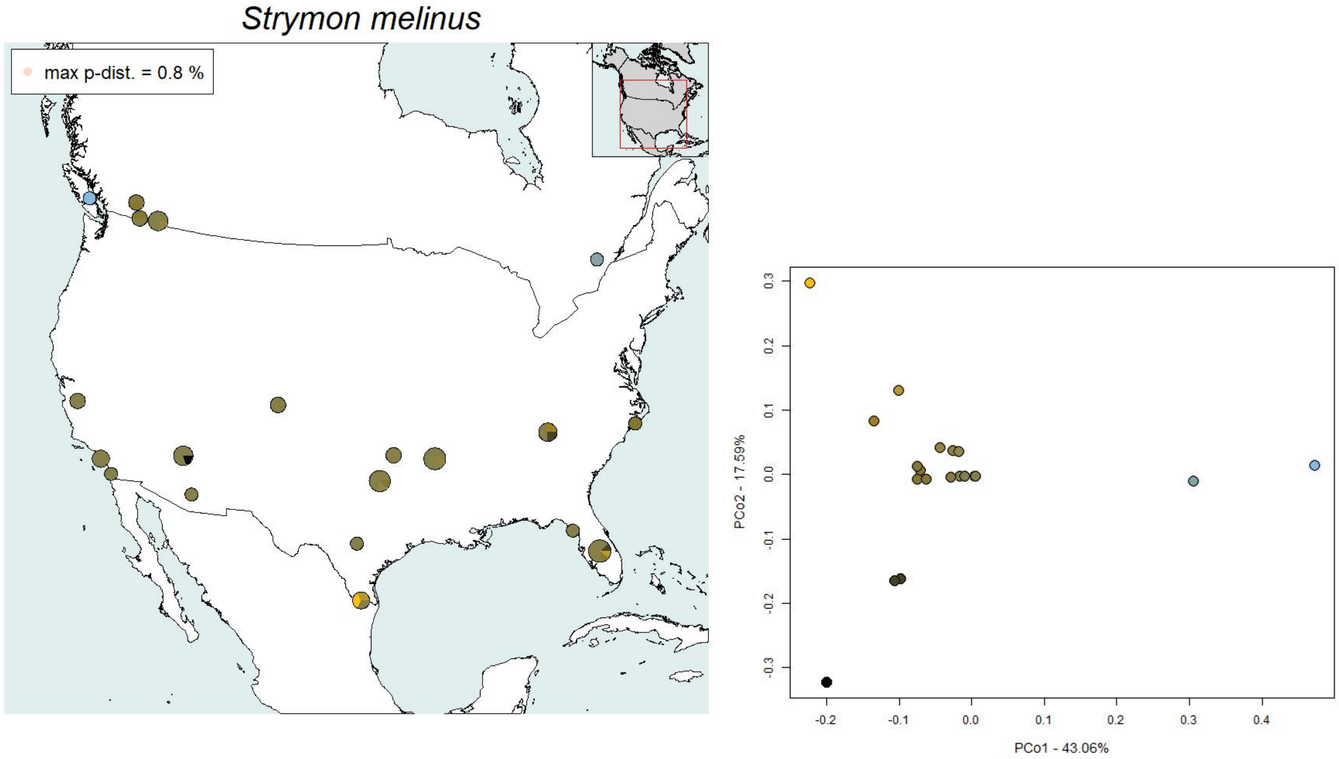

Figure 527 Map of *Strymon melinus* showing the localities of the sequenced specimens (left). Nearby localities are grouped in pies. Colours match the bidimensional colour space of the PCoA projection (right) of max p-dists among sequences (dots). Sequences= 62; Hap obs.= 14; Hap asympt.= 27.3; Hap % obs.= 51.3%; GST= 0.223; DST= 0.0002; HD= 0.497; ND= 0.0011; max p-dist= 0.8%.

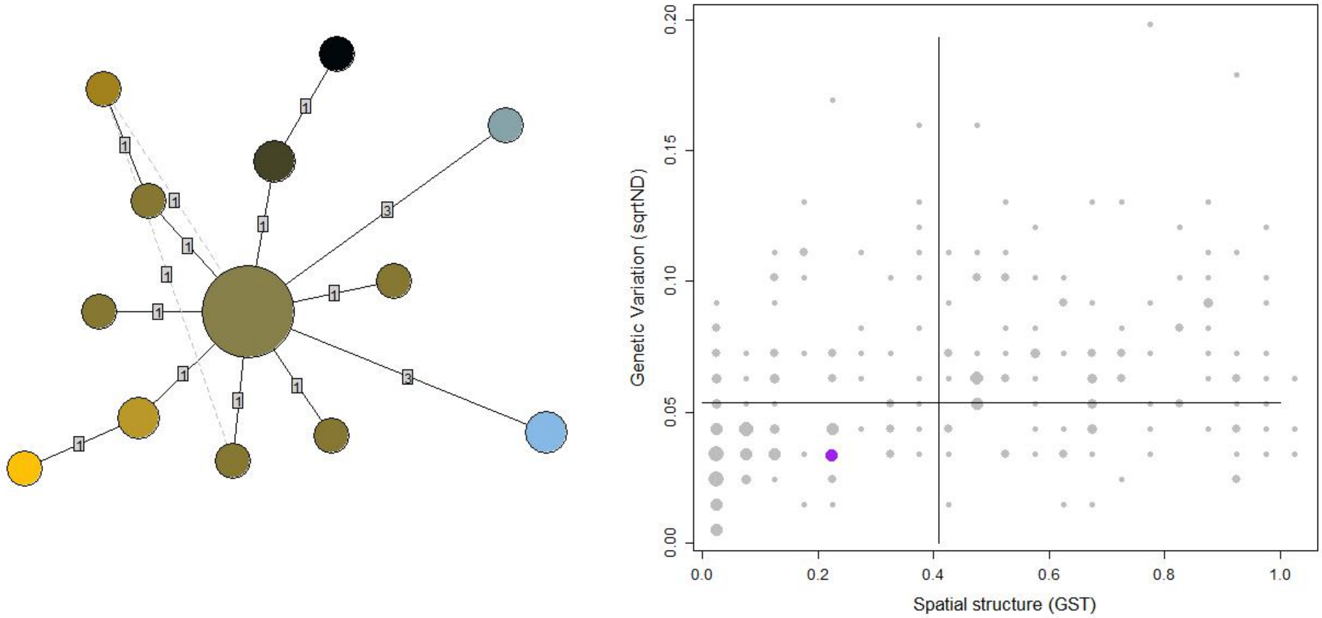

Figure 528: Haplotype network (left) of *Strymon melinus* sequences > 599 bp with colours matching the PCoA colour space (above). The bubble plot for mt-DNA polymorphism (square root transformed nucleotide diversity) and spatial structure (GST) among all species in the atlas and values for *Strymon melinus* (purple dot). The horizontal and vertical lines represent median values of nucleotide diversity and GST, respectively. Sequences > 599 bp= 60.

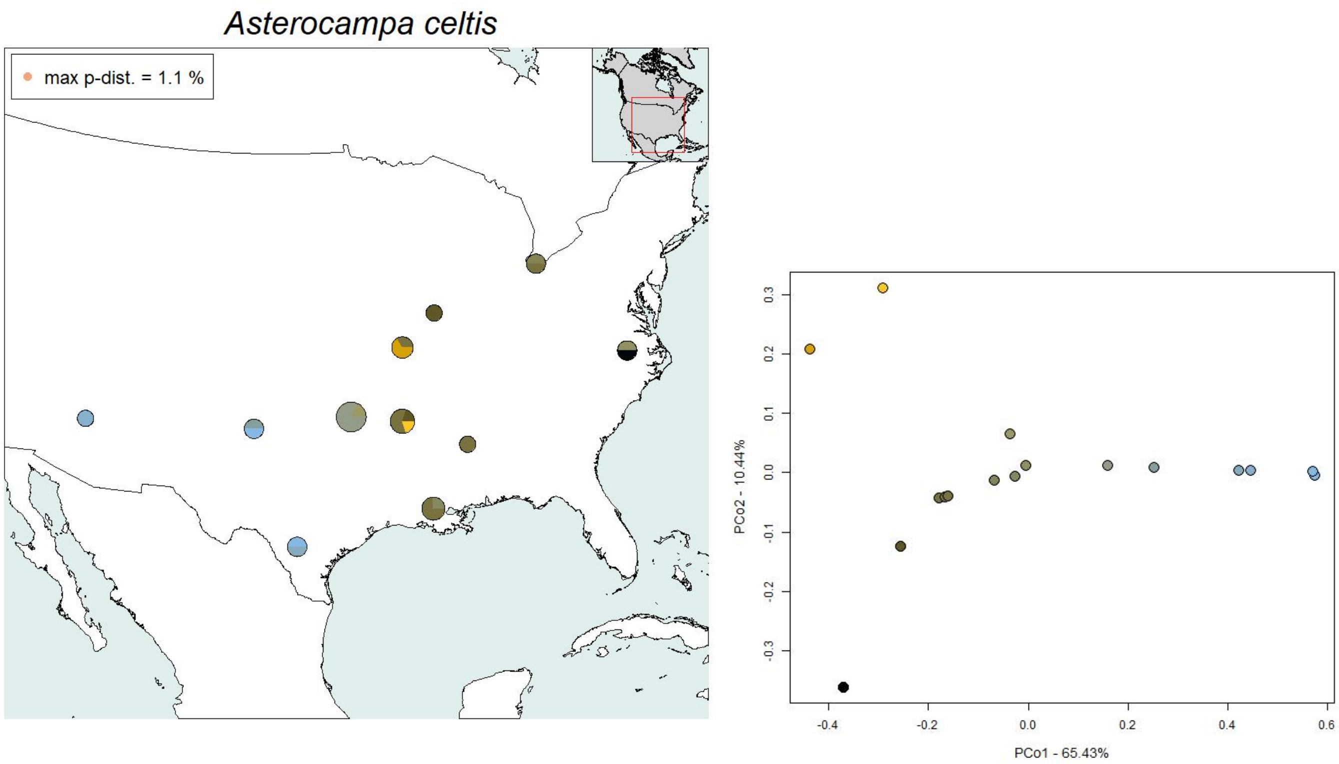

Figure 529 Map of *Asterocampa celtis* showing the localities of the sequenced specimens (left). Nearby localities are grouped in pies. Colours match the bidimensional colour space of the PCoA projection (right) of max p-dists among sequences (dots). Sequences= 34; Hap obs.= 15; Hap asympt.= 73.7; Hap % obs.= 20.3%; GST= 0.548; DST= 0.0015; HD= 0.865; ND= 0.0035; max p-dist= 1.1%.

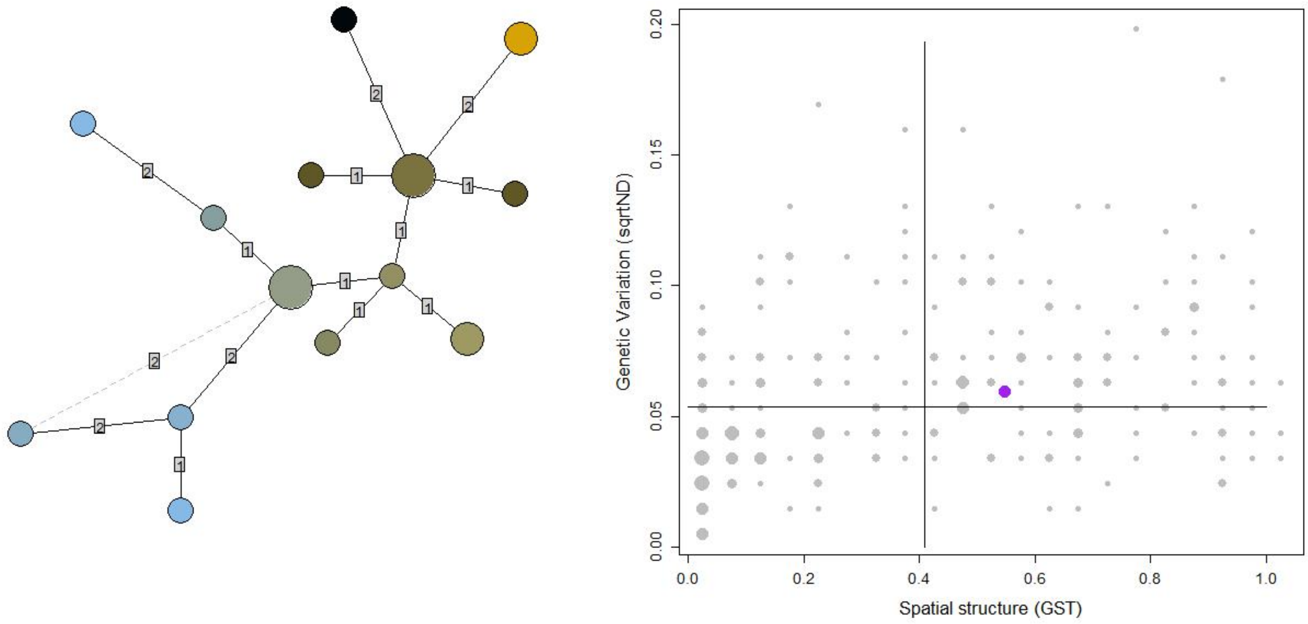

Figure 530: Haplotype network (left) of *Asterocampa celtis* sequences > 599 bp with colours matching the PCoA colour space (above). The bubble plot for mt-DNA polymorphism (square root transformed nucleotide diversity) and spatial structure (GST) among all species in the atlas and values for *Asterocampa celtis* (purple dot). The horizontal and vertical lines represent median values of nucleotide diversity and GST, respectively. Sequences > 599 bp= 34.

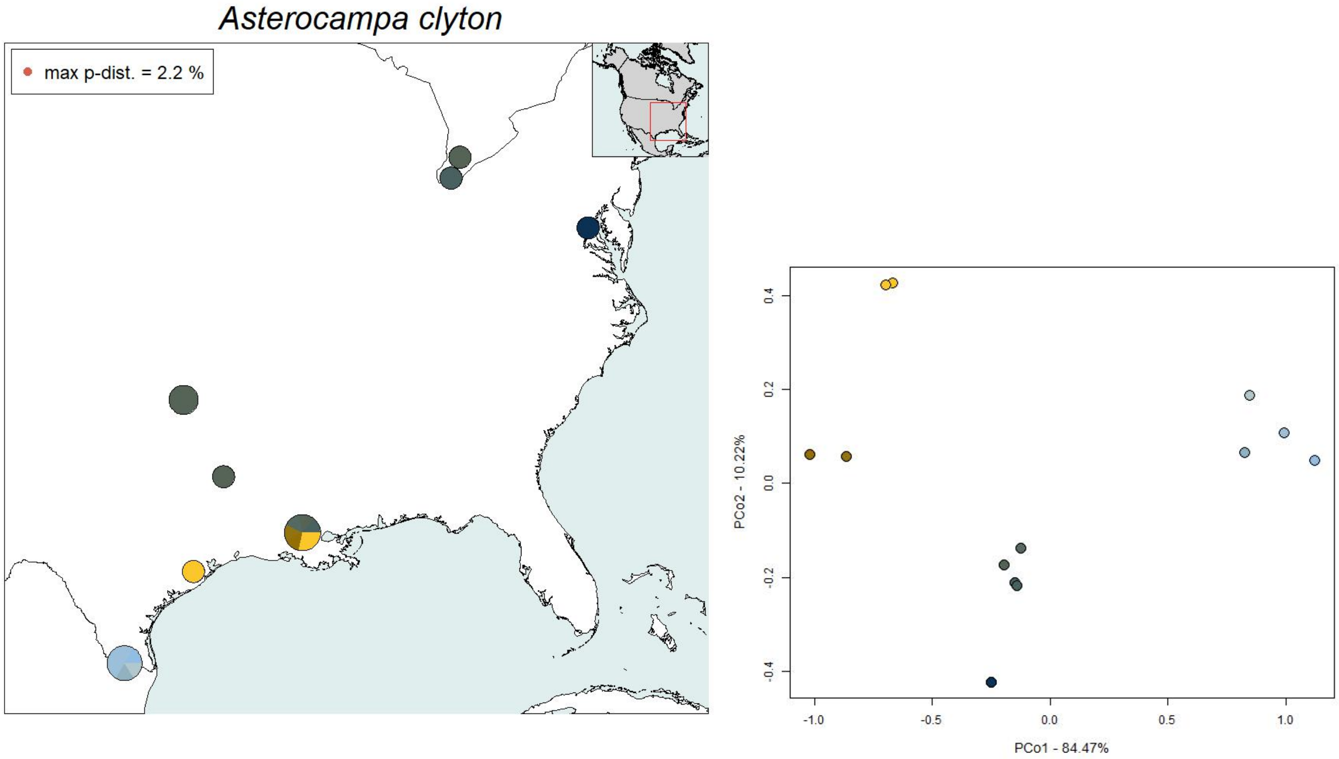

Figure 531 Map of *Asterocampa clyton* showing the localities of the sequenced specimens (left). Nearby localities are grouped in pies. Colours match the bidimensional colour space of the PCoA projection (right) of max p-dists among sequences (dots). Sequences= 21; Hap obs.= 9; Hap asympt.= 23.3; Hap % obs.= 38.6%; GST= 0.625; DST= 0.0055; HD= 0.8; ND= 0.008; max p-dist= 2.2%.

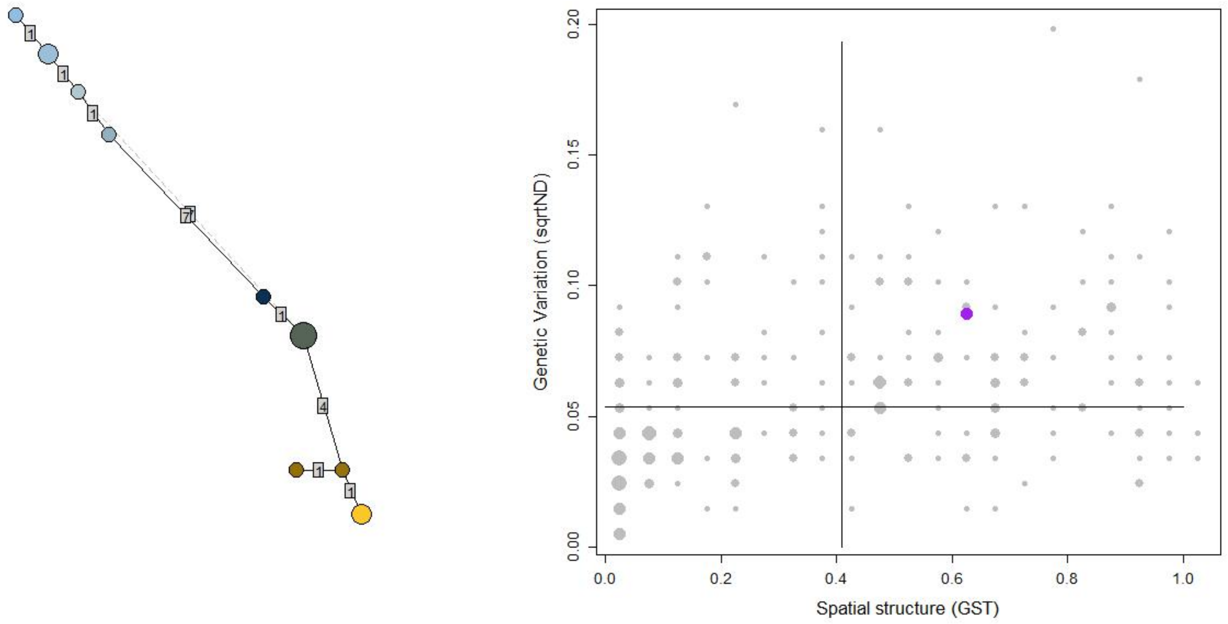

Figure 532: Haplotype network (left) of *Asterocampa clyton* sequences > 599 bp with colours matching the PCoA colour space (above). The bubble plot for mt-DNA polymorphism (square root transformed nucleotide diversity) and spatial structure (GST) among all species in the atlas and values for *Asterocampa clyton* (purple dot). The horizontal and vertical lines represent median values of nucleotide diversity and GST, respectively. Sequences > 599 bp= 21.

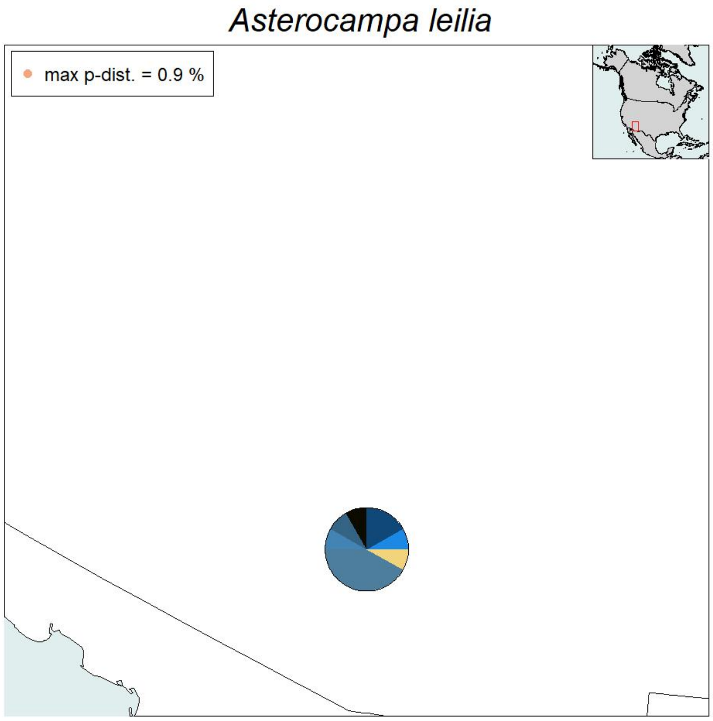

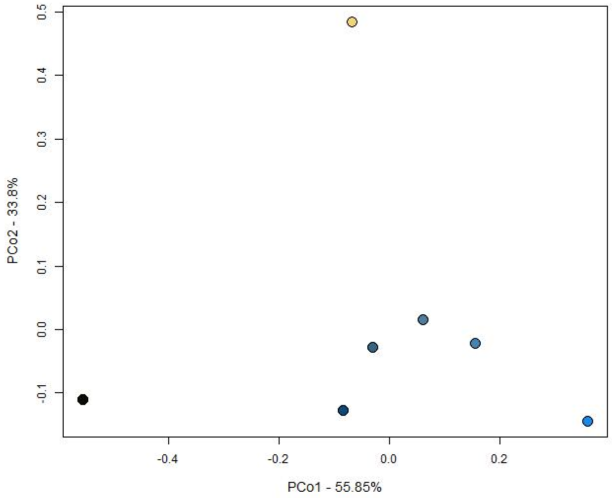

Figure 533 Map of *Asterocampa leilia* showing the localities of the sequenced specimens (left). Nearby localities are grouped in pies. Colours match the bidimensional colour space of the PCoA projection (right) of max p-dists among sequences (dots). Sequences= 12; Hap obs.= 7; Hap asympt.= 18.5; Hap % obs.= 37.9%; GST= 0; DST= 0; HD= 0.833; ND= 0.0028; max p-dist= 0.9%.

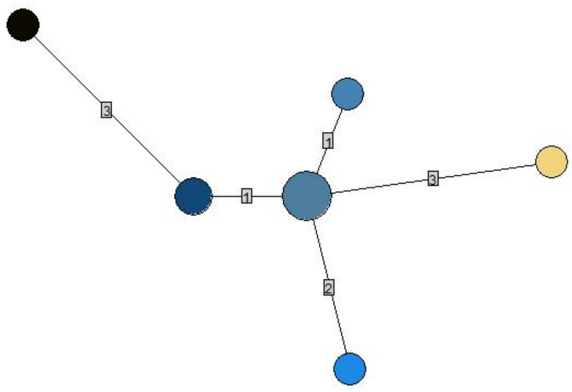

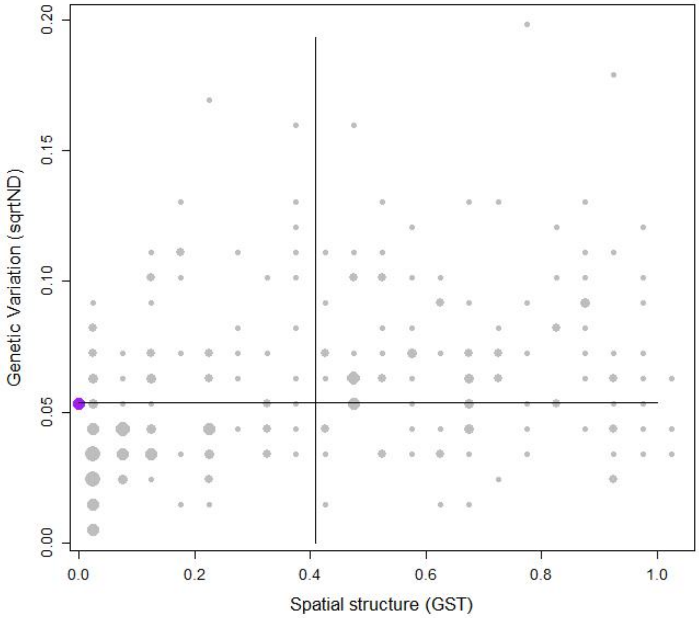

Figure 534: Haplotype network (left) of *Asterocampa leilia* sequences > 599 bp with colours matching the PCoA colour space (above). The bubble plot for mt-DNA polymorphism (square root transformed nucleotide diversity) and spatial structure (GST) among all species in the atlas and values for *Asterocampa leilia* (purple dot). The horizontal and vertical lines represent median values of nucleotide diversity and GST, respectively. Sequences > 599 bp= 12.

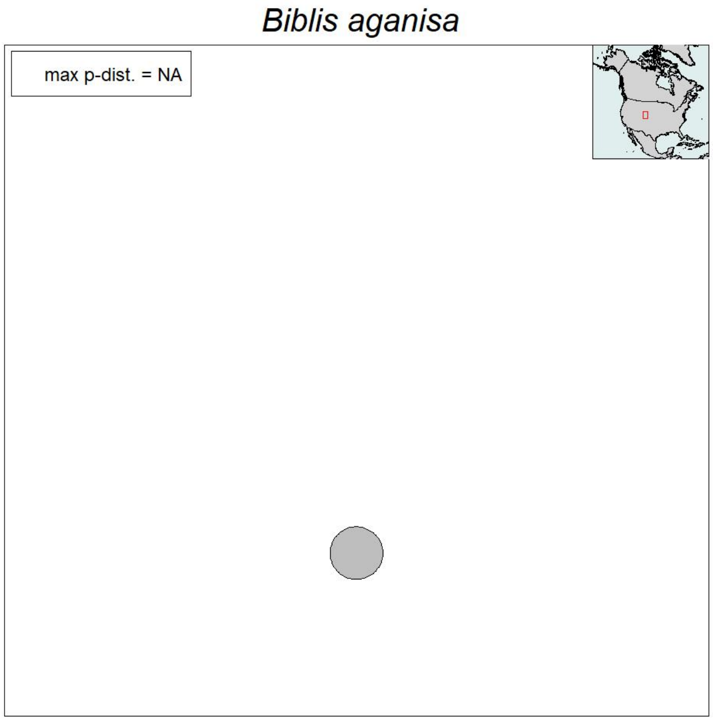

Figure 535: Map of *Biblis aganisa* showing the localities of the sequenced specimens. Nearby localities are grouped in pies. Due to the presence of a single haplotype PCoA projection was not done and a single grey colour was plotted on the map. Sequences= 1; Hap obs.= NA; Hap asympt.= NA; Hap % obs.= NA; GST= NaN; DST= NaN; HD= NA; ND= NA; max p-dist= NA.

Haplotype network analysis and bubble plot of *Biblis aganisa* were not possible. Sequences > 599 bp = 0.

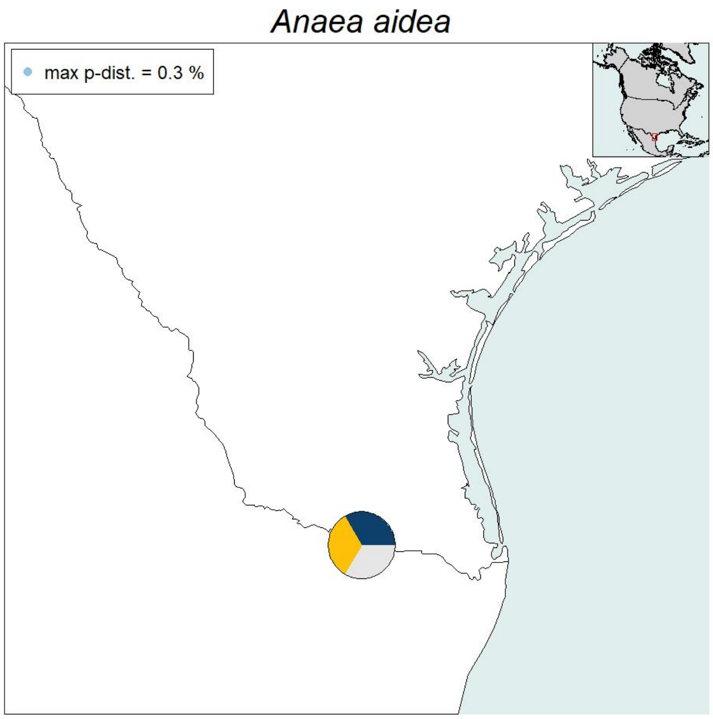

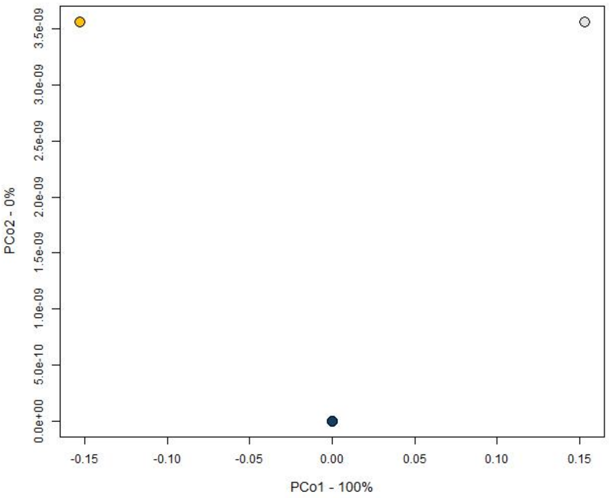

Figure 536 Map of *Anaea aidea* showing the localities of the sequenced specimens (left). Nearby localities are grouped in pies. Colours match the bidimensional colour space of the PCoA projection (right) of max p-dists among sequences (dots). Sequences= 3; Hap obs.= 3; Hap asympt.= NA; Hap % obs.= NA%; GST= NaN; DST= NaN; HD= NA; ND= NA; max p-dist= 0.3%.

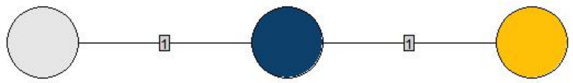

Figure 537: Haplotype network of *Anaea aidea*. Sequences > 599 bp= 3.

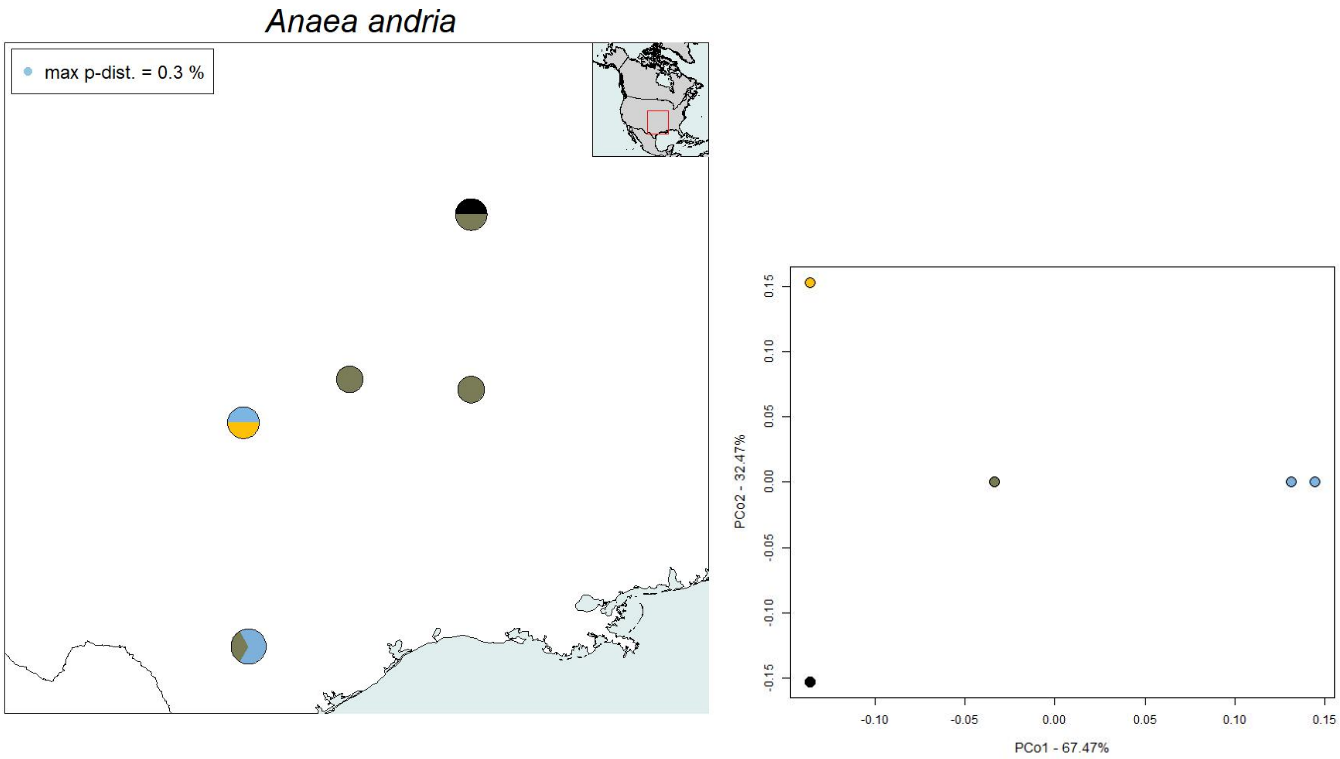

Figure 538 Map of *Anaea andria* showing the localities of the sequenced specimens (left). Nearby localities are grouped in pies. Colours match the bidimensional colour space of the PCoA projection (right) of max p-dists among sequences (dots). Sequences= 9; Hap obs.= 4; Hap asympt.= NA; Hap % obs.= NA%; GST= NaN; DST= NaN; HD= NA; ND= NA; max p-dist= 0.3%.

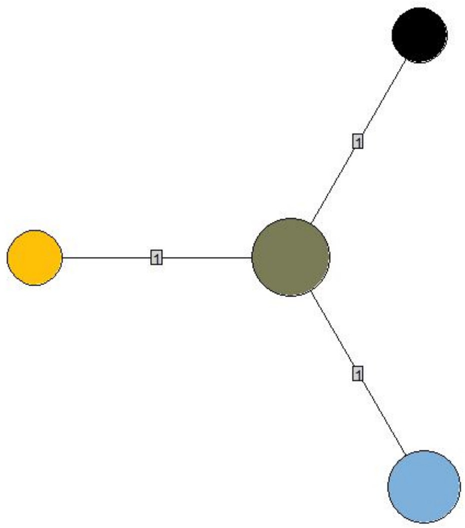

Figure 539: Haplotype network of *Anaea andria*. Sequences > 599 bp= 9.

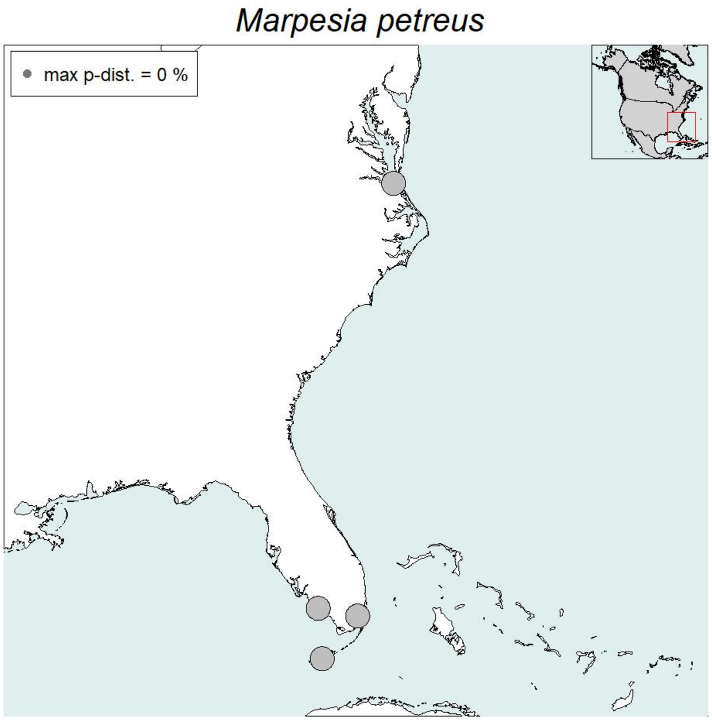

Figure 540: Map of *Marpesia petreus* showing the localities of the sequenced specimens. Nearby localities are grouped in pies. Due to the presence of a single haplotype PCoA projection was not done and a single grey colour was plotted on the map. Sequences= 4; Hap obs.= 1; Hap asympt.= NA; Hap % obs.= NA%; GST= NaN; DST= NaN; HD= NA; ND= NA; max p-dist= 0%.

Haplotype network analysis and bubble plot of *Marpesia petreus* were not possible. Sequences > 599 bp = 4.

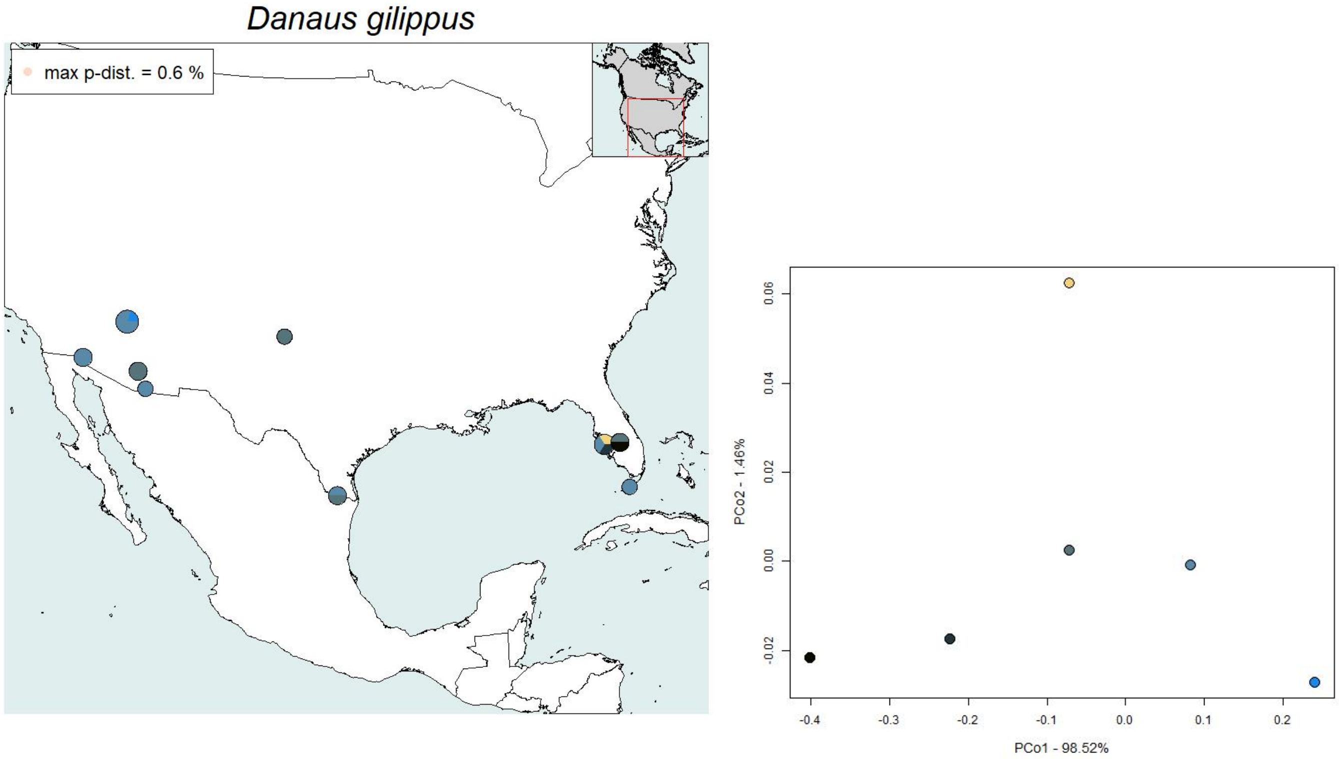

Figure 541 Map of *Danaus gilippus* showing the localities of the sequenced specimens (left). Nearby localities are grouped in pies. Colours match the bidimensional colour space of the PCoA projection (right) of max p-dists among sequences (dots). Sequences= 19; Hap obs.= 5; Hap asympt.= 7.8; Hap % obs.= 63.8%; GST= 0.217; DST= 0.0004; HD= 0.649; ND= 0.0014; max p-dist= 0.6%.

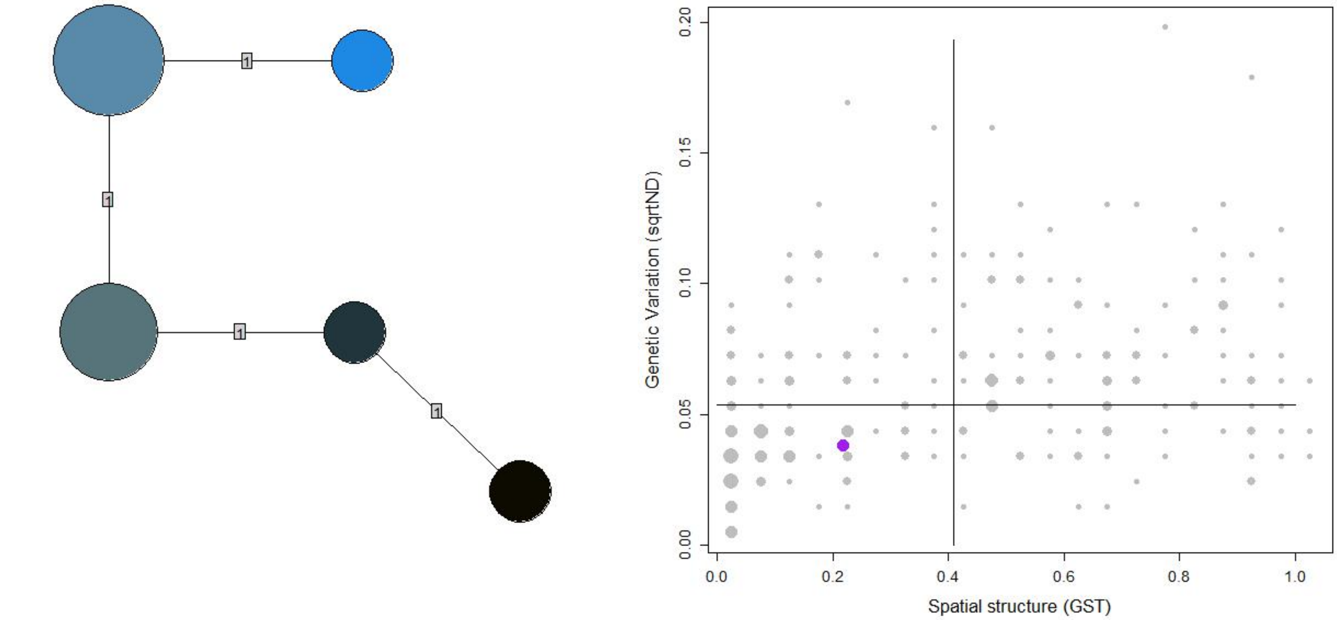

Figure 542: Haplotype network (left) of *Danaus gilippus* sequences > 599 bp with colours matching the PCoA colour space (above). The bubble plot for mt-DNA polymorphism (square root transformed nucleotide diversity) and spatial structure (GST) among all species in the atlas and values for *Danaus gilippus* (purple dot). The horizontal and vertical lines represent median values of nucleotide diversity and GST, respectively. Sequences > 599 bp= 19.

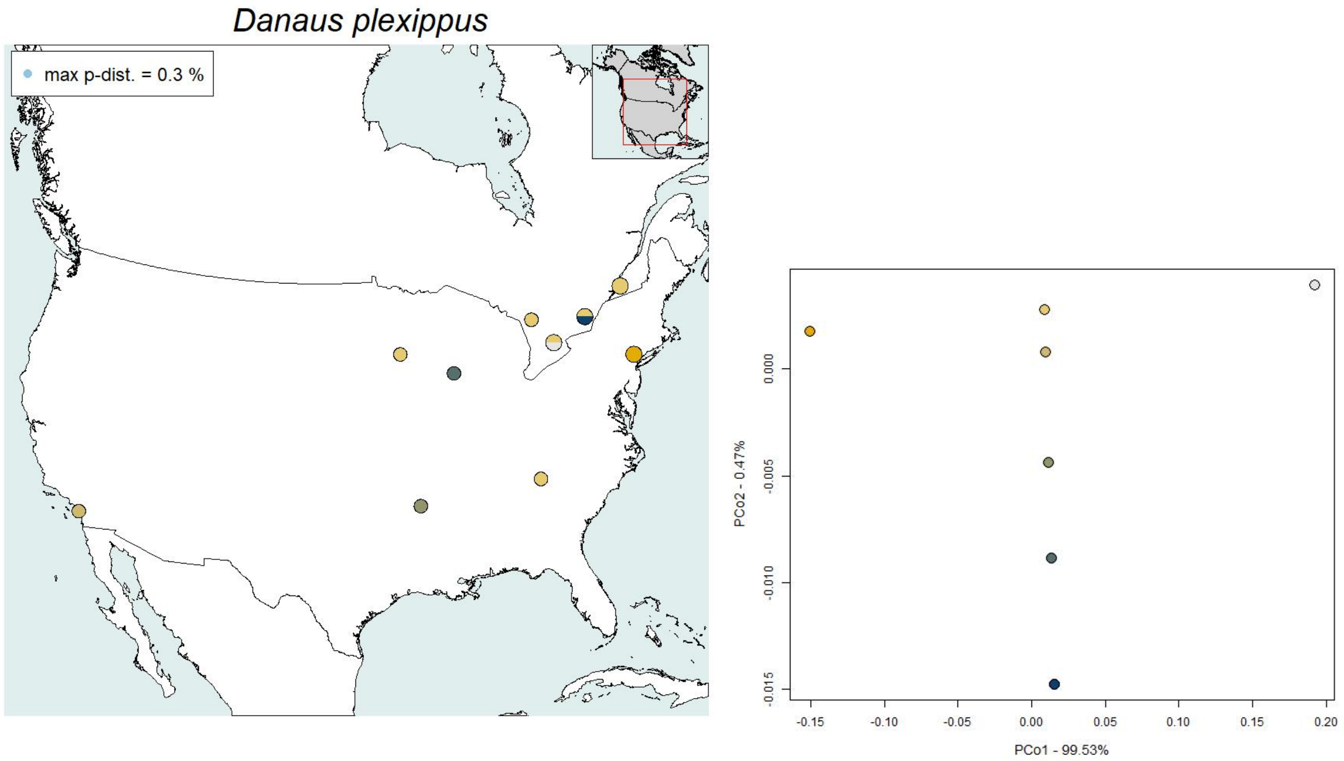

Figure 543 Map of *Danaus plexippus* showing the localities of the sequenced specimens (left). Nearby localities are grouped in pies. Colours match the bidimensional colour space of the PCoA projection (right) of max p-dists among sequences (dots). Sequences= 14; Hap obs.= 3; Hap asympt.= 3.5; Hap % obs.= 86.6%; GST= NaN; DST= NaN; HD= 0.385; ND= 0.0007; max p-dist= 0.3%.

Haplotype network analysis and bubble plot of *Danaus plexippus* were not possible. Sequences > 599 bp = 13.

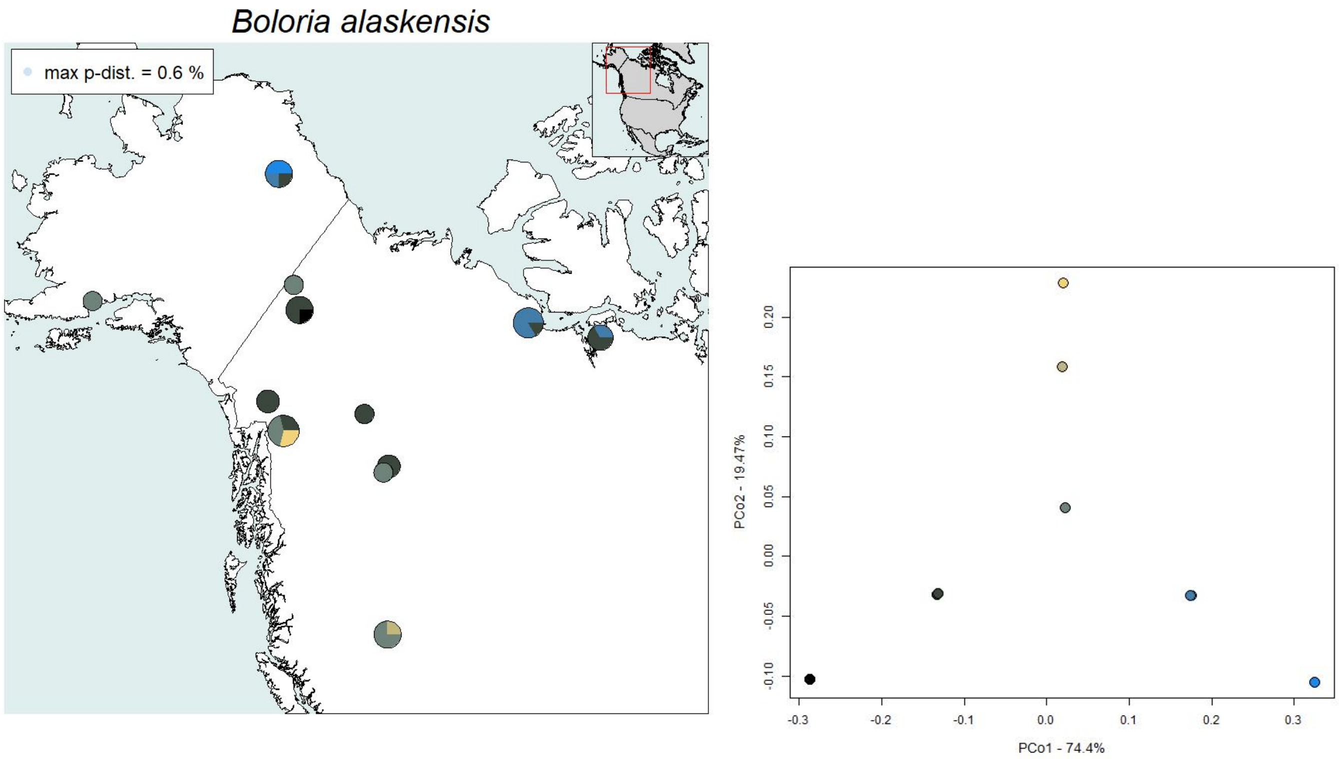

Figure 544 Map of *Boloria alaskensis* showing the localities of the sequenced specimens (left). Nearby localities are grouped in pies. Colours match the bidimensional colour space of the PCoA projection (right) of max p-dists among sequences (dots). Sequences= 36; Hap obs.= 7; Hap asympt.= 8; Hap % obs.= 87.8%; GST= 0.348; DST= 0.0007; HD= 0.762; ND= 0.0019; max p-dist= 0.6%.

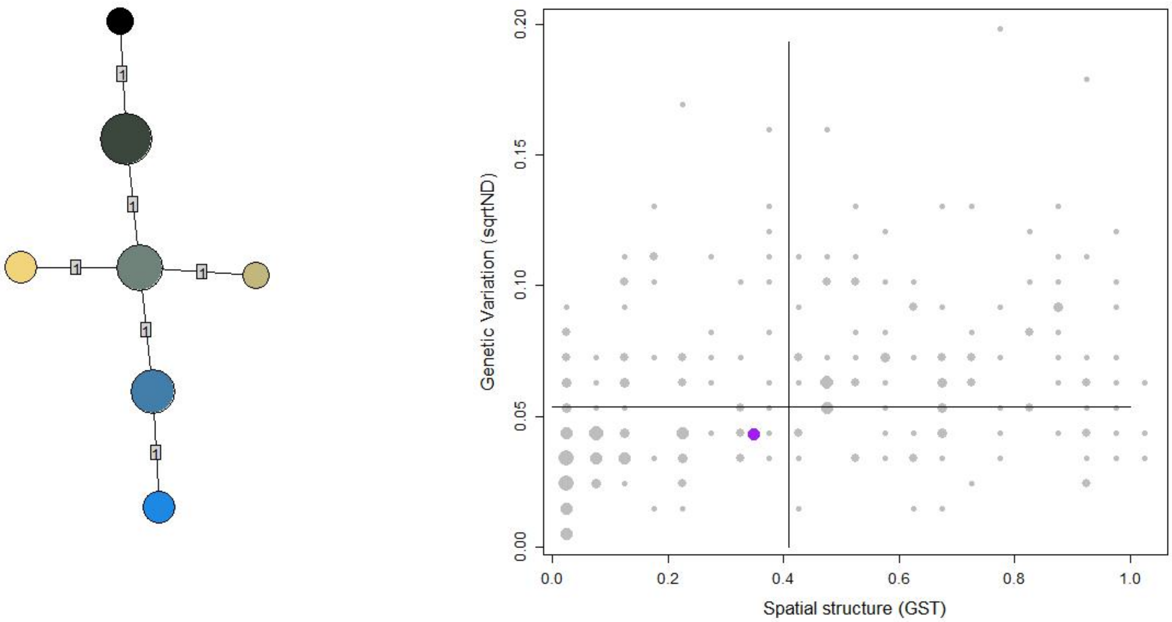

Figure 545: Haplotype network (left) of *Boloria alaskensis* sequences > 599 bp with colours matching the PCoA colour space (above). The bubble plot for mt-DNA polymorphism (square root transformed nucleotide diversity) and spatial structure (GST) among all species in the atlas and values for *Boloria alaskensis* (purple dot). The horizontal and vertical lines represent median values of nucleotide diversity and GST, respectively. Sequences > 599 bp= 36.

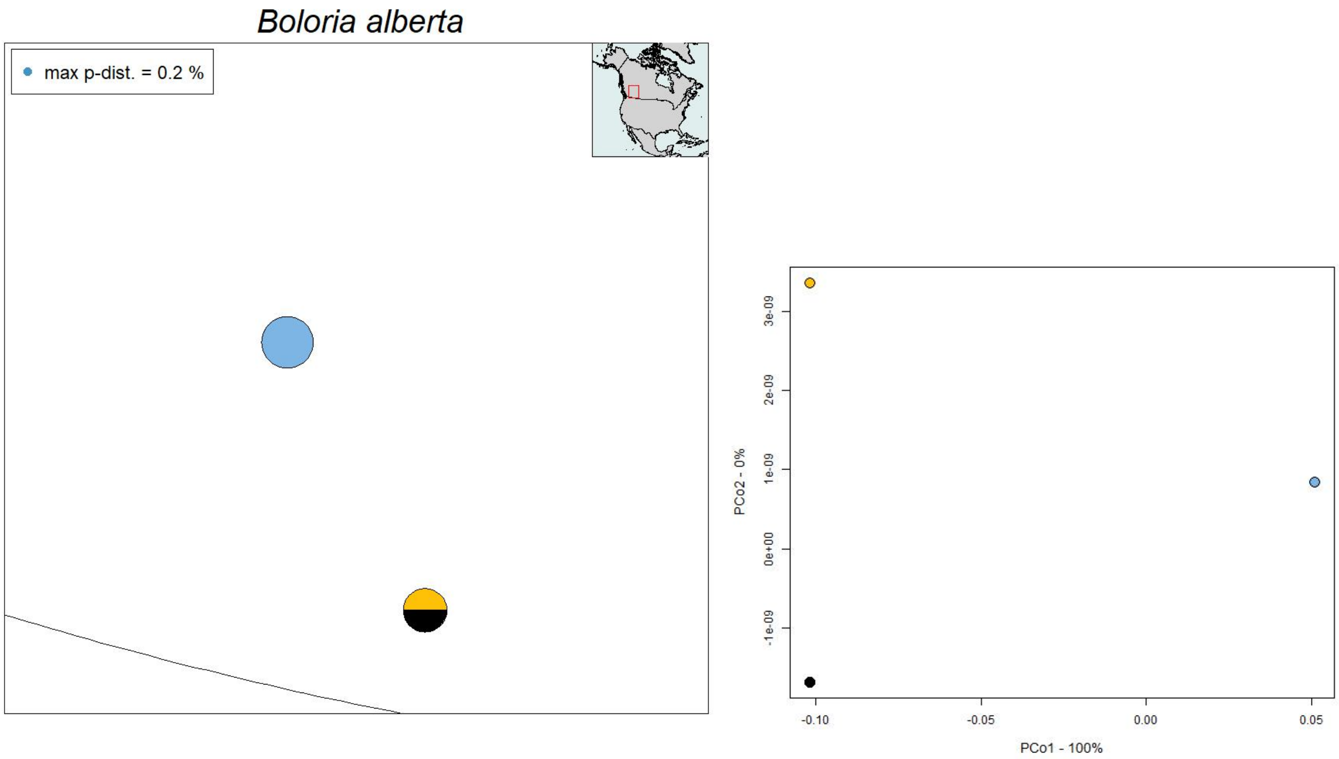

Figure 546 Map of *Boloria alberta* showing the localities of the sequenced specimens (left). Nearby localities are grouped in pies. Colours match the bidimensional colour space of the PCoA projection (right) of max p-dists among sequences (dots). Sequences= 6; Hap obs.= 2; Hap asympt.= NA; Hap % obs.= NA%; GST= NaN; DST= NaN; HD= NA; ND= NA; max p-dist= 0.2%.

Haplotype network analysis and bubble plot of *Boloria alberta* were not possible. Sequences > 599 bp = 6.

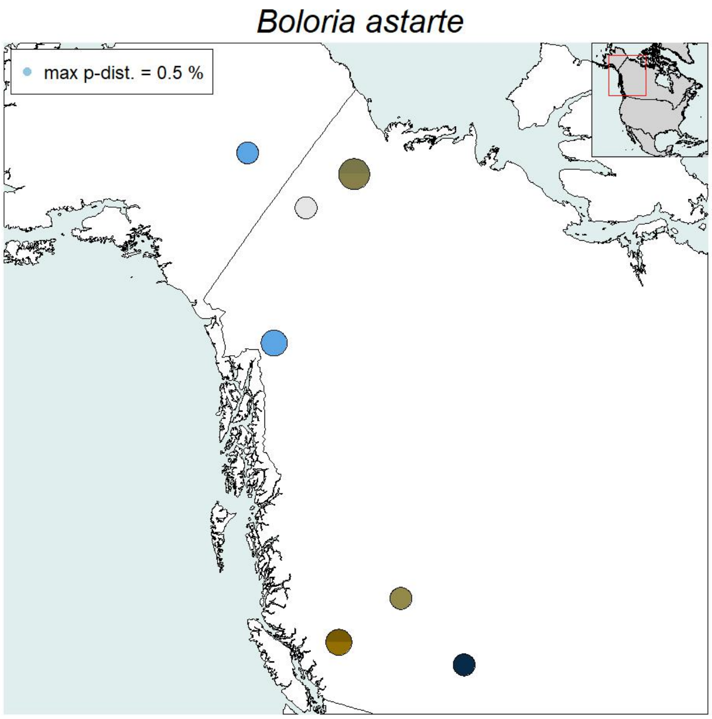

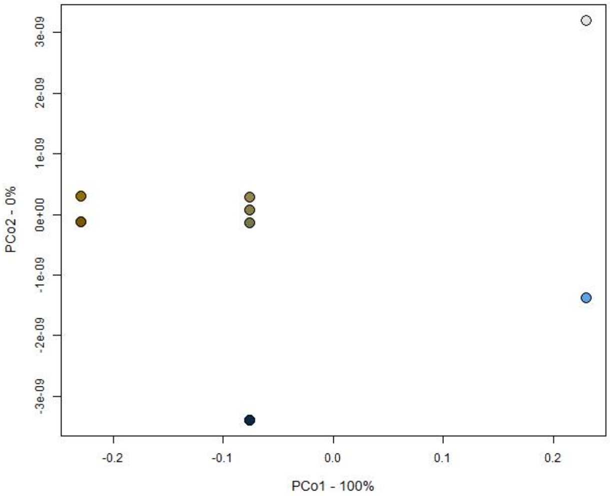

Figure 547 Map of *Boloria astarte* showing the localities of the sequenced specimens (left). Nearby localities are grouped in pies. Colours match the bidimensional colour space of the PCoA projection (right) of max p-dists among sequences (dots). Sequences= 12; Hap obs.= 3; Hap asympt.= 3; Hap % obs.= 100%; GST= NaN; DST= NaN; HD= 0.667; ND= 0.0019; max p-dist= 0.5%.

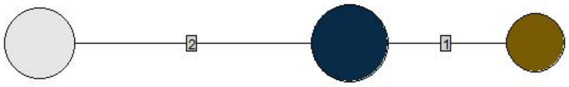

Figure 548: Haplotype network of *Boloria astarte*. Sequences > 599 bp= 12.

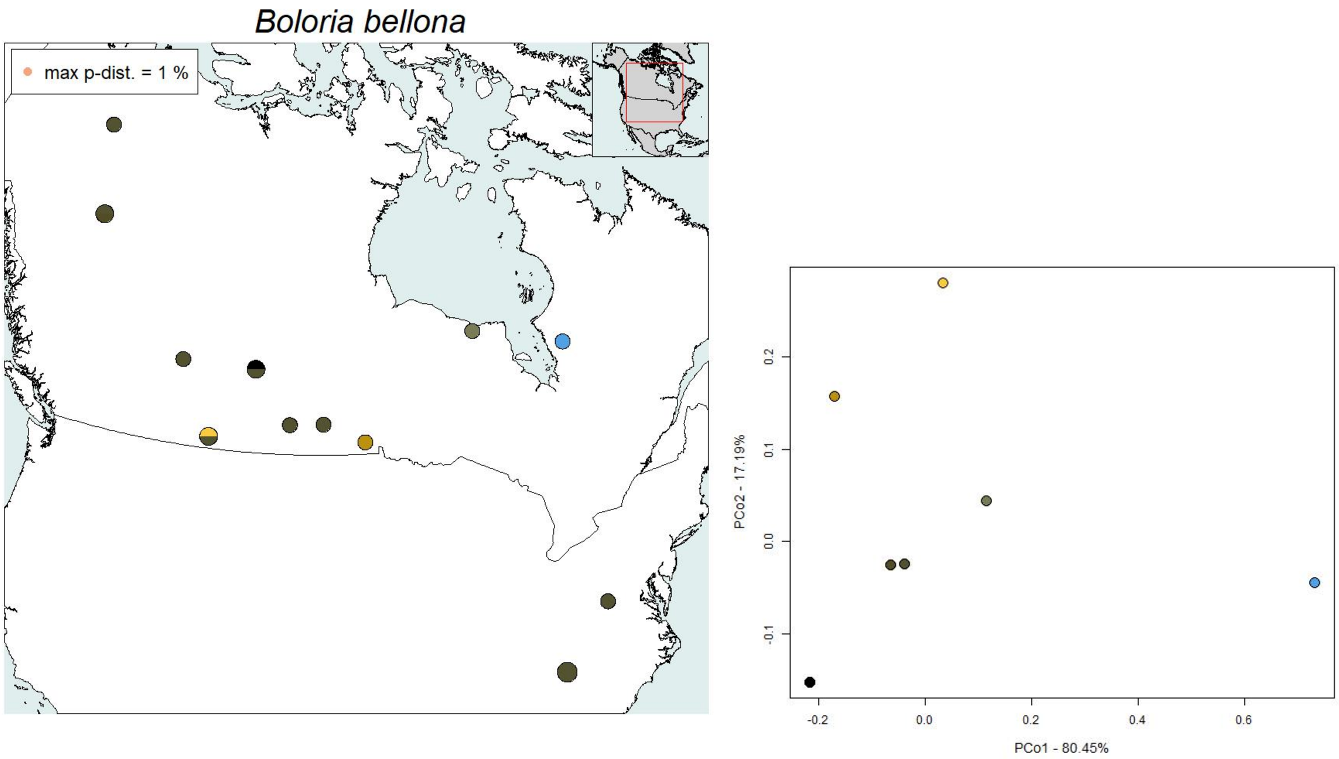

Figure 549 Map of *Boloria bellona* showing the localities of the sequenced specimens (left). Nearby localities are grouped in pies. Colours match the bidimensional colour space of the PCoA projection (right) of max p-dists among sequences (dots). Sequences= 17; Hap obs.= 6; Hap asympt.= 15.4; Hap % obs.= 38.9%; GST= NaN; DST= NaN; HD= 0.515; ND= 0.0017; max p-dist= 1%.

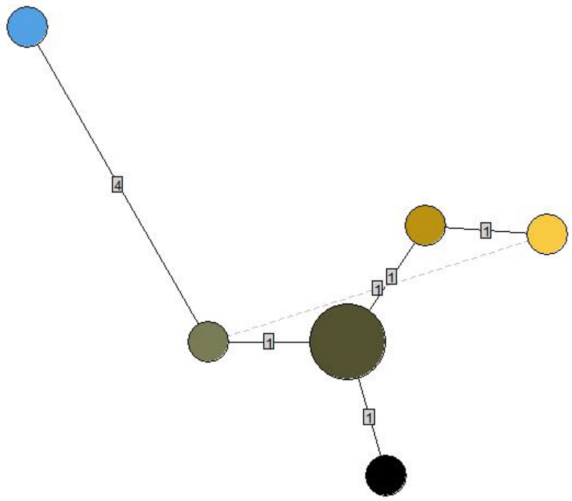

Figure 550: Haplotype network of *Boloria bellona*. Sequences > 599 bp= 17.

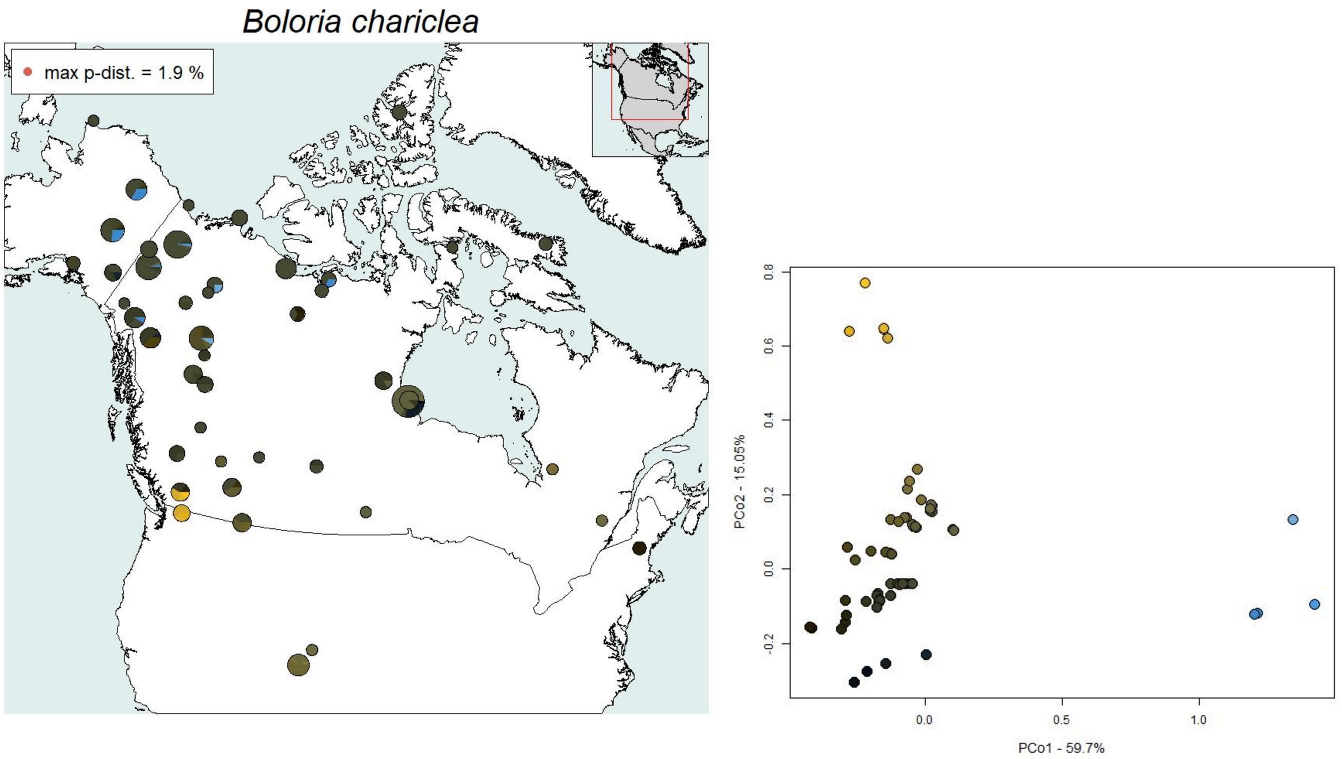

Figure 551 Map of *Boloria chariclea* showing the localities of the sequenced specimens (left). Nearby localities are grouped in pies. Colours match the bidimensional colour space of the PCoA projection (right) of max p-dists among sequences (dots). Sequences= 335; Hap obs.= 42; Hap asympt.= 145.9; Hap % obs.= 28.8%; GST= 0.345; DST= 0.0011; HD= 0.796; ND= 0.003; max p-dist= 1.9%.

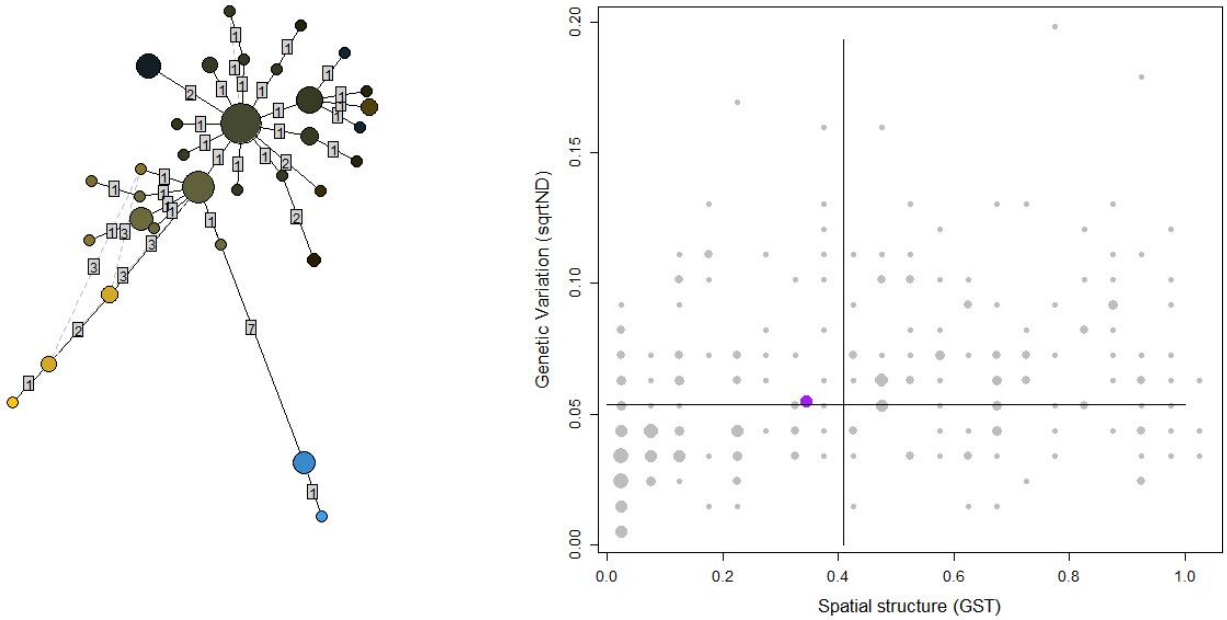

Figure 552: Haplotype network (left) of *Boloria chariclea* sequences > 599 bp with colours matching the PCoA colour space (above). The bubble plot for mt-DNA polymorphism (square root transformed nucleotide diversity) and spatial structure (GST) among all species in the atlas and values for *Boloria chariclea* (purple dot). The horizontal and vertical lines represent median values of nucleotide diversity and GST, respectively. Sequences > 599 bp= 301.

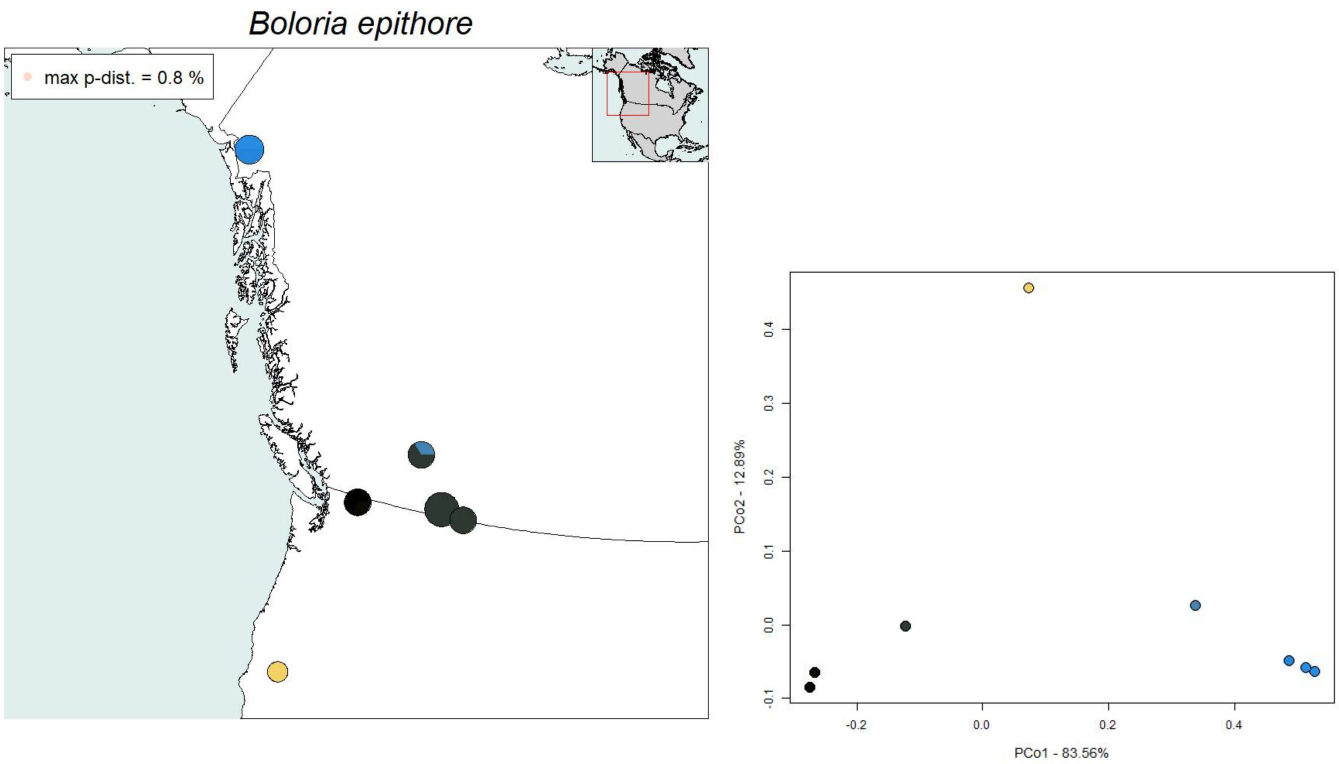

Figure 553 Map of *Boloria epithore* showing the localities of the sequenced specimens (left). Nearby localities are grouped in pies. Colours match the bidimensional colour space of the PCoA projection (right) of max p-dists among sequences (dots). Sequences= 22; Hap obs.= 6; Hap asympt.= 10.3; Hap % obs.= 58.3%; GST= 0.804; DST= 0.0022; HD= 0.632; ND= 0.003; max p-dist= 0.8%.

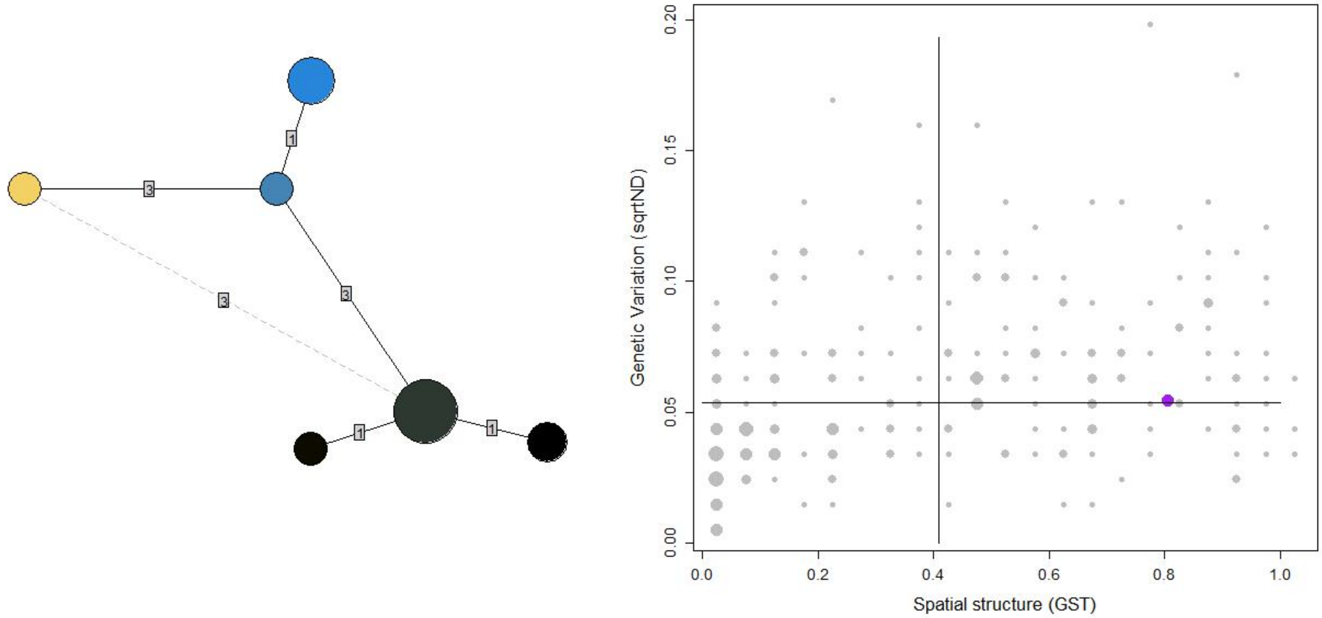

Figure 554: Haplotype network (left) of *Boloria epithore* sequences > 599 bp with colours matching the PCoA colour space (above). The bubble plot for mt-DNA polymorphism (square root transformed nucleotide diversity) and spatial structure (GST) among all species in the atlas and values for *Boloria epithore* (purple dot). The horizontal and vertical lines represent median values of nucleotide diversity and GST, respectively. Sequences > 599 bp= 22.

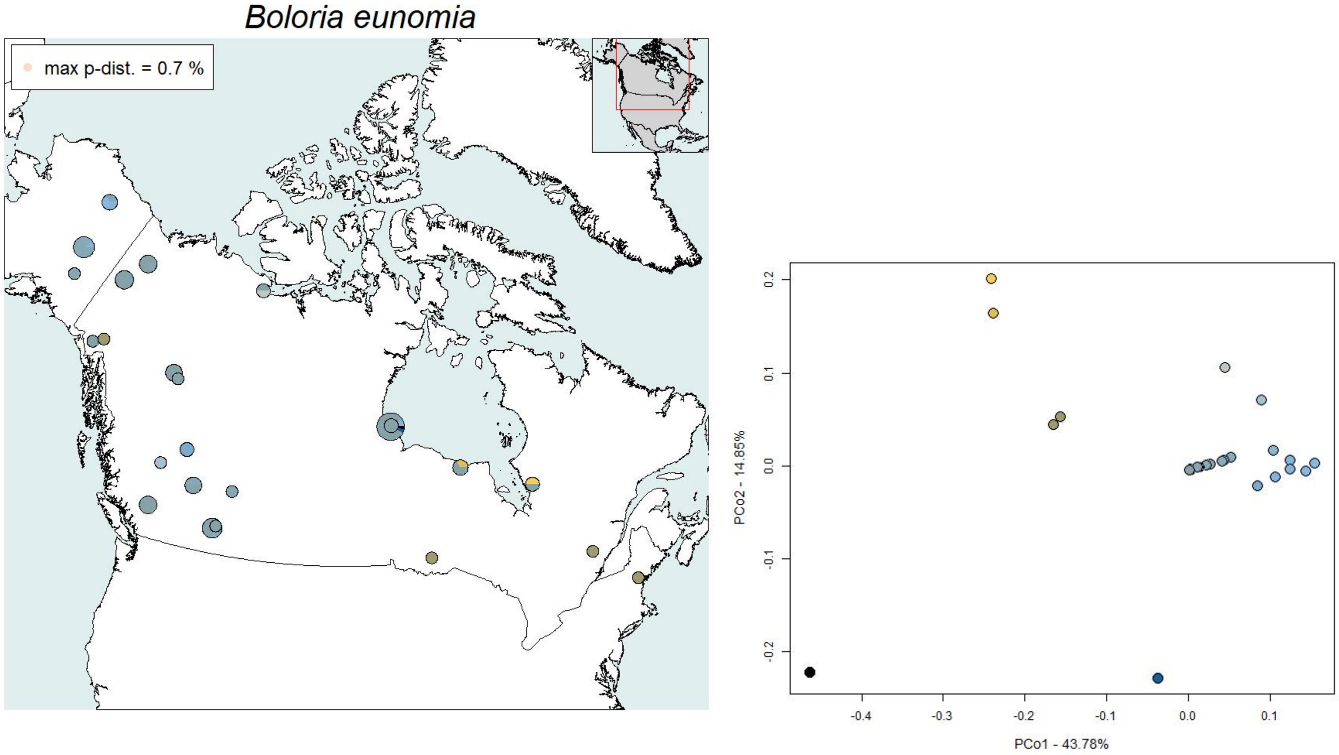

Figure 555 Map of *Boloria eunomia* showing the localities of the sequenced specimens (left). Nearby localities are grouped in pies. Colours match the bidimensional colour space of the PCoA projection (right) of max p-dists among sequences (dots). Sequences= 96; Hap obs.= 18; Hap asympt.= 59.8; Hap % obs.= 30.1%; GST= 0; DST= 0; HD= 0.577; ND= 0.0007; max p-dist= 0.7%.

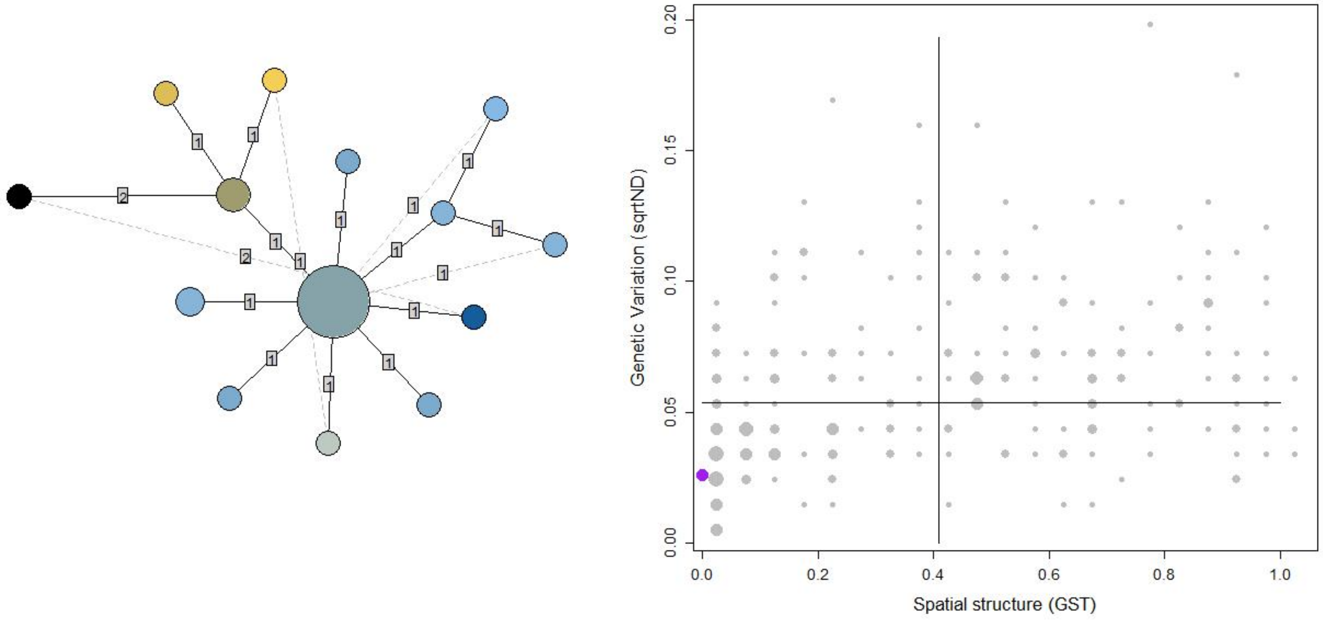

Figure 556: Haplotype network (left) of *Boloria eunomia* sequences > 599 bp with colours matching the PCoA colour space (above). The bubble plot for mt-DNA polymorphism (square root transformed nucleotide diversity) and spatial structure (GST) among all species in the atlas and values for *Boloria eunomia* (purple dot). The horizontal and vertical lines represent median values of nucleotide diversity and GST, respectively. Sequences > 599 bp= 91.

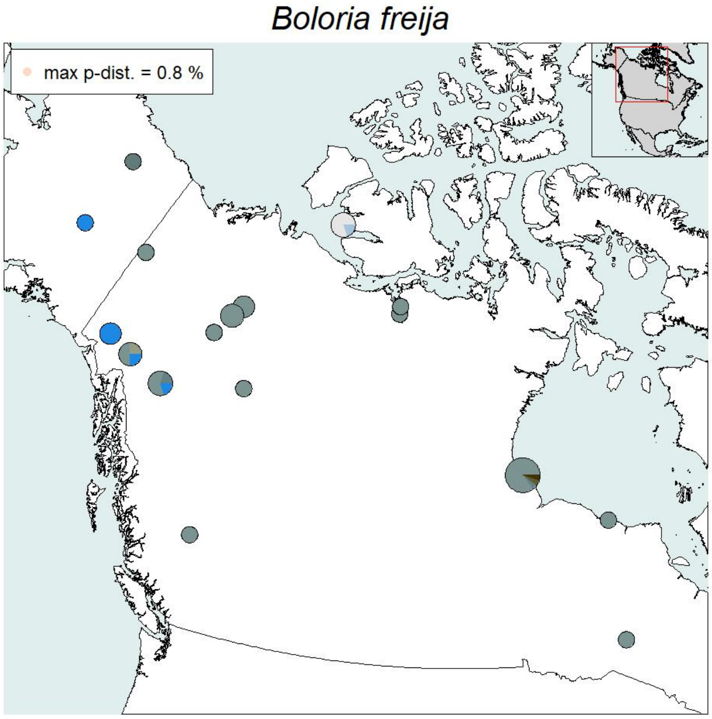

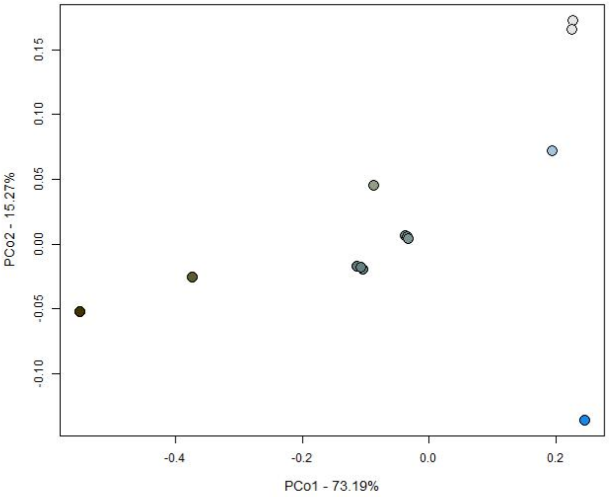

Figure 557 Map of *Boloria freija* showing the localities of the sequenced specimens (left). Nearby localities are grouped in pies. Colours match the bidimensional colour space of the PCoA projection (right) of max p-dists among sequences (dots). Sequences= 54; Hap obs.= 7; Hap asympt.= 9.9; Hap % obs.= 70.4%; GST= 0.551; DST= 0.0008; HD= 0.515; ND= 0.0014; max p-dist= 0.8%.

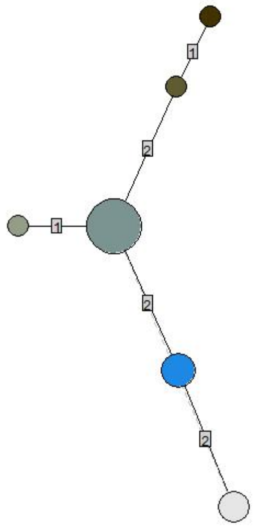

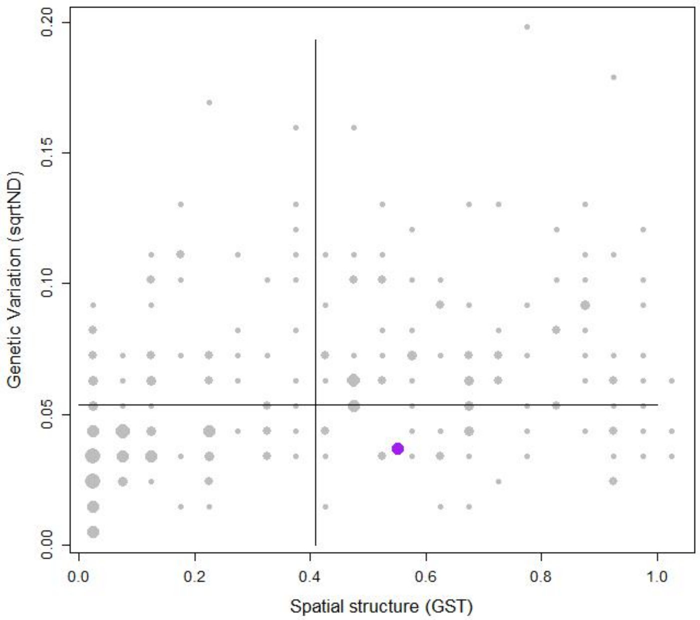

Figure 558: Haplotype network (left) of *Boloria freija* sequences > 599 bp with colours matching the PCoA colour space (above). The bubble plot for mt-DNA polymorphism (square root transformed nucleotide diversity) and spatial structure (GST) among all species in the atlas and values for *Boloria freija* (purple dot). The horizontal and vertical lines represent median values of nucleotide diversity and GST, respectively. Sequences > 599 bp= 53.

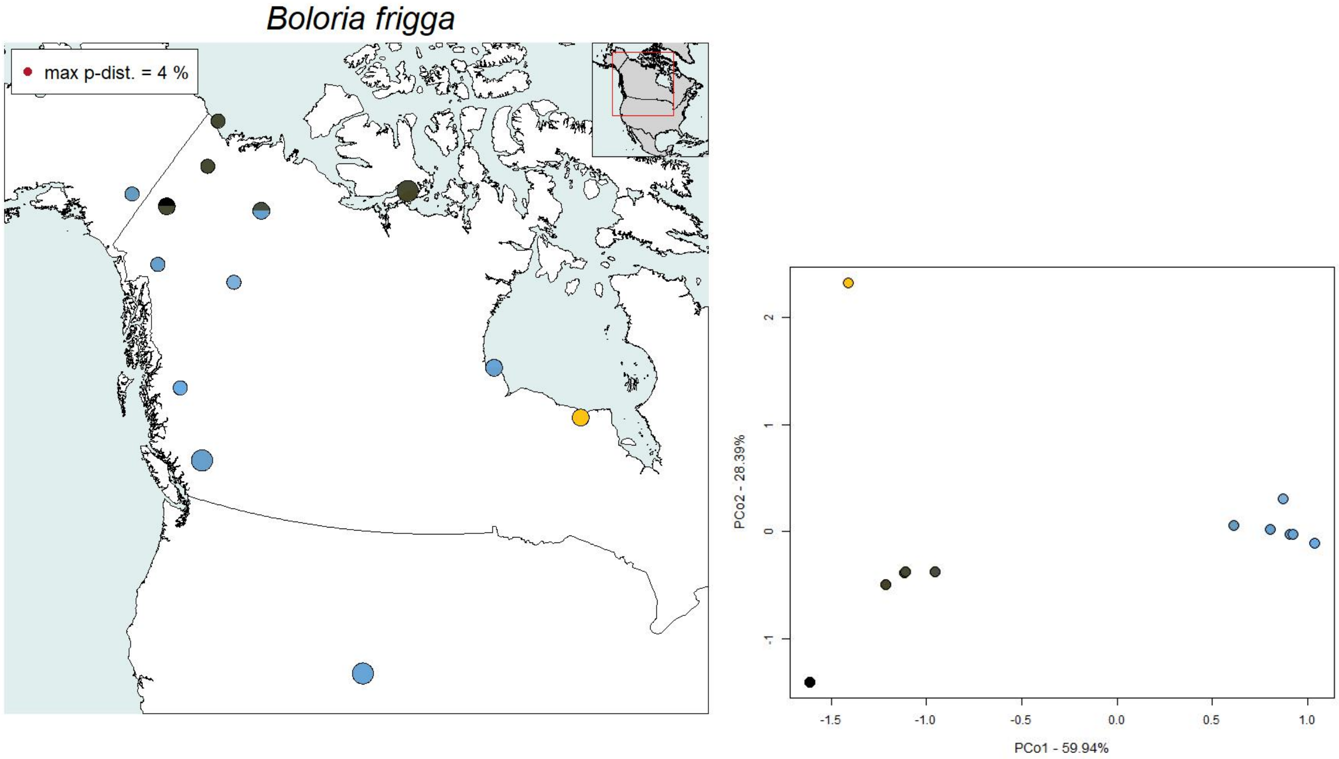

Figure 559 Map of *Boloria frigga* showing the localities of the sequenced specimens (left). Nearby localities are grouped in pies. Colours match the bidimensional colour space of the PCoA projection (right) of max p-dists among sequences (dots). Sequences= 29; Hap obs.= 10; Hap asympt.= 16; Hap % obs.= 62.4%; GST= 0.956; DST= 0.0117; HD= 0.845; ND= 0.0153; max p-dist= 4%.

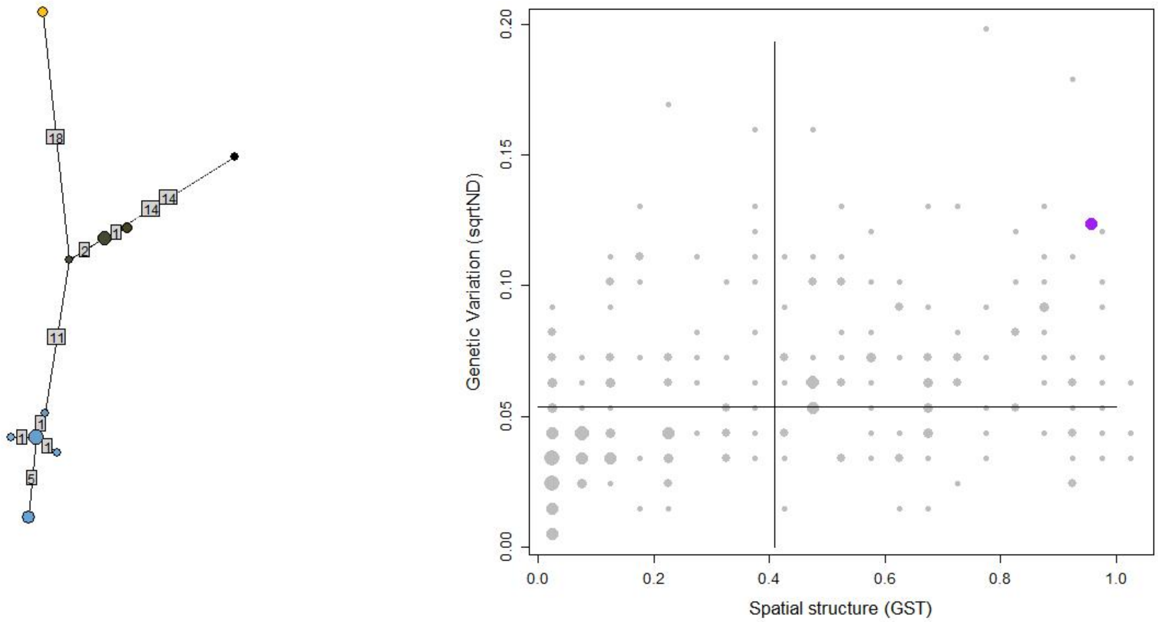

Figure 560: Haplotype network (left) of *Boloria frigga* sequences > 599 bp with colours matching the PCoA colour space (above). The bubble plot for mt-DNA polymorphism (square root transformed nucleotide diversity) and spatial structure (GST) among all species in the atlas and values for *Boloria frigga* (purple dot). The horizontal and vertical lines represent median values of nucleotide diversity and GST, respectively. Sequences > 599 bp= 29.

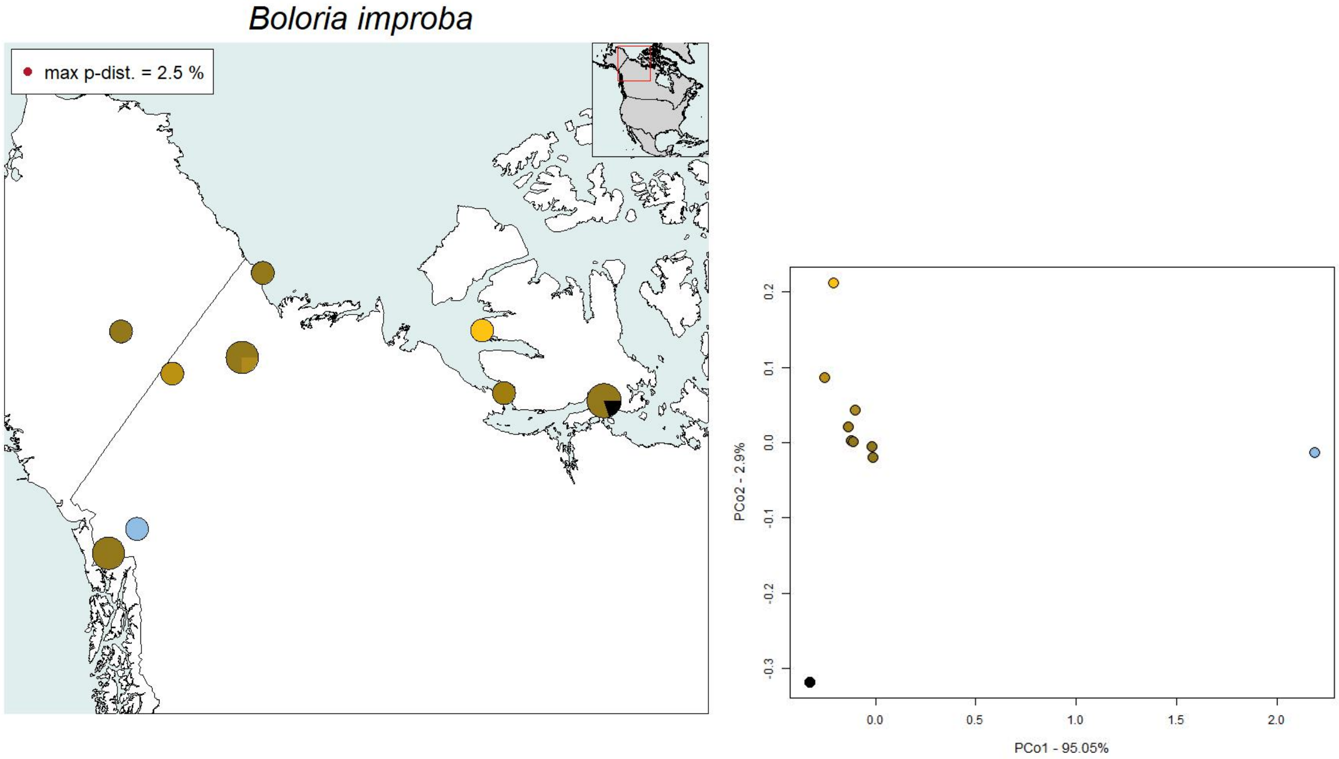

Figure 561 Map of *Boloria improba* showing the localities of the sequenced specimens (left). Nearby localities are grouped in pies. Colours match the bidimensional colour space of the PCoA projection (right) of max p-dists among sequences (dots). Sequences= 19; Hap obs.= 6; Hap asympt.= 15.5; Hap % obs.= 38.8%; GST= 0; DST= 0; HD= 0.468; ND= 0.0032; max p-dist= 2.5%.

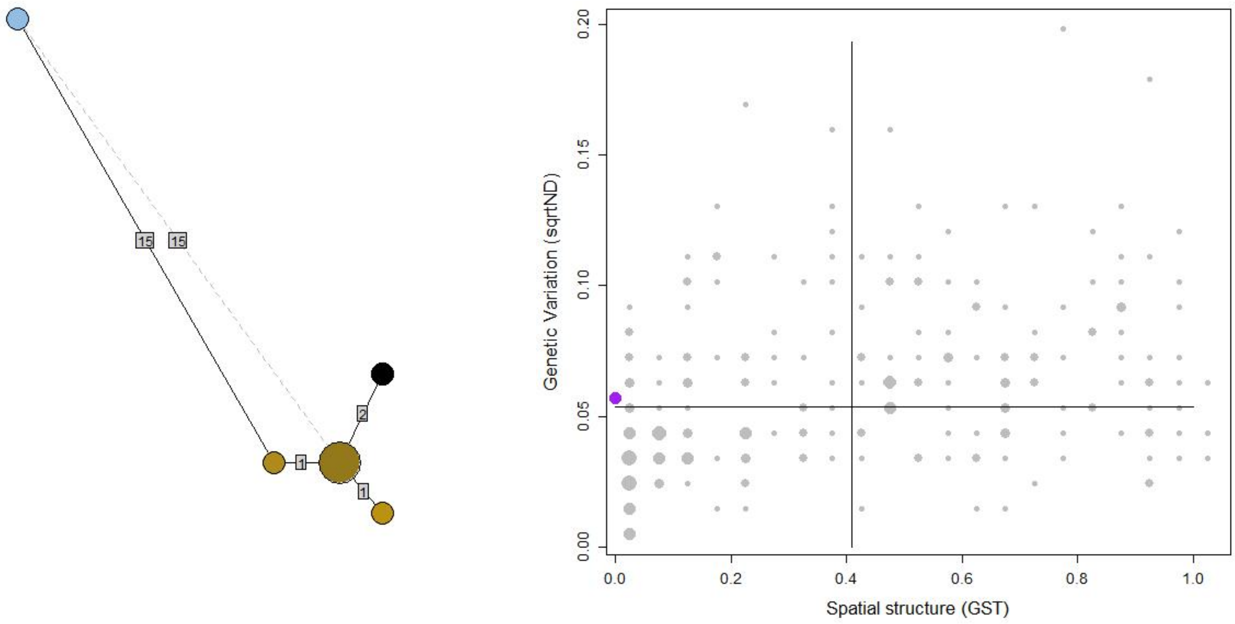

Figure 562: Haplotype network (left) of *Boloria improba* sequences > 599 bp with colours matching the PCoA colour space (above). The bubble plot for mt-DNA polymorphism (square root transformed nucleotide diversity) and spatial structure (GST) among all species in the atlas and values for *Boloria improba* (purple dot). The horizontal and vertical lines represent median values of nucleotide diversity and GST, respectively. Sequences > 599 bp= 17.

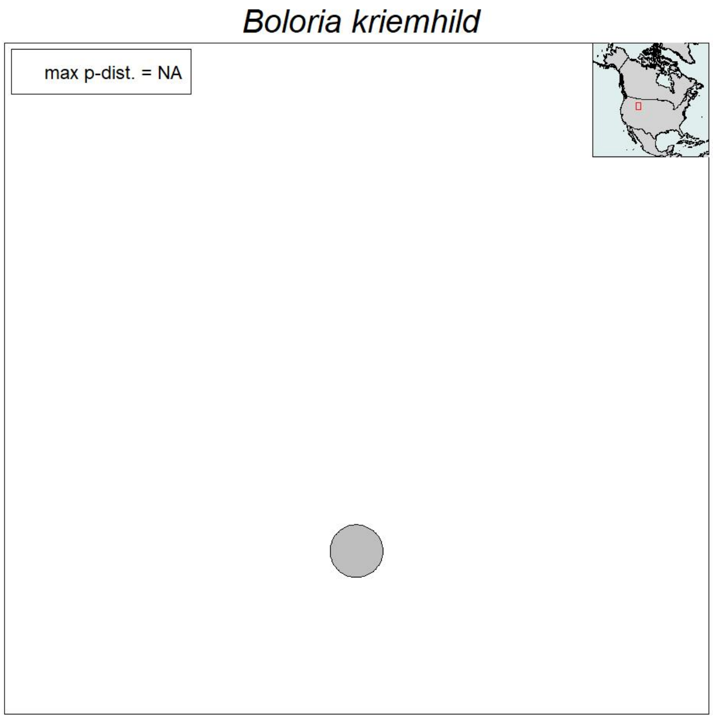

Figure 563: Map of *Boloria kriemhild* showing the localities of the sequenced specimens. Nearby localities are grouped in pies. Due to the presence of a single haplotype PCoA projection was not done and a single grey colour was plotted on the map. Sequences= 1; Hap obs.= NA; Hap asympt.= NA; Hap % obs.= NA; GST= NaN; DST= NaN; HD= NA; ND= NA; max p-dist= NA.

Haplotype network analysis and bubble plot of *Boloria kriemhild* were not possible. Sequences > 599 bp = 0.

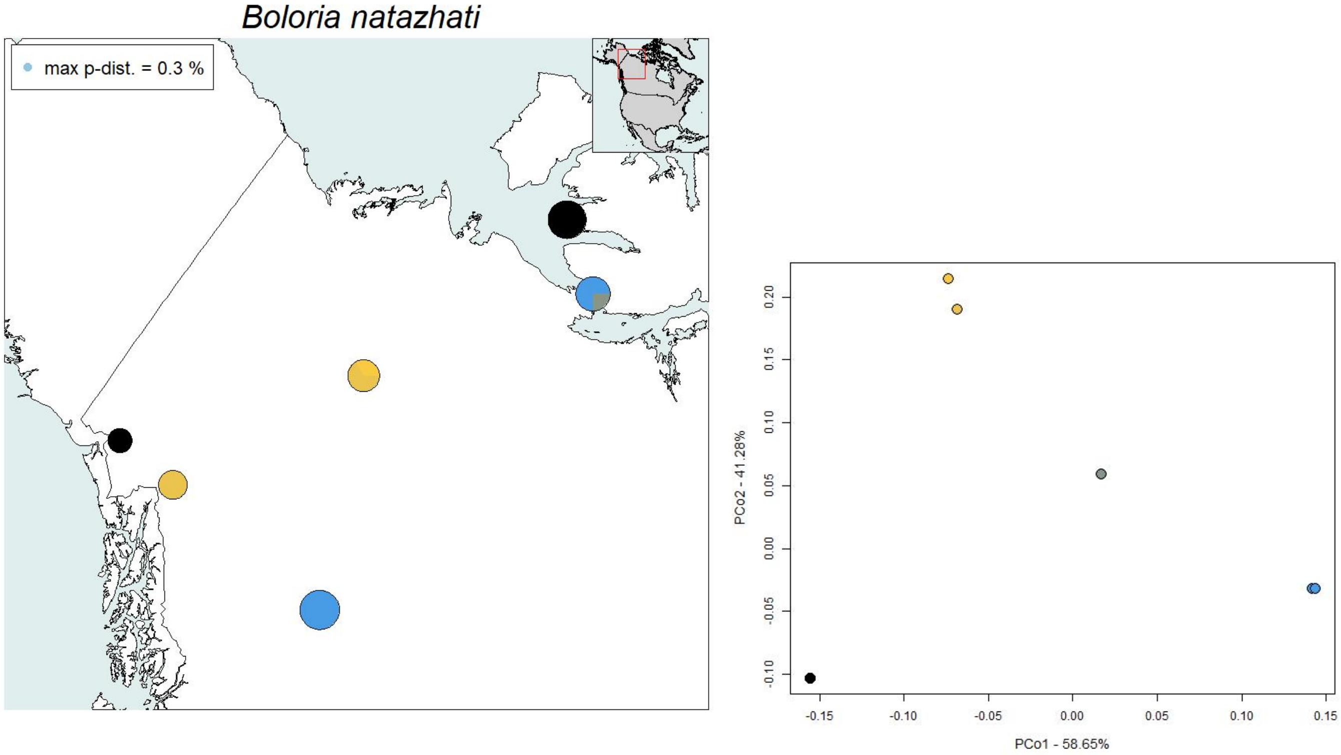

Figure 564 Map of *Boloria natazhati* showing the localities of the sequenced specimens (left). Nearby localities are grouped in pies. Colours match the bidimensional colour space of the PCoA projection (right) of max p-dists among sequences (dots). Sequences= 23; Hap obs.= 4; Hap asympt.= 4; Hap % obs.= 100%; GST= 0.918; DST= 0.0016; HD= 0.7; ND= 0.0019; max p-dist= 0.3%.

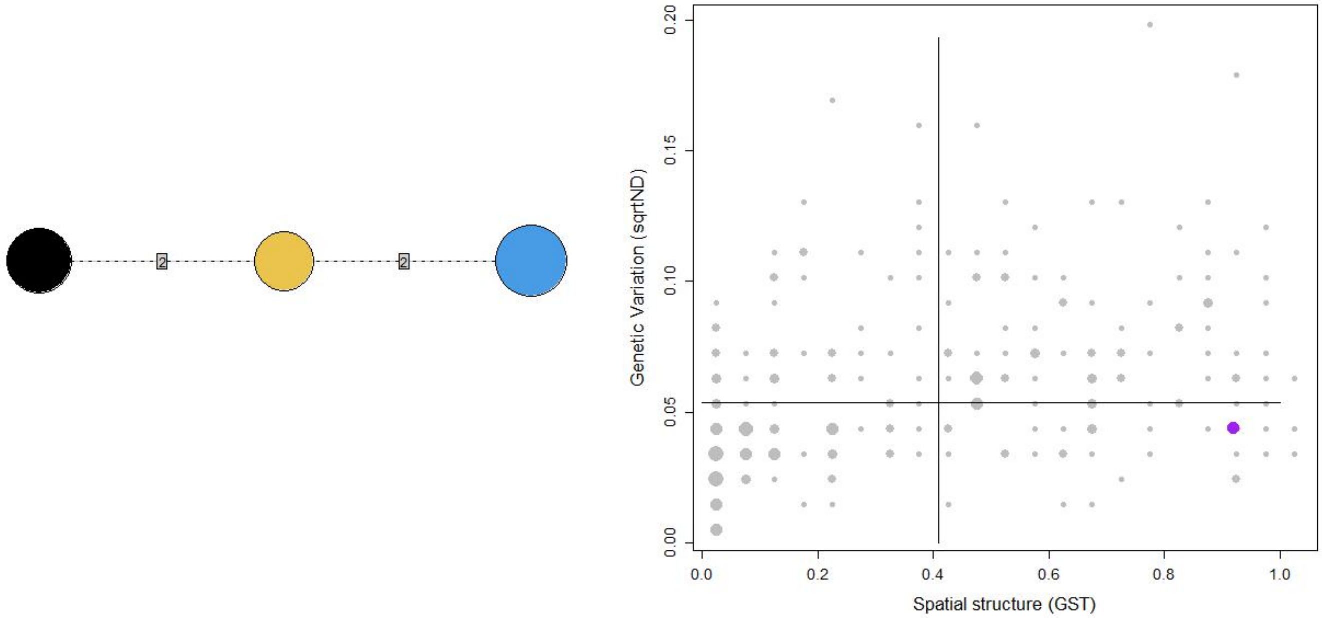

Figure 565: Haplotype network (left) of *Boloria natazhati* sequences > 599 bp with colours matching the PCoA colour space (above). The bubble plot for mt-DNA polymorphism (square root transformed nucleotide diversity) and spatial structure (GST) among all species in the atlas and values for *Boloria natazhati* (purple dot). The horizontal and vertical lines represent median values of nucleotide diversity and GST, respectively. Sequences > 599 bp= 22.

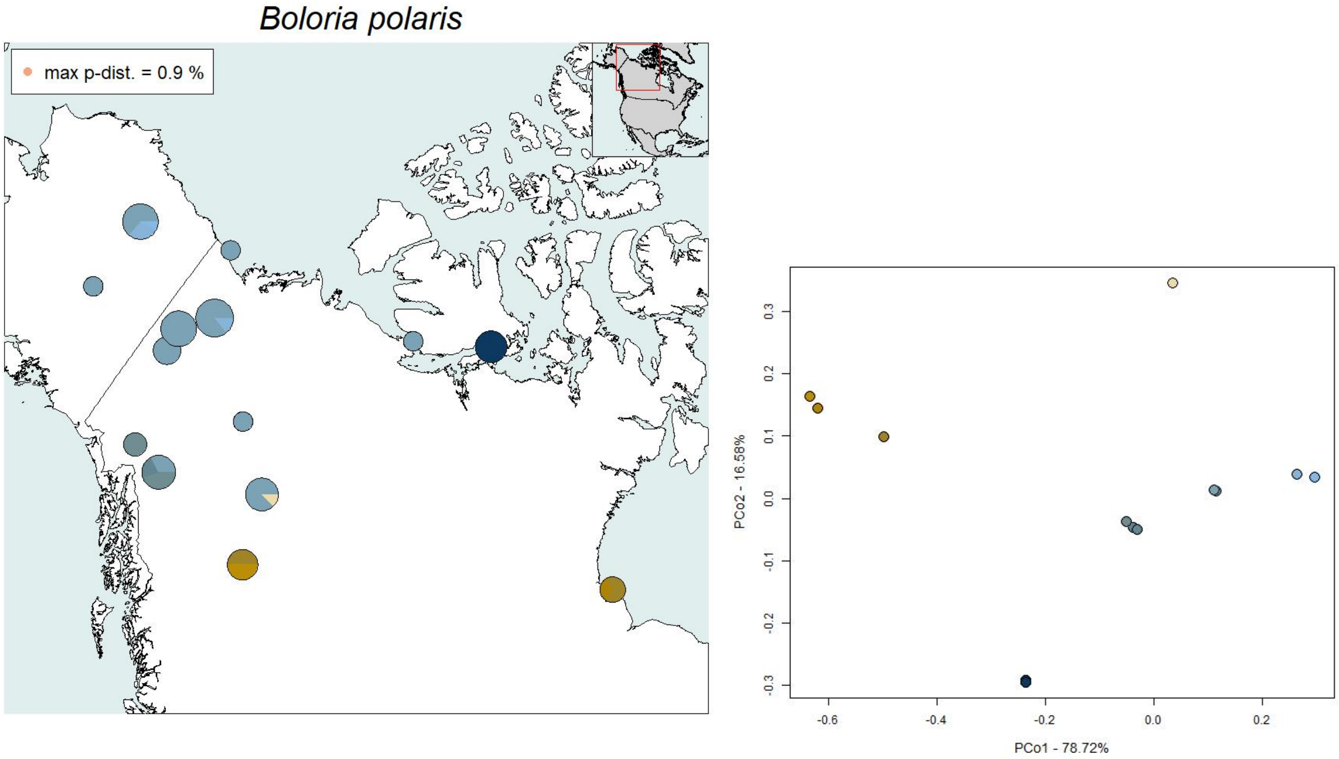

Figure 566 Map of *Boloria polaris* showing the localities of the sequenced specimens (left). Nearby localities are grouped in pies. Colours match the bidimensional colour space of the PCoA projection (right) of max p-dists among sequences (dots). Sequences= 79; Hap obs.= 9; Hap asympt.= 10; Hap % obs.= 90.1%; GST= 0.847; DST= 0.0022; HD= 0.612; ND= 0.0025; max p-dist= 0.9%.

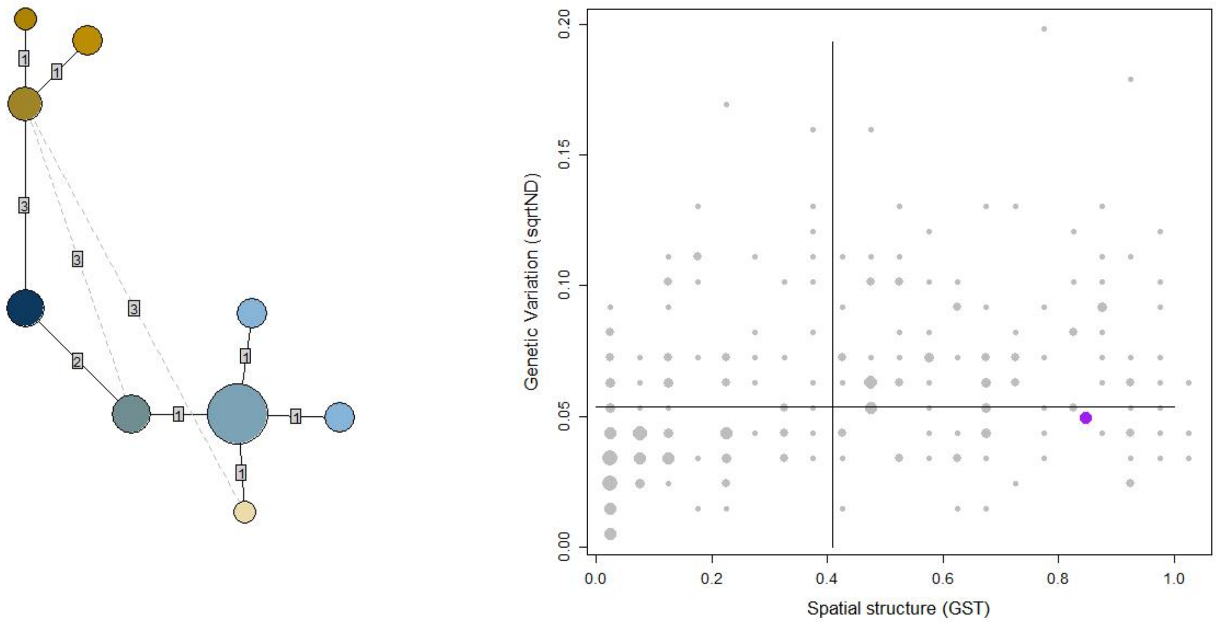

Figure 567: Haplotype network (left) of *Boloria polaris* sequences > 599 bp with colours matching the PCoA colour space (above). The bubble plot for mt-DNA polymorphism (square root transformed nucleotide diversity) and spatial structure (GST) among all species in the atlas and values for *Boloria polaris* (purple dot). The horizontal and vertical lines represent median values of nucleotide diversity and GST, respectively. Sequences > 599 bp= 79.

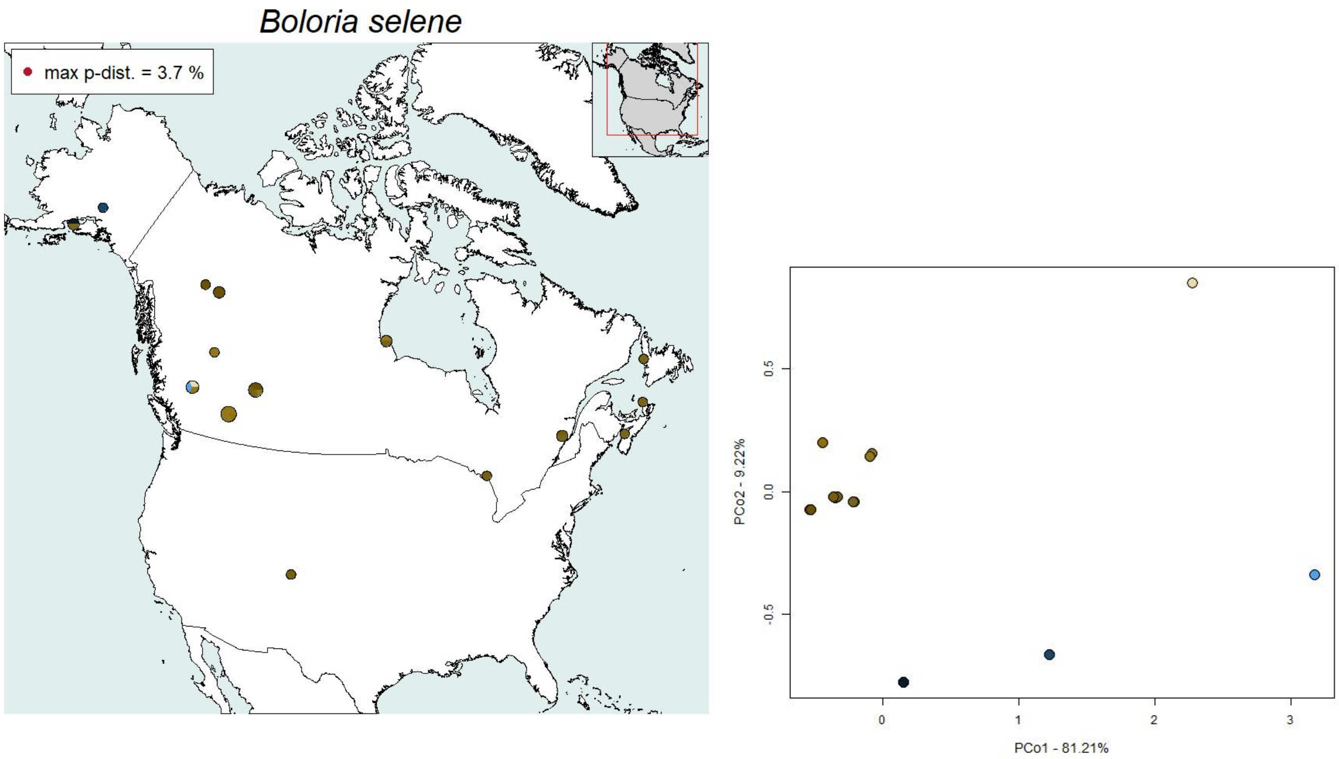

Figure 568 Map of *Boloria selene* showing the localities of the sequenced specimens (left). Nearby localities are grouped in pies. Colours match the bidimensional colour space of the PCoA projection (right) of max p-dists among sequences (dots). Sequences= 31; Hap obs.= 13; Hap asympt.= 44; Hap % obs.= 29.6%; GST= 0.567; DST= 0.0042; HD= 0.888; ND= 0.0071; max p-dist= 3.7%.

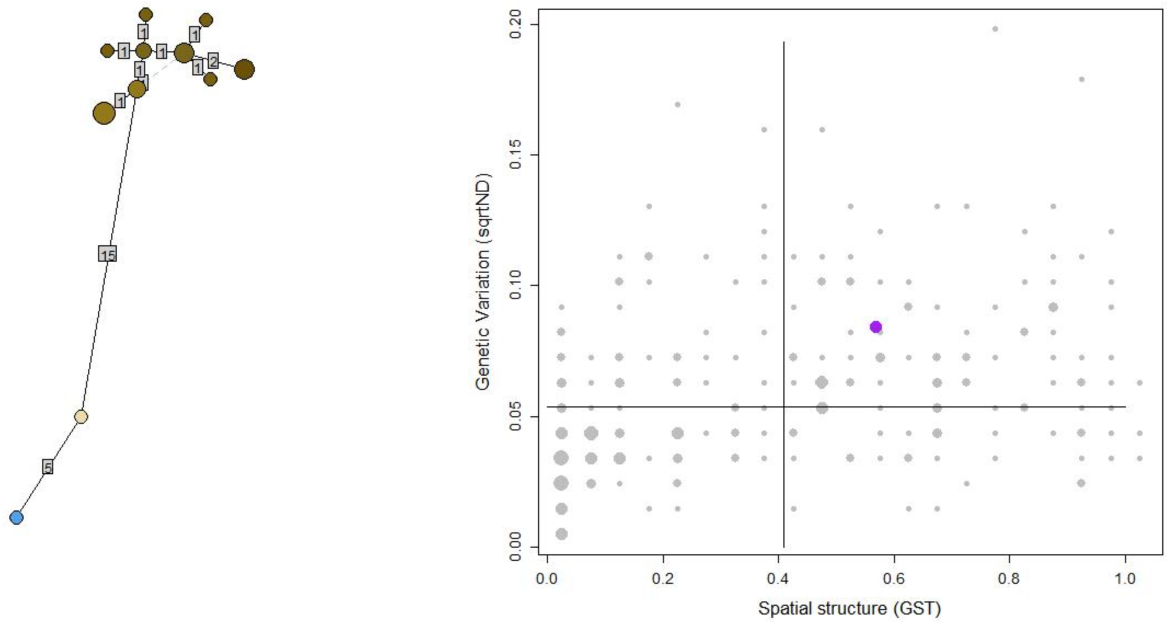

Figure 569: Haplotype network (left) of *Boloria selene* sequences > 599 bp with colours matching the PCoA colour space (above). The bubble plot for mt-DNA polymorphism (square root transformed nucleotide diversity) and spatial structure (GST) among all species in the atlas and values for *Boloria selene* (purple dot). The horizontal and vertical lines represent median values of nucleotide diversity and GST, respectively. Sequences > 599 bp= 28.

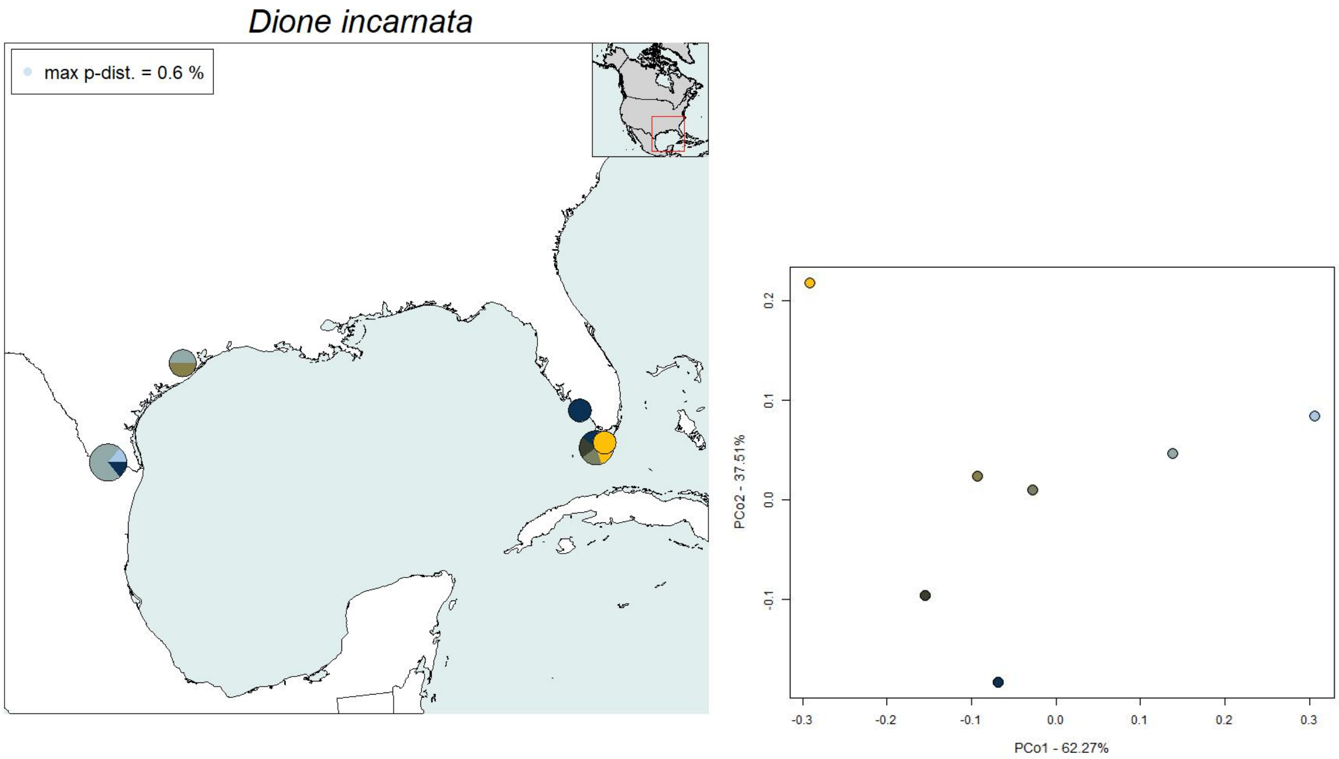

Figure 570 Map of *Dione incarnata* showing the localities of the sequenced specimens (left). Nearby localities are grouped in pies. Colours match the bidimensional colour space of the PCoA projection (right) of max p-dists among sequences (dots). Sequences= 16; Hap obs.= 6; Hap asympt.= 10.2; Hap % obs.= 58.7%; GST= 0.341; DST= 0.0008; HD= 0.783; ND= 0.0023; max p-dist= 0.6%.

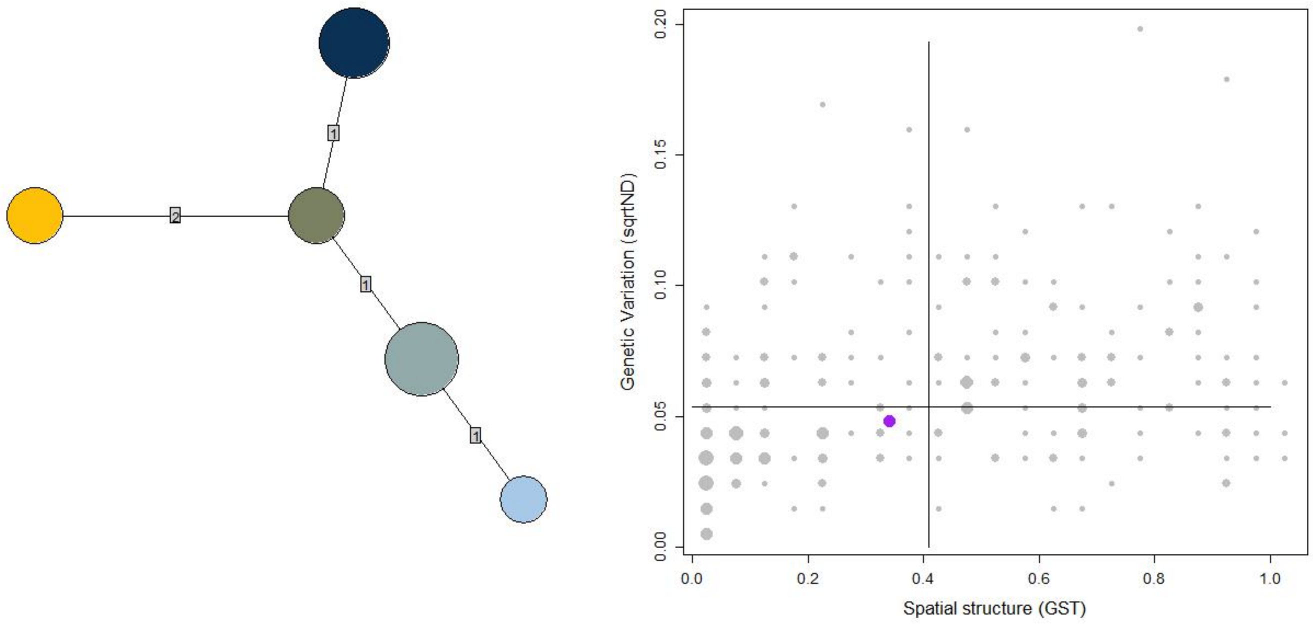

Figure 571: Haplotype network (left) of *Dione incarnata* sequences > 599 bp with colours matching the PCoA colour space (above). The bubble plot for mt-DNA polymorphism (square root transformed nucleotide diversity) and spatial structure (GST) among all species in the atlas and values for *Dione incarnata* (purple dot). The horizontal and vertical lines represent median values of nucleotide diversity and GST, respectively. Sequences > 599 bp= 16.

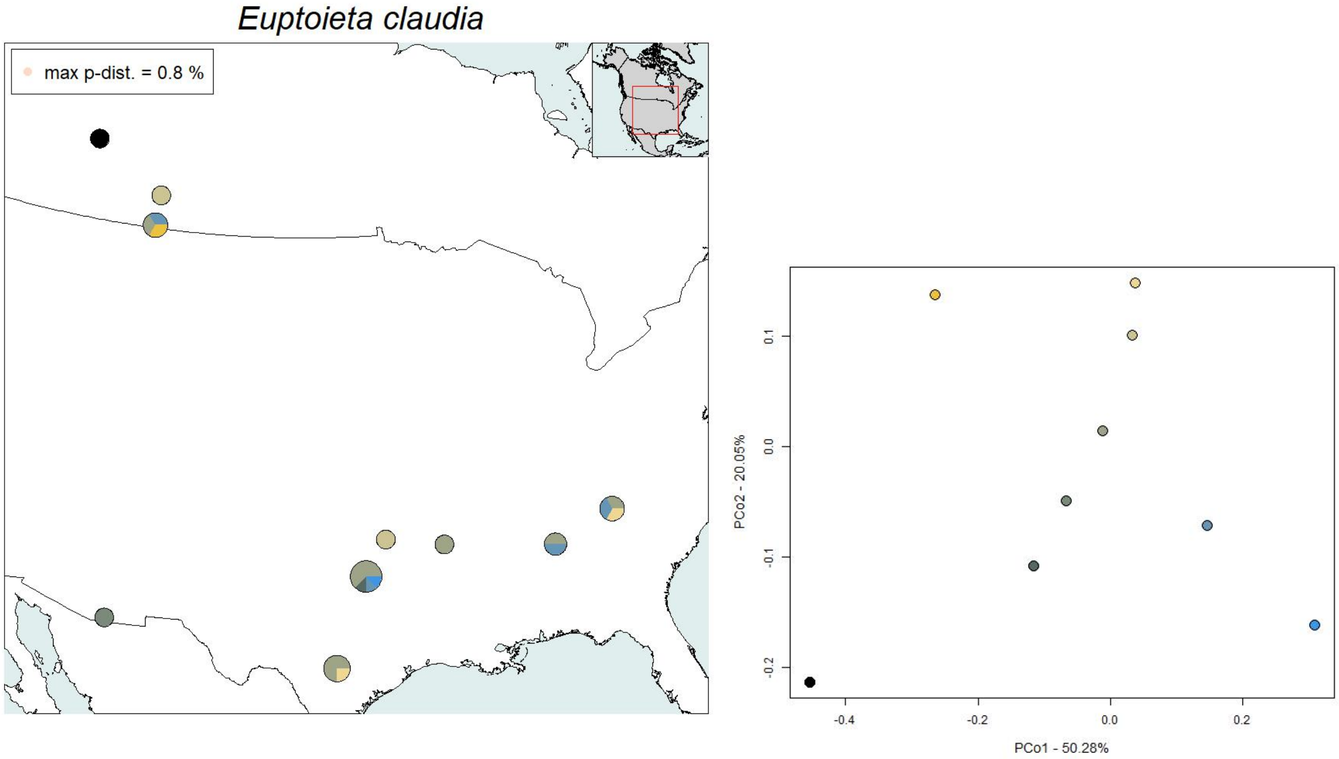

Figure 572 Map of *Euptoieta claudia* showing the localities of the sequenced specimens (left). Nearby localities are grouped in pies. Colours match the bidimensional colour space of the PCoA projection (right) of max p-dists among sequences (dots). Sequences= 25; Hap obs.= 9; Hap asympt.= 26.3; Hap % obs.= 34.2%; GST= 0.051; DST= 0.0001; HD= 0.717; ND= 0.0019; max p-dist= 0.8%.

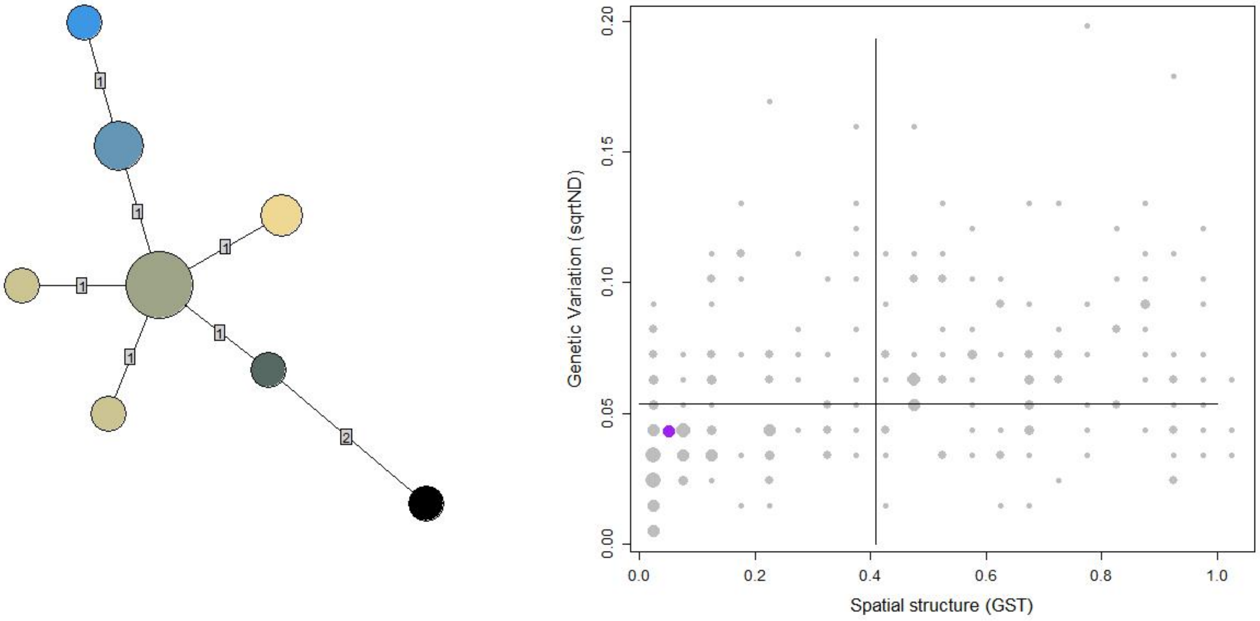

Figure 573: Haplotype network (left) of *Euptoieta claudia* sequences > 599 bp with colours matching the PCoA colour space (above). The bubble plot for mt-DNA polymorphism (square root transformed nucleotide diversity) and spatial structure (GST) among all species in the atlas and values for *Euptoieta claudia* (purple dot). The horizontal and vertical lines represent median values of nucleotide diversity and GST, respectively. Sequences > 599 bp= 24.

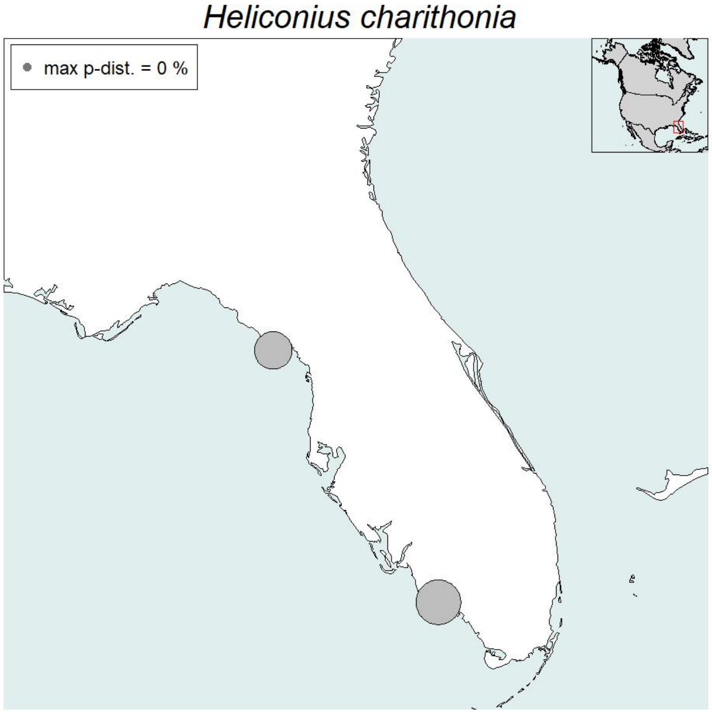

Figure 574: Map of *Heliconius charithonia* showing the localities of the sequenced specimens. Nearby localities are grouped in pies. Due to the presence of a single haplotype PCoA projection was not done and a single grey colour was plotted on the map. Sequences= 3; Hap obs.= 1; Hap asympt.= NA; Hap % obs.= NA%; GST= NaN; DST= NaN; HD= NA; ND= NA; max p-dist= 0%.

Haplotype network analysis and bubble plot of *Heliconius charithonia* were not possible. Sequences > 599 bp = 3.

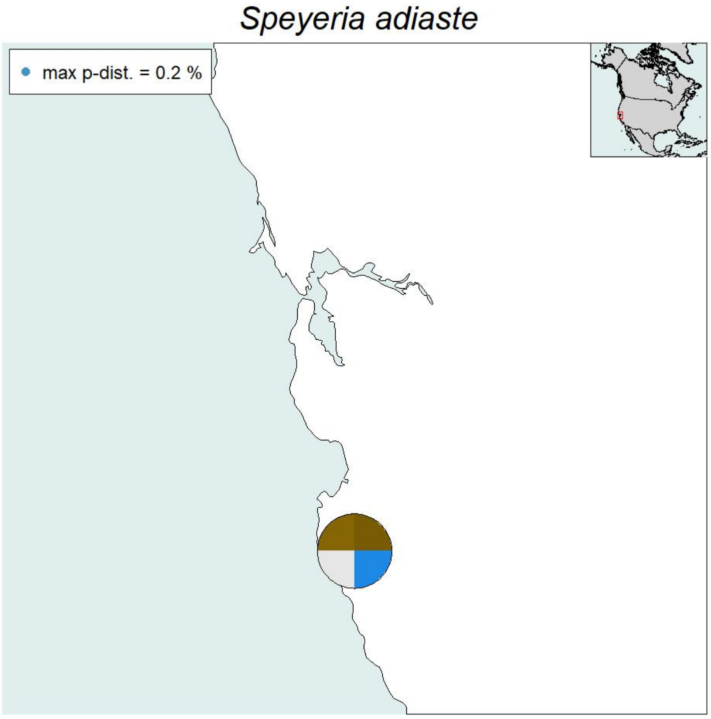

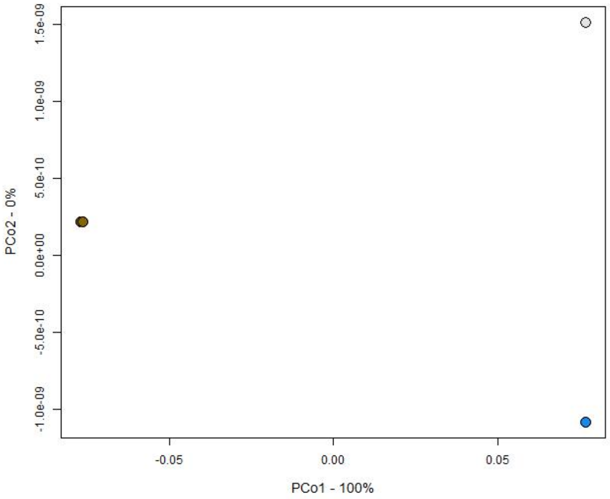

Figure 575 Map of *Speyeria adiaste* showing the localities of the sequenced specimens (left). Nearby localities are grouped in pies. Colours match the bidimensional colour space of the PCoA projection (right) of max p-dists among sequences (dots). Sequences= 4; Hap obs.= 2; Hap asympt.= NA; Hap % obs.= NA%; GST= NaN; DST= NaN; HD= NA; ND= NA; max p-dist= 0.2%.

Haplotype network analysis and bubble plot of *Speyeria adiaste* were not possible. Sequences > 599 bp = 4.

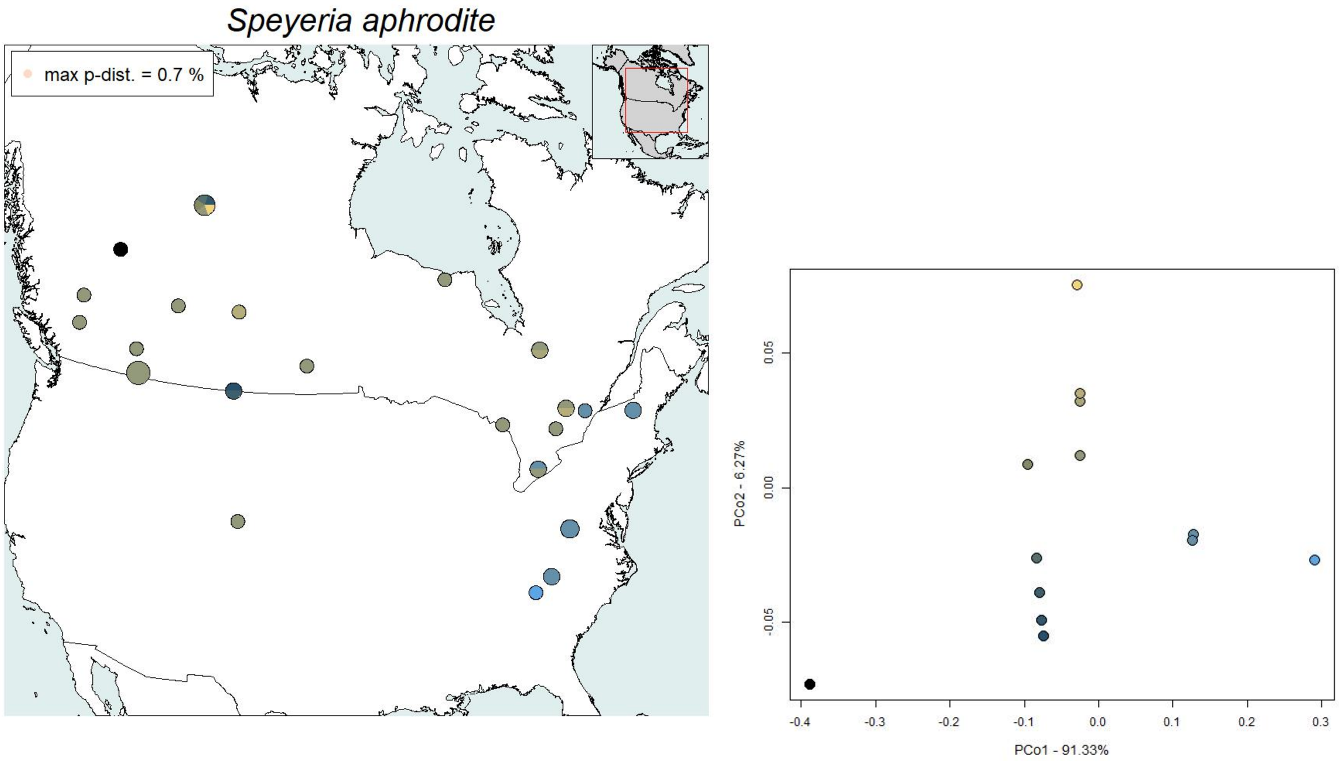

Figure 576 Map of *Speyeria aphrodite* showing the localities of the sequenced specimens (left). Nearby localities are grouped in pies. Colours match the bidimensional colour space of the PCoA projection (right) of max p-dists among sequences (dots). Sequences= 41; Hap obs.= 4; Hap asympt.= 5; Hap % obs.= 80.4%; GST= 0.941; DST= 0.0006; HD= 0.426; ND= 0.0008; max p-dist= 0.7%.

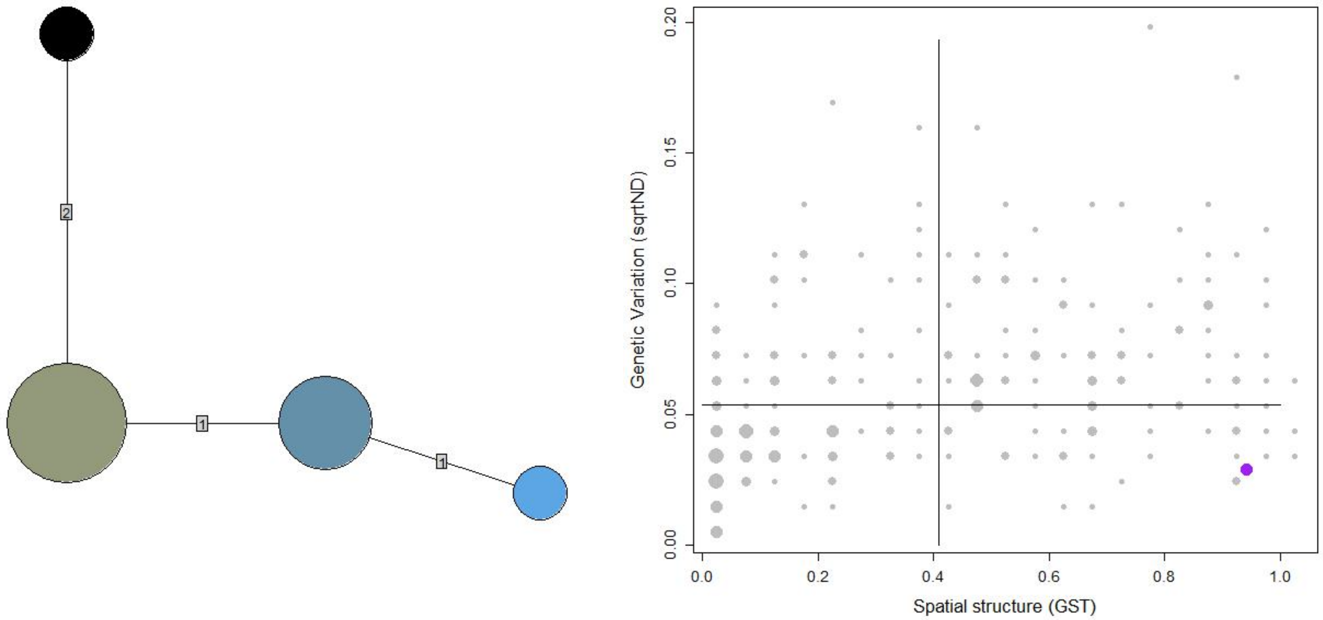

Figure 577: Haplotype network (left) of *Speyeria aphrodite* sequences > 599 bp with colours matching the PCoA colour space (above). The bubble plot for mt-DNA polymorphism (square root transformed nucleotide diversity) and spatial structure (GST) among all species in the atlas and values for *Speyeria aphrodite* (purple dot). The horizontal and vertical lines represent median values of nucleotide diversity and GST, respectively. Sequences > 599 bp= 35.

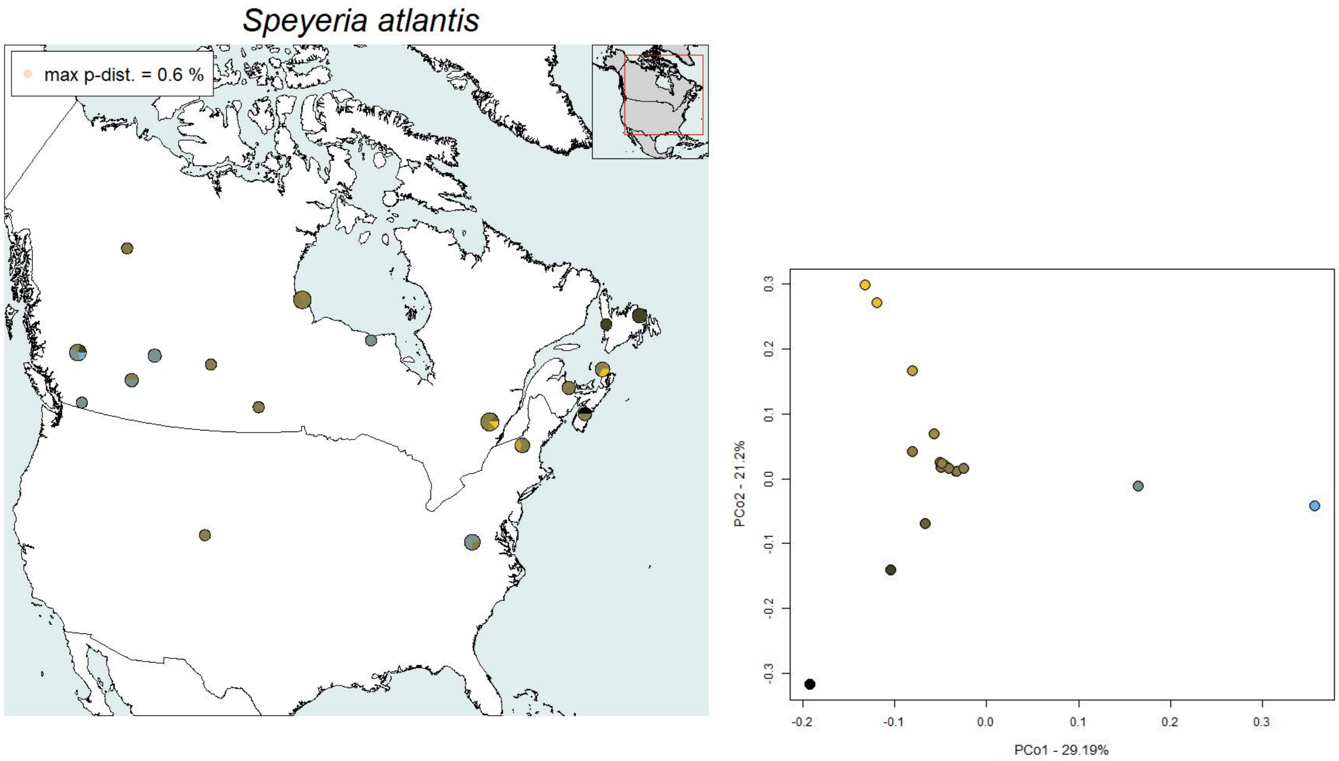

Figure 578 Map of *Speyeria atlantis* showing the localities of the sequenced specimens (left). Nearby localities are grouped in pies. Colours match the bidimensional colour space of the PCoA projection (right) of max p-dists among sequences (dots). Sequences= 46; Hap obs.= 17; Hap asympt.= 46.6; Hap % obs.= 36.5%; GST= 0.246; DST= 0.0006; HD= 0.867; ND= 0.0023; max p-dist= 0.6%.

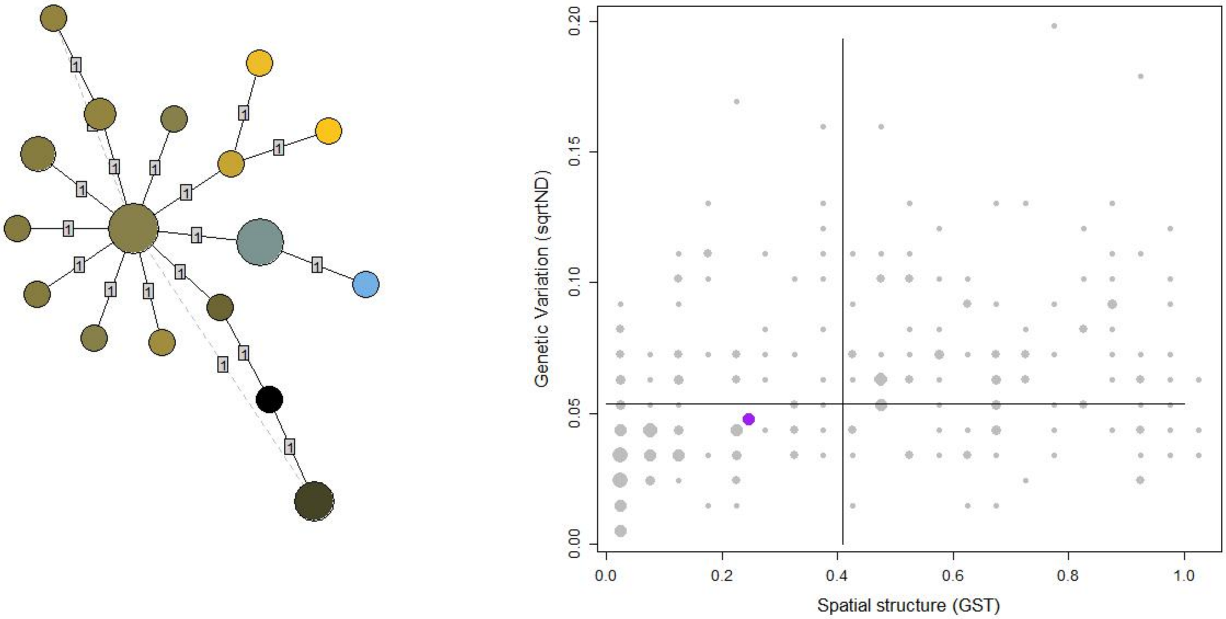

Figure 579: Haplotype network (left) of *Speyeria atlantis* sequences > 599 bp with colours matching the PCoA colour space (above). The bubble plot for mt-DNA polymorphism (square root transformed nucleotide diversity) and spatial structure (GST) among all species in the atlas and values for *Speyeria atlantis* (purple dot). The horizontal and vertical lines represent median values of nucleotide diversity and GST, respectively. Sequences > 599 bp= 45.

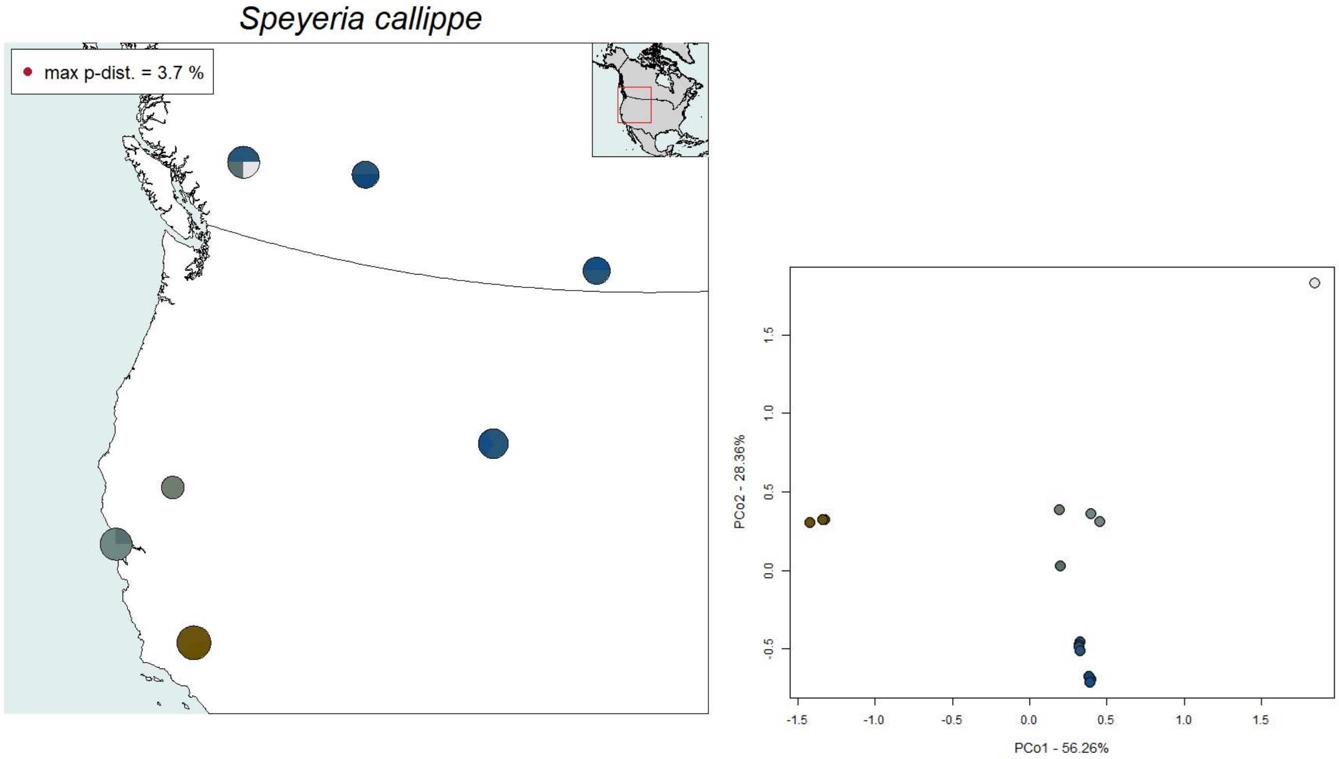

Figure 580 Map of *Speyeria callippe* showing the localities of the sequenced specimens (left). Nearby localities are grouped in pies. Colours match the bidimensional colour space of the PCoA projection (right) of max p-dists among sequences (dots). Sequences= 21; Hap obs.= 10; Hap asympt.= 14; Hap % obs.= 71.6%; GST= 0.431; DST= 0.0063; HD= 0.886; ND= 0.0129; max p-dist= 3.7%.

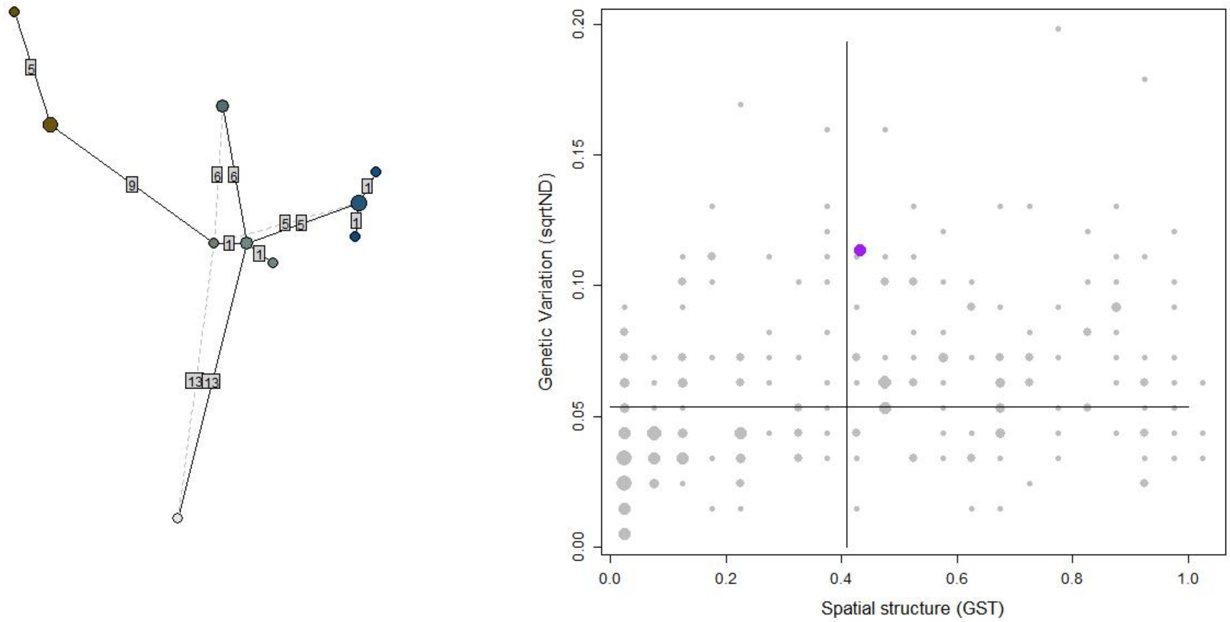

Figure 581: Haplotype network (left) of *Speyeria callippe* sequences > 599 bp with colours matching the PCoA colour space (above). The bubble plot for mt-DNA polymorphism (square root transformed nucleotide diversity) and spatial structure (GST) among all species in the atlas and values for *Speyeria callippe* (purple dot). The horizontal and vertical lines represent median values of nucleotide diversity and GST, respectively. Sequences > 599 bp= 19.

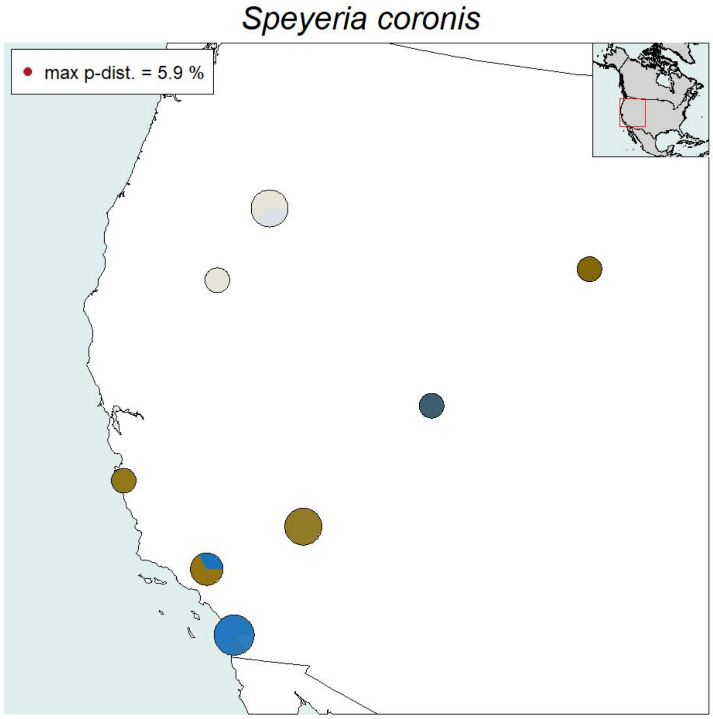

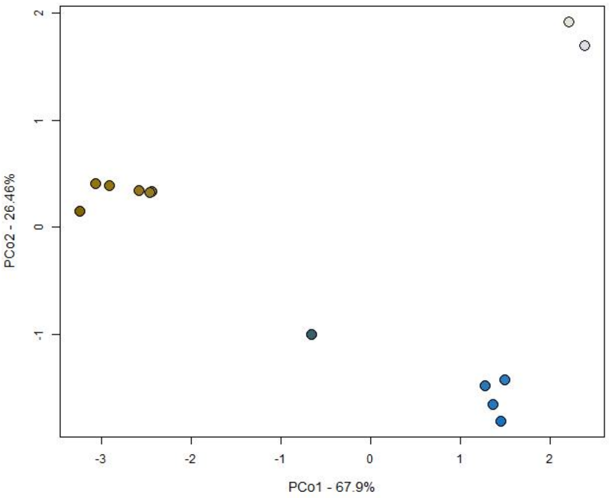

Figure 582 Map of *Speyeria coronis* showing the localities of the sequenced specimens (left). Nearby localities are grouped in pies. Colours match the bidimensional colour space of the PCoA projection (right) of max p-dists among sequences (dots). Sequences= 24; Hap obs.= 12; Hap asympt.= 42.7; Hap % obs.= 28.1%; GST= 0.936; DST= 0.0278; HD= 0.902; ND= 0.0321; max p-dist= 5.9%.

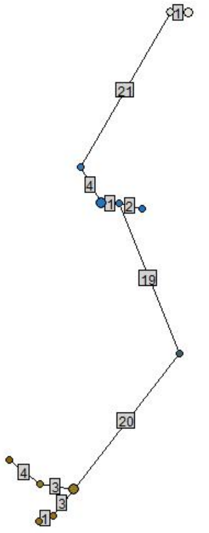

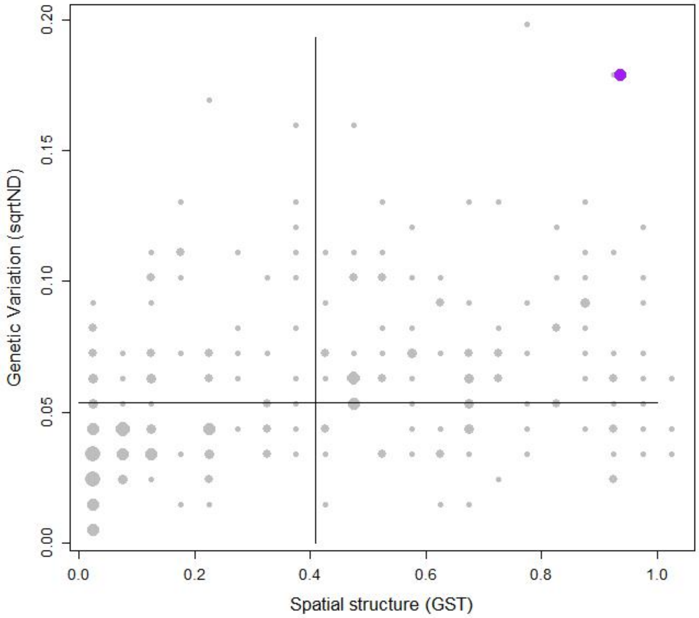

Figure 583: Haplotype network (left) of *Speyeria coronis* sequences > 599 bp with colours matching the PCoA colour space (above). The bubble plot for mt-DNA polymorphism (square root transformed nucleotide diversity) and spatial structure (GST) among all species in the atlas and values for *Speyeria coronis* (purple dot). The horizontal and vertical lines represent median values of nucleotide diversity and GST, respectively. Sequences > 599 bp= 24.

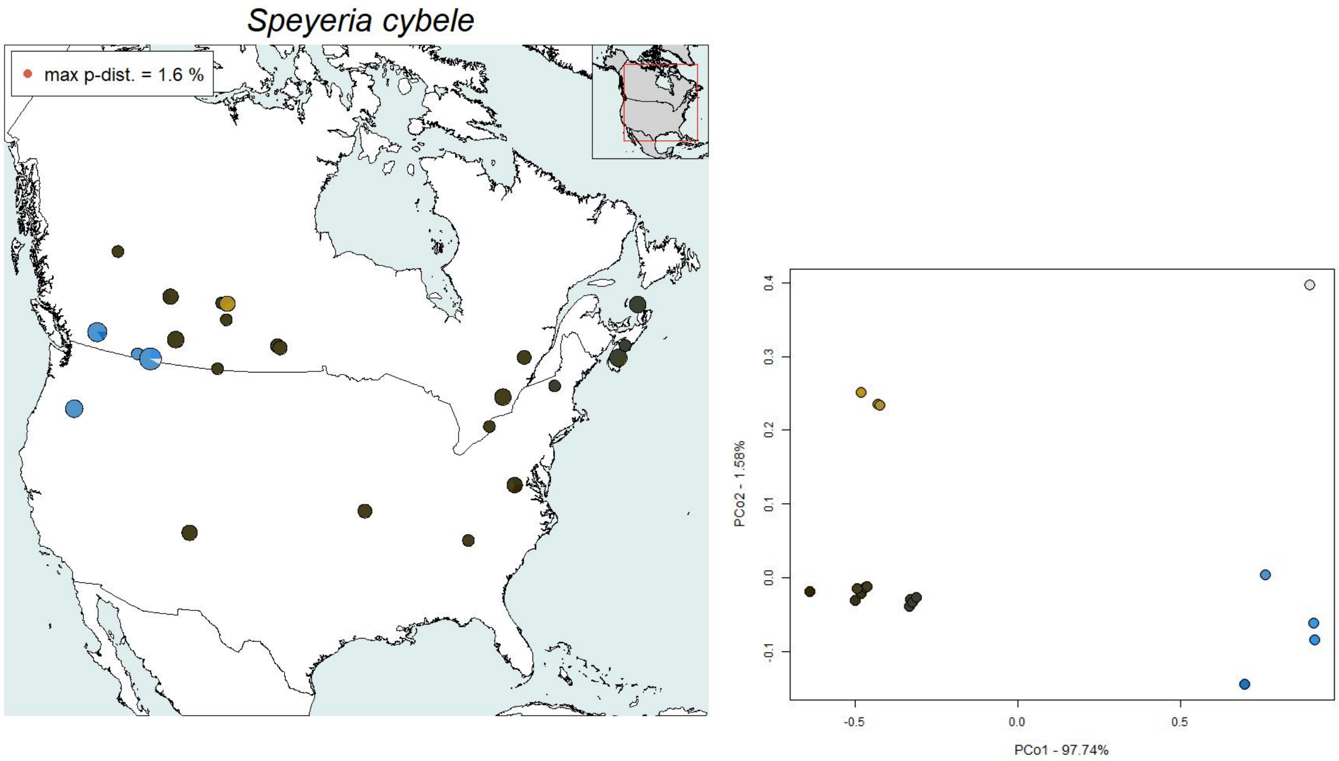

Figure 584 Map of *Speyeria cybele* showing the localities of the sequenced specimens (left). Nearby localities are grouped in pies. Colours match the bidimensional colour space of the PCoA projection (right) of max p-dists among sequences (dots). Sequences= 70; Hap obs.= 8; Hap asympt.= 13.9; Hap % obs.= 57.5%; GST= 0.857; DST= 0.0055; HD= 0.677; ND= 0.006; max p-dist= 1.6%.

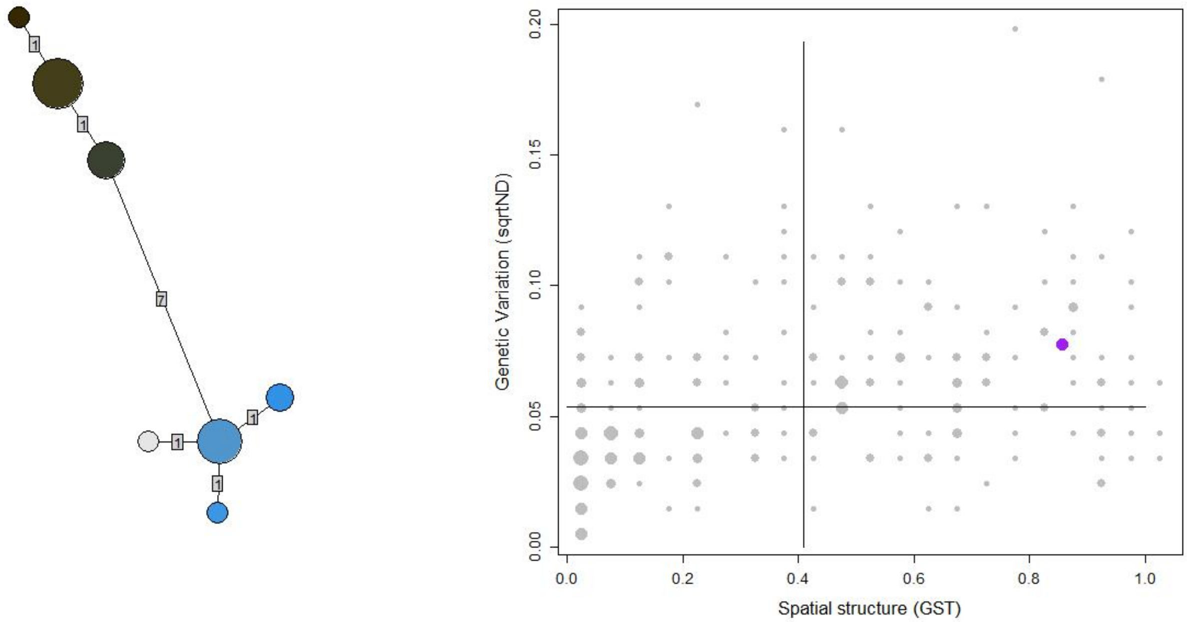

Figure 585: Haplotype network (left) of *Speyeria cybele* sequences > 599 bp with colours matching the PCoA colour space (above). The bubble plot for mt-DNA polymorphism (square root transformed nucleotide diversity) and spatial structure (GST) among all species in the atlas and values for *Speyeria cybele* (purple dot). The horizontal and vertical lines represent median values of nucleotide diversity and GST, respectively. Sequences > 599 bp= 67.

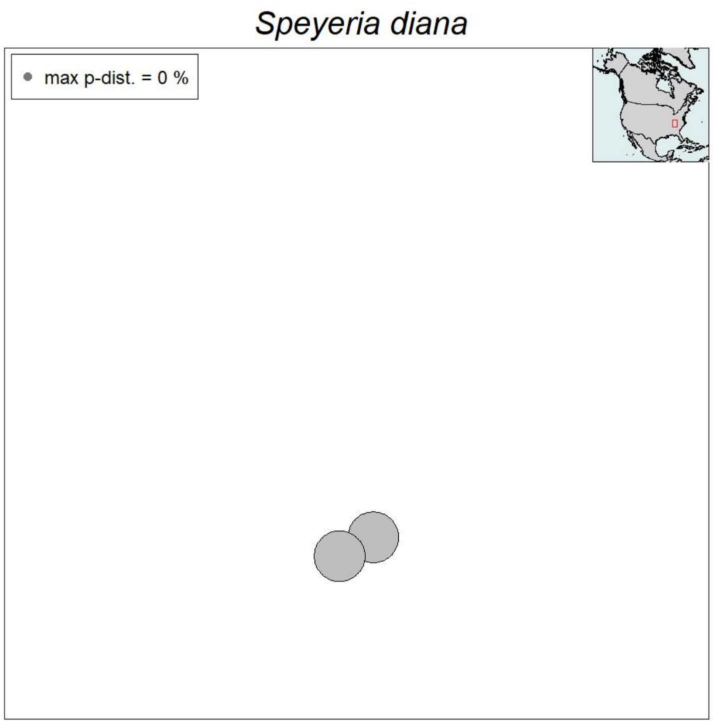

Figure 586: Map of *Speyeria diana* showing the localities of the sequenced specimens. Nearby localities are grouped in pies. Due to the presence of a single haplotype PCoA projection was not done and a single grey colour was plotted on the map. Sequences= 2; Hap obs.= 1; Hap asympt.= NA; Hap % obs.= NA%; GST= NaN; DST= NaN; HD= NA; ND= NA; max p-dist= 0%.

Haplotype network analysis and bubble plot of *Speyeria diana* were not possible. Sequences > 599 bp = 2.

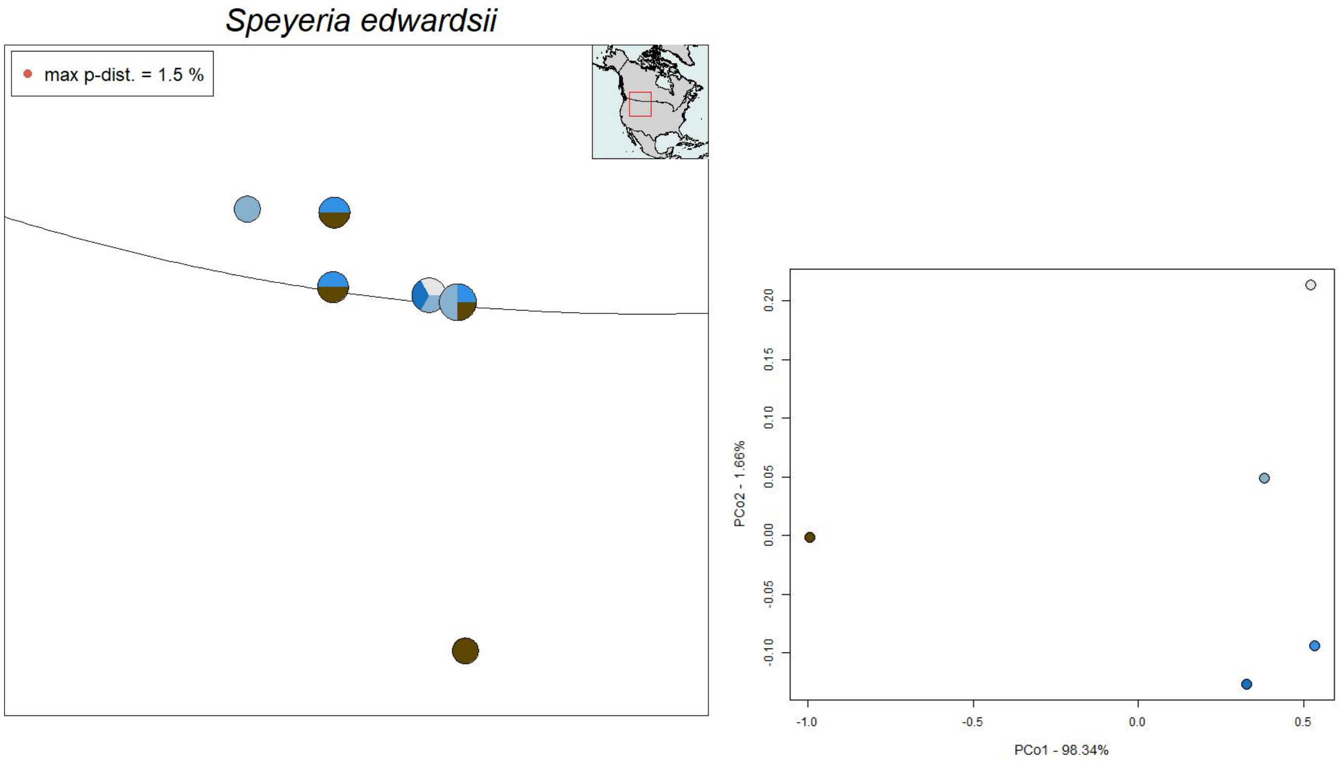

Figure 587 Map of *Speyeria edwardsii* showing the localities of the sequenced specimens (left). Nearby localities are grouped in pies. Colours match the bidimensional colour space of the PCoA projection (right) of max p-dists among sequences (dots). Sequences= 13; Hap obs.= 4; Hap asympt.= 4; Hap % obs.= 100%; GST= 0.042; DST= 0.0003; HD= 0.769; ND= 0.0072; max p-dist= 1.5%.

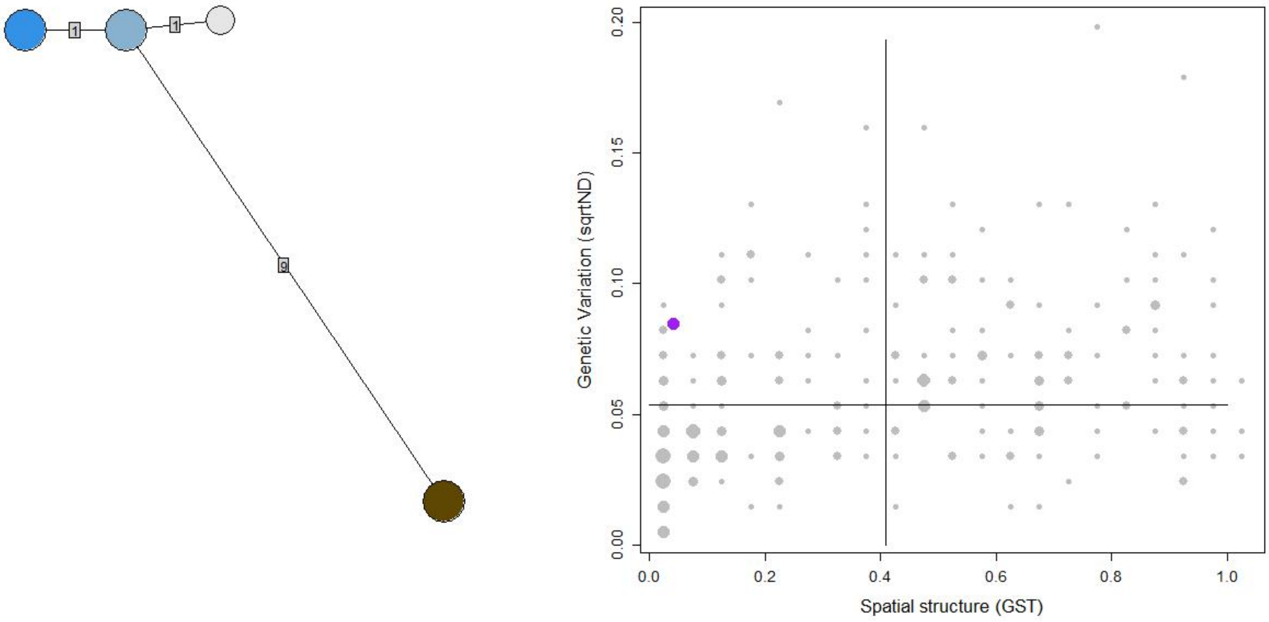

Figure 588: Haplotype network (left) of *Speyeria edwardsii* sequences > 599 bp with colours matching the PCoA colour space (above). The bubble plot for mt-DNA polymorphism (square root transformed nucleotide diversity) and spatial structure (GST) among all species in the atlas and values for *Speyeria edwardsii* (purple dot). The horizontal and vertical lines represent median values of nucleotide diversity and GST, respectively. Sequences > 599 bp= 13.

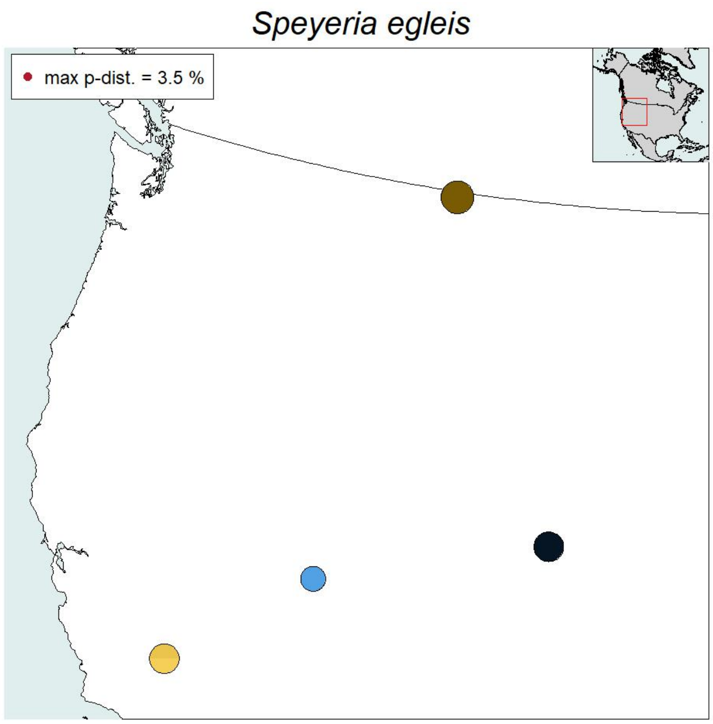

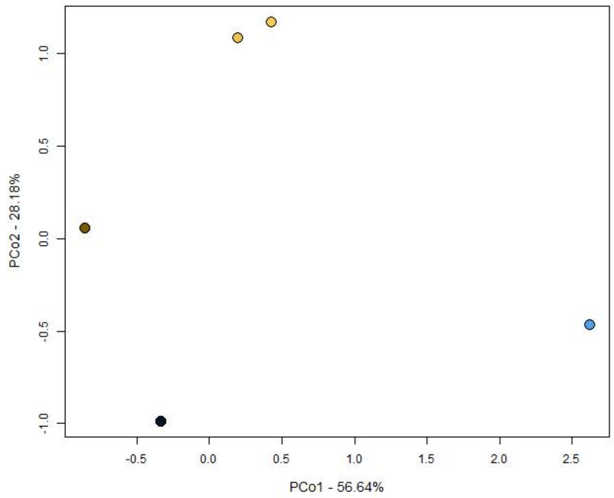

Figure 589 Map of *Speyeria egleis* showing the localities of the sequenced specimens (left). Nearby localities are grouped in pies. Colours match the bidimensional colour space of the PCoA projection (right) of max p-dists among sequences (dots). Sequences= 8; Hap obs.= 5; Hap asympt.= NA; Hap % obs.= NA%; GST= NaN; DST= NaN; HD= NA; ND= NA; max p-dist= 3.5%.

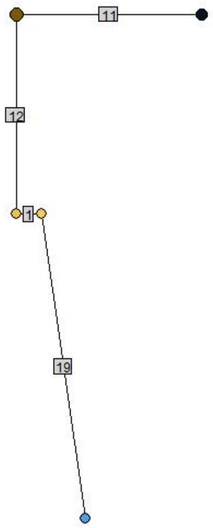

Figure 590: Haplotype network of *Speyeria egleis*. Sequences > 599 bp= 8.

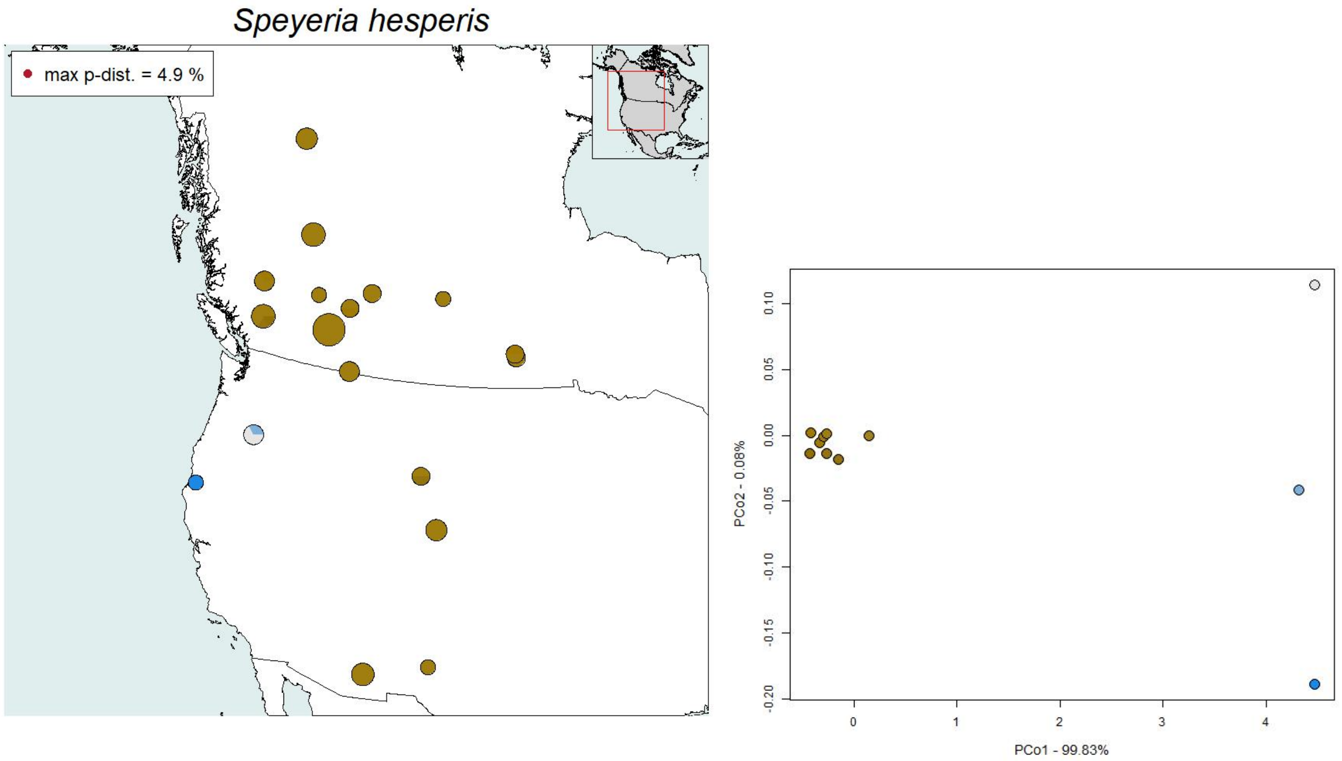

Figure 591 Map of *Speyeria hesperis* showing the localities of the sequenced specimens (left). Nearby localities are grouped in pies. Colours match the bidimensional colour space of the PCoA projection (right) of max p-dists among sequences (dots). Sequences= 69; Hap obs.= 6; Hap asympt.= 13.9; Hap % obs.= 43.2%; GST= 0.982; DST= 0.005; HD= 0.167; ND= 0.0053; max p-dist= 4.9%.

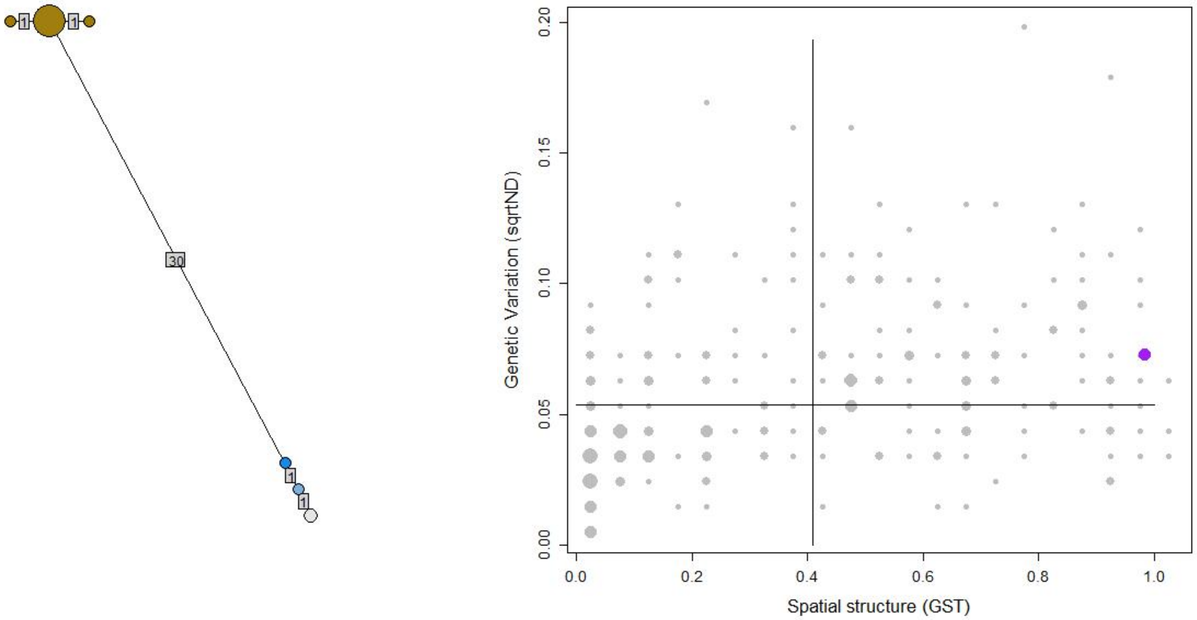

Figure 592: Haplotype network (left) of *Speyeria hesperis* sequences > 599 bp with colours matching the PCoA colour space (above). The bubble plot for mt-DNA polymorphism (square root transformed nucleotide diversity) and spatial structure (GST) among all species in the atlas and values for *Speyeria hesperis* (purple dot). The horizontal and vertical lines represent median values of nucleotide diversity and GST, respectively. Sequences > 599 bp= 69.

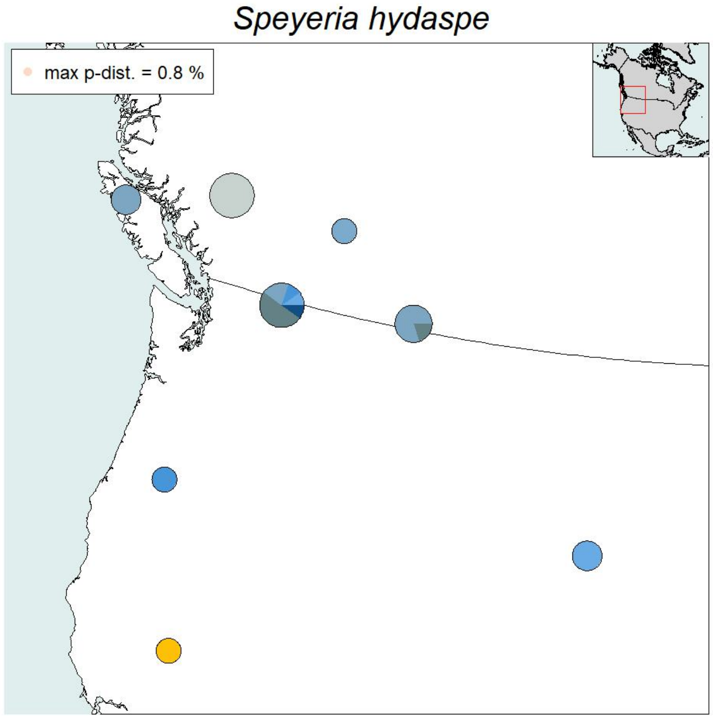

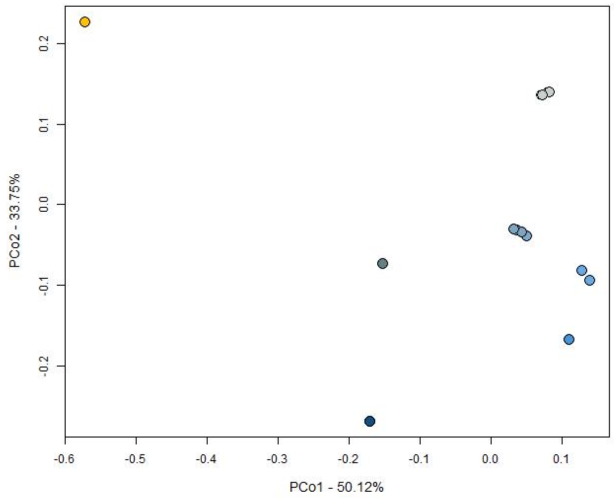

Figure 593 Map of *Speyeria hydaspe* showing the localities of the sequenced specimens (left). Nearby localities are grouped in pies. Colours match the bidimensional colour space of the PCoA projection (right) of max p-dists among sequences (dots). Sequences= 32; Hap obs.= 8; Hap asympt.= 10.2; Hap % obs.= 78.6%; GST= 0.656; DST= 0.0012; HD= 0.802; ND= 0.0021; max p-dist= 0.8%.

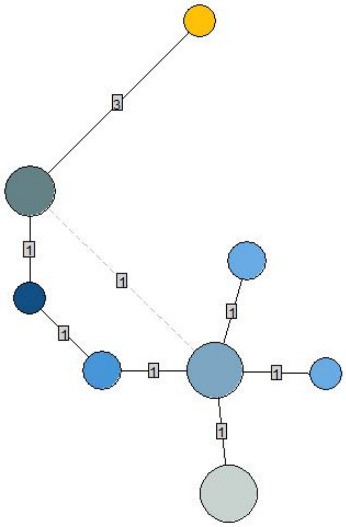

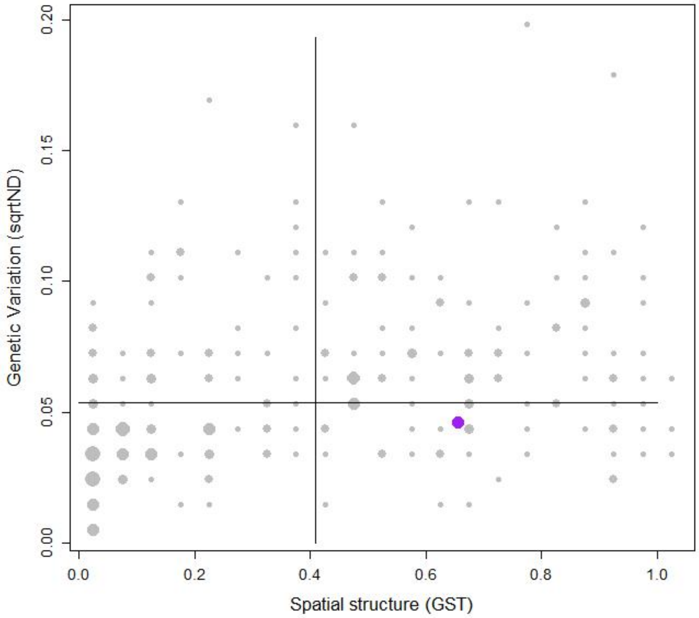

Figure 594: Haplotype network (left) of *Speyeria hydaspe* sequences > 599 bp with colours matching the PCoA colour space (above). The bubble plot for mt-DNA polymorphism (square root transformed nucleotide diversity) and spatial structure (GST) among all species in the atlas and values for *Speyeria hydaspe* (purple dot). The horizontal and vertical lines represent median values of nucleotide diversity and GST, respectively. Sequences > 599 bp= 32.

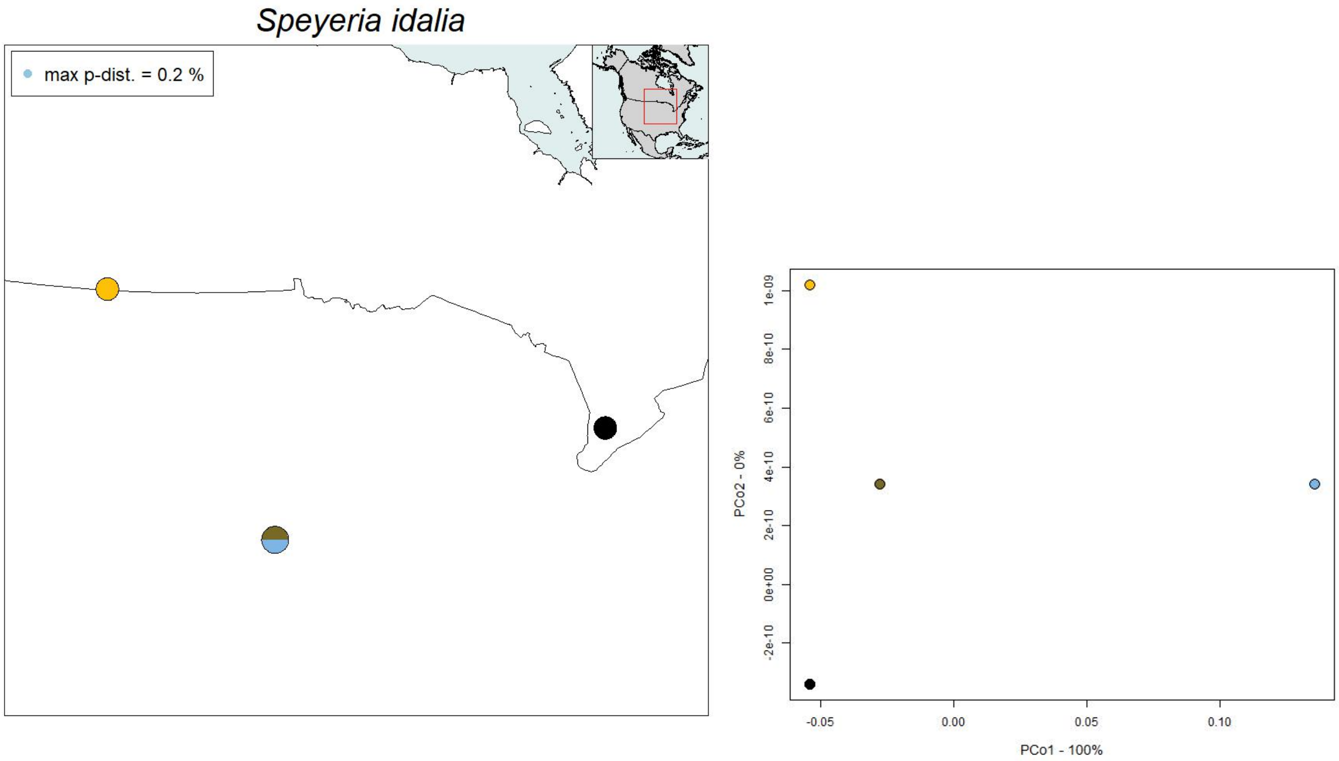

Figure 595 Map of *Speyeria idalia* showing the localities of the sequenced specimens (left). Nearby localities are grouped in pies. Colours match the bidimensional colour space of the PCoA projection (right) of max p-dists among sequences (dots). Sequences= 4; Hap obs.= 2; Hap asympt.= NA; Hap % obs.= NA%; GST= NaN; DST= NaN; HD= NA; ND= NA; max p-dist= 0.2%.

Haplotype network analysis and bubble plot of *Speyeria idalia* were not possible. Sequences > 599 bp = 2.

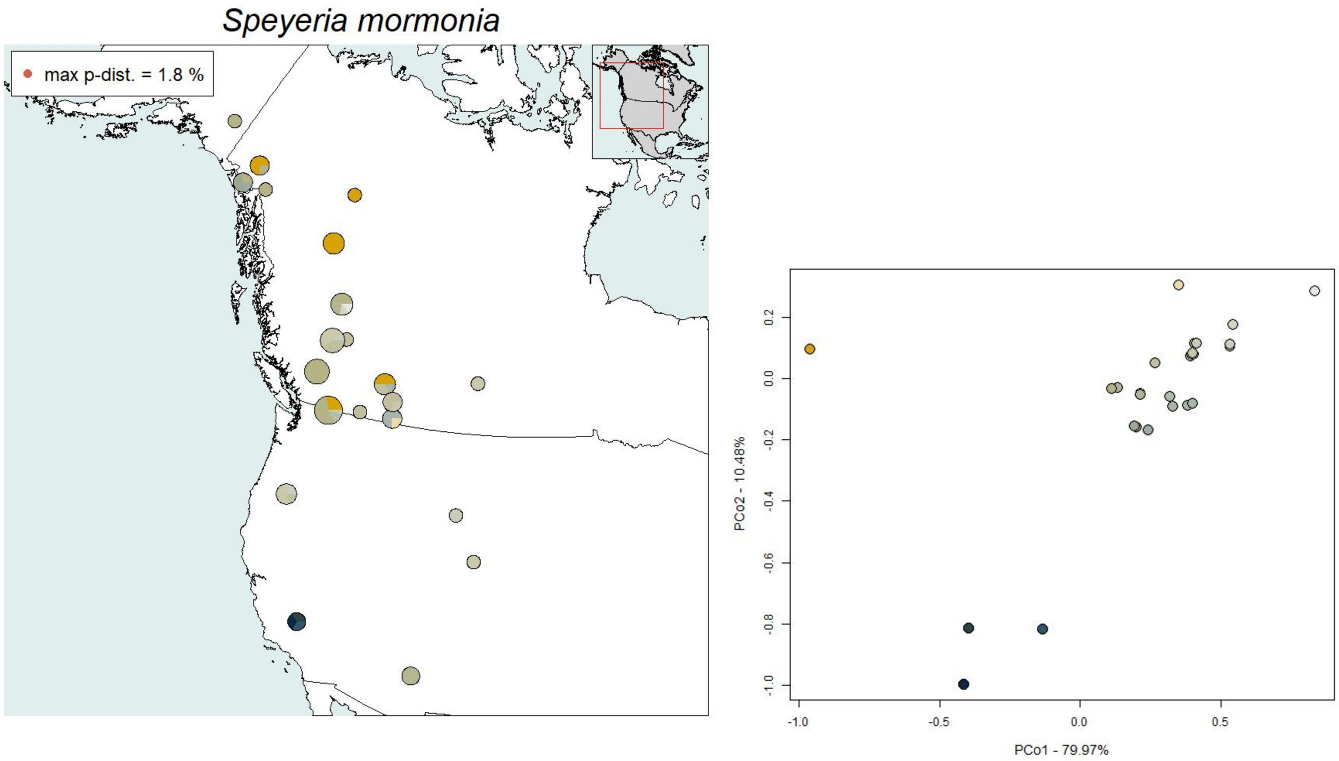

Figure 596 Map of *Speyeria mormonia* showing the localities of the sequenced specimens (left). Nearby localities are grouped in pies. Colours match the bidimensional colour space of the PCoA projection (right) of max p-dists among sequences (dots). Sequences= 93; Hap obs.= 21; Hap asympt.= 111; Hap % obs.= 18.9%; GST= 0.451; DST= 0.0026; HD= 0.832; ND= 0.0057; max p-dist= 1.8%.

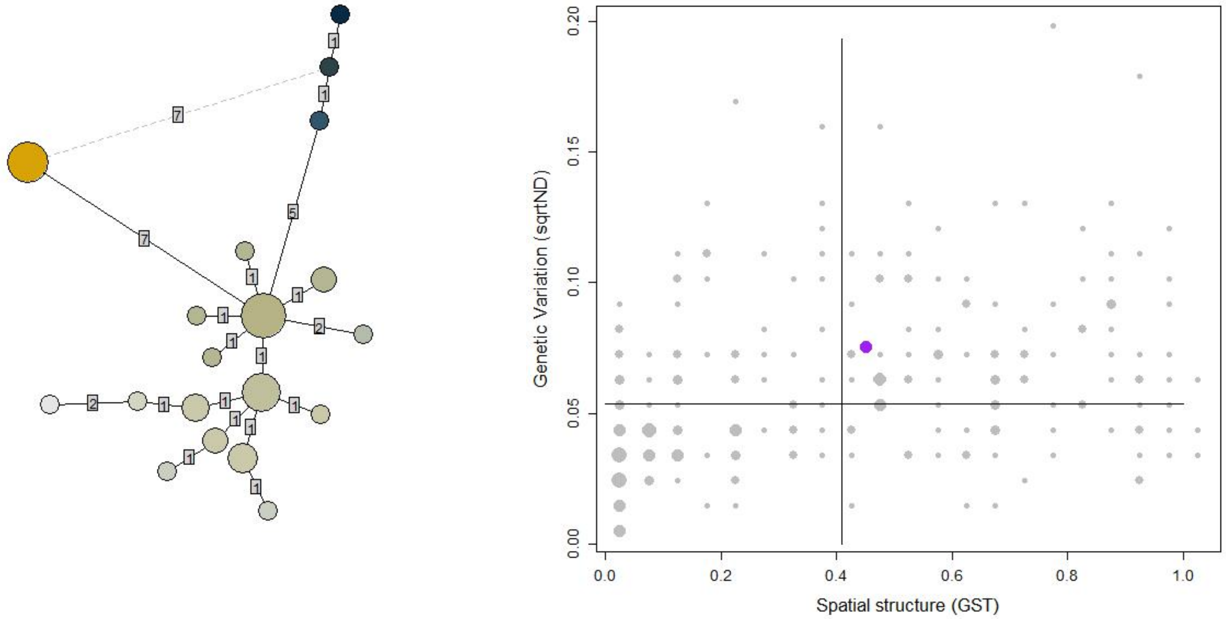

Figure 597: Haplotype network (left) of *Speyeria mormonia* sequences > 599 bp with colours matching the PCoA colour space (above). The bubble plot for mt-DNA polymorphism (square root transformed nucleotide diversity) and spatial structure (GST) among all species in the atlas and values for *Speyeria mormonia* (purple dot). The horizontal and vertical lines represent median values of nucleotide diversity and GST, respectively. Sequences > 599 bp= 87.

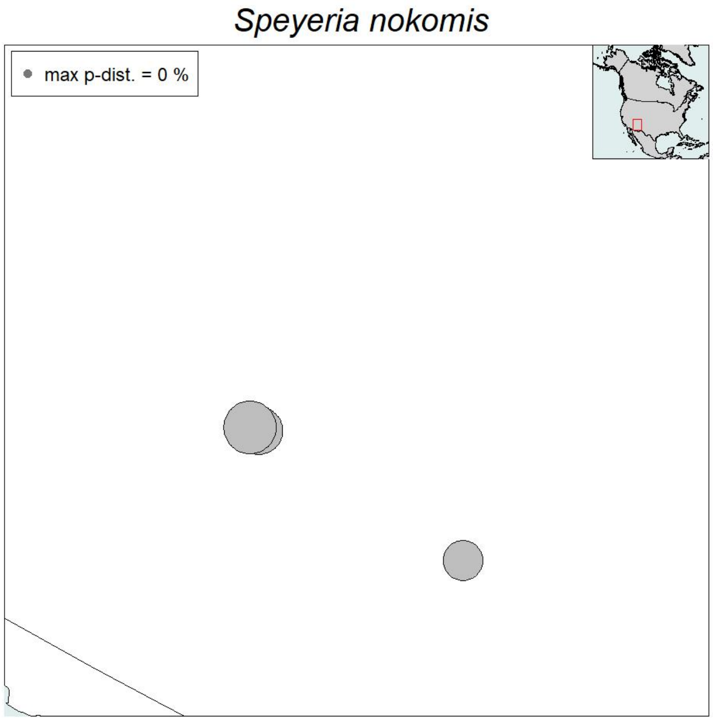

Figure 598: Map of *Speyeria nokomis* showing the localities of the sequenced specimens. Nearby localities are grouped in pies. Due to the presence of a single haplotype PCoA projection was not done and a single grey colour was plotted on the map. Sequences= 6; Hap obs.= 1; Hap asympt.= NA; Hap % obs.= NA%; GST= NaN; DST= NaN; HD= NA; ND= NA; max p-dist= 0%.

Haplotype network analysis and bubble plot of *Speyeria nokomis* were not possible. Sequences > 599 bp = 6.

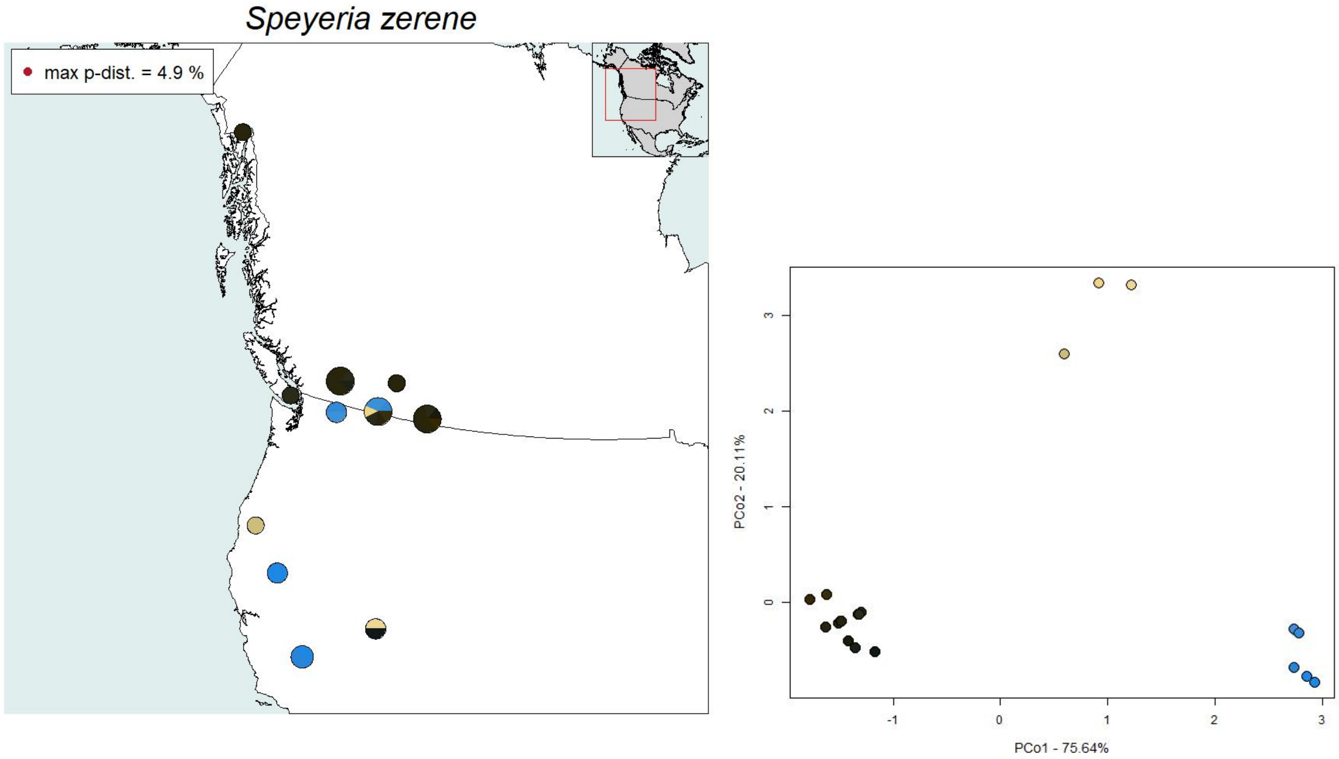

Figure 599 Map of *Speyeria zerene* showing the localities of the sequenced specimens (left). Nearby localities are grouped in pies. Colours match the bidimensional colour space of the PCoA projection (right) of max p-dists among sequences (dots). Sequences= 34; Hap obs.= 21; Hap asympt.= 52.1; Hap % obs.= 40.3%; GST= 0.352; DST= 0.0073; HD= 0.913; ND= 0.0248; max p-dist= 4.9%.

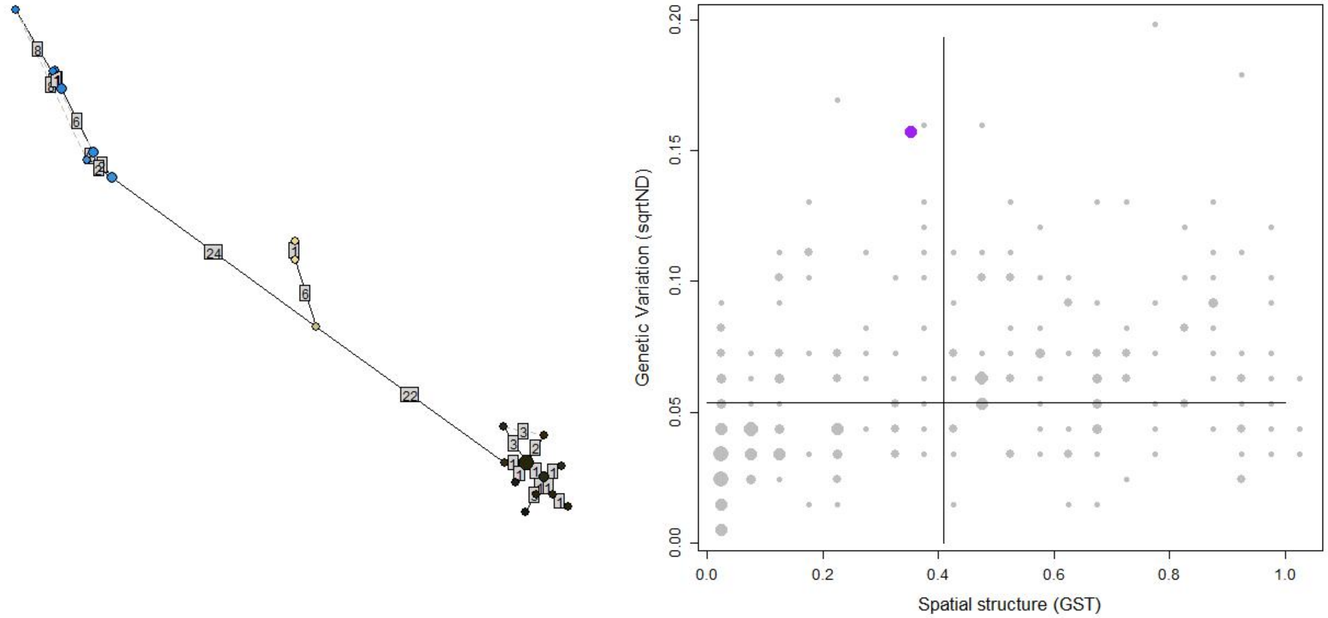

Figure 600: Haplotype network (left) of *Speyeria zerene* sequences > 599 bp with colours matching the PCoA colour space (above). The bubble plot for mt-DNA polymorphism (square root transformed nucleotide diversity) and spatial structure (GST) among all species in the atlas and values for *Speyeria zerene* (purple dot). The horizontal and vertical lines represent median values of nucleotide diversity and GST, respectively. Sequences > 599 bp= 33.

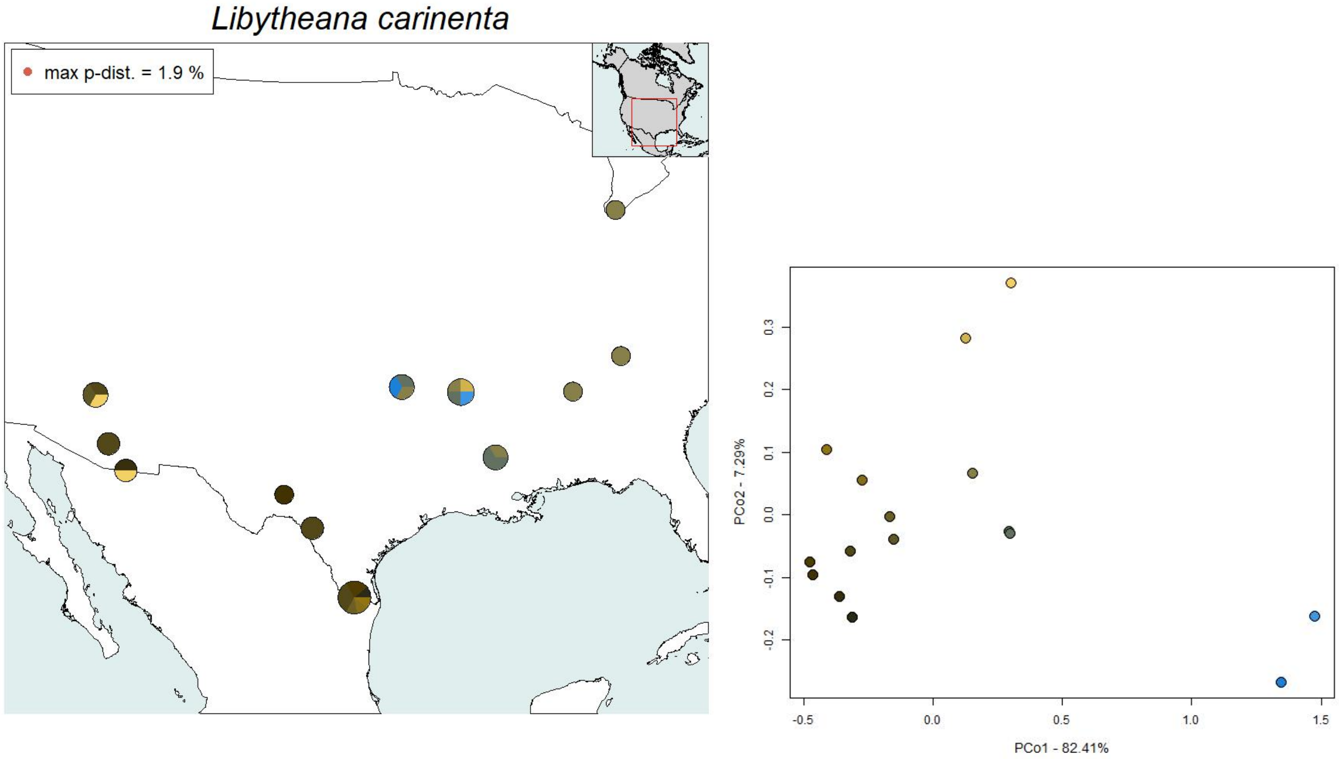

Figure 601 Map of *Libytheana carinenta* showing the localities of the sequenced specimens (left). Nearby localities are grouped in pies. Colours match the bidimensional colour space of the PCoA projection (right) of max p-dists among sequences (dots). Sequences= 32; Hap obs.= 15; Hap asympt.= 31.1; Hap % obs.= 48.2%; GST= 0.435; DST= 0.0026; HD= 0.873; ND= 0.0052; max p-dist= 1.9%.

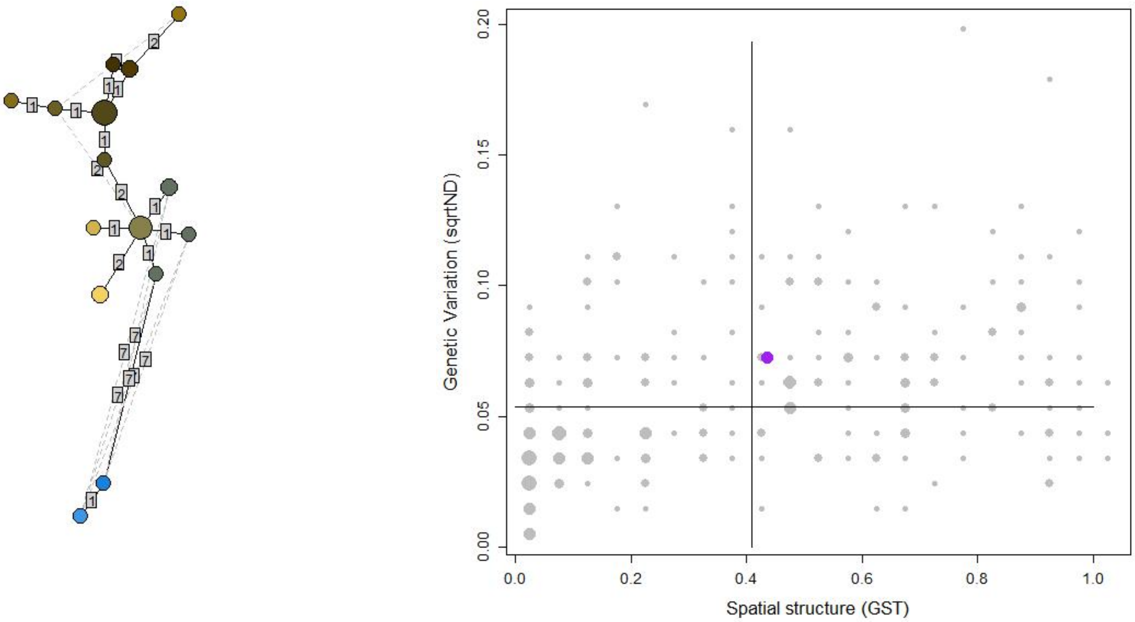

Figure 602: Haplotype network (left) of *Libytheana carinenta* sequences > 599 bp with colours matching the PCoA colour space (above). The bubble plot for mt-DNA polymorphism (square root transformed nucleotide diversity) and spatial structure (GST) among all species in the atlas and values for *Libytheana carinenta* (purple dot). The horizontal and vertical lines represent median values of nucleotide diversity and GST, respectively. Sequences > 599 bp= 31.

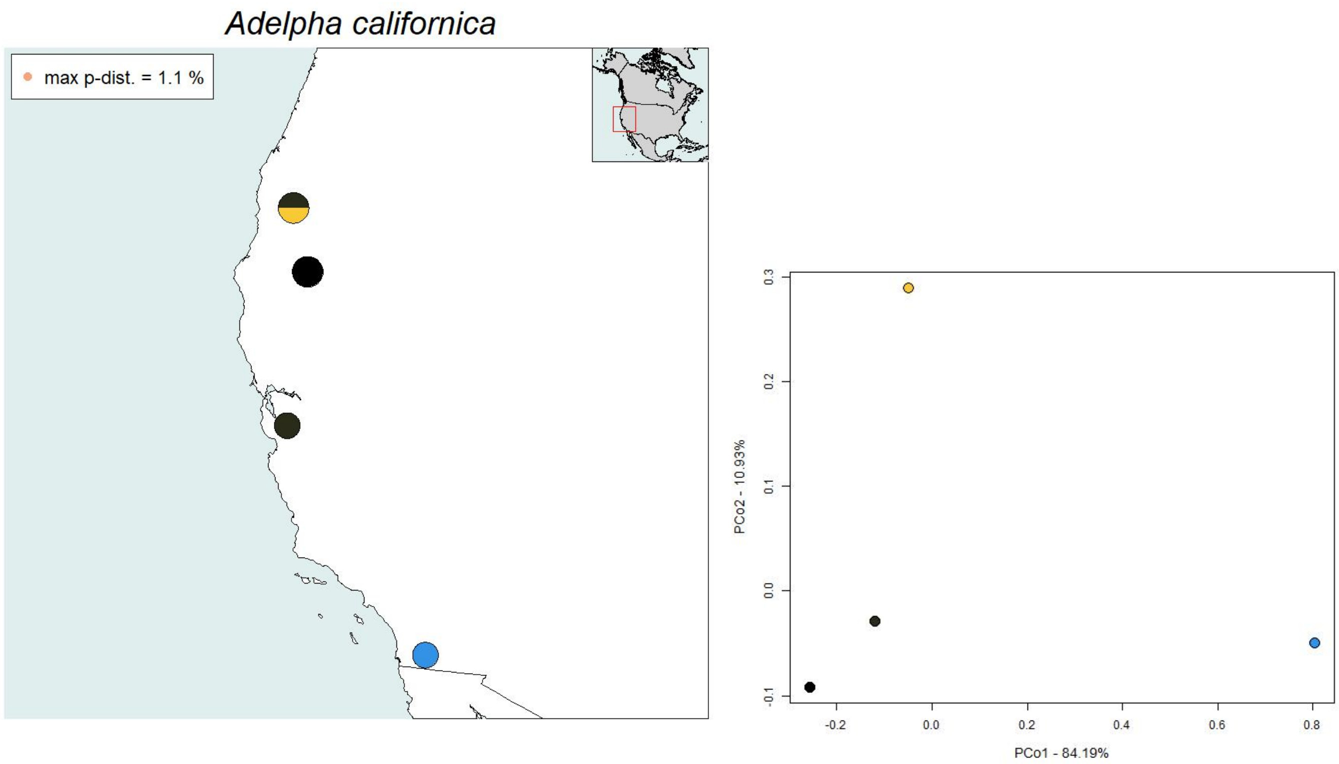

Figure 603 Map of *Adelpha californica* showing the localities of the sequenced specimens (left). Nearby localities are grouped in pies. Colours match the bidimensional colour space of the PCoA projection (right) of max p-dists among sequences (dots). Sequences= 6; Hap obs.= 5; Hap asympt.= NA; Hap % obs.= NA%; GST= NaN; DST= NaN; HD= NA; ND= NA; max p-dist= 1.1%.

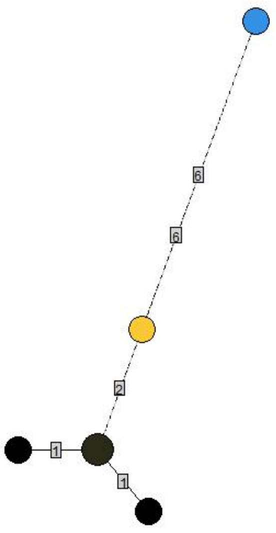

Figure 604: Haplotype network of *Adelpha californica*. Sequences > 599 bp= 6.

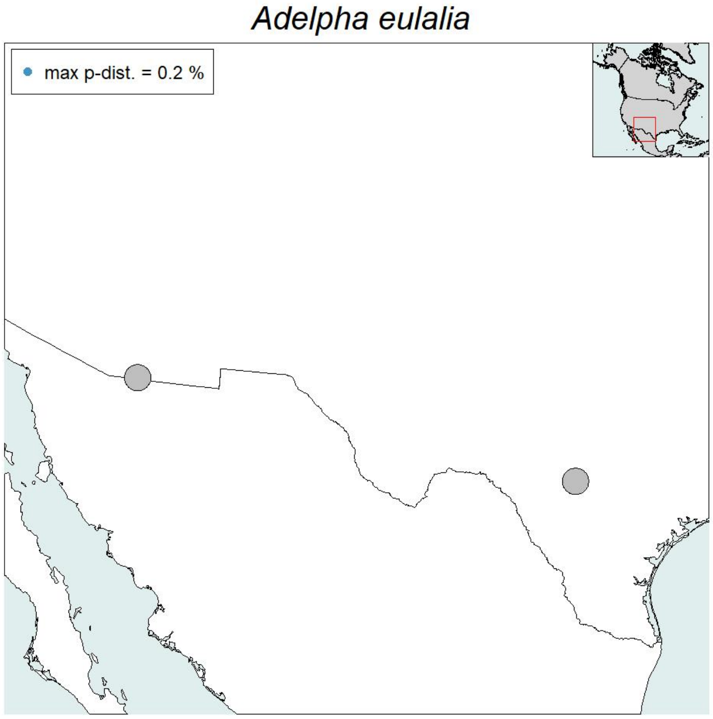

Figure 605: Map of *Adelpha eulalia* showing the localities of the sequenced specimens. Nearby localities are grouped in pies. Due to the presence of a single haplotype PCoA projection was not done and a single grey colour was plotted on the map. Sequences= 2; Hap obs.= 2; Hap asympt.= NA; Hap % obs.= NA%; GST= NaN; DST= NaN; HD= NA; ND= NA; max p-dist= 0.2%.

Haplotype network analysis and bubble plot of *Adelpha eulalia* were not possible. Sequences > 599 bp = 2.

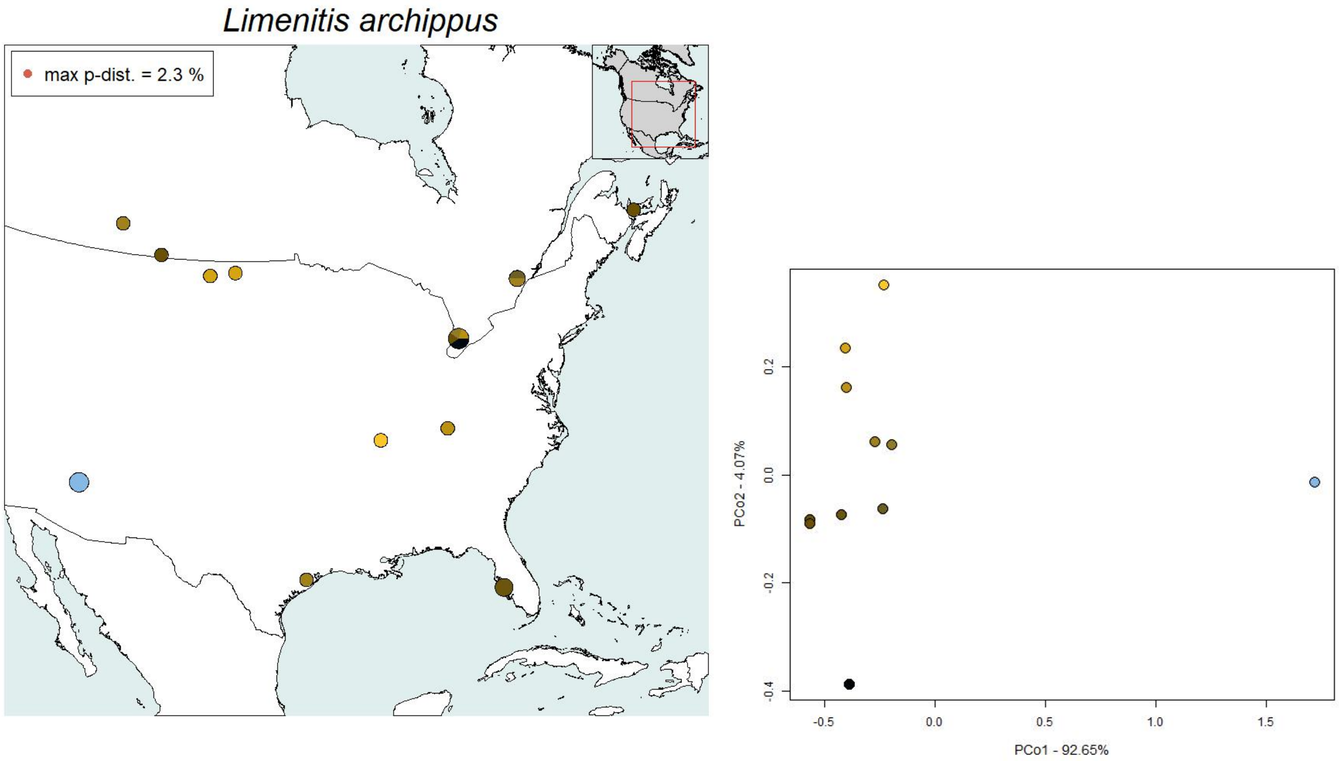

Figure 606 Map of *Limenitis archippus* showing the localities of the sequenced specimens (left). Nearby localities are grouped in pies. Colours match the bidimensional colour space of the PCoA projection (right) of max p-dists among sequences (dots). Sequences= 22; Hap obs.= 11; Hap asympt.= 15; Hap % obs.= 73.4%; GST= 0.876; DST= 0.0104; HD= 0.922; ND= 0.0087; max p-dist= 2.3%.

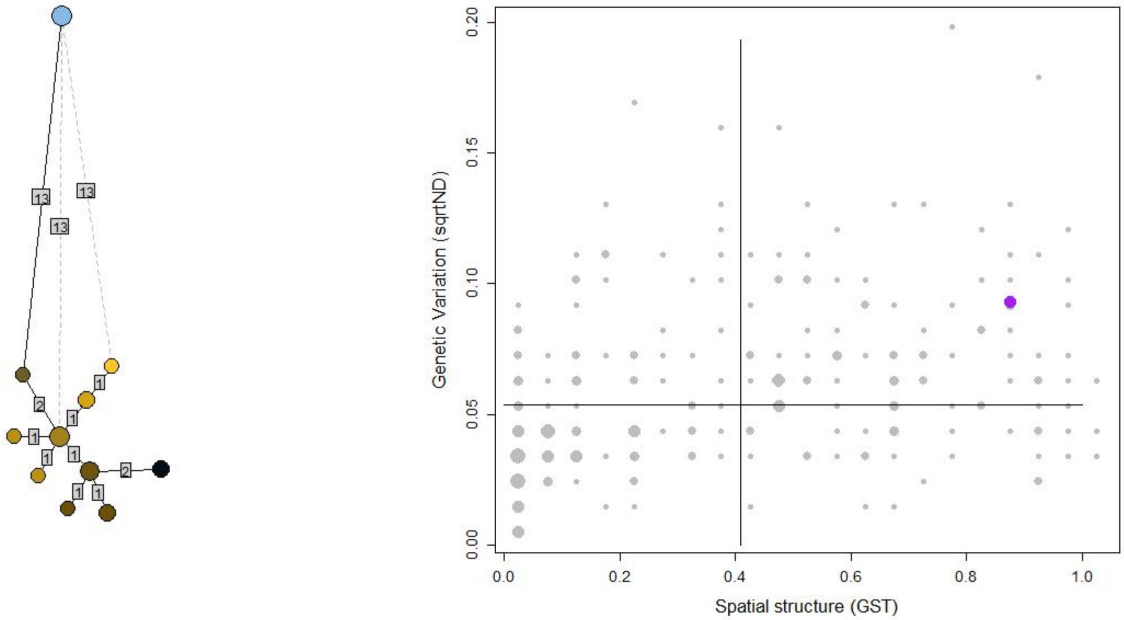

Figure 607: Haplotype network (left) of *Limenitis archippus* sequences > 599 bp with colours matching the PCoA colour space (above). The bubble plot for mt-DNA polymorphism (square root transformed nucleotide diversity) and spatial structure (GST) among all species in the atlas and values for *Limenitis archippus* (purple dot). The horizontal and vertical lines represent median values of nucleotide diversity and GST, respectively. Sequences > 599 bp= 22.

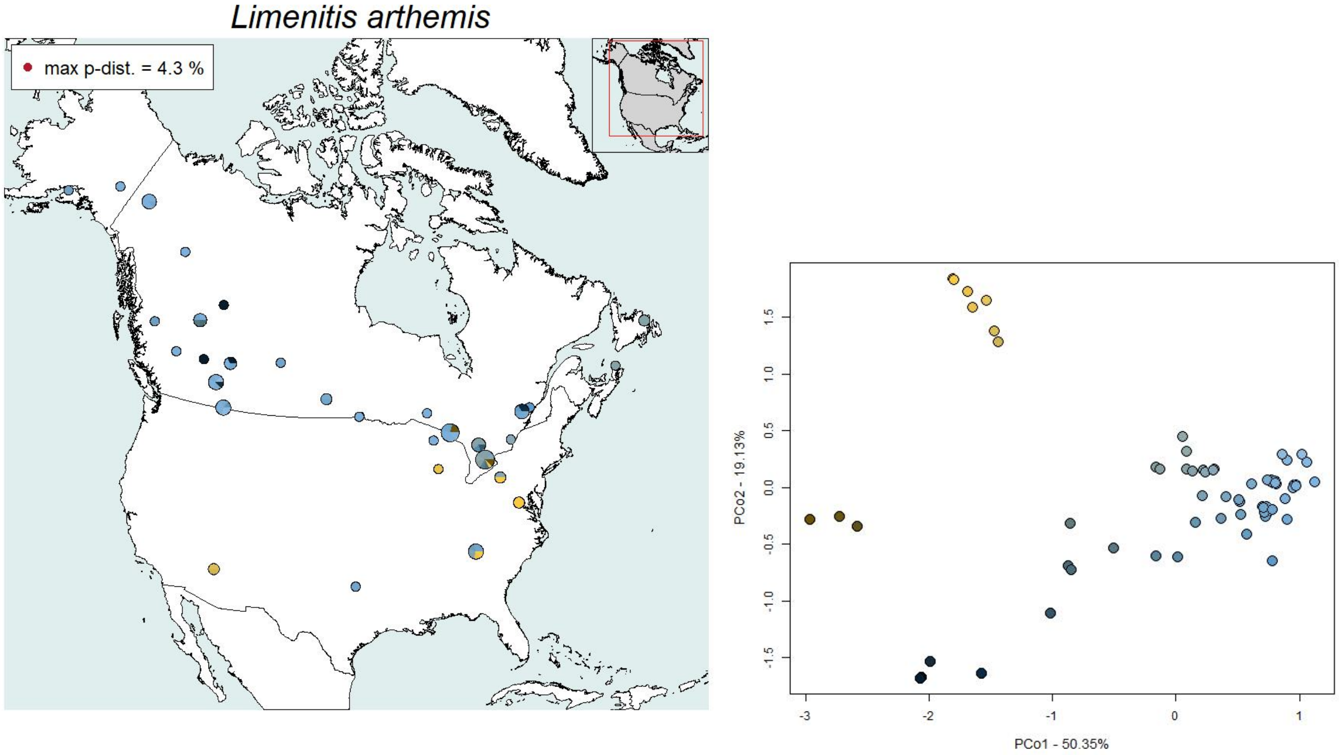

Figure 608 Map of *Limenitis arthemis* showing the localities of the sequenced specimens (left). Nearby localities are grouped in pies. Colours match the bidimensional colour space of the PCoA projection (right) of max p-dists among sequences (dots). Sequences= 102; Hap obs.= 55; Hap asympt.= 404.2; Hap % obs.= 13.6%; GST= 0.163; DST= 0.0025; HD= 0.939; ND= 0.0164; max p-dist= 4.3%.

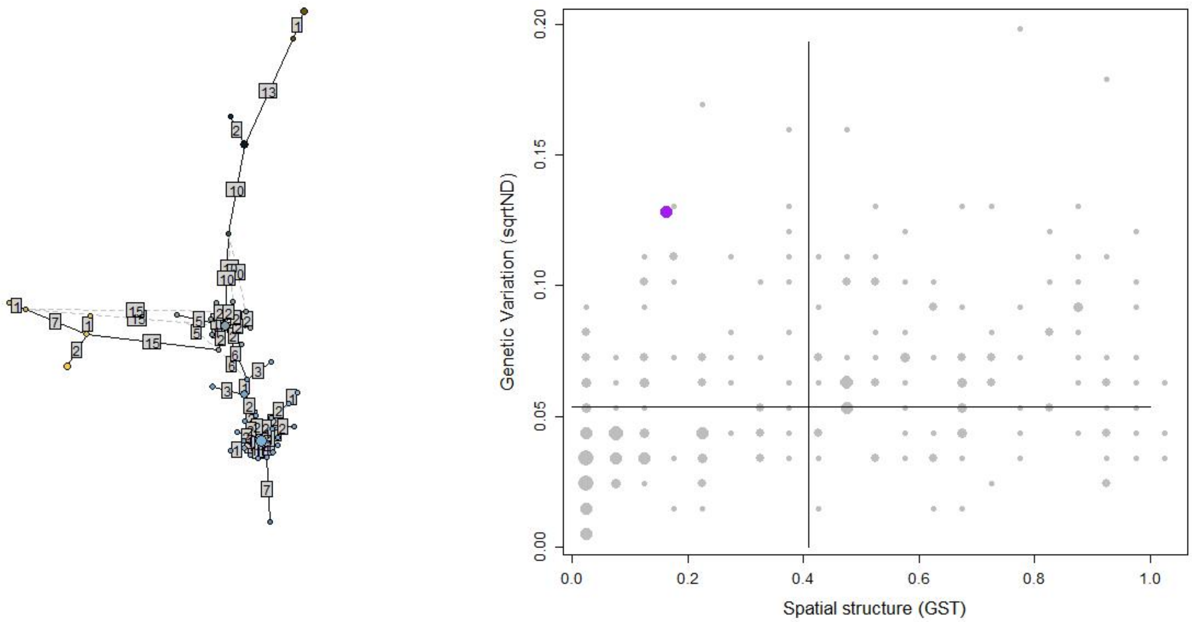

Figure 609: Haplotype network (left) of *Limenitis arthemis* sequences > 599 bp with colours matching the PCoA colour space (above). The bubble plot for mt-DNA polymorphism (square root transformed nucleotide diversity) and spatial structure (GST) among all species in the atlas and values for *Limenitis arthemis* (purple dot). The horizontal and vertical lines represent median values of nucleotide diversity and GST, respectively. Sequences > 599 bp= 91.

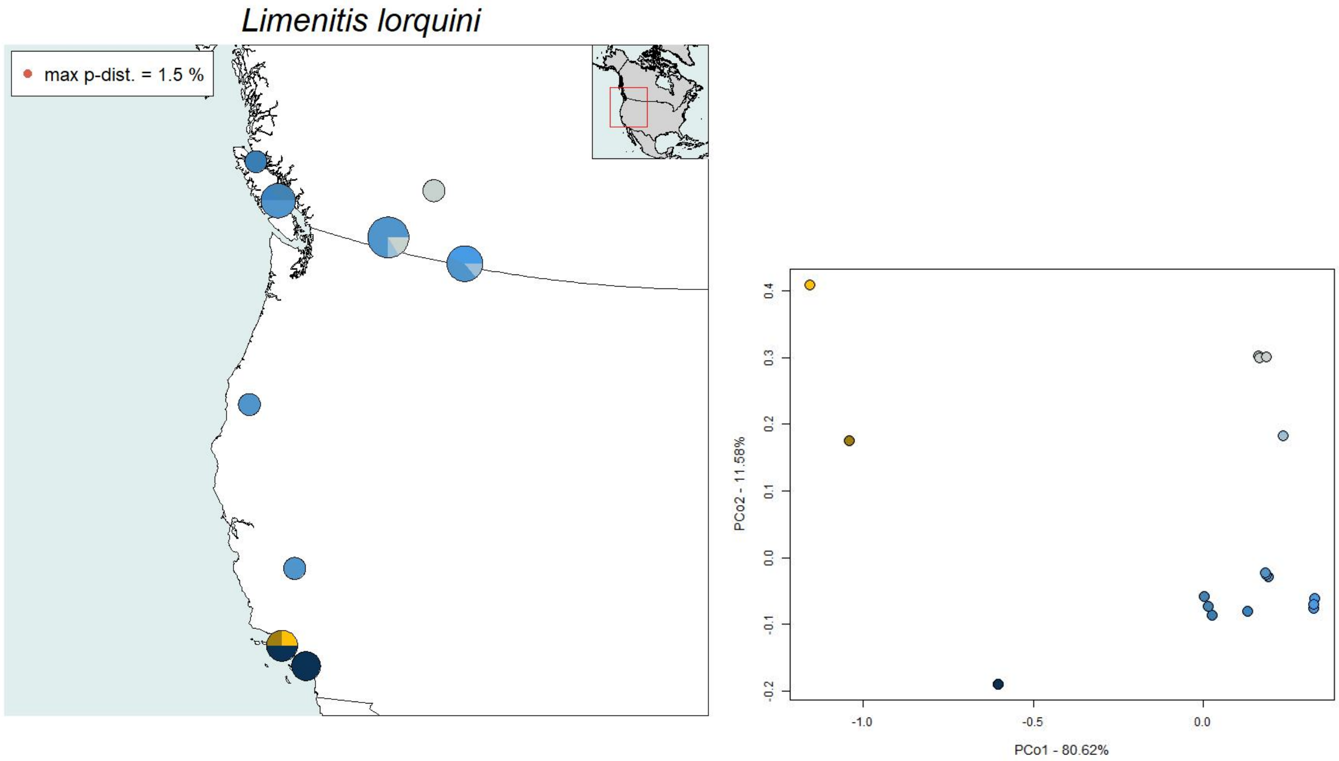

Figure 610 Map of *Limenitis lorquini* showing the localities of the sequenced specimens (left). Nearby localities are grouped in pies. Colours match the bidimensional colour space of the PCoA projection (right) of max p-dists among sequences (dots). Sequences= 36; Hap obs.= 11; Hap asympt.= 28.5; Hap % obs.= 38.6%; GST= 0.7; DST= 0.0028; HD= 0.757; ND= 0.0036; max p-dist= 1.5%.

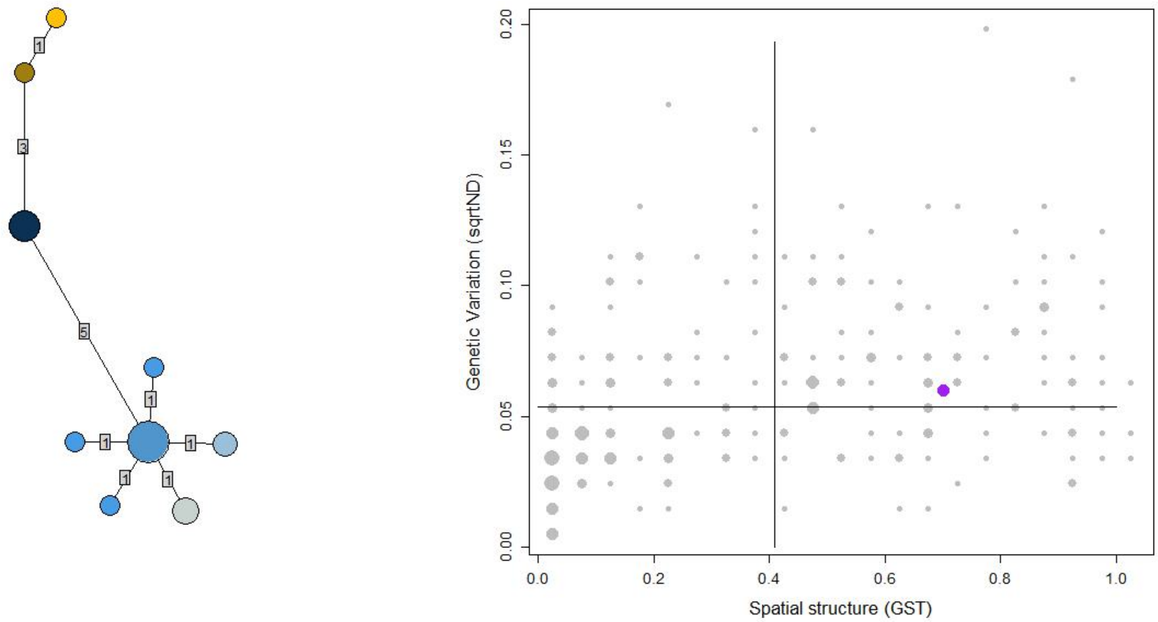

Figure 611: Haplotype network (left) of *Limenitis lorquini* sequences > 599 bp with colours matching the PCoA colour space (above). The bubble plot for mt-DNA polymorphism (square root transformed nucleotide diversity) and spatial structure (GST) among all species in the atlas and values for *Limenitis lorquini* (purple dot). The horizontal and vertical lines represent median values of nucleotide diversity and GST, respectively. Sequences > 599 bp= 32.

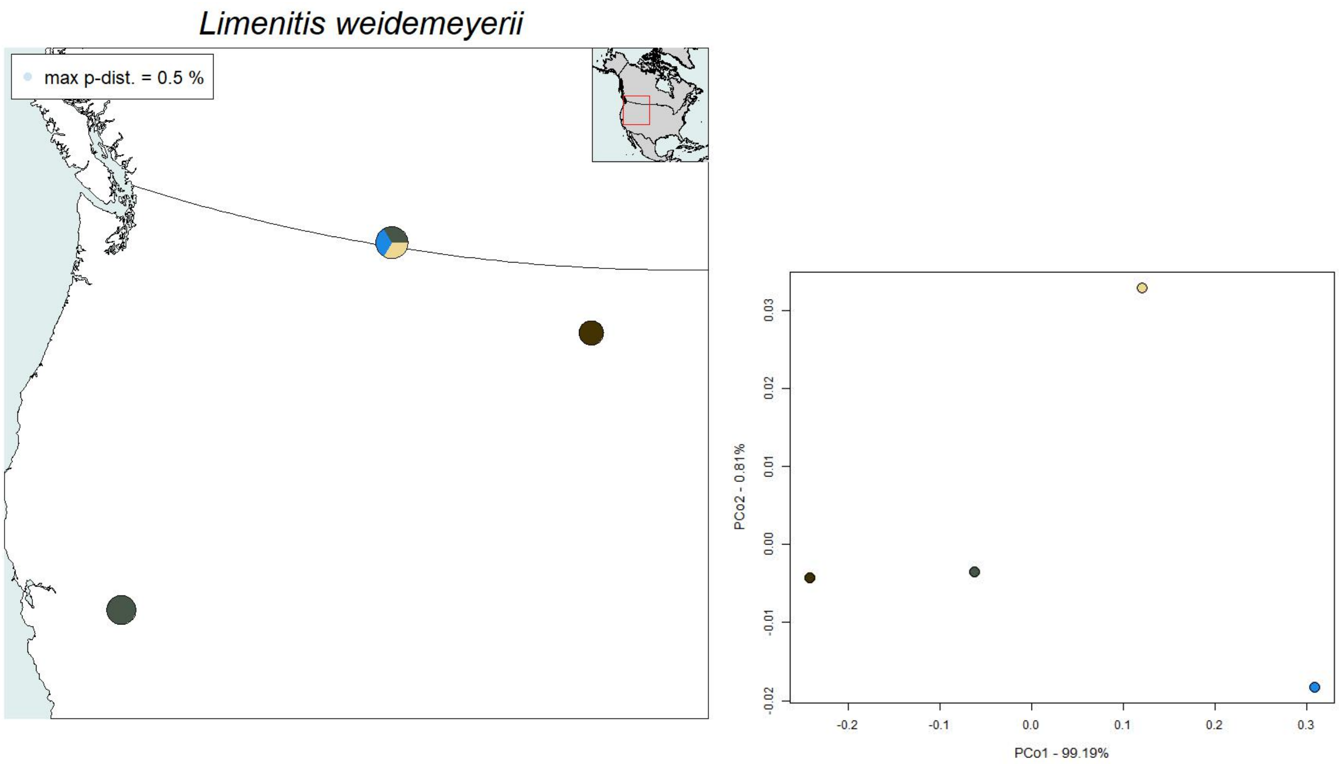

Figure 612 Map of *Limenitis weidemeyerii* showing the localities of the sequenced specimens (left). Nearby localities are grouped in pies. Colours match the bidimensional colour space of the PCoA projection (right) of max p-dists among sequences (dots). Sequences= 6; Hap obs.= 4; Hap asympt.= NA; Hap % obs.= NA%; GST= NaN; DST= NaN; HD= NA; ND= NA; max p-dist= 0.5%.

Haplotype network analysis and bubble plot of *Limenitis weidemeyerii* were not possible. Sequences > 599 bp = 4.

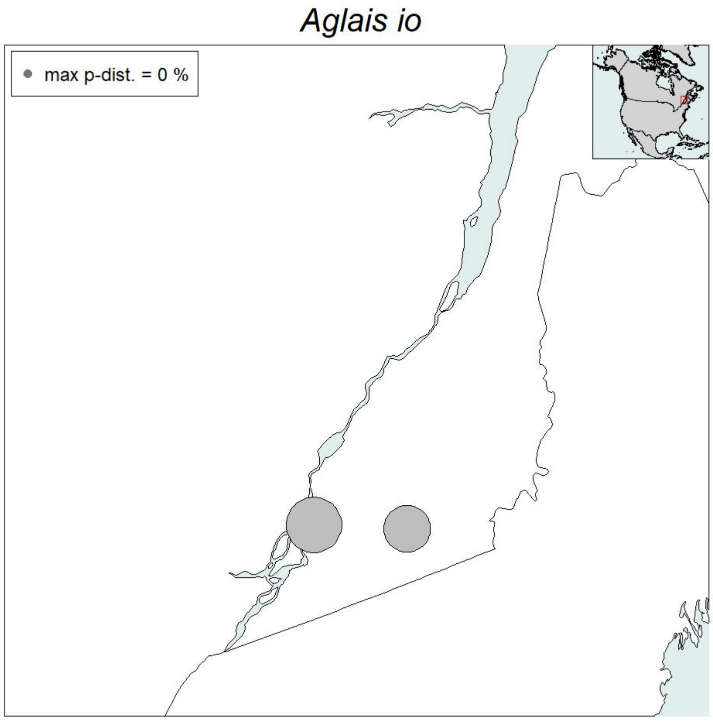

Figure 613: Map of *Aglais io* showing the localities of the sequenced specimens. Nearby localities are grouped in pies. Due to the presence of a single haplotype PCoA projection was not done and a single grey colour was plotted on the map. Sequences= 3; Hap obs.= 1; Hap asympt.= NA; Hap % obs.= NA%; GST= NaN; DST= NaN; HD= NA; ND= NA; max p-dist= 0%.

Haplotype network analysis and bubble plot of *Aglais io* were not possible. Sequences > 599 bp = 3.

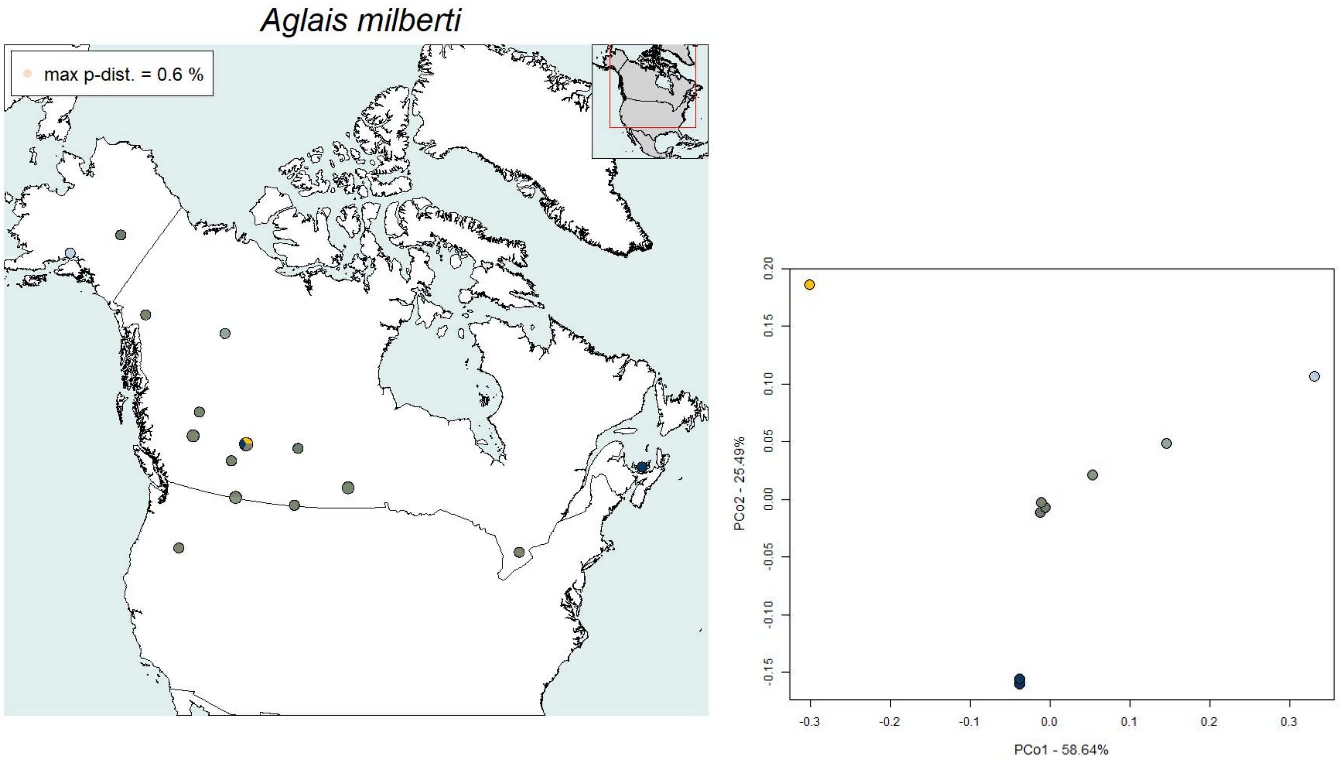

Figure 614 Map of *Aglais milberti* showing the localities of the sequenced specimens (left). Nearby localities are grouped in pies. Colours match the bidimensional colour space of the PCoA projection (right) of max p-dists among sequences (dots). Sequences= 20; Hap obs.= 6; Hap asympt.= 15.5; Hap % obs.= 38.7%; GST= NaN; DST= NaN; HD= 0.447; ND= 0.0011; max p-dist= 0.6%.

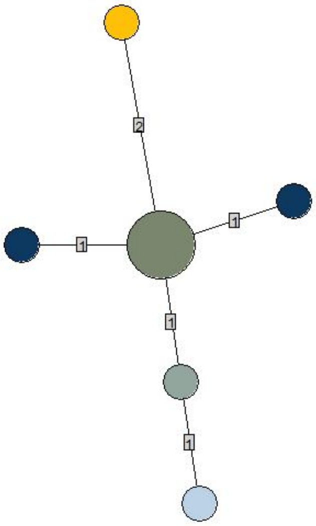

Figure 615: Haplotype network of *Aglais milberti*. Sequences > 599 bp= 19.

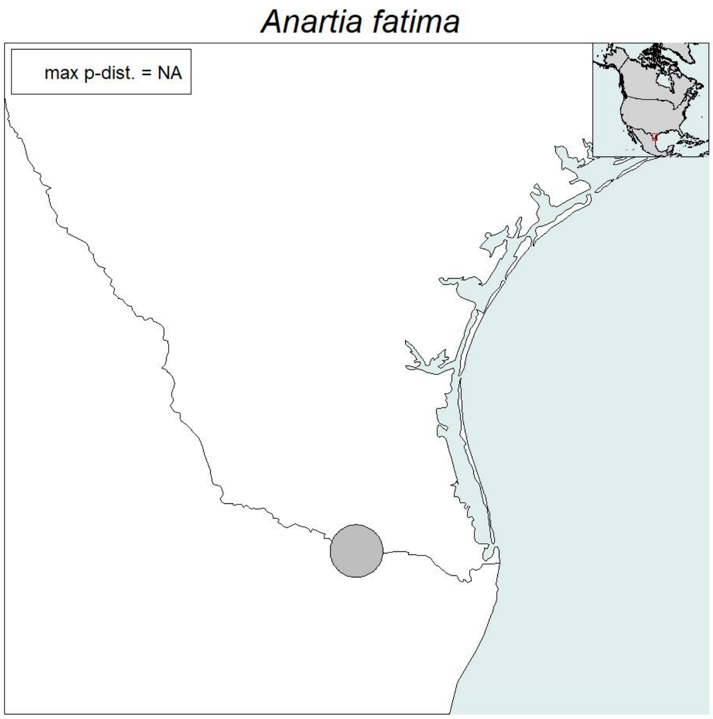

Figure 616: Map of *Anartia fatima* showing the localities of the sequenced specimens. Nearby localities are grouped in pies. Due to the presence of a single haplotype PCoA projection was not done and a single grey colour was plotted on the map. Sequences= 1; Hap obs.= NA; Hap asympt.= NA; Hap % obs.= NA; GST= NaN; DST= NaN; HD= NA; ND= NA; max p-dist= NA.

Haplotype network analysis and bubble plot of *Anartia fatima* were not possible. Sequences > 599 bp = 1.

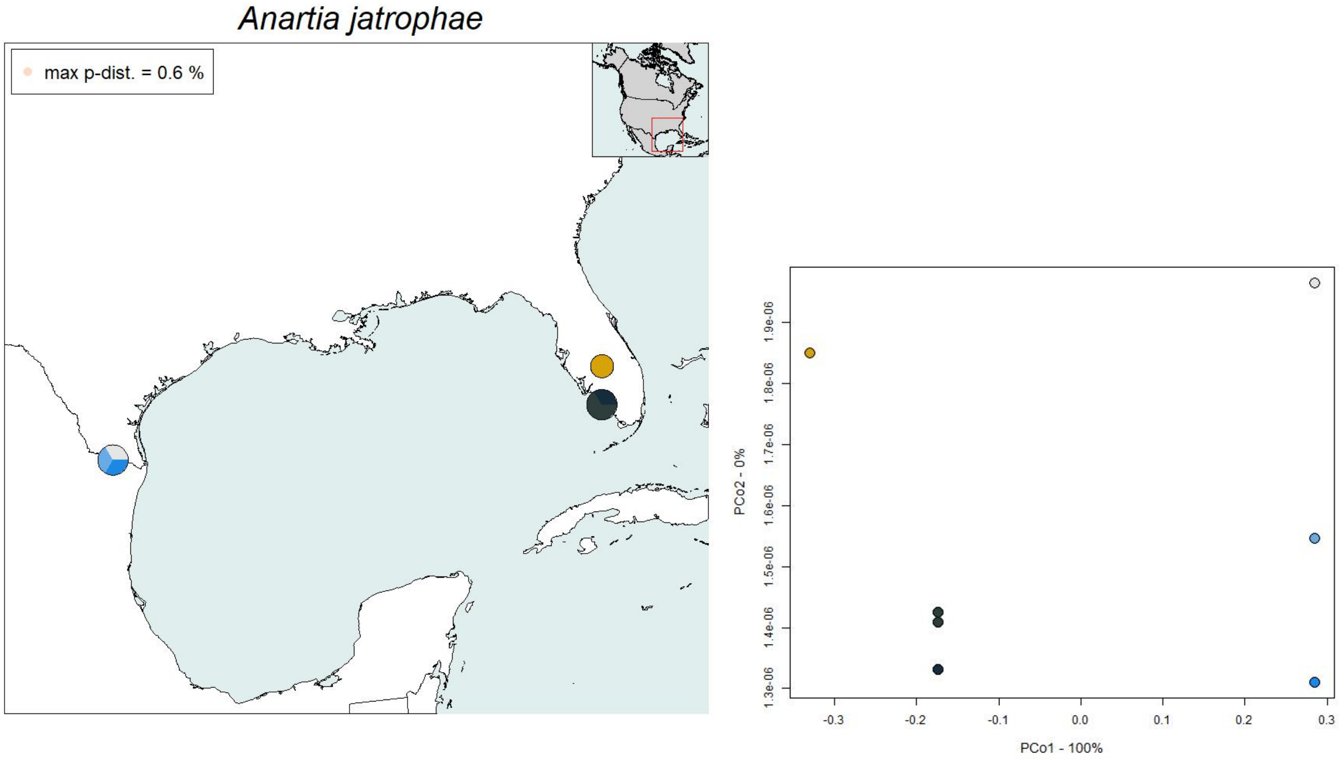

Figure 617 Map of *Anartia jatrophae* showing the localities of the sequenced specimens (left). Nearby localities are grouped in pies. Colours match the bidimensional colour space of the PCoA projection (right) of max p-dists among sequences (dots). Sequences= 7; Hap obs.= 3; Hap asympt.= NA; Hap % obs.= NA%; GST= NaN; DST= NaN; HD= NA; ND= NA; max p-dist= 0.6%.

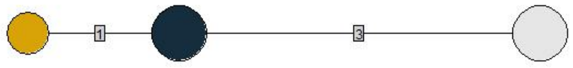

Figure 618: Haplotype network of *Anartia jatrophae*. Sequences > 599 bp= 7.

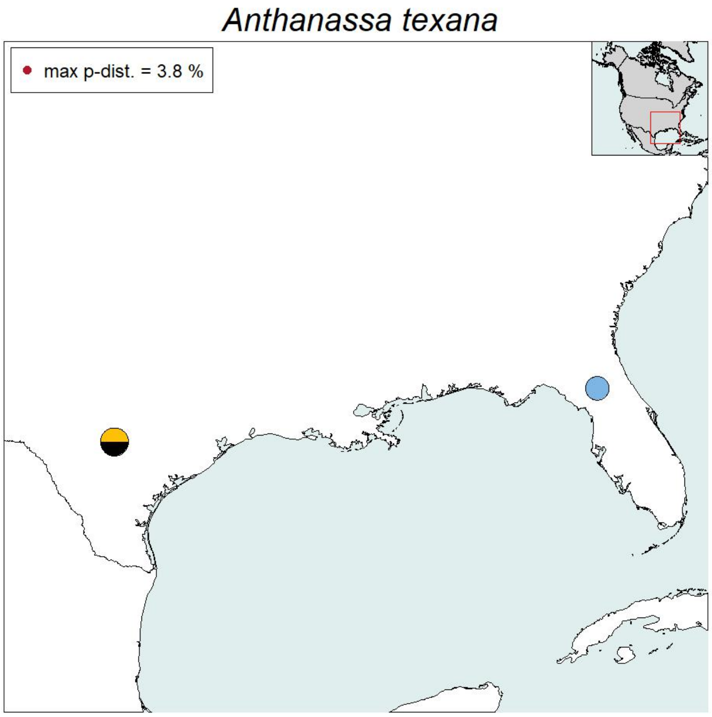

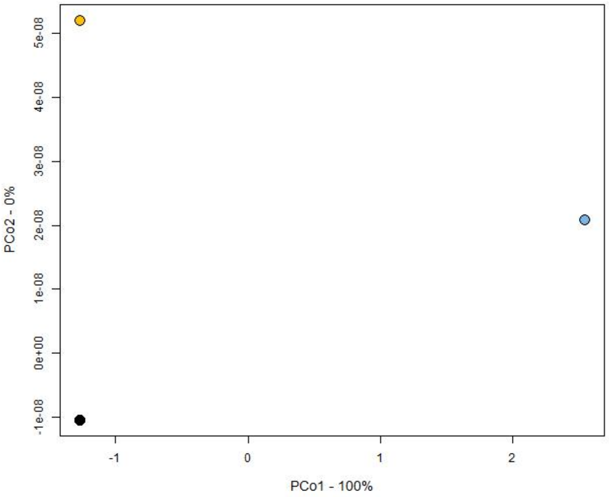

Figure 619 Map of *Anthanassa texana* showing the localities of the sequenced specimens (left). Nearby localities are grouped in pies. Colours match the bidimensional colour space of the PCoA projection (right) of max p-dists among sequences (dots). Sequences= 3; Hap obs.= 2; Hap asympt.= NA; Hap % obs.= NA%; GST= NaN; DST= NaN; HD= NA; ND= NA; max p-dist= 3.8%.

Haplotype network analysis and bubble plot of *Anthanassa texana* were not possible. Sequences > 599 bp = 3.

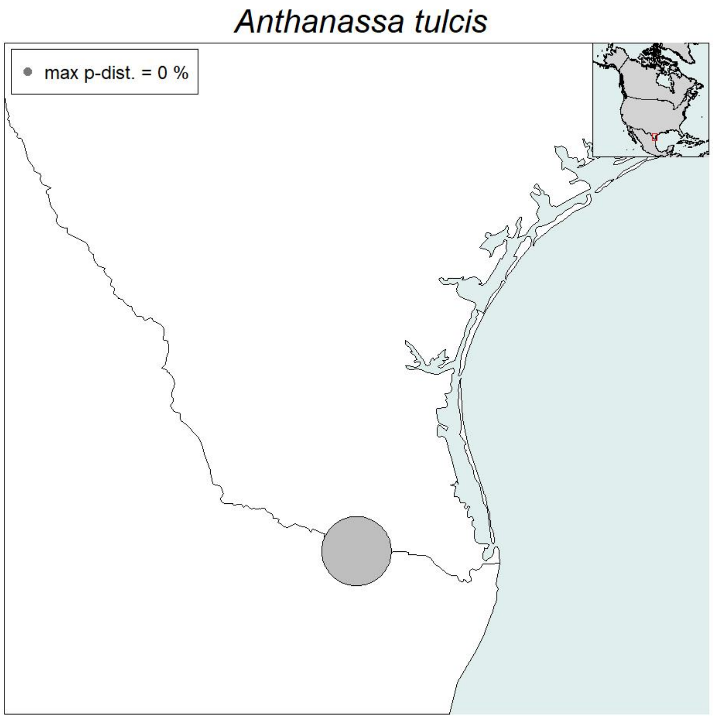

Figure 620: Map of *Anthanassa tulcis* showing the localities of the sequenced specimens. Nearby localities are grouped in pies. Due to the presence of a single haplotype PCoA projection was not done and a single grey colour was plotted on the map. Sequences= 3; Hap obs.= 1; Hap asympt.= NA; Hap % obs.= NA%; GST= NaN; DST= NaN; HD= NA; ND= NA; max p-dist= 0%.

Haplotype network analysis and bubble plot of *Anthanassa tulcis* were not possible. Sequences > 599 bp = 3.

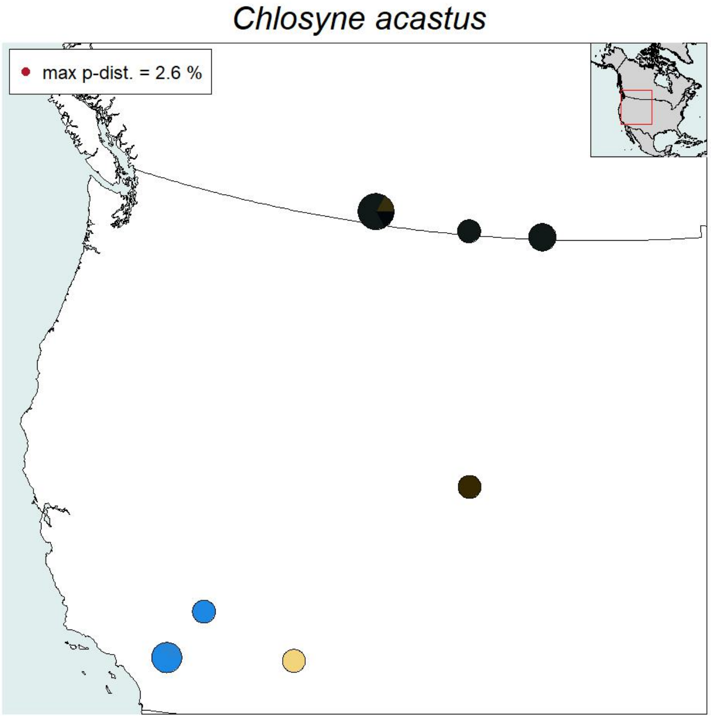

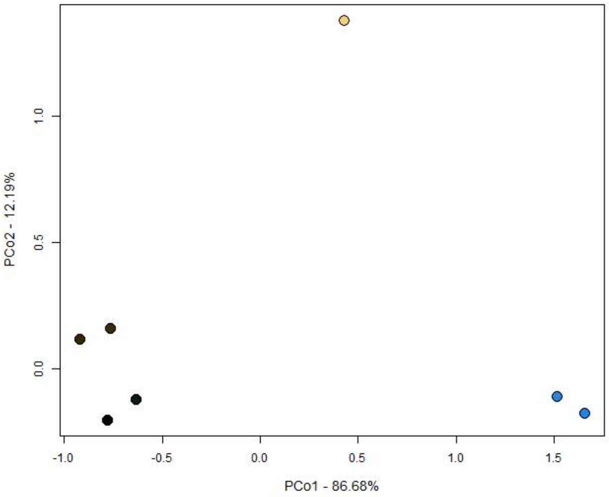

Figure 621 Map of *Chlosyne acastus* showing the localities of the sequenced specimens (left). Nearby localities are grouped in pies. Colours match the bidimensional colour space of the PCoA projection (right) of max p-dists among sequences (dots). Sequences= 15; Hap obs.= 9; Hap asympt.= 35.1; Hap % obs.= 25.6%; GST= 0.891; DST= 0.0098; HD= 0.8; ND= 0.012; max p-dist= 2.6%.

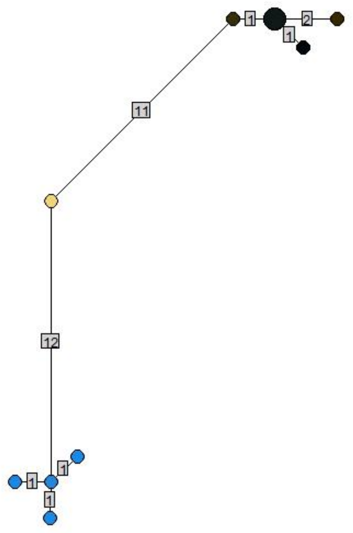

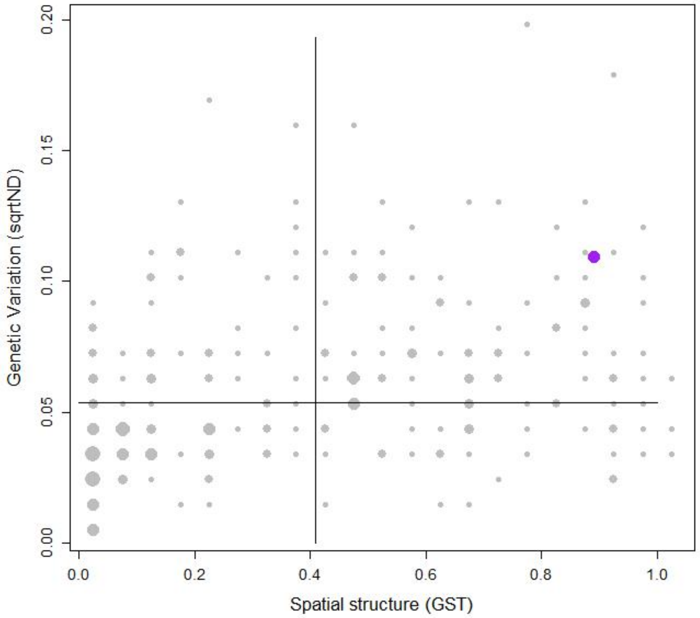

Figure 622: Haplotype network (left) of *Chlosyne acastus* sequences > 599 bp with colours matching the PCoA colour space (above). The bubble plot for mt-DNA polymorphism (square root transformed nucleotide diversity) and spatial structure (GST) among all species in the atlas and values for *Chlosyne acastus* (purple dot). The horizontal and vertical lines represent median values of nucleotide diversity and GST, respectively. Sequences > 599 bp= 15.

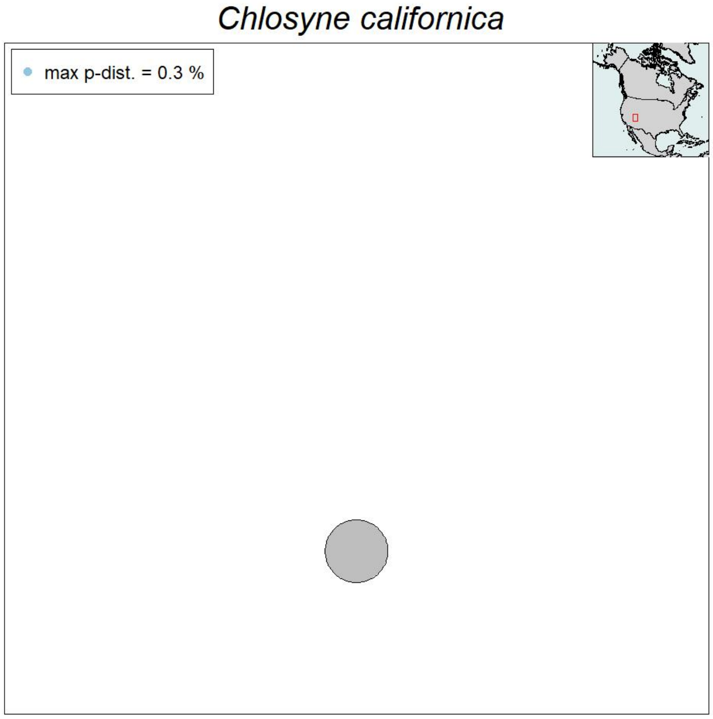

Figure 623: Map of *Chlosyne californica* showing the localities of the sequenced specimens. Nearby localities are grouped in pies. Due to the presence of a single haplotype PCoA projection was not done and a single grey colour was plotted on the map. Sequences= 2; Hap obs.= 2; Hap asympt.= NA; Hap % obs.= NA%; GST= NaN; DST= NaN; HD= NA; ND= NA; max p-dist= 0.3%.

Haplotype network analysis and bubble plot of *Chlosyne californica* were not possible. Sequences > 599 bp = 2.

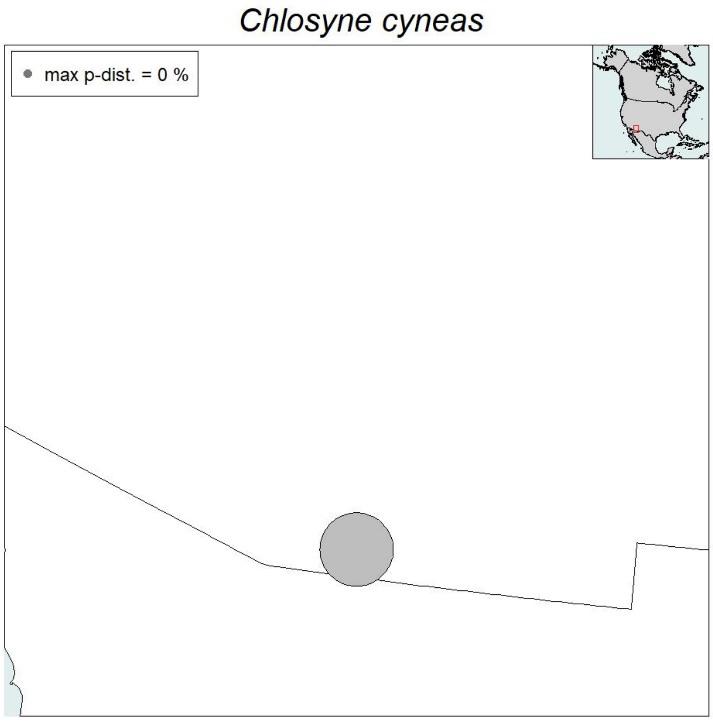

Figure 624: Map of *Chlosyne cyneas* showing the localities of the sequenced specimens. Nearby localities are grouped in pies. Due to the presence of a single haplotype PCoA projection was not done and a single grey colour was plotted on the map. Sequences= 4; Hap obs.= 1; Hap asympt.= NA; Hap % obs.= NA%; GST= NaN; DST= NaN; HD= NA; ND= NA; max p-dist= 0%.

Haplotype network analysis and bubble plot of *Chlosyne cyneas* were not possible. Sequences > 599 bp = 4.

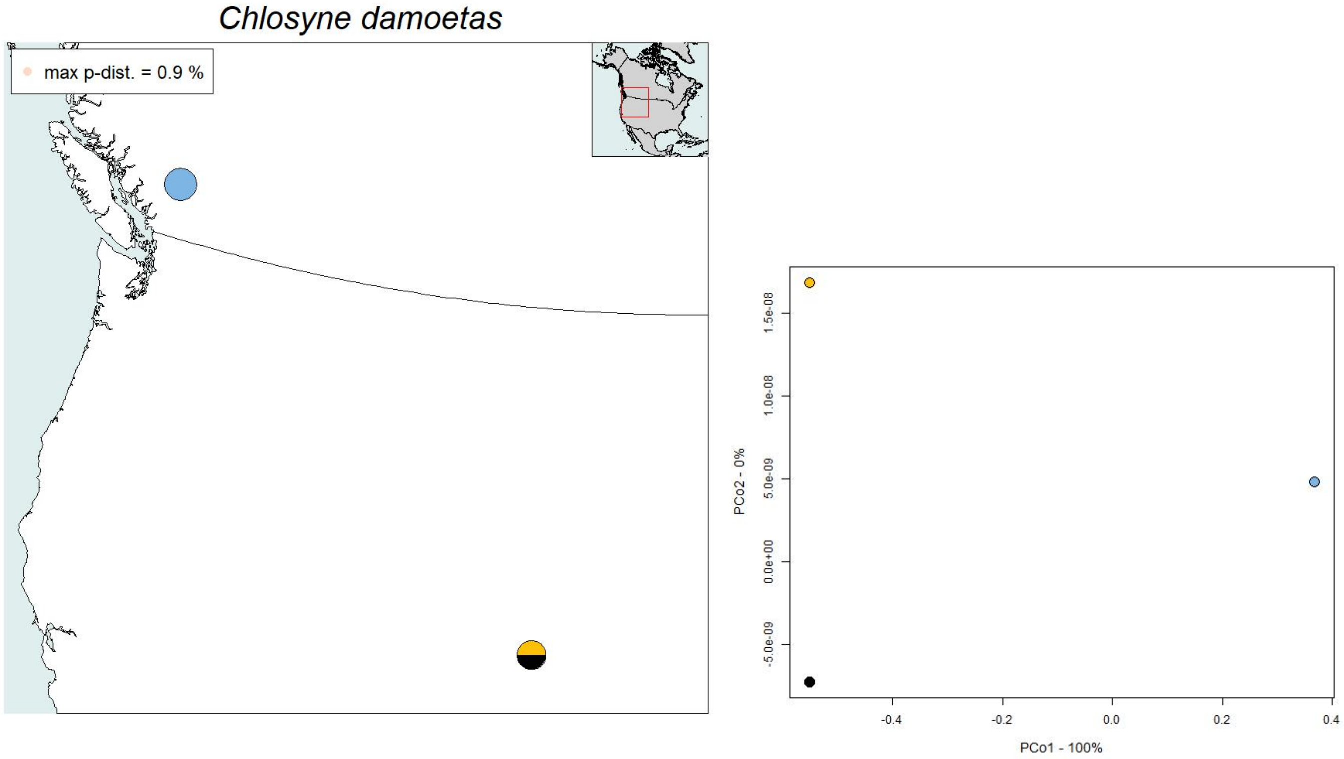

Figure 625 Map of *Chlosyne damoetas* showing the localities of the sequenced specimens (left). Nearby localities are grouped in pies. Colours match the bidimensional colour space of the PCoA projection (right) of max p-dists among sequences (dots). Sequences= 5; Hap obs.= 2; Hap asympt.= NA; Hap % obs.= NA%; GST= NaN; DST= NaN; HD= NA; ND= NA; max p-dist= 0.9%.

Haplotype network analysis and bubble plot of *Chlosyne damoetas* were not possible. Sequences > 599 bp = 5.

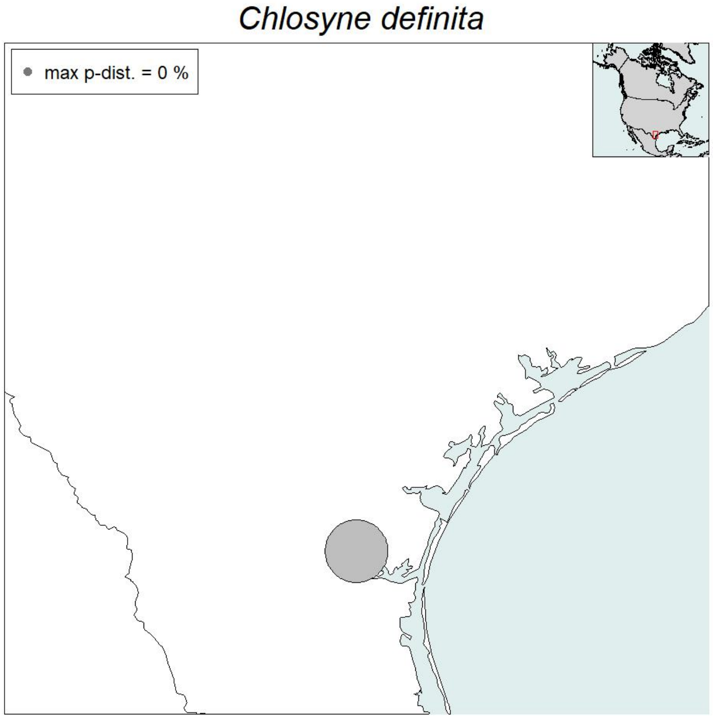

Figure 626: Map of *Chlosyne definita* showing the localities of the sequenced specimens. Nearby localities are grouped in pies. Due to the presence of a single haplotype PCoA projection was not done and a single grey colour was plotted on the map. Sequences= 2; Hap obs.= 1; Hap asympt.= NA; Hap % obs.= NA%; GST= NaN; DST= NaN; HD= NA; ND= NA; max p-dist= 0%.

Haplotype network analysis and bubble plot of *Chlosyne definita* were not possible. Sequences > 599 bp = 2.

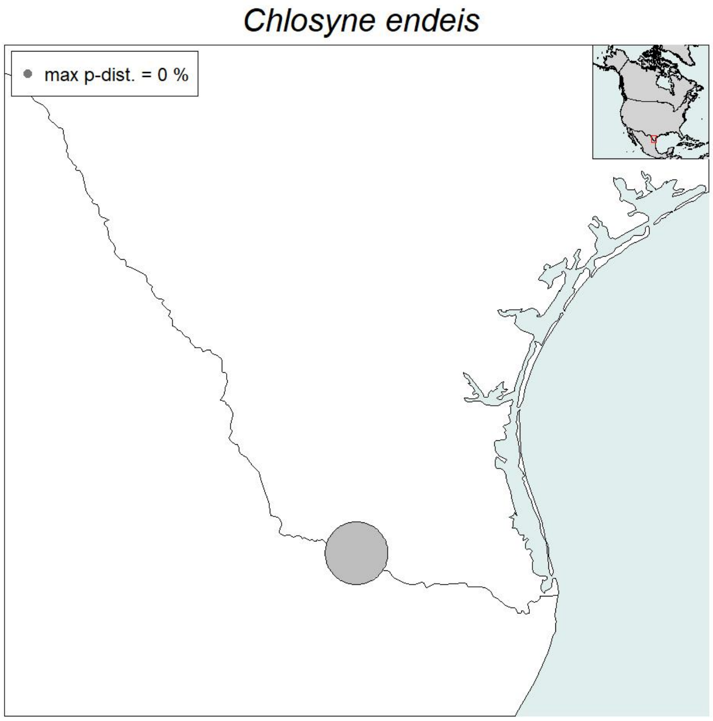

Figure 627: Map of *Chlosyne endeis* showing the localities of the sequenced specimens. Nearby localities are grouped in pies. Due to the presence of a single haplotype PCoA projection was not done and a single grey colour was plotted on the map. Sequences= 2; Hap obs.= 1; Hap asympt.= NA; Hap % obs.= NA%; GST= NaN; DST= NaN; HD= NA; ND= NA; max p-dist= 0%.

Haplotype network analysis and bubble plot of *Chlosyne endeis* were not possible. Sequences > 599 bp = 2.

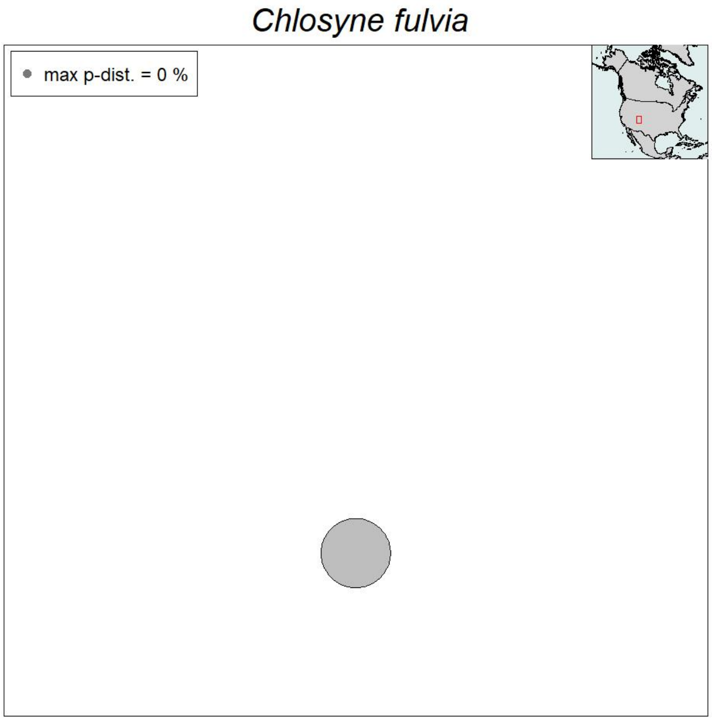

Figure 628: Map of *Chlosyne fulvia* showing the localities of the sequenced specimens. Nearby localities are grouped in pies. Due to the presence of a single haplotype PCoA projection was not done and a single grey colour was plotted on the map. Sequences= 3; Hap obs.= 1; Hap asympt.= NA; Hap % obs.= NA%; GST= NaN; DST= NaN; HD= NA; ND= NA; max p-dist= 0%.

Haplotype network analysis and bubble plot of *Chlosyne fulvia* were not possible. Sequences > 599 bp = 3.

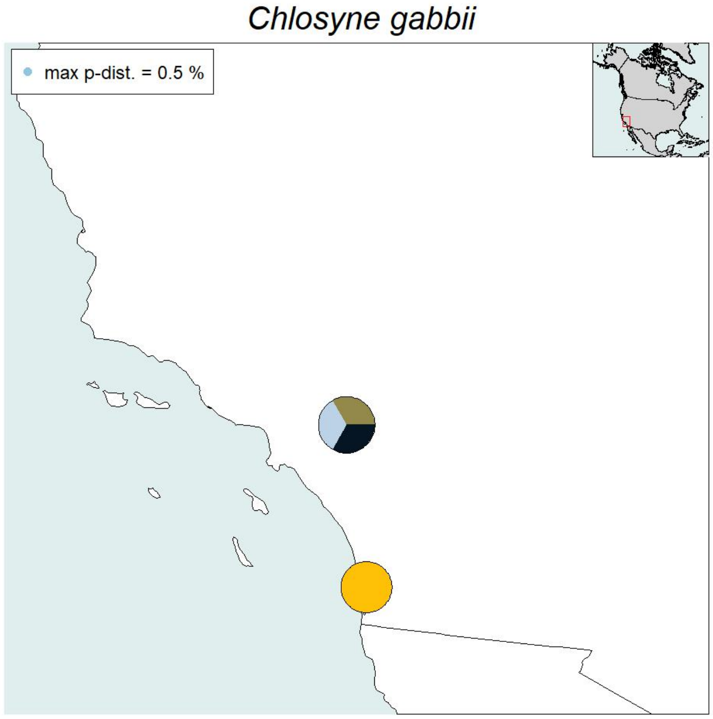

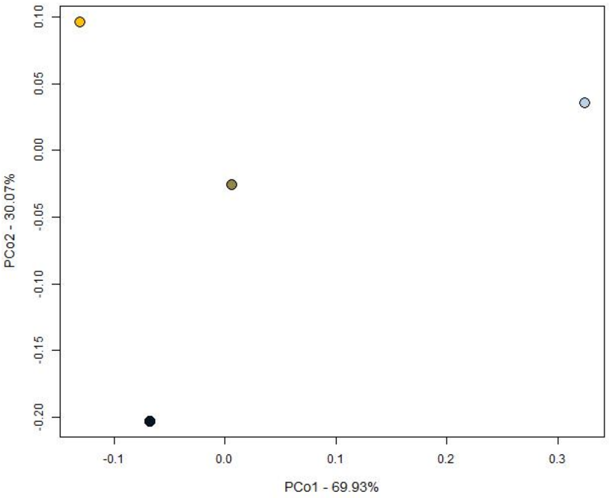

Figure 629 Map of *Chlosyne gabbii* showing the localities of the sequenced specimens (left). Nearby localities are grouped in pies. Colours match the bidimensional colour space of the PCoA projection (right) of max p-dists among sequences (dots). Sequences= 5; Hap obs.= 4; Hap asympt.= NA; Hap % obs.= NA%; GST= NaN; DST= NaN; HD= NA; ND= NA; max p-dist= 0.5%.

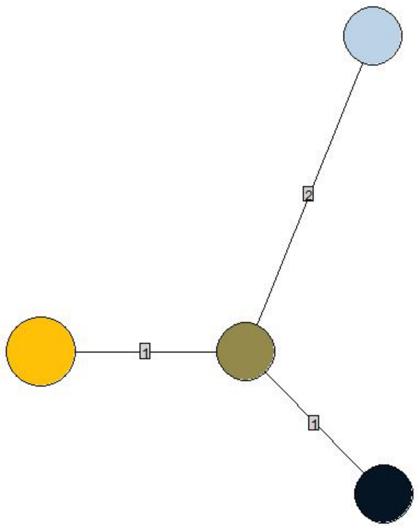

Figure 630: Haplotype network of *Chlosyne gabbii*. Sequences > 599 bp= 5.

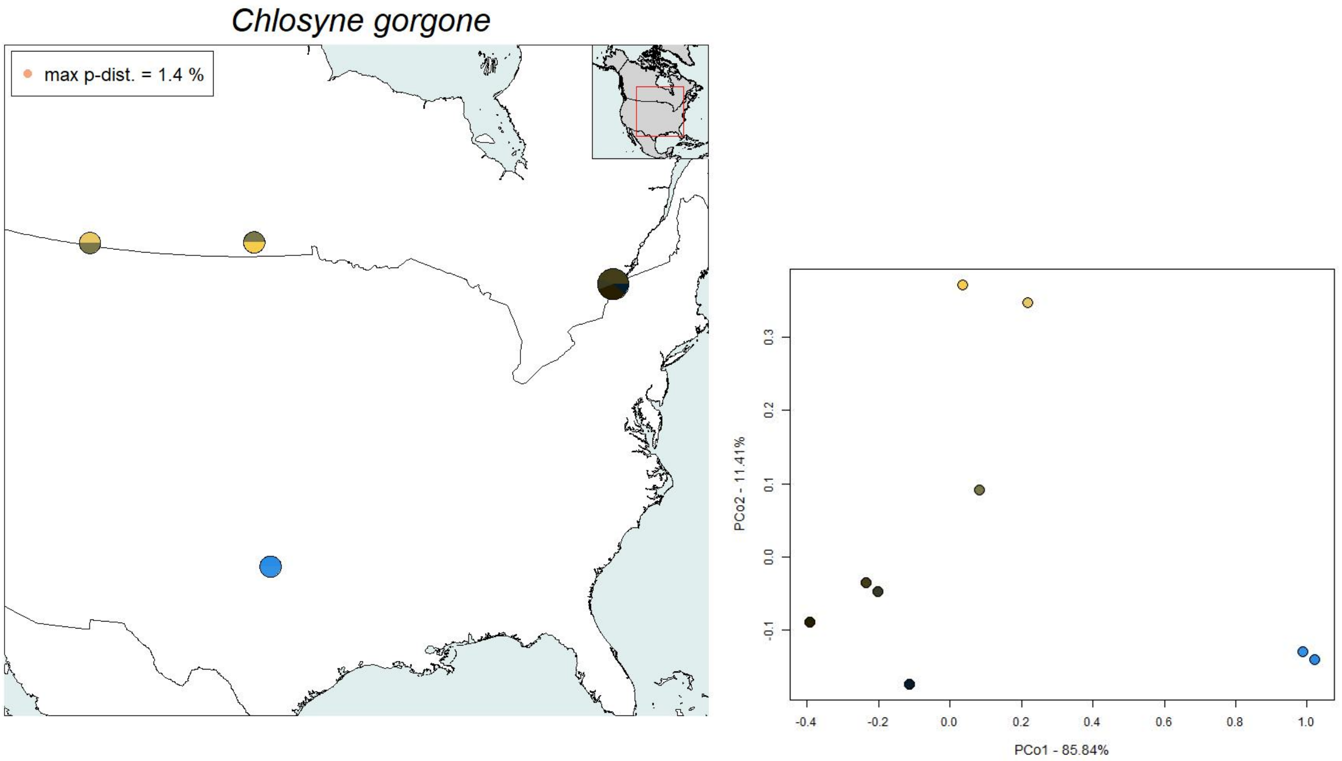

Figure 631 Map of *Chlosyne gorgone* showing the localities of the sequenced specimens (left). Nearby localities are grouped in pies. Colours match the bidimensional colour space of the PCoA projection (right) of max p-dists among sequences (dots). Sequences= 15; Hap obs.= 6; Hap asympt.= 6.9; Hap % obs.= 86.5%; GST= NaN; DST= NaN; HD= 0.81; ND= 0.0049; max p-dist= 1.4%.

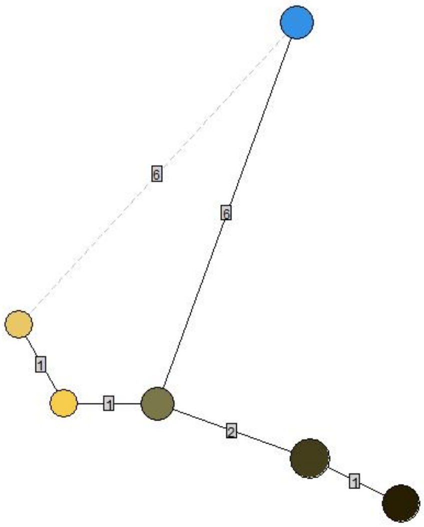

Figure 632: Haplotype network of *Chlosyne gorgone*. Sequences > 599 bp= 13.

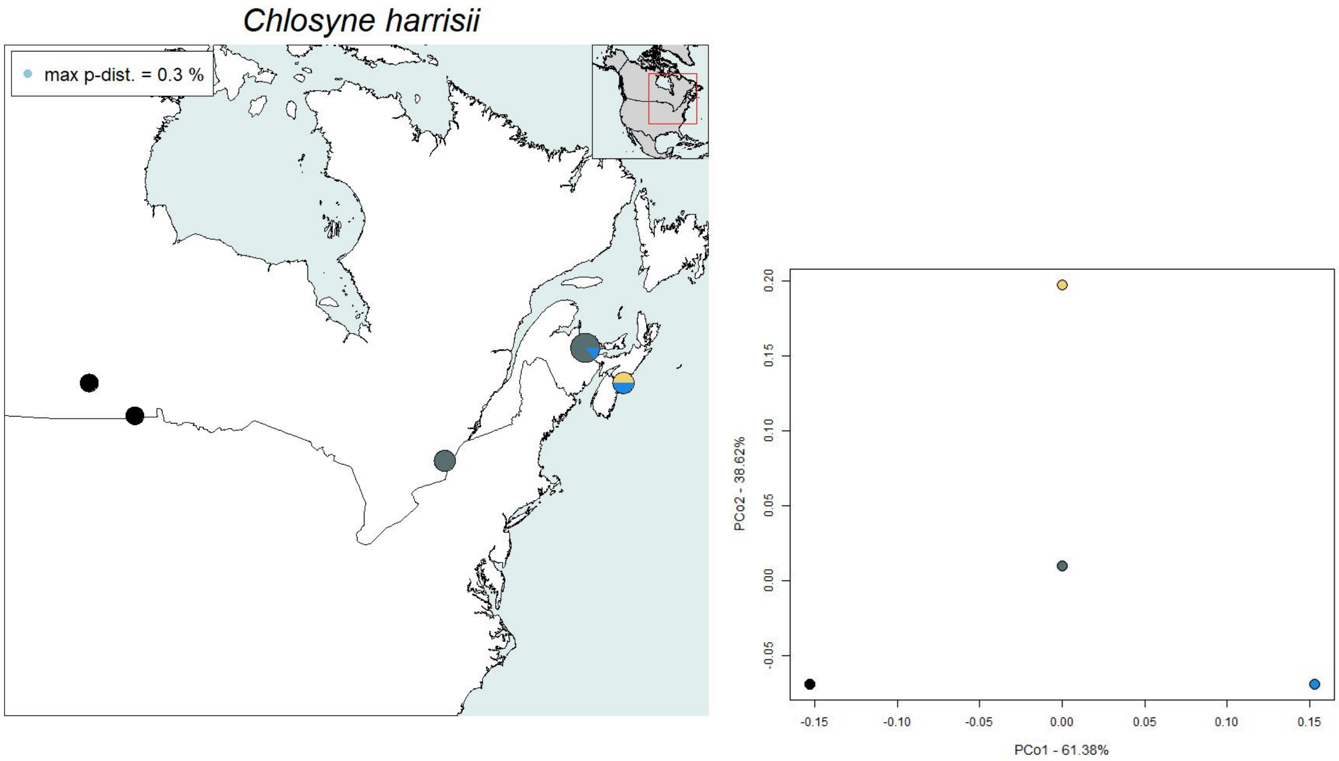

Figure 633 Map of *Chlosyne harrisii* showing the localities of the sequenced specimens (left). Nearby localities are grouped in pies. Colours match the bidimensional colour space of the PCoA projection (right) of max p-dists among sequences (dots). Sequences= 13; Hap obs.= 4; Hap asympt.= 4.2; Hap % obs.= 94.5%; GST= NaN; DST= NaN; HD= 0.615; ND= 0.0011; max p-dist= 0.3%.

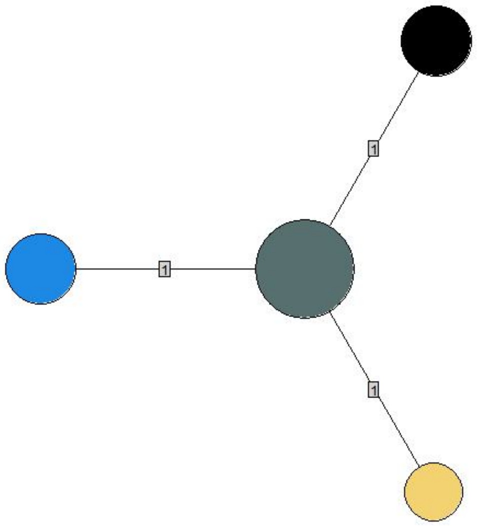

Figure 634: Haplotype network of *Chlosyne harrisii*. Sequences > 599 bp= 13.

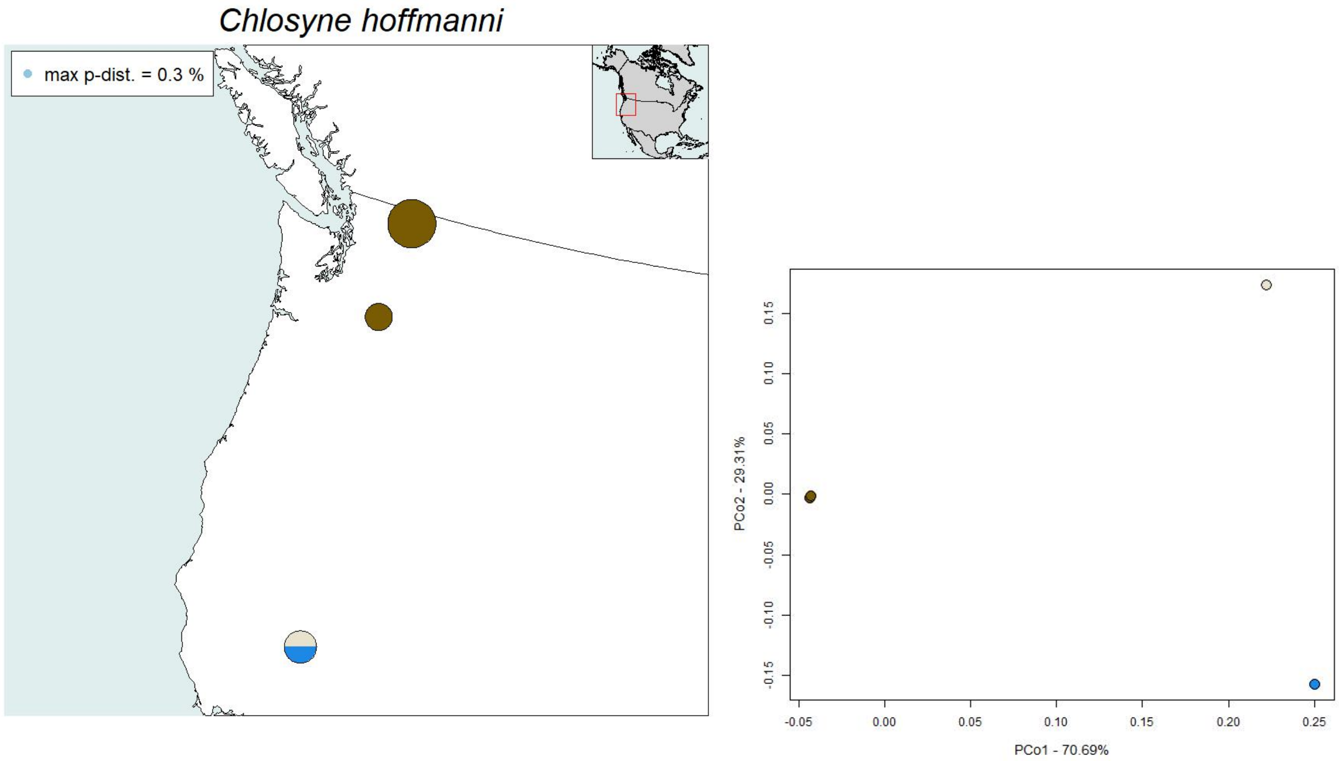

Figure 635 Map of *Chlosyne hoffmanni* showing the localities of the sequenced specimens (left). Nearby localities are grouped in pies. Colours match the bidimensional colour space of the PCoA projection (right) of max p-dists among sequences (dots). Sequences= 13; Hap obs.= 3; Hap asympt.= 3.9; Hap % obs.= 76.5%; GST= NaN; DST= NaN; HD= 0.295; ND= 0.001; max p-dist= 0.3%.

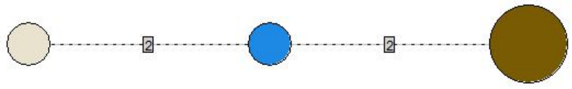

Figure 636: Haplotype network of *Chlosyne hoffmanni*. Sequences > 599 bp= 13.

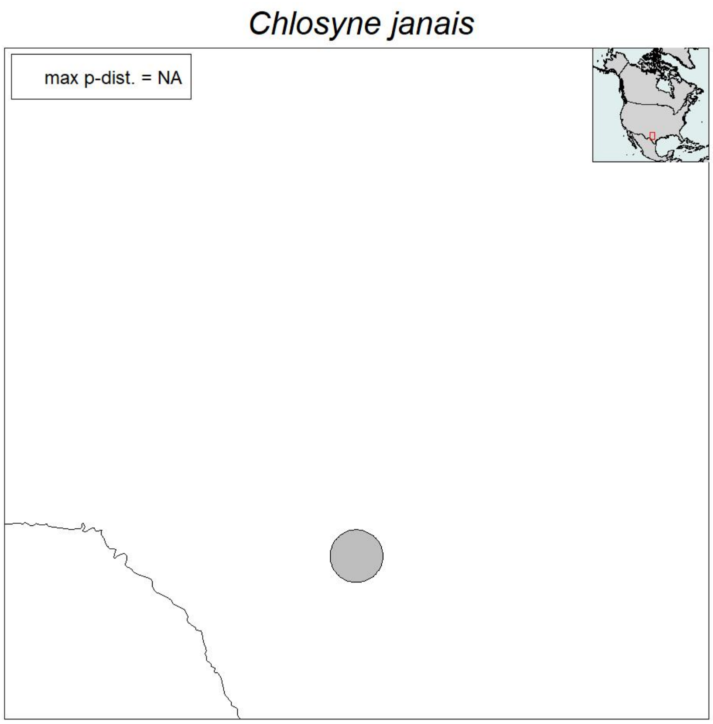

Figure 637: Map of *Chlosyne janais* showing the localities of the sequenced specimens. Nearby localities are grouped in pies. Due to the presence of a single haplotype PCoA projection was not done and a single grey colour was plotted on the map. Sequences= 1; Hap obs.= NA; Hap asympt.= NA; Hap % obs.= NA; GST= NaN; DST= NaN; HD= NA; ND= NA; max p-dist= NA.

Haplotype network analysis and bubble plot of *Chlosyne janais* were not possible. Sequences > 599 bp = 1.

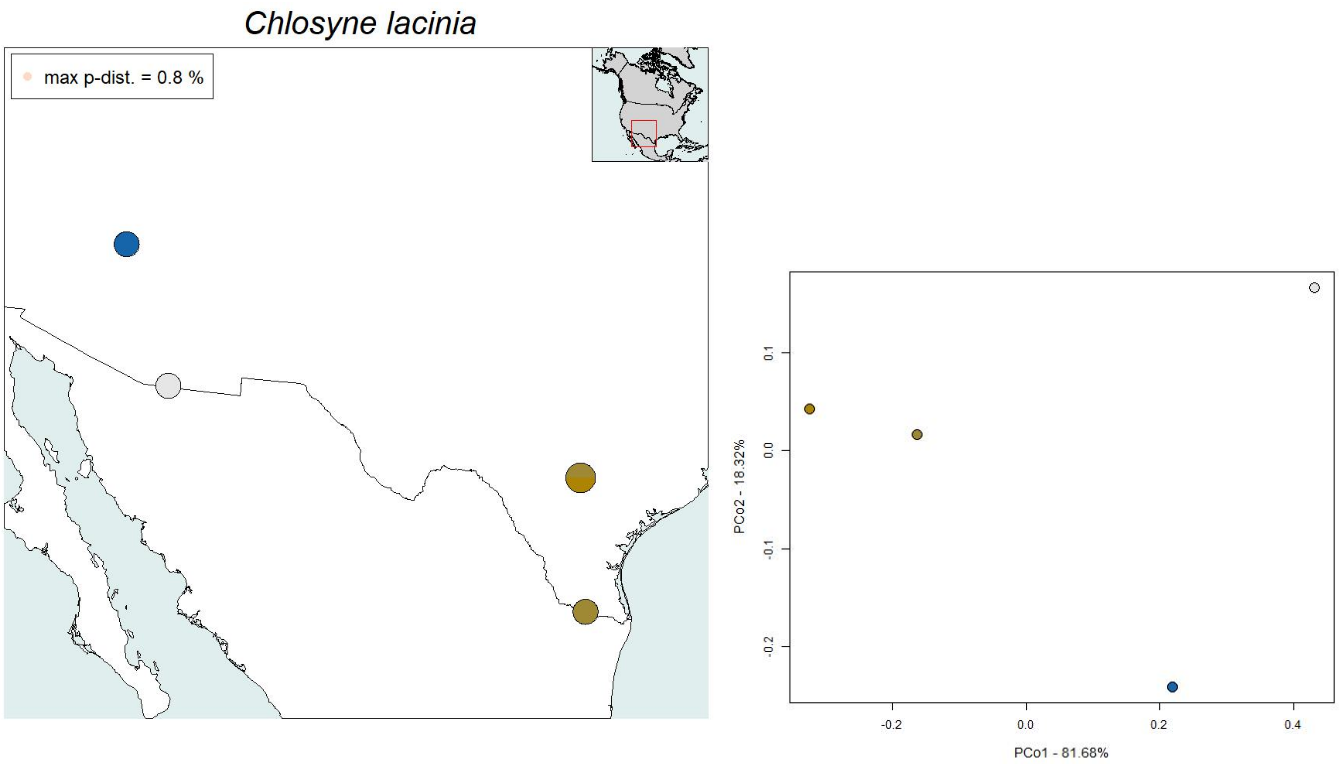

Figure 638 Map of *Chlosyne lacinia* showing the localities of the sequenced specimens (left). Nearby localities are grouped in pies. Colours match the bidimensional colour space of the PCoA projection (right) of max p-dists among sequences (dots). Sequences= 5; Hap obs.= 4; Hap asympt.= NA; Hap % obs.= NA%; GST= NaN; DST= NaN; HD= NA; ND= NA; max p-dist= 0.8%.

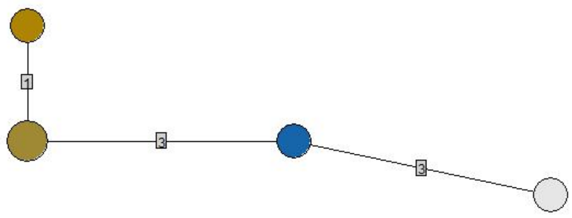

Figure 639: Haplotype network of *Chlosyne lacinia*. Sequences > 599 bp= 5.

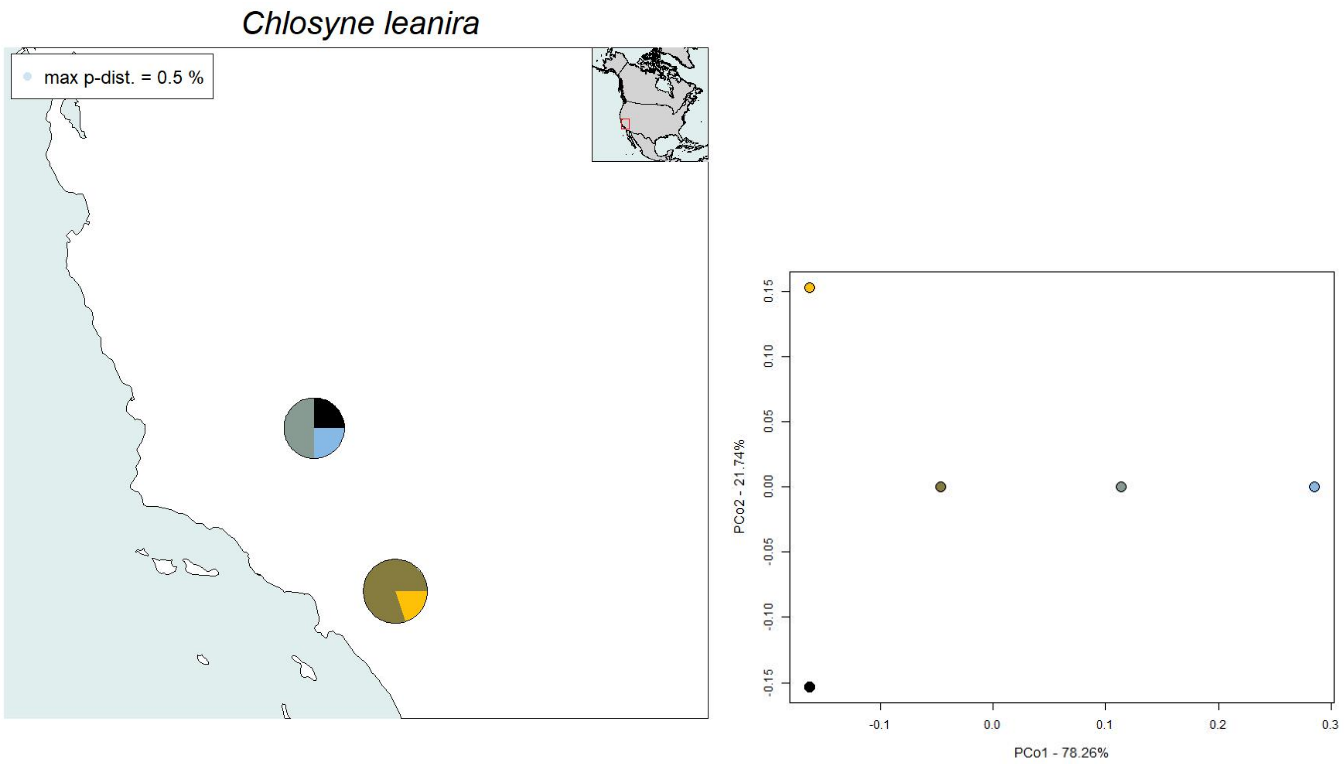

Figure 640 Map of *Chlosyne leanira* showing the localities of the sequenced specimens (left). Nearby localities are grouped in pies. Colours match the bidimensional colour space of the PCoA projection (right) of max p-dists among sequences (dots). Sequences= 9; Hap obs.= 5; Hap asympt.= NA; Hap % obs.= NA%; GST= NaN; DST= NaN; HD= NA; ND= NA; max p-dist= 0.5%.

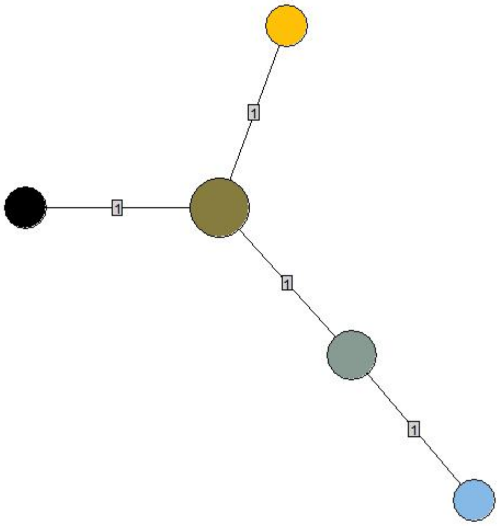

Figure 641: Haplotype network of *Chlosyne leanira*. Sequences > 599 bp= 9.

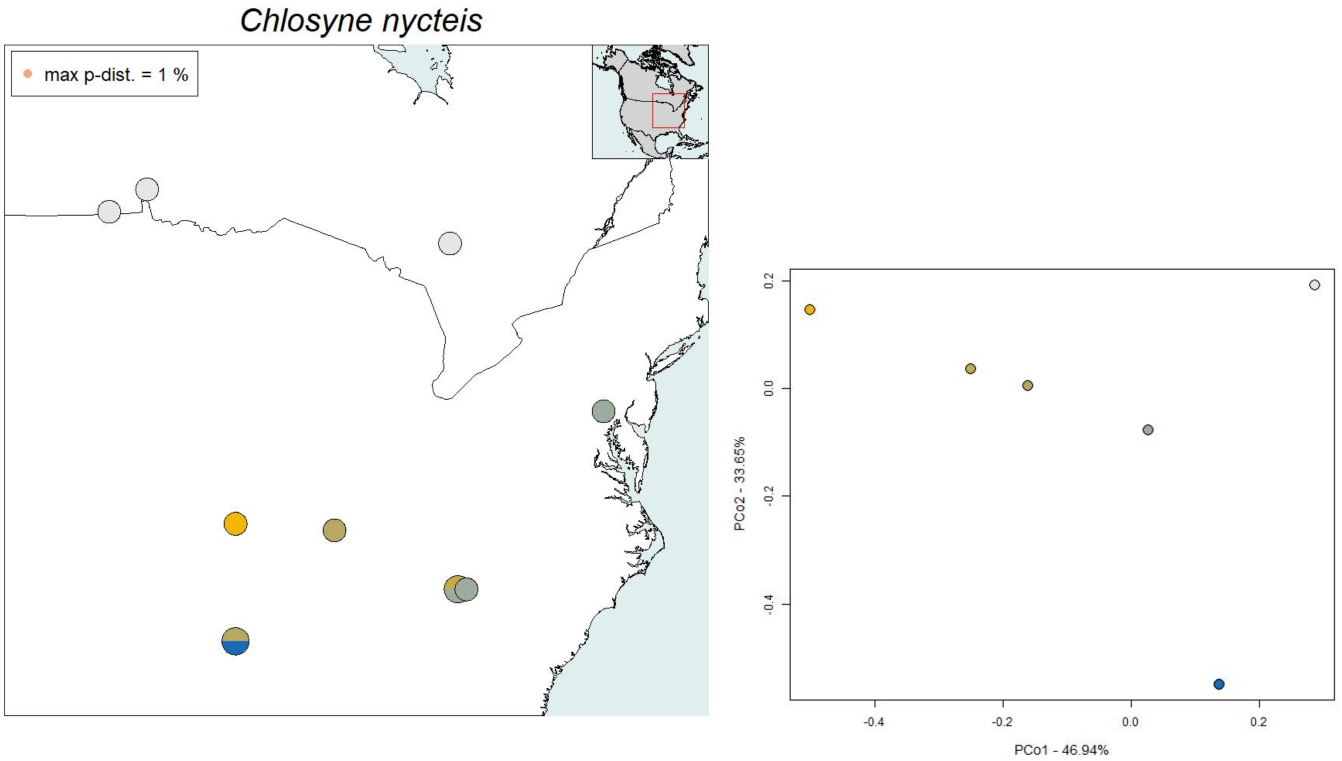

Figure 642 Map of *Chlosyne nycteis* showing the localities of the sequenced specimens (left). Nearby localities are grouped in pies. Colours match the bidimensional colour space of the PCoA projection (right) of max p-dists among sequences (dots). Sequences= 11; Hap obs.= 6; Hap asympt.= 10.1; Hap % obs.= 59.5%; GST= NaN; DST= NaN; HD= 0.873; ND= 0.0045; max p-dist= 1%.

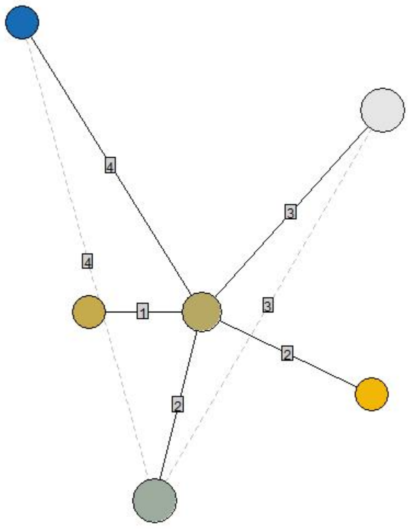

Figure 643: Haplotype network of *Chlosyne nycteis*. Sequences > 599 bp= 11.

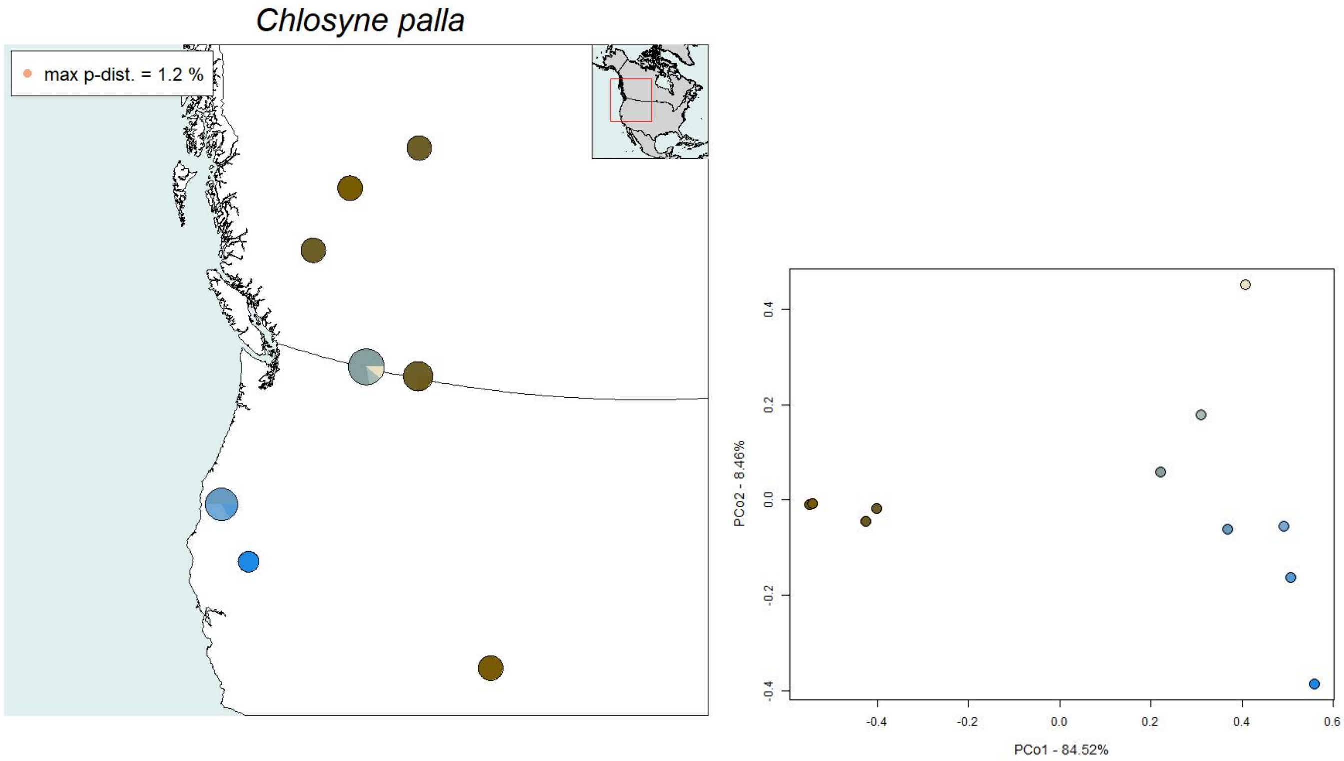

Figure 644 Map of *Chlosyne palla* showing the localities of the sequenced specimens (left). Nearby localities are grouped in pies. Colours match the bidimensional colour space of the PCoA projection (right) of max p-dists among sequences (dots). Sequences= 28; Hap obs.= 12; Hap asympt.= 42.9; Hap % obs.= 28%; GST= 0.666; DST= 0.0027; HD= 0.86; ND= 0.005; max p-dist= 1.2%.

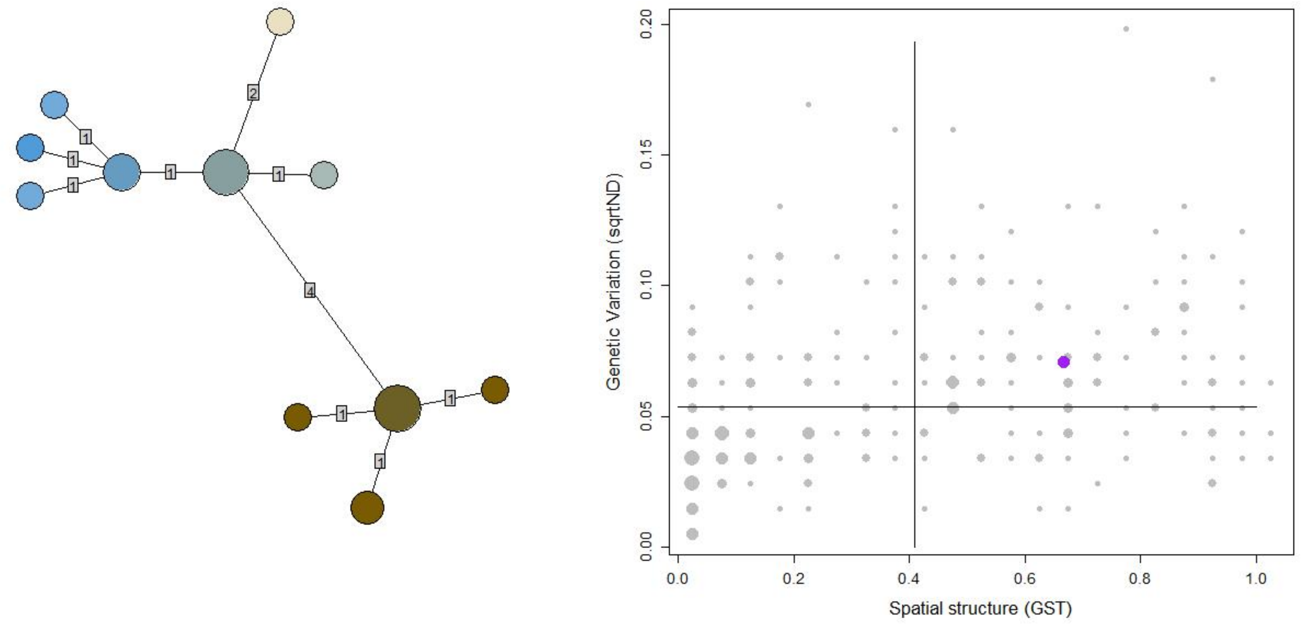

Figure 645: Haplotype network (left) of *Chlosyne palla* sequences > 599 bp with colours matching the PCoA colour space (above). The bubble plot for mt-DNA polymorphism (square root transformed nucleotide diversity) and spatial structure (GST) among all species in the atlas and values for *Chlosyne palla* (purple dot). The horizontal and vertical lines represent median values of nucleotide diversity and GST, respectively. Sequences > 599 bp= 27.

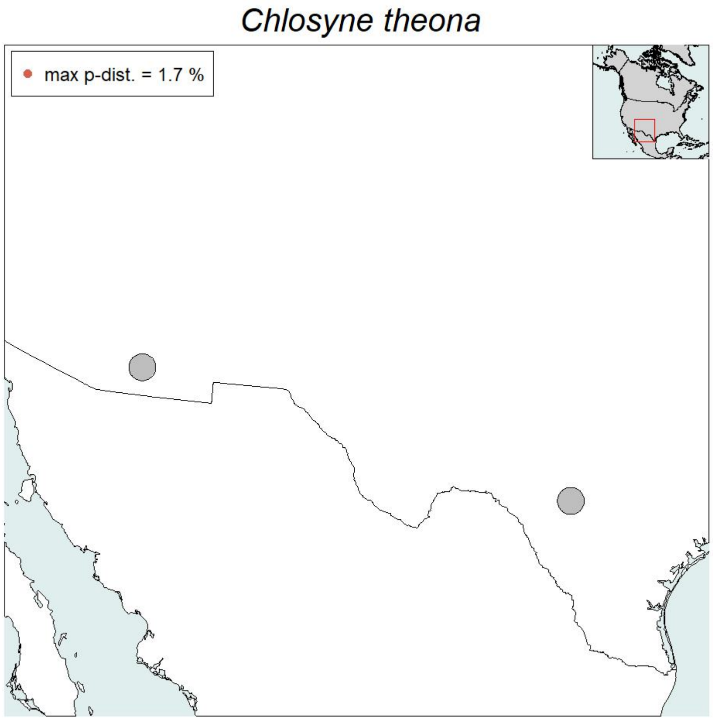

Figure 646: Map of *Chlosyne theona* showing the localities of the sequenced specimens. Nearby localities are grouped in pies. Due to the presence of a single haplotype PCoA projection was not done and a single grey colour was plotted on the map. Sequences= 2; Hap obs.= 2; Hap asympt.= NA; Hap % obs.= NA%; GST= NaN; DST= NaN; HD= NA; ND= NA; max p-dist= 1.7%.

Haplotype network analysis and bubble plot of *Chlosyne theona* were not possible. Sequences > 599 bp = 2.

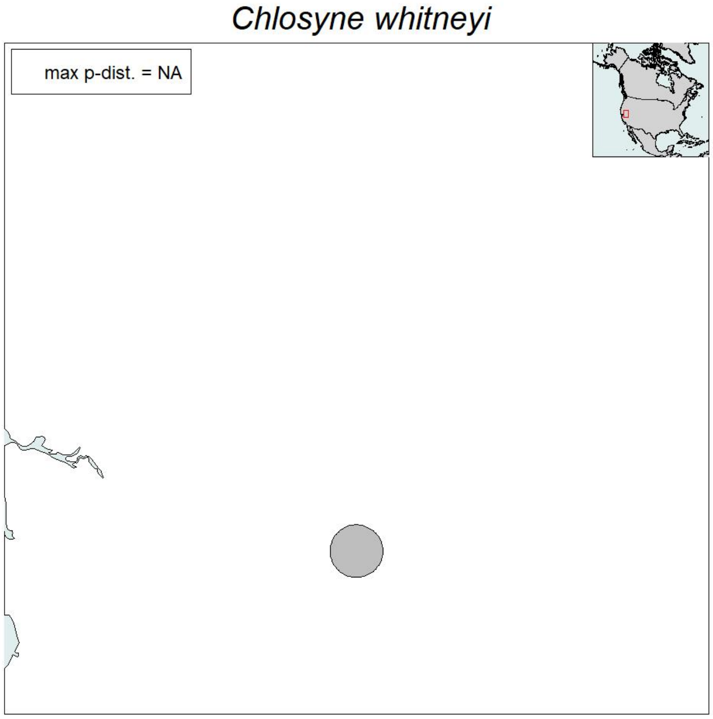

Figure 647: Map of *Chlosyne whitneyi* showing the localities of the sequenced specimens. Nearby localities are grouped in pies. Due to the presence of a single haplotype PCoA projection was not done and a single grey colour was plotted on the map. Sequences= 1; Hap obs.= NA; Hap asympt.= NA; Hap % obs.= NA; GST= NaN; DST= NaN; HD= NA; ND= NA; max p-dist= NA.

Haplotype network analysis and bubble plot of *Chlosyne whitneyi* were not possible. Sequences > 599 bp = 1.

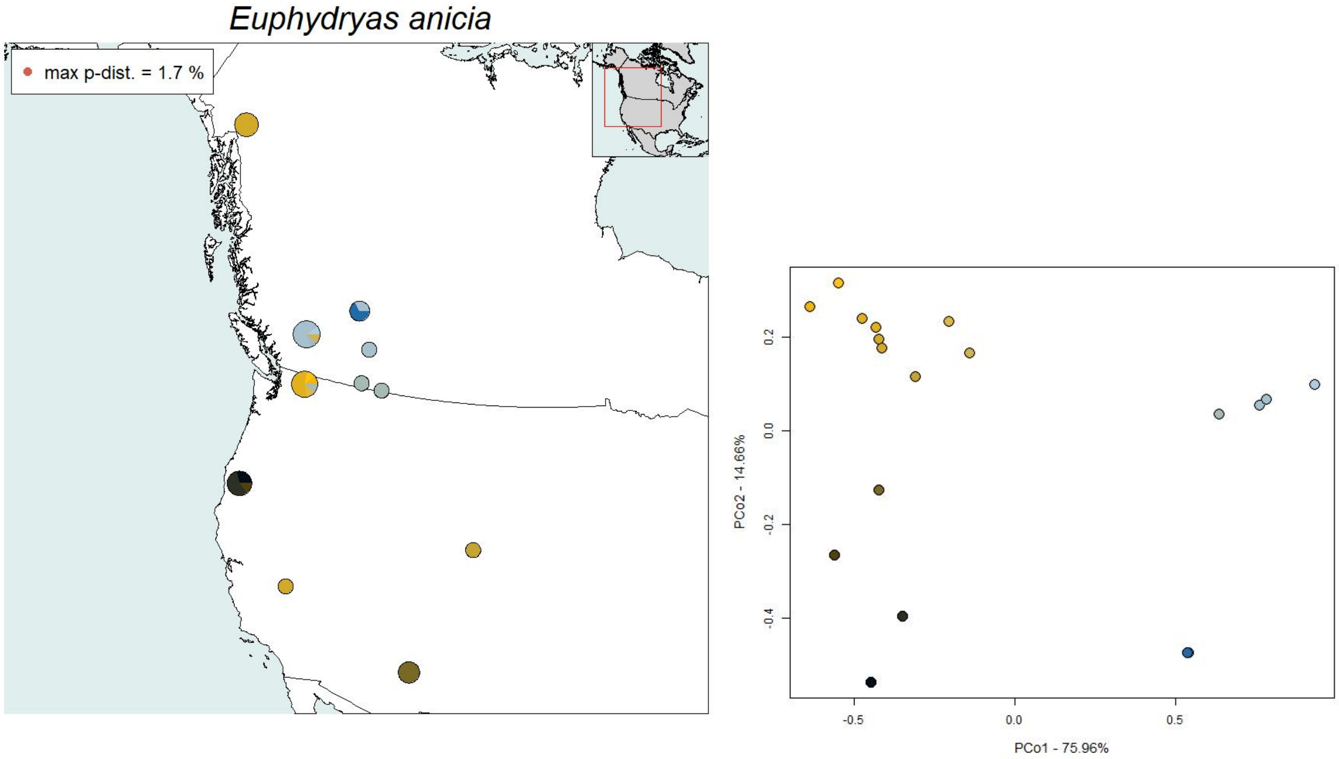

Figure 648 Map of *Euphydryas anicia* showing the localities of the sequenced specimens (left). Nearby localities are grouped in pies. Colours match the bidimensional colour space of the PCoA projection (right) of max p-dists among sequences (dots). Sequences= 44; Hap obs.= 18; Hap asympt.= 42.4; Hap % obs.= 42.4%; GST= 0.704; DST= 0.0053; HD= 0.93; ND= 0.0075; max p-dist= 1.7%.

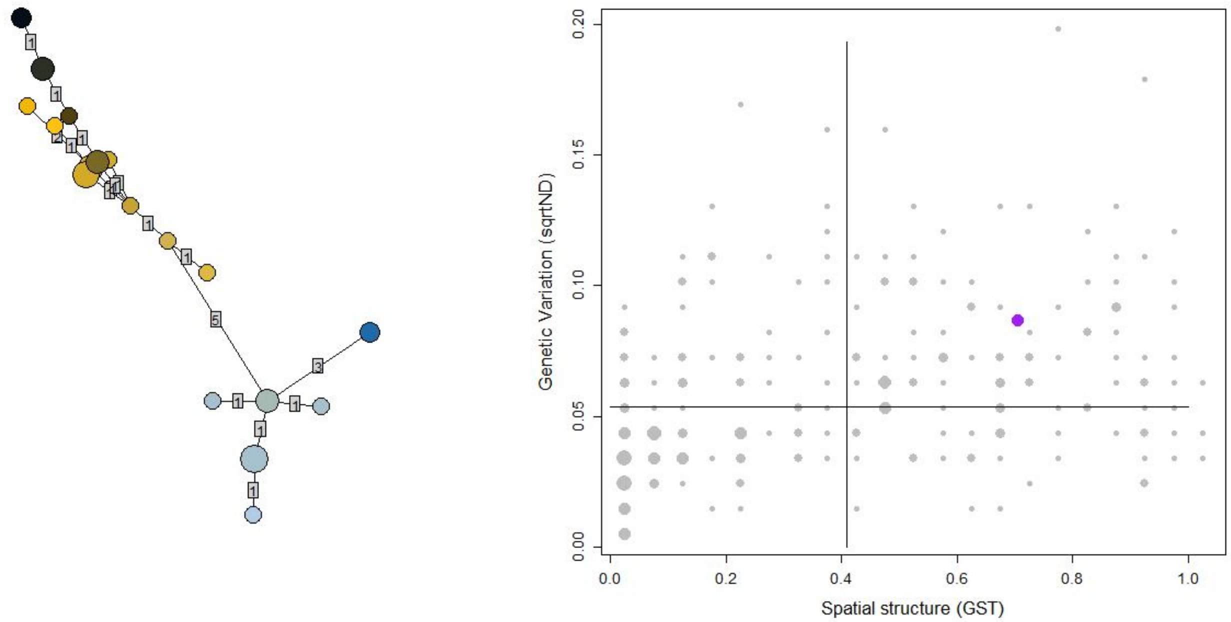

Figure 649: Haplotype network (left) of *Euphydryas anicia* sequences > 599 bp with colours matching the PCoA colour space (above). The bubble plot for mt-DNA polymorphism (square root transformed nucleotide diversity) and spatial structure (GST) among all species in the atlas and values for *Euphydryas anicia* (purple dot). The horizontal and vertical lines represent median values of nucleotide diversity and GST, respectively. Sequences > 599 bp= 44.

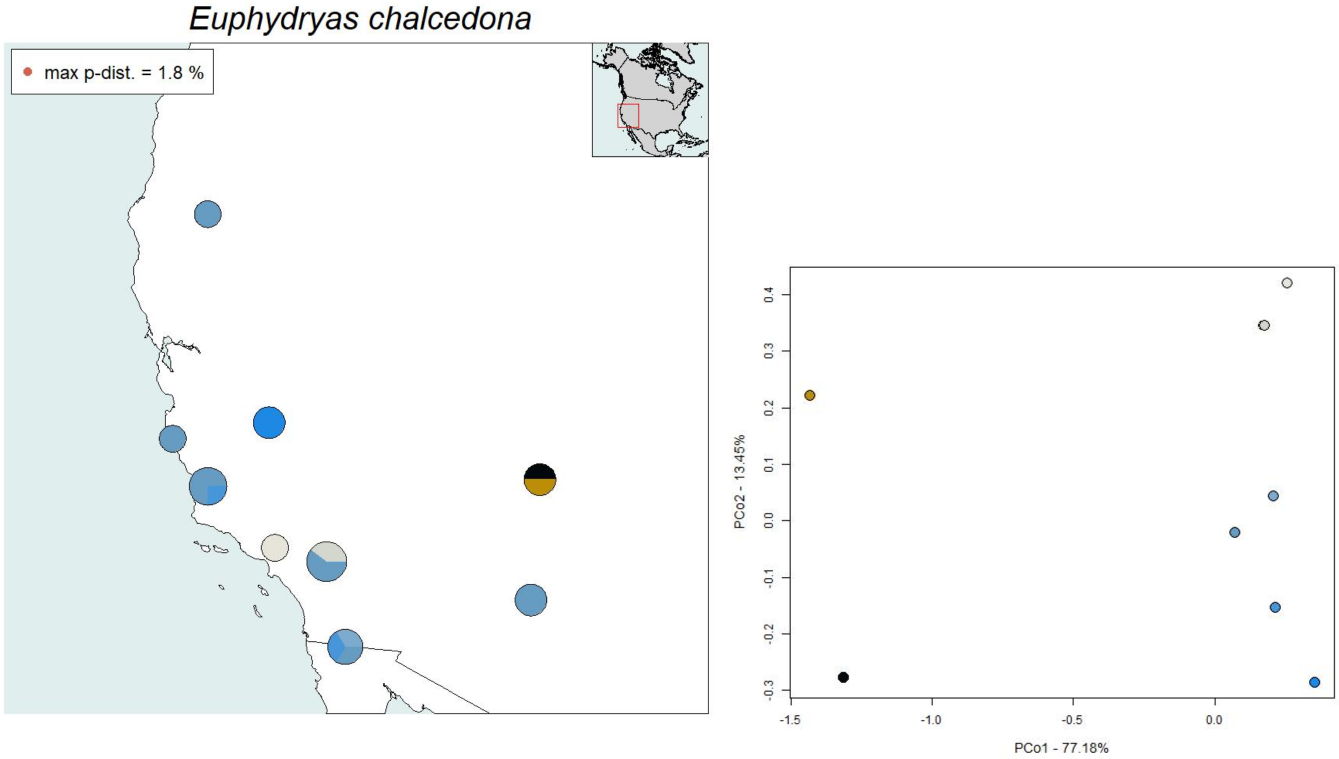

Figure 650 Map of *Euphydryas chalcedona* showing the localities of the sequenced specimens (left). Nearby localities are grouped in pies. Colours match the bidimensional colour space of the PCoA projection (right) of max p-dists among sequences (dots). Sequences= 21; Hap obs.= 8; Hap asympt.= 10.5; Hap % obs.= 75.9%; GST= 0.107; DST= 0.0002; HD= 0.724; ND= 0.0046; max p-dist= 1.8%.

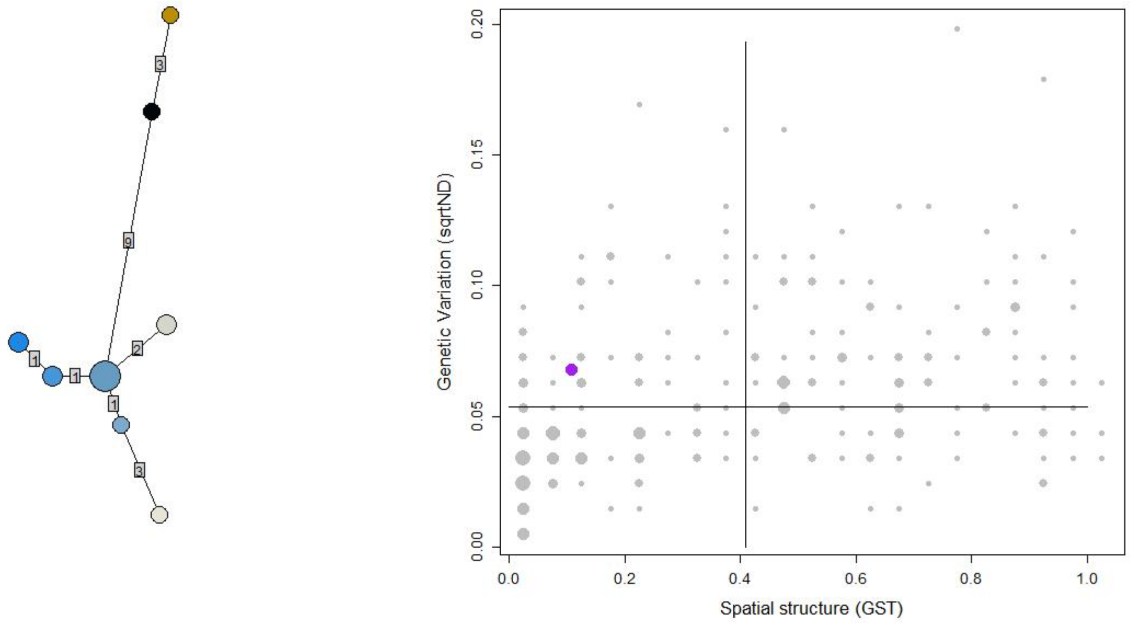

Figure 651: Haplotype network (left) of *Euphydryas chalcedona* sequences > 599 bp with colours matching the PCoA colour space (above). The bubble plot for mt-DNA polymorphism (square root transformed nucleotide diversity) and spatial structure (GST) among all species in the atlas and values for *Euphydryas chalcedona* (purple dot). The horizontal and vertical lines represent median values of nucleotide diversity and GST, respectively. Sequences > 599 bp= 21.

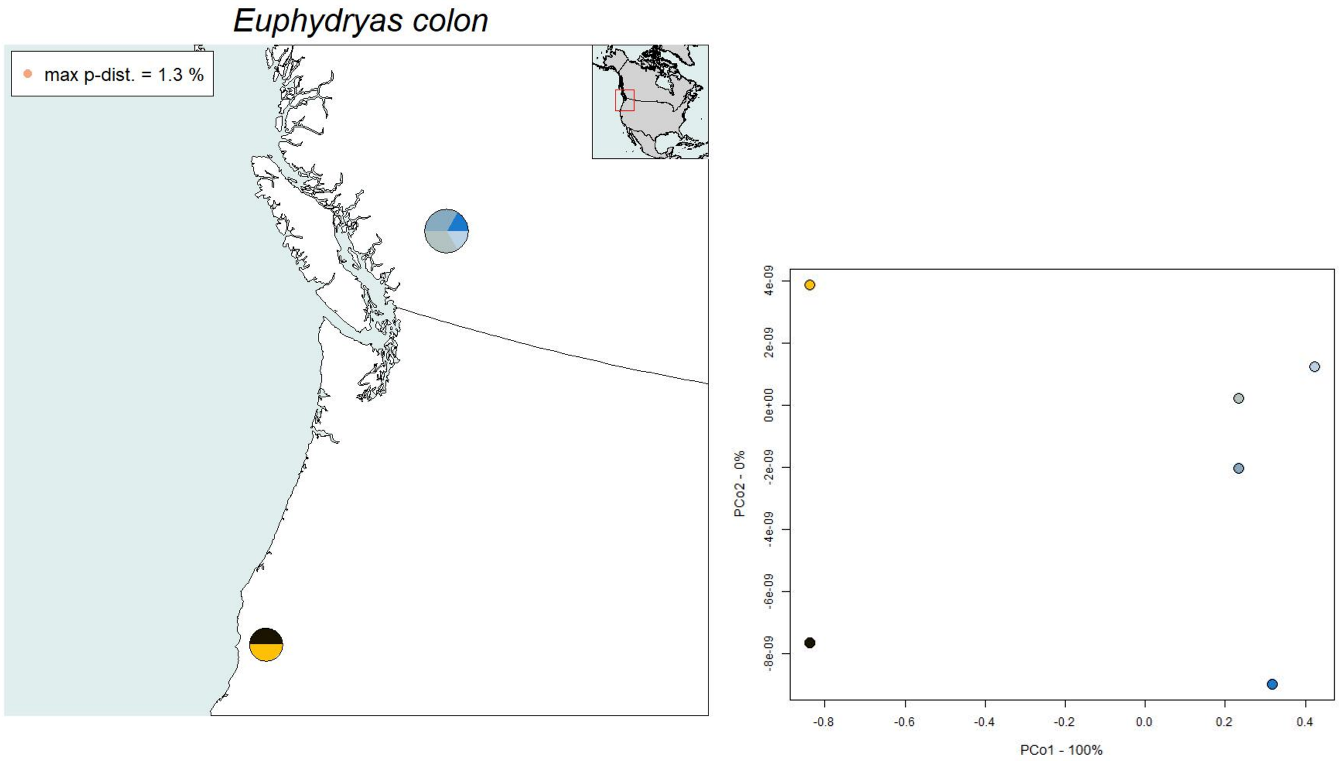

Figure 652 Map of *Euphydryas colon* showing the localities of the sequenced specimens (left). Nearby localities are grouped in pies. Colours match the bidimensional colour space of the PCoA projection (right) of max p-dists among sequences (dots). Sequences= 8; Hap obs.= 2; Hap asympt.= NA; Hap % obs.= NA%; GST= NaN; DST= NaN; HD= NA; ND= NA; max p-dist= 1.3%.

Haplotype network analysis and bubble plot of *Euphydryas colon* were not possible. Sequences > 599 bp = 7.

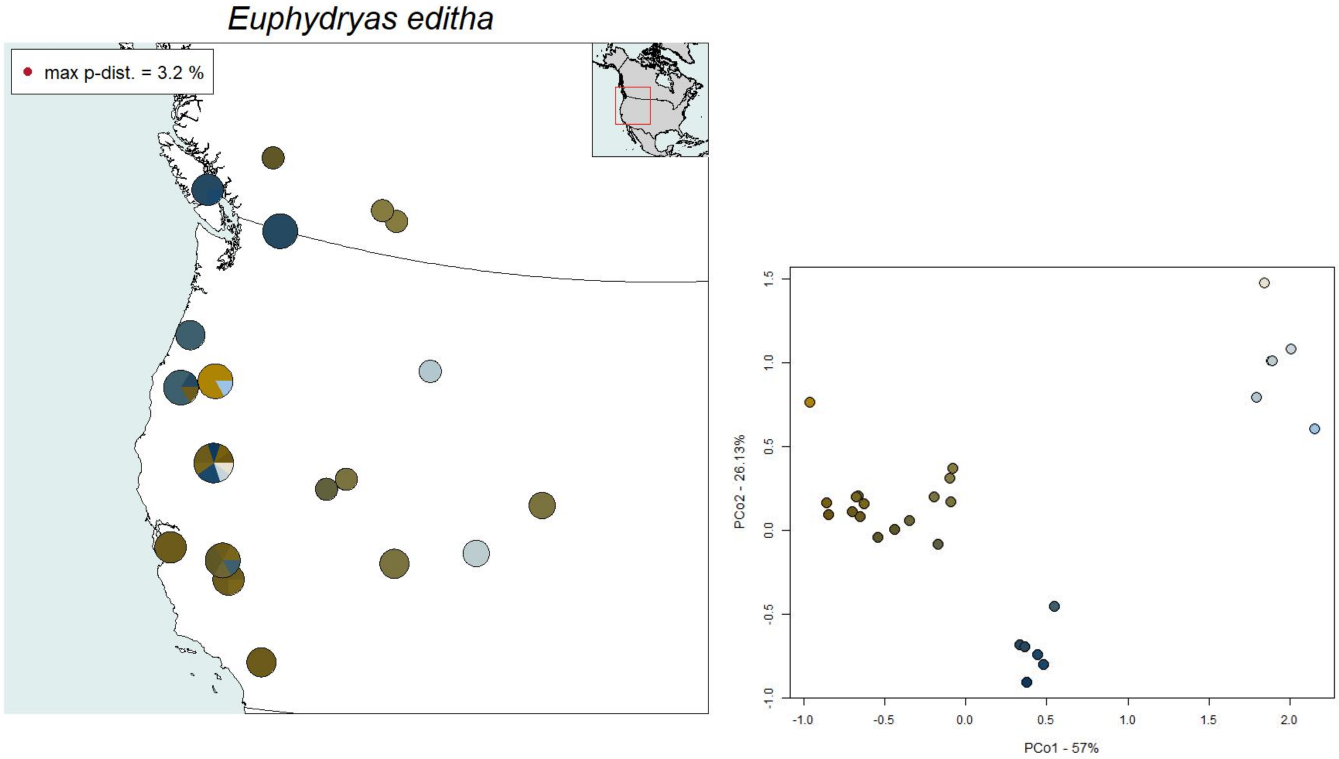

Figure 653 Map of *Euphydryas editha* showing the localities of the sequenced specimens (left). Nearby localities are grouped in pies. Colours match the bidimensional colour space of the PCoA projection (right) of max p-dists among sequences (dots). Sequences= 65; Hap obs.= 18; Hap asympt.= 24; Hap % obs.= 74.9%; GST= 0.27; DST= 0.0031; HD= 0.884; ND= 0.0123; max p-dist= 3.2%.

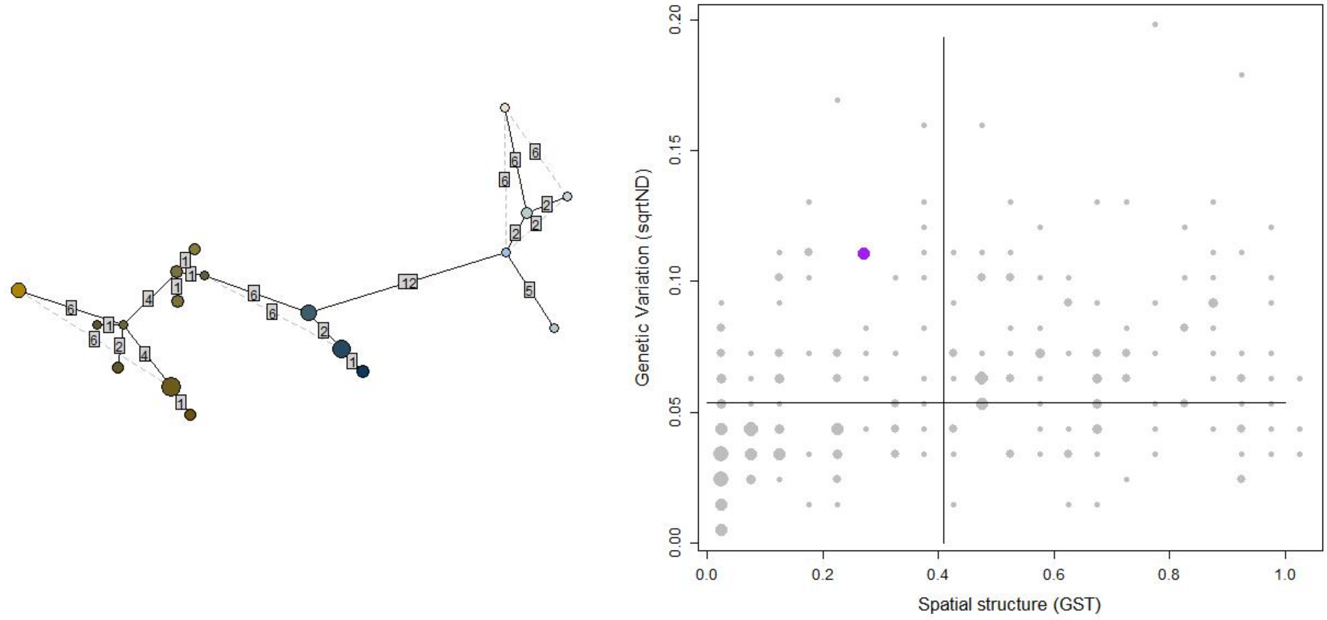

Figure 654: Haplotype network (left) of *Euphydryas editha* sequences > 599 bp with colours matching the PCoA colour space (above). The bubble plot for mt-DNA polymorphism (square root transformed nucleotide diversity) and spatial structure (GST) among all species in the atlas and values for *Euphydryas editha* (purple dot). The horizontal and vertical lines represent median values of nucleotide diversity and GST, respectively. Sequences > 599 bp= 65.

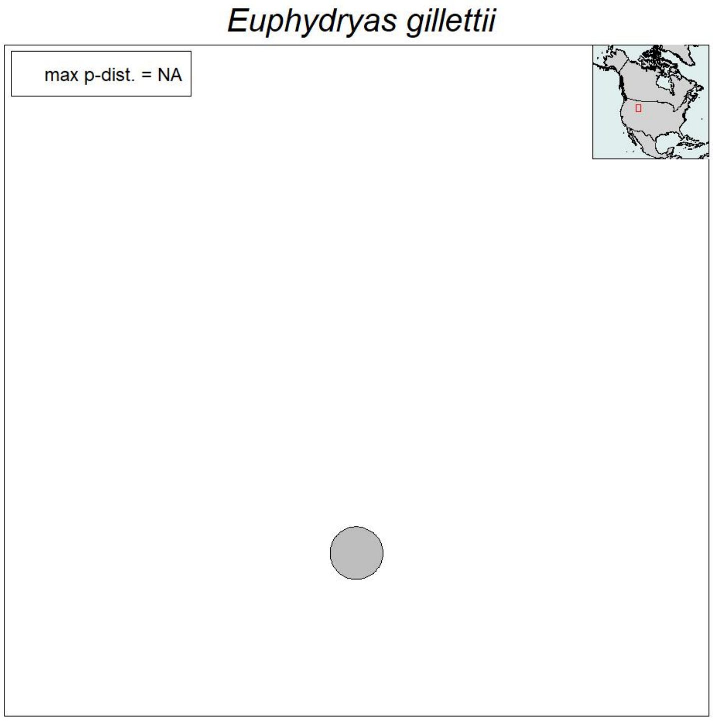

Figure 655: Map of *Euphydryas gillettii* showing the localities of the sequenced specimens. Nearby localities are grouped in pies. Due to the presence of a single haplotype PCoA projection was not done and a single grey colour was plotted on the map. Sequences= 1; Hap obs.= NA; Hap asympt.= NA; Hap % obs.= NA; GST= NaN; DST= NaN; HD= NA; ND= NA; max p-dist= NA.

Haplotype network analysis and bubble plot of *Euphydryas gillettii* were not possible. Sequences > 599 bp = 1.

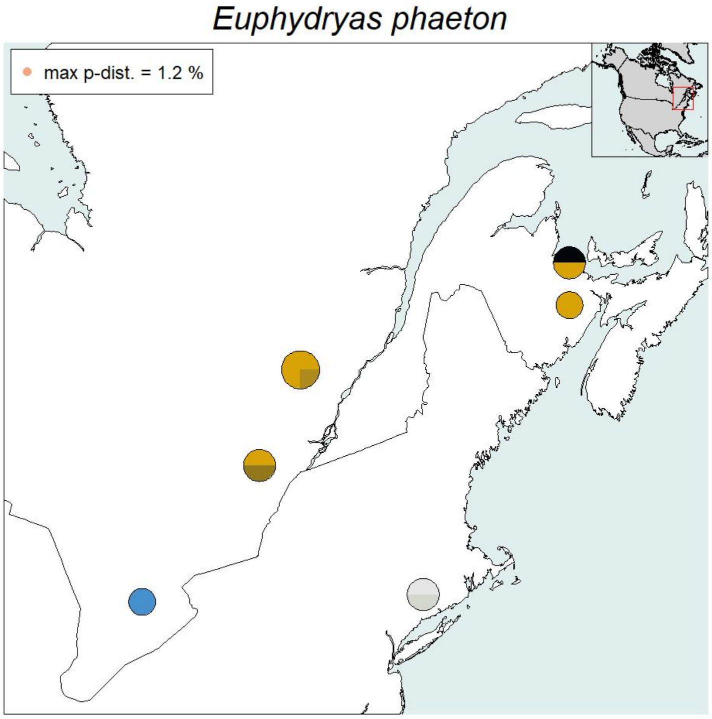

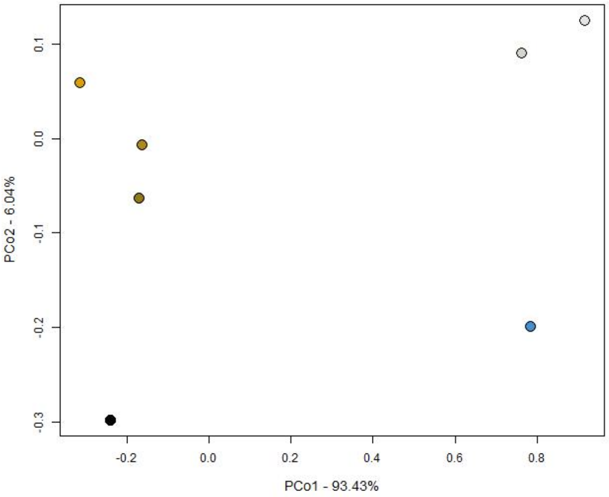

Figure 656 Map of *Euphydryas phaeton* showing the localities of the sequenced specimens (left). Nearby localities are grouped in pies. Colours match the bidimensional colour space of the PCoA projection (right) of max p-dists among sequences (dots). Sequences= 12; Hap obs.= 6; Hap asympt.= 13.3; Hap % obs.= 45%; GST= NaN; DST= NaN; HD= 0.758; ND= 0.005; max p-dist= 1.2%.

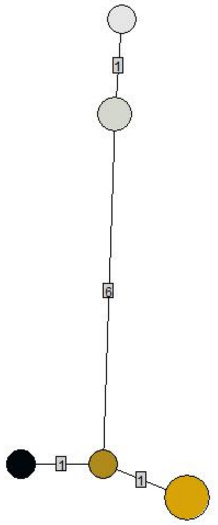

Figure 657: Haplotype network of *Euphydryas phaeton*. Sequences > 599 bp= 11.

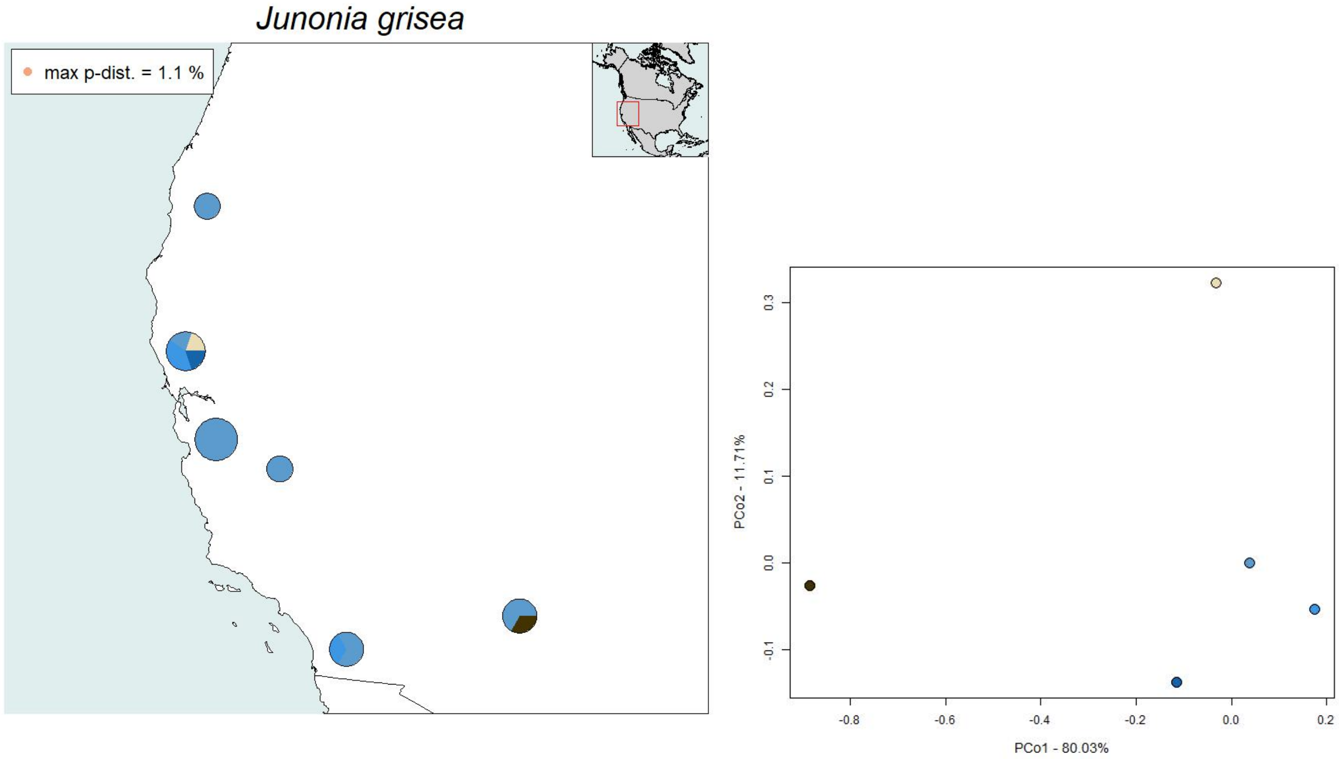

Figure 658 Map of *Junonia grisea* showing the localities of the sequenced specimens (left). Nearby localities are grouped in pies. Colours match the bidimensional colour space of the PCoA projection (right) of max p-dists among sequences (dots). Sequences= 20; Hap obs.= 7; Hap asympt.= 21.2; Hap % obs.= 32.9%; GST= 0.231; DST= 0.0004; HD= 0.521; ND= 0.0018; max p-dist= 1.1%.

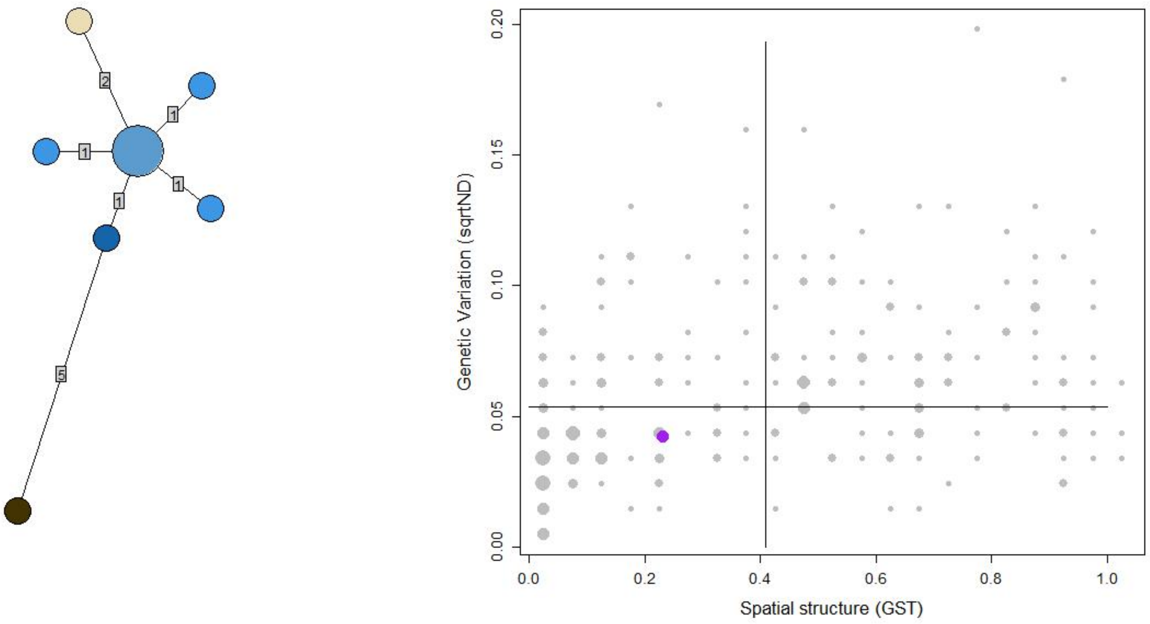

Figure 659: Haplotype network (left) of *Junonia grisea* sequences > 599 bp with colours matching the PCoA colour space (above). The bubble plot for mt-DNA polymorphism (square root transformed nucleotide diversity) and spatial structure (GST) among all species in the atlas and values for *Junonia grisea* (purple dot). The horizontal and vertical lines represent median values of nucleotide diversity and GST, respectively. Sequences > 599 bp= 20.

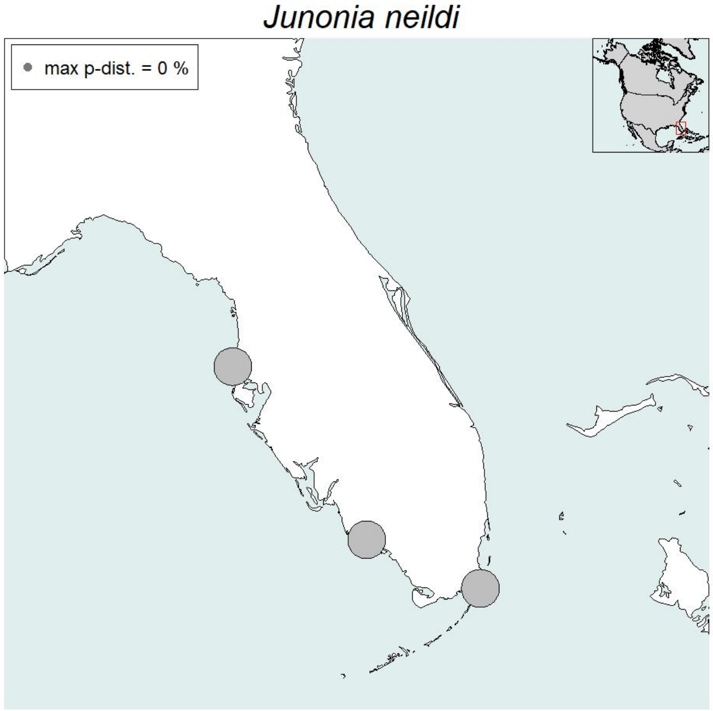

Figure 660: Map of *Junonia neildi* showing the localities of the sequenced specimens. Nearby localities are grouped in pies. Due to the presence of a single haplotype PCoA projection was not done and a single grey colour was plotted on the map. Sequences= 3; Hap obs.= 1; Hap asympt.= NA; Hap % obs.= NA%; GST= NaN; DST= NaN; HD= NA; ND= NA; max p-dist= 0%.

Haplotype network analysis and bubble plot of *Junonia neildi* were not possible. Sequences > 599 bp = 3.

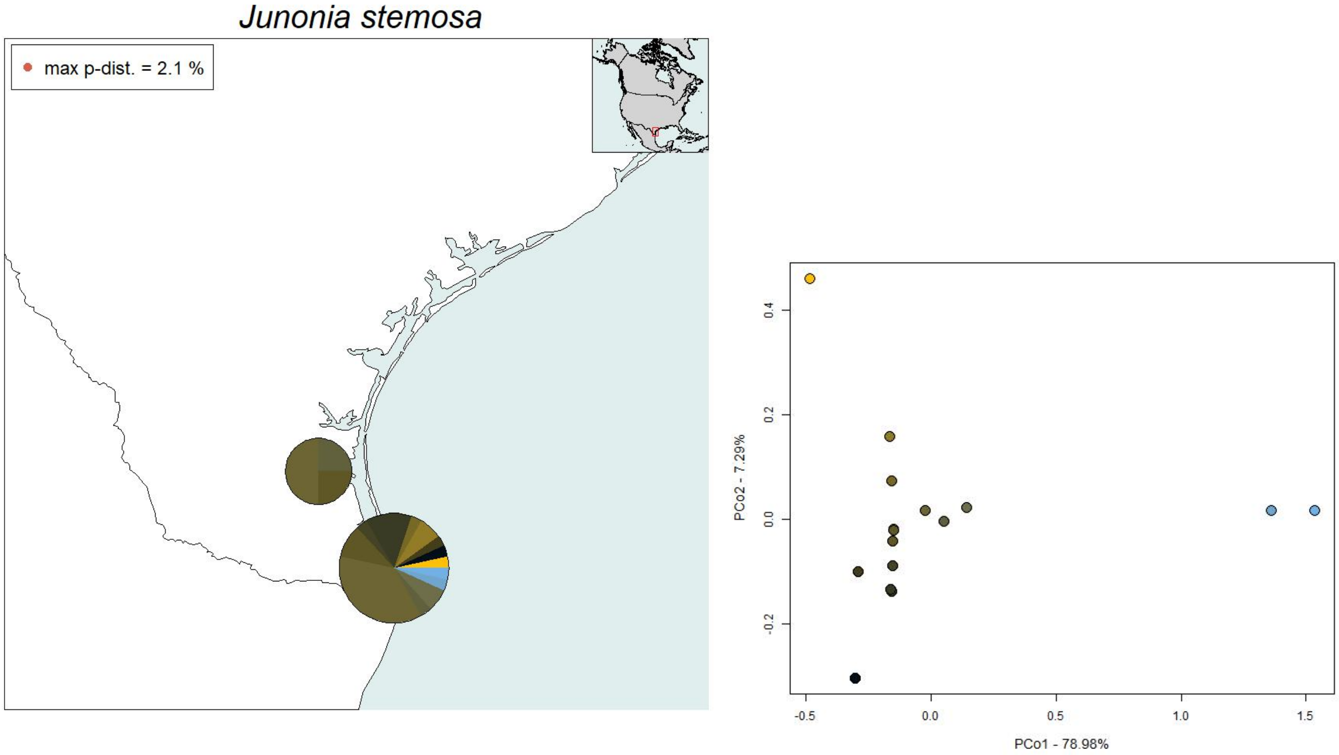

Figure 661 Map of *Junonia stemosa* showing the localities of the sequenced specimens (left). Nearby localities are grouped in pies. Colours match the bidimensional colour space of the PCoA projection (right) of max p-dists among sequences (dots). Sequences= 34; Hap obs.= 16; Hap asympt.= 35.6; Hap % obs.= 45%; GST= 0; DST= 0; HD= 0.845; ND= 0.0037; max p-dist= 2.1%.

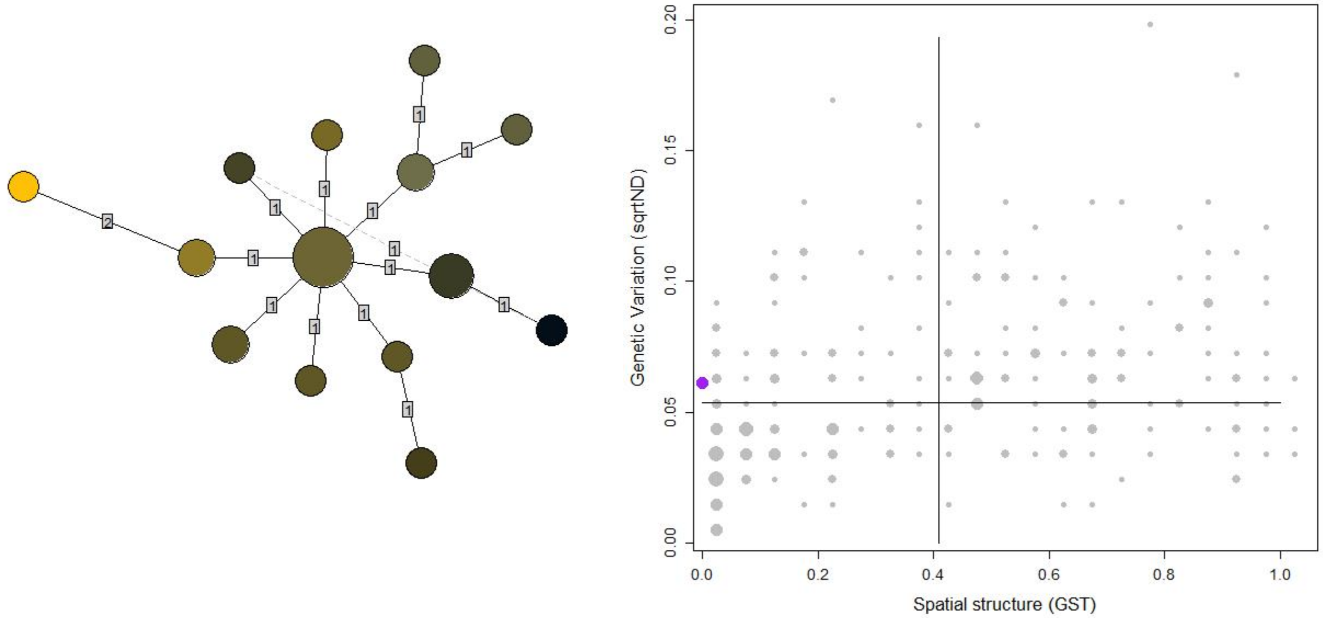

Figure 662: Haplotype network (left) of *Junonia stemosa* sequences > 599 bp with colours matching the PCoA colour space (above). The bubble plot for mt-DNA polymorphism (square root transformed nucleotide diversity) and spatial structure (GST) among all species in the atlas and values for *Junonia stemosa* (purple dot). The horizontal and vertical lines represent median values of nucleotide diversity and GST, respectively. Sequences > 599 bp= 32.

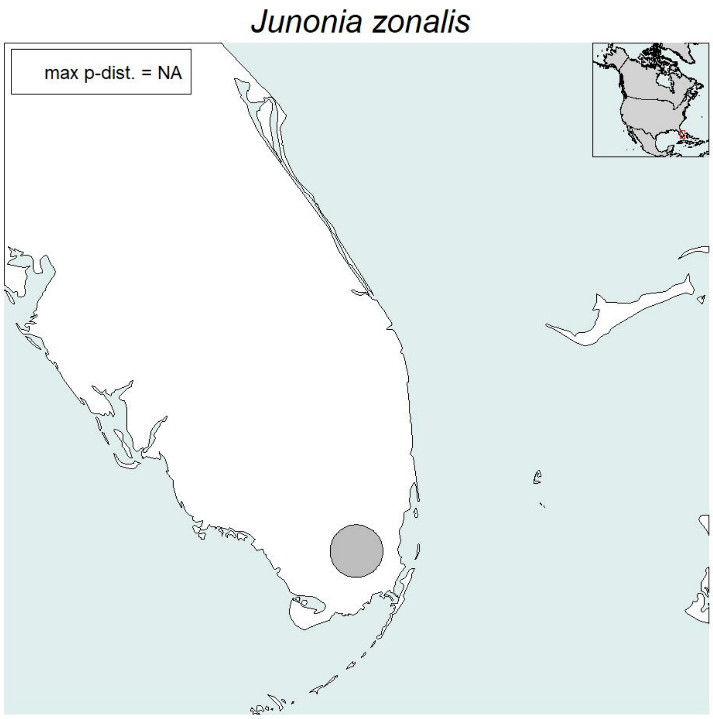

Figure 663: Map of *Junonia zonalis* showing the localities of the sequenced specimens. Nearby localities are grouped in pies. Due to the presence of a single haplotype PCoA projection was not done and a single grey colour was plotted on the map. Sequences= 1; Hap obs.= NA; Hap asympt.= NA; Hap % obs.= NA; GST= NaN; DST= NaN; HD= NA; ND= NA; max p-dist= NA.

Haplotype network analysis and bubble plot of *Junonia zonalis* were not possible. Sequences > 599 bp = 1.

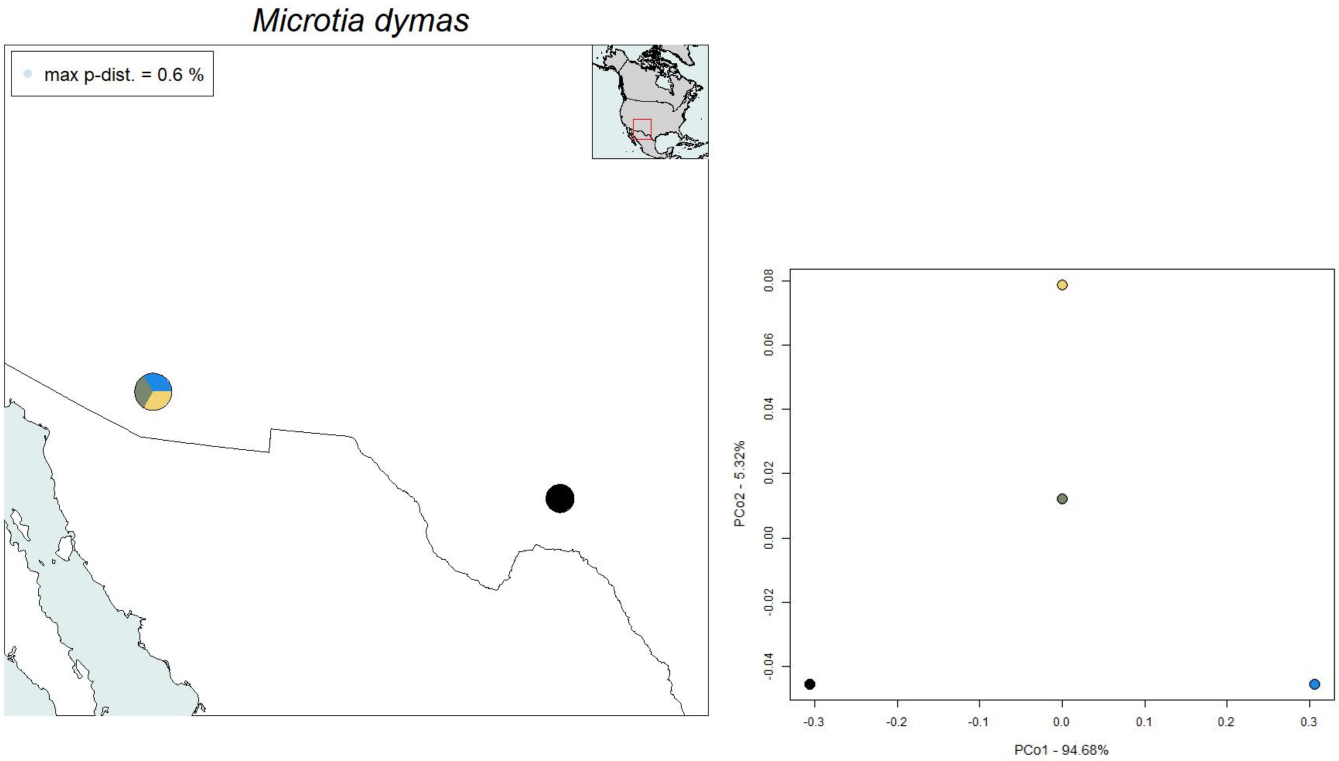

Figure 664 Map of *Microtia dymas* showing the localities of the sequenced specimens (left). Nearby localities are grouped in pies. Colours match the bidimensional colour space of the PCoA projection (right) of max p-dists among sequences (dots). Sequences= 4; Hap obs.= 3; Hap asympt.= NA; Hap % obs.= NA%; GST= NaN; DST= NaN; HD= NA; ND= NA; max p-dist= 0.6%.

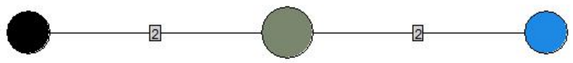

Figure 665: Haplotype network of *Microtia dymas*. Sequences > 599 bp= 4.

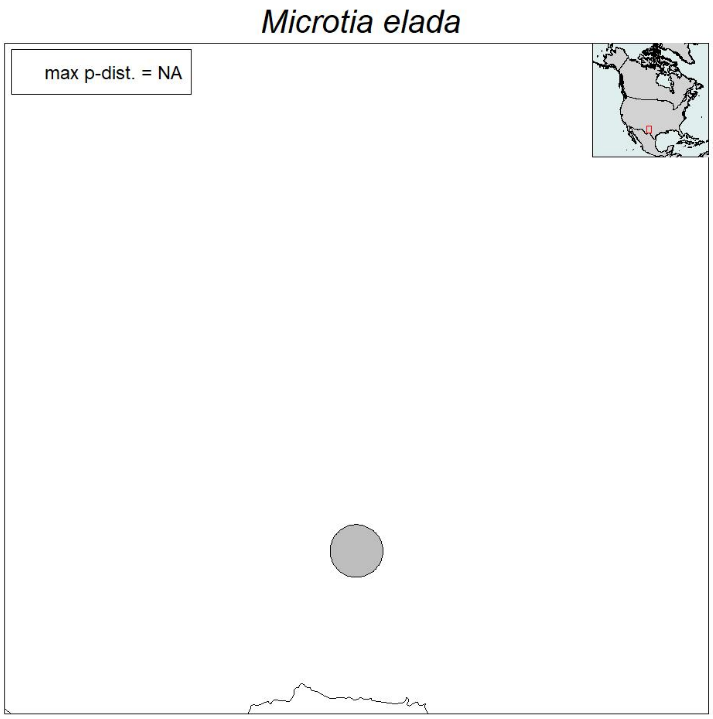

Figure 666: Map of *Microtia elada* showing the localities of the sequenced specimens. Nearby localities are grouped in pies. Due to the presence of a single haplotype PCoA projection was not done and a single grey colour was plotted on the map. Sequences= 1; Hap obs.= NA; Hap asympt.= NA; Hap % obs.= NA; GST= NaN; DST= NaN; HD= NA; ND= NA; max p-dist= NA.

Haplotype network analysis and bubble plot of *Microtia elada* were not possible. Sequences > 599 bp = 1.

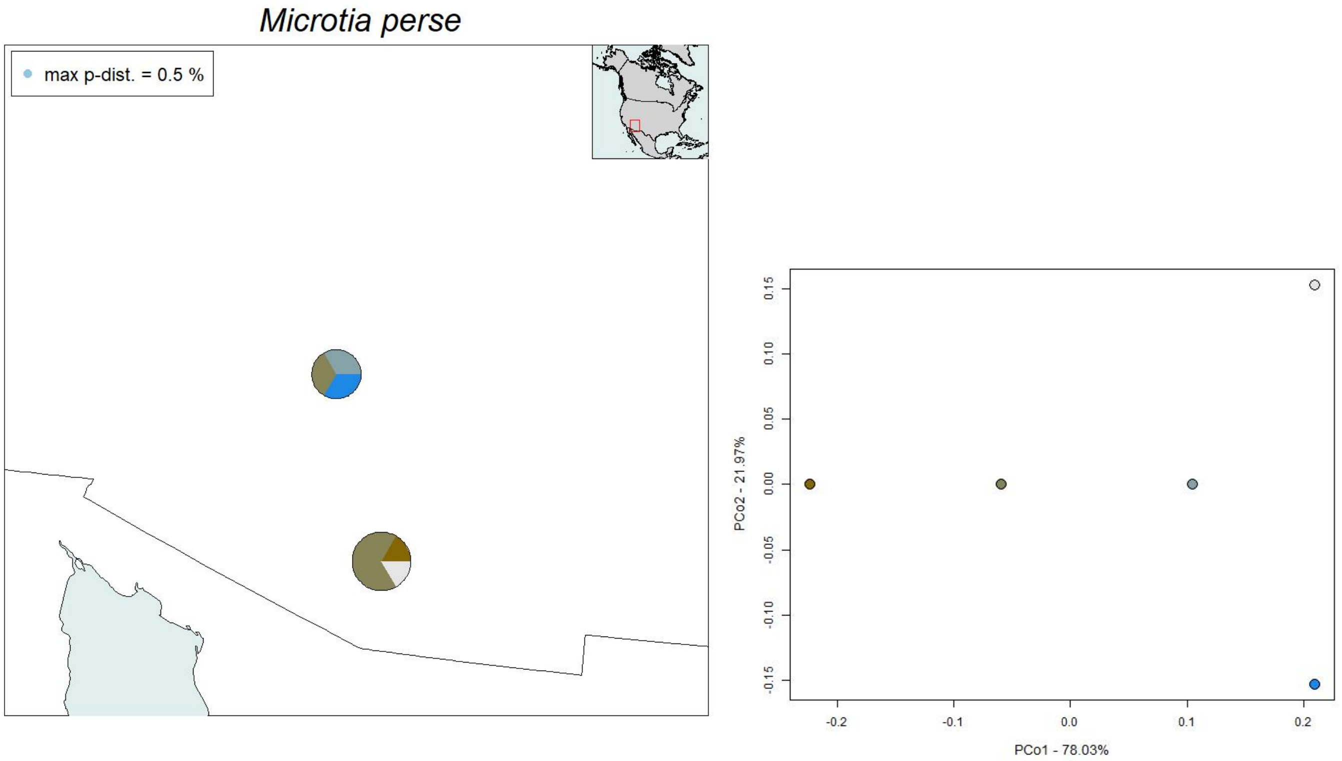

Figure 667 Map of *Microtia perse* showing the localities of the sequenced specimens (left). Nearby localities are grouped in pies. Colours match the bidimensional colour space of the PCoA projection (right) of max p-dists among sequences (dots). Sequences= 9; Hap obs.= 5; Hap asympt.= NA; Hap % obs.= NA%; GST= NaN; DST= NaN; HD= NA; ND= NA; max p-dist= 0.5%.

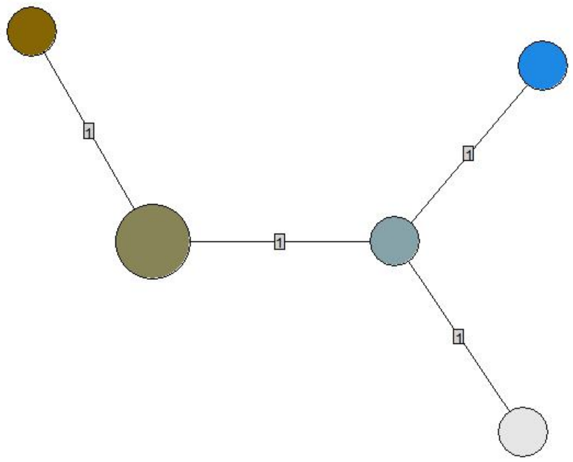

Figure 668: Haplotype network of *Microtia perse*. Sequences > 599 bp= 9.

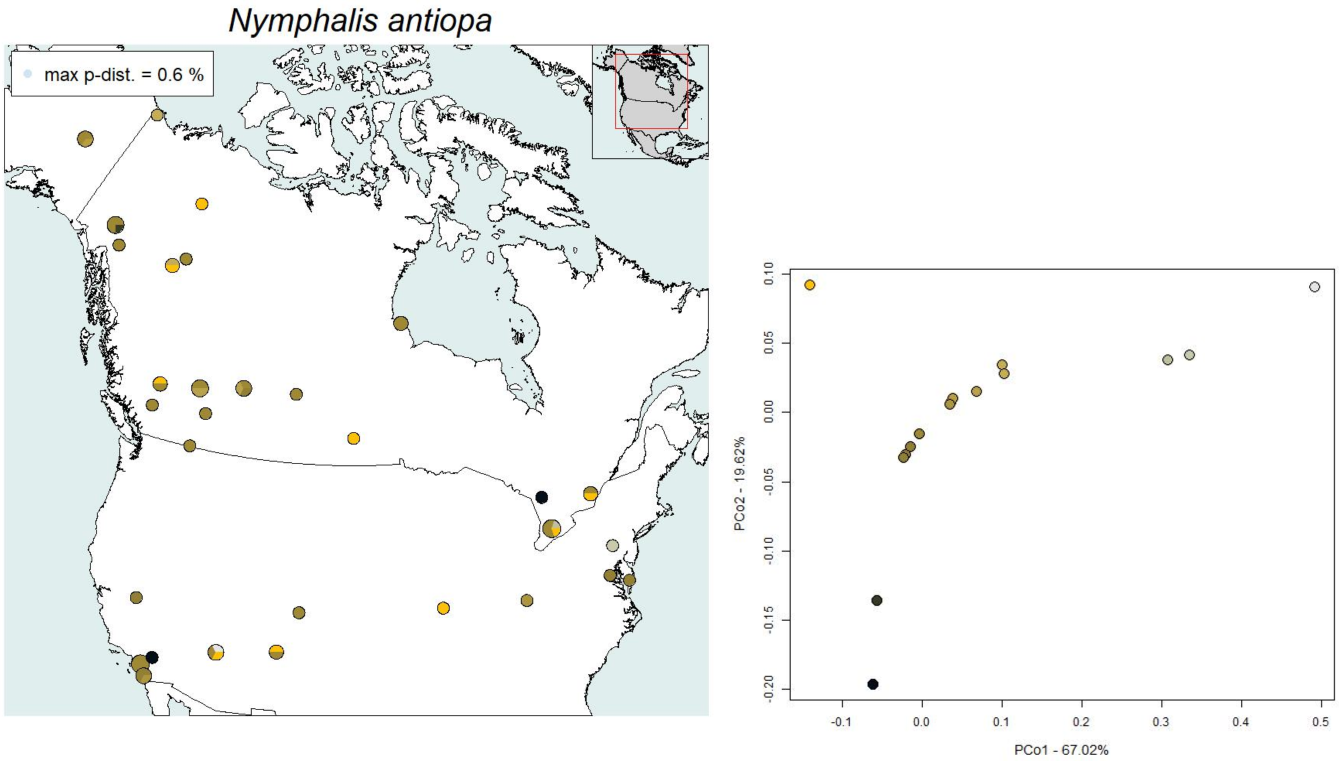

Figure 669 Map of *Nymphalis antiopa* showing the localities of the sequenced specimens (left). Nearby localities are grouped in pies. Colours match the bidimensional colour space of the PCoA projection (right) of max p-dists among sequences (dots). Sequences= 58; Hap obs.= 7; Hap asympt.= 14.9; Hap % obs.= 47.1%; GST= 0.193; DST= 0.0002; HD= 0.431; ND= 0.0009; max p-dist= 0.6%.

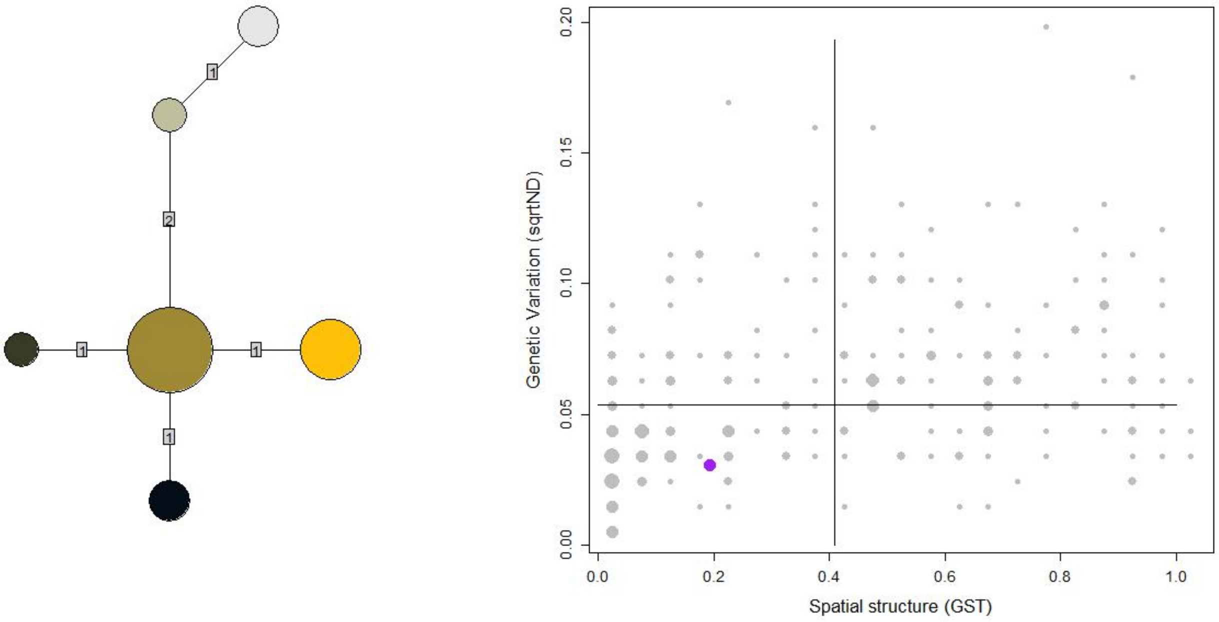

Figure 670: Haplotype network (left) of *Nymphalis antiopa* sequences > 599 bp with colours matching the PCoA colour space (above). The bubble plot for mt-DNA polymorphism (square root transformed nucleotide diversity) and spatial structure (GST) among all species in the atlas and values for *Nymphalis antiopa* (purple dot). The horizontal and vertical lines represent median values of nucleotide diversity and GST, respectively. Sequences > 599 bp= 52.

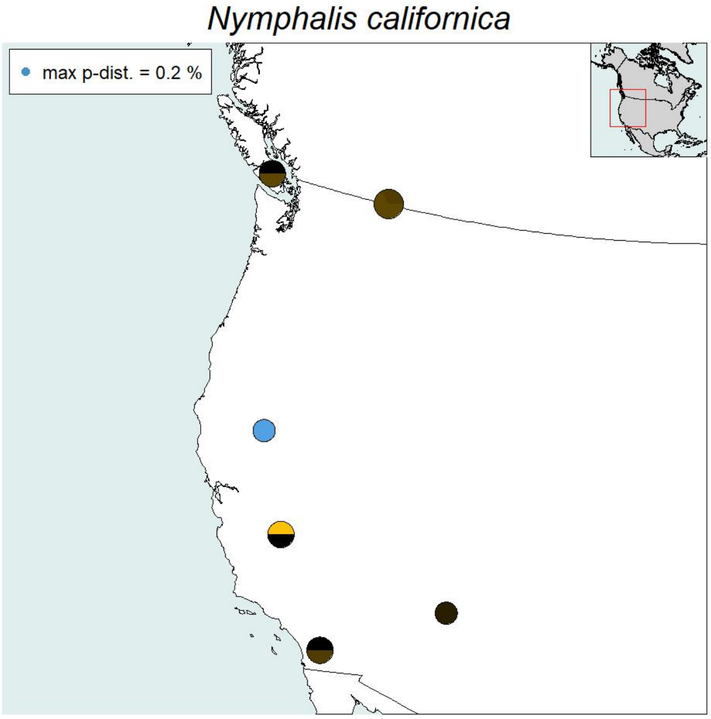

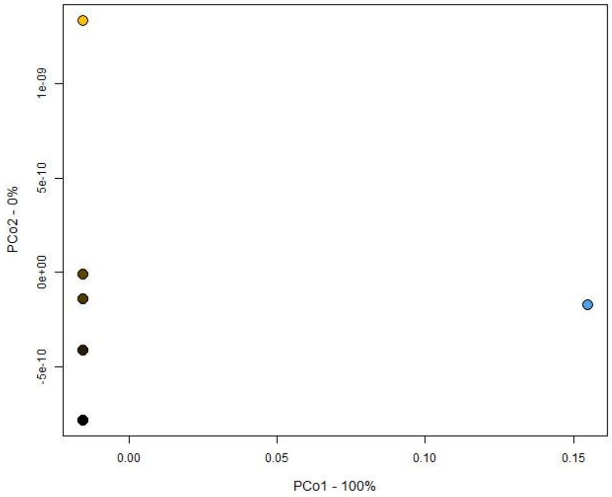

Figure 671 Map of *Nymphalis californica* showing the localities of the sequenced specimens (left). Nearby localities are grouped in pies. Colours match the bidimensional colour space of the PCoA projection (right) of max p-dists among sequences (dots). Sequences= 11; Hap obs.= 2; Hap asympt.= 2; Hap % obs.= 100%; GST= NaN; DST= NaN; HD= 0.182; ND= 0.0003; max p-dist= 0.2%.

Haplotype network analysis and bubble plot of *Nymphalis californica* were not possible. Sequences > 599 bp = 10.

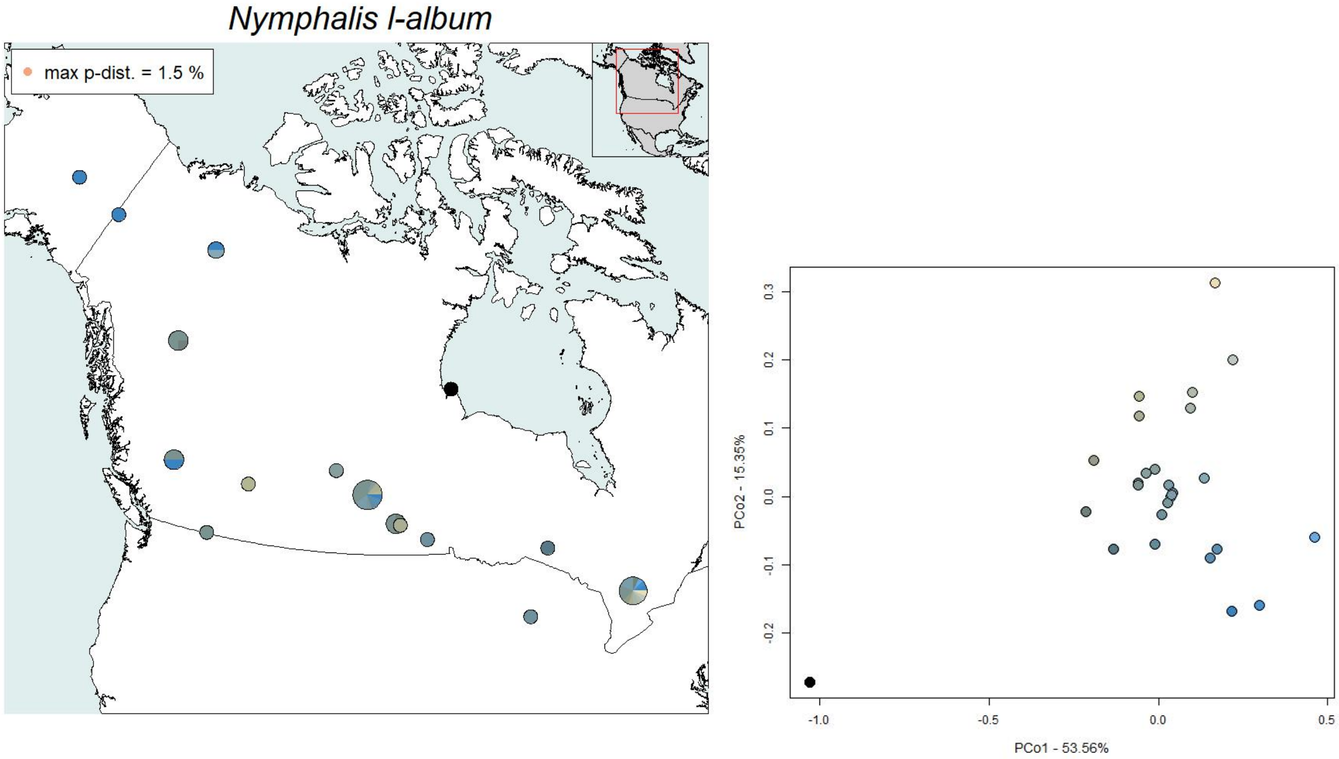

Figure 672 Map of *Nymphalis l-album* showing the localities of the sequenced specimens (left). Nearby localities are grouped in pies. Colours match the bidimensional colour space of the PCoA projection (right) of max p-dists among sequences (dots). Sequences= 60; Hap obs.= 18; Hap asympt.= 88.8; Hap % obs.= 20.3%; GST= 0.033; DST= 0.0001; HD= 0.832; ND= 0.0021; max p-dist= 1.5%.

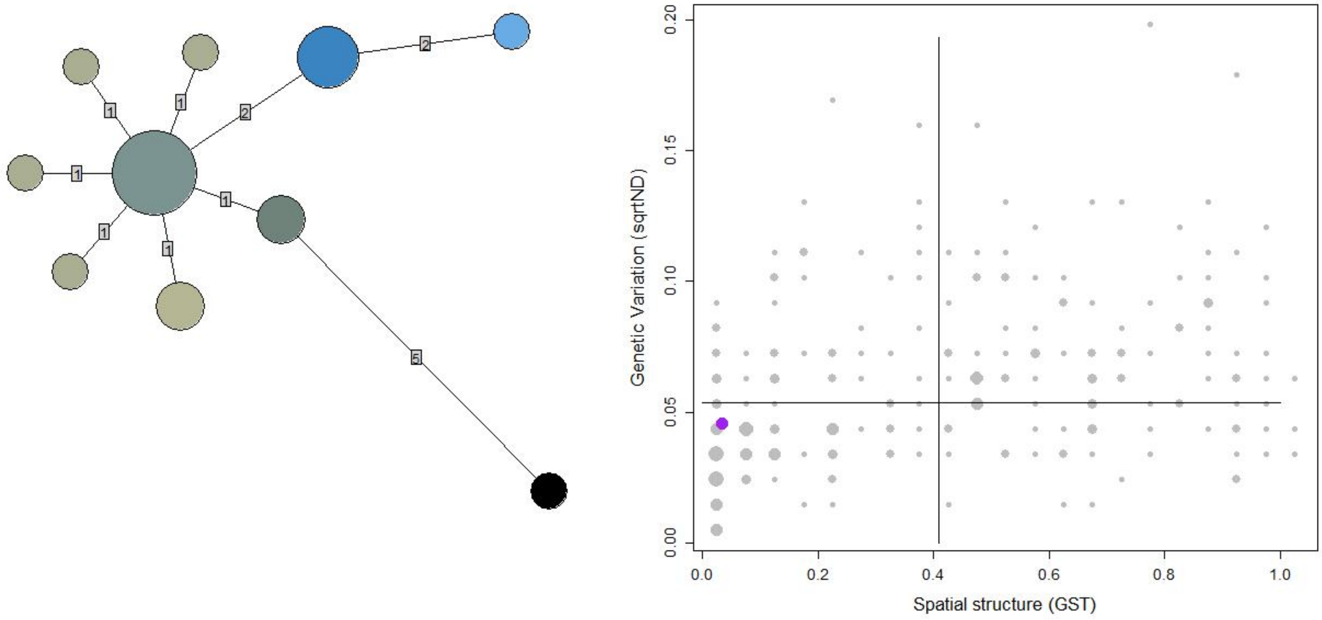

Figure 673: Haplotype network (left) of *Nymphalis l-album* sequences > 599 bp with colours matching the PCoA colour space (above). The bubble plot for mt-DNA polymorphism (square root transformed nucleotide diversity) and spatial structure (GST) among all species in the atlas and values for *Nymphalis l-album* (purple dot). The horizontal and vertical lines represent median values of nucleotide diversity and GST, respectively. Sequences > 599 bp= 47.

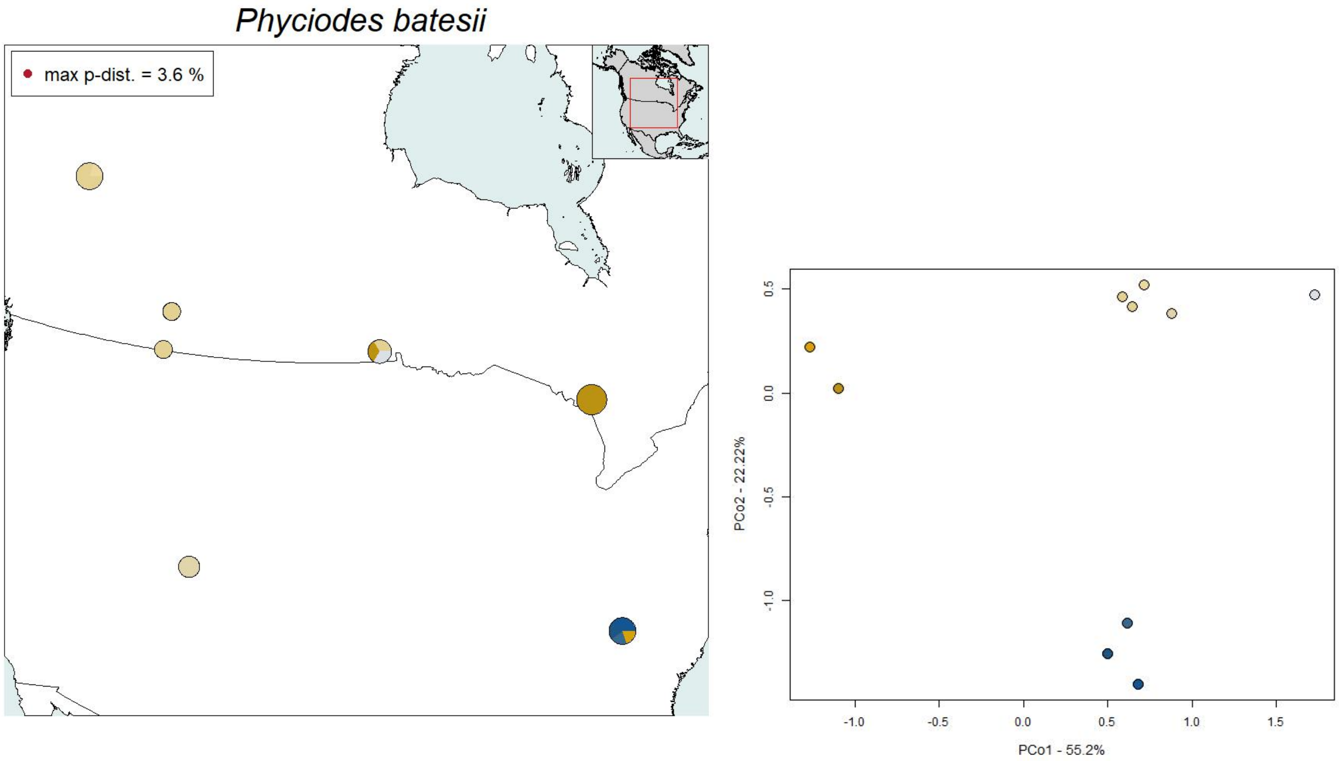

Figure 674 Map of *Phyciodes batesii* showing the localities of the sequenced specimens (left). Nearby localities are grouped in pies. Colours match the bidimensional colour space of the PCoA projection (right) of max p-dists among sequences (dots). Sequences= 25; Hap obs.= 8; Hap asympt.= 15.7; Hap % obs.= 51%; GST= 0.8; DST= 0.011; HD= 0.807; ND= 0.0153; max p-dist= 3.6%.

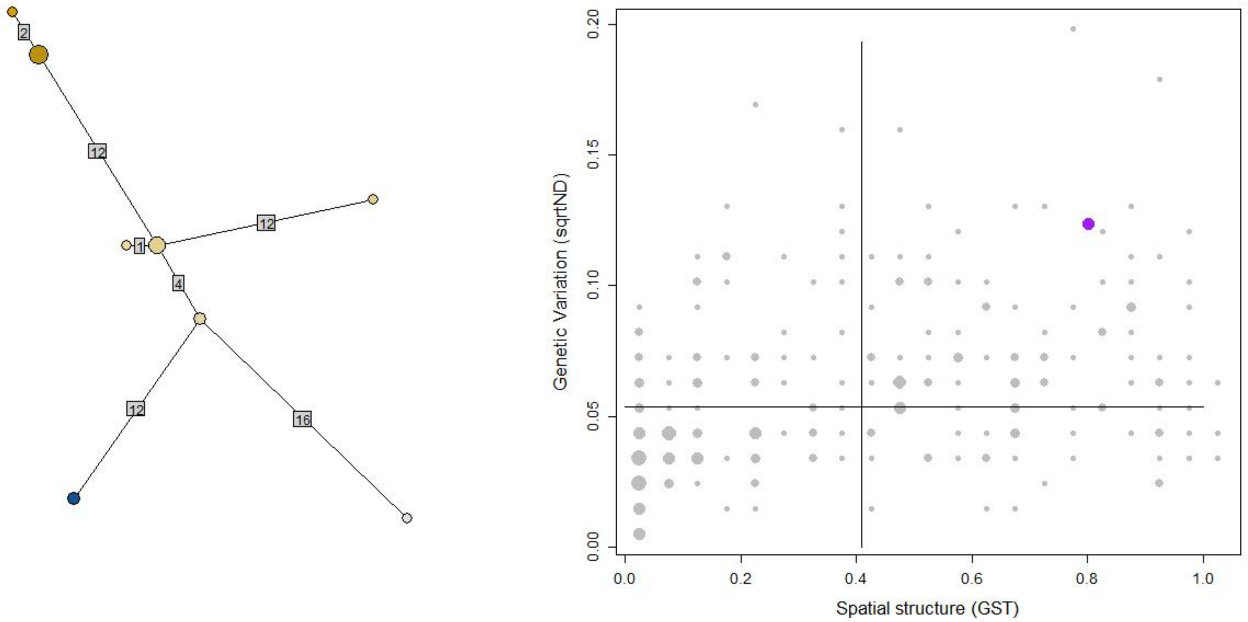

Figure 675: Haplotype network (left) of *Phyciodes batesii* sequences > 599 bp with colours matching the PCoA colour space (above). The bubble plot for mt-DNA polymorphism (square root transformed nucleotide diversity) and spatial structure (GST) among all species in the atlas and values for *Phyciodes batesii* (purple dot). The horizontal and vertical lines represent median values of nucleotide diversity and GST, respectively. Sequences > 599 bp= 23.

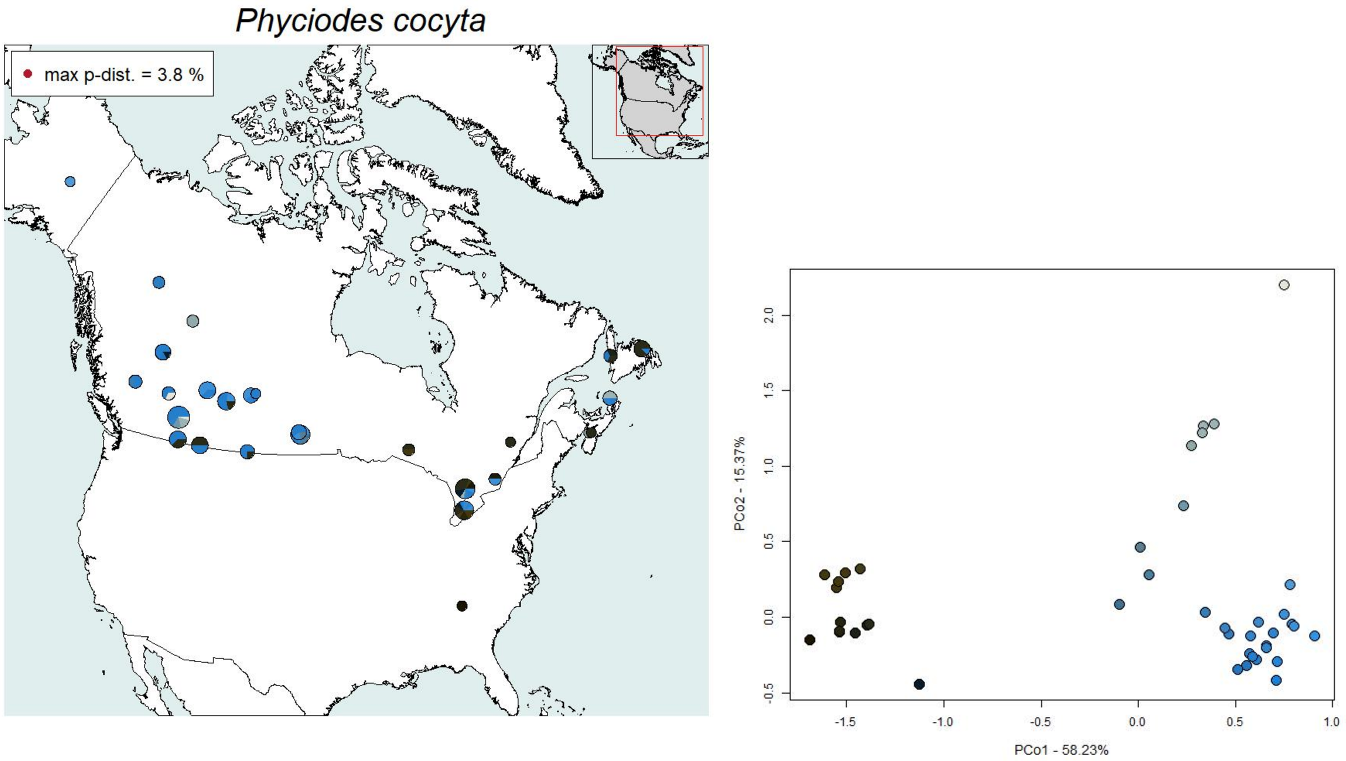

Figure 676 Map of *Phyciodes cocyta* showing the localities of the sequenced specimens (left). Nearby localities are grouped in pies. Colours match the bidimensional colour space of the PCoA projection (right) of max p-dists among sequences (dots). Sequences= 150; Hap obs.= 35; Hap asympt.= 64.9; Hap % obs.= 53.9%; GST= 0.189; DST= 0.0025; HD= 0.814; ND= 0.0134; max p-dist= 3.8%.

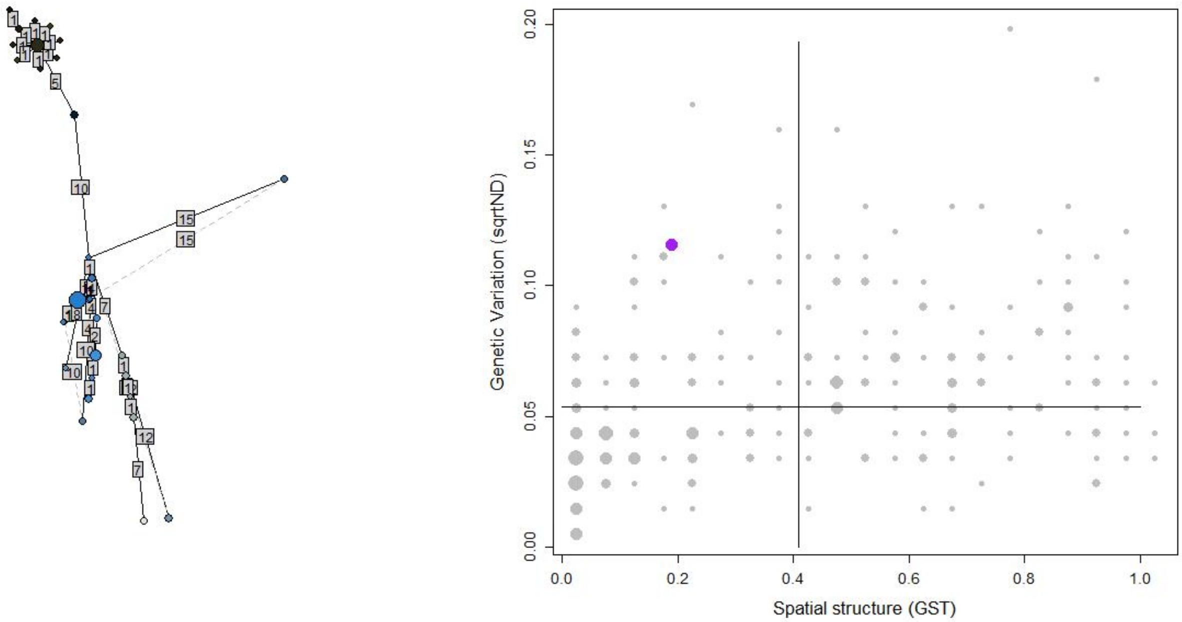

Figure 677: Haplotype network (left) of *Phyciodes cocyta* sequences > 599 bp with colours matching the PCoA colour space (above). The bubble plot for mt-DNA polymorphism (square root transformed nucleotide diversity) and spatial structure (GST) among all species in the atlas and values for *Phyciodes cocyta* (purple dot). The horizontal and vertical lines represent median values of nucleotide diversity and GST, respectively. Sequences > 599 bp= 145.

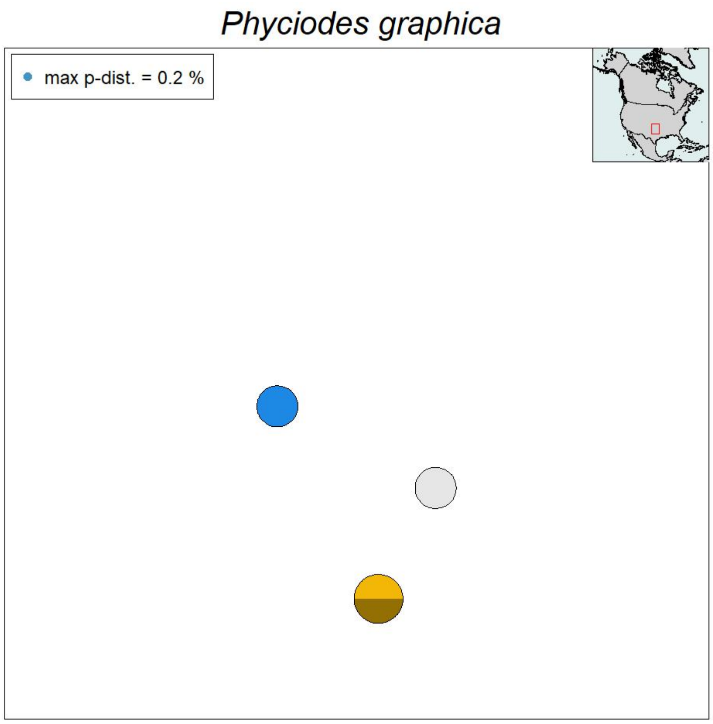

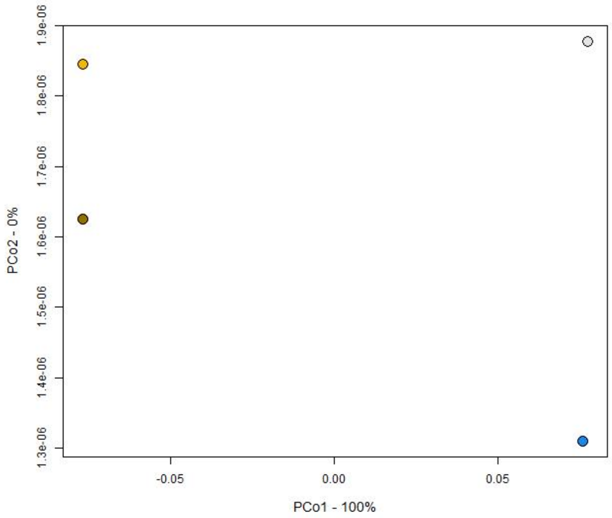

Figure 678 Map of *Phyciodes graphica* showing the localities of the sequenced specimens (left). Nearby localities are grouped in pies. Colours match the bidimensional colour space of the PCoA projection (right) of max p-dists among sequences (dots). Sequences= 4; Hap obs.= 2; Hap asympt.= NA; Hap % obs.= NA%; GST= NaN; DST= NaN; HD= NA; ND= NA; max p-dist= 0.2%.

Haplotype network analysis and bubble plot of *Phyciodes graphica* were not possible. Sequences > 599 bp = 4.

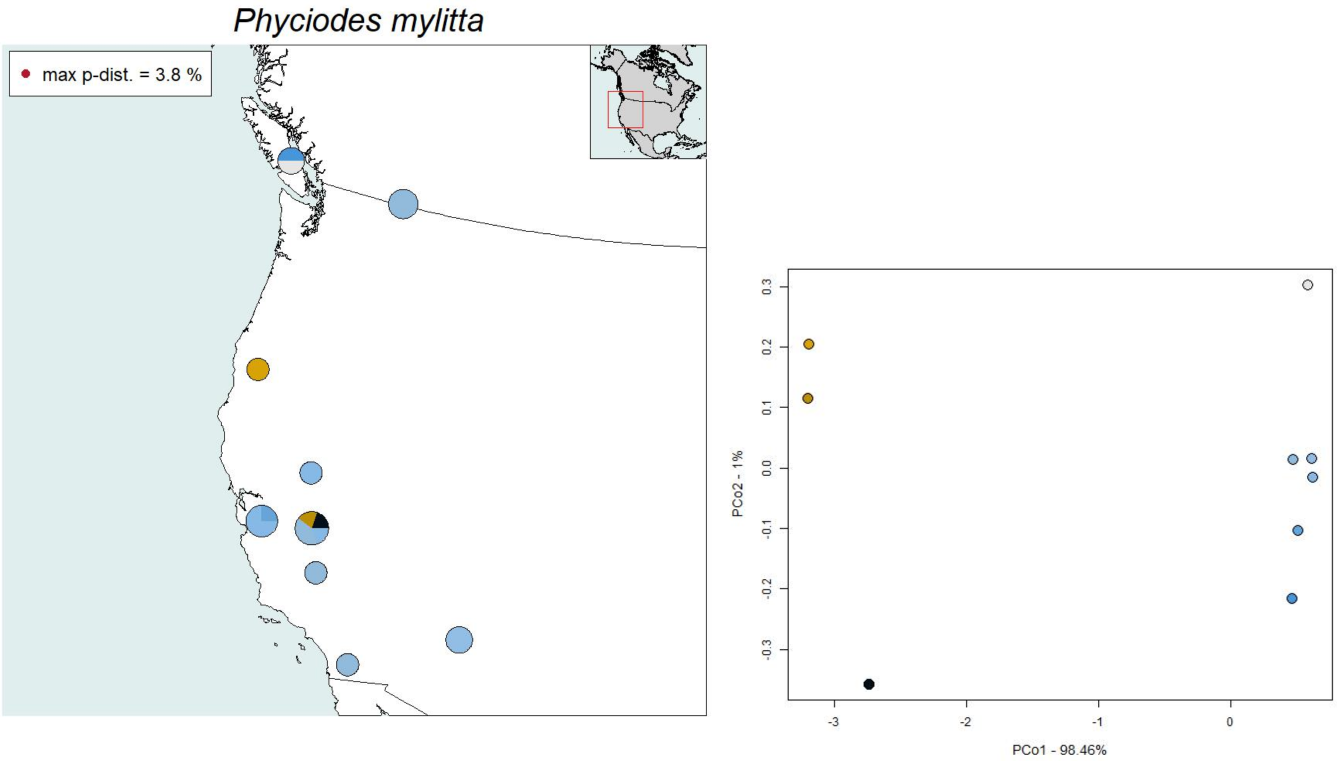

Figure 679 Map of *Phyciodes mylitta* showing the localities of the sequenced specimens (left). Nearby localities are grouped in pies. Colours match the bidimensional colour space of the PCoA projection (right) of max p-dists among sequences (dots). Sequences= 20; Hap obs.= 6; Hap asympt.= 10.3; Hap % obs.= 58.4%; GST= 0.103; DST= 0.0014; HD= 0.726; ND= 0.0105; max p-dist= 3.8%.

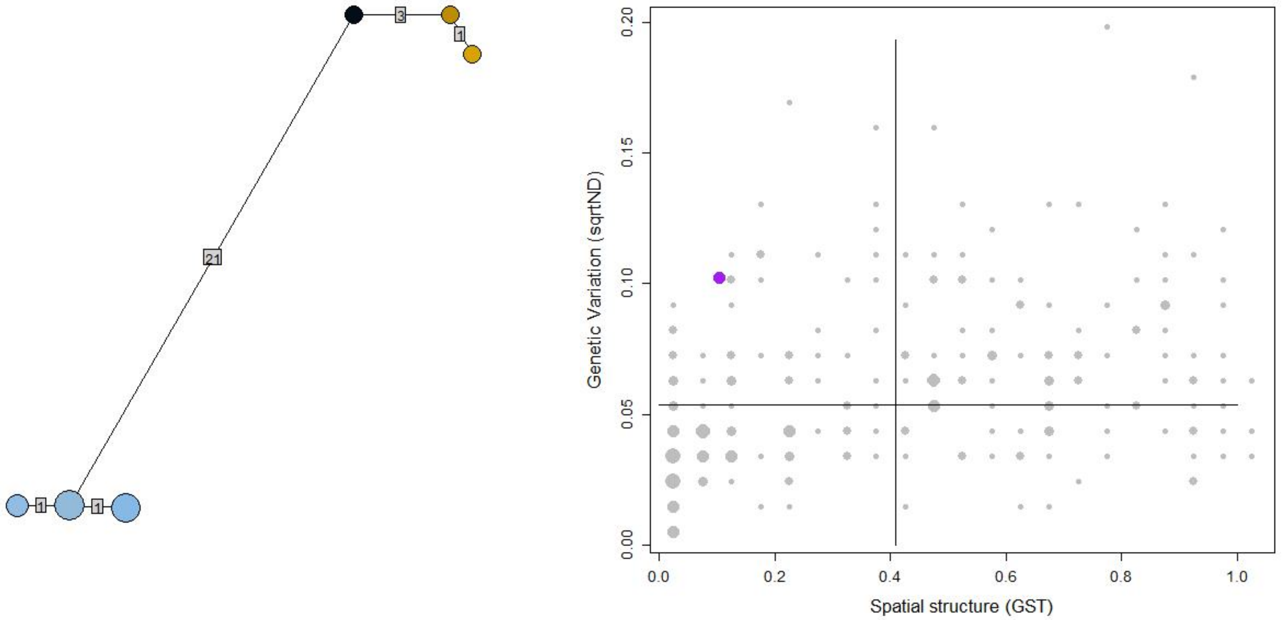

Figure 680: Haplotype network (left) of *Phyciodes mylitta* sequences > 599 bp with colours matching the PCoA colour space (above). The bubble plot for mt-DNA polymorphism (square root transformed nucleotide diversity) and spatial structure (GST) among all species in the atlas and values for *Phyciodes mylitta* (purple dot). The horizontal and vertical lines represent median values of nucleotide diversity and GST, respectively. Sequences > 599 bp= 18.

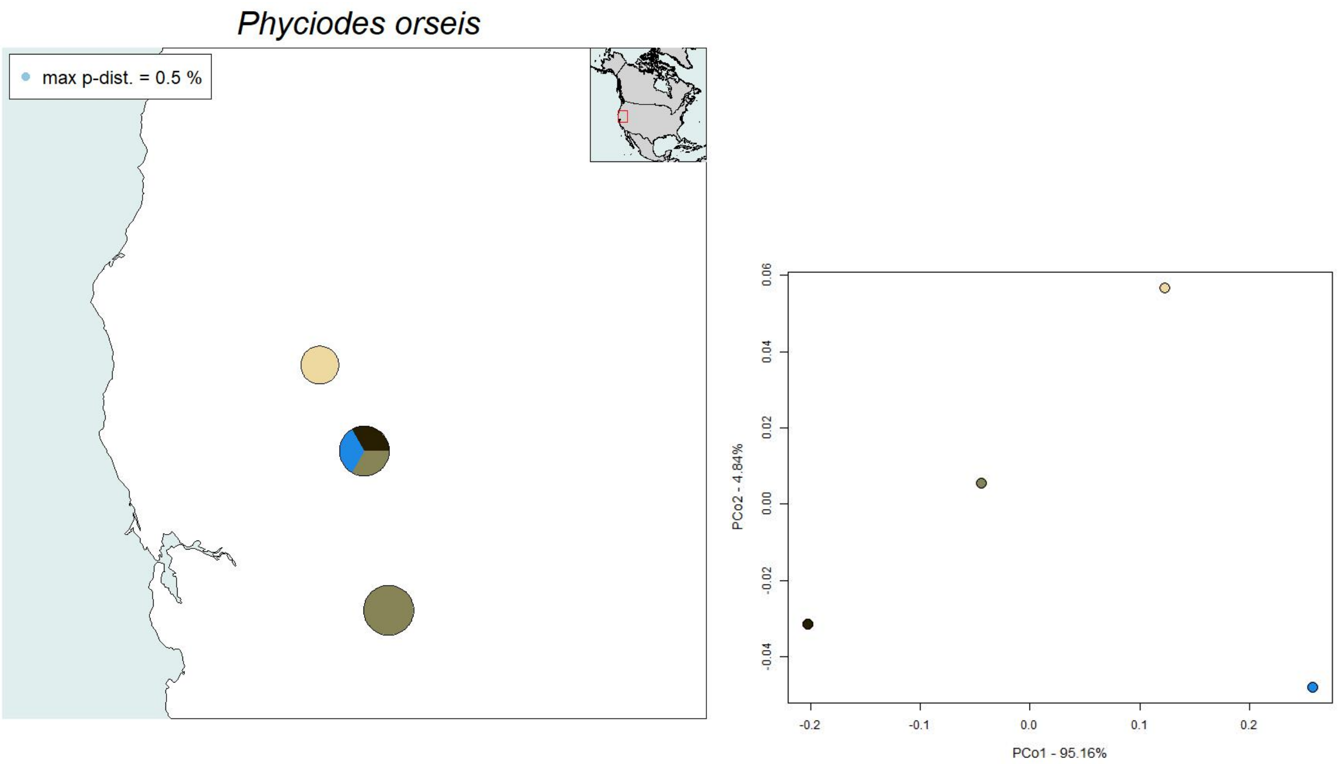

Figure 681 Map of *Phyciodes orseis* showing the localities of the sequenced specimens (left). Nearby localities are grouped in pies. Colours match the bidimensional colour space of the PCoA projection (right) of max p-dists among sequences (dots). Sequences= 7; Hap obs.= 4; Hap asympt.= NA; Hap % obs.= NA%; GST= NaN; DST= NaN; HD= NA; ND= NA; max p-dist= 0.5%.

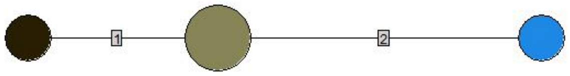

Figure 682: Haplotype network of *Phyciodes orseis*. Sequences > 599 bp= 6.

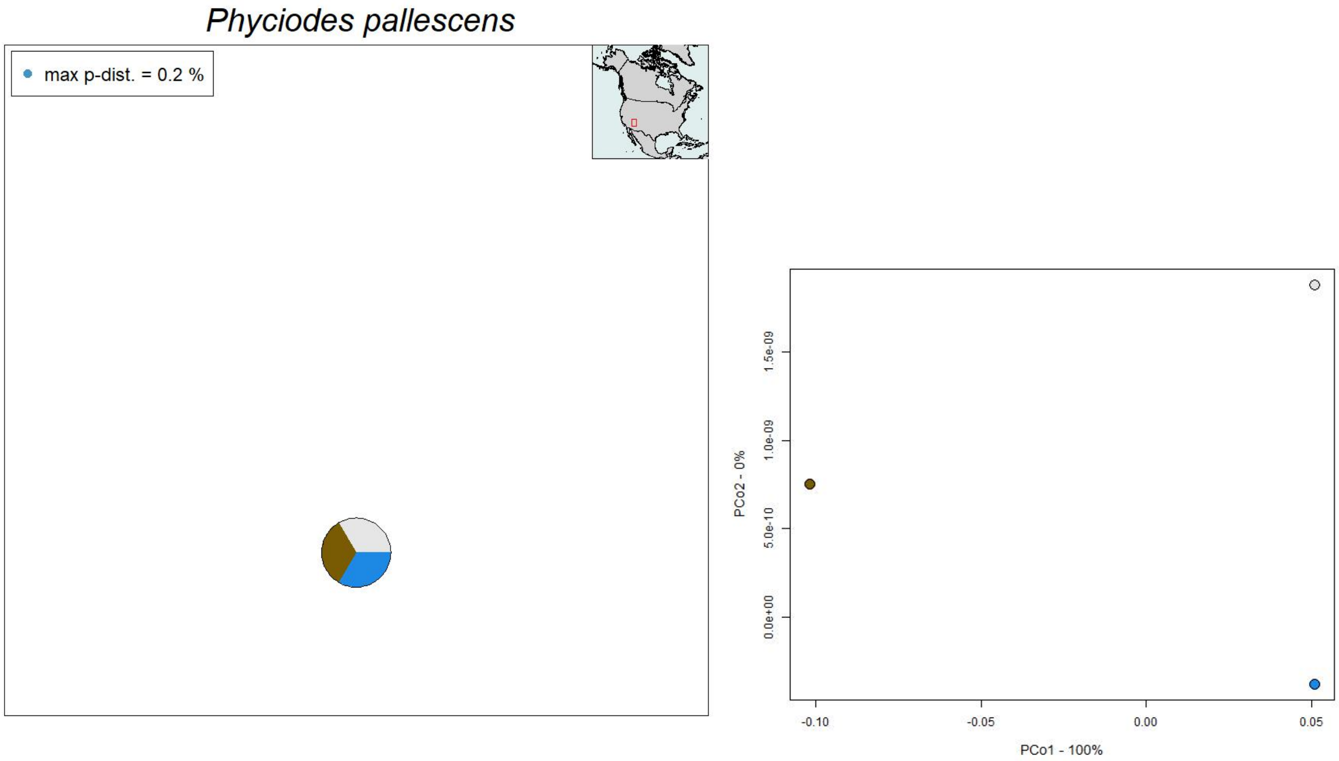

Figure 683 Map of *Phyciodes pallescens* showing the localities of the sequenced specimens (left). Nearby localities are grouped in pies. Colours match the bidimensional colour space of the PCoA projection (right) of max p-dists among sequences (dots). Sequences= 3; Hap obs.= 2; Hap asympt.= NA; Hap % obs.= NA%; GST= NaN; DST= NaN; HD= NA; ND= NA; max p-dist= 0.2%.

Haplotype network analysis and bubble plot of *Phyciodes pallescens* were not possible. Sequences > 599 bp = 3.

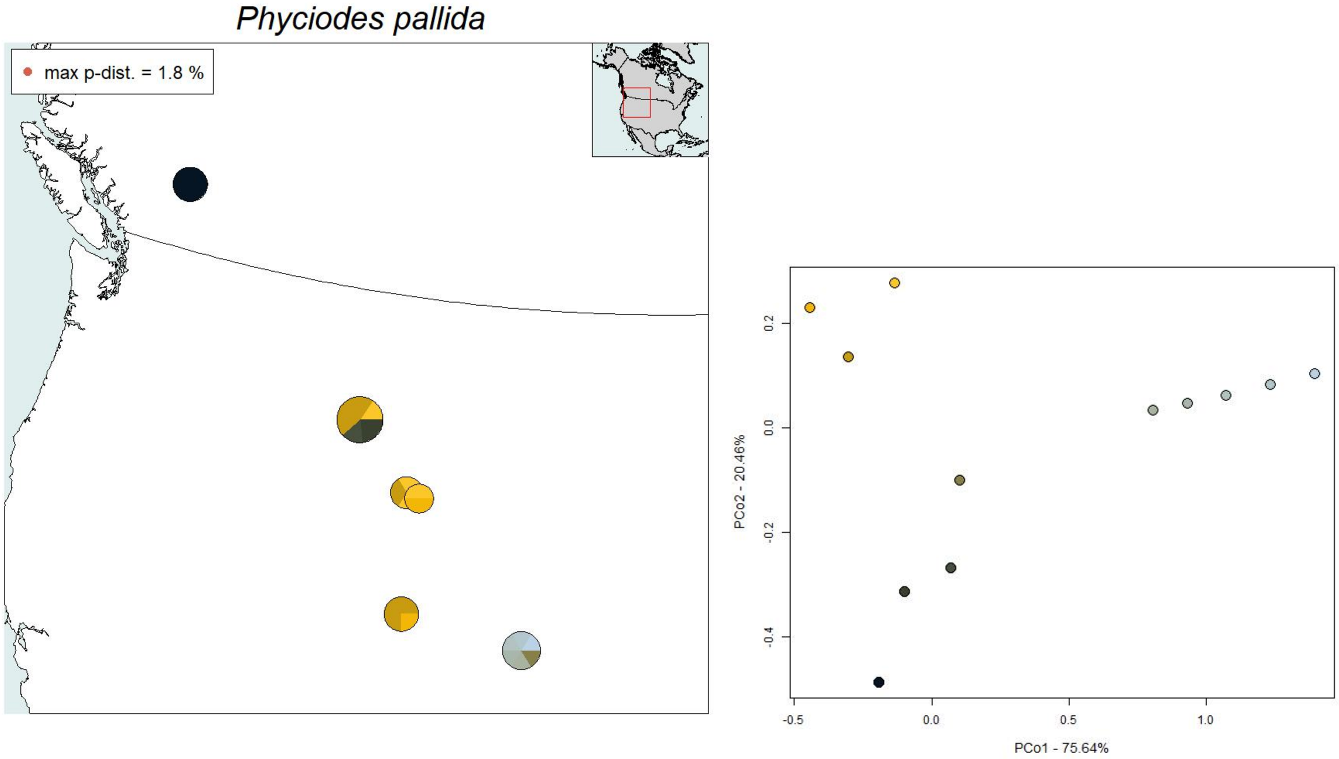

Figure 684 Map of *Phyciodes pallida* showing the localities of the sequenced specimens (left). Nearby localities are grouped in pies. Colours match the bidimensional colour space of the PCoA projection (right) of max p-dists among sequences (dots). Sequences= 32; Hap obs.= 11; Hap asympt.= 23.1; Hap % obs.= 47.6%; GST= 0.524; DST= 0.003; HD= 0.863; ND= 0.006; max p-dist= 1.8%.

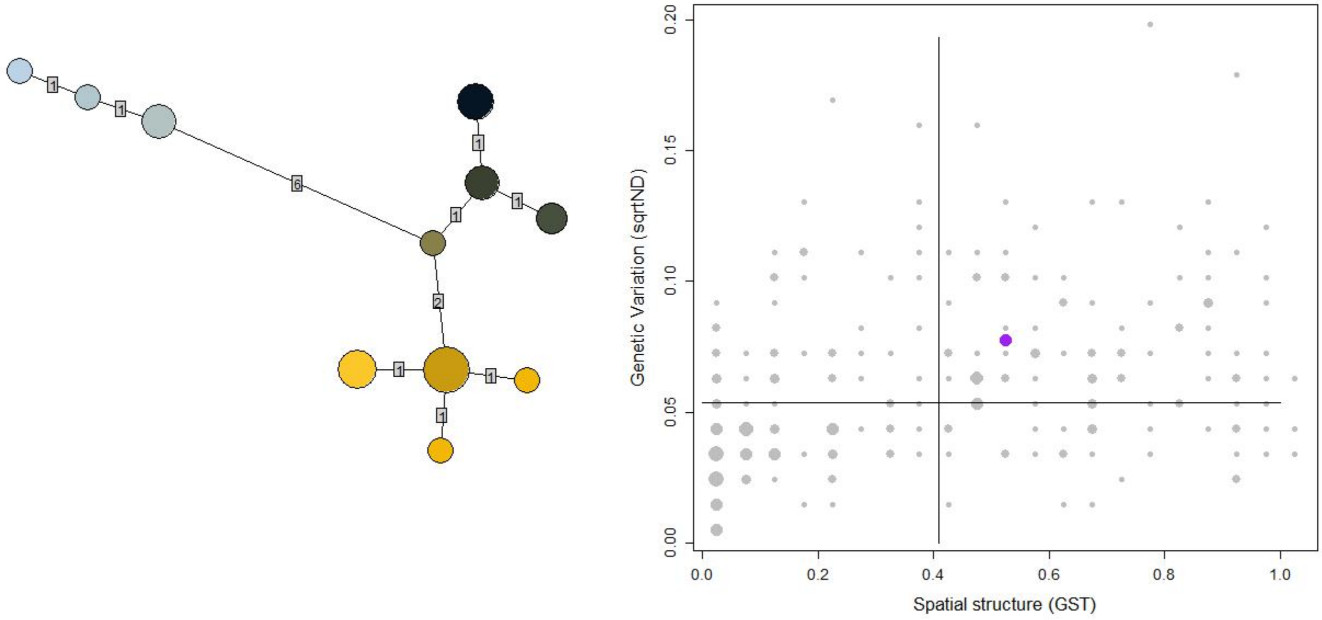

Figure 685: Haplotype network (left) of *Phyciodes pallida* sequences > 599 bp with colours matching the PCoA colour space (above). The bubble plot for mt-DNA polymorphism (square root transformed nucleotide diversity) and spatial structure (GST) among all species in the atlas and values for *Phyciodes pallida* (purple dot). The horizontal and vertical lines represent median values of nucleotide diversity and GST, respectively. Sequences > 599 bp= 32.

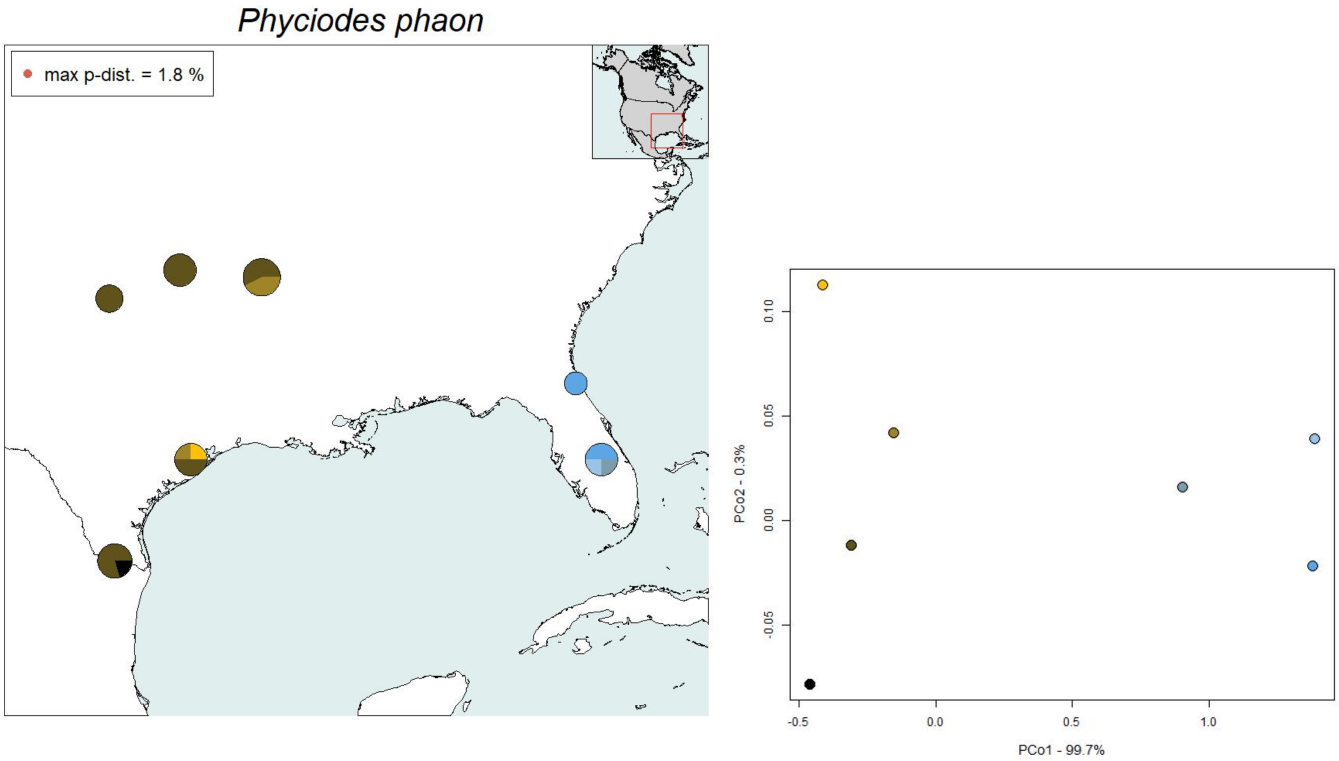

Figure 686 Map of *Phyciodes phaon* showing the localities of the sequenced specimens (left). Nearby localities are grouped in pies. Colours match the bidimensional colour space of the PCoA projection (right) of max p-dists among sequences (dots). Sequences= 27; Hap obs.= 4; Hap asympt.= 4; Hap % obs.= 100%; GST= 0.86; DST= 0.0038; HD= 0.567; ND= 0.0053; max p-dist= 1.8%.

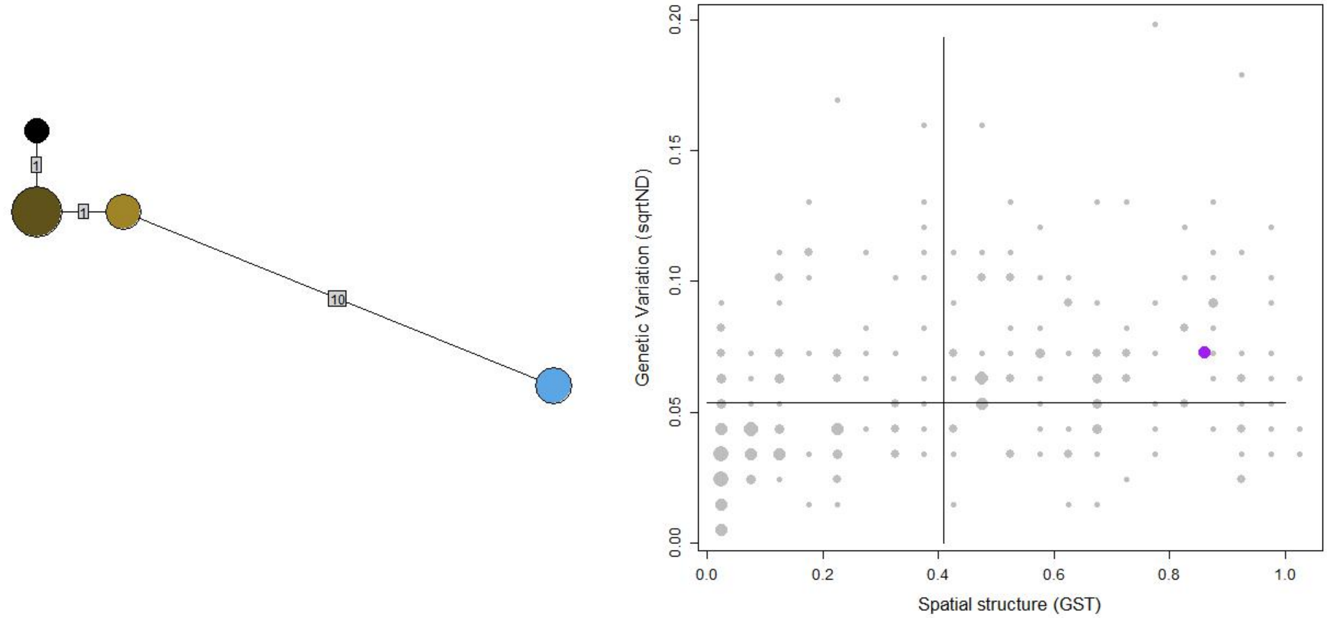

Figure 687: Haplotype network (left) of *Phyciodes phaon* sequences > 599 bp with colours matching the PCoA colour space (above). The bubble plot for mt-DNA polymorphism (square root transformed nucleotide diversity) and spatial structure (GST) among all species in the atlas and values for *Phyciodes phaon* (purple dot). The horizontal and vertical lines represent median values of nucleotide diversity and GST, respectively. Sequences > 599 bp= 27.

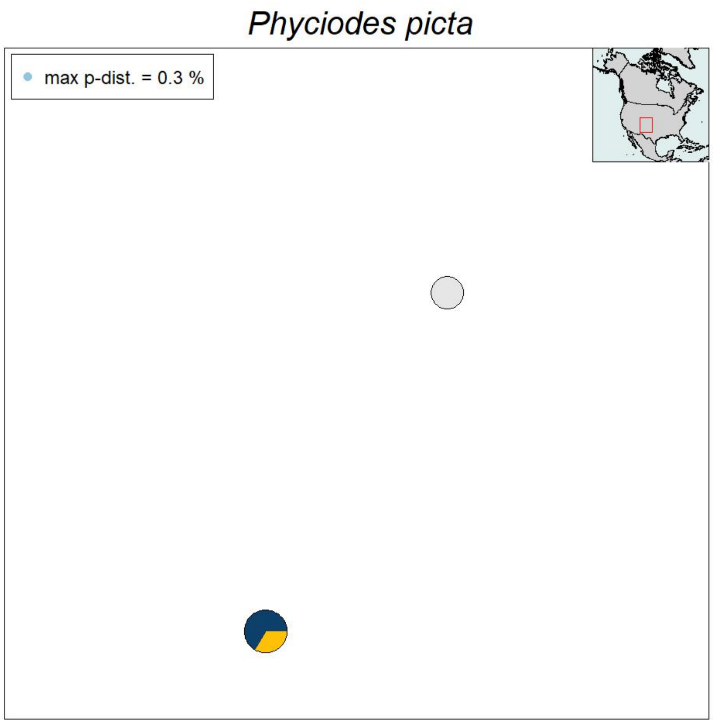

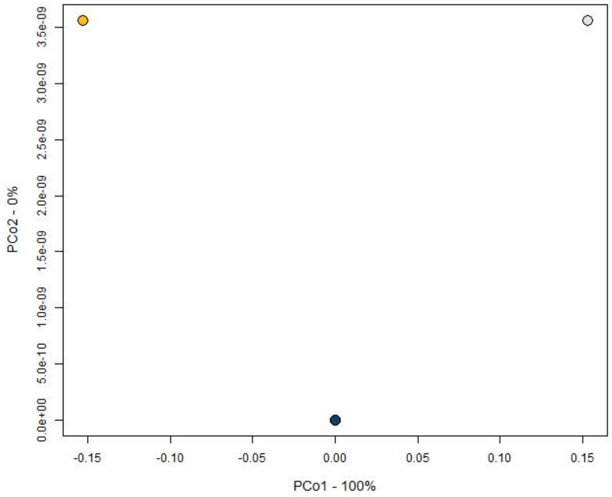

Figure 688 Map of *Phyciodes picta* showing the localities of the sequenced specimens (left). Nearby localities are grouped in pies. Colours match the bidimensional colour space of the PCoA projection (right) of max p-dists among sequences (dots). Sequences= 4; Hap obs.= 3; Hap asympt.= NA; Hap % obs.= NA%; GST= NaN; DST= NaN; HD= NA; ND= NA; max p-dist= 0.3%.

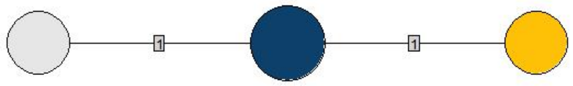

Figure 689: Haplotype network of *Phyciodes picta*. Sequences > 599 bp= 4.

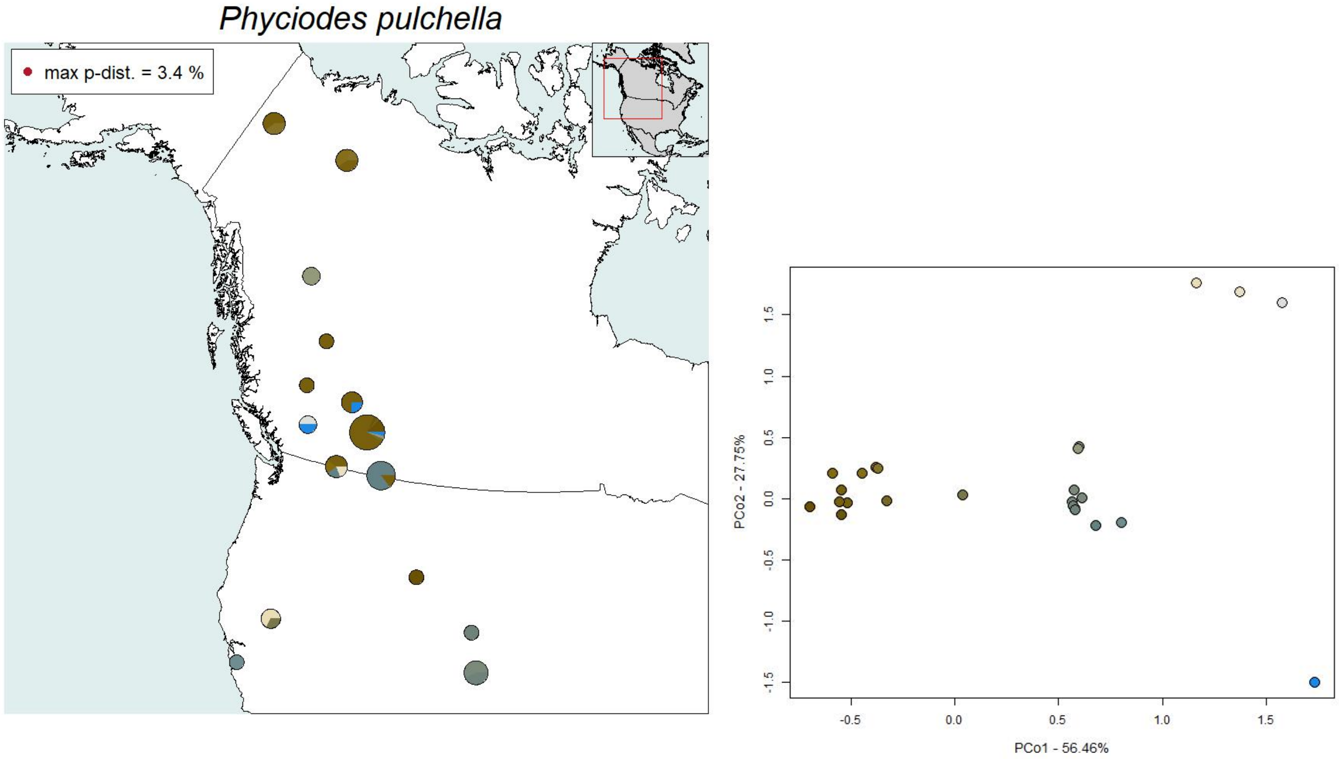

Figure 690 Map of *Phyciodes pulchella* showing the localities of the sequenced specimens (left). Nearby localities are grouped in pies. Colours match the bidimensional colour space of the PCoA projection (right) of max p-dists among sequences (dots). Sequences= 84; Hap obs.= 20; Hap asympt.= 26.7; Hap % obs.= 75%; GST= 0.538; DST= 0.0046; HD= 0.759; ND= 0.0095; max p-dist= 3.4%.

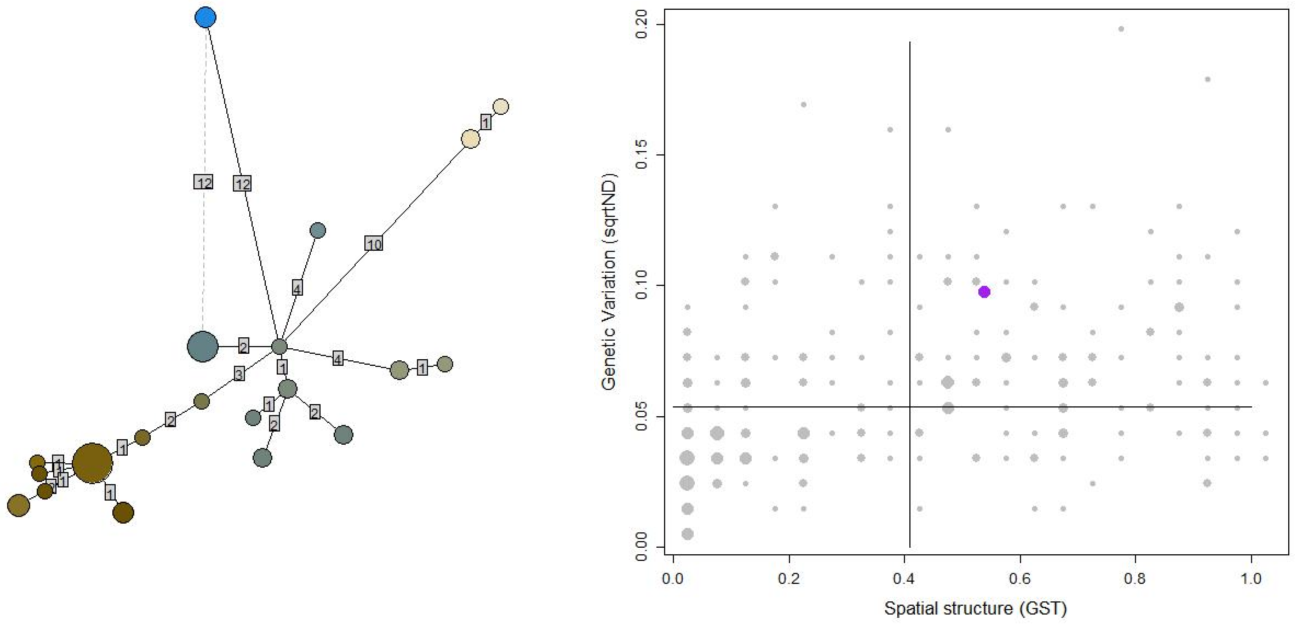

Figure 691: Haplotype network (left) of *Phyciodes pulchella* sequences > 599 bp with colours matching the PCoA colour space (above). The bubble plot for mt-DNA polymorphism (square root transformed nucleotide diversity) and spatial structure (GST) among all species in the atlas and values for *Phyciodes pulchella* (purple dot). The horizontal and vertical lines represent median values of nucleotide diversity and GST, respectively. Sequences > 599 bp= 82.

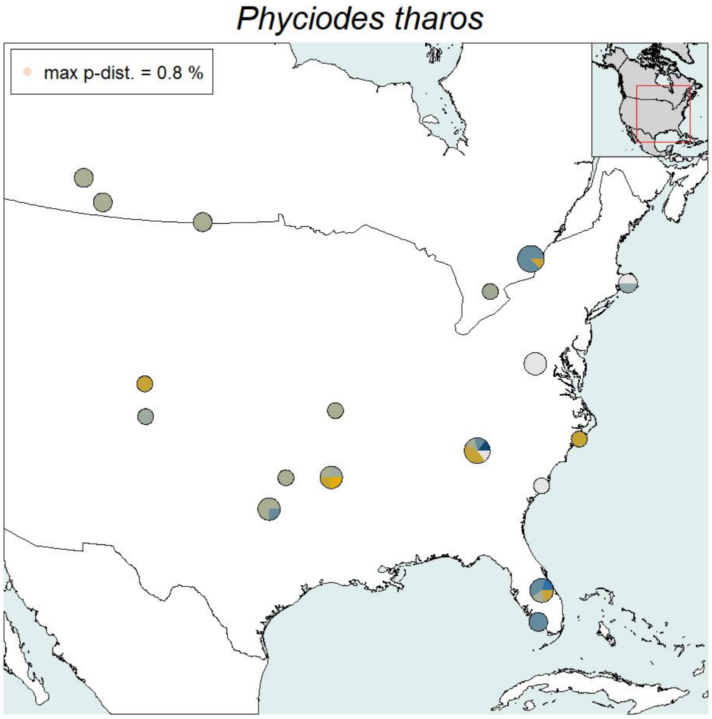

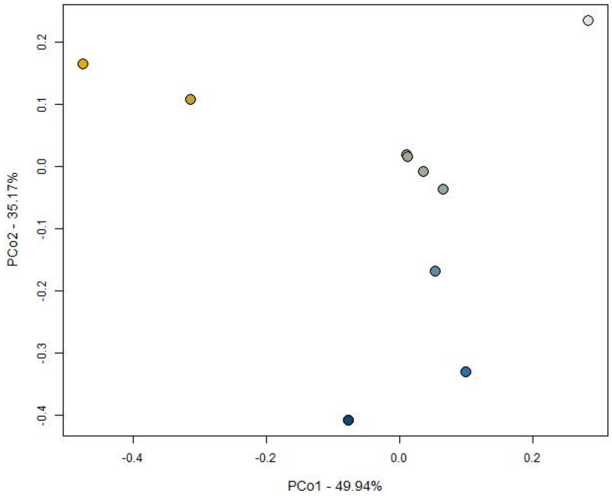

Figure 692 Map of *Phyciodes tharos* showing the localities of the sequenced specimens (left). Nearby localities are grouped in pies. Colours match the bidimensional colour space of the PCoA projection (right) of max p-dists among sequences (dots). Sequences= 49; Hap obs.= 10; Hap asympt.= 24.7; Hap % obs.= 40.5%; GST= 0.308; DST= 0.0009; HD= 0.803; ND= 0.0028; max p-dist= 0.8%.

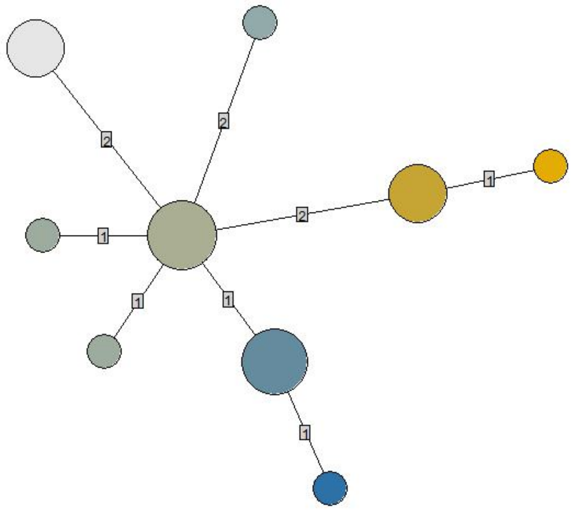

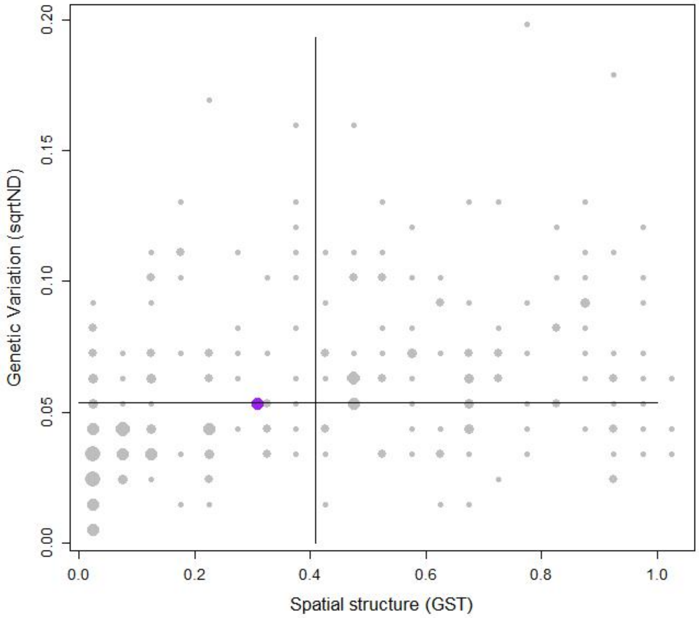

Figure 693: Haplotype network (left) of *Phyciodes tharos* sequences > 599 bp with colours matching the PCoA colour space (above). The bubble plot for mt-DNA polymorphism (square root transformed nucleotide diversity) and spatial structure (GST) among all species in the atlas and values for *Phyciodes tharos* (purple dot). The horizontal and vertical lines represent median values of nucleotide diversity and GST, respectively. Sequences > 599 bp= 48.

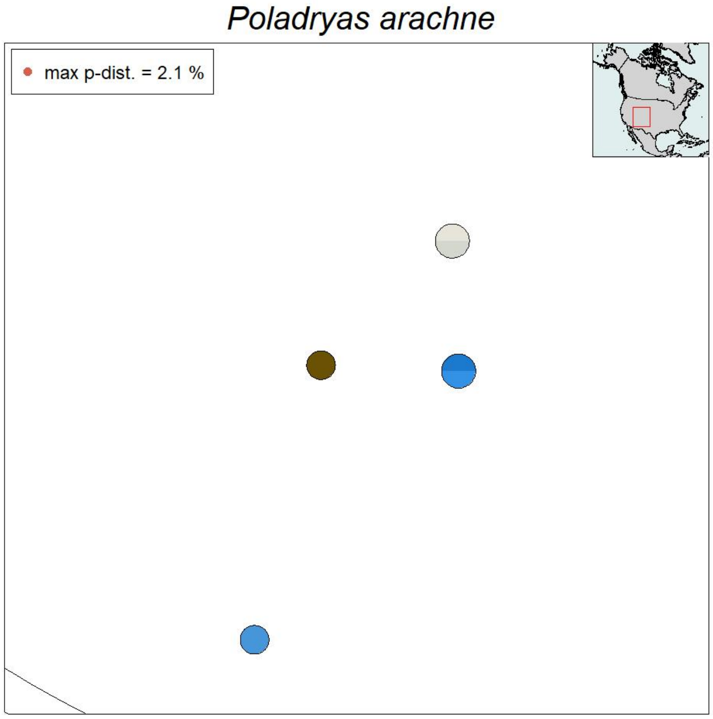

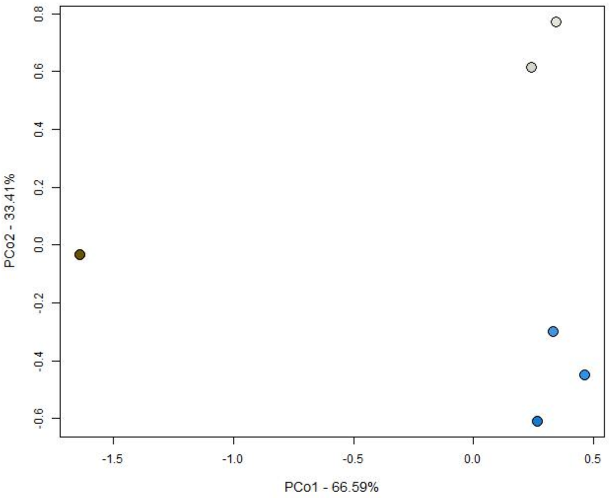

Figure 694 Map of *Poladryas arachne* showing the localities of the sequenced specimens (left). Nearby localities are grouped in pies. Colours match the bidimensional colour space of the PCoA projection (right) of max p-dists among sequences (dots). Sequences= 6; Hap obs.= 6; Hap asympt.= NA; Hap % obs.= NA%; GST= NaN; DST= NaN; HD= NA; ND= NA; max p-dist= 2.1%.

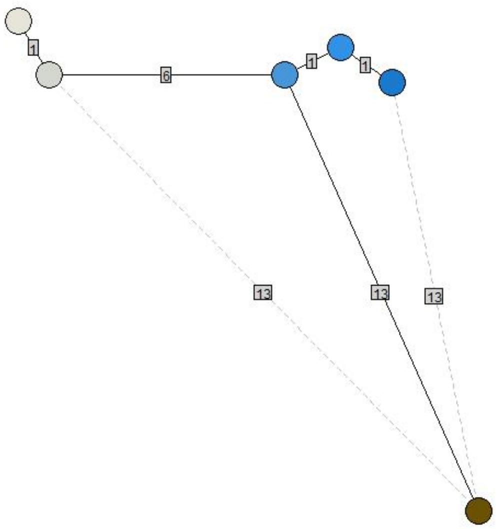

Figure 695: Haplotype network of *Poladryas arachne*. Sequences > 599 bp= 6.

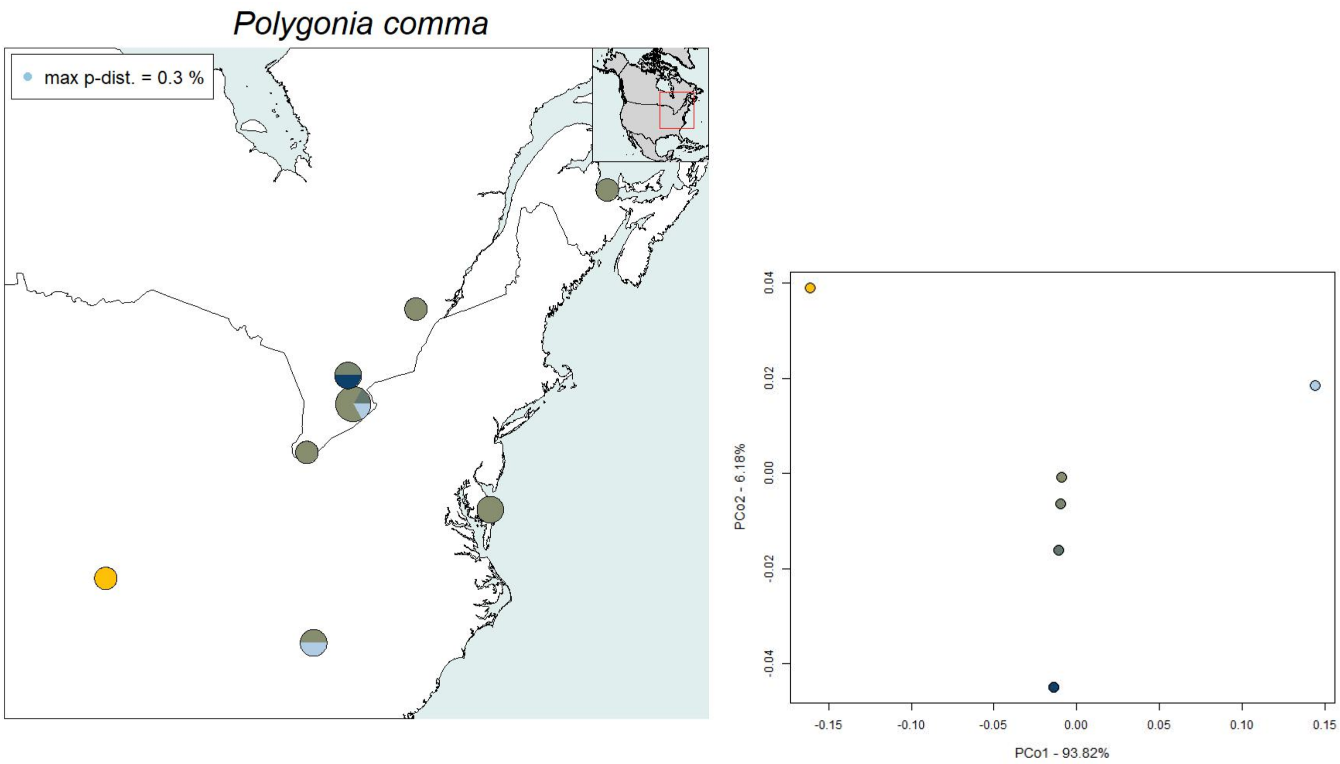

Figure 696 Map of *Polygonia comma* showing the localities of the sequenced specimens (left). Nearby localities are grouped in pies. Colours match the bidimensional colour space of the PCoA projection (right) of max p-dists among sequences (dots). Sequences= 16; Hap obs.= 3; Hap asympt.= 3.5; Hap % obs.= 86.5%; GST= NaN; DST= NaN; HD= 0.342; ND= 0.0006; max p-dist= 0.3%.

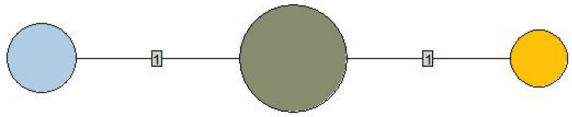

Figure 697: Haplotype network of *Polygonia comma*. Sequences > 599 bp= 15.

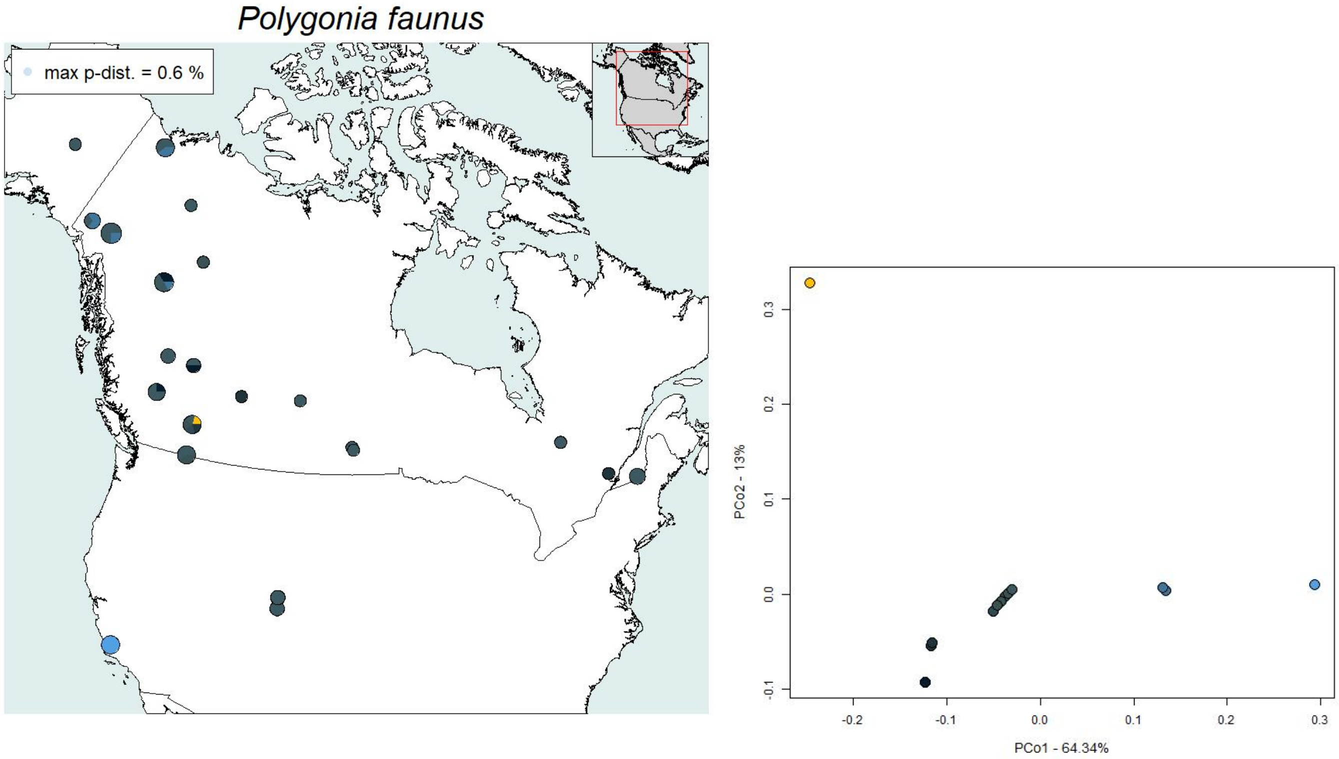

Figure 698 Map of *Polygonia faunus* showing the localities of the sequenced specimens (left). Nearby localities are grouped in pies. Colours match the bidimensional colour space of the PCoA projection (right) of max p-dists among sequences (dots). Sequences= 61; Hap obs.= 10; Hap asympt.= 16.1; Hap % obs.= 61.9%; GST= 0.511; DST= 0.0007; HD= 0.556; ND= 0.0012; max p-dist= 0.6%.

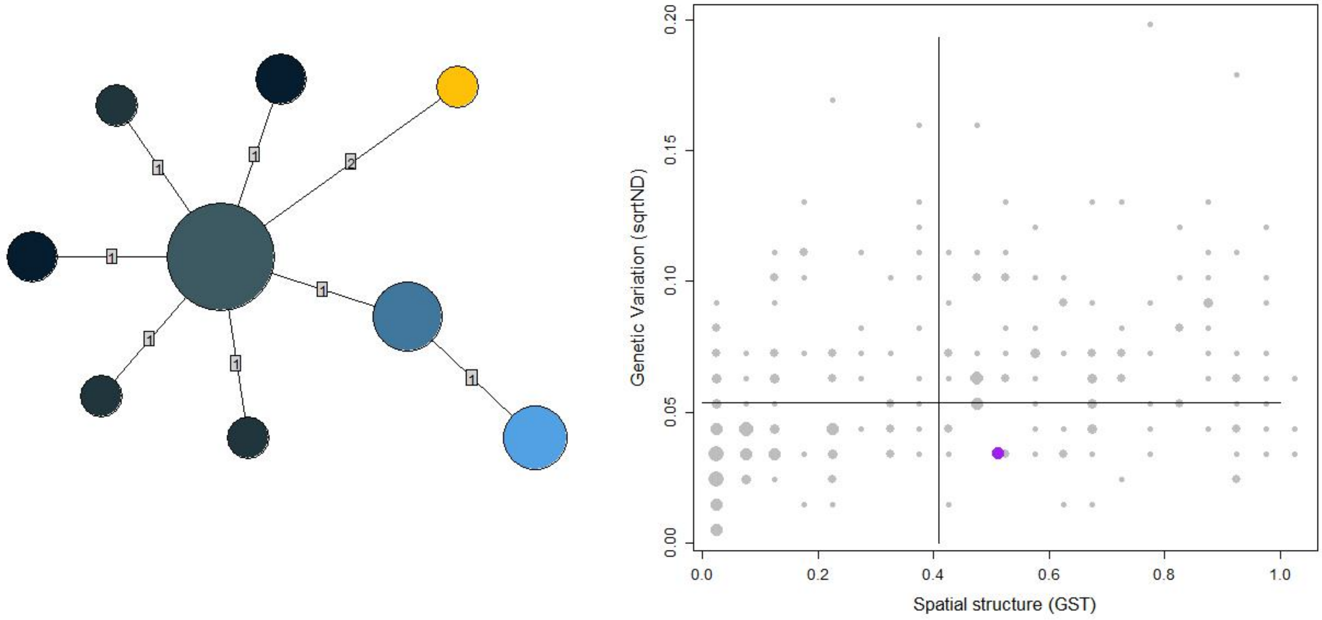

Figure 699: Haplotype network (left) of *Polygonia faunus* sequences > 599 bp with colours matching the PCoA colour space (above). The bubble plot for mt-DNA polymorphism (square root transformed nucleotide diversity) and spatial structure (GST) among all species in the atlas and values for *Polygonia faunus* (purple dot). The horizontal and vertical lines represent median values of nucleotide diversity and GST, respectively. Sequences > 599 bp= 59.

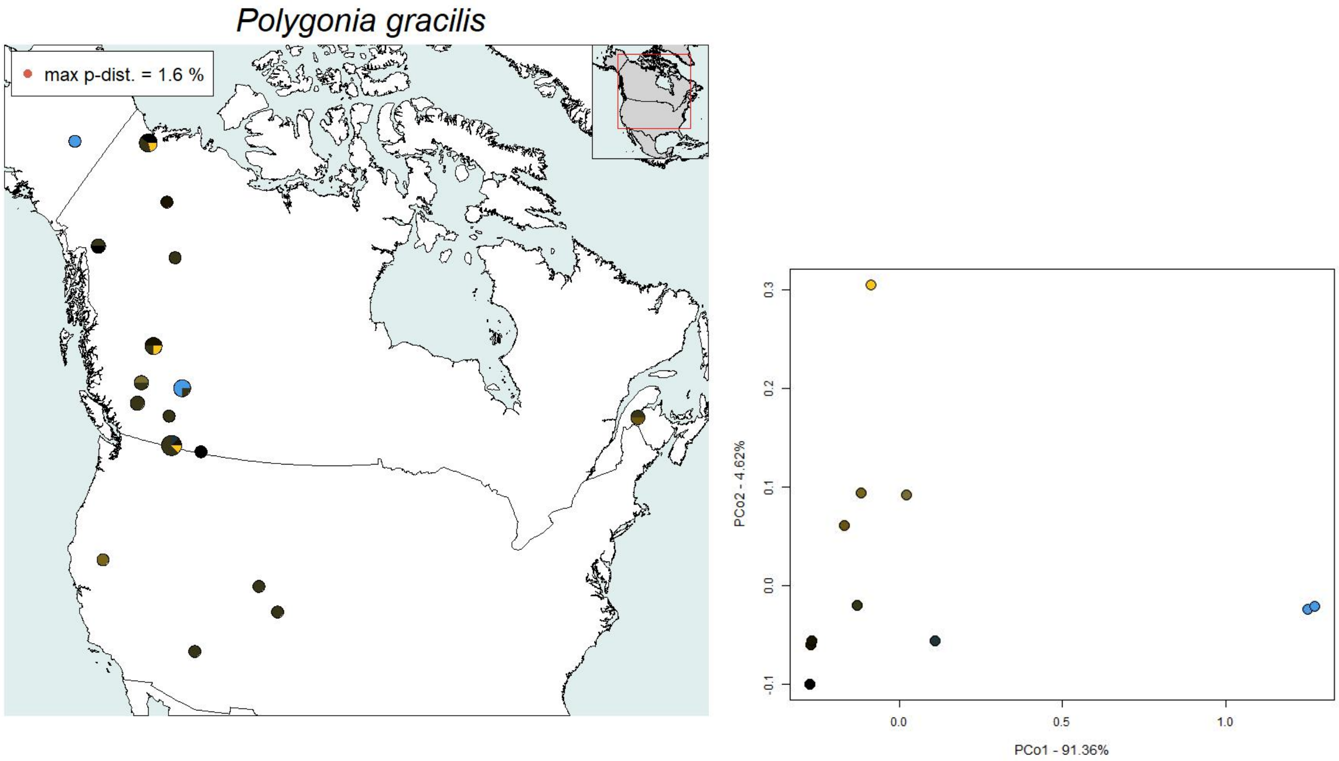

Figure 700 Map of *Polygonia gracilis* showing the localities of the sequenced specimens (left). Nearby localities are grouped in pies. Colours match the bidimensional colour space of the PCoA projection (right) of max p-dists among sequences (dots). Sequences= 38; Hap obs.= 11; Hap asympt.= 28.5; Hap % obs.= 38.6%; GST= 0.466; DST= 0.0022; HD= 0.734; ND= 0.0038; max p-dist= 1.6%.

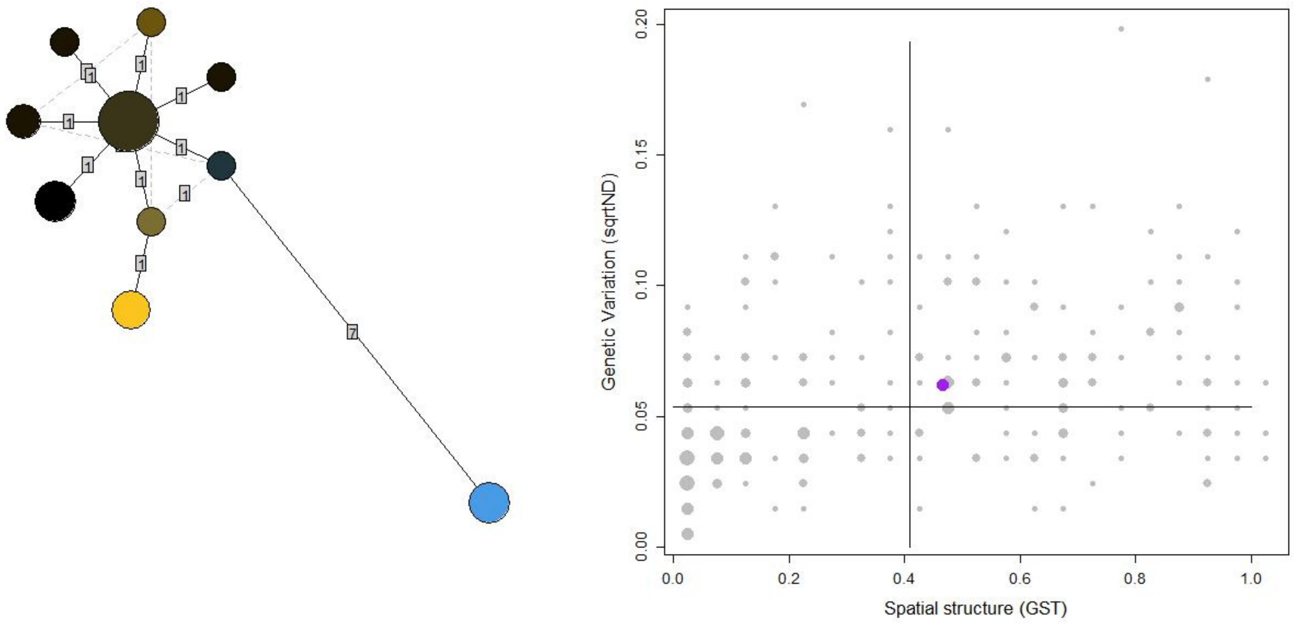

Figure 701: Haplotype network (left) of *Polygonia gracilis* sequences > 599 bp with colours matching the PCoA colour space (above). The bubble plot for mt-DNA polymorphism (square root transformed nucleotide diversity) and spatial structure (GST) among all species in the atlas and values for *Polygonia gracilis* (purple dot). The horizontal and vertical lines represent median values of nucleotide diversity and GST, respectively. Sequences > 599 bp= 37.

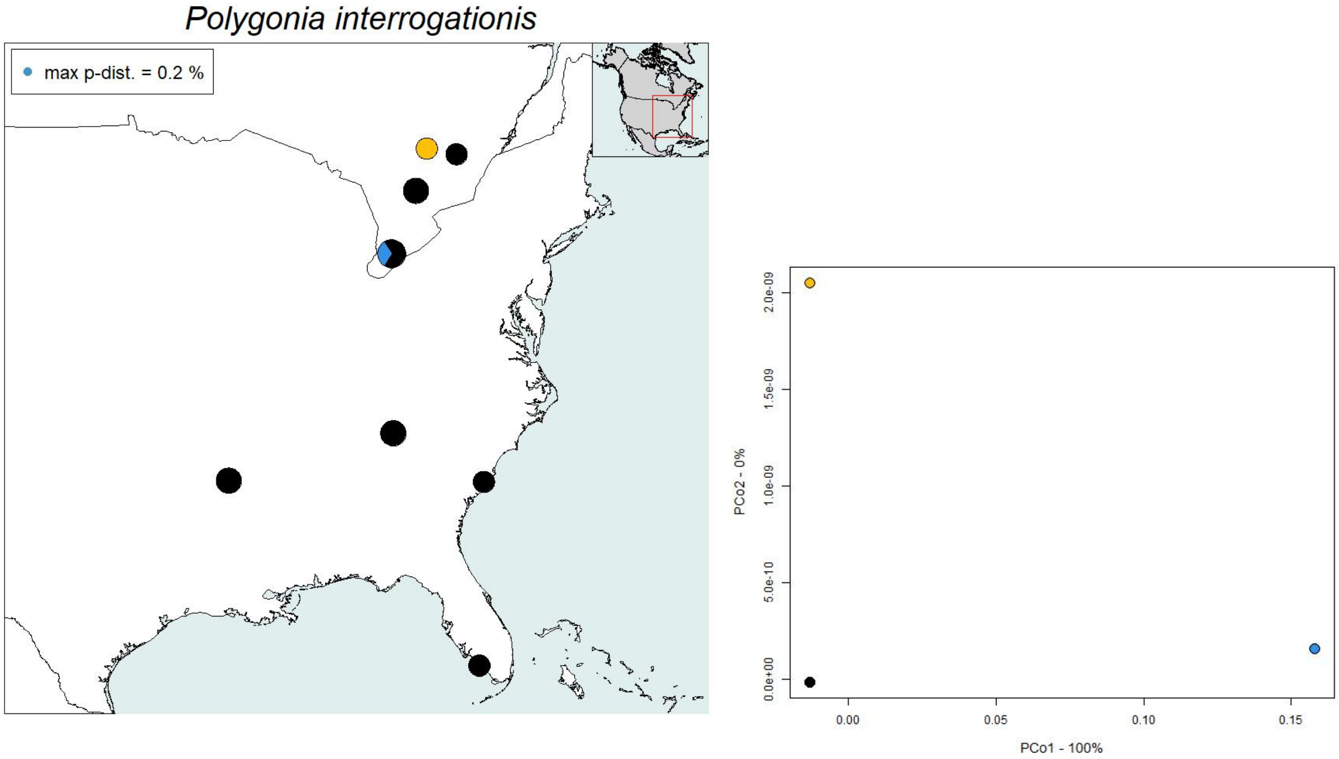

Figure 702 Map of *Polygonia interrogationis* showing the localities of the sequenced specimens (left). Nearby localities are grouped in pies. Colours match the bidimensional colour space of the PCoA projection (right) of max p-dists among sequences (dots). Sequences= 13; Hap obs.= 2; Hap asympt.= 2; Hap % obs.= 100%; GST= NaN; DST= NaN; HD= 0.154; ND= 0.0003; max p-dist= 0.2%.

Haplotype network analysis and bubble plot of *Polygonia interrogationis* were not possible. Sequences > 599 bp = 11.

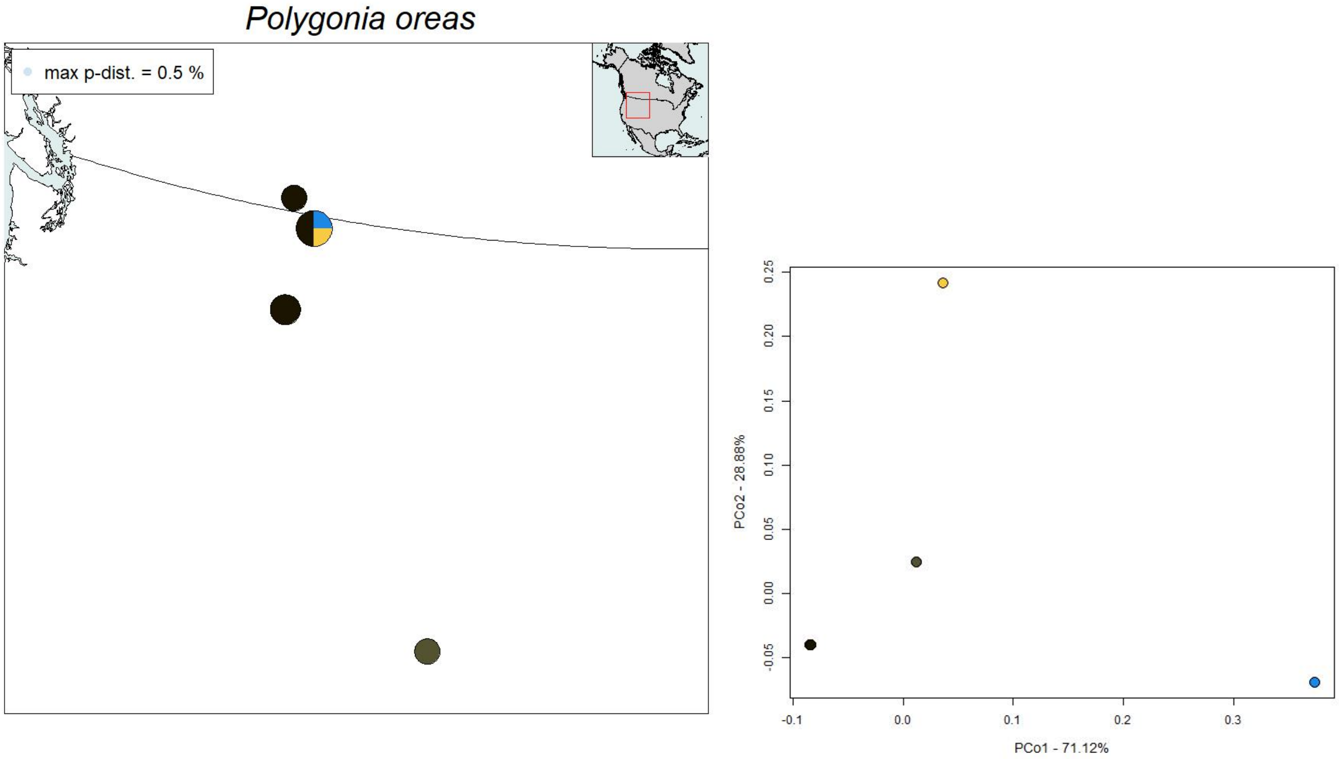

Figure 703 Map of *Polygonia oreas* showing the localities of the sequenced specimens (left). Nearby localities are grouped in pies. Colours match the bidimensional colour space of the PCoA projection (right) of max p-dists among sequences (dots). Sequences= 8; Hap obs.= 3; Hap asympt.= NA; Hap % obs.= NA%; GST= NaN; DST= NaN; HD= NA; ND= NA; max p-dist= 0.5%.

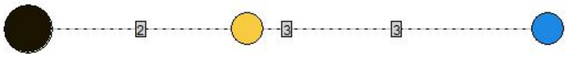

Figure 704: Haplotype network of *Polygonia oreas*. Sequences > 599 bp= 7.

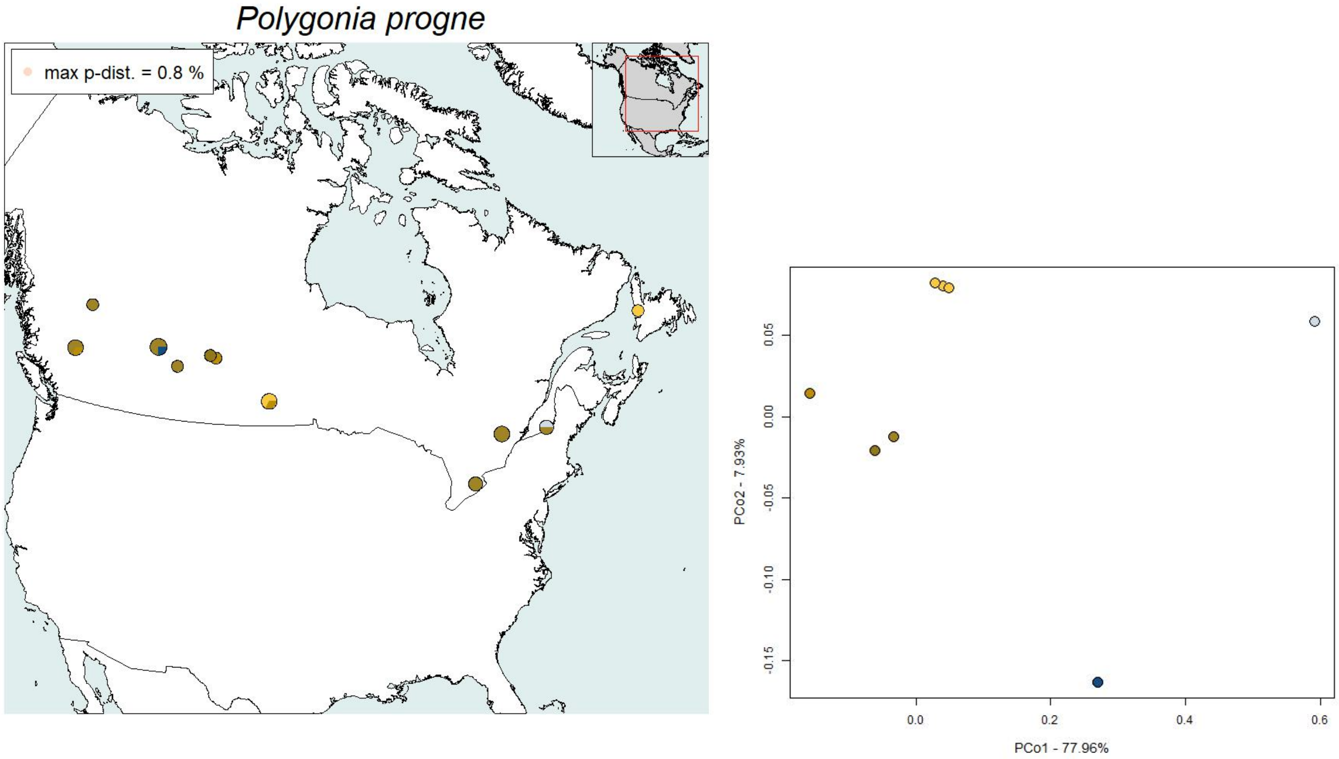

Figure 705 Map of *Polygonia progne* showing the localities of the sequenced specimens (left). Nearby localities are grouped in pies. Colours match the bidimensional colour space of the PCoA projection (right) of max p-dists among sequences (dots). Sequences= 22; Hap obs.= 6; Hap asympt.= 15.5; Hap % obs.= 38.6%; GST= 0; DST= 0; HD= 0.411; ND= 0.0012; max p-dist= 0.8%.

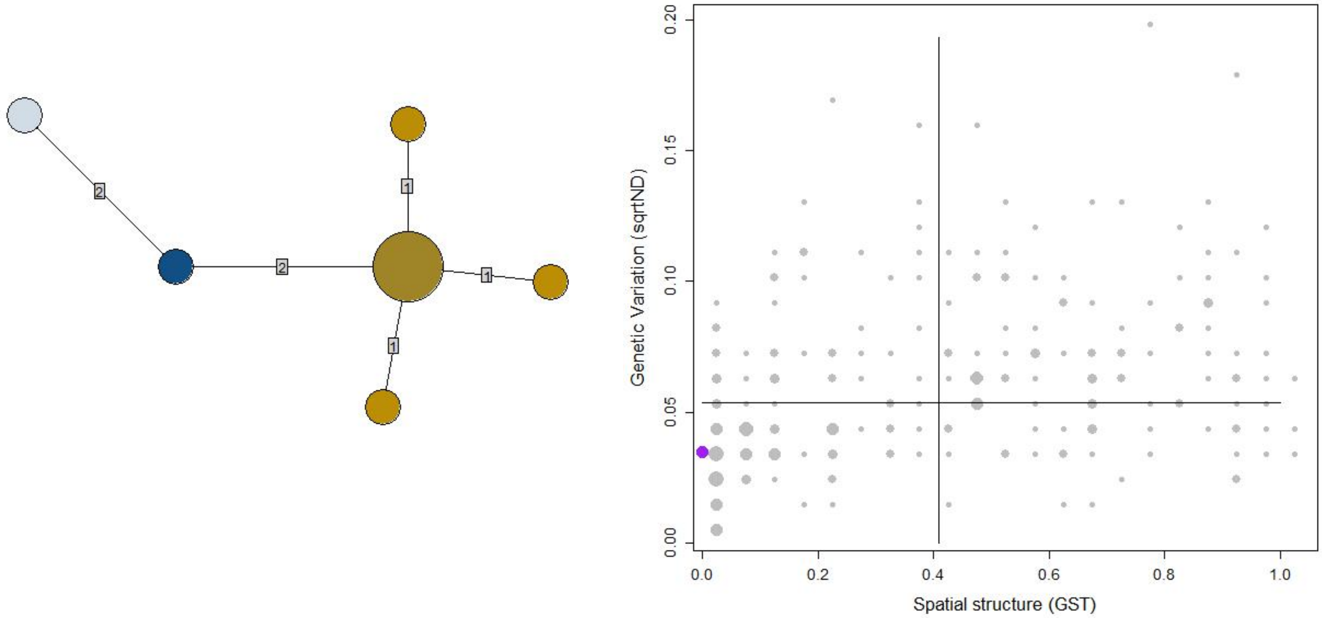

Figure 706: Haplotype network (left) of *Polygonia progne* sequences > 599 bp with colours matching the PCoA colour space (above). The bubble plot for mt-DNA polymorphism (square root transformed nucleotide diversity) and spatial structure (GST) among all species in the atlas and values for *Polygonia progne* (purple dot). The horizontal and vertical lines represent median values of nucleotide diversity and GST, respectively. Sequences > 599 bp= 21.

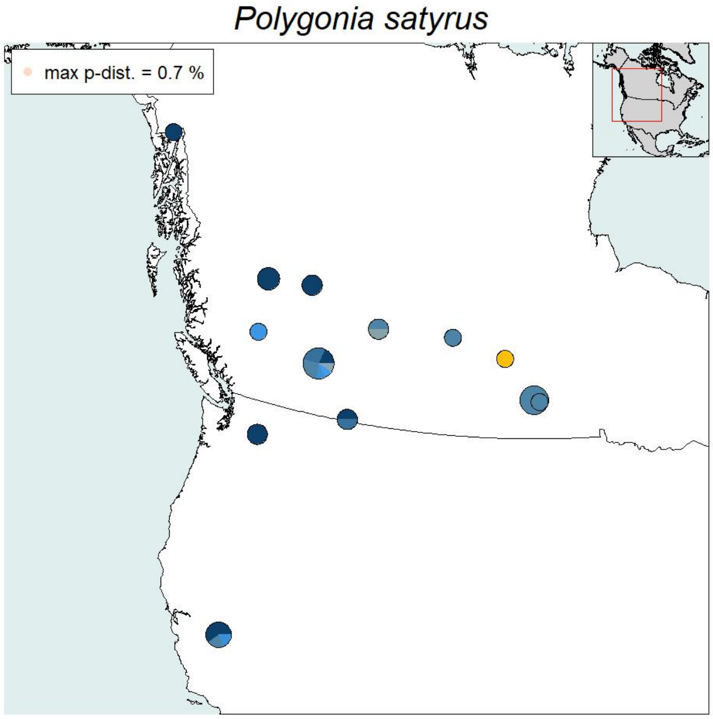

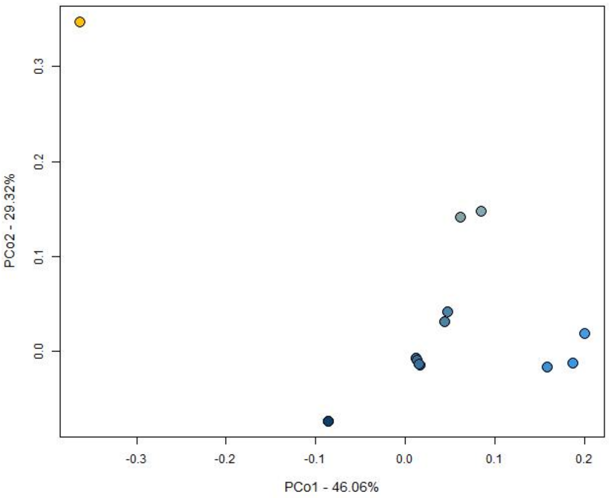

Figure 707 Map of *Polygonia satyrus* showing the localities of the sequenced specimens (left). Nearby localities are grouped in pies. Colours match the bidimensional colour space of the PCoA projection (right) of max p-dists among sequences (dots). Sequences= 40; Hap obs.= 10; Hap asympt.= 27.6; Hap % obs.= 36.3%; GST= 0.354; DST= 0.0004; HD= 0.758; ND= 0.0013; max p-dist= 0.7%.

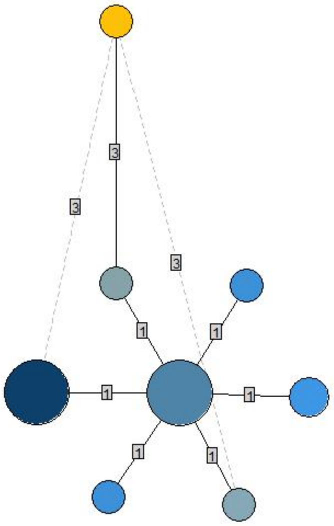

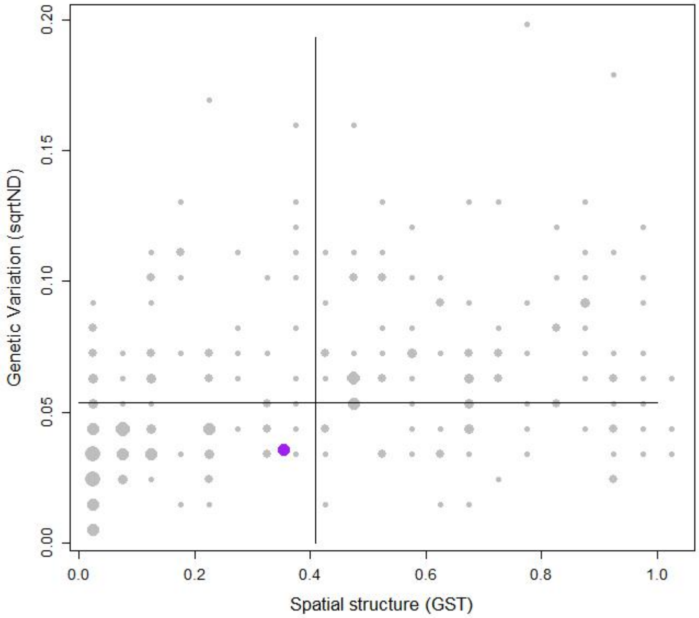

Figure 708: Haplotype network (left) of *Polygonia satyrus* sequences > 599 bp with colours matching the PCoA colour space (above). The bubble plot for mt-DNA polymorphism (square root transformed nucleotide diversity) and spatial structure (GST) among all species in the atlas and values for *Polygonia satyrus* (purple dot). The horizontal and vertical lines represent median values of nucleotide diversity and GST, respectively. Sequences > 599 bp= 36.

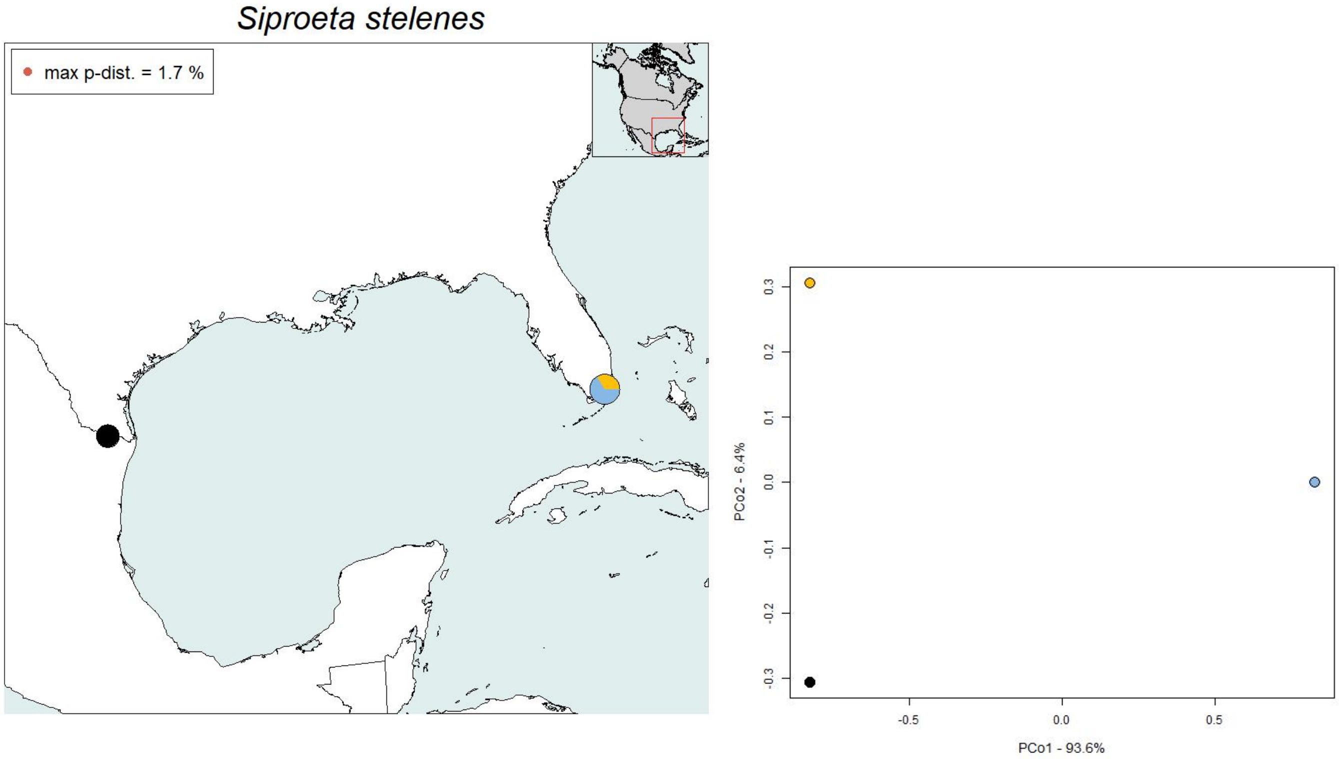

Figure 709 Map of *Siproeta stelenes* showing the localities of the sequenced specimens (left). Nearby localities are grouped in pies. Colours match the bidimensional colour space of the PCoA projection (right) of max p-dists among sequences (dots). Sequences= 4; Hap obs.= 3; Hap asympt.= NA; Hap % obs.= NA%; GST= NaN; DST= NaN; HD= NA; ND= NA; max p-dist= 1.7%.

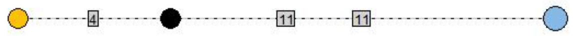

Figure 710: Haplotype network of *Siproeta stelenes*. Sequences > 599 bp= 4.

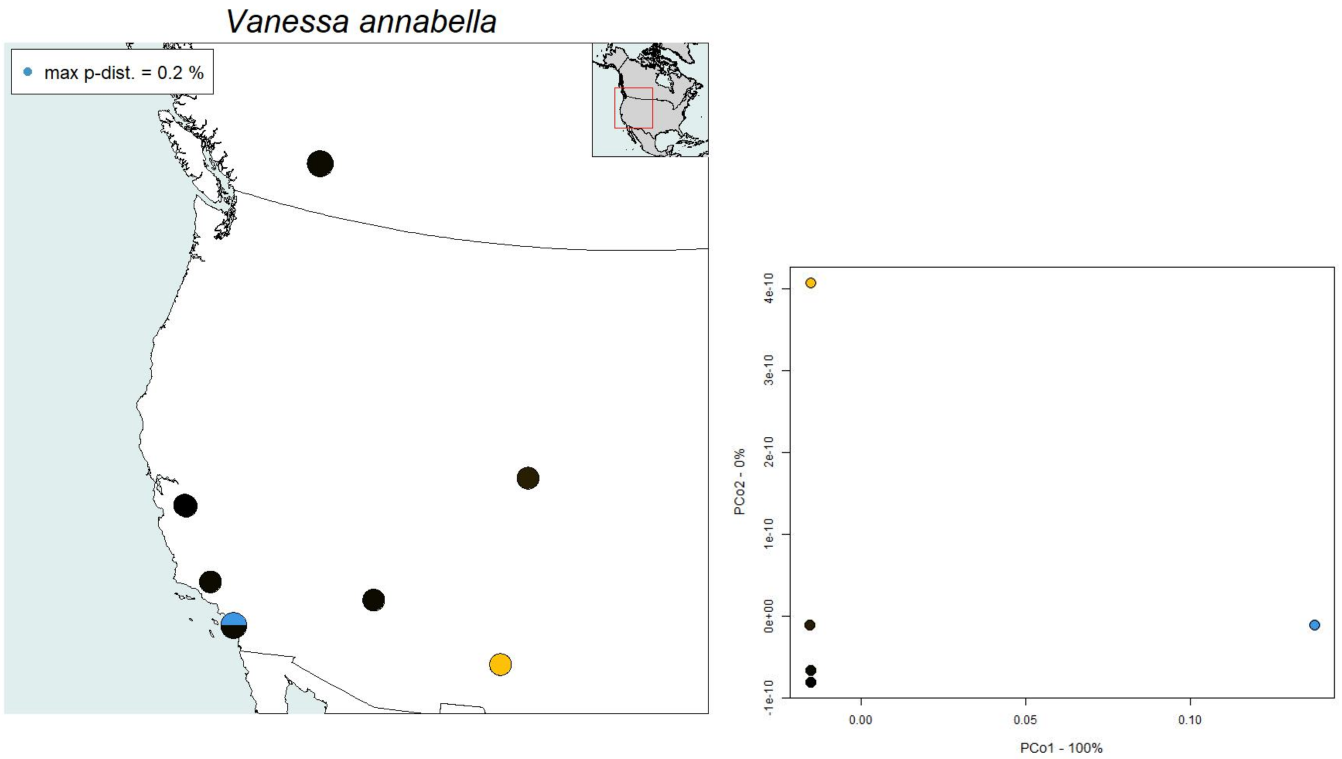

Figure 711 Map of *Vanessa annabella* showing the localities of the sequenced specimens (left). Nearby localities are grouped in pies. Colours match the bidimensional colour space of the PCoA projection (right) of max p-dists among sequences (dots). Sequences= 10; Hap obs.= 2; Hap asympt.= 2; Hap % obs.= 100%; GST= NaN; DST= NaN; HD= 0.2; ND= 0.0003; max p-dist= 0.2%.

Haplotype network analysis and bubble plot of *Vanessa annabella* were not possible. Sequences > 599 bp = 10.

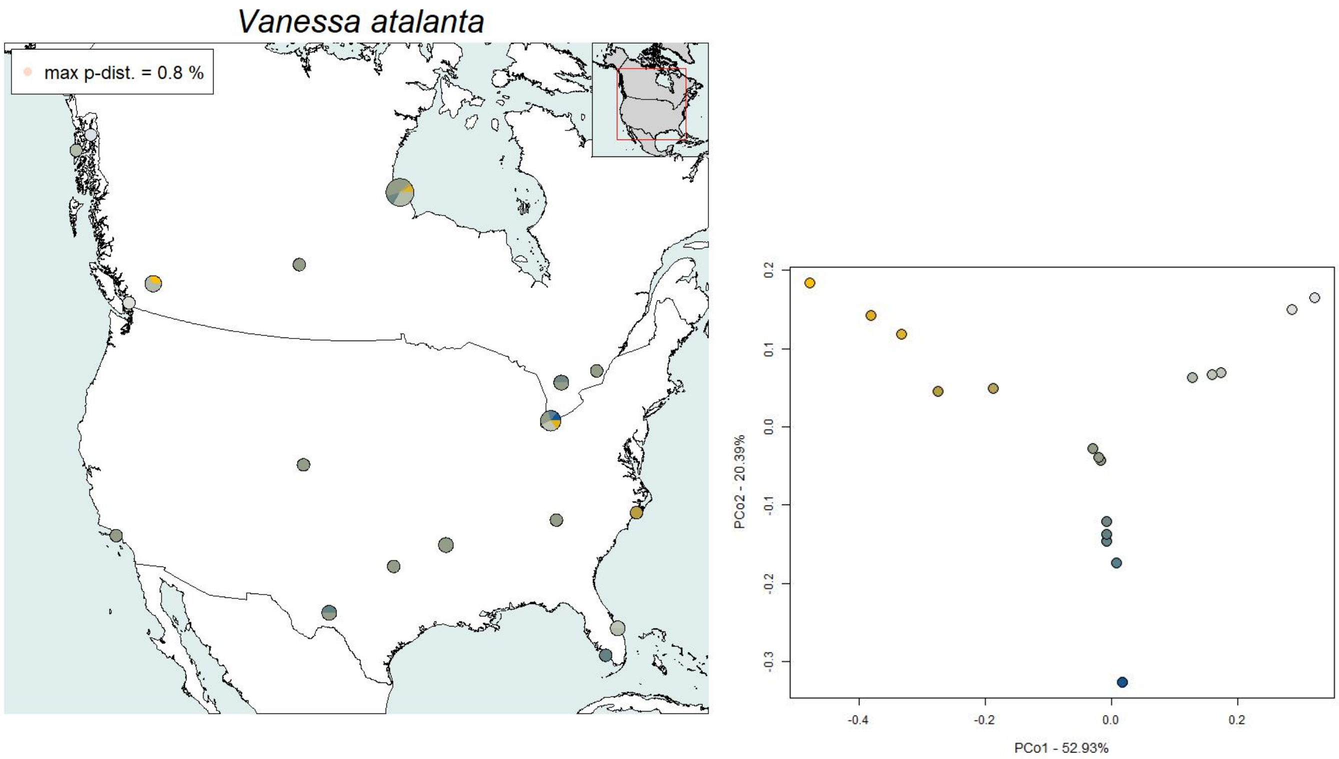

Figure 712 Map of *Vanessa atalanta* showing the localities of the sequenced specimens (left). Nearby localities are grouped in pies. Colours match the bidimensional colour space of the PCoA projection (right) of max p-dists among sequences (dots). Sequences= 53; Hap obs.= 13; Hap asympt.= 21; Hap % obs.= 61.9%; GST= 0.091; DST= 0.0002; HD= 0.752; ND= 0.002; max p-dist= 0.8%.

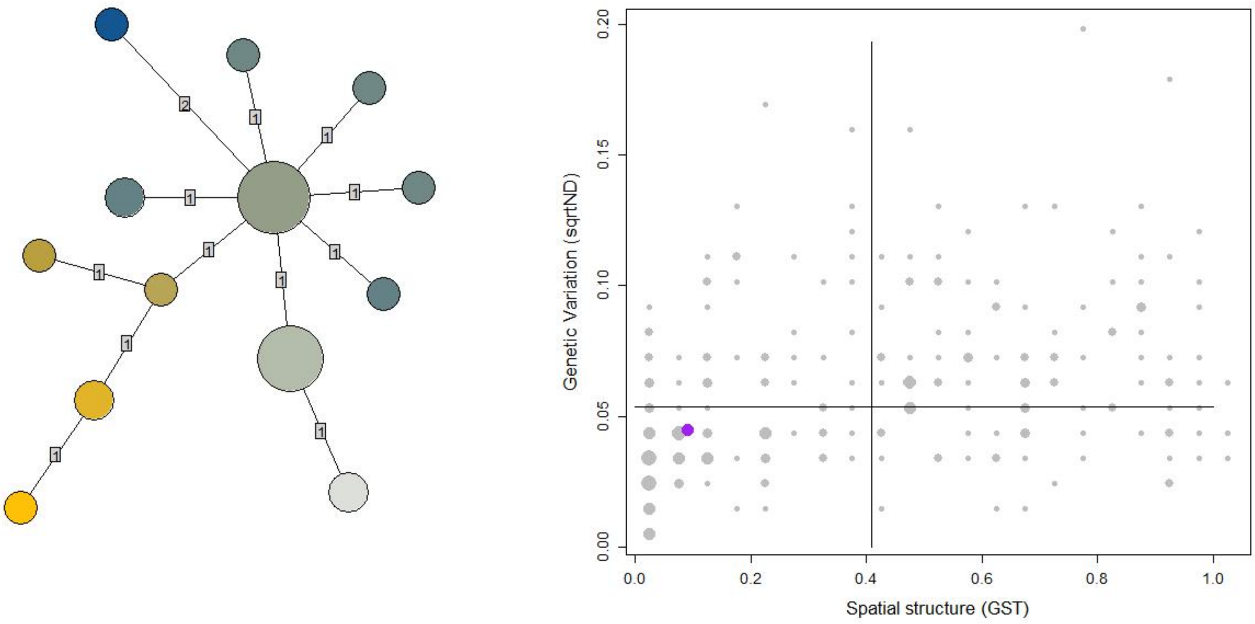

Figure 713: Haplotype network (left) of *Vanessa atalanta* sequences > 599 bp with colours matching the PCoA colour space (above). The bubble plot for mt-DNA polymorphism (square root transformed nucleotide diversity) and spatial structure (GST) among all species in the atlas and values for *Vanessa atalanta* (purple dot). The horizontal and vertical lines represent median values of nucleotide diversity and GST, respectively. Sequences > 599 bp= 49.

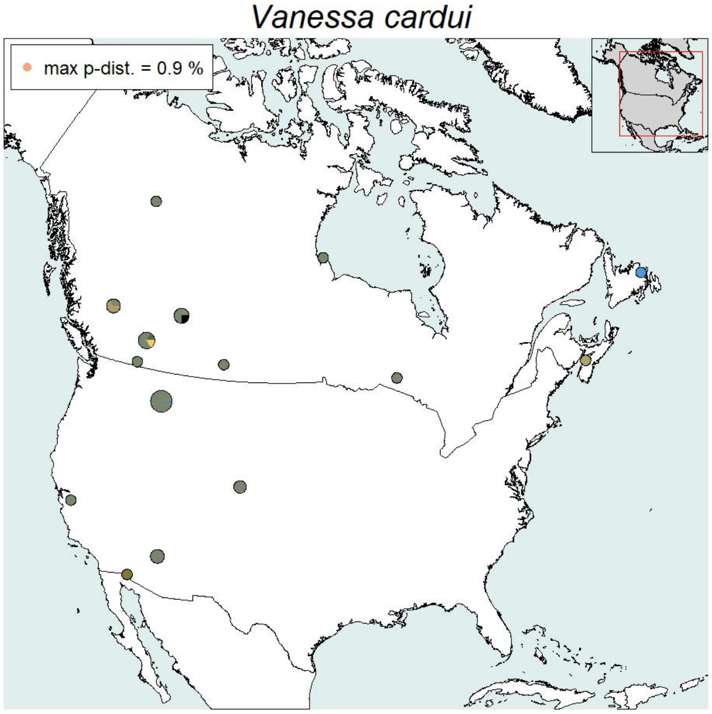

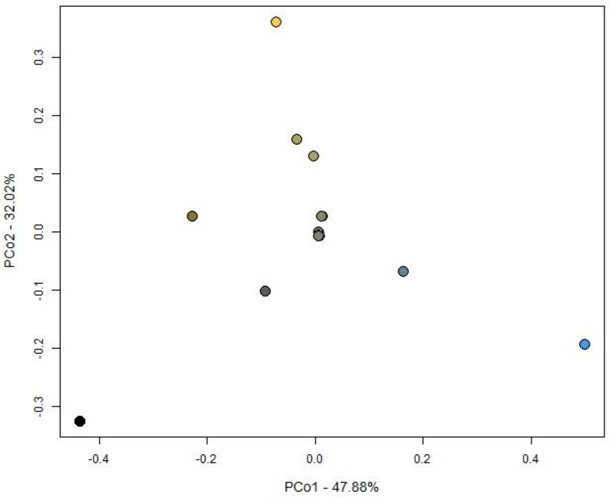

Figure 714 Map of *Vanessa cardui* showing the localities of the sequenced specimens (left). Nearby localities are grouped in pies. Colours match the bidimensional colour space of the PCoA projection (right) of max p-dists among sequences (dots). Sequences= 45; Hap obs.= 8; Hap asympt.= 20.2; Hap % obs.= 39.6%; GST= 0.41; DST= 0.0003; HD= 0.395; ND= 0.001; max p-dist= 0.9%.

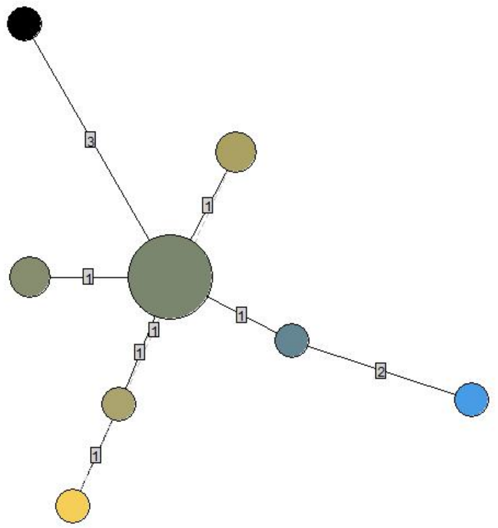

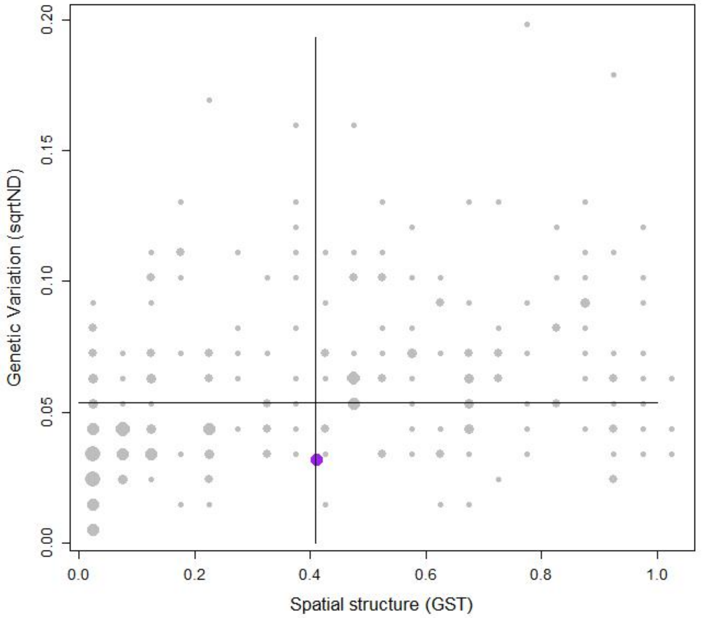

Figure 715: Haplotype network (left) of *Vanessa cardui* sequences > 599 bp with colours matching the PCoA colour space (above). The bubble plot for mt-DNA polymorphism (square root transformed nucleotide diversity) and spatial structure (GST) among all species in the atlas and values for *Vanessa cardui* (purple dot). The horizontal and vertical lines represent median values of nucleotide diversity and GST, respectively. Sequences > 599 bp= 44.

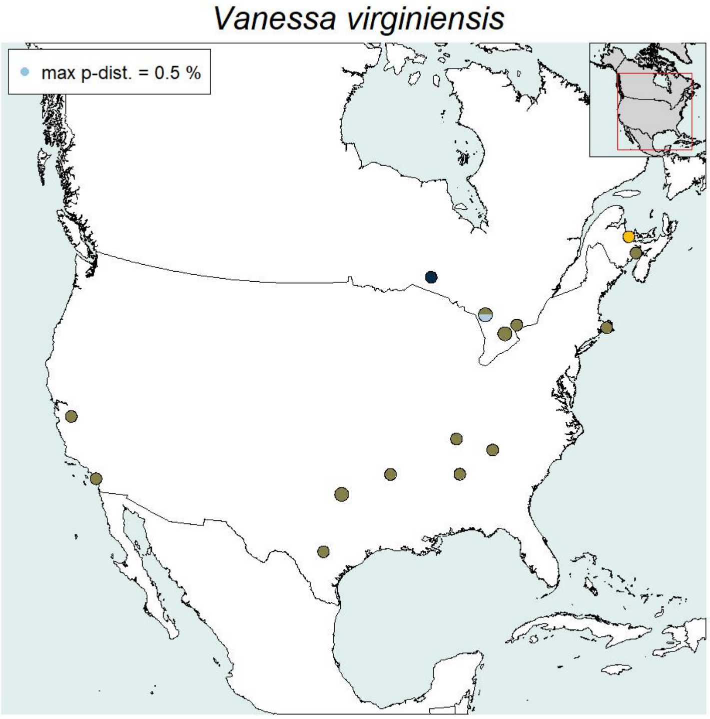

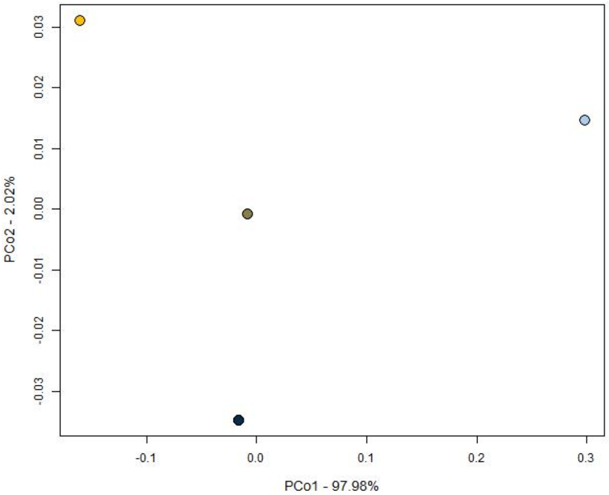

Figure 716 Map of *Vanessa virginiensis* showing the localities of the sequenced specimens (left). Nearby localities are grouped in pies. Colours match the bidimensional colour space of the PCoA projection (right) of max p-dists among sequences (dots). Sequences= 18; Hap obs.= 3; Hap asympt.= 3.9; Hap % obs.= 76.1%; GST= NaN; DST= NaN; HD= 0.216; ND= 0.0005; max p-dist= 0.5%.

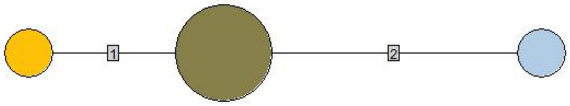

Figure 717: Haplotype network of *Vanessa virginiensis*. Sequences > 599 bp= 18.

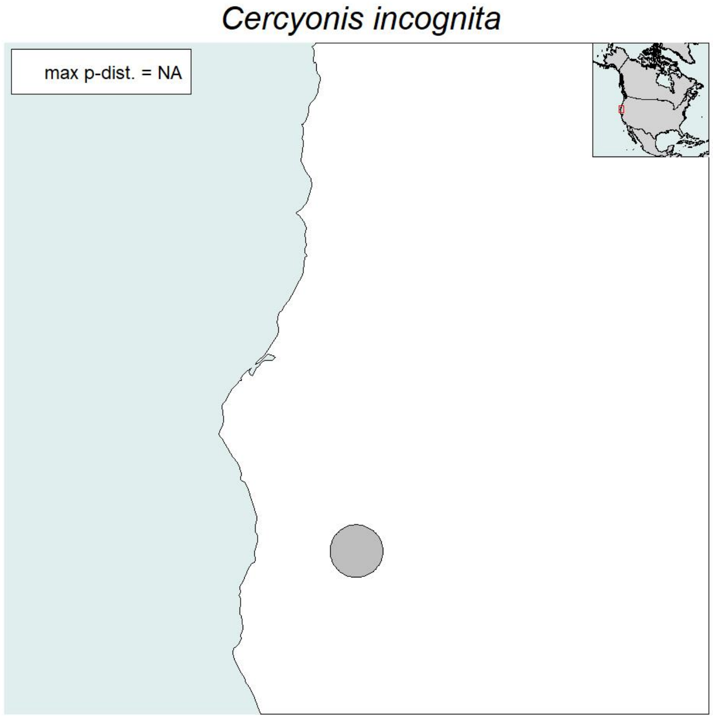

Figure 718: Map of *Cercyonis incognita* showing the localities of the sequenced specimens. Nearby localities are grouped in pies. Due to the presence of a single haplotype PCoA projection was not done and a single grey colour was plotted on the map. Sequences= 1; Hap obs.= NA; Hap asympt.= NA; Hap % obs.= NA; GST= NaN; DST= NaN; HD= NA; ND= NA; max p-dist= NA.

Haplotype network analysis and bubble plot of *Cercyonis incognita* were not possible. Sequences > 599 bp = 1.

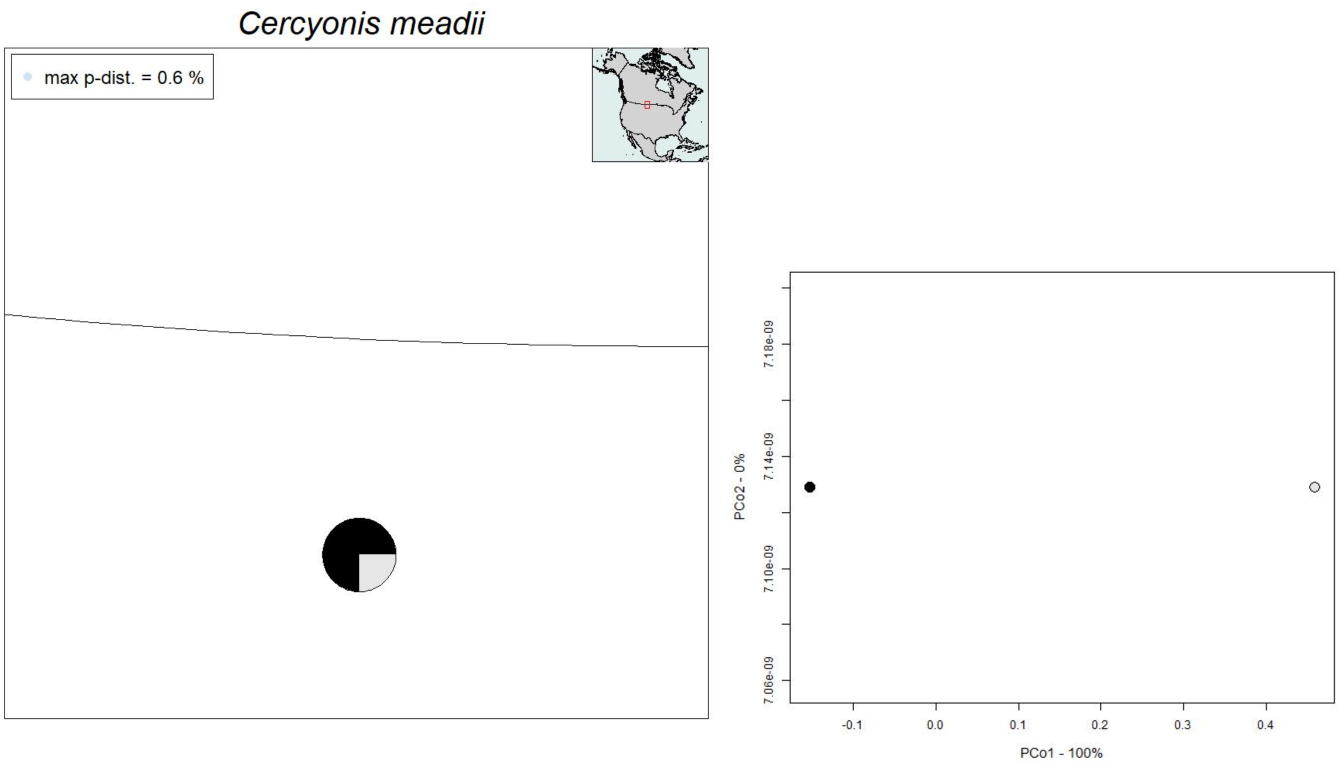

Figure 719 Map of *Cercyonis meadii* showing the localities of the sequenced specimens (left). Nearby localities are grouped in pies. Colours match the bidimensional colour space of the PCoA projection (right) of max p-dists among sequences (dots). Sequences= 4; Hap obs.= 2; Hap asympt.= NA; Hap % obs.= NA%; GST= NaN; DST= NaN; HD= NA; ND= NA; max p-dist= 0.6%.

Haplotype network analysis and bubble plot of *Cercyonis meadii* were not possible. Sequences > 599 bp = 4.

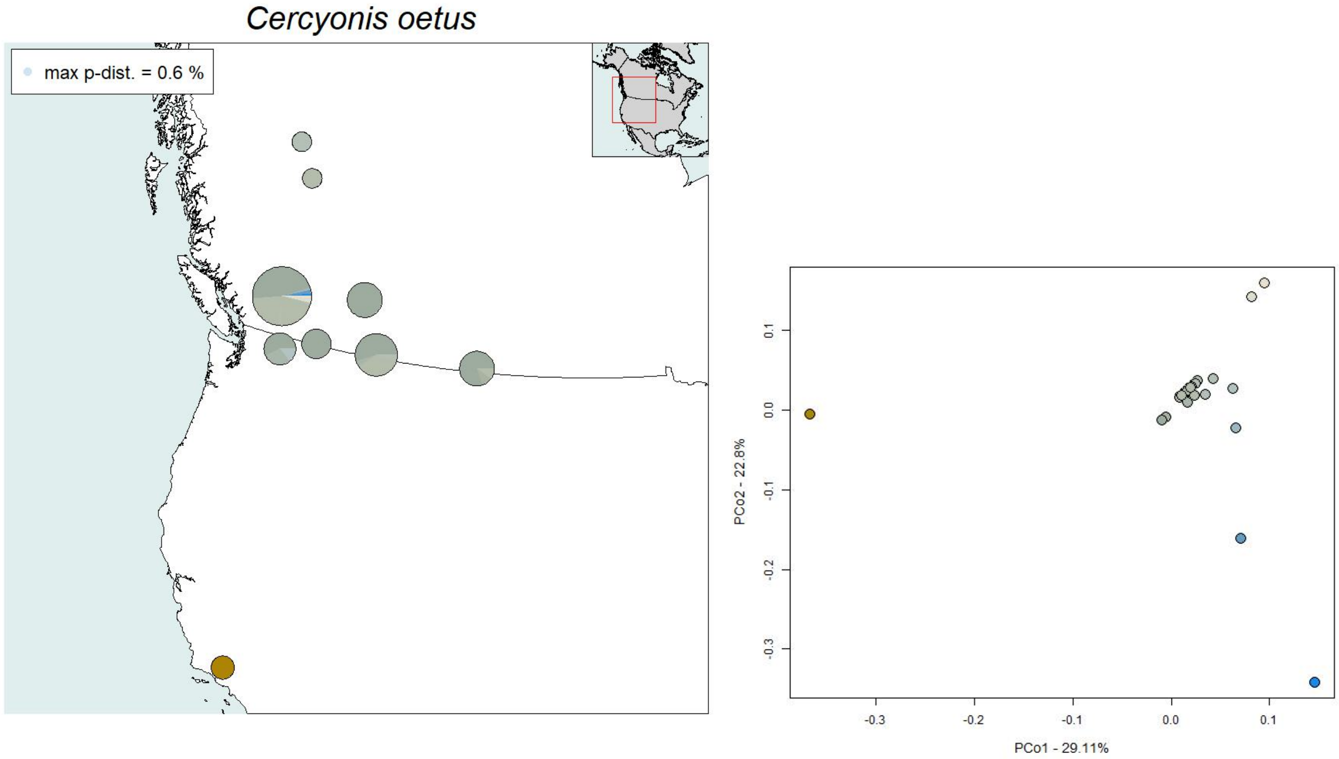

Figure 720 Map of *Cercyonis oetus* showing the localities of the sequenced specimens (left). Nearby localities are grouped in pies. Colours match the bidimensional colour space of the PCoA projection (right) of max p-dists among sequences (dots). Sequences= 140; Hap obs.= 13; Hap asympt.= 21.1; Hap % obs.= 61.6%; GST= 0.095; DST= 0; HD= 0.584; ND= 0.0004; max p-dist= 0.6%.

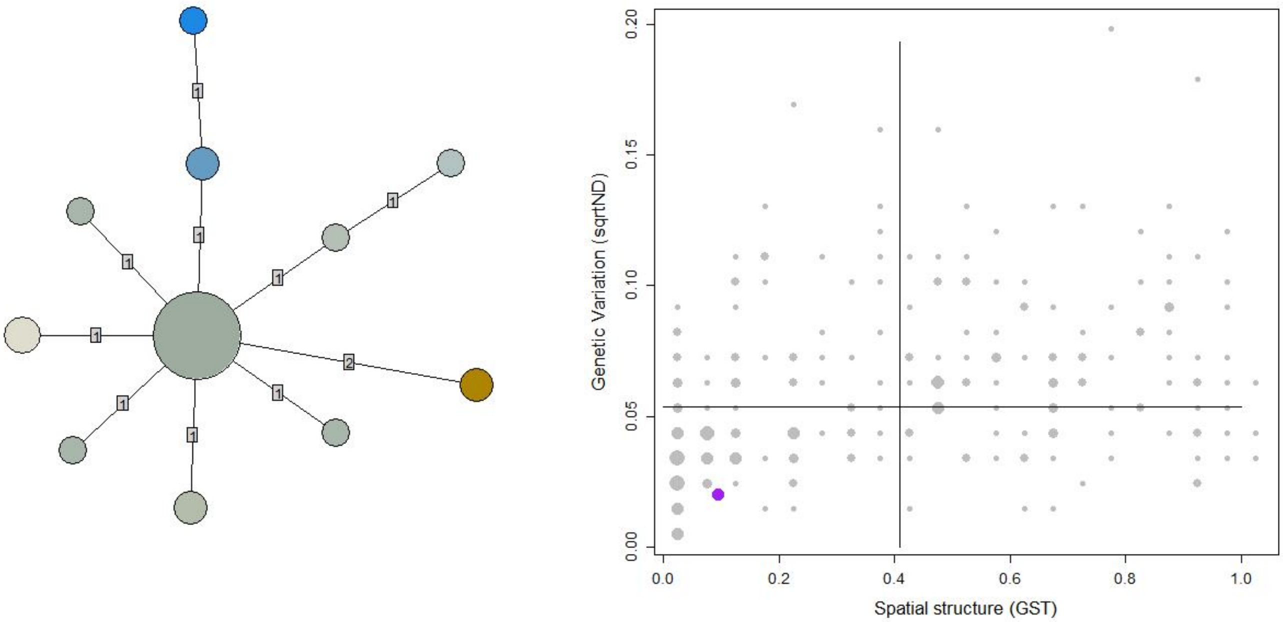

Figure 721: Haplotype network (left) of *Cercyonis oetus* sequences > 599 bp with colours matching the PCoA colour space (above). The bubble plot for mt-DNA polymorphism (square root transformed nucleotide diversity) and spatial structure (GST) among all species in the atlas and values for *Cercyonis oetus* (purple dot). The horizontal and vertical lines represent median values of nucleotide diversity and GST, respectively. Sequences > 599 bp= 114.

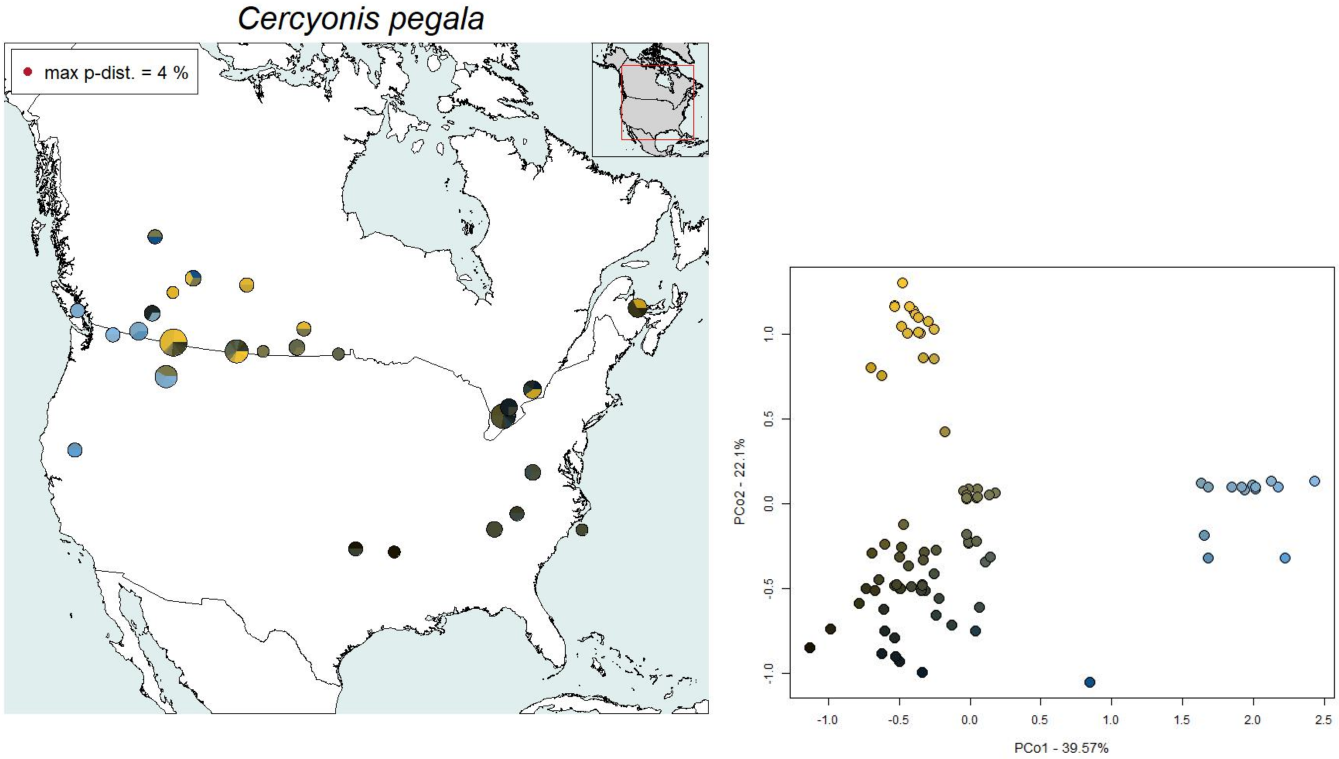

Figure 722 Map of *Cercyonis pegala* showing the localities of the sequenced specimens (left). Nearby localities are grouped in pies. Colours match the bidimensional colour space of the PCoA projection (right) of max p-dists among sequences (dots). Sequences= 126; Hap obs.= 63; Hap asympt.= 135.9; Hap % obs.= 46.4%; GST= 0.383; DST= 0.0059; HD= 0.962; ND= 0.0164; max p-dist= 4%.

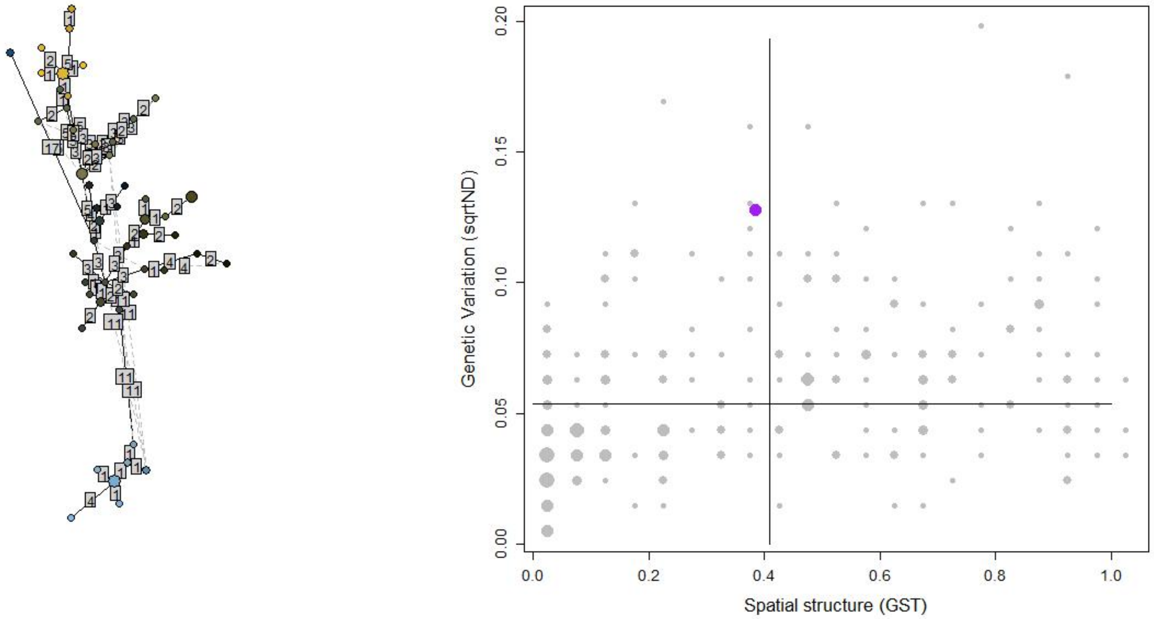

Figure 723: Haplotype network (left) of *Cercyonis pegala* sequences > 599 bp with colours matching the PCoA colour space (above). The bubble plot for mt-DNA polymorphism (square root transformed nucleotide diversity) and spatial structure (GST) among all species in the atlas and values for *Cercyonis pegala* (purple dot). The horizontal and vertical lines represent median values of nucleotide diversity and GST, respectively. Sequences > 599 bp= 92.

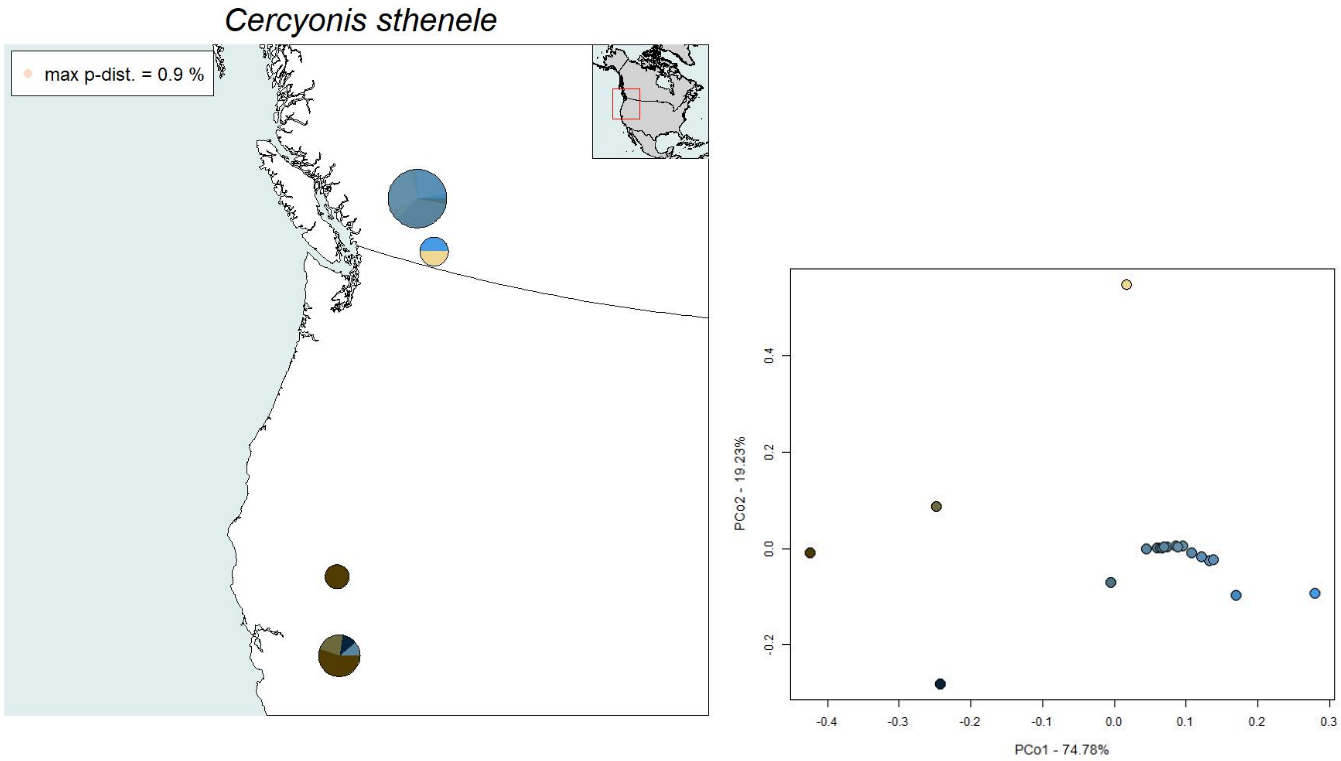

Figure 724 Map of *Cercyonis sthenele* showing the localities of the sequenced specimens (left). Nearby localities are grouped in pies. Colours match the bidimensional colour space of the PCoA projection (right) of max p-dists among sequences (dots). Sequences= 47; Hap obs.= 8; Hap asympt.= 15.8; Hap % obs.= 50.5%; GST= 0.857; DST= 0.0013; HD= 0.524; ND= 0.0018; max p-dist= 0.9%.

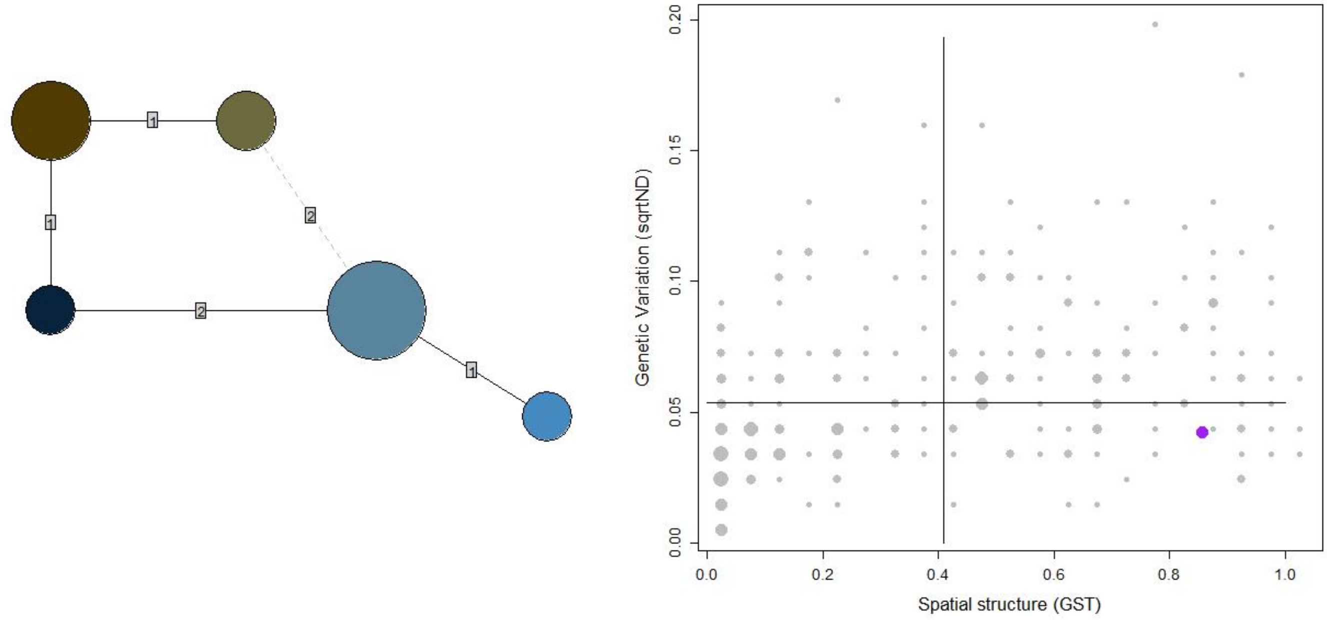

Figure 725: Haplotype network (left) of *Cercyonis sthenele* sequences > 599 bp with colours matching the PCoA colour space (above). The bubble plot for mt-DNA polymorphism (square root transformed nucleotide diversity) and spatial structure (GST) among all species in the atlas and values for *Cercyonis sthenele* (purple dot). The horizontal and vertical lines represent median values of nucleotide diversity and GST, respectively. Sequences > 599 bp= 25.

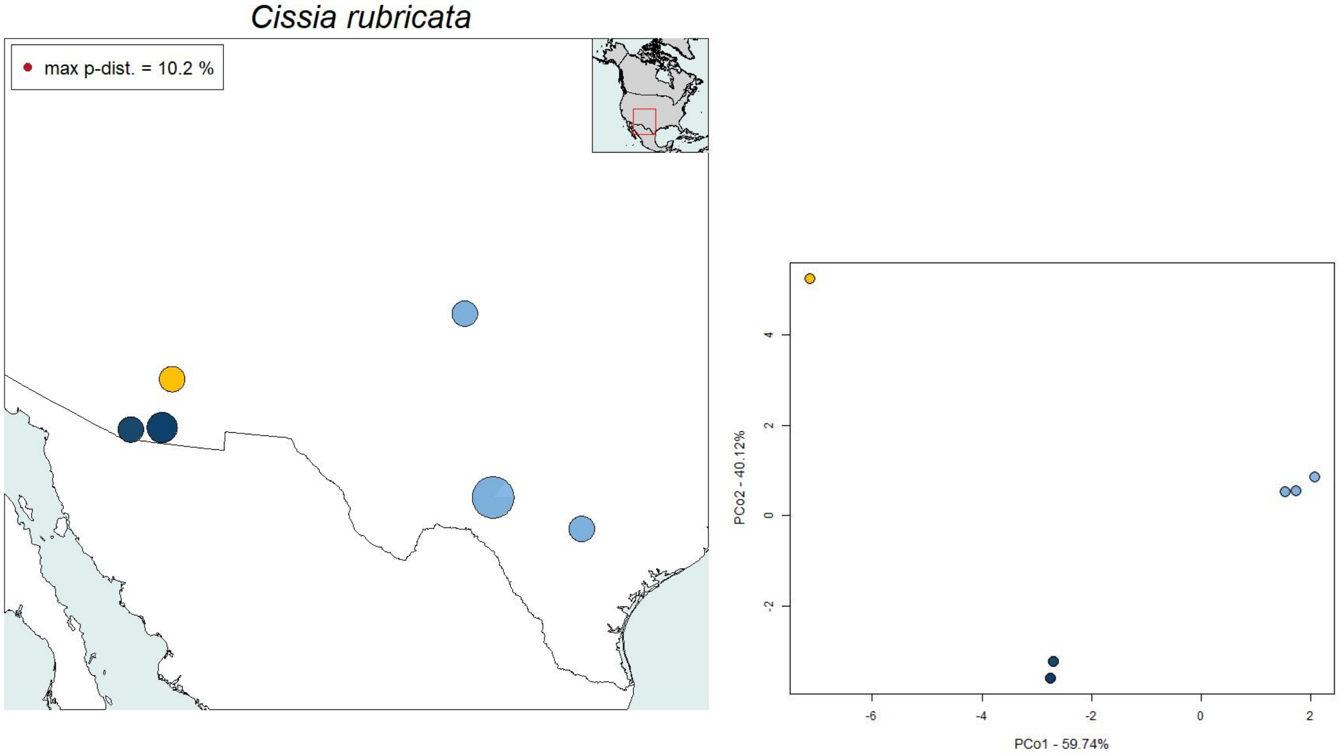

Figure 726 Map of *Cissia rubricata* showing the localities of the sequenced specimens (left). Nearby localities are grouped in pies. Colours match the bidimensional colour space of the PCoA projection (right) of max p-dists among sequences (dots). Sequences= 13; Hap obs.= 8; Hap asympt.= 13.8; Hap % obs.= 58.1%; GST= 0.753; DST= 0.0283; HD= 0.897; ND= 0.0374; max p-dist= 10.2%.

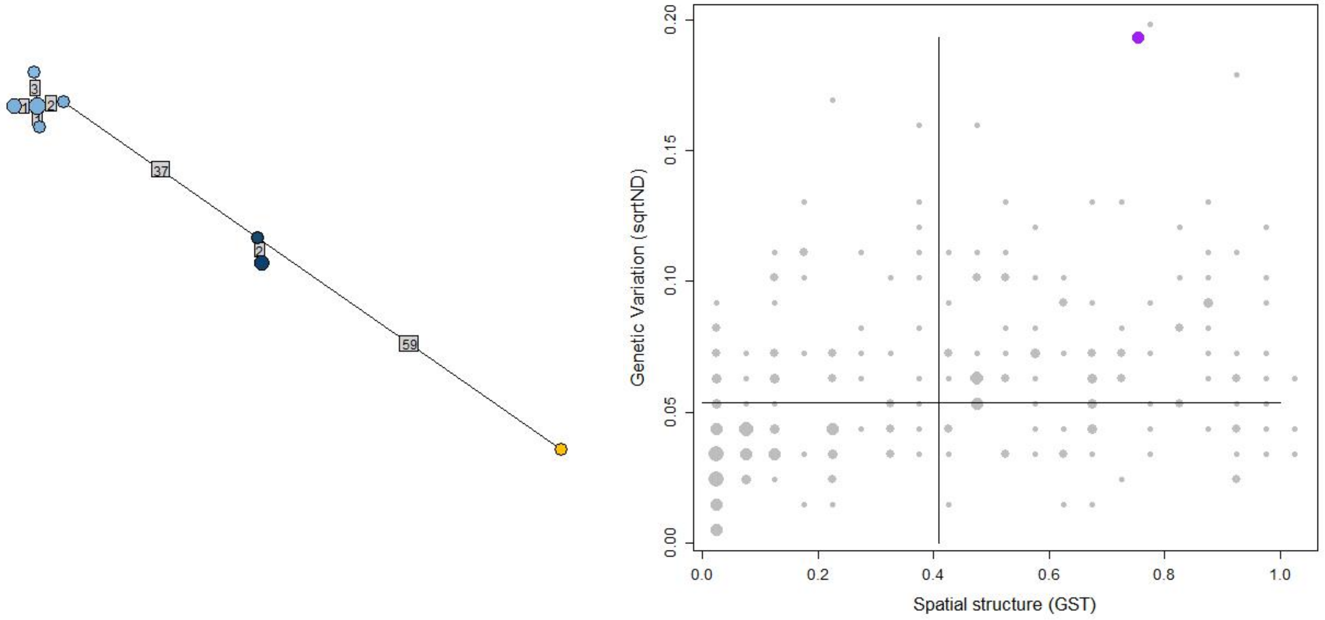

Figure 727: Haplotype network (left) of *Cissia rubricata* sequences > 599 bp with colours matching the PCoA colour space (above). The bubble plot for mt-DNA polymorphism (square root transformed nucleotide diversity) and spatial structure (GST) among all species in the atlas and values for *Cissia rubricata* (purple dot). The horizontal and vertical lines represent median values of nucleotide diversity and GST, respectively. Sequences > 599 bp= 13.

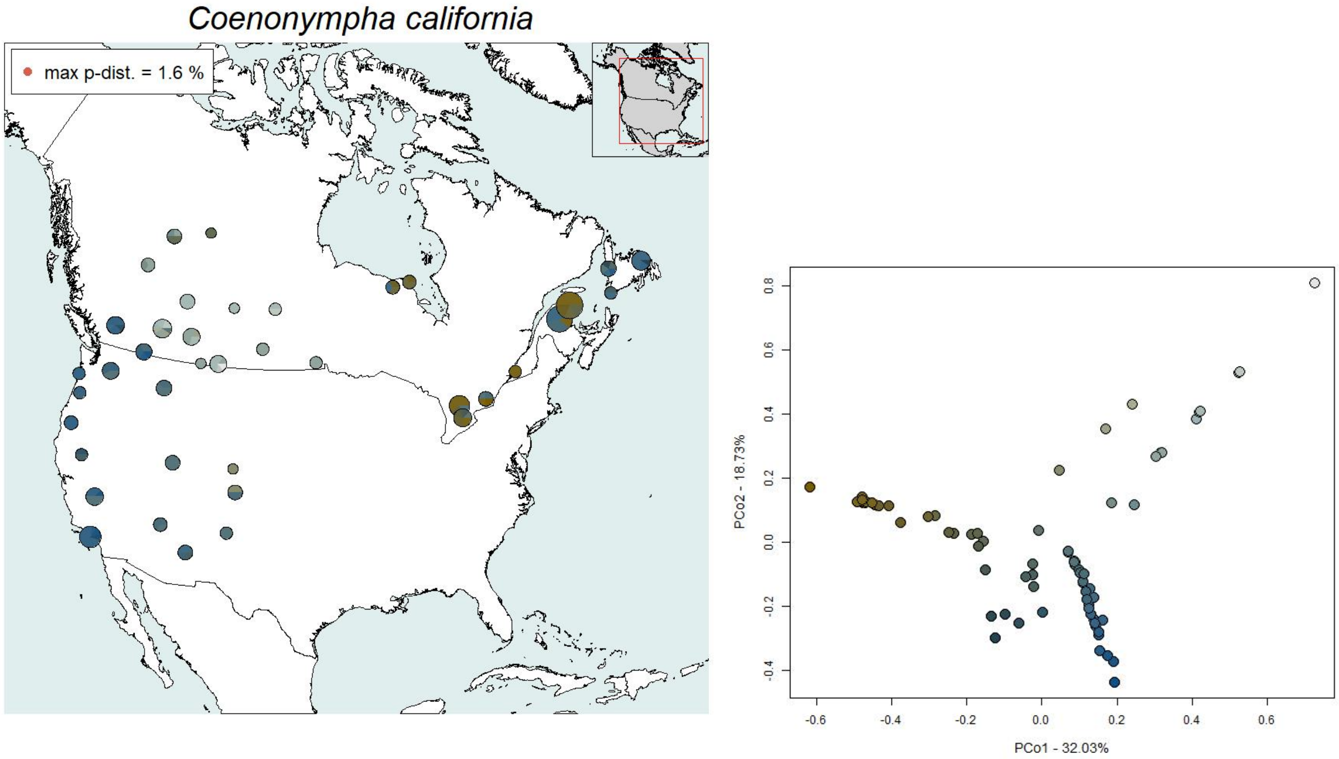

Figure 728 Map of *Coenonympha california* showing the localities of the sequenced specimens (left). Nearby localities are grouped in pies. Colours match the bidimensional colour space of the PCoA projection (right) of max p-dists among sequences (dots). Sequences= 257; Hap obs.= 56; Hap asympt.= 183.5; Hap % obs.= 30.5%; GST= 0.554; DST= 0.0032; HD= 0.941; ND= 0.0058; max p-dist= 1.6%.

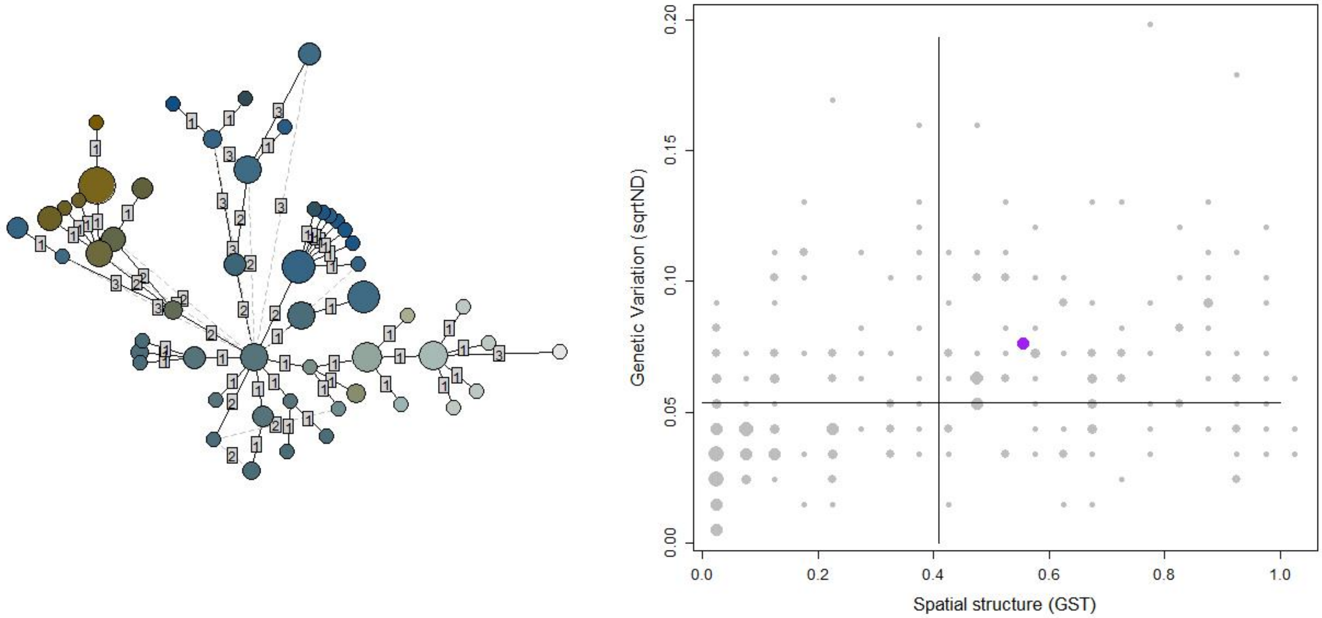

Figure 729: Haplotype network (left) of *Coenonympha california* sequences > 599 bp with colours matching the PCoA colour space (above). The bubble plot for mt-DNA polymorphism (square root transformed nucleotide diversity) and spatial structure (GST) among all species in the atlas and values for *Coenonympha california* (purple dot). The horizontal and vertical lines represent median values of nucleotide diversity and GST, respectively. Sequences > 599 bp= 250.

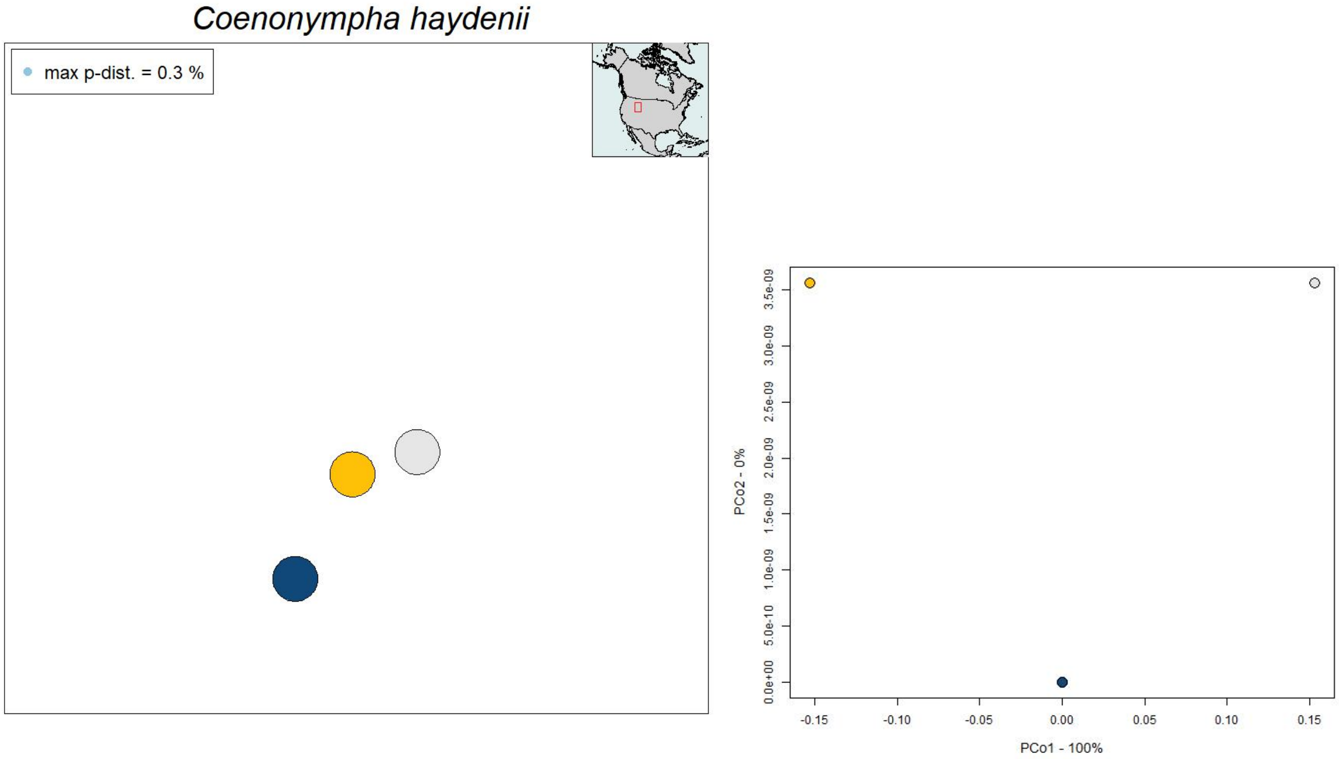

Figure 730 Map of *Coenonympha haydenii* showing the localities of the sequenced specimens (left). Nearby localities are grouped in pies. Colours match the bidimensional colour space of the PCoA projection (right) of max p-dists among sequences (dots). Sequences= 3; Hap obs.= 3; Hap asympt.= NA; Hap % obs.= NA%; GST= NaN; DST= NaN; HD= NA; ND= NA; max p-dist= 0.3%.

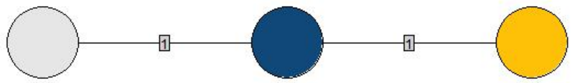

Figure 731: Haplotype network of *Coenonympha haydenii*. Sequences > 599 bp= 3.

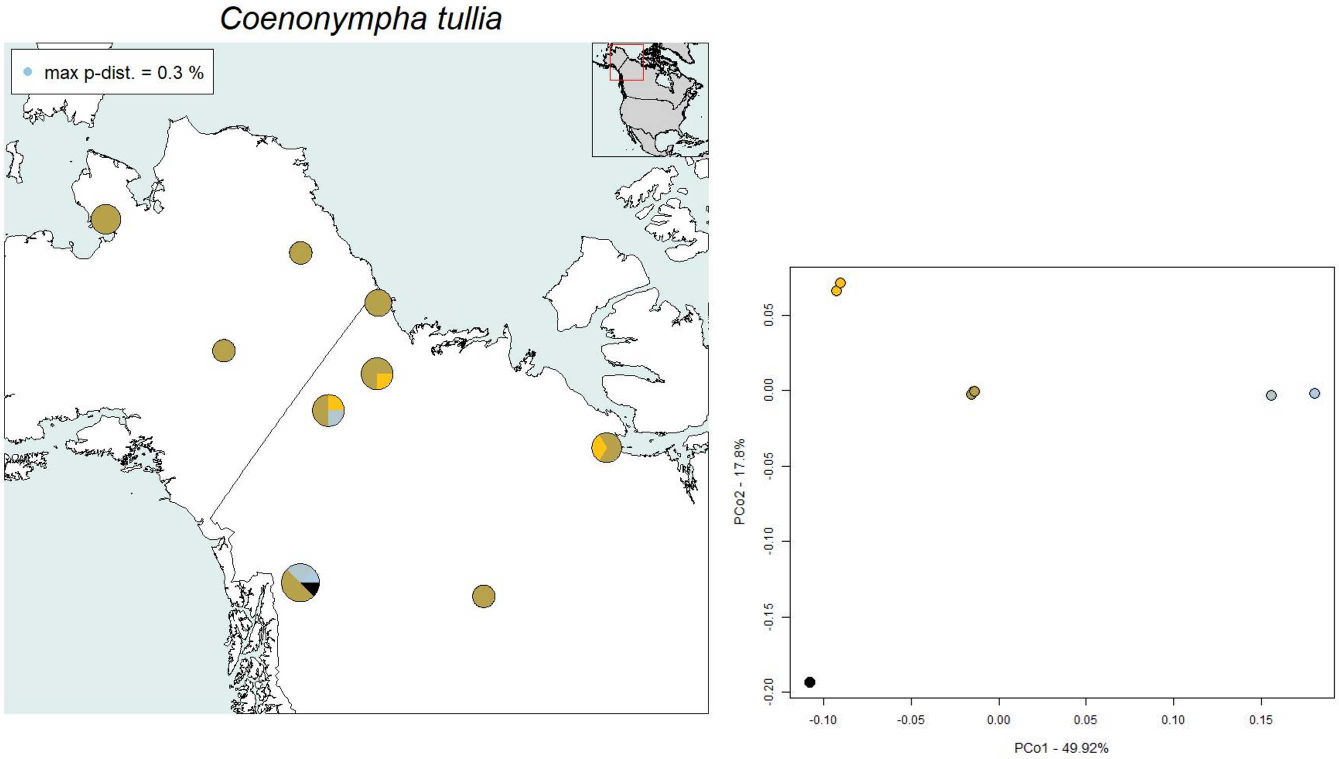

Figure 732 Map of *Coenonympha tullia* showing the localities of the sequenced specimens (left). Nearby localities are grouped in pies. Colours match the bidimensional colour space of the PCoA projection (right) of max p-dists among sequences (dots). Sequences= 27; Hap obs.= 6; Hap asympt.= 11.8; Hap % obs.= 50.9%; GST= 0.051; DST= 0.0001; HD= 0.496; ND= 0.0009; max p-dist= 0.3%.

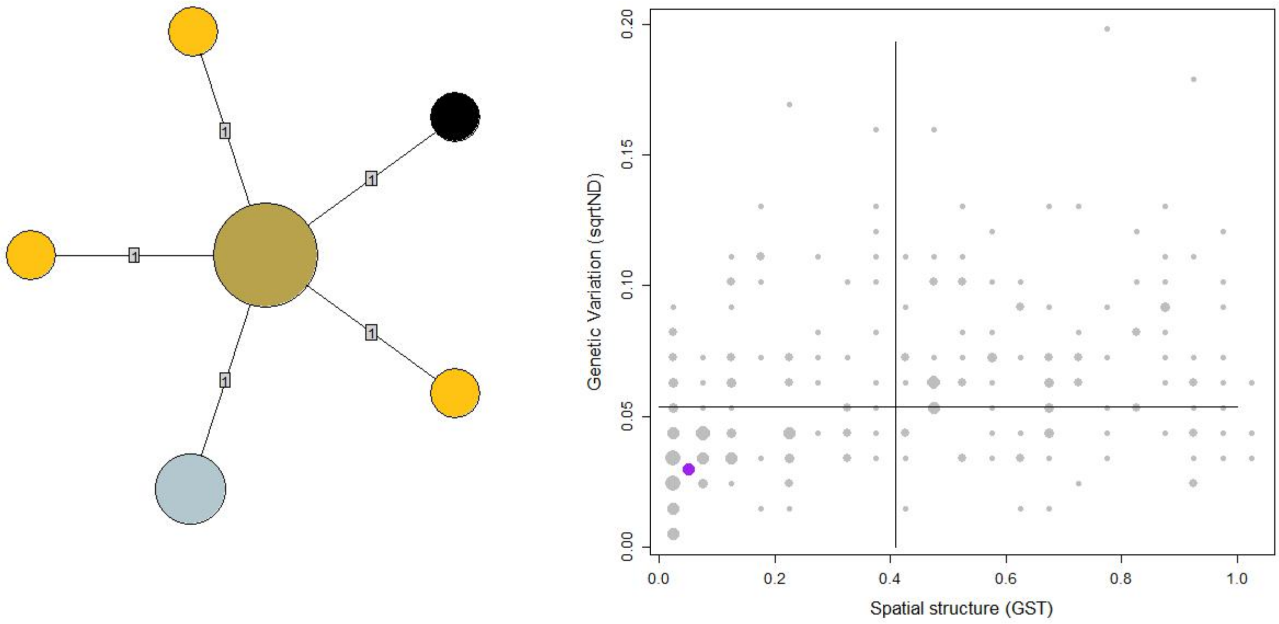

Figure 733: Haplotype network (left) of *Coenonympha tullia* sequences > 599 bp with colours matching the PCoA colour space (above). The bubble plot for mt-DNA polymorphism (square root transformed nucleotide diversity) and spatial structure (GST) among all species in the atlas and values for *Coenonympha tullia* (purple dot). The horizontal and vertical lines represent median values of nucleotide diversity and GST, respectively. Sequences > 599 bp= 27.

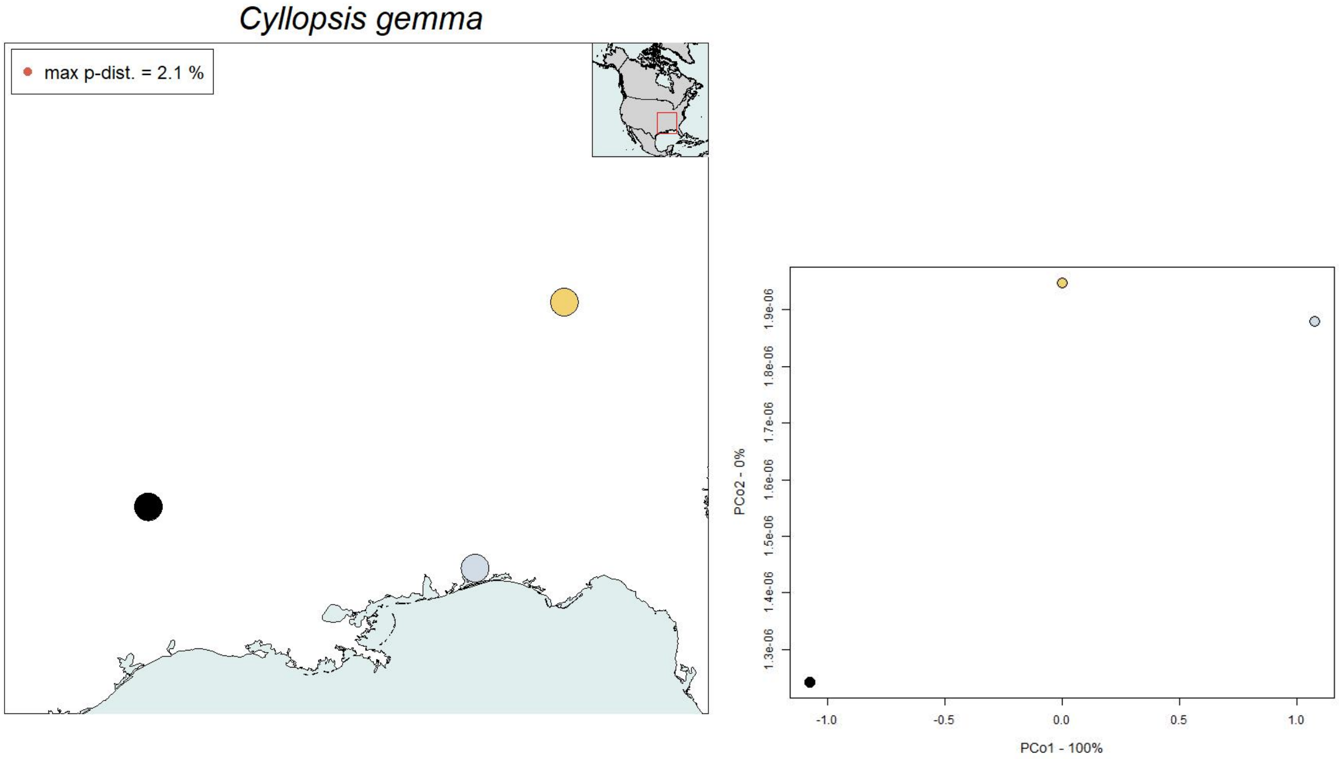

Figure 734 Map of *Cyllopsis gemma* showing the localities of the sequenced specimens (left). Nearby localities are grouped in pies. Colours match the bidimensional colour space of the PCoA projection (right) of max p-dists among sequences (dots). Sequences= 3; Hap obs.= 3; Hap asympt.= NA; Hap % obs.= NA%; GST= NaN; DST= NaN; HD= NA; ND= NA; max p-dist= 2.1%.

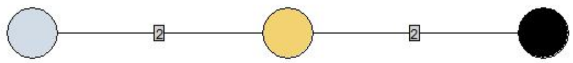

Figure 735: Haplotype network of *Cyllopsis gemma*. Sequences > 599 bp= 3.

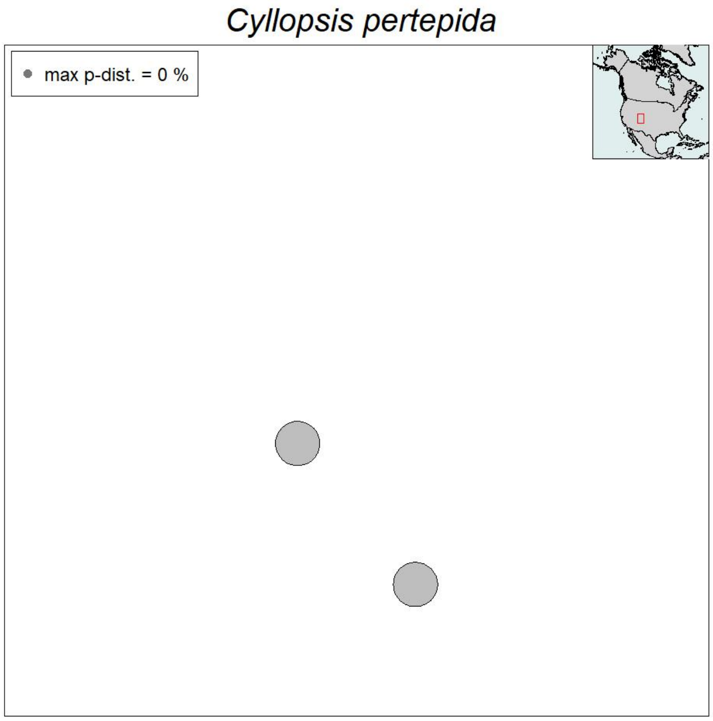

Figure 736: Map of *Cyllopsis pertepida* showing the localities of the sequenced specimens. Nearby localities are grouped in pies. Due to the presence of a single haplotype PCoA projection was not done and a single grey colour was plotted on the map. Sequences= 2; Hap obs.= 1; Hap asympt.= NA; Hap % obs.= NA%; GST= NaN; DST= NaN; HD= NA; ND= NA; max p-dist= 0%.

Haplotype network analysis and bubble plot of *Cyllopsis pertepida* were not possible. Sequences > 599 bp = 2.

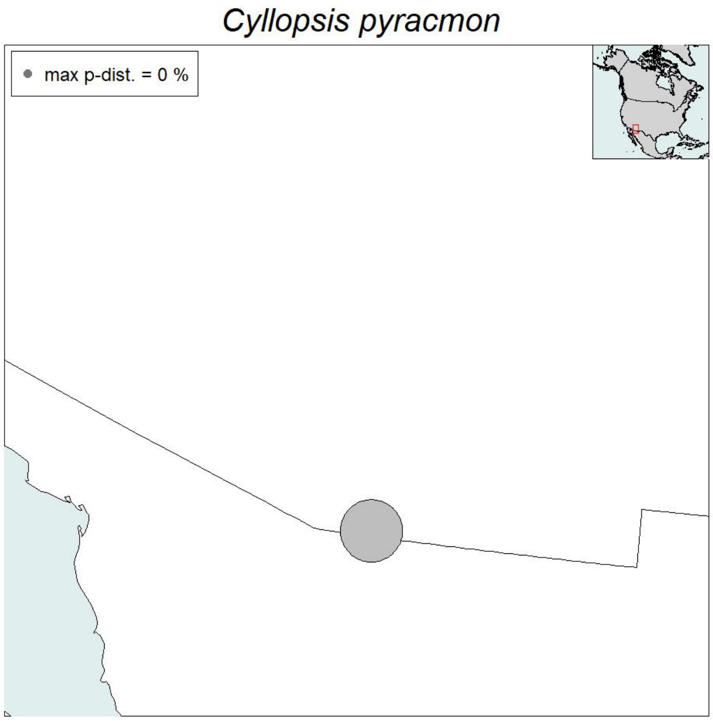

Figure 737: Map of *Cyllopsis pyracmon* showing the localities of the sequenced specimens. Nearby localities are grouped in pies. Due to the presence of a single haplotype PCoA projection was not done and a single grey colour was plotted on the map. Sequences= 3; Hap obs.= 1; Hap asympt.= NA; Hap % obs.= NA%; GST= NaN; DST= NaN; HD= NA; ND= NA; max p-dist= 0%.

Haplotype network analysis and bubble plot of *Cyllopsis pyracmon* were not possible. Sequences > 599 bp = 3.

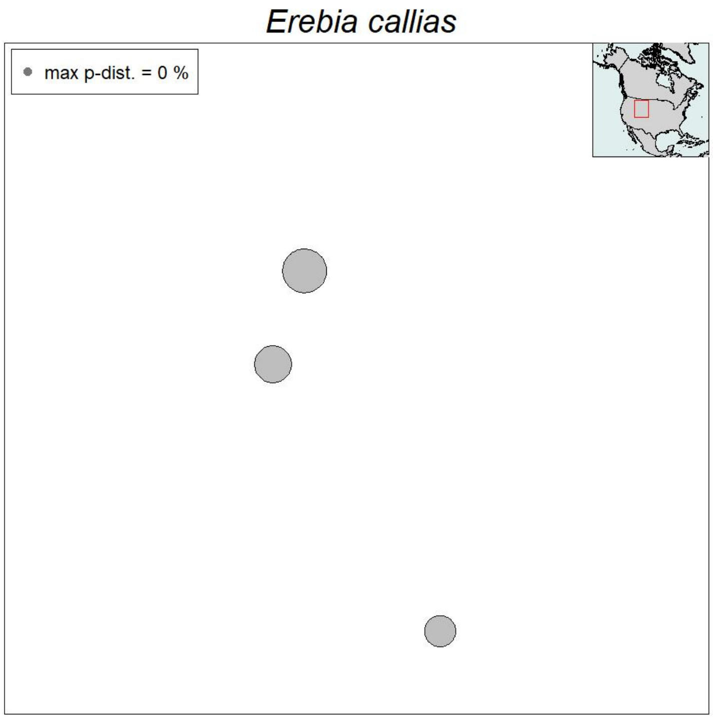

Figure 738: Map of *Erebia callias* showing the localities of the sequenced specimens. Nearby localities are grouped in pies. Due to the presence of a single haplotype PCoA projection was not done and a single grey colour was plotted on the map. Sequences= 7; Hap obs.= 1; Hap asympt.= NA; Hap % obs.= NA%; GST= NaN; DST= NaN; HD= NA; ND= NA; max p-dist= 0%.

Haplotype network analysis and bubble plot of *Erebia callias* were not possible. Sequences > 599 bp = 7.

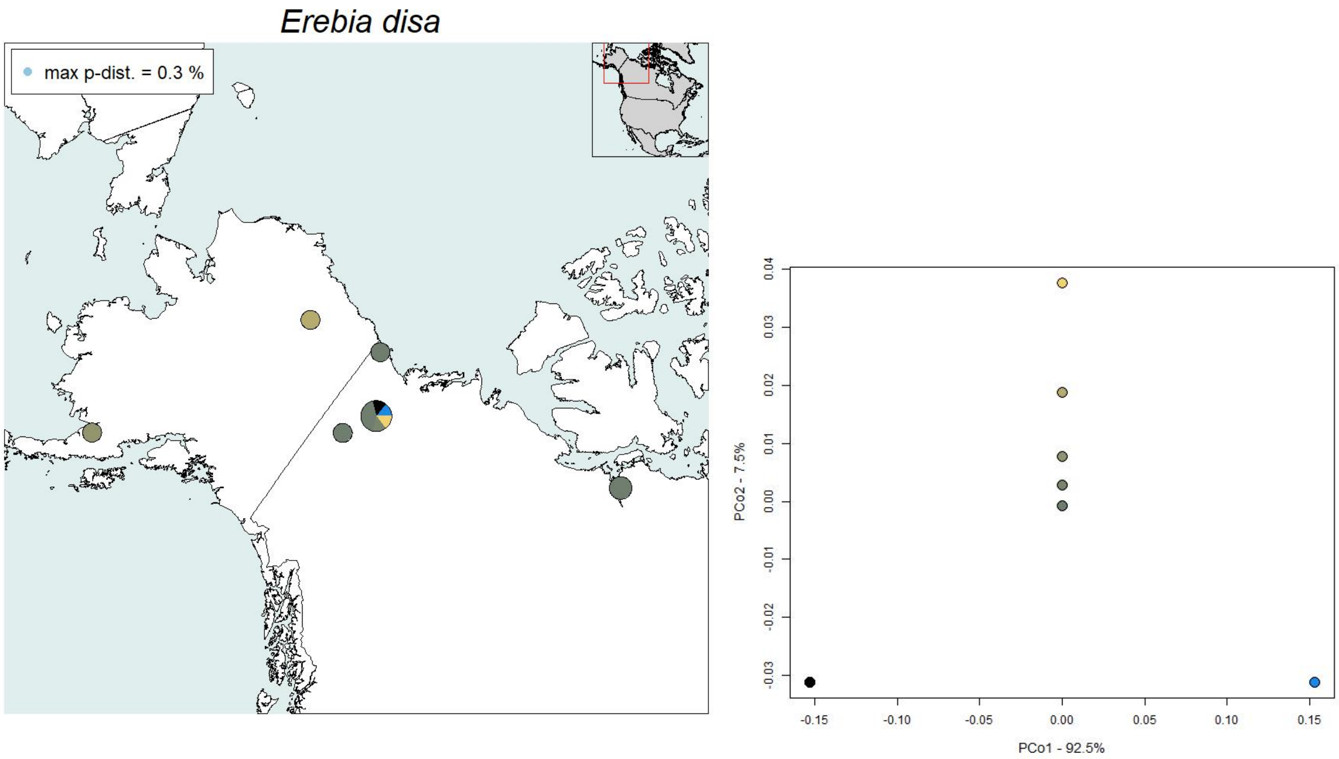

Figure 739 Map of *Erebia disa* showing the localities of the sequenced specimens (left). Nearby localities are grouped in pies. Colours match the bidimensional colour space of the PCoA projection (right) of max p-dists among sequences (dots). Sequences= 13; Hap obs.= 3; Hap asympt.= 3.9; Hap % obs.= 76.5%; GST= NaN; DST= NaN; HD= 0.295; ND= 0.0005; max p-dist= 0.3%.

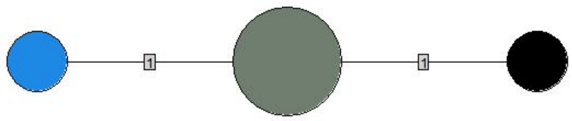

Figure 740: Haplotype network of *Erebia disa*. Sequences > 599 bp= 12.

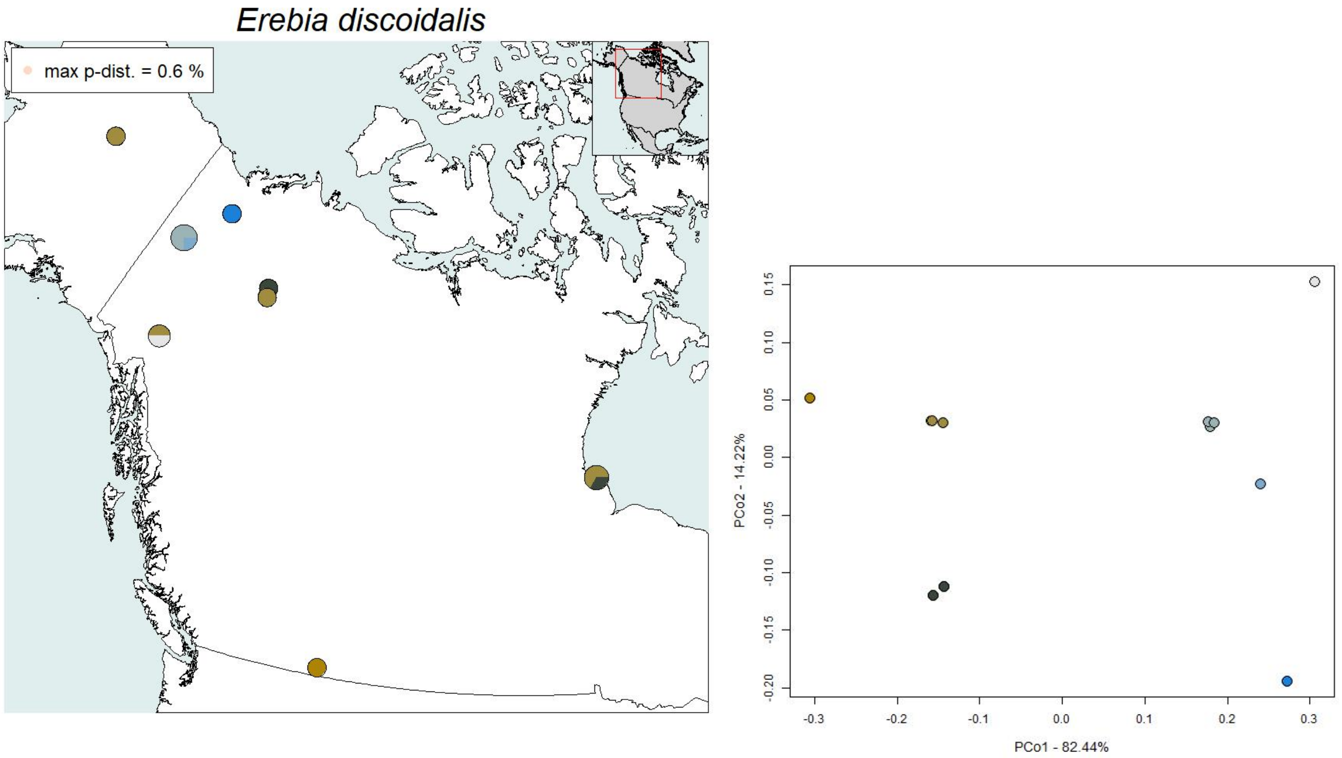

Figure 741 Map of *Erebia discoidalis* showing the localities of the sequenced specimens (left). Nearby localities are grouped in pies. Colours match the bidimensional colour space of the PCoA projection (right) of max p-dists among sequences (dots). Sequences= 14; Hap obs.= 6; Hap asympt.= 11.6; Hap % obs.= 51.9%; GST= NaN; DST= NaN; HD= 0.736; ND= 0.0024; max p-dist= 0.6%.

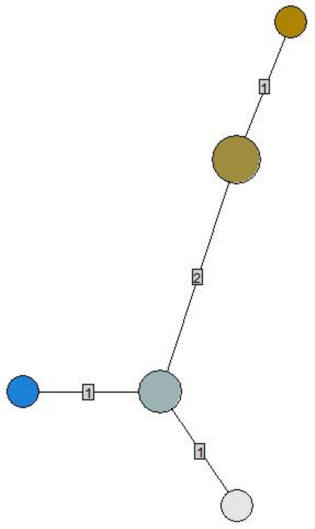

Figure 742: Haplotype network of *Erebia discoidalis*. Sequences > 599 bp= 11.

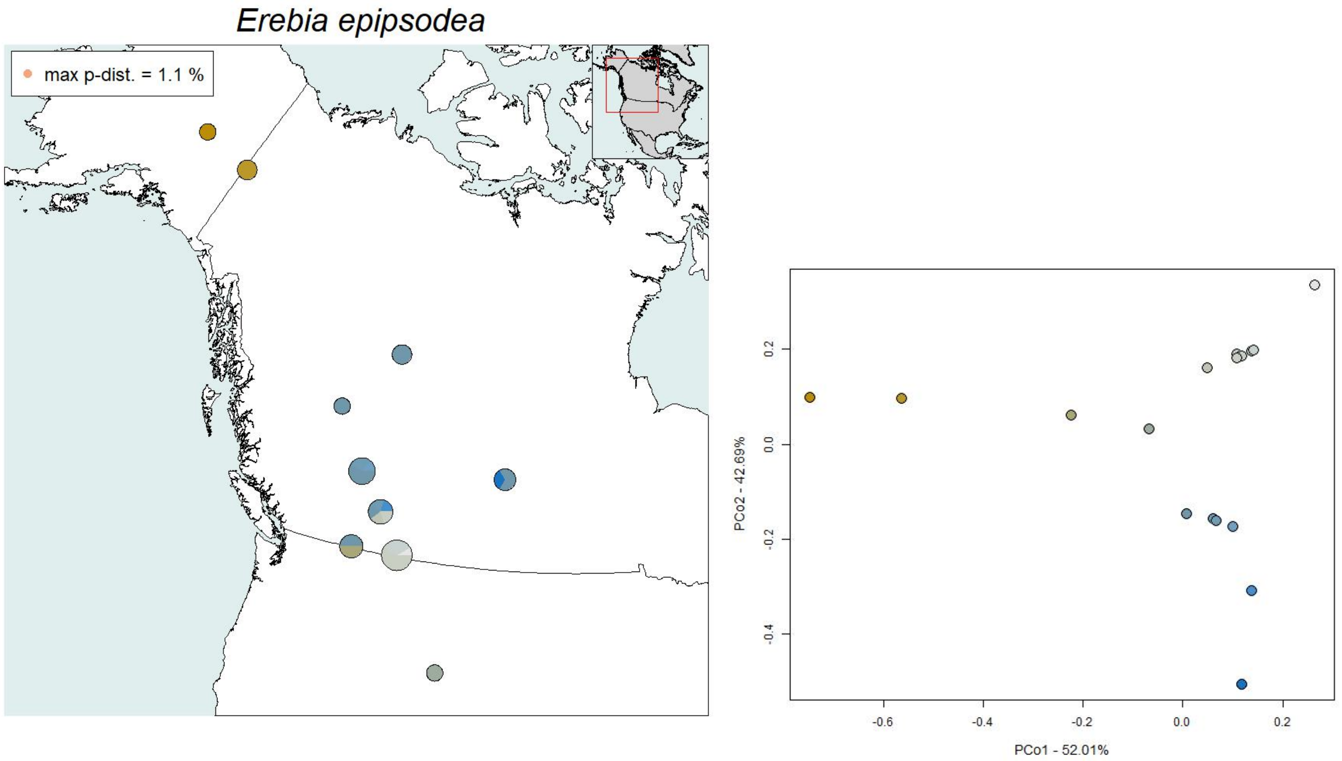

Figure 743 Map of *Erebia epipsodea* showing the localities of the sequenced specimens (left). Nearby localities are grouped in pies. Colours match the bidimensional colour space of the PCoA projection (right) of max p-dists among sequences (dots). Sequences= 38; Hap obs.= 10; Hap asympt.= 18.8; Hap % obs.= 53.3%; GST= 0.699; DST= 0.0017; HD= 0.733; ND= 0.003; max p-dist= 1.1%.

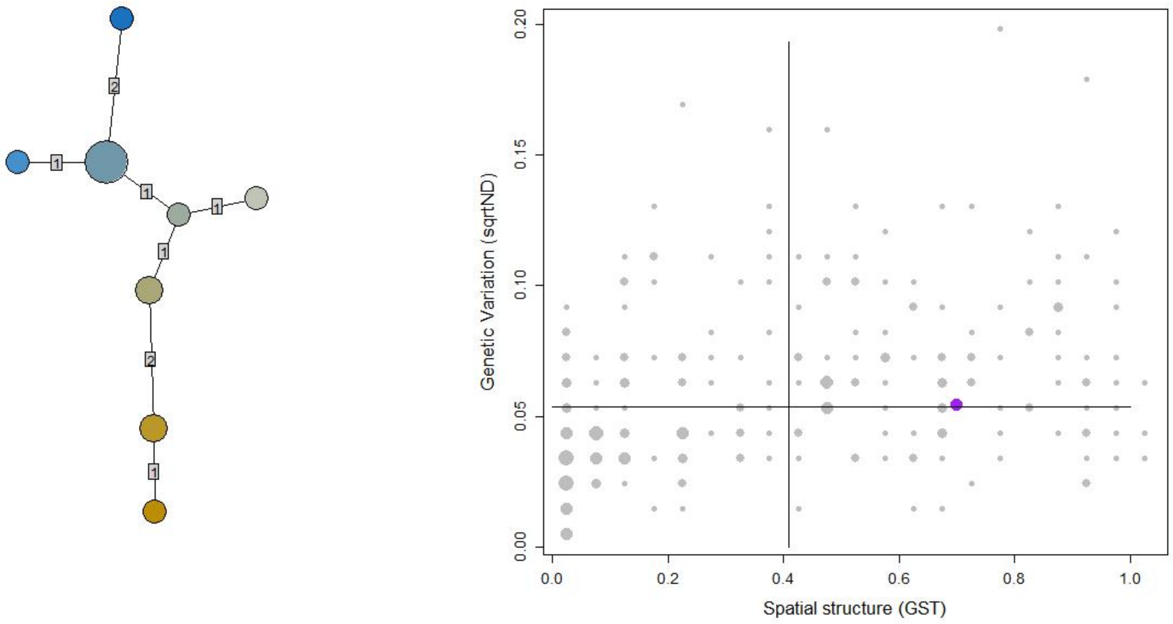

Figure 744: Haplotype network (left) of *Erebia epipsodea* sequences > 599 bp with colours matching the PCoA colour space (above). The bubble plot for mt-DNA polymorphism (square root transformed nucleotide diversity) and spatial structure (GST) among all species in the atlas and values for *Erebia epipsodea* (purple dot). The horizontal and vertical lines represent median values of nucleotide diversity and GST, respectively. Sequences > 599 bp= 21.

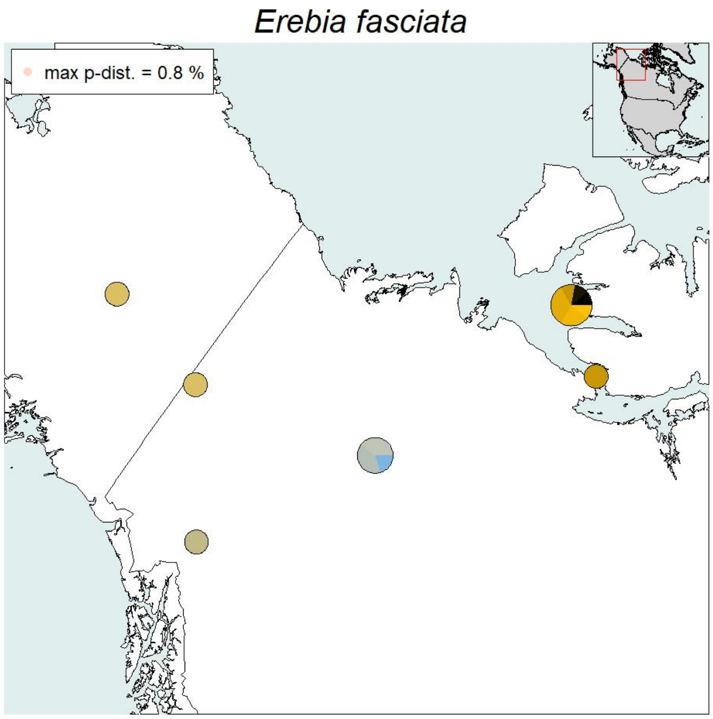

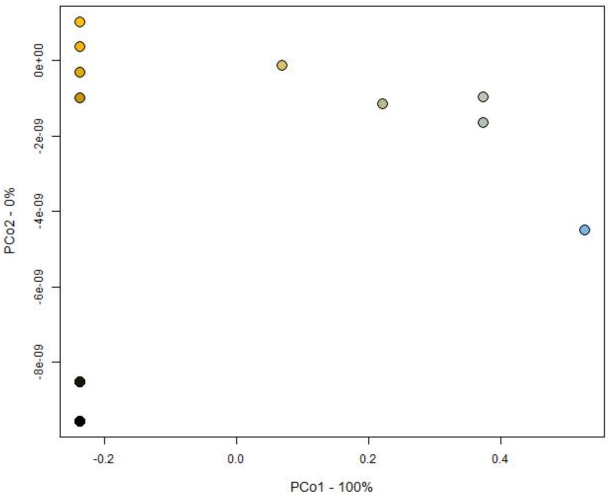

Figure 745 Map of *Erebia fasciata* showing the localities of the sequenced specimens (left). Nearby localities are grouped in pies. Colours match the bidimensional colour space of the PCoA projection (right) of max p-dists among sequences (dots). Sequences= 18; Hap obs.= 5; Hap asympt.= 6.9; Hap % obs.= 72.6%; GST= 0.975; DST= 0.003; HD= 0.66; ND= 0.0031; max p-dist= 0.8%.

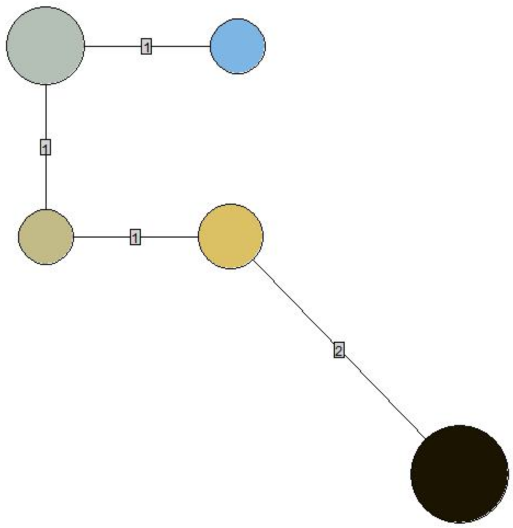

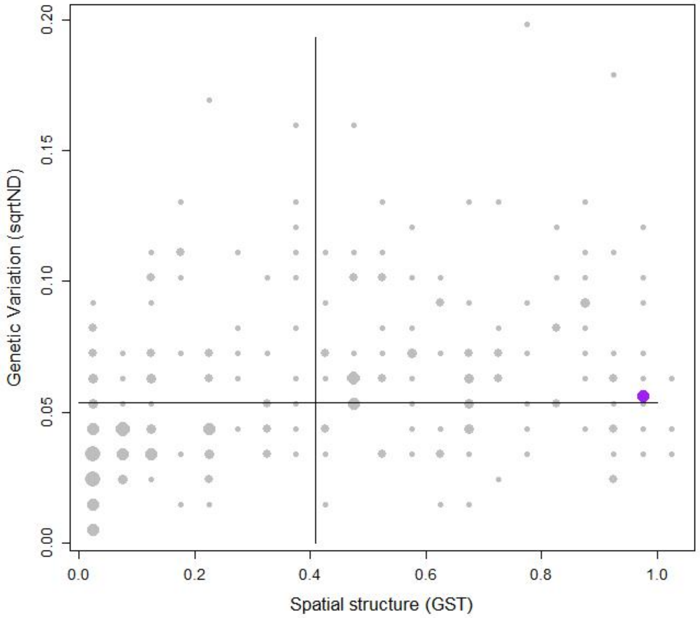

Figure 746: Haplotype network (left) of *Erebia fasciata* sequences > 599 bp with colours matching the PCoA colour space (above). The bubble plot for mt-DNA polymorphism (square root transformed nucleotide diversity) and spatial structure (GST) among all species in the atlas and values for *Erebia fasciata* (purple dot). The horizontal and vertical lines represent median values of nucleotide diversity and GST, respectively. Sequences > 599 bp= 18.

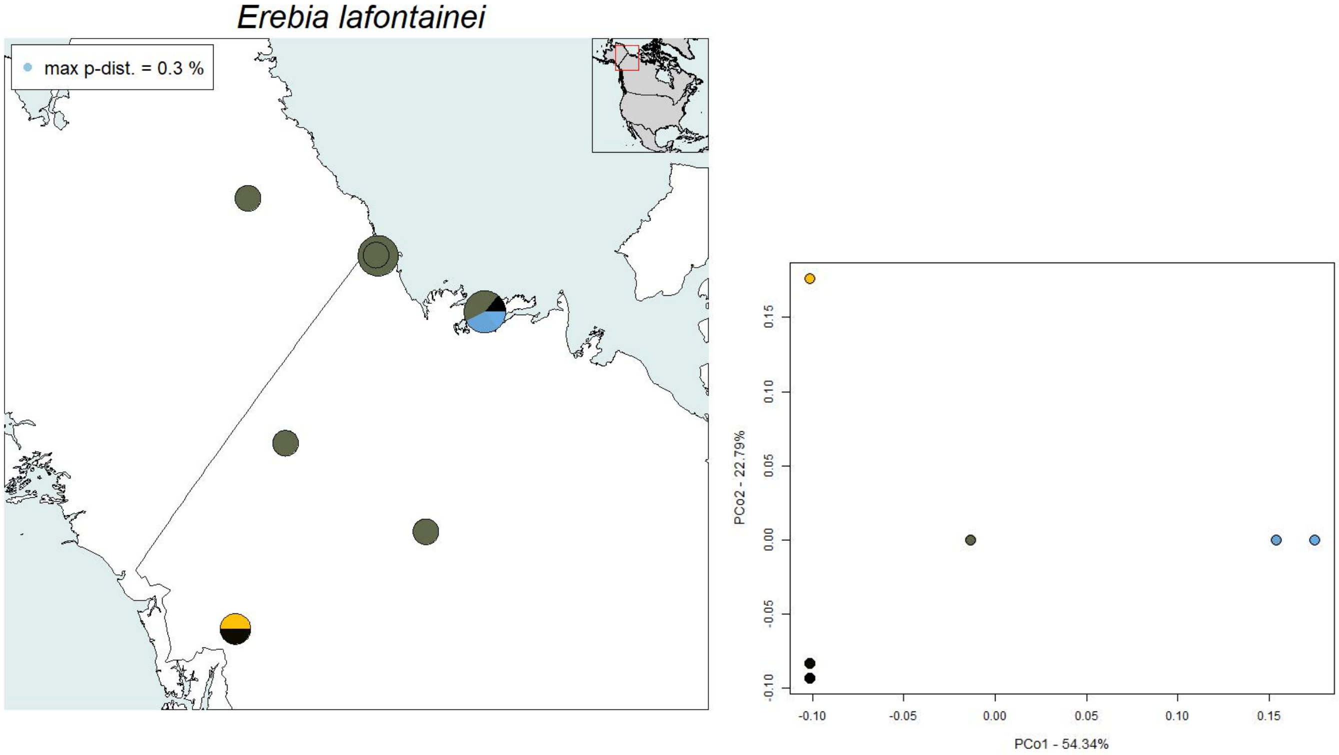

Figure 747 Map of *Erebia lafontainei* showing the localities of the sequenced specimens (left). Nearby localities are grouped in pies. Colours match the bidimensional colour space of the PCoA projection (right) of max p-dists among sequences (dots). Sequences= 19; Hap obs.= 5; Hap asympt.= 7.8; Hap % obs.= 63.8%; GST= 0.086; DST= 0.0001; HD= 0.526; ND= 0.0009; max p-dist= 0.3%.

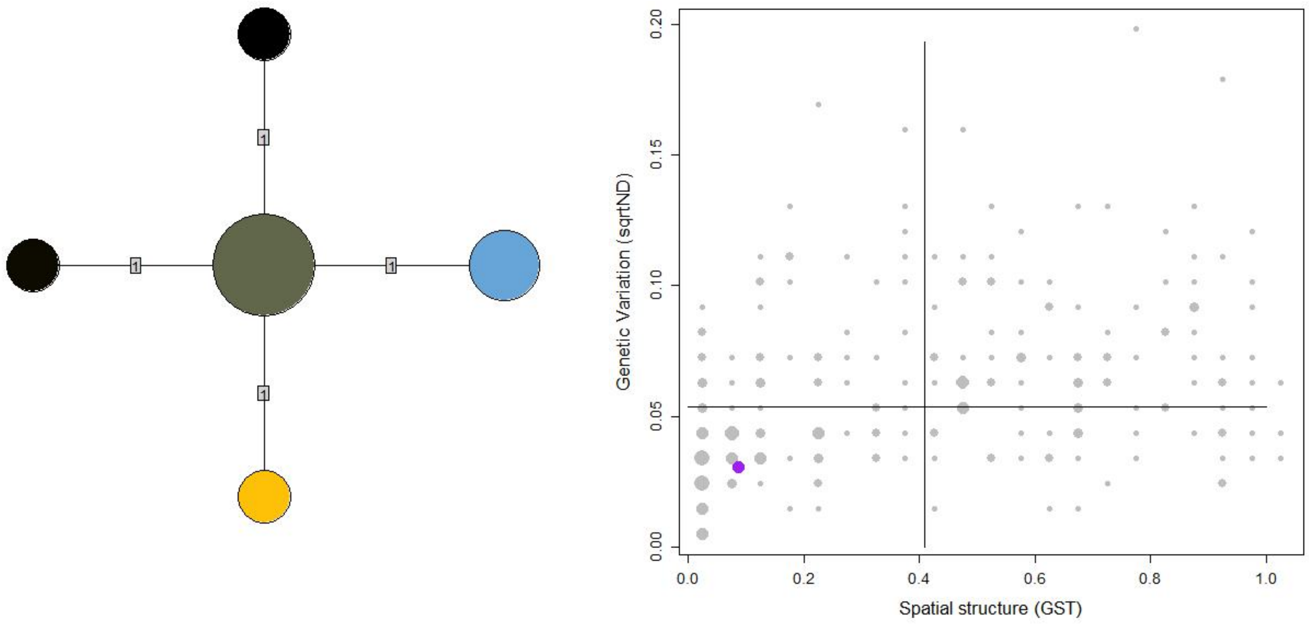

Figure 748: Haplotype network (left) of *Erebia lafontainei* sequences > 599 bp with colours matching the PCoA colour space (above). The bubble plot for mt-DNA polymorphism (square root transformed nucleotide diversity) and spatial structure (GST) among all species in the atlas and values for *Erebia lafontainei* (purple dot). The horizontal and vertical lines represent median values of nucleotide diversity and GST, respectively. Sequences > 599 bp= 19.

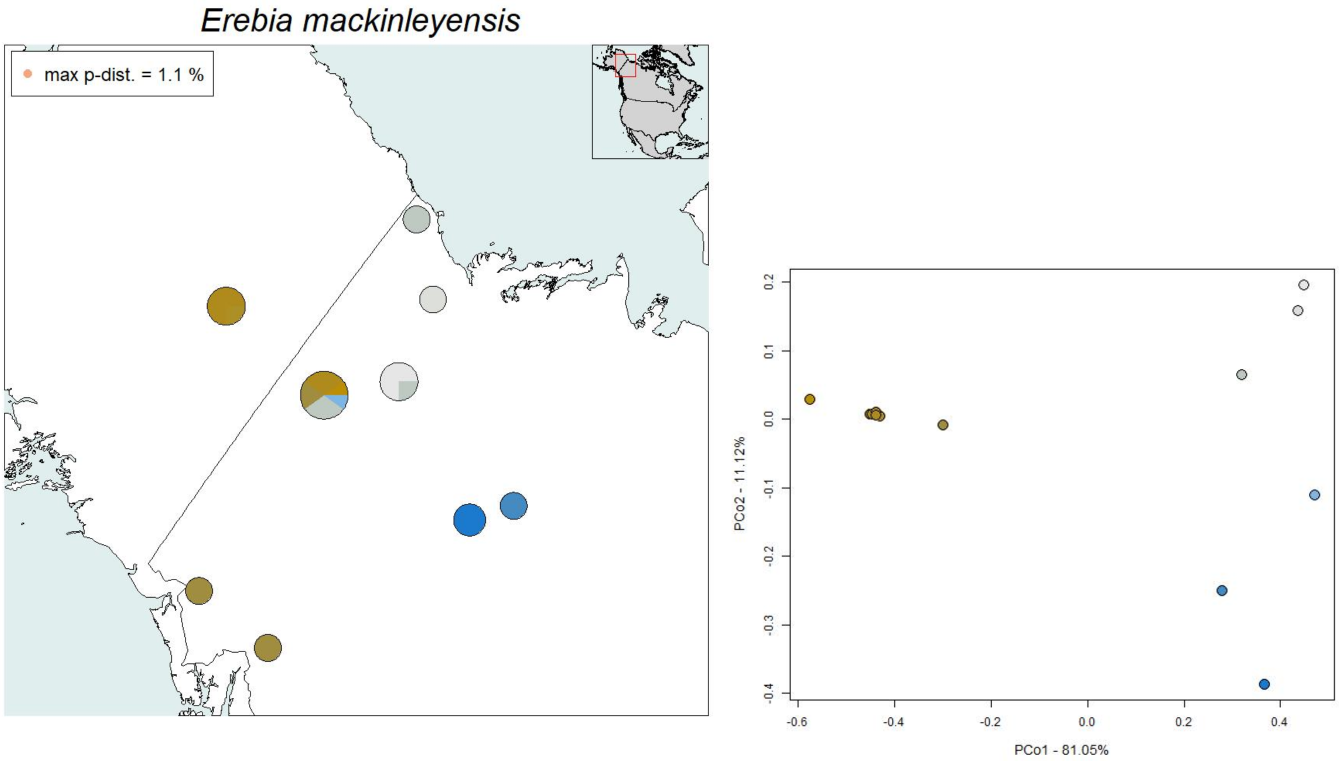

Figure 749 Map of *Erebia mackinleyensis* showing the localities of the sequenced specimens (left). Nearby localities are grouped in pies. Colours match the bidimensional colour space of the PCoA projection (right) of max p-dists among sequences (dots). Sequences= 25; Hap obs.= 11; Hap asympt.= 25.4; Hap % obs.= 43.3%; GST= 0.301; DST= 0.0016; HD= 0.907; ND= 0.0053; max p-dist= 1.1%.

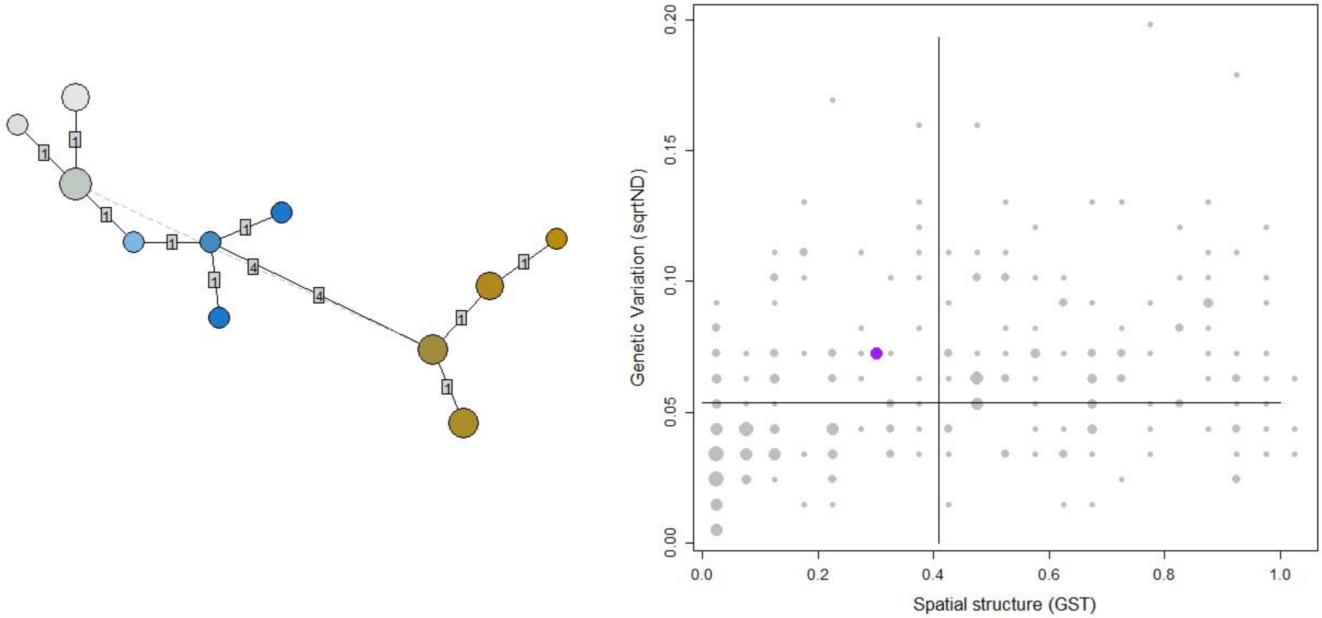

Figure 750: Haplotype network (left) of *Erebia mackinleyensis* sequences > 599 bp with colours matching the PCoA colour space (above). The bubble plot for mt-DNA polymorphism (square root transformed nucleotide diversity) and spatial structure (GST) among all species in the atlas and values for *Erebia mackinleyensis* (purple dot). The horizontal and vertical lines represent median values of nucleotide diversity and GST, respectively. Sequences > 599 bp= 25.

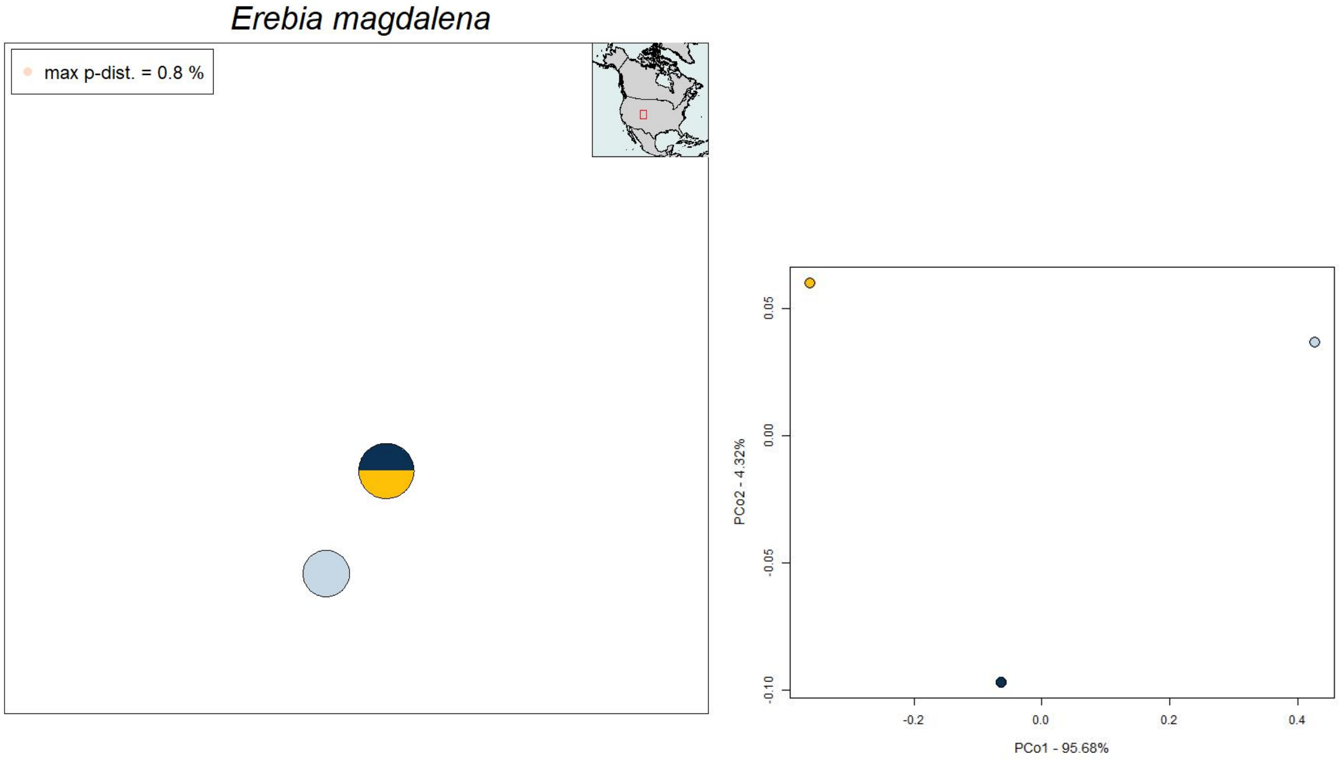

Figure 751 Map of *Erebia magdalena* showing the localities of the sequenced specimens (left). Nearby localities are grouped in pies. Colours match the bidimensional colour space of the PCoA projection (right) of max p-dists among sequences (dots). Sequences= 3; Hap obs.= 3; Hap asympt.= NA; Hap % obs.= NA%; GST= NaN; DST= NaN; HD= NA; ND= NA; max p-dist= 0.8%.

Haplotype network analysis and bubble plot of *Erebia magdalena* were not possible. Sequences > 599 bp = 2.

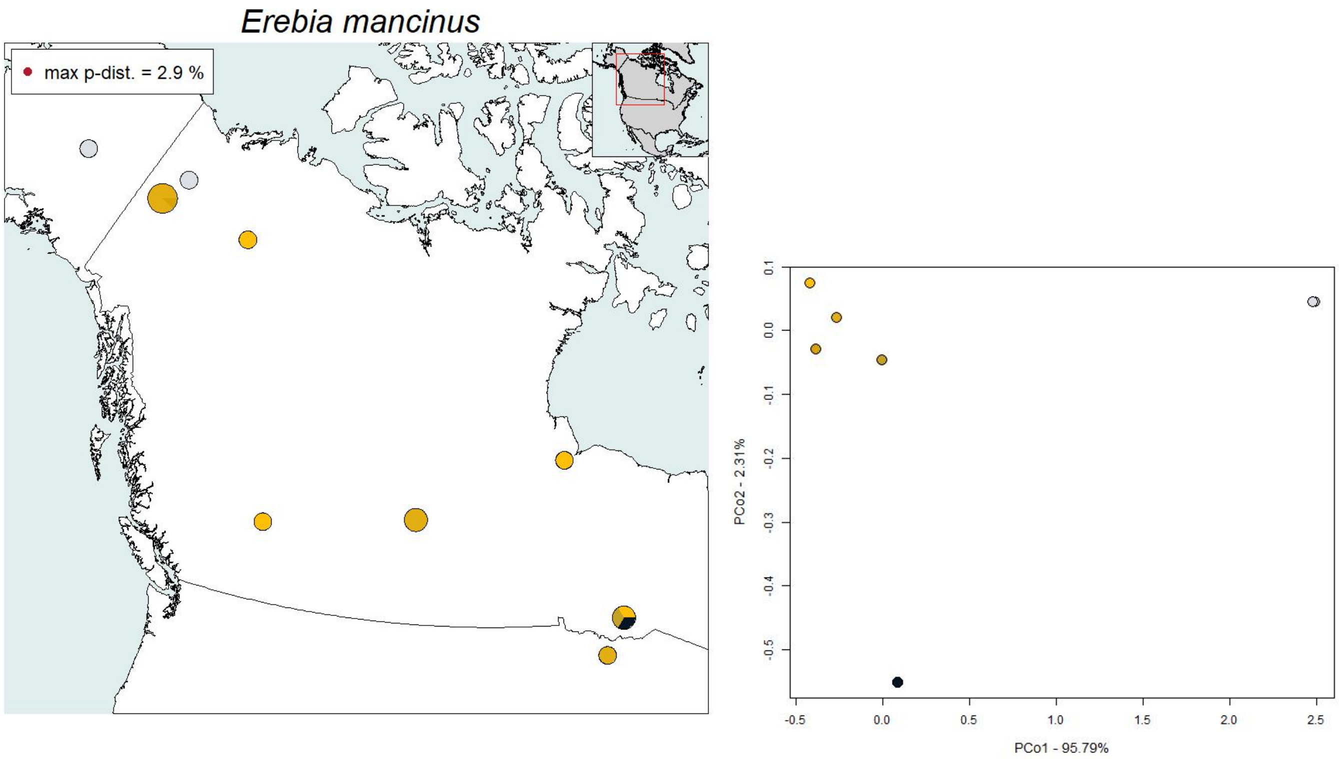

Figure 752 Map of *Erebia mancinus* showing the localities of the sequenced specimens (left). Nearby localities are grouped in pies. Colours match the bidimensional colour space of the PCoA projection (right) of max p-dists among sequences (dots). Sequences= 20; Hap obs.= 7; Hap asympt.= 18.9; Hap % obs.= 37.1%; GST= 0.392; DST= 0.0005; HD= 0.584; ND= 0.0064; max p-dist= 2.9%.

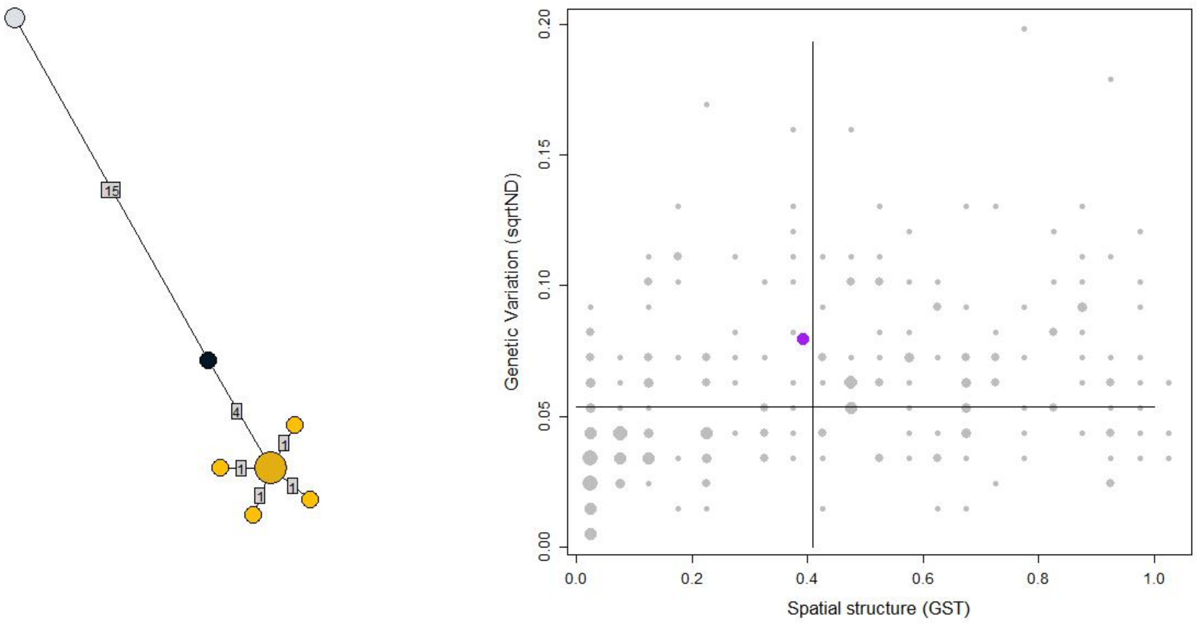

Figure 753: Haplotype network (left) of *Erebia mancinus* sequences > 599 bp with colours matching the PCoA colour space (above). The bubble plot for mt-DNA polymorphism (square root transformed nucleotide diversity) and spatial structure (GST) among all species in the atlas and values for *Erebia mancinus* (purple dot). The horizontal and vertical lines represent median values of nucleotide diversity and GST, respectively. Sequences > 599 bp= 20.

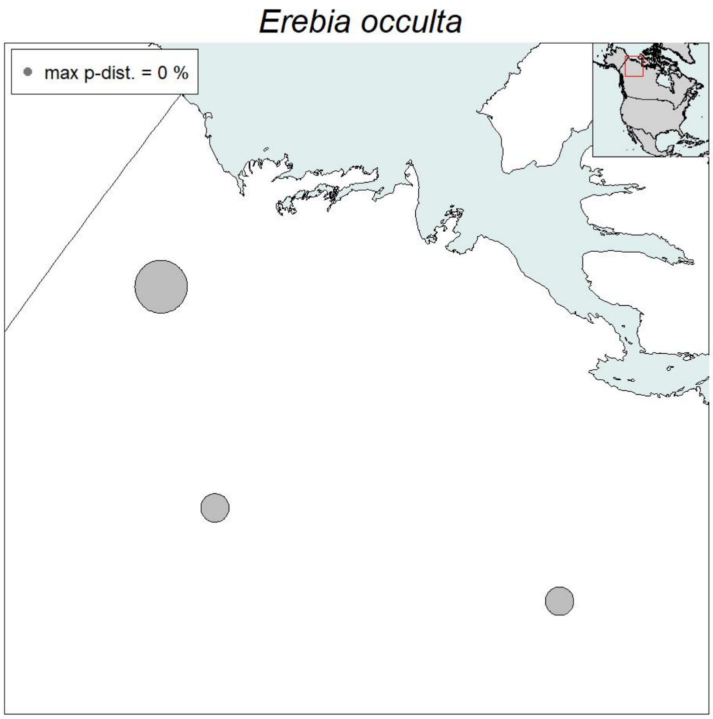

Figure 754: Map of *Erebia occulta* showing the localities of the sequenced specimens. Nearby localities are grouped in pies. Due to the presence of a single haplotype PCoA projection was not done and a single grey colour was plotted on the map. Sequences= 14; Hap obs.= 1; Hap asympt.= NA; Hap % obs.= NA%; GST= 0; DST= 0; HD= 0; ND= 0; max p-dist= 0%.

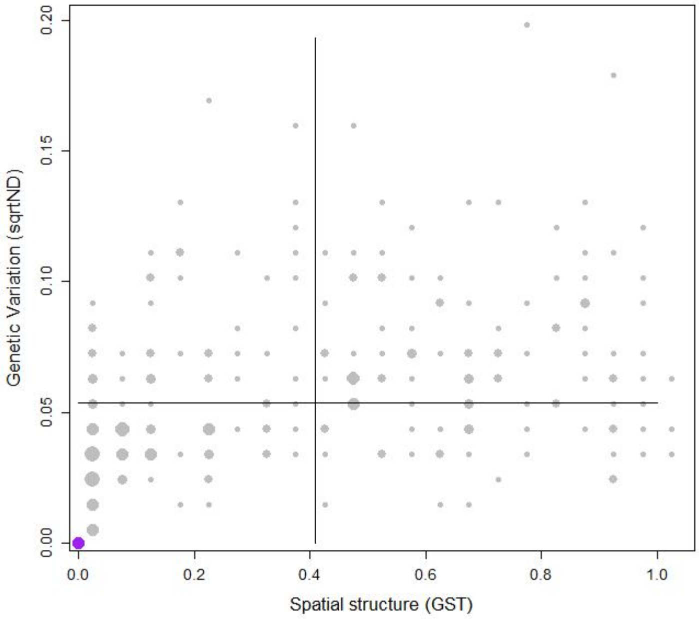

Figure 755: The bubble plot for mt-DNA polymorphism (square root transformed nucleotide diversity) and spatial structuree (GST) among all species in the atlas and values for *Erebia occulta* (purple dot). The horizontal and vertical lines represent median values of nucleotide diversity and GST, respectively. Haplotype network analysis was not possible. Sequences > 599 bp = 14.

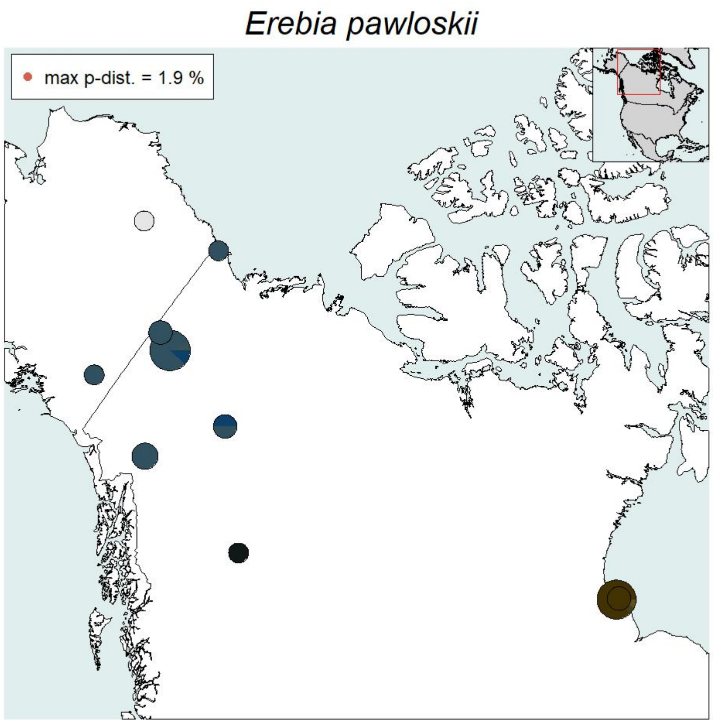

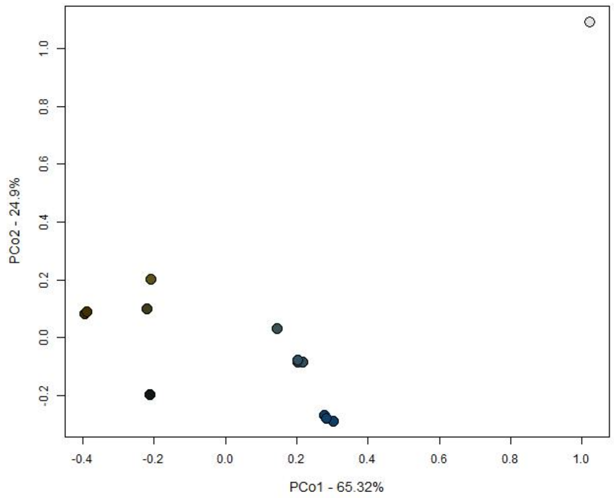

Figure 756 Map of *Erebia pawloskii* showing the localities of the sequenced specimens (left). Nearby localities are grouped in pies. Colours match the bidimensional colour space of the PCoA projection (right) of max p-dists among sequences (dots). Sequences= 45; Hap obs.= 9; Hap asympt.= 29.5; Hap % obs.= 30.5%; GST= 0.91; DST= 0.0029; HD= 0.645; ND= 0.0039; max p-dist= 1.9%.

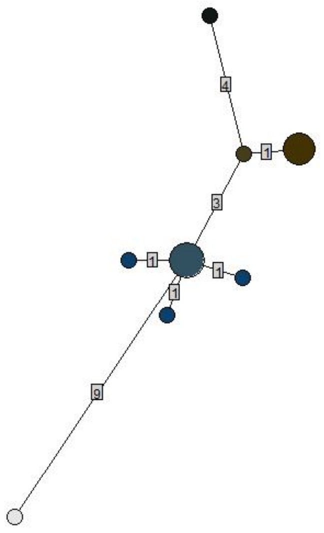

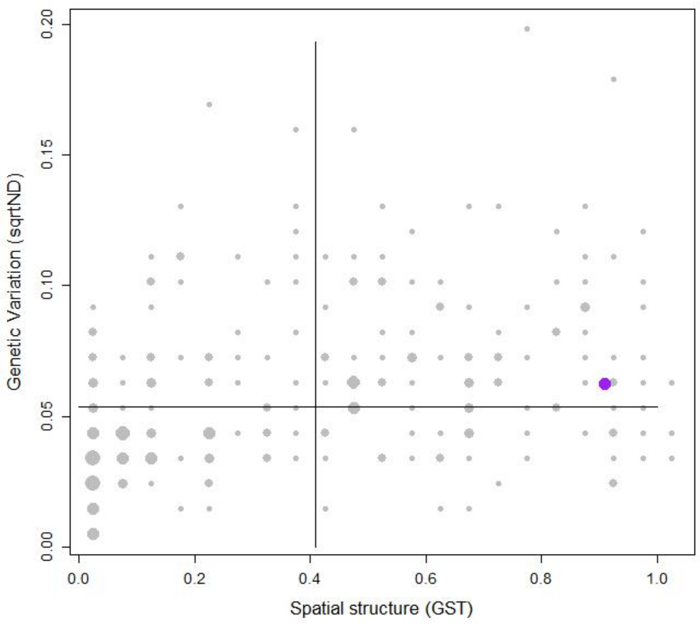

Figure 757: Haplotype network (left) of *Erebia pawloskii* sequences > 599 bp with colours matching the PCoA colour space (above). The bubble plot for mt-DNA polymorphism (square root transformed nucleotide diversity) and spatial structure (GST) among all species in the atlas and values for *Erebia pawloskii* (purple dot). The horizontal and vertical lines represent median values of nucleotide diversity and GST, respectively. Sequences > 599 bp= 45.

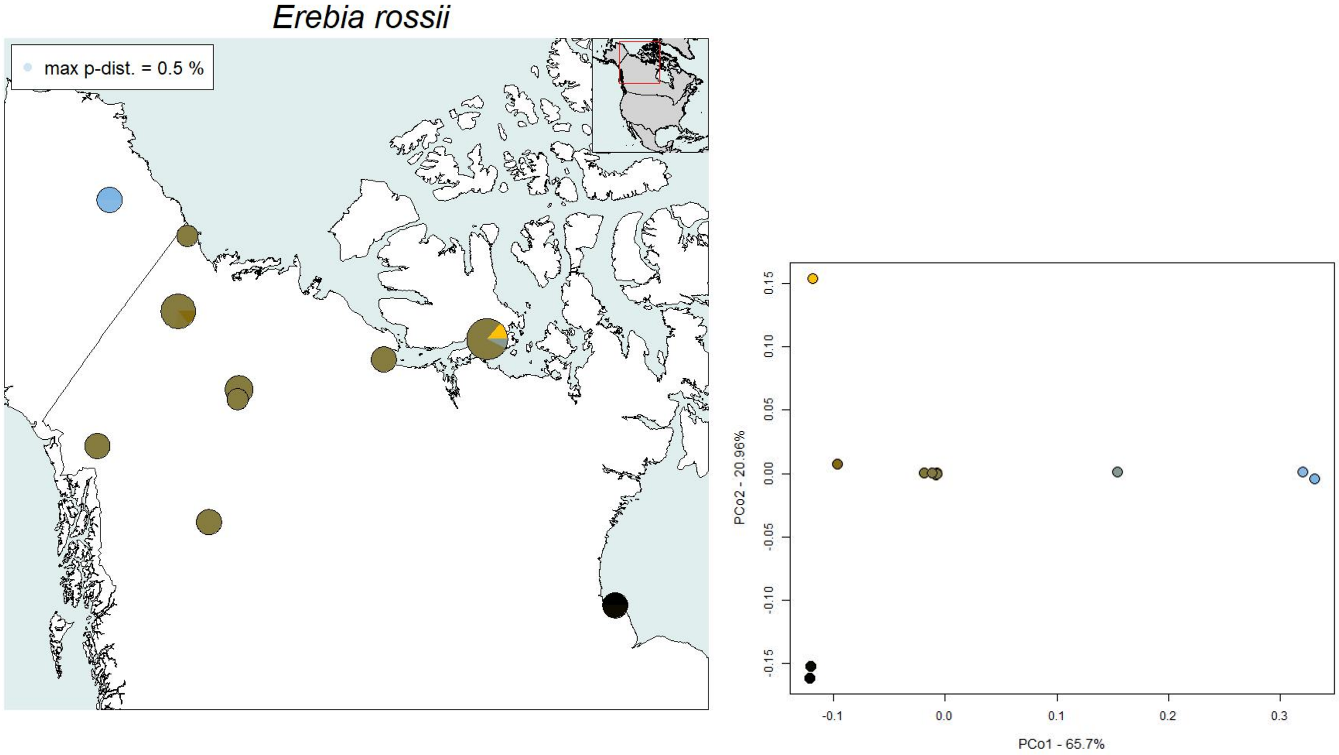

Figure 758 Map of *Erebia rossii* showing the localities of the sequenced specimens (left). Nearby localities are grouped in pies. Colours match the bidimensional colour space of the PCoA projection (right) of max p-dists among sequences (dots). Sequences= 36; Hap obs.= 6; Hap asympt.= 6.6; Hap % obs.= 90.3%; GST= 0; DST= 0; HD= 0.395; ND= 0.0008; max p-dist= 0.5%.

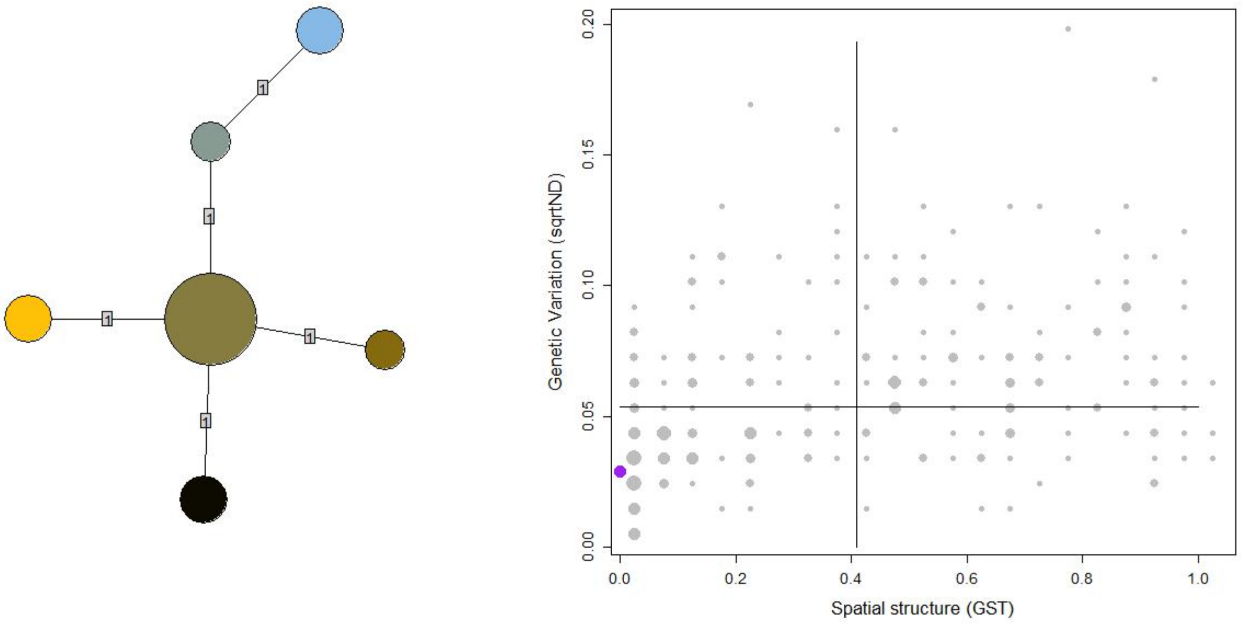

Figure 759: Haplotype network (left) of *Erebia rossii* sequences > 599 bp with colours matching the PCoA colour space (above). The bubble plot for mt-DNA polymorphism (square root transformed nucleotide diversity) and spatial structure (GST) among all species in the atlas and values for *Erebia rossii* (purple dot). The horizontal and vertical lines represent median values of nucleotide diversity and GST, respectively. Sequences > 599 bp= 36.

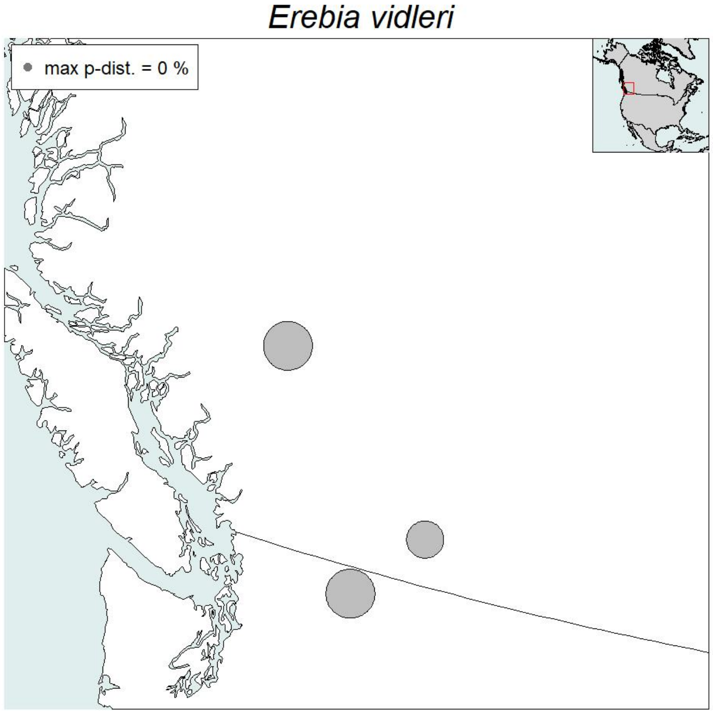

Figure 760: Map of *Erebia vidleri* showing the localities of the sequenced specimens. Nearby localities are grouped in pies. Due to the presence of a single haplotype PCoA projection was not done and a single grey colour was plotted on the map. Sequences= 7; Hap obs.= 1; Hap asympt.= NA; Hap % obs.= NA%; GST= NaN; DST= NaN; HD= NA; ND= NA; max p-dist= 0%.

Haplotype network analysis and bubble plot of *Erebia vidleri* were not possible. Sequences > 599 bp = 7.

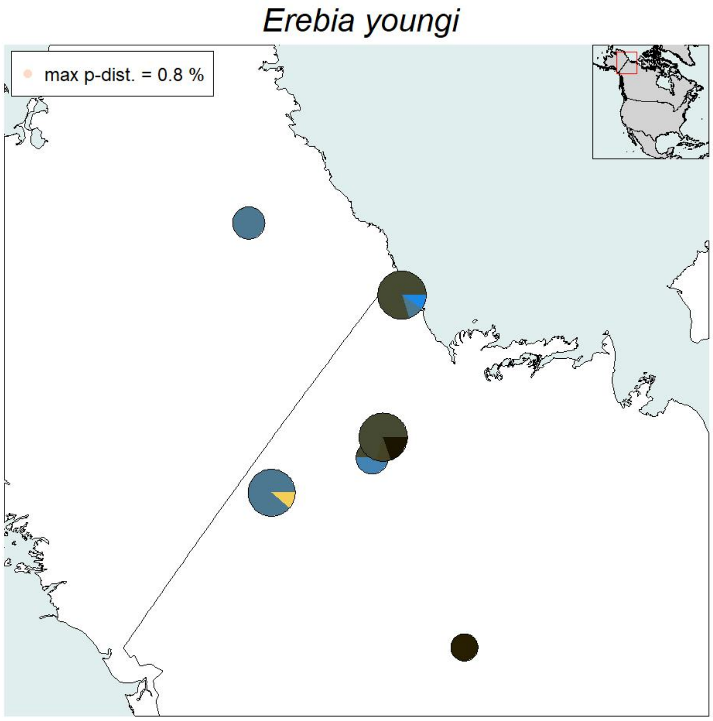

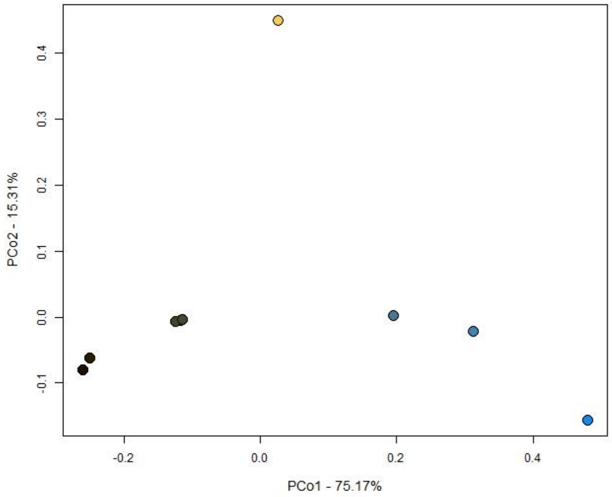

Figure 761 Map of *Erebia youngi* showing the localities of the sequenced specimens (left). Nearby localities are grouped in pies. Colours match the bidimensional colour space of the PCoA projection (right) of max p-dists among sequences (dots). Sequences= 36; Hap obs.= 7; Hap asympt.= 14.8; Hap % obs.= 47.4%; GST= 0.356; DST= 0.0007; HD= 0.64; ND= 0.0022; max p-dist= 0.8%.

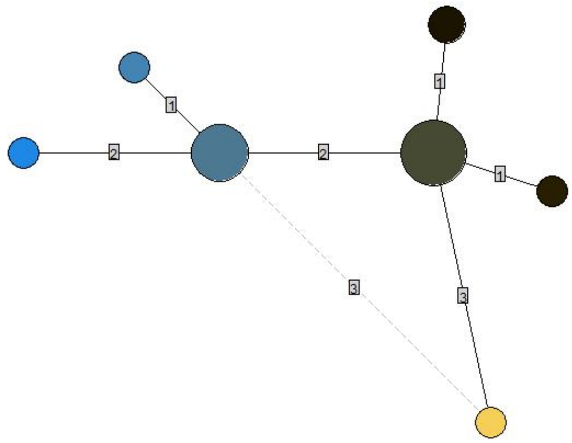

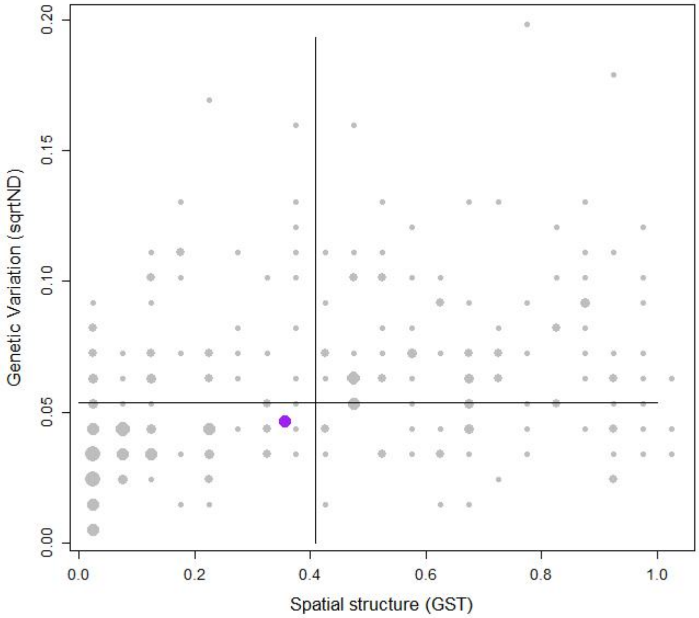

Figure 762: Haplotype network (left) of *Erebia youngi* sequences > 599 bp with colours matching the PCoA colour space (above). The bubble plot for mt-DNA polymorphism (square root transformed nucleotide diversity) and spatial structure (GST) among all species in the atlas and values for *Erebia youngi* (purple dot). The horizontal and vertical lines represent median values of nucleotide diversity and GST, respectively. Sequences > 599 bp= 36.

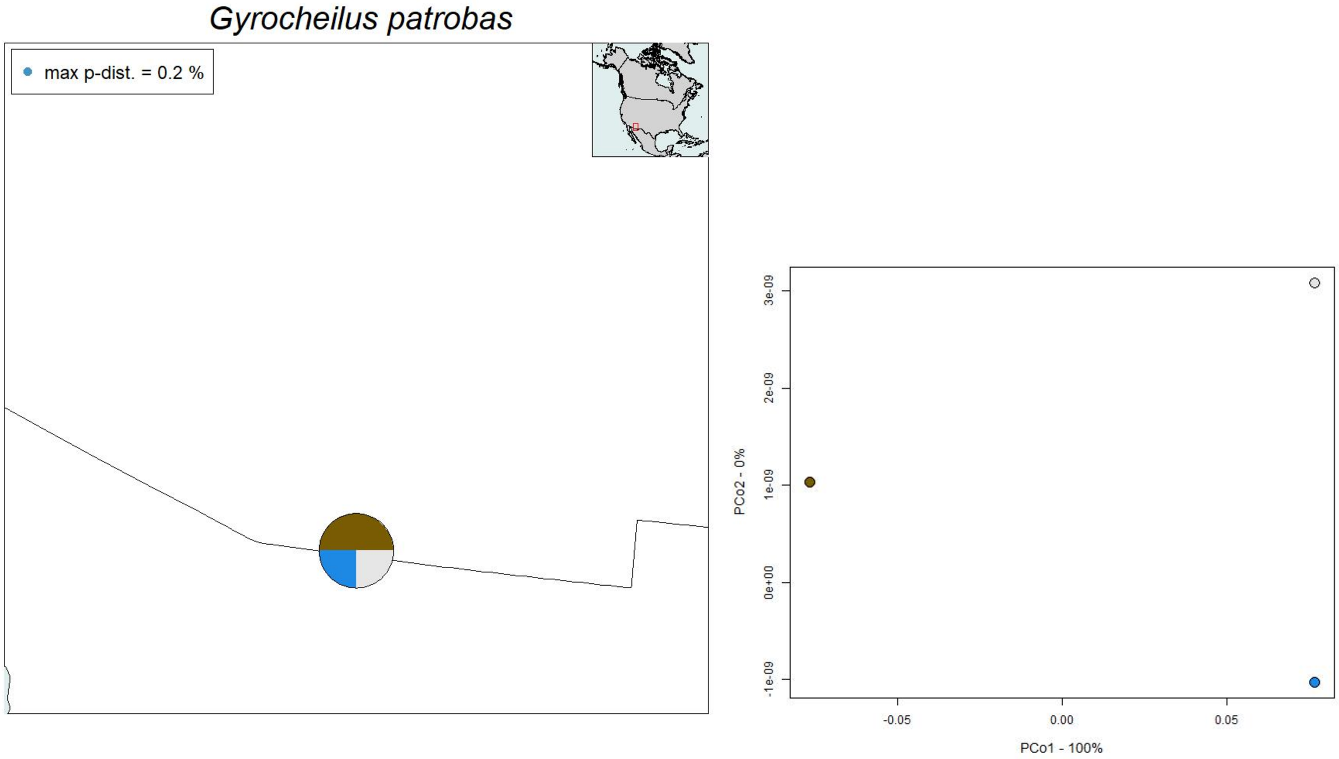

Figure 763 Map of *Gyrocheilus patrobas* showing the localities of the sequenced specimens (left). Nearby localities are grouped in pies. Colours match the bidimensional colour space of the PCoA projection (right) of max p-dists among sequences (dots). Sequences= 4; Hap obs.= 2; Hap asympt.= NA; Hap % obs.= NA%; GST= NaN; DST= NaN; HD= NA; ND= NA; max p-dist= 0.2%.

Haplotype network analysis and bubble plot of *Gyrocheilus patrobas* were not possible. Sequences > 599 bp = 4.

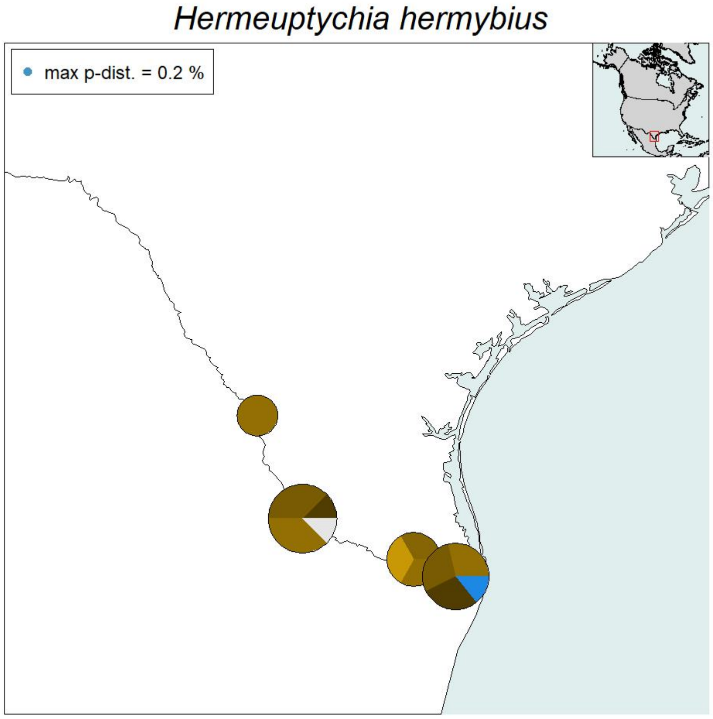

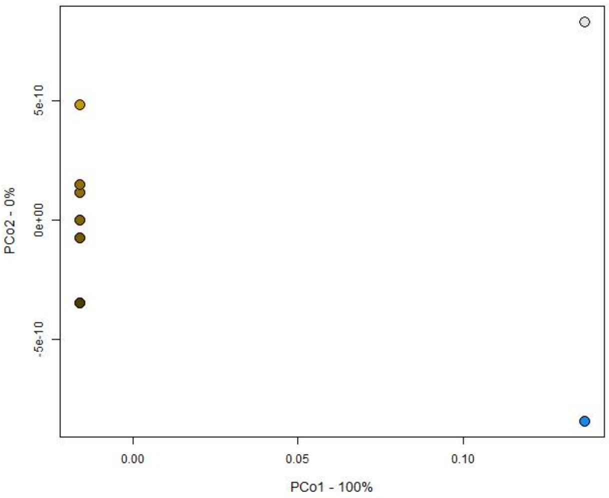

Figure 764 Map of *Hermeuptychia hermybius* showing the localities of the sequenced specimens (left). Nearby localities are grouped in pies. Colours match the bidimensional colour space of the PCoA projection (right) of max p-dists among sequences (dots). Sequences= 19; Hap obs.= 2; Hap asympt.= 2; Hap % obs.= 100%; GST= 0; DST= 0; HD= 0.199; ND= 0.0003; max p-dist= 0.2%.

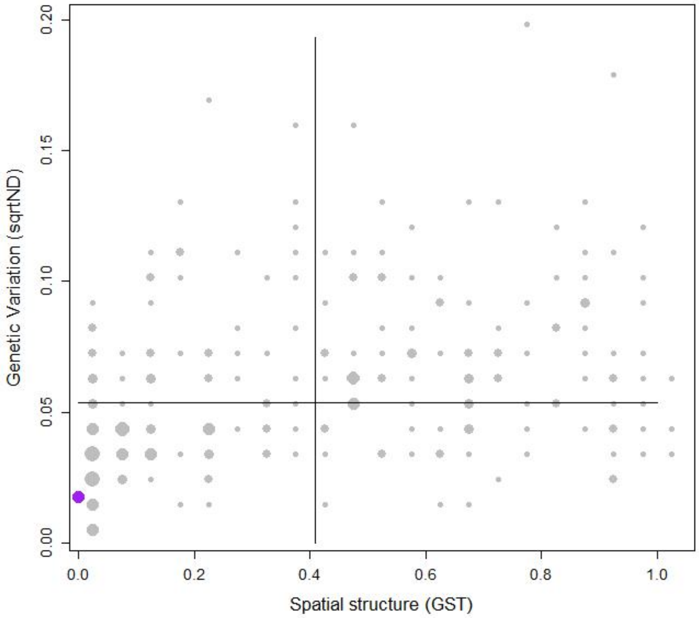

Figure 765: The bubble plot for mt-DNA polymorphism (square root transformed nucleotide diversity) and spatial structuree (GST) among all species in the atlas and values for *Hermeuptychia hermybius* (purple dot). The horizontal and vertical lines represent median values of nucleotide diversity and GST, respectively. Haplotype network analysis was not possible. Sequences > 599 bp = 19.

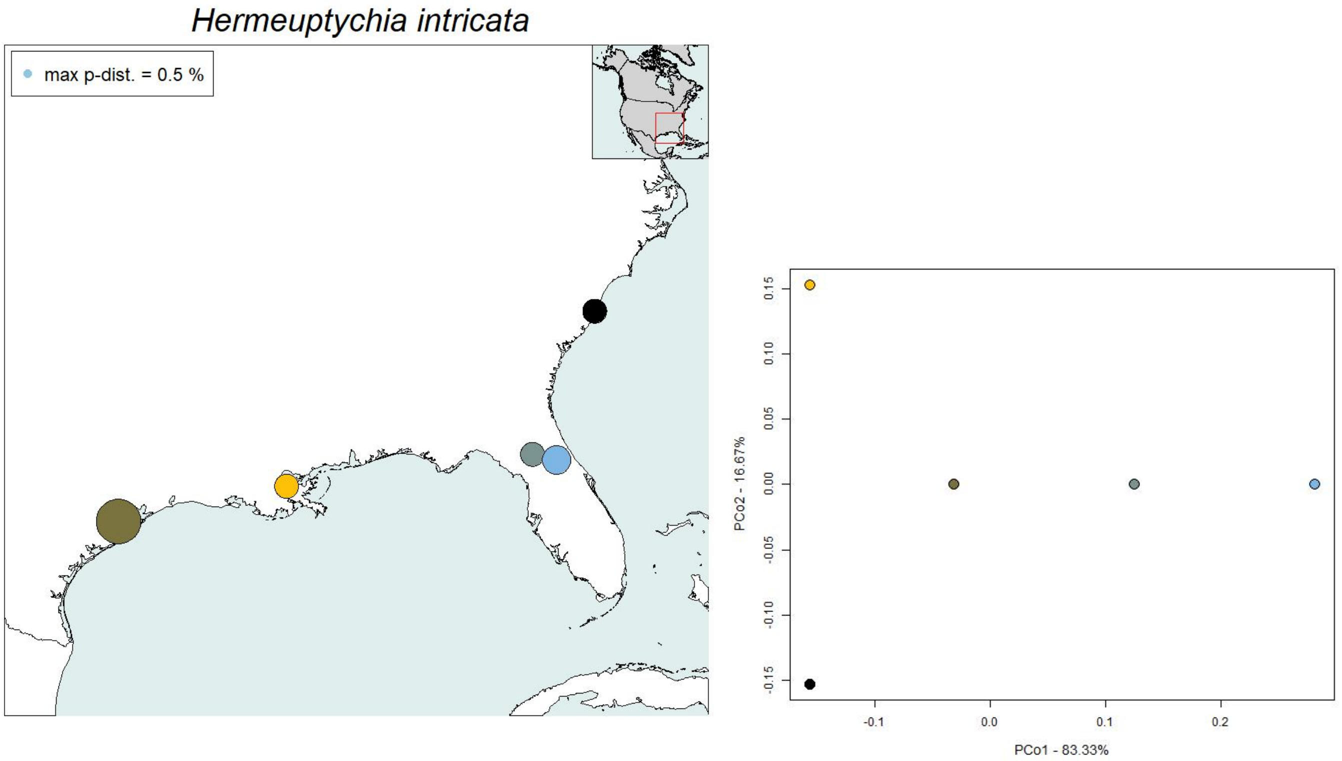

Figure 766 Map of *Hermeuptychia intricata* showing the localities of the sequenced specimens (left). Nearby localities are grouped in pies. Colours match the bidimensional colour space of the PCoA projection (right) of max p-dists among sequences (dots). Sequences= 17; Hap obs.= 5; Hap asympt.= 9.2; Hap % obs.= 54.1%; GST= 0.951; DST= 0.0009; HD= 0.507; ND= 0.0012; max p-dist= 0.5%.

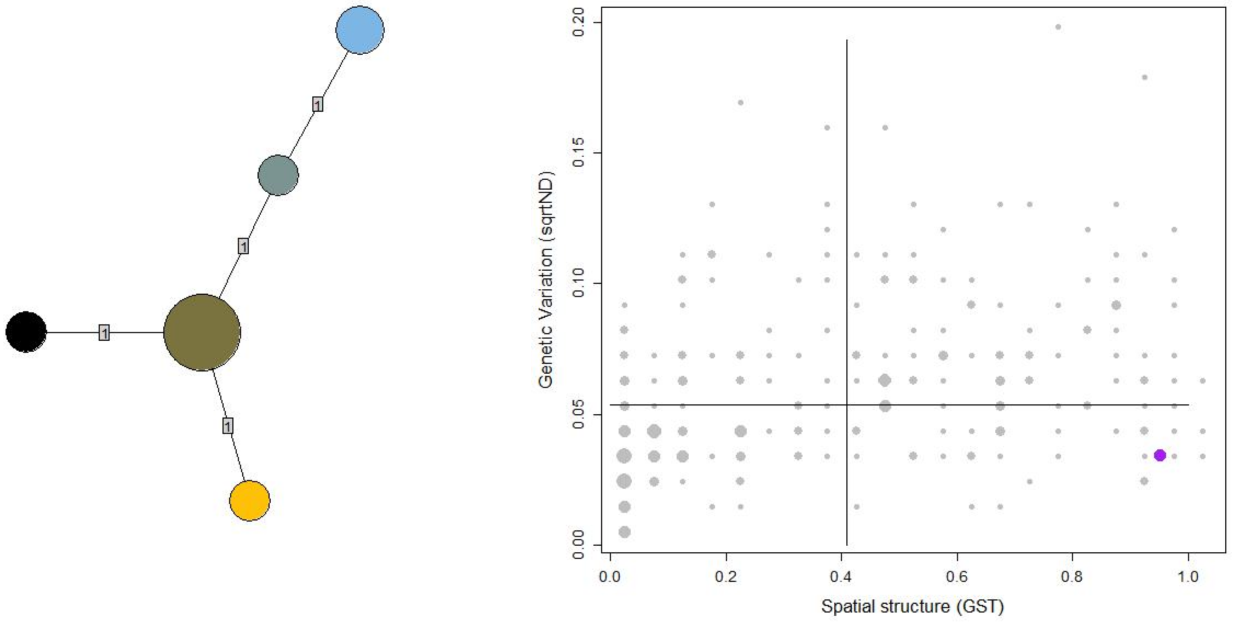

Figure 767: Haplotype network (left) of *Hermeuptychia intricata* sequences > 599 bp with colours matching the PCoA colour space (above). The bubble plot for mt-DNA polymorphism (square root transformed nucleotide diversity) and spatial structure (GST) among all species in the atlas and values for *Hermeuptychia intricata* (purple dot). The horizontal and vertical lines represent median values of nucleotide diversity and GST, respectively. Sequences > 599 bp= 17.

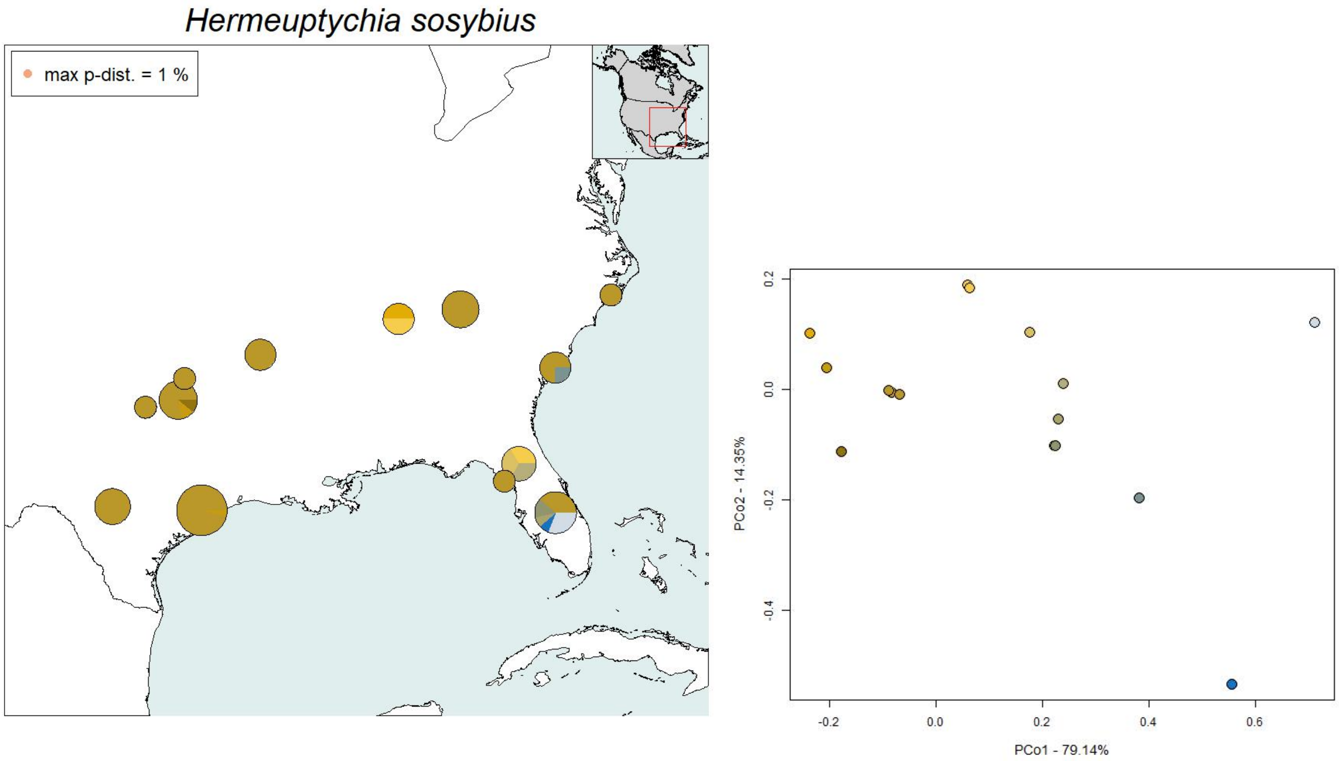

Figure 768 Map of *Hermeuptychia sosybius* showing the localities of the sequenced specimens (left). Nearby localities are grouped in pies. Colours match the bidimensional colour space of the PCoA projection (right) of max p-dists among sequences (dots). Sequences= 87; Hap obs.= 12; Hap asympt.= 16.1; Hap % obs.= 74.4%; GST= 0.514; DST= 0.0006; HD= 0.439; ND= 0.0012; max p-dist= 1%.

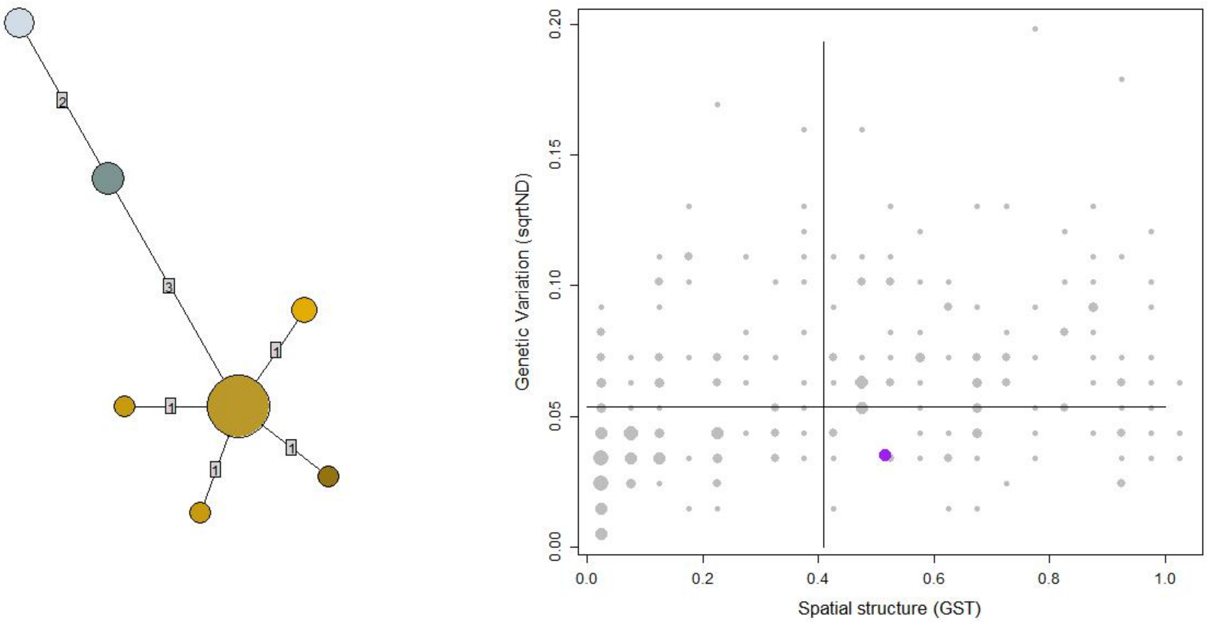

Figure 769: Haplotype network (left) of *Hermeuptychia sosybius* sequences > 599 bp with colours matching the PCoA colour space (above). The bubble plot for mt-DNA polymorphism (square root transformed nucleotide diversity) and spatial structure (GST) among all species in the atlas and values for *Hermeuptychia sosybius* (purple dot). The horizontal and vertical lines represent median values of nucleotide diversity and GST, respectively. Sequences > 599 bp= 84.

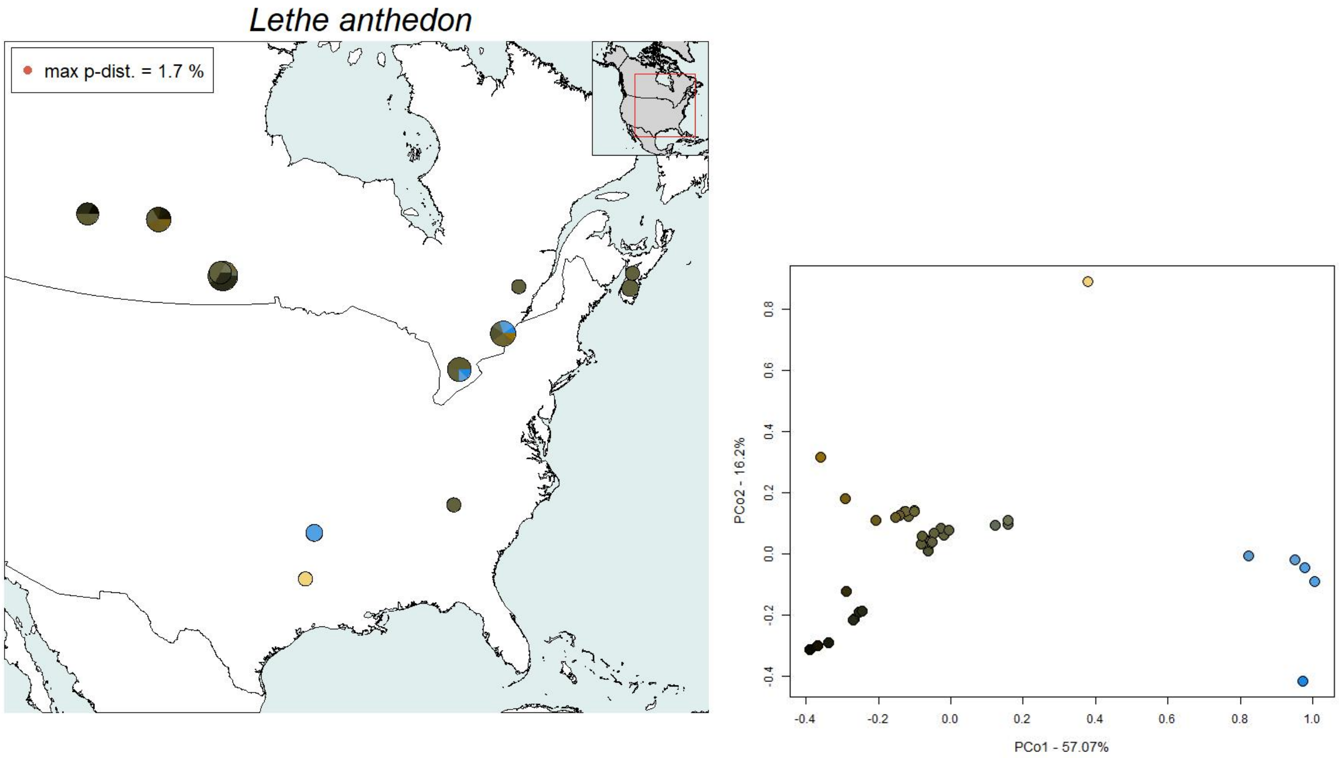

Figure 770 Map of *Lethe anthedon* showing the localities of the sequenced specimens (left). Nearby localities are grouped in pies. Colours match the bidimensional colour space of the PCoA projection (right) of max p-dists among sequences (dots). Sequences= 66; Hap obs.= 21; Hap asympt.= 35.9; Hap % obs.= 58.5%; GST= 0.243; DST= 0.001; HD= 0.899; ND= 0.0045; max p-dist= 1.7%.

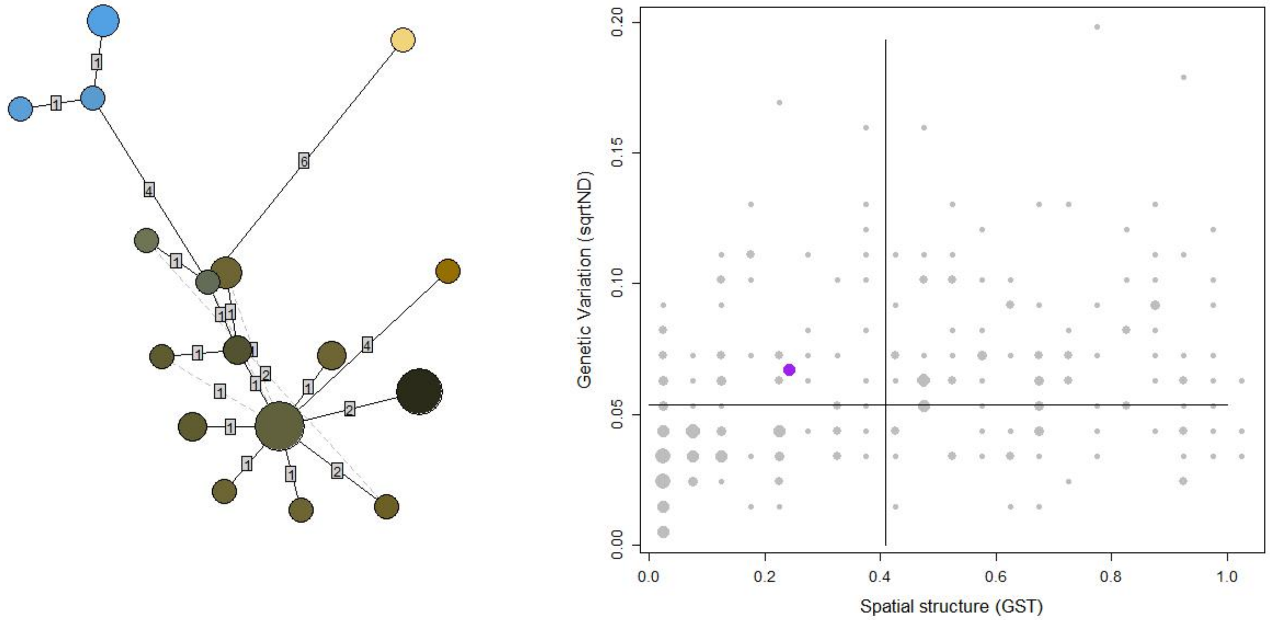

Figure 771: Haplotype network (left) of *Lethe anthedon* sequences > 599 bp with colours matching the PCoA colour space (above). The bubble plot for mt-DNA polymorphism (square root transformed nucleotide diversity) and spatial structure (GST) among all species in the atlas and values for *Lethe anthedon* (purple dot). The horizontal and vertical lines represent median values of nucleotide diversity and GST, respectively. Sequences > 599 bp= 50.

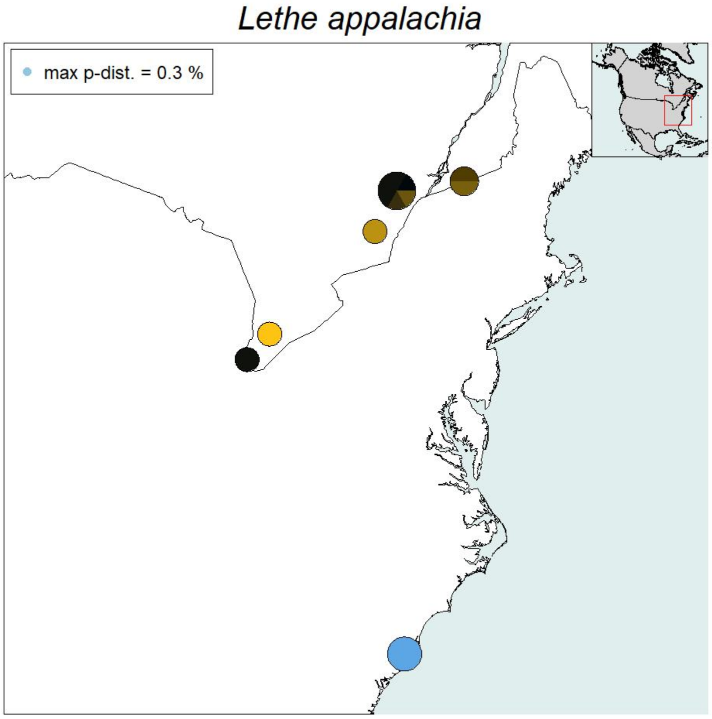

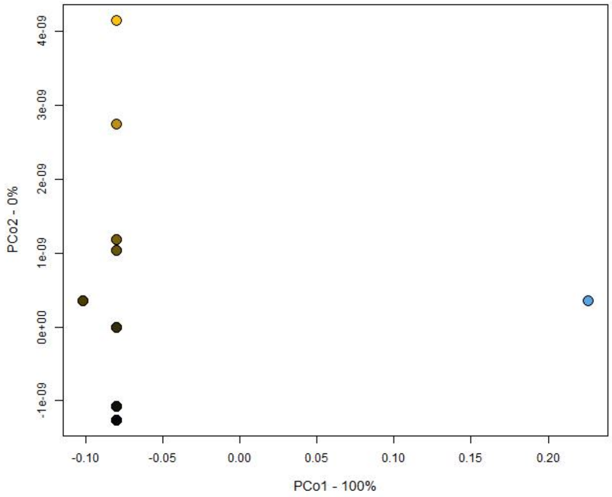

Figure 772 Map of *Lethe appalachia* showing the localities of the sequenced specimens (left). Nearby localities are grouped in pies. Colours match the bidimensional colour space of the PCoA projection (right) of max p-dists among sequences (dots). Sequences= 15; Hap obs.= 2; Hap asympt.= 2; Hap % obs.= 100%; GST= 1; DST= 0.0016; HD= 0.419; ND= 0.0013; max p-dist= 0.3%.

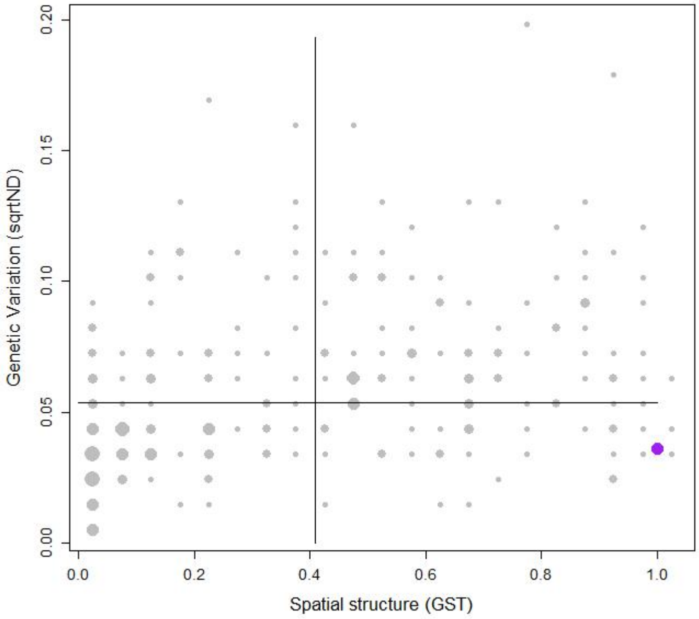

Figure 773: The bubble plot for mt-DNA polymorphism (square root transformed nucleotide diversity) and spatial structuree (GST) among all species in the atlas and values for *Lethe appalachia* (purple dot). The horizontal and vertical lines represent median values of nucleotide diversity and GST, respectively. Haplotype network analysis was not possible. Sequences > 599 bp = 15.

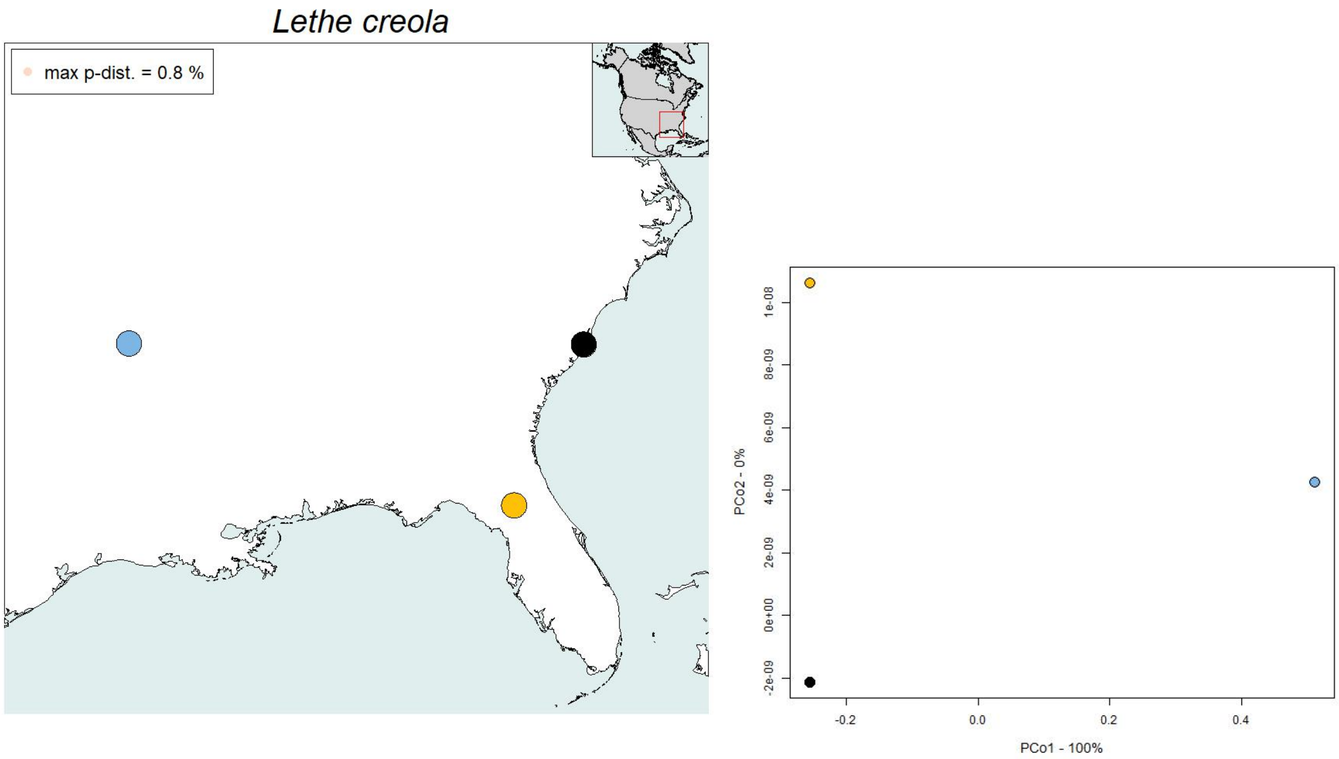

Figure 774 Map of *Lethe creola* showing the localities of the sequenced specimens (left). Nearby localities are grouped in pies. Colours match the bidimensional colour space of the PCoA projection (right) of max p-dists among sequences (dots). Sequences= 3; Hap obs.= 2; Hap asympt.= NA; Hap % obs.= NA%; GST= NaN; DST= NaN; HD= NA; ND= NA; max p-dist= 0.8%.

Haplotype network analysis and bubble plot of *Lethe creola* were not possible. Sequences > 599 bp = 3.

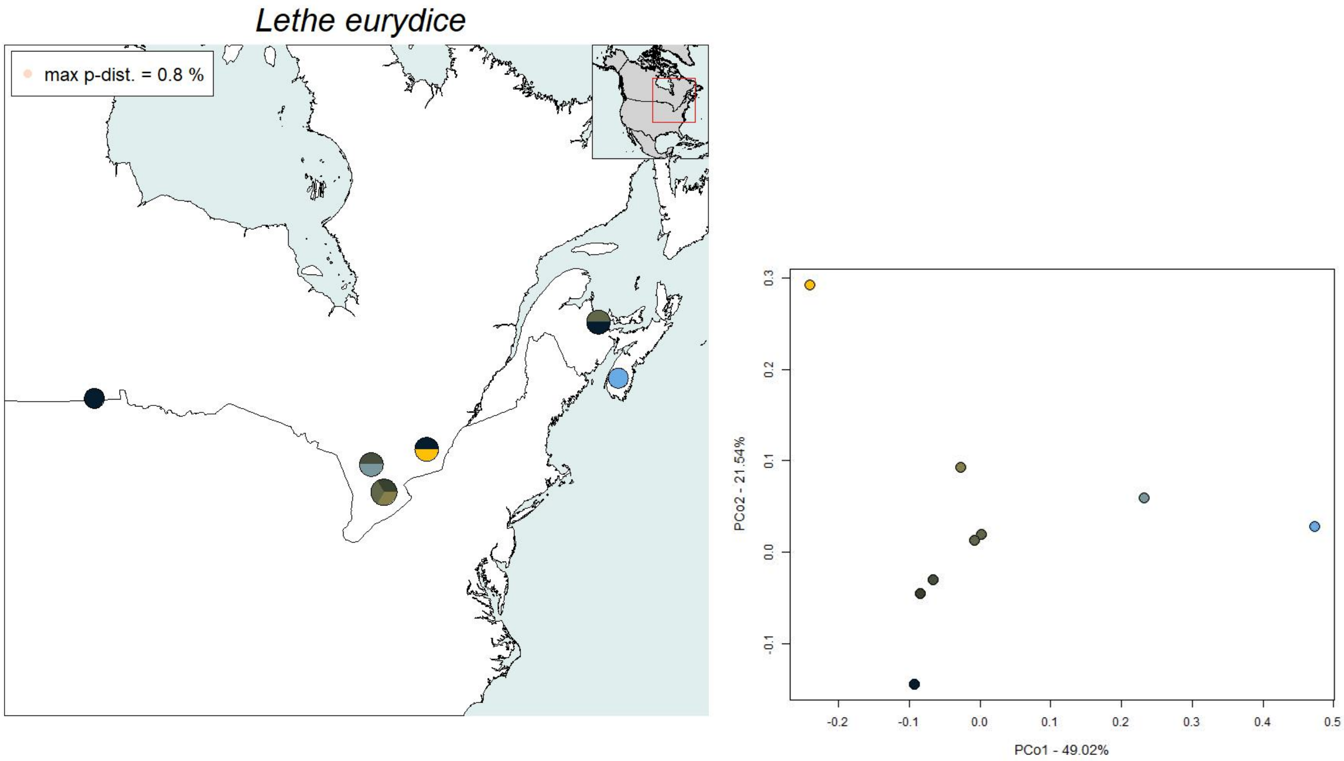

Figure 775 Map of *Lethe eurydice* showing the localities of the sequenced specimens (left). Nearby localities are grouped in pies. Colours match the bidimensional colour space of the PCoA projection (right) of max p-dists among sequences (dots). Sequences= 11; Hap obs.= 7; Hap asympt.= 16.1; Hap % obs.= 43.5%; GST= NaN; DST= NaN; HD= 0.891; ND= 0.0031; max p-dist= 0.8%.

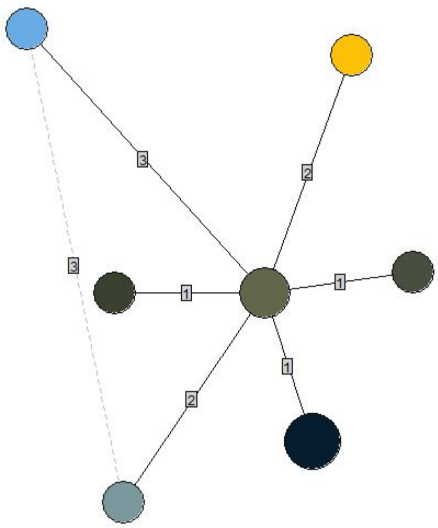

Figure 776: Haplotype network of *Lethe eurydice*. Sequences > 599 bp= 10.

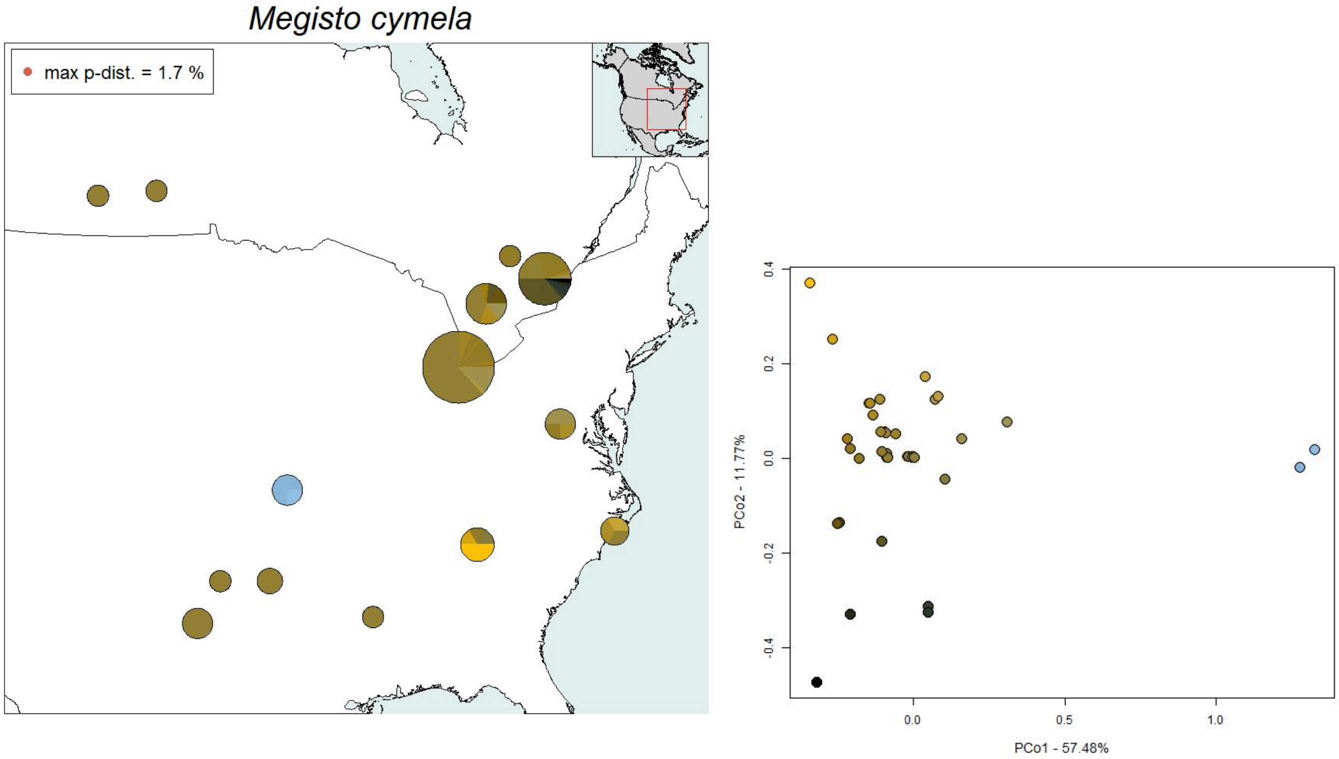

Figure 777 Map of *Megisto cymela* showing the localities of the sequenced specimens (left). Nearby localities are grouped in pies. Colours match the bidimensional colour space of the PCoA projection (right) of max p-dists among sequences (dots). Sequences= 203; Hap obs.= 32; Hap asympt.= 67.9; Hap % obs.= 47.1%; GST= 0.441; DST= 0.0009; HD= 0.728; ND= 0.002; max p-dist= 1.7%.

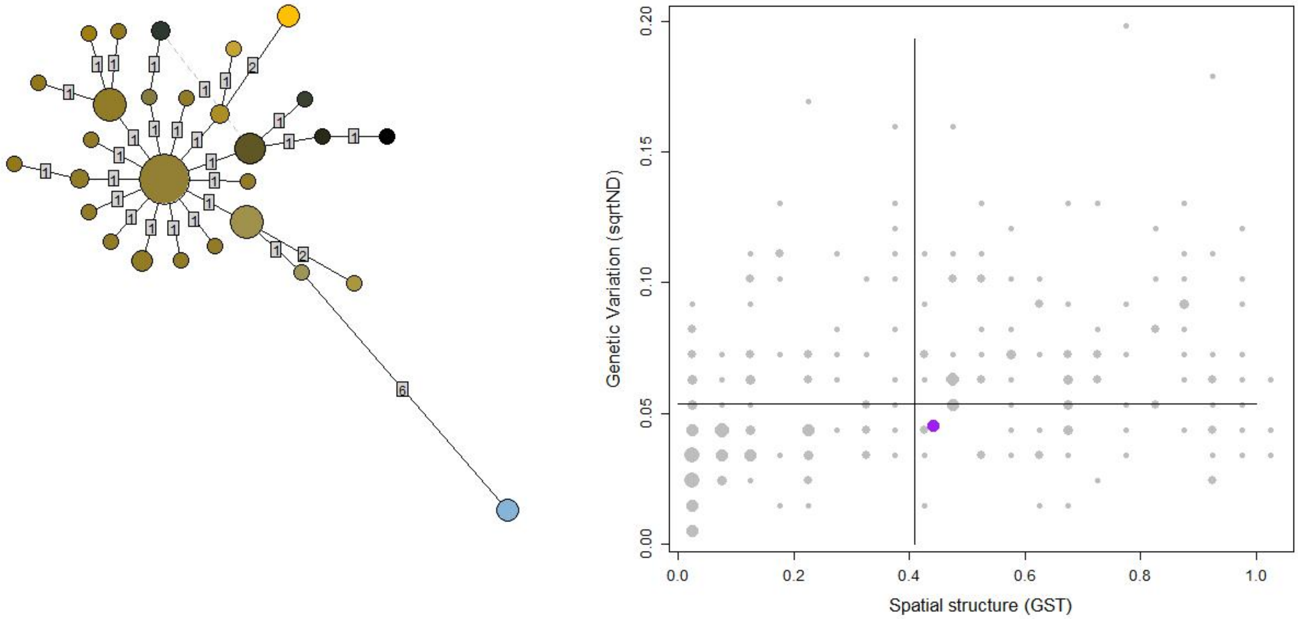

Figure 778: Haplotype network (left) of *Megisto cymela* sequences > 599 bp with colours matching the PCoA colour space (above). The bubble plot for mt-DNA polymorphism (square root transformed nucleotide diversity) and spatial structure (GST) among all species in the atlas and values for *Megisto cymela* (purple dot). The horizontal and vertical lines represent median values of nucleotide diversity and GST, respectively. Sequences > 599 bp= 190.

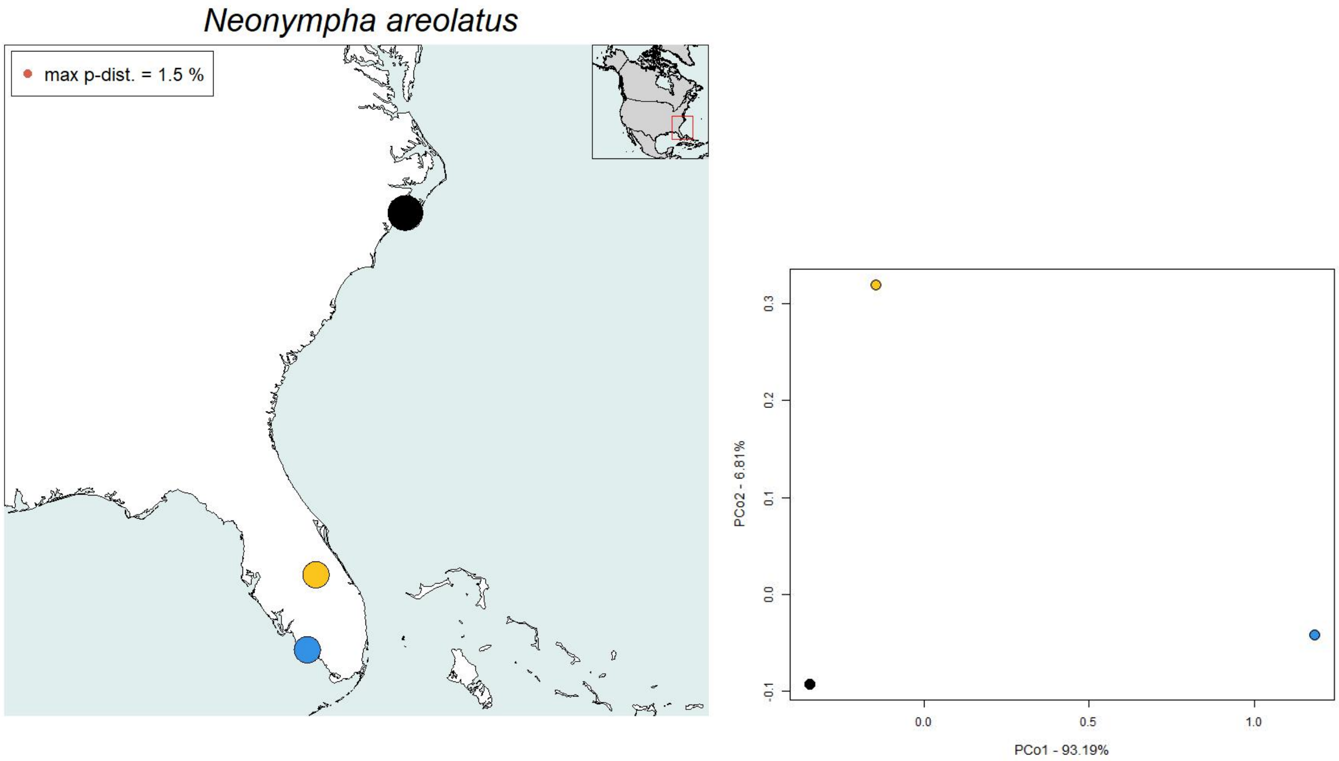

Figure 779 Map of *Neonympha areolatus* showing the localities of the sequenced specimens (left). Nearby localities are grouped in pies. Colours match the bidimensional colour space of the PCoA projection (right) of max p-dists among sequences (dots). Sequences= 5; Hap obs.= 3; Hap asympt.= NA; Hap % obs.= NA%; GST= NaN; DST= NaN; HD= NA; ND= NA; max p-dist= 1.5%.

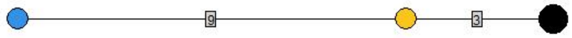

Figure 780: Haplotype network of *Neonympha areolatus*. Sequences > 599 bp= 5.

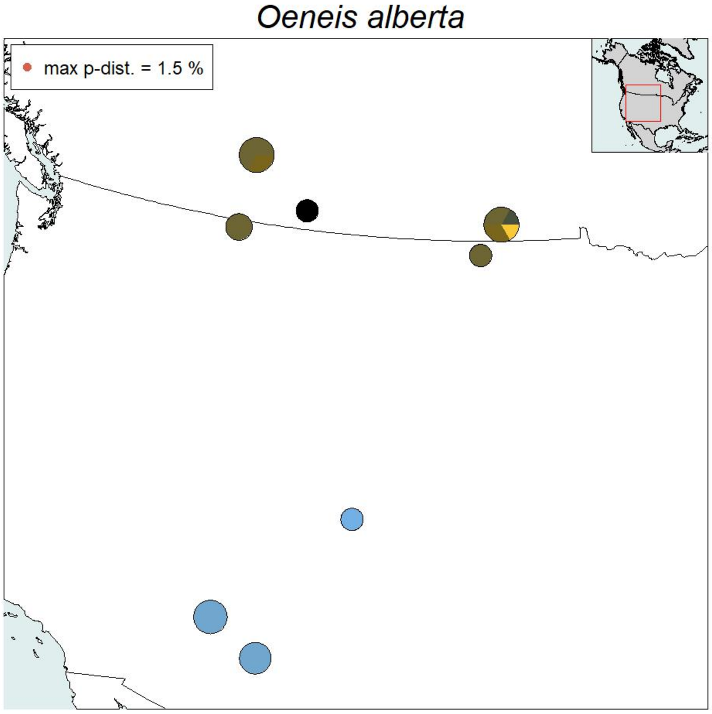

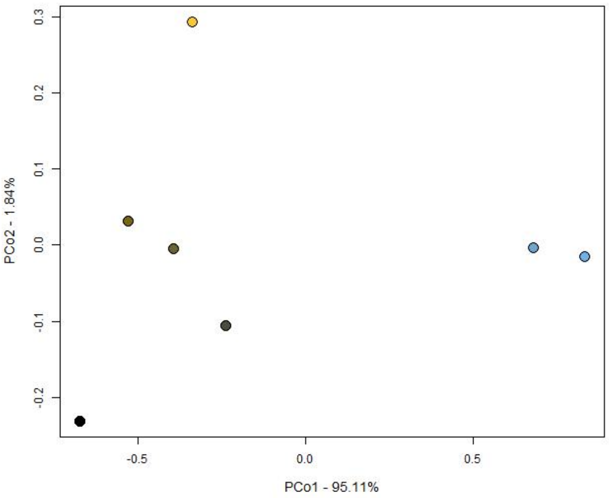

Figure 781 Map of *Oeneis alberta* showing the localities of the sequenced specimens (left). Nearby localities are grouped in pies. Colours match the bidimensional colour space of the PCoA projection (right) of max p-dists among sequences (dots). Sequences= 26; Hap obs.= 10; Hap asympt.= 36.9; Hap % obs.= 27.1%; GST= 0.803; DST= 0.005; HD= 0.778; ND= 0.0063; max p-dist= 1.5%.

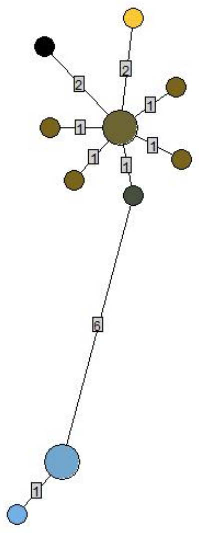

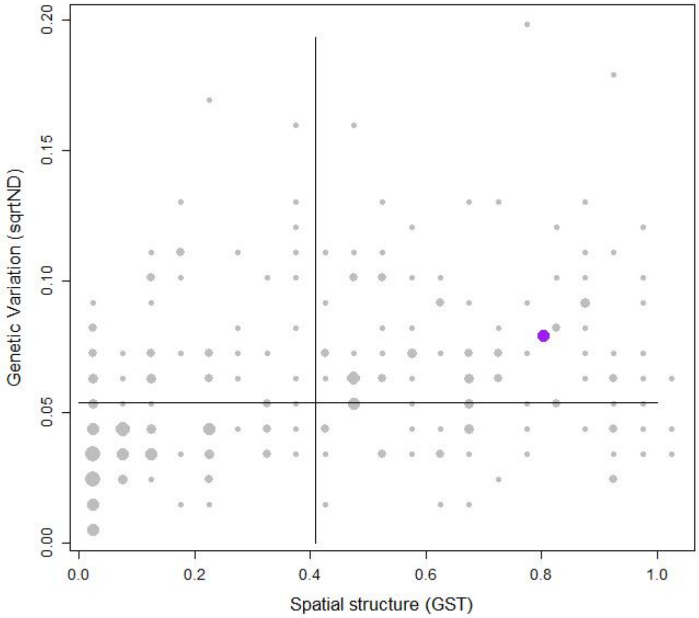

Figure 782: Haplotype network (left) of *Oeneis alberta* sequences > 599 bp with colours matching the PCoA colour space (above). The bubble plot for mt-DNA polymorphism (square root transformed nucleotide diversity) and spatial structure (GST) among all species in the atlas and values for *Oeneis alberta* (purple dot). The horizontal and vertical lines represent median values of nucleotide diversity and GST, respectively. Sequences > 599 bp= 26.

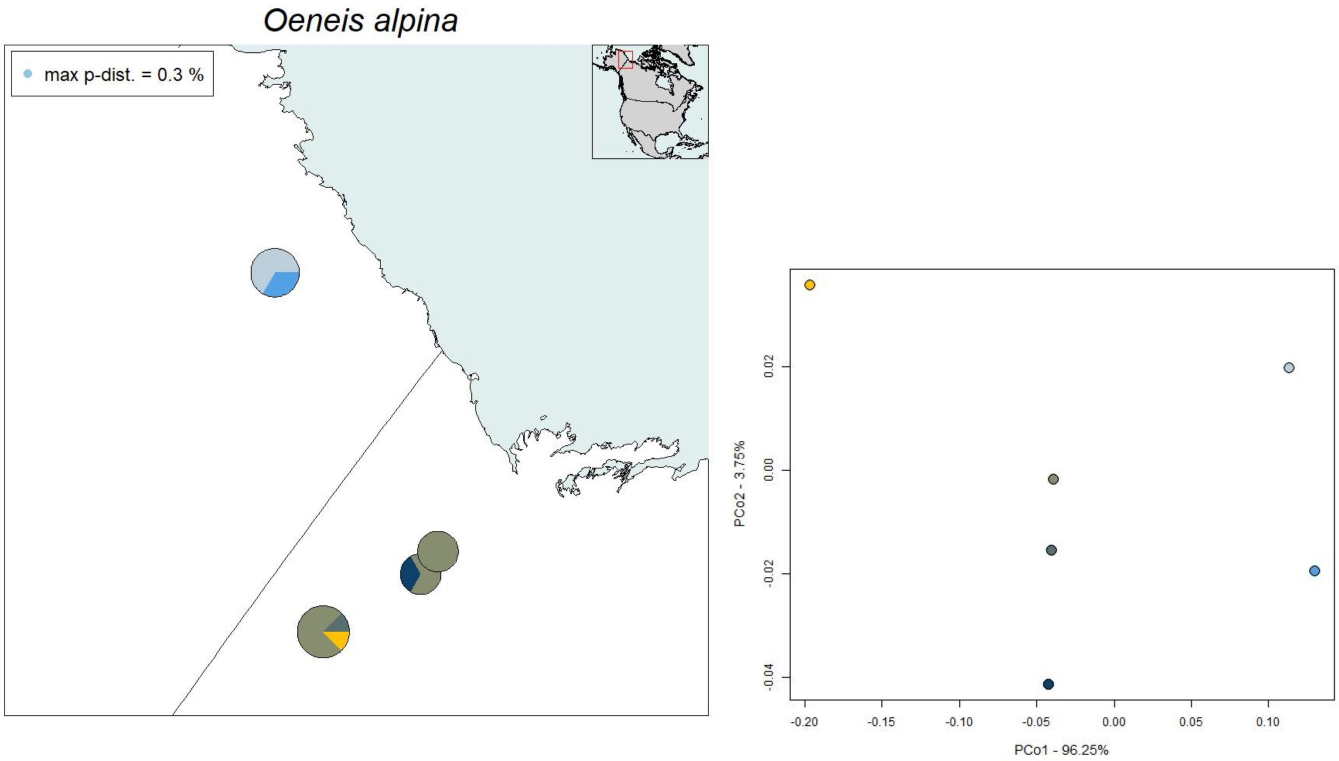

Figure 783 Map of *Oeneis alpina* showing the localities of the sequenced specimens (left). Nearby localities are grouped in pies. Colours match the bidimensional colour space of the PCoA projection (right) of max p-dists among sequences (dots). Sequences= 20; Hap obs.= 3; Hap asympt.= 3; Hap % obs.= 100%; GST= 0.763; DST= 0.0007; HD= 0.511; ND= 0.0009; max p-dist= 0.3%.

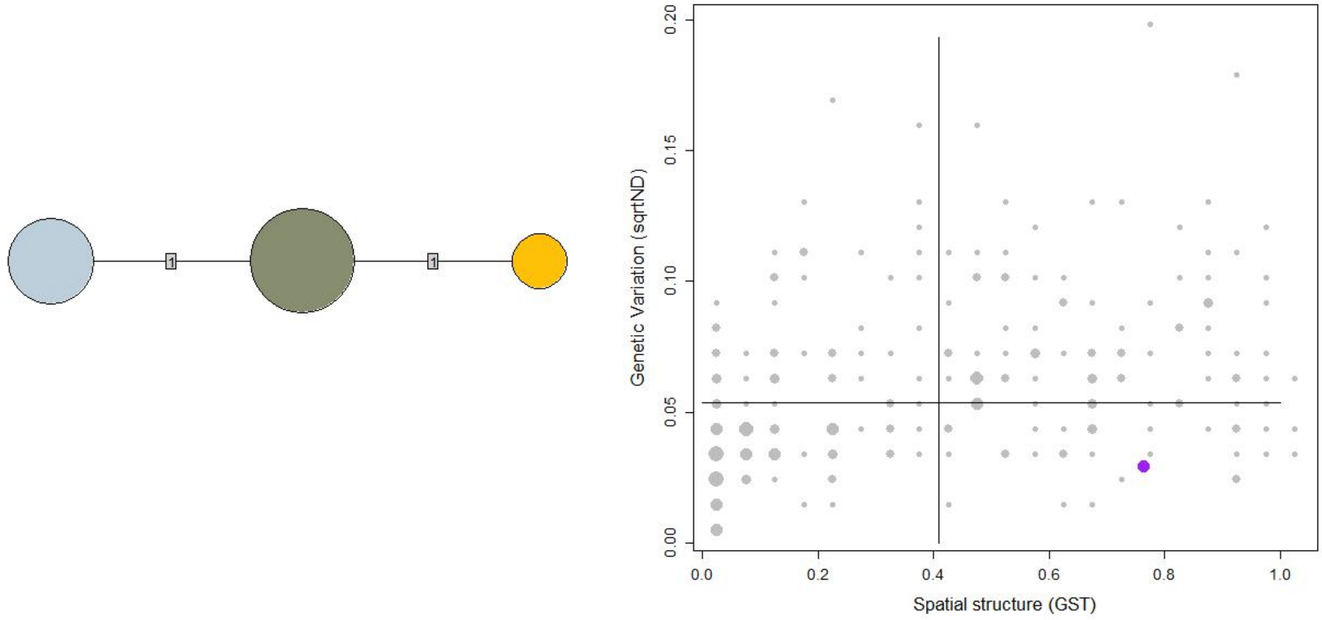

Figure 784: Haplotype network (left) of *Oeneis alpina* sequences > 599 bp with colours matching the PCoA colour space (above). The bubble plot for mt-DNA polymorphism (square root transformed nucleotide diversity) and spatial structure (GST) among all species in the atlas and values for *Oeneis alpina* (purple dot). The horizontal and vertical lines represent median values of nucleotide diversity and GST, respectively. Sequences > 599 bp= 20.

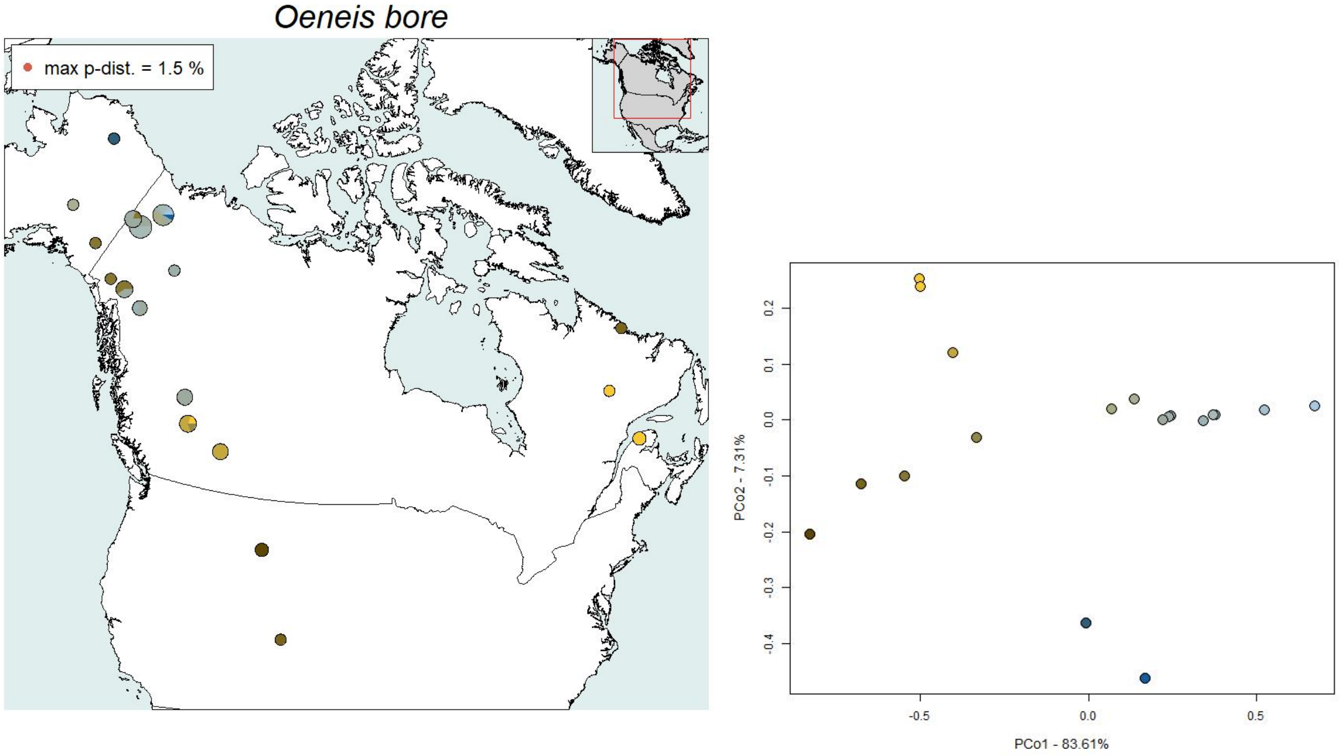

Figure 785 Map of *Oeneis bore* showing the localities of the sequenced specimens (left). Nearby localities are grouped in pies. Colours match the bidimensional colour space of the PCoA projection (right) of max p-dists among sequences (dots). Sequences= 66; Hap obs.= 20; Hap asympt.= 61.6; Hap % obs.= 32.5%; GST= 0.518; DST= 0.0019; HD= 0.851; ND= 0.0045; max p-dist= 1.5%.

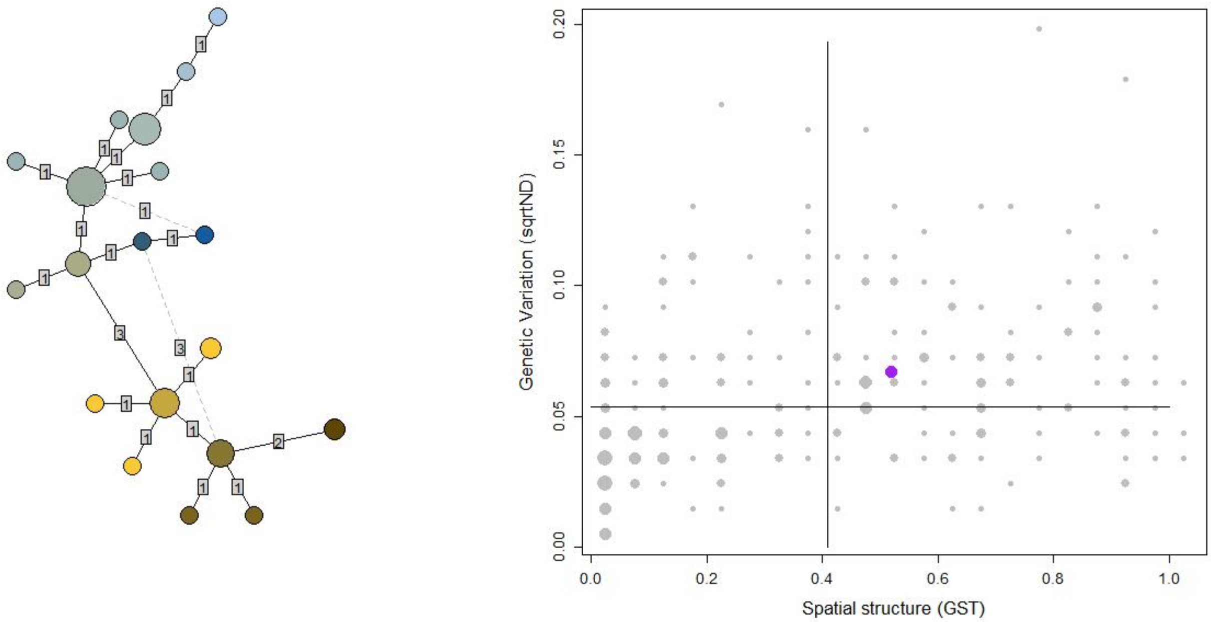

Figure 786: Haplotype network (left) of *Oeneis bore* sequences > 599 bp with colours matching the PCoA colour space (above). The bubble plot for mt-DNA polymorphism (square root transformed nucleotide diversity) and spatial structure (GST) among all species in the atlas and values for *Oeneis bore* (purple dot). The horizontal and vertical lines represent median values of nucleotide diversity and GST, respectively. Sequences > 599 bp= 66.

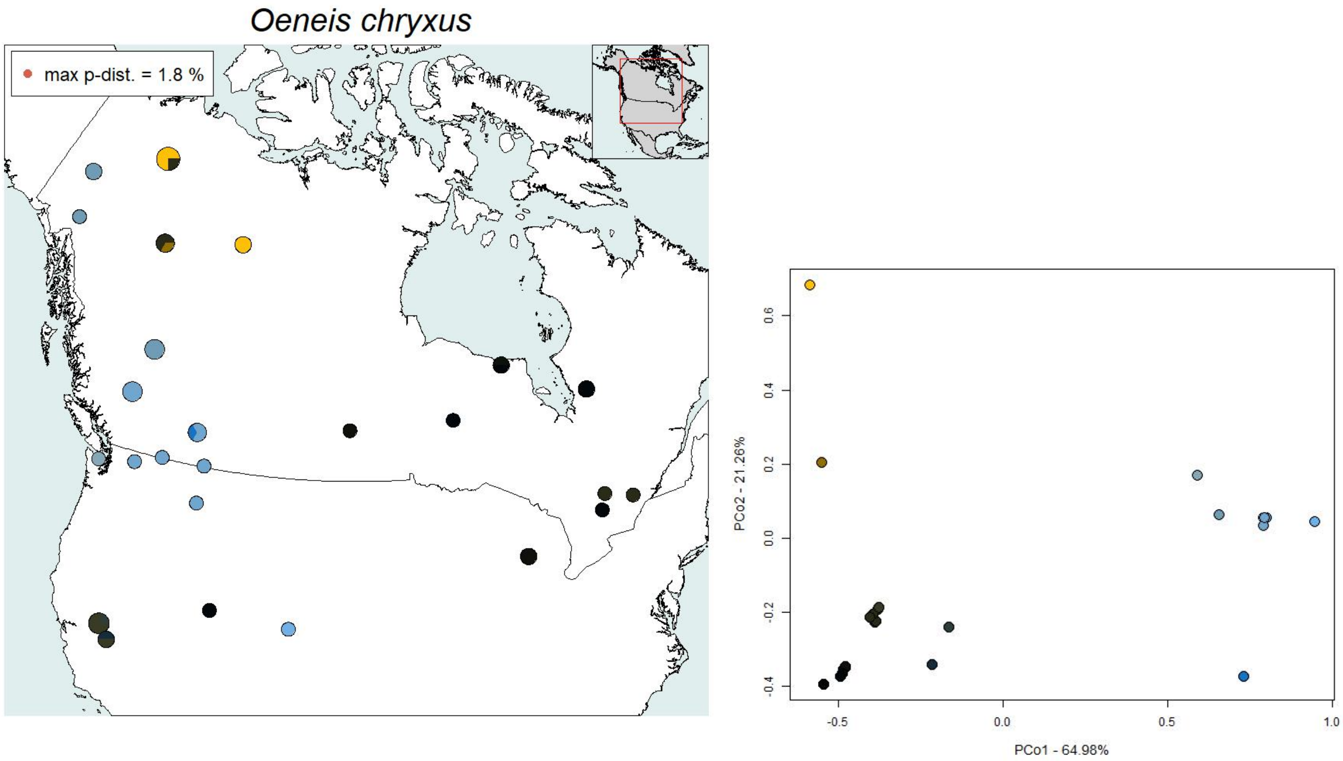

Figure 787 Map of *Oeneis chryxus* showing the localities of the sequenced specimens (left). Nearby localities are grouped in pies. Colours match the bidimensional colour space of the PCoA projection (right) of max p-dists among sequences (dots). Sequences= 52; Hap obs.= 20; Hap asympt.= 36.3; Hap % obs.= 55%; GST= 0.863; DST= 0.0073; HD= 0.934; ND= 0.0089; max p-dist= 1.8%.

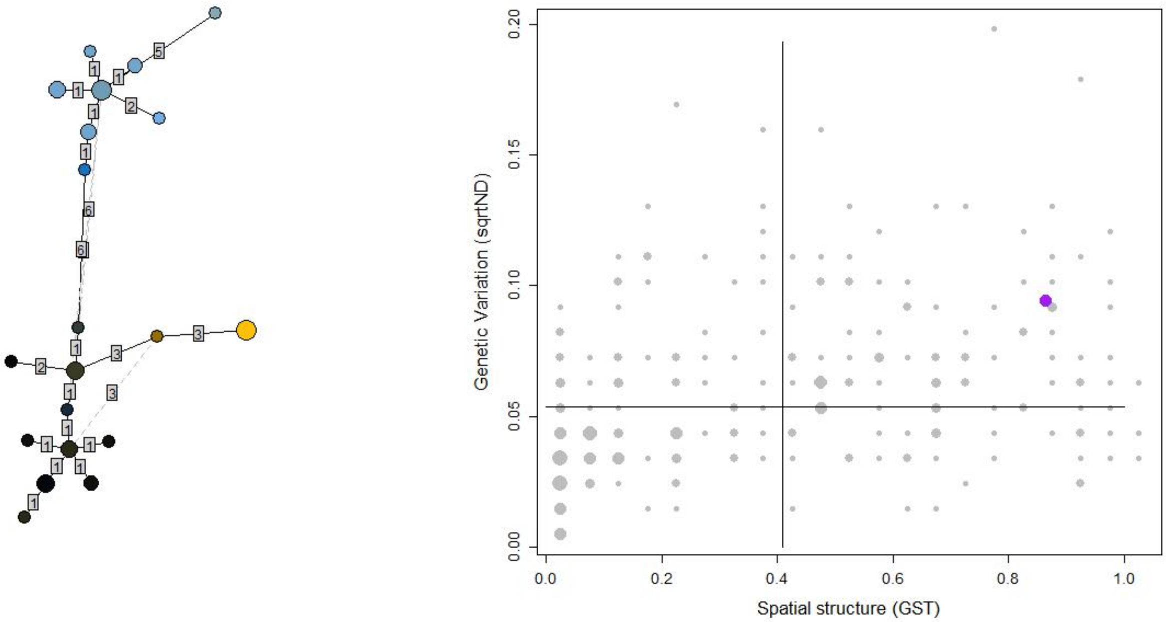

Figure 788: Haplotype network (left) of *Oeneis chryxus* sequences > 599 bp with colours matching the PCoA colour space (above). The bubble plot for mt-DNA polymorphism (square root transformed nucleotide diversity) and spatial structure (GST) among all species in the atlas and values for *Oeneis chryxus* (purple dot). The horizontal and vertical lines represent median values of nucleotide diversity and GST, respectively. Sequences > 599 bp= 51.

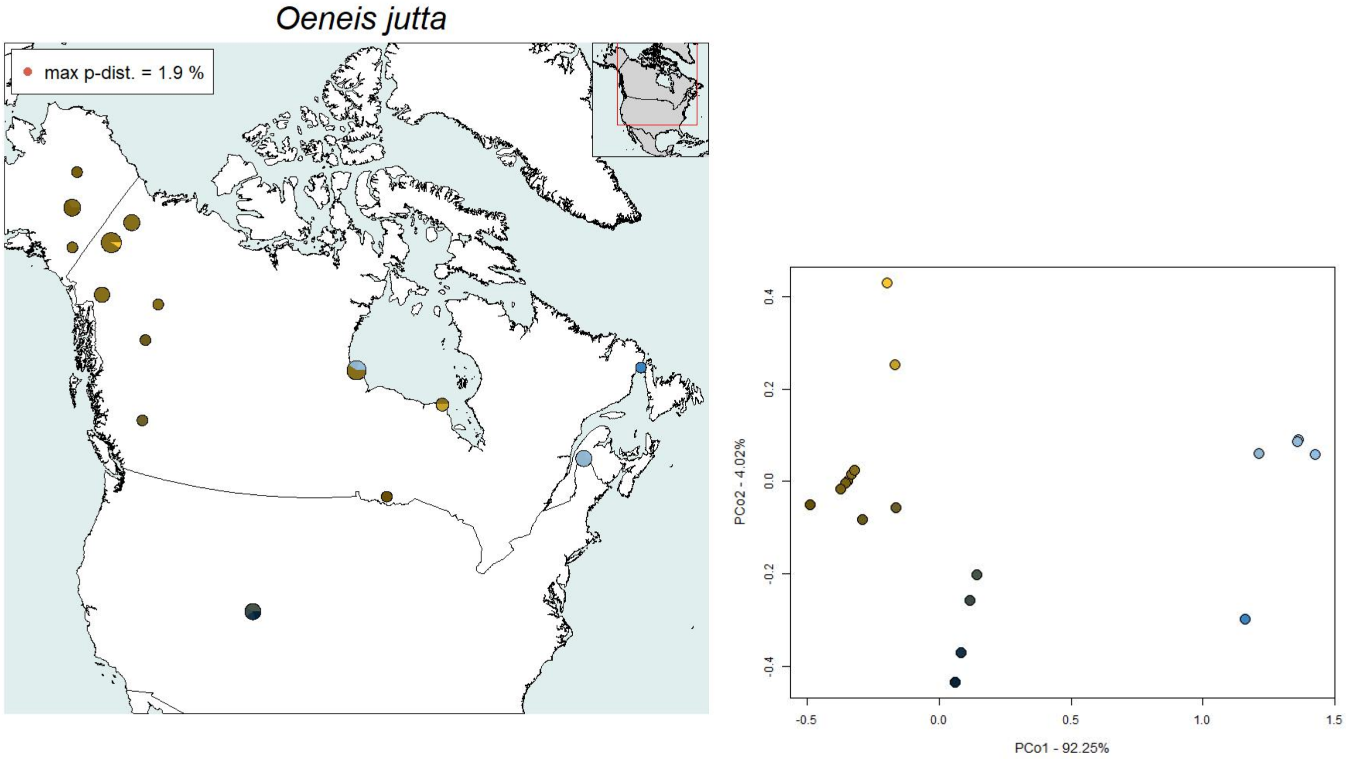

Figure 789 Map of *Oeneis jutta* showing the localities of the sequenced specimens (left). Nearby localities are grouped in pies. Colours match the bidimensional colour space of the PCoA projection (right) of max p-dists among sequences (dots). Sequences= 56; Hap obs.= 12; Hap asympt.= 43.4; Hap % obs.= 27.6%; GST= 0.409; DST= 0.0024; HD= 0.555; ND= 0.0055; max p-dist= 1.9%.

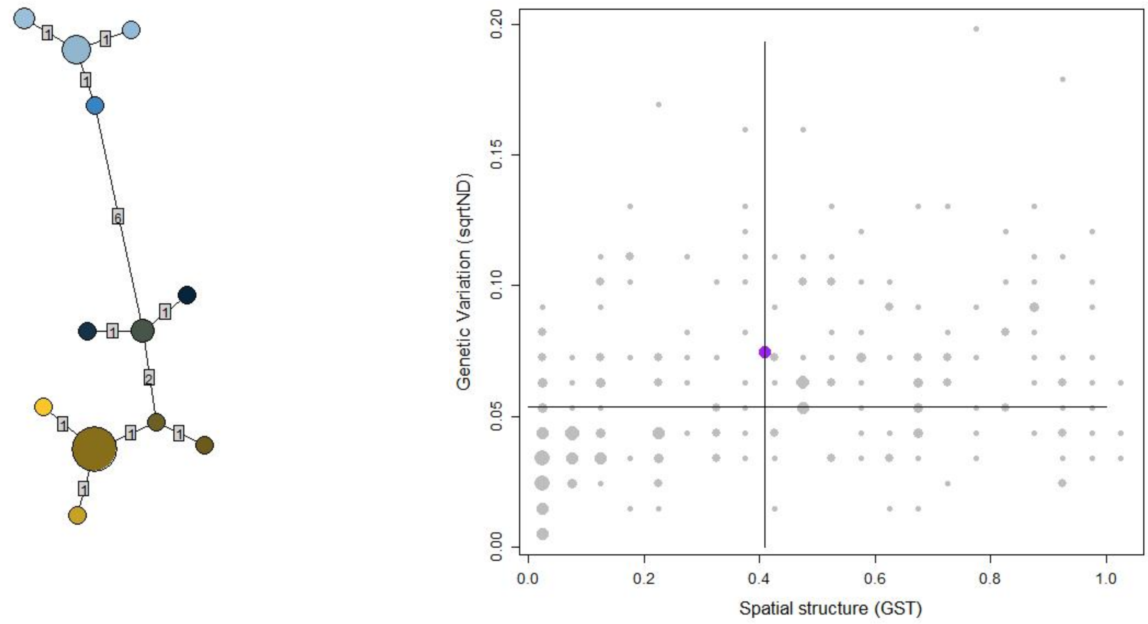

Figure 790: Haplotype network (left) of *Oeneis jutta* sequences > 599 bp with colours matching the PCoA colour space (above). The bubble plot for mt-DNA polymorphism (square root transformed nucleotide diversity) and spatial structure (GST) among all species in the atlas and values for *Oeneis jutta* (purple dot). The horizontal and vertical lines represent median values of nucleotide diversity and GST, respectively. Sequences > 599 bp= 55.

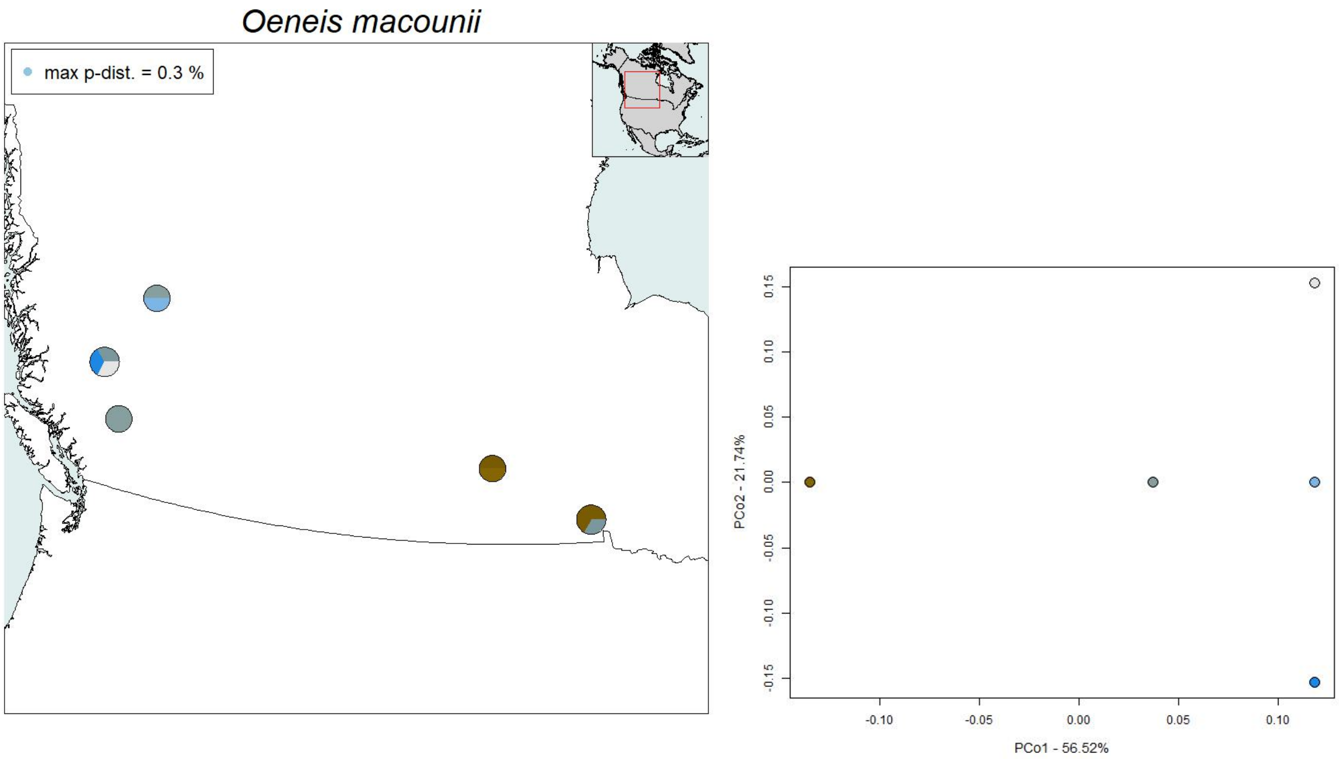

Figure 791 Map of *Oeneis macounii* showing the localities of the sequenced specimens (left). Nearby localities are grouped in pies. Colours match the bidimensional colour space of the PCoA projection (right) of max p-dists among sequences (dots). Sequences= 12; Hap obs.= 5; Hap asympt.= 7.8; Hap % obs.= 64.5%; GST= NaN; DST= NaN; HD= 0.758; ND= 0.0015; max p-dist= 0.3%.

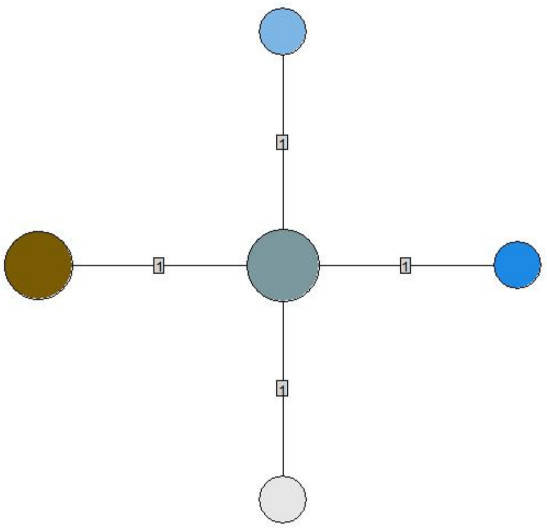

Figure 792: Haplotype network of *Oeneis macounii*. Sequences > 599 bp= 12.

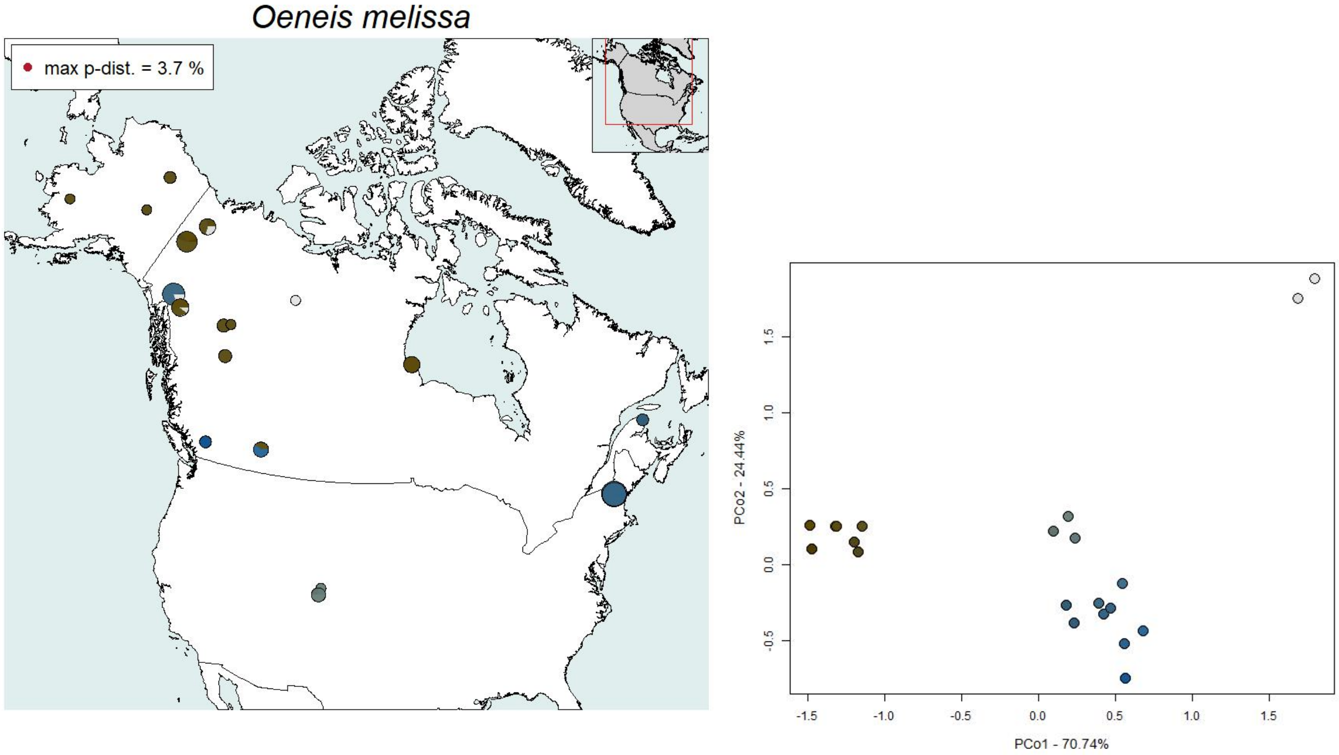

Figure 793 Map of *Oeneis melissa* showing the localities of the sequenced specimens (left). Nearby localities are grouped in pies. Colours match the bidimensional colour space of the PCoA projection (right) of max p-dists among sequences (dots). Sequences= 166; Hap obs.= 20; Hap asympt.= 35.9; Hap % obs.= 55.7%; GST= 0.814; DST= 0.0084; HD= 0.758; ND= 0.0109; max p-dist= 3.7%.

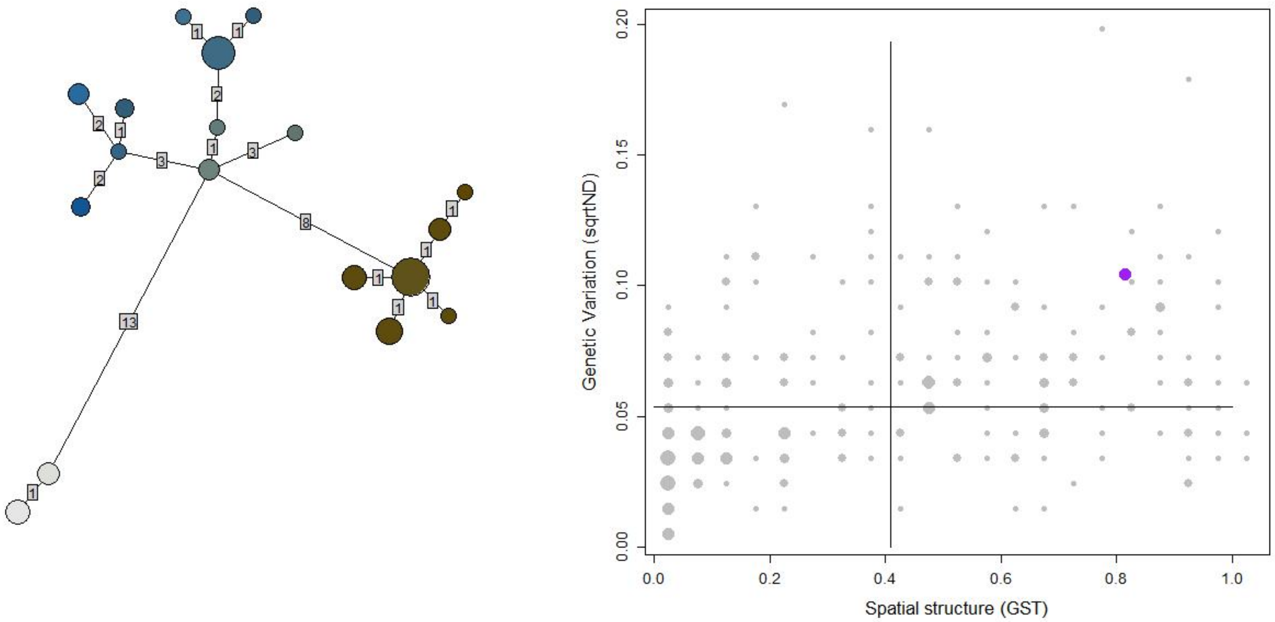

Figure 794: Haplotype network (left) of *Oeneis melissa* sequences > 599 bp with colours matching the PCoA colour space (above). The bubble plot for mt-DNA polymorphism (square root transformed nucleotide diversity) and spatial structure (GST) among all species in the atlas and values for *Oeneis melissa* (purple dot). The horizontal and vertical lines represent median values of nucleotide diversity and GST, respectively. Sequences > 599 bp= 92.

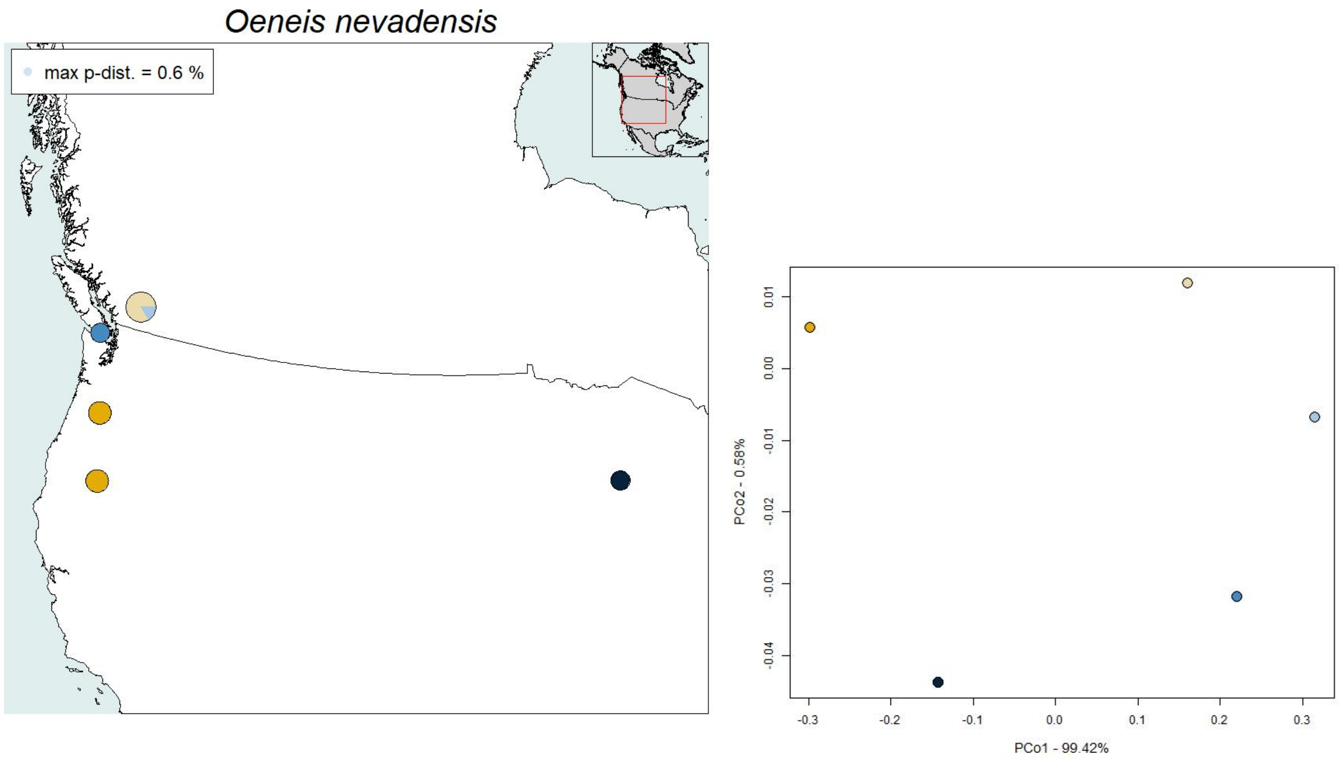

Figure 795 Map of *Oeneis nevadensis* showing the localities of the sequenced specimens (left). Nearby localities are grouped in pies. Colours match the bidimensional colour space of the PCoA projection (right) of max p-dists among sequences (dots). Sequences= 12; Hap obs.= 5; Hap asympt.= 7.8; Hap % obs.= 64.5%; GST= NaN; DST= NaN; HD= 0.758; ND= 0.0026; max p-dist= 0.6%.

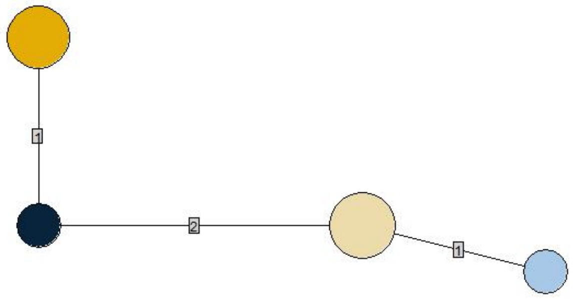

Figure 796: Haplotype network of *Oeneis nevadensis*. Sequences > 599 bp= 11.

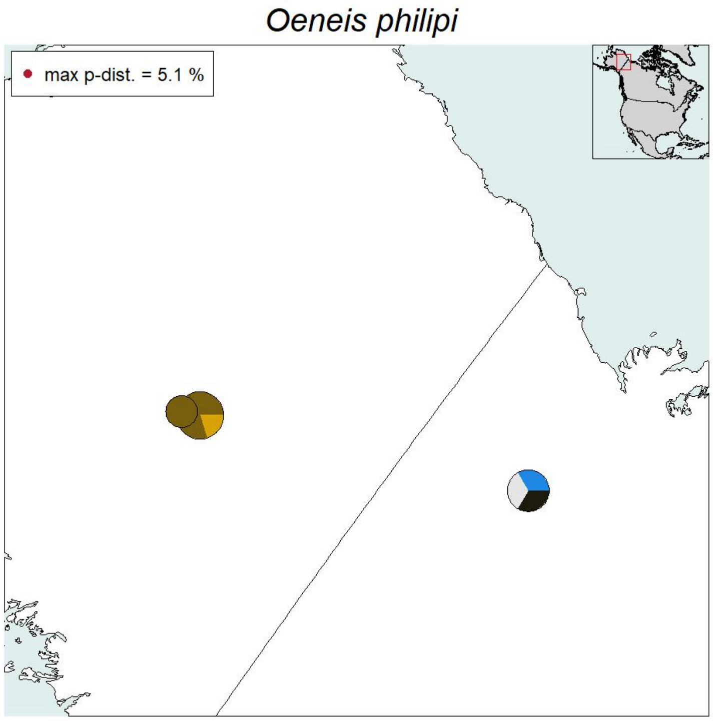

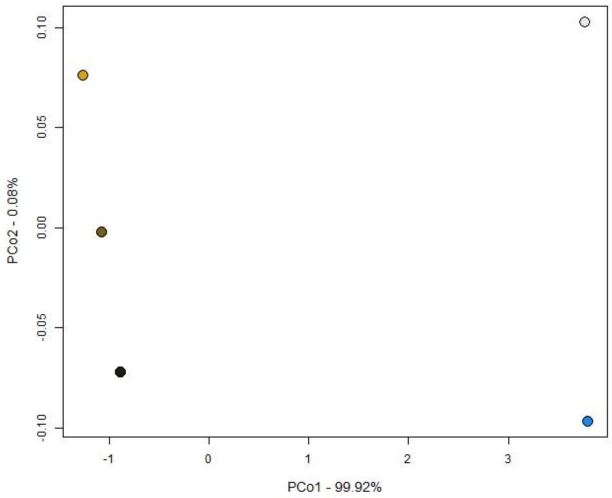

Figure 797 Map of *Oeneis philipi* showing the localities of the sequenced specimens (left). Nearby localities are grouped in pies. Colours match the bidimensional colour space of the PCoA projection (right) of max p-dists among sequences (dots). Sequences= 9; Hap obs.= 4; Hap asympt.= NA; Hap % obs.= NA%; GST= NaN; DST= NaN; HD= NA; ND= NA; max p-dist= 5.1%.

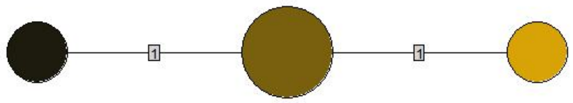

Figure 798: Haplotype network of *Oeneis philipi*. Sequences > 599 bp= 7.

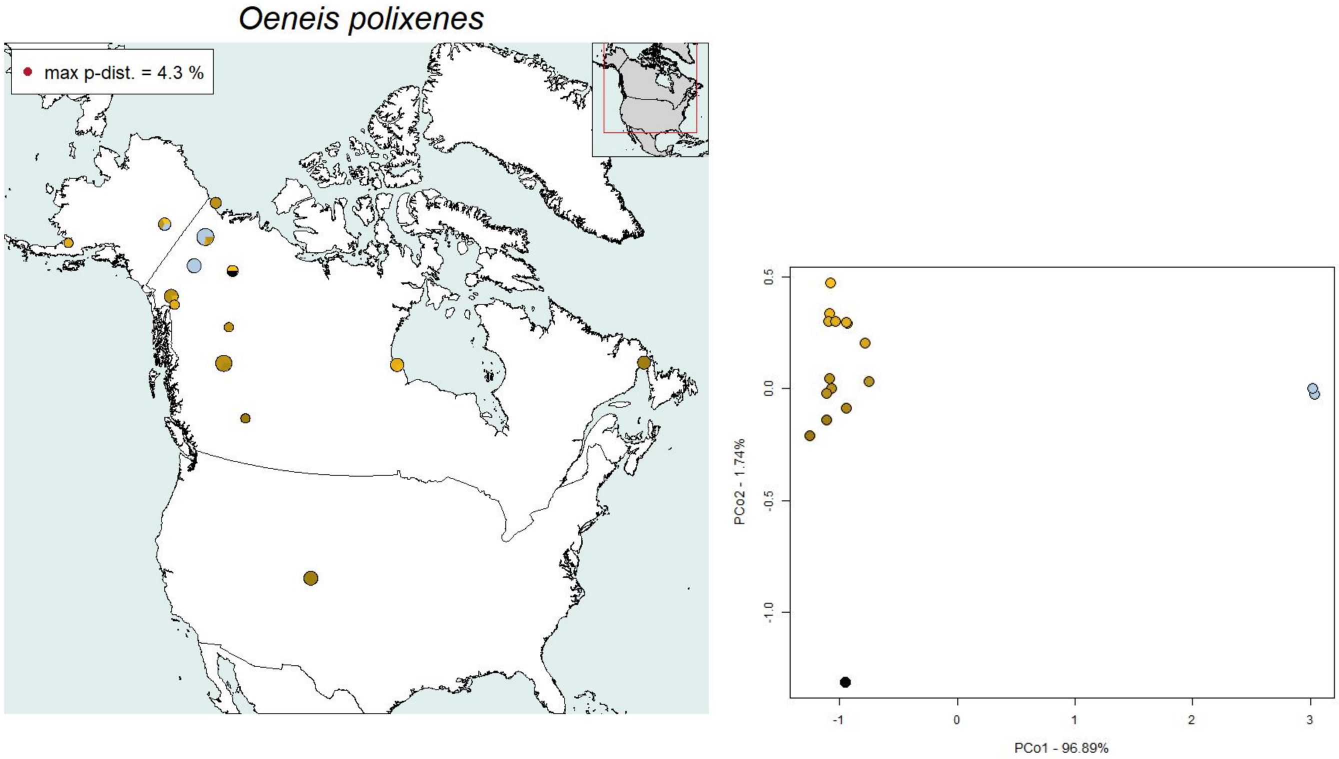

Figure 799 Map of *Oeneis polixenes* showing the localities of the sequenced specimens (left). Nearby localities are grouped in pies. Colours match the bidimensional colour space of the PCoA projection (right) of max p-dists among sequences (dots). Sequences= 54; Hap obs.= 14; Hap asympt.= 45.4; Hap % obs.= 30.8%; GST= 0.516; DST= 0.0098; HD= 0.847; ND= 0.018; max p-dist= 4.3%.

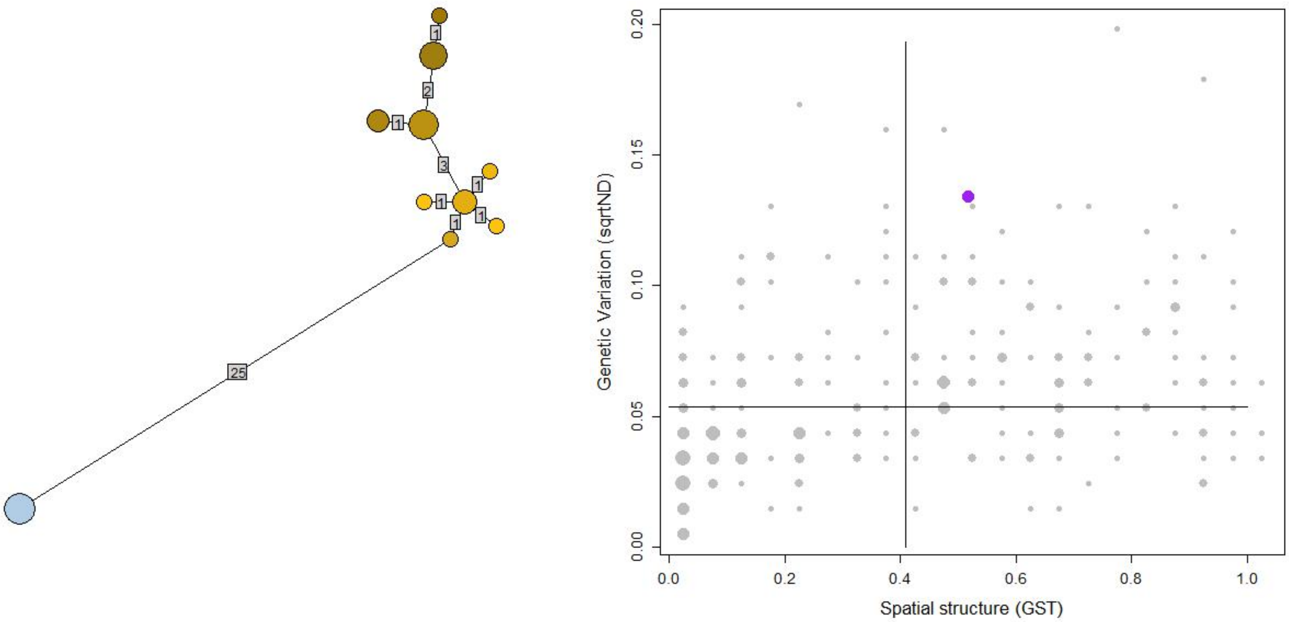

Figure 800: Haplotype network (left) of *Oeneis polixenes* sequences > 599 bp with colours matching the PCoA colour space (above). The bubble plot for mt-DNA polymorphism (square root transformed nucleotide diversity) and spatial structure (GST) among all species in the atlas and values for *Oeneis polixenes* (purple dot). The horizontal and vertical lines represent median values of nucleotide diversity and GST, respectively. Sequences > 599 bp= 51.

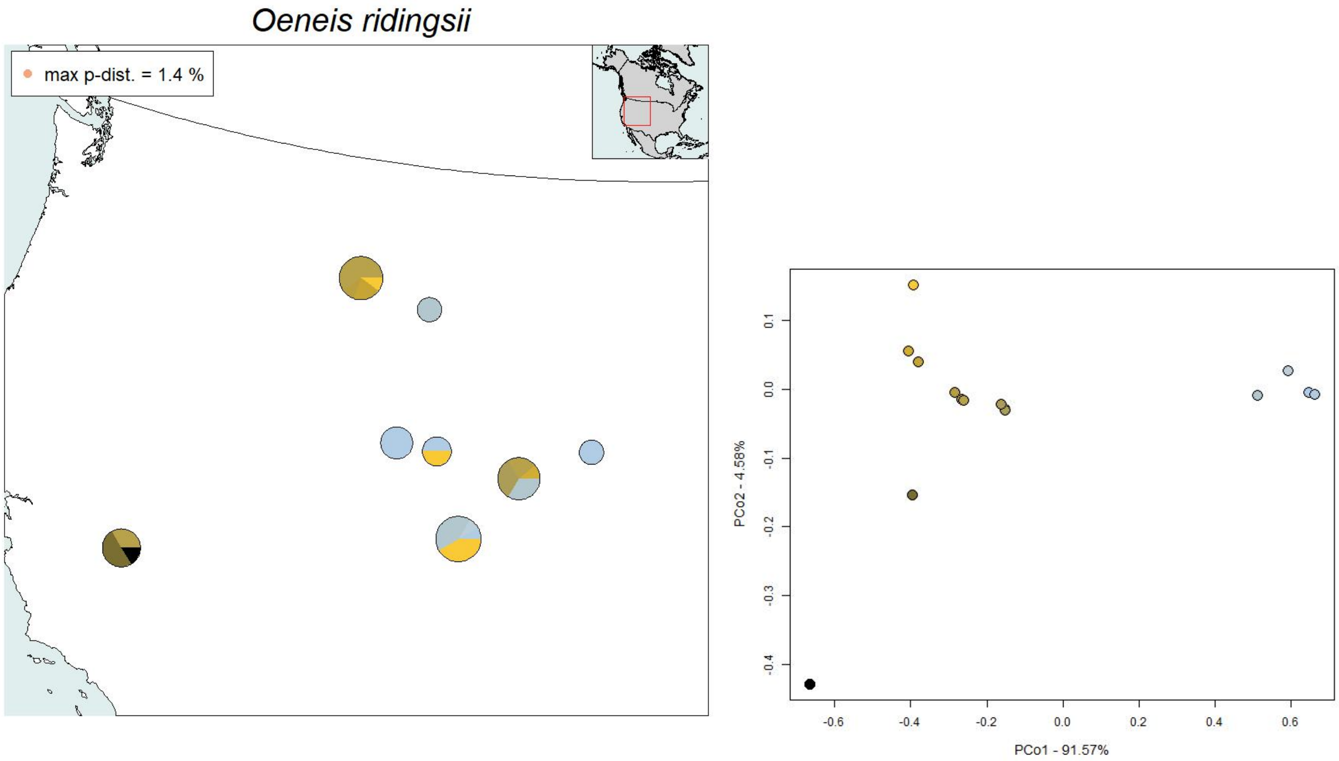

Figure 801 Map of *Oeneis ridingsii* showing the localities of the sequenced specimens (left). Nearby localities are grouped in pies. Colours match the bidimensional colour space of the PCoA projection (right) of max p-dists among sequences (dots). Sequences= 44; Hap obs.= 10; Hap asympt.= 19.8; Hap % obs.= 50.6%; GST= 0.563; DST= 0.0027; HD= 0.82; ND= 0.005; max p-dist= 1.4%.

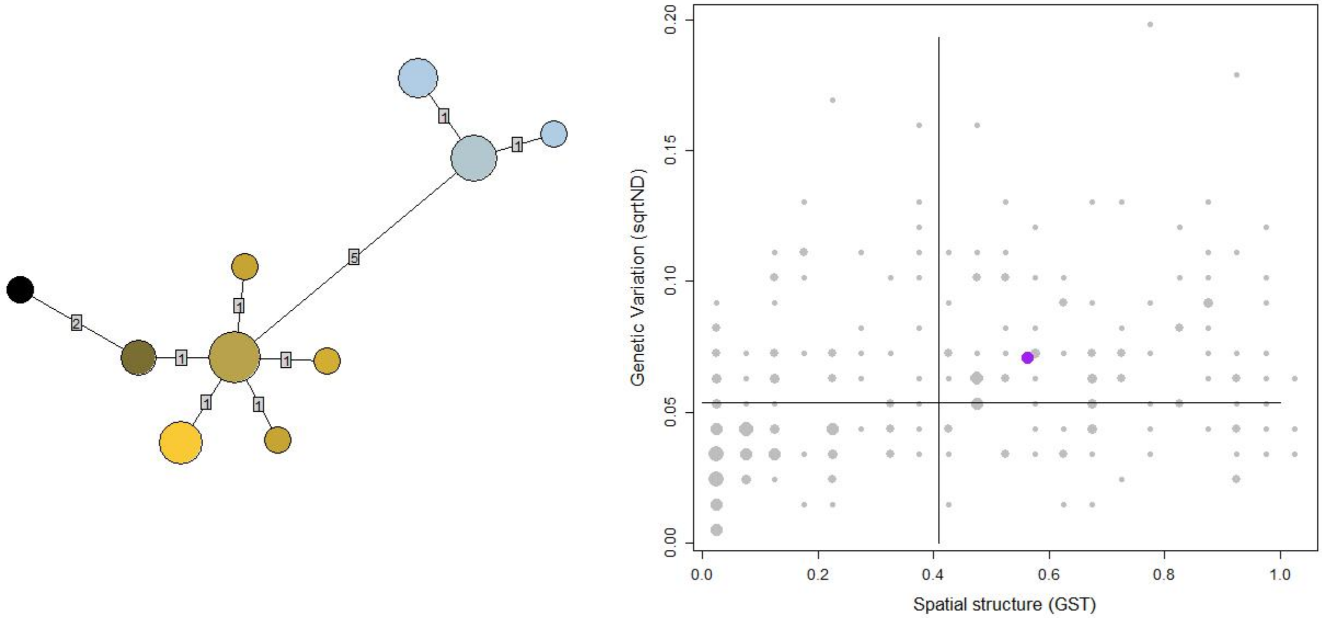

Figure 802: Haplotype network (left) of *Oeneis ridingsii* sequences > 599 bp with colours matching the PCoA colour space (above). The bubble plot for mt-DNA polymorphism (square root transformed nucleotide diversity) and spatial structure (GST) among all species in the atlas and values for *Oeneis ridingsii* (purple dot). The horizontal and vertical lines represent median values of nucleotide diversity and GST, respectively. Sequences > 599 bp= 43.

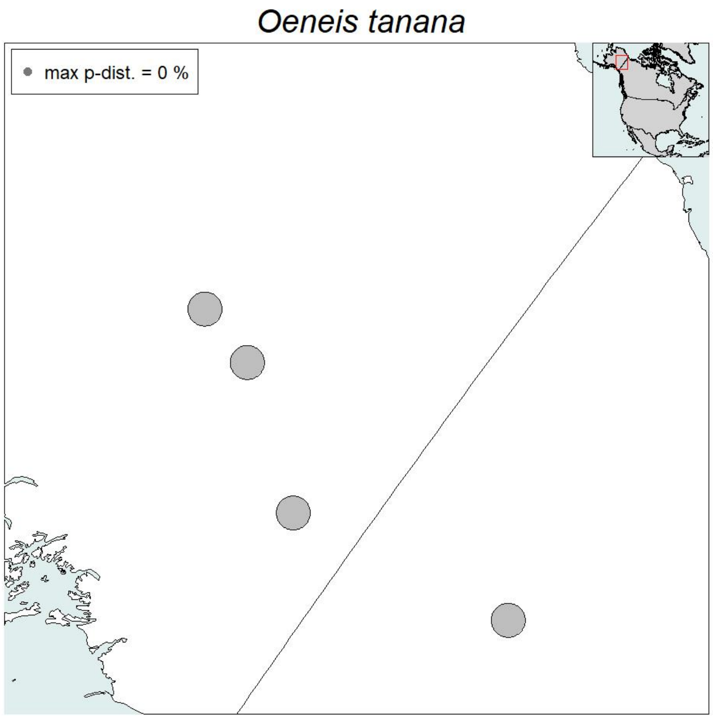

Figure 803: Map of *Oeneis tanana* showing the localities of the sequenced specimens. Nearby localities are grouped in pies. Due to the presence of a single haplotype PCoA projection was not done and a single grey colour was plotted on the map. Sequences= 4; Hap obs.= 1; Hap asympt.= NA; Hap % obs.= NA%; GST= NaN; DST= NaN; HD= NA; ND= NA; max p-dist= 0%.

Haplotype network analysis and bubble plot of *Oeneis tanana* were not possible. Sequences > 599 bp = 4.

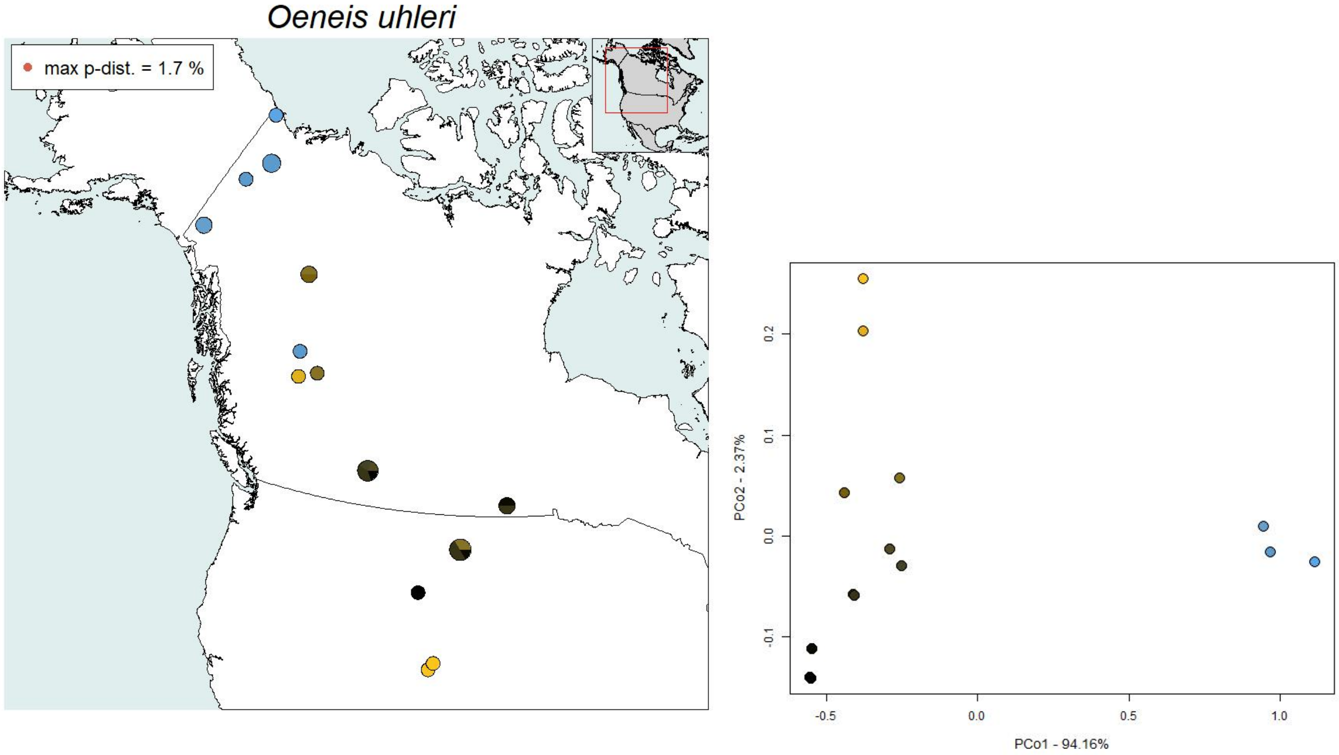

Figure 804 Map of *Oeneis uhleri* showing the localities of the sequenced specimens (left). Nearby localities are grouped in pies. Colours match the bidimensional colour space of the PCoA projection (right) of max p-dists among sequences (dots). Sequences= 28; Hap obs.= 13; Hap asympt.= 20.9; Hap % obs.= 62.3%; GST= 0.832; DST= 0.0049; HD= 0.91; ND= 0.0069; max p-dist= 1.7%.

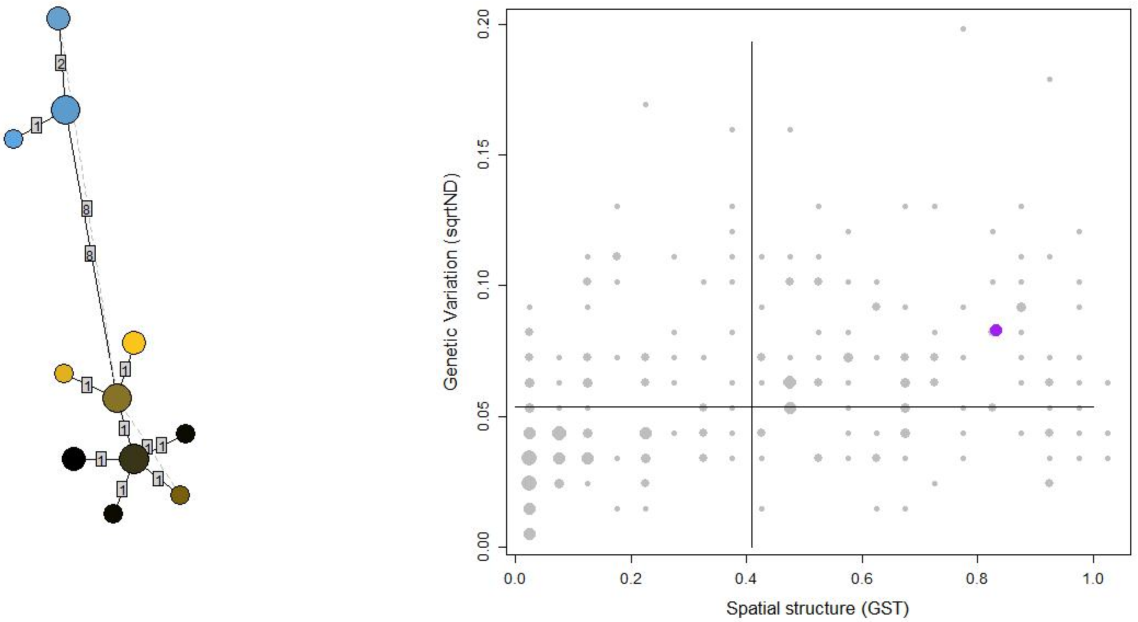

Figure 805: Haplotype network (left) of *Oeneis uhleri* sequences > 599 bp with colours matching the PCoA colour space (above). The bubble plot for mt-DNA polymorphism (square root transformed nucleotide diversity) and spatial structure (GST) among all species in the atlas and values for *Oeneis uhleri* (purple dot). The horizontal and vertical lines represent median values of nucleotide diversity and GST, respectively. Sequences > 599 bp= 27.

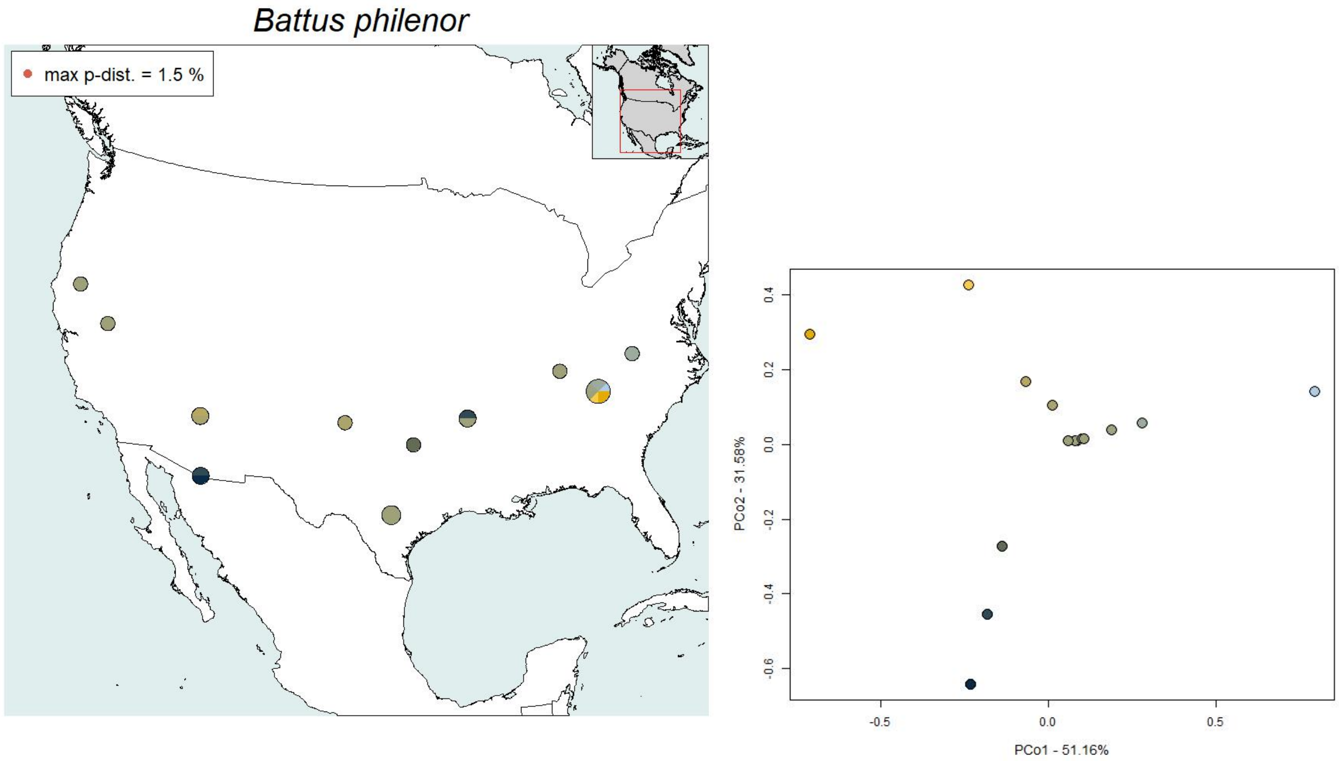

Figure 806 Map of *Battus philenor* showing the localities of the sequenced specimens (left). Nearby localities are grouped in pies. Colours match the bidimensional colour space of the PCoA projection (right) of max p-dists among sequences (dots). Sequences= 23; Hap obs.= 12; Hap asympt.= 19.8; Hap % obs.= 60.6%; GST= 0.011; DST= 0.0001; HD= 0.893; ND= 0.0048; max p-dist= 1.5%.

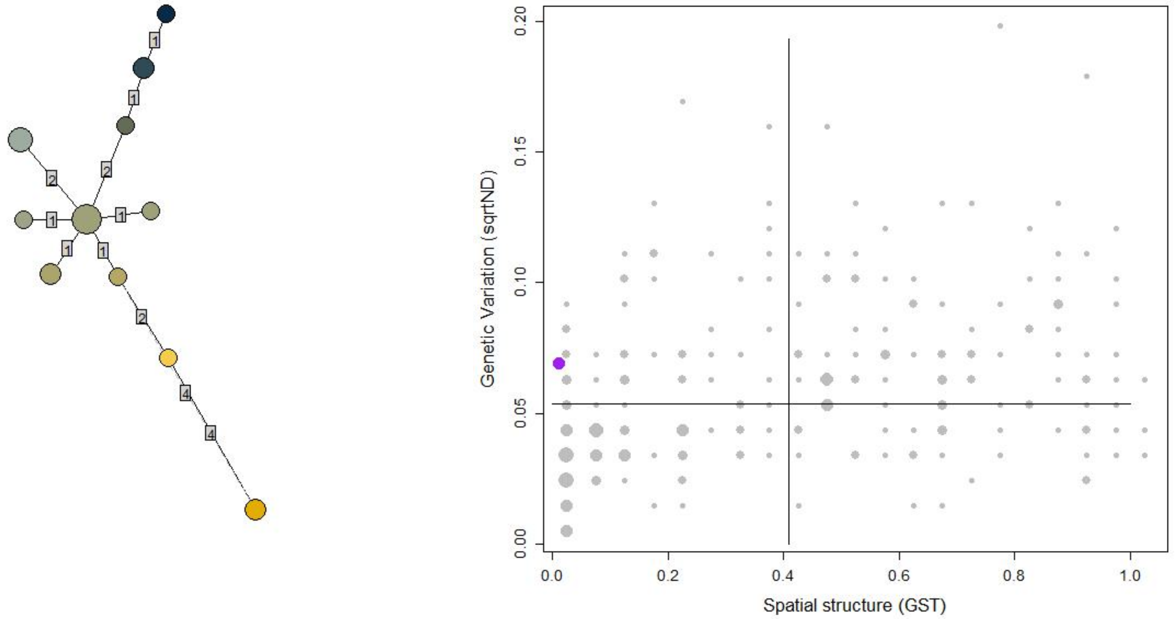

Figure 807: Haplotype network (left) of *Battus philenor* sequences > 599 bp with colours matching the PCoA colour space (above). The bubble plot for mt-DNA polymorphism (square root transformed nucleotide diversity) and spatial structure (GST) among all species in the atlas and values for *Battus philenor* (purple dot). The horizontal and vertical lines represent median values of nucleotide diversity and GST, respectively. Sequences > 599 bp= 22.

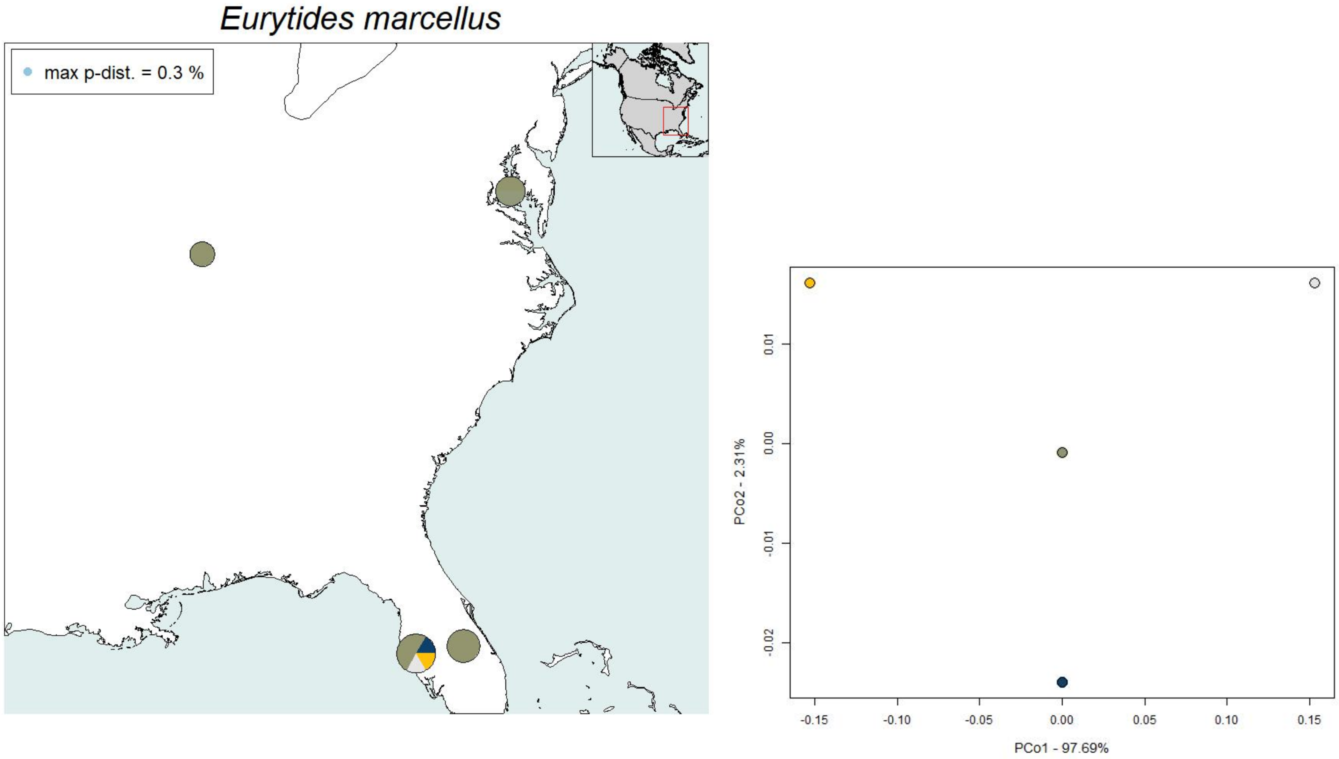

Figure 808 Map of *Eurytides marcellus* showing the localities of the sequenced specimens (left). Nearby localities are grouped in pies. Colours match the bidimensional colour space of the PCoA projection (right) of max p-dists among sequences (dots). Sequences= 12; Hap obs.= 3; Hap asympt.= 3.9; Hap % obs.= 76.6%; GST= NaN; DST= NaN; HD= 0.318; ND= 0.0005; max p-dist= 0.3%.

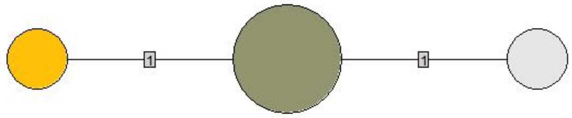

Figure 809: Haplotype network of *Eurytides marcellus*. Sequences > 599 bp= 12.

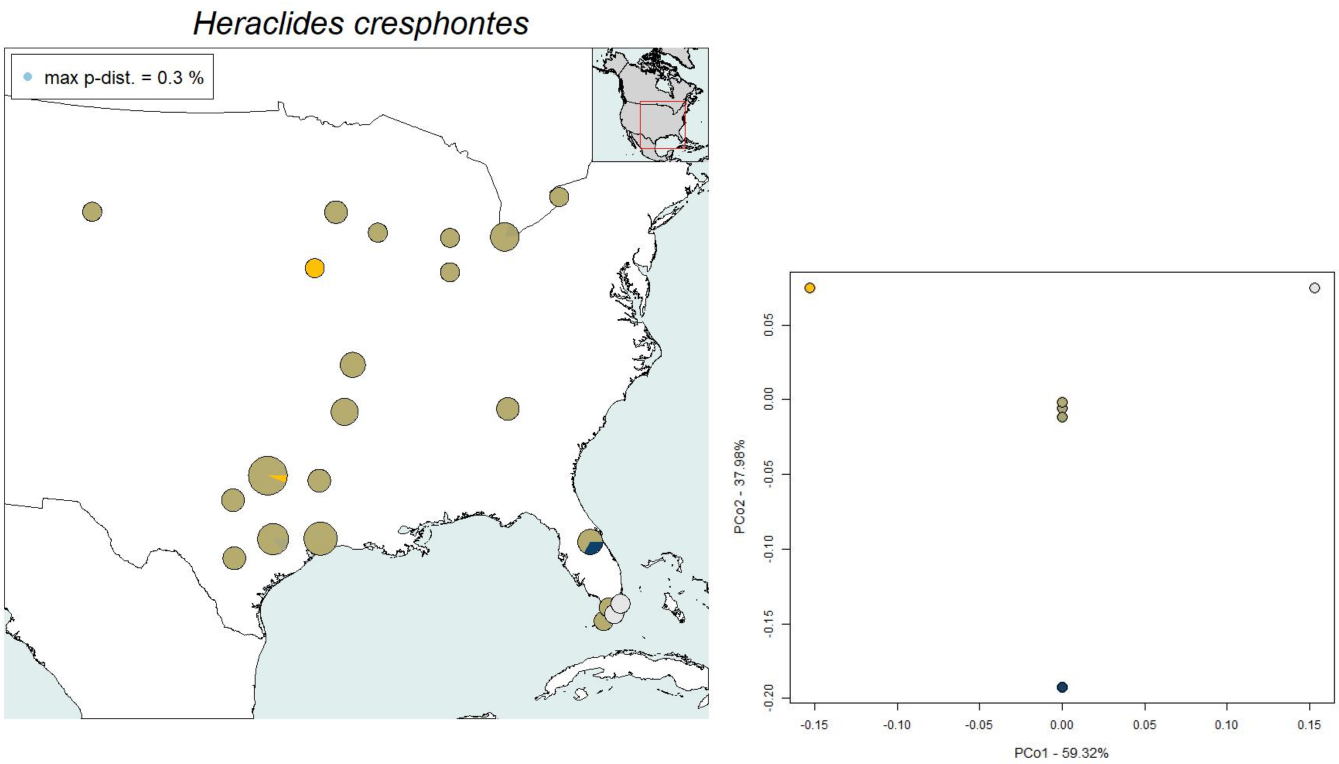

Figure 810 Map of *Heraclides cresphontes* showing the localities of the sequenced specimens (left). Nearby localities are grouped in pies. Colours match the bidimensional colour space of the PCoA projection (right) of max p-dists among sequences (dots). Sequences= 68; Hap obs.= 4; Hap asympt.= 4.2; Hap % obs.= 94.2%; GST= 0.168; DST= 0; HD= 0.142; ND= 0.0002; max p-dist= 0.3%.

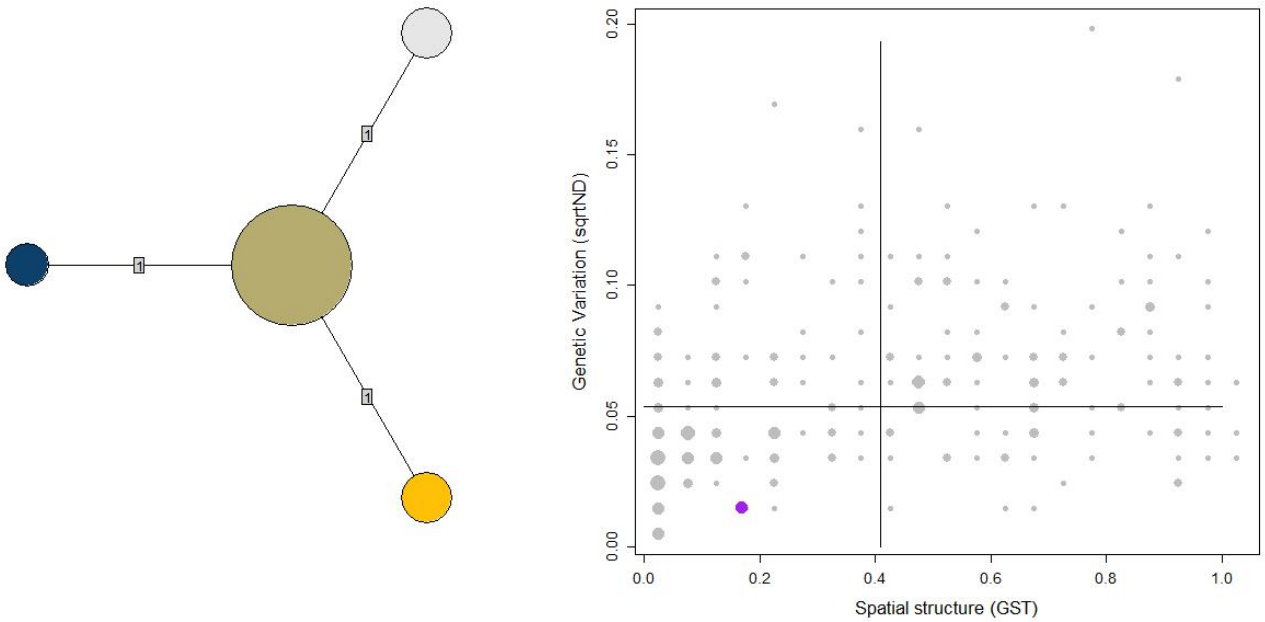

Figure 811: Haplotype network (left) of *Heraclides cresphontes* sequences > 599 bp with colours matching the PCoA colour space (above). The bubble plot for mt-DNA polymorphism (square root transformed nucleotide diversity) and spatial structure (GST) among all species in the atlas and values for *Heraclides cresphontes* (purple dot). The horizontal and vertical lines represent median values of nucleotide diversity and GST, respectively. Sequences > 599 bp= 66.

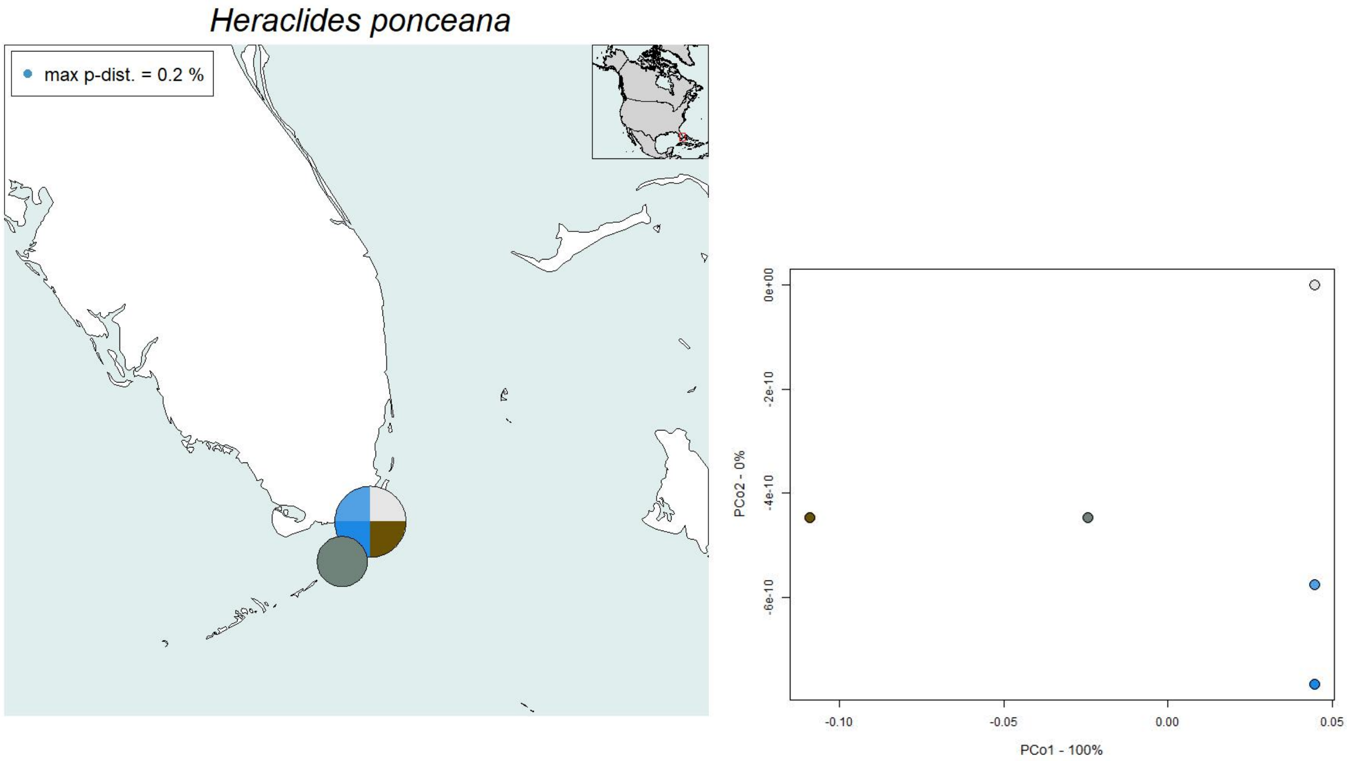

Figure 812 Map of *Heraclides ponceana* showing the localities of the sequenced specimens (left). Nearby localities are grouped in pies. Colours match the bidimensional colour space of the PCoA projection (right) of max p-dists among sequences (dots). Sequences= 5; Hap obs.= 3; Hap asympt.= NA; Hap % obs.= NA%; GST= NaN; DST= NaN; HD= NA; ND= NA; max p-dist= 0.2%.

Haplotype network analysis and bubble plot of *Heraclides ponceana* were not possible. Sequences > 599 bp = 4.

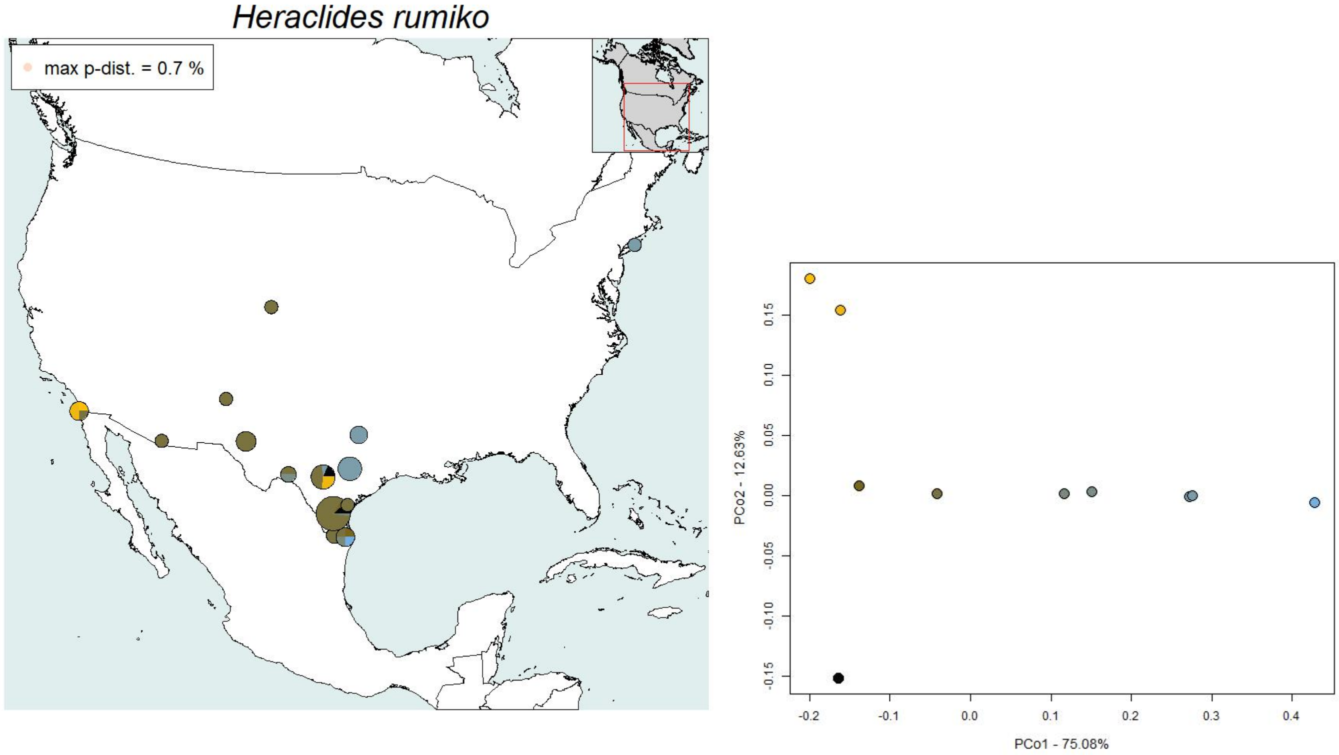

Figure 813 Map of *Heraclides rumiko* showing the localities of the sequenced specimens (left). Nearby localities are grouped in pies. Colours match the bidimensional colour space of the PCoA projection (right) of max p-dists among sequences (dots). Sequences= 90; Hap obs.= 10; Hap asympt.= 22.4; Hap % obs.= 44.7%; GST= 0.556; DST= 0.0008; HD= 0.594; ND= 0.0016; max p-dist= 0.7%.

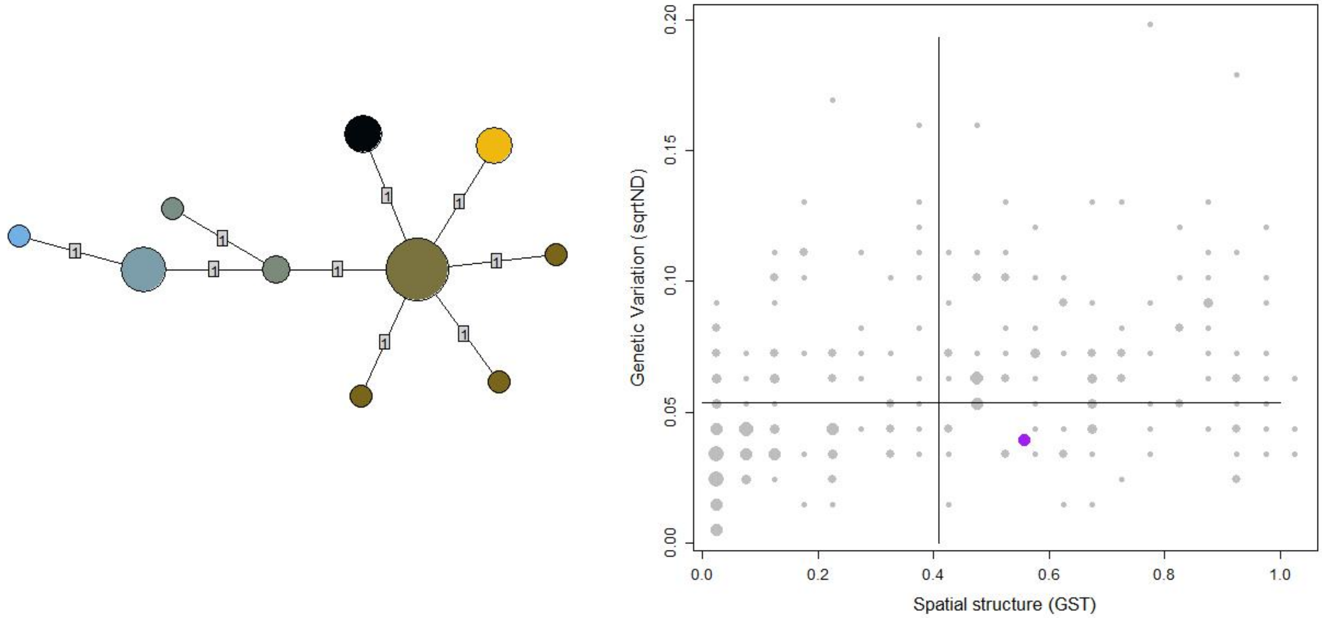

Figure 814: Haplotype network (left) of *Heraclides rumiko* sequences > 599 bp with colours matching the PCoA colour space (above). The bubble plot for mt-DNA polymorphism (square root transformed nucleotide diversity) and spatial structure (GST) among all species in the atlas and values for *Heraclides rumiko* (purple dot). The horizontal and vertical lines represent median values of nucleotide diversity and GST, respectively. Sequences > 599 bp= 90.

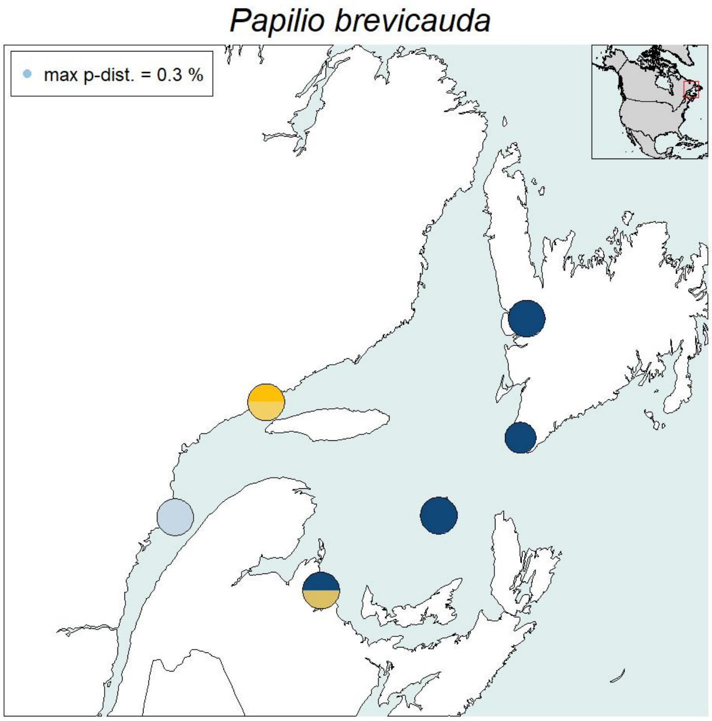

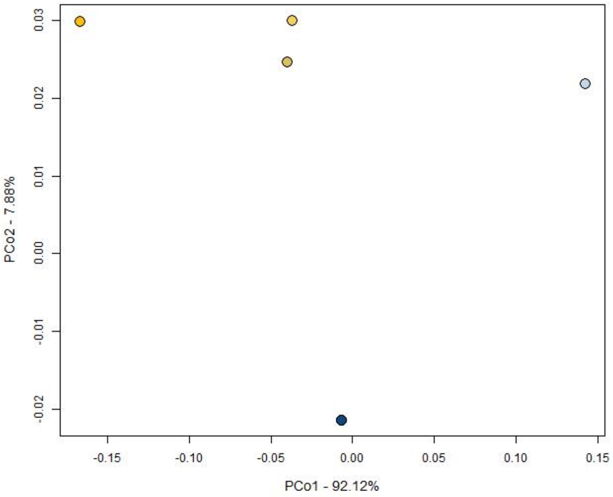

Figure 815 Map of *Papilio brevicauda* showing the localities of the sequenced specimens (left). Nearby localities are grouped in pies. Colours match the bidimensional colour space of the PCoA projection (right) of max p-dists among sequences (dots). Sequences= 11; Hap obs.= 4; Hap asympt.= 4.2; Hap % obs.= 94.6%; GST= NaN; DST= NaN; HD= 0.691; ND= 0.0007; max p-dist= 0.3%.

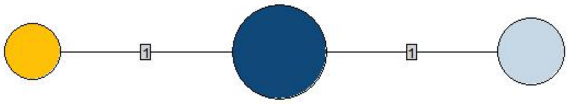

Figure 816: Haplotype network of *Papilio brevicauda*. Sequences > 599 bp= 11.

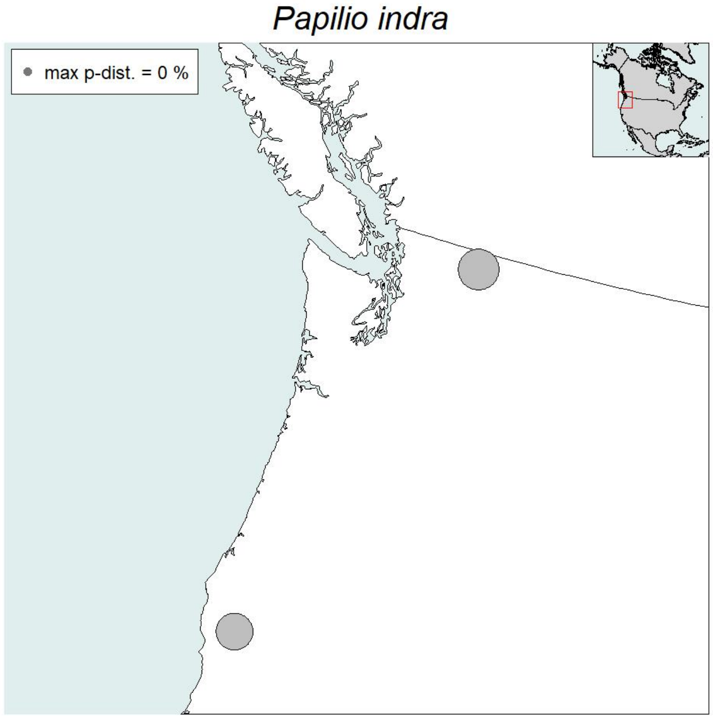

Figure 817: Map of *Papilio indra* showing the localities of the sequenced specimens. Nearby localities are grouped in pies. Due to the presence of a single haplotype PCoA projection was not done and a single grey colour was plotted on the map. Sequences= 5; Hap obs.= 1; Hap asympt.= NA; Hap % obs.= NA%; GST= NaN; DST= NaN; HD= NA; ND= NA; max p-dist= 0%.

Haplotype network analysis and bubble plot of *Papilio indra* were not possible. Sequences > 599 bp = 5.

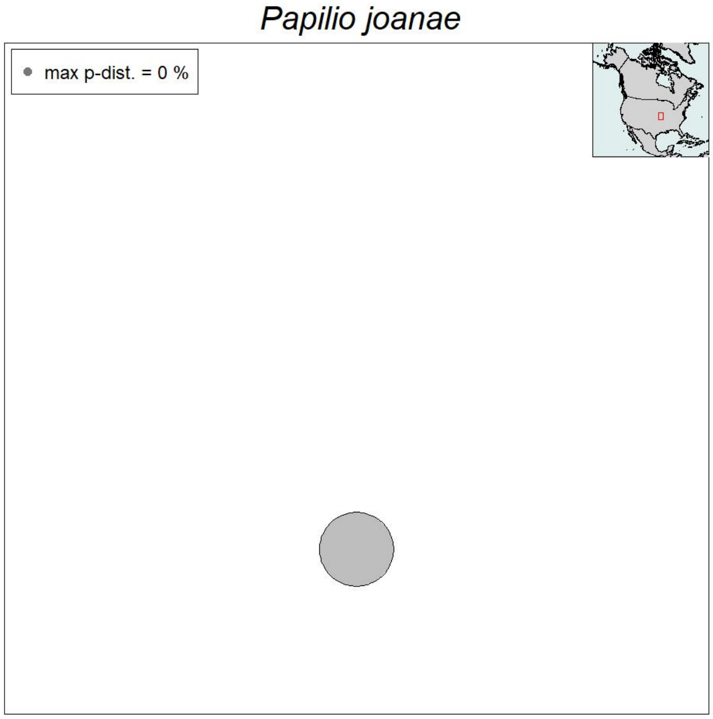

Figure 818: Map of *Papilio joanae* showing the localities of the sequenced specimens. Nearby localities are grouped in pies. Due to the presence of a single haplotype PCoA projection was not done and a single grey colour was plotted on the map. Sequences= 4; Hap obs.= 1; Hap asympt.= NA; Hap % obs.= NA%; GST= NaN; DST= NaN; HD= NA; ND= NA; max p-dist= 0%.

Haplotype network analysis and bubble plot of *Papilio joanae* were not possible. Sequences > 599 bp = 4.

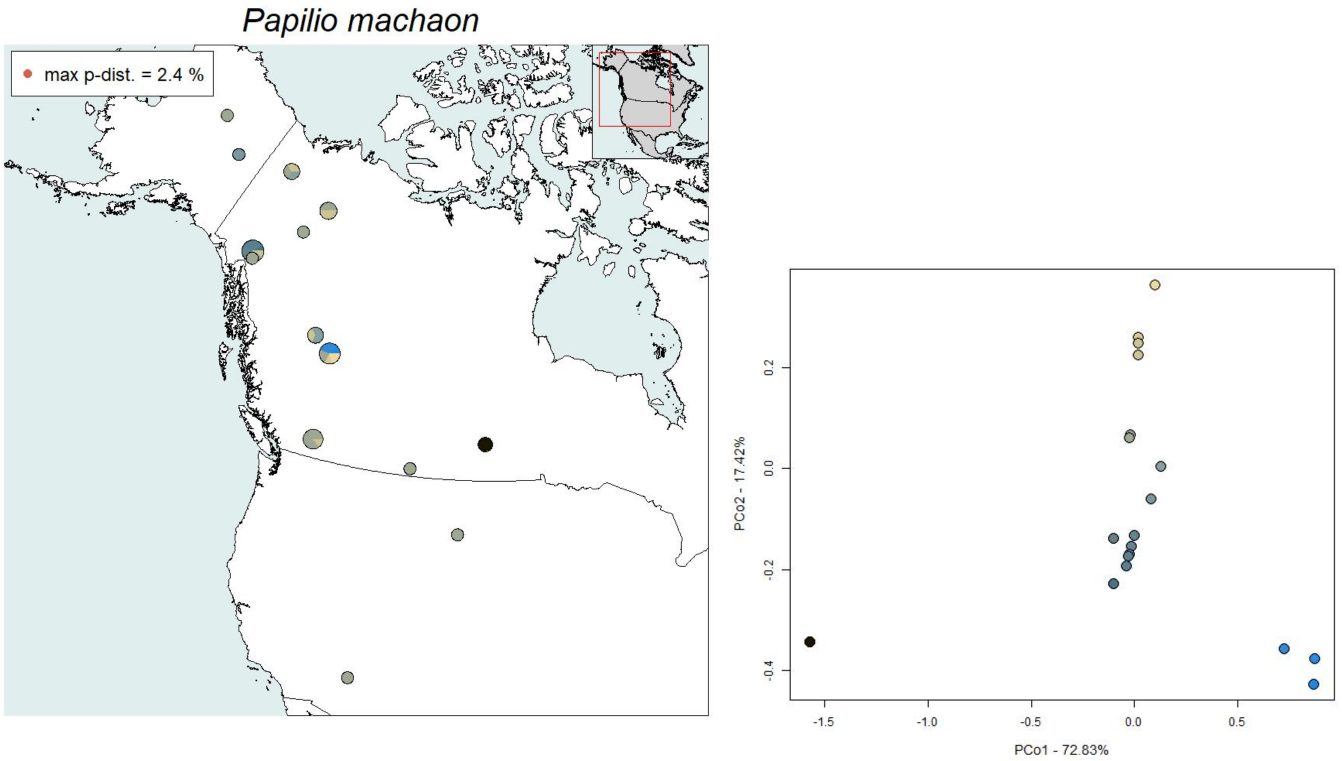

Figure 819 Map of *Papilio machaon* showing the localities of the sequenced specimens (left). Nearby localities are grouped in pies. Colours match the bidimensional colour space of the PCoA projection (right) of max p-dists among sequences (dots). Sequences= 46; Hap obs.= 10; Hap asympt.= 12.6; Hap % obs.= 79.3%; GST= 0.101; DST= 0.0003; HD= 0.641; ND= 0.0034; max p-dist= 2.4%.

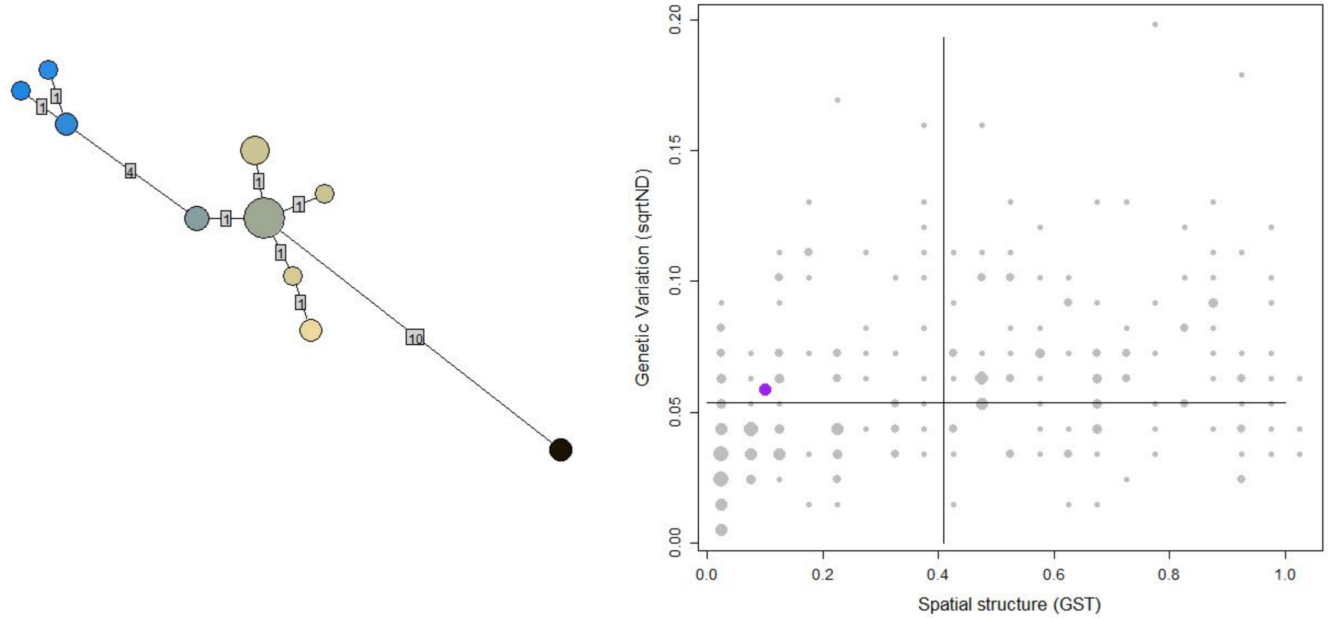

Figure 820: Haplotype network (left) of *Papilio machaon* sequences > 599 bp with colours matching the PCoA colour space (above). The bubble plot for mt-DNA polymorphism (square root transformed nucleotide diversity) and spatial structure (GST) among all species in the atlas and values for *Papilio machaon* (purple dot). The horizontal and vertical lines represent median values of nucleotide diversity and GST, respectively. Sequences > 599 bp= 38.

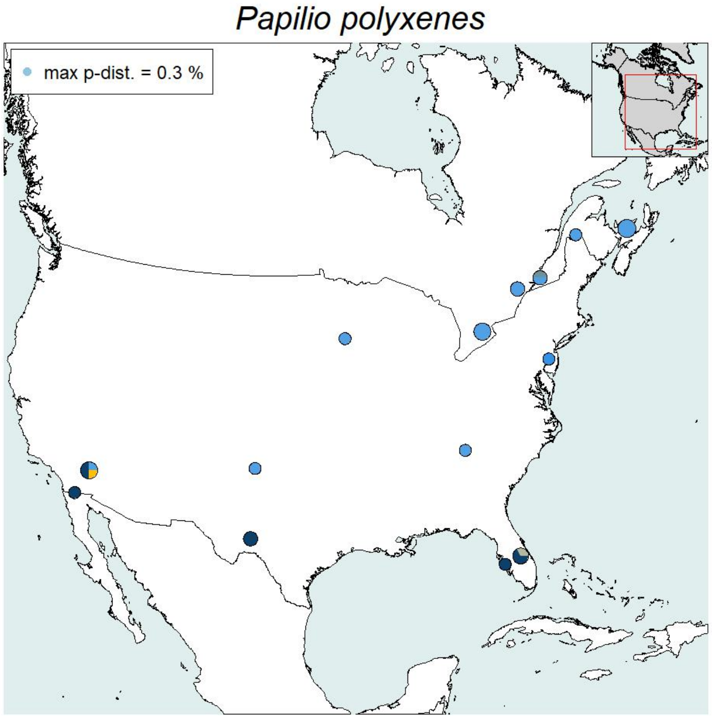

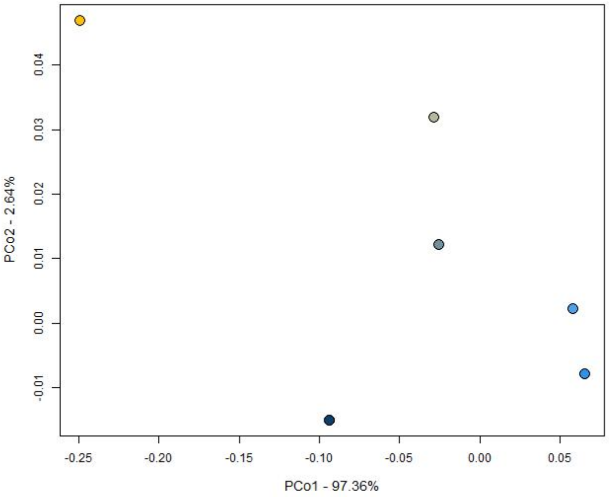

Figure 821 Map of *Papilio polyxenes* showing the localities of the sequenced specimens (left). Nearby localities are grouped in pies. Colours match the bidimensional colour space of the PCoA projection (right) of max p-dists among sequences (dots). Sequences= 29; Hap obs.= 4; Hap asympt.= 4.5; Hap % obs.= 89.2%; GST= 0.732; DST= 0.0006; HD= 0.552; ND= 0.0007; max p-dist= 0.3%.

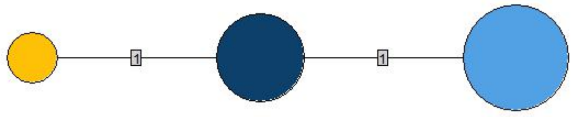

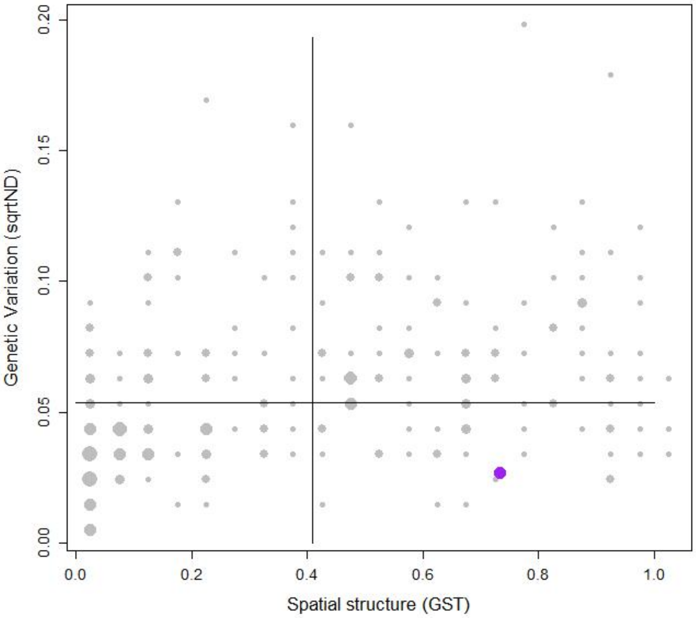

Figure 822: Haplotype network (left) of *Papilio polyxenes* sequences > 599 bp with colours matching the PCoA colour space (above). The bubble plot for mt-DNA polymorphism (square root transformed nucleotide diversity) and spatial structure (GST) among all species in the atlas and values for *Papilio polyxenes* (purple dot). The horizontal and vertical lines represent median values of nucleotide diversity and GST, respectively. Sequences > 599 bp= 28.

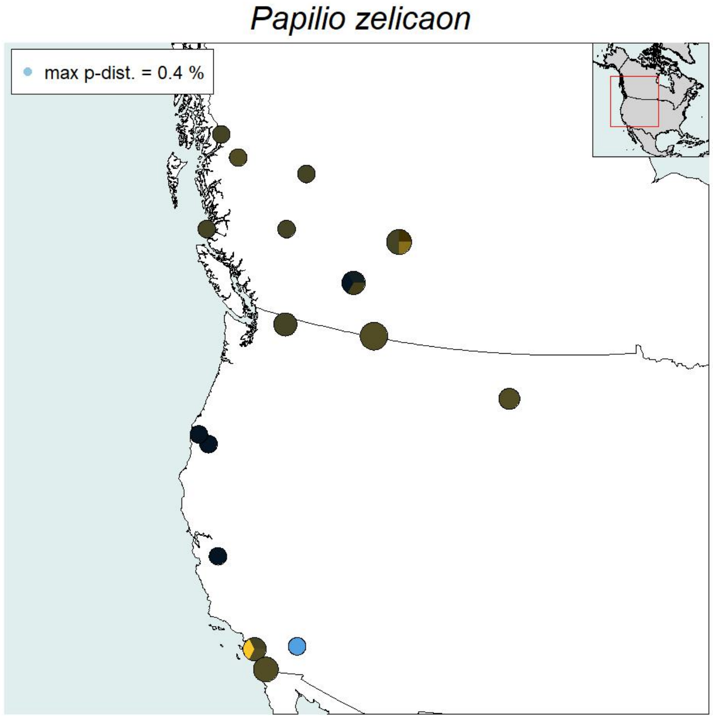

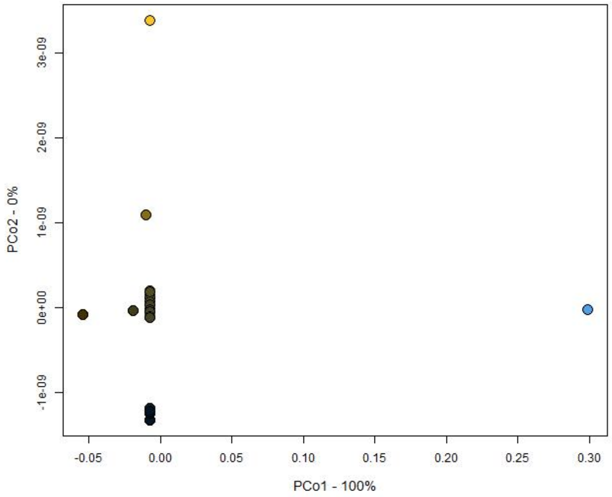

Figure 823 Map of *Papilio zelicaon* showing the localities of the sequenced specimens (left). Nearby localities are grouped in pies. Colours match the bidimensional colour space of the PCoA projection (right) of max p-dists among sequences (dots). Sequences= 34; Hap obs.= 2; Hap asympt.= 2; Hap % obs.= 100%; GST= 0; DST= 0; HD= 0.059; ND= 0.0002; max p-dist= 0.4%.

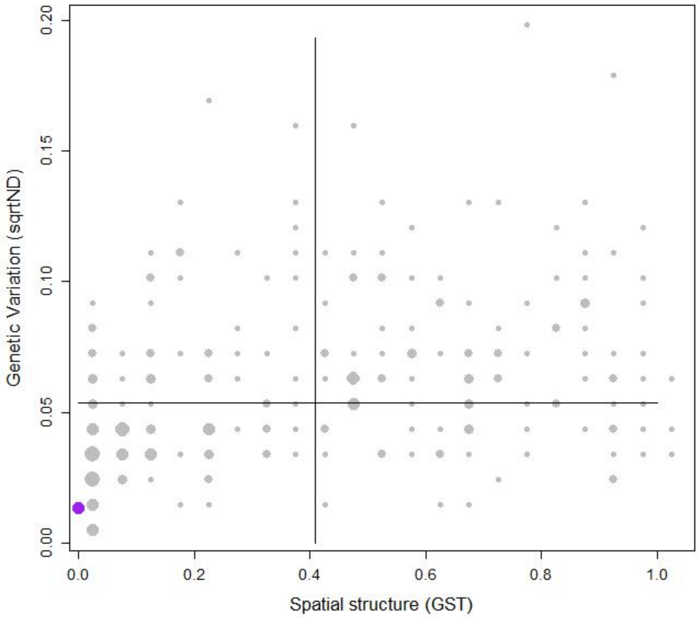

Figure 824: The bubble plot for mt-DNA polymorphism (square root transformed nucleotide diversity) and spatial structuree (GST) among all species in the atlas and values for *Papilio zelicaon* (purple dot). The horizontal and vertical lines represent median values of nucleotide diversity and GST, respectively. Haplotype network analysis was not possible. Sequences > 599 bp = 33.

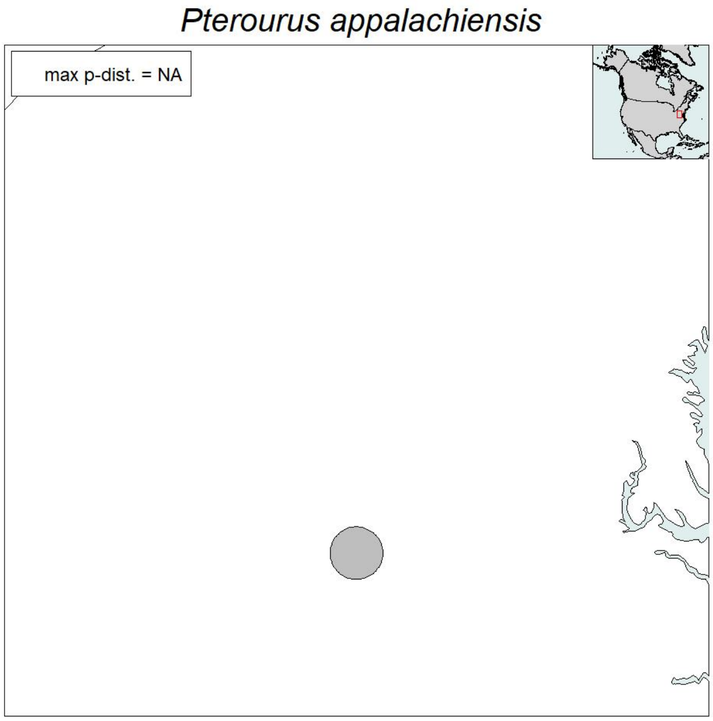

Figure 825: Map of *Pterourus appalachiensis* showing the localities of the sequenced specimens. Nearby localities are grouped in pies. Due to the presence of a single haplotype PCoA projection was not done and a single grey colour was plotted on the map. Sequences= 1; Hap obs.= NA; Hap asympt.= NA; Hap % obs.= NA; GST= NaN; DST= NaN; HD= NA; ND= NA; max p-dist= NA.

Haplotype network analysis and bubble plot of *Pterourus appalachiensis* were not possible. Sequences > 599 bp = 1.

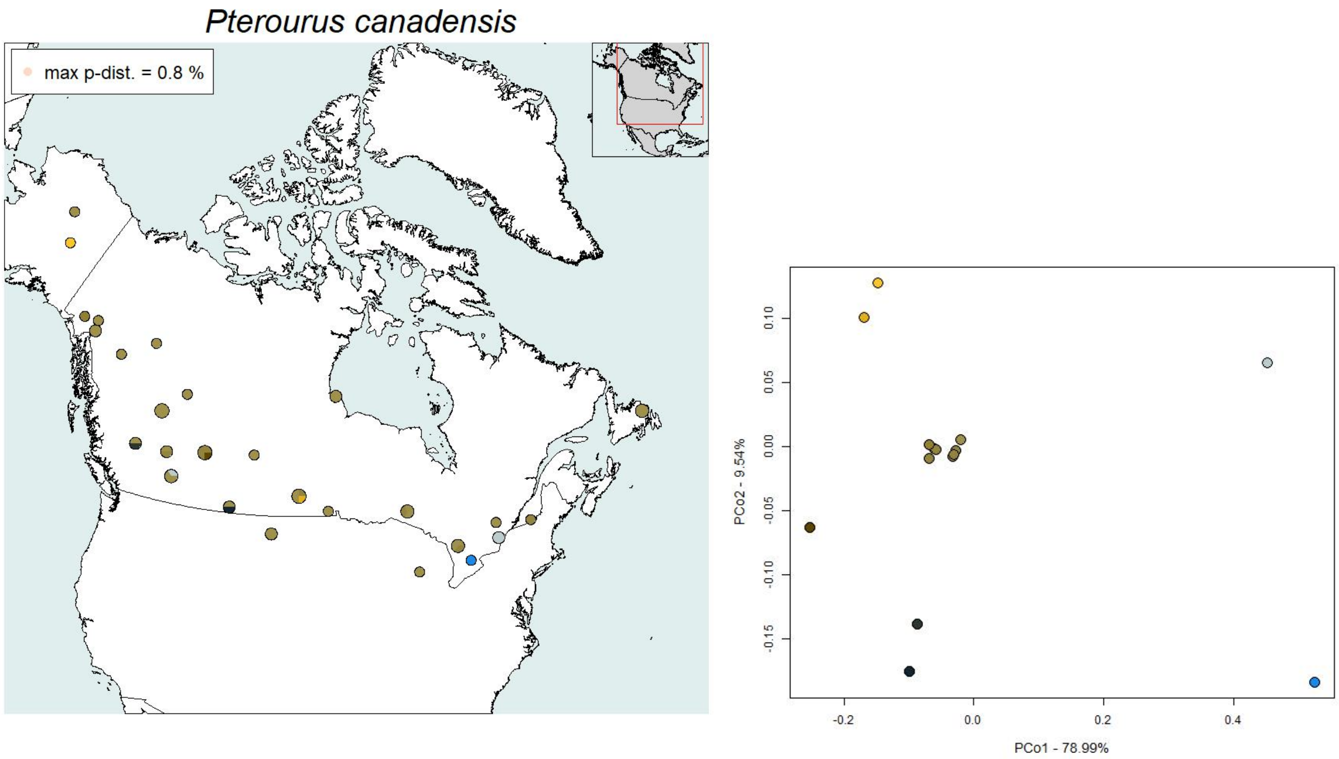

Figure 826 Map of *Pterourus canadensis* showing the localities of the sequenced specimens (left). Nearby localities are grouped in pies. Colours match the bidimensional colour space of the PCoA projection (right) of max p-dists among sequences (dots). Sequences= 51; Hap obs.= 7; Hap asympt.= 16.8; Hap % obs.= 41.7%; GST= 0.094; DST= 0.0001; HD= 0.32; ND= 0.001; max p-dist= 0.8%.

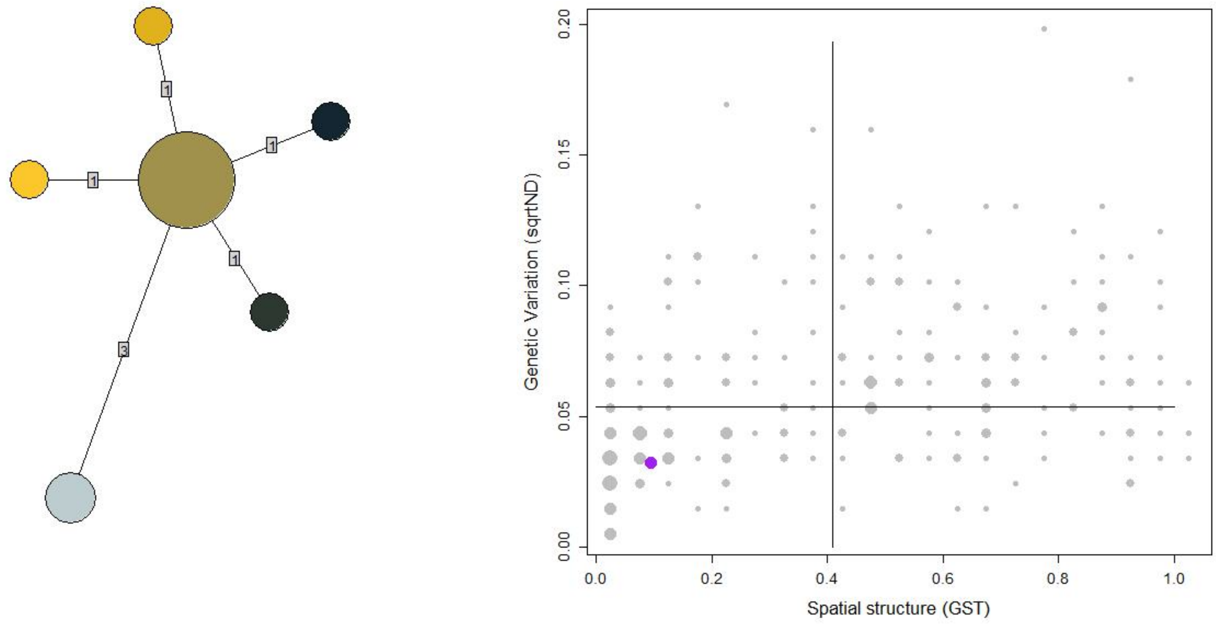

Figure 827: Haplotype network (left) of *Pterourus canadensis* sequences > 599 bp with colours matching the PCoA colour space (above). The bubble plot for mt-DNA polymorphism (square root transformed nucleotide diversity) and spatial structure (GST) among all species in the atlas and values for *Pterourus canadensis* (purple dot). The horizontal and vertical lines represent median values of nucleotide diversity and GST, respectively. Sequences > 599 bp= 44.

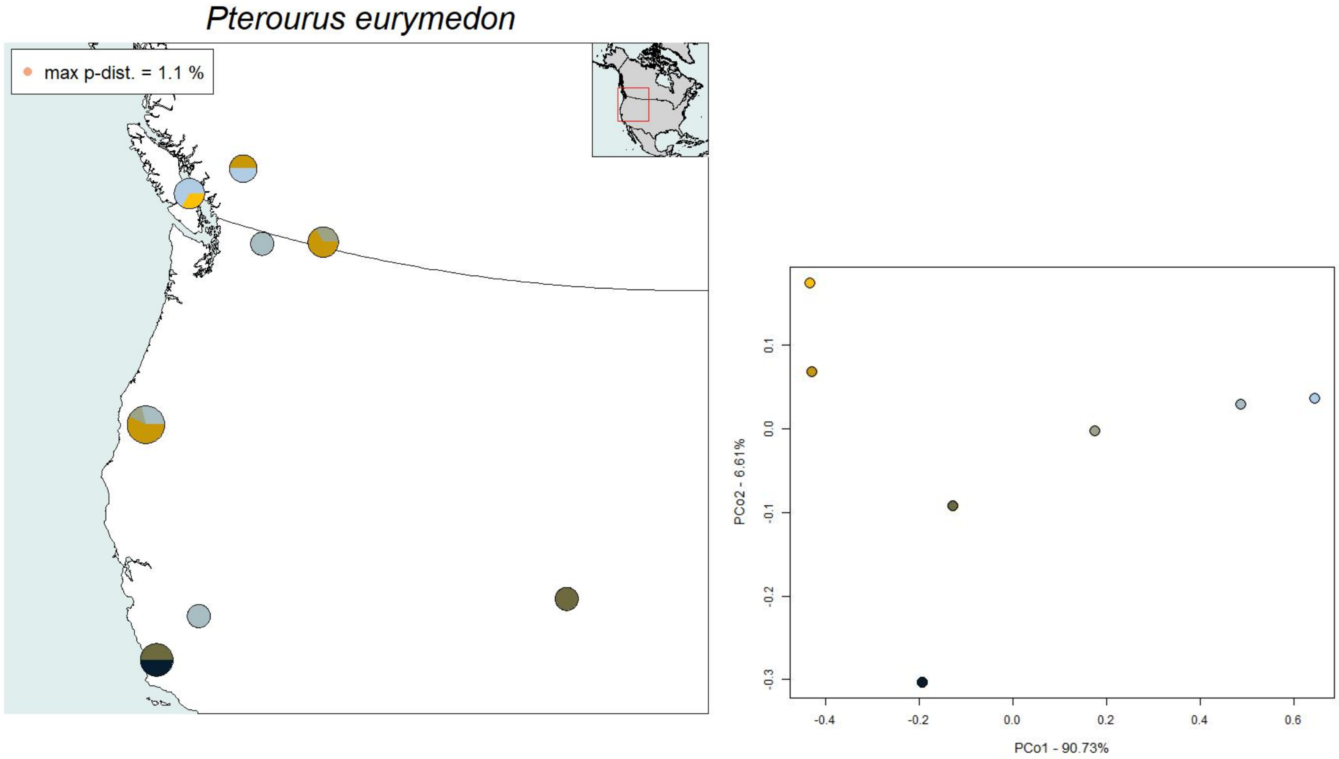

Figure 828 Map of *Pterourus eurymedon* showing the localities of the sequenced specimens (left). Nearby localities are grouped in pies. Colours match the bidimensional colour space of the PCoA projection (right) of max p-dists among sequences (dots). Sequences= 22; Hap obs.= 7; Hap asympt.= 8.9; Hap % obs.= 78.6%; GST= 0.091; DST= 0.0005; HD= 0.823; ND= 0.0051; max p-dist= 1.1%.

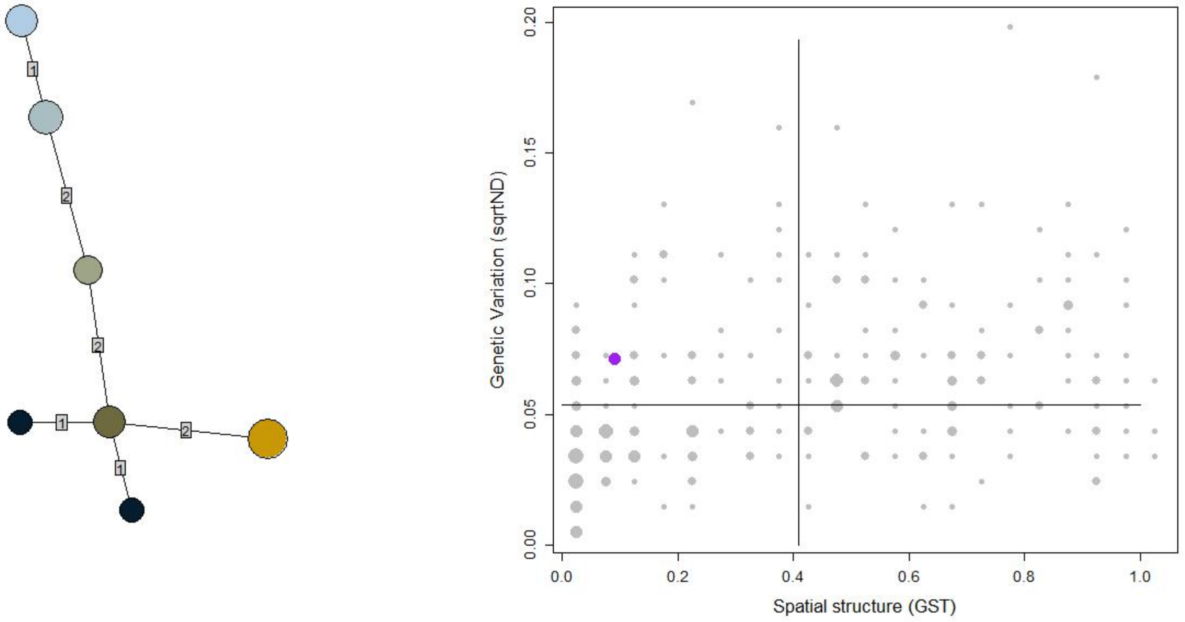

Figure 829: Haplotype network (left) of *Pterourus eurymedon* sequences > 599 bp with colours matching the PCoA colour space (above). The bubble plot for mt-DNA polymorphism (square root transformed nucleotide diversity) and spatial structure (GST) among all species in the atlas and values for *Pterourus eurymedon* (purple dot). The horizontal and vertical lines represent median values of nucleotide diversity and GST, respectively. Sequences > 599 bp= 21.

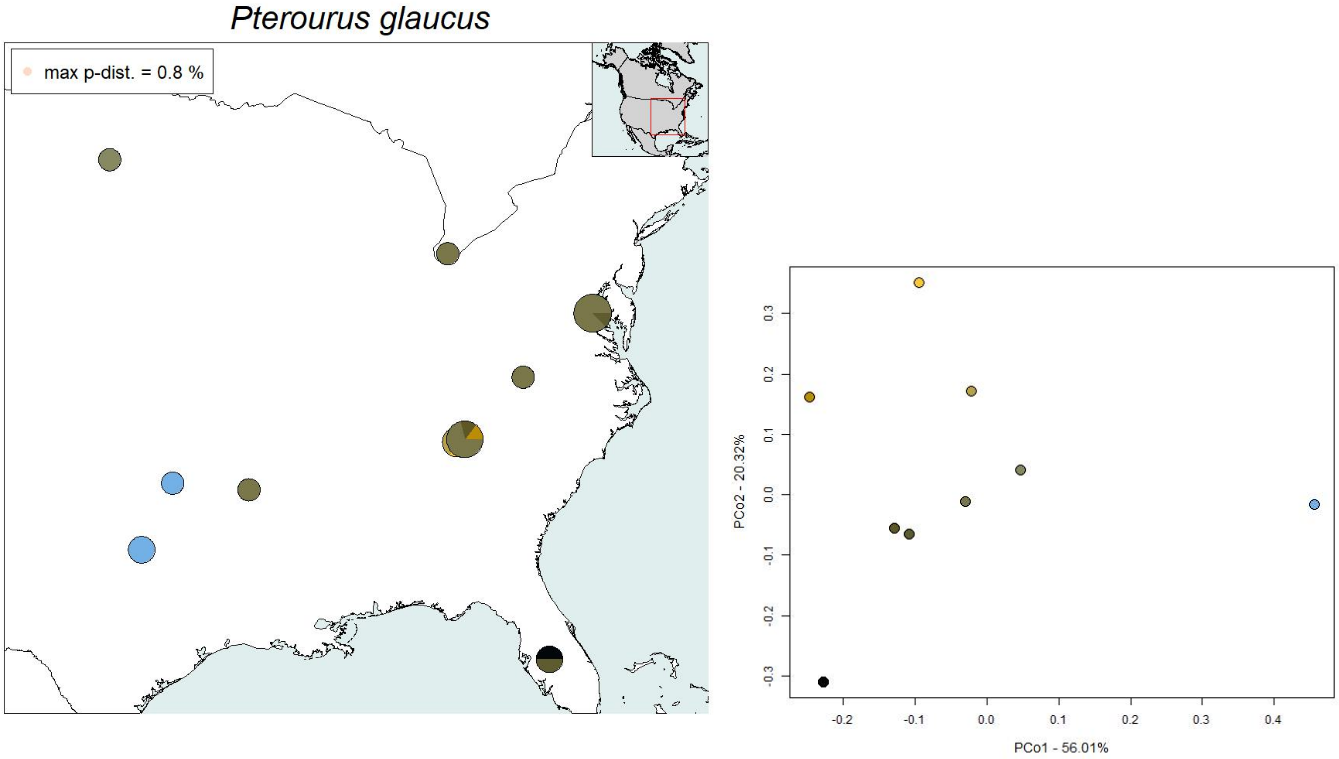

Figure 830 Map of *Pterourus glaucus* showing the localities of the sequenced specimens (left). Nearby localities are grouped in pies. Colours match the bidimensional colour space of the PCoA projection (right) of max p-dists among sequences (dots). Sequences= 27; Hap obs.= 9; Hap asympt.= 29.2; Hap % obs.= 30.8%; GST= 0.059; DST= 0.0001; HD= 0.604; ND= 0.0021; max p-dist= 0.8%.

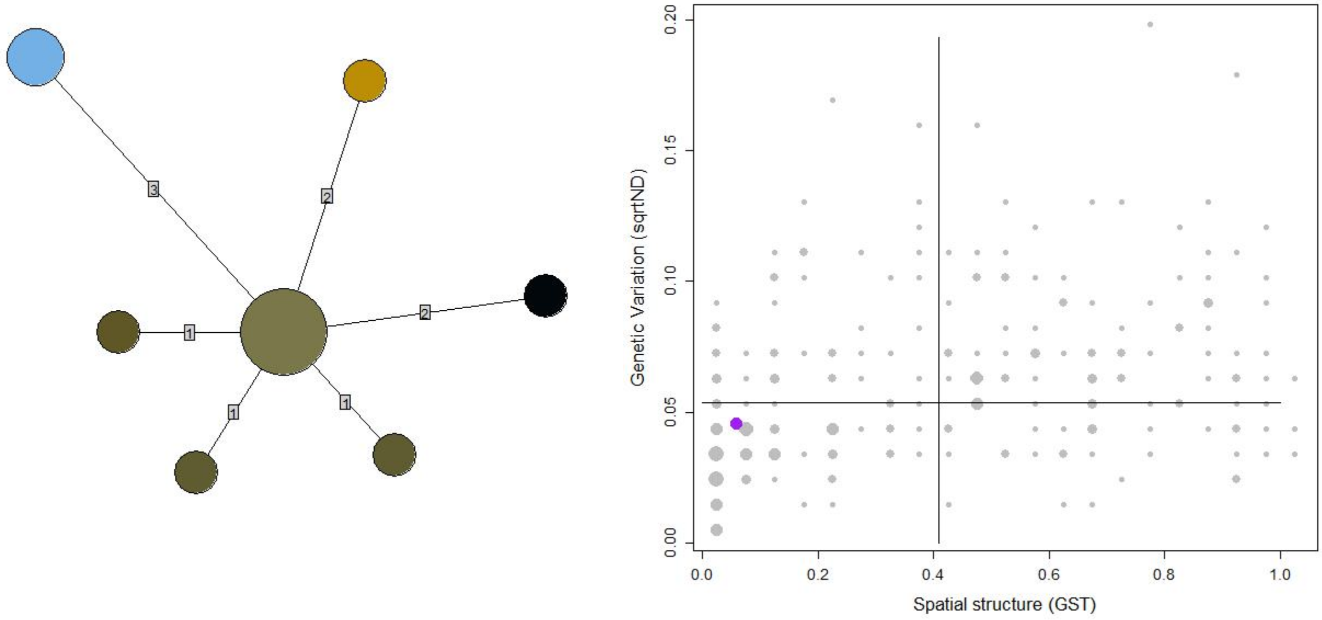

Figure 831: Haplotype network (left) of *Pterourus glaucus* sequences > 599 bp with colours matching the PCoA colour space (above). The bubble plot for mt-DNA polymorphism (square root transformed nucleotide diversity) and spatial structure (GST) among all species in the atlas and values for *Pterourus glaucus* (purple dot). The horizontal and vertical lines represent median values of nucleotide diversity and GST, respectively. Sequences > 599 bp= 24.

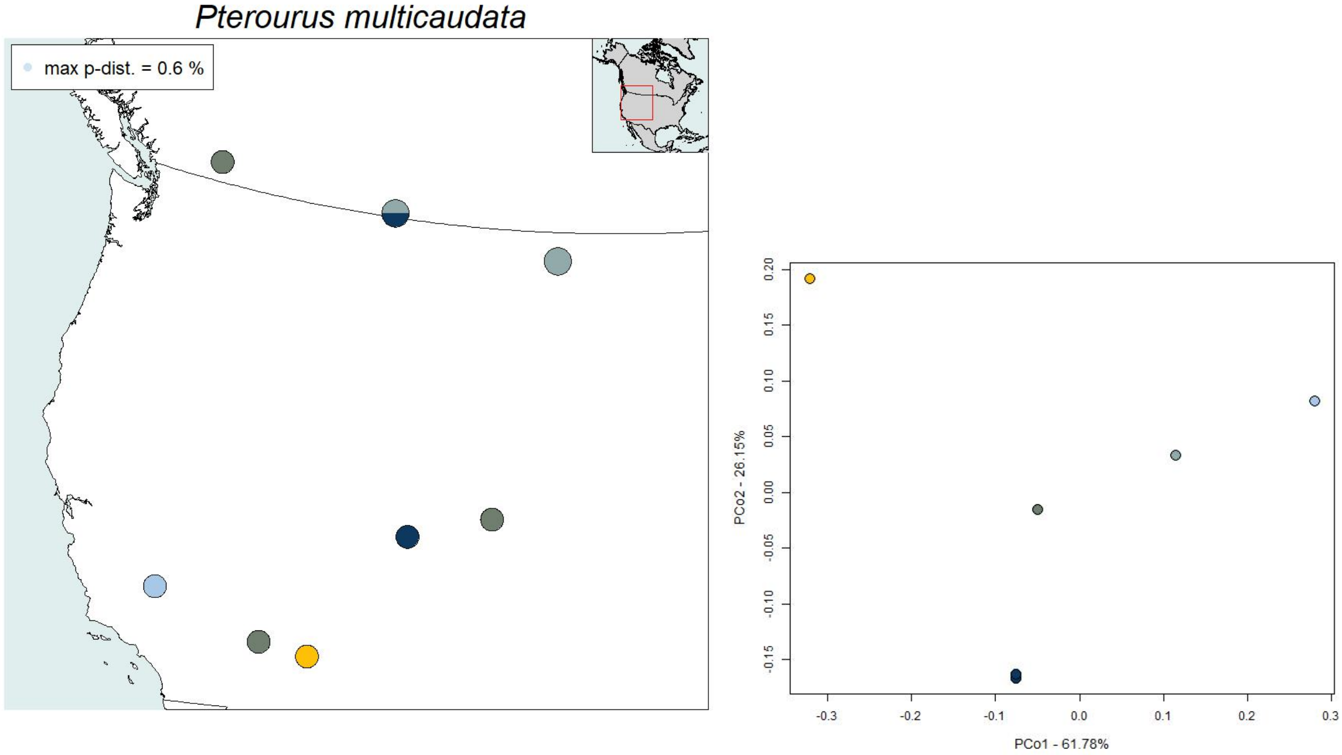

Figure 832 Map of *Pterourus multicaudata* showing the localities of the sequenced specimens (left). Nearby localities are grouped in pies. Colours match the bidimensional colour space of the PCoA projection (right) of max p-dists among sequences (dots). Sequences= 10; Hap obs.= 6; Hap asympt.= 11.4; Hap % obs.= 52.6%; GST= NaN; DST= NaN; HD= 0.867; ND= 0.0023; max p-dist= 0.6%.

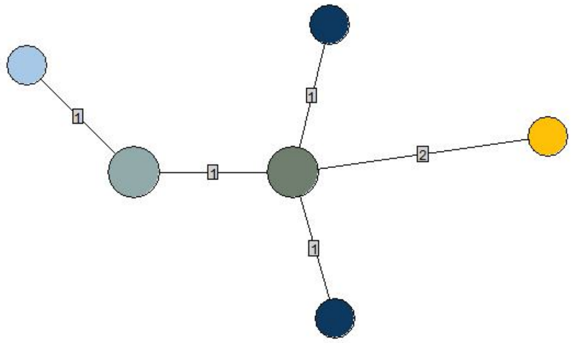

Figure 833: Haplotype network of *Pterourus multicaudata*. Sequences > 599 bp= 10.

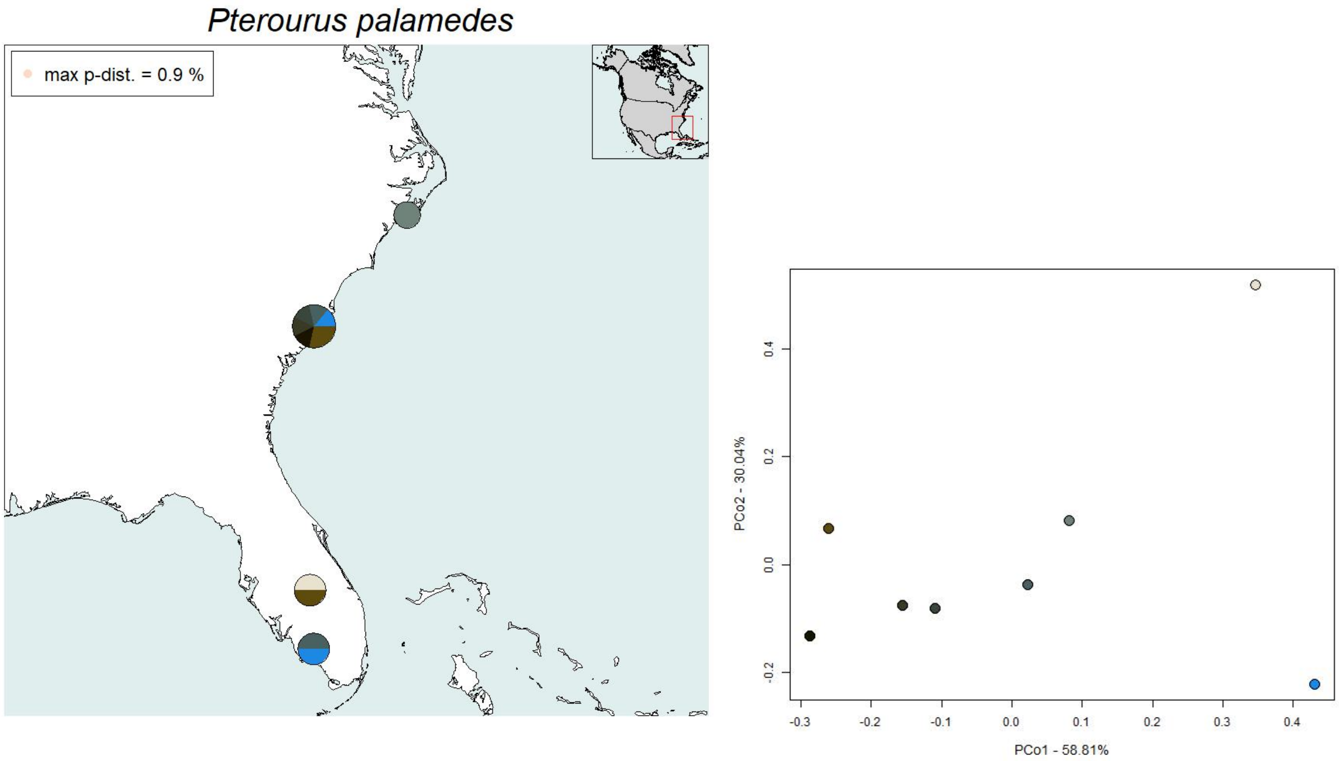

Figure 834 Map of *Pterourus palamedes* showing the localities of the sequenced specimens (left). Nearby localities are grouped in pies. Colours match the bidimensional colour space of the PCoA projection (right) of max p-dists among sequences (dots). Sequences= 12; Hap obs.= 9; Hap asympt.= 31.5; Hap % obs.= 28.6%; GST= 0.116; DST= 0.0005; HD= 0.939; ND= 0.0038; max p-dist= 0.9%.

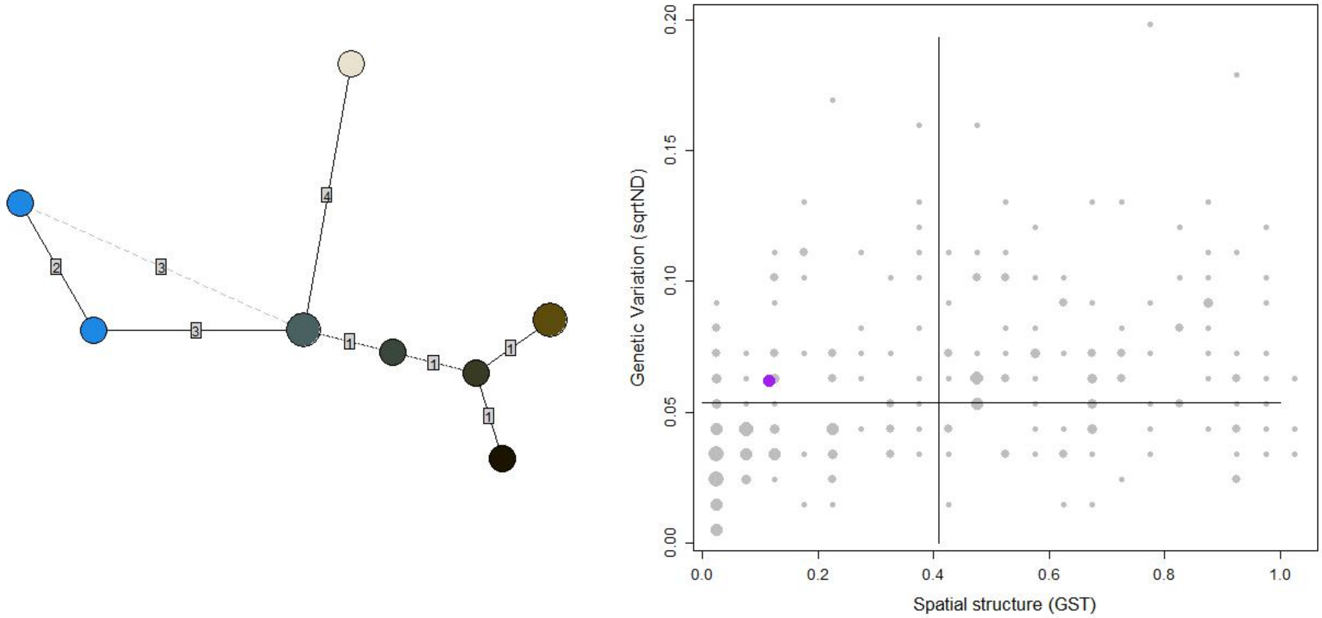

Figure 835: Haplotype network (left) of *Pterourus palamedes* sequences > 599 bp with colours matching the PCoA colour space (above). The bubble plot for mt-DNA polymorphism (square root transformed nucleotide diversity) and spatial structure (GST) among all species in the atlas and values for *Pterourus palamedes* (purple dot). The horizontal and vertical lines represent median values of nucleotide diversity and GST, respectively. Sequences > 599 bp= 12.

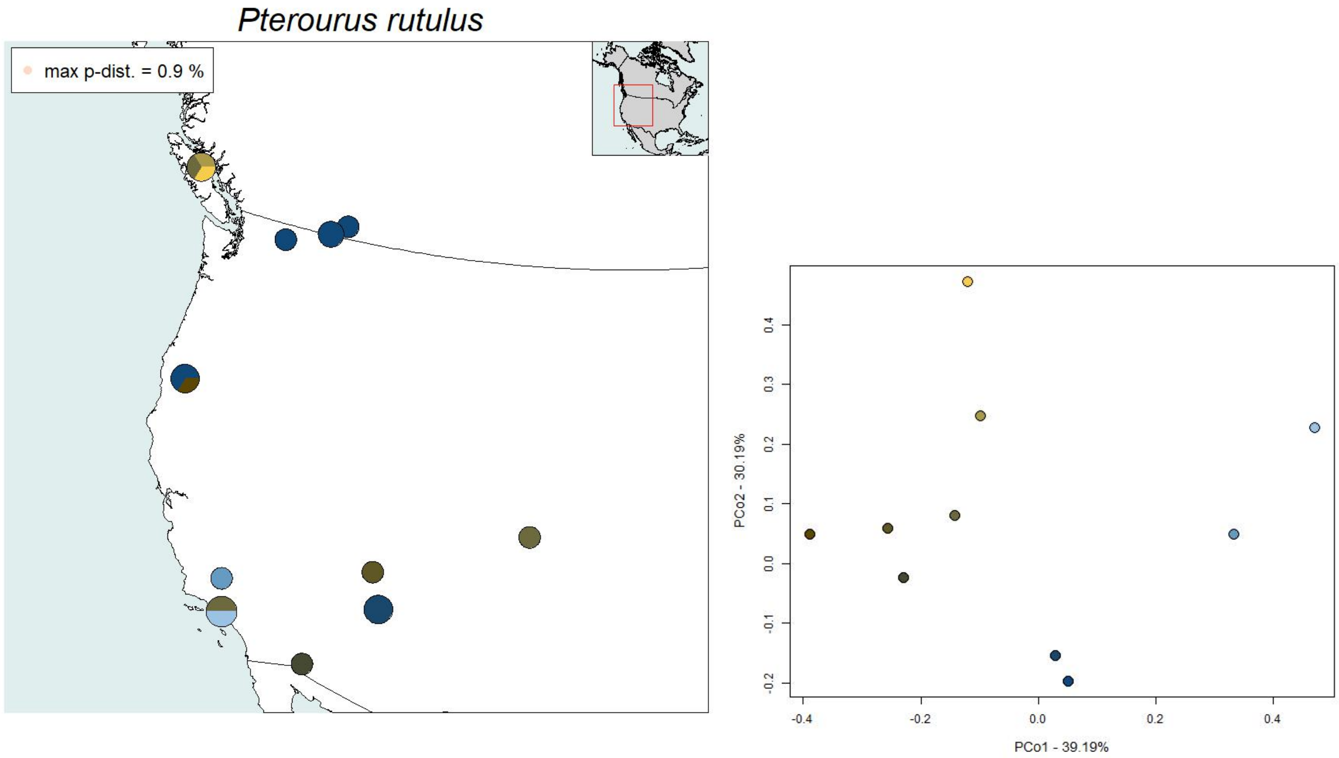

Figure 836 Map of *Pterourus rutulus* showing the localities of the sequenced specimens (left). Nearby localities are grouped in pies. Colours match the bidimensional colour space of the PCoA projection (right) of max p-dists among sequences (dots). Sequences= 21; Hap obs.= 9; Hap asympt.= 20.9; Hap % obs.= 43.1%; GST= 0.3; DST= 0.0012; HD= 0.862; ND= 0.0039; max p-dist= 0.9%.

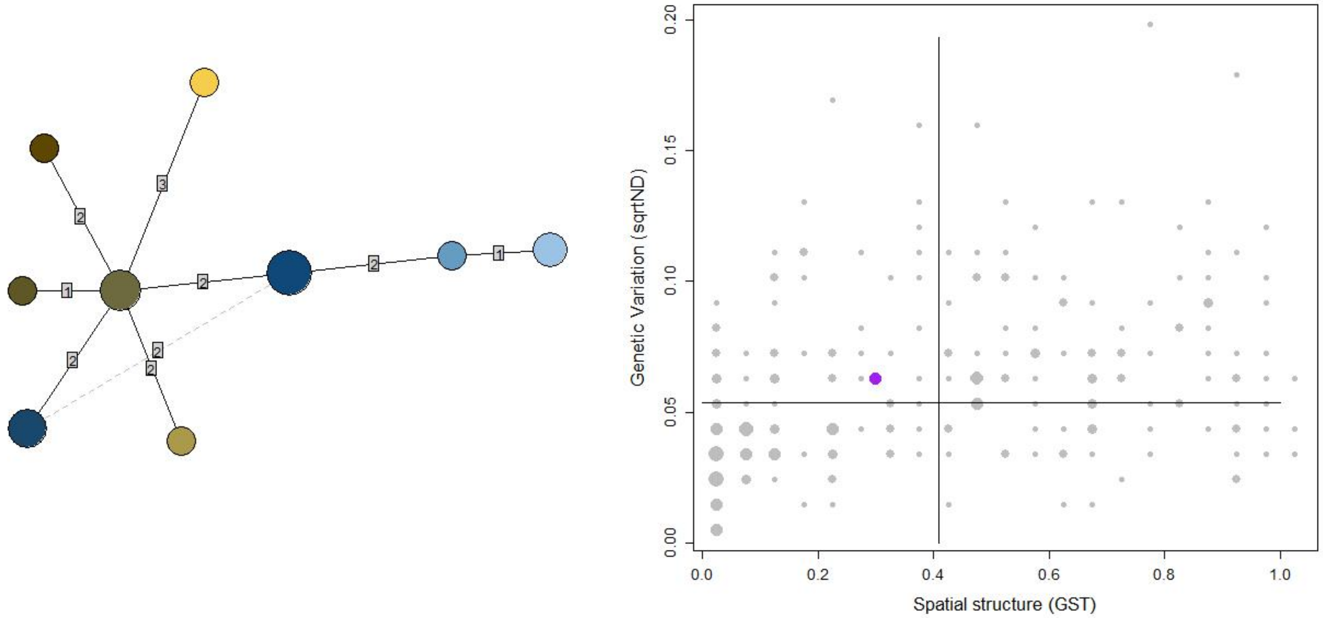

Figure 837: Haplotype network (left) of *Pterourus rutulus* sequences > 599 bp with colours matching the PCoA colour space (above). The bubble plot for mt-DNA polymorphism (square root transformed nucleotide diversity) and spatial structure (GST) among all species in the atlas and values for *Pterourus rutulus* (purple dot). The horizontal and vertical lines represent median values of nucleotide diversity and GST, respectively. Sequences > 599 bp= 20.

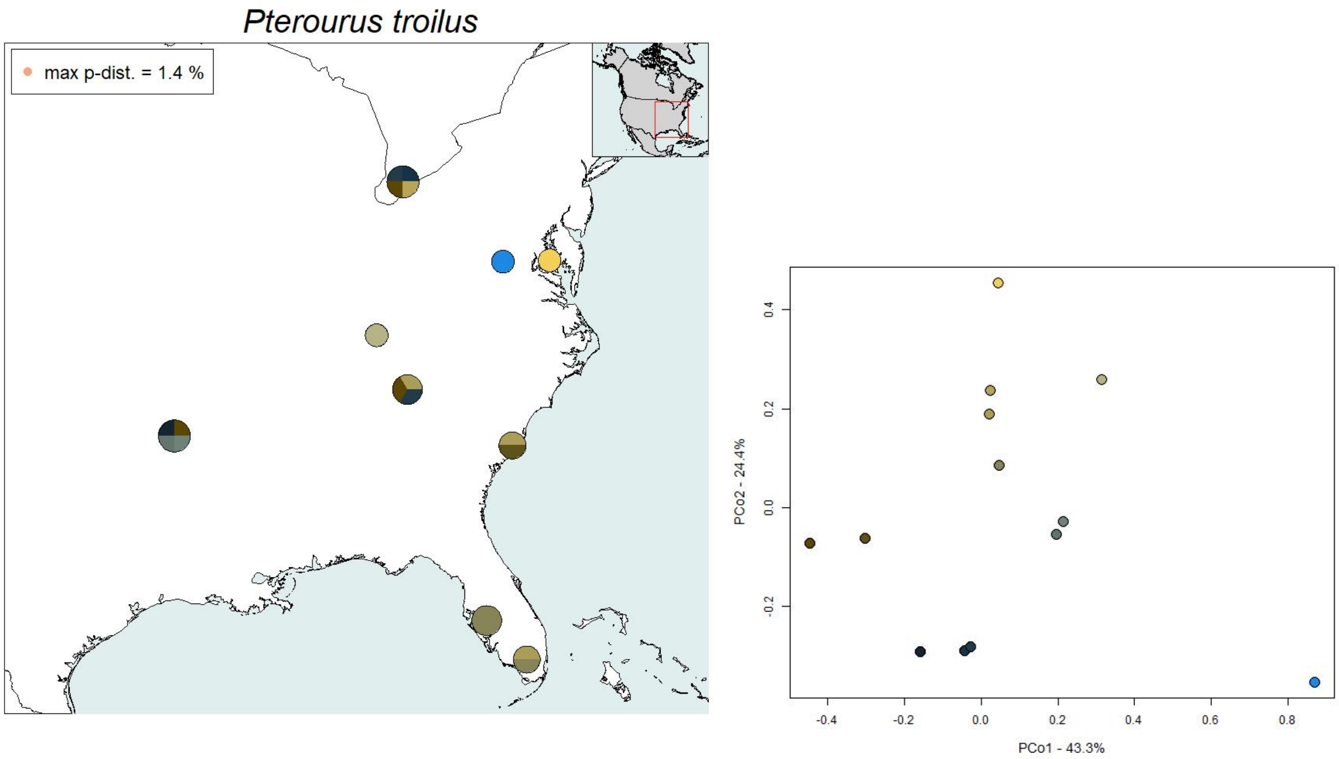

Figure 838 Map of *Pterourus troilus* showing the localities of the sequenced specimens (left). Nearby localities are grouped in pies. Colours match the bidimensional colour space of the PCoA projection (right) of max p-dists among sequences (dots). Sequences= 21; Hap obs.= 16; Hap asympt.= 102.7; Hap % obs.= 15.6%; GST= 0; DST= 0; HD= 0.957; ND= 0.0051; max p-dist= 1.4%.

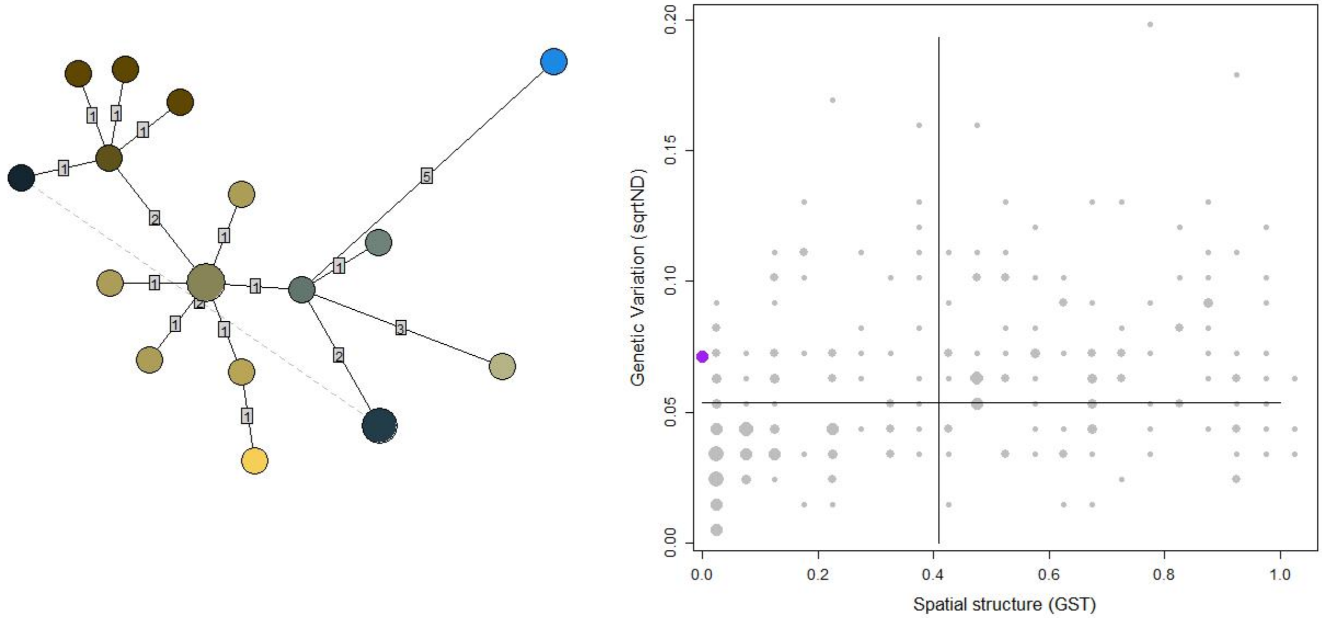

Figure 839: Haplotype network (left) of *Pterourus troilus* sequences > 599 bp with colours matching the PCoA colour space (above). The bubble plot for mt-DNA polymorphism (square root transformed nucleotide diversity) and spatial structure (GST) among all species in the atlas and values for *Pterourus troilus* (purple dot). The horizontal and vertical lines represent median values of nucleotide diversity and GST, respectively. Sequences > 599 bp= 21.

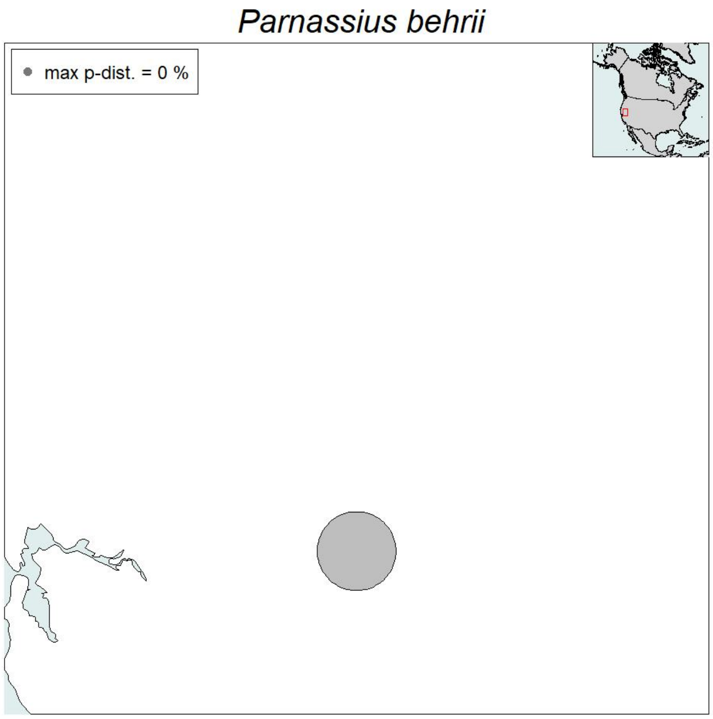

Figure 840: Map of *Parnassius behrii* showing the localities of the sequenced specimens. Nearby localities are grouped in pies. Due to the presence of a single haplotype PCoA projection was not done and a single grey colour was plotted on the map. Sequences= 5; Hap obs.= 1; Hap asympt.= NA; Hap % obs.= NA%; GST= NaN; DST= NaN; HD= NA; ND= NA; max p-dist= 0%.

Haplotype network analysis and bubble plot of *Parnassius behrii* were not possible. Sequences > 599 bp = 5.

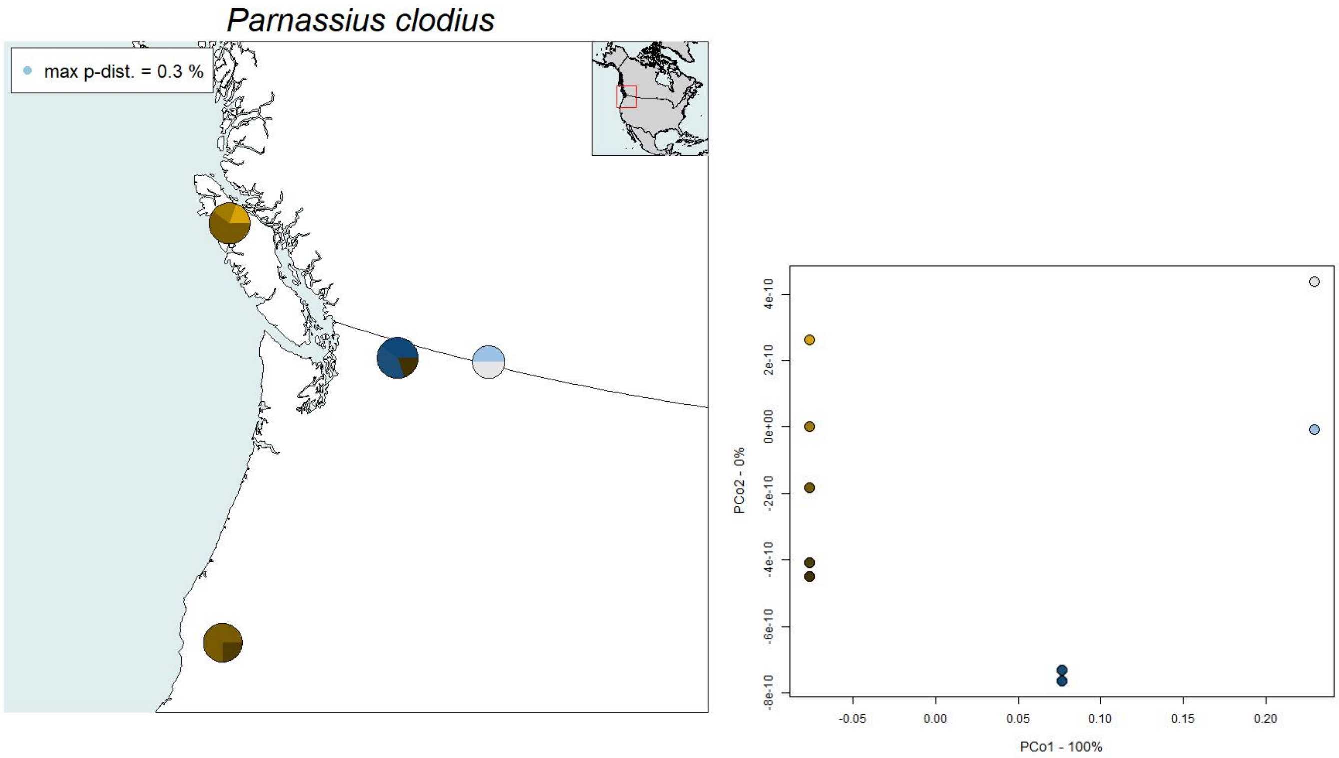

Figure 841 Map of *Parnassius clodius* showing the localities of the sequenced specimens (left). Nearby localities are grouped in pies. Colours match the bidimensional colour space of the PCoA projection (right) of max p-dists among sequences (dots). Sequences= 16; Hap obs.= 3; Hap asympt.= 3; Hap % obs.= 100%; GST= 0.619; DST= 0.0004; HD= 0.567; ND= 0.0011; max p-dist= 0.3%.

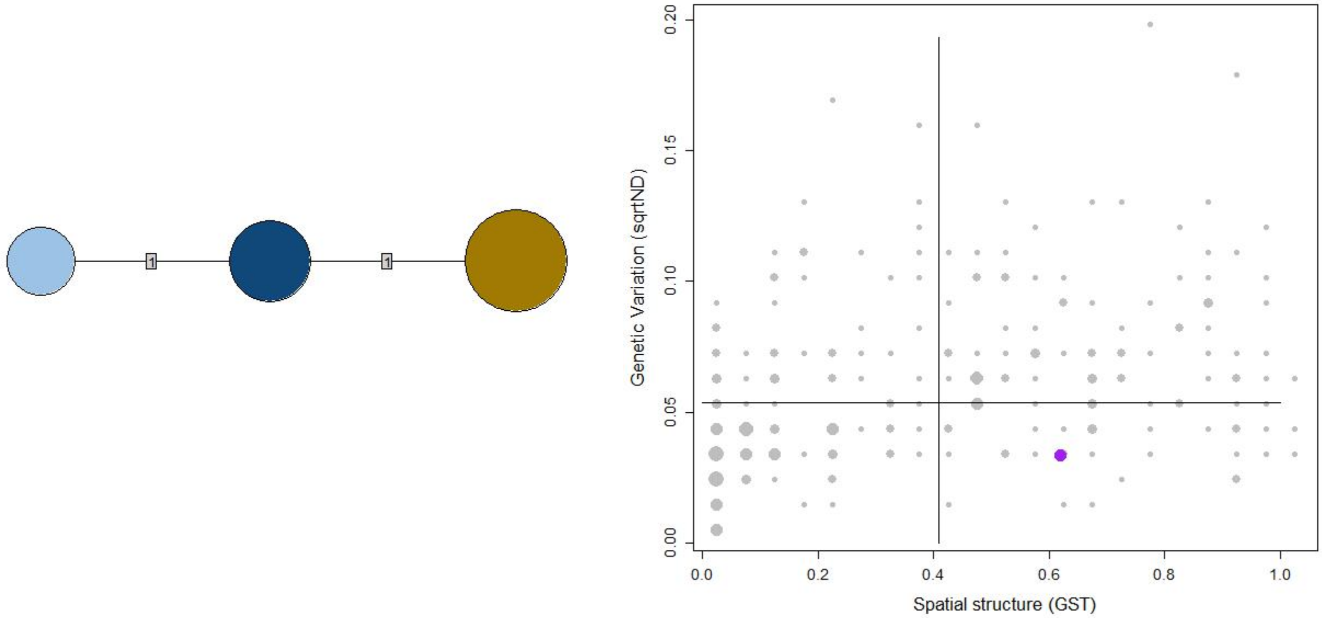

Figure 842: Haplotype network (left) of *Parnassius clodius* sequences > 599 bp with colours matching the PCoA colour space (above). The bubble plot for mt-DNA polymorphism (square root transformed nucleotide diversity) and spatial structure (GST) among all species in the atlas and values for *Parnassius clodius* (purple dot). The horizontal and vertical lines represent median values of nucleotide diversity and GST, respectively. Sequences > 599 bp= 16.

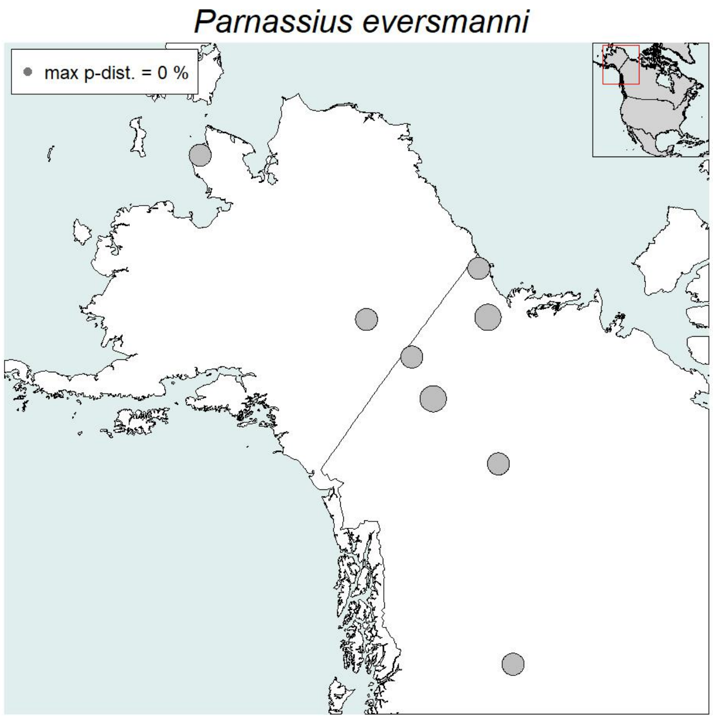

Figure 843: Map of *Parnassius eversmanni* showing the localities of the sequenced specimens. Nearby localities are grouped in pies. Due to the presence of a single haplotype PCoA projection was not done and a single grey colour was plotted on the map. Sequences= 10; Hap obs.= 1; Hap asympt.= NA; Hap % obs.= NA%; GST= NaN; DST= NaN; HD= 0; ND= 0; max p-dist= 0%.

Haplotype network analysis and bubble plot of *Parnassius eversmanni* were not possible. Sequences > 599 bp = 8.

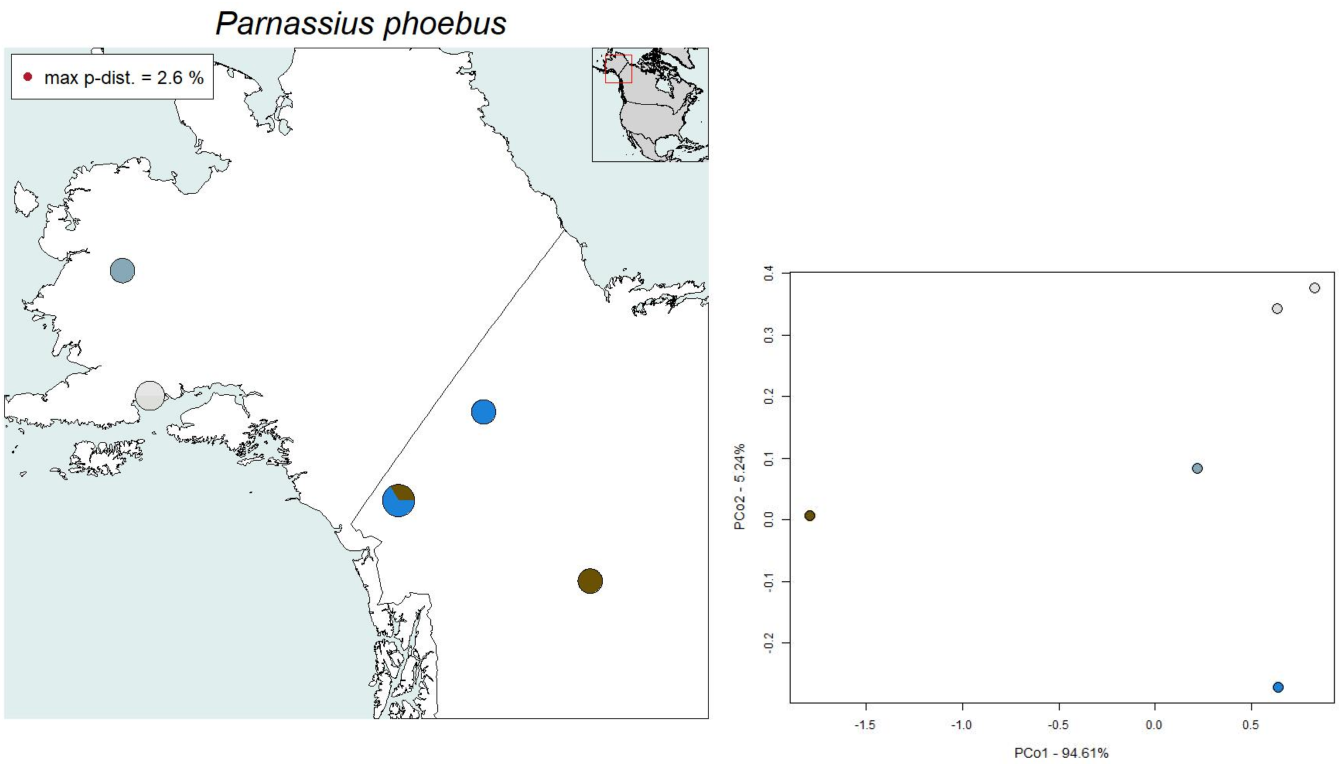

Figure 844 Map of *Parnassius phoebus* showing the localities of the sequenced specimens (left). Nearby localities are grouped in pies. Colours match the bidimensional colour space of the PCoA projection (right) of max p-dists among sequences (dots). Sequences= 8; Hap obs.= 4; Hap asympt.= NA; Hap % obs.= NA%; GST= NaN; DST= NaN; HD= NA; ND= NA; max p-dist= 2.6%.

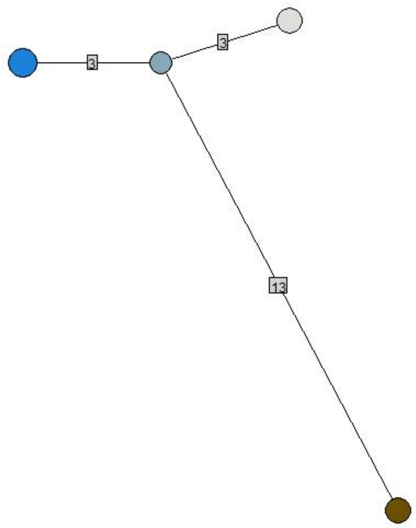

Figure 845: Haplotype network of *Parnassius phoebus*. Sequences > 599 bp= 8.

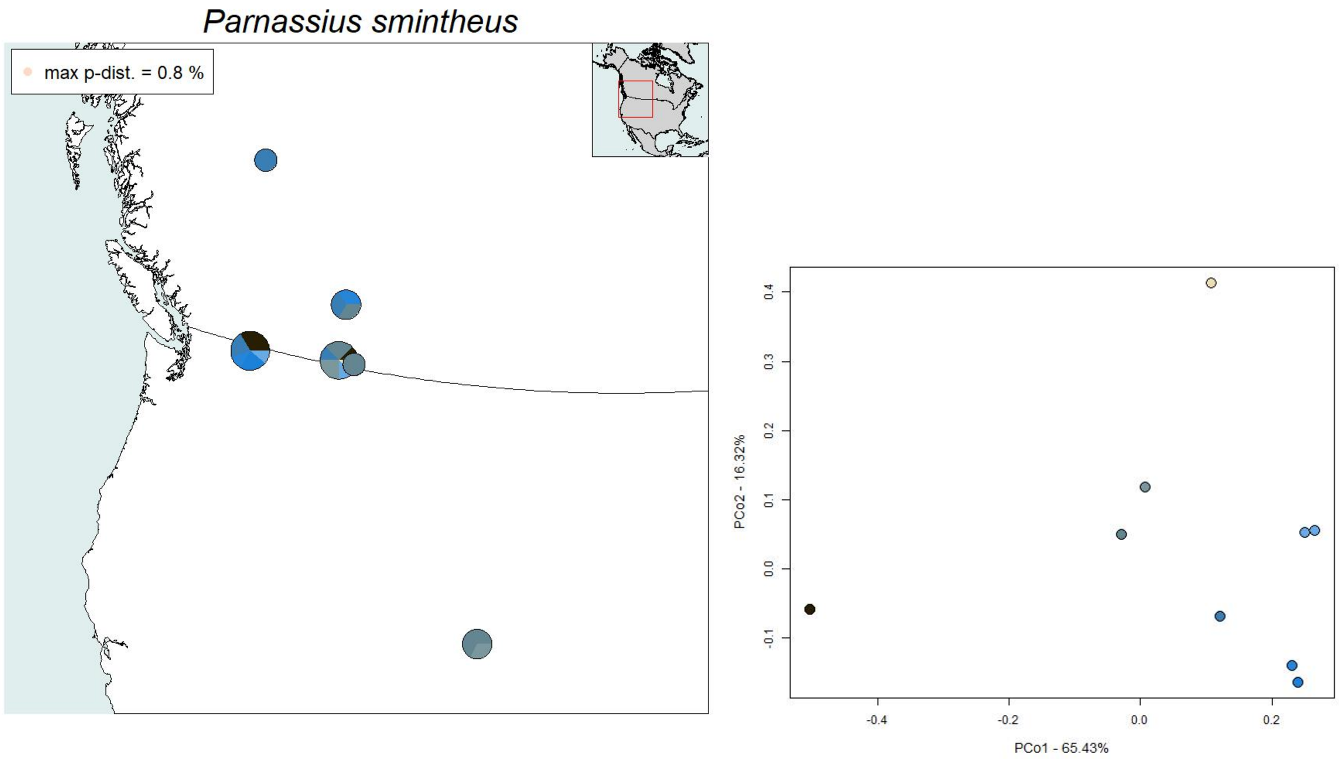

Figure 846 Map of *Parnassius smintheus* showing the localities of the sequenced specimens (left). Nearby localities are grouped in pies. Colours match the bidimensional colour space of the PCoA projection (right) of max p-dists among sequences (dots). Sequences= 25; Hap obs.= 11; Hap asympt.= 19.6; Hap % obs.= 56%; GST= 0.216; DST= 0.0008; HD= 0.89; ND= 0.0034; max p-dist= 0.8%.

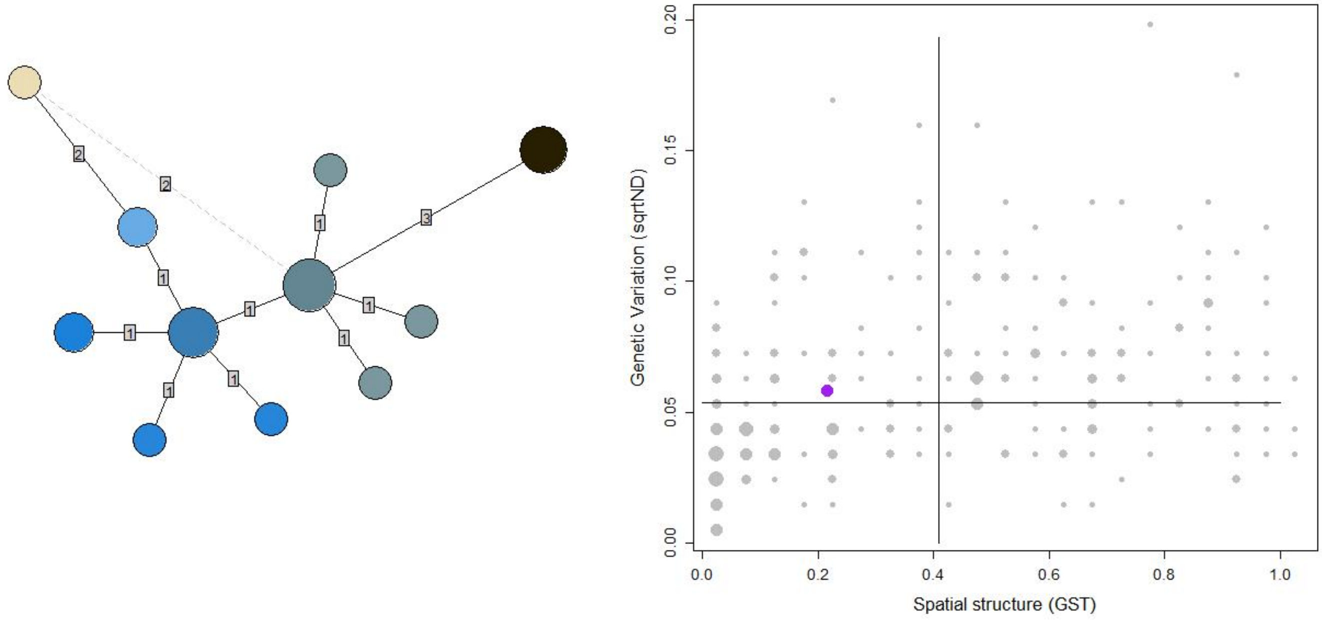

Figure 847: Haplotype network (left) of *Parnassius smintheus* sequences > 599 bp with colours matching the PCoA colour space (above). The bubble plot for mt-DNA polymorphism (square root transformed nucleotide diversity) and spatial structure (GST) among all species in the atlas and values for *Parnassius smintheus* (purple dot). The horizontal and vertical lines represent median values of nucleotide diversity and GST, respectively. Sequences > 599 bp= 25.

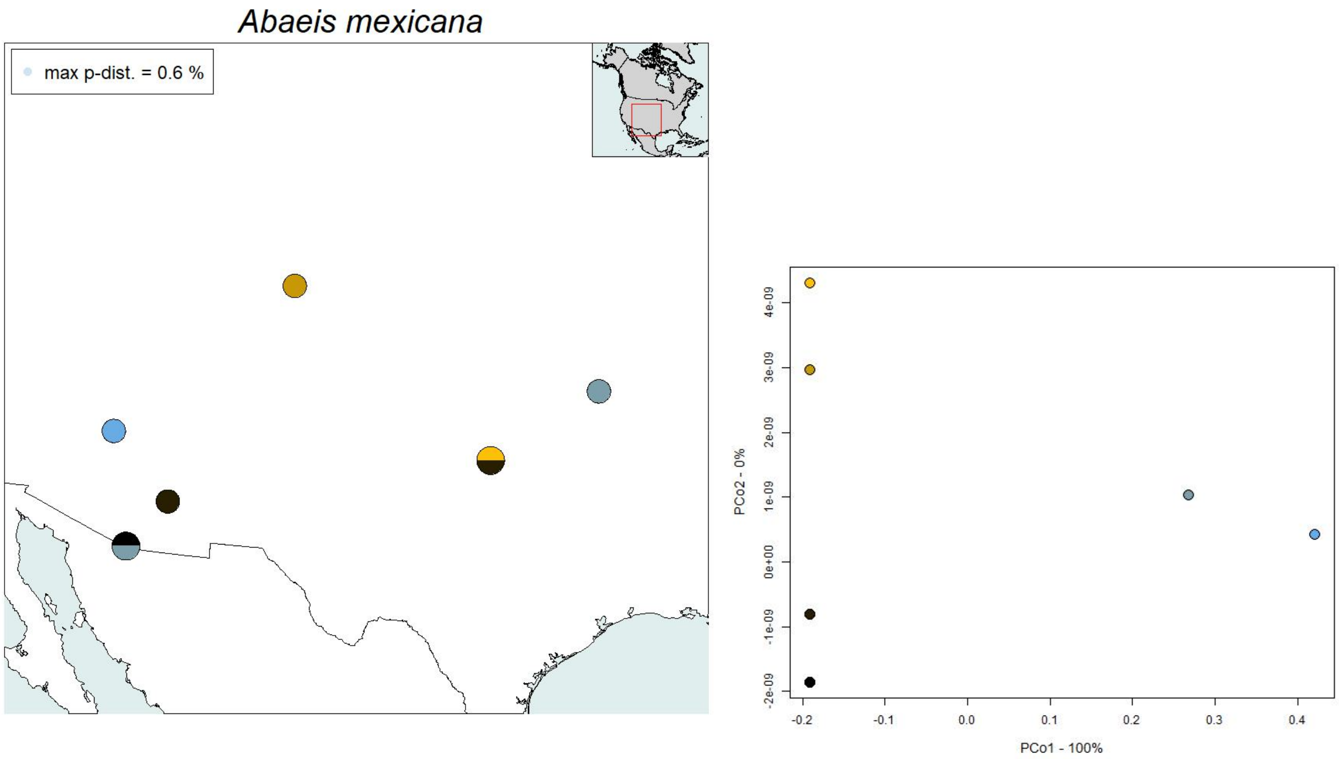

Figure 848 Map of *Abaeis mexicana* showing the localities of the sequenced specimens (left). Nearby localities are grouped in pies. Colours match the bidimensional colour space of the PCoA projection (right) of max p-dists among sequences (dots). Sequences= 8; Hap obs.= 3; Hap asympt.= NA; Hap % obs.= NA%; GST= NaN; DST= NaN; HD= NA; ND= NA; max p-dist= 0.6%.

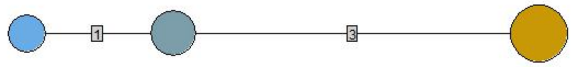

Figure 849: Haplotype network of *Abaeis mexicana*. Sequences > 599 bp= 8.

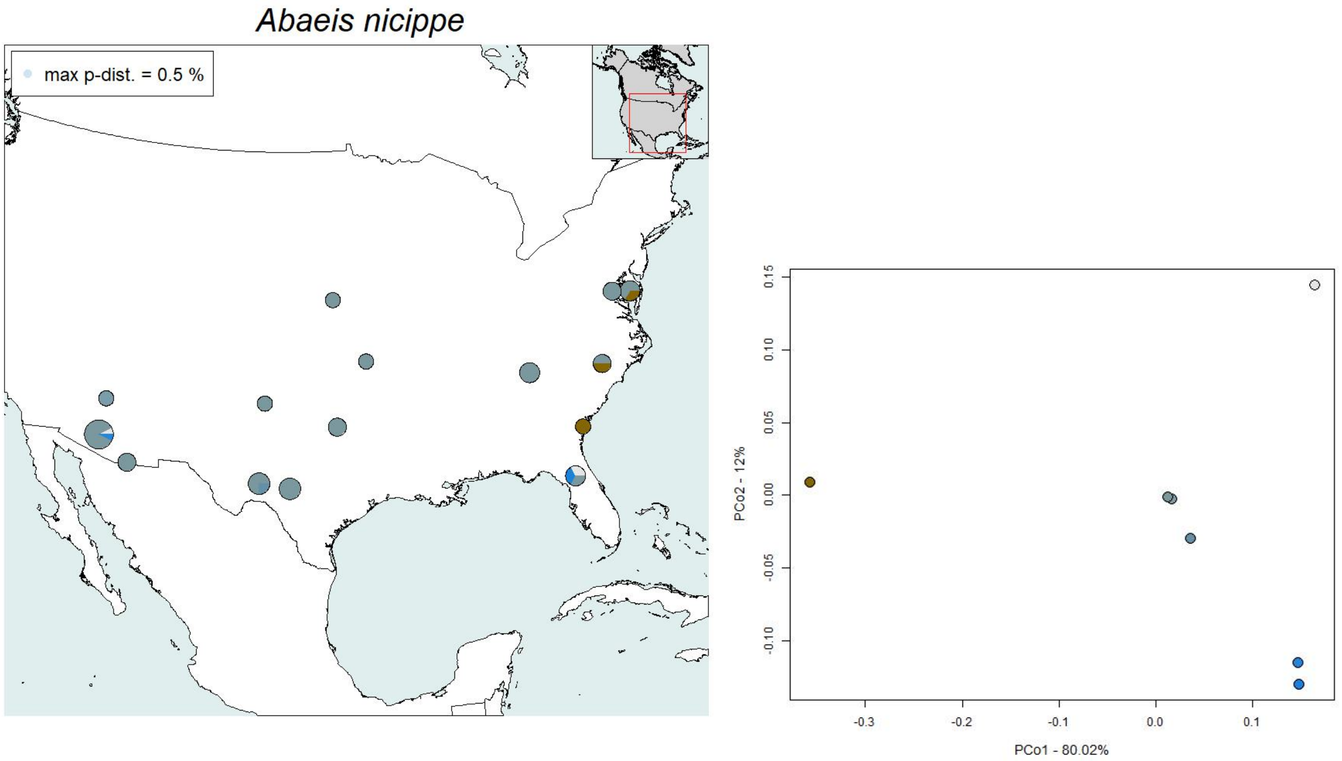

Figure 850 Map of *Abaeis nicippe* showing the localities of the sequenced specimens (left). Nearby localities are grouped in pies. Colours match the bidimensional colour space of the PCoA projection (right) of max p-dists among sequences (dots). Sequences= 43; Hap obs.= 6; Hap asympt.= 10.4; Hap % obs.= 57.7%; GST= 0.1; DST= 0.0001; HD= 0.337; ND= 0.0008; max p-dist= 0.5%.

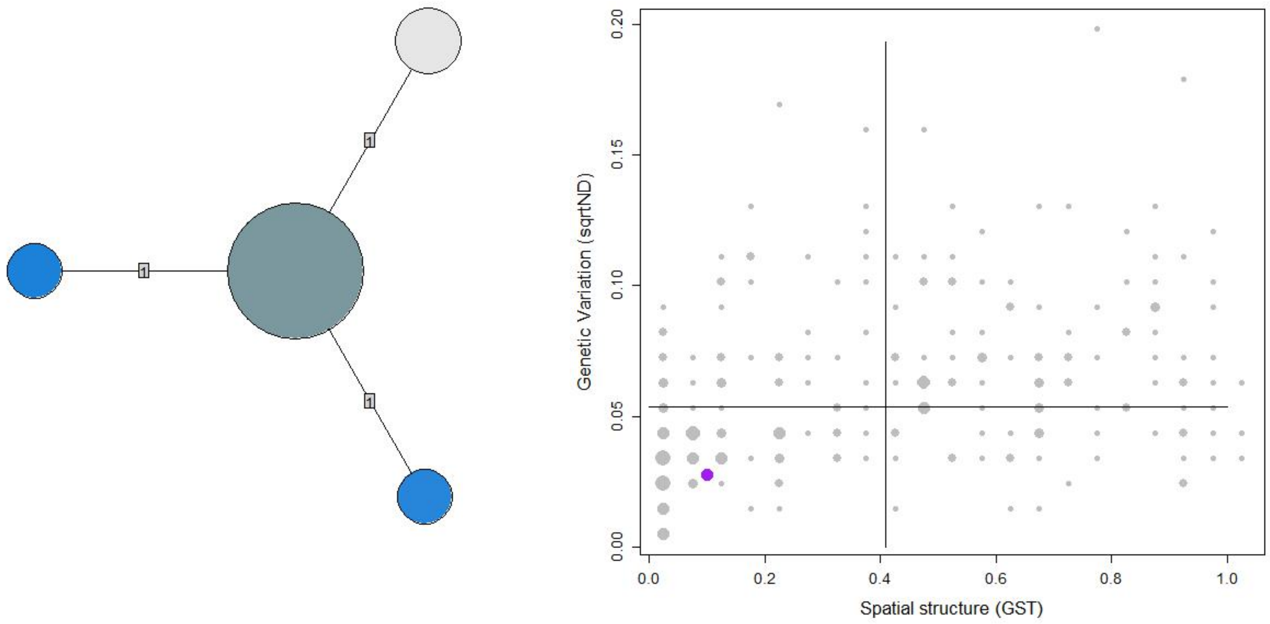

Figure 851: Haplotype network (left) of *Abaeis nicippe* sequences > 599 bp with colours matching the PCoA colour space (above). The bubble plot for mt-DNA polymorphism (square root transformed nucleotide diversity) and spatial structure (GST) among all species in the atlas and values for *Abaeis nicippe* (purple dot). The horizontal and vertical lines represent median values of nucleotide diversity and GST, respectively. Sequences > 599 bp= 40.

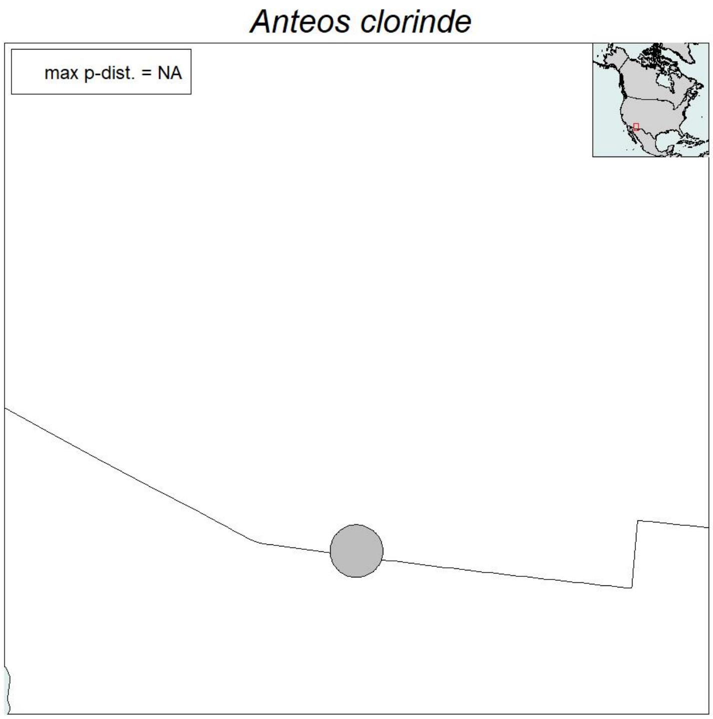

Figure 852: Map of *Anteos clorinde* showing the localities of the sequenced specimens. Nearby localities are grouped in pies. Due to the presence of a single haplotype PCoA projection was not done and a single grey colour was plotted on the map. Sequences= 1; Hap obs.= NA; Hap asympt.= NA; Hap % obs.= NA; GST= NaN; DST= NaN; HD= NA; ND= NA; max p-dist= NA.

Haplotype network analysis and bubble plot of *Anteos clorinde* were not possible. Sequences > 599 bp = 1.

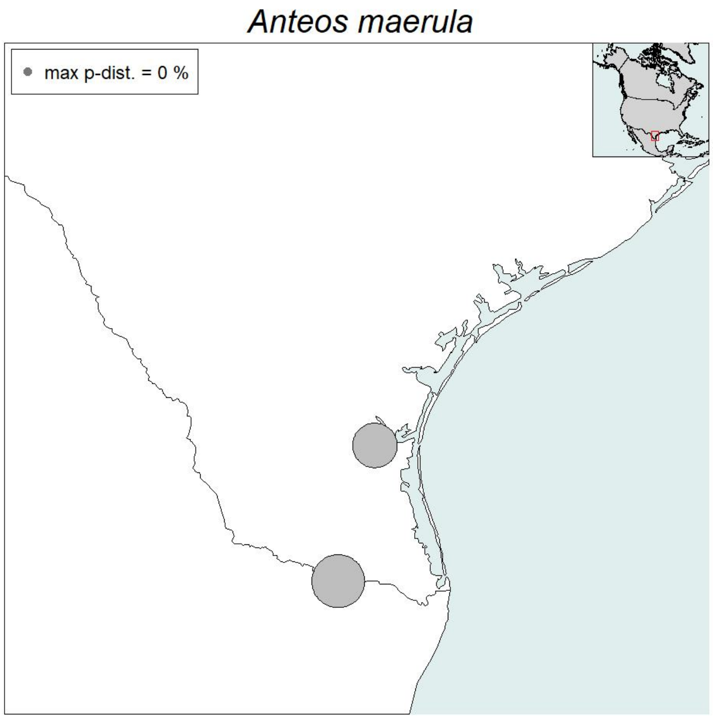

Figure 853: Map of *Anteos maerula* showing the localities of the sequenced specimens. Nearby localities are grouped in pies. Due to the presence of a single haplotype PCoA projection was not done and a single grey colour was plotted on the map. Sequences= 3; Hap obs.= 1; Hap asympt.= NA; Hap % obs.= NA%; GST= NaN; DST= NaN; HD= NA; ND= NA; max p-dist= 0%.

Haplotype network analysis and bubble plot of *Anteos maerula* were not possible. Sequences > 599 bp = 3.

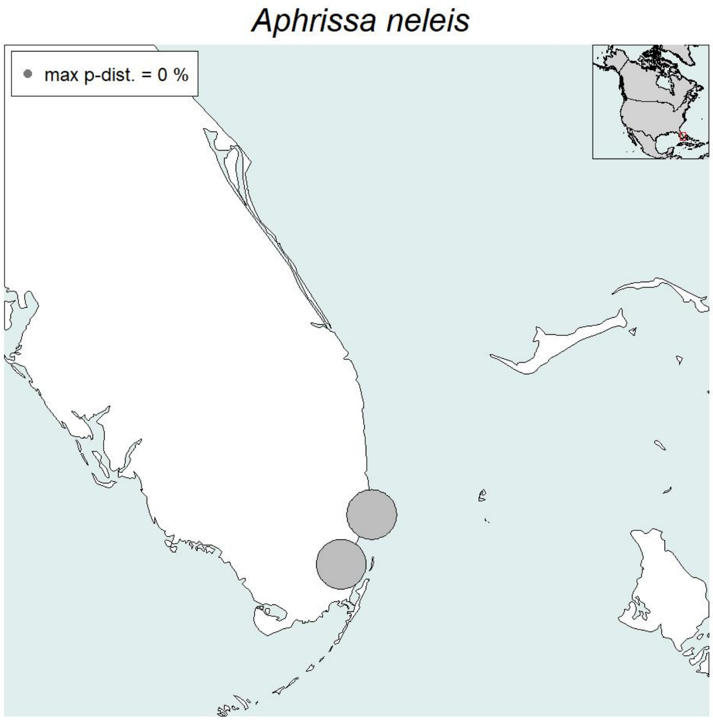

Figure 854: Map of *Aphrissa neleis* showing the localities of the sequenced specimens. Nearby localities are grouped in pies. Due to the presence of a single haplotype PCoA projection was not done and a single grey colour was plotted on the map. Sequences= 2; Hap obs.= 1; Hap asympt.= NA; Hap % obs.= NA%; GST= NaN; DST= NaN; HD= NA; ND= NA; max p-dist= 0%.

Haplotype network analysis and bubble plot of *Aphrissa neleis* were not possible. Sequences > 599 bp = 2.

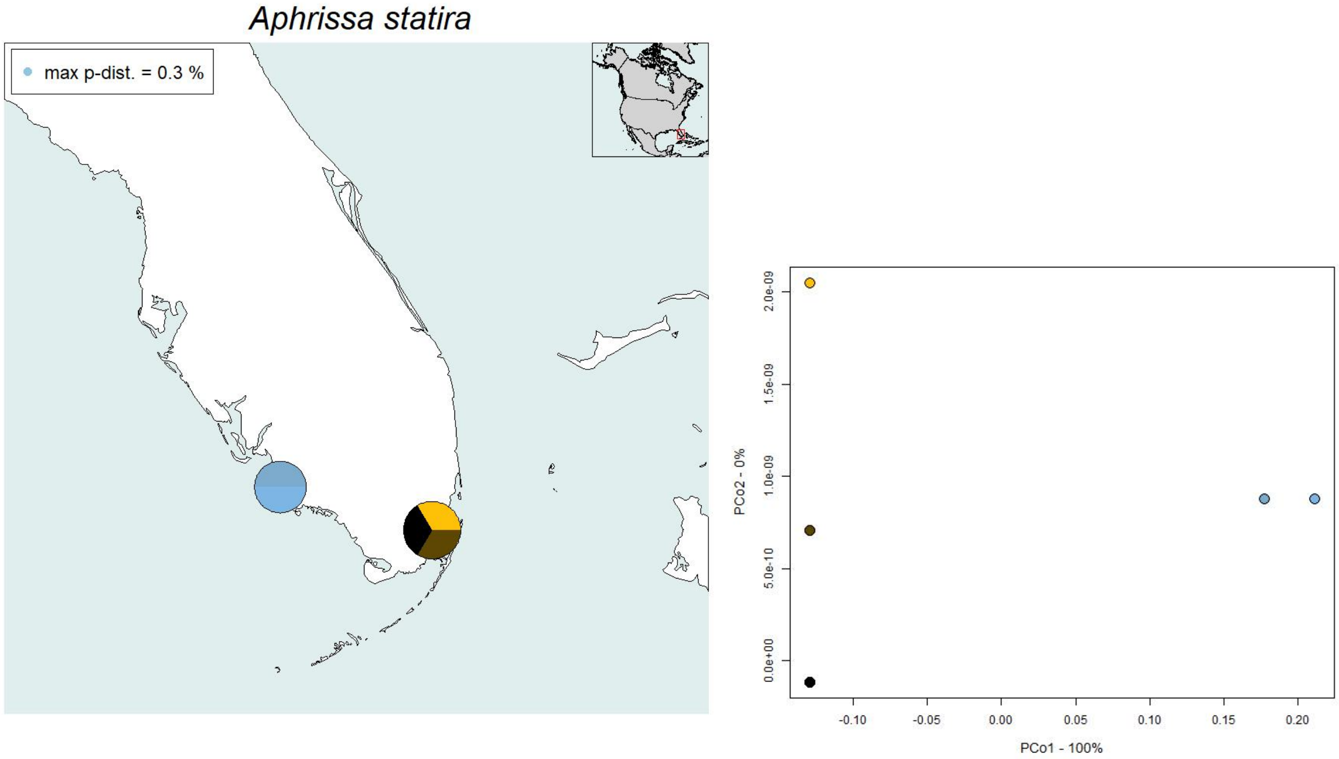

Figure 855 Map of *Aphrissa statira* showing the localities of the sequenced specimens (left). Nearby localities are grouped in pies. Colours match the bidimensional colour space of the PCoA projection (right) of max p-dists among sequences (dots). Sequences= 5; Hap obs.= 2; Hap asympt.= NA; Hap % obs.= NA%; GST= NaN; DST= NaN; HD= NA; ND= NA; max p-dist= 0.3%.

Haplotype network analysis and bubble plot of *Aphrissa statira* were not possible. Sequences > 599 bp = 4.

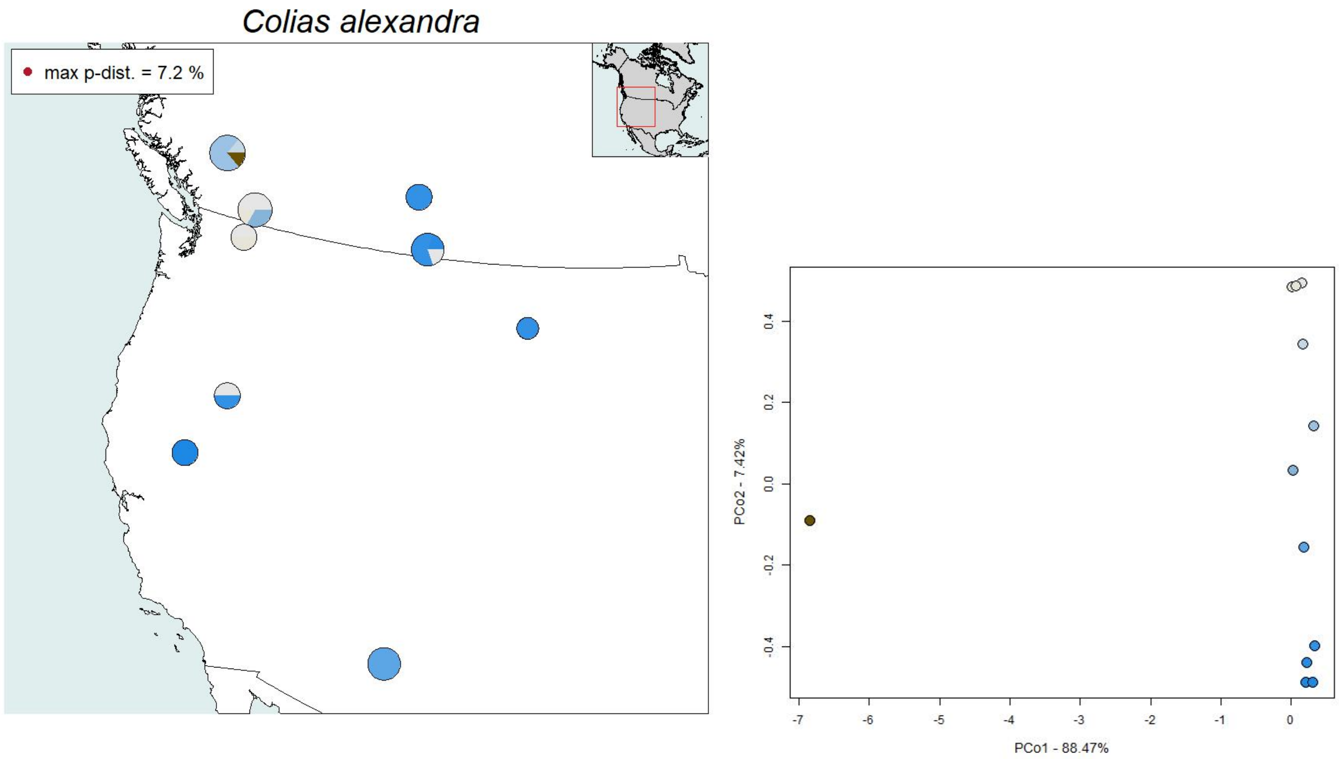

Figure 856 Map of *Colias alexandra* showing the localities of the sequenced specimens (left). Nearby localities are grouped in pies. Colours match the bidimensional colour space of the PCoA projection (right) of max p-dists among sequences (dots). Sequences= 32; Hap obs.= 7; Hap asympt.= 8.9; Hap % obs.= 78.3%; GST= 0.156; DST= 0.0017; HD= 0.81; ND= 0.0097; max p-dist= 7.2%.

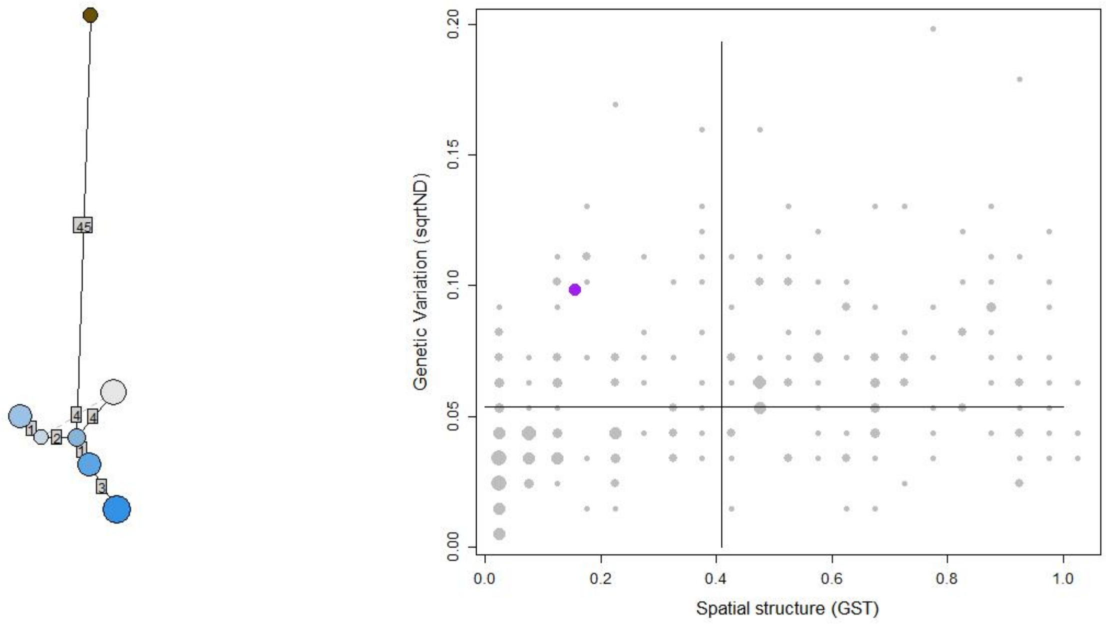

Figure 857: Haplotype network (left) of *Colias alexandra* sequences > 599 bp with colours matching the PCoA colour space (above). The bubble plot for mt-DNA polymorphism (square root transformed nucleotide diversity) and spatial structure (GST) among all species in the atlas and values for *Colias alexandra* (purple dot). The horizontal and vertical lines represent median values of nucleotide diversity and GST, respectively. Sequences > 599 bp= 32.

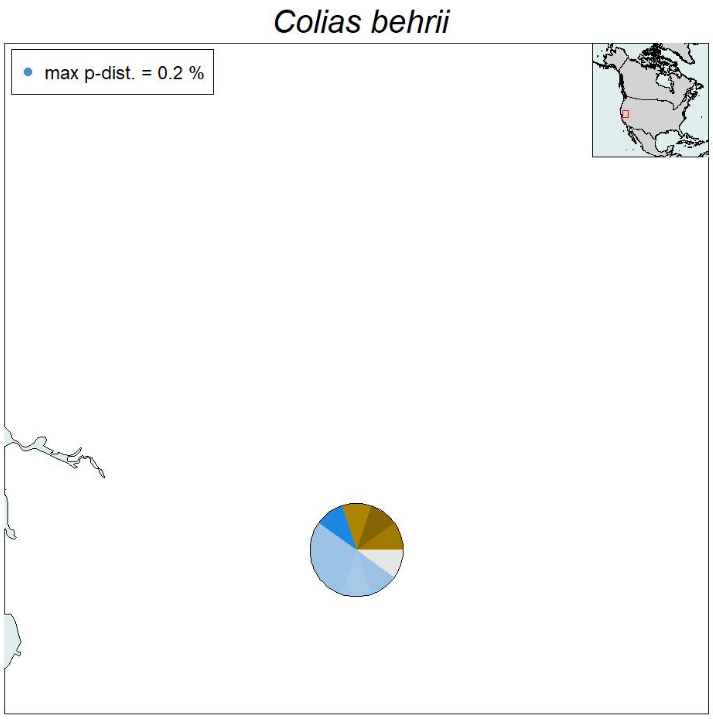

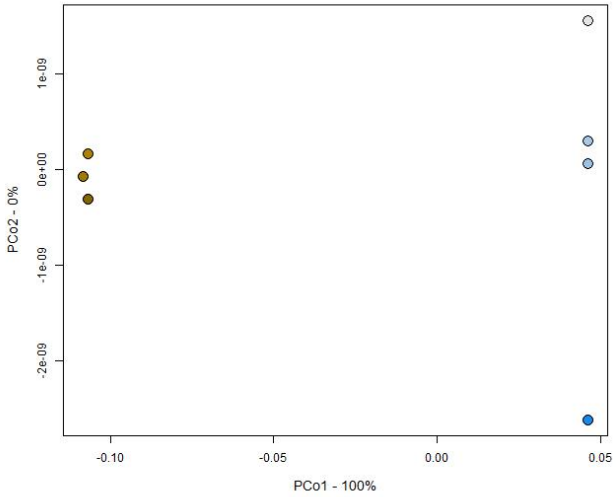

Figure 858 Map of *Colias behrii* showing the localities of the sequenced specimens (left). Nearby localities are grouped in pies. Colours match the bidimensional colour space of the PCoA projection (right) of max p-dists among sequences (dots). Sequences= 10; Hap obs.= 2; Hap asympt.= 2; Hap % obs.= 100%; GST= NaN; DST= NaN; HD= 0.467; ND= 0.0007; max p-dist= 0.2%.

Haplotype network analysis and bubble plot of *Colias behrii* were not possible. Sequences > 599 bp = 10.

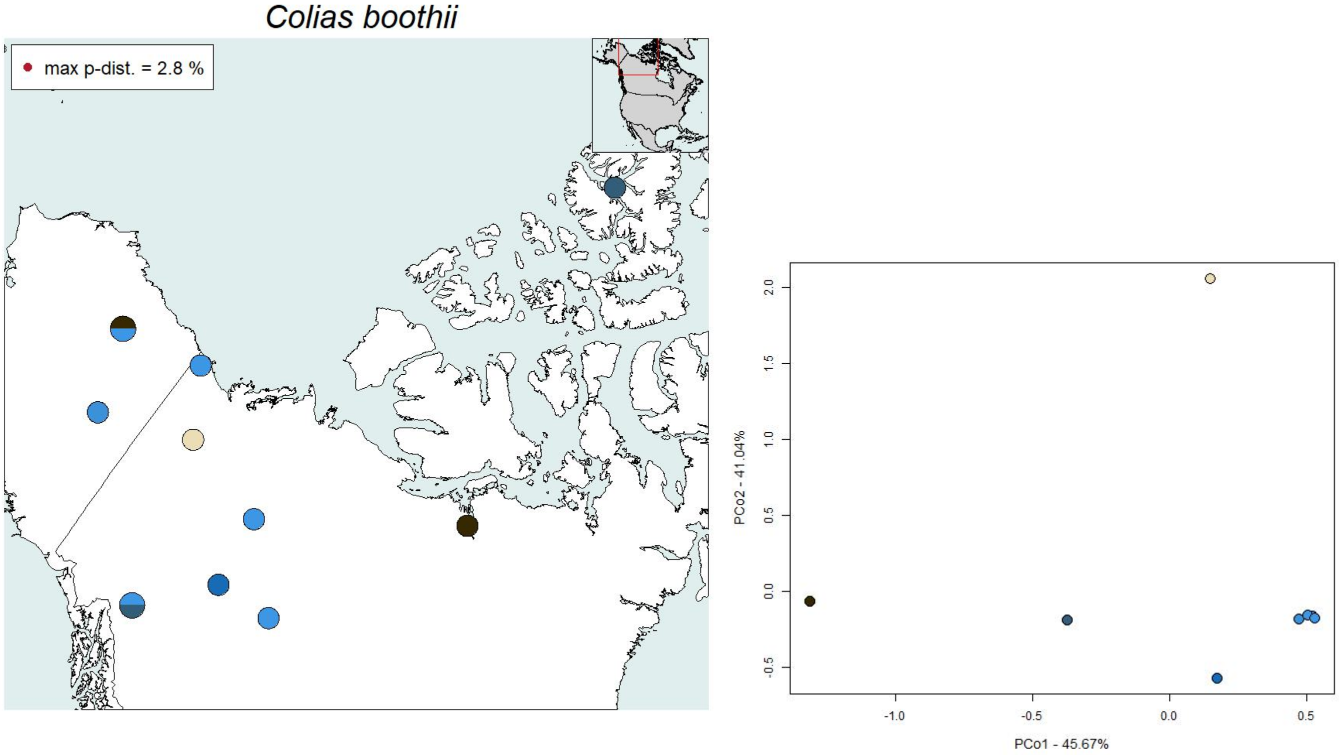

Figure 859 Map of *Colias boothii* showing the localities of the sequenced specimens (left). Nearby localities are grouped in pies. Colours match the bidimensional colour space of the PCoA projection (right) of max p-dists among sequences (dots). Sequences= 12; Hap obs.= 6; Hap asympt.= 8.1; Hap % obs.= 74.4%; GST= NaN; DST= NaN; HD= 0.818; ND= 0.0119; max p-dist= 2.8%.

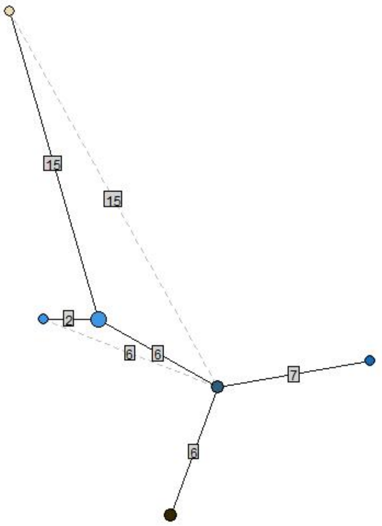

Figure 860: Haplotype network of *Colias boothii*. Sequences > 599 bp= 12.

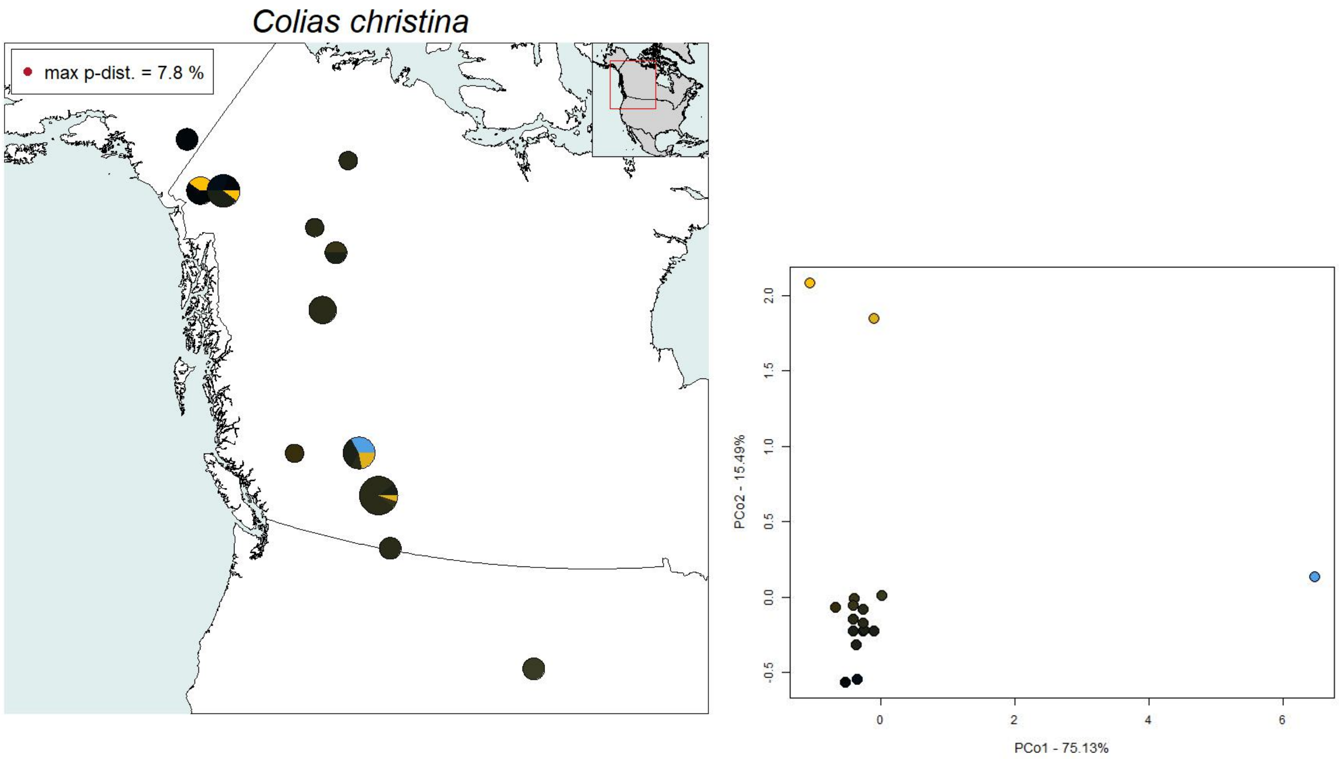

Figure 861 Map of *Colias christina* showing the localities of the sequenced specimens (left). Nearby localities are grouped in pies. Colours match the bidimensional colour space of the PCoA projection (right) of max p-dists among sequences (dots). Sequences= 59; Hap obs.= 14; Hap asympt.= 17.9; Hap % obs.= 78.1%; GST= 0.378; DST= 0.006; HD= 0.799; ND= 0.0147; max p-dist= 7.8%.

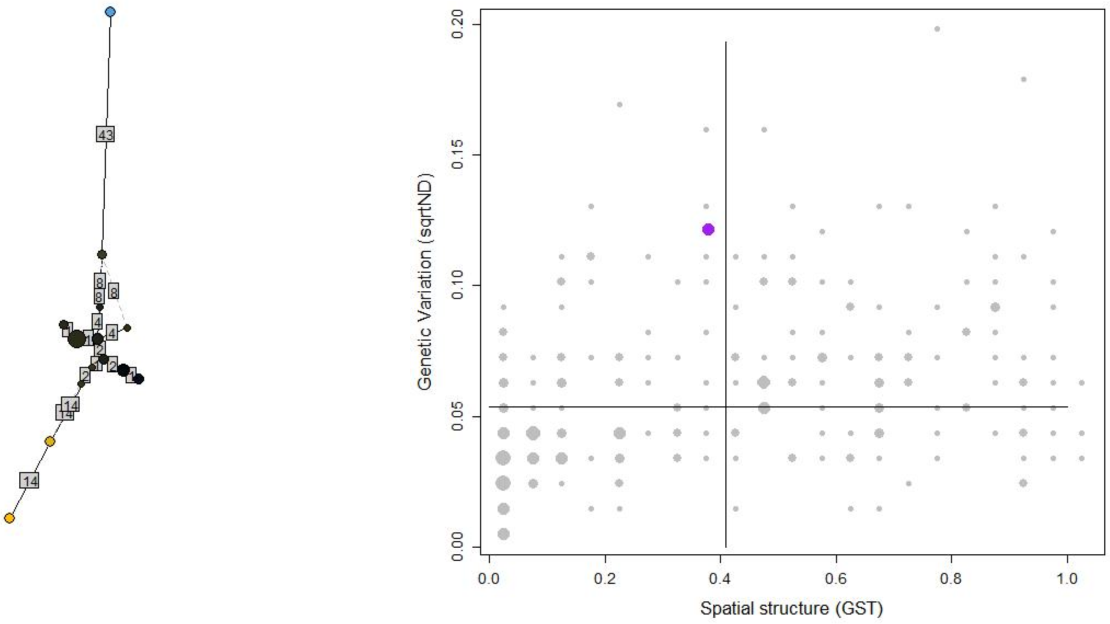

Figure 862: Haplotype network (left) of *Colias christina* sequences > 599 bp with colours matching the PCoA colour space (above). The bubble plot for mt-DNA polymorphism (square root transformed nucleotide diversity) and spatial structure (GST) among all species in the atlas and values for *Colias christina* (purple dot). The horizontal and vertical lines represent median values of nucleotide diversity and GST, respectively. Sequences > 599 bp= 58.

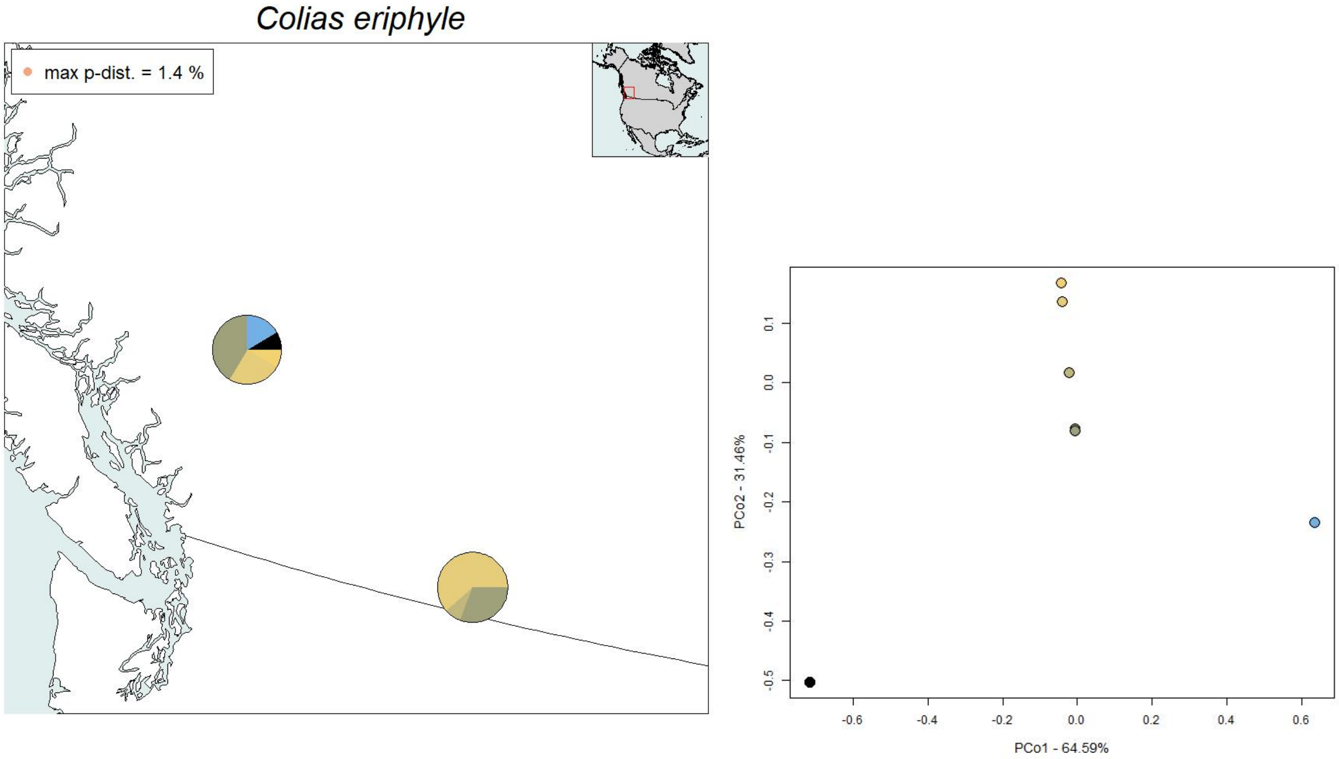

Figure 863 Map of *Colias eriphyle* showing the localities of the sequenced specimens (left). Nearby localities are grouped in pies. Colours match the bidimensional colour space of the PCoA projection (right) of max p-dists among sequences (dots). Sequences= 25; Hap obs.= 5; Hap asympt.= 6.9; Hap % obs.= 72.3%; GST= 0.056; DST= 0.0001; HD= 0.657; ND= 0.0025; max p-dist= 1.4%.

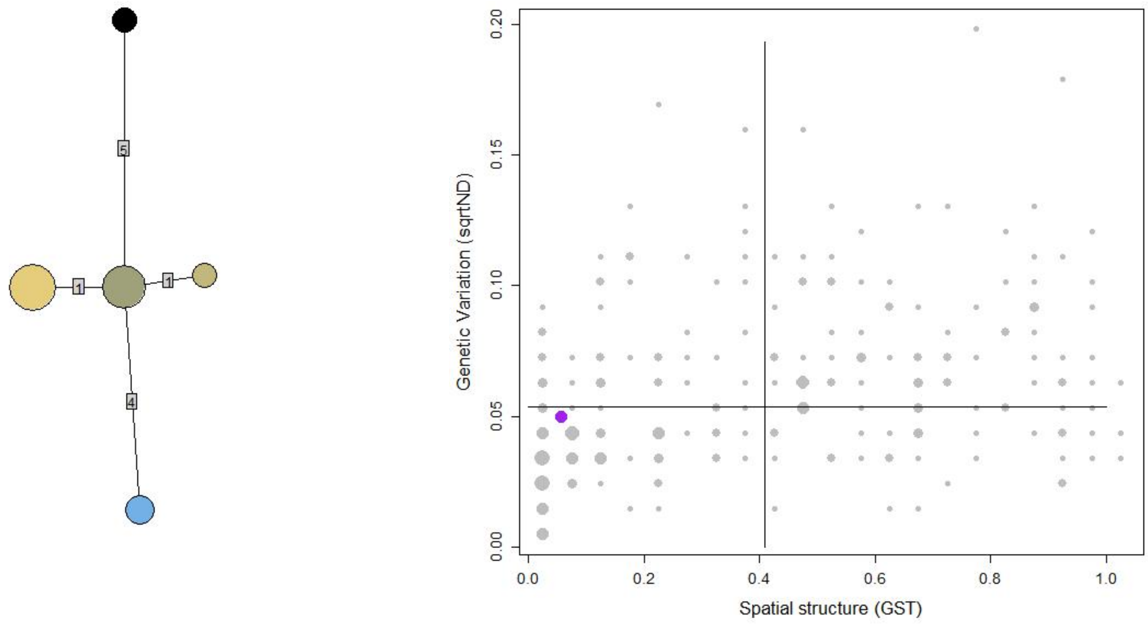

Figure 864: Haplotype network (left) of *Colias eriphyle* sequences > 599 bp with colours matching the PCoA colour space (above). The bubble plot for mt-DNA polymorphism (square root transformed nucleotide diversity) and spatial structure (GST) among all species in the atlas and values for *Colias eriphyle* (purple dot). The horizontal and vertical lines represent median values of nucleotide diversity and GST, respectively. Sequences > 599 bp= 25.

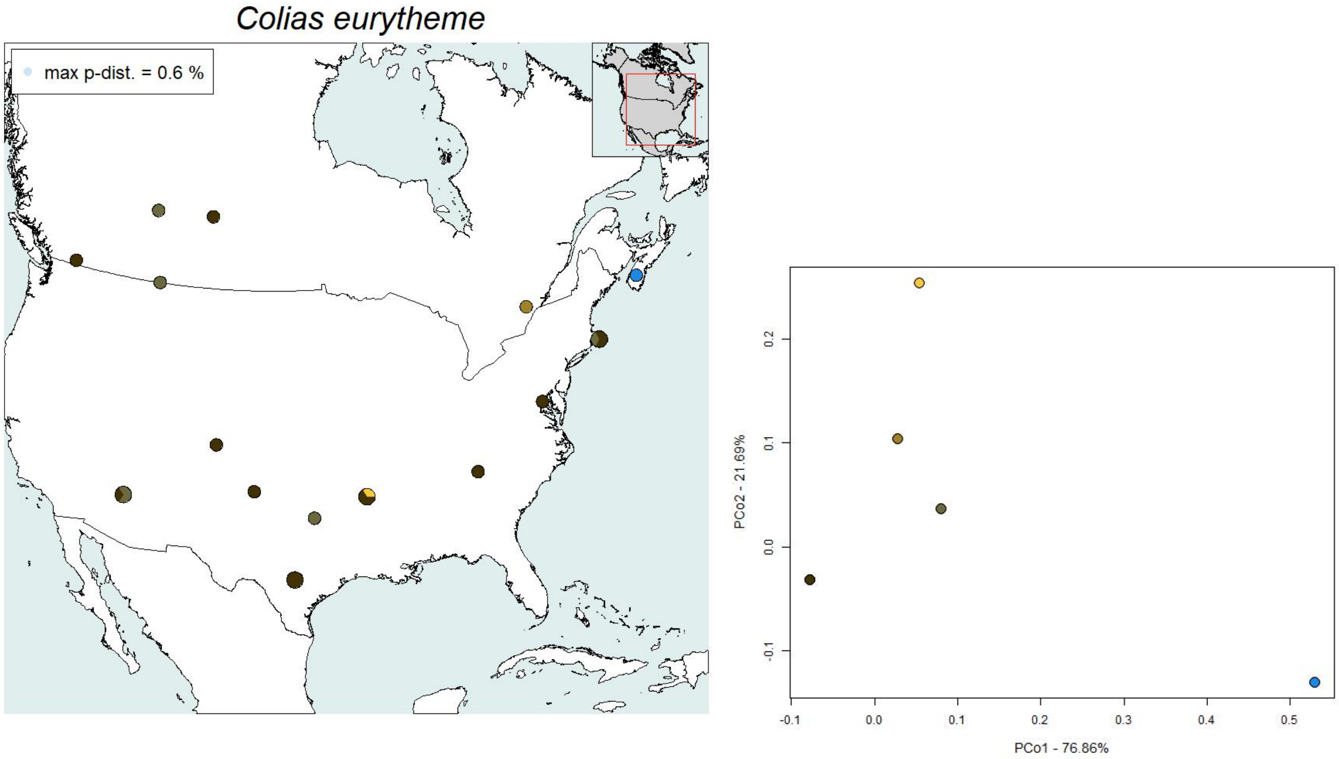

Figure 865 Map of *Colias eurytheme* showing the localities of the sequenced specimens (left). Nearby localities are grouped in pies. Colours match the bidimensional colour space of the PCoA projection (right) of max p-dists among sequences (dots). Sequences= 23; Hap obs.= 4; Hap asympt.= 5; Hap % obs.= 80.7%; GST= 0; DST= 0; HD= 0.557; ND= 0.0013; max p-dist= 0.6%.

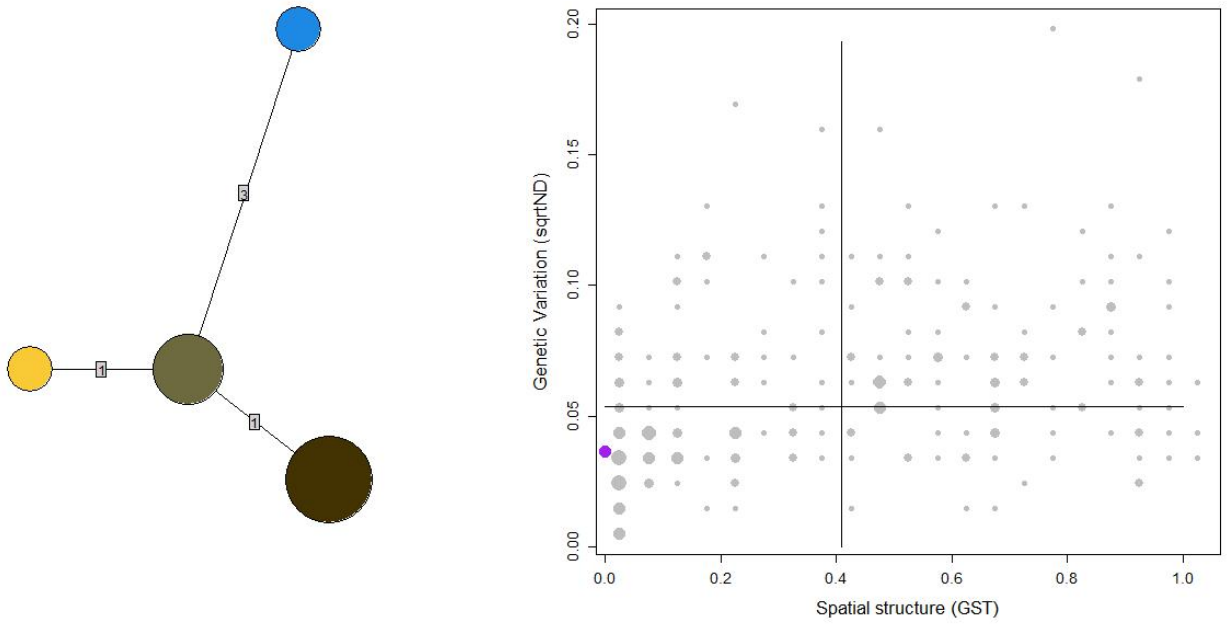

Figure 866: Haplotype network (left) of *Colias eurytheme* sequences > 599 bp with colours matching the PCoA colour space (above). The bubble plot for mt-DNA polymorphism (square root transformed nucleotide diversity) and spatial structure (GST) among all species in the atlas and values for *Colias eurytheme* (purple dot). The horizontal and vertical lines represent median values of nucleotide diversity and GST, respectively. Sequences > 599 bp= 22.

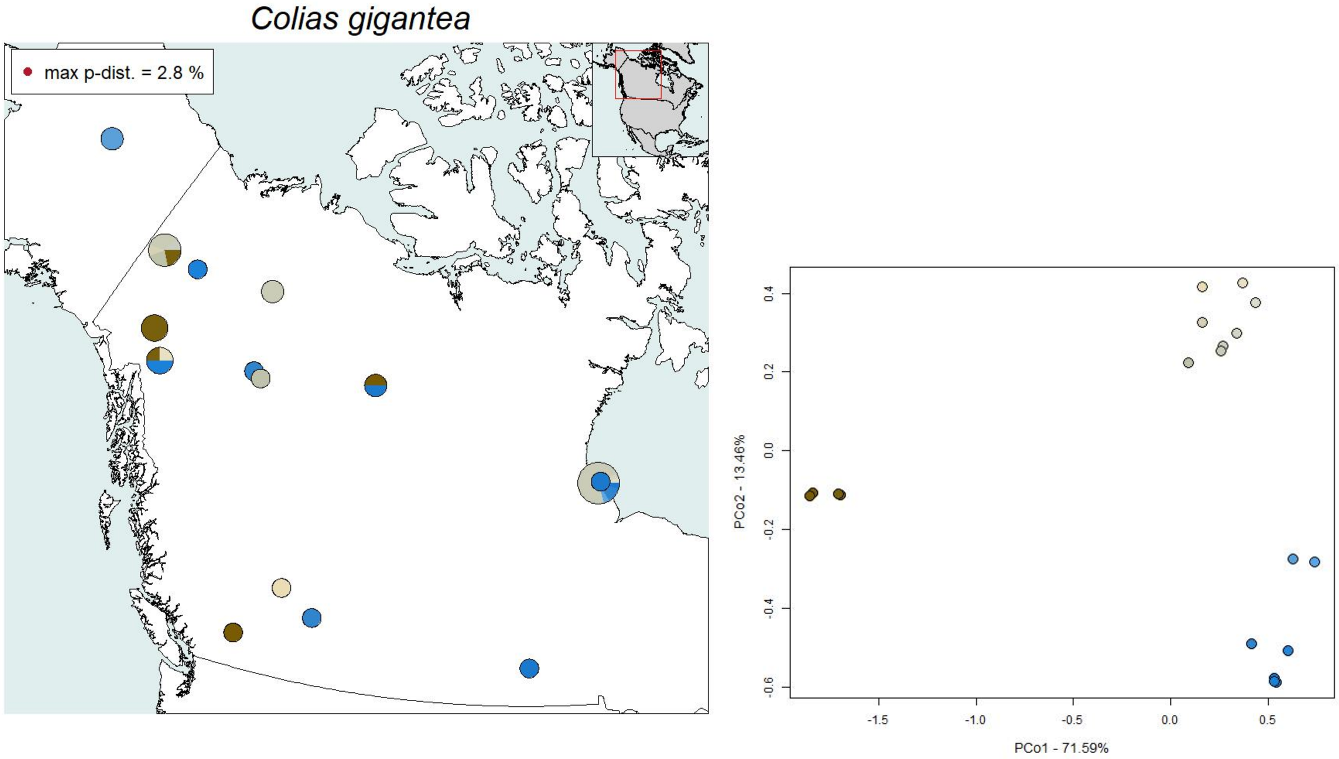

Figure 867 Map of *Colias gigantea* showing the localities of the sequenced specimens (left). Nearby localities are grouped in pies. Colours match the bidimensional colour space of the PCoA projection (right) of max p-dists among sequences (dots). Sequences= 55; Hap obs.= 16; Hap asympt.= 26.5; Hap % obs.= 60.4%; GST= 0.511; DST= 0.0046; HD= 0.803; ND= 0.0102; max p-dist= 2.8%.

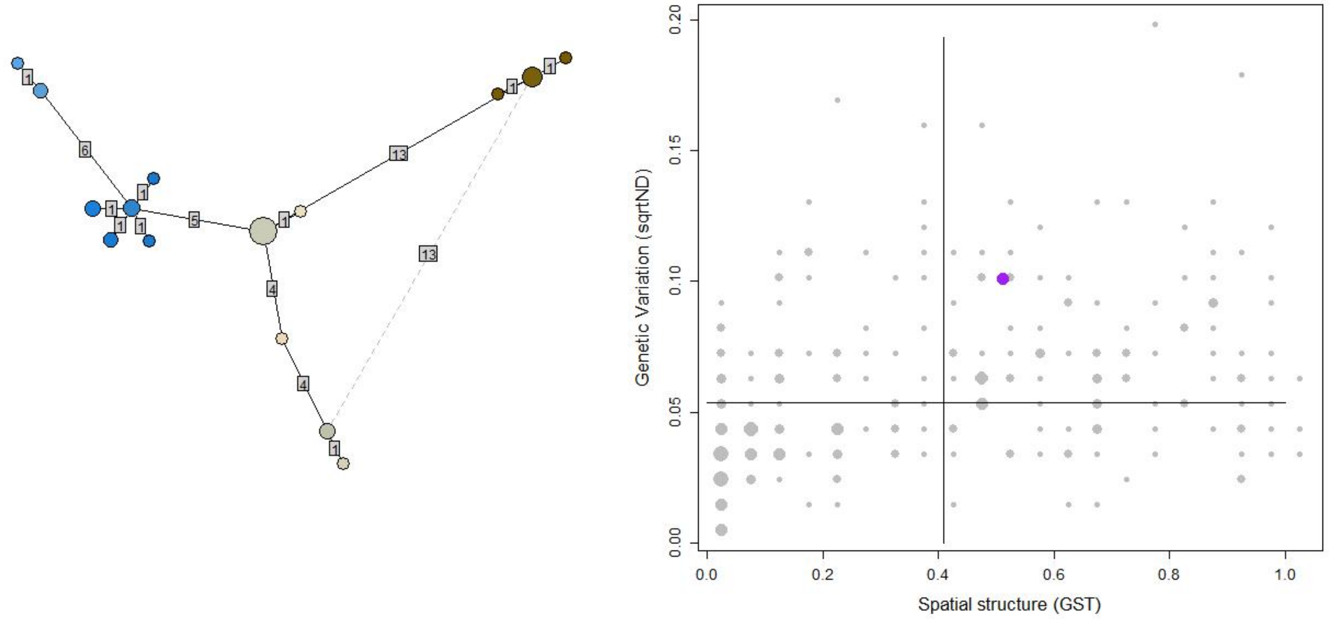

Figure 868: Haplotype network (left) of *Colias gigantea* sequences > 599 bp with colours matching the PCoA colour space (above). The bubble plot for mt-DNA polymorphism (square root transformed nucleotide diversity) and spatial structure (GST) among all species in the atlas and values for *Colias gigantea* (purple dot). The horizontal and vertical lines represent median values of nucleotide diversity and GST, respectively. Sequences > 599 bp= 53.

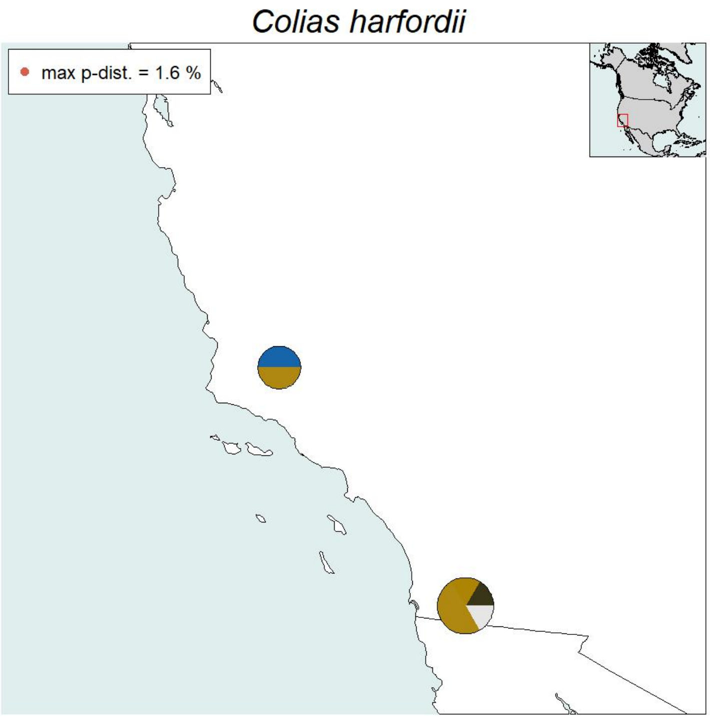

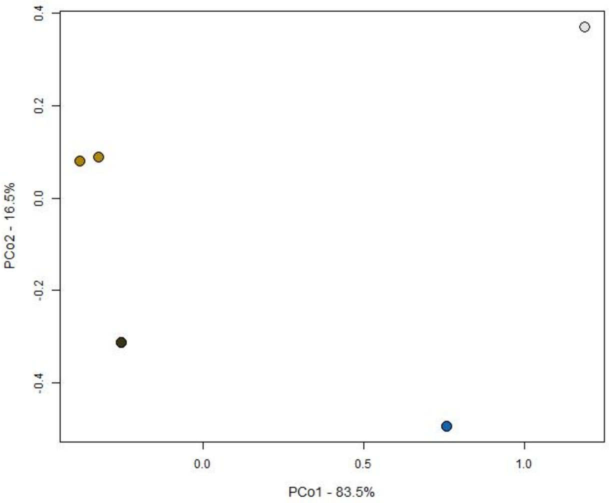

Figure 869 Map of *Colias harfordii* showing the localities of the sequenced specimens (left). Nearby localities are grouped in pies. Colours match the bidimensional colour space of the PCoA projection (right) of max p-dists among sequences (dots). Sequences= 8; Hap obs.= 3; Hap asympt.= NA; Hap % obs.= NA%; GST= NaN; DST= NaN; HD= NA; ND= NA; max p-dist= 1.6%.

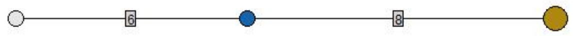

Figure 870: Haplotype network of *Colias harfordii*. Sequences > 599 bp= 7.

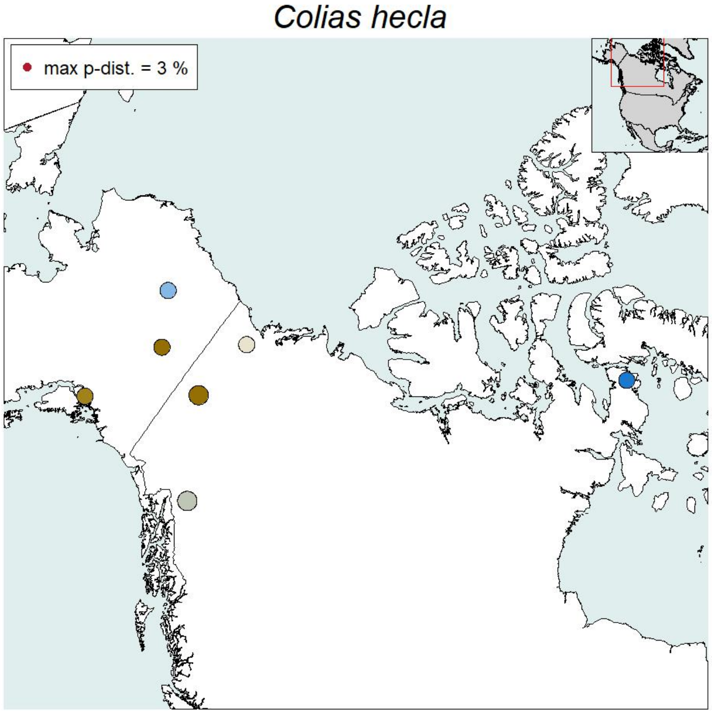

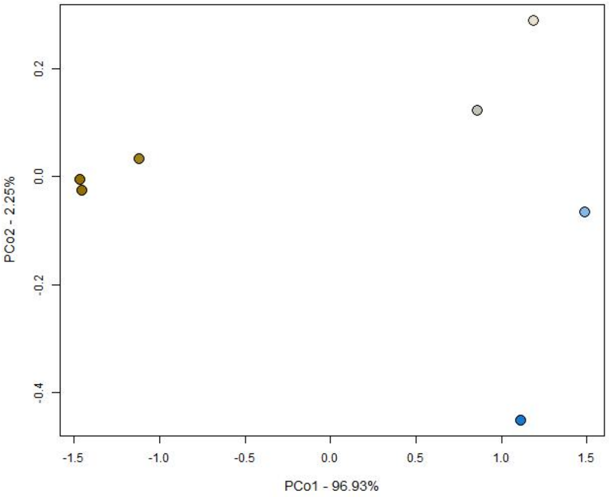

Figure 871 Map of *Colias hecla* showing the localities of the sequenced specimens (left). Nearby localities are grouped in pies. Colours match the bidimensional colour space of the PCoA projection (right) of max p-dists among sequences (dots). Sequences= 9; Hap obs.= 5; Hap asympt.= NA; Hap % obs.= NA%; GST= NaN; DST= NaN; HD= NA; ND= NA; max p-dist= 3%.

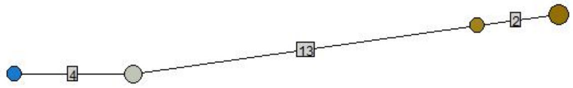

Figure 872: Haplotype network of *Colias hecla*. Sequences > 599 bp= 7.

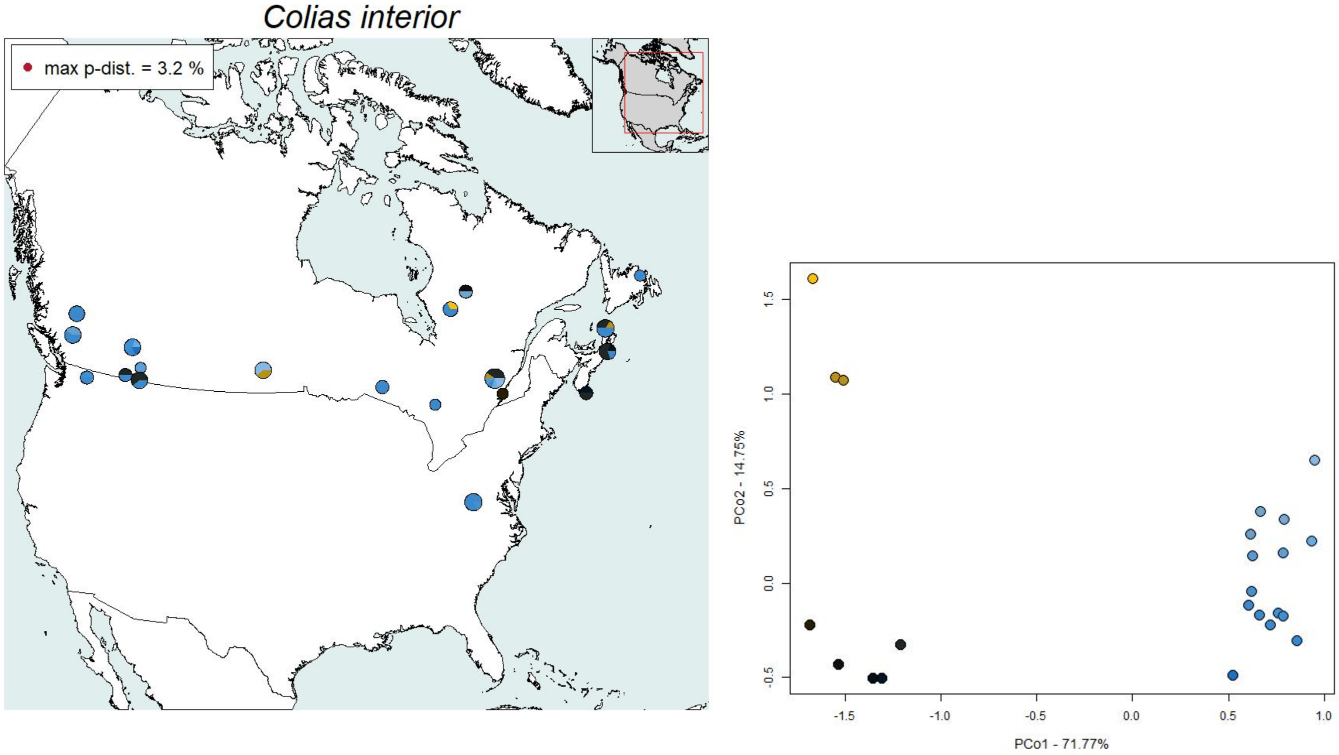

Figure 873 Map of *Colias interior* showing the localities of the sequenced specimens (left). Nearby localities are grouped in pies. Colours match the bidimensional colour space of the PCoA projection (right) of max p-dists among sequences (dots). Sequences= 68; Hap obs.= 16; Hap asympt.= 65.3; Hap % obs.= 24.5%; GST= 0.134; DST= 0.0017; HD= 0.76; ND= 0.0123; max p-dist= 3.2%.

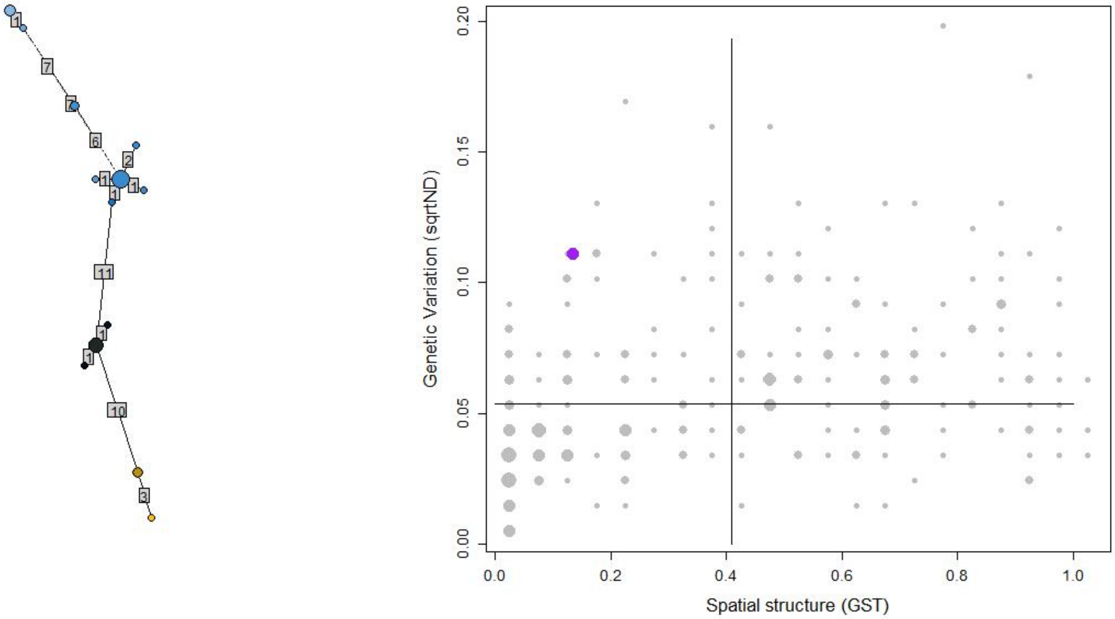

Figure 874: Haplotype network (left) of *Colias interior* sequences > 599 bp with colours matching the PCoA colour space (above). The bubble plot for mt-DNA polymorphism (square root transformed nucleotide diversity) and spatial structure (GST) among all species in the atlas and values for *Colias interior* (purple dot). The horizontal and vertical lines represent median values of nucleotide diversity and GST, respectively. Sequences > 599 bp= 64.

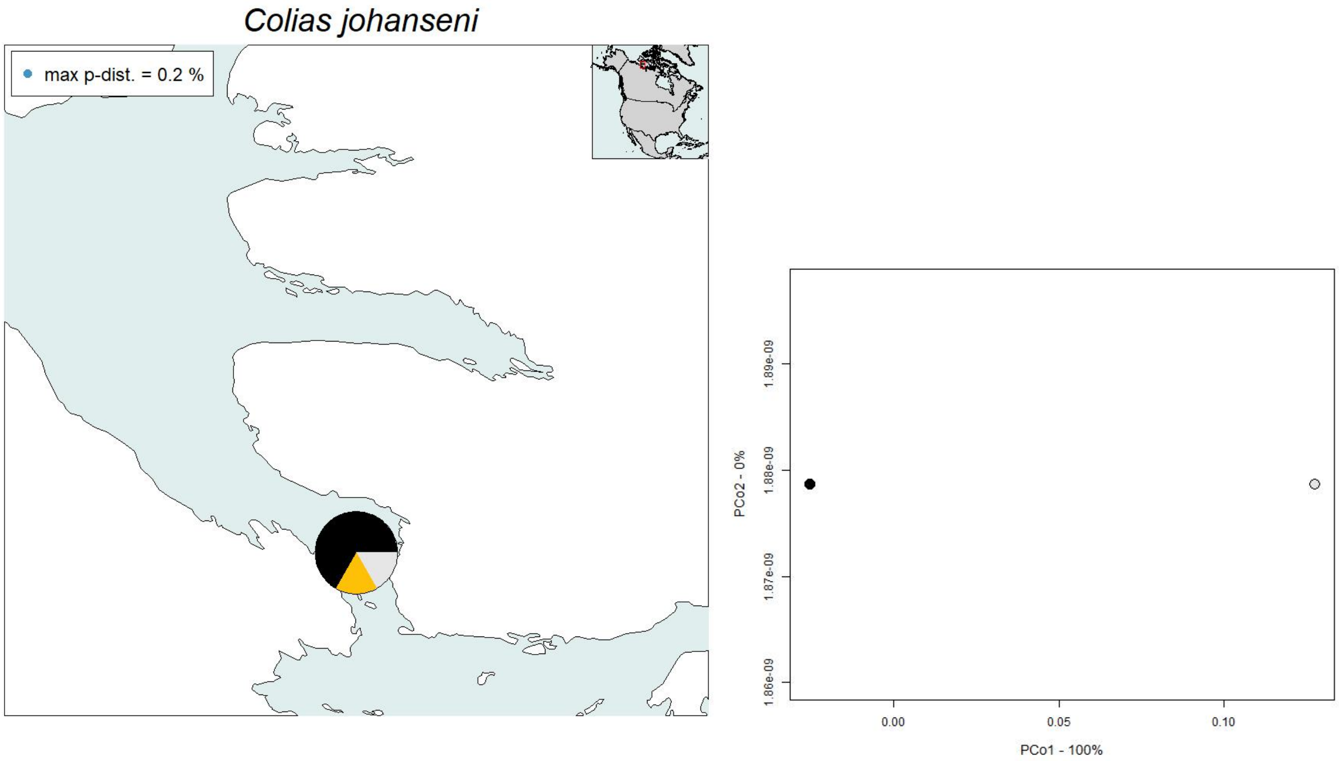

Figure 875 Map of *Colias johanseni* showing the localities of the sequenced specimens (left). Nearby localities are grouped in pies. Colours match the bidimensional colour space of the PCoA projection (right) of max p-dists among sequences (dots). Sequences= 6; Hap obs.= 2; Hap asympt.= NA; Hap % obs.= NA%; GST= NaN; DST= NaN; HD= NA; ND= NA; max p-dist= 0.2%.

Haplotype network analysis and bubble plot of *Colias johanseni* were not possible. Sequences > 599 bp = 6.

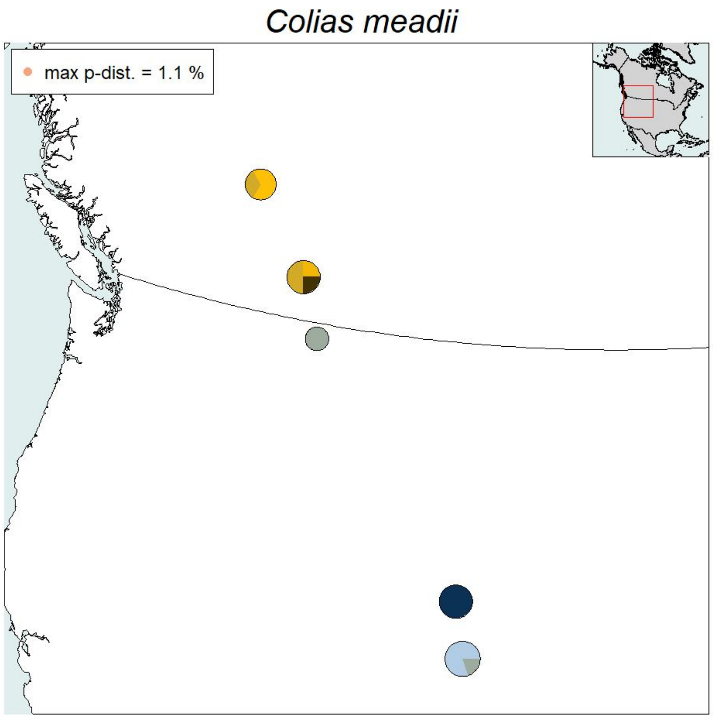

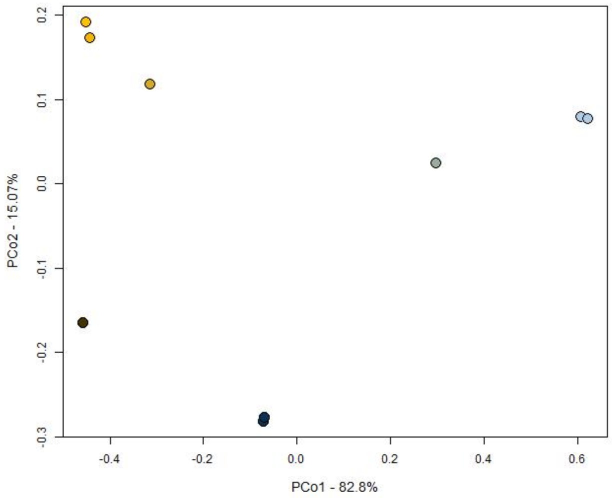

Figure 876 Map of *Colias meadii* showing the localities of the sequenced specimens (left). Nearby localities are grouped in pies. Colours match the bidimensional colour space of the PCoA projection (right) of max p-dists among sequences (dots). Sequences= 17; Hap obs.= 7; Hap asympt.= 7.9; Hap % obs.= 88.2%; GST= 0.741; DST= 0.0041; HD= 0.875; ND= 0.0054; max p-dist= 1.1%.

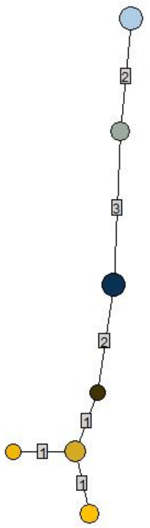

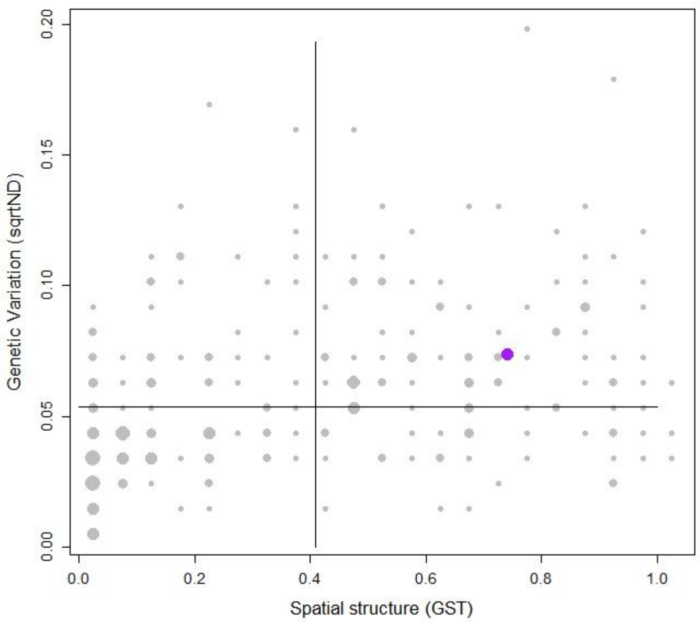

Figure 877: Haplotype network (left) of *Colias meadii* sequences > 599 bp with colours matching the PCoA colour space (above). The bubble plot for mt-DNA polymorphism (square root transformed nucleotide diversity) and spatial structure (GST) among all species in the atlas and values for *Colias meadii* (purple dot). The horizontal and vertical lines represent median values of nucleotide diversity and GST, respectively. Sequences > 599 bp= 17.

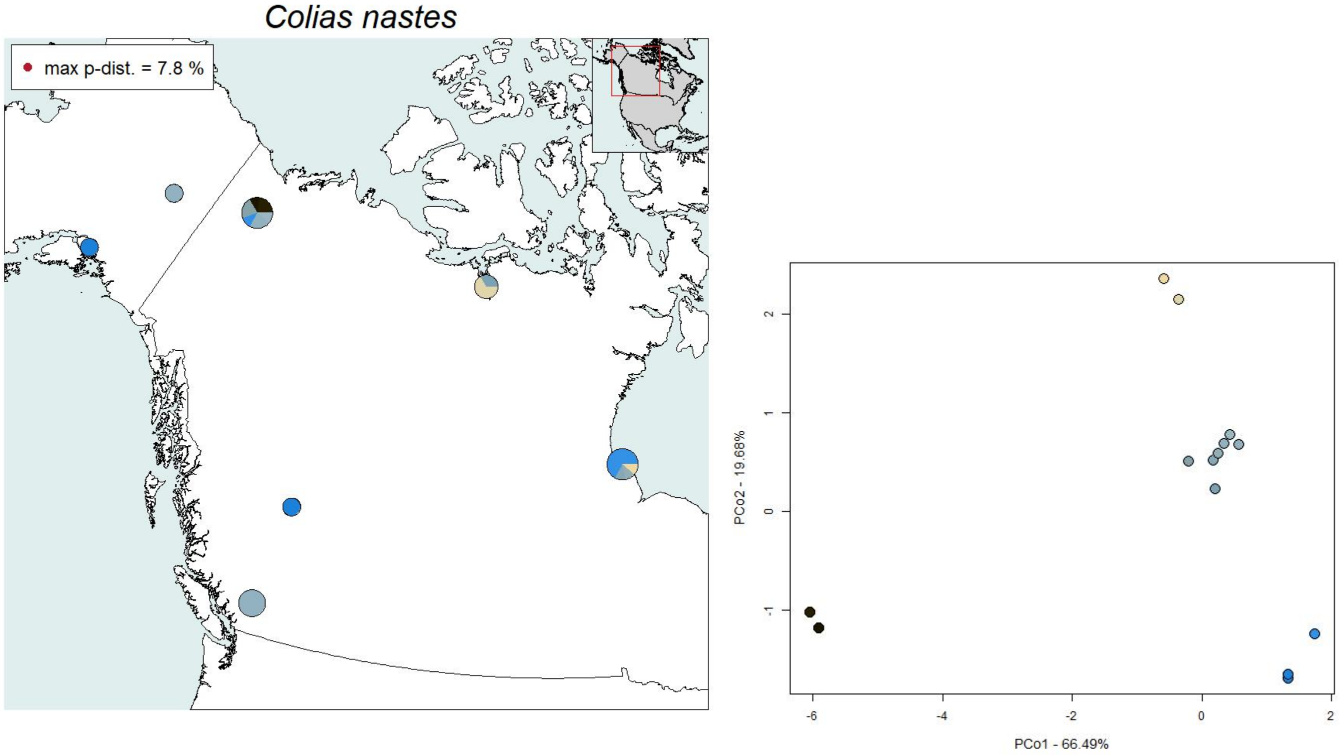

Figure 878 Map of *Colias nastes* showing the localities of the sequenced specimens (left). Nearby localities are grouped in pies. Colours match the bidimensional colour space of the PCoA projection (right) of max p-dists among sequences (dots). Sequences= 29; Hap obs.= 11; Hap asympt.= 13.6; Hap % obs.= 81%; GST= 0.237; DST= 0.0073; HD= 0.894; ND= 0.03; max p-dist= 7.8%.

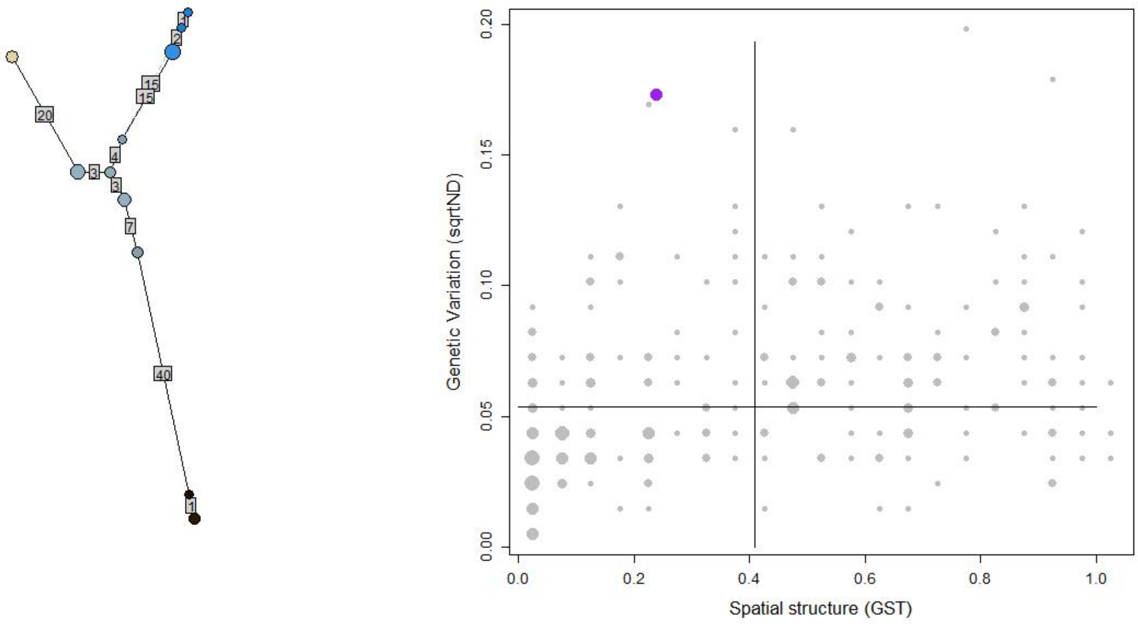

Figure 879: Haplotype network (left) of *Colias nastes* sequences > 599 bp with colours matching the PCoA colour space (above). The bubble plot for mt-DNA polymorphism (square root transformed nucleotide diversity) and spatial structure (GST) among all species in the atlas and values for *Colias nastes* (purple dot). The horizontal and vertical lines represent median values of nucleotide diversity and GST, respectively. Sequences > 599 bp= 29.

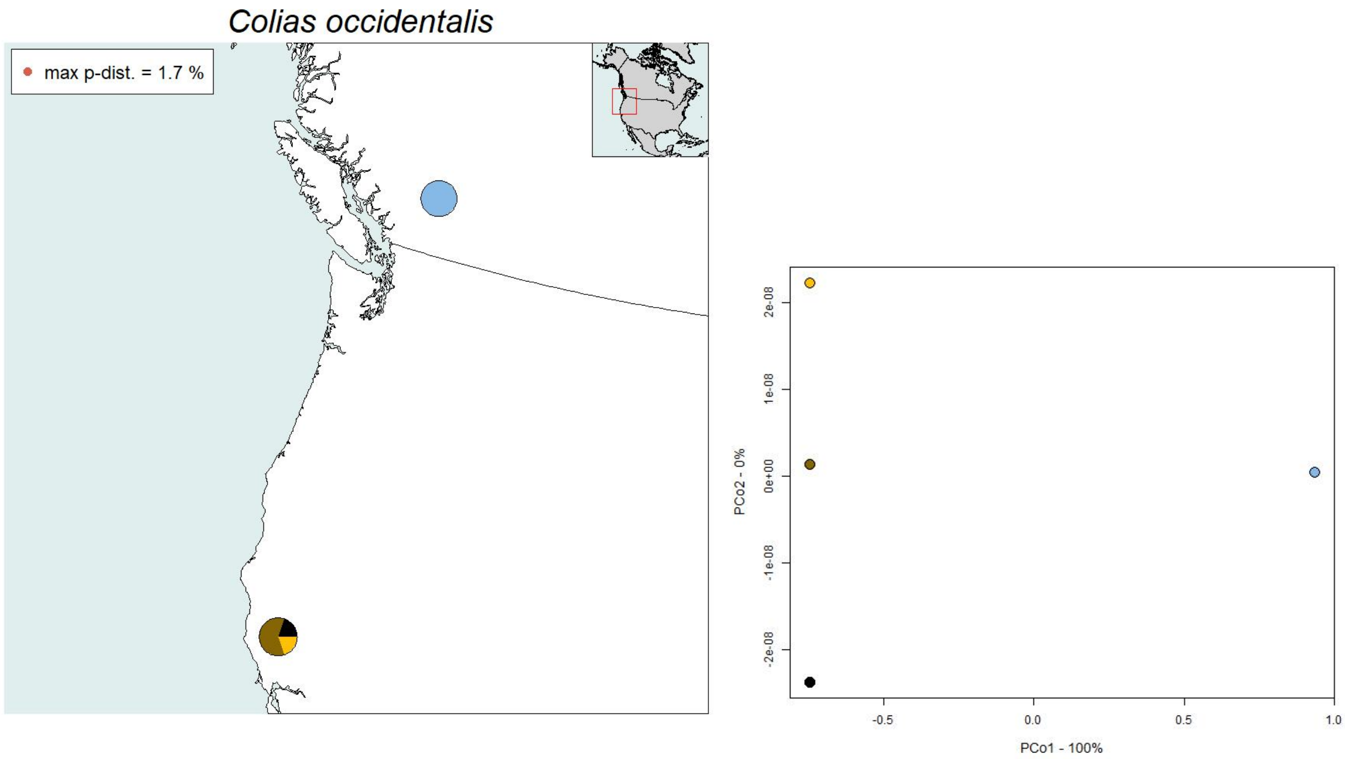

Figure 880 Map of *Colias occidentalis* showing the localities of the sequenced specimens (left). Nearby localities are grouped in pies. Colours match the bidimensional colour space of the PCoA projection (right) of max p-dists among sequences (dots). Sequences= 9; Hap obs.= 2; Hap asympt.= NA; Hap % obs.= NA%; GST= NaN; DST= NaN; HD= NA; ND= NA; max p-dist= 1.7%.

Haplotype network analysis and bubble plot of *Colias occidentalis* were not possible. Sequences > 599 bp = 9.

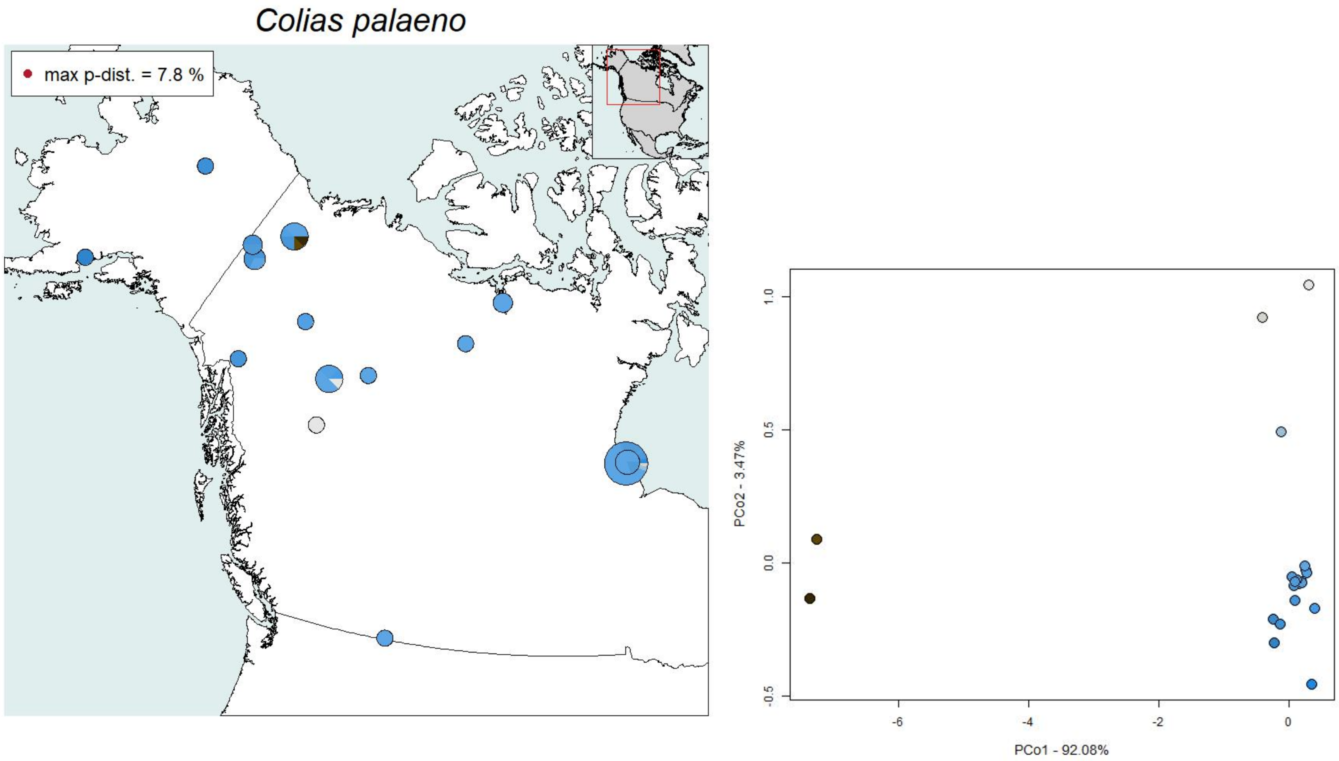

Figure 881 Map of *Colias palaeno* showing the localities of the sequenced specimens (left). Nearby localities are grouped in pies. Colours match the bidimensional colour space of the PCoA projection (right) of max p-dists among sequences (dots). Sequences= 86; Hap obs.= 13; Hap asympt.= 15.5; Hap % obs.= 84%; GST= 0.613; DST= 0.0033; HD= 0.564; ND= 0.0052; max p-dist= 7.8%.

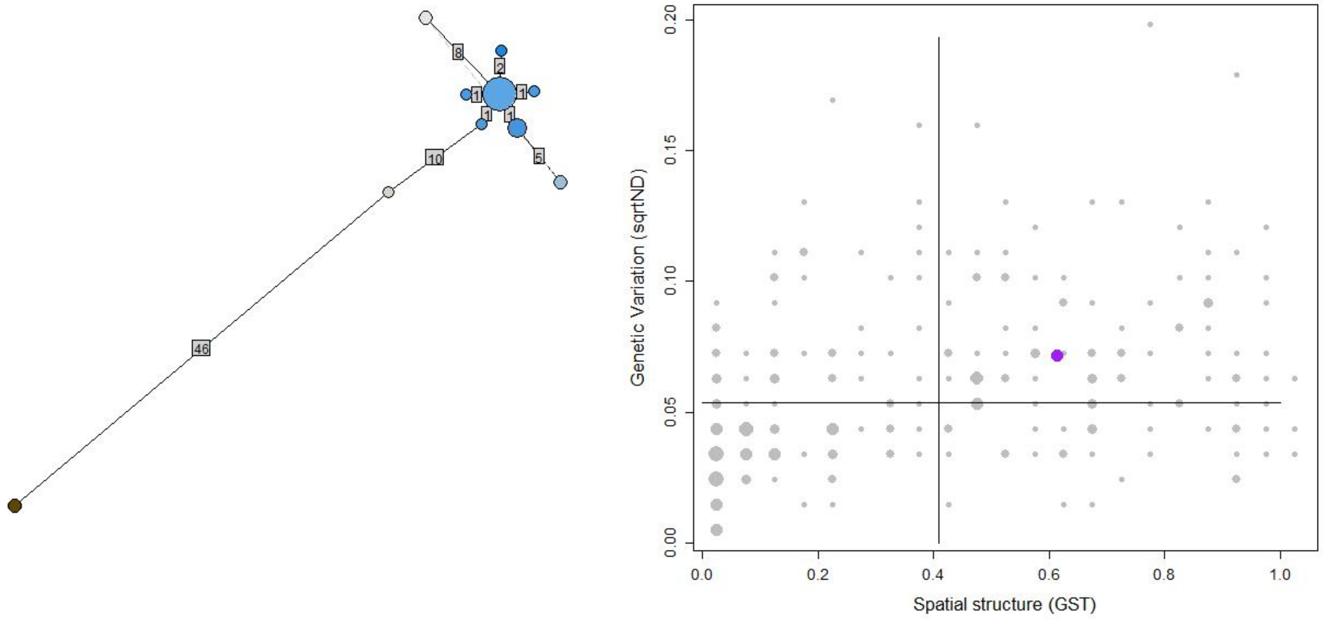

Figure 882: Haplotype network (left) of *Colias palaeno* sequences > 599 bp with colours matching the PCoA colour space (above). The bubble plot for mt-DNA polymorphism (square root transformed nucleotide diversity) and spatial structure (GST) among all species in the atlas and values for *Colias palaeno* (purple dot). The horizontal and vertical lines represent median values of nucleotide diversity and GST, respectively. Sequences > 599 bp= 84.

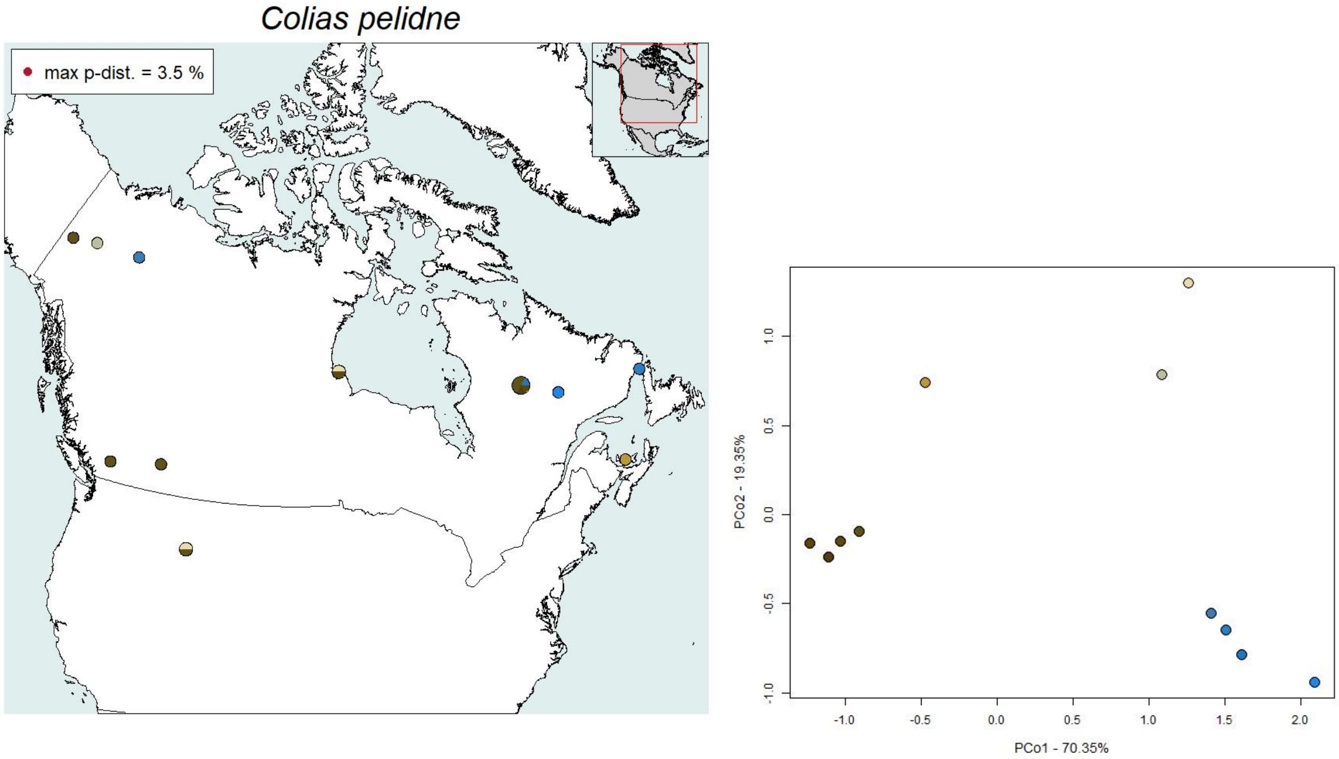

Figure 883 Map of *Colias pelidne* showing the localities of the sequenced specimens (left). Nearby localities are grouped in pies. Colours match the bidimensional colour space of the PCoA projection (right) of max p-dists among sequences (dots). Sequences= 18; Hap obs.= 10; Hap asympt.= 40.2; Hap % obs.= 24.9%; GST= NaN; DST= NaN; HD= 0.81; ND= 0.0165; max p-dist= 3.5%.

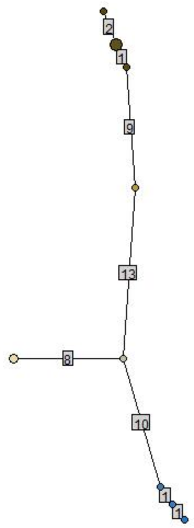

Figure 884: Haplotype network of *Colias pelidne*. Sequences > 599 bp= 16.

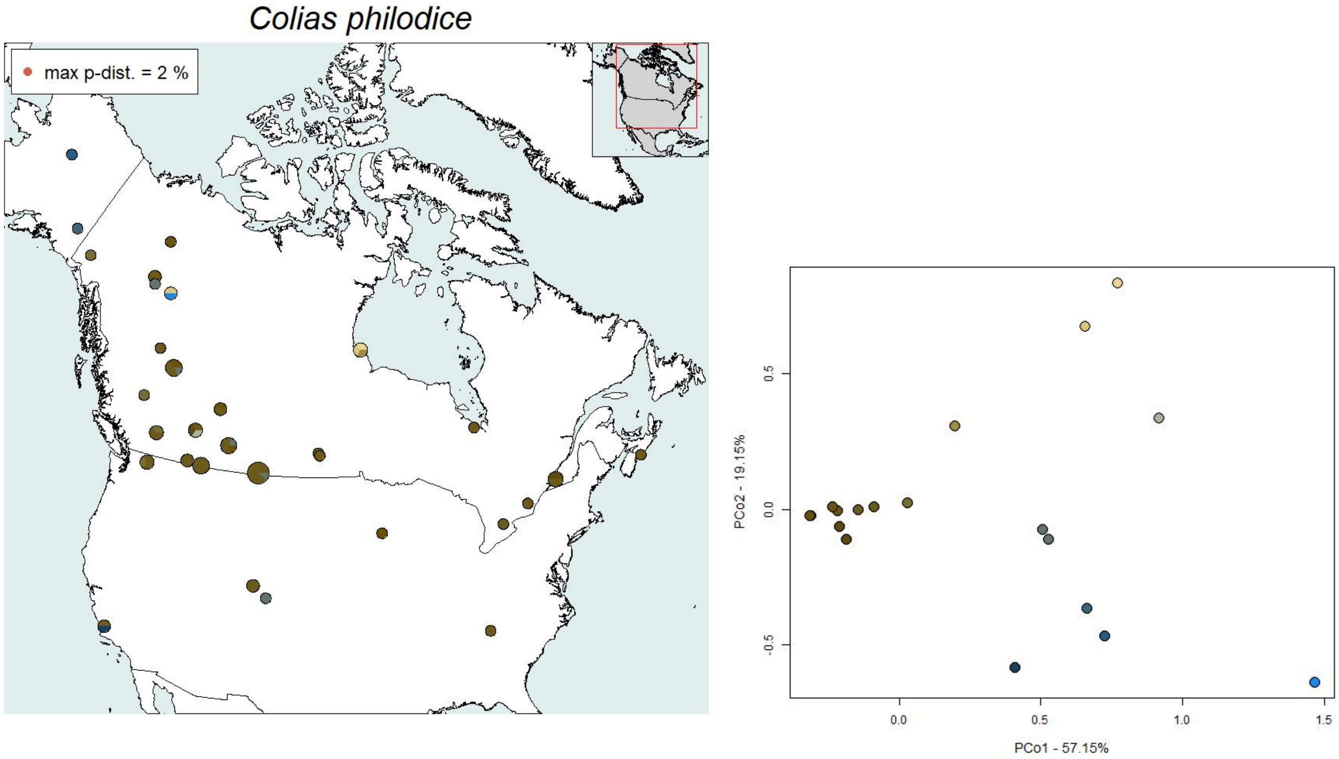

Figure 885 Map of *Colias philodice* showing the localities of the sequenced specimens (left). Nearby localities are grouped in pies. Colours match the bidimensional colour space of the PCoA projection (right) of max p-dists among sequences (dots). Sequences= 78; Hap obs.= 21; Hap asympt.= 53.2; Hap % obs.= 39.4%; GST= 0.456; DST= 0.0014; HD= 0.648; ND= 0.0039; max p-dist= 2%.

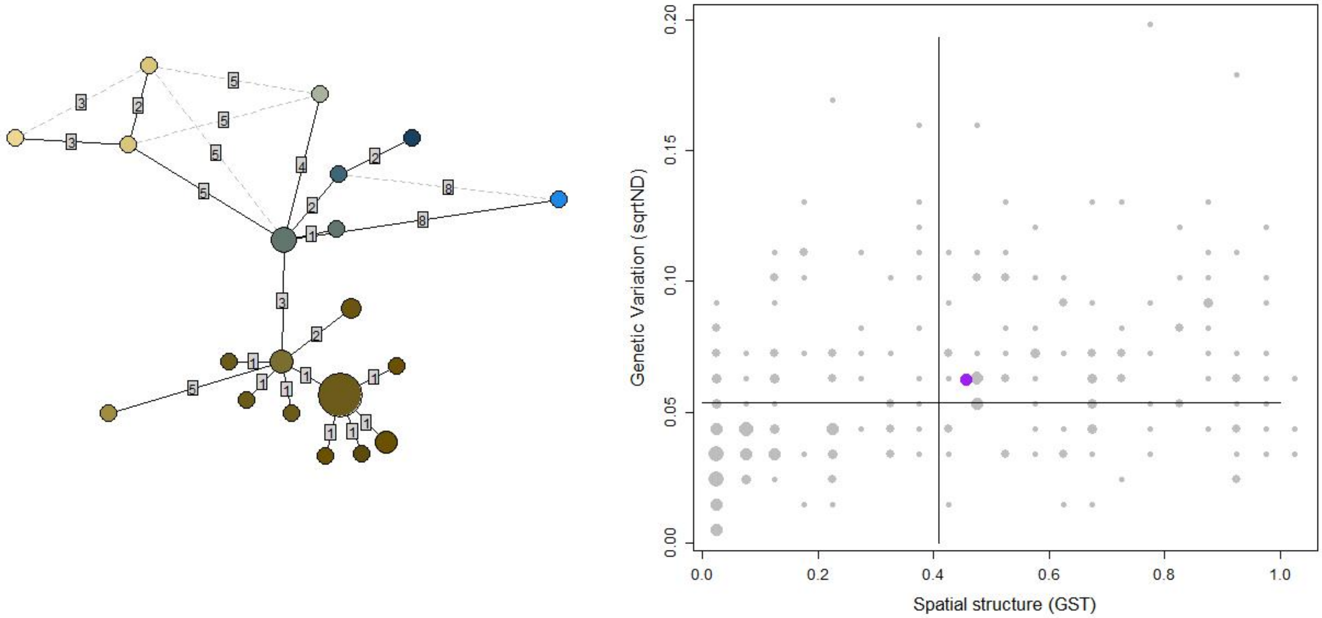

Figure 886: Haplotype network (left) of *Colias philodice* sequences > 599 bp with colours matching the PCoA colour space (above). The bubble plot for mt-DNA polymorphism (square root transformed nucleotide diversity) and spatial structure (GST) among all species in the atlas and values for *Colias philodice* (purple dot). The horizontal and vertical lines represent median values of nucleotide diversity and GST, respectively. Sequences > 599 bp= 75.

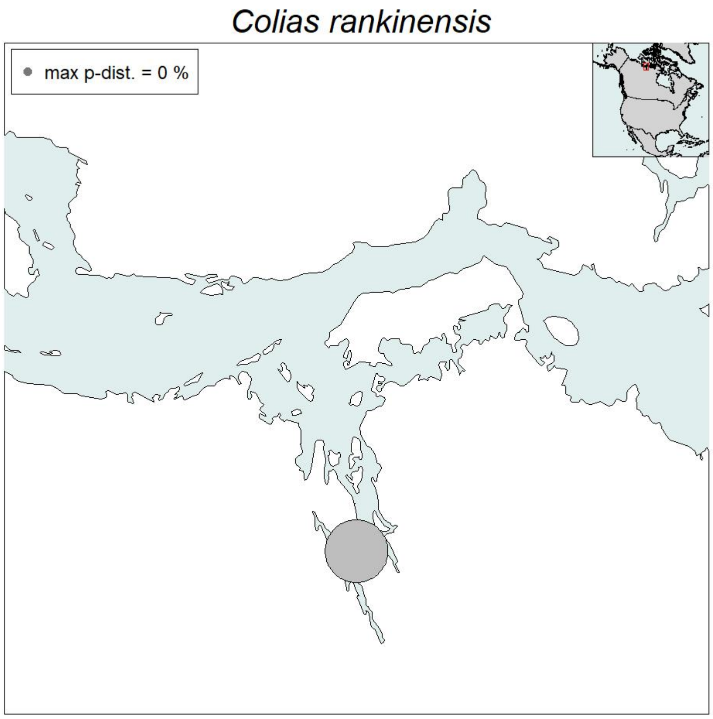

Figure 887: Map of *Colias rankinensis* showing the localities of the sequenced specimens. Nearby localities are grouped in pies. Due to the presence of a single haplotype PCoA projection was not done and a single grey colour was plotted on the map. Sequences= 2; Hap obs.= 1; Hap asympt.= NA; Hap % obs.= NA%; GST= NaN; DST= NaN; HD= NA; ND= NA; max p-dist= 0%.

Haplotype network analysis and bubble plot of *Colias rankinensis* were not possible. Sequences > 599 bp = 2.

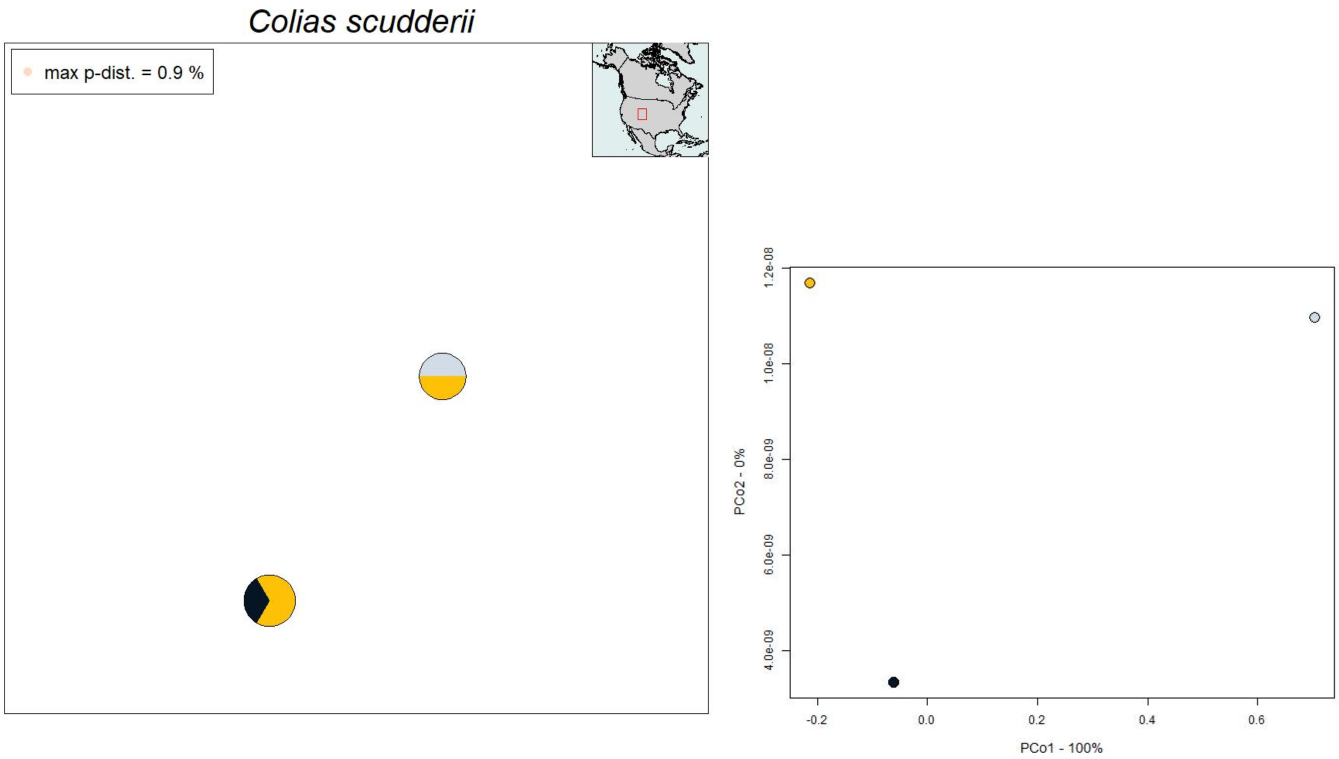

Figure 888 Map of *Colias scudderii* showing the localities of the sequenced specimens (left). Nearby localities are grouped in pies. Colours match the bidimensional colour space of the PCoA projection (right) of max p-dists among sequences (dots). Sequences= 5; Hap obs.= 3; Hap asympt.= NA; Hap % obs.= NA%; GST= NaN; DST= NaN; HD= NA; ND= NA; max p-dist= 0.9%.

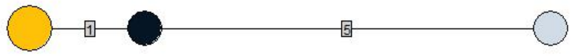

Figure 889: Haplotype network of *Colias scudderii*. Sequences > 599 bp= 5.

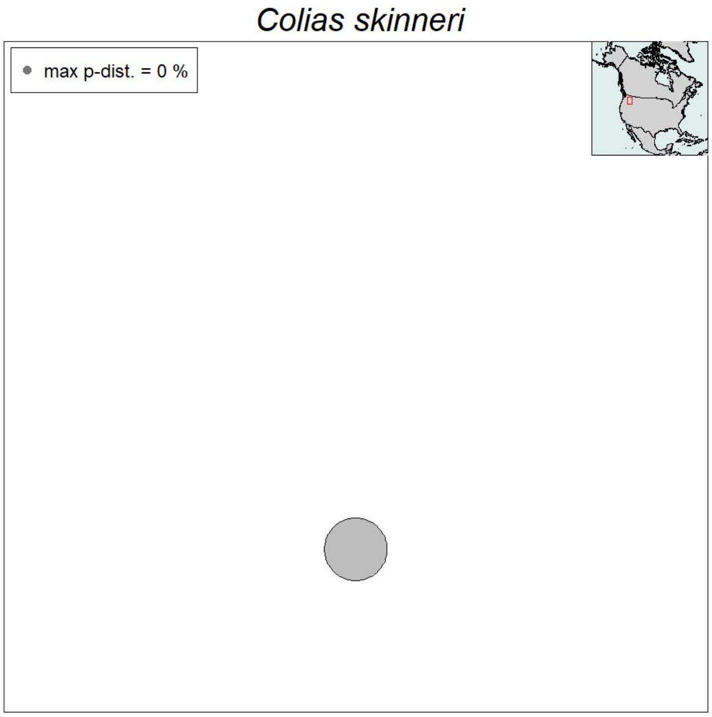

Figure 890: Map of *Colias skinneri* showing the localities of the sequenced specimens. Nearby localities are grouped in pies. Due to the presence of a single haplotype PCoA projection was not done and a single grey colour was plotted on the map. Sequences= 2; Hap obs.= 1; Hap asympt.= NA; Hap % obs.= NA%; GST= NaN; DST= NaN; HD= NA; ND= NA; max p-dist= 0%.

Haplotype network analysis and bubble plot of *Colias skinneri* were not possible. Sequences > 599 bp = 2.

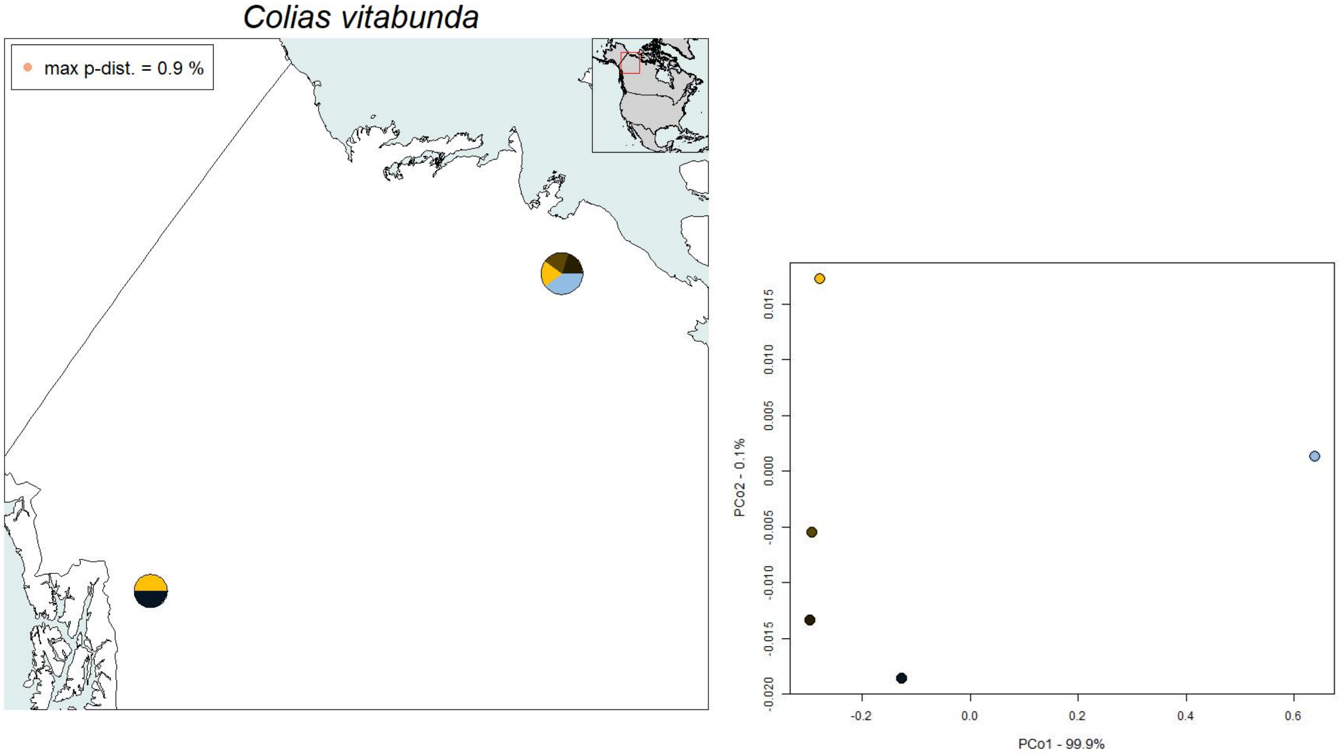

Figure 891 Map of *Colias vitabunda* showing the localities of the sequenced specimens (left). Nearby localities are grouped in pies. Colours match the bidimensional colour space of the PCoA projection (right) of max p-dists among sequences (dots). Sequences= 7; Hap obs.= 3; Hap asympt.= NA; Hap % obs.= NA%; GST= NaN; DST= NaN; HD= NA; ND= NA; max p-dist= 0.9%.

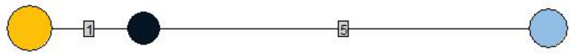

Figure 892: Haplotype network of *Colias vitabunda*. Sequences > 599 bp= 7.

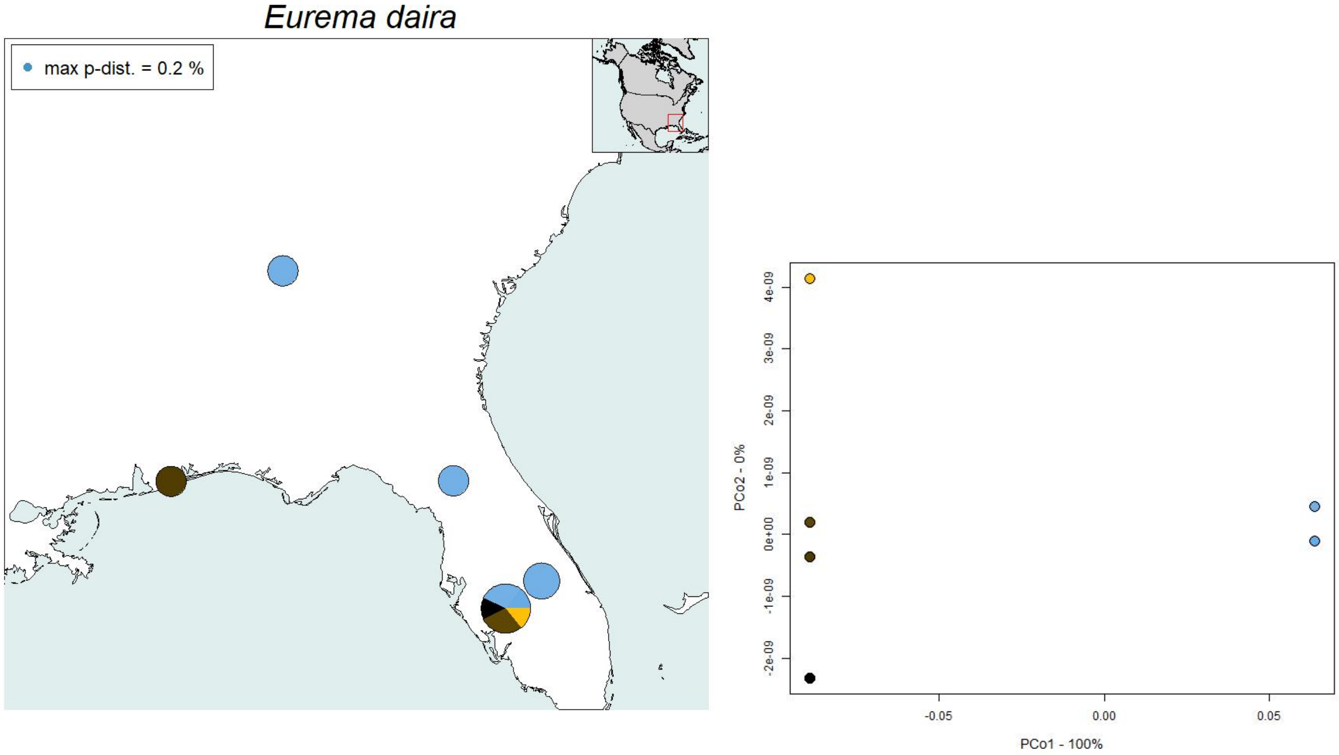

Figure 893 Map of *Eurema daira* showing the localities of the sequenced specimens (left). Nearby localities are grouped in pies. Colours match the bidimensional colour space of the PCoA projection (right) of max p-dists among sequences (dots). Sequences= 12; Hap obs.= 2; Hap asympt.= 2; Hap % obs.= 100%; GST= NaN; DST= NaN; HD= 0.53; ND= 0.0008; max p-dist= 0.2%.

Haplotype network analysis and bubble plot of *Eurema daira* were not possible. Sequences > 599 bp = 12.

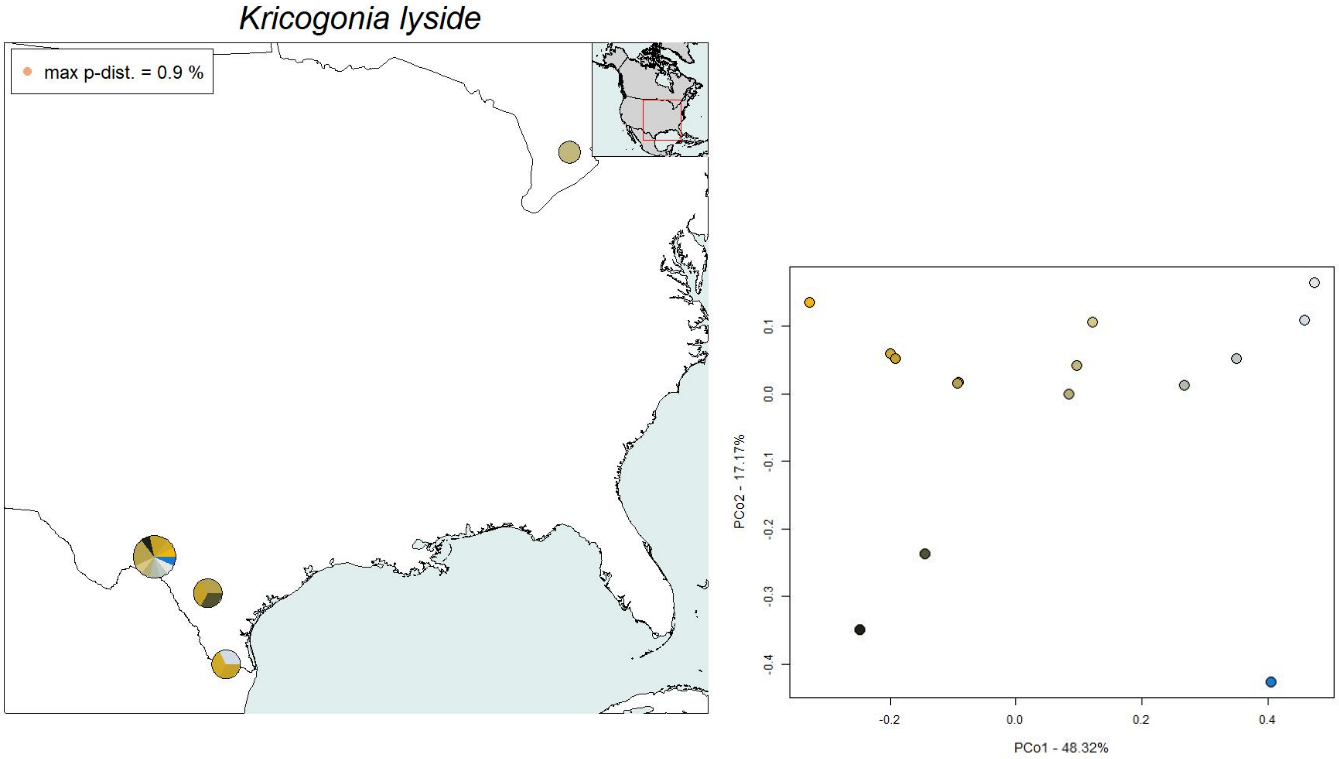

Figure 894 Map of *Kricogonia lyside* showing the localities of the sequenced specimens (left). Nearby localities are grouped in pies. Colours match the bidimensional colour space of the PCoA projection (right) of max p-dists among sequences (dots). Sequences= 21; Hap obs.= 17; Hap asympt.= 124.1; Hap % obs.= 13.7%; GST= 0.001; DST= 0; HD= 0.967; ND= 0.0042; max p-dist= 0.9%.

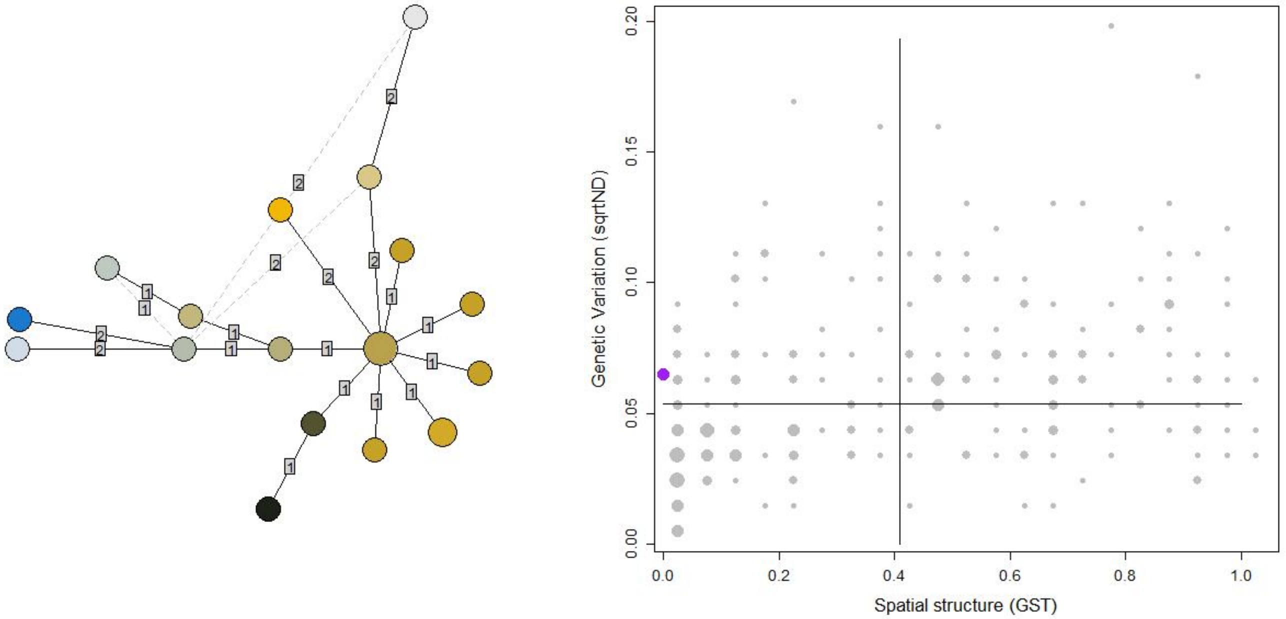

Figure 895: Haplotype network (left) of *Kricogonia lyside* sequences > 599 bp with colours matching the PCoA colour space (above). The bubble plot for mt-DNA polymorphism (square root transformed nucleotide diversity) and spatial structure (GST) among all species in the atlas and values for *Kricogonia lyside* (purple dot). The horizontal and vertical lines represent median values of nucleotide diversity and GST, respectively. Sequences > 599 bp= 21.

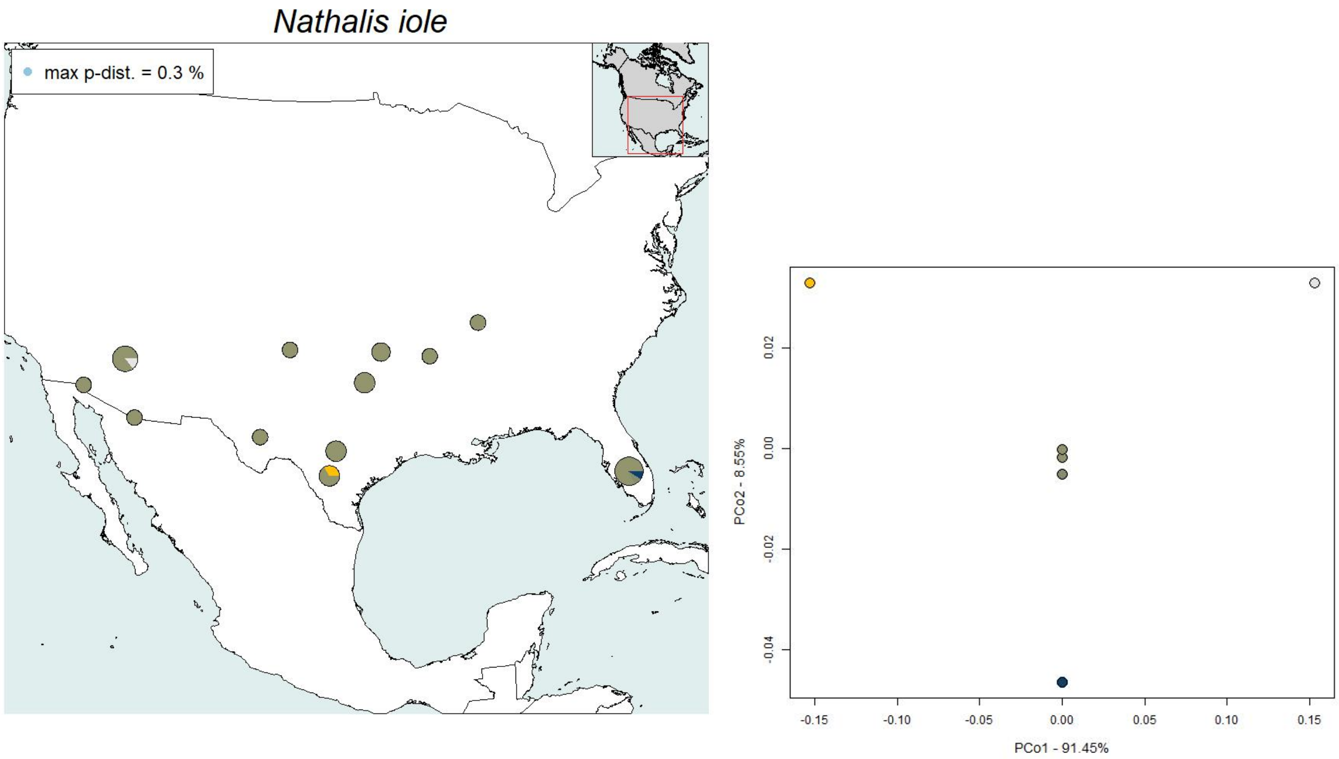

Figure 896 Map of *Nathalis iole* showing the localities of the sequenced specimens (left). Nearby localities are grouped in pies. Colours match the bidimensional colour space of the PCoA projection (right) of max p-dists among sequences (dots). Sequences= 35; Hap obs.= 3; Hap asympt.= 4; Hap % obs.= 75.5%; GST= 0.445; DST= 0.0001; HD= 0.113; ND= 0.0002; max p-dist= 0.3%.

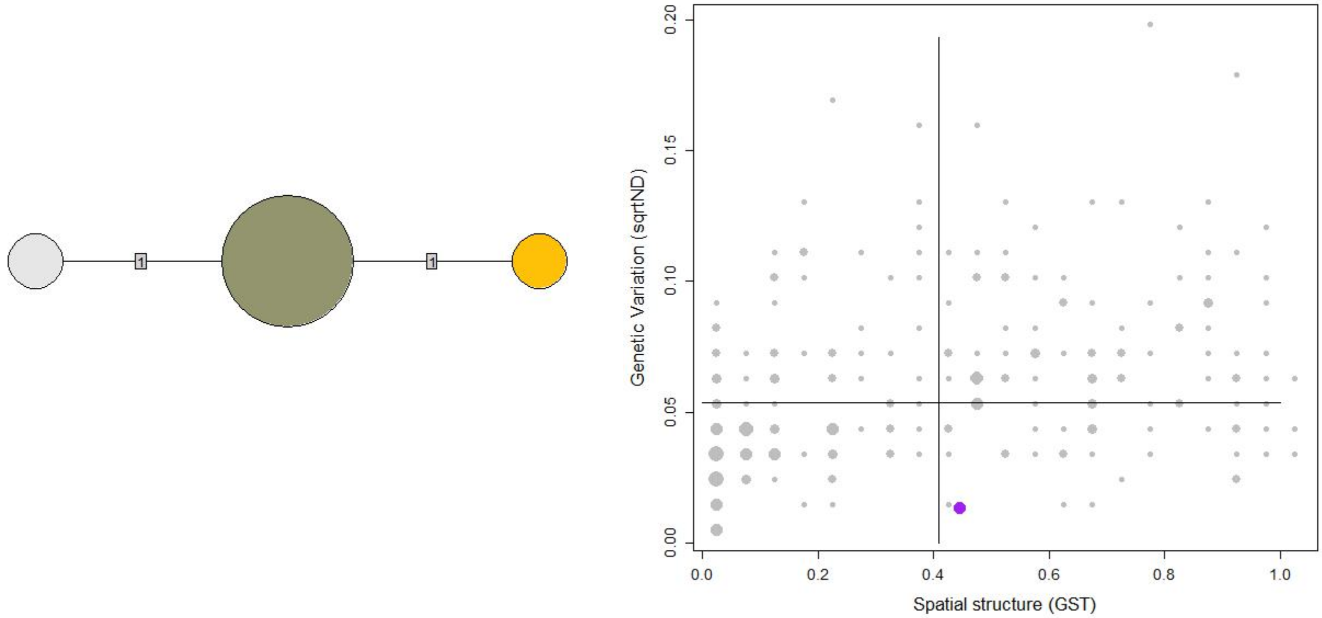

Figure 897: Haplotype network (left) of *Nathalis iole* sequences > 599 bp with colours matching the PCoA colour space (above). The bubble plot for mt-DNA polymorphism (square root transformed nucleotide diversity) and spatial structure (GST) among all species in the atlas and values for *Nathalis iole* (purple dot). The horizontal and vertical lines represent median values of nucleotide diversity and GST, respectively. Sequences > 599 bp= 34.

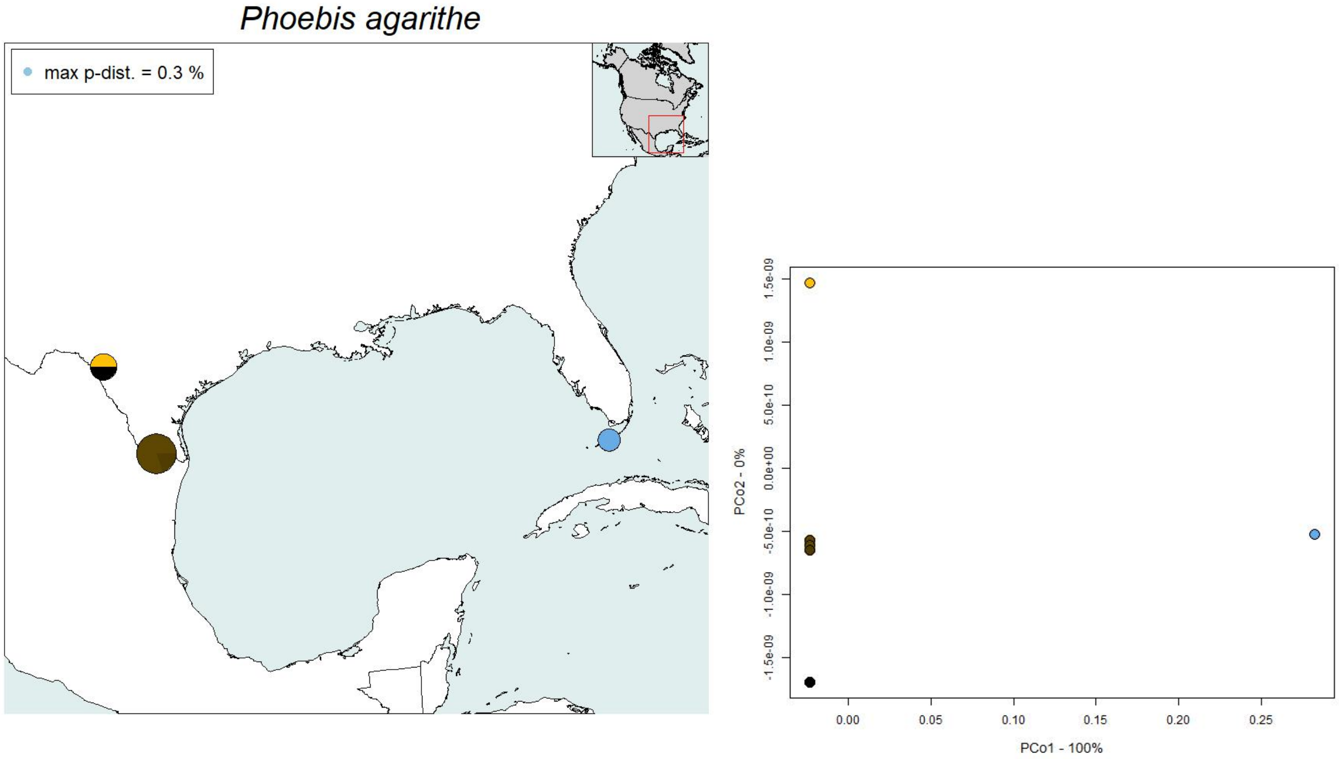

Figure 898 Map of *Phoebis agarithe* showing the localities of the sequenced specimens (left). Nearby localities are grouped in pies. Colours match the bidimensional colour space of the PCoA projection (right) of max p-dists among sequences (dots). Sequences= 13; Hap obs.= 2; Hap asympt.= 2; Hap % obs.= 100%; GST= NaN; DST= NaN; HD= 0.154; ND= 0.0005; max p-dist= 0.3%.

Haplotype network analysis and bubble plot of *Phoebis agarithe* were not possible. Sequences > 599 bp = 13.

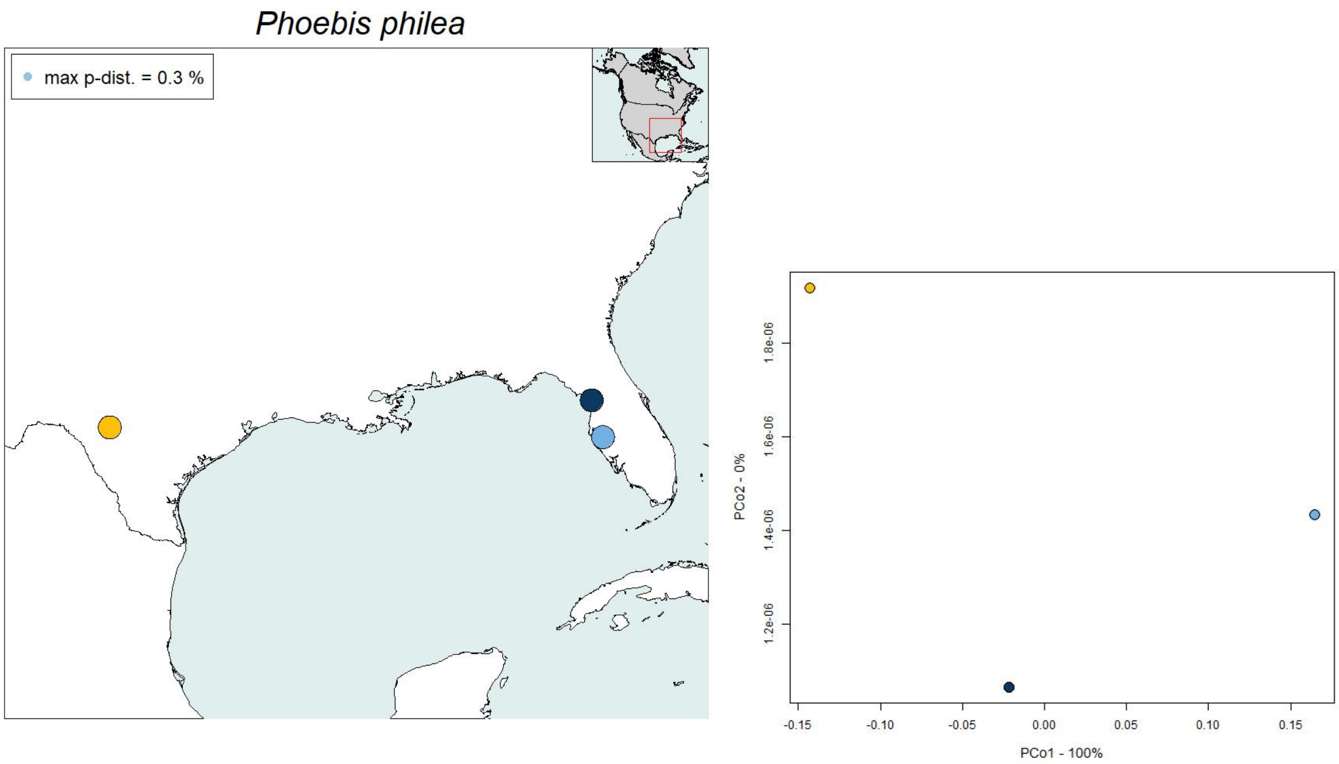

Figure 899 Map of *Phoebis philea* showing the localities of the sequenced specimens (left). Nearby localities are grouped in pies. Colours match the bidimensional colour space of the PCoA projection (right) of max p-dists among sequences (dots). Sequences= 3; Hap obs.= 2; Hap asympt.= NA; Hap % obs.= NA%; GST= NaN; DST= NaN; HD= NA; ND= NA; max p-dist= 0.3%.

Haplotype network analysis and bubble plot of *Phoebis philea* were not possible. Sequences > 599 bp = 3.

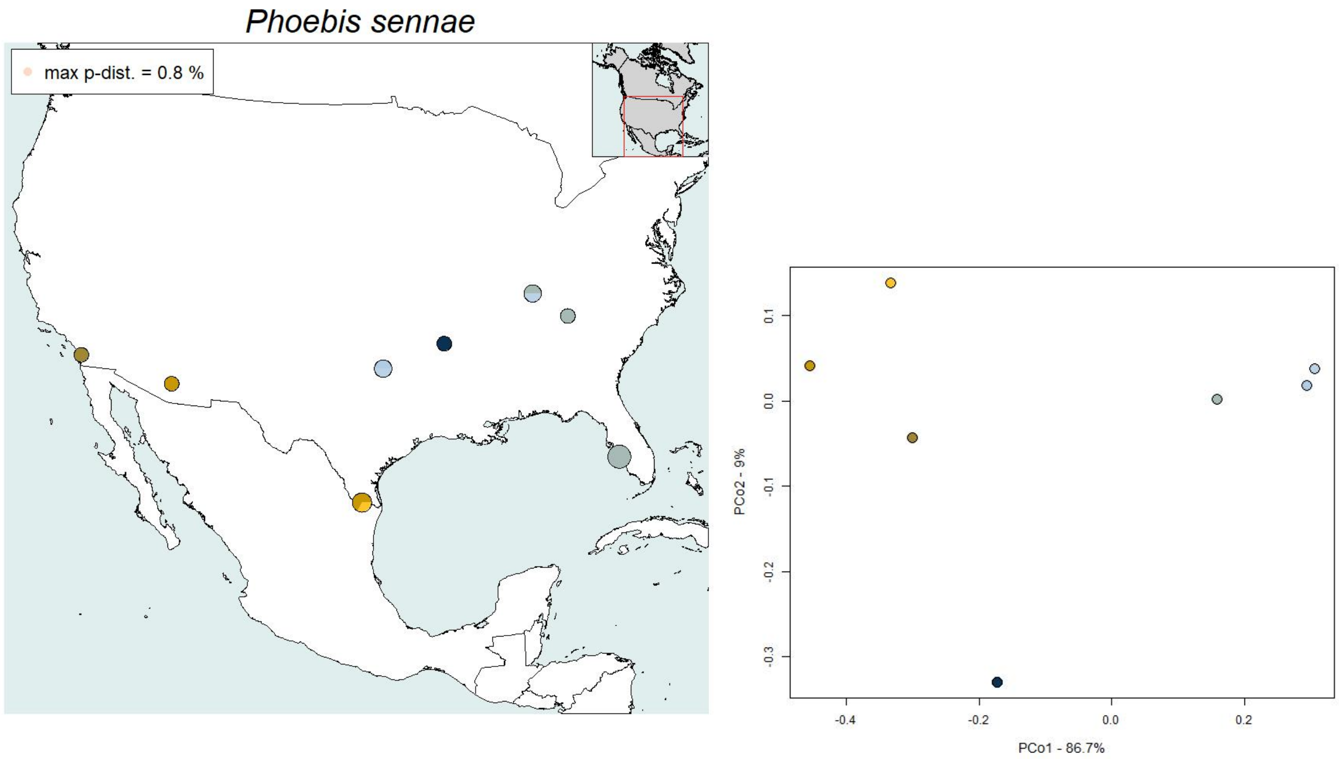

Figure 900 Map of *Phoebis sennae* showing the localities of the sequenced specimens (left). Nearby localities are grouped in pies. Colours match the bidimensional colour space of the PCoA projection (right) of max p-dists among sequences (dots). Sequences= 17; Hap obs.= 6; Hap asympt.= 10.2; Hap % obs.= 58.6%; GST= 0.931; DST= 0.0026; HD= 0.743; ND= 0.0033; max p-dist= 0.8%.

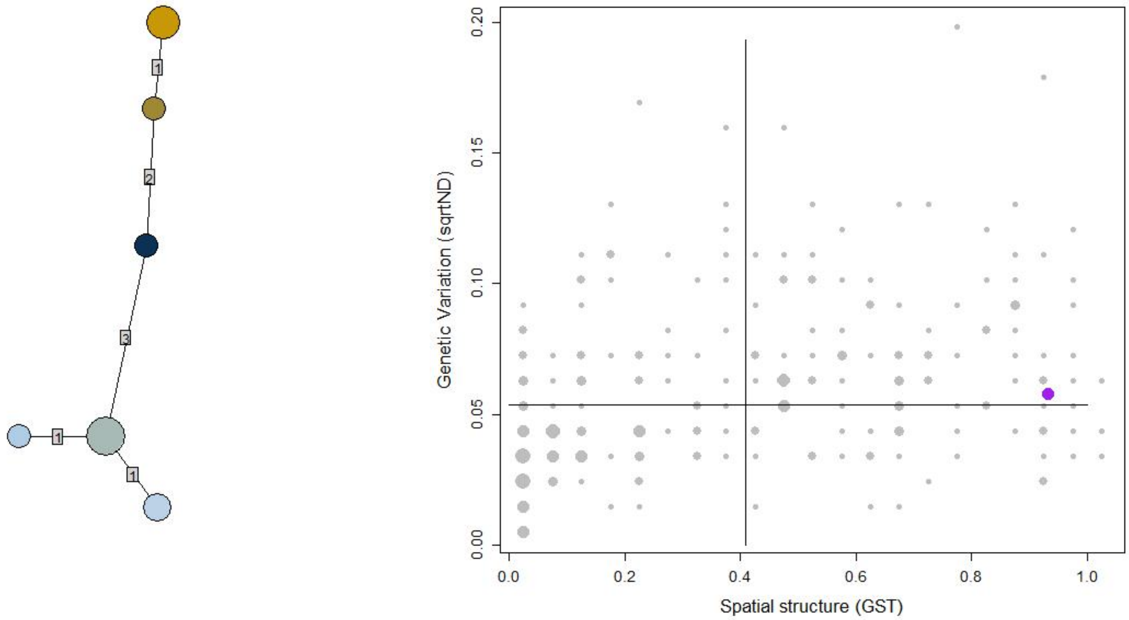

Figure 901: Haplotype network (left) of *Phoebis sennae* sequences > 599 bp with colours matching the PCoA colour space (above). The bubble plot for mt-DNA polymorphism (square root transformed nucleotide diversity) and spatial structure (GST) among all species in the atlas and values for *Phoebis sennae* (purple dot). The horizontal and vertical lines represent median values of nucleotide diversity and GST, respectively. Sequences > 599 bp= 17.

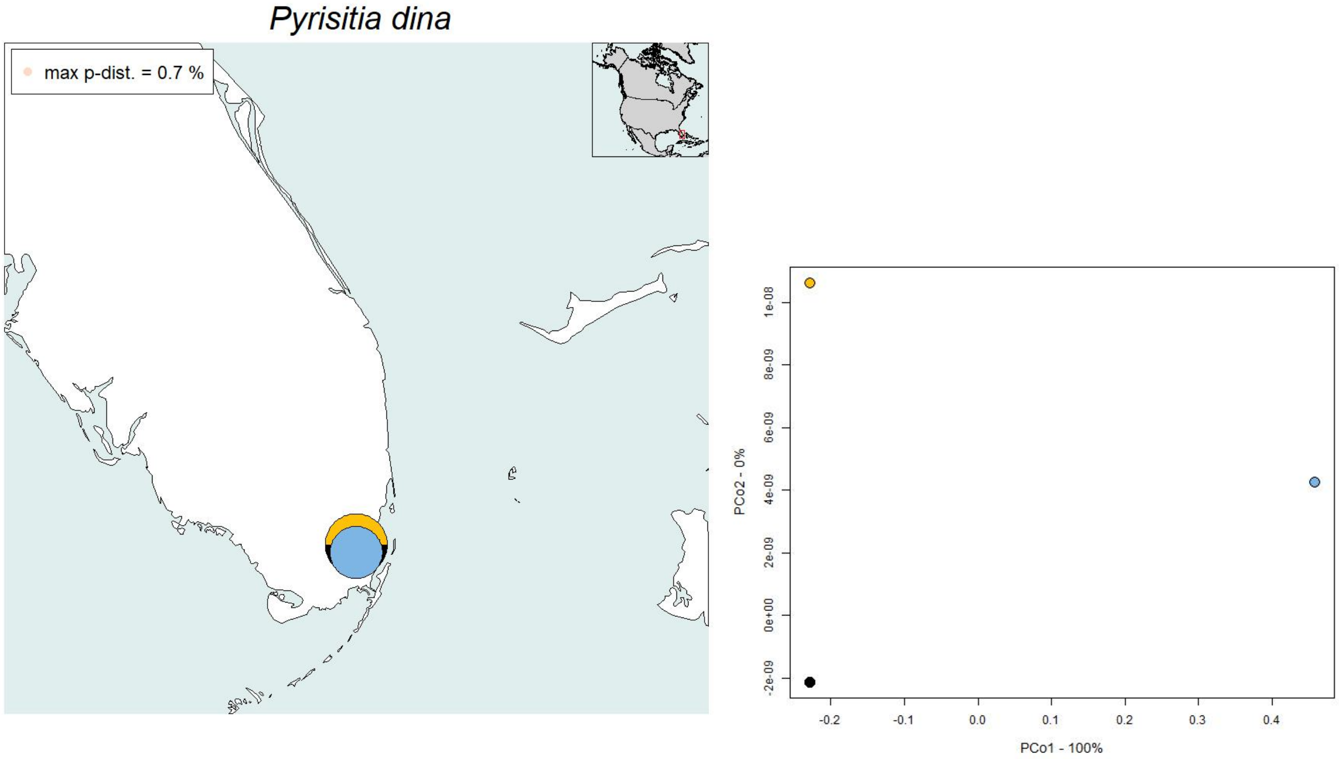

Figure 902 Map of *Pyrisitia dina* showing the localities of the sequenced specimens (left). Nearby localities are grouped in pies. Colours match the bidimensional colour space of the PCoA projection (right) of max p-dists among sequences (dots). Sequences= 3; Hap obs.= 2; Hap asympt.= NA; Hap % obs.= NA%; GST= NaN; DST= NaN; HD= NA; ND= NA; max p-dist= 0.7%.

Haplotype network analysis and bubble plot of *Pyrisitia dina* were not possible. Sequences > 599 bp = 2.

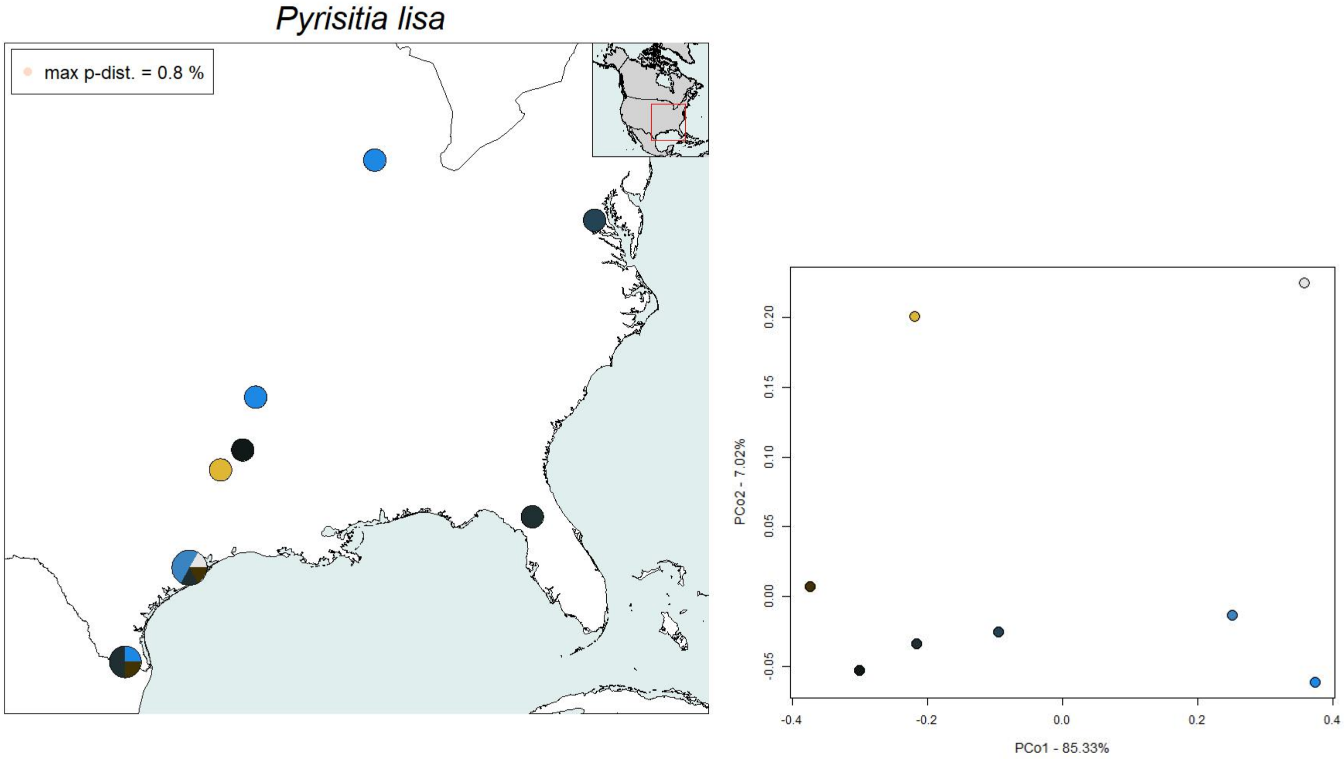

Figure 903 Map of *Pyrisitia lisa* showing the localities of the sequenced specimens (left). Nearby localities are grouped in pies. Colours match the bidimensional colour space of the PCoA projection (right) of max p-dists among sequences (dots). Sequences= 16; Hap obs.= 8; Hap asympt.= 17.4; Hap % obs.= 46%; GST= 0.028; DST= 0.0001; HD= 0.867; ND= 0.0036; max p-dist= 0.8%.

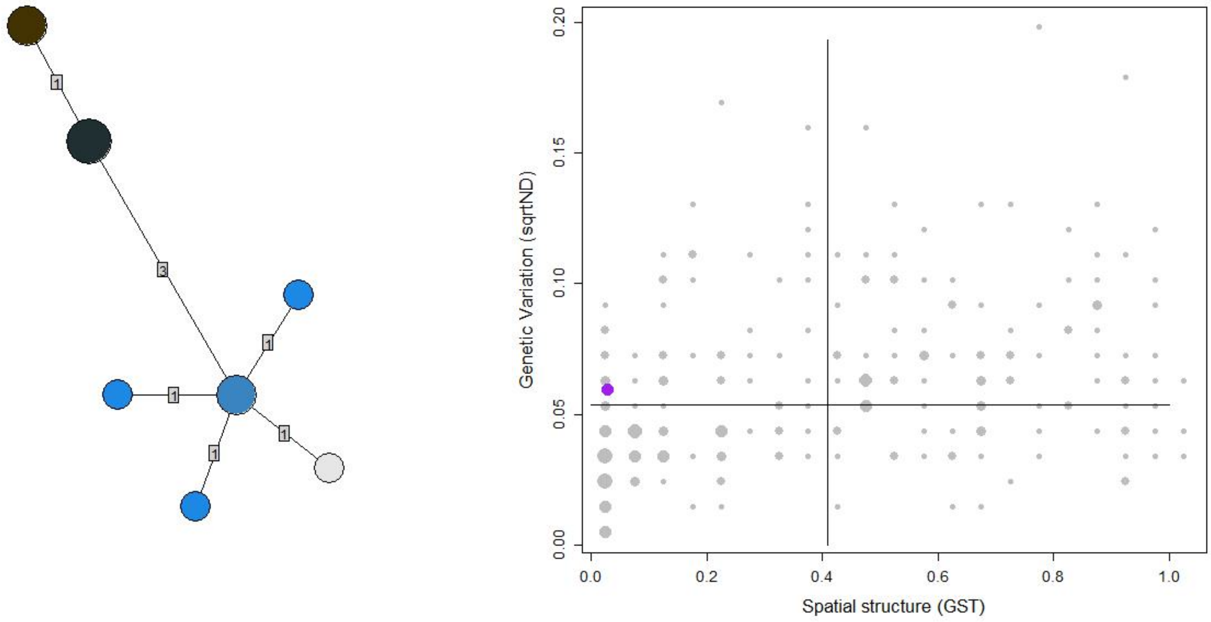

Figure 904: Haplotype network (left) of *Pyrisitia lisa* sequences > 599 bp with colours matching the PCoA colour space (above). The bubble plot for mt-DNA polymorphism (square root transformed nucleotide diversity) and spatial structure (GST) among all species in the atlas and values for *Pyrisitia lisa* (purple dot). The horizontal and vertical lines represent median values of nucleotide diversity and GST, respectively. Sequences > 599 bp= 15.

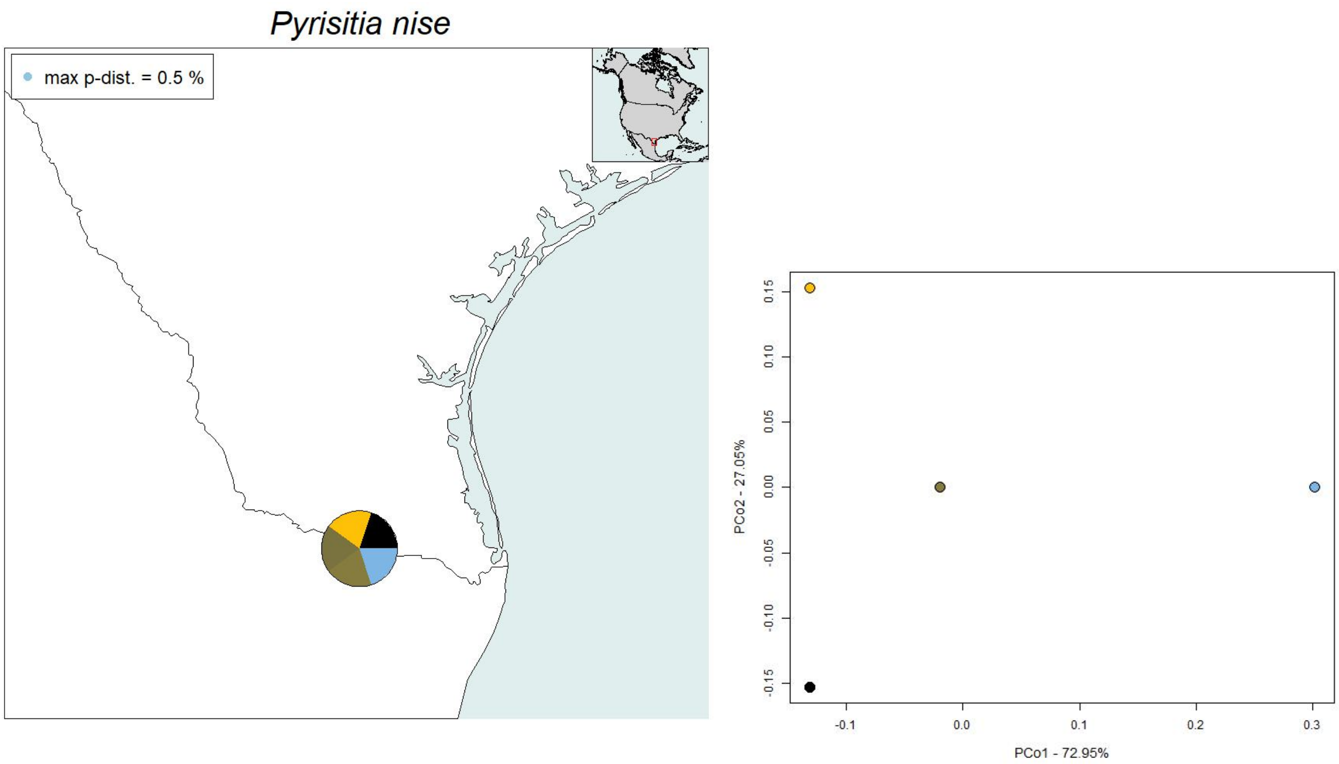

Figure 905 Map of *Pyrisitia nise* showing the localities of the sequenced specimens (left). Nearby localities are grouped in pies. Colours match the bidimensional colour space of the PCoA projection (right) of max p-dists among sequences (dots). Sequences= 5; Hap obs.= 4; Hap asympt.= NA; Hap % obs.= NA%; GST= NaN; DST= NaN; HD= NA; ND= NA; max p-dist= 0.5%.

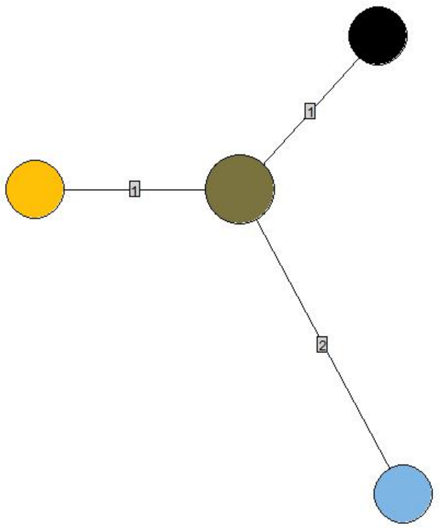

Figure 906: Haplotype network of *Pyrisitia nise*. Sequences > 599 bp= 5.

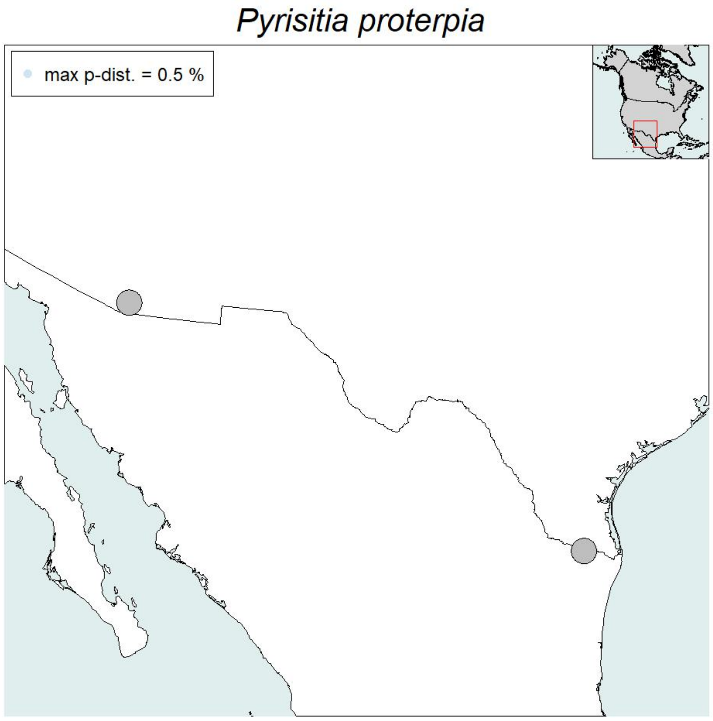

Figure 907: Map of *Pyrisitia proterpia* showing the localities of the sequenced specimens. Nearby localities are grouped in pies. Due to the presence of a single haplotype PCoA projection was not done and a single grey colour was plotted on the map. Sequences= 2; Hap obs.= 2; Hap asympt.= NA; Hap % obs.= NA%; GST= NaN; DST= NaN; HD= NA; ND= NA; max p-dist= 0.5%.

Haplotype network analysis and bubble plot of *Pyrisitia proterpia* were not possible. Sequences > 599 bp = 2.

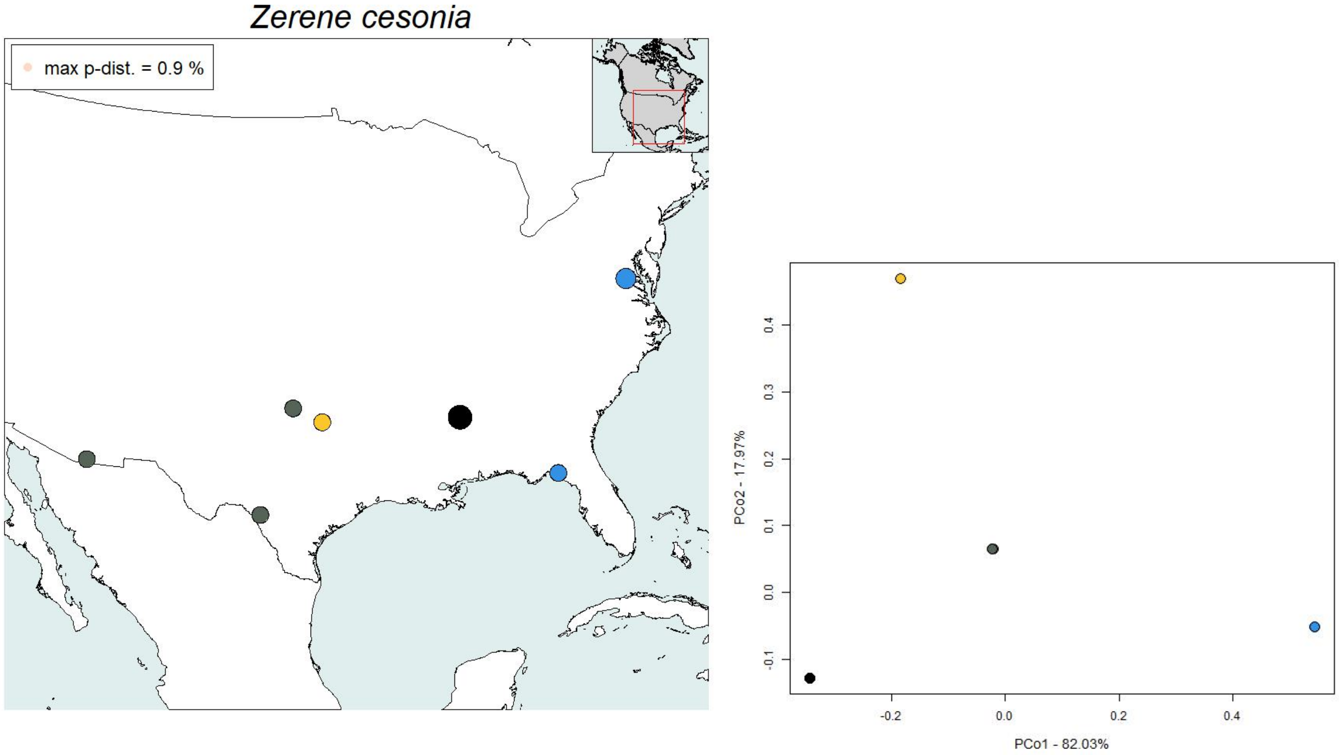

Figure 908 Map of *Zerene cesonia* showing the localities of the sequenced specimens (left). Nearby localities are grouped in pies. Colours match the bidimensional colour space of the PCoA projection (right) of max p-dists among sequences (dots). Sequences= 11; Hap obs.= 4; Hap asympt.= 4; Hap % obs.= 100%; GST= NaN; DST= NaN; HD= 0.782; ND= 0.0046; max p-dist= 0.9%.

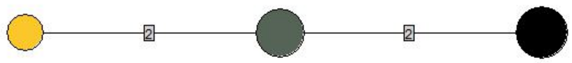

Figure 909: Haplotype network of *Zerene cesonia*. Sequences > 599 bp= 8.

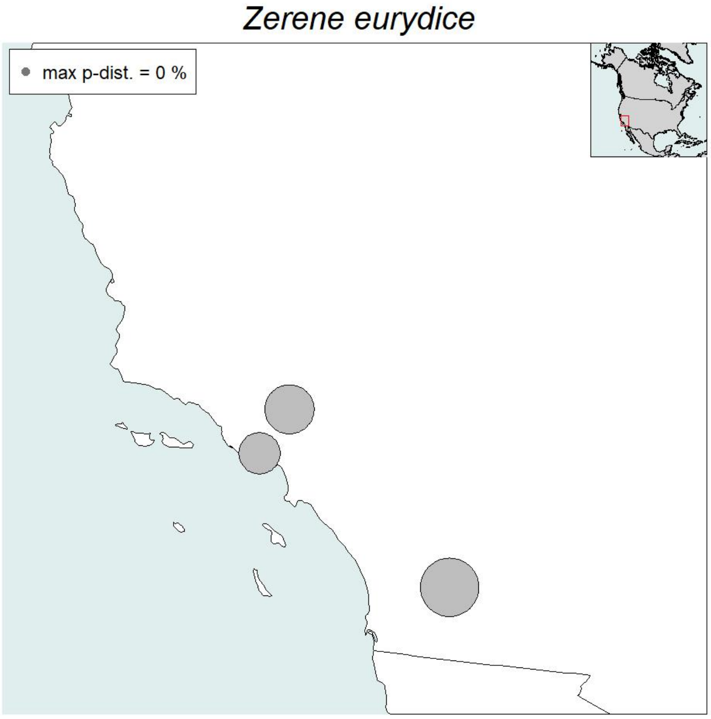

Figure 910: Map of *Zerene eurydice* showing the localities of the sequenced specimens. Nearby localities are grouped in pies. Due to the presence of a single haplotype PCoA projection was not done and a single grey colour was plotted on the map. Sequences= 7; Hap obs.= 1; Hap asympt.= NA; Hap % obs.= NA%; GST= NaN; DST= NaN; HD= NA; ND= NA; max p-dist= 0%.

Haplotype network analysis and bubble plot of *Zerene eurydice* were not possible. Sequences > 599 bp = 5.

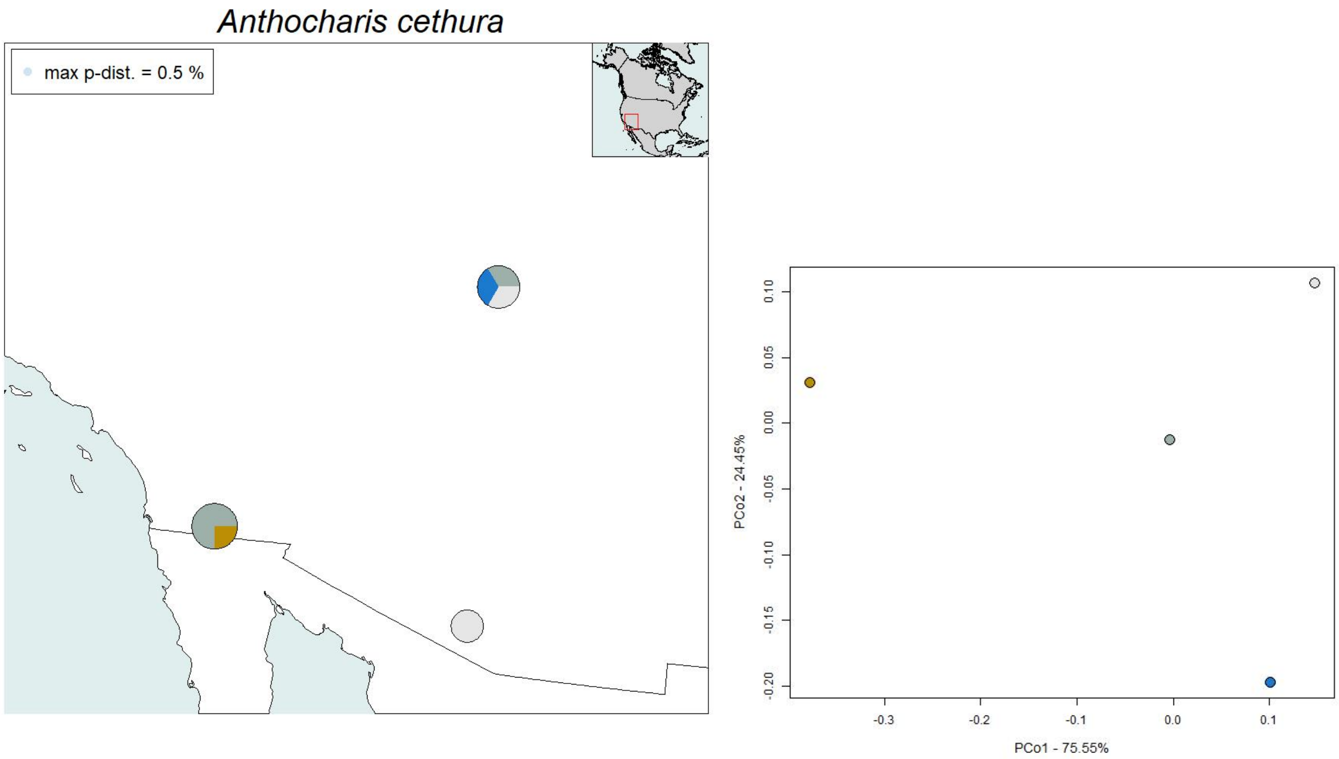

Figure 911 Map of *Anthocharis cethura* showing the localities of the sequenced specimens (left). Nearby localities are grouped in pies. Colours match the bidimensional colour space of the PCoA projection (right) of max p-dists among sequences (dots). Sequences= 8; Hap obs.= 4; Hap asympt.= NA; Hap % obs.= NA%; GST= NaN; DST= NaN; HD= NA; ND= NA; max p-dist= 0.5%.

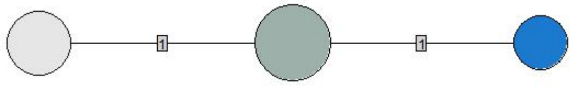

Figure 912: Haplotype network of *Anthocharis cethura*. Sequences > 599 bp= 7.

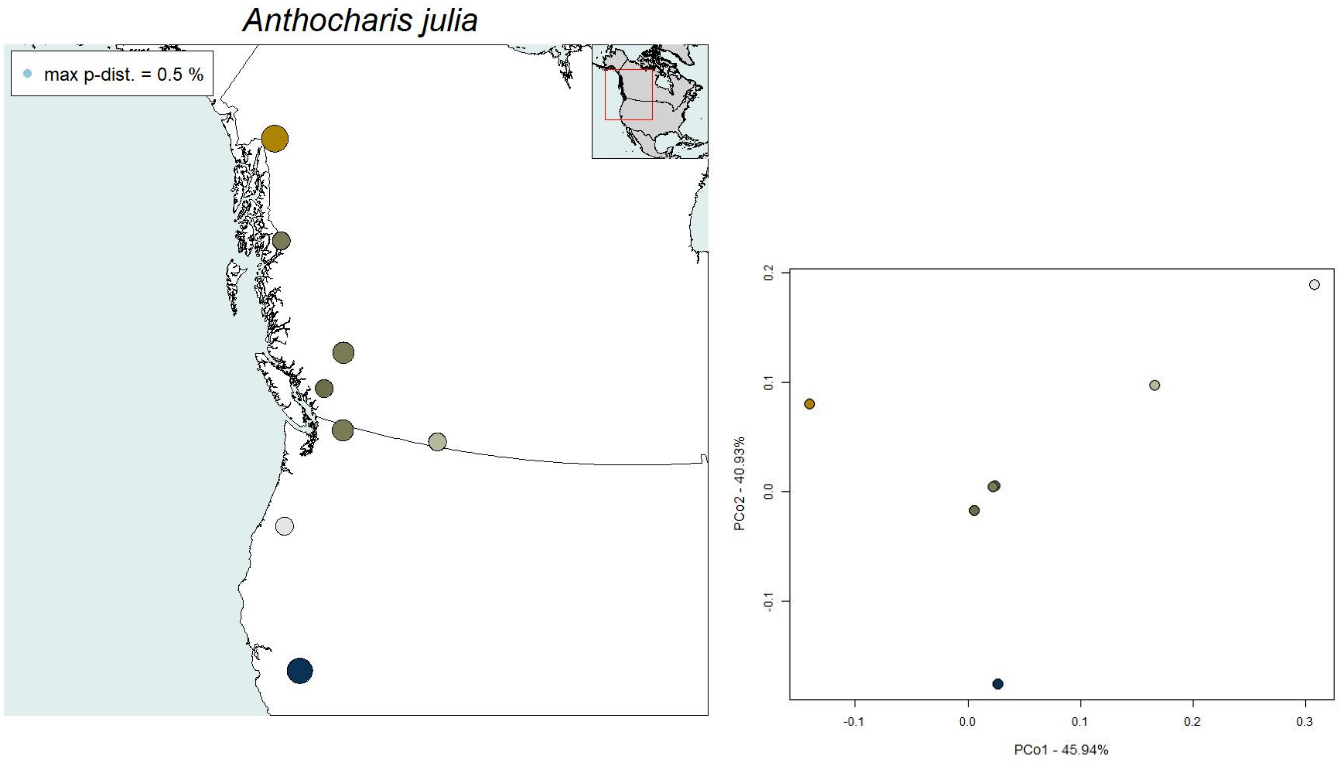

Figure 913 Map of *Anthocharis julia* showing the localities of the sequenced specimens (left). Nearby localities are grouped in pies. Colours match the bidimensional colour space of the PCoA projection (right) of max p-dists among sequences (dots). Sequences= 17; Hap obs.= 6; Hap asympt.= 8.8; Hap % obs.= 68%; GST= 0.902; DST= 0.0016; HD= 0.809; ND= 0.002; max p-dist= 0.5%.

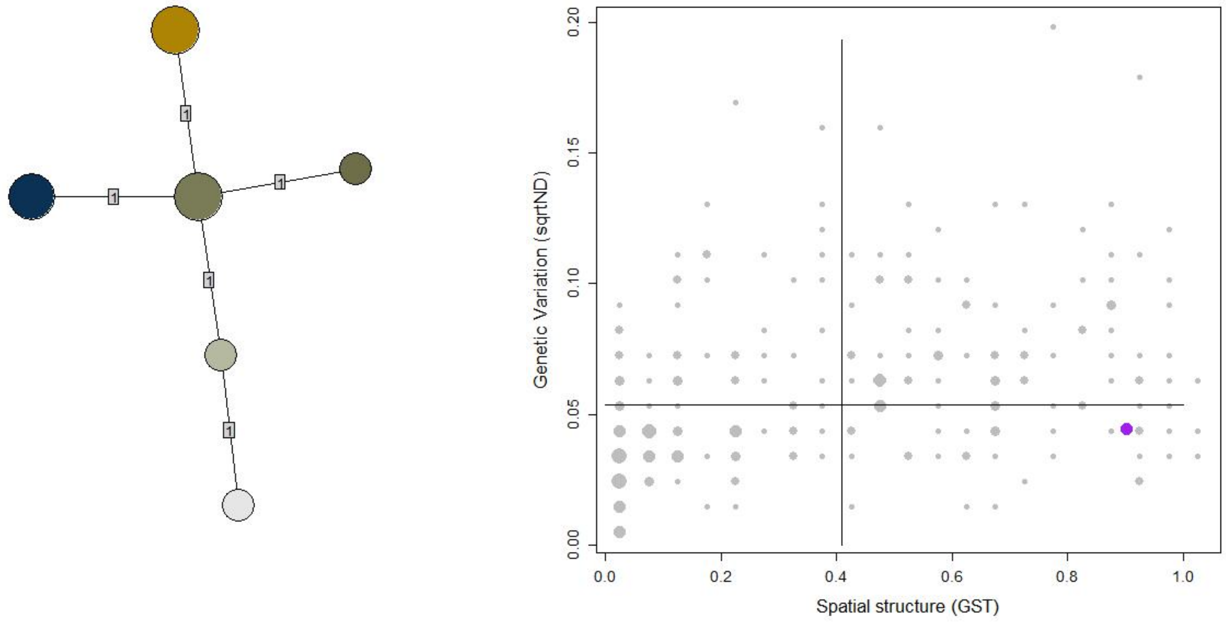

Figure 914: Haplotype network (left) of *Anthocharis julia* sequences > 599 bp with colours matching the PCoA colour space (above). The bubble plot for mt-DNA polymorphism (square root transformed nucleotide diversity) and spatial structure (GST) among all species in the atlas and values for *Anthocharis julia* (purple dot). The horizontal and vertical lines represent median values of nucleotide diversity and GST, respectively. Sequences > 599 bp= 17.

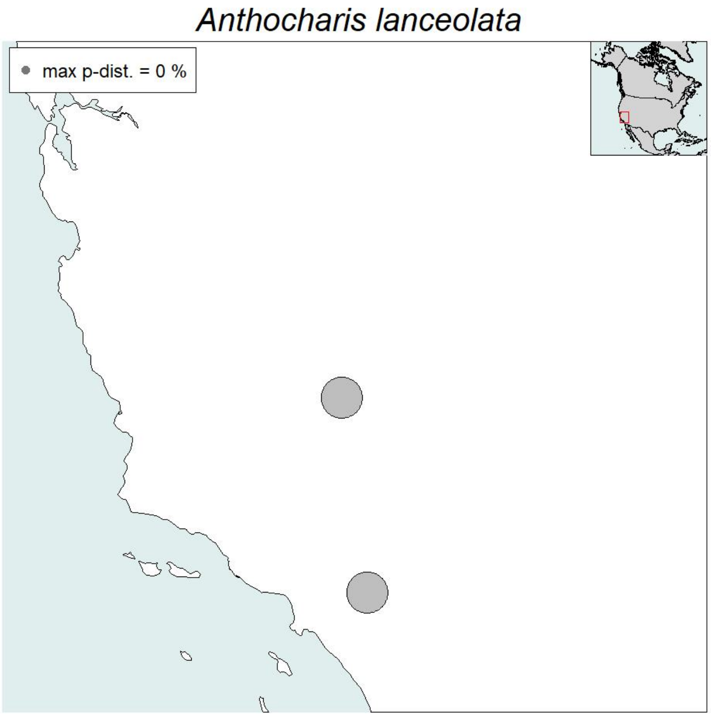

Figure 915: Map of *Anthocharis lanceolata* showing the localities of the sequenced specimens. Nearby localities are grouped in pies. Due to the presence of a single haplotype PCoA projection was not done and a single grey colour was plotted on the map. Sequences= 2; Hap obs.= 1; Hap asympt.= NA; Hap % obs.= NA%; GST= NaN; DST= NaN; HD= NA; ND= NA; max p-dist= 0%.

Haplotype network analysis and bubble plot of *Anthocharis lanceolata* were not possible. Sequences > 599 bp = 2.

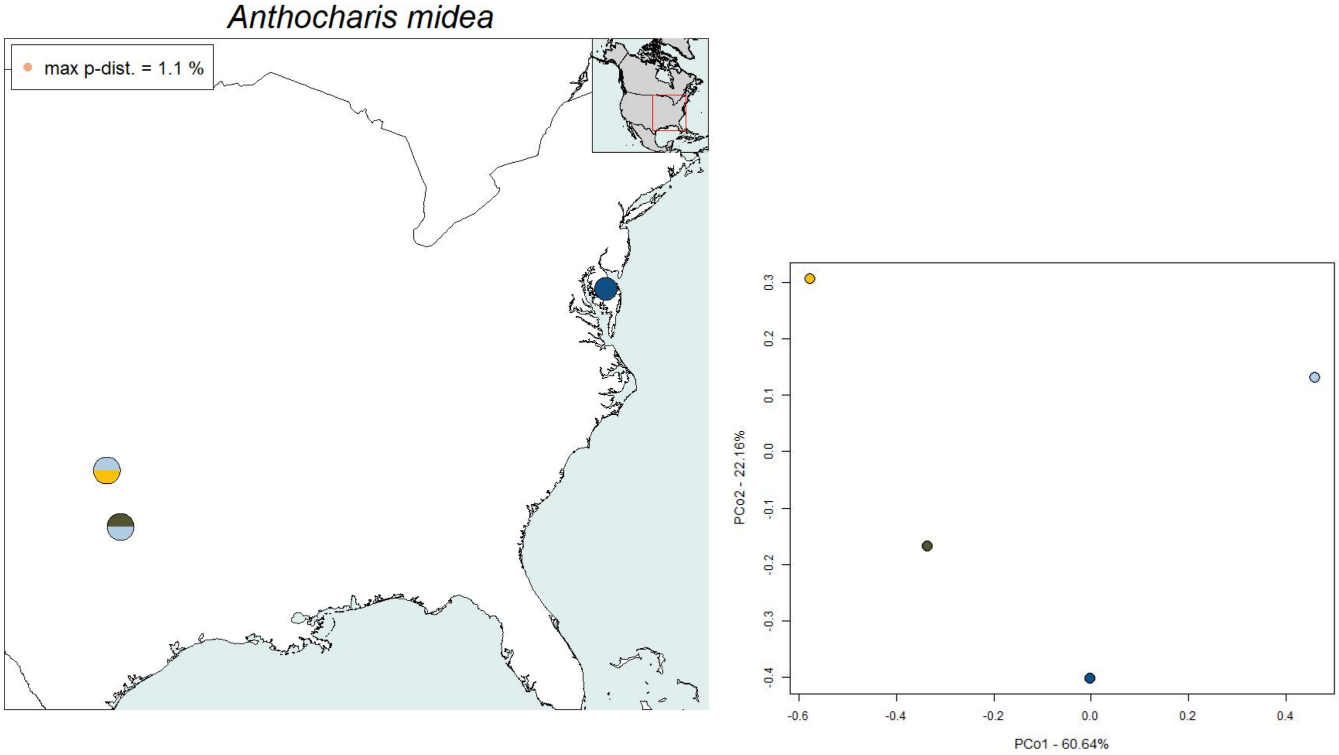

Figure 916 Map of *Anthocharis midea* showing the localities of the sequenced specimens (left). Nearby localities are grouped in pies. Colours match the bidimensional colour space of the PCoA projection (right) of max p-dists among sequences (dots). Sequences= 5; Hap obs.= 4; Hap asympt.= NA; Hap % obs.= NA%; GST= NaN; DST= NaN; HD= NA; ND= NA; max p-dist= 1.1%.

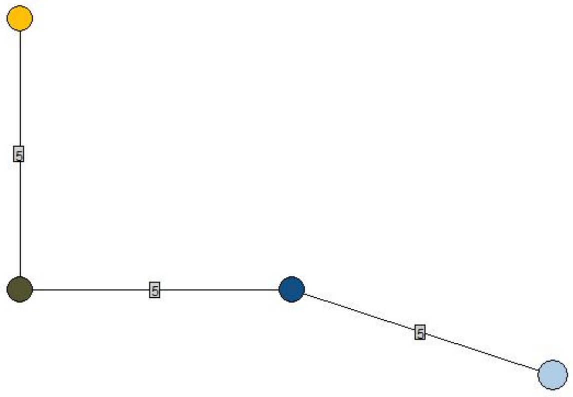

Figure 917: Haplotype network of *Anthocharis midea*. Sequences > 599 bp= 5.

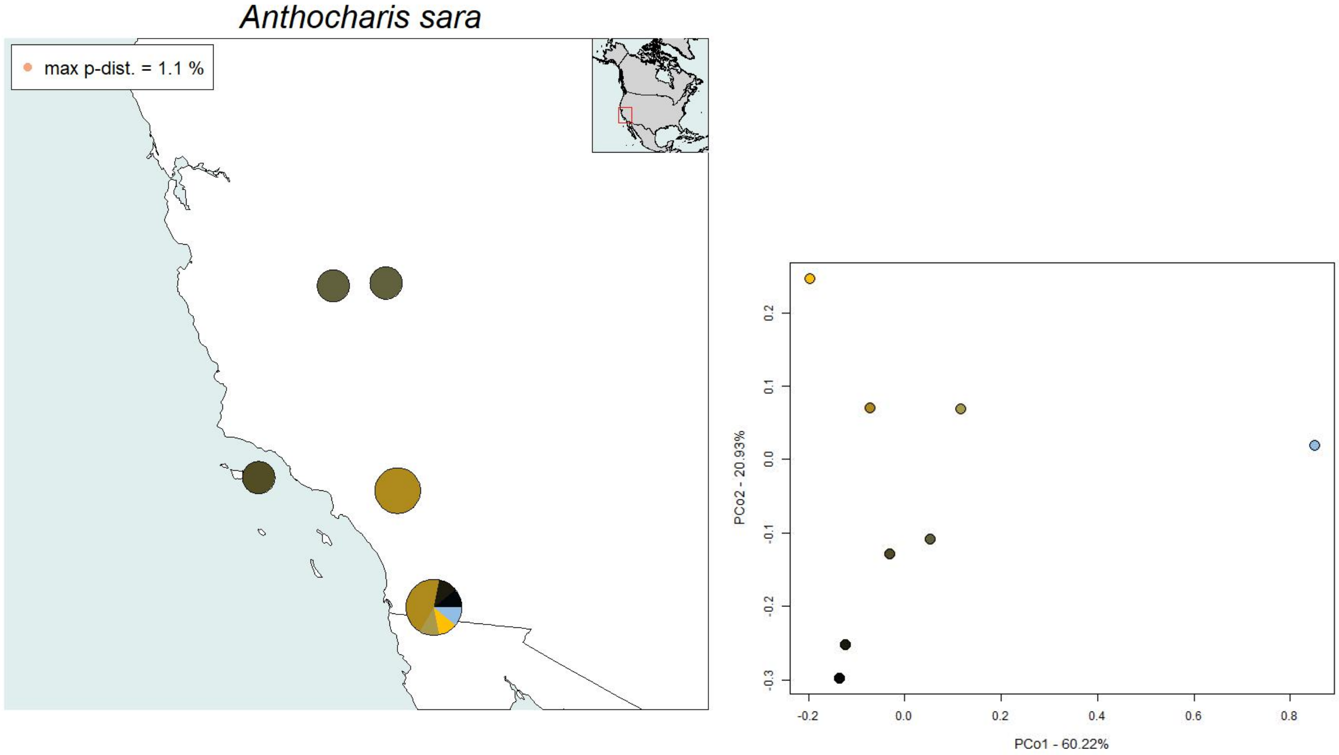

Figure 918 Map of *Anthocharis sara* showing the localities of the sequenced specimens (left). Nearby localities are grouped in pies. Colours match the bidimensional colour space of the PCoA projection (right) of max p-dists among sequences (dots). Sequences= 16; Hap obs.= 7; Hap asympt.= 18.7; Hap % obs.= 37.4%; GST= 0.004; DST= 0; HD= 0.692; ND= 0.0028; max p-dist= 1.1%.

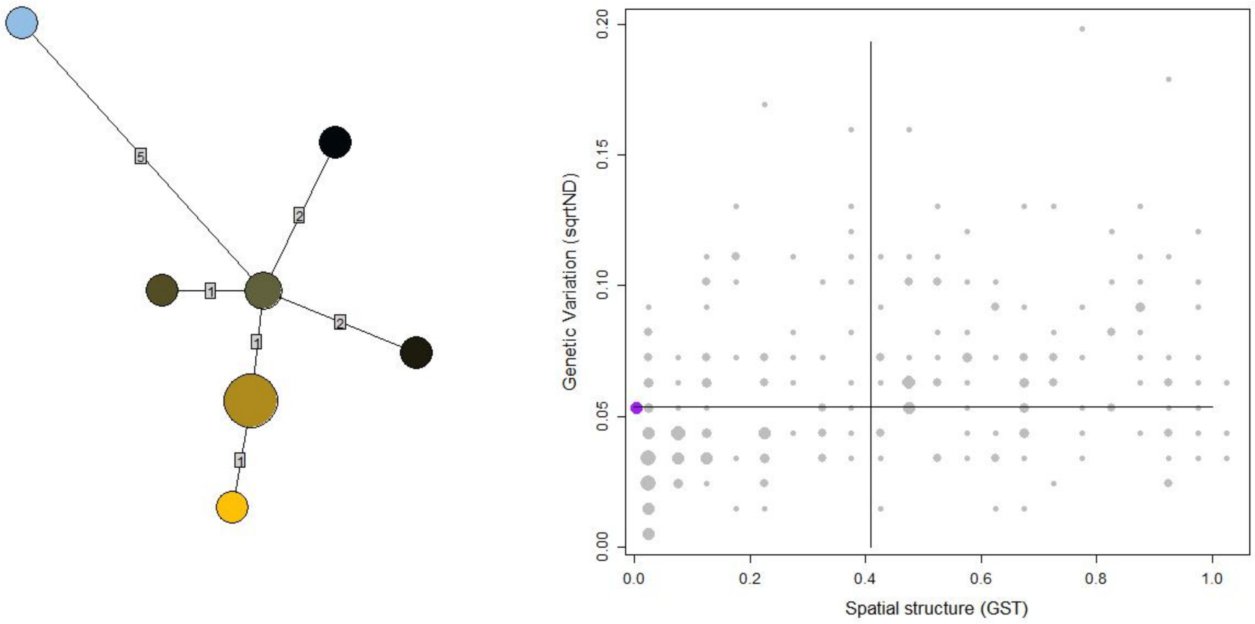

Figure 919: Haplotype network (left) of *Anthocharis sara* sequences > 599 bp with colours matching the PCoA colour space (above). The bubble plot for mt-DNA polymorphism (square root transformed nucleotide diversity) and spatial structure (GST) among all species in the atlas and values for *Anthocharis sara* (purple dot). The horizontal and vertical lines represent median values of nucleotide diversity and GST, respectively. Sequences > 599 bp= 15.

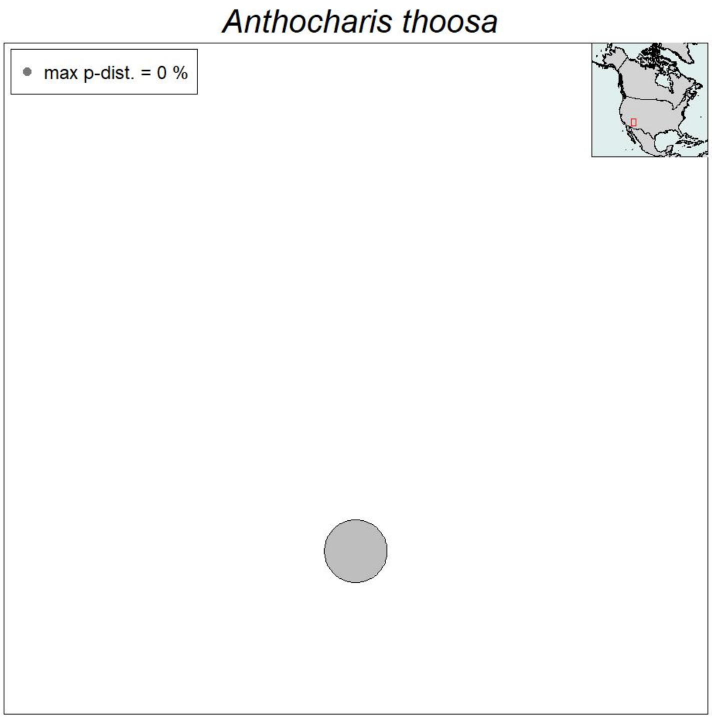

Figure 920: Map of *Anthocharis thoosa* showing the localities of the sequenced specimens. Nearby localities are grouped in pies. Due to the presence of a single haplotype PCoA projection was not done and a single grey colour was plotted on the map. Sequences= 2; Hap obs.= 1; Hap asympt.= NA; Hap % obs.= NA%; GST= NaN; DST= NaN; HD= NA; ND= NA; max p-dist= 0%.

Haplotype network analysis and bubble plot of *Anthocharis thoosa* were not possible. Sequences > 599 bp = 2.

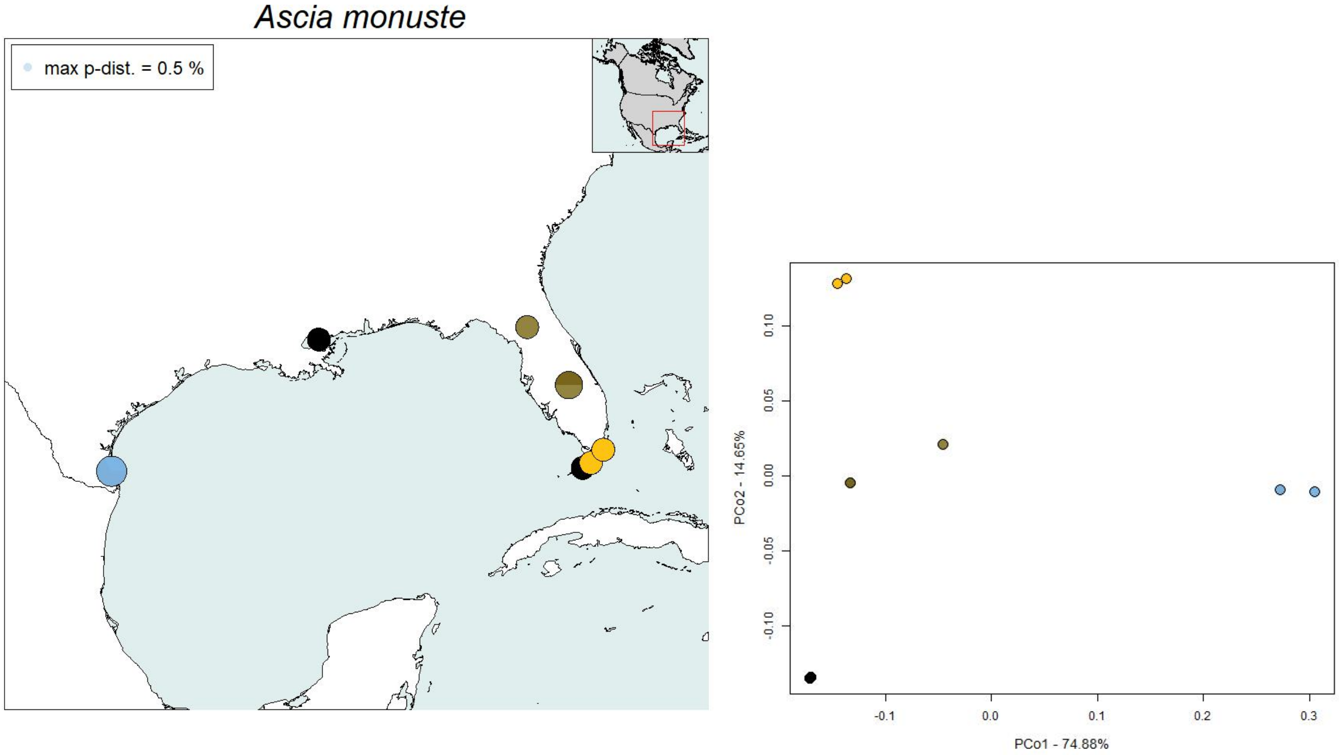

Figure 921 Map of *Ascia monuste* showing the localities of the sequenced specimens (left). Nearby localities are grouped in pies. Colours match the bidimensional colour space of the PCoA projection (right) of max p-dists among sequences (dots). Sequences= 10; Hap obs.= 6; Hap asympt.= 8; Hap % obs.= 74.8%; GST= NaN; DST= NaN; HD= 0.889; ND= 0.0026; max p-dist= 0.5%.

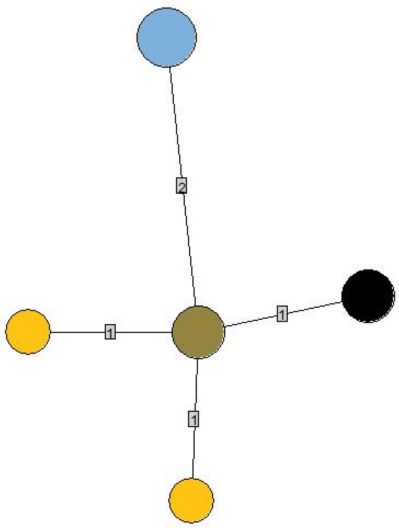

Figure 922: Haplotype network of *Ascia monuste*. Sequences > 599 bp= 9.

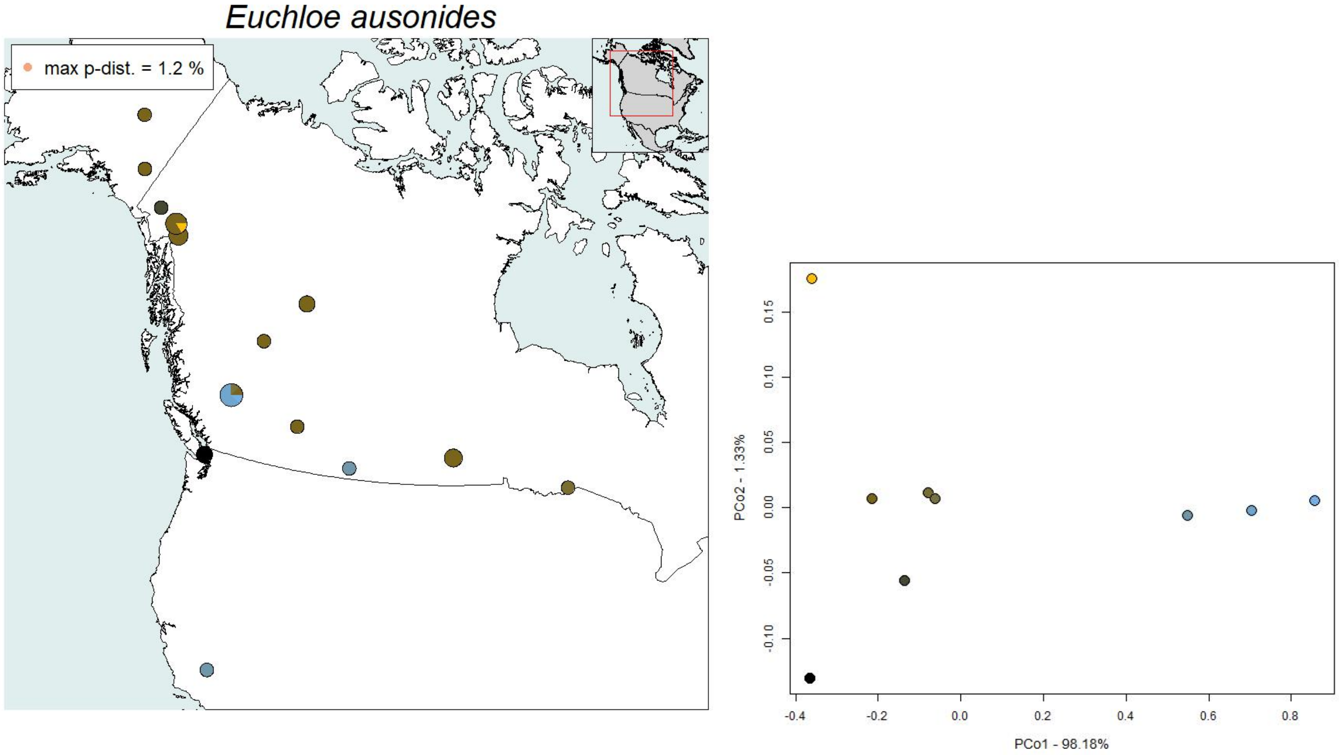

Figure 923 Map of *Euchloe ausonides* showing the localities of the sequenced specimens (left). Nearby localities are grouped in pies. Colours match the bidimensional colour space of the PCoA projection (right) of max p-dists among sequences (dots). Sequences= 33; Hap obs.= 8; Hap asympt.= 9.5; Hap % obs.= 84.6%; GST= 0.655; DST= 0.0029; HD= 0.652; ND= 0.0038; max p-dist= 1.2%.

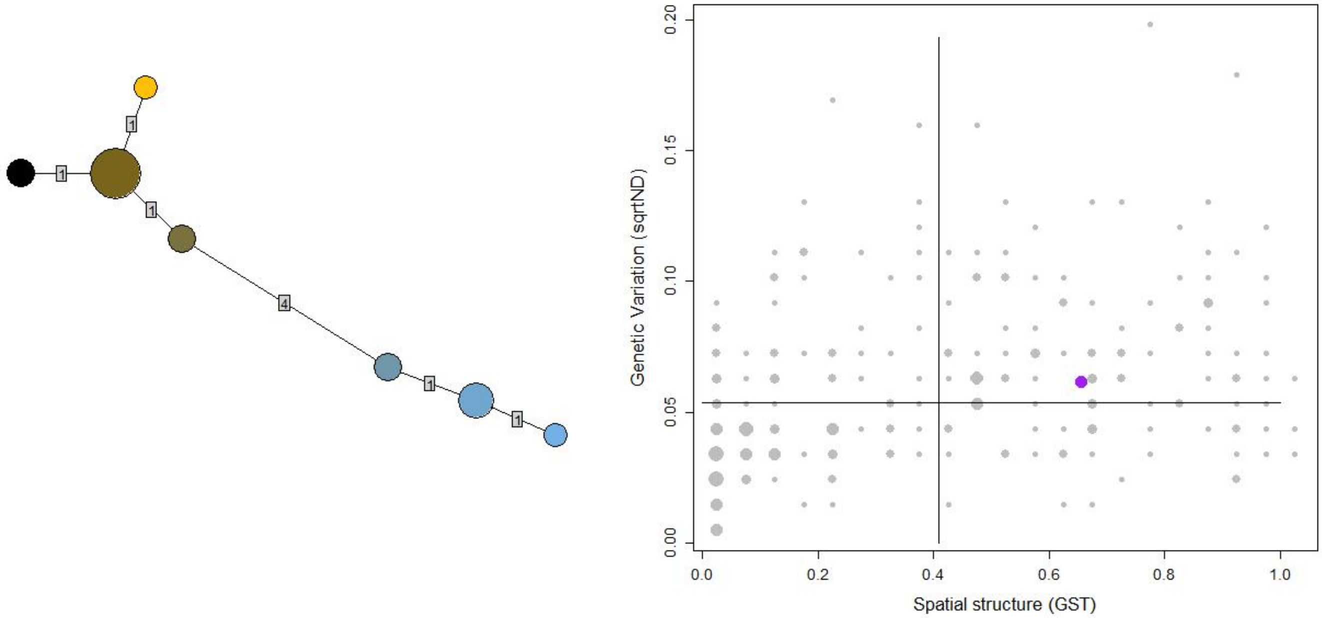

Figure 924: Haplotype network (left) of *Euchloe ausonides* sequences > 599 bp with colours matching the PCoA colour space (above). The bubble plot for mt-DNA polymorphism (square root transformed nucleotide diversity) and spatial structure (GST) among all species in the atlas and values for *Euchloe ausonides* (purple dot). The horizontal and vertical lines represent median values of nucleotide diversity and GST, respectively. Sequences > 599 bp= 33.

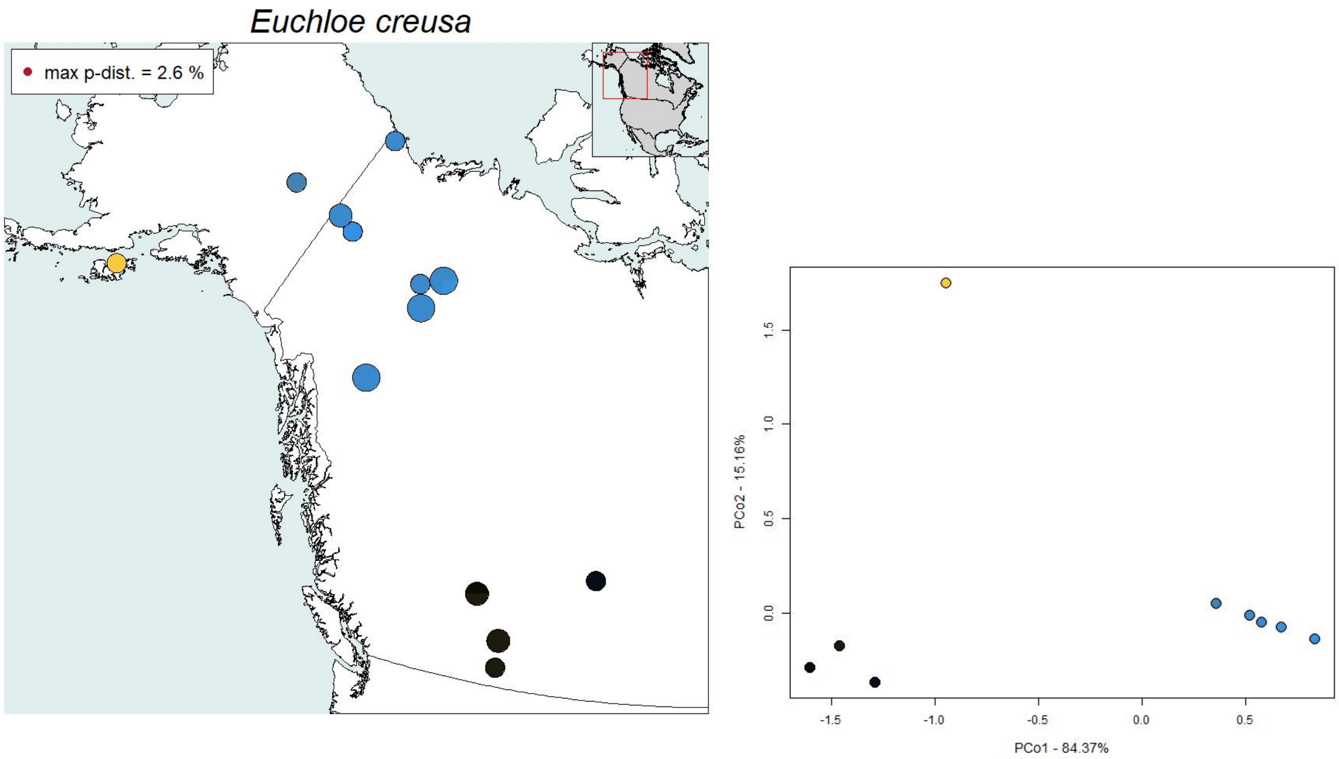

Figure 925 Map of *Euchloe creusa* showing the localities of the sequenced specimens (left). Nearby localities are grouped in pies. Colours match the bidimensional colour space of the PCoA projection (right) of max p-dists among sequences (dots). Sequences= 25; Hap obs.= 8; Hap asympt.= 22.4; Hap % obs.= 35.7%; GST= 0.39; DST= 0.0023; HD= 0.63; ND= 0.0094; max p-dist= 2.6%.

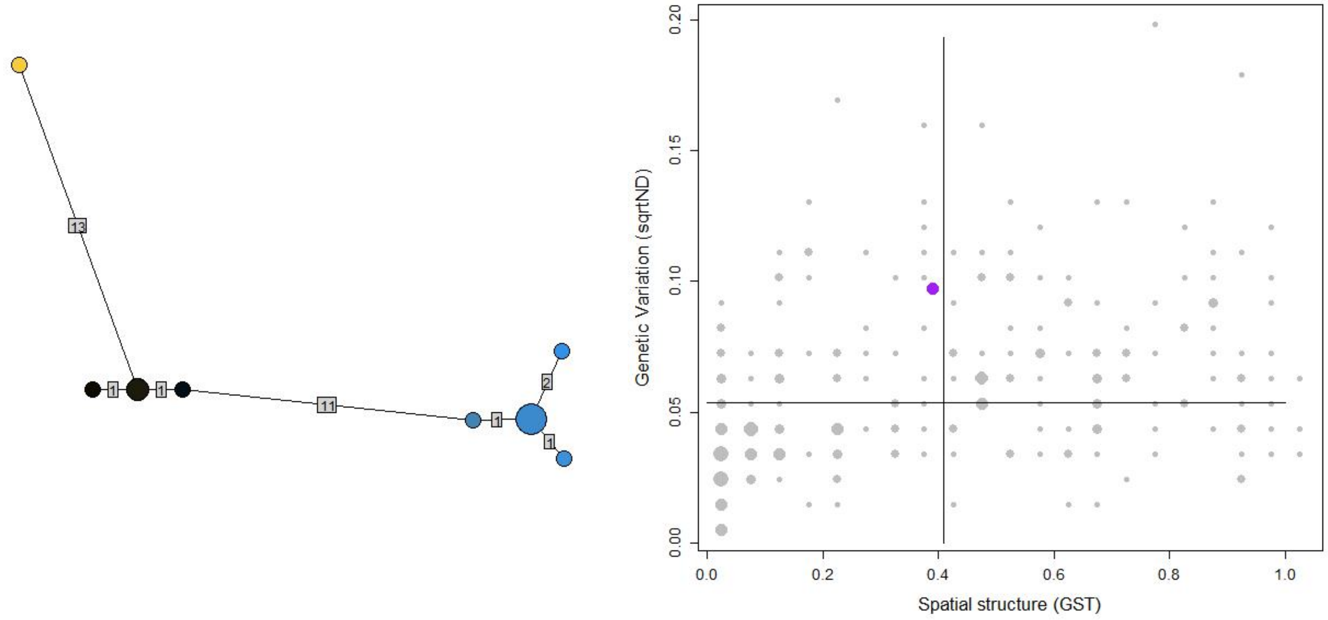

Figure 926: Haplotype network (left) of *Euchloe creusa* sequences > 599 bp with colours matching the PCoA colour space (above). The bubble plot for mt-DNA polymorphism (square root transformed nucleotide diversity) and spatial structure (GST) among all species in the atlas and values for *Euchloe creusa* (purple dot). The horizontal and vertical lines represent median values of nucleotide diversity and GST, respectively. Sequences > 599 bp= 25.

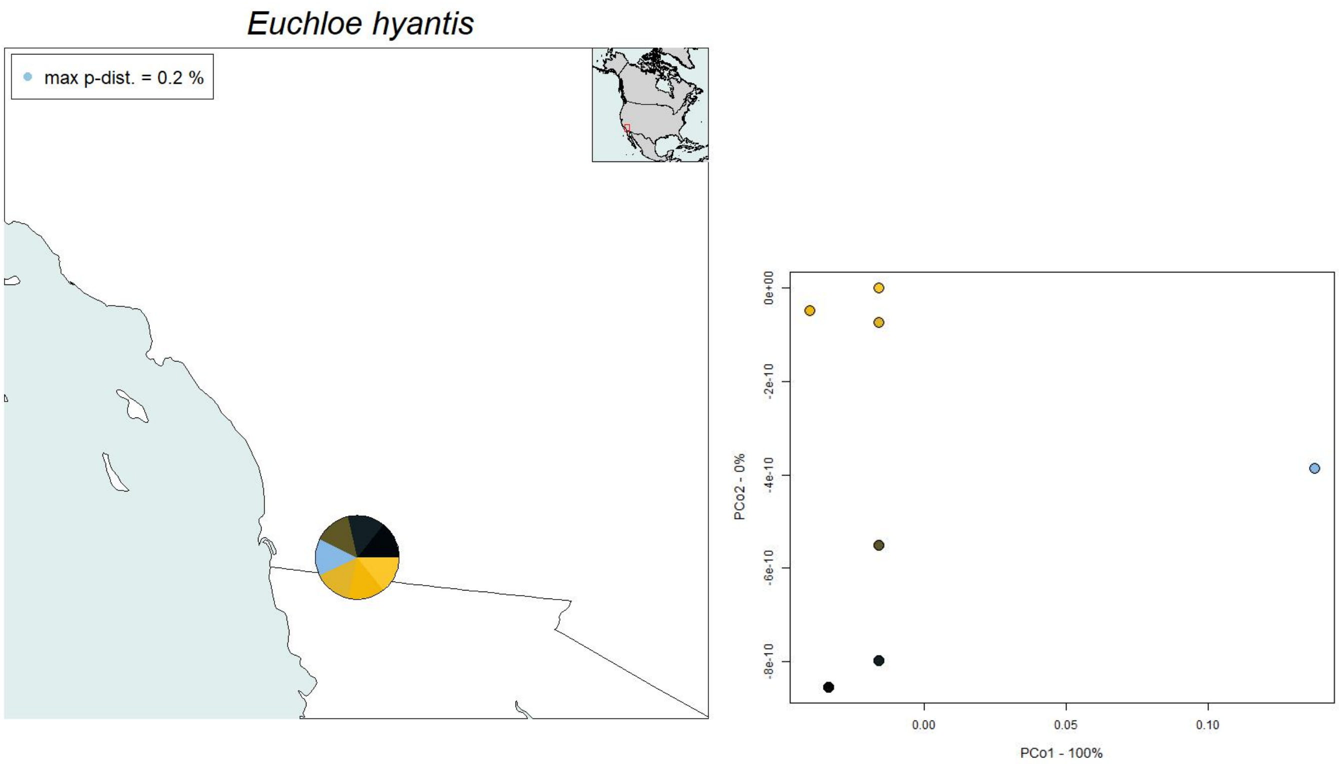

Figure 927 Map of *Euchloe hyantis* showing the localities of the sequenced specimens (left). Nearby localities are grouped in pies. Colours match the bidimensional colour space of the PCoA projection (right) of max p-dists among sequences (dots). Sequences= 7; Hap obs.= 2; Hap asympt.= NA; Hap % obs.= NA%; GST= NaN; DST= NaN; HD= NA; ND= NA; max p-dist= 0.2%.

Haplotype network analysis and bubble plot of *Euchloe hyantis* were not possible. Sequences > 599 bp = 5.

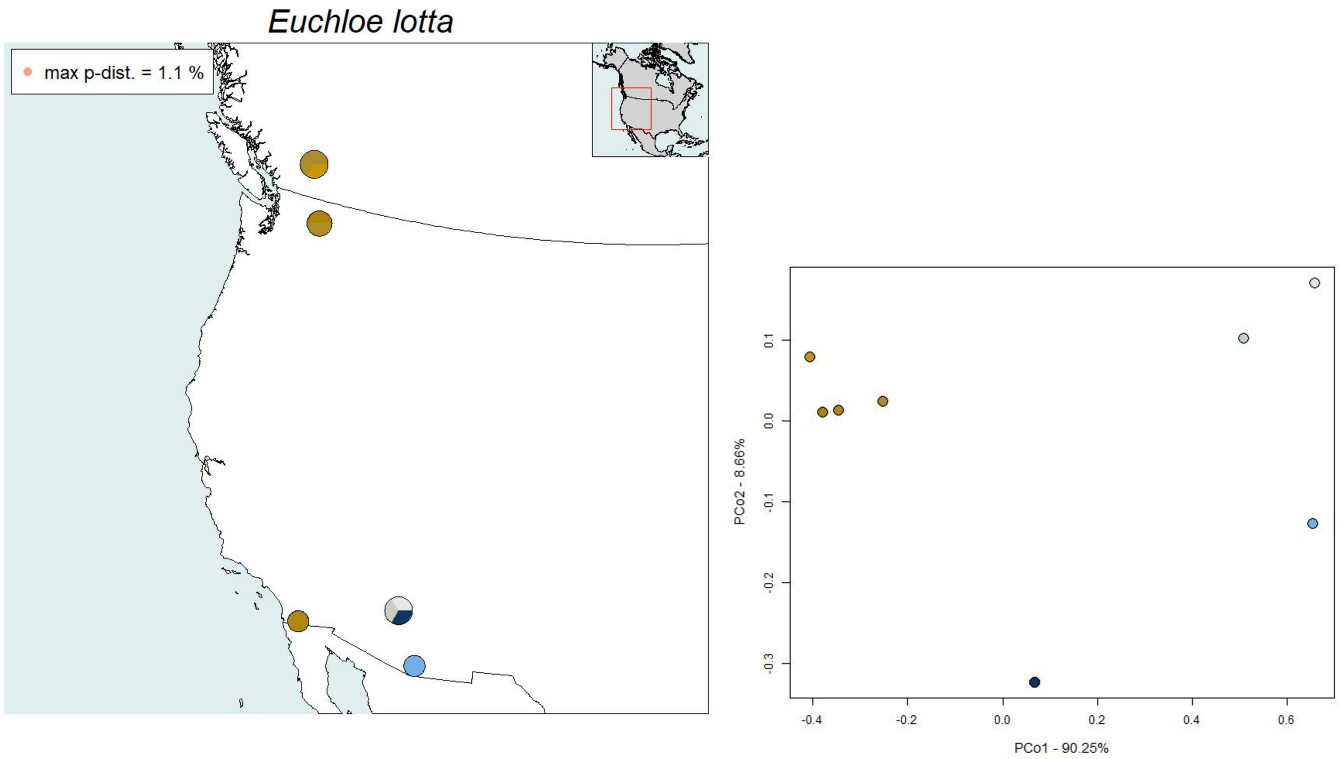

Figure 928 Map of *Euchloe lotta* showing the localities of the sequenced specimens (left). Nearby localities are grouped in pies. Colours match the bidimensional colour space of the PCoA projection (right) of max p-dists among sequences (dots). Sequences= 10; Hap obs.= 6; Hap asympt.= 15; Hap % obs.= 40%; GST= NaN; DST= NaN; HD= 0.778; ND= 0.005; max p-dist= 1.1%.

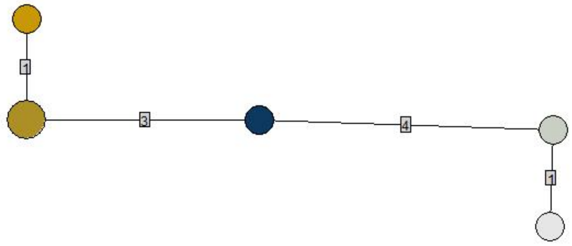

Figure 929: Haplotype network of *Euchloe lotta*. Sequences > 599 bp= 7.

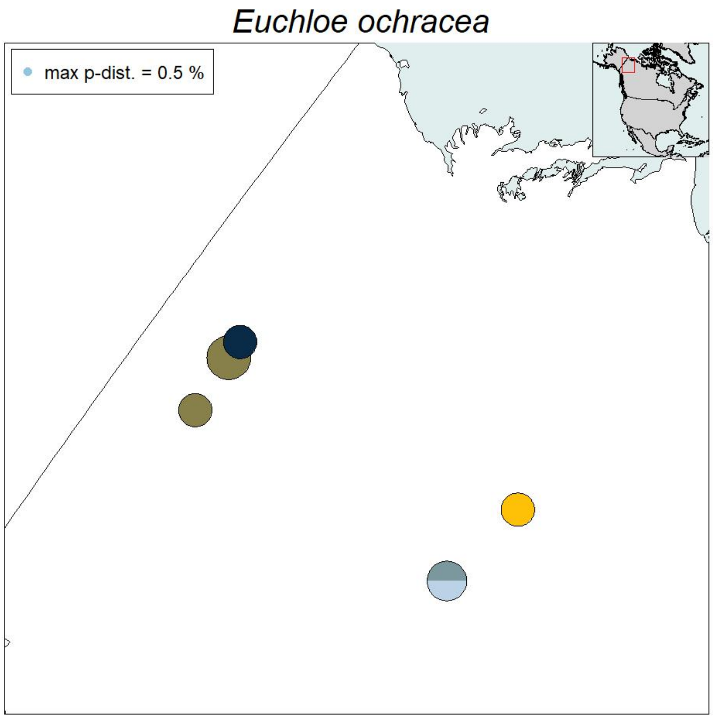

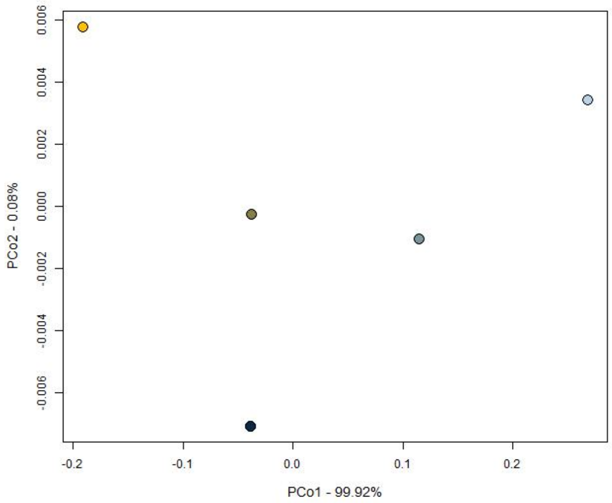

Figure 930 Map of *Euchloe ochracea* showing the localities of the sequenced specimens (left). Nearby localities are grouped in pies. Colours match the bidimensional colour space of the PCoA projection (right) of max p-dists among sequences (dots). Sequences= 8; Hap obs.= 4; Hap asympt.= NA; Hap % obs.= NA%; GST= NaN; DST= NaN; HD= NA; ND= NA; max p-dist= 0.5%.

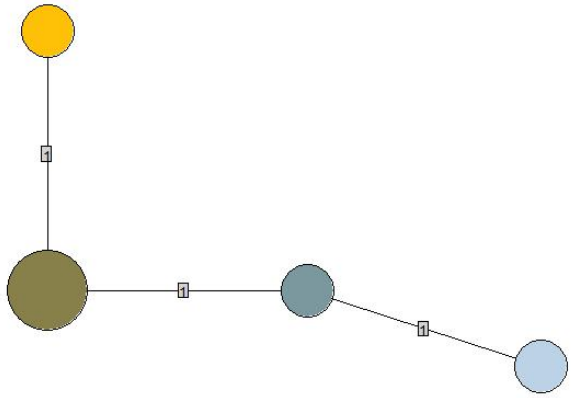

Figure 931: Haplotype network of *Euchloe ochracea*. Sequences > 599 bp= 8.

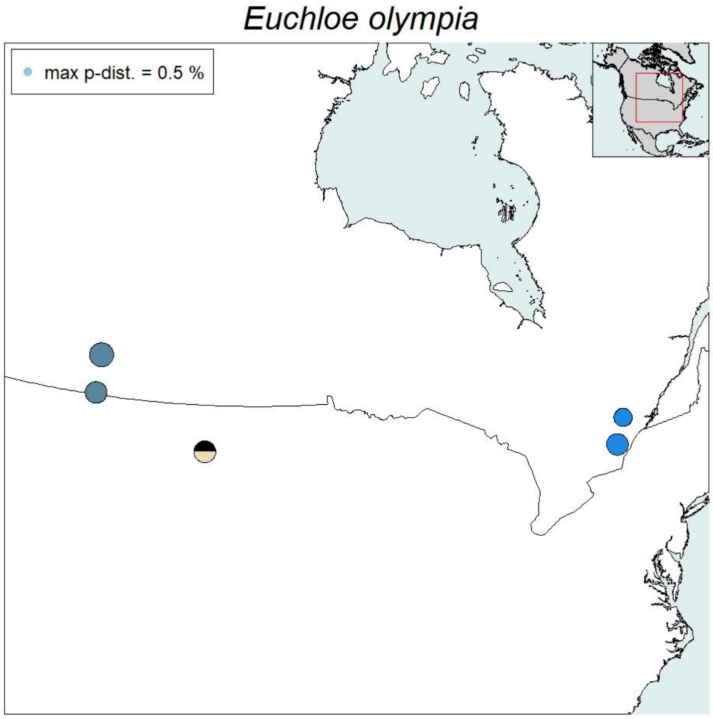

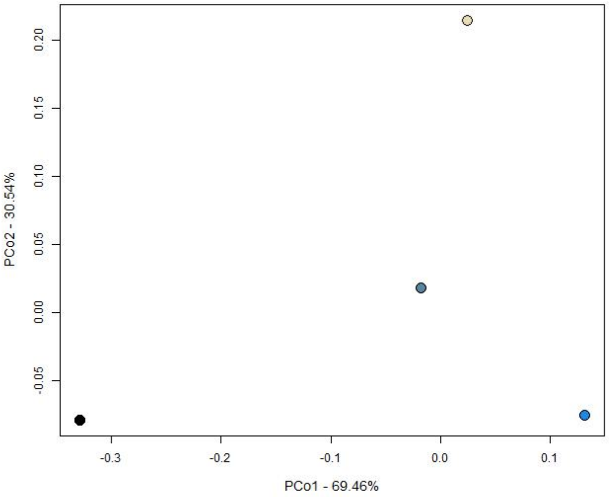

Figure 932 Map of *Euchloe olympia* showing the localities of the sequenced specimens (left). Nearby localities are grouped in pies. Colours match the bidimensional colour space of the PCoA projection (right) of max p-dists among sequences (dots). Sequences= 10; Hap obs.= 4; Hap asympt.= 4.9; Hap % obs.= 81.6%; GST= NaN; DST= NaN; HD= 0.711; ND= 0.0016; max p-dist= 0.5%.

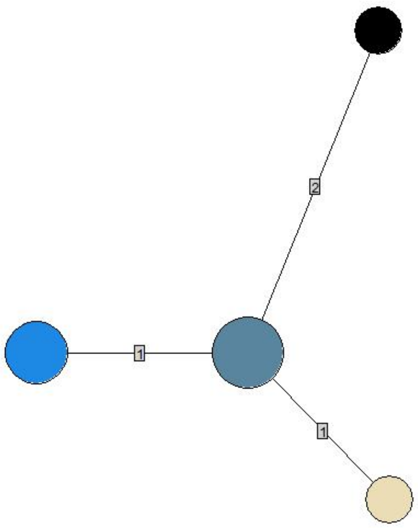

Figure 933: Haplotype network of *Euchloe olympia*. Sequences > 599 bp= 10.

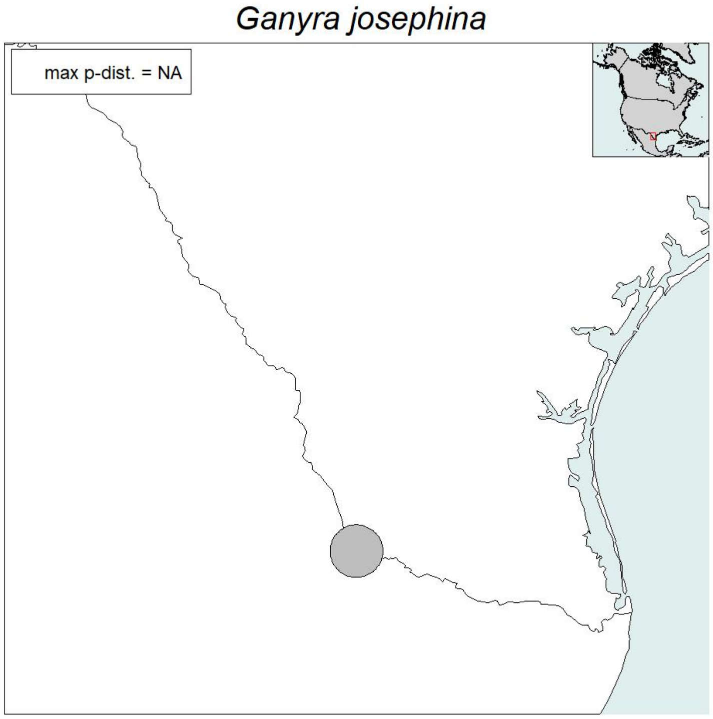

Figure 934: Map of *Ganyra josephina* showing the localities of the sequenced specimens. Nearby localities are grouped in pies. Due to the presence of a single haplotype PCoA projection was not done and a single grey colour was plotted on the map. Sequences= 1; Hap obs.= NA; Hap asympt.= NA; Hap % obs.= NA; GST= NaN; DST= NaN; HD= NA; ND= NA; max p-dist= NA.

Haplotype network analysis and bubble plot of *Ganyra josephina* were not possible. Sequences > 599 bp = 1.

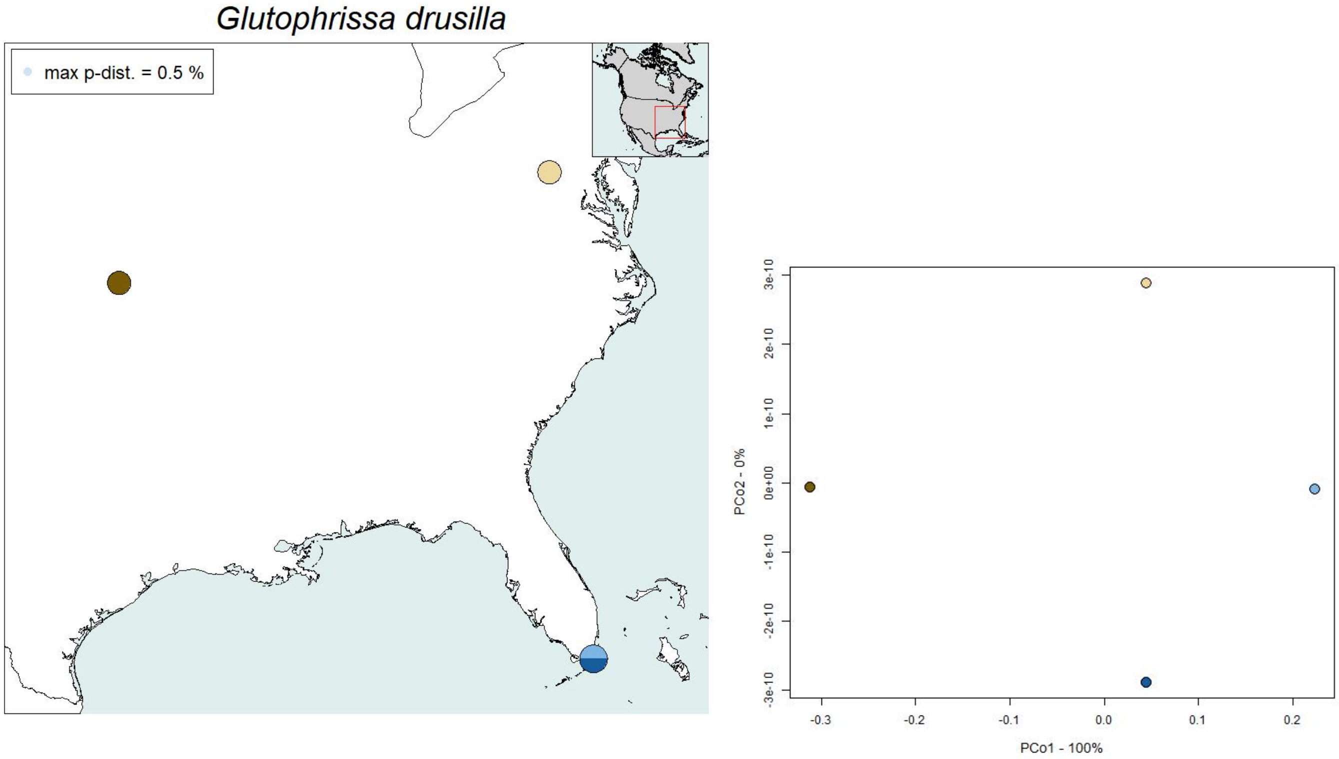

Figure 935 Map of *Glutophrissa drusilla* showing the localities of the sequenced specimens (left). Nearby localities are grouped in pies. Colours match the bidimensional colour space of the PCoA projection (right) of max p-dists among sequences (dots). Sequences= 4; Hap obs.= 3; Hap asympt.= NA; Hap % obs.= NA%; GST= NaN; DST= NaN; HD= NA; ND= NA; max p-dist= 0.5%.

Haplotype network analysis and bubble plot of *Glutophrissa drusilla* were not possible. Sequences > 599 bp = 0.

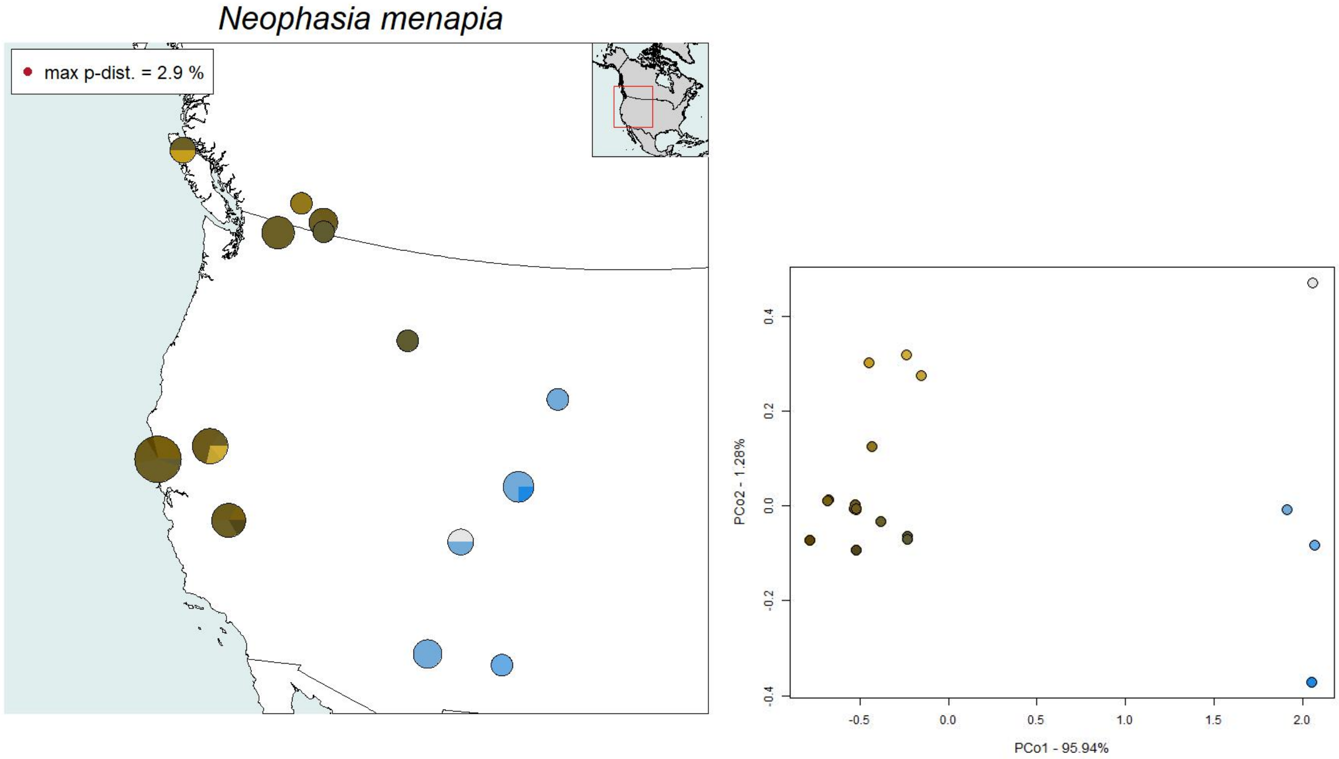

Figure 936 Map of *Neophasia menapia* showing the localities of the sequenced specimens (left). Nearby localities are grouped in pies. Colours match the bidimensional colour space of the PCoA projection (right) of max p-dists among sequences (dots). Sequences= 58; Hap obs.= 19; Hap asympt.= 102; Hap % obs.= 18.6%; GST= 0.754; DST= 0.0054; HD= 0.837; ND= 0.0088; max p-dist= 2.9%.

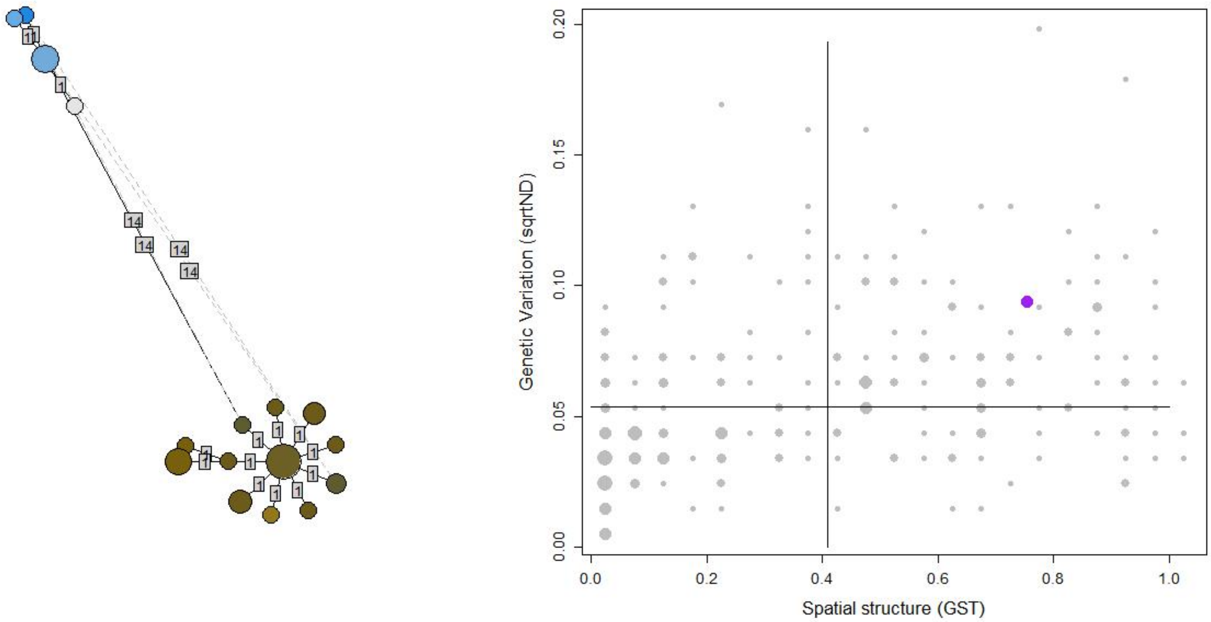

Figure 937: Haplotype network (left) of *Neophasia menapia* sequences > 599 bp with colours matching the PCoA colour space (above). The bubble plot for mt-DNA polymorphism (square root transformed nucleotide diversity) and spatial structure (GST) among all species in the atlas and values for *Neophasia menapia* (purple dot). The horizontal and vertical lines represent median values of nucleotide diversity and GST, respectively. Sequences > 599 bp= 55.

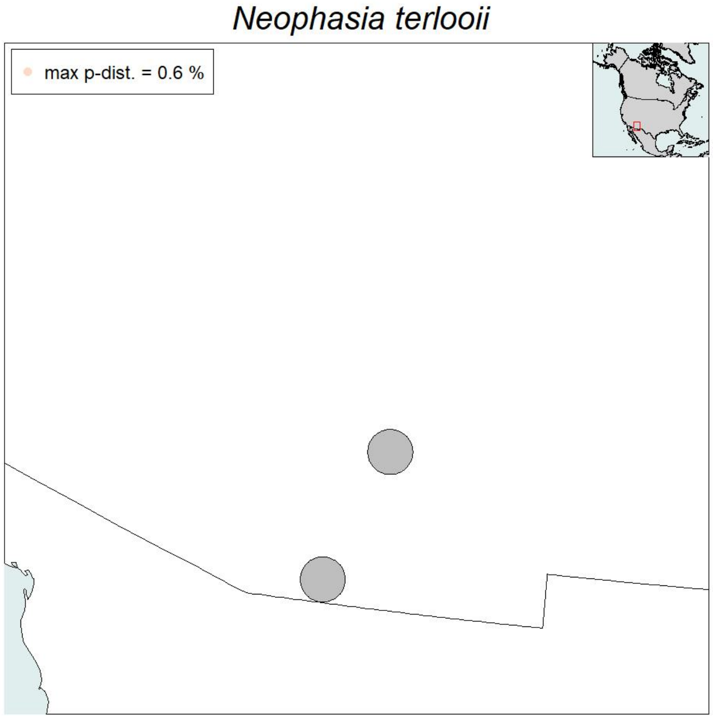

Figure 938: Map of *Neophasia terlooii* showing the localities of the sequenced specimens. Nearby localities are grouped in pies. Due to the presence of a single haplotype PCoA projection was not done and a single grey colour was plotted on the map. Sequences= 2; Hap obs.= 2; Hap asympt.= NA; Hap % obs.= NA%; GST= NaN; DST= NaN; HD= NA; ND= NA; max p-dist= 0.6%.

Haplotype network analysis and bubble plot of *Neophasia terlooii* were not possible. Sequences > 599 bp = 2.

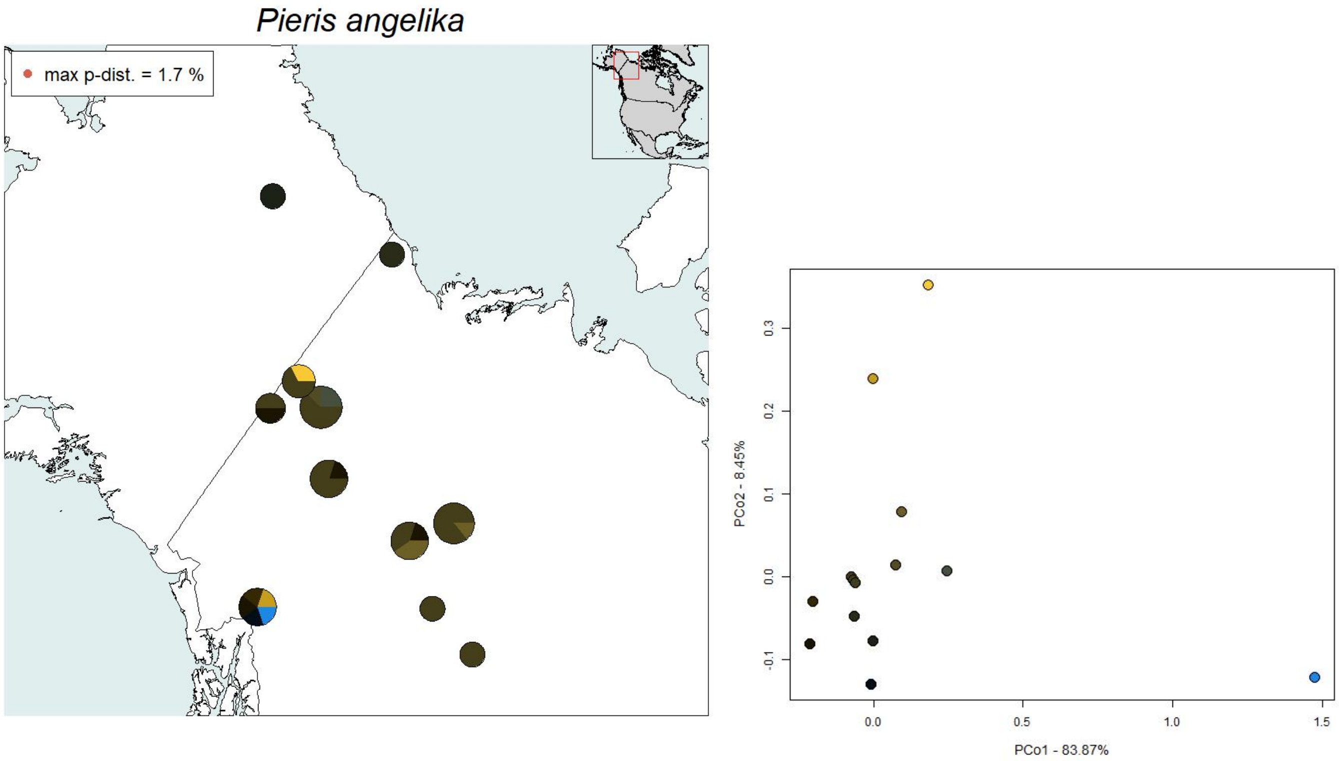

Figure 939 Map of *Pieris angelika* showing the localities of the sequenced specimens (left). Nearby localities are grouped in pies. Colours match the bidimensional colour space of the PCoA projection (right) of max p-dists among sequences (dots). Sequences= 39; Hap obs.= 9; Hap asympt.= 21.2; Hap % obs.= 42.5%; GST= 0.265; DST= 0.0006; HD= 0.582; ND= 0.002; max p-dist= 1.7%.

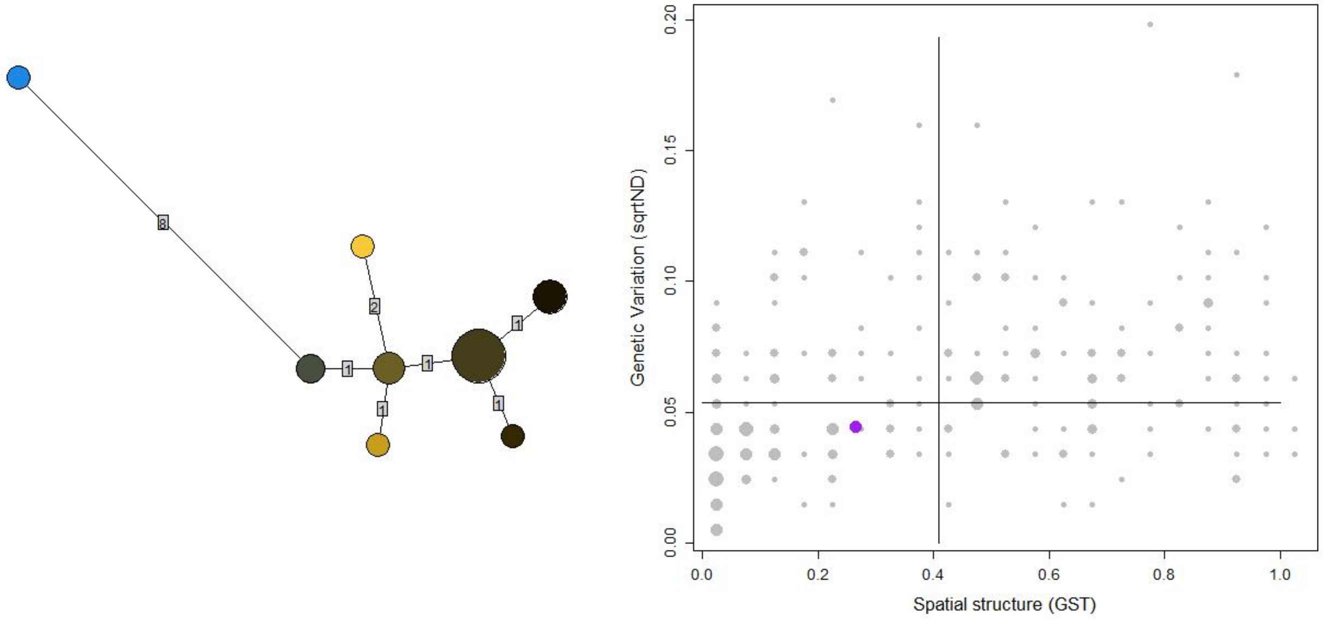

Figure 940: Haplotype network (left) of *Pieris angelika* sequences > 599 bp with colours matching the PCoA colour space (above). The bubble plot for mt-DNA polymorphism (square root transformed nucleotide diversity) and spatial structure (GST) among all species in the atlas and values for *Pieris angelika* (purple dot). The horizontal and vertical lines represent median values of nucleotide diversity and GST, respectively. Sequences > 599 bp= 37.

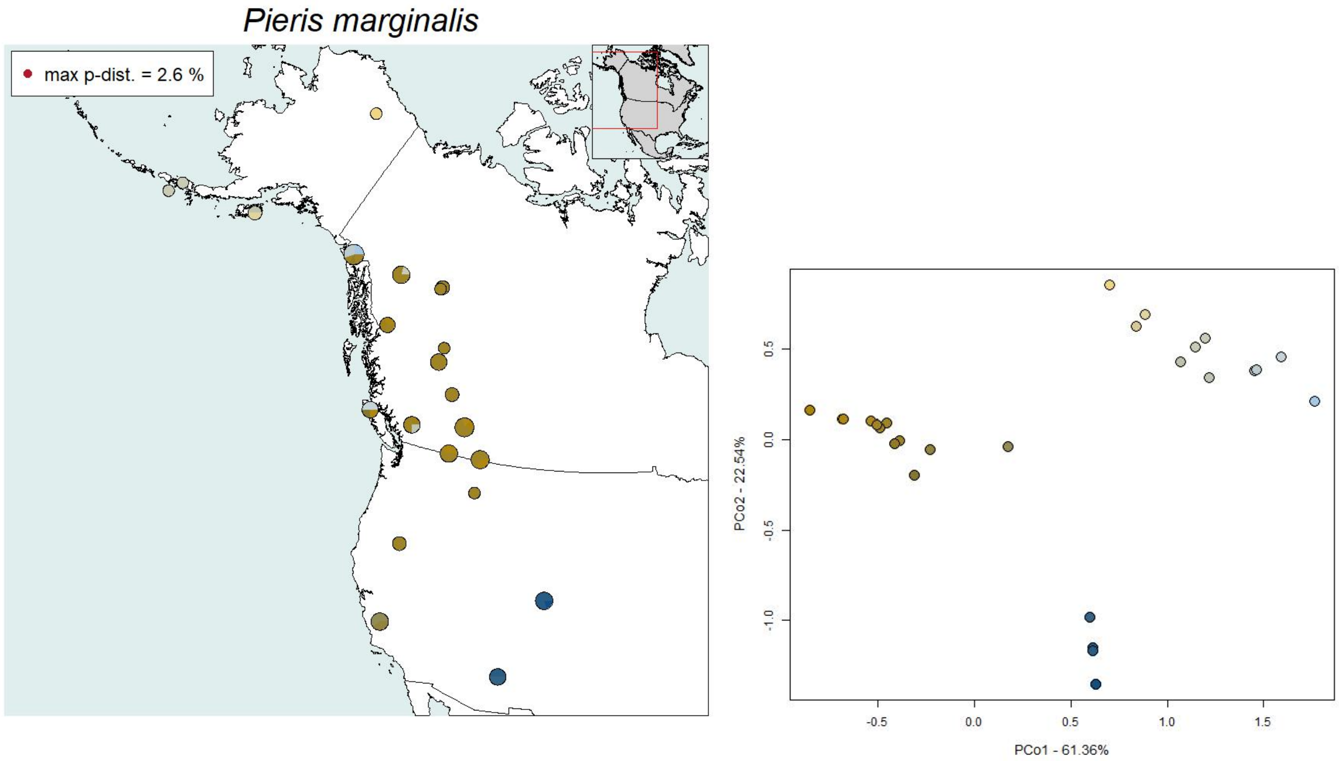

Figure 941 Map of *Pieris marginalis* showing the localities of the sequenced specimens (left). Nearby localities are grouped in pies. Colours match the bidimensional colour space of the PCoA projection (right) of max p-dists among sequences (dots). Sequences= 76; Hap obs.= 20; Hap asympt.= 26.7; Hap % obs.= 75%; GST= 0.468; DST= 0.0046; HD= 0.729; ND= 0.0102; max p-dist= 2.6%.

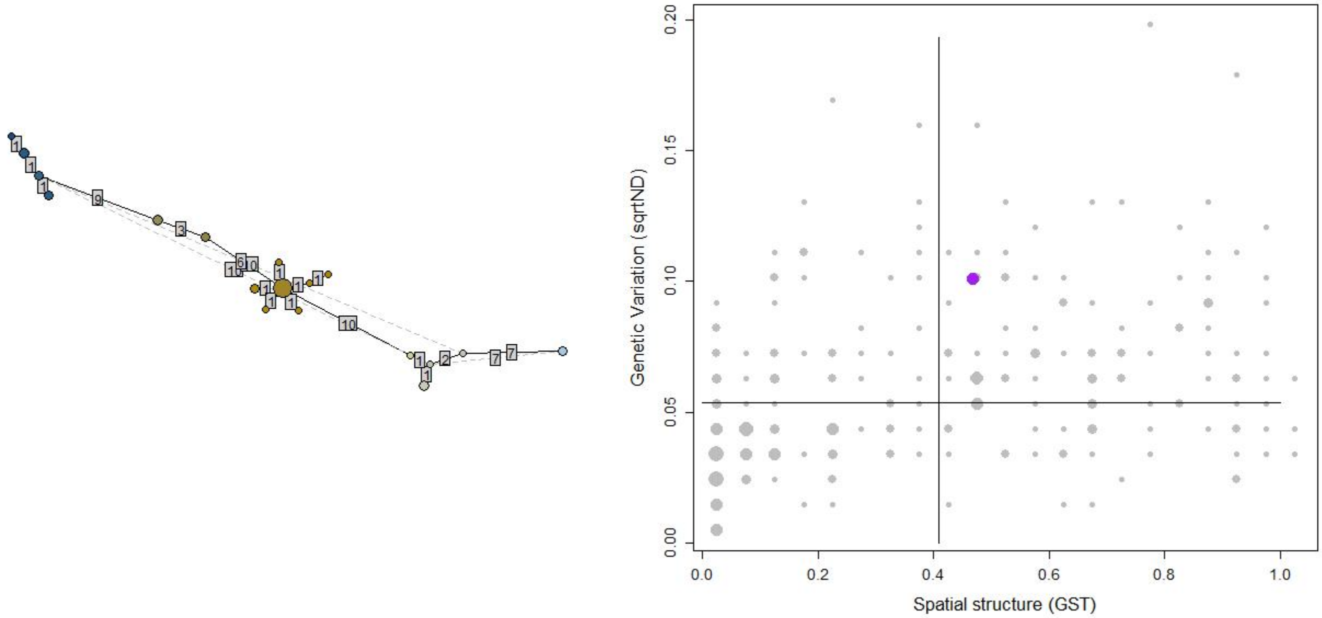

Figure 942: Haplotype network (left) of *Pieris marginalis* sequences > 599 bp with colours matching the PCoA colour space (above). The bubble plot for mt-DNA polymorphism (square root transformed nucleotide diversity) and spatial structure (GST) among all species in the atlas and values for *Pieris marginalis* (purple dot). The horizontal and vertical lines represent median values of nucleotide diversity and GST, respectively. Sequences > 599 bp= 65.

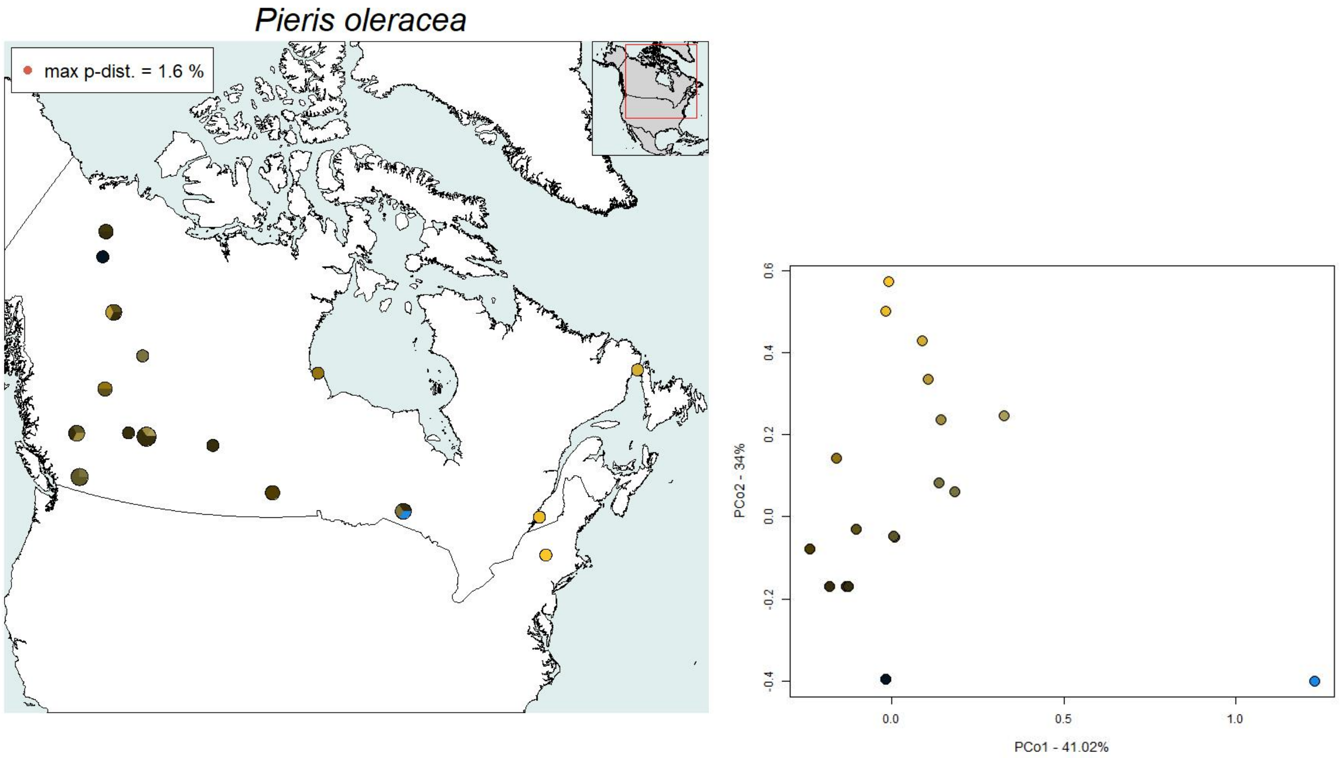

Figure 943 Map of *Pieris oleracea* showing the localities of the sequenced specimens (left). Nearby localities are grouped in pies. Colours match the bidimensional colour space of the PCoA projection (right) of max p-dists among sequences (dots). Sequences= 33; Hap obs.= 15; Hap asympt.= 21.2; Hap % obs.= 70.7%; GST= 0.125; DST= 0.0005; HD= 0.875; ND= 0.0043; max p-dist= 1.6%.

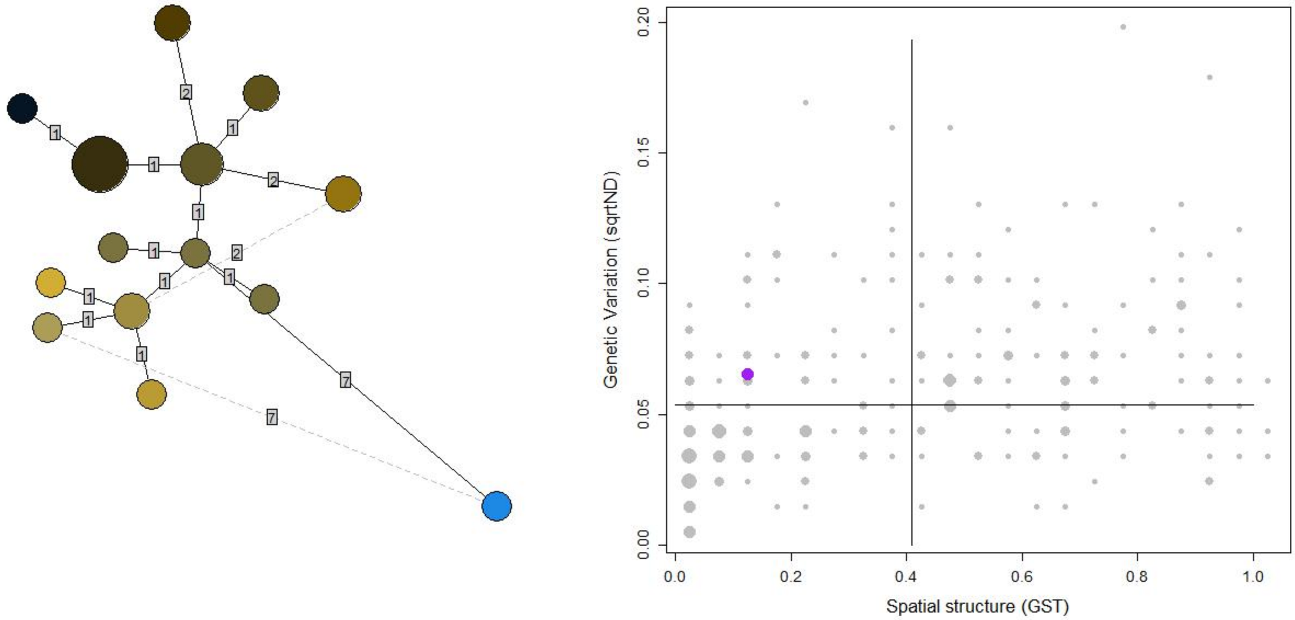

Figure 944: Haplotype network (left) of *Pieris oleracea* sequences > 599 bp with colours matching the PCoA colour space (above). The bubble plot for mt-DNA polymorphism (square root transformed nucleotide diversity) and spatial structure (GST) among all species in the atlas and values for *Pieris oleracea* (purple dot). The horizontal and vertical lines represent median values of nucleotide diversity and GST, respectively. Sequences > 599 bp= 31.

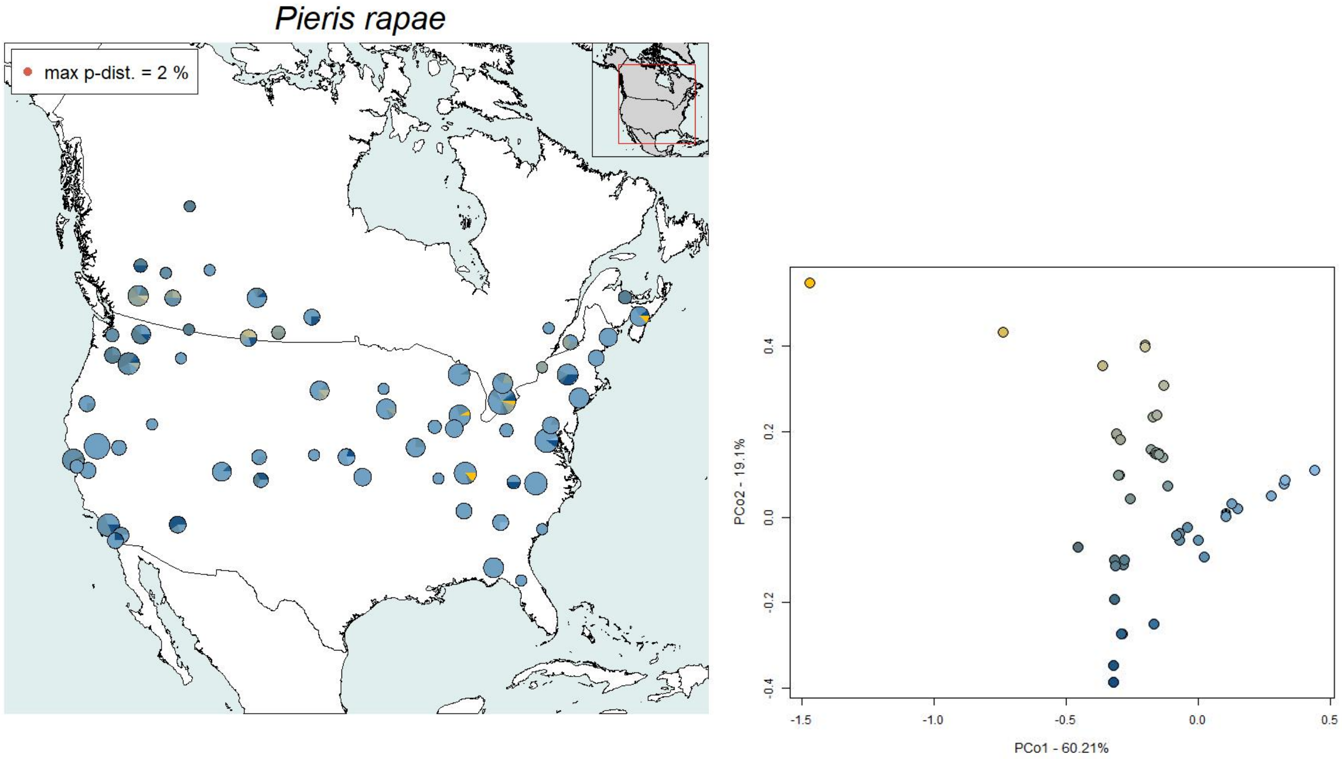

Figure 945 Map of *Pieris rapae* showing the localities of the sequenced specimens (left). Nearby localities are grouped in pies. Colours match the bidimensional colour space of the PCoA projection (right) of max p-dists among sequences (dots). Sequences= 416; Hap obs.= 30; Hap asympt.= 83.9; Hap % obs.= 35.8%; GST= 0.081; DST= 0.0002; HD= 0.567; ND= 0.002; max p-dist= 2%.

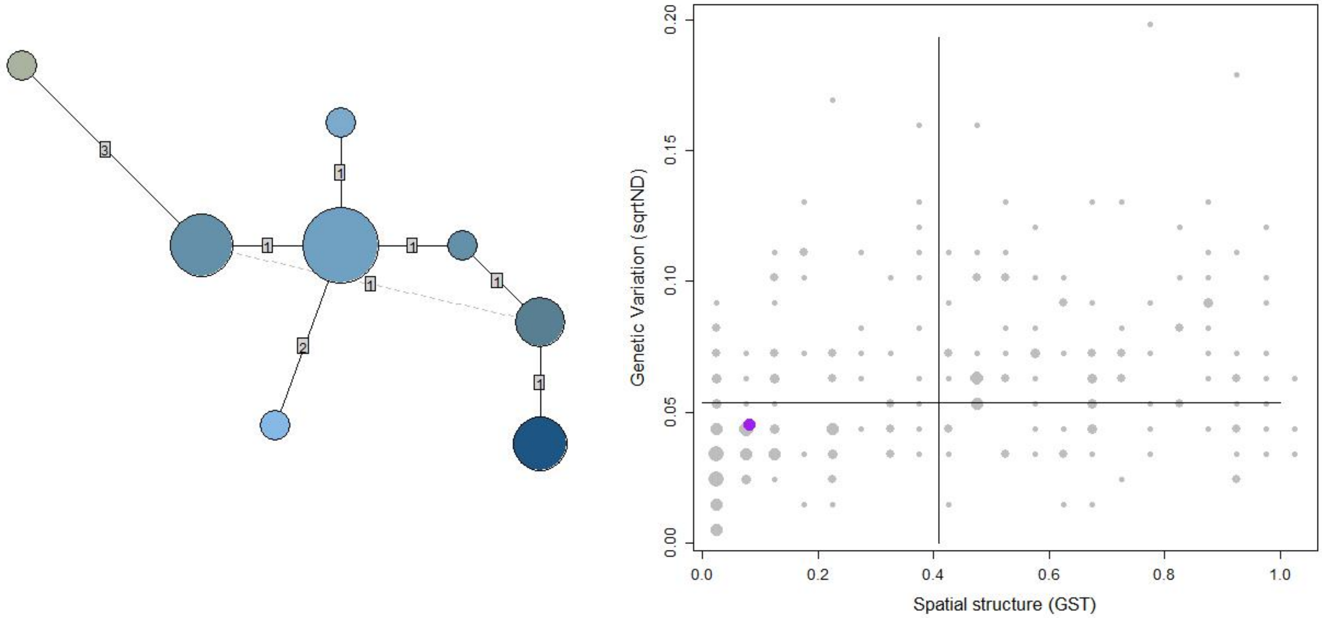

Figure 946: Haplotype network (left) of *Pieris rapae* sequences > 599 bp with colours matching the PCoA colour space (above). The bubble plot for mt-DNA polymorphism (square root transformed nucleotide diversity) and spatial structure (GST) among all species in the atlas and values for *Pieris rapae* (purple dot). The horizontal and vertical lines represent median values of nucleotide diversity and GST, respectively. Sequences > 599 bp= 84.

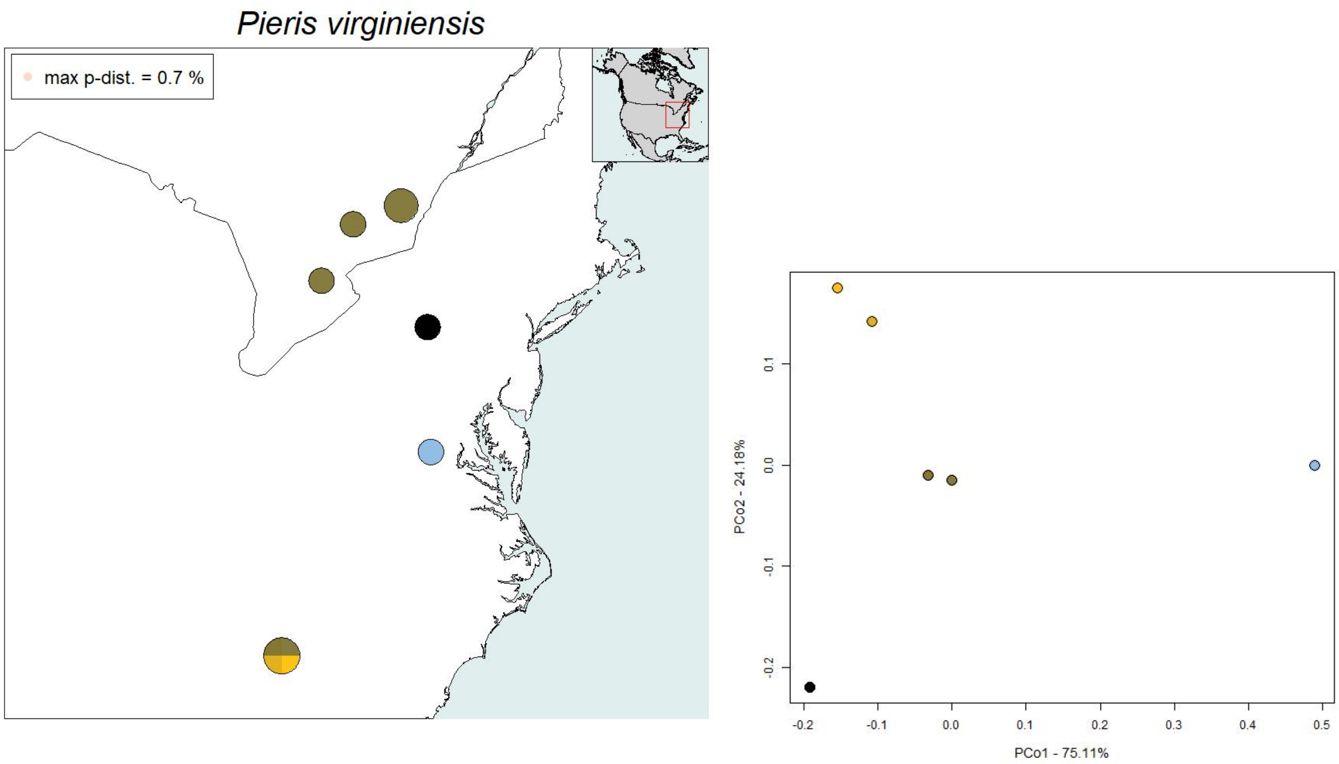

Figure 947 Map of *Pieris virginiensis* showing the localities of the sequenced specimens (left). Nearby localities are grouped in pies. Colours match the bidimensional colour space of the PCoA projection (right) of max p-dists among sequences (dots). Sequences= 11; Hap obs.= 4; Hap asympt.= 5.8; Hap % obs.= 68.8%; GST= NaN; DST= NaN; HD= 0.6; ND= 0.0017; max p-dist= 0.7%.

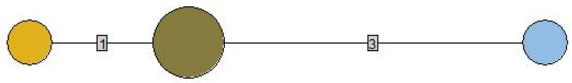

Figure 948: Haplotype network of *Pieris virginiensis*. Sequences > 599 bp= 8.

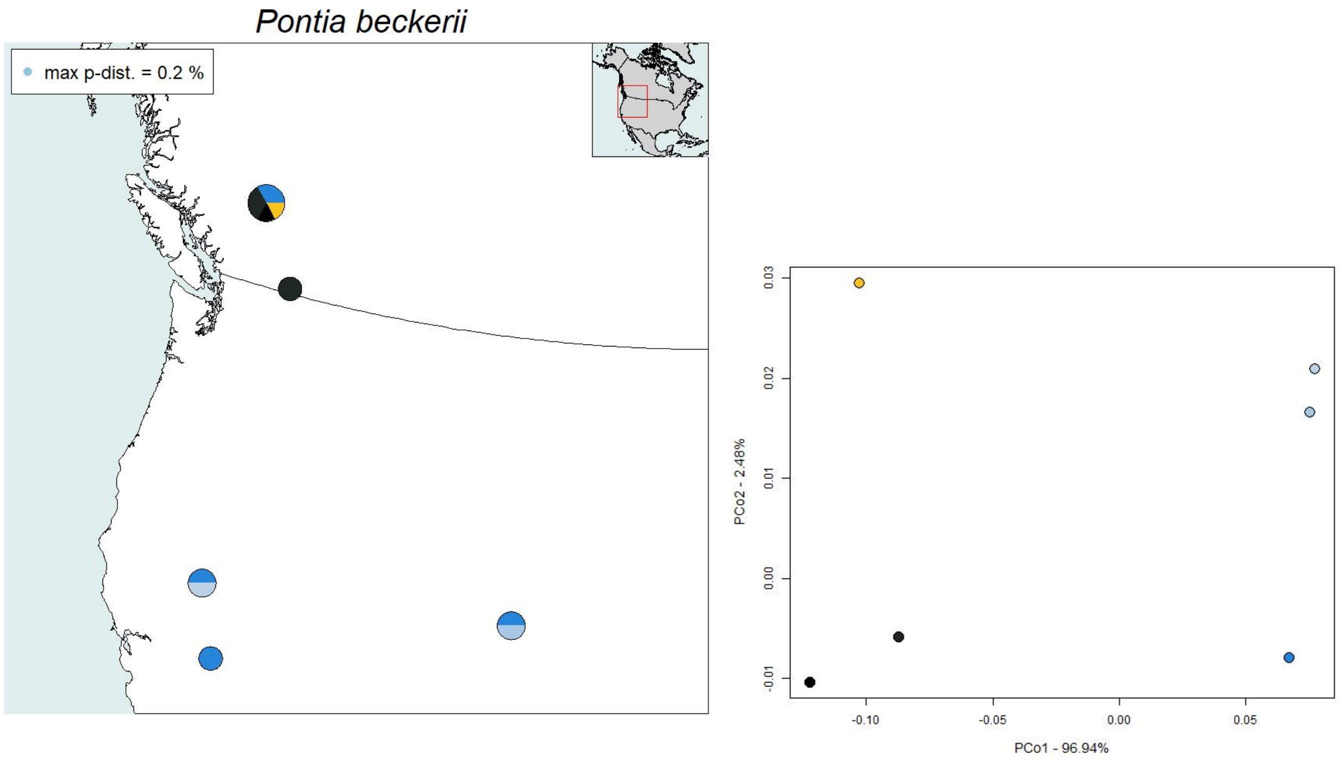

Figure 949 Map of *Pontia beckerii* showing the localities of the sequenced specimens (left). Nearby localities are grouped in pies. Colours match the bidimensional colour space of the PCoA projection (right) of max p-dists among sequences (dots). Sequences= 12; Hap obs.= 2; Hap asympt.= 2; Hap % obs.= 100%; GST= NaN; DST= NaN; HD= 0.53; ND= 0.0009; max p-dist= 0.2%.

Haplotype network analysis and bubble plot of *Pontia beckerii* were not possible. Sequences > 599 bp = 10.

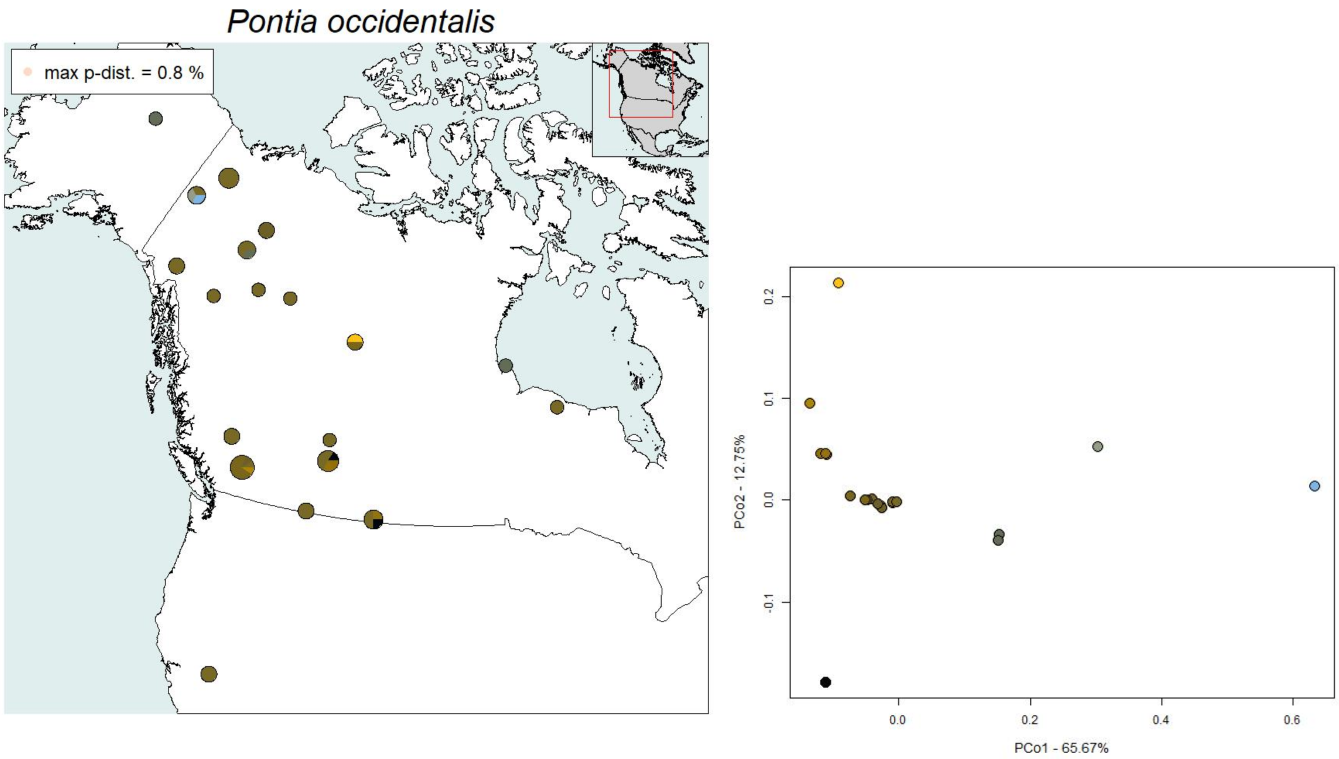

Figure 950 Map of *Pontia occidentalis* showing the localities of the sequenced specimens (left). Nearby localities are grouped in pies. Colours match the bidimensional colour space of the PCoA projection (right) of max p-dists among sequences (dots). Sequences= 50; Hap obs.= 11; Hap asympt.= 19.8; Hap % obs.= 55.5%; GST= 0.217; DST= 0.0002; HD= 0.624; ND= 0.001; max p-dist= 0.8%.

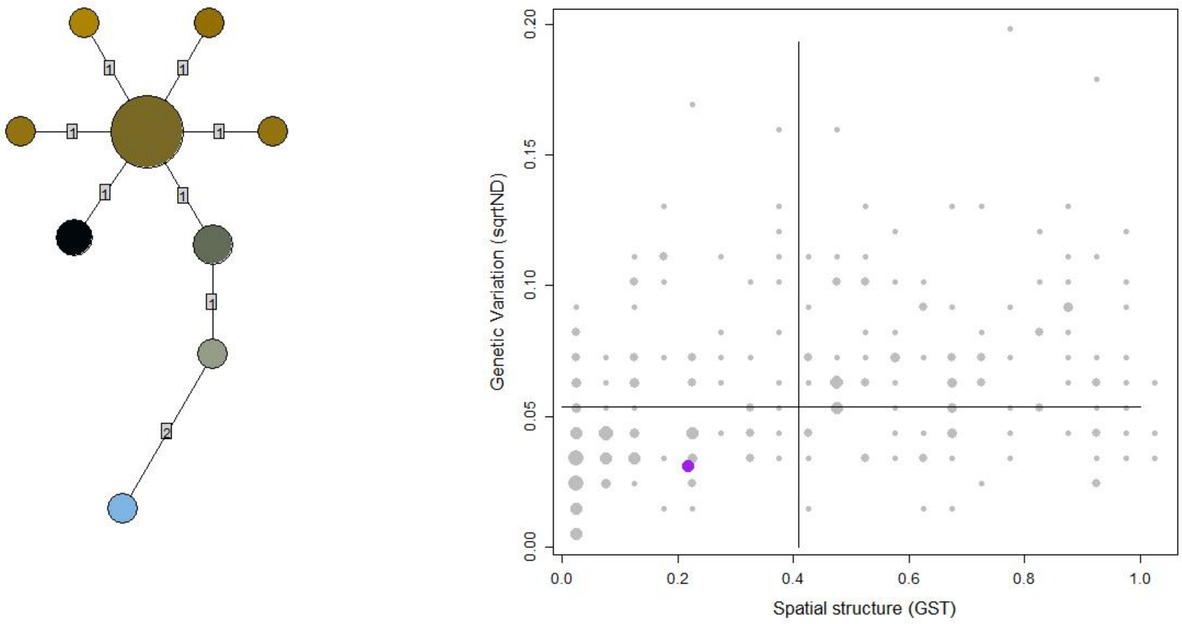

Figure 951: Haplotype network (left) of *Pontia occidentalis* sequences > 599 bp with colours matching the PCoA colour space (above). The bubble plot for mt-DNA polymorphism (square root transformed nucleotide diversity) and spatial structure (GST) among all species in the atlas and values for *Pontia occidentalis* (purple dot). The horizontal and vertical lines represent median values of nucleotide diversity and GST, respectively. Sequences > 599 bp= 42.

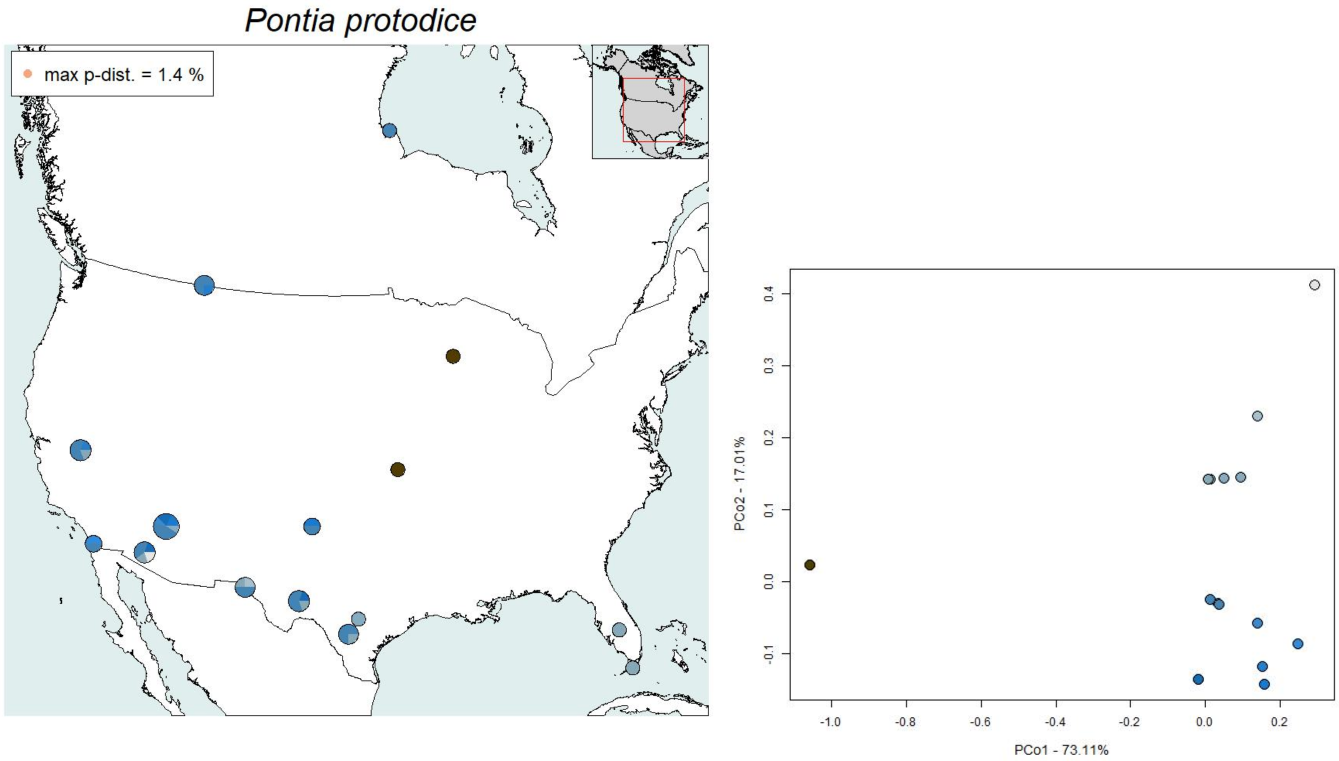

Figure 952 Map of *Pontia protodice* showing the localities of the sequenced specimens (left). Nearby localities are grouped in pies. Colours match the bidimensional colour space of the PCoA projection (right) of max p-dists among sequences (dots). Sequences= 48; Hap obs.= 9; Hap asympt.= 16.8; Hap % obs.= 53.5%; GST= 0.006; DST= 0; HD= 0.671; ND= 0.0021; max p-dist= 1.4%.

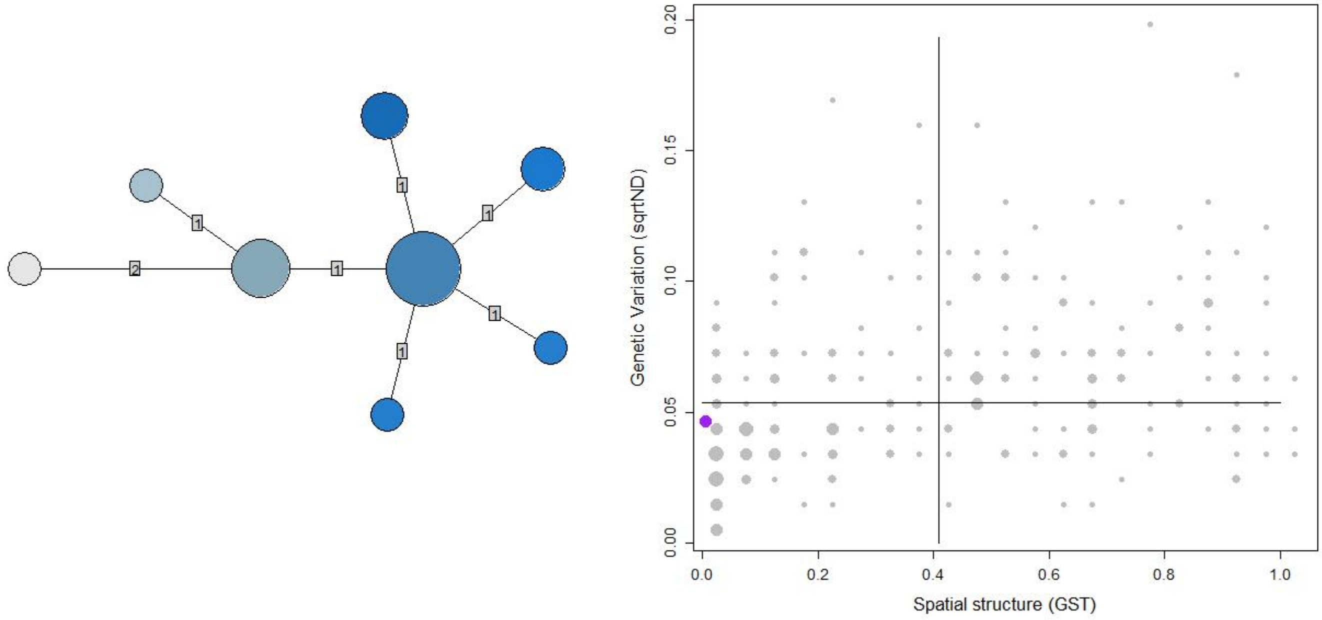

Figure 953: Haplotype network (left) of *Pontia protodice* sequences > 599 bp with colours matching the PCoA colour space (above). The bubble plot for mt-DNA polymorphism (square root transformed nucleotide diversity) and spatial structure (GST) among all species in the atlas and values for *Pontia protodice* (purple dot). The horizontal and vertical lines represent median values of nucleotide diversity and GST, respectively. Sequences > 599 bp= 44.

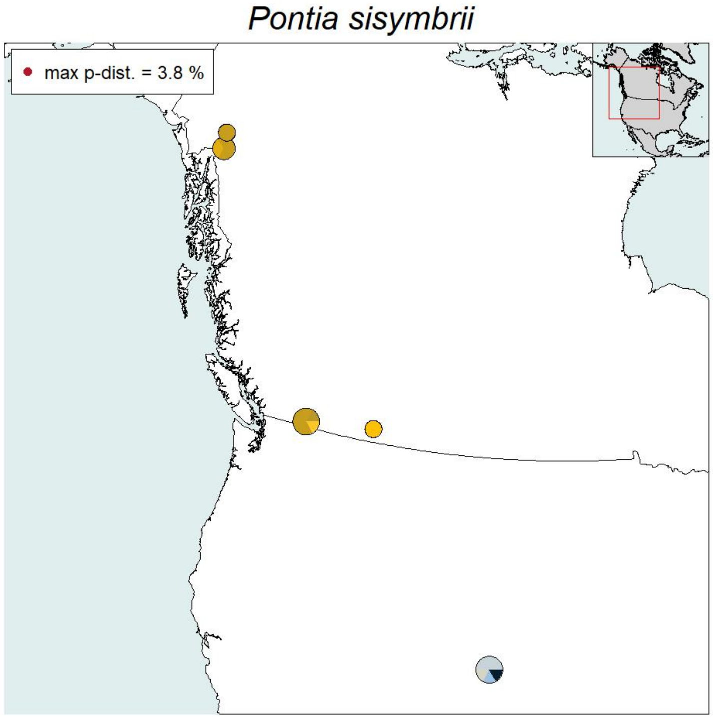

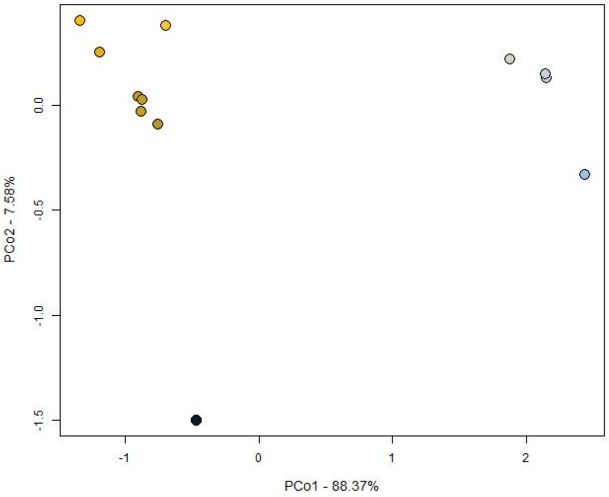

Figure 954 Map of *Pontia sisymbrii* showing the localities of the sequenced specimens (left). Nearby localities are grouped in pies. Colours match the bidimensional colour space of the PCoA projection (right) of max p-dists among sequences (dots). Sequences= 17; Hap obs.= 12; Hap asympt.= 59.1; Hap % obs.= 20.3%; GST= 0.672; DST= 0.0112; HD= 0.919; ND= 0.0166; max p-dist= 3.8%.

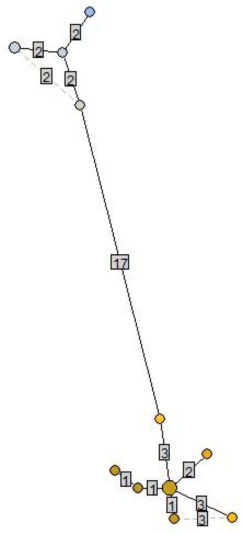

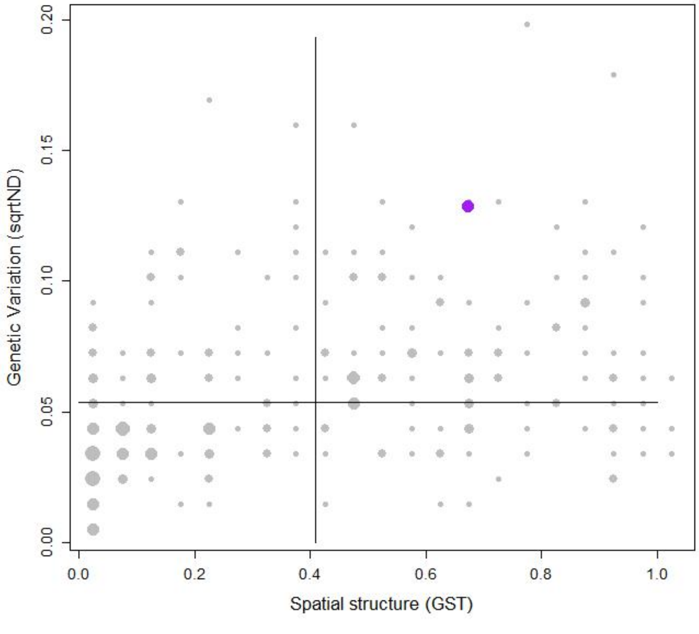

Figure 955: Haplotype network (left) of *Pontia sisymbrii* sequences > 599 bp with colours matching the PCoA colour space (above). The bubble plot for mt-DNA polymorphism (square root transformed nucleotide diversity) and spatial structure (GST) among all species in the atlas and values for *Pontia sisymbrii* (purple dot). The horizontal and vertical lines represent median values of nucleotide diversity and GST, respectively. Sequences > 599 bp= 16.

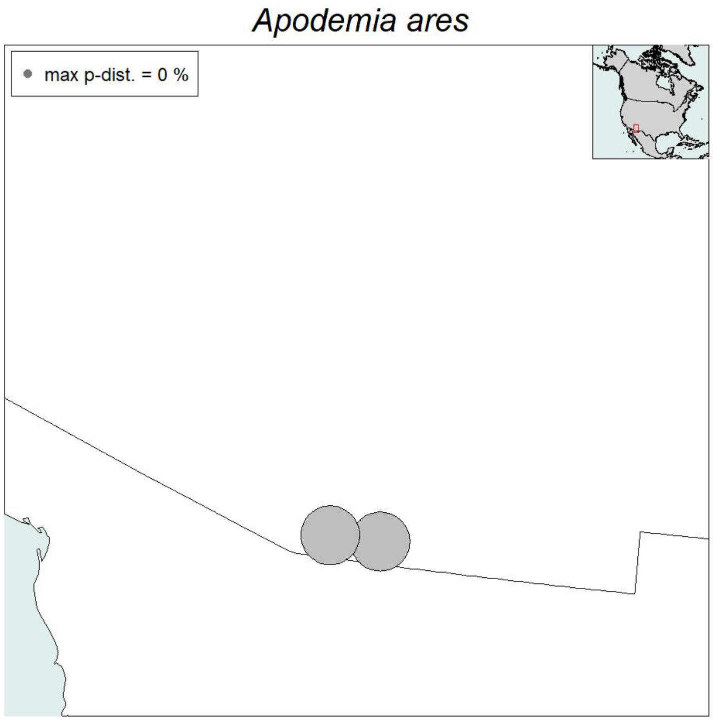

Figure 956: Map of *Apodemia ares* showing the localities of the sequenced specimens. Nearby localities are grouped in pies. Due to the presence of a single haplotype PCoA projection was not done and a single grey colour was plotted on the map. Sequences= 4; Hap obs.= 1; Hap asympt.= NA; Hap % obs.= NA%; GST= NaN; DST= NaN; HD= NA; ND= NA; max p-dist= 0%.

Haplotype network analysis and bubble plot of *Apodemia ares* were not possible. Sequences > 599 bp = 4.

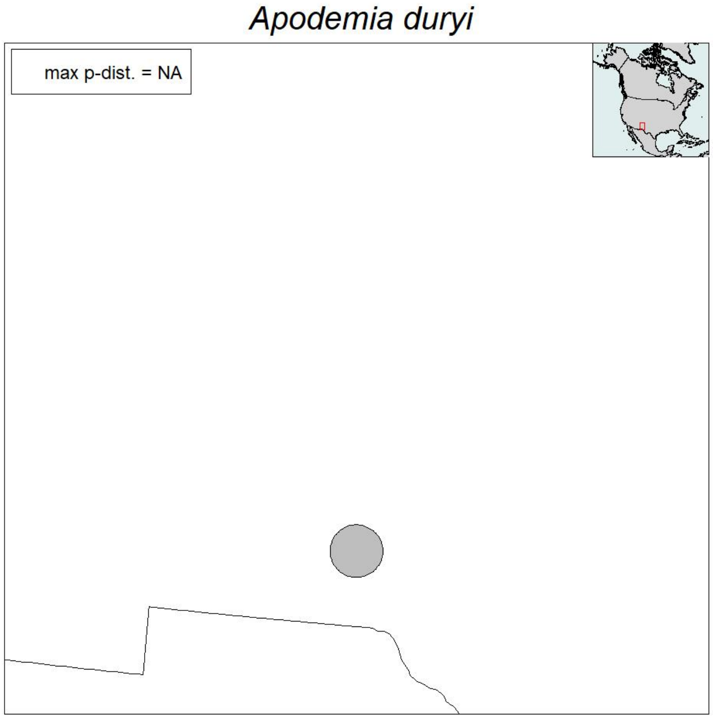

Figure 957: Map of *Apodemia duryi* showing the localities of the sequenced specimens. Nearby localities are grouped in pies. Due to the presence of a single haplotype PCoA projection was not done and a single grey colour was plotted on the map. Sequences= 1; Hap obs.= NA; Hap asympt.= NA; Hap % obs.= NA; GST= NaN; DST= NaN; HD= NA; ND= NA; max p-dist= NA.

Haplotype network analysis and bubble plot of *Apodemia duryi* were not possible. Sequences > 599 bp = 1.

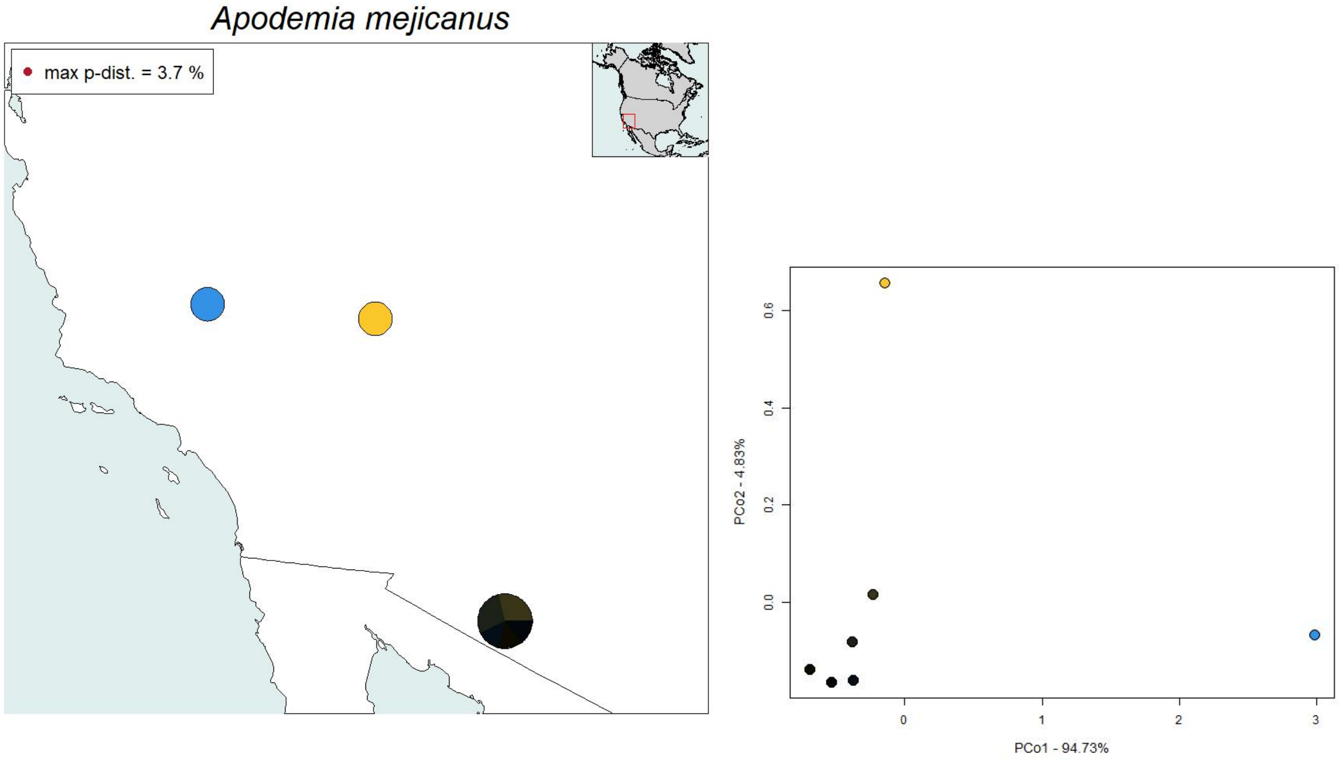

Figure 958 Map of *Apodemia mejicanus* showing the localities of the sequenced specimens (left). Nearby localities are grouped in pies. Colours match the bidimensional colour space of the PCoA projection (right) of max p-dists among sequences (dots). Sequences= 9; Hap obs.= 6; Hap asympt.= NA; Hap % obs.= NA%; GST= NaN; DST= NaN; HD= NA; ND= NA; max p-dist= 3.7%.

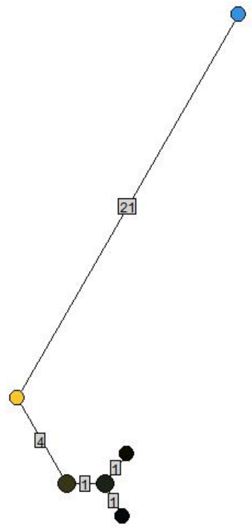

Figure 959: Haplotype network of *Apodemia mejicanus*. Sequences > 599 bp= 8.

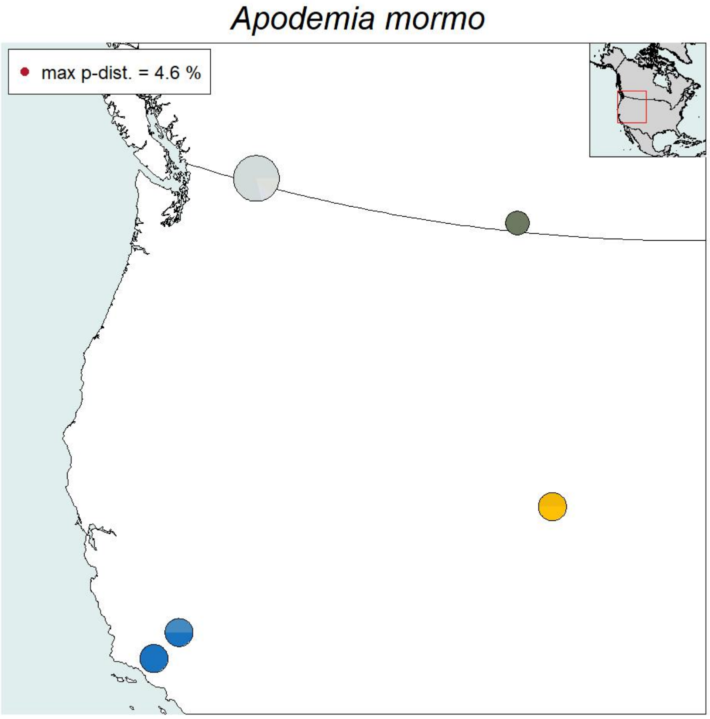

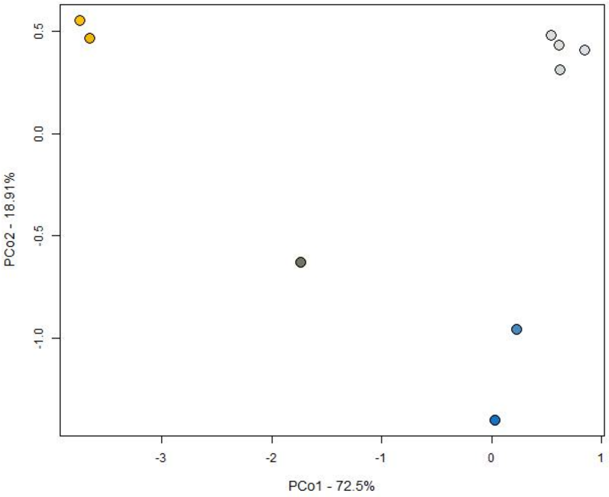

Figure 960 Map of *Apodemia mormo* showing the localities of the sequenced specimens (left). Nearby localities are grouped in pies. Colours match the bidimensional colour space of the PCoA projection (right) of max p-dists among sequences (dots). Sequences= 21; Hap obs.= 5; Hap asympt.= 6.9; Hap % obs.= 72.4%; GST= 0.584; DST= 0.0038; HD= 0.548; ND= 0.015; max p-dist= 4.6%.

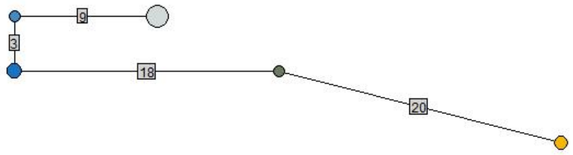

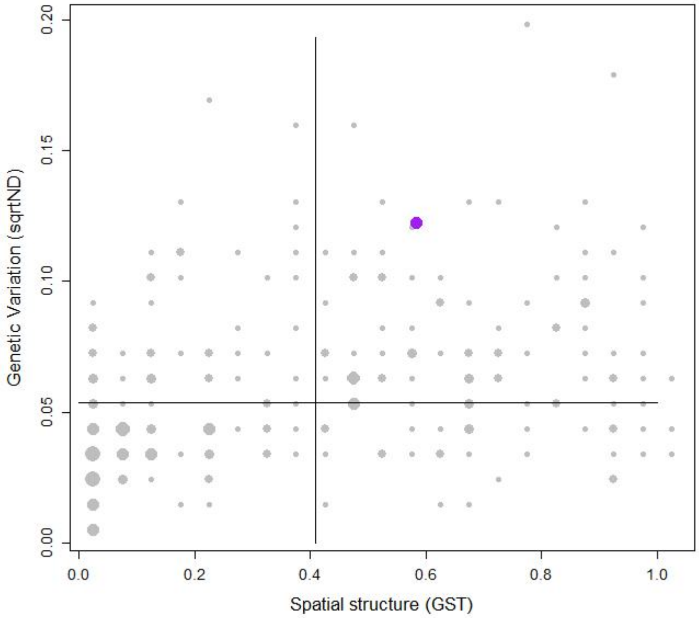

Figure 961: Haplotype network (left) of *Apodemia mormo* sequences > 599 bp with colours matching the PCoA colour space (above). The bubble plot for mt-DNA polymorphism (square root transformed nucleotide diversity) and spatial structure (GST) among all species in the atlas and values for *Apodemia mormo* (purple dot). The horizontal and vertical lines represent median values of nucleotide diversity and GST, respectively. Sequences > 599 bp= 20.

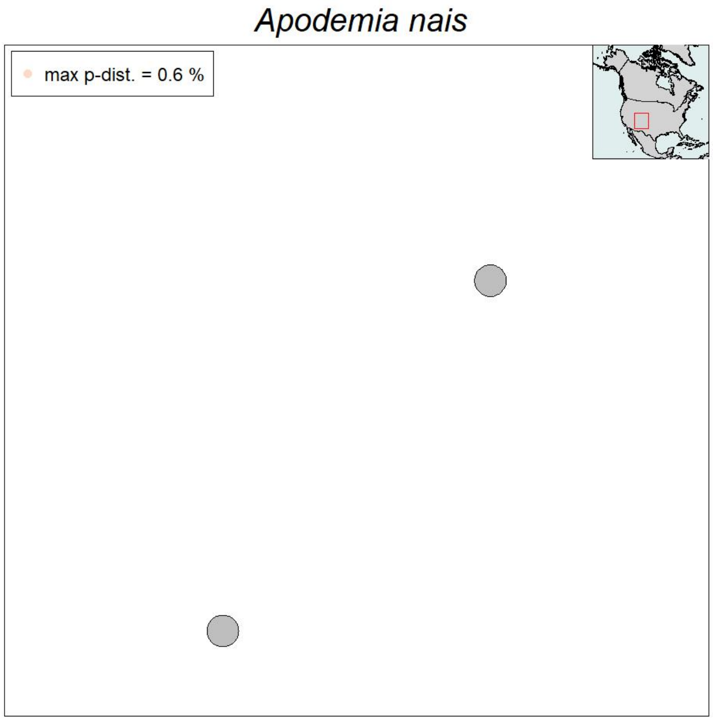

Figure 962: Map of *Apodemia nais* showing the localities of the sequenced specimens. Nearby localities are grouped in pies. Due to the presence of a single haplotype PCoA projection was not done and a single grey colour was plotted on the map. Sequences= 2; Hap obs.= 2; Hap asympt.= NA; Hap % obs.= NA%; GST= NaN; DST= NaN; HD= NA; ND= NA; max p-dist= 0.6%.

Haplotype network analysis and bubble plot of *Apodemia nais* were not possible. Sequences > 599 bp = 2.

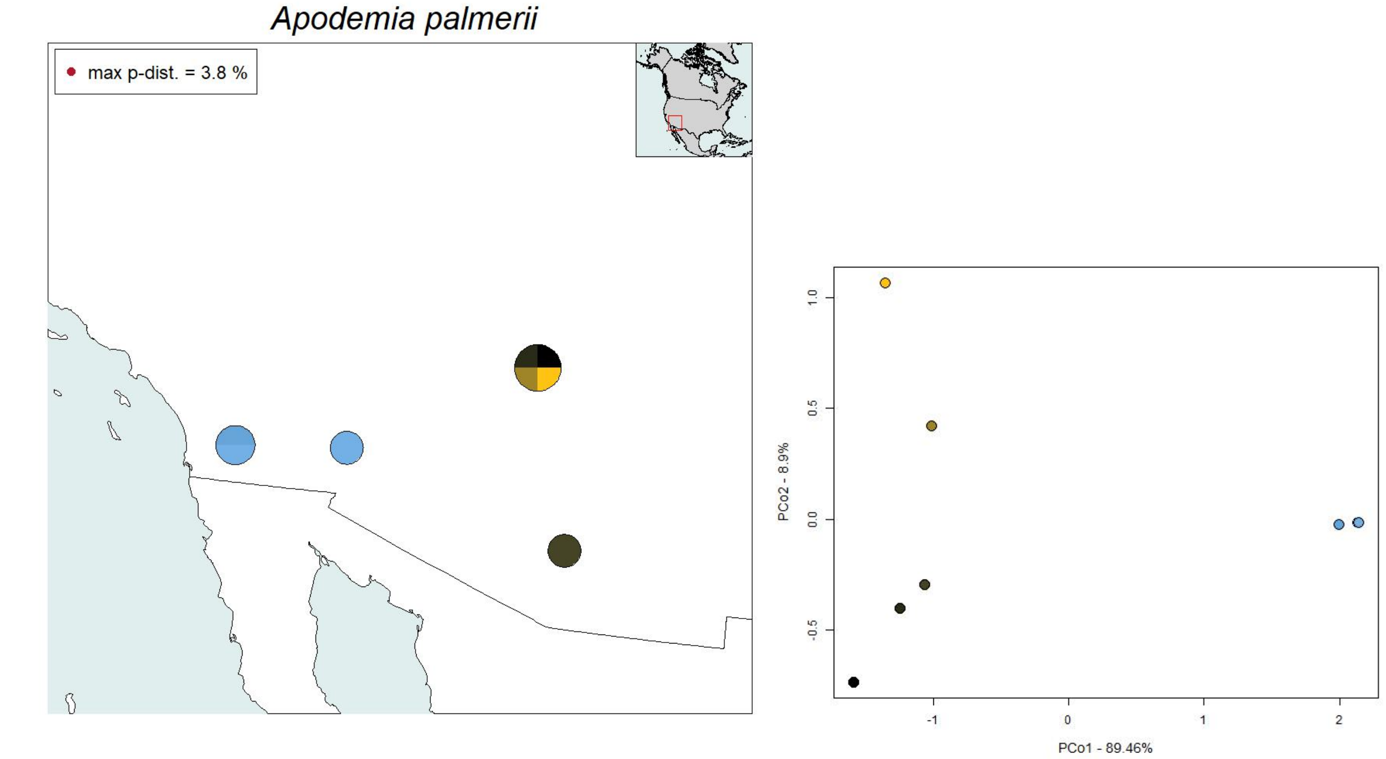

Figure 963 Map of *Apodemia palmerii* showing the localities of the sequenced specimens (left). Nearby localities are grouped in pies. Colours match the bidimensional colour space of the PCoA projection (right) of max p-dists among sequences (dots). Sequences= 8; Hap obs.= 8; Hap asympt.= NA; Hap % obs.= NA%; GST= NaN; DST= NaN; HD= NA; ND= NA; max p-dist= 3.8%.

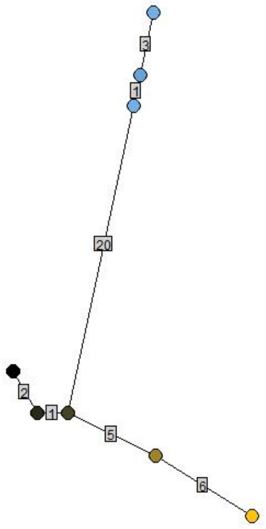

Figure 964: Haplotype network of *Apodemia palmerii*. Sequences > 599 bp= 8.

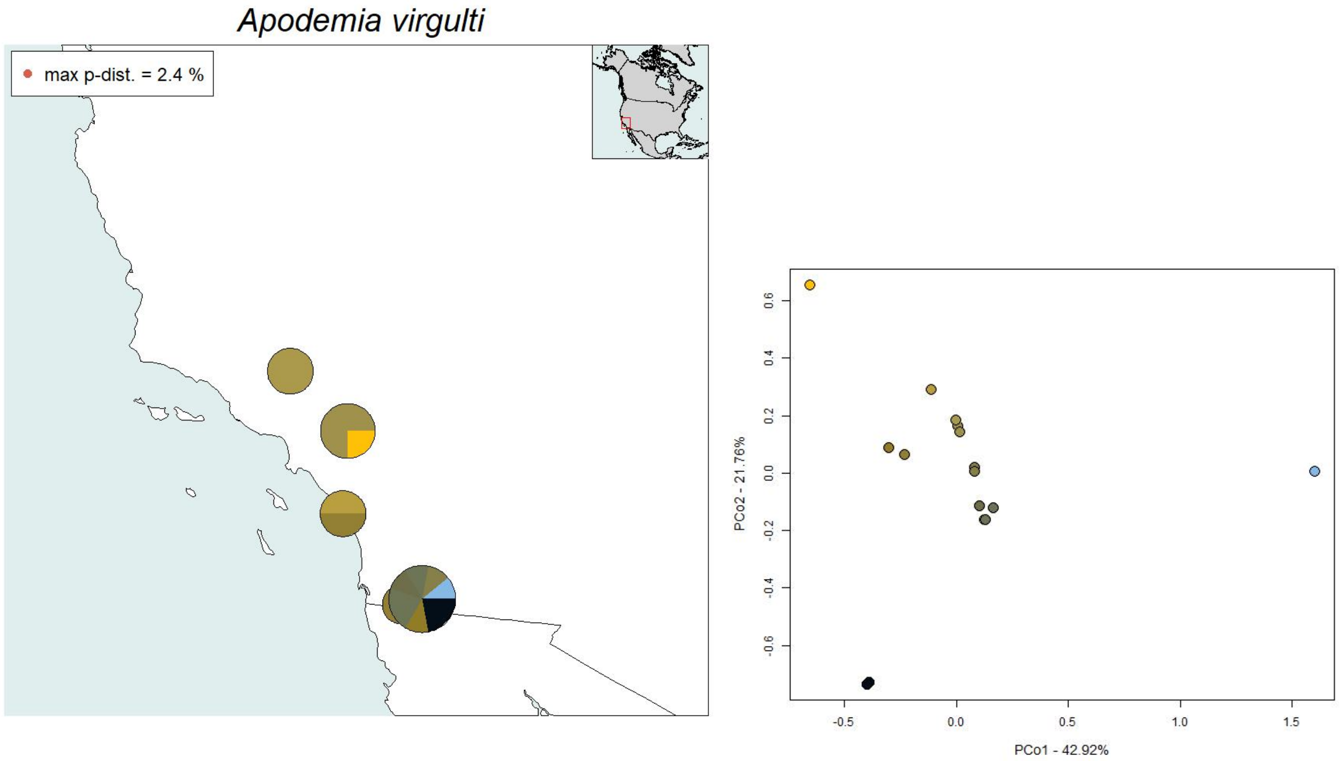

Figure 965 Map of *Apodemia virgulti* showing the localities of the sequenced specimens (left). Nearby localities are grouped in pies. Colours match the bidimensional colour space of the PCoA projection (right) of max p-dists among sequences (dots). Sequences= 19; Hap obs.= 11; Hap asympt.= 19.5; Hap % obs.= 56.3%; GST= 0.048; DST= 0.0004; HD= 0.936; ND= 0.008; max p-dist= 2.4%.

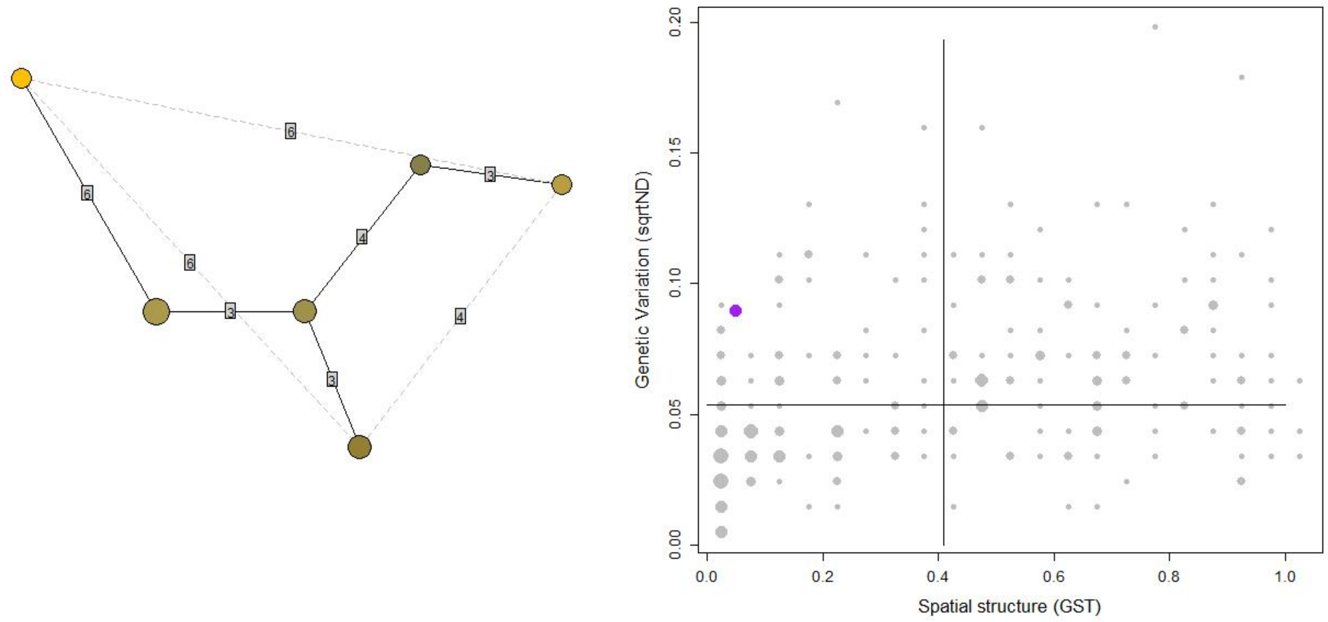

Figure 966: Haplotype network (left) of *Apodemia virgulti* sequences > 599 bp with colours matching the PCoA colour space (above). The bubble plot for mt-DNA polymorphism (square root transformed nucleotide diversity) and spatial structure (GST) among all species in the atlas and values for *Apodemia virgulti* (purple dot). The horizontal and vertical lines represent median values of nucleotide diversity and GST, respectively. Sequences > 599 bp= 10.

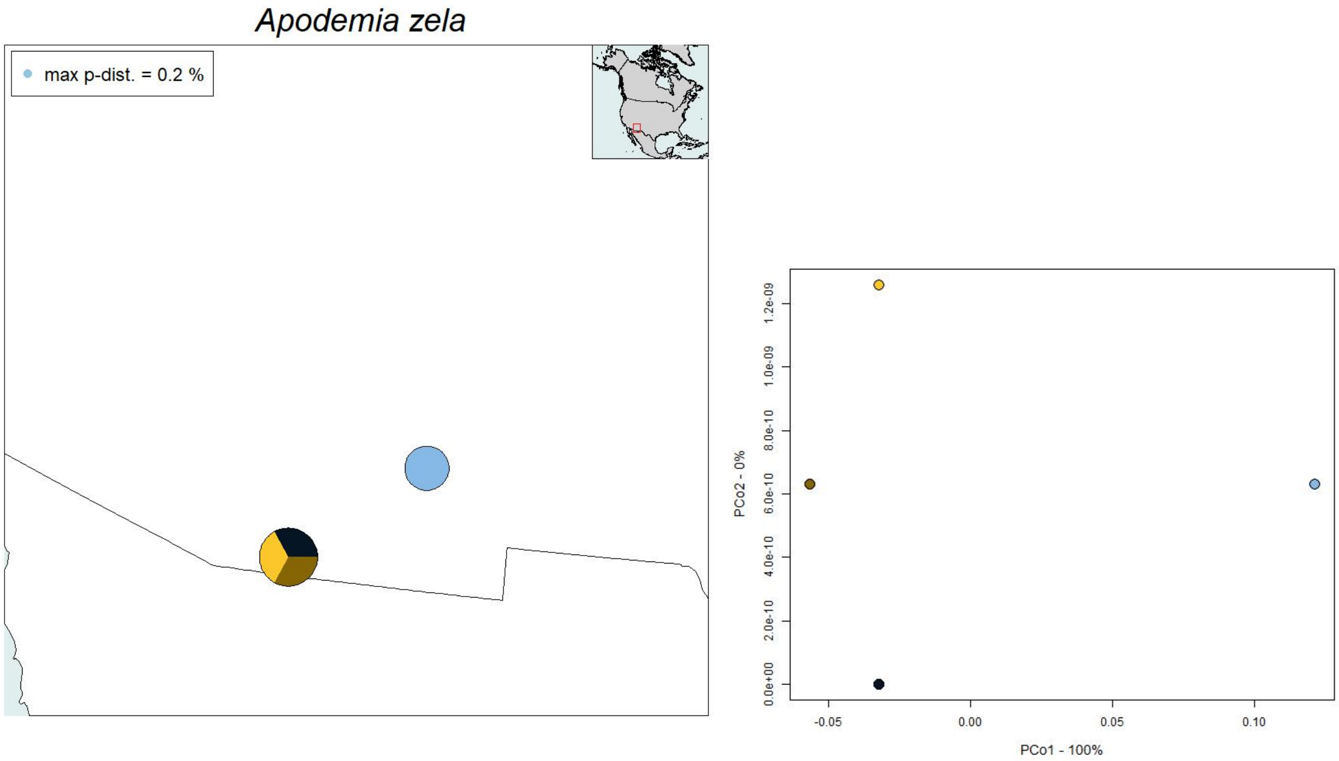

Figure 967 Map of *Apodemia zela* showing the localities of the sequenced specimens (left). Nearby localities are grouped in pies. Colours match the bidimensional colour space of the PCoA projection (right) of max p-dists among sequences (dots). Sequences= 4; Hap obs.= 2; Hap asympt.= NA; Hap % obs.= NA%; GST= NaN; DST= NaN; HD= NA; ND= NA; max p-dist= 0.2%.

Haplotype network analysis and bubble plot of *Apodemia zela* were not possible. Sequences > 599 bp = 3.

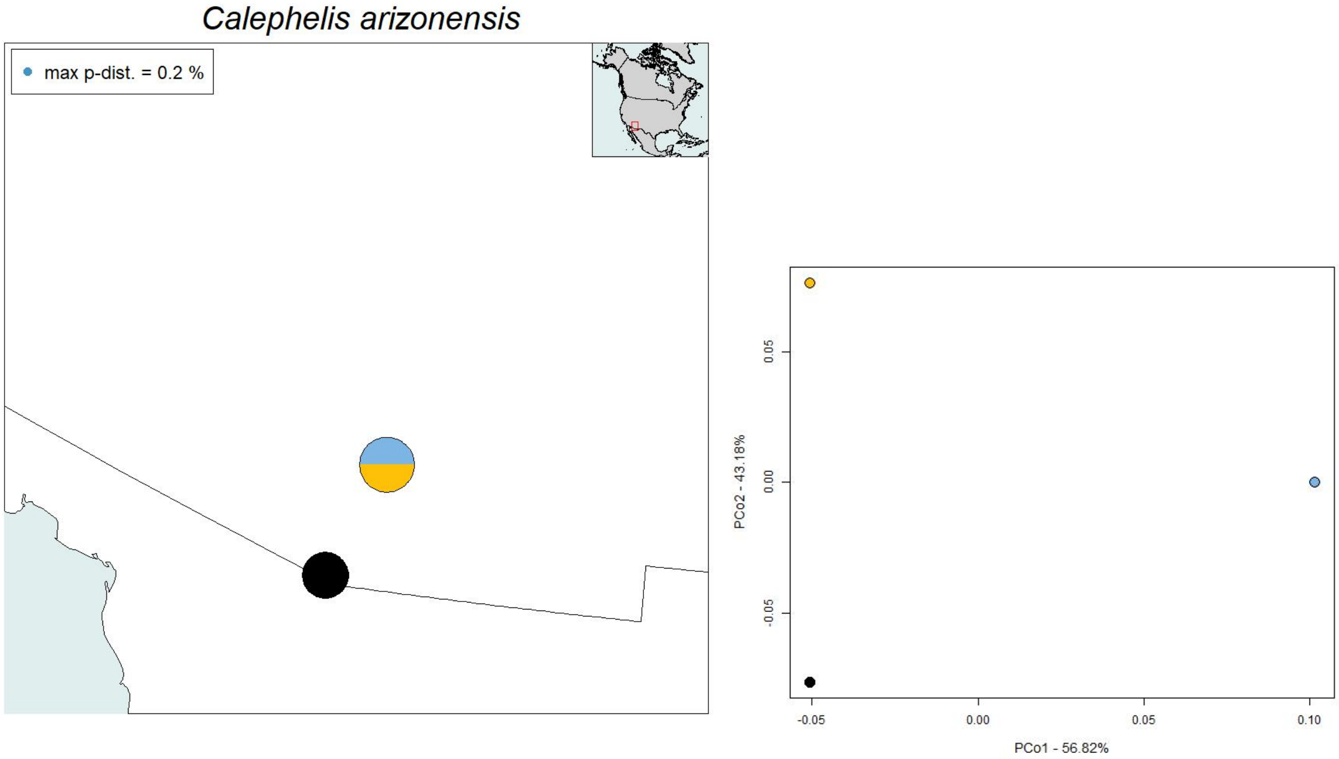

Figure 968 Map of *Calephelis arizonensis* showing the localities of the sequenced specimens (left). Nearby localities are grouped in pies. Colours match the bidimensional colour space of the PCoA projection (right) of max p-dists among sequences (dots). Sequences= 3; Hap obs.= 3; Hap asympt.= NA; Hap % obs.= NA%; GST= NaN; DST= NaN; HD= NA; ND= NA; max p-dist= 0.2%.

Haplotype network analysis and bubble plot of *Calephelis arizonensis* were not possible. Sequences > 599 bp = 2.

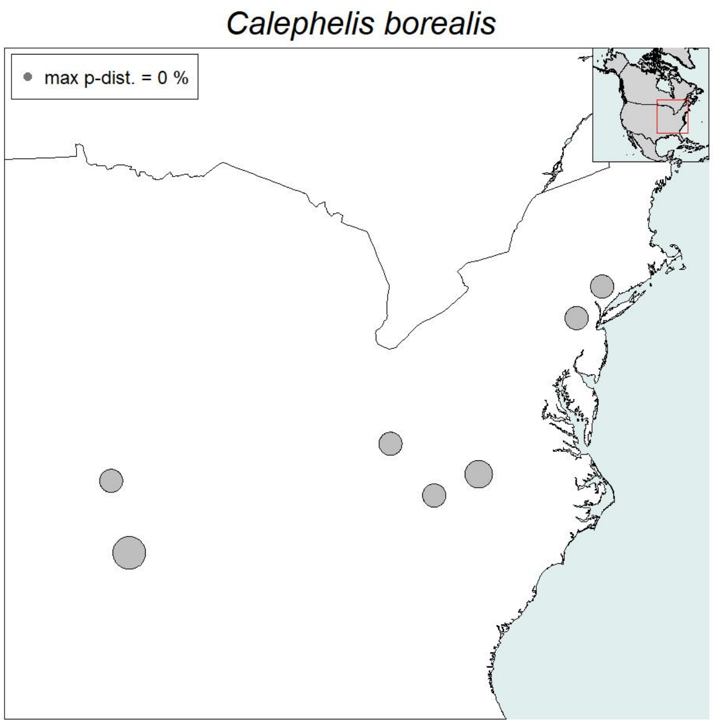

Figure 969: Map of *Calephelis borealis* showing the localities of the sequenced specimens. Nearby localities are grouped in pies. Due to the presence of a single haplotype PCoA projection was not done and a single grey colour was plotted on the map. Sequences= 11; Hap obs.= 1; Hap asympt.= NA; Hap % obs.= NA%; GST= NaN; DST= NaN; HD= 0; ND= 0; max p-dist= 0%.

Haplotype network analysis and bubble plot of *Calephelis borealis* were not possible. Sequences > 599 bp = 11.

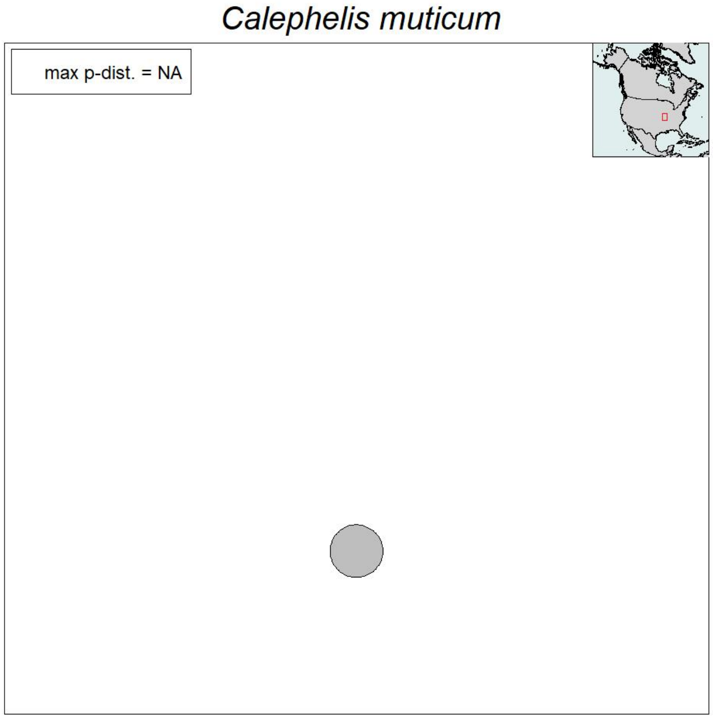

Figure 970: Map of *Calephelis muticum* showing the localities of the sequenced specimens. Nearby localities are grouped in pies. Due to the presence of a single haplotype PCoA projection was not done and a single grey colour was plotted on the map. Sequences= 1; Hap obs.= NA; Hap asympt.= NA; Hap % obs.= NA; GST= NaN; DST= NaN; HD= NA; ND= NA; max p-dist= NA.

Haplotype network analysis and bubble plot of *Calephelis muticum* were not possible. Sequences > 599 bp = 1.

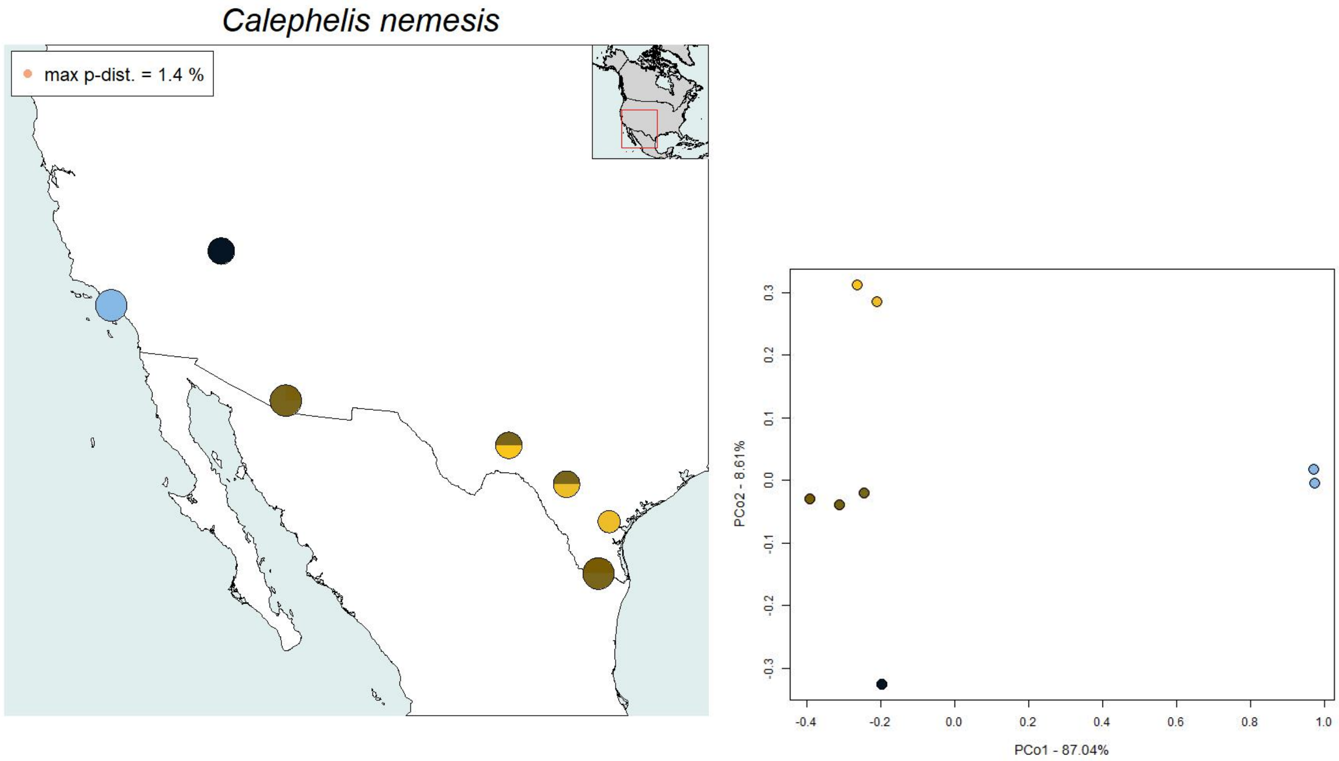

Figure 971 Map of *Calephelis nemesis* showing the localities of the sequenced specimens (left). Nearby localities are grouped in pies. Colours match the bidimensional colour space of the PCoA projection (right) of max p-dists among sequences (dots). Sequences= 19; Hap obs.= 5; Hap asympt.= 5.5; Hap % obs.= 91.3%; GST= 0.933; DST= 0.0052; HD= 0.696; ND= 0.0056; max p-dist= 1.4%.

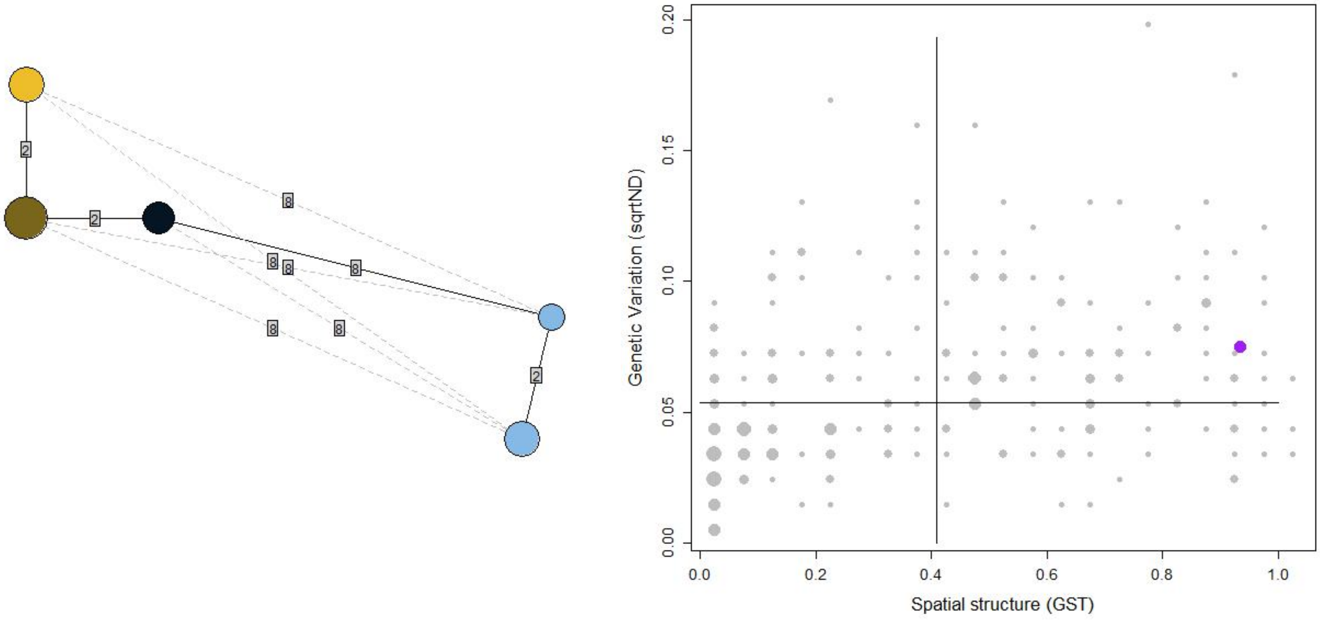

Figure 972: Haplotype network (left) of *Calephelis nemesis* sequences > 599 bp with colours matching the PCoA colour space (above). The bubble plot for mt-DNA polymorphism (square root transformed nucleotide diversity) and spatial structure (GST) among all species in the atlas and values for *Calephelis nemesis* (purple dot). The horizontal and vertical lines represent median values of nucleotide diversity and GST, respectively. Sequences > 599 bp= 16.

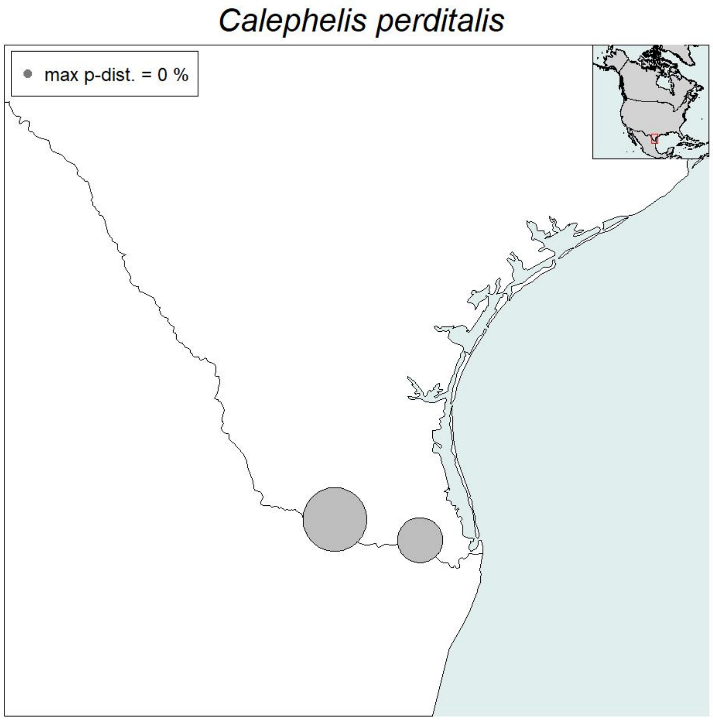

Figure 973: Map of *Calephelis perditalis* showing the localities of the sequenced specimens. Nearby localities are grouped in pies. Due to the presence of a single haplotype PCoA projection was not done and a single grey colour was plotted on the map. Sequences= 5; Hap obs.= 1; Hap asympt.= NA; Hap % obs.= NA%; GST= NaN; DST= NaN; HD= NA; ND= NA; max p-dist= 0%.

Haplotype network analysis and bubble plot of *Calephelis perditalis* were not possible. Sequences > 599 bp = 5.

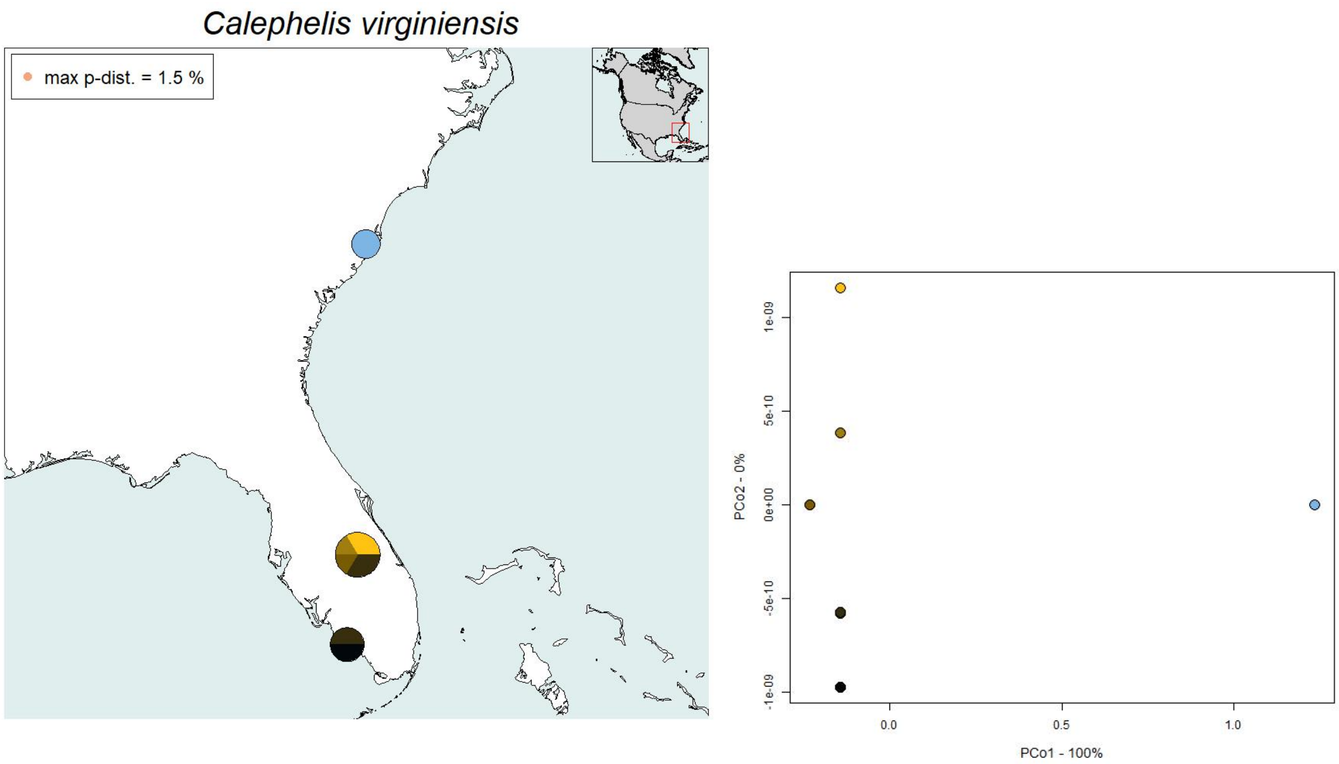

Figure 974 Map of *Calephelis virginiensis* showing the localities of the sequenced specimens (left). Nearby localities are grouped in pies. Colours match the bidimensional colour space of the PCoA projection (right) of max p-dists among sequences (dots). Sequences= 9; Hap obs.= 2; Hap asympt.= NA; Hap % obs.= NA%; GST= NaN; DST= NaN; HD= NA; ND= NA; max p-dist= 1.5%.

Haplotype network analysis and bubble plot of *Calephelis virginiensis* were not possible. Sequences > 599 bp = 9.

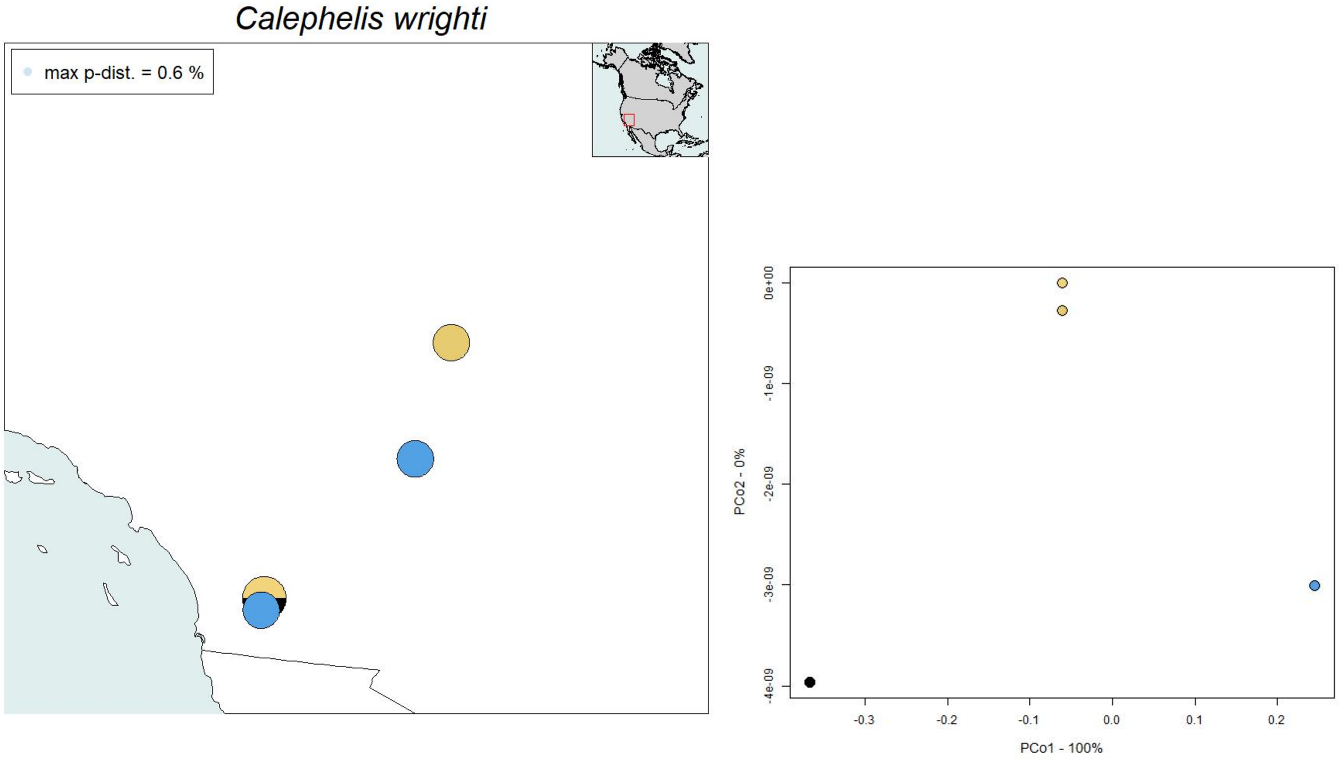

Figure 975 Map of *Calephelis wrighti* showing the localities of the sequenced specimens (left). Nearby localities are grouped in pies. Colours match the bidimensional colour space of the PCoA projection (right) of max p-dists among sequences (dots). Sequences= 5; Hap obs.= 3; Hap asympt.= NA; Hap % obs.= NA%; GST= NaN; DST= NaN; HD= NA; ND= NA; max p-dist= 0.6%.

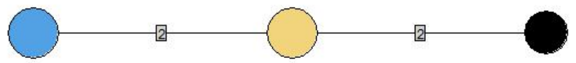

Figure 976: Haplotype network of *Calephelis wrighti*. Sequences > 599 bp= 5.

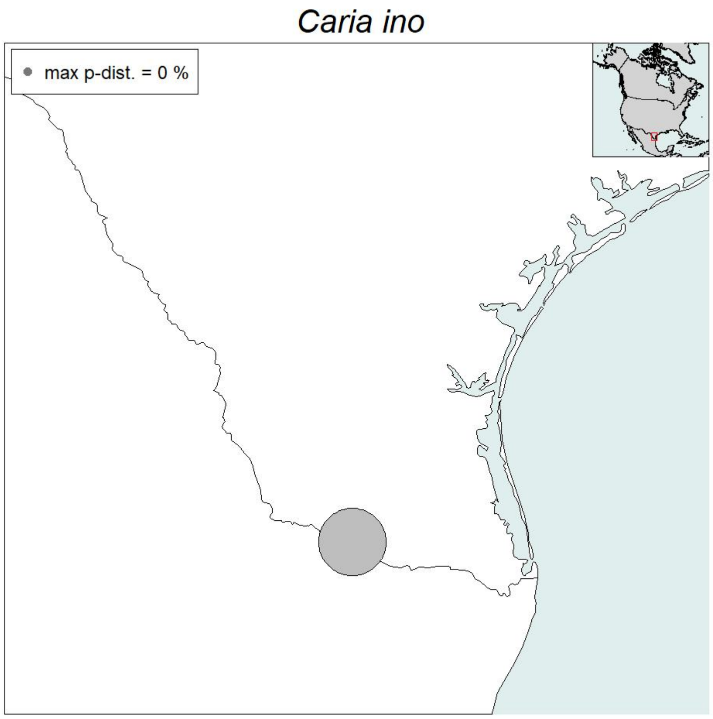

Figure 977: Map of *Caria ino* showing the localities of the sequenced specimens. Nearby localities are grouped in pies. Due to the presence of a single haplotype PCoA projection was not done and a single grey colour was plotted on the map. Sequences= 3; Hap obs.= 1; Hap asympt.= NA; Hap % obs.= NA%; GST= NaN; DST= NaN; HD= NA; ND= NA; max p-dist= 0%.

Haplotype network analysis and bubble plot of *Caria ino* were not possible. Sequences > 599 bp = 3.

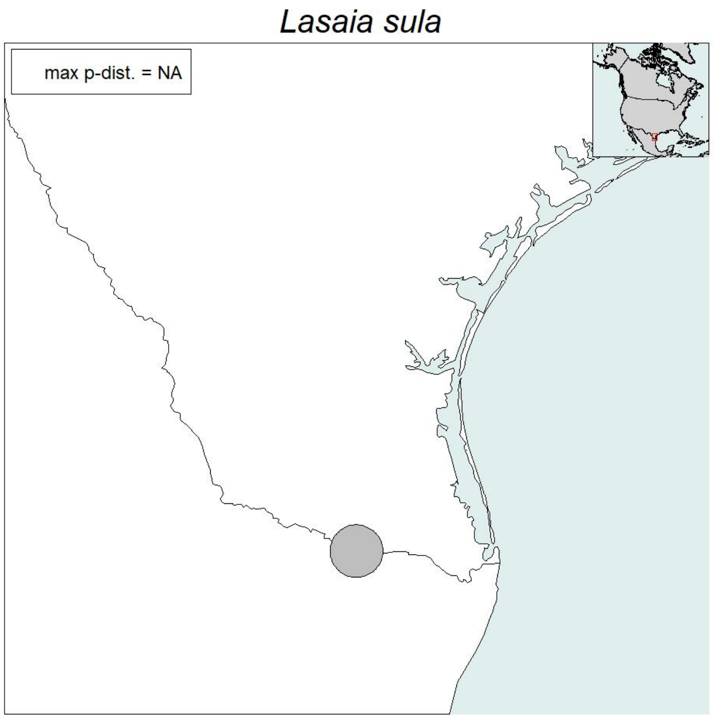

Figure 978: Map of *Lasaia sula* showing the localities of the sequenced specimens. Nearby localities are grouped in pies. Due to the presence of a single haplotype PCoA projection was not done and a single grey colour was plotted on the map. Sequences= 1; Hap obs.= NA; Hap asympt.= NA; Hap % obs.= NA; GST= NaN; DST= NaN; HD= NA; ND= NA; max p-dist= NA.

Haplotype network analysis and bubble plot of *Lasaia sula* were not possible. Sequences > 599 bp = 1.

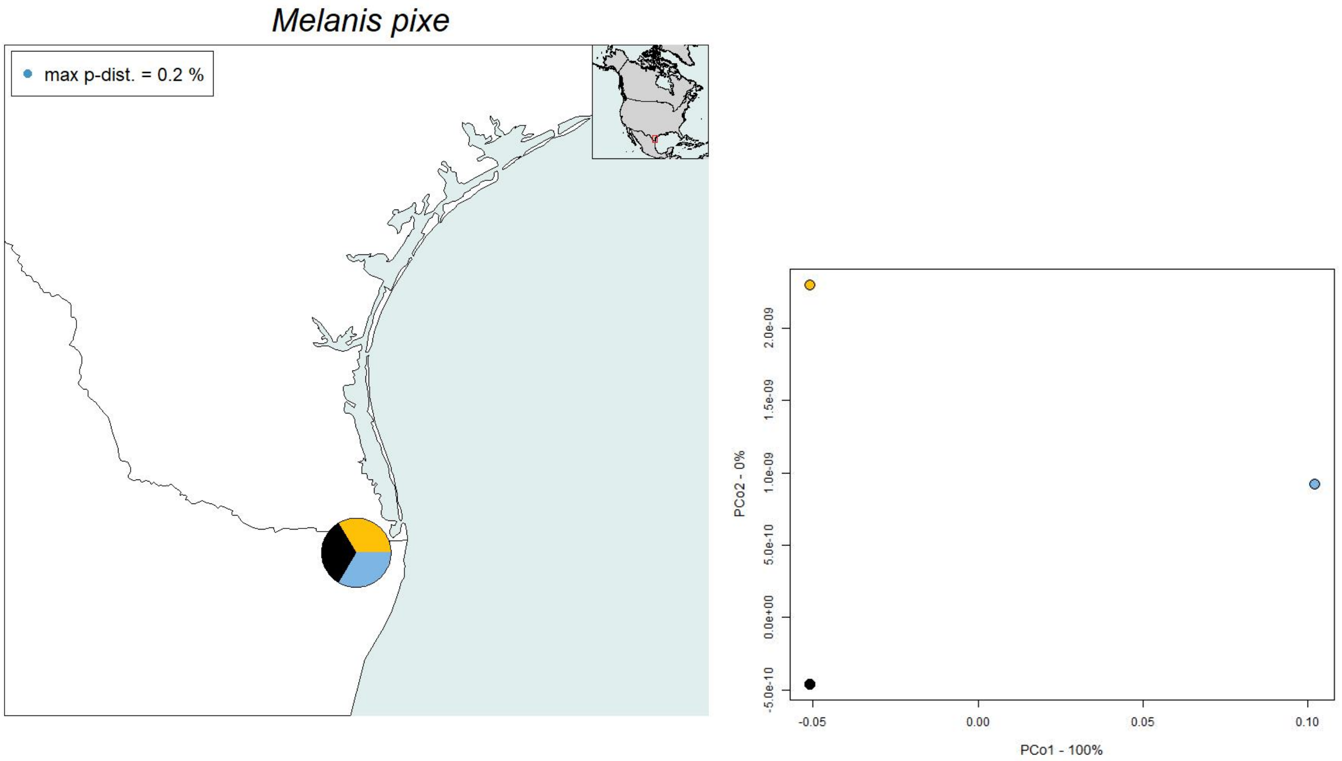

Figure 979 Map of *Melanis pixe* showing the localities of the sequenced specimens (left). Nearby localities are grouped in pies. Colours match the bidimensional colour space of the PCoA projection (right) of max p-dists among sequences (dots). Sequences= 3; Hap obs.= 2; Hap asympt.= NA; Hap % obs.= NA%; GST= NaN; DST= NaN; HD= NA; ND= NA; max p-dist= 0.2%.

Haplotype network analysis and bubble plot of *Melanis pixe* were not possible. Sequences > 599 bp = 3.

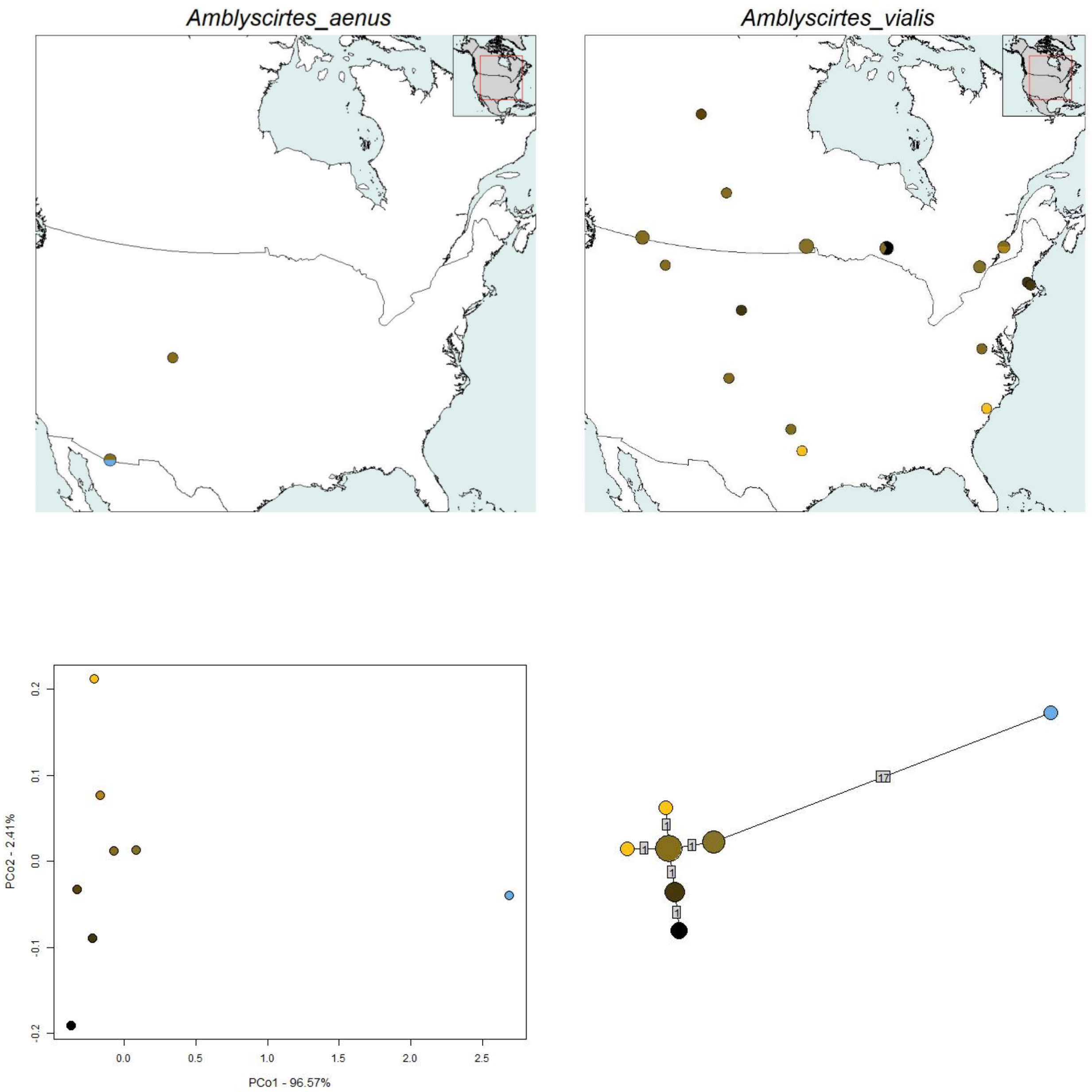

Figure 980: Haplotype maps, PCoA projection, and haplotype network for two *Amblyscirtes* species displaying barcode sharing.

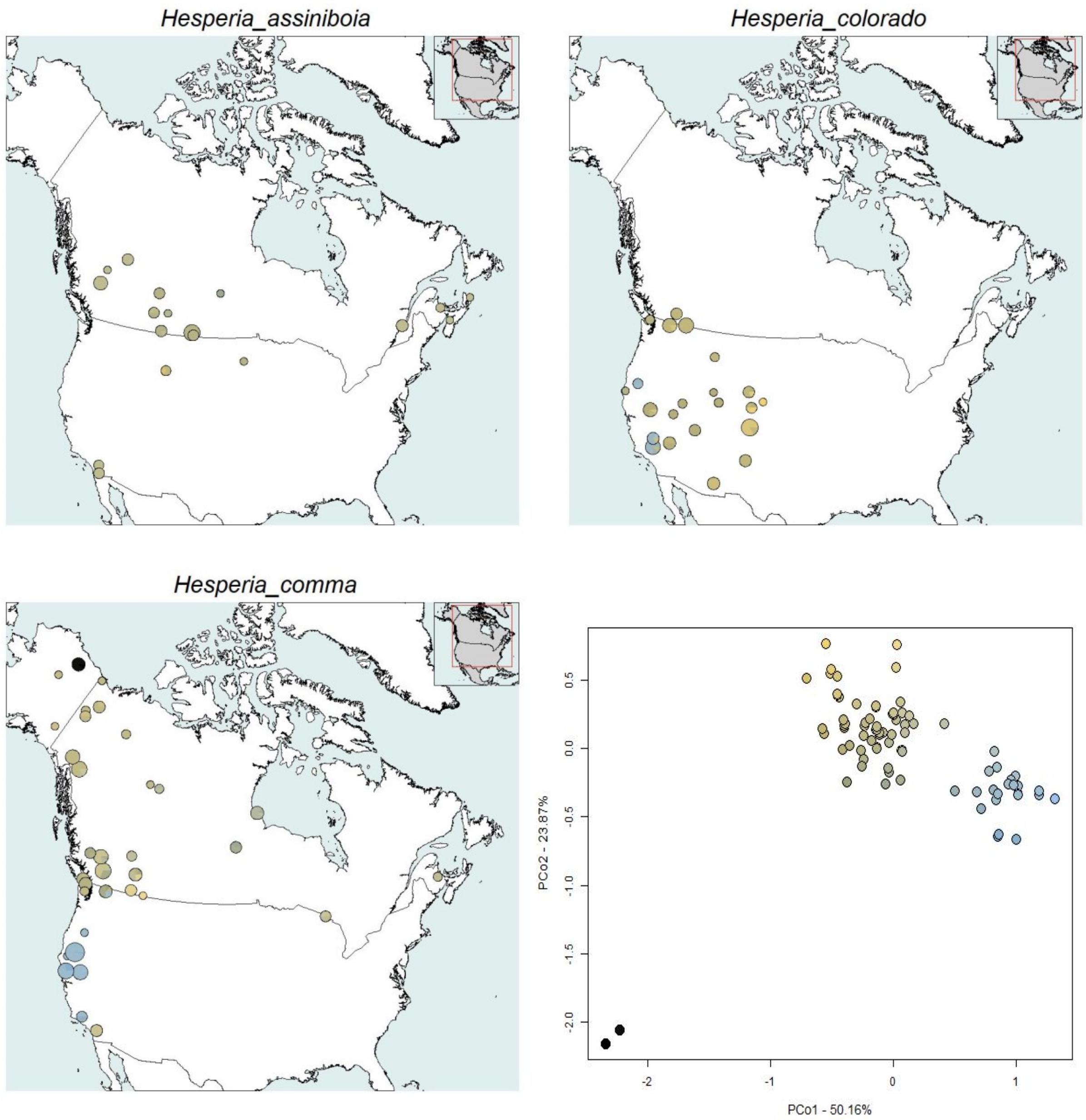
Hesperia_assiniboia
Hesperia_colorado
Hesperia_comma
PCo1 - 50.16%
PCo2 - 23.87%
0.5
0.0
-0.5
-1.0
-1.5
-2.0
-2
-1
0
1

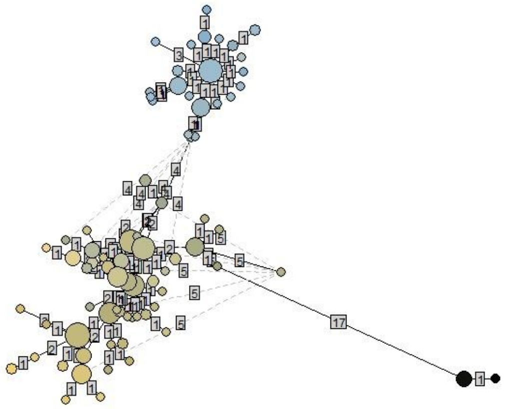

Figure 981: Haplotype maps, PCoA projection, and haplotype network for three *Hesperia* species displaying barcode sharing.

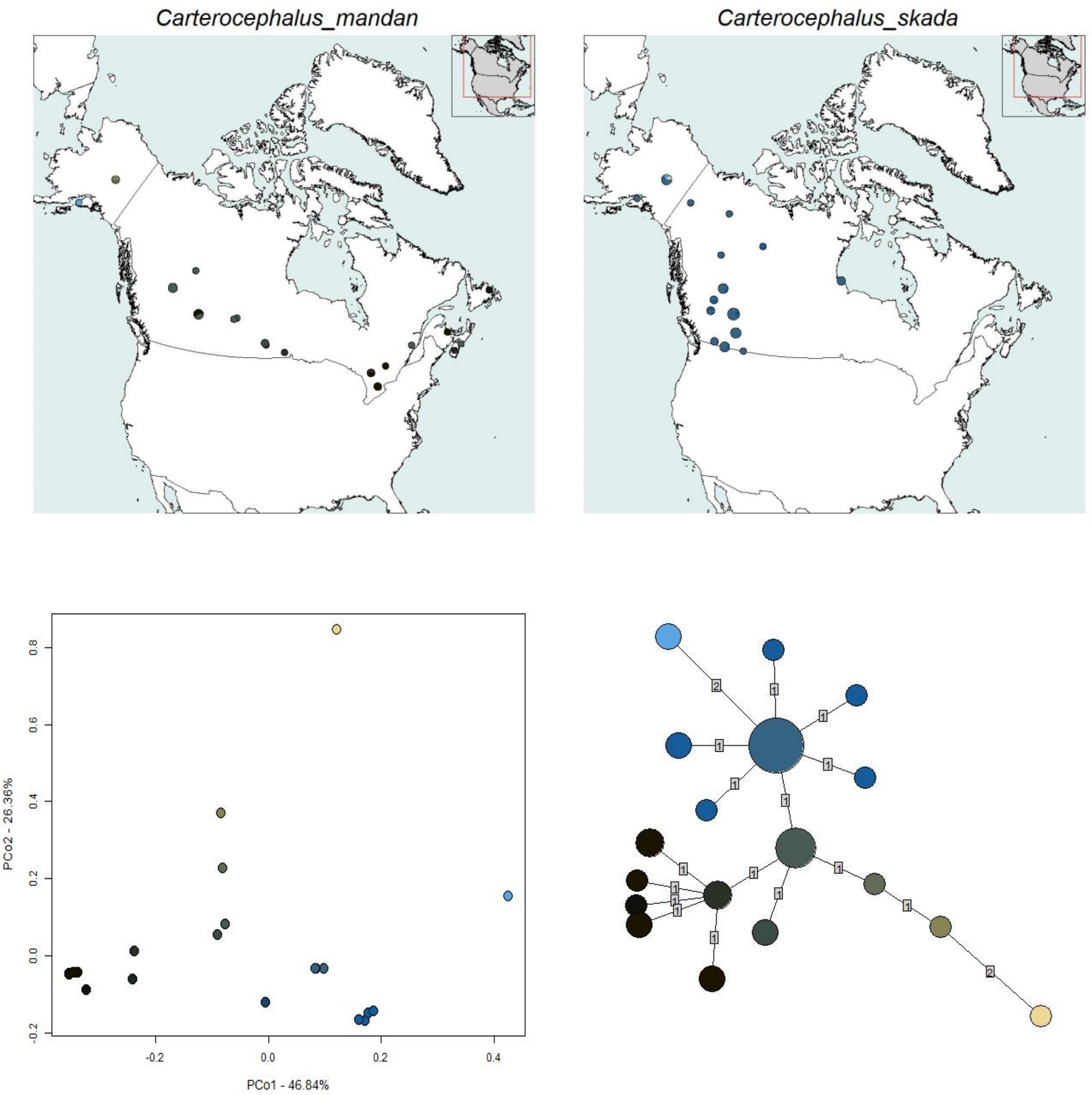

Figure 982: Haplotype maps, PCoA projection, and haplotype network for two *Carterocephalus* species displaying barcode sharing.

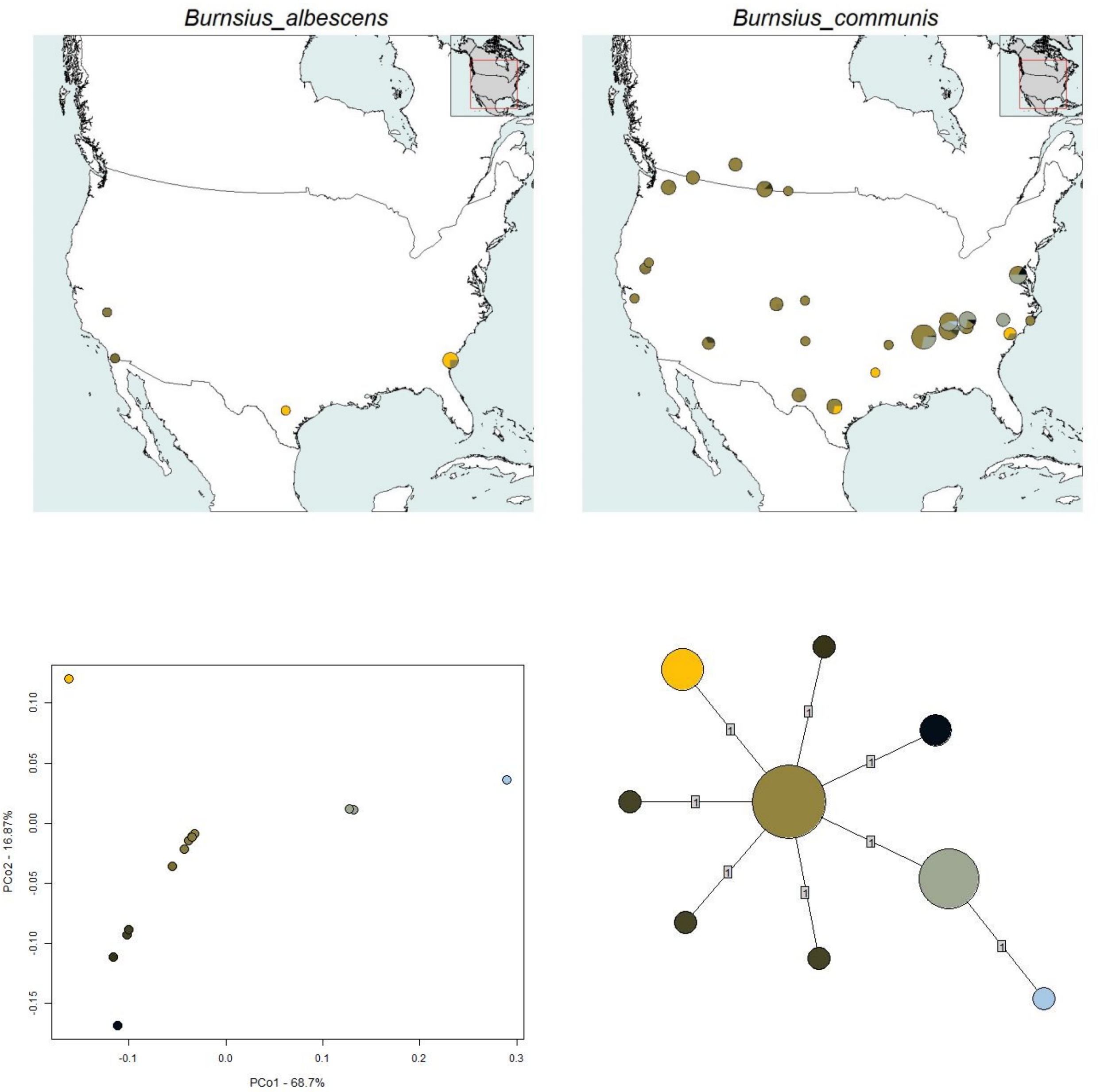

Figure 983: Haplotype maps, PCoA projection, and haplotype network for two *Burnsius* species displaying barcode sharing.

Erynnis_afranius

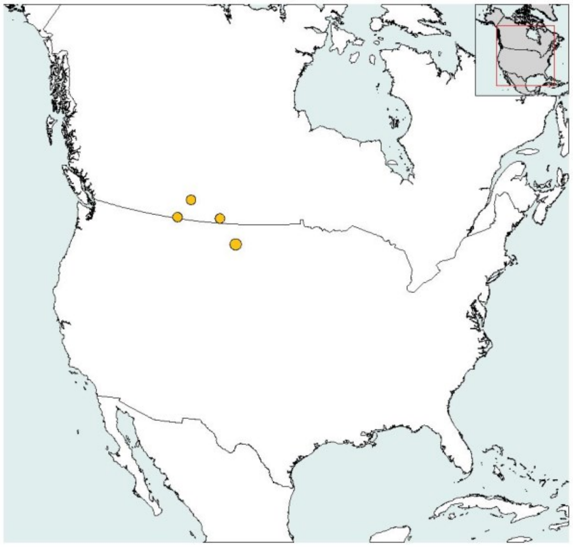

Erynnis_baptisiae

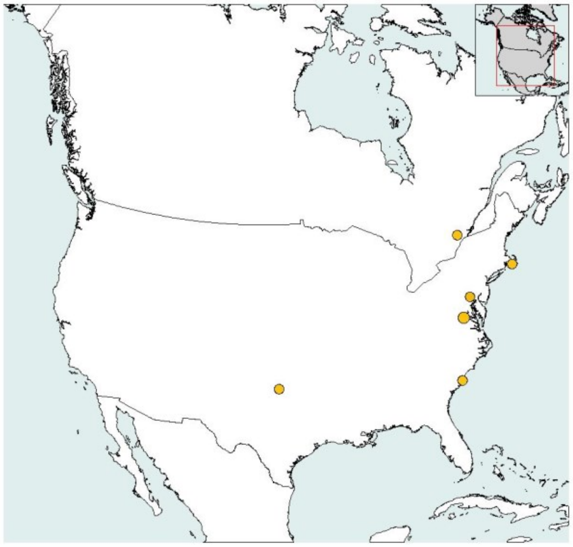

Erynnis_funeralis

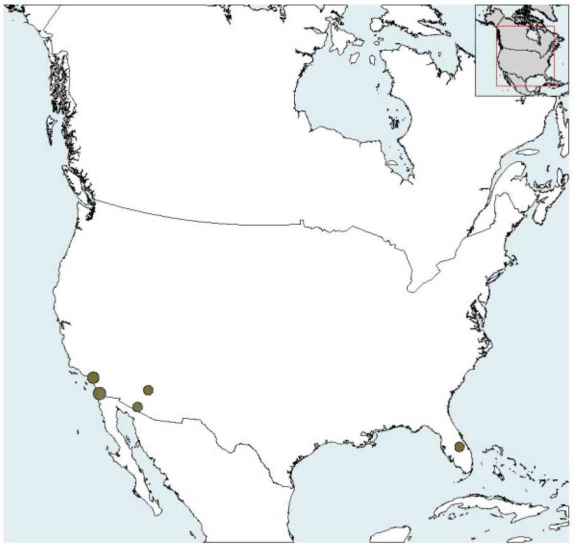

Erynnis_horatius

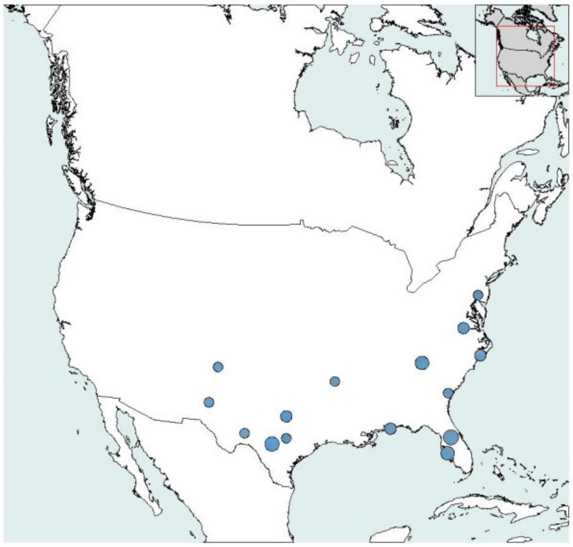

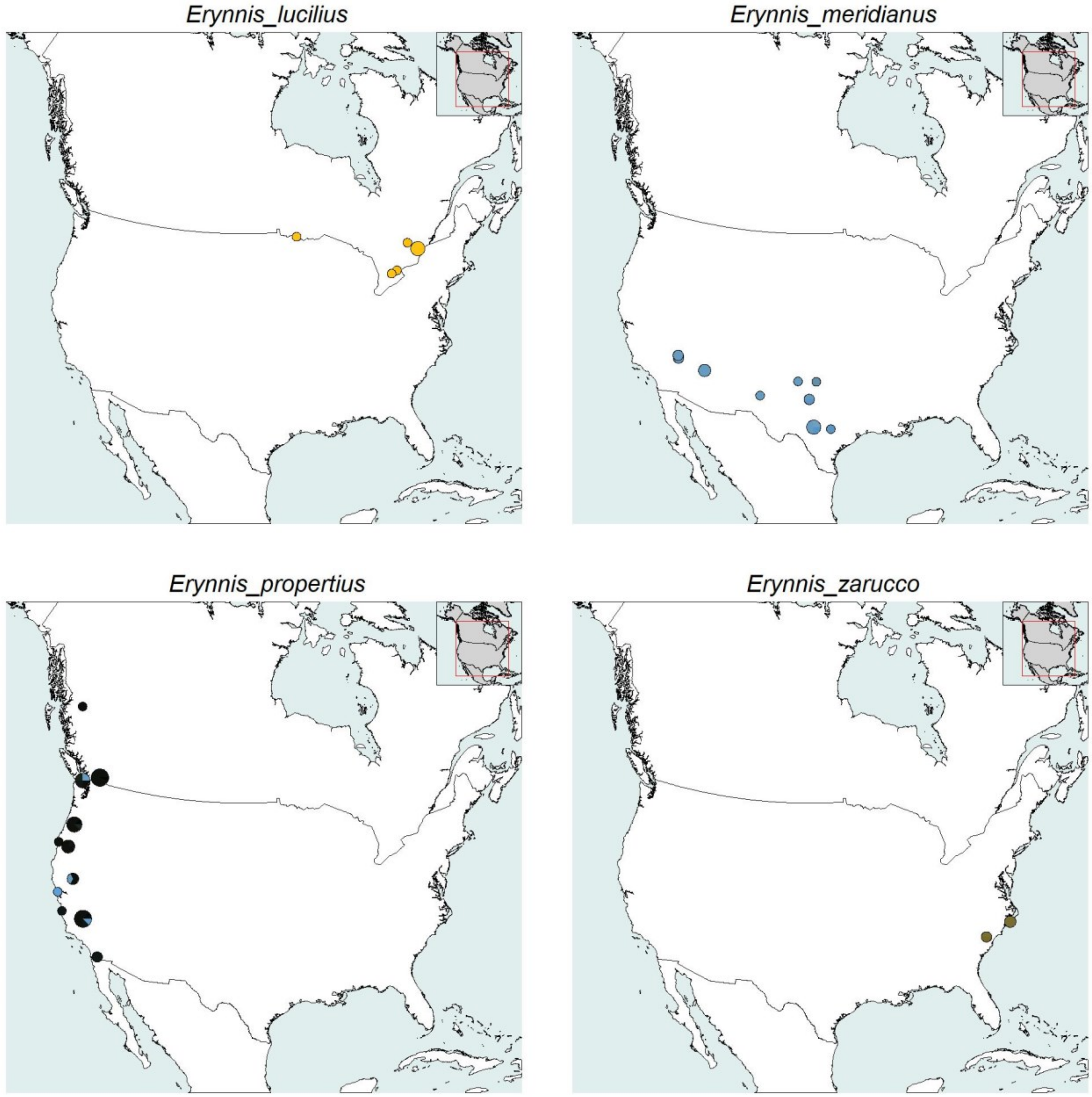
Erynnis_lucilius
Erynnis_meridianus
Erynnis_propertius
Erynnis_zarucco

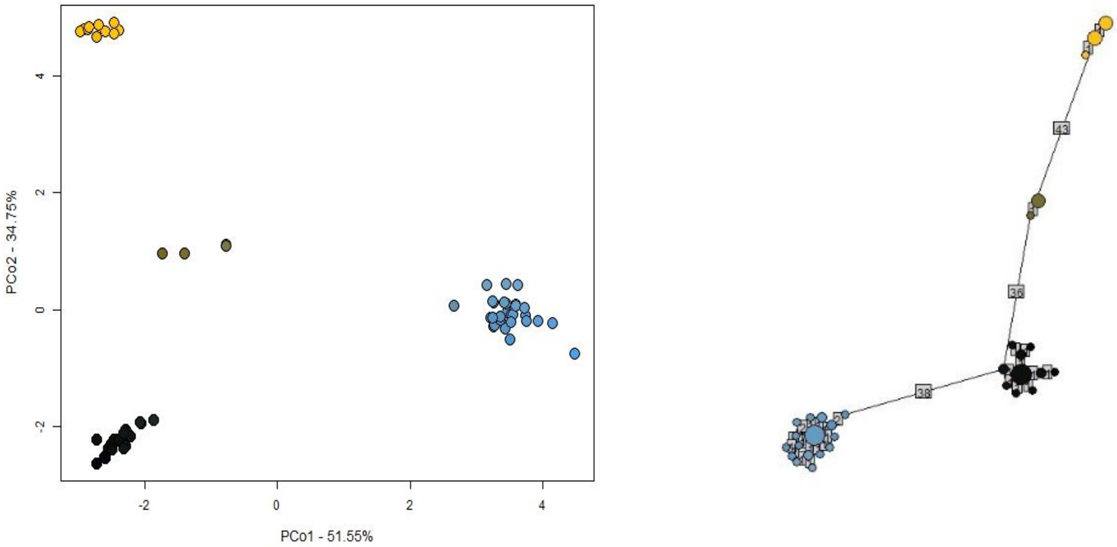

Figure 984: Haplotype maps, PCoA projection, and haplotype network for eight *Erynnis* species displaying barcode sharing.

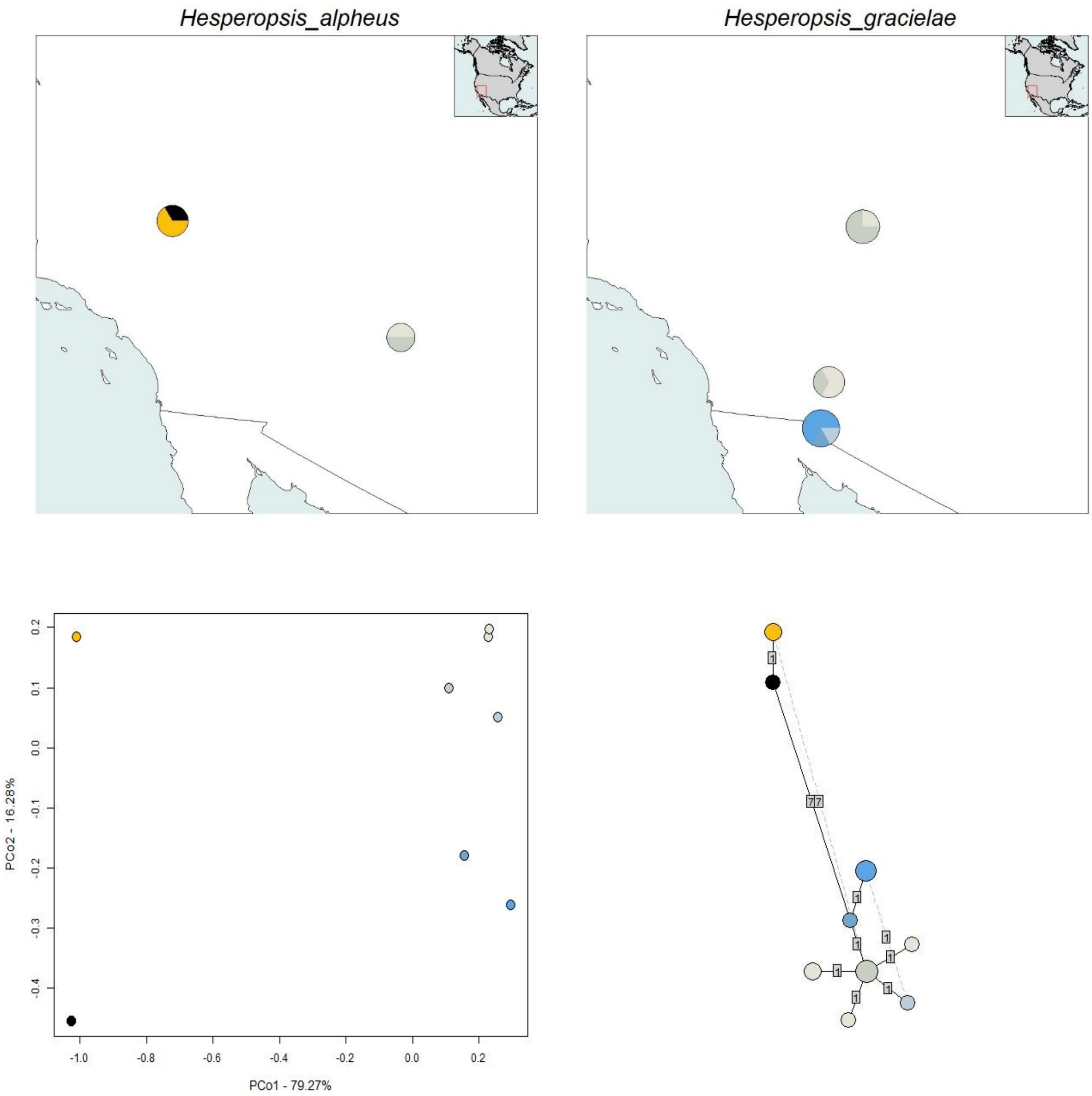

Figure 985: Haplotype maps, PCoA projection, and haplotype network for two *Hesperopsis* species displaying barcode sharing.

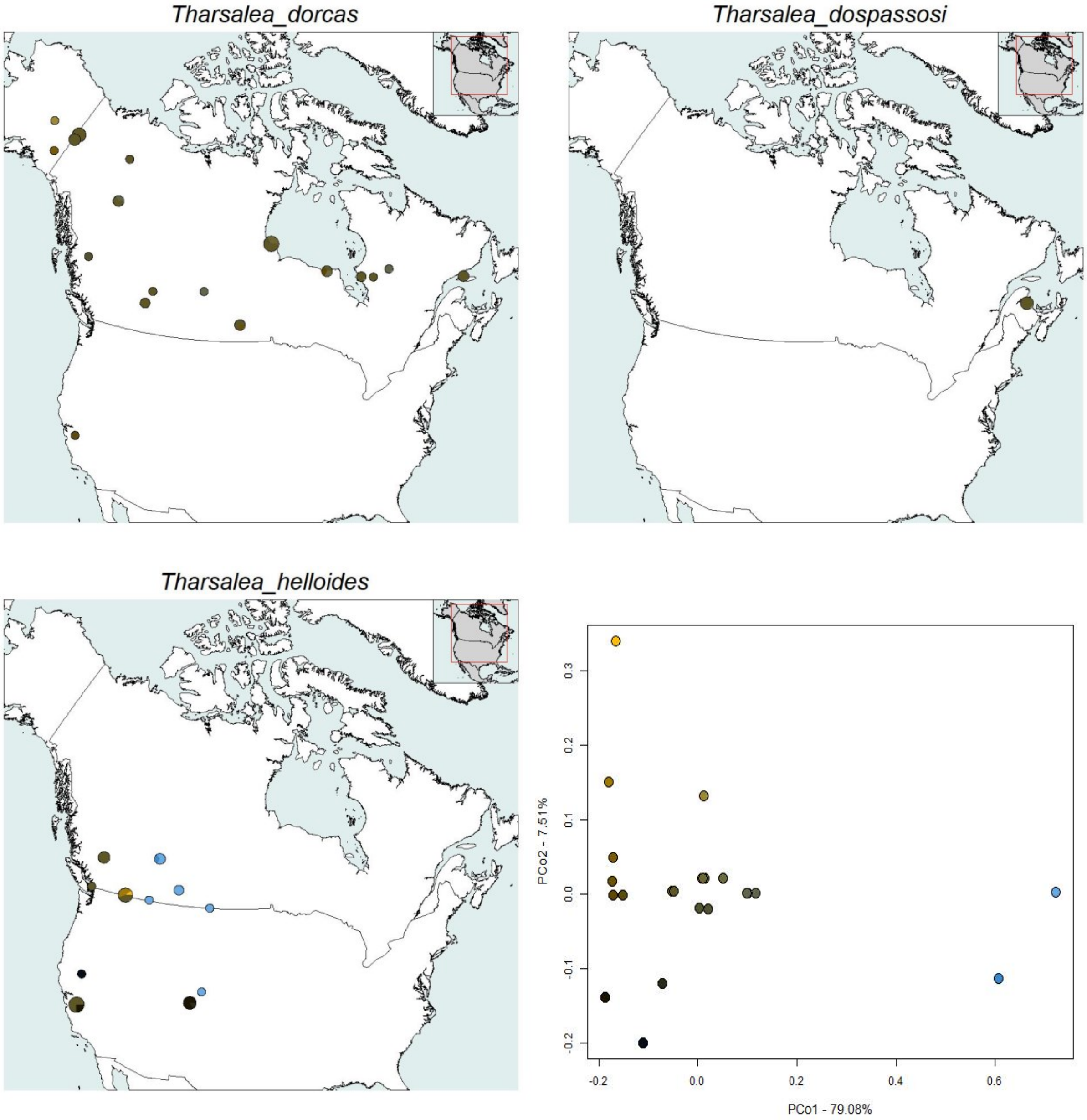
Tharsalea_dorcas
Tharsalea_dospassosi
Tharsalea_helloides
PCo2 - 7.51%
PCo1 - 79.08%
-0.2
0.0
0.2
0.4
0.6
-0.2
-0.1
0.0
0.1
0.2
0.3

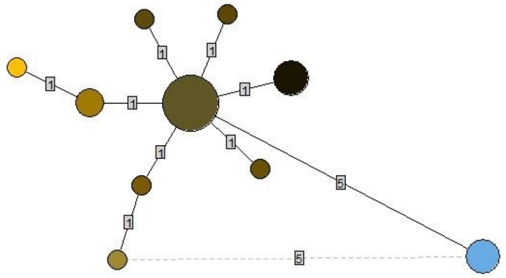

Figure 986: Haplotype maps, PCoA projection, and haplotype network for three *Tharsalea* species displaying barcode sharing.

Celastrina_echo

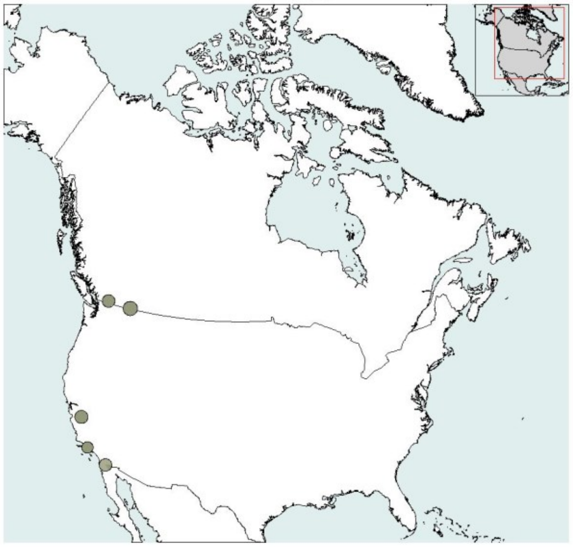

Celastrina_humulus

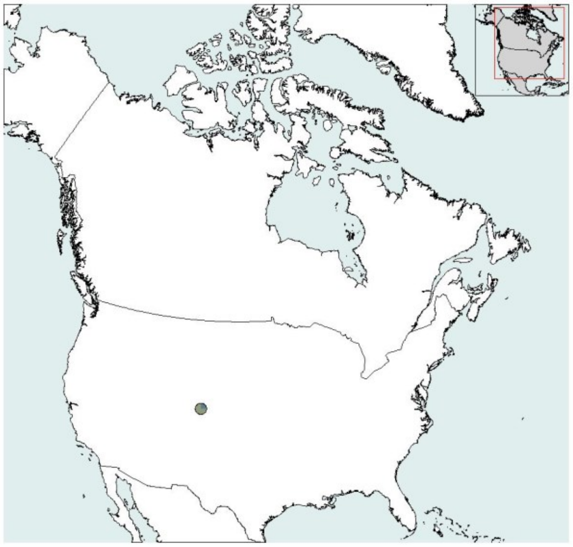

Celastrina_idella

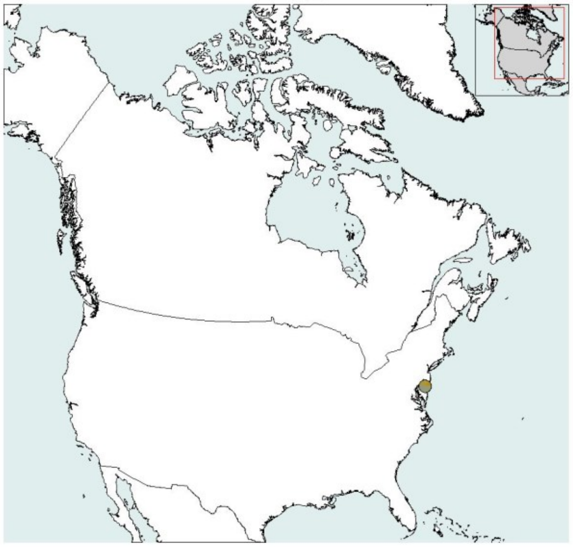

Celastrina_ladon

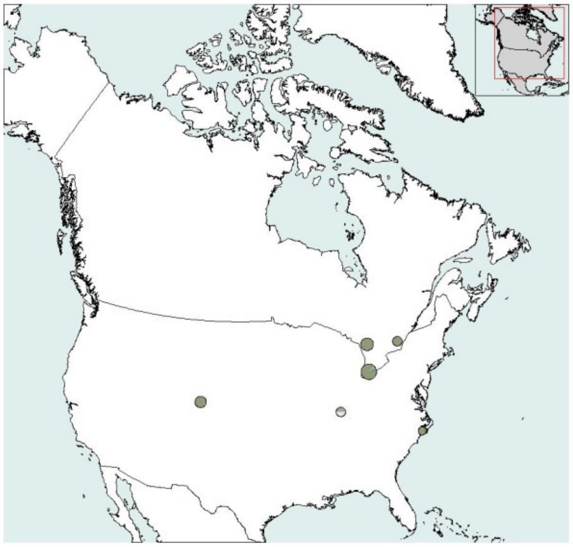

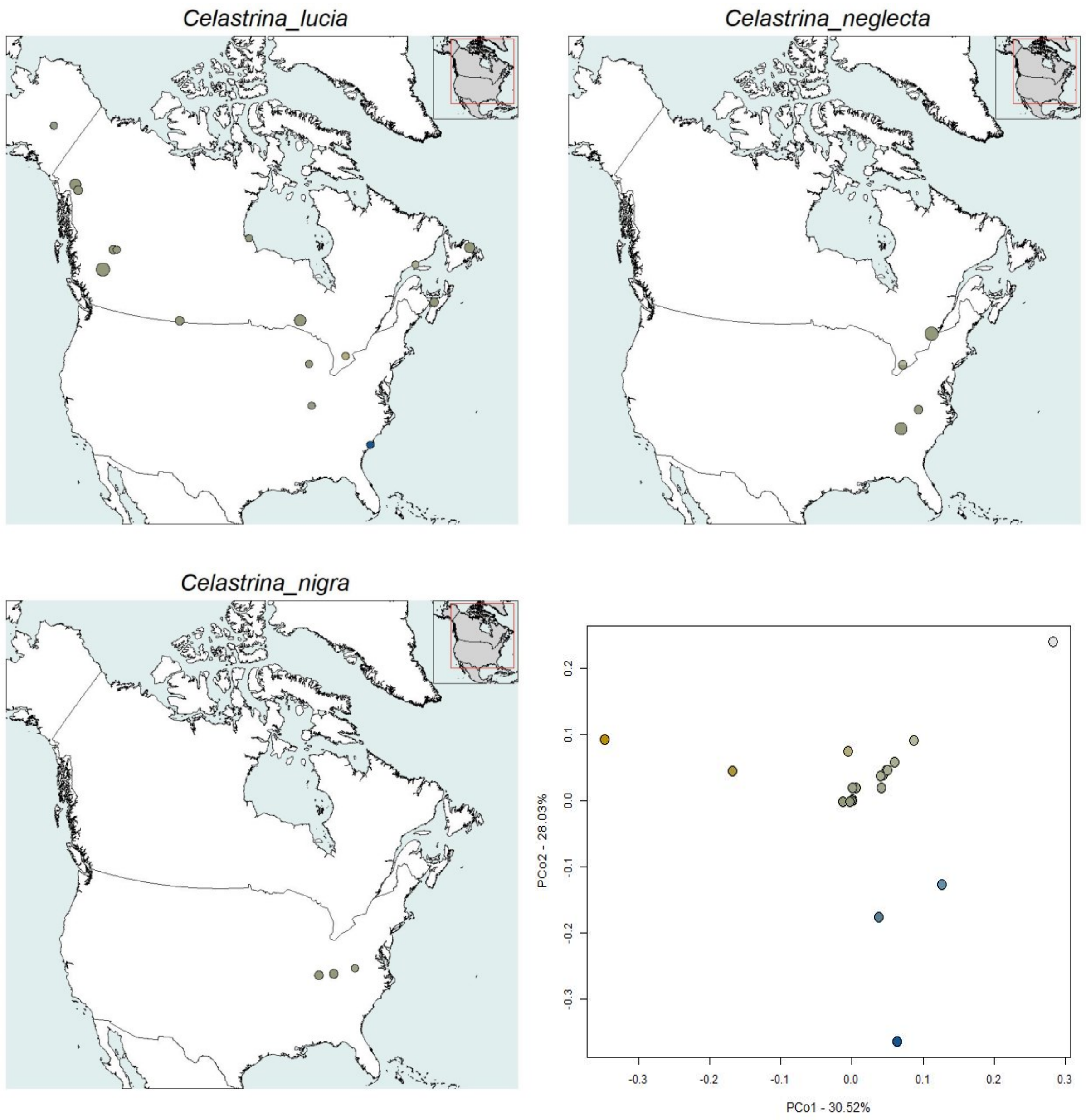
Celastrina_lucia
Celastrina_neglecta
Celastrina_nigra
PCo1 - 30.52%
PCo2 - 28.03%
-0.3
-0.2
-0.1
0.0
0.1
0.2
0.3

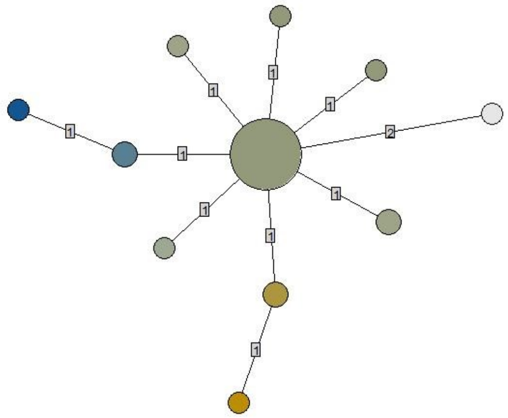

Figure 987: Haplotype maps, PCoA projection, and haplotype network for seven *Celastrina* species displaying barcode sharing.

Euphilotes_ancilla

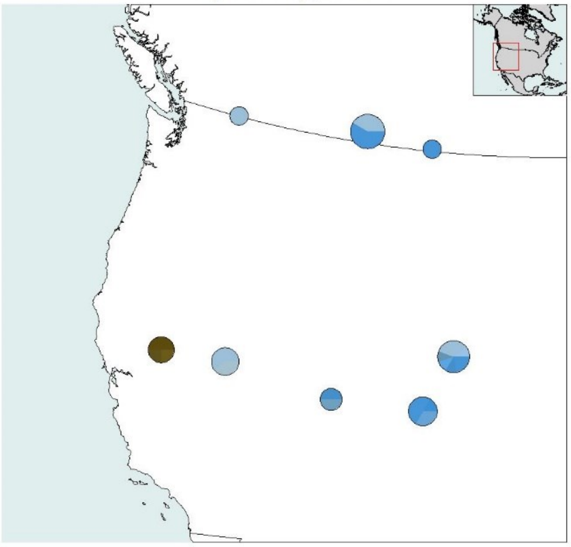

Euphilotes_battoides

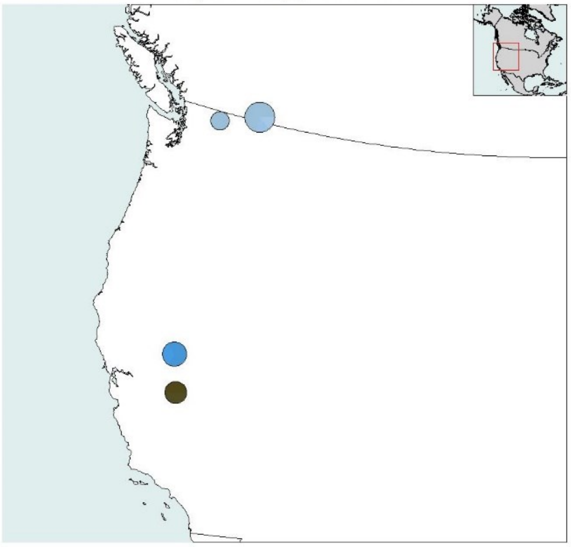

Euphilotes_baueri

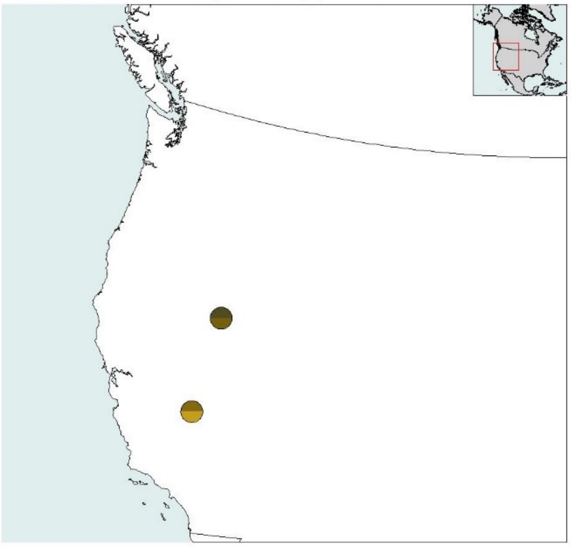

Euphilotes_bernardino

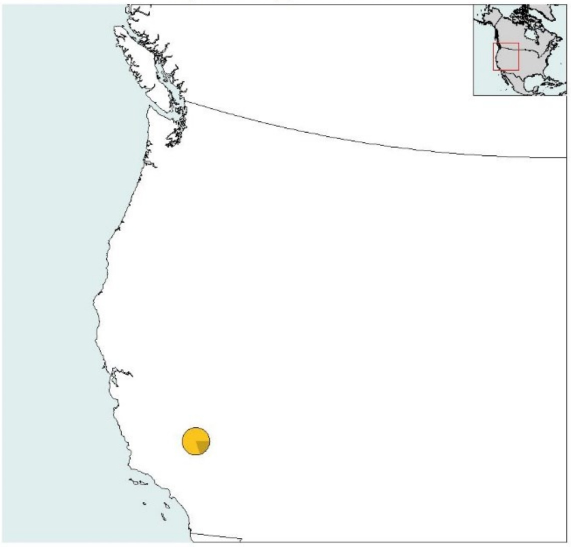

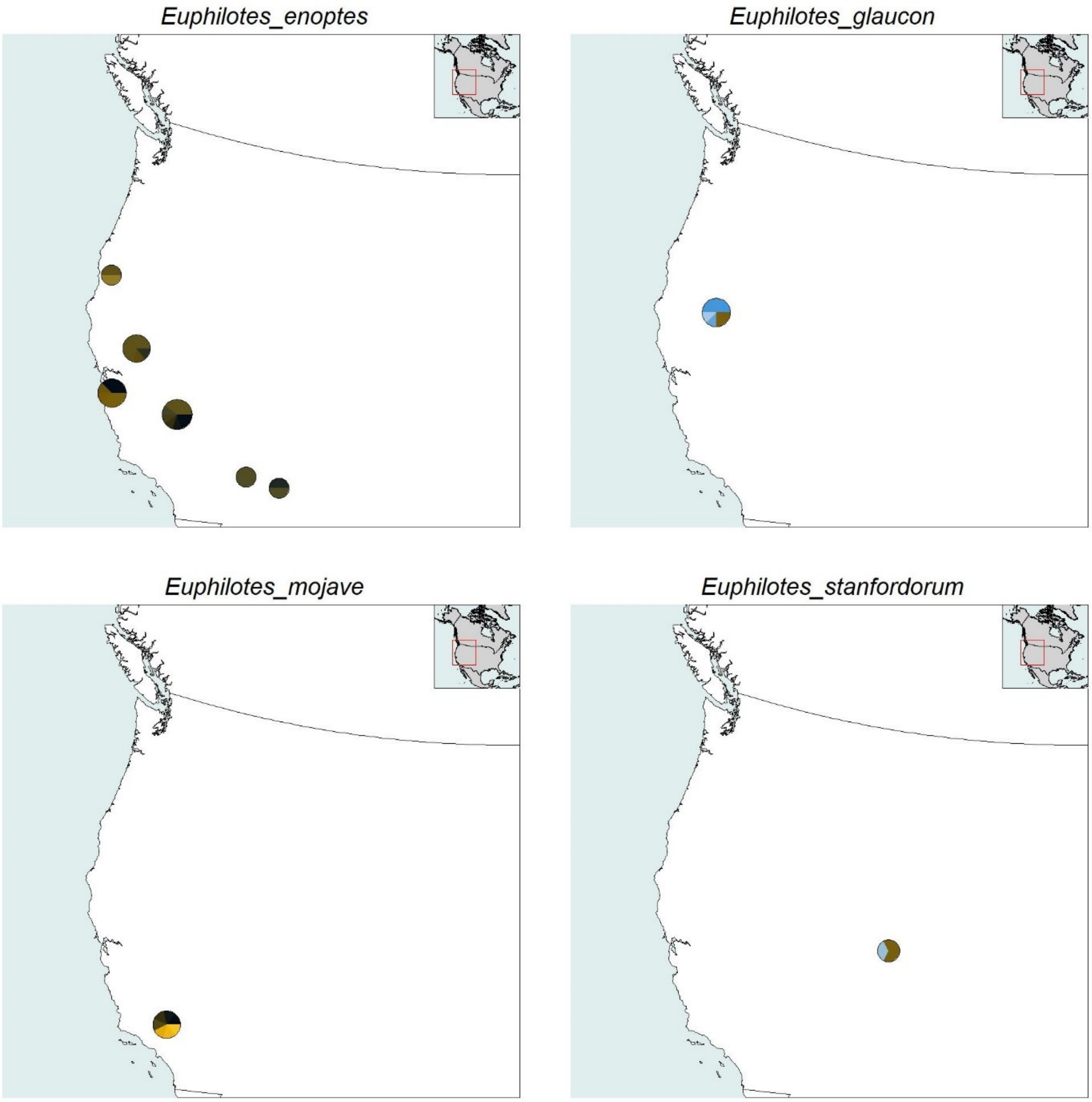
Euphilotes_enoptes
Euphilotes_glaucon
Euphilotes_mojave
Euphilotes_stanfordorum

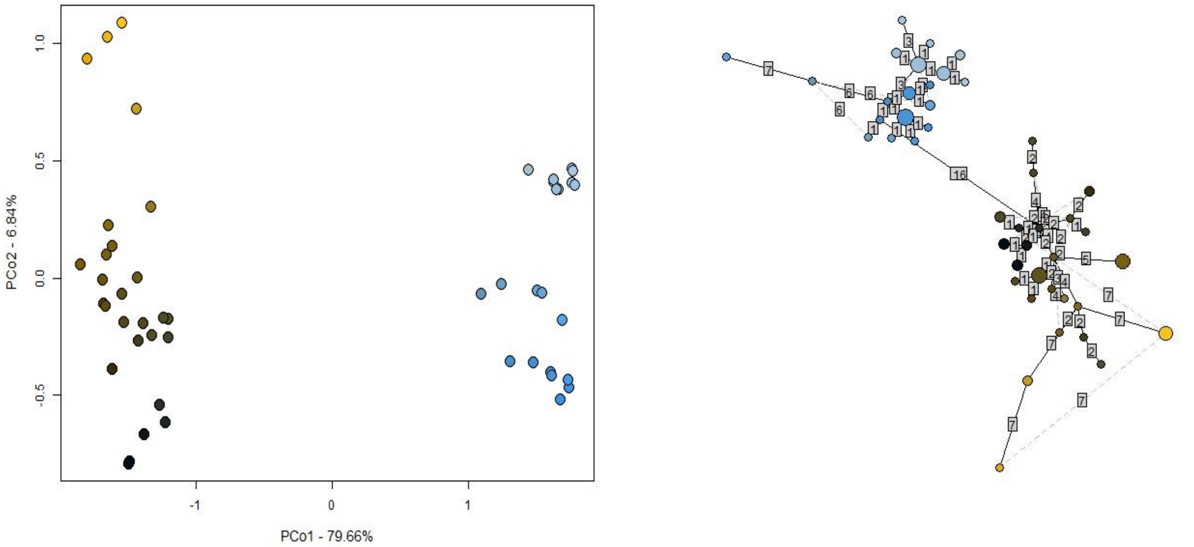

Figure 988: Haplotype maps, PCoA projection, and haplotype network for seven *Euphilotes* species displaying barcode sharing.

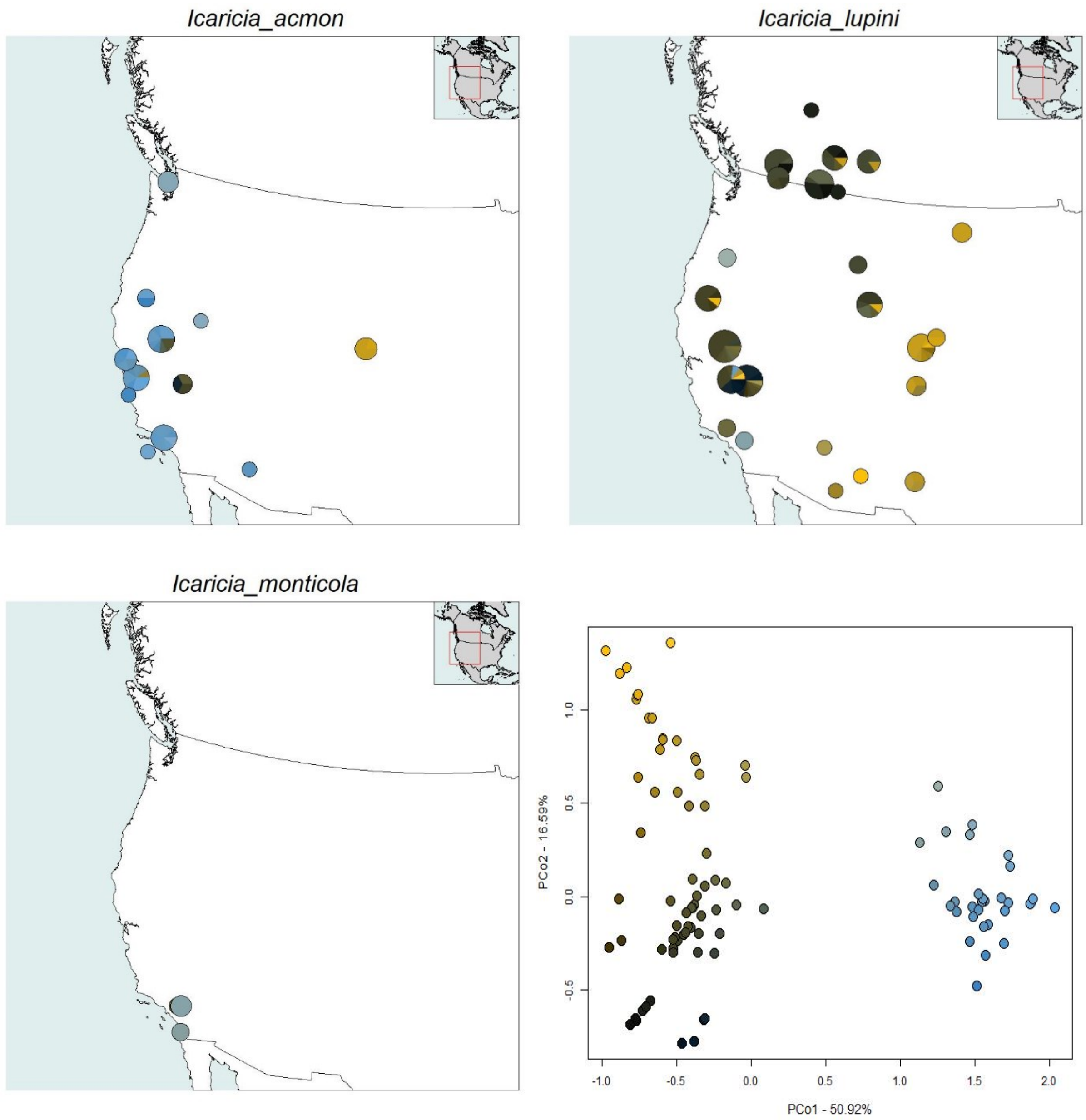
Icaricia_acmon
Icaricia_lupini
Icaricia_monticola
PCo2 - 16.59%
PCo1 - 50.92%
-1.0
-0.5
0.0
0.5
1.0
1.5
2.0
-0.5
0.0
0.5
1.0

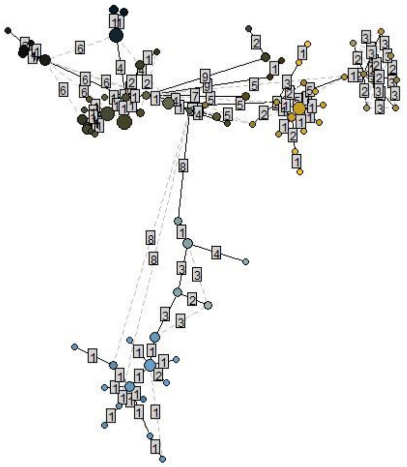

Figure 989: Haplotype maps, PCoA projection, and haplotype network for three *Icaricia* species displaying barcode sharing.

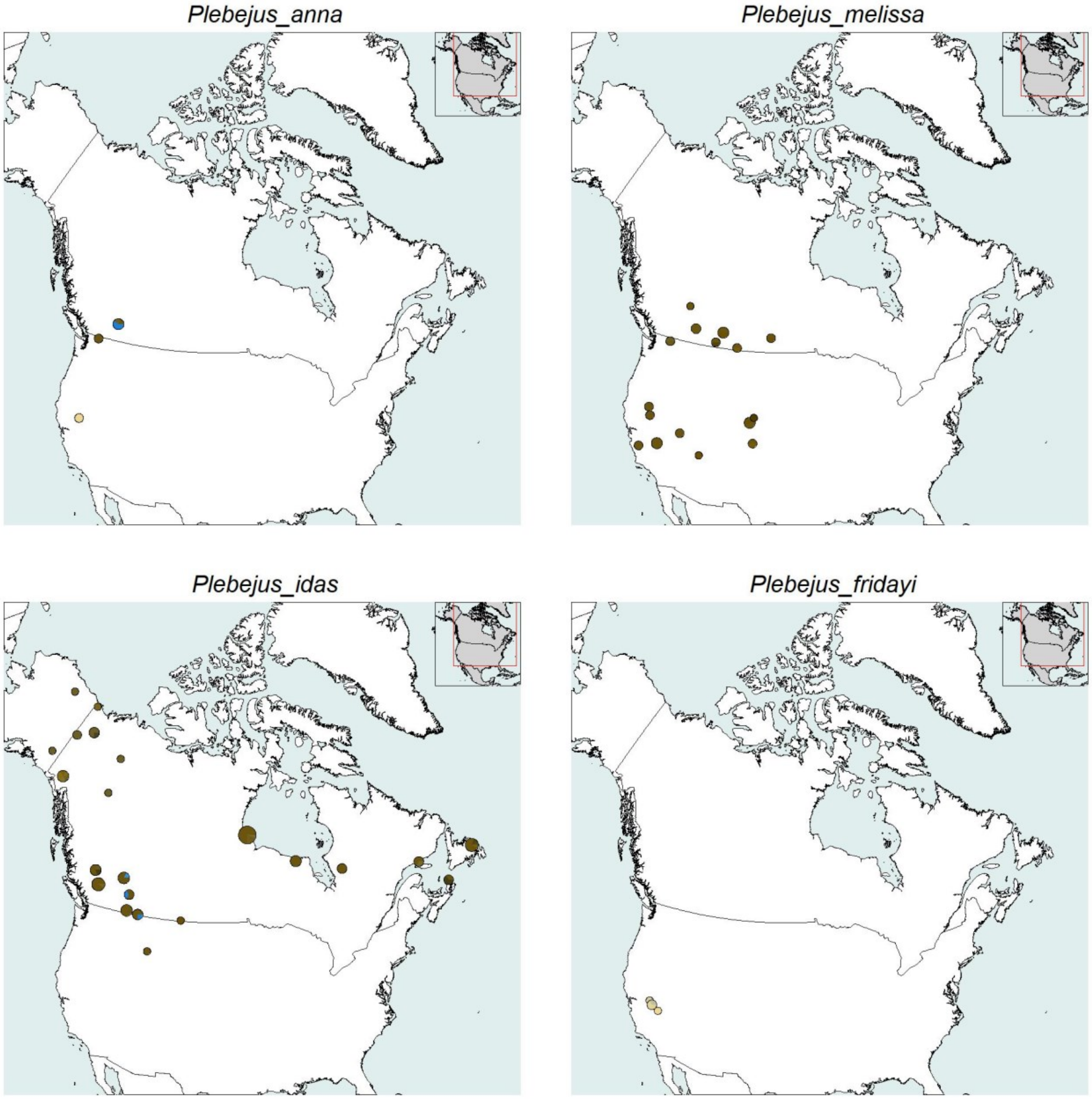
Plebejus_anna
Plebejus_melissa
Plebejus_idas
Plebejus_fridayi

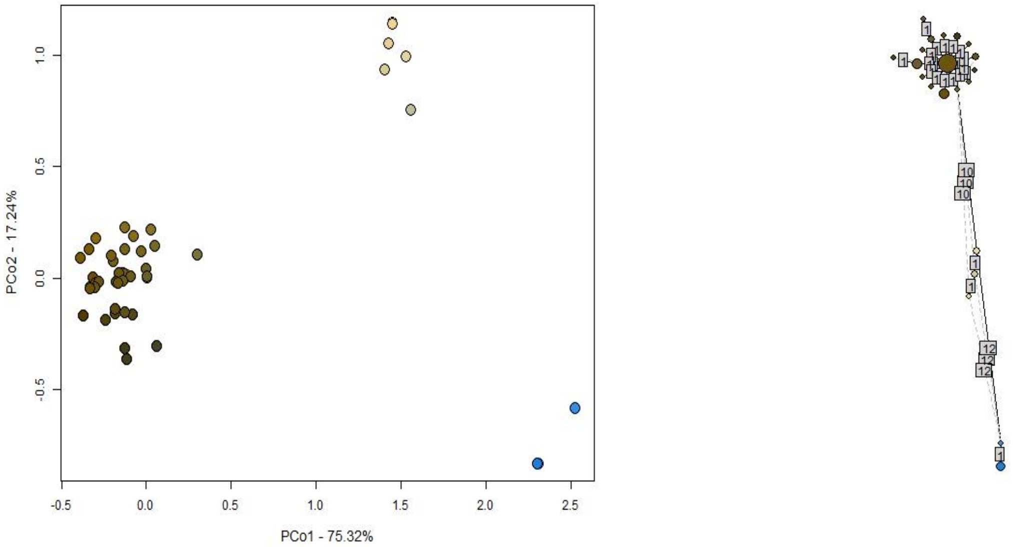

Figure 990: Haplotype maps, PCoA projection, and haplotype network for four *Plebejus* species displaying barcode sharing.

Callophrys_affinis

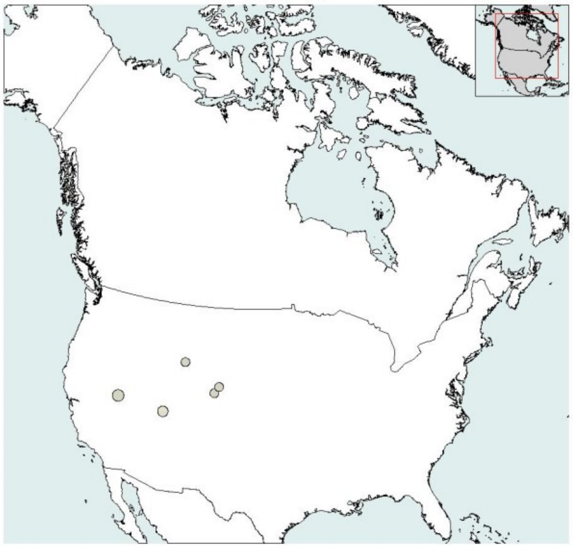

Callophrys_augustinus

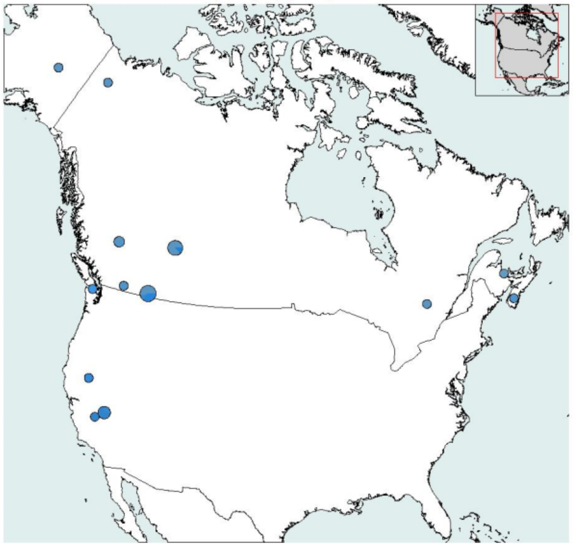

Callophrys_mossii

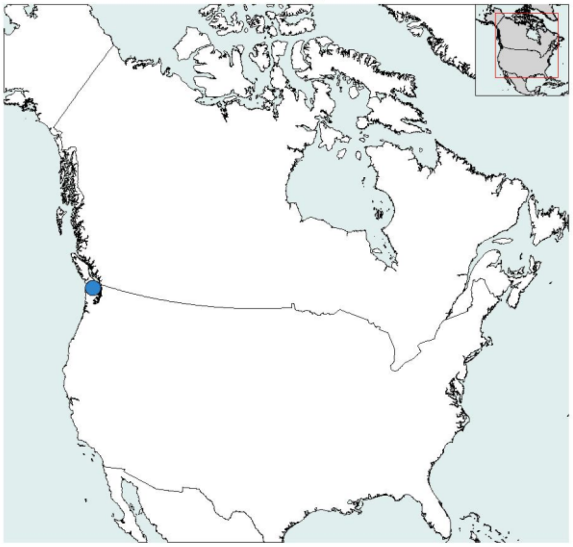

Callophrys_muiri

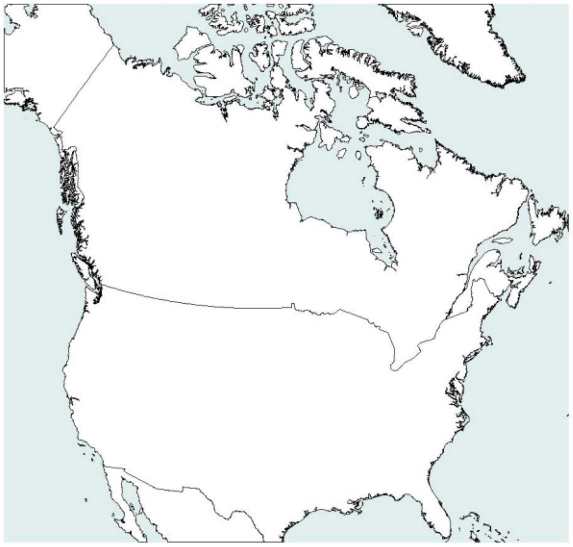

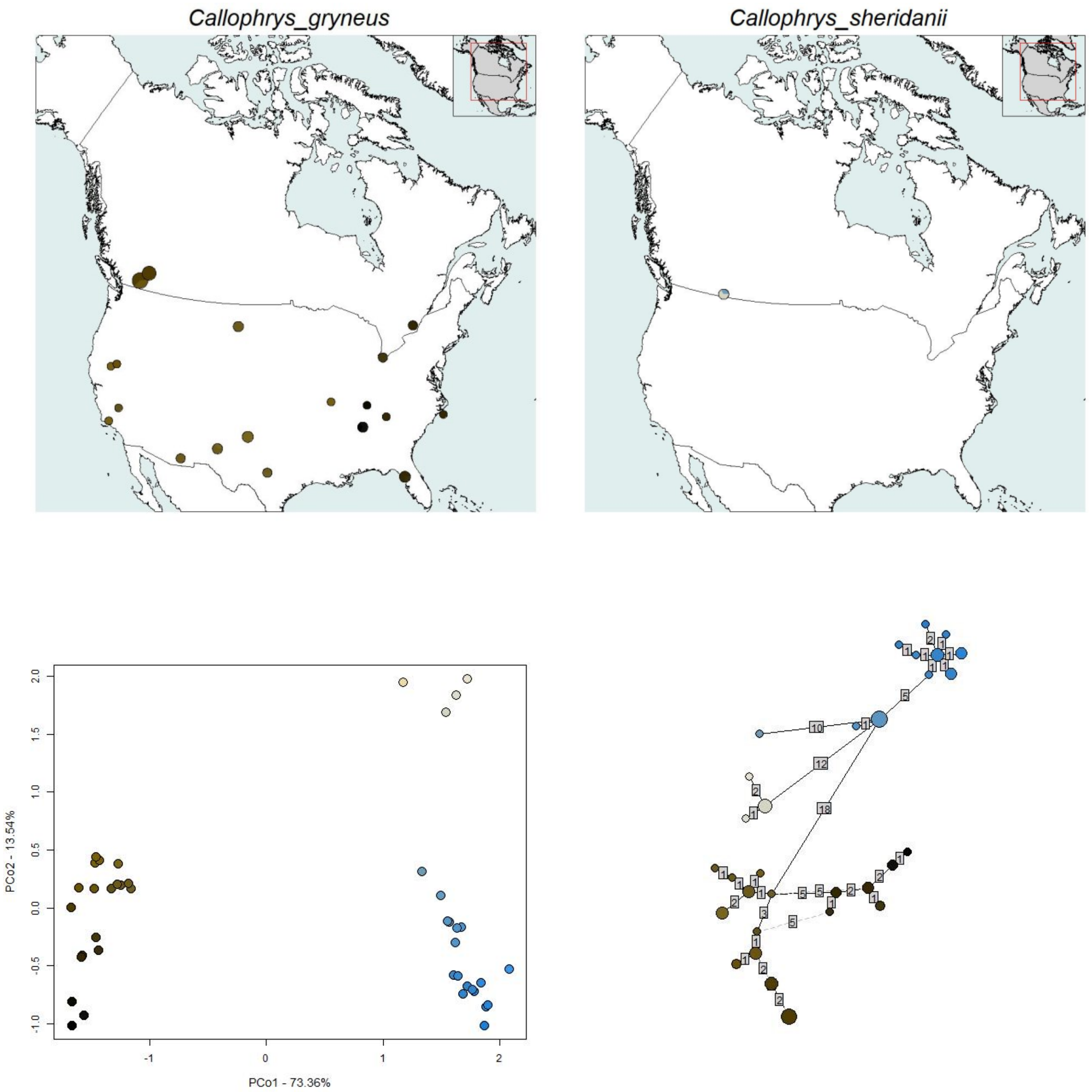

Figure 991: Haplotype maps, PCoA projection, and haplotype network for six *Callophrys* species displaying barcode sharing.

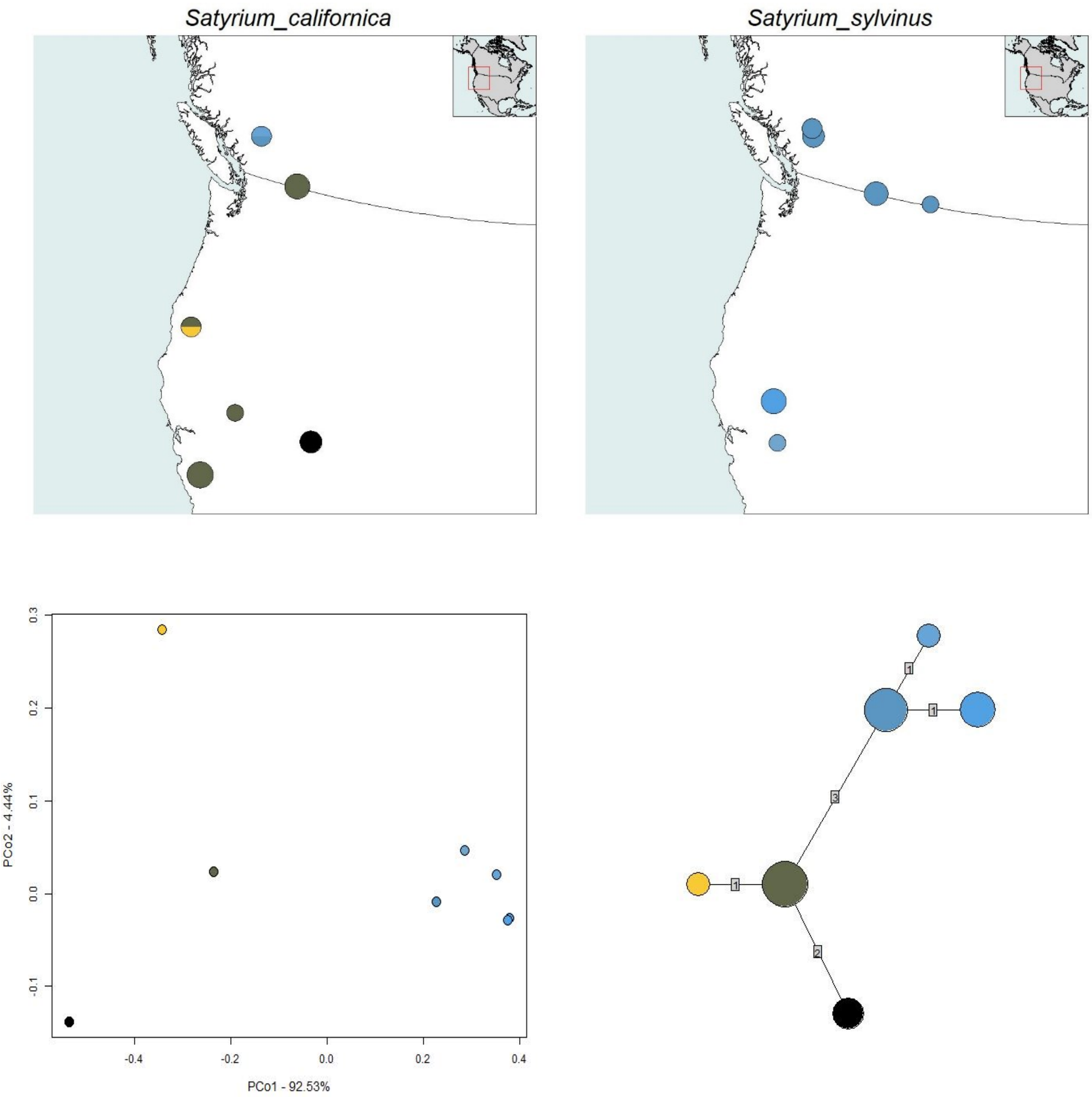

Figure 992: Haplotype maps, PCoA projection, and haplotype network for two *Satyrium* species displaying barcode sharing.

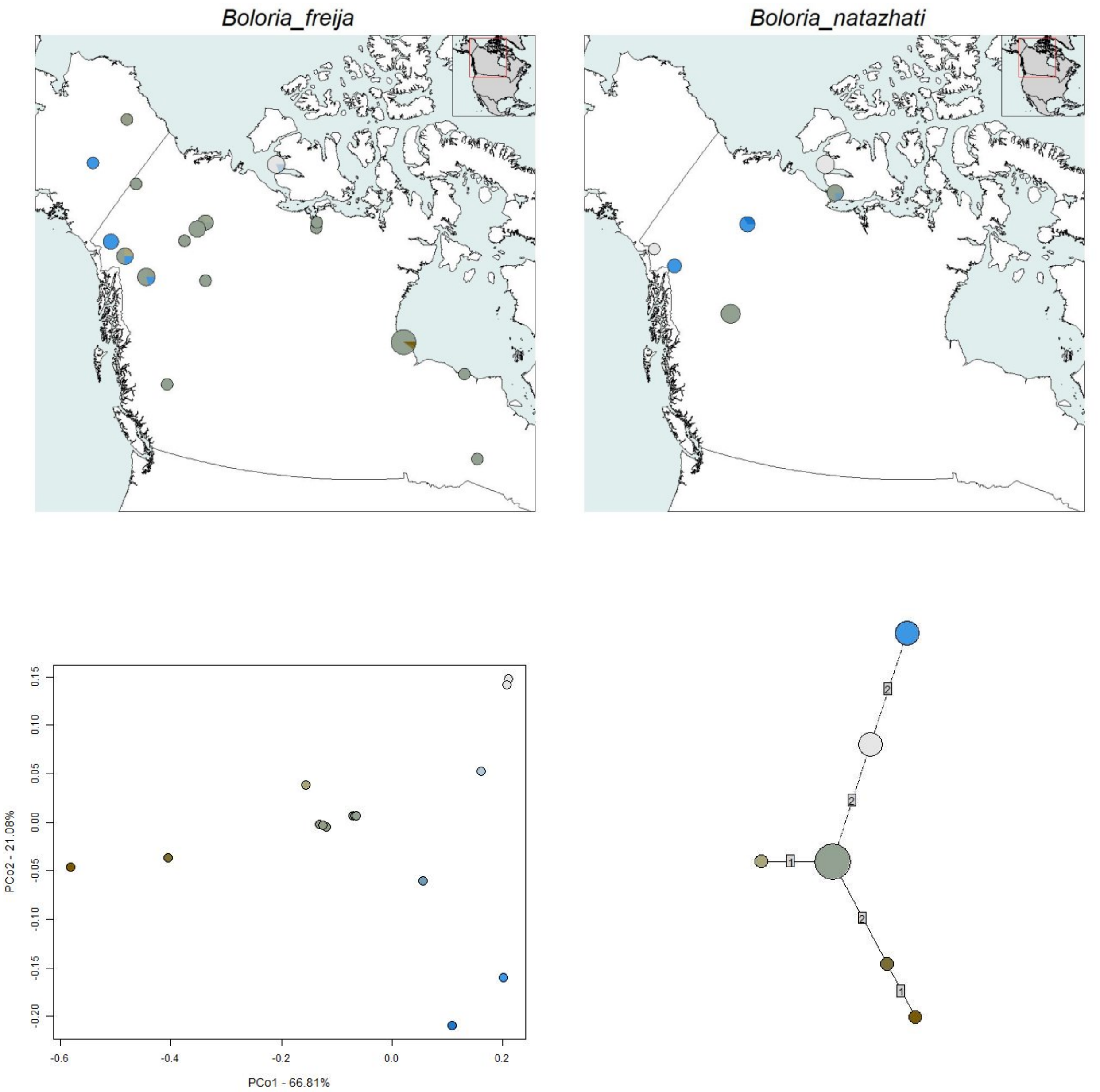

Figure 993: Haplotype maps, PCoA projection, and haplotype network for seven *Boloria* species displaying barcode sharing.

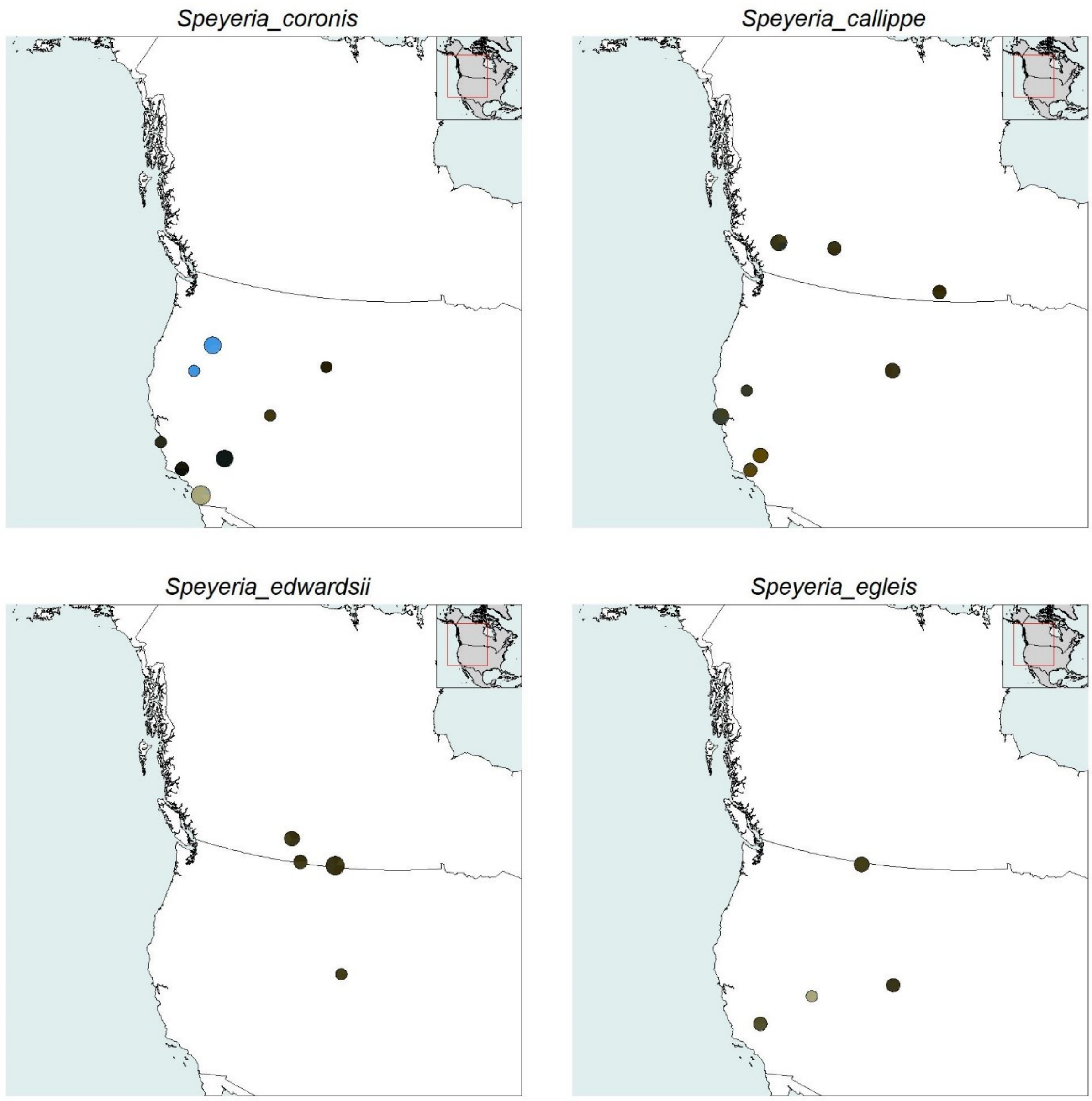
Speyeria_coronis
Speyeria_callippe
Speyeria_edwardsii
Speyeria_egleis

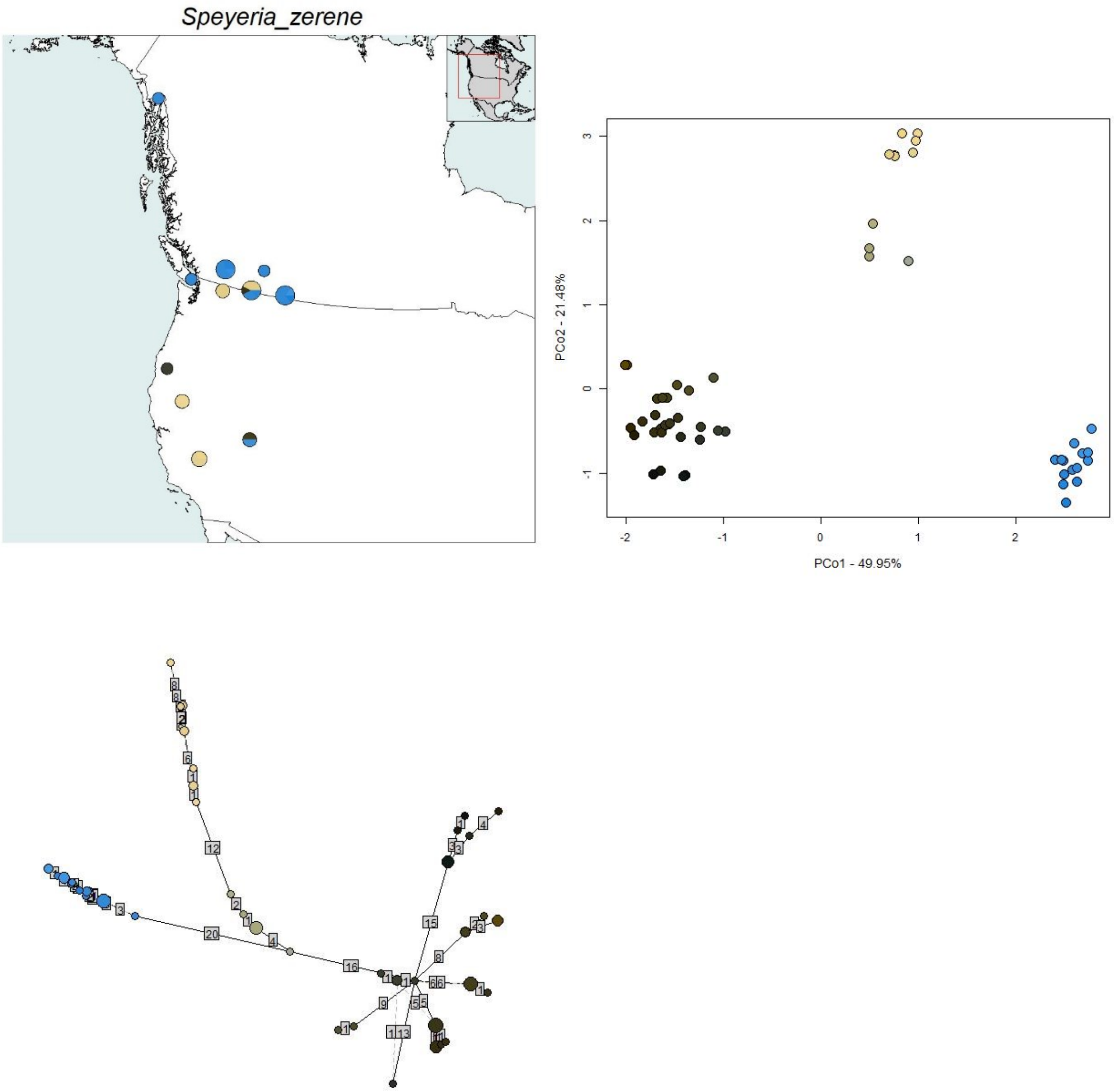

Figure 994: Haplotype maps, PCoA projection, and haplotype network for five *Speyeria* species displaying barcode sharing.

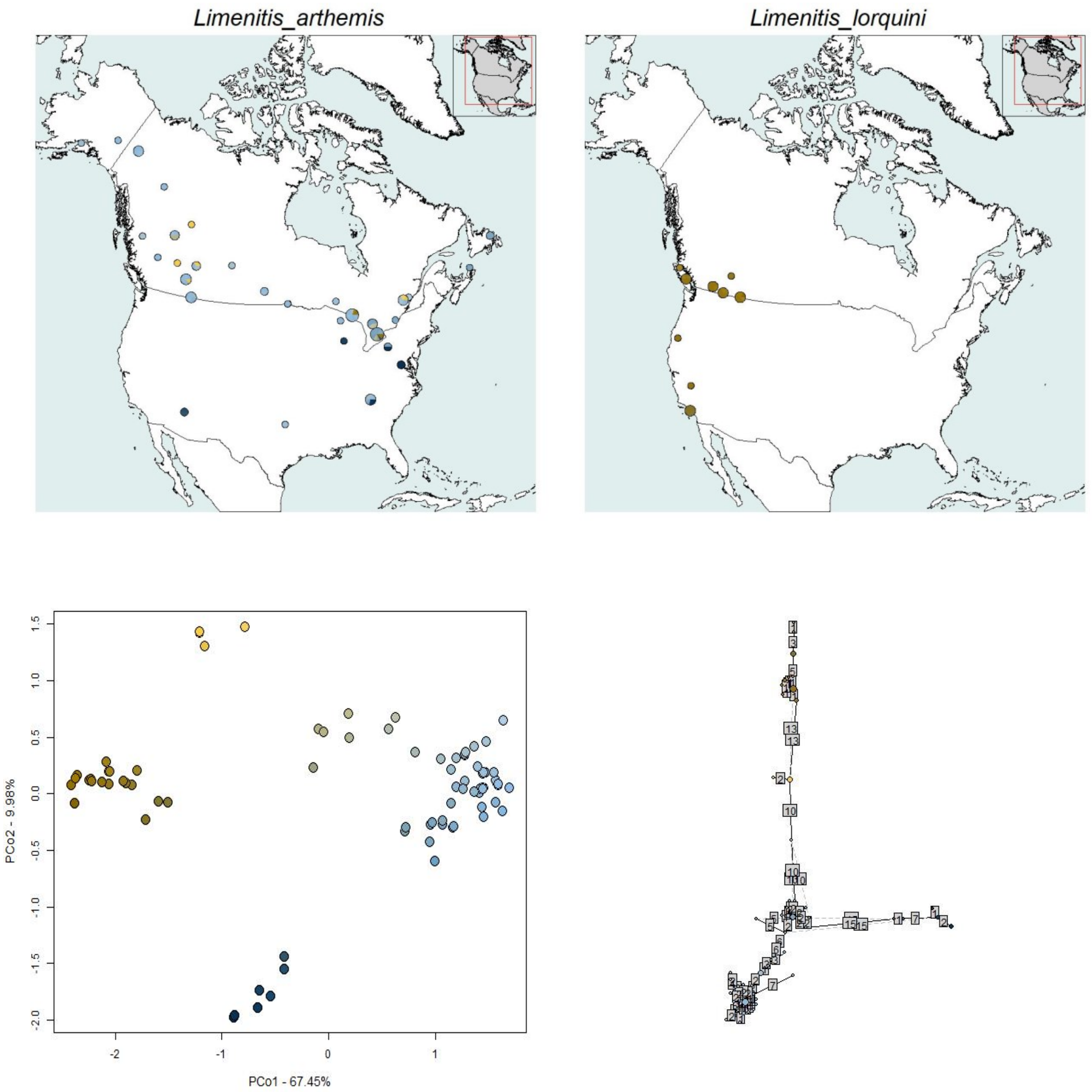

Figure 995: Haplotype maps, PCoA projection, and haplotype network for two *Limenitis* species displaying barcode sharing.

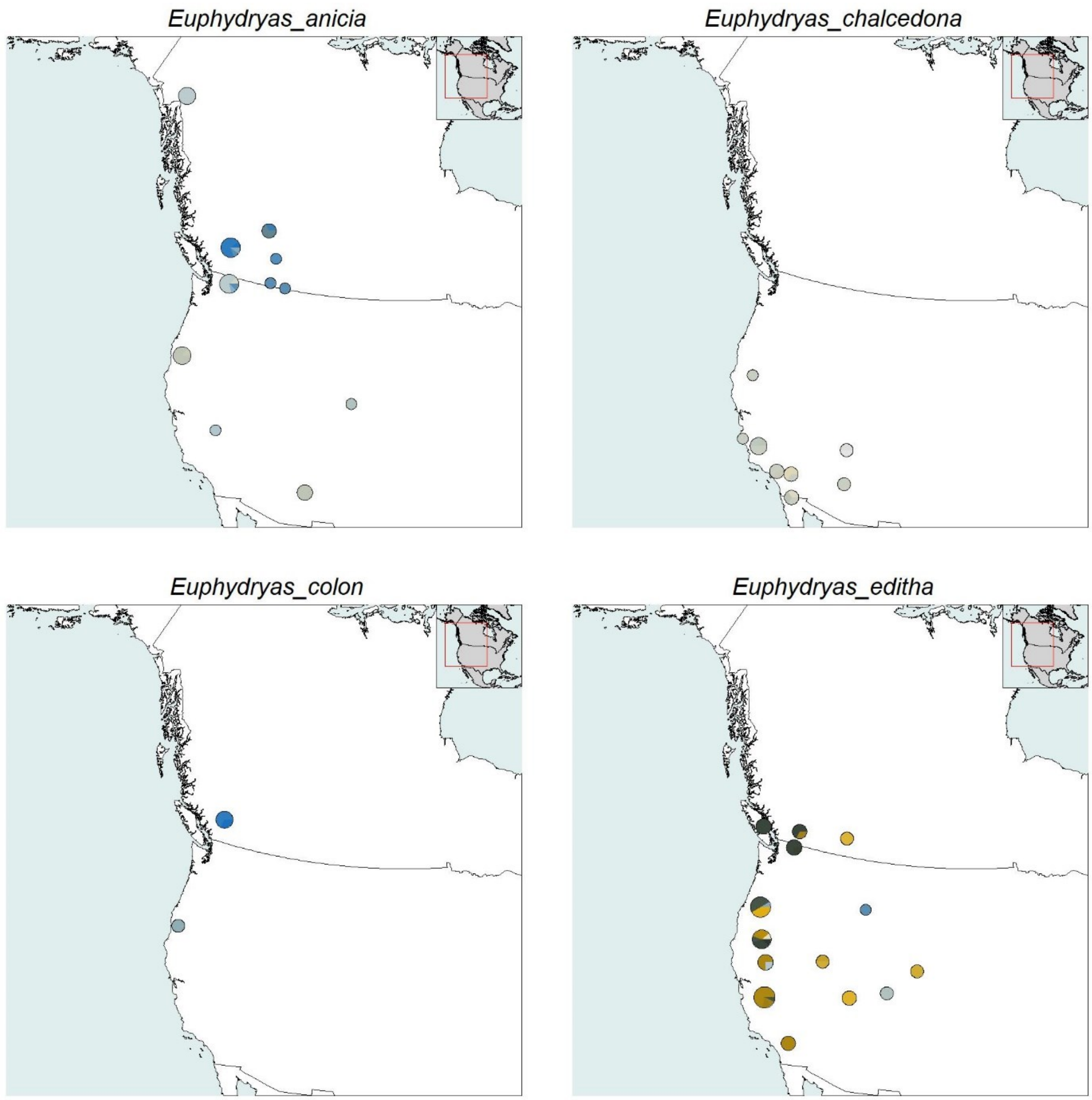
Euphydryas_anicia
Euphydryas_chalcedona
Euphydryas_colon
Euphydryas_editha

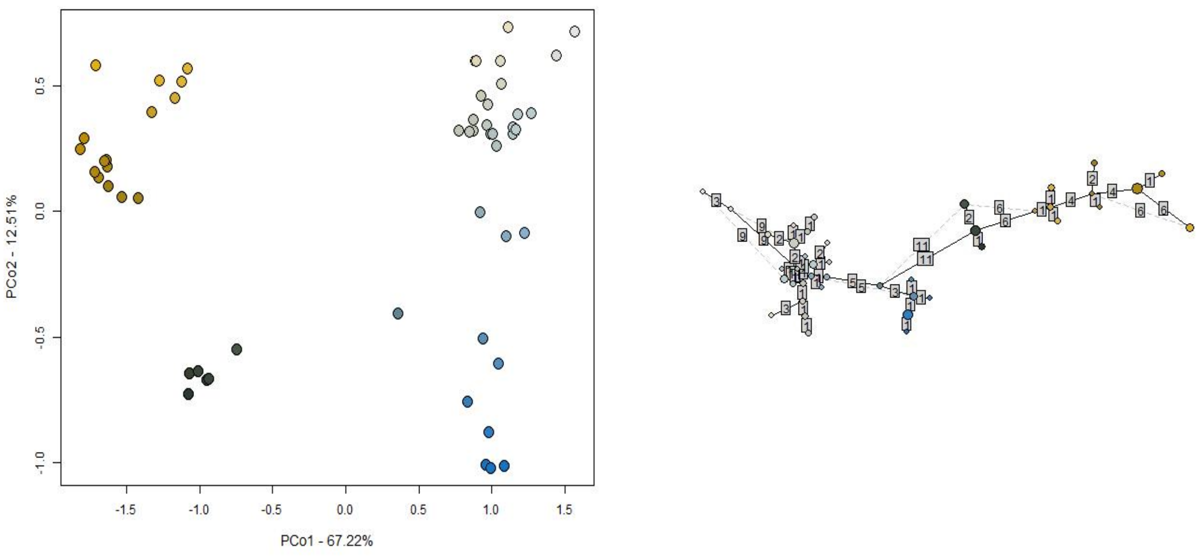

Figure 996: Haplotype maps, PCoA projection, and haplotype network for four *Euphydryas* species displaying barcode sharing.

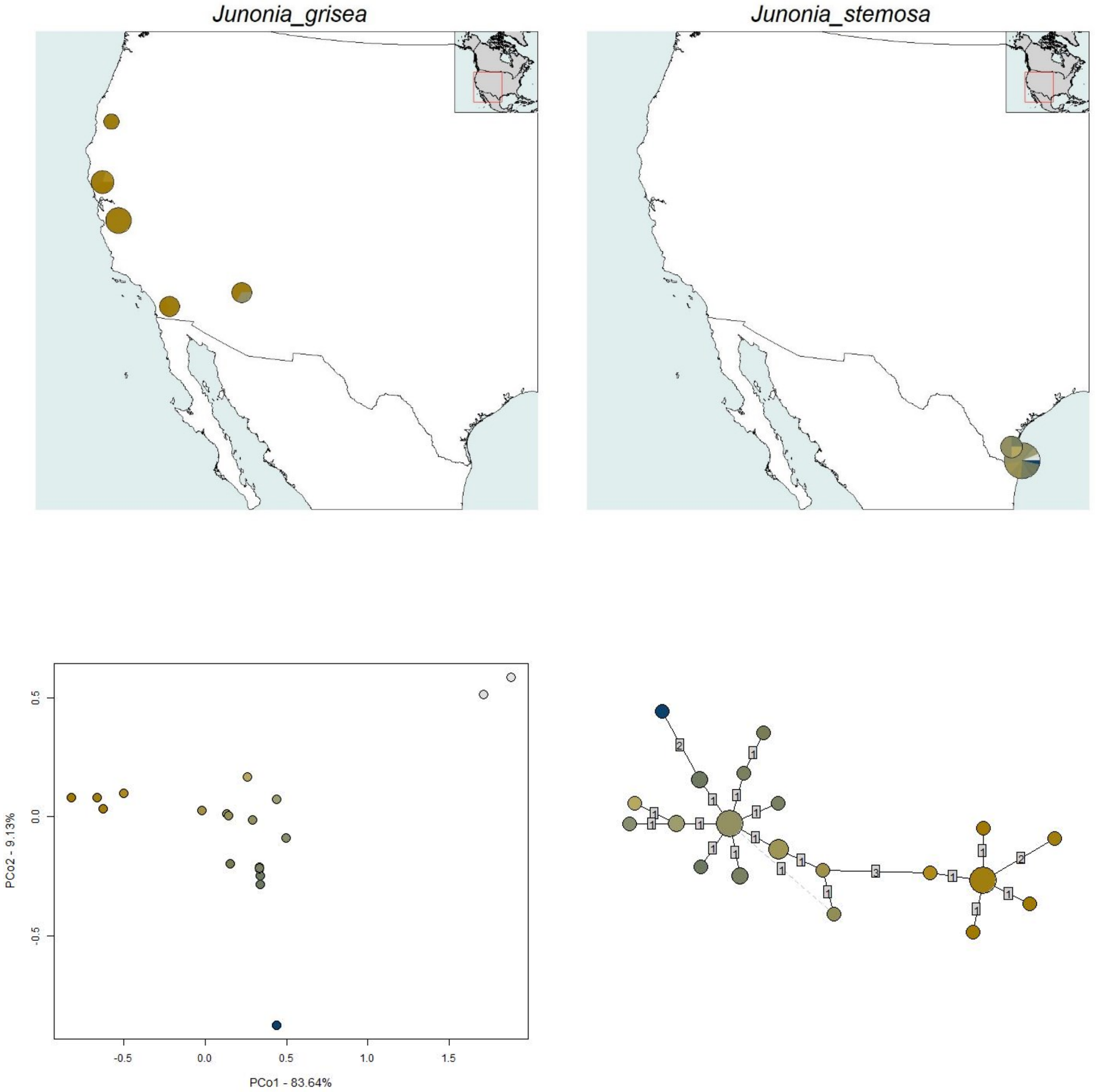

Figure 997: Haplotype maps, PCoA projection, and haplotype network for four *Junonia* species displaying barcode sharing.

Phyciodes_tharos

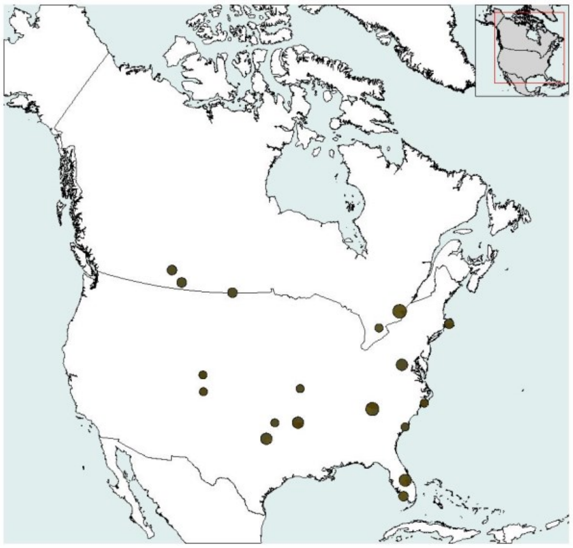

Phyciodes_pulchella

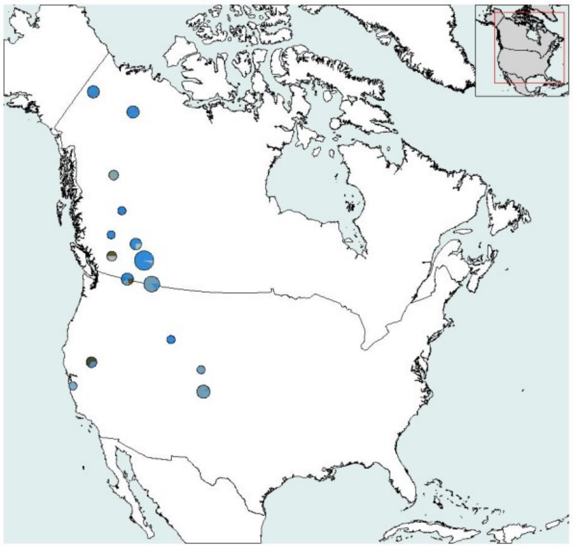

Phyciodes_cocyta

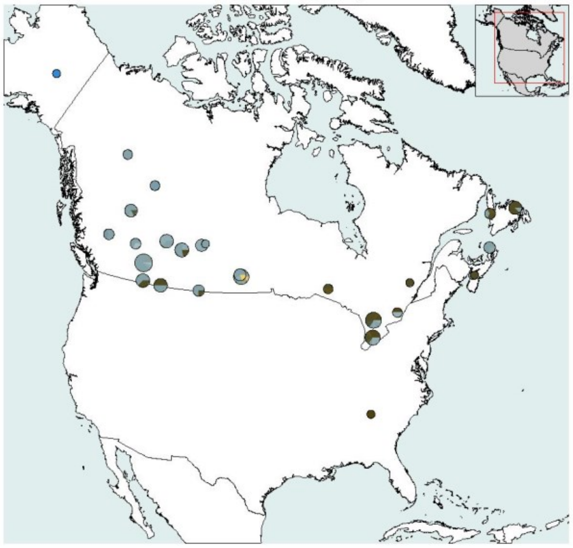

Phyciodes_batesii

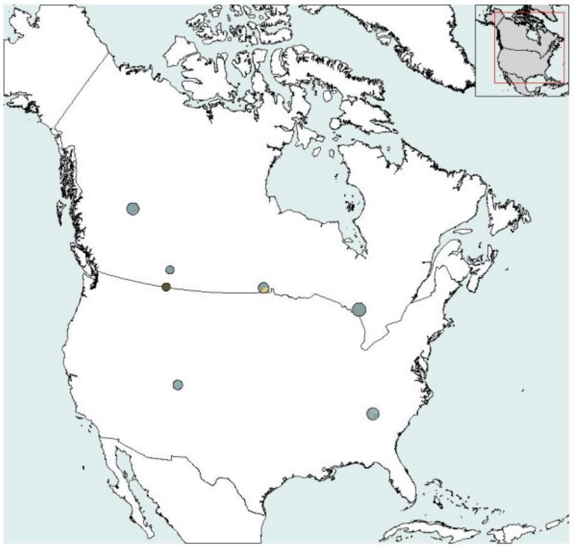

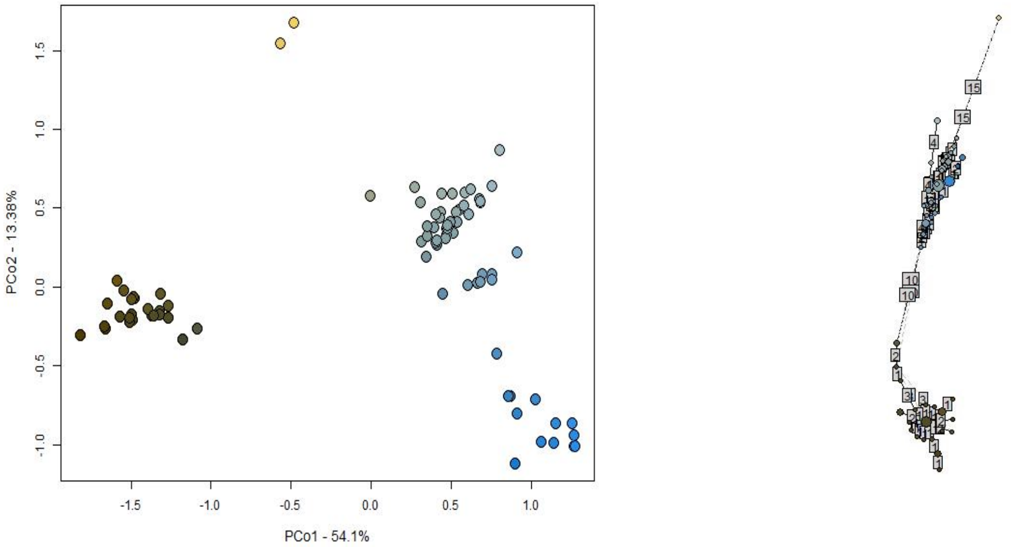

Figure 998: Haplotype maps, PCoA projection, and haplotype network for four *Phyciodes* species displaying barcode sharing.

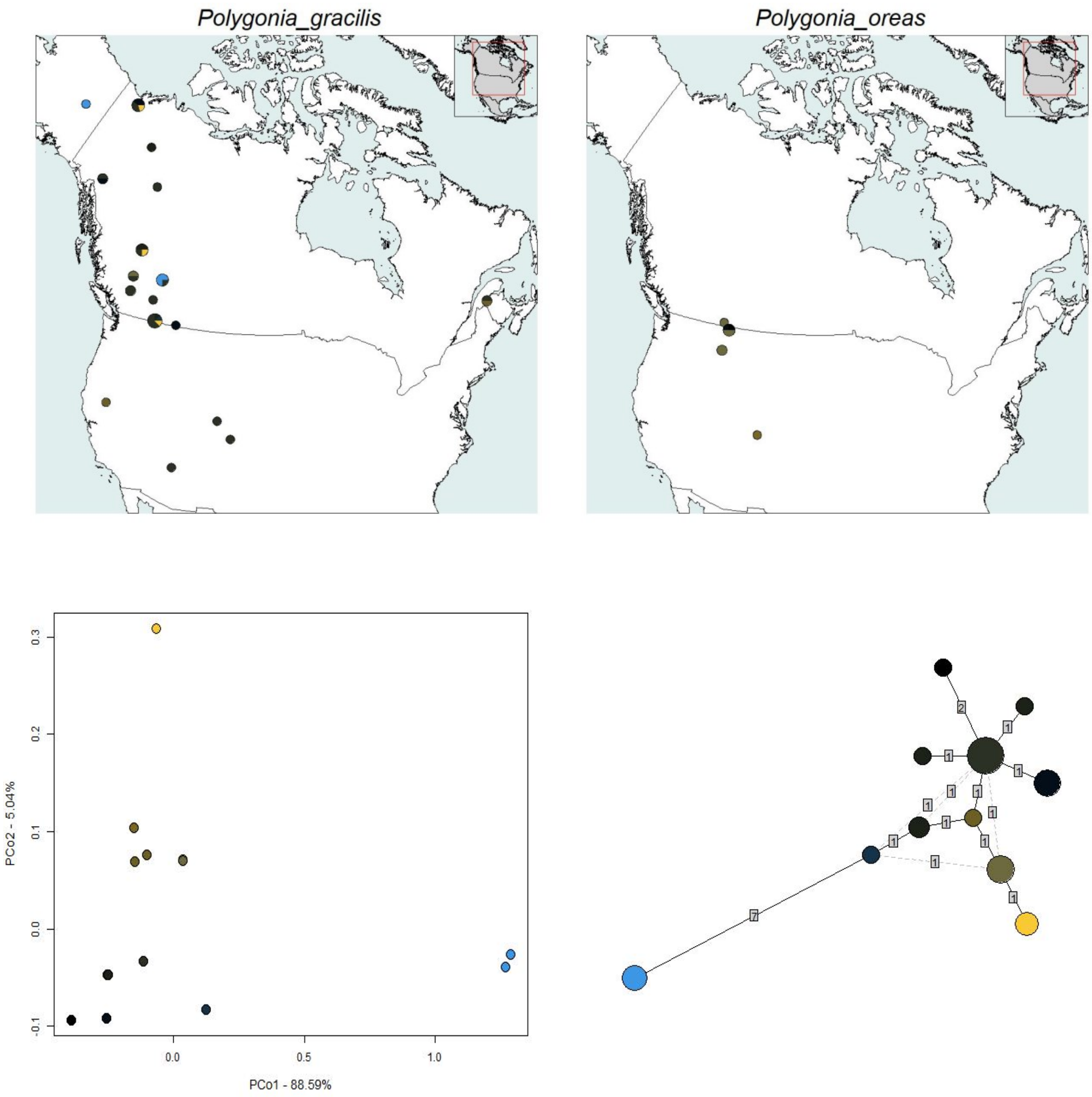

Figure 999: Haplotype maps, PCoA projection, and haplotype network for two *Polygonia* species displaying barcode sharing.

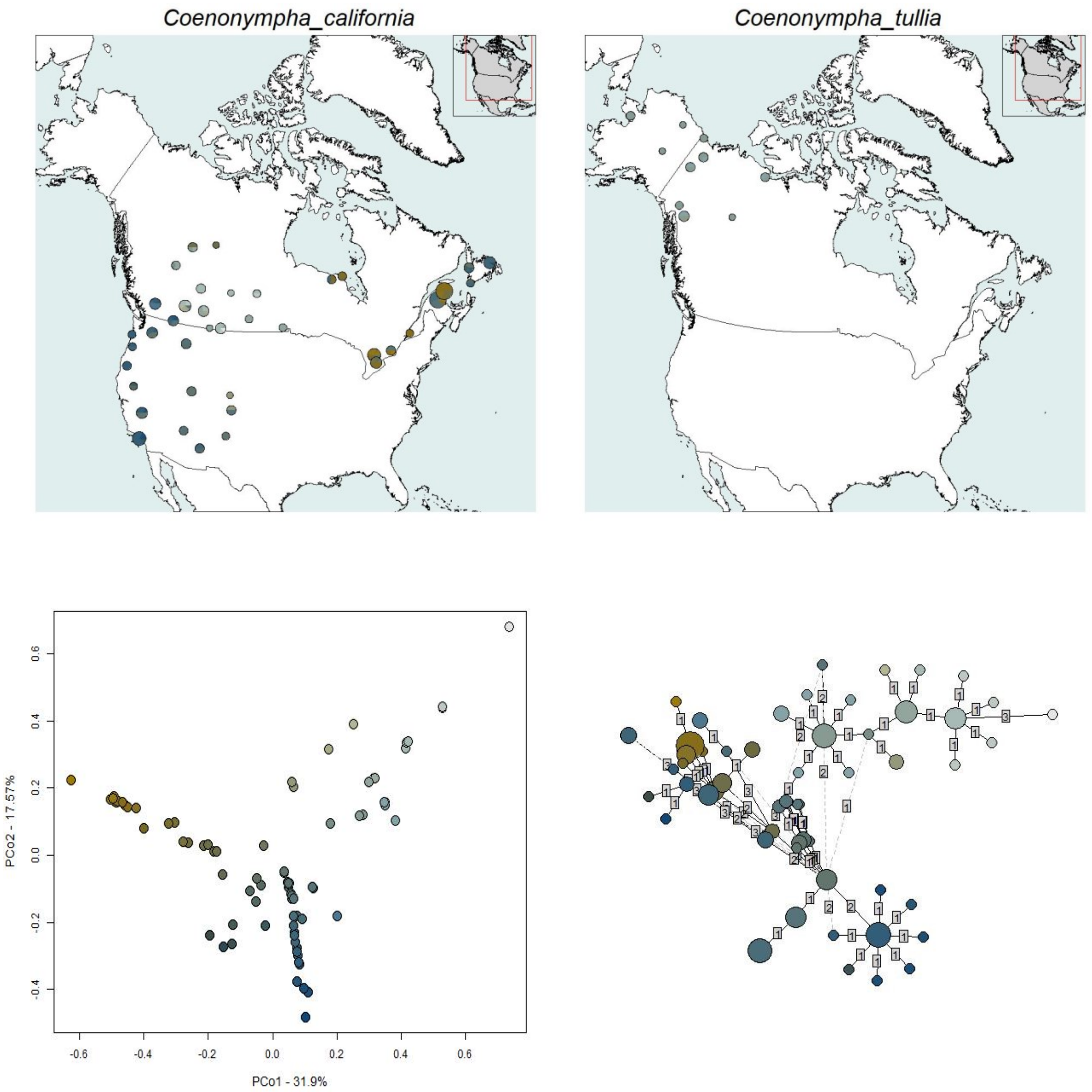

Figure 1000: Haplotype maps, PCoA projection, and haplotype network for two *Coenonympha* species displaying barcode sharing.

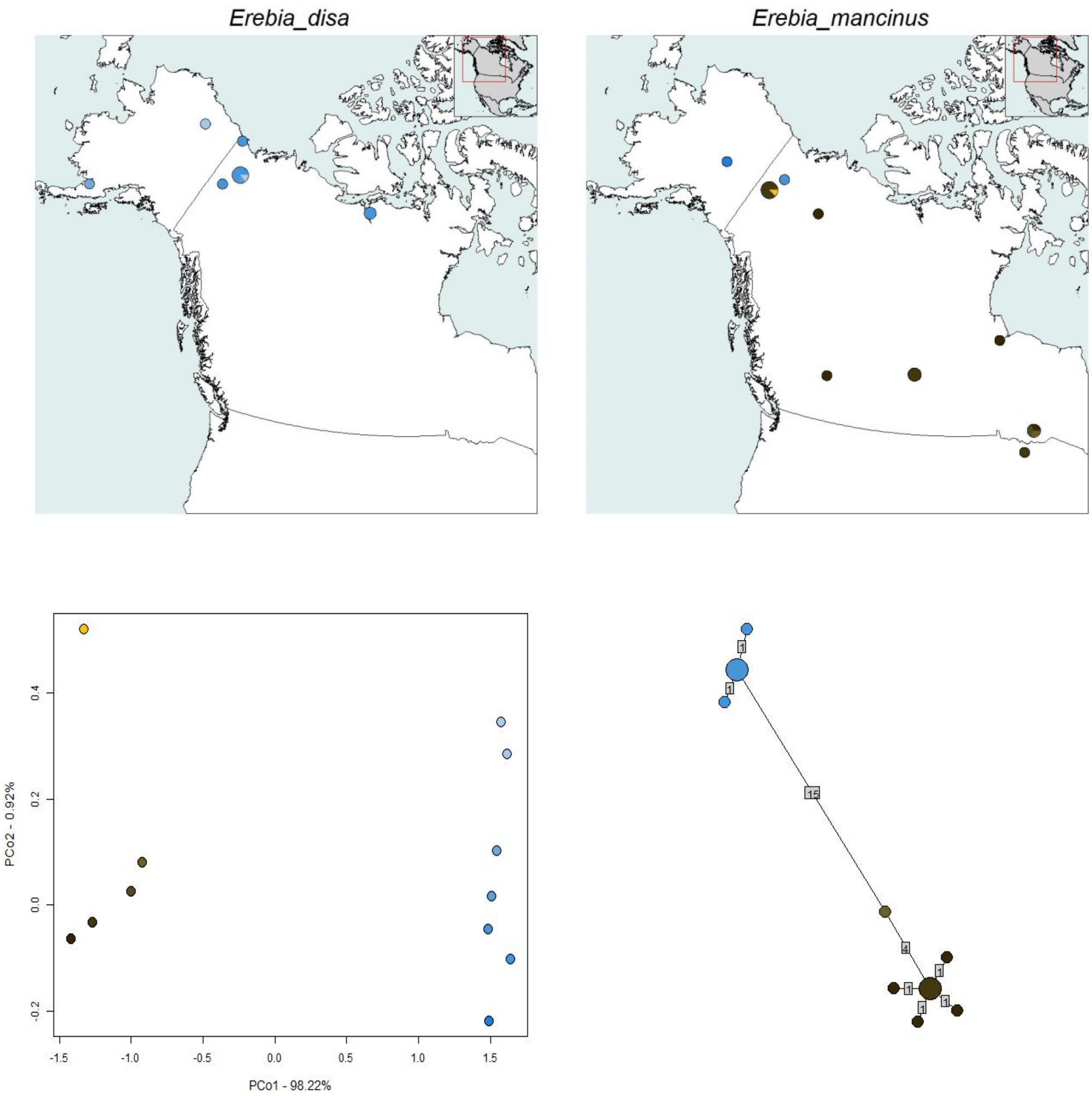

Figure 1001: Haplotype maps, PCoA projection, and haplotype network for two *Erebia* species displaying barcode sharing.

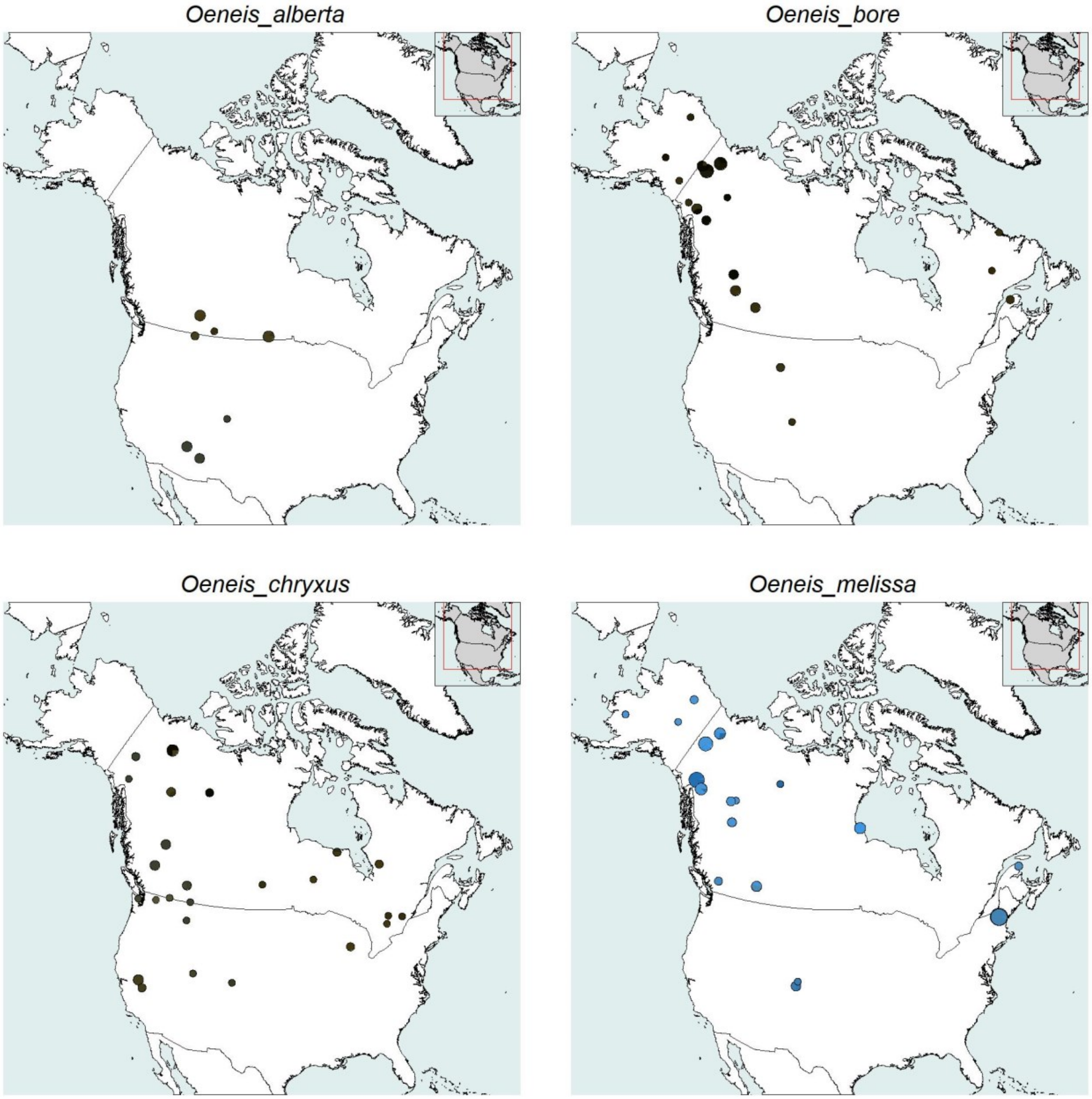
Oeneis_alberta
Oeneis_bore
Oeneis_chryxus
Oeneis_melissa

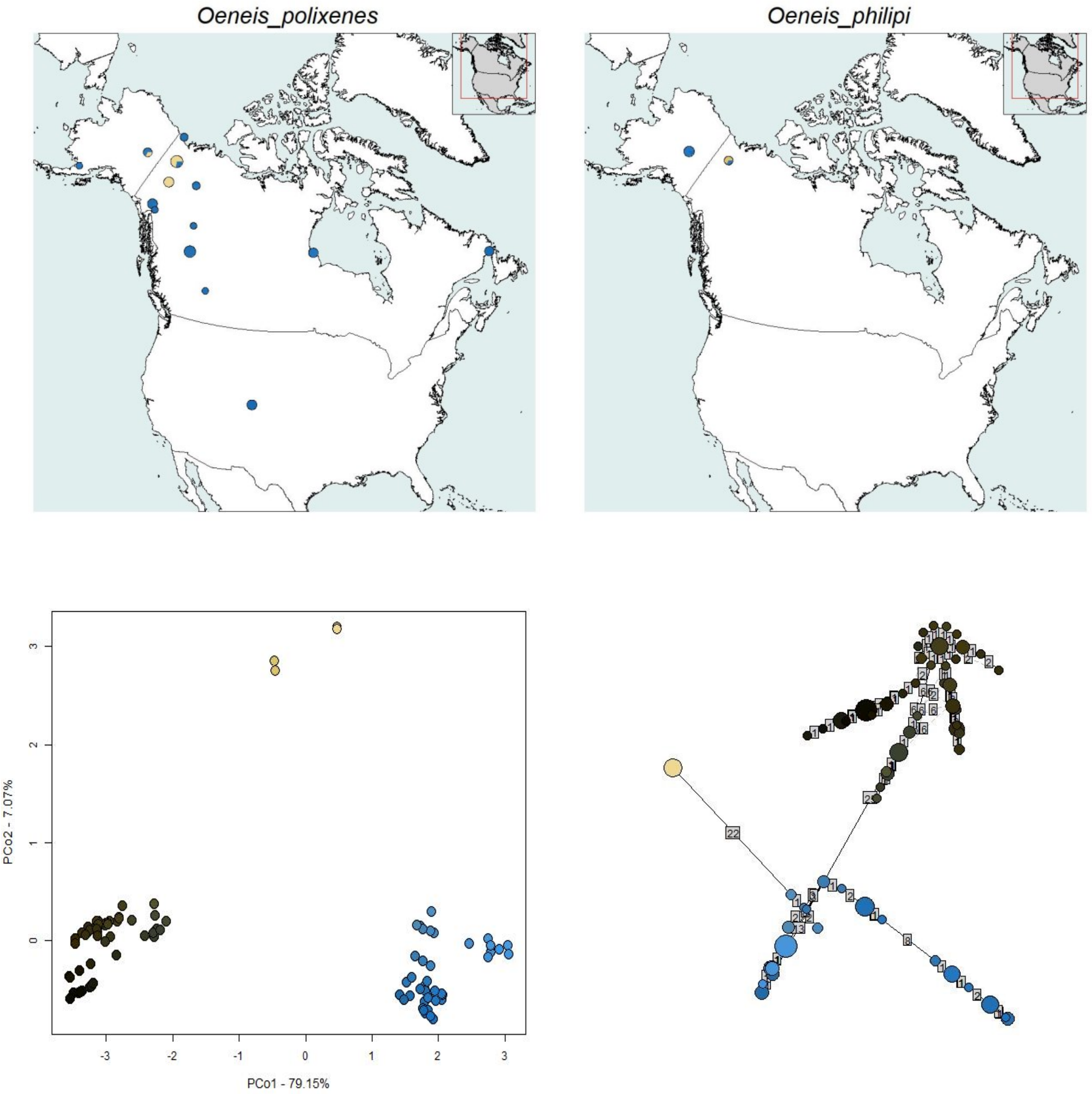

Figure 1002: Haplotype maps, PCoA projection, and haplotype network for six *Oeneis* species displaying barcode sharing.

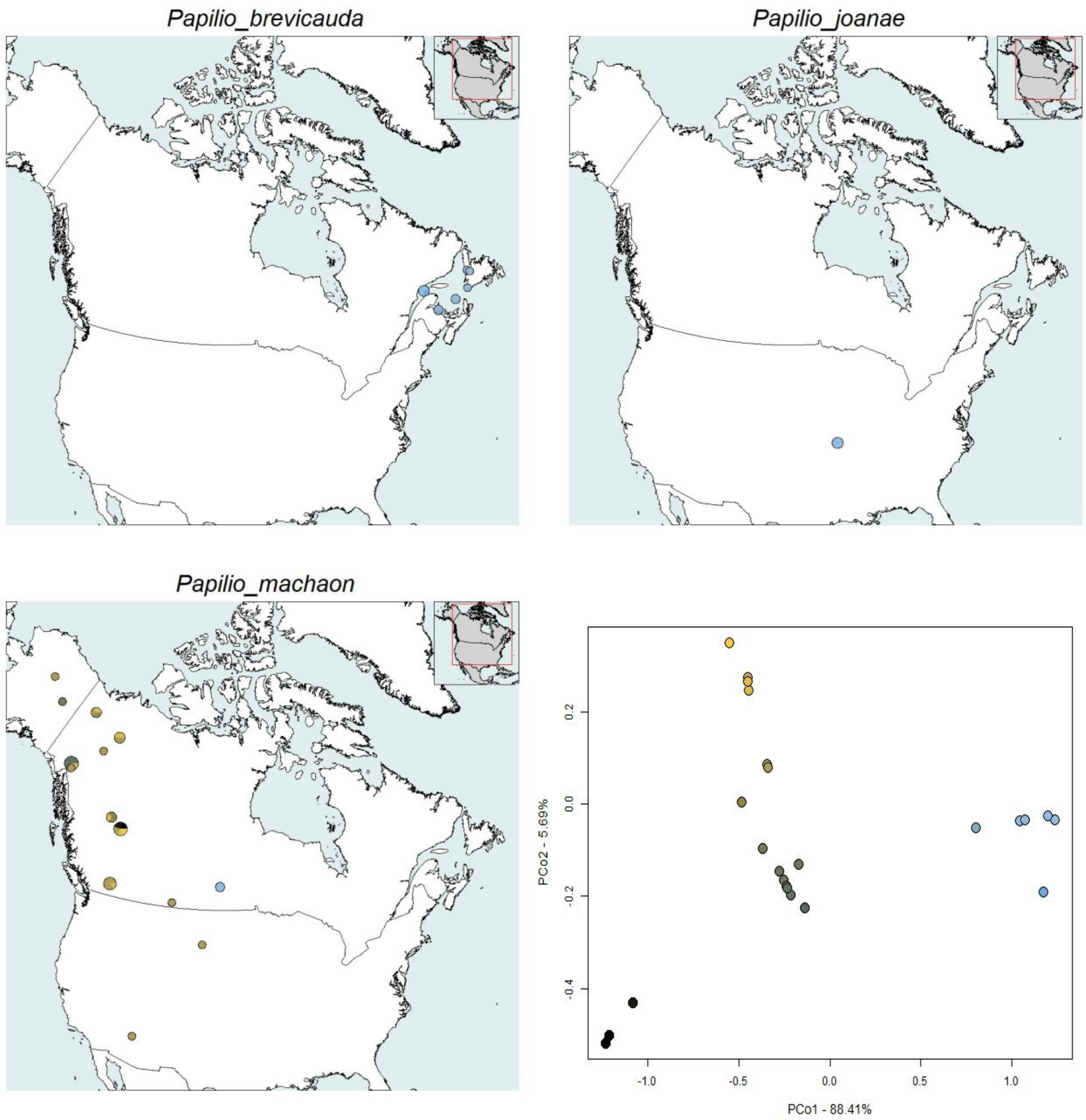
Papilio_brevicauda
Papilio_joanae
Papilio_machaon
PCo2 - 5.69%
PCo1 - 88.41%
0.2
0.0
-0.2
-0.4
-1.0
-0.5
0.0
0.5
1.0

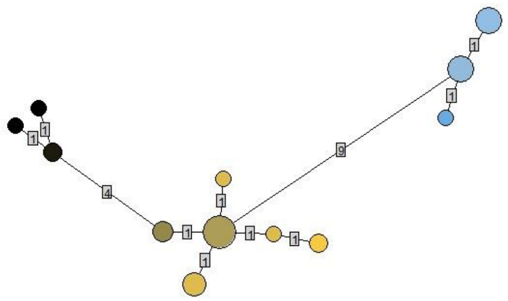

Figure 1003: Haplotype maps, PCoA projection, and haplotype network for three *Papilio* species displaying barcode sharing.

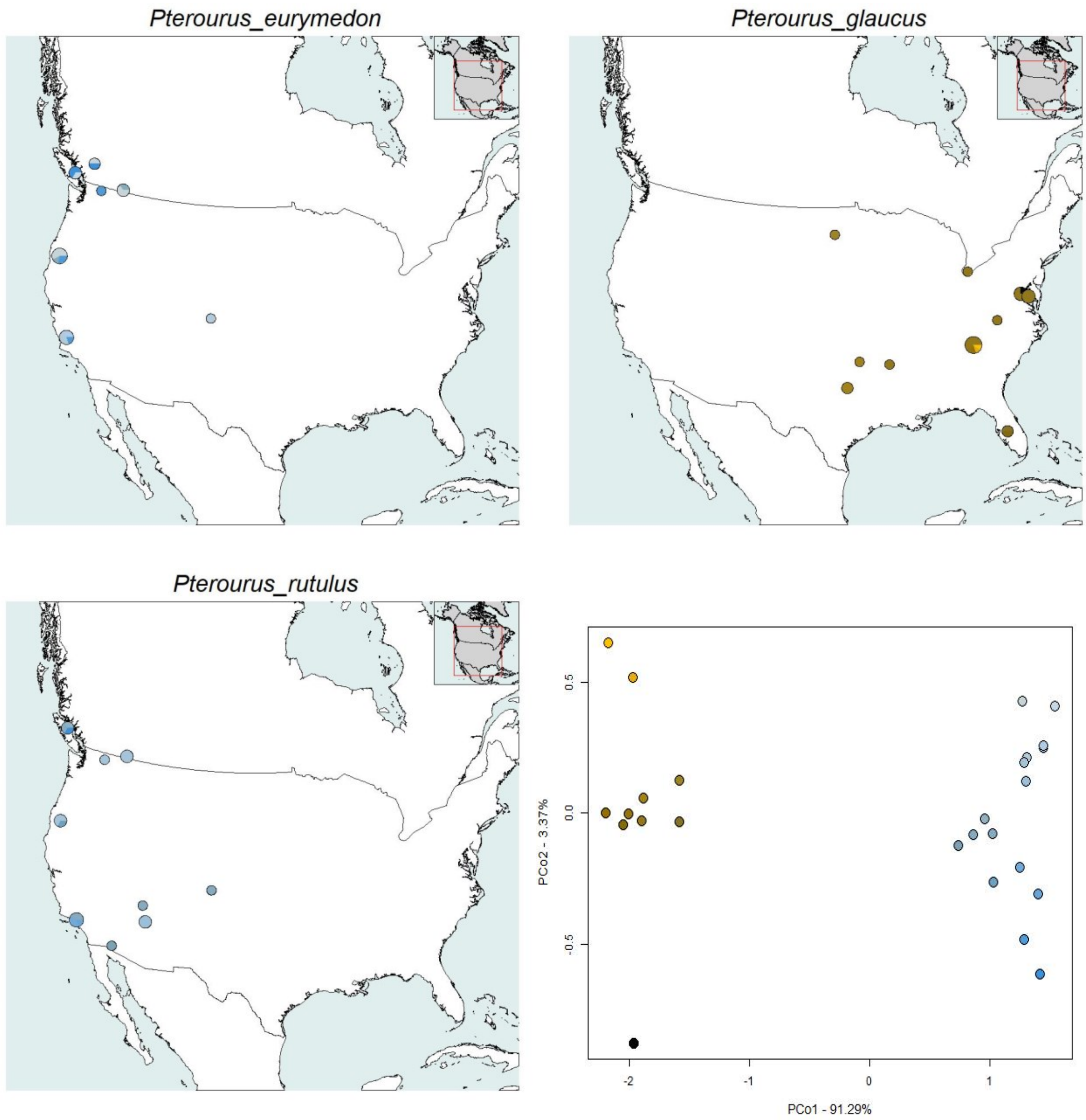
Pterourus_eurymedon
Pterourus_glaucus
Pterourus_rutulus
PCo2 - 3.37%
PCo1 - 91.29%
0.5
0.0
-0.5
-2
-1
0
1

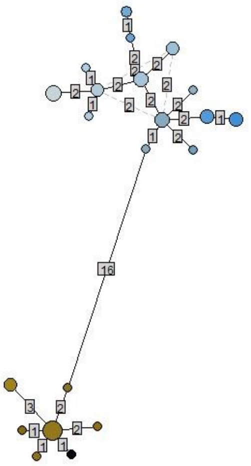

Figure 1004: Haplotype maps, PCoA projection, and haplotype network for three *Pterourus* species displaying barcode sharing.

Colias_alexandra

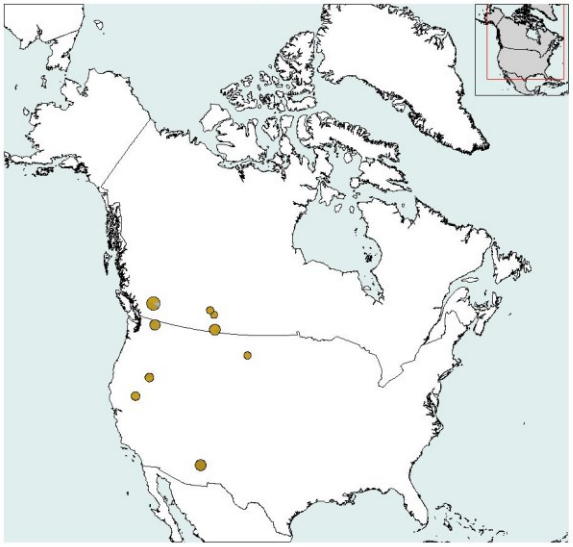

Colias_behrii

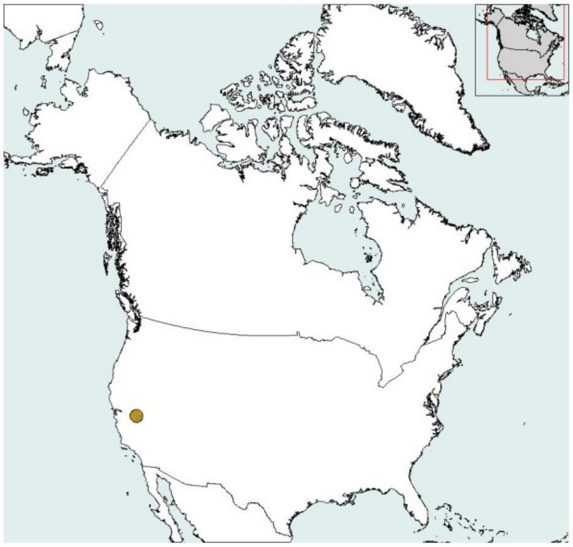

Colias_boothii

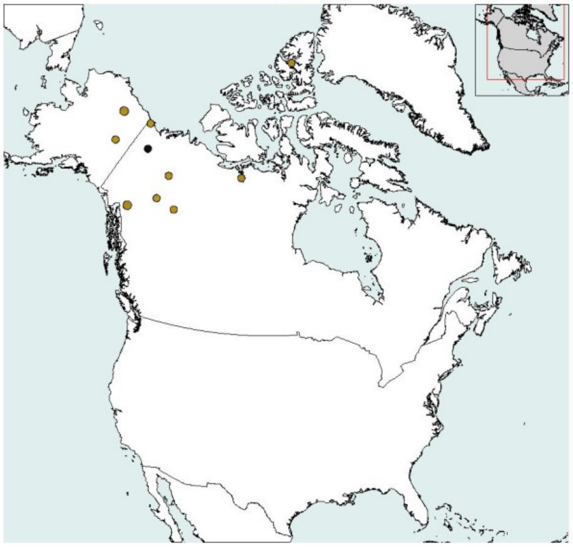

Colias_christina

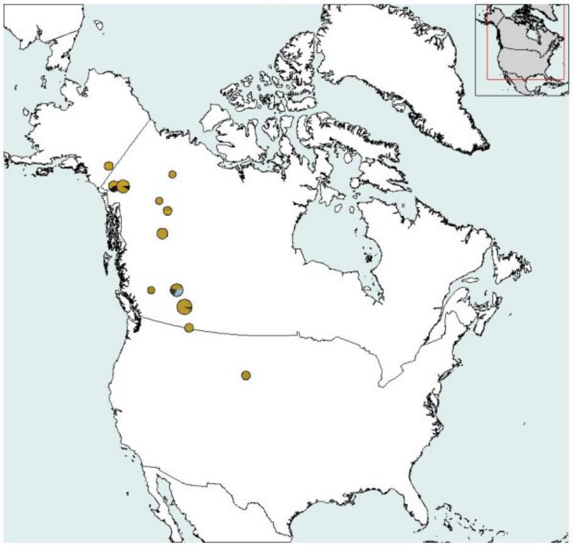

Colias_eriphyle

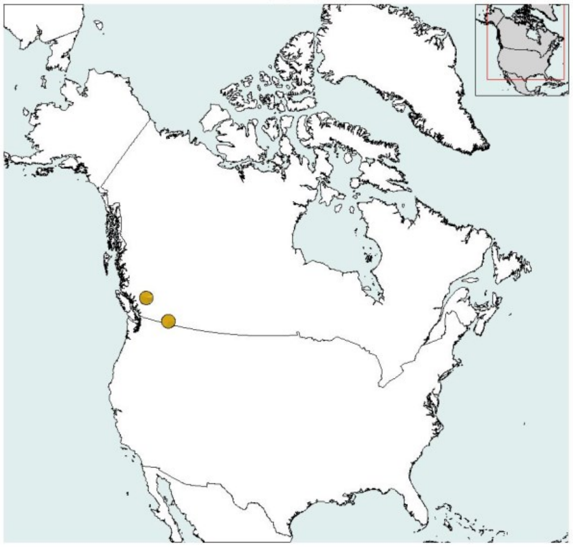

Colias_eurytheme

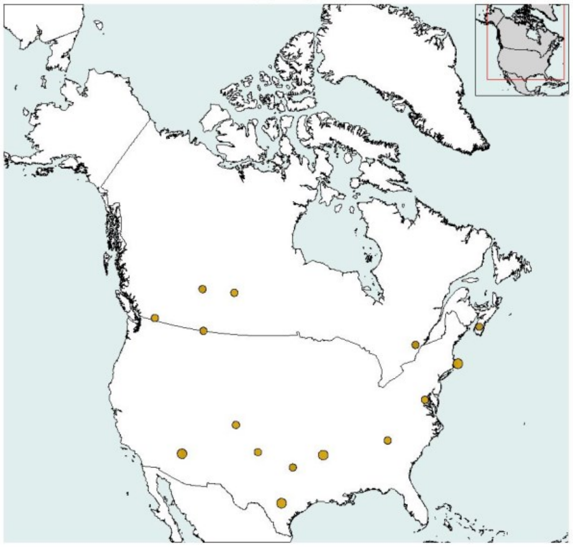

Colias_gigantea

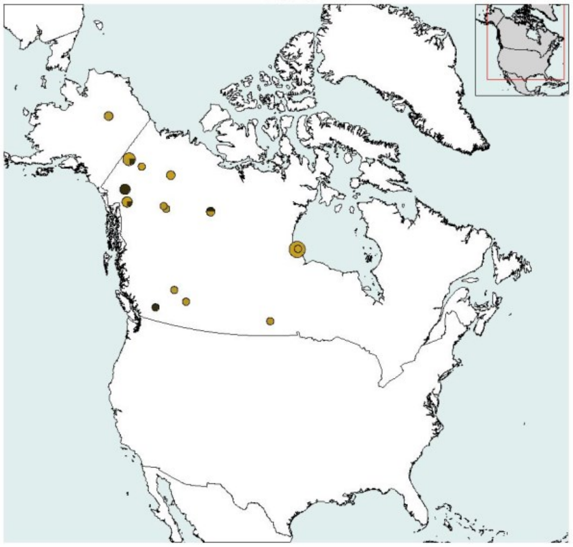

Colias_harfordii

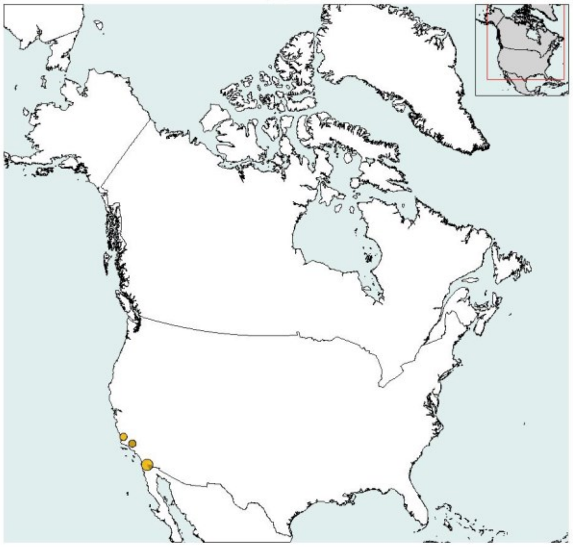

Colias_hecla

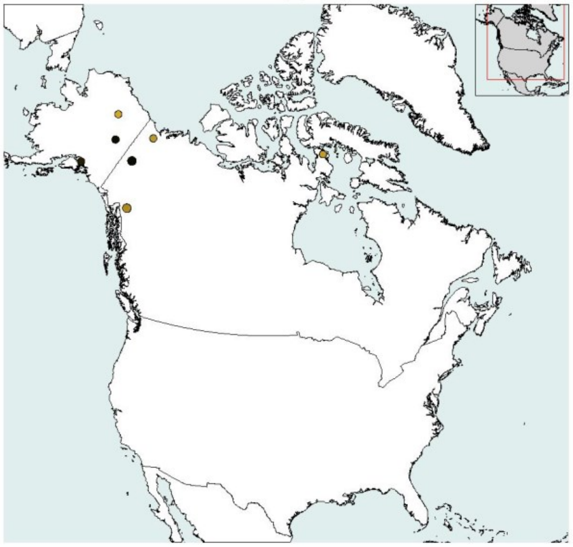

Colias_interior

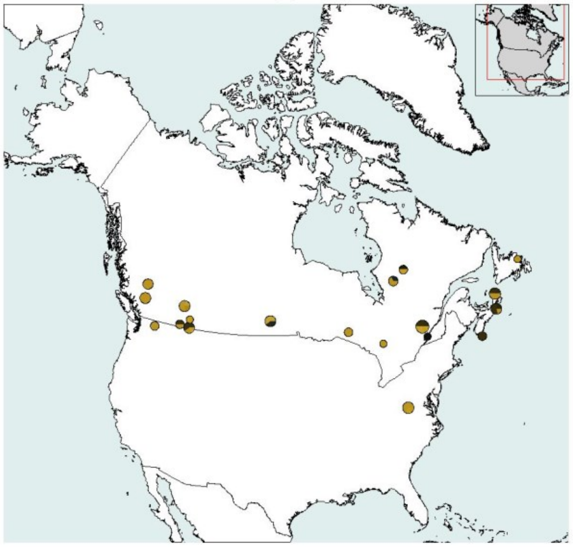

Colias_johanseni

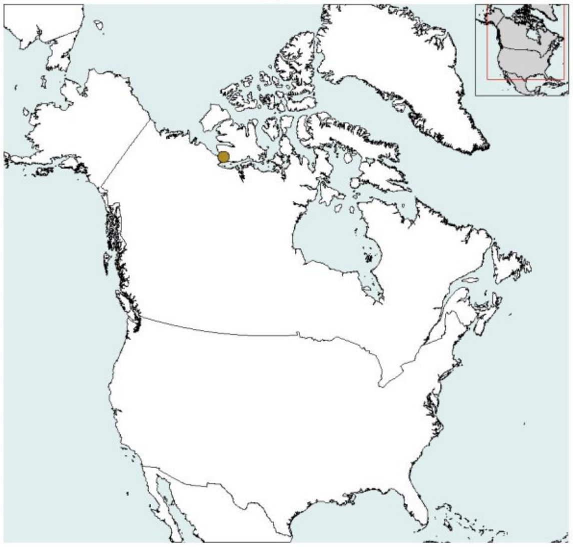

Colias_meadii

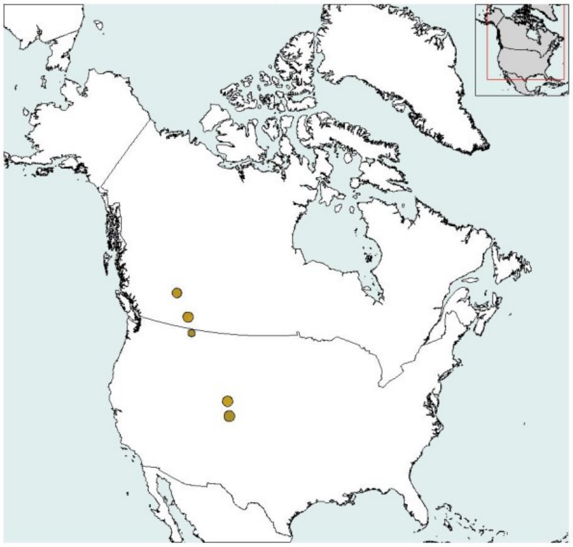

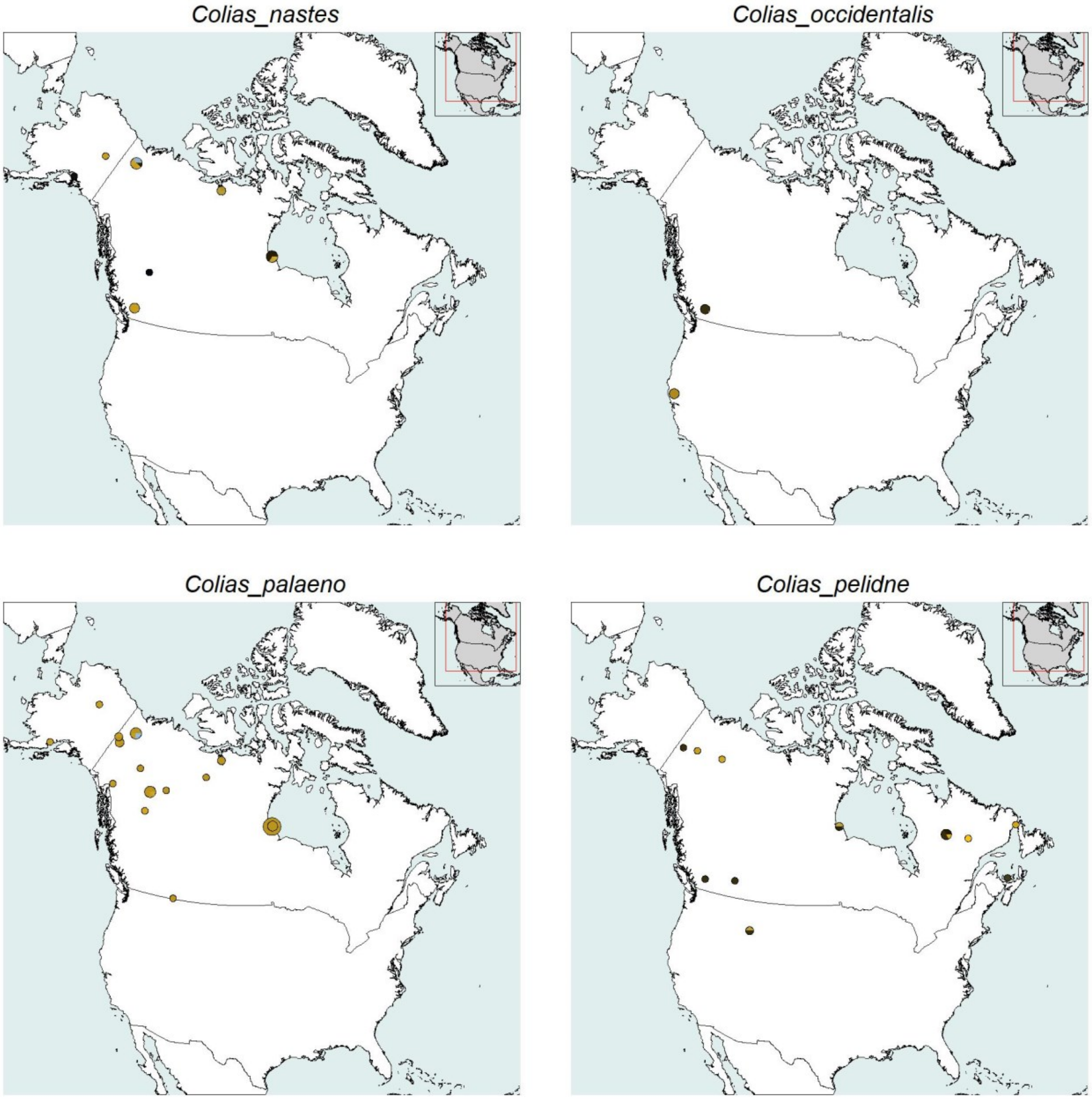
Colias_nastes
Colias_occidentalis
Colias_palaeno
Colias_pelidne

Colias_philodice

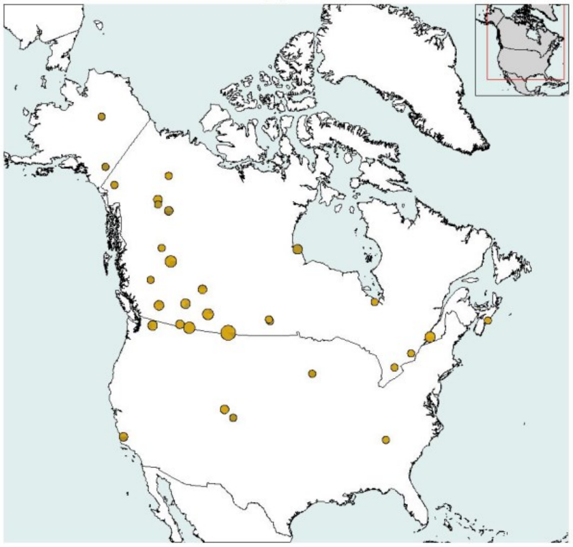

Colias_rankinensis

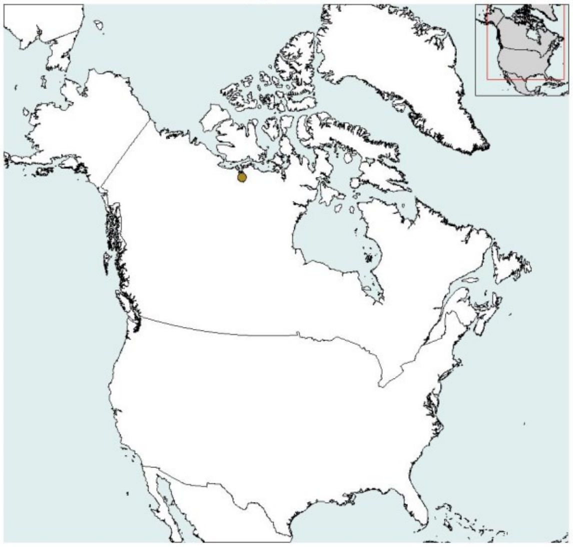

Colias_scudderii

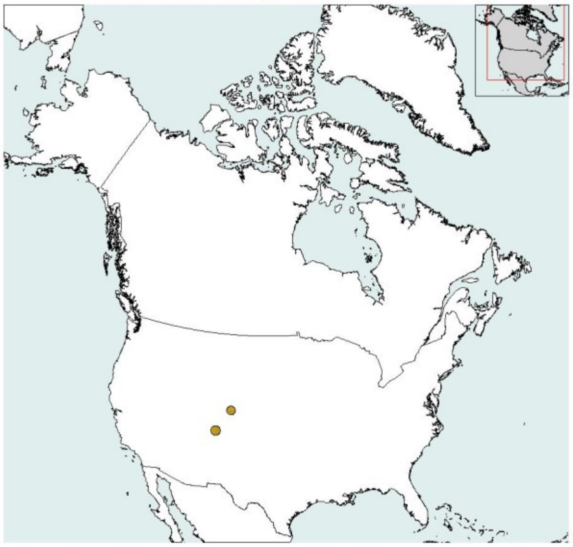

Colias_skinneri

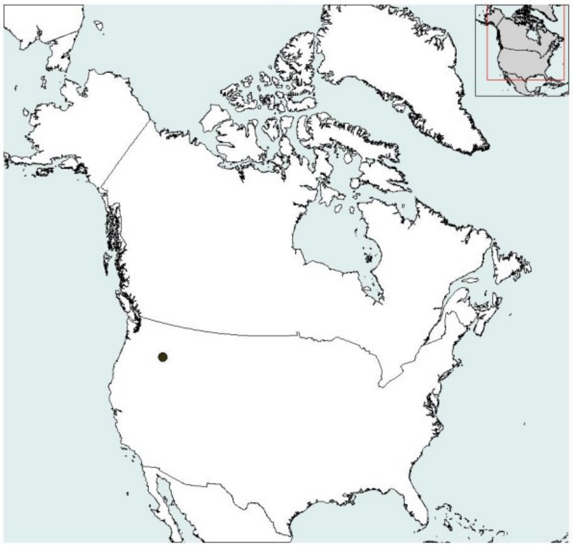

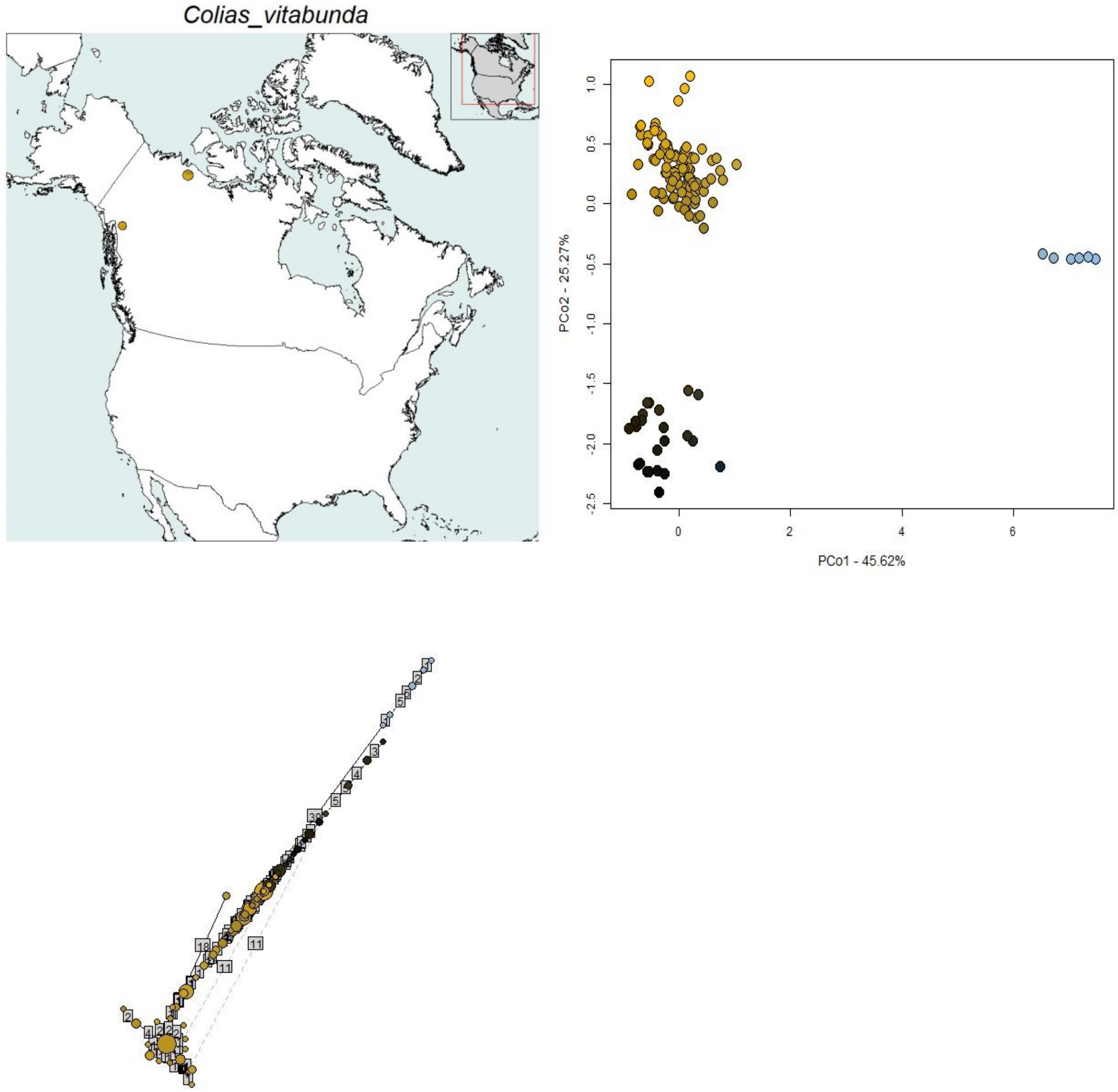

Figure 1005: Haplotype maps, PCoA projection, and haplotype network for 21 *Colias* species displaying barcode sharing.

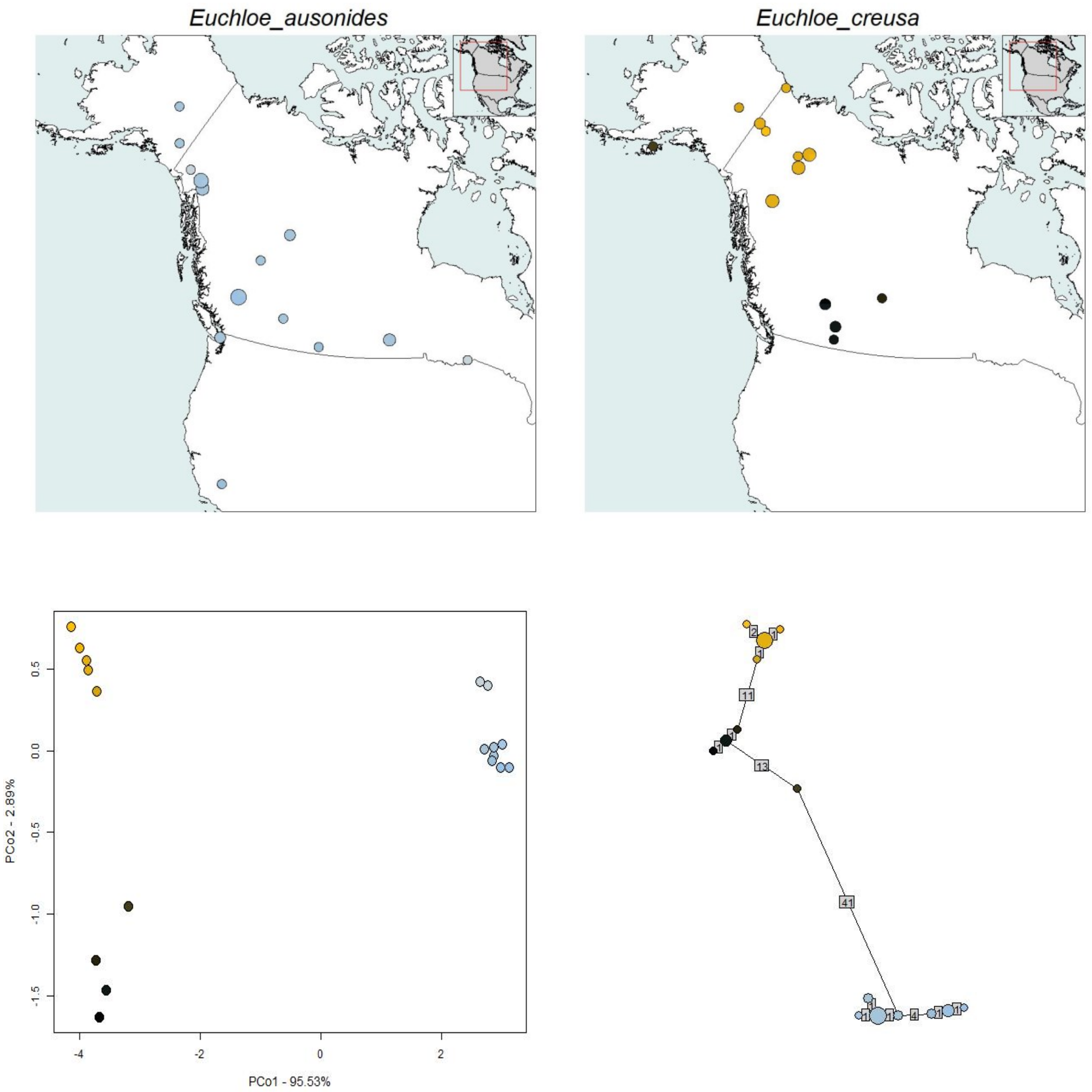

Figure 1006: Haplotype maps, PCoA projection, and haplotype network for two *Euchloe* species displaying barcode sharing.

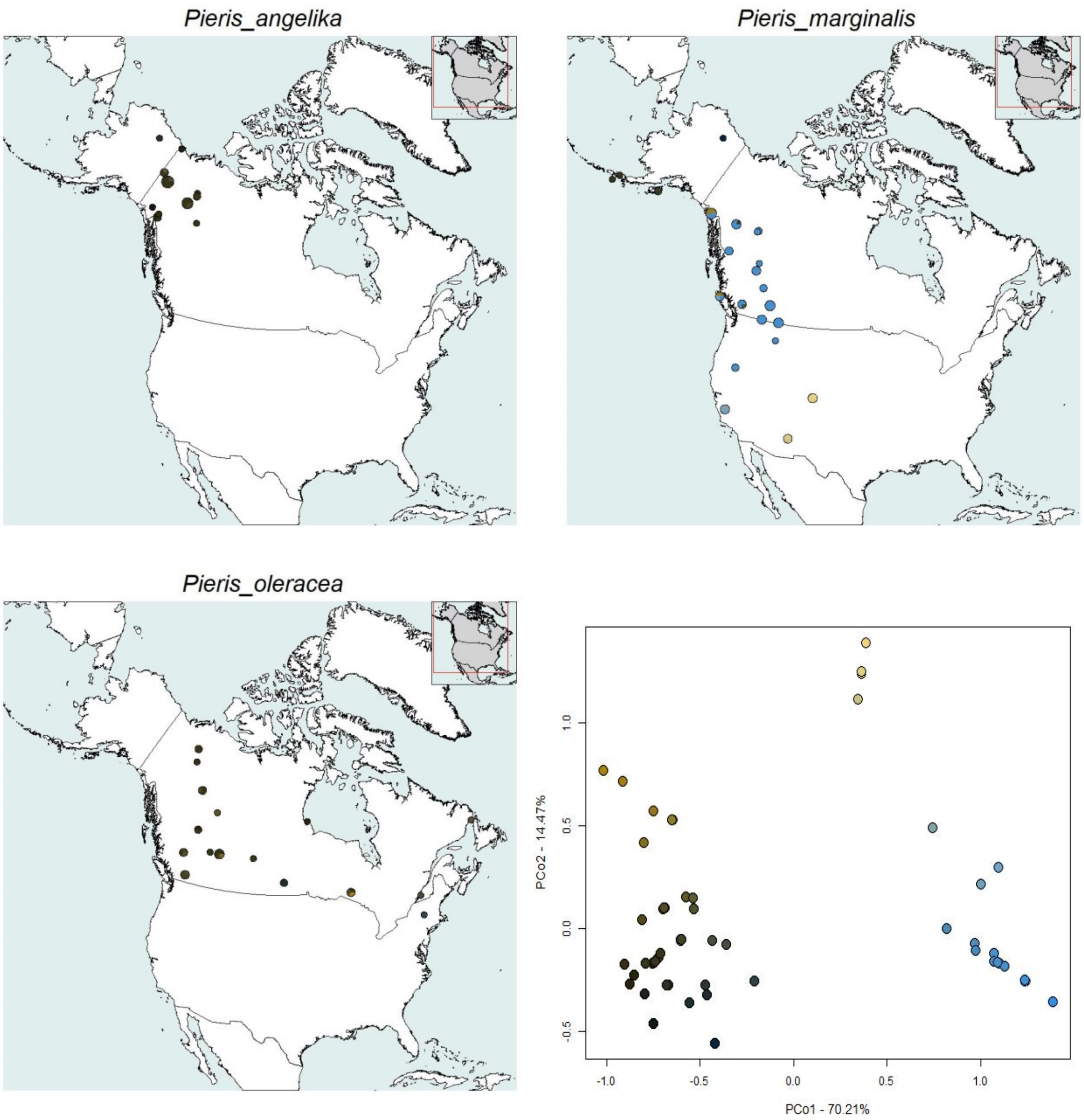
Pieris_angelika
Pieris_marginalis
Pieris_oleracea
PCo2 - 14.47%
PCo1 - 70.21%
-1.0
-0.5
0.0
0.5
1.0

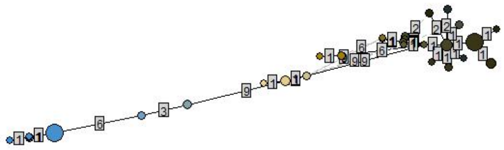

Figure 1007: Haplotype maps, PCoA projection, and haplotype network for three *Pieris* species displaying barcode sharing.

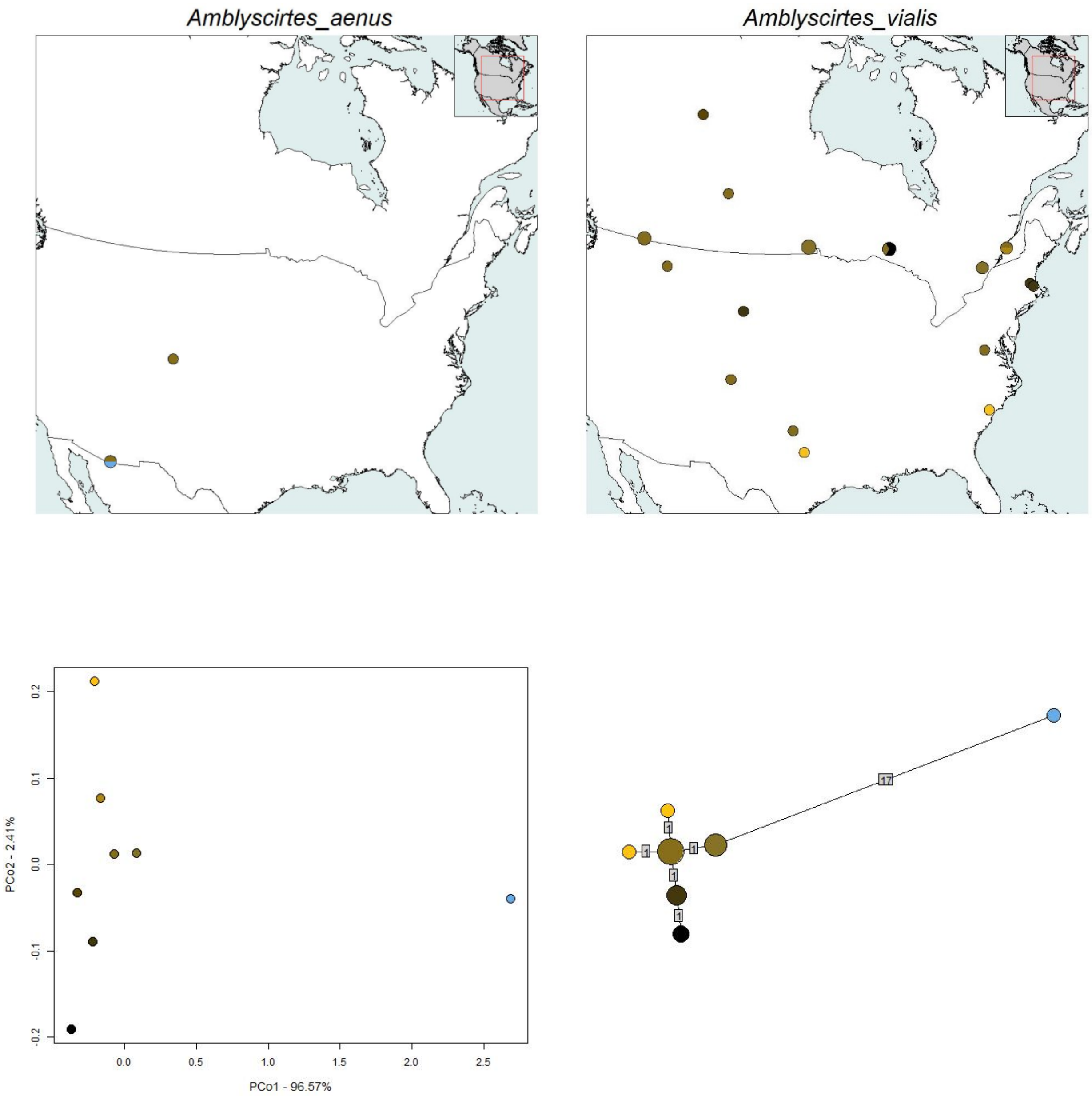

Figure 980: Haplotype maps, PCoA projection, and haplotype network for two *Amblyscirtes* species displaying barcode sharing.

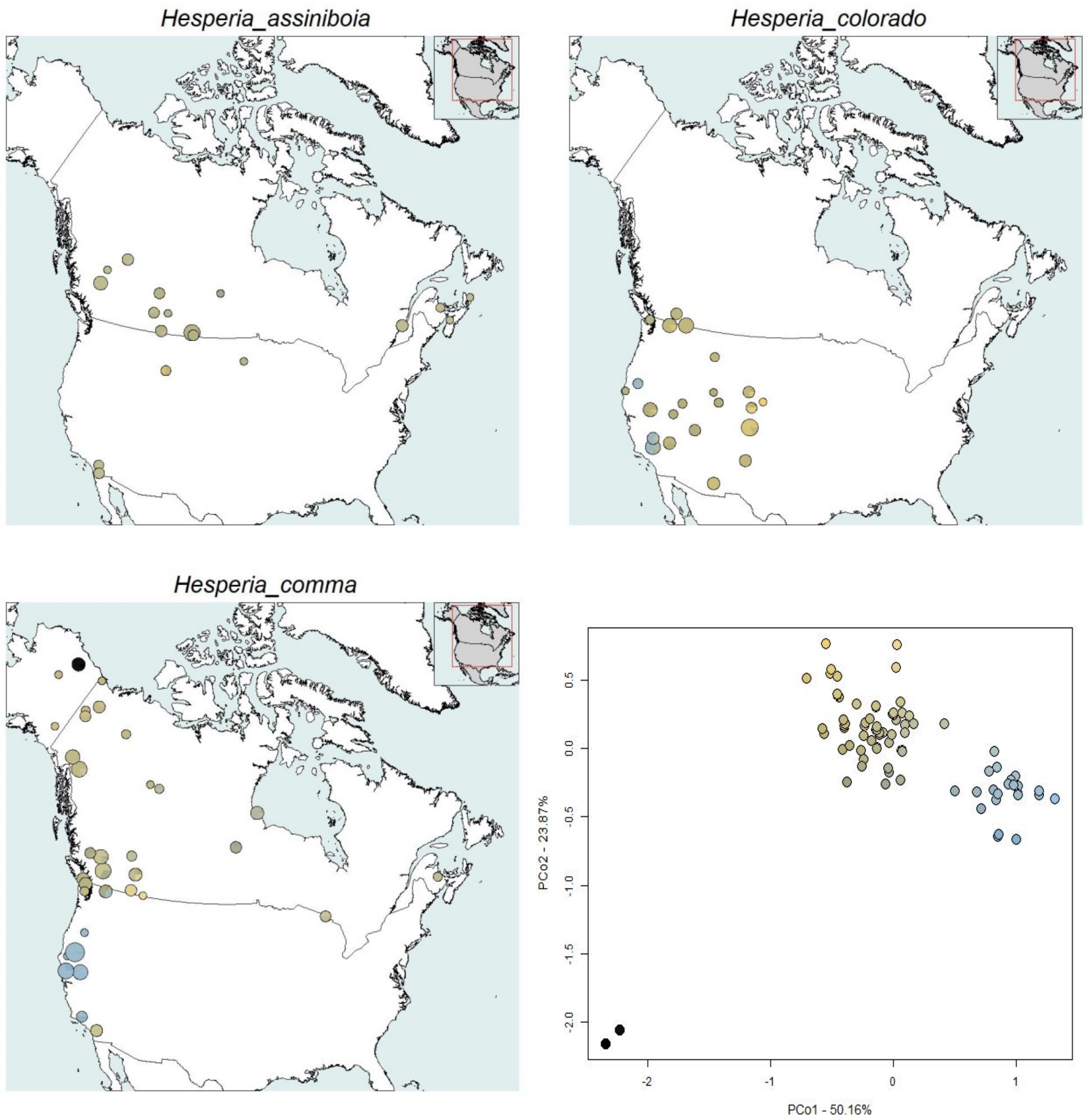

Hesperia_assiniboia
Hesperia_colorado
Hesperia_comma
PCo2 - 23.87%
PCo1 - 50.16%

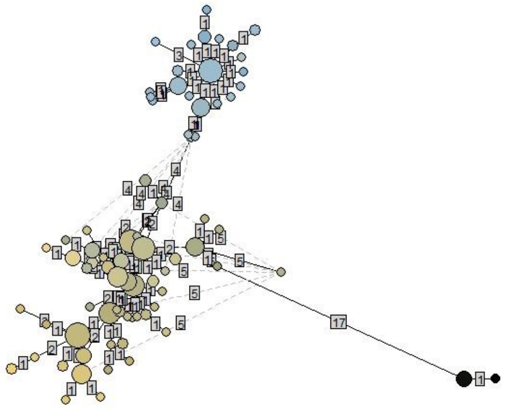

Figure 981: Haplotype maps, PCoA projection, and haplotype network for three *Hesperia* species displaying barcode sharing.

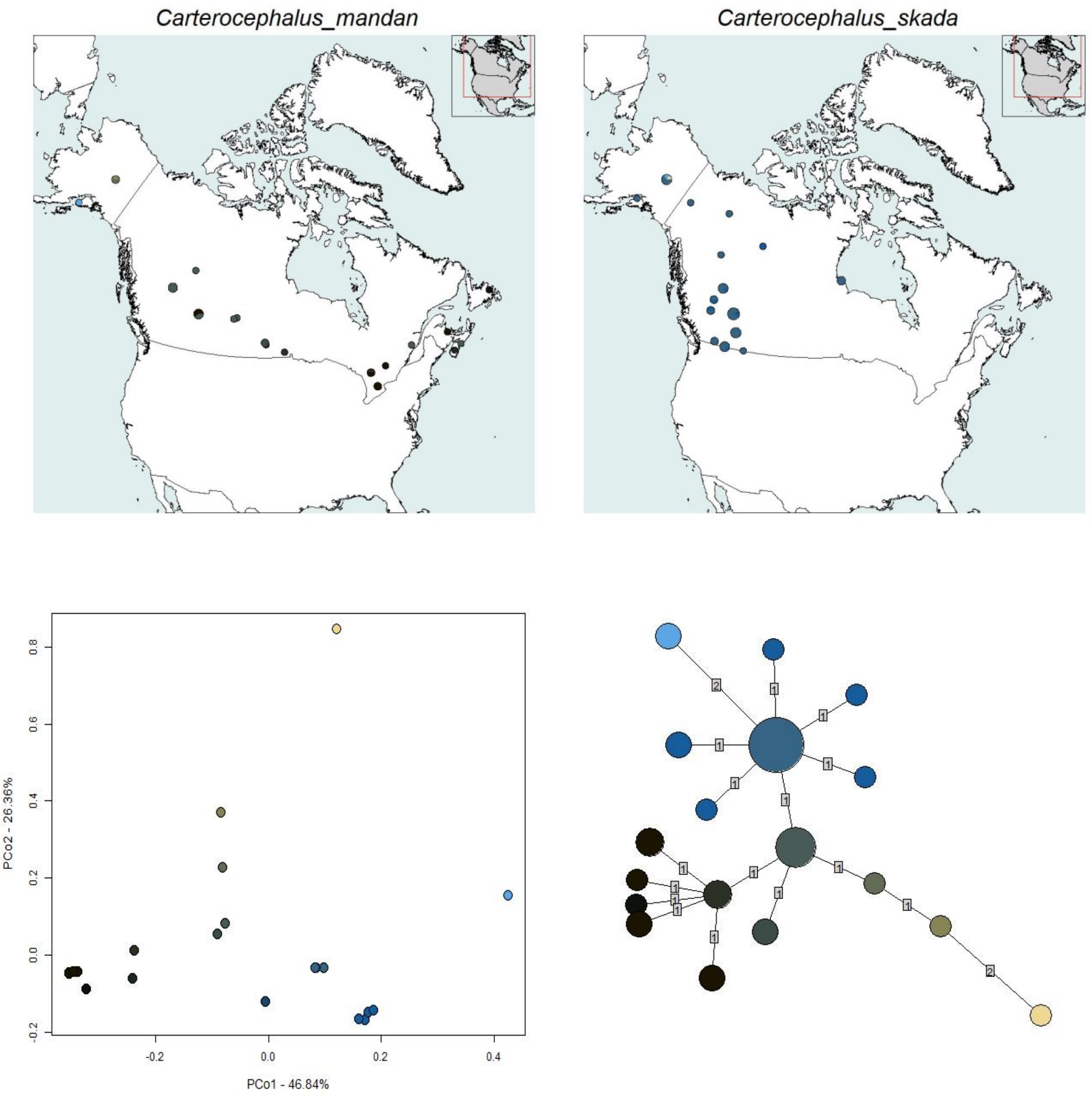

Figure 982: Haplotype maps, PCoA projection, and haplotype network for two *Carterocephalus* species displaying barcode sharing.

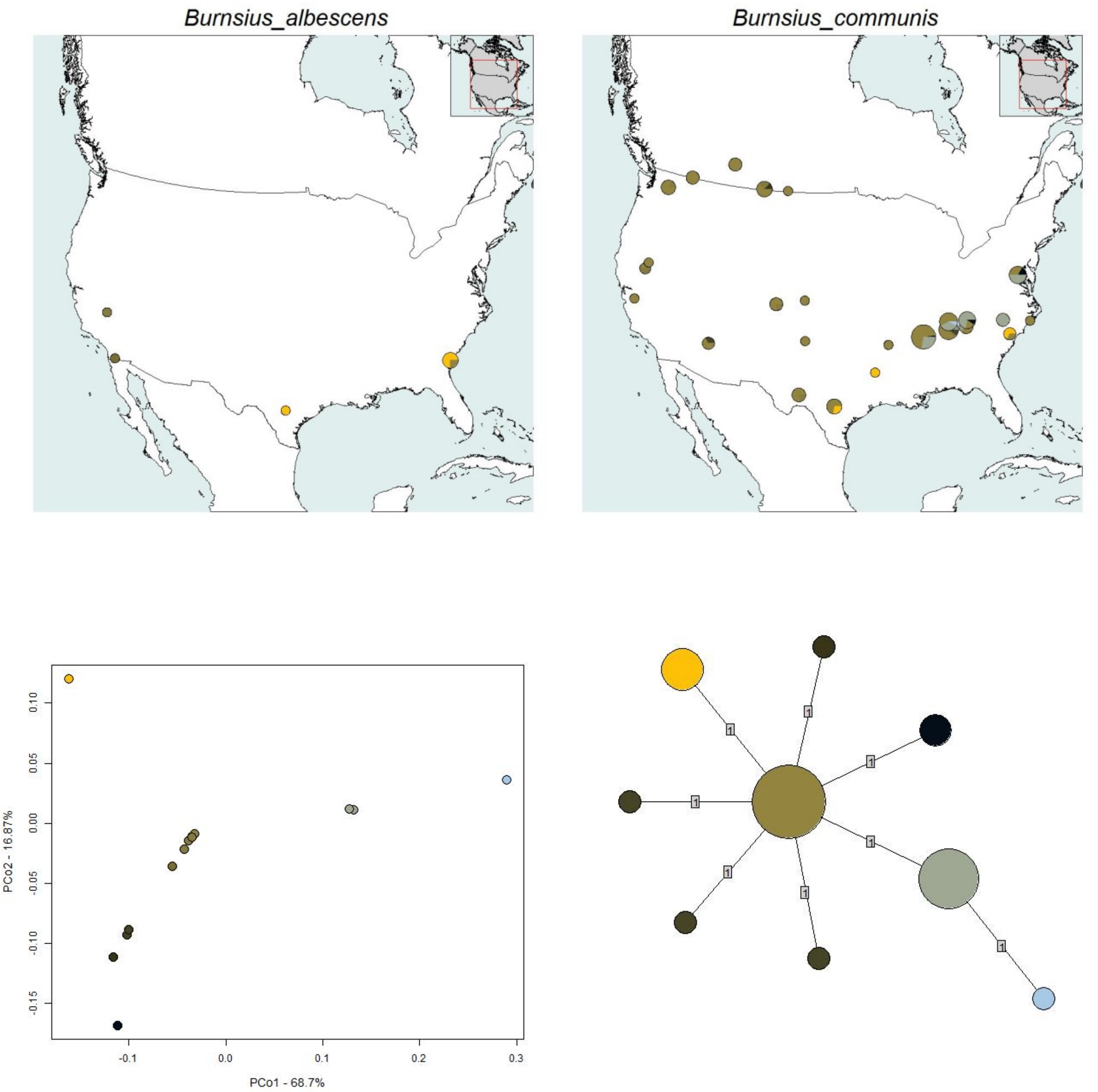

Figure 983: Haplotype maps, PCoA projection, and haplotype network for two *Burnsius* species displaying barcode sharing.

Erynnis_afranius

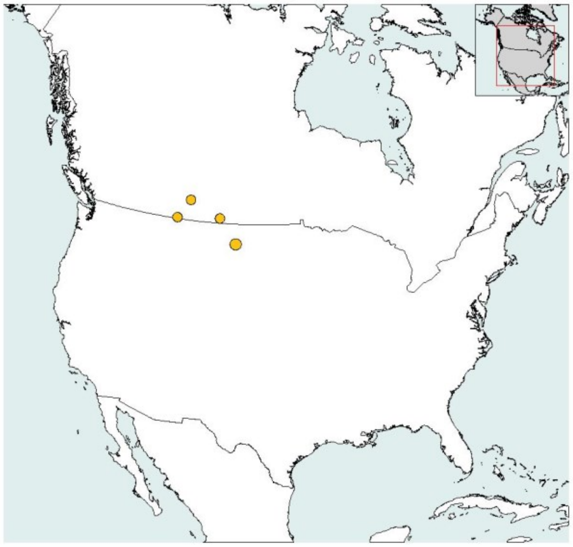

Erynnis_baptisiae

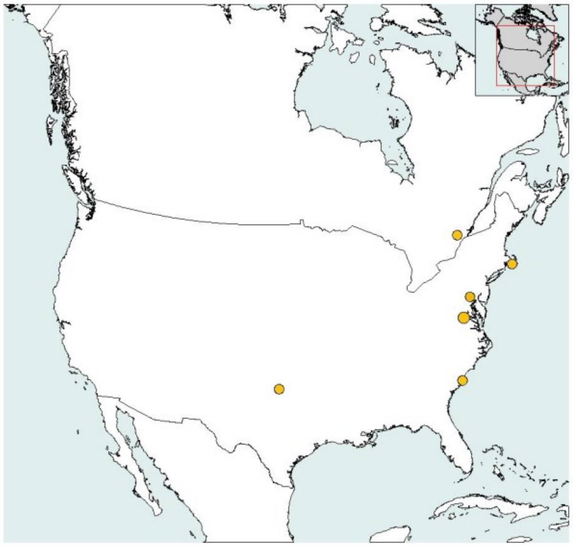

Erynnis_funeralis

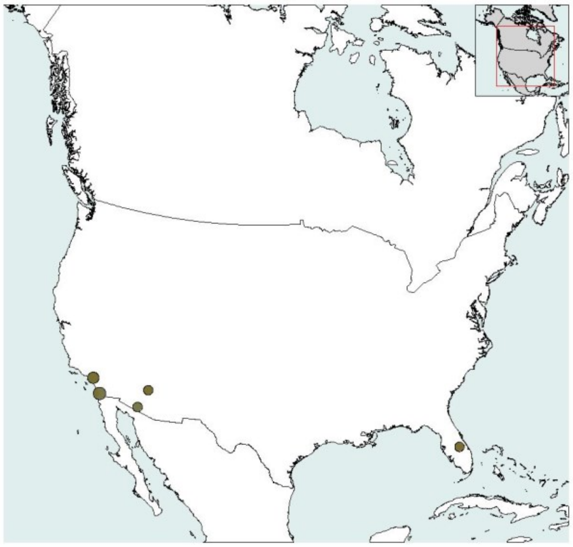

Erynnis_horatius

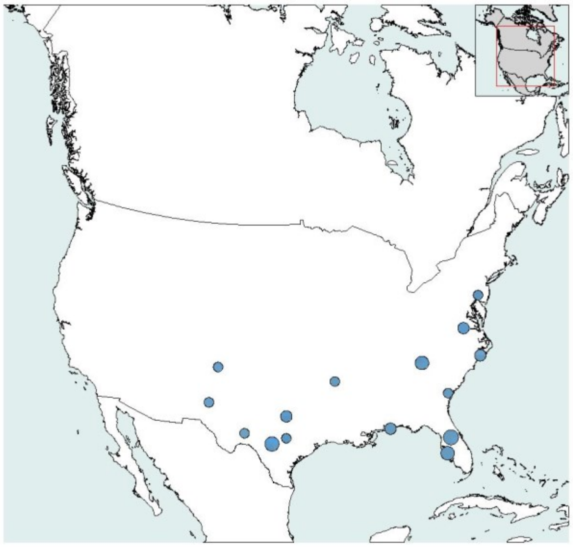

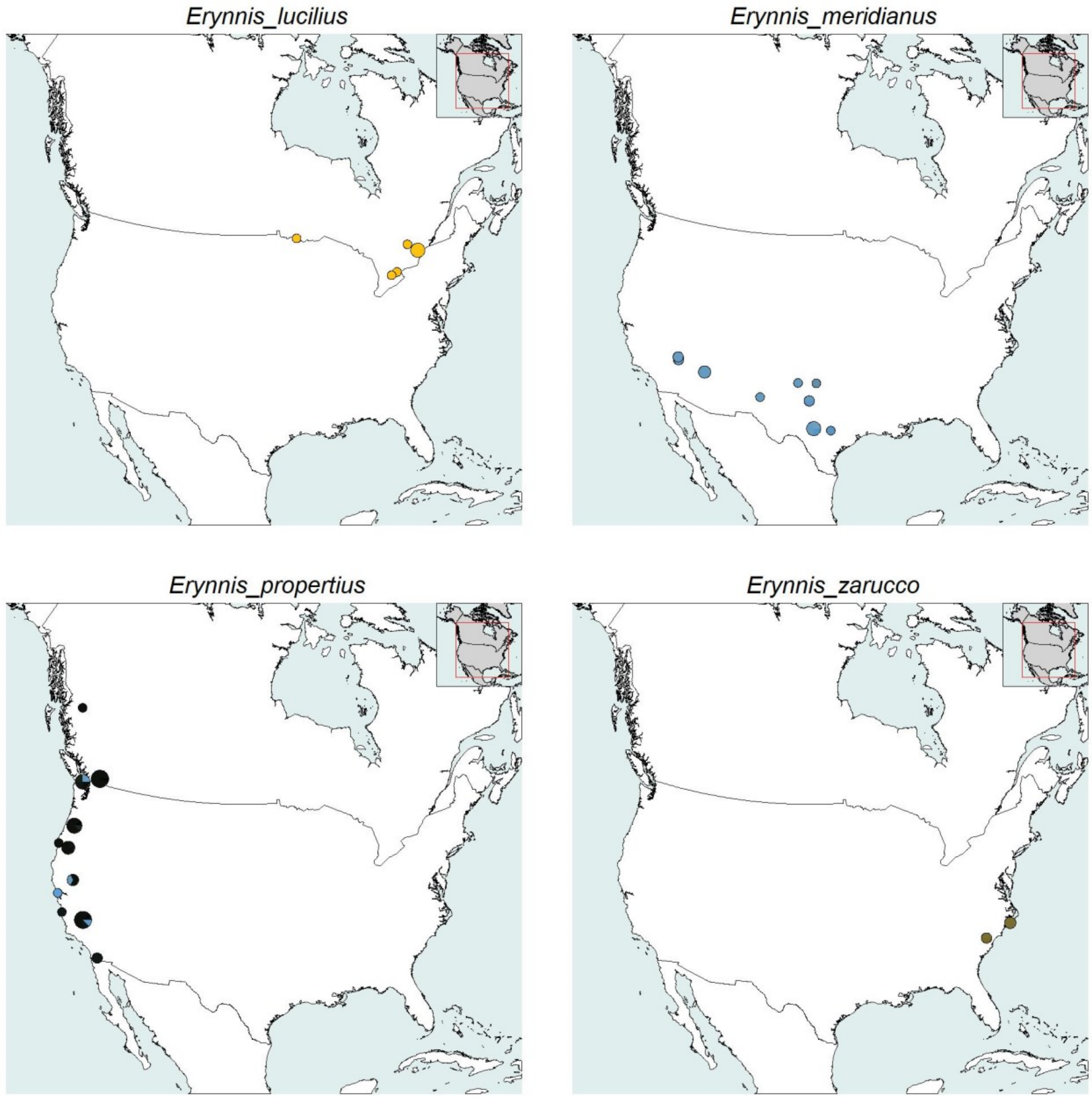
Erynnis_lucilius
Erynnis_meridianus
Erynnis_propertius
Erynnis_zarucco

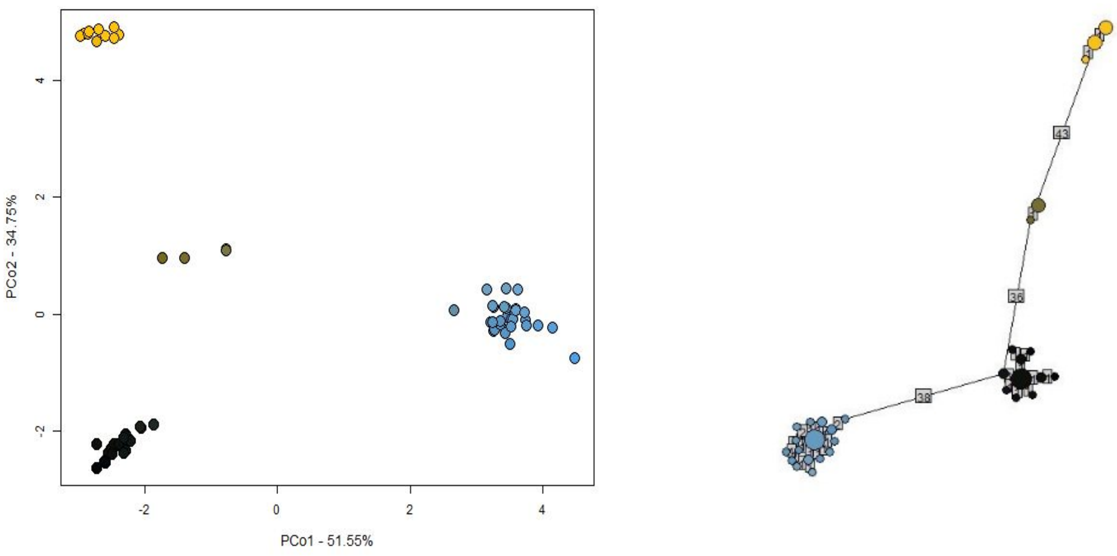

Figure 984: Haplotype maps, PCoA projection, and haplotype network for eight *Erynnis* species displaying barcode sharing.

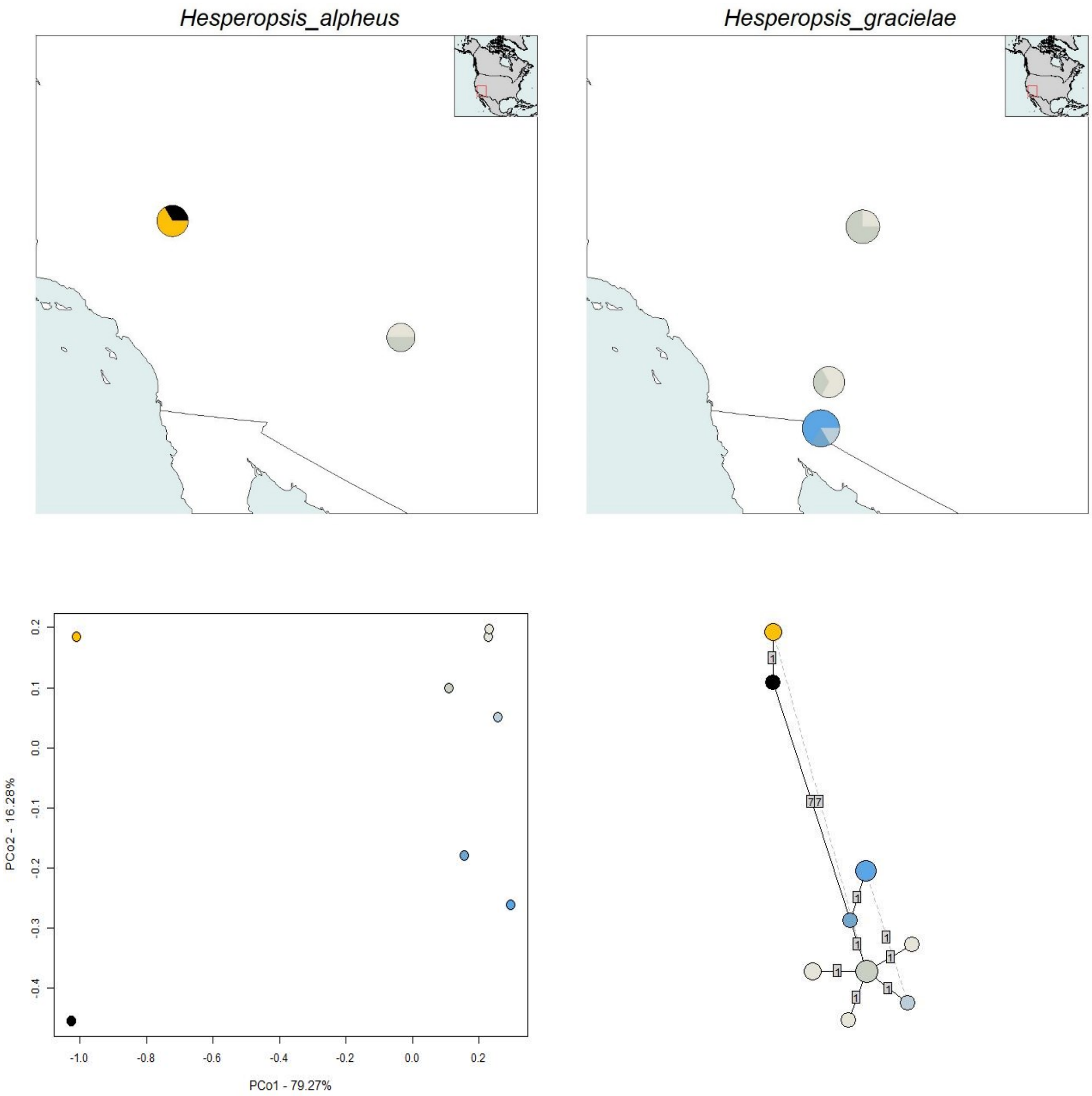

Figure 985: Haplotype maps, PCoA projection, and haplotype network for two *Hesperopsis* species displaying barcode sharing.

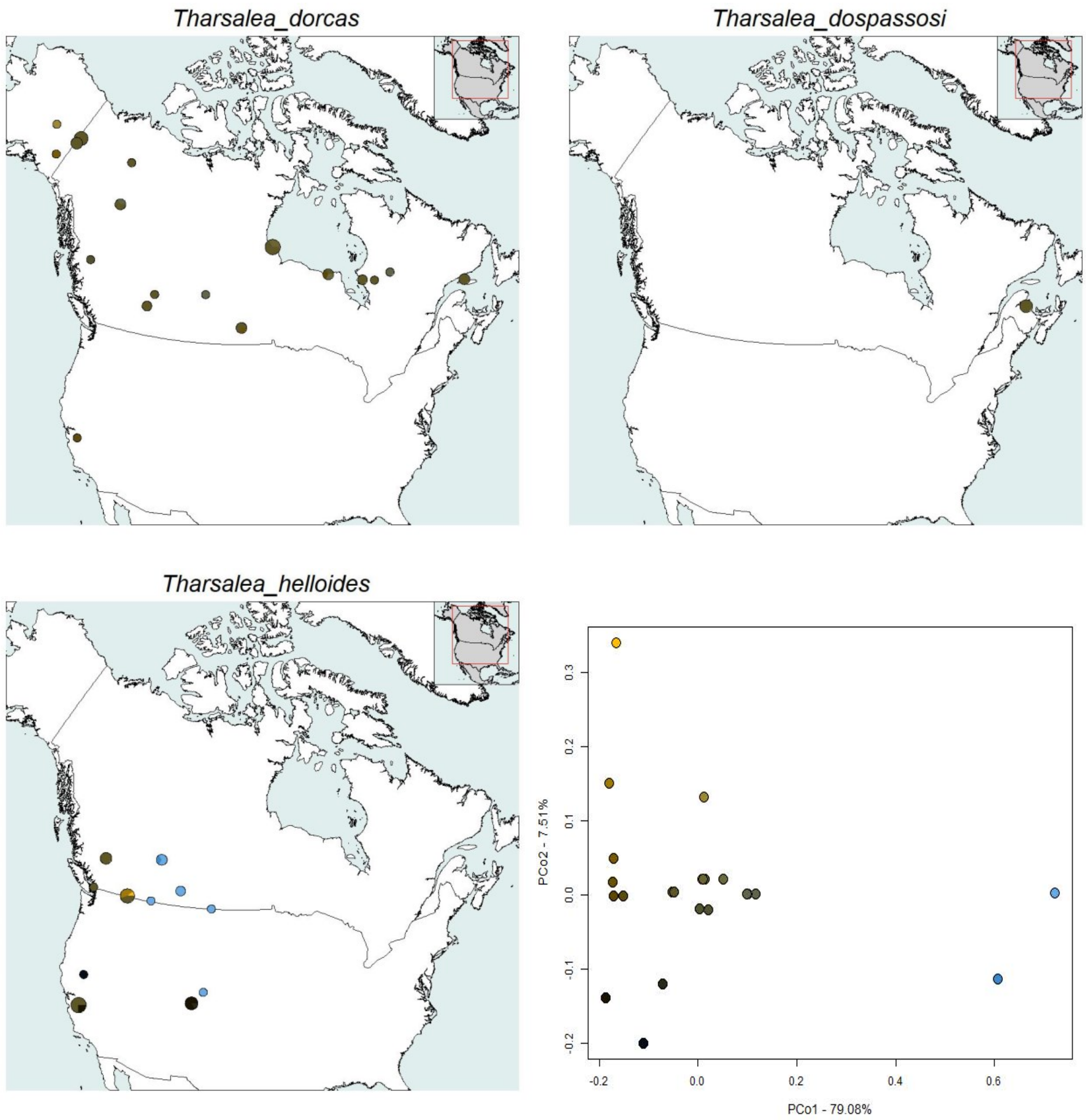
Tharsalea_dorcas
Tharsalea_dospassosi
Tharsalea_helloides
PCo2 - 7.51%
PCo1 - 79.08%
0.3
0.2
0.1
0.0
-0.1
-0.2
-0.2
0.0
0.2
0.4
0.6

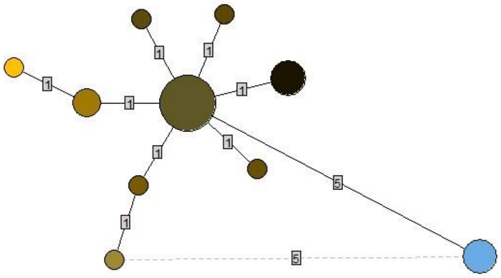

Figure 986: Haplotype maps, PCoA projection, and haplotype network for three *Tharsalea* species displaying barcode sharing.

Celastrina_echo

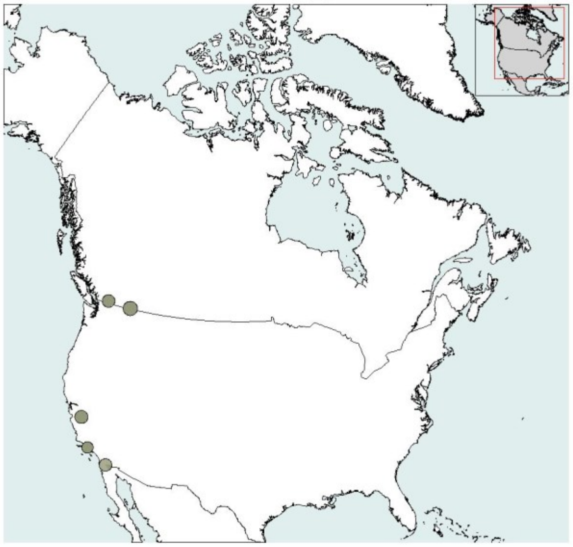

Celastrina_humulus

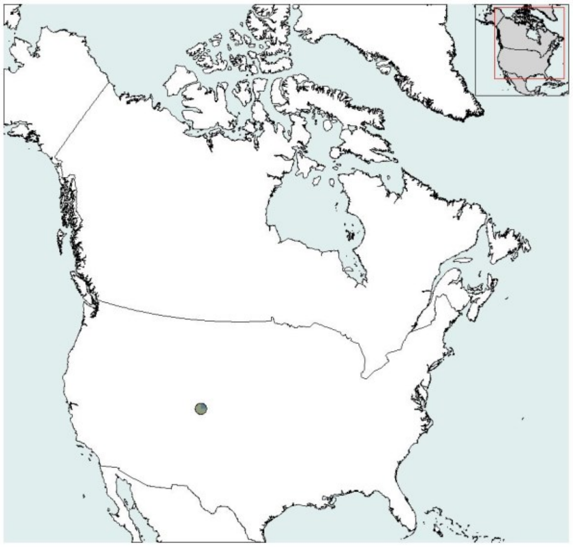

Celastrina_idella

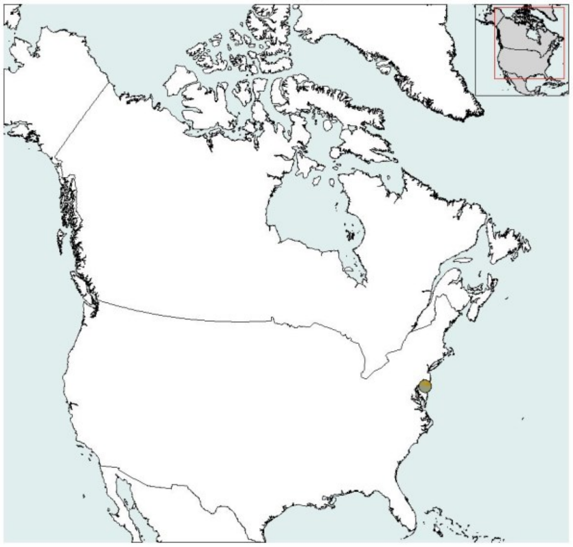

Celastrina_ladon

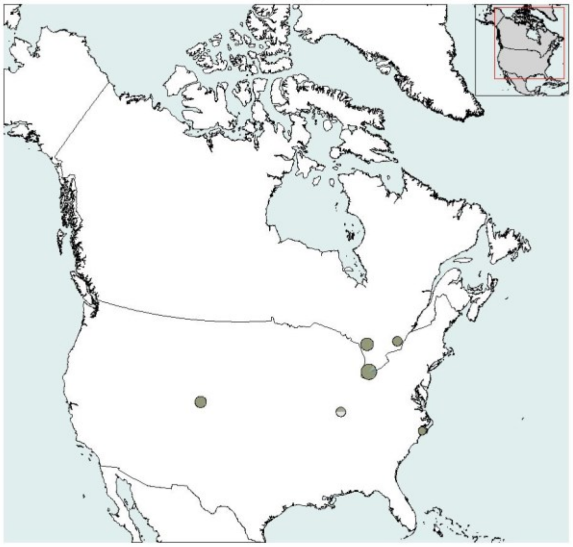

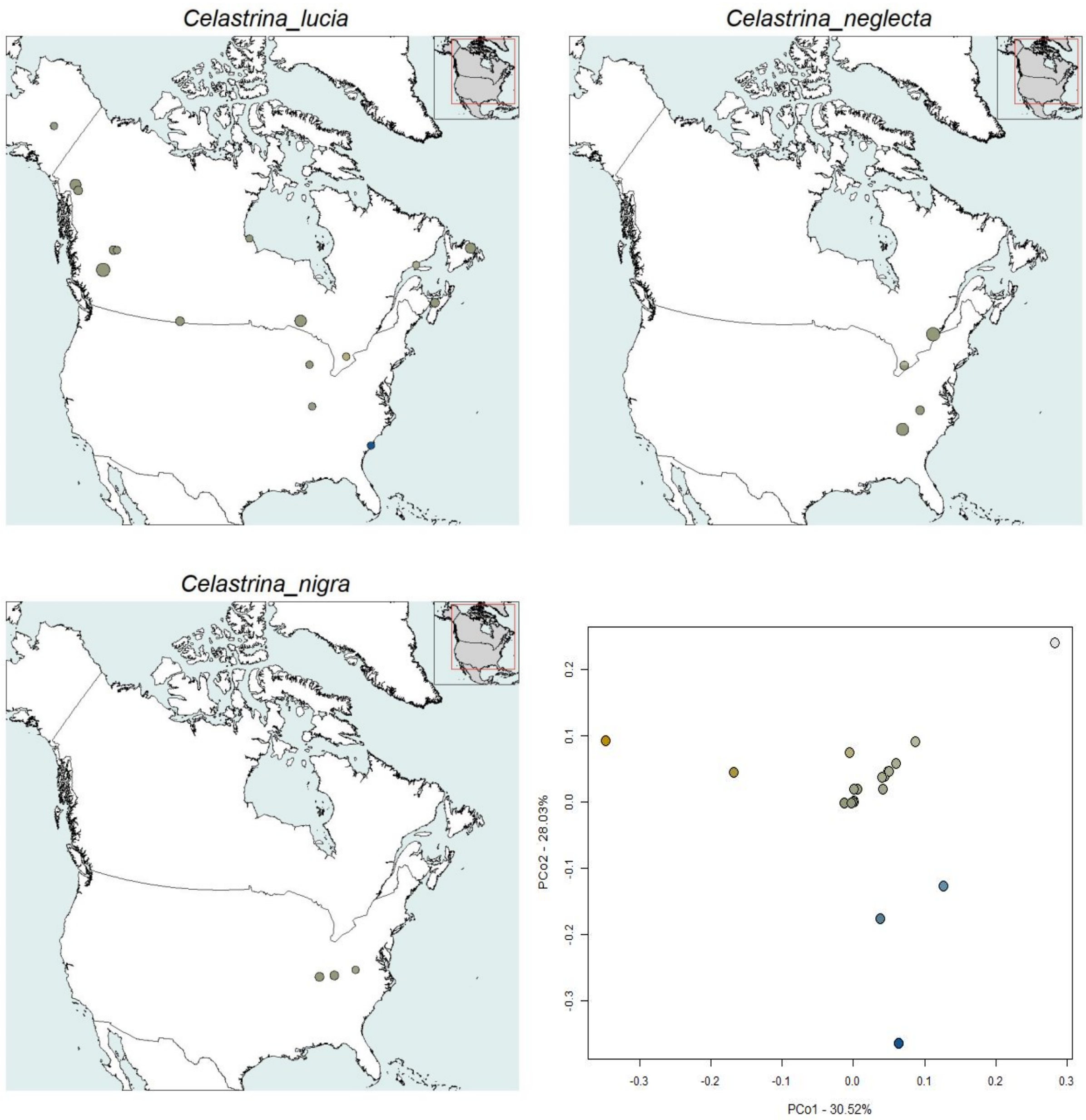
Celastrina_lucia
Celastrina_neglecta
Celastrina_nigra
PCo1 - 30.52%
PCo2 - 28.03%
-0.3
-0.2
-0.1
0.0
0.1
0.2
0.3

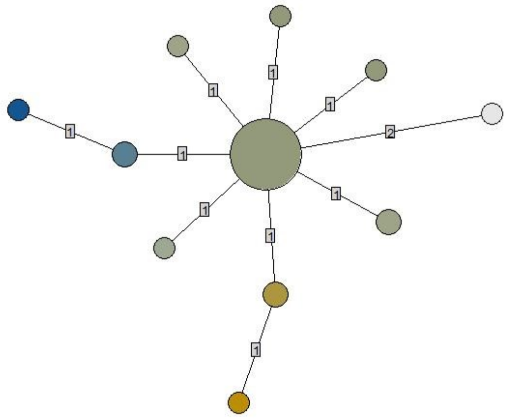

Figure 987: Haplotype maps, PCoA projection, and haplotype network for seven *Celastrina* species displaying barcode sharing.

Euphilotes_ancilla

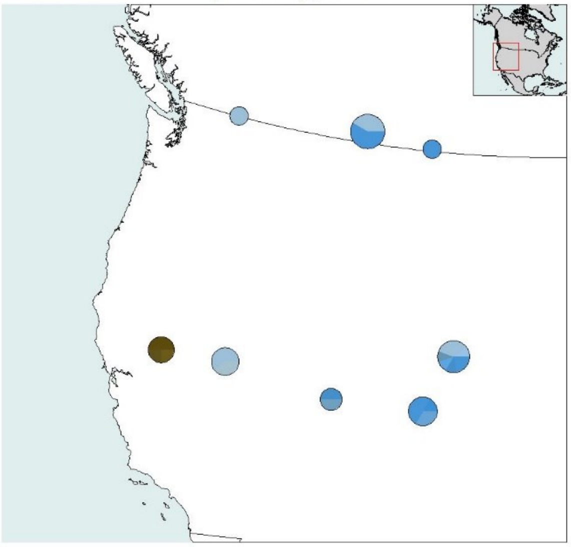

Euphilotes_battoides

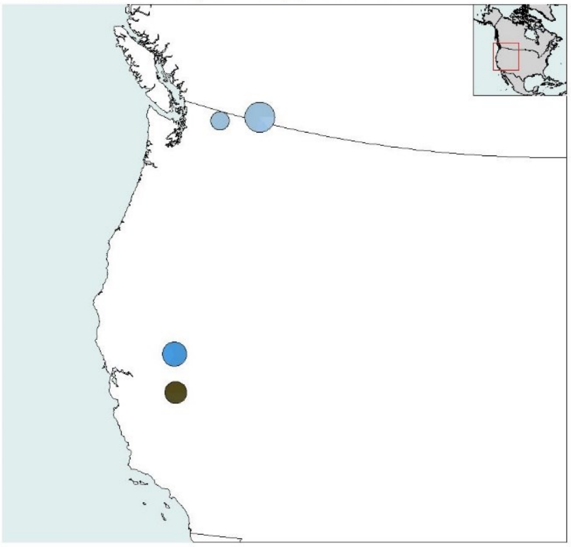

Euphilotes_baueri

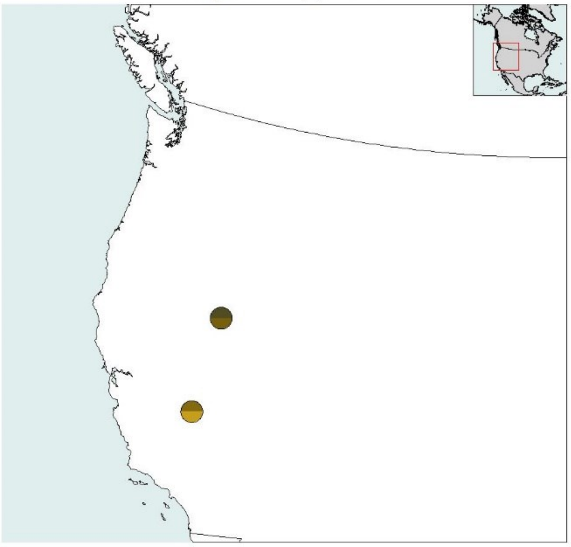

Euphilotes_bernardino

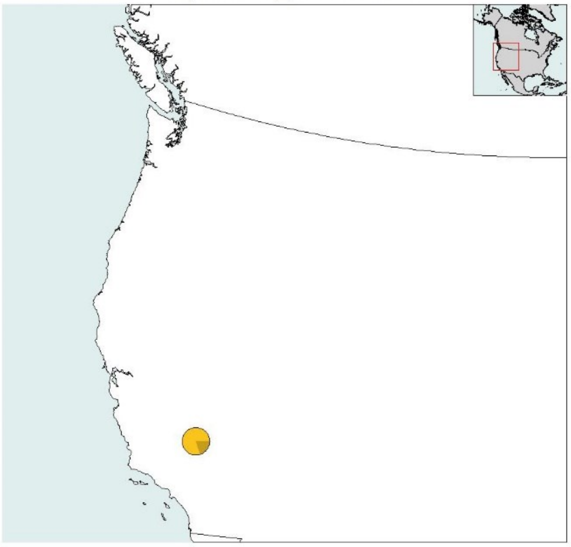

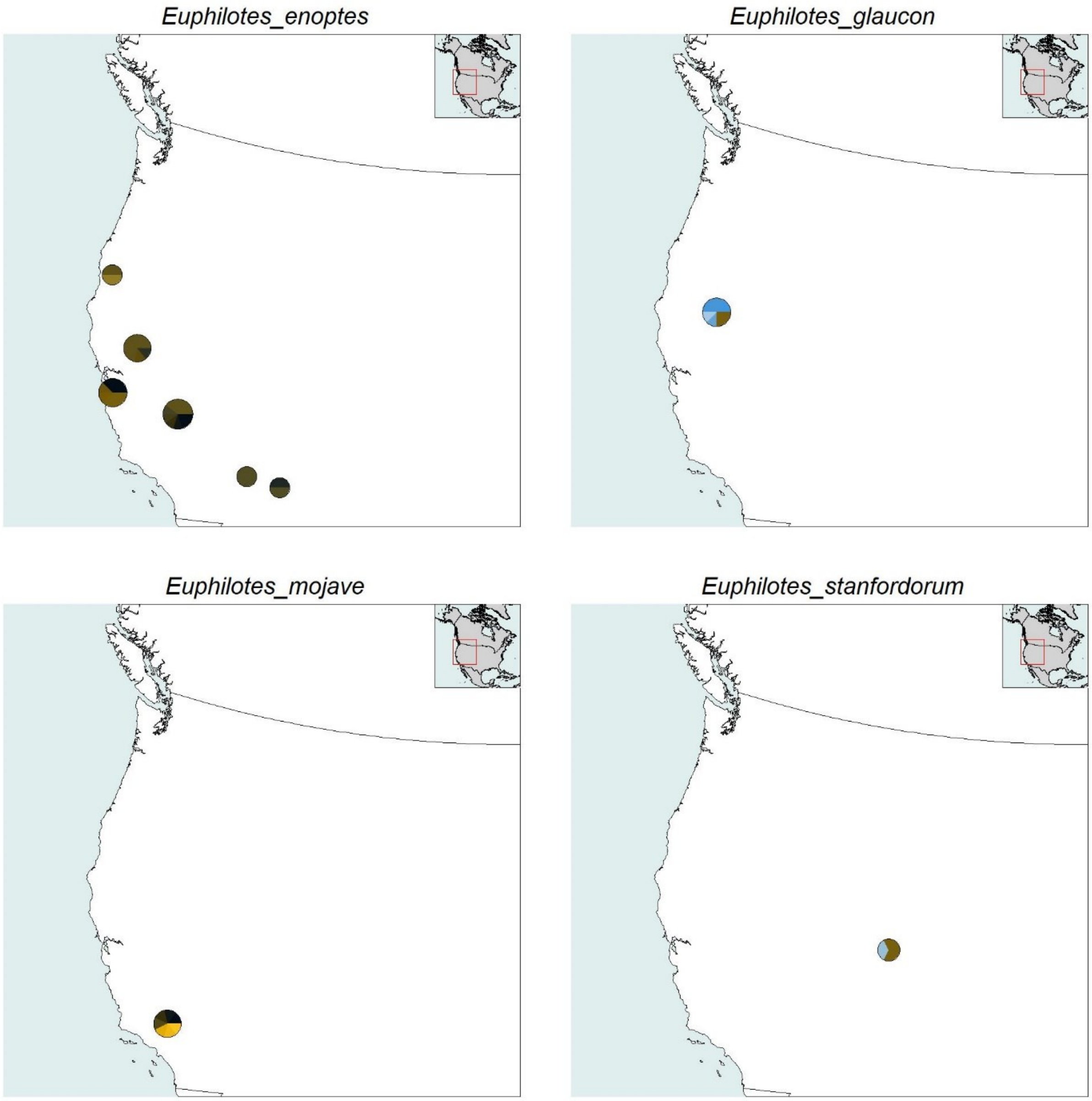
Euphilotes_enoptes
Euphilotes_glaucon
Euphilotes_mojave
Euphilotes_stanfordorum

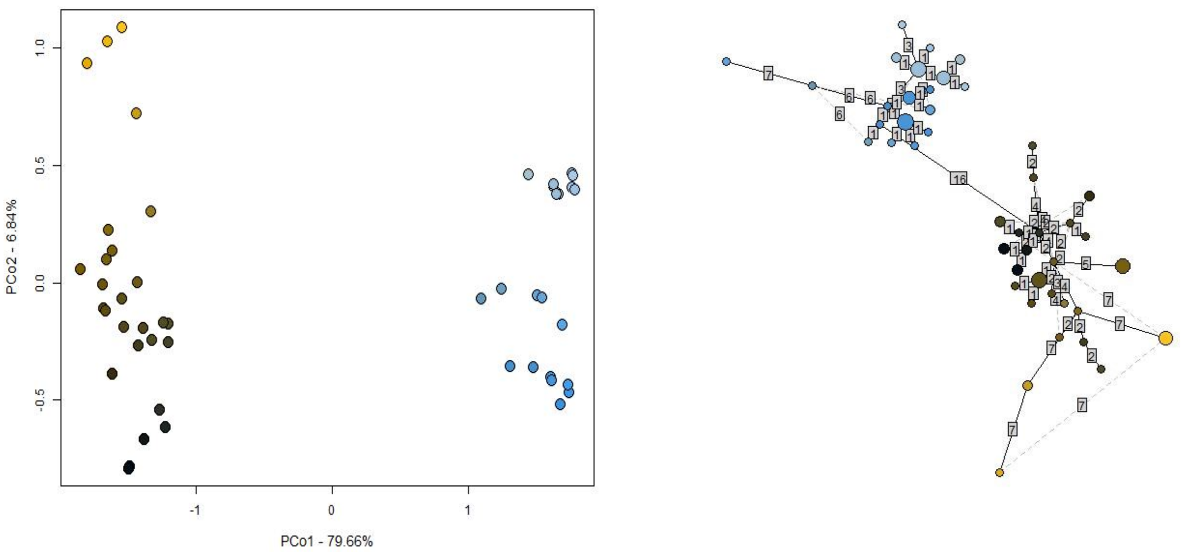

Figure 988: Haplotype maps, PCoA projection, and haplotype network for seven *Euphilotes* species displaying barcode sharing.

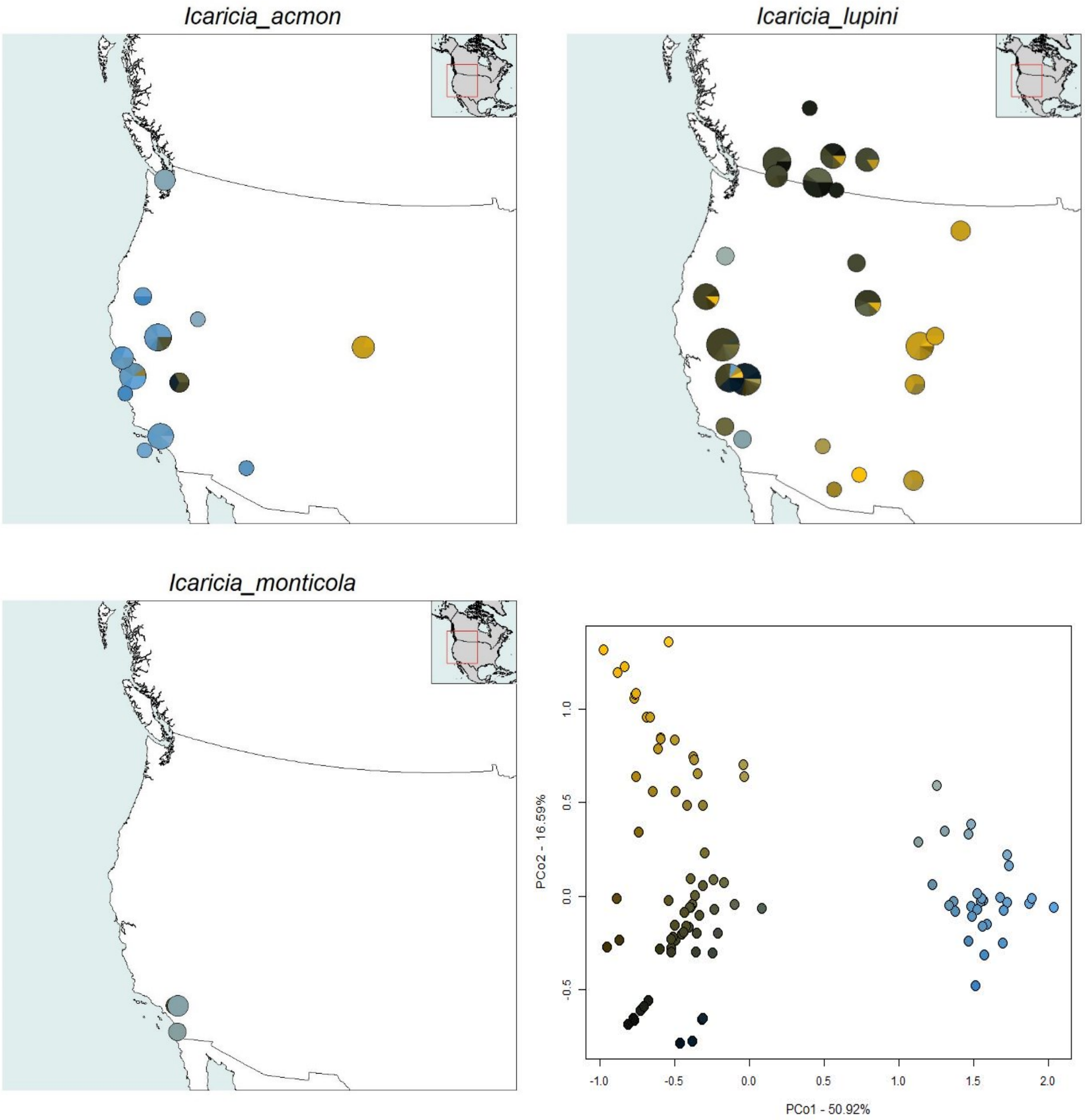
Icaricia_acmon
Icaricia_lupini
Icaricia_monticola
PCo2 - 16.59%
PCo1 - 50.92%
1.0
0.5
0.0
-0.5
-1.0
-0.5
0.0
0.5
1.0
1.5
2.0

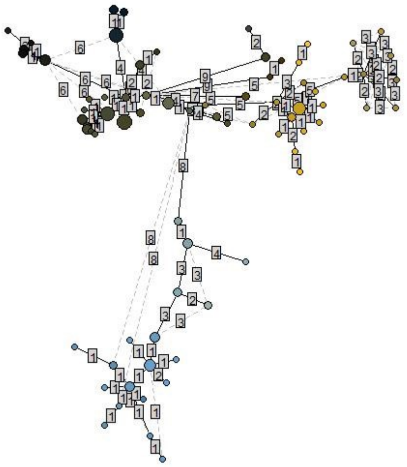

Figure 989: Haplotype maps, PCoA projection, and haplotype network for three *Icaricia* species displaying barcode sharing.

Plebejus_anna

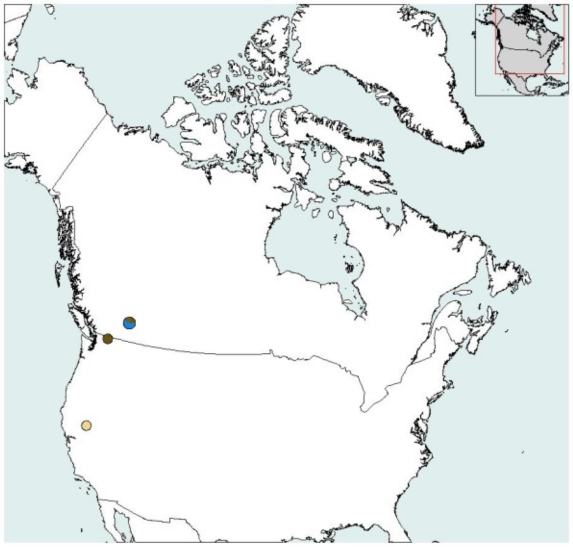

Plebejus_melissa

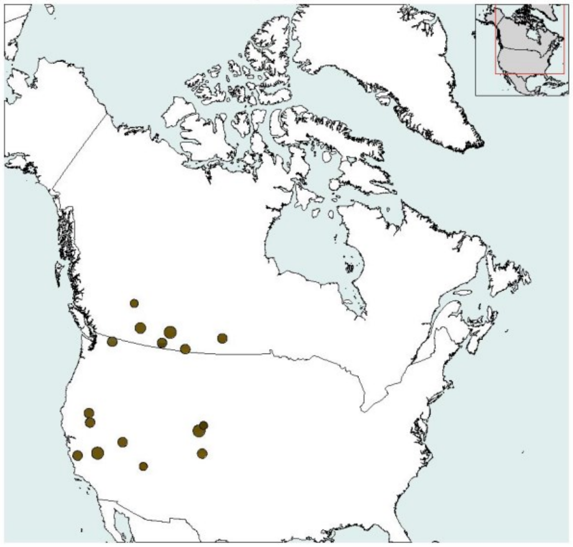

Plebejus_idas

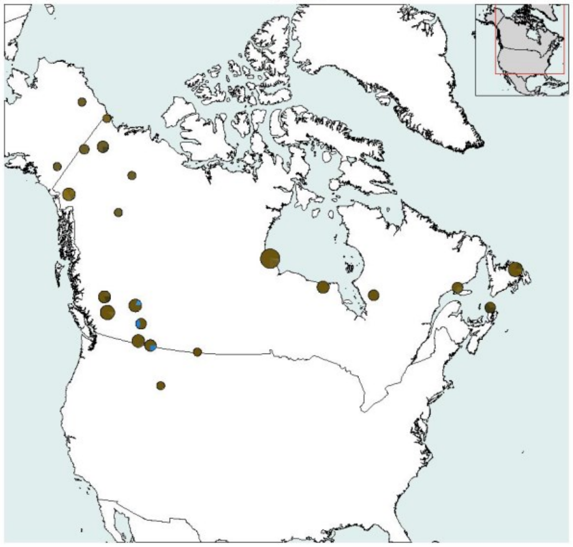

Plebejus_fridayi

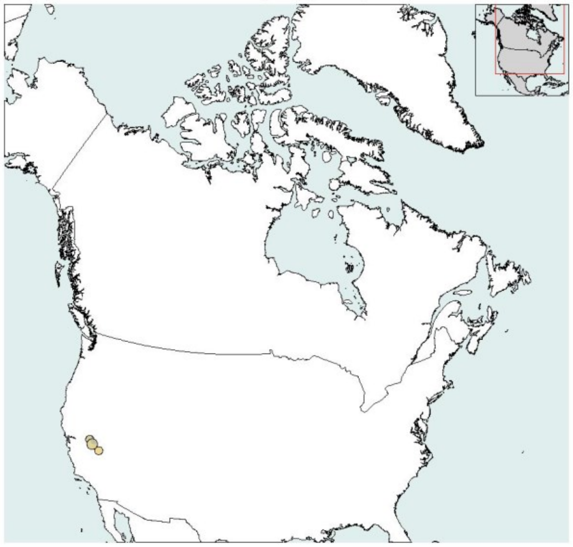

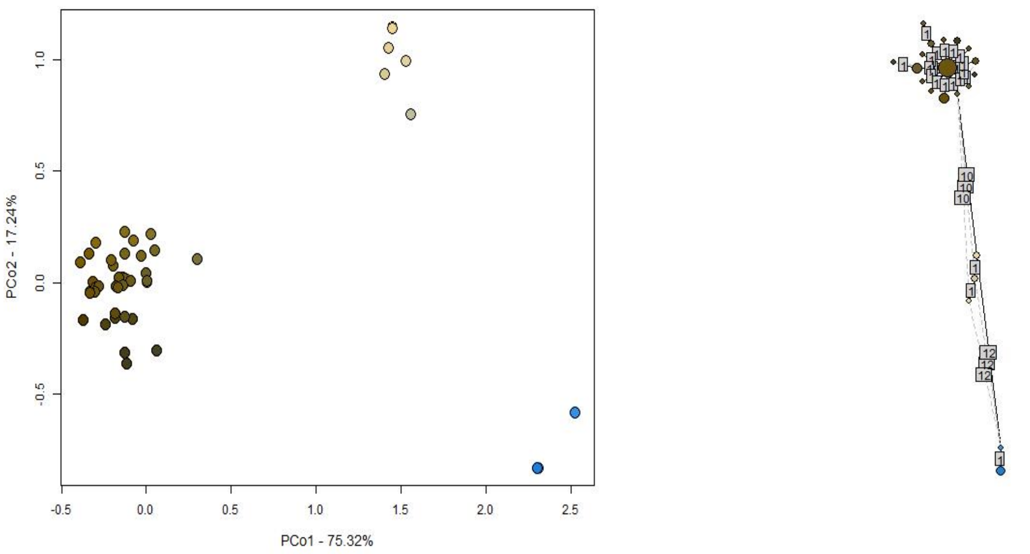

Figure 990: Haplotype maps, PCoA projection, and haplotype network for four *Plebejus* species displaying barcode sharing.

Callophrys_affinis

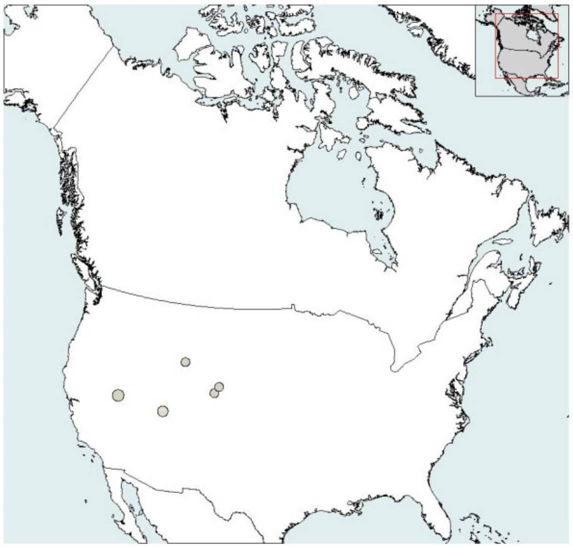

Callophrys_augustinus

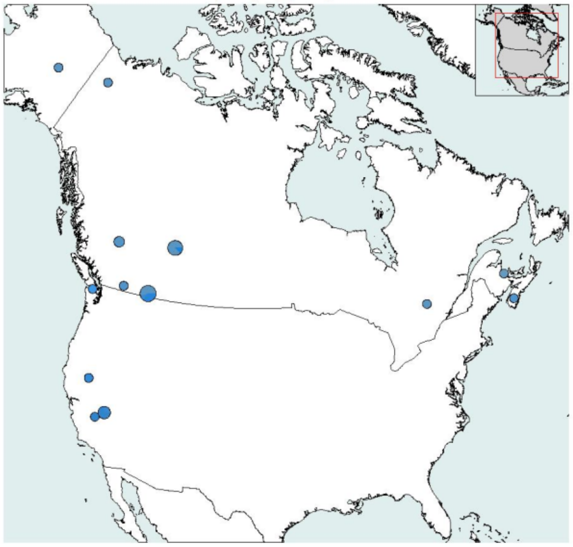

Callophrys_mossii

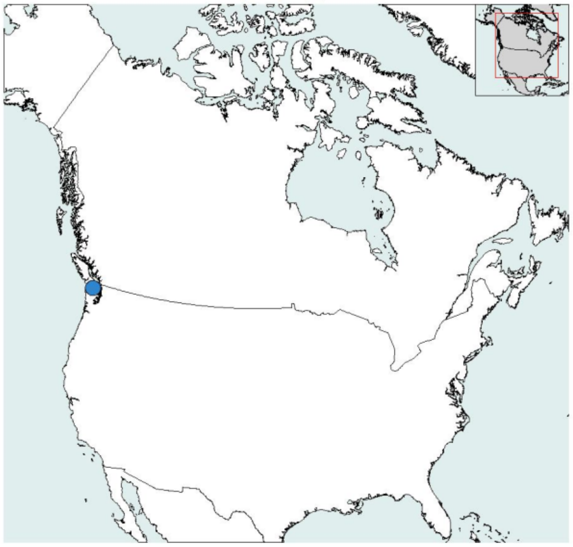

Callophrys_muiri

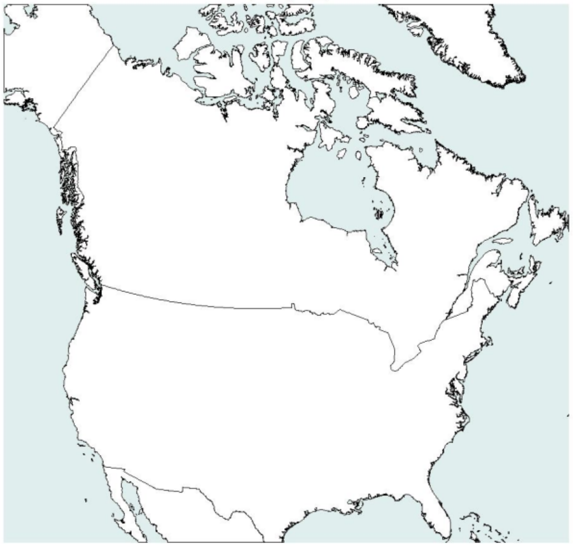

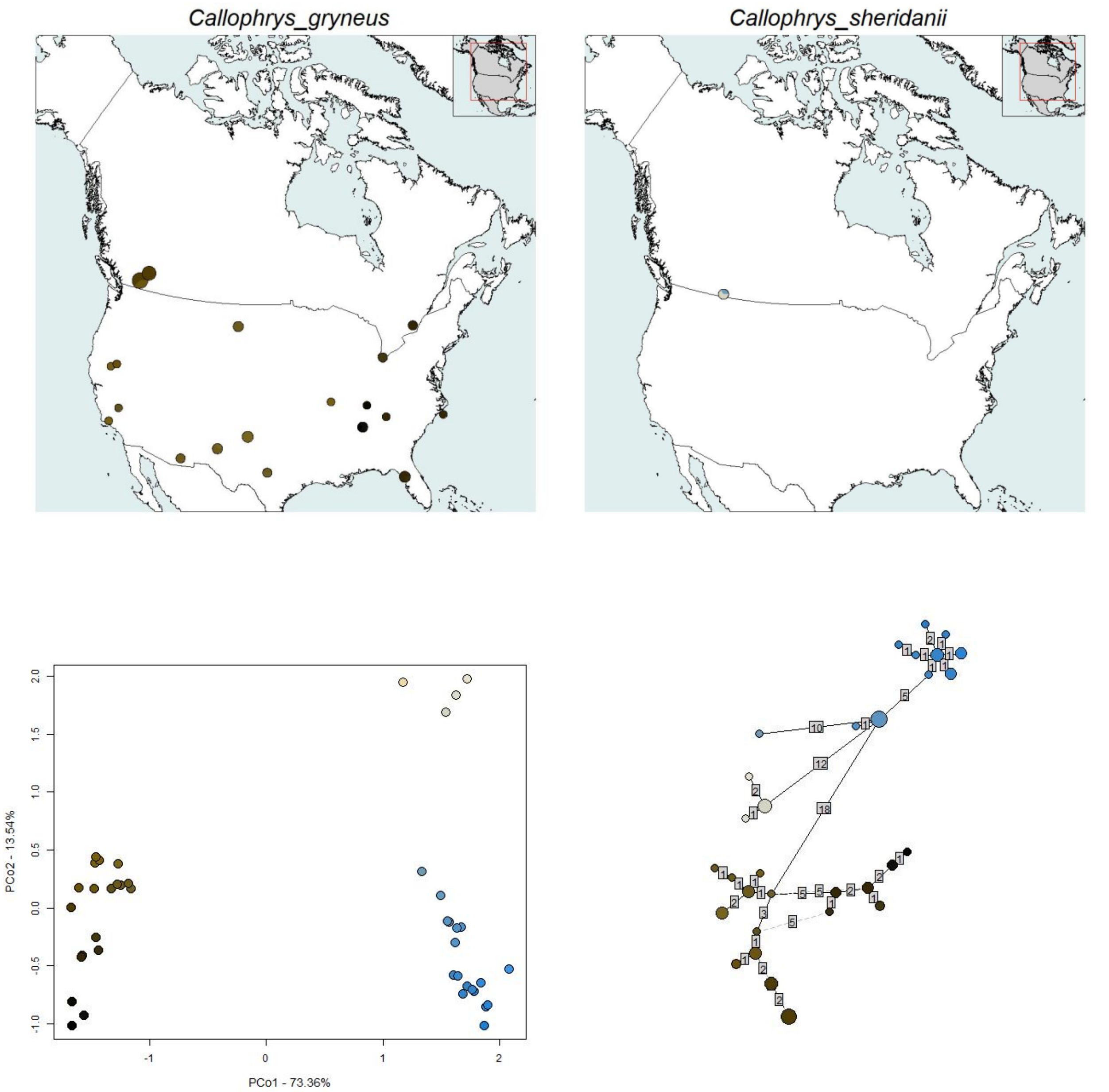

Figure 991: Haplotype maps, PCoA projection, and haplotype network for six *Callophrys* species displaying barcode sharing.

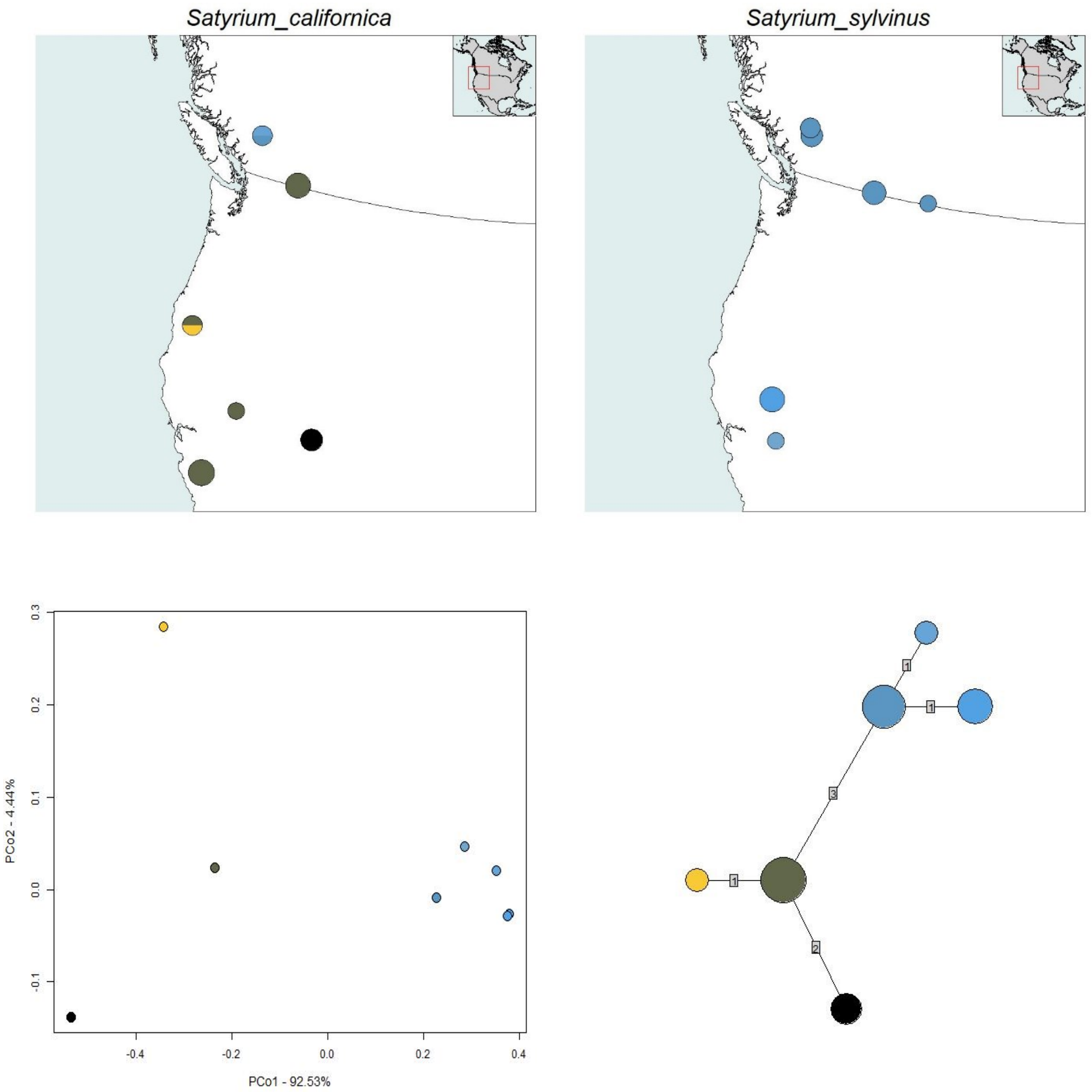

Figure 992: Haplotype maps, PCoA projection, and haplotype network for two *Satyrium* species displaying barcode sharing.

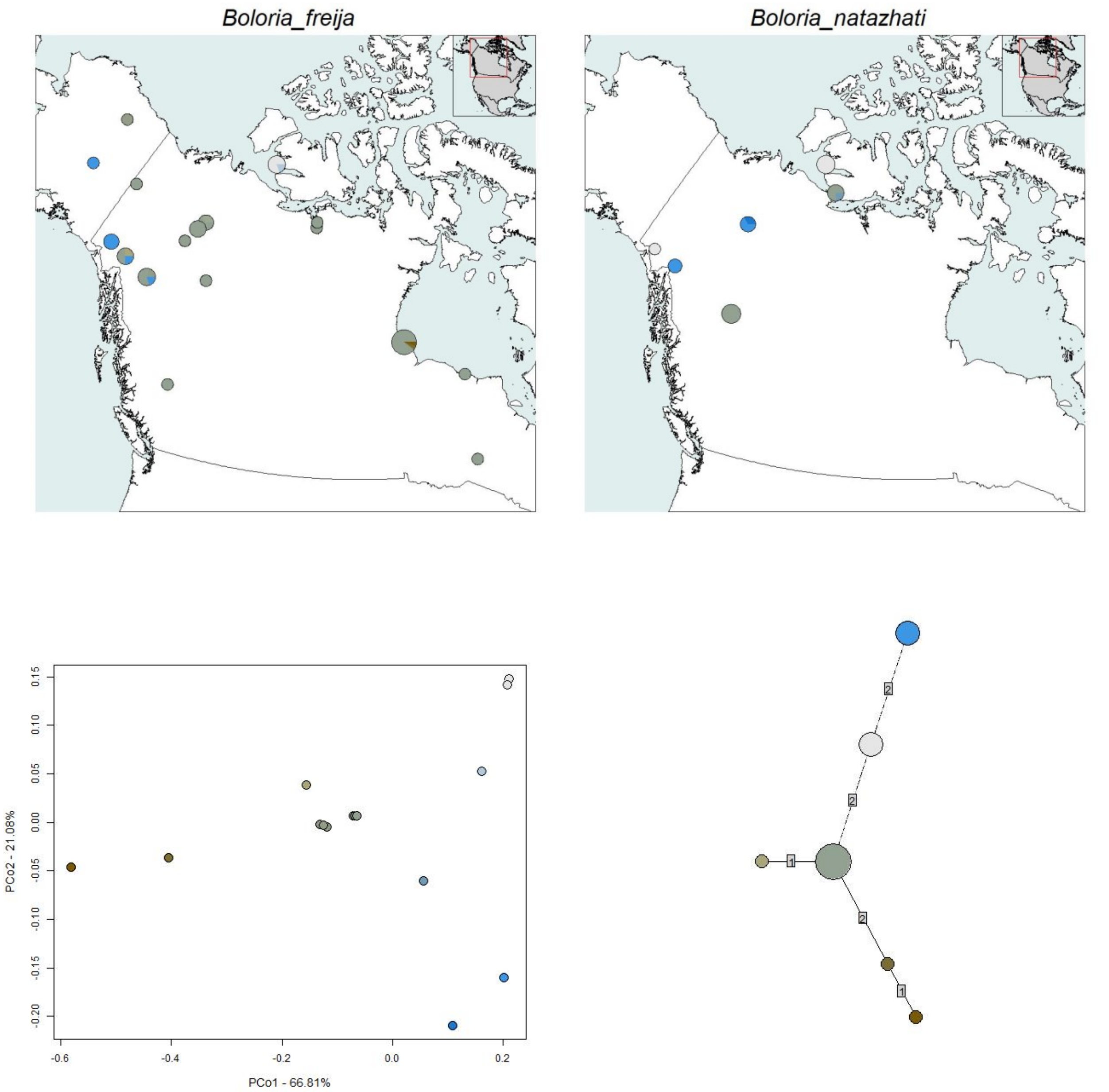

Figure 993: Haplotype maps, PCoA projection, and haplotype network for seven *Boloria* species displaying barcode sharing.

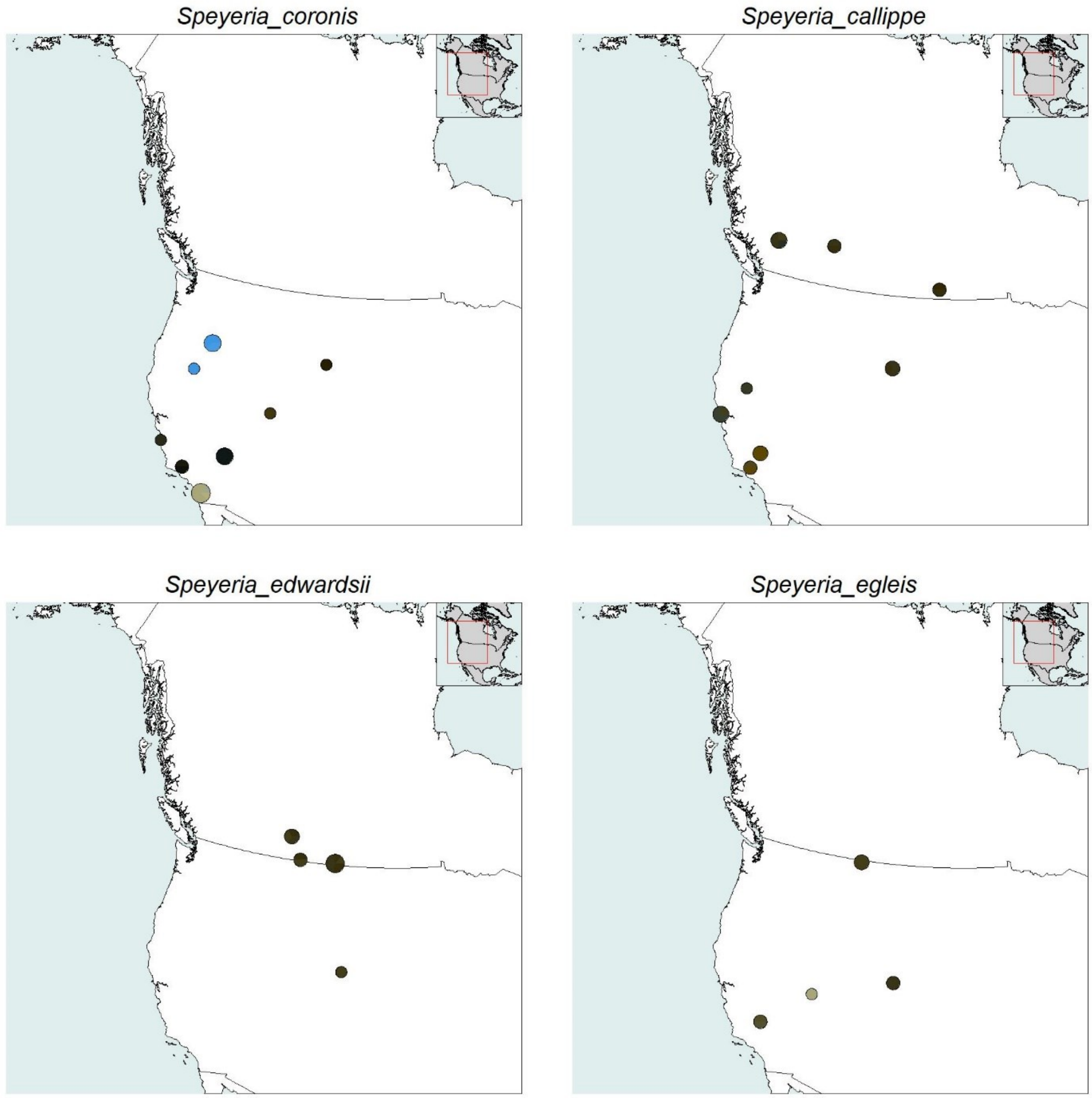
Speyeria_coronis
Speyeria_callippe
Speyeria_edwardsii
Speyeria_egleis

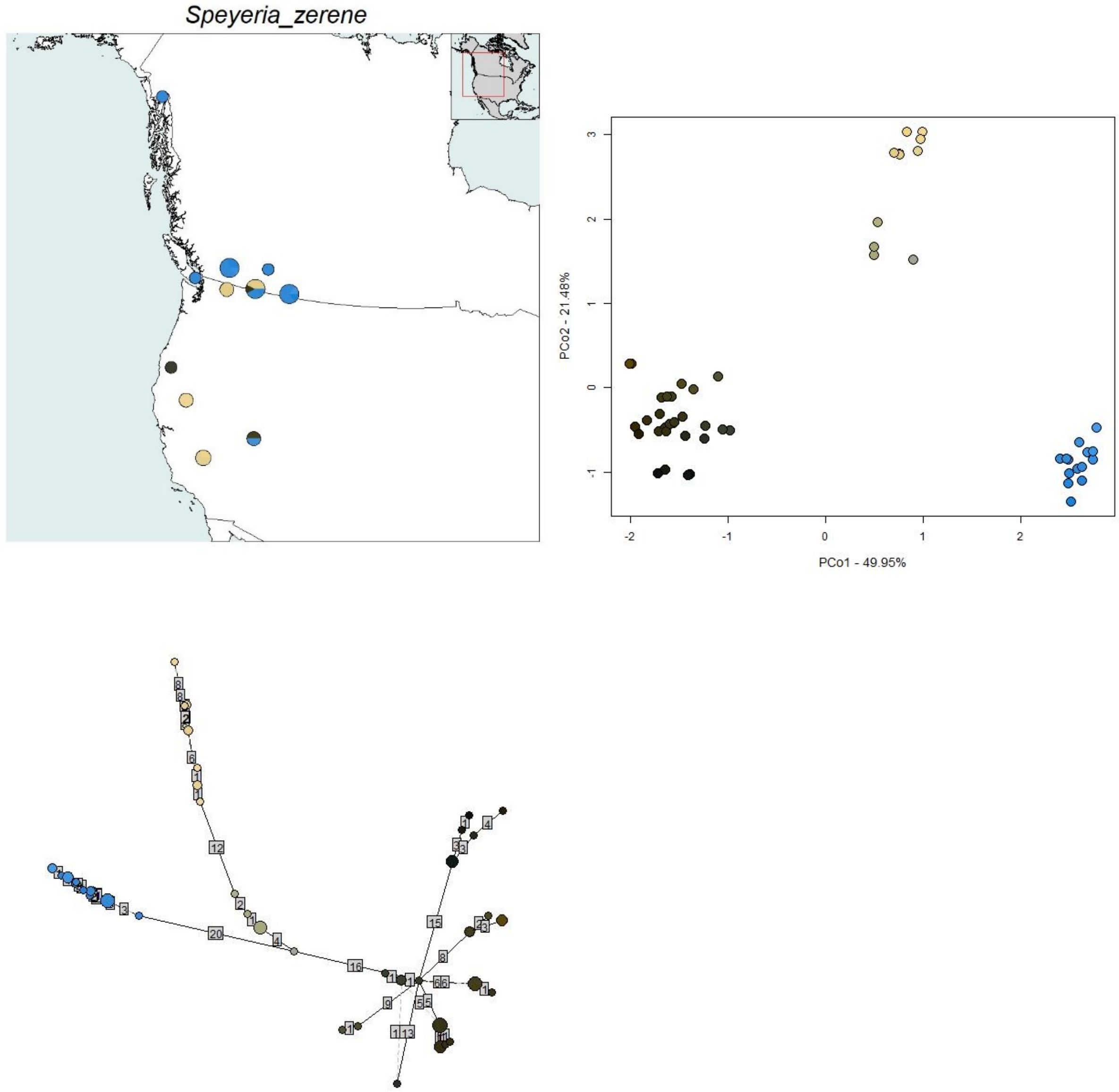

Figure 994: Haplotype maps, PCoA projection, and haplotype network for five *Speyeria* species displaying barcode sharing.

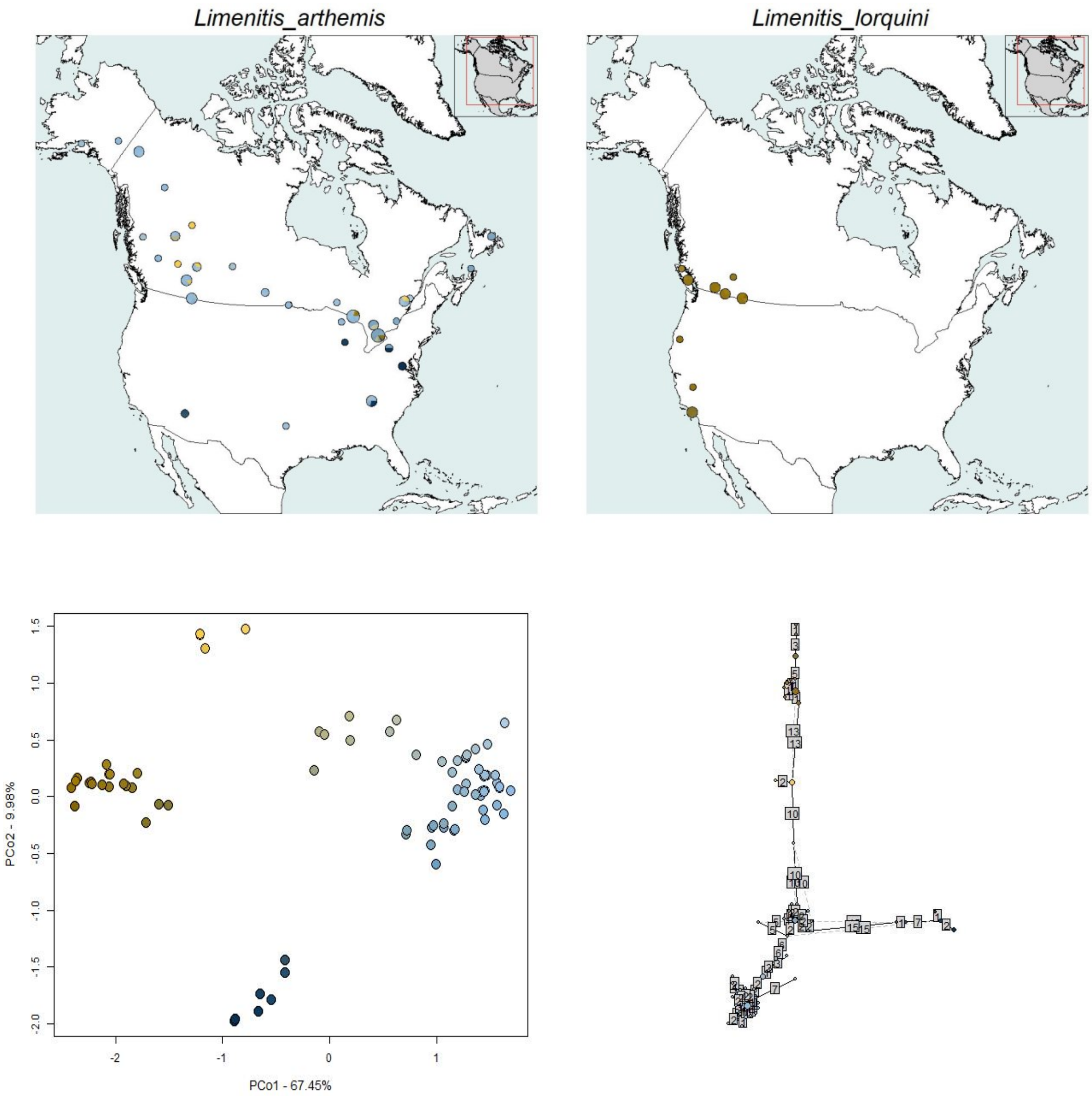

Figure 995: Haplotype maps, PCoA projection, and haplotype network for two *Limenitis* species displaying barcode sharing.

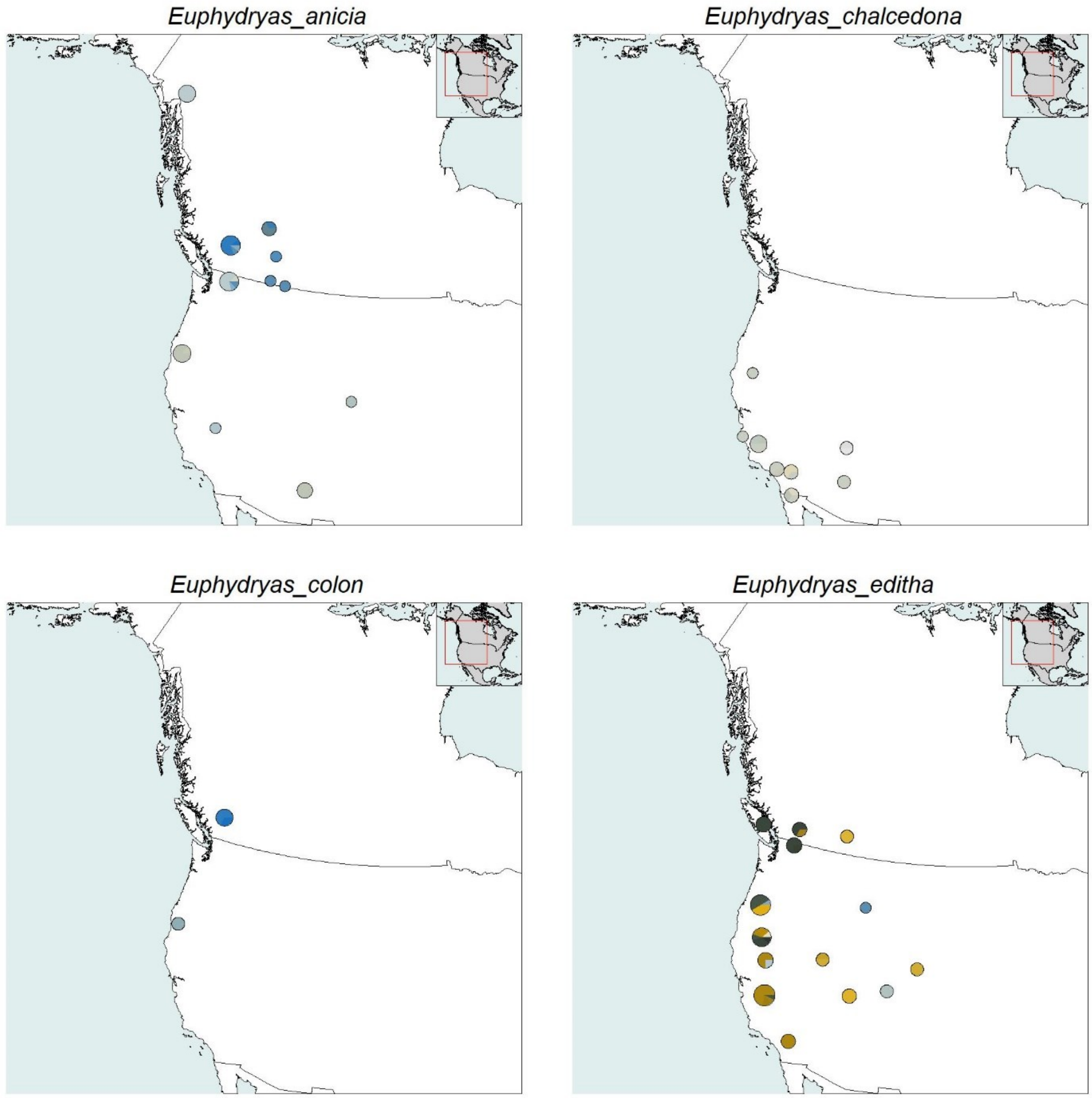
Euphydryas_anicia
Euphydryas_chalcedona
Euphydryas_colon
Euphydryas_editha

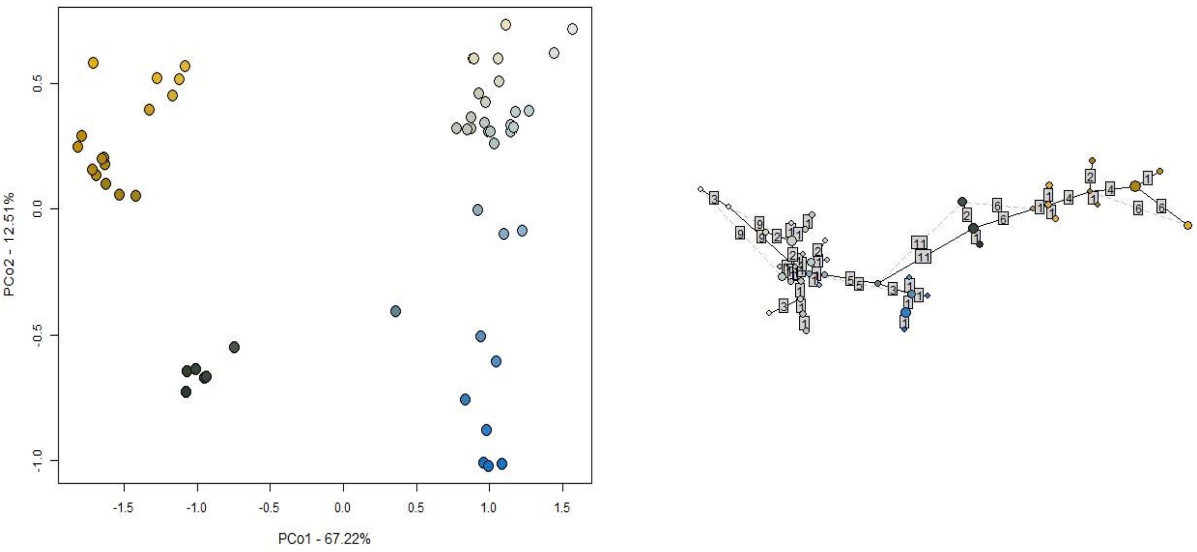

Figure 996: Haplotype maps, PCoA projection, and haplotype network for four *Euphydryas* species displaying barcode sharing.

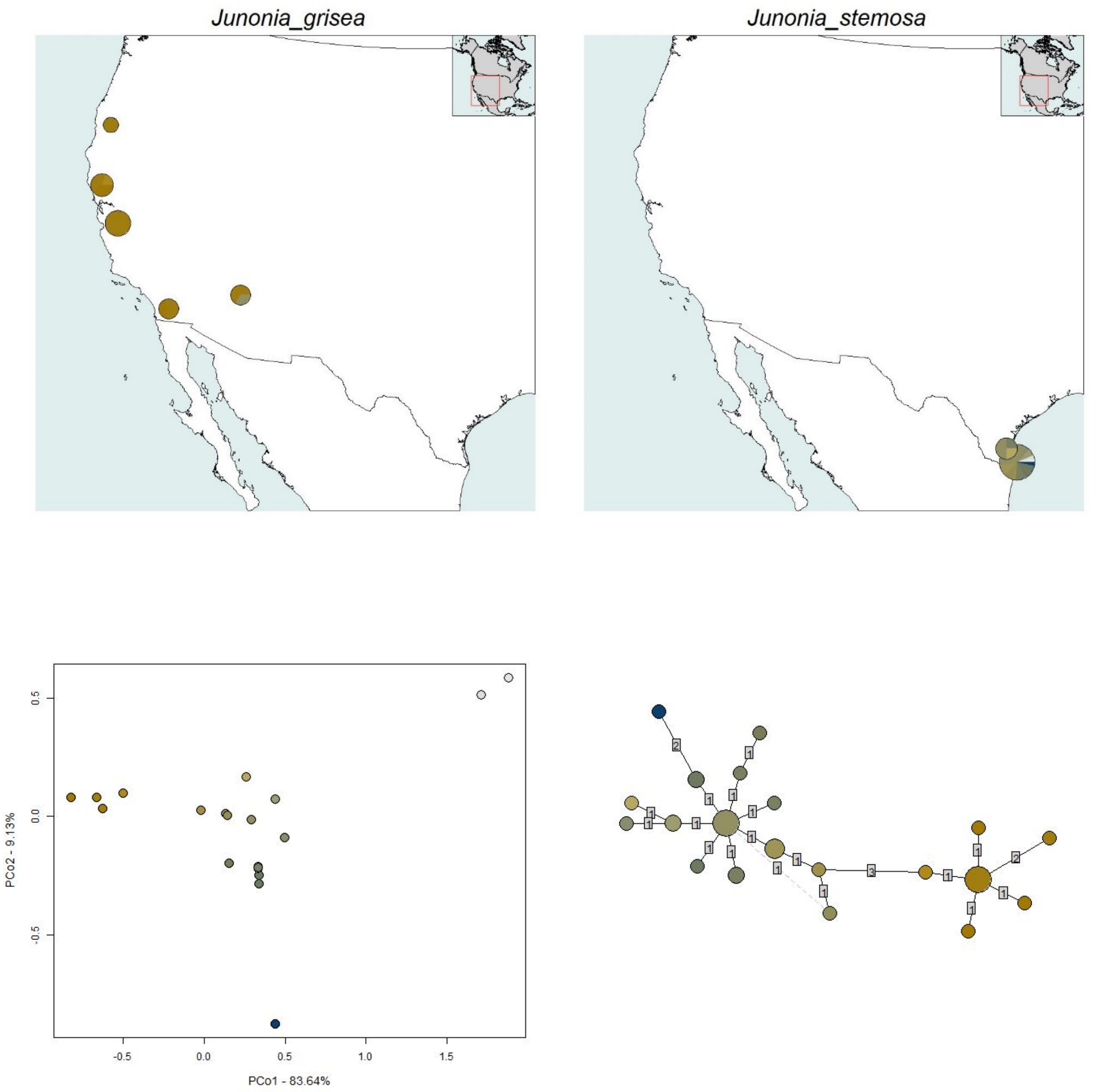

Figure 997: Haplotype maps, PCoA projection, and haplotype network for four *Junonia* species displaying barcode sharing.

Phyciodes_tharos

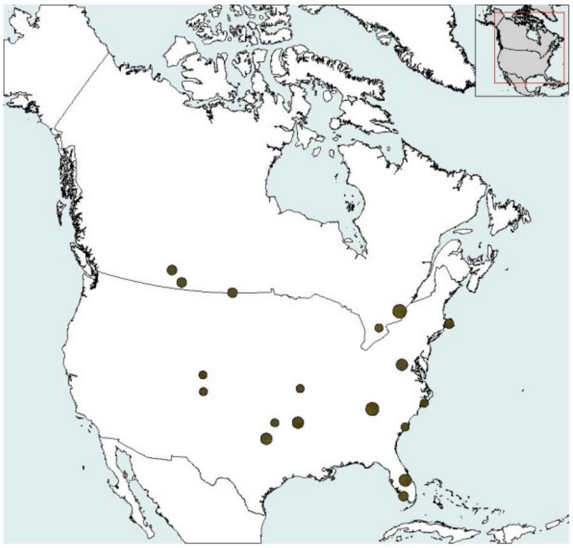

Phyciodes_pulchella

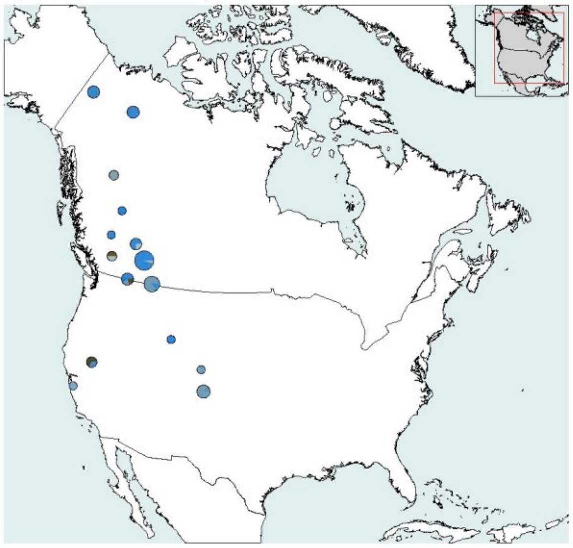

Phyciodes_cocyta

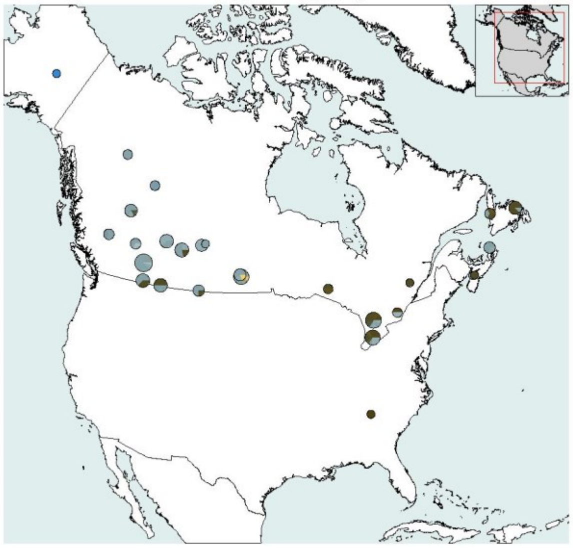

Phyciodes_batesii

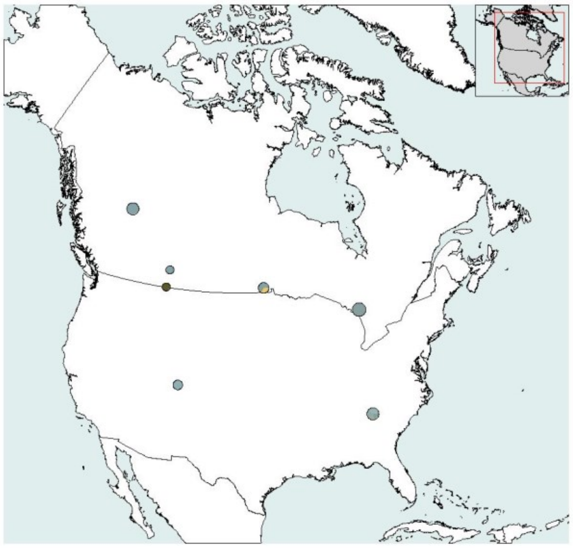

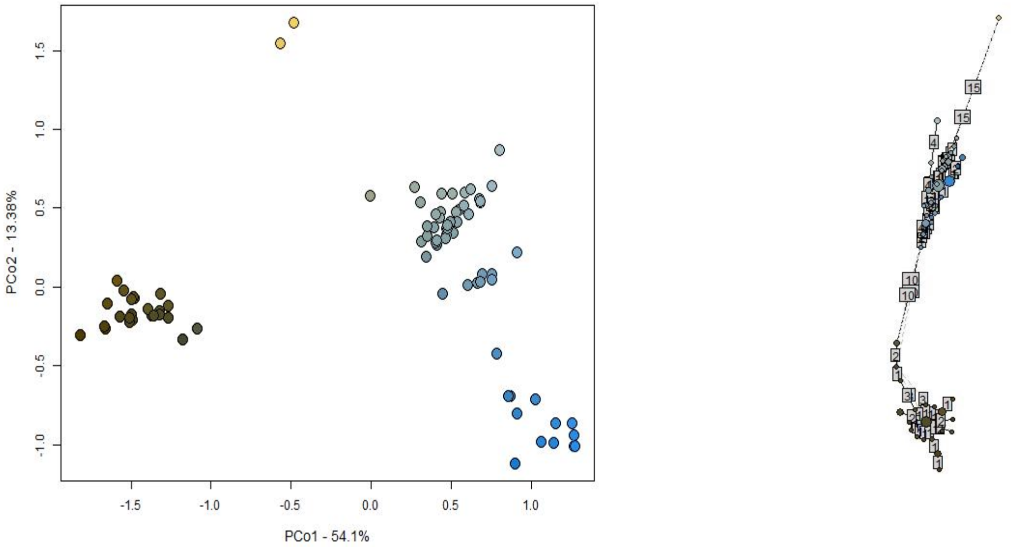

Figure 998: Haplotype maps, PCoA projection, and haplotype network for four *Phyciodes* species displaying barcode sharing.

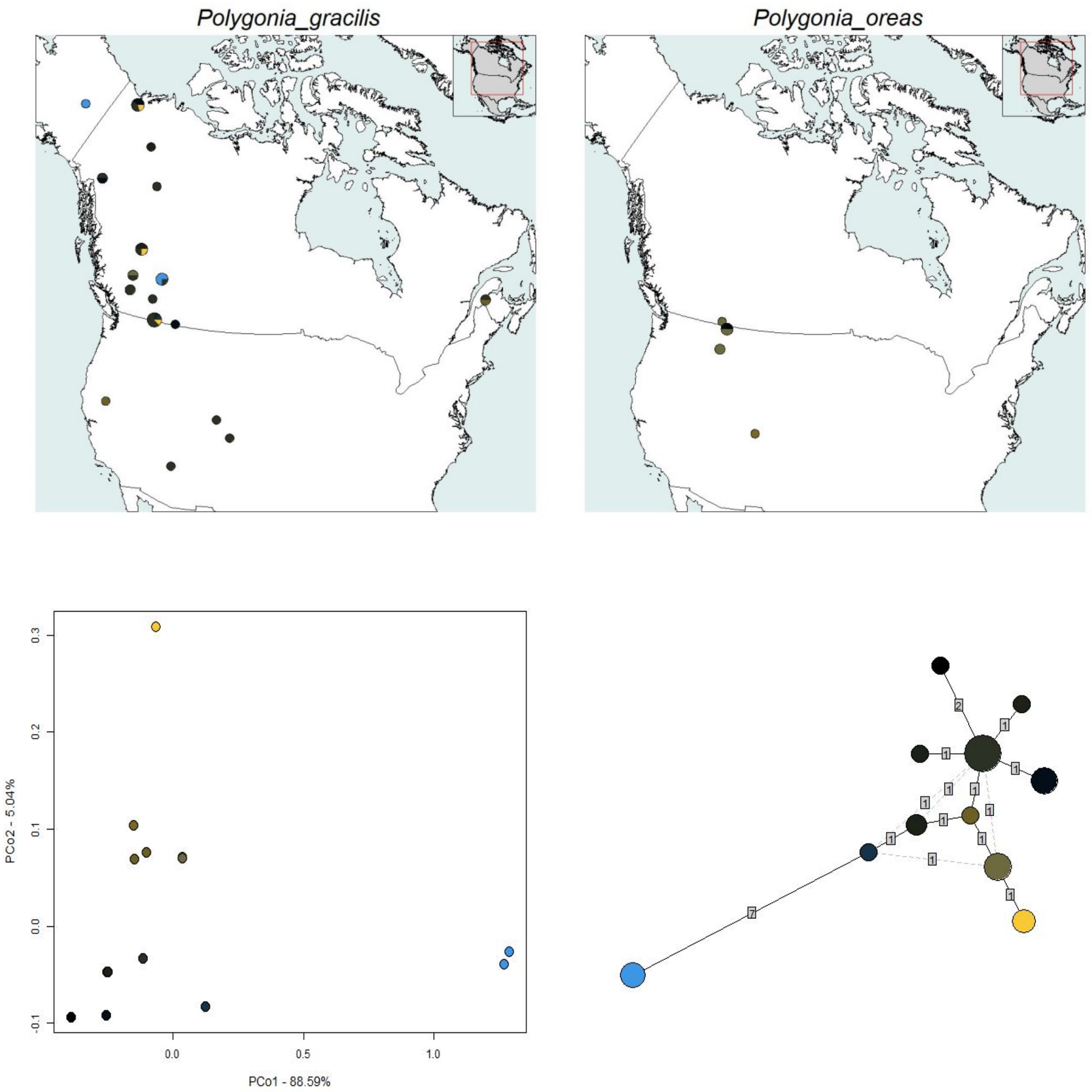

Figure 999: Haplotype maps, PCoA projection, and haplotype network for two *Polygonia* species displaying barcode sharing.

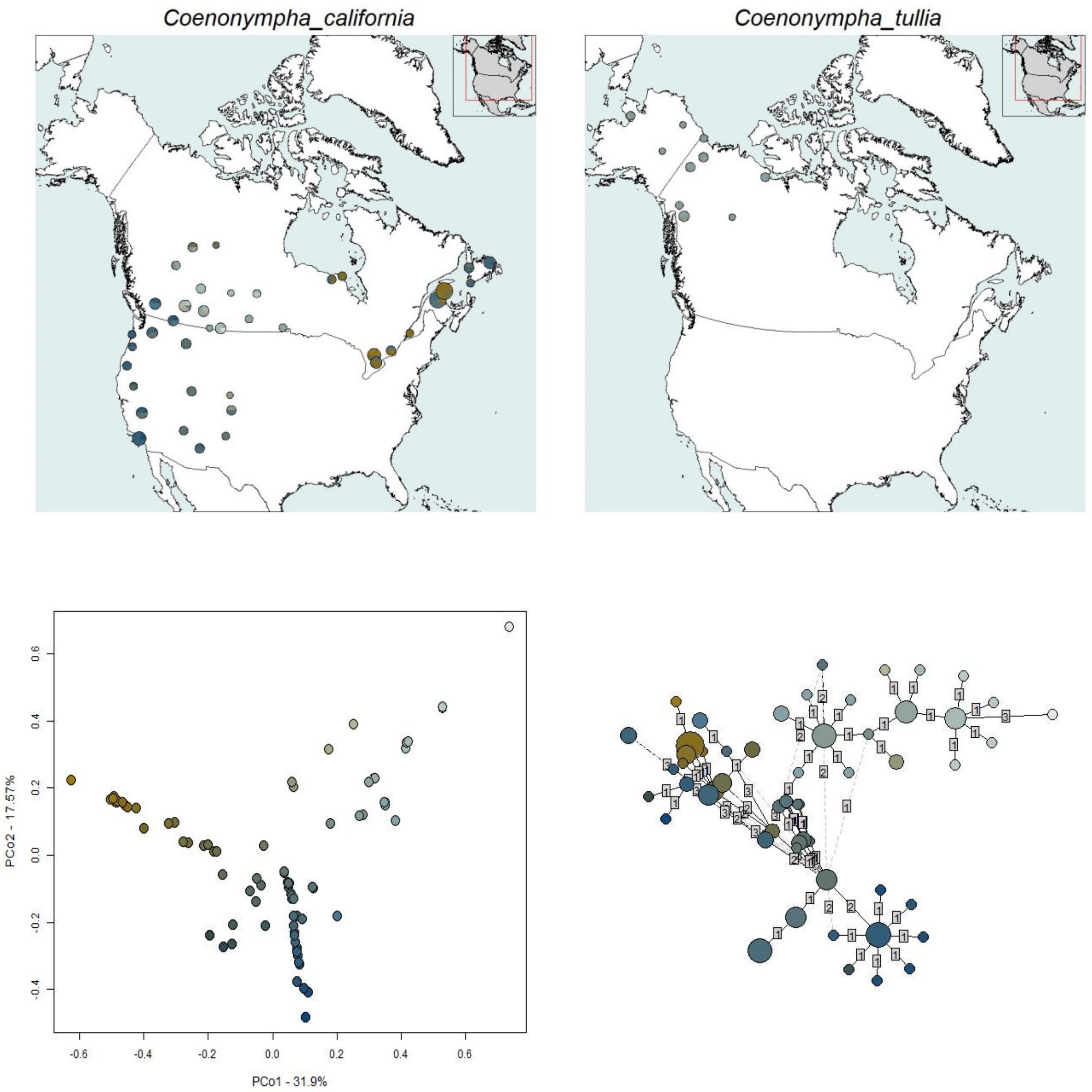

Figure 1000: Haplotype maps, PCoA projection, and haplotype network for two *Coenonympha* species displaying barcode sharing.

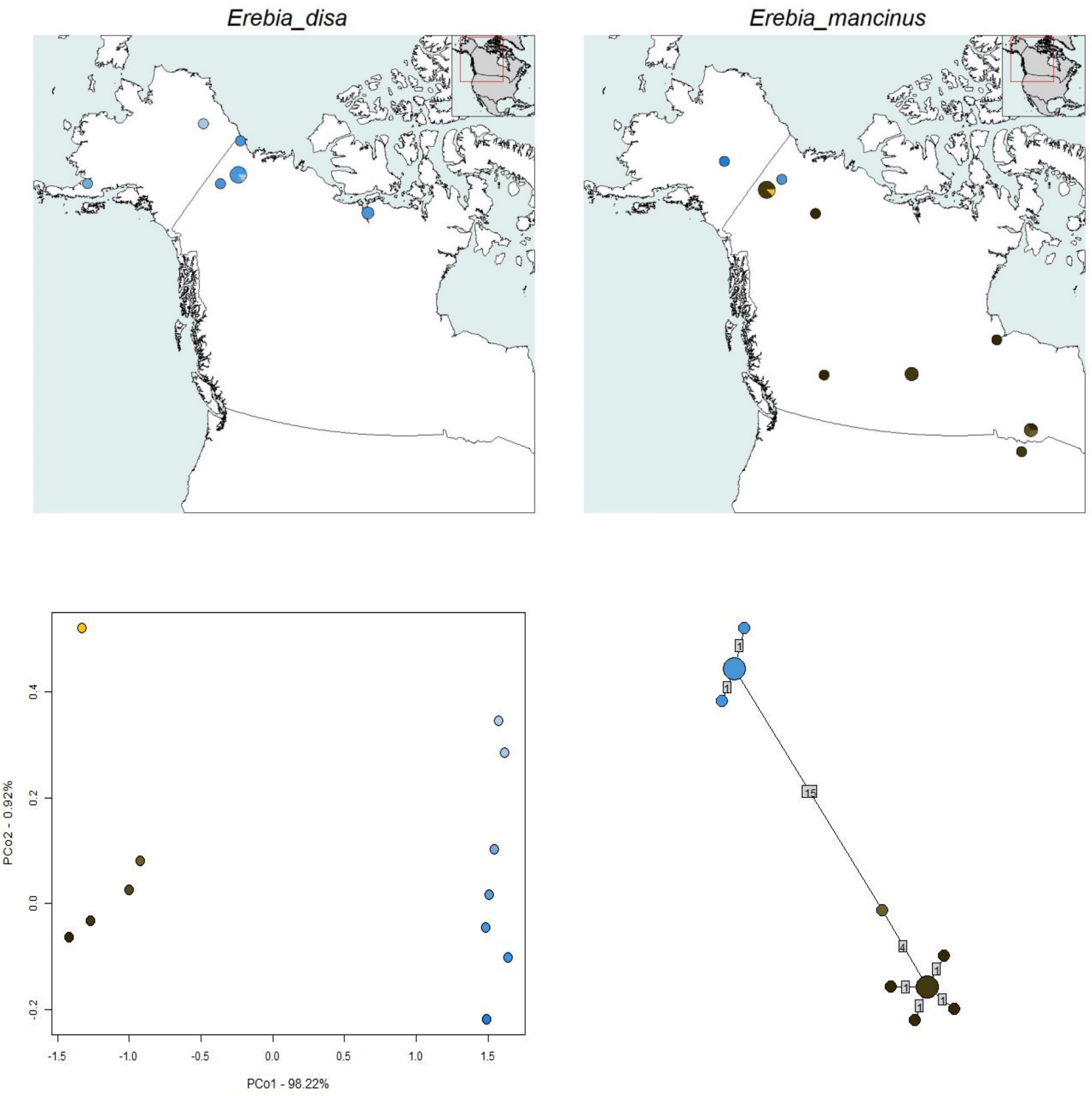

Figure 1001: Haplotype maps, PCoA projection, and haplotype network for two *Erebia* species displaying barcode sharing.

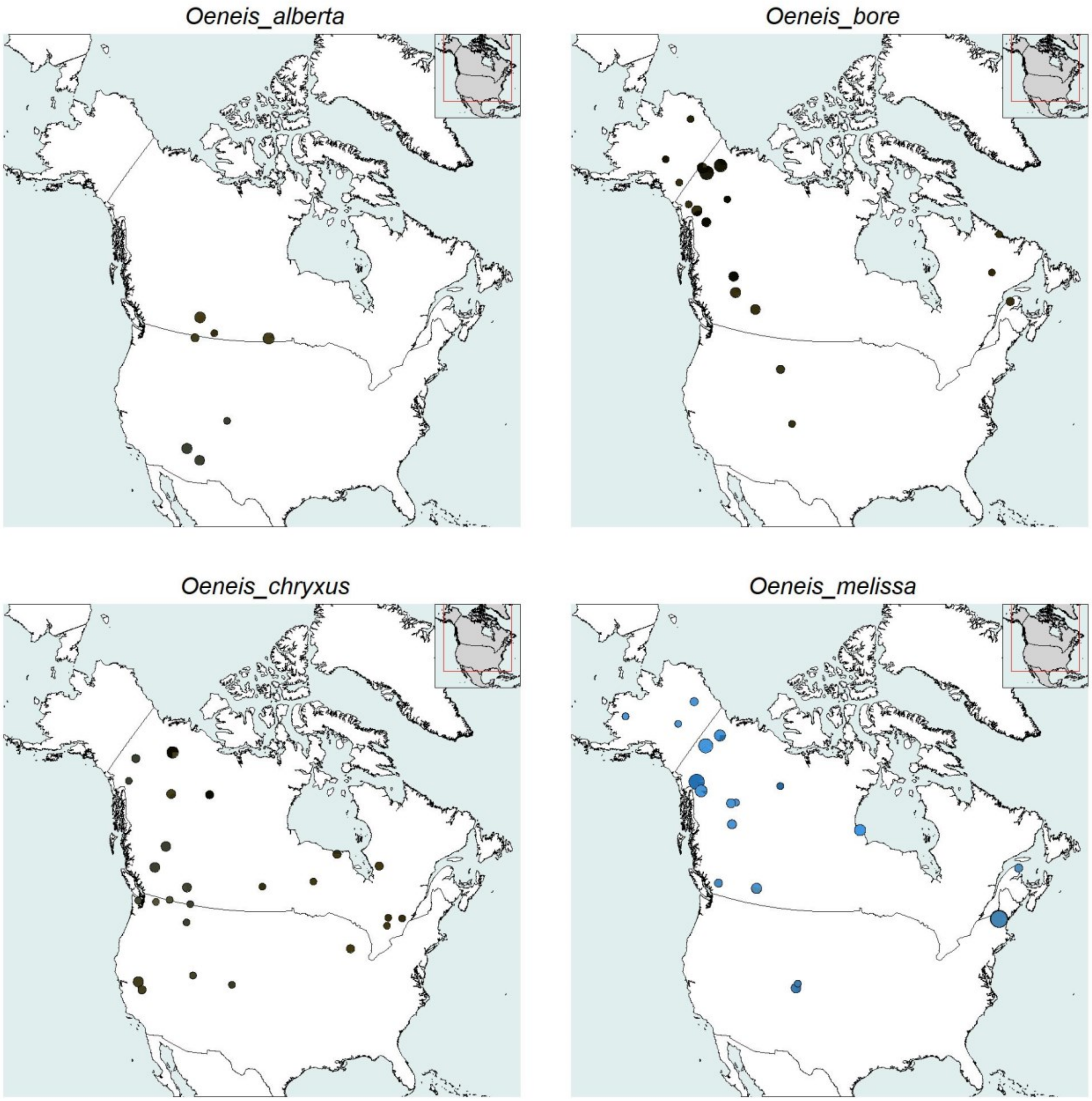
Oeneis_alberta
Oeneis_bore
Oeneis_chryxus
Oeneis_melissa

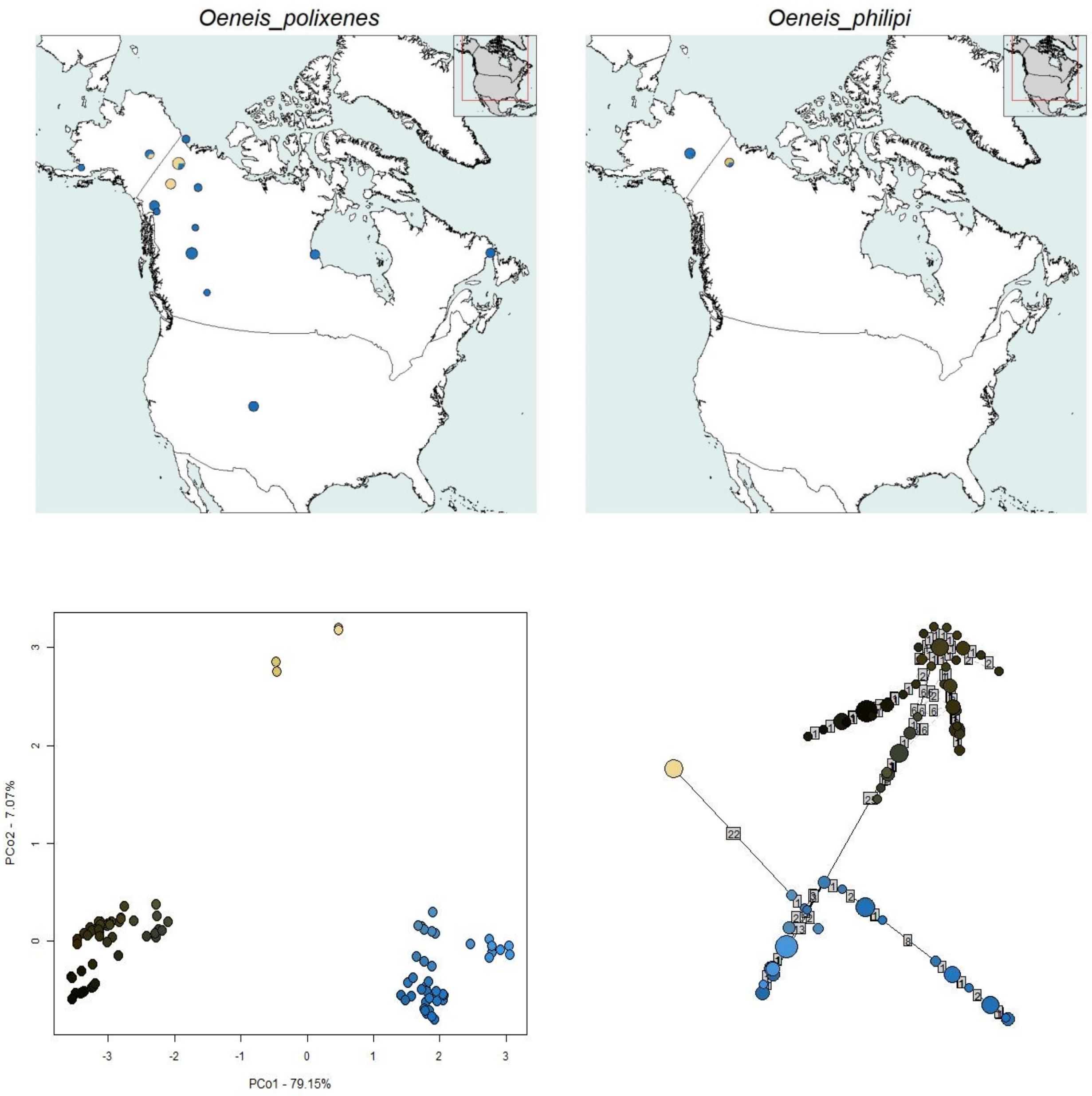

Figure 1002: Haplotype maps, PCoA projection, and haplotype network for six *Oeneis* species displaying barcode sharing.

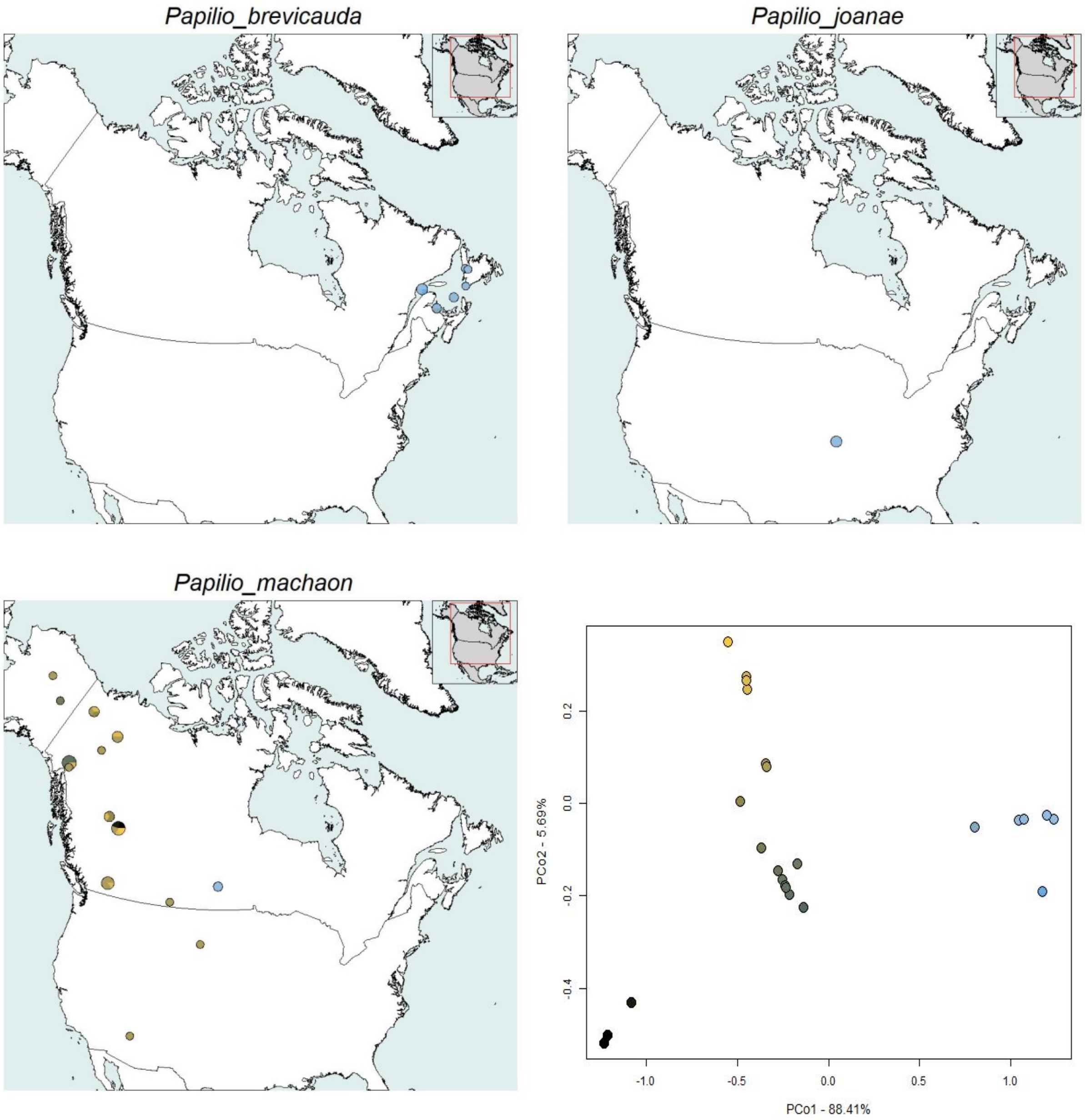
Papilio_brevicauda
Papilio_joanae
Papilio_machaon
PCo1 - 88.41%
PCo2 - 5.69%
-1.0
-0.5
0.0
0.5
1.0
0.2
0.0
-0.2
-0.4

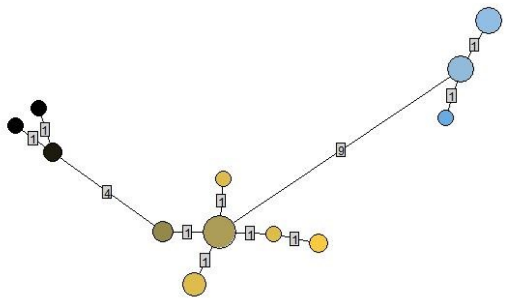

Figure 1003: Haplotype maps, PCoA projection, and haplotype network for three *Papilio* species displaying barcode sharing.

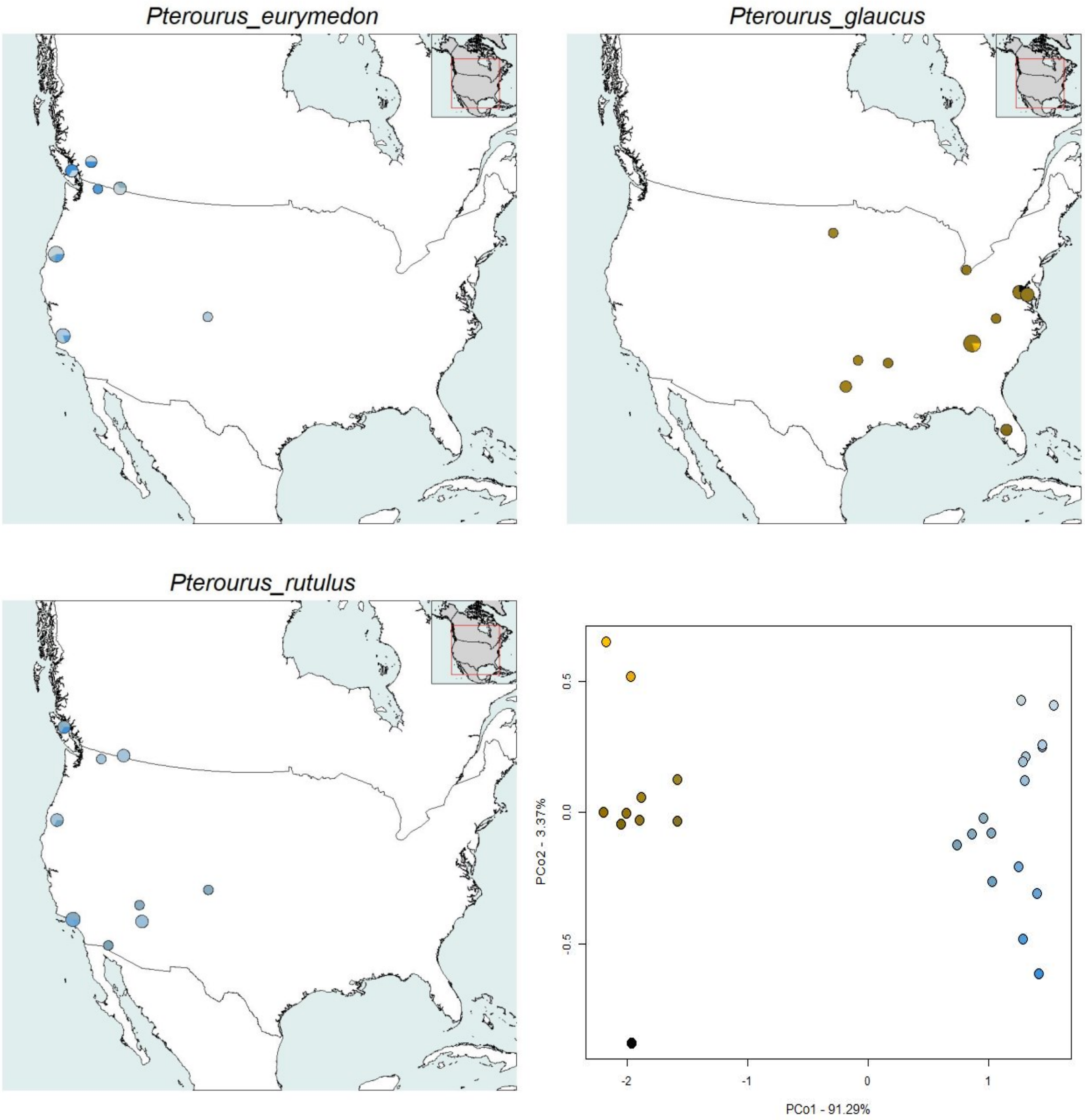
Pterourus_eurymedon
Pterourus_glaucus
Pterourus_rutulus
PCo2 - 3.37%
0.5
0.0
-0.5
-2
-1
0
1
PCo1 - 91.29%

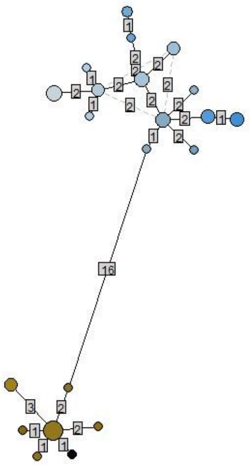

Figure 1004: Haplotype maps, PCoA projection, and haplotype network for three *Pterourus* species displaying barcode sharing.

Colias_alexandra

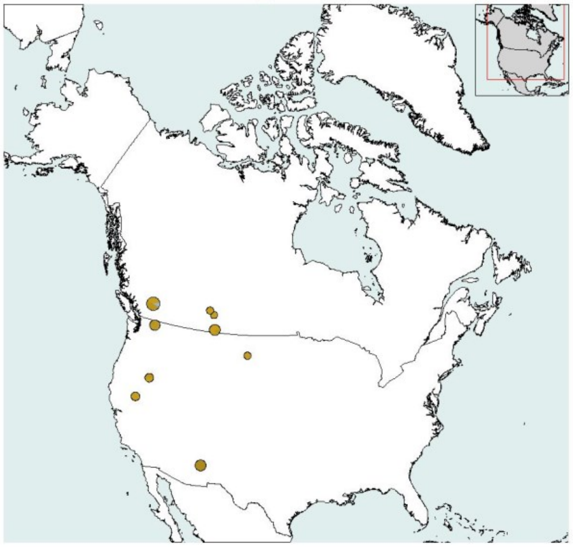

Colias_behrii

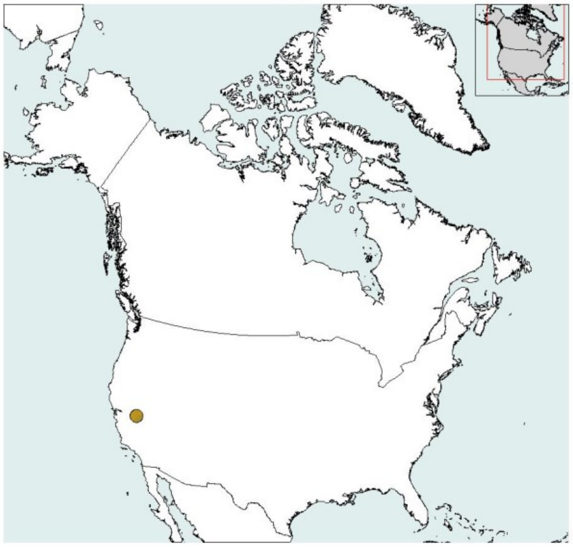

Colias_boothii

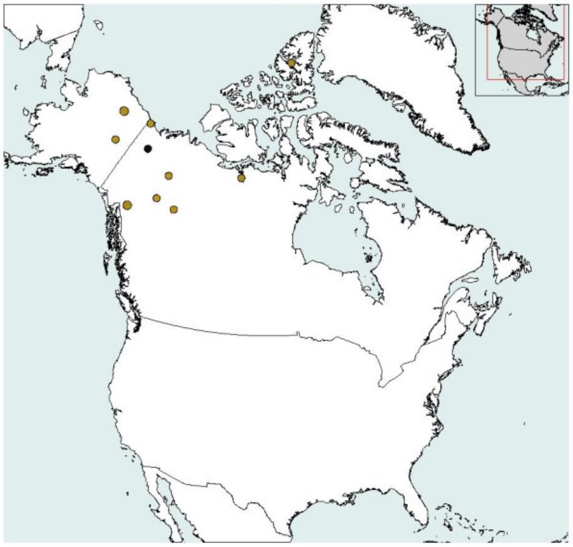

Colias_christina

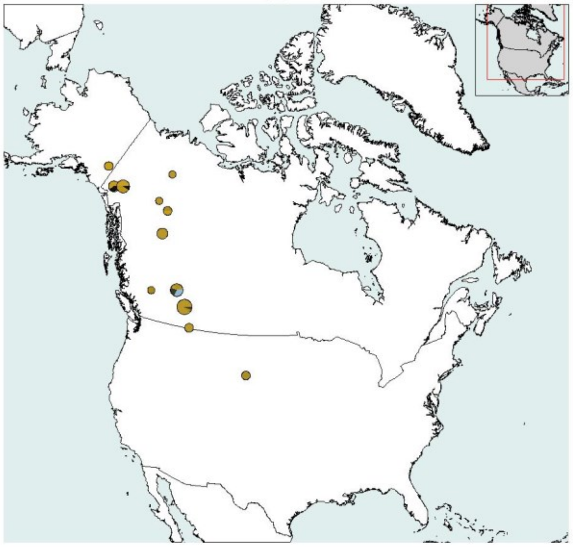

Colias_eriphyle

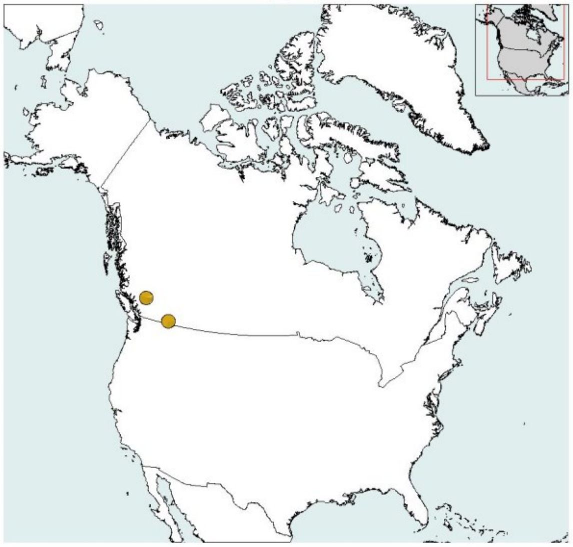

Colias_eurytheme

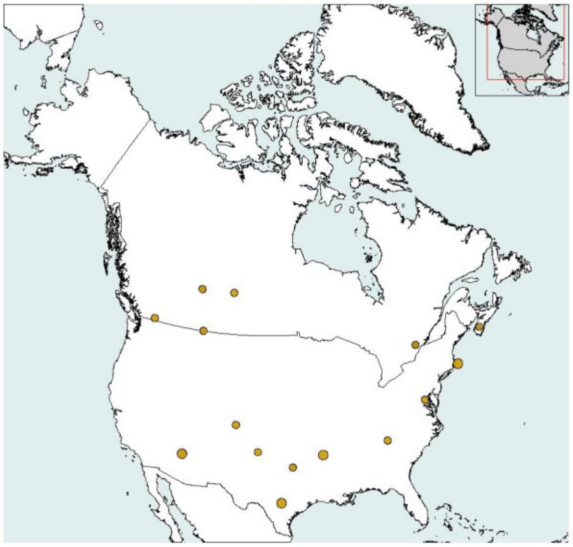

Colias_gigantea

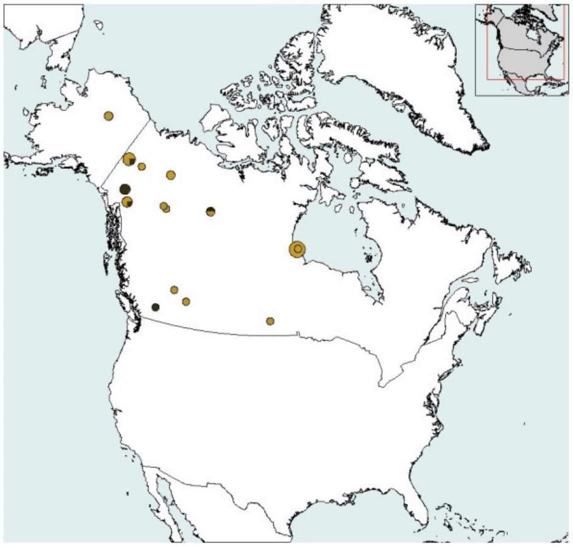

Colias_harfordii

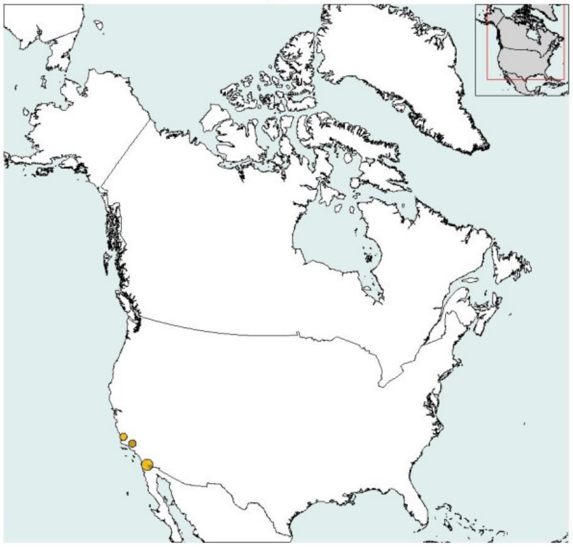

Colias_hecla

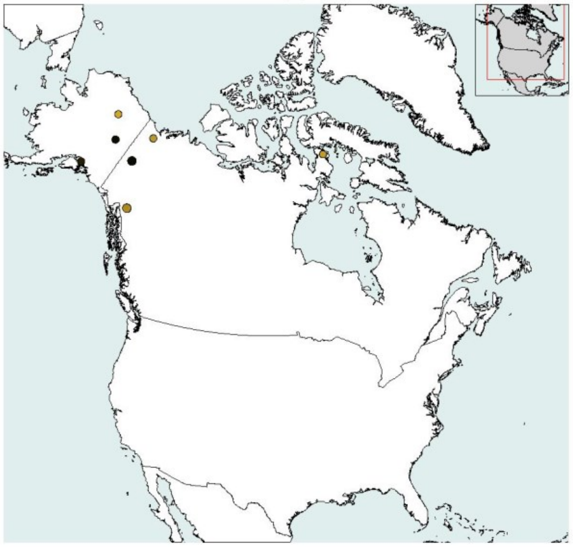

Colias_interior

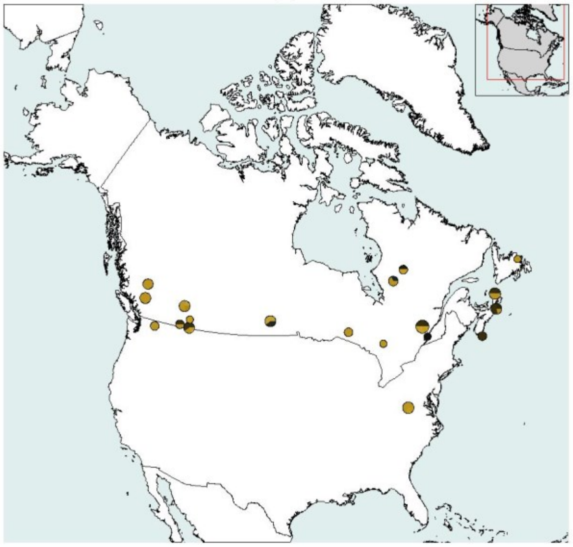

Colias_johanseni

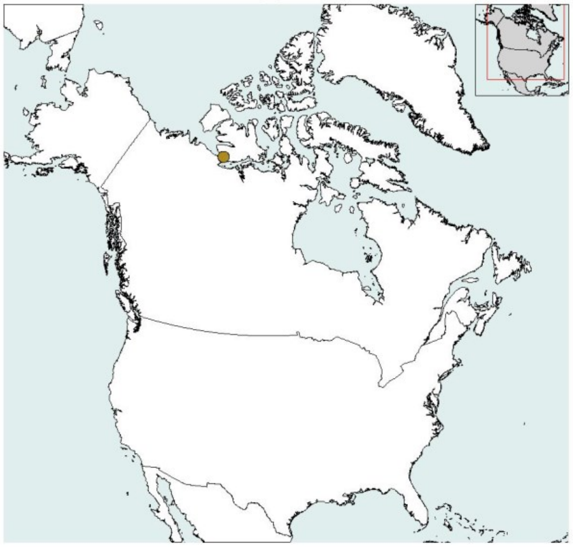

Colias_meadii

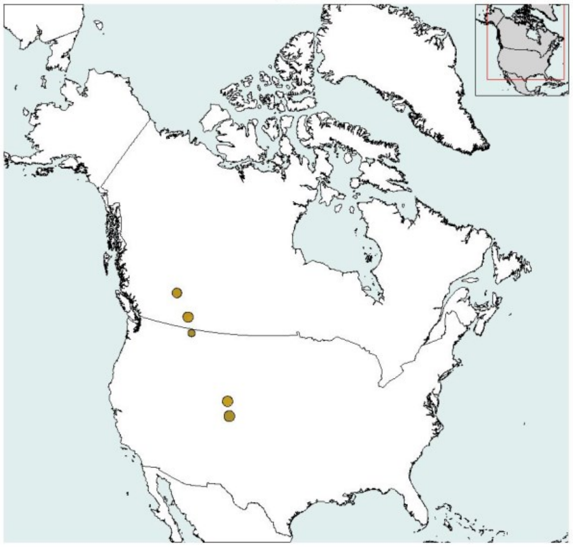

Colias_nastes

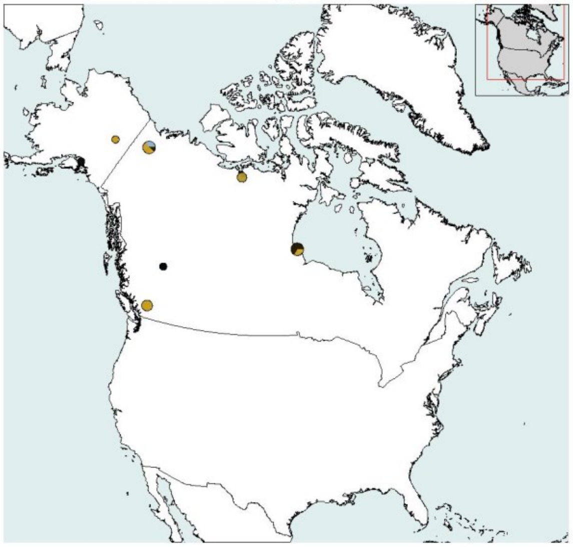

Colias_occidentalis

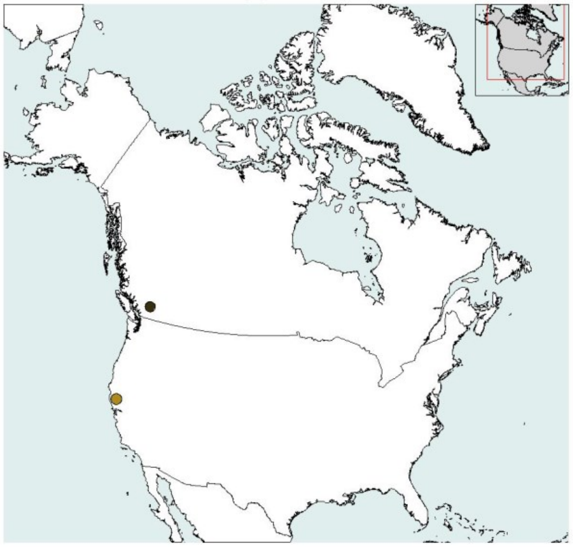

Colias_palaeno

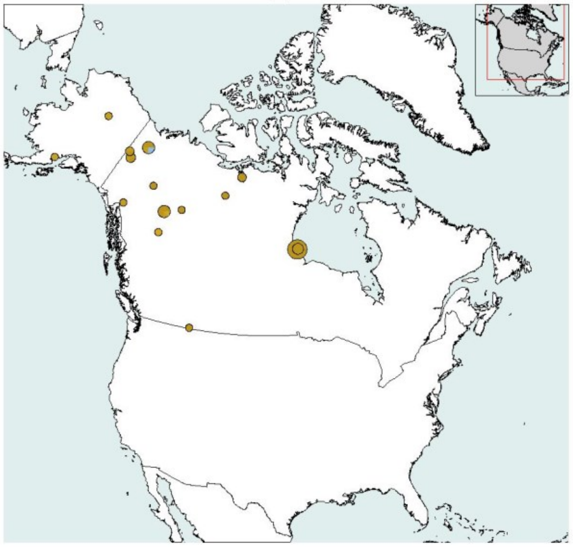

Colias_pelidne

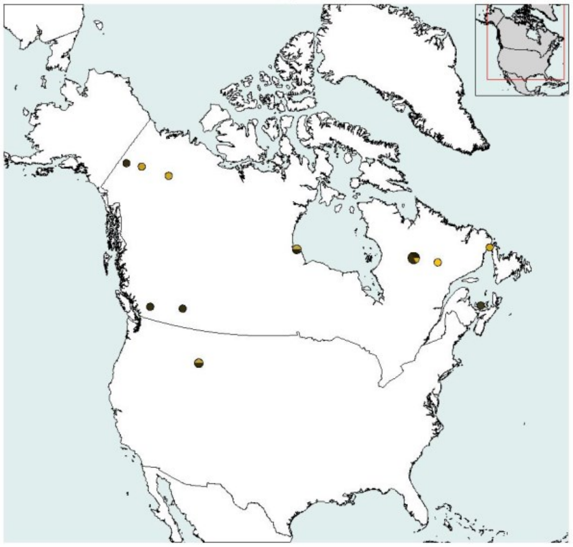

Colias_philodice

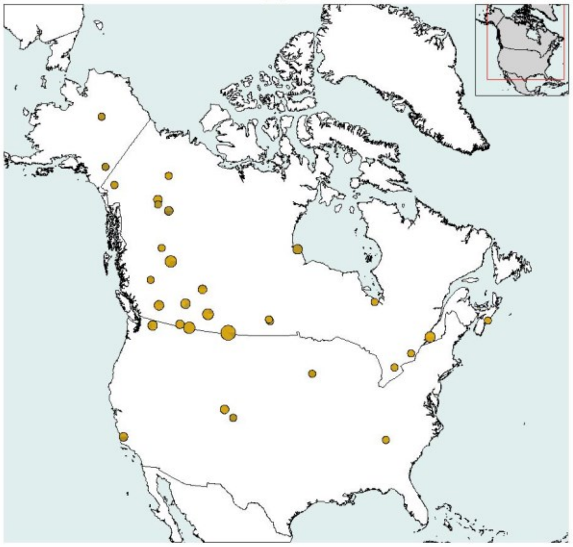

Colias_rankinensis

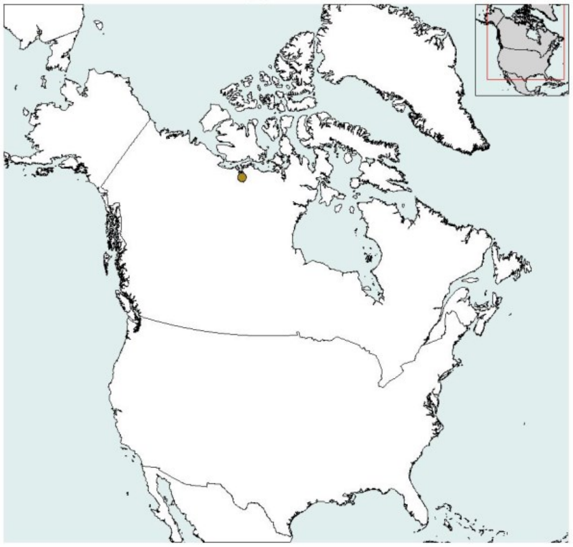

Colias_scudderii

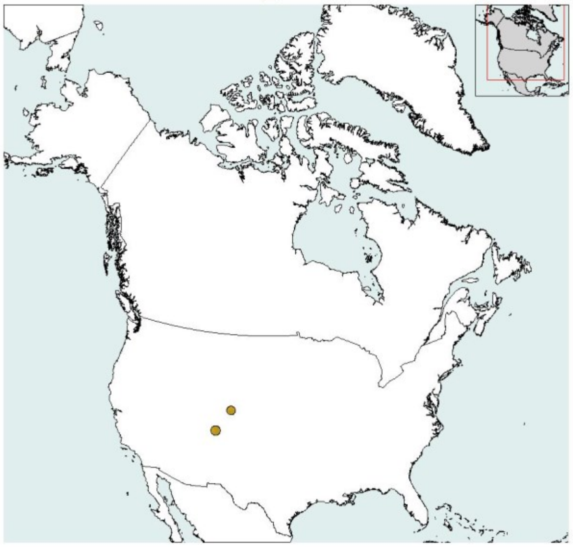

Colias_skinneri

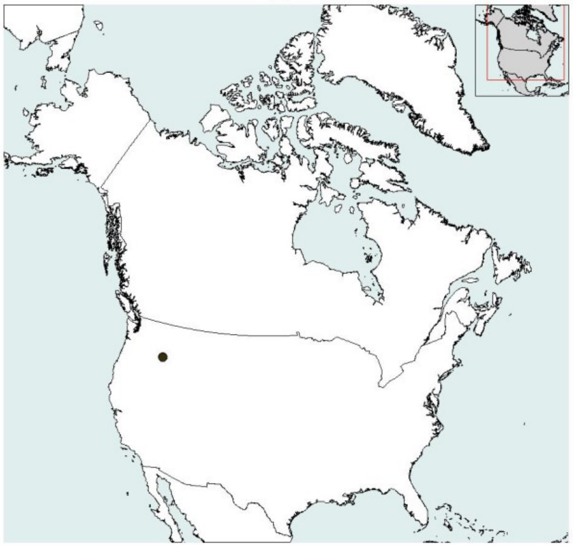

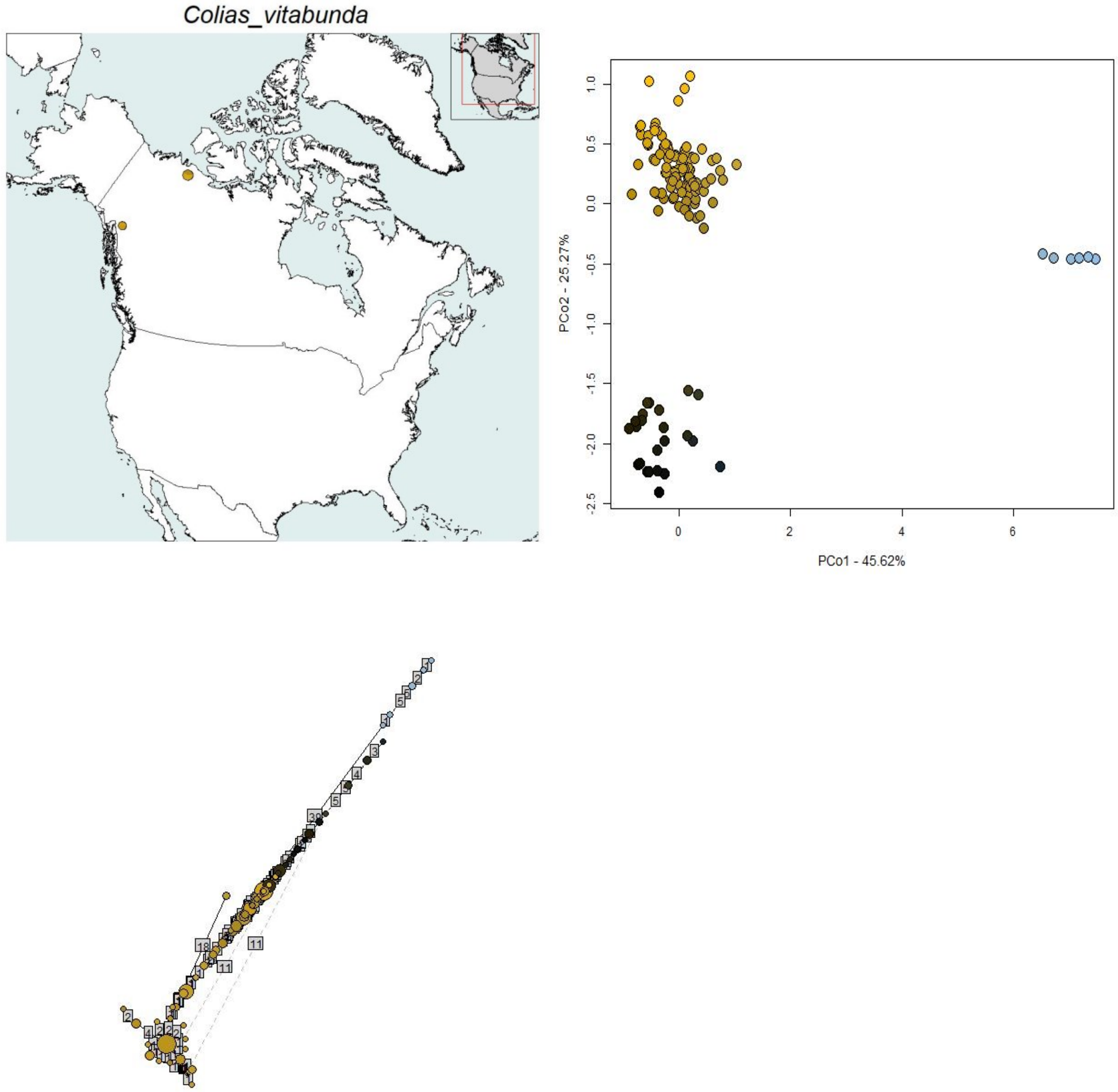

Figure 1005: Haplotype maps, PCoA projection, and haplotype network for 21 *Colias* species displaying barcode sharing.

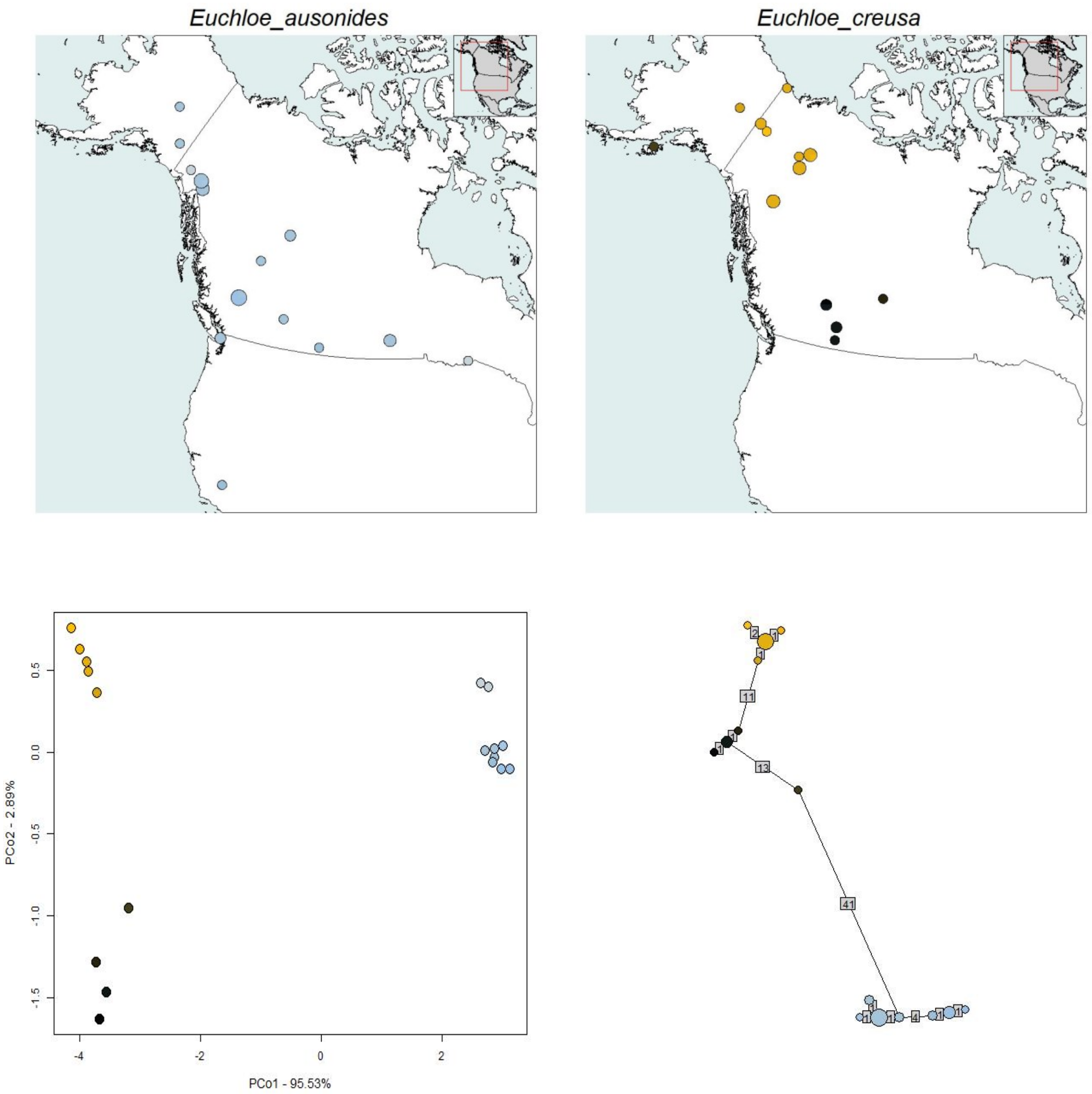

Figure 1006: Haplotype maps, PCoA projection, and haplotype network for two *Euchloe* species displaying barcode sharing.

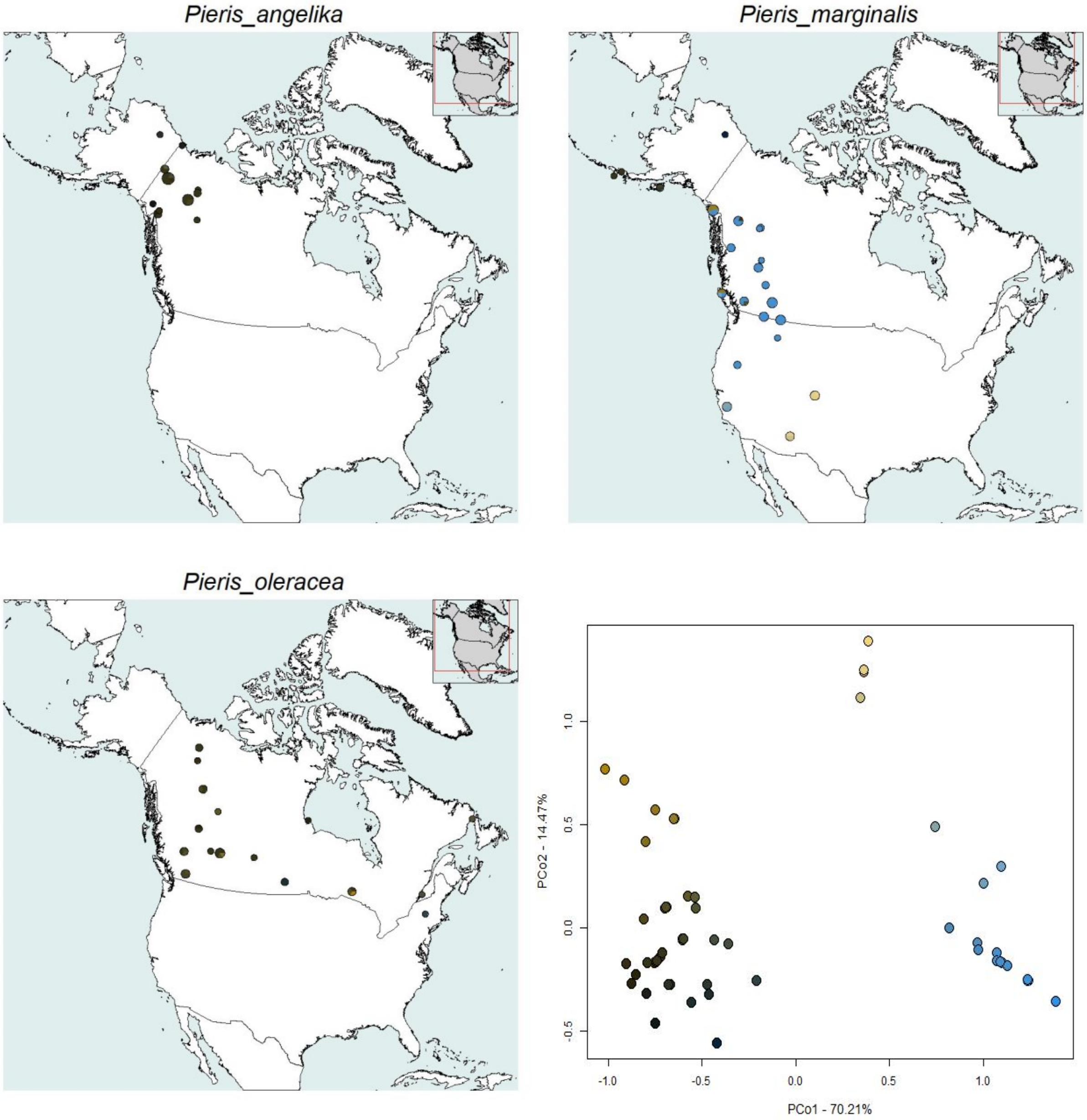
Pieris_angelika
Pieris_marginalis
Pieris_oleracea
PCo2 - 14.47%
PCo1 - 70.21%
-1.0
-0.5
0.0
0.5
1.0

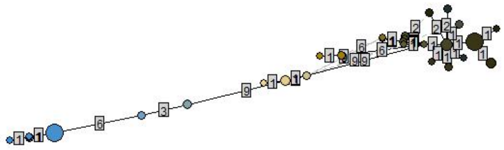

Figure 1007: Haplotype maps, PCoA projection, and haplotype network for three *Pieris* species displaying barcode sharing.
