# Supplementary figures and images for: A genetic atlas for the butterflies of continental Canada and United States
Source: PLoS One. 2024 Apr 3;19(4):e0300811. doi: 10.1371/journal.pone.0300811 (PMC10990199; doi:10.1371/journal.pone.0300811)

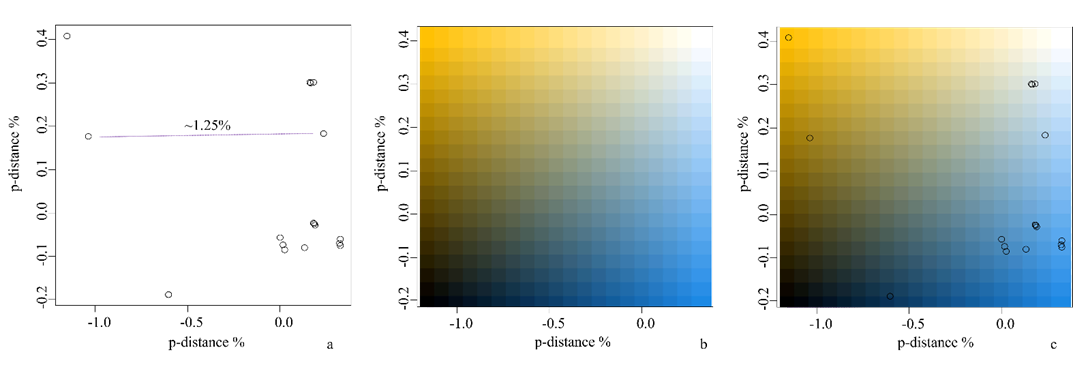

Supplement: S1 Fig — Principal coordinate analysis (PCoA) for Limenitis arthemis (a). The RGB (red blue green) square employed to assign colors to haplotypes (b) and the projection of the PCoA configuration in the RGB space (c). (PNG) [file pone.0300811.s002.png]

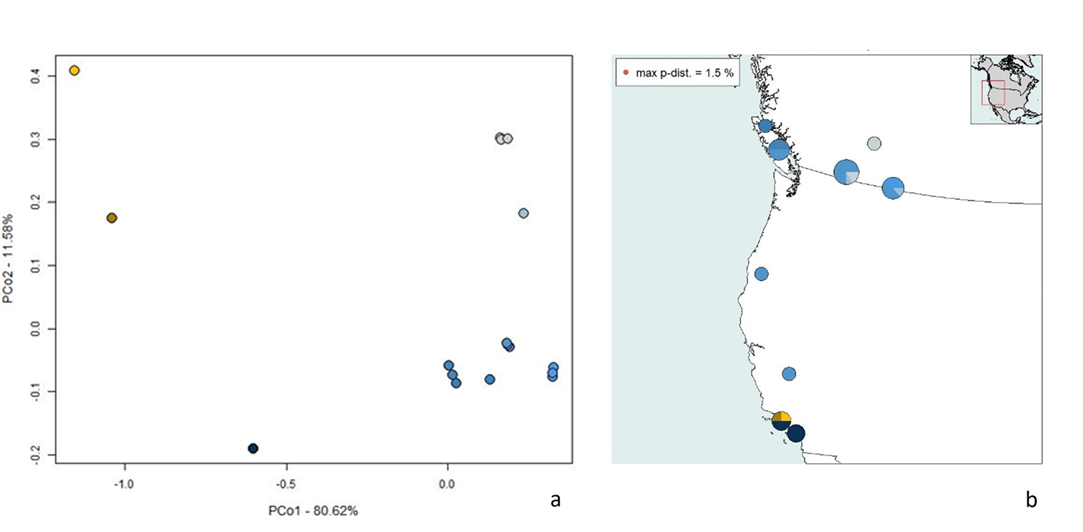

Supplement: S2 Fig — The representation of PCoA in RGB space as showed in the Atlas (a) and the resulting haplotype map (b) for 36 specimens of Limenitis lorquini. (PNG) [file pone.0300811.s003.png]

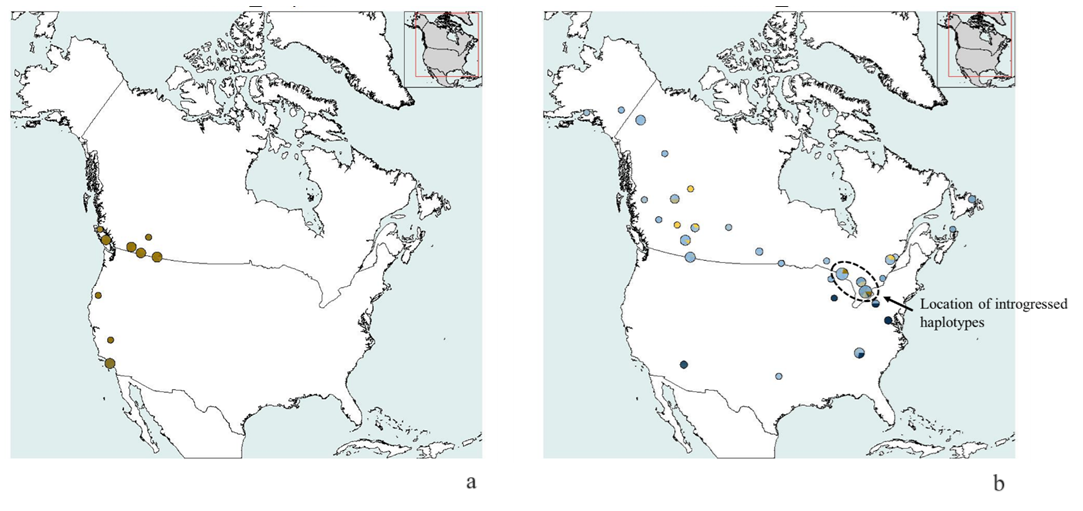

Supplement: S3 Fig — Haplotype maps for 36 specimens of Limenitis lorquini (a) and 102 specimens of Limenitis arthemis (b). (PNG) [file pone.0300811.s004.png]

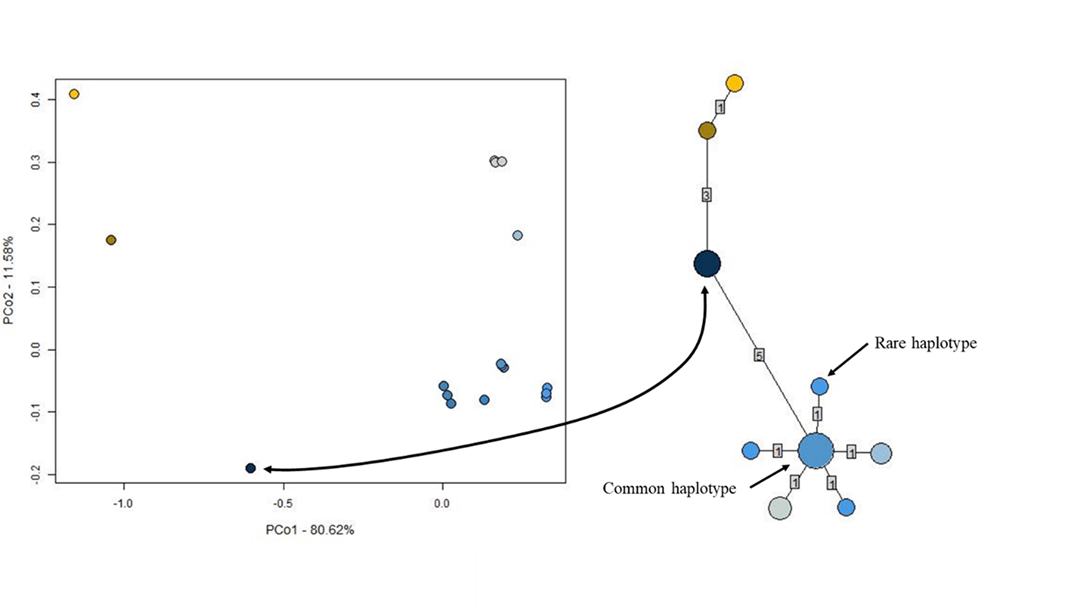

Supplement: S4 Fig — PCoA (a) and haplotype network (b) for 36 specimens of Limenitis lorquini. Because colors assigned to haplotypes are the same for the PCoA and the haplotype network, it is possible to easily identify same haplotypes in the two plots. (PNG) [file pone.0300811.s005.png]

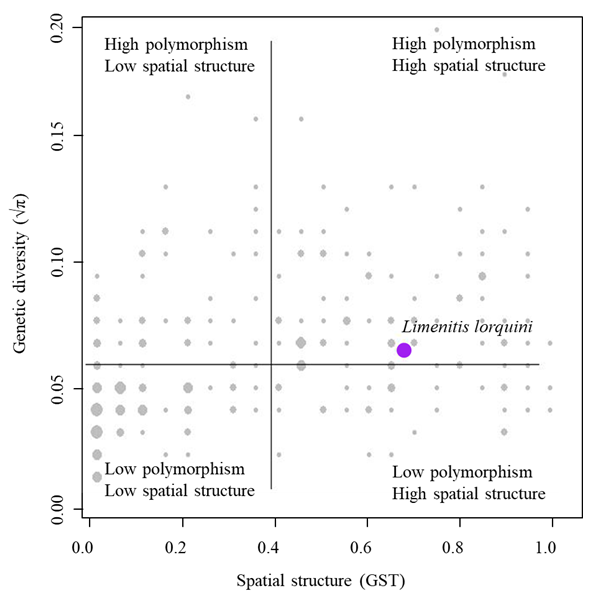

Supplement: S5 Fig — Values for all species in the Atlas are depicted as grey bubbles and the species of interest (Limenitis lorquini) is represented by a purple dot. The black lines represent median values for all species of the Atlas. (PNG) [file pone.0300811.s006.png]
